# Genome-wide generation and use of informative intron-spanning and intron-length polymorphism markers for high-throughput genetic analysis in rice

**DOI:** 10.1038/srep23765

**Published:** 2016-04-01

**Authors:** Saurabh Badoni, Sweta Das, Yogesh K. Sayal, S. Gopalakrishnan, Ashok K. Singh, Atmakuri R. Rao, Pinky Agarwal, Swarup K. Parida, Akhilesh K. Tyagi

**Affiliations:** 1 National Institute of Plant Genome Research (NIPGR), Aruna Asaf Ali Marg, New Delhi 110067, India; 2 Centre for Agricultural Bioinformatics, Indian Council of Agricultural Research (ICAR)-Indian Agricultural Statistics Research Institute, New Delhi 110012, India; 3 Division of Genetics, Rice Section, Indian Agricultural Research Institute (IARI), New Delhi 110012, India

## Abstract

We developed genome-wide 84634 ISM (intron-spanning marker) and 16510 InDel-fragment length polymorphism-based ILP (intron-length polymorphism) markers from genes physically mapped on 12 rice chromosomes. These genic markers revealed much higher amplification-efficiency (80%) and polymorphic-potential (66%) among rice accessions even by a cost-effective agarose gel-based assay. A wider level of functional molecular diversity (17–79%) and well-defined precise admixed genetic structure was assayed by 3052 genome-wide markers in a structured population of *indica, japonica*, aromatic and wild rice. Six major grain weight QTLs (11.9–21.6% phenotypic variation explained) were mapped on five rice chromosomes of a high-density (inter-marker distance: 0.98 cM) genetic linkage map (IR 64 x Sonasal) anchored with 2785 known/candidate gene-derived ISM and ILP markers. The designing of multiple ISM and ILP markers (2 to 4 markers/gene) in an individual gene will broaden the user-preference to select suitable primer combination for efficient assaying of functional allelic variation/diversity and realistic estimation of differential gene expression profiles among rice accessions. The genomic information generated in our study is made publicly accessible through a user-friendly web-resource, “*Oryza ISM-ILP* marker” database. The known/candidate gene-derived ISM and ILP markers can be enormously deployed to identify functionally relevant trait-associated molecular tags by optimal-resource expenses, leading towards genomics-assisted crop improvement in rice.

The development and large-scale genotyping of gene-derived markers is vital for fast-paced identification and fine-mapping/positional cloning of genes/QTLs regulating important agronomic traits and genetic enhancement in rice. Effective deployment of sequence-based robust genic SSR (simple sequence repeat) and SNP (single nucleotide polymorphism) markers has been made at whole genome level to accelerate multi-dimensional high-throughput genetic analysis in rice^1^,^2^,^3^,^4^,^5^,^6^,^7^,^8^,^9^,^10^,^11^,^12^,^13^,^14^,^15^,^16^,^17^,^18^. These genetic markers, despite broader applicability, usually suffer from certain shortcomings, which restrict their use in genomics-assisted breeding applications of rice. Some of these limitations include less abundance and lower polymorphic potential of multi-allelic SSR markers specifically in the genic sequence components of genome and need of specialized cost-intensive infrastructural facilities (genotyping platforms) for large-scale validation and high-throughput genotyping of bi-allelic abundant SNP markers. Therefore, development of multi-allelic gene-derived markers specifically revealing wider genomic distribution as well as higher polymorphic potential among rice accessions by simplified marker genotyping using an affordable assay is a prerequisite.

The introns are abundant in most eukaryotic genomes and widely distributed in diverse sequence components of genes^19^,^20^. Introns being under low purifying selection pressure are evolutionarily less conserved and highly variable than coding sequences, thus can be well-exploited as highly polymorphic genetic markers. Consequently, in recent years, introns of genes have been annotated and targeted to develop successful intron-spanning markers (ISM) and/or intron-length polymorphism (ILP) markers at a genome-wide scale to be utilised for various large-scale genotyping applications in multiple major food crop plants, including rice^21^,^22^,^23^,^24^, wheat^25^, maize^26^, foxtail millet^27^,^28^, *Medicago*^29^, soybean^30^, tomato^31^, cowpea^32^, *Brassica*^33^ and chickpea^34^,^35^,^36^. The ISM and ILP markers are largely preferred in plant genomics and molecular breeding due to a wide spectrum of desirable genetic attributes, including high specificity, robustness and reproducibility nature, co-dominant multi-allelic genetic inheritance pattern and abundant genomic distribution especially in the gene regions of crop genomes^21^,^37^. The efficacy of these markers is more evident from their ability to impart direct reflection of allelic variation/diversity within the genes and thus have utility for rapidly establishing straightforward association between markers and traits of agronomic importance in crop plants. Moreover, the ISM and ILP markers have the potential to differentiate closely-related accessions efficiently by their convenient PCR amplification and simpler cost-effective agarose gel-based assay^21^. The practical utility of ISM and ILP markers for diverse high-throughput genotyping applications like genetic diversity analysis, construction of high-density genetic linkage maps, comparative genome mapping, evolutionary studies, mapping of genes/QTLs regulating important agronomic traits and marker-assisted breeding has been well-demonstrated in various crop plants^21^,^27^,^28^,^32^,^33^,^34^,^35^,^36^.

All the aforesaid crop (including rice) genome studies have adopted common homology-based approaches for development of ISM and ILP markers at a genome-wide scale. These strategies are primarily streamlined towards identification of *in silico* polymorphic introns by comparing the cDNA/EST (expressed sequence tags) sequences with genomic sequences of diverse accessions of a studied crop species and/or their evolutionarily closely-related sequenced model crop plant genomes^21^,^23^,^25^,^26^,^27^,^28^,^29^,^30^,^31^,^32^,^33^,^34^,^35^,^36^. Subsequently, efforts have been made to amplify and validate/genotype the correctly annotated polymorphic introns in diverse accessions by designing ISM and ILP marker primers from the exonic sequences flanking these introns. For instance, genome-wide ISM and ILP markers have been developed successfully in foxtail millet, chickpea and *Brassica* for genomics-assisted breeding applications by utilizing the genomic sequence information of phylogenetically more homologous model crop plant genome species, namely rice, *Medicago* and *Arabidopsis*, respectively, as references. The homology-based approaches for designing such ISM and ILP markers are not efficient enough to target all the genes annotated from the completely sequenced genome and thus usually provide low-resolution genomic coverage at whole genome level. Specifically in rice, ISM and ILP markers with dense genome-wide coverage are yet to be developed involving all the intronic sequence components of genes recently annotated from the completely sequenced genome. The gold standard genomic sequences, including structurally and functionally annotated genes of whole *japonica* rice (Nipponbare) genome and NGS (next-generation sequencing)-based genome resequences of diverse rice accessions are currently accessible. Henceforth, it is now possible to develop ISM initially at a genome-wide scale by targeting all individual introns present in the genes annotated from rice genome. Subsequently, each intron of these genes can be scanned for insertions-deletions (InDels) by comparing the corresponding whole genome sequences of multiple resequenced rice accessions^38^,^39^,^40^ in order to convert ISM into ILP markers. This strategy of developing ISM and ILP markers provides user with a wider flexibility to screen diverse combinations of informative primers from an individual gene exhibiting reproducible amplification as well as higher polymorphic potential for discrimination of rice accessions effectively. Henceforth, ISM and ILP markers are found to be more efficient in targeted mapping and identification of diverse arrays of genes directly on genome for expediting trait-associated genes/QTLs identification and marker-assisted breeding in rice. Considering these, the added advantage of abundant and multi-allelic gene-derived ISM and ILP markers as compared to SSR and SNP markers that were commonly utilized in rice genetic analysis is evident. This could be primarily due to higher efficiency of ISM and ILP markers in detecting polymorphism among rice accessions along with precise assay of differential expression profiles across tissues/stages of accessions by an affordable gel-based assay with optimal expense of resources. The ILP markers, especially targeting multiple InDels within an individual intron at a time for their amplification, thereby have higher likelihood potential of detecting polymorphism than InDel markers among rice accessions. The marker genotyping and differential gene expression profiling can be furthered by assaying identical set of ISM and ILP markers in both of these studies, which will eventually be helpful in molecular mapping of differentially expressed genes directly on the genome for successful rapid quantitative dissection of complex traits and genetic enhancement studies in rice.

In view of the above, the present study made an effort to develop genome-wide ISM and ILP markers by targeting/comparing individual introns of genes recently annotated from the sequenced whole genomes of *japonica* (Nipponbare) and upland *indica*/*aus* (Kasalath) rice accession. Large-scale validation and genotyping of these selected markers were performed to assess their potential to detect polymorphism, molecular diversity and population genetic structure among rice accessions. These informative ISM and ILP markers were further utilized to construct a high-density genetic linkage map for identification and molecular mapping of grain weight QTLs in rice. In addition to these DNA-based marker genotyping applications, the efficacy of genic ISM and ILP markers in accurate assaying and realistic estimation of differential gene expression profiling in diverse seed developmental stages of an *indica* (IR 64) rice accession was evaluated. All the rice ISM and ILP markers developed by us at a genome-wide scale were made available in the public domain through a user-friendly web resource, “*Oryza ISM-ILP* marker” database.

## Results and Discussion

### Genome-wide development and genomic constitution of rice ISM and ILP markers

**Figure 1:**
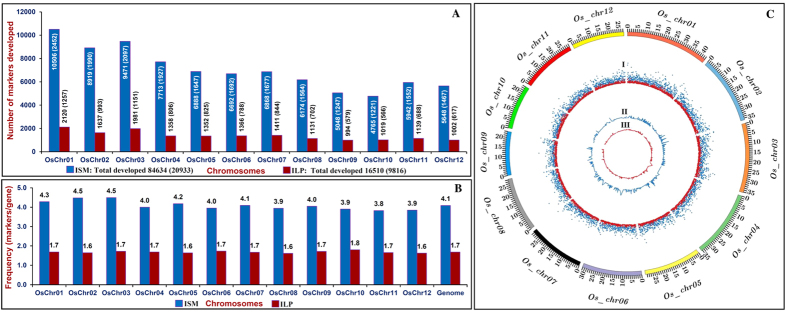
Genome-wide distribution pattern [number (**A**) and frequency (**B**)] of 84634 ISM and 16510 ILP markers designed from the intronic sequences of 20533 and 9816 genes annotated from 12 rice chromosomes. Number in parenthesis specifies rice genes with ISM and ILP markers. (**C**) The relative distribution of 84634 ISM and 16510 ILP markers physically mapped on 12 rice chromosomes are depicted by a Circos circular ideogram. The outermost circle denotes the physical size (Mb) of 12 rice chromosome-pseudomolecules coded with multiple colours. The next circle I signifies the ISM (blue) and ILP (red) markers designed from rice genes, whereas circles II and III indicate the ISM (blue) and ILP (red) markers, respectively, developed from known cloned genes regulating diverse agronomic traits (yield component and stress tolerance traits) in rice.

We developed a total of 84634 genome-wide ISM from the introns of 20533 protein-coding rice genes (6552 and 13981 TE and non-TE associated genes) that are physically mapped on 12 chromosomes (Table S1). Highest number of 10506 ISM were designed from the intronic sequences of 2452 genes annotated in chromosome 1 (Fig. 1A). This was followed by chromosomes 3 (9471 ISM in 2097 genes) and 2 (8919 ISM in 1990 genes), and lowest on chromosome 10 (4765 ISM in 1221 genes). A similar trend of frequency distribution of ISM was observed within the genes present in nearly all 12 rice chromosomes. However, it varied from 3.8 ISM per gene on chromosome 11 to 4.5 ISM per gene on chromosomes 2 and 3, with an average of 4.1 ISM/gene (Fig. 1B). At a whole genome level, the density of physically mapped ISM was found maximum on chromosome 3 (260.1 ISM/Mb), followed by chromosomes 2 (248.2 ISM/Mb) and 1 (242.8 ISM/Mb), and minimum on chromosome 11 (204.7 ISM/gene), with a mean of 226.8 ISM/Mb (Fig. S1). With an effort to convert ISM to ILP markers at whole genome level, we identified 29946 InDels within the introns of genes annotated between Nipponbare and Kasalath genomes. Based on the comparison of genomes for 29946 intronic-InDel polymorphism between Nipponbare and Kasalath, we were able to convert 16510 ISM into ILP markers targeting introns of 9816 rice genes. All these designed 16510 ILP markers were physically mapped on 12 chromosomes and found well-distributed across the rice genome (Table S2). A maximum number of 2120 ILP markers developed from the introns of 1257 rice genes were physically mapped on chromosome 1, whereas it was minimum on chromosome 9 (994 ILP markers in 579 genes) (Fig. 1A). The *in silico* fragment length polymorphism detected by ILP markers between Nipponbare and Kasalath based on sum of InDels-size variation within introns of genes ranged from 1 to 101 bp, with an average of 4.8 bp. The abundance of ILP markers showing 1 to 4-bp (74.2%, 12260 of 16510 markers) InDels-based fragment length polymorphism in the introns of genes between Nipponbare and Kasalath genomes was apparent. About 13.9 and 0.4% ILP markers exhibited 10 to 101-bp and 50 to 101-bp intronic InDels-based fragment length polymorphism, respectively. Even though, a similar trend of frequency distribution of ILP markers was observed in all 12 rice chromosomes; however, this varied from 1.6 to 1.8 ILP/gene, with an average of 1.7 ILP/gene (Fig. 1B). The genome coverage estimation based on density of physically mapped ILP markers revealed their highest density on chromosome 3 (54.4 ILP/Mb), followed by chromosomes 1 (48.9 ILP/Mb) and 7 (47.5 ILP/Mb), and lowest on chromosome 12 (36.4 ILP/Mb), with an average of 44.2 ILP/Mb (Fig. S2).

**Figure 2:**
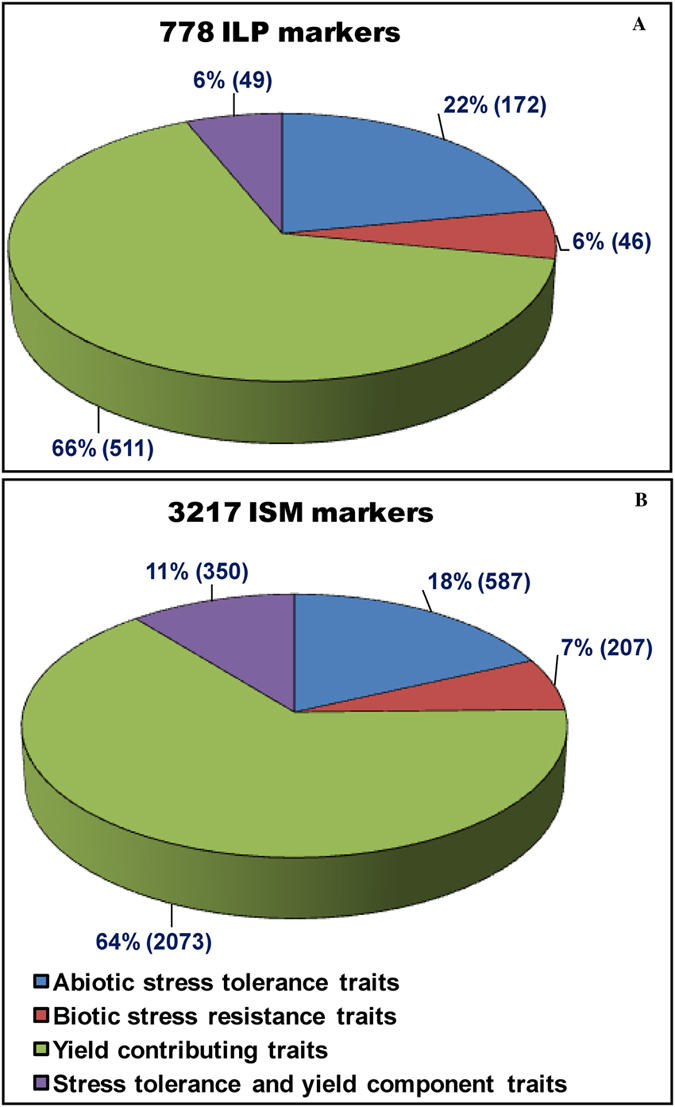
Proportionate distribution of 778 ILP (**A**) and 3217 ISM (**B**) markers designed from various known cloned genes that are functionally well-characterized for diverse agronomic traits in rice. The ILP (66%) and ISM (64%) markers derived particularly from multiple known cloned genes governing yield-contributing traits were abundant. Values in parentheses indicate the number of ILP and ISM markers. The detail information regarding known cloned gene-derived ISM and ILP markers are mentioned in the Tables S3 and S4.

Collectively, a random uneven genomic distribution of physically mapped ISM and ILP markers with regard to their occurrence and relative density across 12 rice chromosomes was evident. Nevertheless, a wider genome coverage of gene-derived ISM (226.8 ISMs/Mb) and ILP markers (44.2 ILPs/Mb) markers than multi-allelic SSR (356.7 genic SSRs/Mb) and InDel (25–30 genic InDels/Mb) markers that are physically mapped on 12 rice chromosomes was evident, which is also coherent with the previous report of Wang *et al*.^21^. Interestingly, we identified 3217 ISM and 778 ILP markers (Intronic InDel-based *in silico* fragment length polymorphism varied from 1 to 54 bp) from the introns of 620 and 415 known cloned genes (https://github.com/venyao/RICENCODE/blob/master/geneKeyword.table), respectively, that regulate diverse biotic and abiotic stress tolerance and yield component traits in rice (Tables S3 and S4, Fig. 2A,B). The designing of multiple ISM (4 ISM/gene) and ILP markers (2 ILP/gene) in individual genes will provide greater flexibility to user for selecting suitable primer combination exhibiting robust marker amplification (with an average amplicon product size of 464 bp) and higher functional allelic polymorphism among accessions in order to accelerate targeted mapping of genes and candidate gene-based association analysis for effective quantitative trait dissection and genetic enhancement in rice. The genome-wide well-distributed ISM and ILP markers, being derived from the diverse trait-regulating known cloned as well as functionally annotated candidate genes, could serve as an instant resource for establishing marker-trait association and identification/fine-mapping of genes/QTLs regulating important agronomic traits to accelerate marker-assisted selection in rice.

### High marker amplification and polymorphic potential

A set of 3217 ISM and 778 ILP markers from the 620 and 415 known cloned genes regulating stress tolerance and yield component traits as well as 1750 ILP markers (one marker per non-TE associated gene) revealing 10 to 101-bp InDel-based *in silico* fragment length polymorphism between Nipponbare and Kasalath genomes were selected. These ISM and ILP markers were experimentally validated using the agarose gel- and amplicon sequencing-based assays to further assess their amplification and polymorphic potential among 26 lowland/upland (*aus*) *indica, japonica*, long- and short-grained aromatics and wild rice accessions. All 5745, including 3217 ISM and 2528 ILP markers, were validated primarily using the genomic DNA of two rice accessions, namely Nipponbare and Kasalath, the genome sequences from which InDel markers were designed originally. Of these, 4629, including 2479 (77%) ISM and 2150 (85%) ILP markers amplified single reproducible PCR amplicons in 2.5% agarose gel with an average amplification success rate of 80.6%. Notably, the amplified 1329 (53.6%) ISM and 1723 (80.1%) ILP markers revealing *in silico* InDel-based fragment length polymorphism between Nipponbare and Kasalath were validated experimentally using agarose gel- and amplicon sequencing-based assays. Specifically, the validation and genotyping of ILP markers overall assured the correspondence of expected *in silico* fragment length polymorphism based on sum of InDels-size present in each intron of genes with their actual amplicon fragment size variation detected experimentally between Nipponbare and Kasalath rice accessions.

A selected set of 3052 polymorphic ISM and ILP markers, as above were genotyped in 26 rice accessions using agarose gel- and amplicon sequencing-based assays to assess their potential to detect polymorphism among these accessions. The markers genotyped overall detected 8243 alleles in 26 accessions with a mean PIC of 0.70. The number of alleles detected by the ILP markers (1 to 4 alleles with a mean 2.7 alleles per marker) among rice accessions was higher than that of ISM (1 to 2 alleles with a mean 1.6 alleles per marker). A higher polymorphic potential of markers was observed among the accessions belonging to long- and short-grained aromatics (2289 markers, 75% polymorphism and mean PIC: 0.64), followed by lowland/upland (*aus*) *indica* (2075, 70% and 0.61), wild (1709, 56% and 0.57) and *japonica* (1556, 51% and 0.52) rice accessions. The potential of markers to detect polymorphism between cultivated and wild (2412 markers, 79% polymorphism and mean PIC: 0.69) as well as between *indica* and *japonica* (2350, 77% and 0.67) rice was much higher as compared to that within cultivated (1984, 65% and 0.58) and wild rice. The degree of marker polymorphism estimated within and/or between *indica*, aromatics, *japonica* and wild rice is consistent with the previous documentation^41^,^42^,^43^,^44^,^45^,^46^. Remarkably, the polymorphic potential detected by the ISM (53%) and ILP (80%) markers among 26 lowland/upland (*aus*) *indica, japonica*, long- and short-grained aromatics and wild rice accessions is much higher than that observed especially with genome-wide ILP (~25%), RM (rice microsatellite) (18–24%), GNMS (genic non-coding microsatellite) (~32%) and highly-variable SSR (42–54%) markers^21^,^41^,^42^,^43^,^44^,^45^,^46^.

The potential of these markers in revealing higher average amplification success rate (80%) and polymorphic potential (66%) among domesticated rice accessions by a simpler cost-effective agarose gel-based assay suggest their immense use in multi-dimensional genomics-assisted breeding applications of rice. The efficient resolution and correspondence between *in silico* and experimental fragment length polymorphism showing ILP markers in gel- and amplicon sequencing-based assay deduce that the practical applicability of these markers for large-scale genotyping applications can be complemented with user-preference marker selection (selecting markers based on their predetermined intronic-InDel size) by optimal expense of resources in rice. Henceforth, the ISM and ILP markers with their simplicity in mining as well as robustness in large-scale genotyping and detecting functional allelic variation in the gene sequence components of diverse accessions could act as a preferred marker resource for high-throughput rice genetic analysis in laboratories with minimal infrastructural facilities.

### Wider functional molecular diversity and admixed population genetic structure assayed by rice ISM and ILP markers

**Figure 3:**
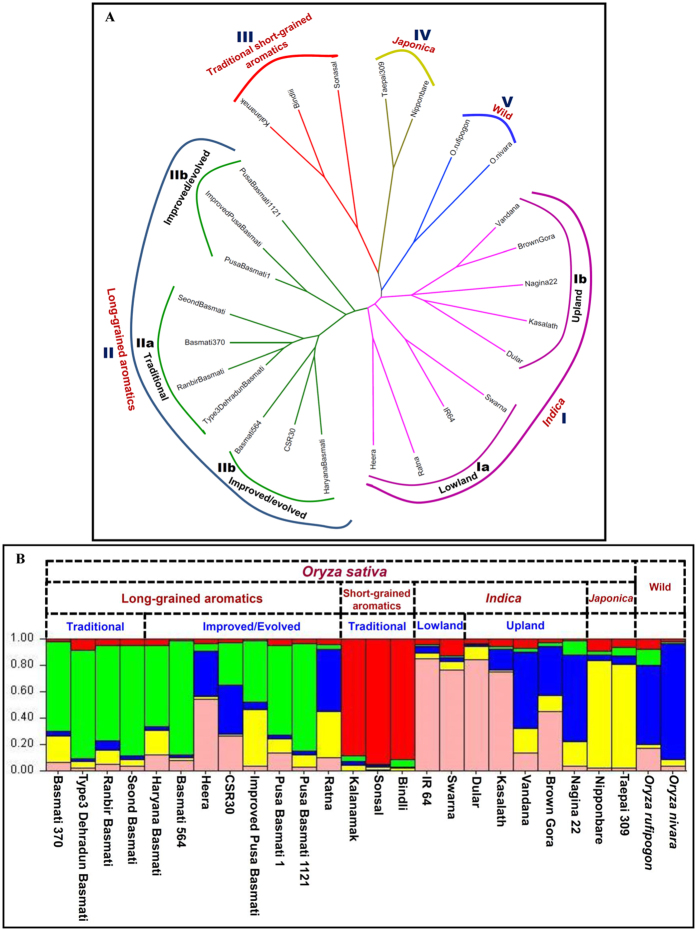
(**A**) Unrooted phylogenetic tree depicting the functional molecular diversity and evolutionary relationships among 26 rice accessions using 3052 informative gene-based ISM and ILP markers. All these accessions differentiated into five major groups- I: *indica* (Ia: lowland and Ib: upland/*aus*), II: long-grained aromatics (IIa: traditional and IIb: improved/evolved), III: traditional short-grained aromatics, IV: *japonica* and V: wild according to their known species/subspecies-specific origination, pedigree relationships and parentage. (**B**) Population genetic structure depicts best possible structure among 26 rice accessions using 3052 informative gene-based ISM and ILP markers. At optimal population number K = 5, these mapped markers grouped rice accessions into five major populations- *indica*, long- and short-grained aromatics, *japonica* and wild according to their known parentage and pedigree relationships. The accessions represented by vertical bars along the horizontal axis were categorized into K colour segments based on their estimated membership fraction in each K cluster. Five different colours signify five major population groups that correspond well with the clustering pattern as obtained by phylogenetic tree construction.

The estimation of genetic diversity level among 26 lowland/upland *indica* (*aus*), long- and short-grained aromatics, *japonica*, and wild rice accessions using 3052 polymorphic ISM and ILP markers (mapped on 12 rice chromosomes) revealed a wide range of diversity/distance coefficient from 0.17 to 0.79 with an average of 0.56. The diversity coefficient among 24 cultivated accessions varied from 0.21 to 0.73 with an average of 0.50. The phylogenetic relationship among 26 lowland/upland *indica* (*aus*), long- and short-grained aromatics, *japonica*, and wild rice accessions was determined and illustrated by an unrooted phylogenetic tree (Fig. 3A). The ISM and ILP markers clearly differentiated all these accessions from each other and clustered into five major groups, namely *indica* (I), long (II) and short (III)-grained aromatics, *japonica* (IV) and wild (V). The *indica* group I was further clustered into two sub-groups, lowland (Ia) and upland/*aus* (Ib) *indica*, whereas aromatics group II was grouped into traditional (IIa) and improved/evolved (IIb) long-grained Basmati.

The population genetic structure analysis among 26 rice accessions using 3052 polymorphic ISM and ILP markers with varying levels of K (K = 2 to 10) at 10 replications was performed. This revealed that at K value of 5, all the accessions were grouped majorly into five distinct populations, lowland (4 accessions) and upland/*aus indica* (5), long-grained aromatics (10) and short-grained aromatics (3), *japonica* (2) and wild (2) (Fig. 3B). The high resolution population groupings corresponded well with expected pedigree relationships and parentage. It was further comparable with the clustering pattern as detected among 26 rice accessions by the neighbor-joining tree analysis using pair-wise genetic distances (Fig. 3A). The estimation of molecular genetic variation among and within six populations using 3052 informative ISM and ILP markers detected a wider level of quantitative genetic differentiation (F_ST_: 0.11 to 0.61 with an average of 0.43) among these population groups. A higher frequency of F_ST_ and thereby molecular diversity between population groups (F_ST_ 0.36) as compared to that within populations (0.27) was observed. Higher allelic diversity was observed among the accessions belonging to aromatic population (0.21) than that of *indica, japonica* and wild population groups. This could be due to inclusion of much diverse traditional (selection from landraces) and improved/evolved high-yielding Basmati accessions (developed through cross-breeding between traditional Basmati and non-Basmati *indica*) in the aromatic rice population group^41^,^45^. All the 26 accessions clearly belonged to a single population in which about 71.3% of their inferred ancestry was derived from one of the model-based population and remaining ~28.7% contained admixed ancestry. This is possibly due to cumulative effects of strong adaptive selection pressure and complex breeding efforts involving inter-crossing and introgression among accessions representing diverse species/sub-species and population groups during their divergence from wild progenitors and subsequent domestication^24^,^43^,^44^,^47^,^48^. Maximum admixture was observed between *indica* and wild population groups (~12%), followed between long- and short-grained aromatics and *japonica* (~9%) populations (Fig. 3B). This suggests more evolutionary closeness of *indica* with wild population and *japonica* with aromatics, which is coherent with studies involving genome-wide SSR and SNP markers^24^,^41^,^42^,^43^,^44^,^45^,^47^,^48^.

The level of molecular diversity (17 to 79%) and F_ST_ (11 to 61%) estimated among rice accessions using the ISM and ILP markers is comparatively much higher than that estimated previously with the genome/gene-derived SSR and ILP markers^21^,^41^,^42^,^45^,^46^. The observed phylogenetic relationship and population genetic structure among *indica*, aromatic, *aus, japonica* and wild rice accessions is consistent with the earlier documentation and also corresponded well with their known population (species/sub-species)-specific origination, pedigree relationships and parentage^24^,^41^,^42^,^43^,^44^,^45^,^47^,^48^. A distinct differentiation between long (traditional and improved/evolved) and traditional short-grained aromatic population groups assayed by ISM and ILP markers suggests their utility in defining varietal identity in Basmati trade and commerce. A higher potential of ISM and ILP markers for assaying realistic estimation of functional molecular diversity, phylogenetics and population genetic structure pattern at genome/gene level infers their significance in establishing distinctness among rice accessions belonging to different *indica*, aromatic, *aus, japonica*, and wild population groups and thus could be employed in genomics-assisted varietal improvement of rice. Specifically, these markers assaying functional allelic variation and diversity in the gene regions of the genome might be directly associated with phenotypic trait variation through genetic association mapping and thereby could be deployed efficiently in selection of desirable cultivar types and trait-associated molecular tags for rice crop improvement. Other than commonly utilized random microsatellite and RAPD (random amplified polymorphic DNA) markers in hybridity assessment, the genic ISM and ILP markers could be useful in improving the predictability of hybrid performance in rice.

In an autogamous crop species like rice, the phenotypic selection for recurrent parent phenotype and genotyping of randomly distributed genome-wide markers in small size back-cross mapping population are commonly adopted to recover recurrent parent genome in background selection. These random markers often detect recurrent parent identity in the non-coding region rather than the coding region due to the fact that bulk of genome mostly consists of the non-coding sequence components. However, any recovery of recurrent parent genome in non-coding region is of less consequence as far as phenotype is concerned. This could be improved if more markers, developed from the coding/non-coding but genic sequence components of genome, are employed for background selection. In this context, generation of informative ISM and ILP markers from the rice genes at a genome-wide scale assumes great significance.

### Construction of a high-density ISM/ILP marker-based genetic linkage map

**Figure 4:**
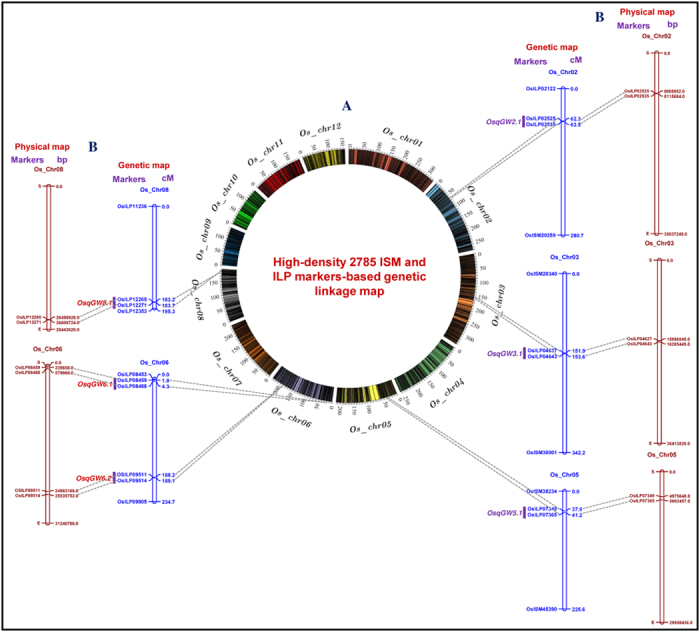
(**A**) A high-density genetic linkage map (IR 64 x Sonasal), generated by anchoring 2785 known cloned/candidate genes-derived ISM and ILP markers on 12 rice chromosomes, is illustrated in a Circos circular ideogram. The outer circle signifies the diverse genetic map length (cM) (spanning 50 cM uniform genetic distance intervals between bins) of 12 chromosomes coded with multiple colours. **(B)** The integration of genetic and physical maps delineated six candidate genes with ILP markers at six major genomic regions harbouring grain weight QTL mapped on five rice chromosomes 2, 3, 5, 6 and 8. The genetic (cM)/physical (bp) distance and identities of markers mapped on chromosomes are denoted on the right and left side of the chromosomes, respectively. The detail information regarding ISM and ILP markers and major grain weight QTLs are provided in the Tables S1,S2 and Table 2.

To construct a high-resolution genetic linkage map, 2785 markers (including 620 ISM and 415 ILP markers from known/cloned and functionally characterized rice genes (https://github.com/venyao/RICENCODE/blob/master/geneKeyword.table) and 1750 random ILP markers, Tables S3,S4 and S5) revealing polymorphism between two parental accessions (*indica* variety, IR 64 and short-grained aromatic landrace, Sonasal) were genotyped among 150 individuals of a F_3_ mapping population (IR 64 x Sonasal). The co-dominant inheritance of parental polymorphic ISM and ILP markers across segregating mapping individuals and their subsequent linkage analysis led to map 2785 markers across 12 chromosomes of a constructed rice genetic map (Fig. 4A, Table 1). The genetic map spanned a total map length of 2730.2 cM with an average inter-marker distance of 0.98 cM. Highest number of markers were mapped on chromosome 1 (398 markers), followed by chromosomes 3 (377) and 2 (298) and lowest on chromosome 10 (130) (Fig. 4A, Table 1). Longest and shortest map length spanning 342.2 and 141.0 cM were obtained in chromosomes 3 and 9, respectively. Chromosomes 1 (mean inter-marker distance: 0.84 cM) and 12 (1.16 cM) had most and least saturated genetic maps, respectively (Fig. 4A, Table 1).

The co-dominant inheritance of genic ISM and ILP markers in discriminating the homozygous and heterozygous mapping individuals implicate the wider practical applicability of these developed genome-wide markers for generation of high-resolution genetic linkage maps and efficient molecular mapping of QTLs. A 2785 ISM and ILP marker-based high-density (inter-marker distance of 0.98 cM) genetic linkage map (IR 64 x Sonasal) constructed by us is comparable/highly saturated than that documented yet in diverse *indica* and aromatics mapping population-led genetic maps of rice^13^,^14^,^22^,^49^,^50^,^51^,^52^,^53^,^54^,^55^,^56^,^57^,^58^,^59^. Despite high-density, the constructed genetic linkage map (IR 64 x Sonasal) (2730.2 cM) covered a much higher total map-length than that of RGP (Rice genome research program) genetic maps (most commonly *indica* x *japonica* inter-crosses used) (http://rgp.dna.affrc.go.jp/) (1500–2000 cM). This is possibly due to varied impact of population-specific genetic inheritance pattern, which especially rely upon the genetic constitution of parental accessions used to develop mapping populations contrasting for agronomic traits between the past and present studies. To delineate such effects and determine the possible cause of higher map length covered in our constructed genetic linkage map, the respective Indian *indica* and aromatic rice population-led genetic map (IR 64 x Sonasal) was compared with that of multiple genomic and genic SSR markers-based genetic linkage maps documented earlier involving similar genetic backgrounds of Indian *indica* and aromatic rice accessions^49^,^50^,^56^,^57^. In contrast to above-mentioned previous genetic linkage maps (2000–2500 cM), a slightly higher total map-length (2730.2 cM) in our presently constructed genetic map was observed. This could be due to exclusive use of a large-scale gene-based ISM and ILP markers that are sparsely distributed on the rice genome for genotyping of parents and segregating individuals of a mapping population in our study to construct the genetic linkage map. Summarily, the high-density genetic linkage map constructed in this study has potential to be utilized as a reference for rapid molecular mapping of high-resolution QTLs/genes governing diverse agronomic traits in rice.

### Molecular mapping of grain weight QTLs

We observed a significant phenotypic variation for grain weight (1000-grain weight: 8 to 31 g with 85% H^2^) trait in 150 mapping individuals (IR 64 x Sonasal) and two parental accessions. A normal frequency distribution, including a bi-directional transgressive segregation of grain weight trait in mapping individuals and parental accessions was evident. The two years multi-location field phenotyping data of grain weight and genotyping information of 2785 ISM and ILP markers genetically mapped on 12 rice chromosomes were integrated for molecular mapping of QTLs. This analysis identified six major genomic regions harbouring six QTLs associated with grain weight that were mapped on five rice chromosomes (2, 3, 5, 6 and 8) (Fig. 4B, Table 2). A maximum number of two major grain weight QTLs was mapped on chromosome 6. The individual major QTL explained 11.9 to 21.6% phenotypic variation (R^2^) for grain weight trait at 9.8–13.7 LOD. The PVE estimated for all six major QTLs in combination was 27.5%. Six major genomic regions underlying these grain weight QTLs spanned (0.5 cM on chromosome 8 to 3.3 cM on chromosome 5) by 20 ISM and ILP markers, were mapped on six different genomic regions on five chromosomes (Fig. 4B, Table 2). In all six major grain weight QTLs, the positive additive gene effect (3.0–4.3) of grain weight with major allelic contribution from a high grain weight parental rice accession IR 64 was observed.

The integration of genetic and physical maps detected six genes with ILP markers tightly linked to six major QTLs regulating grain weight (single marker analysis-based QTL mapping) in rice (Fig. 4B, Table 2). These gene-based markers thus have potential to be deployed in marker-assisted genetic enhancement for increasing grain weight and yield of rice. To assure the validity and robustness of grain weight QTLs identified in our study, the major genomic regions harbouring six grain weight QTLs were compared with that documented by previous QTL mapping studies utilizing multiple *indica* and aromatic rice-derived mapping populations. The correspondence of two major grain weight QTLs (*OsqGW3.1* and *OsqGW8.1*) with previously reported two known major QTLs that harbour two cloned and functionally characterized genes (*GS3* and *GW8*) governing grain size/weight based on their congruent physical positions on rice chromosomes was observed^9^,^13^,^14^,^60^,^61^. This implicates that four grain weight QTLs identified by us are novel and possibly exhibit population-specific genomic distribution. The genes with ISM and ILP markers tightly linked with the major grain weight QTLs mapped on chromosomes could be utilized in genomics-assisted crop improvement for developing rice varieties with higher grain size/weight and yield.

### A higher potential of genic ISM/ILP markers for precise differential gene expression profiling

**Figure 5:**
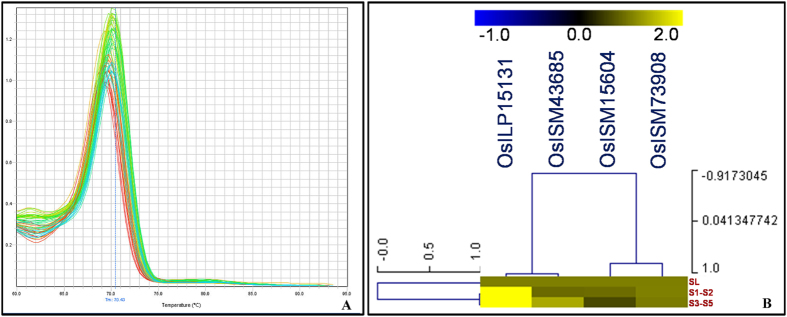
(**A**) Melting-curve analysis of one representative *HAP* gene with ILP marker in quantitative RT-PCR assay using the cDNA-pools of seedlings and two seed developmental stages of IR 64 (at least two biological replicates) produces single peak as desired, confirming the efficacy of ILP marker to amplify single gene-specific PCR product of accurate fragment size. (**B**) Hierarchical cluster display illustrated the differential expression profile of four *HAP* genes with ISM and ILP markers in seedling (SL) and two seed developmental stages (S1–S2: early cell division and S3–S5: late maturation) of one rice accession IR 64. The blue, black and yellow colour scale (mentioned at the top) signify the low, medium and high level of average log signal expression values of genes in various tissues/stages, respectively. The expression values across diverse tissues/development stages of accession were normalized using an endogenous control *Actin1* in RT-PCR assay. The differential expression profiling of genes in two seed developmental stages of IR 64 was compared with their respective vegetative seedling tissue by assigning the gene expression in this tissue as a reference calibrator 1. The detail structural and functional annotation four rice *HAP* genes with markers are mentioned in the Tables S1 and S2. The genes with marker and tissues/stages used for expression profiling are indicated on the top and right side of an expression map, respectively.

To assess the potential of developed genic ISM and ILP markers for accurate assaying of differential expression pattern of genes (from which these markers are derived), semi-quantitative and quantitative RT-PCR assays were performed using the RNA isolated from seedling (control) and two (early cell division S1–S2 and late maturation S3–S5) seed developmental stages of IR 64. A number of *HAP* family genes annotated from whole rice genome have been functionally well characterized and are known to be involved specifically in transcriptional regulation of growth and development, including seed development and grain size/weight variation in rice^62^,^63^,^64^,^65^. Since, our study majorly concerned on identification and genetic mapping of ISM and ILP markers associated with grain weight QTLs in rice, we selected ISM and ILP markers designed specifically from developmental traits-regulating candidate *HAP* genes for differential expression profiling. For this, 13 ISM/ILP markers designed from each of 13 rice *HAP* genes with single spliced form were selected initially for their differential expression profiling in seedling and two seed developmental stages of IR 64 using semi-quantitative RT-PCR assay (Tables S1 and S2). Four of these, 13 *HAP* genes with markers showed high seed-specific expression in IR 64, which is further agreed well with expression pattern of these genes assayed in similar rice accession, as documented by Rice Oligonucleotide Array Database (http://www.ricearray.org). The four *HAP* genes-derived ISM/ILP markers were selected subsequently for expression profiling using the three cDNA samples of afore-mentioned tissues/stages of IR 64 by semi-quantitative and quantitative RT-PCR assays. The semi-quantitative RT-PCR assay using the ISM/ILP markers derived from four *HAP* genes amplified single reproducible and specific PCR amplicons of expected product size across all three cDNA samples in agarose gel. The quantitative RT-PCR assay of these four *HAP* genes-derived ISM/ILP markers using the cDNA of seedlings and two seed developmental stages of IR 64 (at least two biological replicates) with no template control amplified single gene-specific PCR product of desired fragment size, which were further confirmed through single peak-led melting-curve analysis of individual genes (Fig. 5A). The amplification curves and cycle threshold (C_T_) of all gene-based markers across all biological replicates of seedling and two seed developmental stages were measured and compared. All the four *HAP* genes with ISM/ILP markers showed differential up-regulated expression pattern in two seed developmental stages as compared to control vegetative seedling tissue of IR 64 (Fig. 5B). This is consistent with the differential expression pattern of these genes assayed in similar developmental stages/tissues of rice accession IR 64 through global microarray profiling (Rice Oligonucleotide Array Database). Interestingly, one ILP marker-derived from *HAP* gene showing InDel-based *in silico* fragment length polymorphism (3-bp) among rice accessions revealed pronounced differential expression (>10-fold up-regulation) of corresponding *HAP* gene in two seed developmental stages as compared to control seedling tissue of IR 64 (Fig. 5B).

Therefore, the ISM and ILP markers developed targeting the introns of genes have potential to serve as a resource for accurate and robust profiling of differential expression pattern of genes in diverse developmental stages/tissues of rice accessions at a genome-wide scale. The development of multiple ISM and ILP markers from individual genes provides flexibility to users for selecting desirable primer combination for robust amplification and realistic estimation of differential expression pattern of genes especially in RT-PCR assay. The potential of these markers for simultaneous assaying of both DNA-based large-scale genotyping, allelic diversity and expression profiling precisely in a diverse array of rice accessions was apparent. This useful genetic attribute of markers will further expedite the molecular mapping of QTLs/eQTLs (expression QTLs) and differentially expressed genes governing diverse agronomic traits as well as rapid delineation of trait-associated molecular tags at whole genome level for marker-assisted genetic enhancement in rice.

### Attributes and efficacy of rice ISM and ILP marker database

**Figure 6:**
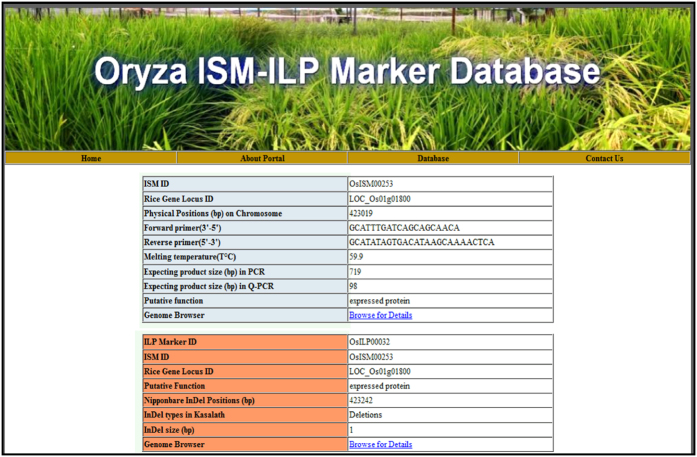
Snapshot illustrating the features and utilities of different interfaces included in a public web-resource “*Oryza ISM-ILP* marker” database. The snapshot was selected from database webpages developed.

In order to facilitate unrestricted access to the ISM and ILP markers developed from the whole genome of rice, an online marker database portal named as “*Oryza ISM-ILP* marker” database was developed for querying and visualization of marker information. The whole application of this database is to provide an elegant and web-based interface containing three types of search options *viz.*, Search by Marker ID, Search by gene locus ID, Search by gene function. Currently, the database contains the information on 84634 ISM and 16510 ILP markers along with their gene annotation, forward/reverse primer sequences, expected PCR product size (bp) and InDel characteristics in the ISM marker regions. Moreover, the search results can be displayed in tabular format along with hyperlinks to genome browser for visualization/download of the ISM and ILP markers designed from gene sequence components of rice genome (rice genome annotation database, MSU release 7.0, http://rice.plantbiology.msu.edu). The database is publicly available via the Internet using web-links: http://webapp.cabgrid.res.in/ismdb/ or http://bioinformatics.iasri.res.in/ismdb/. The snapshot of the Rice ISM and ILP (*Oryza ISM-ILP*) marker database has been provided in the Fig. 6.

The added-advantage of our present investigation over previous study of Wang *et al*.^21^ especially with regard to strategy adopted for designing and broader use of genome-wide ISM and ILP markers in accelerating genomics-assisted breeding applications of rice is evident. This majorly includes designing of multiple ISM and ILP markers with dense genome-wide coverage involving all the intronic sequence components of genes that are structurally and functionally annotated recently from the high-quality gold standard completely sequenced whole rice genomes/pseudomolecules. Consequently, our strategy provides user a wider flexibility to screen diverse combination of informative ISM and ILP markers from an individual gene exhibiting reproducible amplification (80% efficiency) as well as higher polymorphic potential (66%) for discrimination of domesticated accessions effectively to expedite marker-assisted breeding in rice. The utility of genic ISM and ILP markers over other sequence-based markers in terms of their simplicity in discovery/designing and higher potential to detect functional allelic polymorphism among accessions in the gene regions of genome even by cost-effective agarose gel-based assay was apparent. In conjunction, these markers also have significance for assaying wider functional molecular diversity and realistic estimation of admixed population-specific genetic structure in diverse rice population as well as suitability for construction of high-density genetic linkage map and molecular mapping of high-resolution grain weight QTLs in rice. Therefore, unrestricted access of these informative gene-based markers mapped on 12 chromosomes has been made available to the rice scientific community through a user-friendly public web-resource, “*Oryza ISM-ILP* marker” database, with an aim to accelerate multi-dimensional high-throughput genetic analysis, including assessment of hybrid performance and marker-assisted background analysis for genetic enhancement in rice.

## Methods

### ISM and ILP markers designing

**Figure 7:**
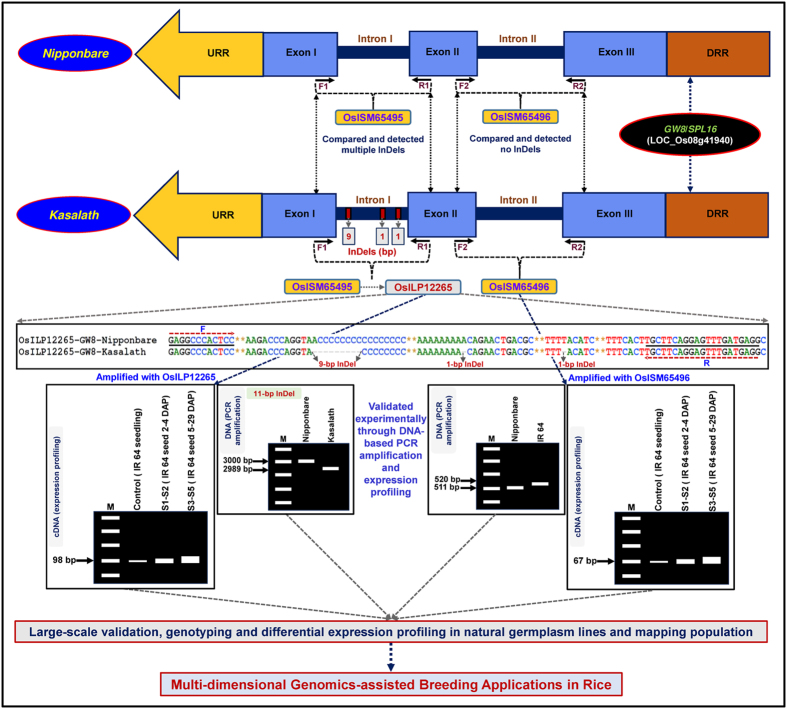
Schematic depicting the key steps followed for successful discovery, large-scale validation and high-throughput genotyping of ISM and ILP markers derived from diverse intronic sequence components of grain weight-regulating known cloned gene as exemplified by *GW8*/*SPL16* (annotated from rice genome), to be utilized for multi-dimensional genomics-assisted breeding applications in rice. The Forward (F) and Reverse (R) primers designed from the exonic sequences flanking the introns (without any InDels) and intronic-InDels were developed as ISM and ILP markers, respectively. URR: Upstream regulatory region and DRR: downstream regulatory region. The identities of ISM and ILP markers with their detailed information are mentioned in the Tables S1 and S2.

For designing ISM and ILP markers at a genome-wide scale, the individual intronic sequence components of each rice gene [transposable element (TE)- and non TE-related gene models] (rice genome annotation database, release 7, http://rice.plantbiology.msu.edu) annotated from completely sequenced *japonica* (Nipponbare) rice genome were retrieved. The forward and reverse primer-pairs from 100-bp exonic sequences flanking each intron were designed individually using custom-made Primer3 perl scripts to develop ISM. For converting ISM into ILP markers based on InDels, the individual intronic sequences of each rice gene annotated from Nipponbare genome were compared with corresponding homologous (*E*-value: 0 and bit score ≥500) genomic sequences of recently sequenced Kasalath (upland *indica*/*aus*) genome^66^ (http://rice50ks.dna.affrc.go.jp/) and intronic-InDels were detected between Nipponbare and Kasalath genomes. The ISM primers designed targeting those introns of genes with single and/or multiple intronic-InDels between Nipponbare and Kasalath genomes, were also considered as primers for corresponding ILP markers. The uniqueness of primers designed both for ISM and ILP markers in the rice genome was assured following the methods of Wang *et al*.^21^. The genomic distribution of ISM and ILP markers in diverse known cloned as well as candidate TE and non-TE associated rice genes that were structurally and functionally annotated on 12 rice chromosomes, was determined. To assess the potential of ISM and ILP markers for precise measurement of differential expression profiling of genes (from which these markers were derived), primer-pairs were designed from 100-bp flanking exonic sequences of introns in such a way that the markers should amplify 60–100 bp amplicon product size in the cDNA of rice accessions used. The major strategies adopted to develop ISM and ILP markers from the introns of genes annotated on the rice genome, to be effectively deployed for large-scale genotyping- and expression profiling-based applications are depicted in the Fig. 7.

### Experimental validation, marker amplification and polymorphic potential

Twenty-six rice accessions representing lowland/upland *aus*/*indica* (9 accessions), *japonica* (2), long (10)- and short (3)-grained aromatics, and wild (2) were utilized for genomic DNA isolation. To determine the amplification and polymorphic potential of markers, ISM and ILP markers designed from various known cloned and functionally characterized genes regulating stress tolerance and yield component traits in rice were selected. In addition, ILP markers derived from various non TE-associated genes (one marker/gene) revealing ≥10-bp InDel-based *in silico* fragment length polymorphism between Nipponbare and Kasalath genomes, and physically mapped on 12 rice chromosomes, were screened. These ISM and ILP markers were PCR amplified with the genomic DNA of rice accessions using standard PCR constituents and touchdown thermal cycling profiling as per Jhanwar *et al*.^67^ and Kujur *et al*.^68^. The PCR products of amplified ISM as well as ILP markers exhibiting 10-bp *in silico* fragment length polymorphism between Nipponbare and Kasalath genomes were resolved in 2.5% agarose gel and their fragment size (bp) was determined against 50-bp DNA ladder size standard. The PCR products of amplified ISM and ILP markers revealing 2 to 9-bp *in silico* fragment length polymorphism was purified and sequenced employing automated 96 capillary ABI 3730xl DNA Analyzer (Applied Biosystems, USA) following Kujur *et al*.^68^ and Saxena *et al*.^69^. The genotyping data of experimentally validated ISM and ILP markers was analysed employing PowerMarker v3.51^70^ to estimate the average polymorphic alleles per marker, per cent polymorphism and polymorphism information content (PIC) among rice accessions.

### Functional molecular diversity and population genetic structure

The validated polymorphic ISM and ILP markers (physically mapped across 12 rice chromosomes) were utilized to determine the molecular diversity, population structure and phylogenetic relationships among 26 rice accessions. The marker genotyping data were analyzed by Nei and Li similarity coefficient-based neighbor joining (NJ) method (with 1000 bootstrap replicates) of PowerMarker v3.51 for clustering analysis and construction of unrooted phylogenetic tree among accessions. To determine the population structure among 26 rice accessions, the marker genotyping data was analyzed in STRUCTURE^71^ using the admixture and correlated allele frequency with varying levels of K (number of populations) = 2 to 10 (burn-in of 50000 iterations, run length of 100000 and 20 independent replications of K). The population structure model representing better relationship among accessions was constructed and various population genetic parameters, including genetic variability (F_ST_) and degree of admixture within and between population groups at the optimum K was estimated.

### Genetic linkage map construction and QTL mapping

The ISM and ILP markers showing polymorphism between high (IR 64 with 1000-grain weight: 25 g) and low (Sonasal: 10 g) grain weight parental accessions of a F_3_ mapping population (IR 64 x Sonasal) were screened from our marker polymorphism study. These informative markers were PCR amplified and genotyped using the genomic DNA of 150 F_3_ mapping individuals and two parental accessions (IR 64 and Sonasal) following aforesaid agarose gel- and amplicon sequencing-based genotyping methods. The χ^2^-test (p < 0.05) of marker genotyping data was performed to screen their goodness-of-fit to the expected Mendelian 1:1 segregation ratio. The MAPMAKER/EXP 3.0^72^ and JoinMap 4.1 (http://www.kyazma.nl/index.php/mc.JoinMap) at higher LOD threshold (4.0) with Kosambi mapping function were deployed to measure linkage analysis among the markers used. The markers were incorporated into defined linkage groups (LGs) according to their centiMorgan (cM) genetic distances and corresponding marker physical positions (bp) on the chromosomes. A high-density genetic map was finally constructed and visualized using MapChart v2.2.

The 150 individuals and parental accessions of a F_3_ mapping population (IR 64 x Sonasal) were grown in the field at least for two consecutive years during the crop growing season and phenotyped for 1000-grain weight (g). The frequency distribution, coefficient of variation (CV) and broad-sense heritability (H^2^) of grain weight were determined in the mapping population following Bajaj *et al*.^73^. For molecular mapping of major grain weight QTLs, the genotyping data of ISM and ILP markers genetically mapped on 12 rice chromosomes were integrated with 1000-grain weight field phenotypic data of 150 mapping individuals and parental accessions using composite interval mapping (CIM) function (LOD threshold score >4.0 at 1000 permutations and p < 0.05 significance) of QTL Cartographer v2.5 and MapQTL 6. The additive effect and phenotypic variation explained (PVE) by each major grain weight QTL at significant LOD were estimated as per Bajaj *et al*.^73^.

### Expression profiling

The total RNA was isolated from vegetative 7-day-old seedlings (considered as control) and two different seed developmental stages [S1–S2: early cell division and organ initiation phase occurring 0–4 days after pollination (DAP) and S3–S5: maturation phase occurring 5–29 DAP, defined as per Agarwal *et al*.^74^)] of one *indica* rice accession (IR 64) following a modified protocol of Singh *et al*.^75^. The isolated RNA was purified using RNeasy MinElute Cleanup Kit (QIAGEN, USA), DNase (QIAGEN, USA) digested and tested high-quality purified RNA for quality on NanoDrop 2000c Spectrophotometer (NanoDrop products, USA). At least two biological replicates of each sample were used for cDNA synthesis as mentioned previously^76^. The cDNA was amplified with ISM and/or ILP marker primers designed from selected rice *HAP* (heme activator protein) genes using the semi-quantitative and quantitative RT-PCR assays. The 1X Fast SYBR Green Master Mix (Applied Biosystems, USA) along with 250 nM of forward and reverse ISM/ILP primers and 10 ng of each cDNA (1:10 dilution) in a total reaction volume of 10 μl were used for quantitative RT-PCR assay in 7500 Fast Real-Time PCR system (Applied Biosystems). *Actin1* gene was used as an internal control for normalization. Relative expression level of genes with markers in different seed developmental stages was ensured by comparative Ct (2^−ΔΔCt^) method. A heat map illustrating the differential expression profiles of *HAP* genes with ISM/ILP marker was constructed using the TIGR MultiExperiment Viewer (MeV, http://www.tm4.org/mev).

### Construction of ISM and ILP marker database

The rice ISM and ILP marker database (*Oryza ISM-ILP* marker) is an online user-friendly web resource developed using MySQL ver. 5.6.12 (www.mysql.com) at back end and PHP ver. 5.4.16 (www.php.net) at front end. This database serves as a repository for all ISM and ILP markers designed from the genes annotated from whole genome of rice in our study. This web application has been developed on three-layered architecture as illustrated in Fig. S3. We have hosted this database currently on a Linux operating system based HP Server with Intel Xeon quad core processors and 256 GB of random access memory. The online database is compatible with various commonly used browsers like Chrome and Firefox.

## Additional Information

**How to cite this article**: Badoni, S. *et al*. Genome-wide generation and use of informative intron-spanning and intron-length polymorphism markers for high-throughput genetic analysis in rice. *Sci. Rep.* **6**, 23765; doi: 10.1038/srep23765 (2016).

## Supplementary Material

Supplementary Information

## Figures and Tables

**Table 1:** Characteristics of a high-density genetic linkage map constructed using a 150 F_3_ rice mapping population (IR 64 x Sonasal).

Linkage groups (LGs)/chromosomes	ILP (known genes + random) markers + ISM mapped	Map length covered (cM)	Mean inter-marker distance (cM)
*Os_Chr01*	(102 + 66) + 230 = 398	334.8	0.84
*Os_Chr02*	(68 + 46) + 184 = 298	280.7	0.94
*Os_Chr03*	(95 + 62) + 220 = 377	342.2	0.91
*Os_Chr04*	(54 + 37) + 146 = 237	256.4	1.08
*Os_Chr05*	(52 + 36) + 136 = 224	225.6	1.01
*Os_Chr06*	(52 + 38) + 140 = 230	234.7	1.02
*Os_Chr07*	(52 + 37) + 155 = 244	231.8	0.95
*Os_Chr08*	(44 + 26) + 123 = 193	195.3	1.01
*Os_Chr09*	(33 + 23) + 101 = 157	141	0.90
*Os_Chr10*	(23 + 16) + 91 = 130	146.4	1.13
*Os_Chr11*	(24 + 15) + 123 = 162	184.4	1.14
*Os_Chr12*	(21 + 13) + 101 = 135	156.9	1.16
Total	(620 + 415) + 1750 = 2785	2730.2	0.98

**Table 2:** Molecular mapping of significant QTLs associated with grain weight in rice.

QTLs	LGs/chromosomes	Marker intervals with genetic positions (cM)	QTL physical intervals (bp)	Markers with genes tightly linked with QTLs	Protein-encoding genes	LOD	PVE (R^2^%)	A
*OsqGW2.1*	*Os_Chr02*	OsILP02525 (62.3)–OsILP02535 (63.5)	OsILP02525 (8008952)–OsILP02535 (8115684)	OsILP02535	Expressed protein	12.9	19.5	3.8
*OsqGW3.1*	*Os_Chr03*	OsILP04627 (151.9)–OsILP04643 (153.6)	OsILP04627 (15986844)–OsILP04643 (16285445)	OsILP04643	Protein kinase	10.8	13.7	2.9
*OsqGW5.1*	*Os_Chr05*	OsILP07349 (37.9)–OsILP07365 (41.2)	OsILP07349 (4975648)–OsILP07365 (5602457)	OsILP07365	Homeobox TF	11.4	16.2	3.2
*OsqGW6.1*	*Os_Chr06*	OsILP08459 (1.76)–OsILP08488 (4.25)	OsILP08459 (239858)–OsILP08488 (579980)	OsILP08488	Cytochrome P450	10.2	12.9	3.0
*OsqGW6.2*	*Os_Chr06*	OsILP09511 (188.2)–OsILP09514 (189.1)	OsILP09511 (24983167)–OsILP09514 (25535752)	OsILP09514	*bZIP* TF	9.8	11.9	3.5
*OsqGW8.1*	*Os_Chr08*	OsILP12265 (183.2)–OsILP12271 (183.7)	OsILP12265 (26498929)–OsILP12271 (26600724)	OsILP12265	*SBP* TF	13.7	21.6	4.3

^*^OsqGW2.1 (*Oryza sativa* QTL for grain weight on chromosome 2 number 1), PVE: Percentage of phenotypic variation explained by QTLs, A: Additive effect; positive additive effect infers alleles from IR 64 with gain weight values. Details regarding ILP markers are mentioned in the Table S2. TF: Transcription factor.
