# Supplementary material for: Genome-wide generation and use of informative intron-spanning and intron-length polymorphism markers for high-throughput genetic analysis in rice
Source: Sci Rep. 2016 Apr 1;6:23765. doi: 10.1038/srep23765 (PMC4817136; doi:10.1038/srep23765)
Supplement: Supplementary Information [file srep23765-s1.pdf]

Genome-wide generation and use of informative intron-spanning and intron-length polymorphism markers for high-throughput genetic analysis in rice

Saurabh Badoni[1], Sweta Das[1], Yogesh K. Sayal[2], S. Gopalakrishnan [3], Ashok K. Singh[3], Atmakuri R. Rao[2], Pinky Agarwal[1], Swarup K. Parida[1*], Akhilesh K. Tyagi[1*]

[1]National Institute of Plant Genome Research (NIPGR), Aruna Asaf Ali Marg, New Delhi 110067, India

[2]Centre for Agricultural Bioinformatics, Indian Council of Agricultural Research (ICAR)-Indian Agricultural Statistics Research Institute, New Delhi 110012, India

[3]Division of Genetics, Rice Section, Indian Agricultural Research Institute (IARI), New Delhi 110012, India

*Corresponding authors

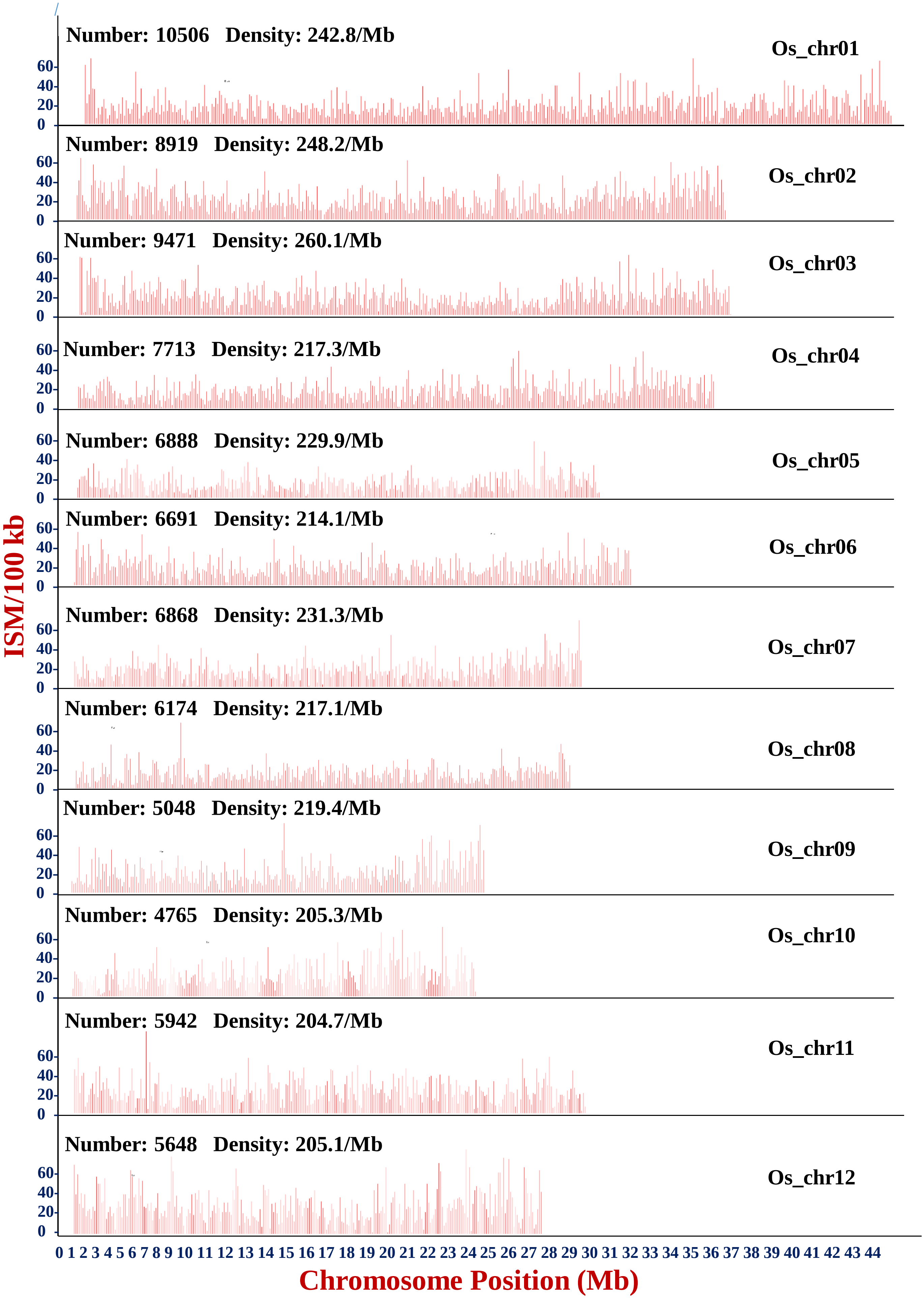

Figure S1: A genome scan plot illustrating the density of 84634 ISM physically mapped across 12 rice chromosomes at 100-kb sliding window. The SNP density is defined as number of SNPs mapped within 1 Mb physical interval of chromosomes.

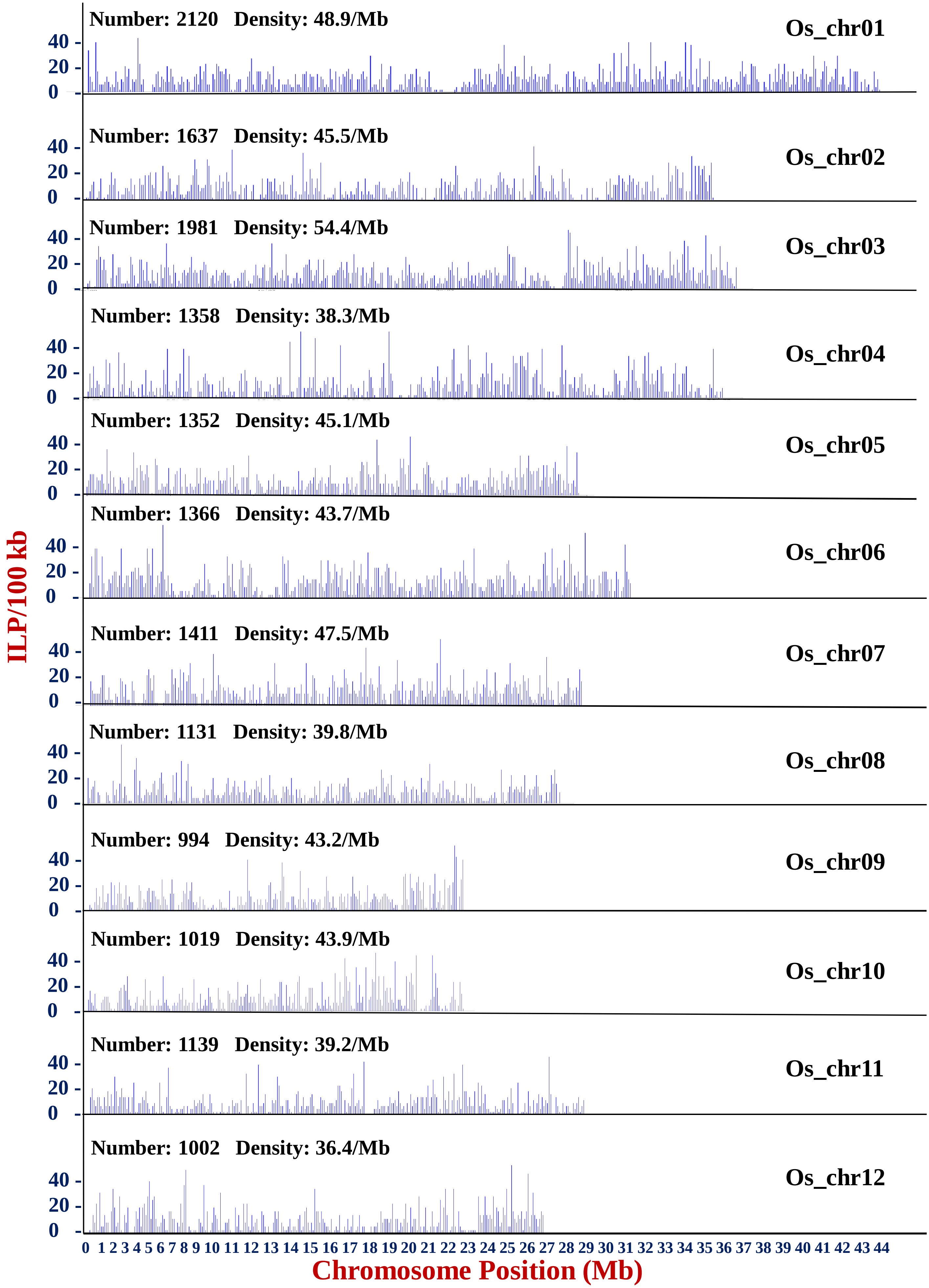

Figure S2: A genome scan plot illustrating the density of 16510 ILP markers physically mapped across 12 rice chromosomes at 100 kb sliding window. The SNP density is defined as number of SNPs mapped within 1 Mb physical interval of chromosomes.

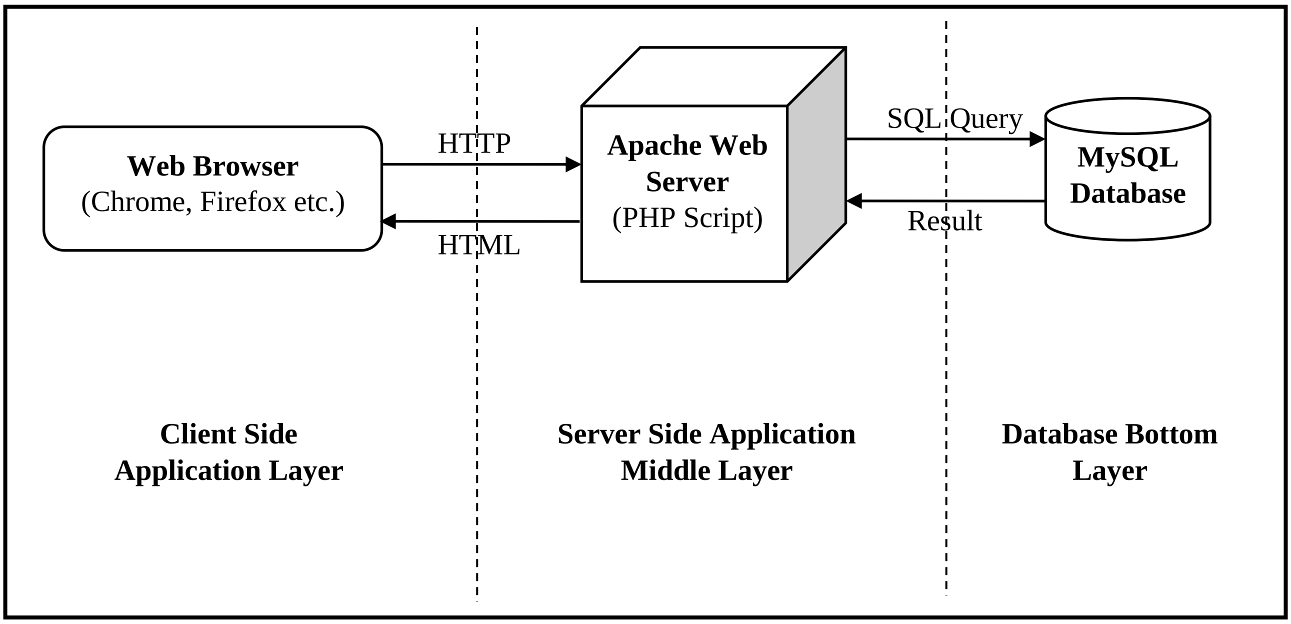

Figure S3. Three-layered architecture-based web application used for constructing rice ISM and ILP marker database (*Oryza ISM-ILP* marker).

Table S1: Genome-wide 84634 ISM markers developed from the introns of genes for large-scale genotyping applications in rice

ISM IDs	Rice gene locus IDs	Physical positions (bp) on chromosomes	Forward primers (5'-3')	Reverse primers (5'-3')	Melting tempearature (T°C)	Expected product size (bp) in PCR	Expected product size (bp) in Q-PCR	Putative Functions
OsISM00001	LOC_Os01g01010	2566	TCCCTCGTCCGTGACATT	CATTTTTCCGGTTTGAGCAT	60.0	820	78	TBC domain containing protein expressed
OsISM00002	LOC_Os01g01010	3420	GGTTTCCGTAAAGCCCTAAAA	AGGCATAGCAGCCAAGAAGA	59.8	1095	92	TBC domain containing protein expressed
OsISM00003	LOC_Os01g01010	4501	TGGCTGCTATGCCTTGAGTA	GCATGCTCTGGCACTGTCTA	59.9	1676	99	TBC domain containing protein expressed
OsISM00004	LOC_Os01g01010	6915	CTGAATGGCTTTGGACACTG	TGACATCCTAGCCATATTTCTTG A	59.6	184	99	TBC domain containing protein expressed
OsISM00005	LOC_Os01g01010	7108	GCAGTTTATGCATGGGTTGA	AAGGCATCTGCATCATCCTC	59.9	182	99	TBC domain containing protein expressed
OsISM00006	LOC_Os01g01010	7281	AGATGCCTTTTGGTGCTTTG	CACAATGCTTGCAACTGCTT	60.2	186	97	TBC domain containing protein expressed
OsISM00007	LOC_Os01g01010	7565	TTCGTCGAGAGCTGTCCTTT	TCATTAAAGTCAGCAGCCCA	59.8	671	68	TBC domain containing protein expressed
OsISM00008	LOC_Os01g01010	8592	CTCGCTCAATAGATGATGCAA	CCTCAGCTTGATAGCCATGC	60.0	578	90	TBC domain containing protein expressed
OsISM00009	LOC_Os01g01010	9147	TACGCATGGCTATCAAGCTG	CAGGGCGATTTCTCAACTTC	59.9	187	99	TBC domain containing protein expressed
OsISM00011	LOC_Os01g01030	12789	CCATCAACCAAGCAAGATCA	GACGGTAATGTCGCCGTAAT	59.7	187	93	monocopper oxidase putative expressed
OsISM00012	LOC_Os01g01030	13228	GTGCAGAGCTACCACTTGGA	GTATGTACCGCGGCTGTTGT	60.0	174	85	monocopper oxidase putative expressed
OsISM00013	LOC_Os01g01030	13398	ATACAACAAATGGGATGGCG	CCGCATTGTCAAGAAAGACA	60.2	620	87	monocopper oxidase putative expressed
OsISM00014	LOC_Os01g01030	14147	GTGGTGCGCTGTCAAGTTTA	TTCCACAGCTGCGTTATCTG	60.0	176	80	monocopper oxidase putative expressed
OsISM00015	LOC_Os01g01040	15943	CCTCGACTGCATCTACGACA	TACTGAGCACGTACCACCCA	60.1	497	89	expressed protein
OsISM00016	LOC_Os01g01040	16415	AGAGTTGGGTGGTACGTGCT	CAATGTTGCGTCTTTGGATG	59.9	178	93	expressed protein
OsISM00017	LOC_Os01g01040	17235	GCAACGAAAATGTGCTAGGC	TGCCACACTGAAGGCATAAA	60.5	313	69	expressed protein
OsISM00018	LOC_Os01g01040	17529	TTATGCCTTCAGTGTGGCAA	CTGGAATCCTCGTCTCGAAG	60.1	480	82	expressed protein

ISM IDs	Rice gene locus IDs	Physical positions (bp) on chromosomes	Forward primers (5'-3')	Reverse primers (5'-3')	Melting tempearature (T°C)	Expected product size (bp) in PCR	Expected product size (bp) in Q-PCR	Putative Functions
OsISM00019	LOC_Os01g01040	17989	CTTCGAGACGAGGATTCCAG	GGCCCCAGGTATACATGACA	60.3	184	98	expressed protein
OsISM00020	LOC_Os01g01040	18265	CCGATTAGACGAGCAACCTC	TTTCCCCAACTCTTTCGATG	59.9	297	86	expressed protein
OsISM00021	LOC_Os01g01050	22231	TATGATCAATTTCGCCGTCC	AAGTTTCGTTCGCACAGAGC	60.7	371	79	R3H domain containing protein expressed
OsISM00022	LOC_Os01g01050	22793	AACCTGCCAGCTCCTATCAA	TCGATCTTCACCTTCACCAA	59.5	206	90	R3H domain containing protein expressed
OsISM00023	LOC_Os01g01050	22979	TTGGTGAAGGTGAAGATCGAC	TACGTCGCTTACGAGGACAG	59.6	538	78	R3H domain containing protein expressed
OsISM00024	LOC_Os01g01050	23504	CGTAAGCGACGTATTATGGGA	TTCCTTCCAGGCTTCTTCAA	60.0	964	95	R3H domain containing protein expressed
OsISM00025	LOC_Os01g01050	24487	CTGCTGAAATCTCGCATTTG	TTCCCTCTCCTTGAGTGACG	60.0	455	90	R3H domain containing protein expressed
OsISM00026	LOC_Os01g01060	26259	GCTCTTCAACTGCTGGTCCT	GTAGTCGGCGAGGGATATGT	59.3	138	59	40S ribosomal protein S5 putative expressed
OsISM00027	LOC_Os01g01060	26617	TCATCGTAGATGCCATCATCA	AGATATCCACAGCCTGCCTC	59.7	541	91	40S ribosomal protein S5 putative expressed
OsISM00028	LOC_Os01g01060	27233	AGACCATTGCTGAGTGCCTT	GCAACACGCTCAATCTCATC	59.6	171	98	40S ribosomal protein S5 putative expressed
OsISM00029	LOC_Os01g01070	28946	CTGGGAAAGATCGTCATCGT	ACATCCCTAAAATCGGGGAG	60.1	264	93	expressed protein
OsISM00030	LOC_Os01g01070	29205	GATGTGCTCTCTGGTGCCTT	ATAGCAAGTGAGCAGCGTGA	60.1	597	89	expressed protein
OsISM00031	LOC_Os01g01070	29782	TCACGCTGCTCACTTGCTAT	TGACCTGGACAAAAGTTGGA	59.4	145	65	expressed protein
OsISM00032	LOC_Os01g01070	29934	TTGCTATTGTCACCGTCGAT	GATAGCCCCAGCAACAACAG	59.9	368	72	expressed protein
OsISM00033	LOC_Os01g01070	30282	CTGTTGTTGCTGGGGCTATC	CACGCTTCGTCACAAACATC	60.5	263	88	expressed protein
OsISM00034	LOC_Os01g01070	30538	AAGCGTGGCTTATCTGATGC	TATTCTTCCTGCCAGATGCC	60.3	869	97	expressed protein
OsISM00035	LOC_Os01g01070	31435	GCCAGGAAATTACAGAATCCA	TTCCTCCTGGAAGGCACTTA	59.4	149	72	expressed protein
OsISM00036	LOC_Os01g01070	31572	TTCCAGGAGGAAATGCAATC	TGGGTATATGAAAGACGTCCAA	59.7	172	75	expressed protein

ISM IDs	Rice gene locus IDs	Physical positions (bp) on chromosomes	Forward primers (5'-3')	Reverse primers (5'-3')	Melting tempearature (T˚C)	Expected product size (bp) in PCR	Expected product size (bp) in Q-PCR	Putative Functions
OsISM00037	LOC_Os01g01070	31716	CAAAGCTTGGACGTCTTTCA	GGGCTCTTATCCAGCCTTTT	59.4	174	89	expressed protein
OsISM00038	LOC_Os01g01070	31870	AAAAGGCTGGATAAGAGCCC	TGGCAATTCAATAGCTGGAA	59.5	446	76	expressed protein
OsISM00039	LOC_Os01g01070	32296	TTCCAGCTATTGAATTGCCA	CCAGAAAACTCCGGTTCTGA	59.7	145	74	expressed protein
OsISM00040	LOC_Os01g01070	32421	TCAGAACCGGAGTTTTCTGG	TCCTGATGCCGACATAGTTG	59.9	158	85	expressed protein
OsISM00041	LOC_Os01g01070	32559	CAACTATGTCGGCATCAGGA	GAACTTGCTCGGCTCAAAAC	59.8	439	80	expressed protein
OsISM00042	LOC_Os01g01080	34908	GTCCTCCTCGCTCTAGCCAT	TGATGTAGGCGCTAACTCCC	60.6	148	59	decarboxylase putative expressed
OsISM00043	LOC_Os01g01080	35036	GGGAGTTAGCGCCTACATCA	CGGTCCGCAGAGTACTTTTT	59.8	536	90	decarboxylase putative expressed
OsISM00044	LOC_Os01g01080	35638	TAAACTGGCAGGGAAAATGC	TCAATGCAAAATGACCTTCG	59.9	221	70	decarboxylase putative expressed
OsISM00045	LOC_Os01g01080	35839	CGAAGGTCATTTTGCATTGA	GTGAAGTGGGTTGGTGTGTG	59.8	780	62	decarboxylase putative expressed
OsISM00046	LOC_Os01g01080	36775	CGCGACTATATGCGAACAAA	TCGTATGCAGAATGTGCTGA	59.4	150	75	decarboxylase putative expressed
OsISM00047	LOC_Os01g01080	36985	TGCAGATGTGAAAGGATTCAA	ATGAGGAAATCCTGGTGCAG	59.7	321	77	decarboxylase putative expressed
OsISM00048	LOC_Os01g01080	37291	CCAGGATTTCCTCATGGTTT	CACCAAGGCAGAGATCAACA	59.3	204	95	decarboxylase putative expressed
OsISM00049	LOC_Os01g01080	37482	CTCTGCCTTGGTGGATTTGT	CCCTTGACACAGAAGTCGAA	59.5	873	78	decarboxylase putative expressed
OsISM00050	LOC_Os01g01080	38391	ATTAGCCCCAAAAGGGACAA	TTGAAGCTTACCAGCAACAAA	59.6	189	80	decarboxylase putative expressed
OsISM00051	LOC_Os01g01080	38503	TCCCTCCATCTATTTGGCAC	CATAGAGCCCACCAGTCCAT	59.9	463	91	decarboxylase putative expressed
OsISM00052	LOC_Os01g01080	38988	TAGGCCTGGTGGGTTGATAG	TCGACACTTCCATGATATGACC	59.9	186	96	decarboxylase putative expressed
OsISM00053	LOC_Os01g01080	39119	TGTGCTTGGTTTCCAGGTTA	TCACAAACAACCCTGGGATA	59.0	366	98	decarboxylase putative expressed
OsISM00054	LOC_Os01g01080	39555	AATGTCATCAAAAGGCTGGC	GTGCTGCAGTGTCACACAAA	59.8	176	74	decarboxylase putative expressed

ISM IDs	Rice gene locus IDs	Physical positions (bp) on chromosomes	Forward primers (5'-3')	Reverse primers (5'-3')	Melting tempearature (T˚C)	Expected product size (bp) in PCR	Expected product size (bp) in Q-PCR	Putative Functions
OsISM00055	LOC_Os01g01080	39720	CACTGCAGCACACGGTCATA	ATAGGCCCTGGATTTGCTTT	60.9	188	83	decarboxylase putative expressed
OsISM00056	LOC_Os01g01090	44167	ACTGCCAATGGGAGATGAAG	TCAAACTCAAAAGCTGTGCG	60.1	159	79	retrotransposon protein putative unclassified
OsISM00057	LOC_Os01g01090	44424	GCTATTCACATCTGCTGCCC	GCATTGAGCAATTGGAGGAT	60.4	184	96	retrotransposon protein putative unclassified
OsISM00058	LOC_Os01g01090	44787	CCCAAAGCCAACTAATCCAA	CGGCTTCCTACGAGTTTGAG	60.0	158	92	retrotransposon protein putative unclassified
OsISM00059	LOC_Os01g01090	45257	ACACCAAAATCCACTCGTCC	TCCGGAGGATCTCTTCTTCA	59.9	153	74	retrotransposon protein putative unclassified
OsISM00060	LOC_Os01g01090	45504	GGACGAAGAGTTATGCCTGC	TTACCGTTTTGGCTTCGTTC	60.0	166	90	retrotransposon protein putative unclassified
OsISM00061	LOC_Os01g01090	45780	GCTGAGCTCCAGGAATCAAA	TTGAATCTGCTTCTCCAACTGA	60.2	158	85	retrotransposon protein putative unclassified
OsISM00062	LOC_Os01g01090	45965	GCAAGACAAAGAGACCTGGC	AACCAGGACGTCGAATTGAG	60.1	151	76	retrotransposon protein putative unclassified
OsISM00064	LOC_Os01g01120	61265	CTCCTCCGAGCTTCCTGAC	GAAGGAGATGGGCGTCGT	60.6	168	77	enolase-phosphatase E1 putative expressed
OsISM00065	LOC_Os01g01120	61877	CTCTGCGGATTCTTCGACAC	CGTAGTAGCTGCTAACCTCGC	60.1	148	56	enolase-phosphatase E1 putative expressed
OsISM00066	LOC_Os01g01120	62077	AGACGTCTACCAAGAAGCCG	ATGATTATCAGGGAGGGGTG	59.1	227	92	enolase-phosphatase E1 putative expressed
OsISM00067	LOC_Os01g01140	68913	AGAAGATGTCGCATGCTCCTA	ATTCACCTCCATCTCCCCAC	60.6	154	88	hypothetical protein
OsISM00068	LOC_Os01g01150	72905	GATTCCAAGGCTGCAGGAG	TGGGCTTAGGTTGCTAATCTG	60.1	631	97	RNA recognition motif putative expressed
OsISM00069	LOC_Os01g01150	73950	GTCGAAGAGAAGGATGCCAG	GATCTACGGGCAGAAGGTGA	60.1	696	57	RNA recognition motif putative expressed
OsISM00070	LOC_Os01g01160	81910	GCGTCGCCCCCTACTACT	TCTCCTCCAGCCAGACTTGT	60.1	838	51	heat shock protein DnaJ putative expressed
OsISM00071	LOC_Os01g01170	84549	ATCTGCAGAGACGACGCC	GAGCTTCGACCGCTGGAC	61.1	209	71	expressed protein
OsISM00072	LOC_Os01g01170	84802	CCTCAACCAACTGACCAACA	GCTTCAGATTCAGATTCGGC	59.7	200	94	expressed protein
OsISM00073	LOC_Os01g01170	85031	GCACCACCAAGGAAGAAATC	GGTGGTGAACAAACCTGGAC	59.9	221	94	expressed protein

ISM IDs	Rice gene locus IDs	Physical positions (bp) on chromosomes	Forward primers (5'-3')	Reverse primers (5'-3')	Melting tempearature (T˚C)	Expected product size (bp) in PCR	Expected product size (bp) in Q-PCR	Putative Functions
OsISM00074	LOC_Os01g01170	85232	GTCCAGGTTTGTTCACCACC	TTTTGGTCCTGTGACCTTCC	60.1	200	99	expressed protein
OsISM00075	LOC_Os01g01170	86657	TCTCATGGAGAAATCTGCTGG	ACATCTGCCTACGACGCTCT	60.2	710	99	expressed protein
OsISM00076	LOC_Os01g01170	87374	GCTGAACTCACATTGCAGGA	GACGCCATCCTCTCTTCAAC	59.9	173	70	expressed protein
OsISM00077	LOC_Os01g01200	95047	CCCGTCCCTCCAATTTTTAT	CTCCACGGTTGTGCTGTTC	60.2	273	80	retrotransposon protein putative unclassified
OsISM00078	LOC_Os01g01200	95400	CGAACGTGAAGCTGCCAT	CGATTTTCTCCTTCAGCGAC	60.5	162	93	retrotransposon protein putative unclassified
OsISM00079	LOC_Os01g01200	95648	ATCGGACATCGTCGAAAGAC	TTCTCACCAAGCGTCACAAG	60.1	162	90	retrotransposon protein putative unclassified
OsISM00080	LOC_Os01g01200	95788	GGCTTGTGACGCTTGGTG	GTCGAGGCCATAGTAGGGGA	61.4	168	92	retrotransposon protein putative unclassified
OsISM00081	LOC_Os01g01200	96587	CGCAAGGATGTGATGAAAGA	GCTGAAGAAAGCCAAGGTGA	60.2	164	83	retrotransposon protein putative unclassified
OsISM00082	LOC_Os01g01200	96836	GATCGTTCCGATACTTGCGT	GTTTCGCGACCTCAAACTTC	60.0	277	69	retrotransposon protein putative unclassified
OsISM00083	LOC_Os01g01240	107645	AACCCTGGGAGACTTACCGT	TGACACATGATATCGACCGC	60.2	1614	82	retrotransposon protein putative Ty3-gypsy subclass
OsISM00084	LOC_Os01g01240	109233	TAGTCGGCGGTCGATATCAT	GCGTGAACCATTCCTTGTCT	60.3	200	51	retrotransposon protein putative Ty3-gypsy subclass
OsISM00085	LOC_Os01g01250	117663	CCTCCAAGGTGGTCATTCAC	GATCTTCGGCCTCGGACT	60.3	186	92	transposon protein putative unclassified
OsISM00086	LOC_Os01g01280	133567	CTTACTCCCAATCCCAATCG	GGGTTTGTGGACACGCAT	59.9	182	98	expressed protein
OsISM00087	LOC_Os01g01280	133798	AATACACCAACGGACAAGGC	TTCCTCTGCGTCTTGAGGAT	59.9	149	60	expressed protein
OsISM00088	LOC_Os01g01295	141602	CAACGACCTCGTCGACCT	ACTTGGCCCAACTATCTCCA	59.7	660	99	expressed protein
OsISM00089	LOC_Os01g01295	142401	GGCAACCGAGTTCAAGAGAG	TCCTCATCAACCATTGCATC	59.7	200	67	expressed protein
OsISM00090	LOC_Os01g01295	142623	CACCGCCCTAGAGGAGAAAT	CAAAGGCTCATAATGGTCGG	60.5	232	94	expressed protein
OsISM00091	LOC_Os01g01302	144724	TCTTCCTCTCCTCCTCCTCC	AAGCTCCTCCAGAGTTTGCTC	60.0	207	87	shikimate kinase putative expressed

ISM IDs	Rice gene locus IDs	Physical positions (bp) on chromosomes	Forward primers (5'-3')	Reverse primers (5'-3')	Melting tempearature (T°C)	Expected product size (bp) in PCR	Expected product size (bp) in Q-PCR	Putative Functions
OsISM00092	LOC_Os01g01302	144922	GAGGAGCTTAACCCTTCCGT	GACCGACGATGTAGATGGGT	59.8	164	86	shikimate kinase putative expressed
OsISM00093	LOC_Os01g01302	145051	CGATTTCAGGAAAACACCCA	ATGATGGAATTGGCAAGGAG	60.4	183	85	shikimate kinase putative expressed
OsISM00094	LOC_Os01g01302	145271	GTTCTTGGAGGCAAAGATGC	GTGAGCTGTTTCAGGCCTTC	59.9	202	90	shikimate kinase putative expressed
OsISM00095	LOC_Os01g01302	145509	TGCTGTCATGAACTCAACCA	TCATTCACCACCAATTCCAG	59.1	165	85	shikimate kinase putative expressed
OsISM00096	LOC_Os01g01302	145653	TCTGGAATTGGTGGTGAATG	GTGTCGCTGTCGGACTCTG	60.0	480	92	shikimate kinase putative expressed
OsISM00097	LOC_Os01g01302	146107	CAATGAGCAGAGTCCGACAG	GTAGCCCAGTTGAGATGCCA	60.4	227	99	shikimate kinase putative expressed
OsISM00098	LOC_Os01g01302	146337	AGCATCGACTCAGTGACCCTA	AGCAGCTTCCATCATCGACT	59.9	224	91	shikimate kinase putative expressed
OsISM00099	LOC_Os01g01307	147286	CCCTCGACCTCTACAACCAG	CCTCCTTTGGCCAAGTATCA	59.9	1211	73	translocon-associated protein beta domain containing protein expressed
OsISM00100	LOC_Os01g01307	148492	GGAGGCATTTGAGCTTGTCT	CAAGACAAATGCATGCGAAG	59.9	173	86	translocon-associated protein beta domain containing protein expressed
OsISM00101	LOC_Os01g01307	148689	CAAGGTTCACCTGCGGTTAT	AAGGATGGGGGTTGAATAGG	60.0	191	76	translocon-associated protein beta domain containing protein expressed
OsISM00102	LOC_Os01g01307	148897	TGAGAGGCCTCCAGAAAAGA	GCACTAGAAAAGTTGCTCCTTG	59.2	213	83	translocon-associated protein beta domain containing protein expressed
OsISM00103	LOC_Os01g01307	149038	CTCTCCTTGTTGGTTCAGGC	AACACTCCAACCAAGCCAAC	59.9	170	78	translocon-associated protein beta domain containing protein expressed
OsISM00104	LOC_Os01g01312	150875	CCGCAGACATCCACGATATT	GGCTGATGACCTCCATTGAT	60.4	229	95	retrotransposon protein putative Ty1-copia subclass expressed
OsISM00105	LOC_Os01g01312	151503	AGTGCTTGATGGAGCCTCTG	GGAATGGTGTCAACAAACCC	60.3	169	78	retrotransposon protein putative Ty1-copia subclass expressed
OsISM00106	LOC_Os01g01312	151976	TGGCAAGAAATGGAGGAAAG	CTGAAACTGACACAGCTGCC	59.9	238	84	retrotransposon protein putative Ty1-copia subclass expressed
OsISM00107	LOC_Os01g01312	153588	CTGGTGCAAACCGGATTATT	CTCTAGTTTGCCCAATTCGC	59.8	459	94	retrotransposon protein putative Ty1-copia subclass expressed
OsISM00108	LOC_Os01g01312	154424	AGCCTTTTTGATGCTCAAGG	GCTTCTAGGCGCAGCTTCT	59.7	181	87	retrotransposon protein putative Ty1-copia subclass expressed
OsISM00109	LOC_Os01g01390	197193	AGATTCCACTCCCACCCAC	TGACAAGCCAGACAAACAGC	59.9	673	67	expressed protein

ISM IDs	Rice gene locus IDs	Physical positions (bp) on chromosomes	Forward primers (5'-3')	Reverse primers (5'-3')	Melting tempearature (T˚C)	Expected product size (bp) in PCR	Expected product size (bp) in Q-PCR	Putative Functions
OsISM00110	LOC_Os01g01390	198986	AGCTTTCAAATGGGTCTCCA	CTCACACACATCCCCTCCTT	59.8	293	75	expressed protein
OsISM00111	LOC_Os01g01400	201051	CTCTGCTTCCTGCTCGACAT	AGGTTGGCGAGGTGGAGT	60.7	224	81	expressed protein
OsISM00112	LOC_Os01g01400	201312	CTCGCCCTCTGCTACGTC	CTCCAGGCCTATACACCACC	59.6	720	71	expressed protein
OsISM00113	LOC_Os01g01400	202085	GCCTTTCGTCCTAACCAACA	TCATCAGTGGCCCAAGAATA	59.6	275	99	expressed protein
OsISM00114	LOC_Os01g01400	202397	CAAAACACACAAGCTCTCCG	CAGCTCCTGTTTCCAGCAAT	59.9	169	86	expressed protein
OsISM00115	LOC_Os01g01400	202626	TTGAAAACTGTGTGGTACGAAGA	GCCTCCTCCTTGTCATCCTT	60.1	201	98	expressed protein
OsISM00116	LOC_Os01g01400	202896	TGCTTCAGCAAATCACATCA	TTAGCAAGGTCAATAGGGCG	59.6	345	79	expressed protein
OsISM00117	LOC_Os01g01400	203395	TCTTTTGACGTGATTGAGCG	ATCACAATATGAGGCGTCCC	59.9	179	75	expressed protein
OsISM00118	LOC_Os01g01400	203591	TTGAGGTTGCTCTTGATGATG	GGAGCACACAAGTCCCTGAT	59.5	195	99	expressed protein
OsISM00119	LOC_Os01g01400	203842	CCAACCATCTGAGAAGGGAC	TTGCAAGGTCGACTCACAAC	59.7	256	88	expressed protein
OsISM00120	LOC_Os01g01400	204104	CGGTACCAAGGAAGTCCAGA	GAGGGTTGTAATCCTTCAGCA	59.6	211	69	expressed protein
OsISM00121	LOC_Os01g01400	204335	CACTCGAGACTGAATTGTCTGG	GATCCTTCGCGCCACAAG	61.4	249	92	expressed protein
OsISM00122	LOC_Os01g01420	209507	CCACGCTCTGCTACATCAAA	GGACAGTCCTTAAAGCGCAA	60.2	182	85	expressed protein
OsISM00123	LOC_Os01g01420	209855	GAGAGAGGGGCTTTTCTGGT	TGAGCCTTGACTTTGCACTTT	59.9	203	77	expressed protein
OsISM00124	LOC_Os01g01420	210136	TGTTAGGAATGCTTGGGGAC	GAAGGAGCATCTCCCAAGGT	60.3	1153	47	expressed protein
OsISM00125	LOC_Os01g01420	211323	CTCTCGAAATTGCCCAAAAA	GAGTGGAGGTCCCCATCATA	60.0	181	99	expressed protein
OsISM00126	LOC_Os01g01420	211547	GCAGCAAAAGAACACTCAGC	TGCTTTCGTTCCCAAGATTT	59.2	1010	98	expressed protein
OsISM00127	LOC_Os01g01420	212614	CTGGAGAGGCCAGTCTTGAG	AACCGTGCATATCACTTGTCA	59.6	186	83	expressed protein

ISM IDs	Rice gene locus IDs	Physical positions (bp) on chromosomes	Forward primers (5'-3')	Reverse primers (5'-3')	Melting tempearature (T˚C)	Expected product size (bp) in PCR	Expected product size (bp) in Q-PCR	Putative Functions
OsISM00128	LOC_Os01g01430	215731	TCATCATCATCGTCCAGCTC	CTGGTTGGCCTCCTTCTTCT	60.3	204	88	No apical meristem protein putative expressed
OsISM00129	LOC_Os01g01450	226584	AGAGATGGCAAGGTCTGCCT	CGTCTTTCACTCTCGTGCTG	60.6	201	92	stress responsive protein putative expressed
OsISM00130	LOC_Os01g01450	226834	CCTCAAGCACTCCATCGG	CACAGCACAGACTCGTCCAT	60.1	773	79	stress responsive protein putative expressed
OsISM00131	LOC_Os01g01470	241056	TGGATCTCAACAAGTGCGAG	ACCACTCCTTCTCCCCATTC	60.1	176	67	no apical meristem protein putative expressed
OsISM00132	LOC_Os01g01510	260585	GAAGTGGAAGAGGCCCCC	GATCATGTCGCGGTGCTT	61.4	205	98	sas10/Utp3 family protein expressed
OsISM00133	LOC_Os01g01510	260759	ATTTCCAGTCCACAAGCACC	TACCTCGAGATCGAAGACGG	60.2	186	95	sas10/Utp3 family protein expressed
OsISM00134	LOC_Os01g01510	260899	GGAGTCTGAGGATGACCTGG	CCTCGCTGTCATCAGTTTCA	59.8	717	75	sas10/Utp3 family protein expressed
OsISM00135	LOC_Os01g01510	261615	GGAGATGAGGATGGCAACAT	GCAATTTGTTTCACTGCCCT	60.0	344	99	sas10/Utp3 family protein expressed
OsISM00136	LOC_Os01g01510	262148	TGGTGAAAGTGATGAAGAGG A	CACCATCAGGAACTTTGACTTG	59.0	411	66	sas10/Utp3 family protein expressed
OsISM00137	LOC_Os01g01510	262613	GAGATGAGAAGATGGGTGTG C	AGCTCGGACAACAATCCAAC	59.9	437	62	sas10/Utp3 family protein expressed
OsISM00138	LOC_Os01g01510	263072	ATGGCGATAGGACCAGTCAC	TCCAAAGGCTGCATTTTACC	60.0	1211	72	sas10/Utp3 family protein expressed
OsISM00139	LOC_Os01g01510	264350	GGATTGTCTGTGCAGGATCA	TCTTGGAAAGTTTACATTGGCA	59.6	186	94	sas10/Utp3 family protein expressed
OsISM00140	LOC_Os01g01510	264589	GGCAGACATGATTTCTTTGGA	TGTCAACTCTGCAACTTCCAC	59.5	175	83	sas10/Utp3 family protein expressed
OsISM00141	LOC_Os01g01510	264787	TCACCATGAAATTGCCAAAA	AGGCTTTGCGAACCAATATG	60.0	148	55	sas10/Utp3 family protein expressed
OsISM00142	LOC_Os01g01510	264997	TGACGCGATTGAAACGTAAG	ATCCAACGTTTGCAGGTCA	60.0	163	69	sas10/Utp3 family protein expressed
OsISM00143	LOC_Os01g01510	265223	GCGGCAAAATCCAATAAAAA	AATTCGTGCTTTCGTCGTCT	59.9	1082	90	sas10/Utp3 family protein expressed
OsISM00144	LOC_Os01g01510	266472	GGAGGACTGAAAAGCTCTCG	CCTCTTGCCATCTCCTTCAG	59.6	171	93	sas10/Utp3 family protein expressed
OsISM00145	LOC_Os01g01510	266623	CTGAAGGAGATGGCAAGAGG	ATTTCTCGAGCGAGTCAACC	59.7	147	69	sas10/Utp3 family protein expressed

ISM IDs	Rice gene locus IDs	Physical positions (bp) on chromosomes	Forward primers (5'-3')	Reverse primers (5'-3')	Melting tempearature (T˚C)	Expected product size (bp) in PCR	Expected product size (bp) in Q-PCR	Putative Functions
OsISM00146	LOC_Os01g01510	266750	GGTTGACTCGCTCGAGAAAT	TCCCCGCTTAACAAGTTTTG	59.8	163	84	sas10/Utp3 family protein expressed
OsISM00147	LOC_Os01g01570	290532	AACCTGTGCCAAGGAGGTTA	ATCTGAAGGTGCTTCACCAA	58.9	154	79	kinesin heavy chain putative expressed
OsISM00148	LOC_Os01g01570	290724	ATCGTGCCTCCTCTTCTGAG	TGCATTGTCTCGTTGCTTCT	59.6	180	99	kinesin heavy chain putative expressed
OsISM00149	LOC_Os01g01570	290897	CAATGCACAGTCGAAACTTGA	GTCAGGGTGCTTGCTACCTC	59.9	225	99	kinesin heavy chain putative expressed
OsISM00150	LOC_Os01g01590	292802	GGATCCACCAAGAGAGGCTT	GGAAATGGGCATCAACACTC	60.5	637	85	expressed protein
OsISM00151	LOC_Os01g01600	302438	GATTGAACCACCAATGGAGC	CAGCTGACTTTCAACTGATTGG	60.1	610	99	ethylene-responsive protein related putative expressed
OsISM00152	LOC_Os01g01600	303465	GGCCCTCCTGCTCATCTAAT	CTTGAGCTGCTGCATTCGTA	60.4	1138	74	ethylene-responsive protein related putative expressed
OsISM00153	LOC_Os01g01600	304689	CACTCCTCCTCAATGCTTCC	AAGGGTGAGACCAGTTGCTG	60.1	204	87	ethylene-responsive protein related putative expressed
OsISM00154	LOC_Os01g01600	304873	CAGCAACTGGTCTCACCCTT	TTTGATGTAACCGATGGCCT	60.3	165	70	ethylene-responsive protein related putative expressed
OsISM00155	LOC_Os01g01600	305018	AGGCCATCGGTTACATCAAA	CCCTGCAGGCTAATTTCTTG	60.1	211	97	ethylene-responsive protein related putative expressed
OsISM00156	LOC_Os01g01600	305224	CAGGGCAGCACAACAACA	GGCAGAGTCCTCTACTTCGG	59.8	193	84	ethylene-responsive protein related putative expressed
OsISM00157	LOC_Os01g01640	319215	GCCTCCCCGAGTTCAAGT	GCCAGAAGACAGGAACATCA	59.5	1330	97	Rer1 protein putative expressed
OsISM00158	LOC_Os01g01640	320622	TATGTTCCCTTCAGCGTTGG	CTTTCGAAGAACTGCTGCCT	60.4	349	68	Rer1 protein putative expressed
OsISM00159	LOC_Os01g01689	334841	CGAGAAGCTCTCCTCCGA	AGGCGGCAGAAGGAGAAG	60.0	187	97	phosphatidylinositol 3- and 4-kinase family protein expressed
OsISM00160	LOC_Os01g01689	335038	AACACCGCCAACCTCAAG	GATGACGCATTTGGTGAGC	60.0	168	82	phosphatidylinositol 3- and 4-kinase family protein expressed
OsISM00161	LOC_Os01g01689	335272	CAGTGCGCTCAAGACTTCC	TTCTTAGCGACGGAAATCAAA	59.8	1099	60	phosphatidylinositol 3- and 4-kinase family protein expressed
OsISM00162	LOC_Os01g01689	336468	CGGTATCAGATGAAACCTCGA A	TTCATCAAAACCAGTCGCAA	60.8	156	76	phosphatidylinositol 3- and 4-kinase family protein expressed
OsISM00163	LOC_Os01g01689	336701	ACCGGGTGATTACCCTGATA	TAGCCCTAGCAAGTTCCAGC	59.4	528	81	phosphatidylinositol 3- and 4-kinase family protein expressed

ISM IDs	Rice gene locus IDs	Physical positions (bp) on chromosomes	Forward primers (5'-3')	Reverse primers (5'-3')	Melting tempearature (T˚C)	Expected product size (bp) in PCR	Expected product size (bp) in Q-PCR	Putative Functions
OsISM00164	LOC_Os01g01689	337278	CCAAAATTTTAATACTGGAGCCG	AGGAGCCAGCTTTCTCGTCT	60.8	142	66	phosphatidylinositol 3- and 4-kinase family protein expressed
OsISM00165	LOC_Os01g01689	337404	GAGAAAGCTGGCTCCTTGAG	GTCCTCGGTGTGCATTTGTA	59.4	206	90	phosphatidylinositol 3- and 4-kinase family protein expressed
OsISM00166	LOC_Os01g01689	337655	CATCATGGATAGCCTGCTGA	TCCACTCGGTACCCATACTTG	59.8	739	82	phosphatidylinositol 3- and 4-kinase family protein expressed
OsISM00167	LOC_Os01g01689	338419	AAGGCGACCTGTAAGGGTTT	AATCTTGAAACCGTAGGGCA	59.8	679	62	phosphatidylinositol 3- and 4-kinase family protein expressed
OsISM00168	LOC_Os01g01689	339078	TGCCCTACGGTTTCAAGATT	AGCGCAGAGAACTCCTTGAG	59.7	208	61	phosphatidylinositol 3- and 4-kinase family protein expressed
OsISM00169	LOC_Os01g01689	339266	CTCAAGGAGTTCTCTGCGCT	TCTTGCCAATAACCTTCCAAA	59.7	282	99	phosphatidylinositol 3- and 4-kinase family protein expressed
OsISM00170	LOC_Os01g01689	339569	CGCACTTCCTTTGTTTAGCA	TTGGGAATGAGAAGCTCACC	59.7	461	93	phosphatidylinositol 3- and 4-kinase family protein expressed
OsISM00171	LOC_Os01g01689	339982	TGAGGTTGTTGGGTGAAATG	CTGACACAAATGATGCATGAAC	59.2	174	55	phosphatidylinositol 3- and 4-kinase family protein expressed
OsISM00172	LOC_Os01g01689	340136	TCATGCATCATTTGTGTCAGA	TCTTTGAAAAGTAACAAGCAATGAA	59.0	1418	99	phosphatidylinositol 3- and 4-kinase family protein expressed
OsISM00173	LOC_Os01g01689	341533	TTGCTTGTTACTTTTCAAAGATAGG	GGAGTGCACTAACTCAAAAGTTG	58.2	906	99	phosphatidylinositol 3- and 4-kinase family protein expressed
OsISM00174	LOC_Os01g01689	342369	GGGCGATGTATCCAATTCC	CACTTTGTTCATTCAAAAGAGAGA	58.9	808	99	phosphatidylinositol 3- and 4-kinase family protein expressed
OsISM00175	LOC_Os01g01689	343225	CGCCAGTGATTAACTCAGCA	TTCTTCCTCGTGAGACCAGC	60.3	195	87	phosphatidylinositol 3- and 4-kinase family protein expressed
OsISM00176	LOC_Os01g01689	343400	GCTGGTCTCACGAGGAAGAA	CTCGGCTGCATTTCTCAGAC	60.8	791	97	phosphatidylinositol 3- and 4-kinase family protein expressed
OsISM00177	LOC_Os01g01689	344323	GCAATCTAATTCATTGTTCGCT	GGCATTGGTGACATAATCCA	58.8	326	87	phosphatidylinositol 3- and 4-kinase family protein expressed
OsISM00178	LOC_Os01g01689	345122	CTCTGTTGTGCCGCTTCATA	TTGTGCAAAGTTCAAGCAAGA	59.8	186	99	phosphatidylinositol 3- and 4-kinase family protein expressed
OsISM00179	LOC_Os01g01689	345301	TGCACAAATATCTCTGCCTCA	TCTGTGAAACTGATCTCTGTAGCC	59.7	1484	96	phosphatidylinositol 3- and 4-kinase family protein expressed
OsISM00180	LOC_Os01g01689	346783	GATTGGTTTTTCCGCATCAG	GCATGCTAAGCAAATGACCA	60.1	293	98	phosphatidylinositol 3- and 4-kinase family protein expressed
OsISM00181	LOC_Os01g01689	347137	AATATGGGAAGGACACAGCG	TGCACCATCTTGACACCAAT	60.0	876	64	phosphatidylinositol 3- and 4-kinase family protein expressed

ISM IDs	Rice gene locus IDs	Physical positions (bp) on chromosomes	Forward primers (5'-3')	Reverse primers (5'-3')	Melting tempearature (T˚C)	Expected product size (bp) in PCR	Expected product size (bp) in Q-PCR	Putative Functions
OsISM00182	LOC_Os01g01689	348088	TGCTCTGATTACACTAGCTCATTTG	ATAGCAGCAACAGCGGAAAT	59.9	286	85	phosphatidylinositol 3- and 4-kinase family protein expressed
OsISM00183	LOC_Os01g01689	348354	ATTTCCGCTGTTGCTGCTAT	TCGTGAAGCATAATTGAGCC	59.4	176	91	phosphatidylinositol 3- and 4-kinase family protein expressed
OsISM00184	LOC_Os01g01689	348510	GGCTCAATTATGCTTCACGA	CTCACTTCACAAGCAACCCA	59.4	237	80	phosphatidylinositol 3- and 4-kinase family protein expressed
OsISM00185	LOC_Os01g01689	348727	TGGGTTGCTTGTGAAGTGAG	GCTCATCCGATTTAAAGCCA	60.0	599	71	phosphatidylinositol 3- and 4-kinase family protein expressed
OsISM00186	LOC_Os01g01689	349373	ATTCTGAGGGGTGAGACTGC	TCCAAAGATCGGGACAAAGT	59.4	481	97	phosphatidylinositol 3- and 4-kinase family protein expressed
OsISM00187	LOC_Os01g01689	349938	GAAATTTCTGAGCCTGAGCG	TGGCTGACGTGAAGAAGATG	60.0	1292	94	phosphatidylinositol 3- and 4-kinase family protein expressed
OsISM00188	LOC_Os01g01689	351276	CAGTTGTTGACGGATTTATGGA	AAAACCCTGTCTGAACGGAA	59.7	180	92	phosphatidylinositol 3- and 4-kinase family protein expressed
OsISM00189	LOC_Os01g01689	351436	TTCCGTTCAGACAGGGTTTT	CGAATATGCCTAGGATGGCT	59.4	594	78	phosphatidylinositol 3- and 4-kinase family protein expressed
OsISM00190	LOC_Os01g01689	352068	TTGGGCATAGAGTGGCTCTT	CATTGGTCTTGCAATGGTTG	59.9	574	89	phosphatidylinositol 3- and 4-kinase family protein expressed
OsISM00191	LOC_Os01g01689	352657	CAAGTTGTTGGCTGCTTTCA	CAAAGCTAGGAAGTGGCTCC	59.6	433	93	phosphatidylinositol 3- and 4-kinase family protein expressed
OsISM00192	LOC_Os01g01689	353113	TTCCATGCTTCACTTTCTGG	AAACAGCTCTGGTGGCAAGT	59.4	165	88	phosphatidylinositol 3- and 4-kinase family protein expressed
OsISM00193	LOC_Os01g01689	353258	ACTTGCCACCAGAGCTGTTT	GAAGCTTCATTTGCCACAGA	59.5	367	53	phosphatidylinositol 3- and 4-kinase family protein expressed
OsISM00194	LOC_Os01g01689	353624	CATCGGTGCTTGCTGATTTT	TCAGTAAGATTCGGCACATGA	60.2	187	80	phosphatidylinositol 3- and 4-kinase family protein expressed
OsISM00195	LOC_Os01g01689	353905	TTGAAAGCTTGTTTGTCTGATGA	CAGAGCATTCAGACCCCTTG	60.4	817	97	phosphatidylinositol 3- and 4-kinase family protein expressed
OsISM00196	LOC_Os01g01689	354714	ATGCTCTGCATTGCCTTGAT	CCTGAGCATTTATGCCTCGT	60.5	150	70	phosphatidylinositol 3- and 4-kinase family protein expressed
OsISM00197	LOC_Os01g01689	354845	CGAGGCATAAATGCTCAGGT	AAATGTCATACCATAGGGAGCA	59.4	421	94	phosphatidylinositol 3- and 4-kinase family protein expressed
OsISM00198	LOC_Os01g01689	355369	CCTTGGTGGGCTCACTAATTT	TGCAAGCAAAAGTTCAGCAA	60.5	207	99	phosphatidylinositol 3- and 4-kinase family protein expressed
OsISM00199	LOC_Os01g01689	355602	TTTGGATTCAAATGCTGGC	TCATCAAATGATTGGAGTCACA	59.3	170	81	phosphatidylinositol 3- and 4-kinase family protein expressed

ISM IDs	Rice gene locus IDs	Physical positions (bp) on chromosomes	Forward primers (5'-3')	Reverse primers (5'-3')	Melting tempearature (T˚C)	Expected product size (bp) in PCR	Expected product size (bp) in Q-PCR	Putative Functions
OsISM00200	LOC_Os01g01689	355825	GCAGAAAAAGCCAGAGGTTG	GGTGGTTGGTGATCGACTTT	59.9	469	79	phosphatidylinositol 3- and 4-kinase family protein expressed
OsISM00201	LOC_Os01g01689	356274	AAAGTCGATCACCAACCACC	TGGCAGCCACAAGATAATCA	60.0	568	79	phosphatidylinositol 3- and 4-kinase family protein expressed
OsISM00202	LOC_Os01g01689	356826	TATCTTGTGGCTGCCAGAGC	CCTCACACCAAAGCTCAACA	60.7	798	77	phosphatidylinositol 3- and 4-kinase family protein expressed
OsISM00203	LOC_Os01g01689	357648	GACCTCCAGATTTTTCGCAC	GATCAATATGTGGGGGCAAC	59.8	150	97	phosphatidylinositol 3- and 4-kinase family protein expressed
OsISM00204	LOC_Os01g01689	357828	AGCTGGACAGCATTTATGGG	CTCCAATCACCCTCATGCTC	60.4	313	80	phosphatidylinositol 3- and 4-kinase family protein expressed
OsISM00205	LOC_Os01g01689	358338	GCCAAGGTTTAAGCAACCAA	AATTTCCAGCTCTCCAAGCA	60.0	172	88	phosphatidylinositol 3- and 4-kinase family protein expressed
OsISM00206	LOC_Os01g01689	358553	CCATTCCCAAAGTTACTGCC	CTTCACATGGAACTCCTCGG	60.0	578	89	phosphatidylinositol 3- and 4-kinase family protein expressed
OsISM00207	LOC_Os01g01689	359099	AGAATGGTGATTCCGAGGAG	CATTTGAAAGAGCCAGCACA	59.5	162	73	phosphatidylinositol 3- and 4-kinase family protein expressed
OsISM00208	LOC_Os01g01689	359259	TGCTAGCAAGGAGAGCACAA	TTTCCATCGTAGGTCCCAAG	59.9	178	92	phosphatidylinositol 3- and 4-kinase family protein expressed
OsISM00209	LOC_Os01g01689	359484	TTCTCCAGTTCCAACTGTTCC	TTGACACAGGATGAAAGTCCA	59.1	714	74	phosphatidylinositol 3- and 4-kinase family protein expressed
OsISM00210	LOC_Os01g01689	360296	TCAATCTGCTTCAGCACTCC	GCAGCCAAAGAAAATCTTGC	59.5	128	46	phosphatidylinositol 3- and 4-kinase family protein expressed
OsISM00211	LOC_Os01g01689	360462	TGGATCATCAAGCTATGCCA	ATATCATGCTGCCCTTGAGC	60.2	1127	83	phosphatidylinositol 3- and 4-kinase family protein expressed
OsISM00212	LOC_Os01g01689	361667	CAAGTGGTTGGCTGAAACAA	GGGCACTTGTGCAATTCAGT	60.4	193	94	phosphatidylinositol 3- and 4-kinase family protein expressed
OsISM00213	LOC_Os01g01689	361966	CAAGCAGCTCTGCGATTAAG	TGCAGTGCTTACCTTTGATGA	59.2	173	88	phosphatidylinositol 3- and 4-kinase family protein expressed
OsISM00214	LOC_Os01g01689	362072	AATGCAGTCTAAGGAGCTGGA	TTCTGTAACTCTTGGATTTTCACTG	59.0	187	97	phosphatidylinositol 3- and 4-kinase family protein expressed
OsISM00215	LOC_Os01g01689	362269	CTTGACGAAGAAGAGGCAGG	GCGCTAAATTTGGCATGTGT	60.6	367	51	phosphatidylinositol 3- and 4-kinase family protein expressed
OsISM00216	LOC_Os01g01689	362609	ACAGTGCACACATGCCAAAT	CCCTGAAGCGCTAGATTCAA	60.3	731	85	phosphatidylinositol 3- and 4-kinase family protein expressed
OsISM00217	LOC_Os01g01689	363339	GGTACCAACGCTCACTCGTT	CCATAGTGAGACCAACCGGA	60.5	575	69	phosphatidylinositol 3- and 4-kinase family protein expressed

ISM IDs	Rice gene locus IDs	Physical positions (bp) on chromosomes	Forward primers (5'-3')	Reverse primers (5'-3')	Melting tempearature (T˚C)	Expected product size (bp) in PCR	Expected product size (bp) in Q-PCR	Putative Functions
OsISM00218	LOC_Os01g01689	363940	TGTGAAAGCAATGATGAAAACA	GCGCATCTTTTGAGCTACCA	60.3	174	99	phosphatidylinositol 3- and 4-kinase family protein expressed
OsISM00219	LOC_Os01g01689	364093	TTGGTAGCTCAAAAGATGCG	TGTATGATAAGGATGATCAATGGC	59.6	178	93	phosphatidylinositol 3- and 4-kinase family protein expressed
OsISM00220	LOC_Os01g01689	364206	TTTTCTTGCAGAATGCGCTA	GGTCACCATTTGCTAGAGCC	59.7	1057	97	phosphatidylinositol 3- and 4-kinase family protein expressed
OsISM00221	LOC_Os01g01689	365355	TCTCATGGAGCTTTGATTCG	TTCTTGGTCTCTAGTTCAGCCA	59.0	192	75	phosphatidylinositol 3- and 4-kinase family protein expressed
OsISM00222	LOC_Os01g01689	365525	TGGCTGAACTAGAGACCAAGAA	TTCAAGCTGACAAATGCTGC	59.6	170	81	phosphatidylinositol 3- and 4-kinase family protein expressed
OsISM00223	LOC_Os01g01689	365657	CACTACCTAGGGAGATCCGC	TGACAATTAGGATCGACTGGAA	59.2	478	83	phosphatidylinositol 3- and 4-kinase family protein expressed
OsISM00224	LOC_Os01g01689	366138	TGAAGAAGGTTCCTTTCCACAT	CCTTCGGAGCATTAATTCCA	60.0	166	75	phosphatidylinositol 3- and 4-kinase family protein expressed
OsISM00225	LOC_Os01g01689	366358	GAAATGATGACCTGCGACAA	TCAGATGTATCTCTGTGATTTTGC	59.0	771	86	phosphatidylinositol 3- and 4-kinase family protein expressed
OsISM00226	LOC_Os01g01689	367098	TGTTCTTGCAAAATCACAGAGA	CCATTCAACAACACCAGCAC	59.3	249	96	phosphatidylinositol 3- and 4-kinase family protein expressed
OsISM00227	LOC_Os01g01689	367327	GTGCTGGTGTTGTTGAATGG	TCCATATCTTCCATGTGCTCC	60.0	187	93	phosphatidylinositol 3- and 4-kinase family protein expressed
OsISM00228	LOC_Os01g01689	367530	ATCATCAATGCAGGGAGACC	AAACGCCCTCCTCTTGTCTT	60.1	153	50	phosphatidylinositol 3- and 4-kinase family protein expressed
OsISM00229	LOC_Os01g01689	367663	AAGACAAGAGGAGGGCGTTT	GCAGGCTGCAAGAATCTCTC	60.2	185	86	phosphatidylinositol 3- and 4-kinase family protein expressed
OsISM00230	LOC_Os01g01689	367874	ACACAAGAAGTGTGGCAGCA	TTCTGGGAATGACGATCTCC	60.3	171	68	phosphatidylinositol 3- and 4-kinase family protein expressed
OsISM00231	LOC_Os01g01689	368096	TGCATTTGAACAGGGTCTCA	CCTTCAACGCCAGTAACTCC	60.0	297	97	phosphatidylinositol 3- and 4-kinase family protein expressed
OsISM00232	LOC_Os01g01689	368435	GGCAAATAAAGAAGCGTTGG	GCCTTCAGTGGTGAAAGAGC	59.9	167	82	phosphatidylinositol 3- and 4-kinase family protein expressed
OsISM00233	LOC_Os01g01689	368582	GCTCTTTCACCACTGAAGGC	GTCATCCAAGCACGAGTCAA	59.9	767	67	phosphatidylinositol 3- and 4-kinase family protein expressed
OsISM00234	LOC_Os01g01689	369392	TCCTCCGTGTTAAGCAGAAA	GTCCGCATCTACTGCATCCT	59.4	218	99	phosphatidylinositol 3- and 4-kinase family protein expressed
OsISM00235	LOC_Os01g01689	369590	AGGATGCAGTAGATGCGGAC	TTTTTGCCTTGGCTCAGATT	60.0	118	80	phosphatidylinositol 3- and 4-kinase family protein expressed

ISM IDs	Rice gene locus IDs	Physical positions (bp) on chromosomes	Forward primers (5'-3')	Reverse primers (5'-3')	Melting tempearature (T˚C)	Expected product size (bp) in PCR	Expected product size (bp) in Q-PCR	Putative Functions
OsISM00236	LOC_Os01g01700	371032	AGATCAAGAAGTGGAACGCC	AGATCCATGATGTGGTTGCG	60.6	185	86	anaphase-promoting complex subunit 11 putative expressed
OsISM00237	LOC_Os01g01700	371191	ATCTGCCGCAACCACATC	GACAGTGCACTCCTCACTGG	60.1	186	79	anaphase-promoting complex subunit 11 putative expressed
OsISM00238	LOC_Os01g01700	371361	TGAGGAGTGCACTGTCGCT	GCTGATGCAATGGAAGTGAA	60.3	1635	59	anaphase-promoting complex subunit 11 putative expressed
OsISM00239	LOC_Os01g01700	373002	CTCAAGACTCGCCAAGTGTG	AGCAGGTGGTGTCTAGTGCC	60.0	163	76	anaphase-promoting complex subunit 11 putative expressed
OsISM00241	LOC_Os01g01780	411886	CTTCCACTTCCATCCTCCAG	TGTTGTAAACCCAGATGGCA	59.8	238	51	exostosin family protein putative expressed
OsISM00242	LOC_Os01g01780	412184	ATGGAGGAAACTGACCCTCC	GAGAAAAGGAGCTGATTCCG	59.7	250	61	exostosin family protein putative expressed
OsISM00243	LOC_Os01g01780	412413	GCGGAATCAGCTCCTTTTCT	GGTCCTTCATGTCATGTCCC	60.5	167	92	exostosin family protein putative expressed
OsISM00244	LOC_Os01g01780	412564	CATGACATGAAGGACCATGC	CAGCTGCTGTTGACCAAAGA	60.1	170	71	exostosin family protein putative expressed
OsISM00245	LOC_Os01g01780	412811	CTGTTCCGCAATGTGTCTGT	TGGAAAATAGGTTTTGCTCCAT	59.8	580	96	exostosin family protein putative expressed
OsISM00246	LOC_Os01g01780	413602	TCCACCAAGTTCCCCTATTG	TCATGCAATTTGGTAGTGTATGG	59.8	239	93	exostosin family protein putative expressed
OsISM00247	LOC_Os01g01800	421642	ACCACCCCTCCTCCTCCT	CTACTTCAGTGGTGTGCGGA	60.4	235	72	expressed protein
OsISM00248	LOC_Os01g01800	421853	TATTTCCGCACACCACTGAA	CGCTGGGTGTTACTACATGG	59.6	195	97	expressed protein
OsISM00249	LOC_Os01g01800	422247	AGCAGACCATCAGGCAAAAC	AGCAAACAAGACCATCCCAC	60.1	240	94	expressed protein
OsISM00250	LOC_Os01g01800	422521	CAGGCATAGAAAAGAAGGCG	AGGGATGATGGATGATACAGG	59.3	154	76	expressed protein
OsISM00251	LOC_Os01g01800	422654	CCTGTATCATCCATCATCCCT	GGGAAAGGCAACAAAAATGA	59.3	178	80	expressed protein
OsISM00253	LOC_Os01g01800	423019	GCATTTGATCAGCAGCAACA	GCATATAGTGACATAAGCAAAACTCA	59.9	719	98	expressed protein
OsISM00254	LOC_Os01g01800	423767	TTCATATCTTCGTTGCTGCCT	CCTGCAGAAAGGGACACATT	60.0	379	89	expressed protein
OsISM00255	LOC_Os01g01800	424072	TCAACACATCAAGTTGCTTCTG	TGCACAAAAGGAAACTTCAAAA	59.4	292	97	expressed protein

ISM IDs	Rice gene locus IDs	Physical positions (bp) on chromosomes	Forward primers (5'-3')	Reverse primers (5'-3')	Melting tempearature (T°C)	Expected product size (bp) in PCR	Expected product size (bp) in Q-PCR	Putative Functions
OsISM00256	LOC_Os01g01800	424406	GATTACTCCATGGCAAACTGAA	AAATAGGTTAGAGCATTTCTGCAA	58.8	152	79	expressed protein
OsISM00257	LOC_Os01g01800	424648	AGGTGATTGGGGTGTAGCAG	CGTTTAATGTTCTTGTGATGCC	59.8	832	72	expressed protein
OsISM00258	LOC_Os01g01800	425697	TAATTTGGTATCCGGCATTG	CATCCTCAGCAGCTACATATTCA	58.9	184	99	expressed protein
OsISM00259	LOC_Os01g01800	425894	CATAGAGGGCCTTCGAATCA	TTTGGACAGTGAGATTGCCA	60.2	202	73	expressed protein
OsISM00260	LOC_Os01g01800	426066	CAAACTTTCATGGCAATCTCA	TGCTCCTATGCCAACAGAAA	59.1	204	98	expressed protein
OsISM00261	LOC_Os01g01800	426258	GGCATAGGAGCAAGACAACC	CCCTGCAATAGGAAACTCAAA	59.5	1075	99	expressed protein
OsISM00262	LOC_Os01g01800	427286	TTCCAATGTTAAGATTCGTCCTT	TTATTCAAAGTCGGTTGGGTG	59.4	321	76	expressed protein
OsISM00263	LOC_Os01g01800	427586	CACCCAACCGACTTTGAATAA	TGCATGAAATCATAAGAAATTAGTGAG	59.9	175	77	expressed protein
OsISM00264	LOC_Os01g01800	427810	GTGGGAACCAGCTATTTTGG	CCACATTTTTGTATGTCTACAGCA	59.3	740	65	expressed protein
OsISM00265	LOC_Os01g01800	428582	CCAGATTAATATTCCTTACGGGC	GCCTGCAAAATTGGAGAACA	60.6	686	93	expressed protein
OsISM00266	LOC_Os01g01800	429201	GCCAATCATAATTCATAACGAACA	TTTAAGGAACGTGAGACGACA	59.3	695	79	expressed protein
OsISM00267	LOC_Os01g01830	440397	GACATGTACCATGGCGTCC	CTAGCTCCACCTGACTTGCC	59.9	176	98	OsPOP1 - Putative Prolyl Oligopeptidase homologue expressed
OsISM00268	LOC_Os01g01830	440693	ACAACTCCGGACTCCAAGC	CCCATCCTTGCTTAGTGCAT	60.2	972	99	OsPOP1 - Putative Prolyl Oligopeptidase homologue expressed
OsISM00269	LOC_Os01g01830	441754	TCGTGTGATGAACATTGCTG	TTTCCATCATGGGTCCAACT	59.7	199	88	OsPOP1 - Putative Prolyl Oligopeptidase homologue expressed
OsISM00270	LOC_Os01g01830	441933	AGTTGGACCCATGATGGAAA	TGTTTCAGTTCCAGCATCCA	60.2	156	82	OsPOP1 - Putative Prolyl Oligopeptidase homologue expressed
OsISM00271	LOC_Os01g01830	442158	TGGAAAGATCCTGAACACCC	GACAGGATCGCAACCTTCAT	60.0	2047	97	OsPOP1 - Putative Prolyl Oligopeptidase homologue expressed
OsISM00272	LOC_Os01g01830	444717	TTCAGTTCCTGGATTTGATCG	GGAATTTTGGTTCCATCCTT	58.9	187	76	OsPOP1 - Putative Prolyl Oligopeptidase homologue expressed
OsISM00273	LOC_Os01g01830	445206	GTGGAAGCAATGGTGGTCTT	GCACACCCAAAGAGATCAGG	60.3	390	65	OsPOP1 - Putative Prolyl Oligopeptidase homologue expressed

ISM IDs	Rice gene locus IDs	Physical positions (bp) on chromosomes	Forward primers (5'-3')	Reverse primers (5'-3')	Melting tempearature (T˚C)	Expected product size (bp) in PCR	Expected product size (bp) in Q-PCR	Putative Functions
OsISM00274	LOC_Os01g01830	445618	GACATGCTCCGTTTTCACAA	CCATAGTCGGTAGTCCAGGC	59.6	143	54	OsPOP1 - Putative Prolyl Oligopeptidase homologue expressed
OsISM00275	LOC_Os01g01830	445741	GCCTGGACTACCGACTATGG	TCCTCACATTATGAAGGGGG	59.7	619	83	OsPOP1 - Putative Prolyl Oligopeptidase homologue expressed
OsISM00276	LOC_Os01g01830	446437	GTTGTGCCATTGCACTCATT	CCTGTGGTGTATCCTCAATGC	60.0	1275	77	OsPOP1 - Putative Prolyl Oligopeptidase homologue expressed
OsISM00277	LOC_Os01g01830	447753	GGAGCTGGCAGACCAACTAA	CGCCATGAAGCTATACCGAT	60.2	134	61	OsPOP1 - Putative Prolyl Oligopeptidase homologue expressed
OsISM00278	LOC_Os01g01840	454877	CATCCCCAAAGAACACTGCT	GAGCTTCTTGATGTAGGCCG	60.0	865	95	helix-loop-helix DNA-binding domain containing protein expressed
OsISM00279	LOC_Os01g01840	456043	ACATCATCTCCGCCAACTTC	CGAAGCCTCTATGCCAATTC	59.9	268	93	helix-loop-helix DNA-binding domain containing protein expressed
OsISM00280	LOC_Os01g01840	456291	GAATTGGCATAGAGGCTTCG	TTACTCCACGTTGCAAGAGC	59.4	1914	71	helix-loop-helix DNA-binding domain containing protein expressed
OsISM00281	LOC_Os01g01840	458233	TTTGGTGTTGGGTCTGGAGT	GTCTTCGGATCTCGCATCTC	60.2	1238	97	helix-loop-helix DNA-binding domain containing protein expressed
OsISM00282	LOC_Os01g01850	467611	ATTGCCACATGCGACGAG	CTCCATGCGAAGACGTCAG	61.2	149	75	transposon protein putative unclassified
OsISM00283	LOC_Os01g01870	477777	CTCCACCTCCTCCTCCTCAT	TTGAGCTGCTTGATGTACGC	60.4	1594	97	helix-loop-helix DNA-binding domain containing protein expressed
OsISM00284	LOC_Os01g01890	484238	CGGACAACGTAGTCAGGATCT	AAGCTGAGGAAGCTCTCGAA	59.3	244	95	expressed protein
OsISM00285	LOC_Os01g01910	495113	AGCGAGGACGATGGTGAC	GTGCATCTGTAGGTTCGCGT	60.5	448	98	hypothetical protein
OsISM00286	LOC_Os01g01910	495579	AAGTACAAGAGCGTGCCCAT	CCTGCACCTGTCCCAATTT	60.3	280	88	hypothetical protein
OsISM00287	LOC_Os01g01920	497847	CTACTACGGCTGCTTCGACC	CCGTATCCACAAACTCACGA	59.8	1622	99	HD domain containing protein 2 putative expressed
OsISM00288	LOC_Os01g01920	499429	TTCTCTGATGTGCAGTTGGC	CCAAGTTGTTTTAGATCCCGC	60.4	171	75	HD domain containing protein 2 putative expressed
OsISM00289	LOC_Os01g01920	499559	TGCATTGTGTCATTCAGGCT	CTCAAAACGTGTGTGAACGG	60.2	1644	85	HD domain containing protein 2 putative expressed
OsISM00290	LOC_Os01g01920	501229	TGGGGAGGCTATGAACAATC	TATCAACACGGTCAATGCCA	60.4	158	63	HD domain containing protein 2 putative expressed
OsISM00291	LOC_Os01g01920	501354	AGGGAGAAGAGCTTGGCATT	AAACAAATGACTGAAGGGGC	59.7	165	96	HD domain containing protein 2 putative expressed

ISM IDs	Rice gene locus IDs	Physical positions (bp) on chromosomes	Forward primers (5'-3')	Reverse primers (5'-3')	Melting tempearature (T˚C)	Expected product size (bp) in PCR	Expected product size (bp) in Q-PCR	Putative Functions
OsISM00292	LOC_Os01g01920	501533	TCCTCGTGTTGTTCCTGGAT	TCCAGCAACAGTGCAGACAT	60.5	410	58	HD domain containing protein 2 putative expressed
OsISM00293	LOC_Os01g01920	501932	CTGTTGCTGGACAGCATTGT	GCAGTGAACTTAGAGCTGGCA	60.3	351	99	HD domain containing protein 2 putative expressed
OsISM00294	LOC_Os01g01920	502249	AGGAAATGATTGTTGCCAGC	TCGATACCATTCCGACCATT	60.1	193	98	HD domain containing protein 2 putative expressed
OsISM00295	LOC_Os01g01920	502417	TTGCTAATGGTCGGAATGGT	CAGACCACATGCTCTGCAAT	60.1	170	72	HD domain containing protein 2 putative expressed
OsISM00296	LOC_Os01g01920	502545	AGGTTTGACTACATTGGCCG	ATGACTCGCATGCCTTGTAA	59.6	764	87	HD domain containing protein 2 putative expressed
OsISM00297	LOC_Os01g01920	503288	CTTACAAGGCATGCGAGTCA	TGAAGATCAGCACGTGTCGT	60.2	194	94	HD domain containing protein 2 putative expressed
OsISM00298	LOC_Os01g01920	503473	TGATCTTCACCGCACTGTCT	AGTGCATCCACAAGCATCAG	59.6	162	67	HD domain containing protein 2 putative expressed
OsISM00299	LOC_Os01g01920	503665	TTTGCATGCACAAGATCCAG	TGTTCAGTTCATCGTTGGGA	60.5	169	87	HD domain containing protein 2 putative expressed
OsISM00300	LOC_Os01g01920	503836	GCAAAAGGGATCATTCAACG	TCCAGCTTGTCCTTTGGAAC	60.3	250	81	HD domain containing protein 2 putative expressed
OsISM00301	LOC_Os01g01920	504066	GTTCCAAAGGACAAGCTGGA	CTTCTTCCTTCAGCAGCACC	60.2	180	92	HD domain containing protein 2 putative expressed
OsISM00302	LOC_Os01g01920	504401	CTATCCCCAGCATCAAGTTCT	TTGTACGCAGGTAGCAGGTG	59.1	171	92	HD domain containing protein 2 putative expressed
OsISM00303	LOC_Os01g01920	504555	CTGCTACCTGCGTACAACCA	TCTCGAAGGCTTCTGACACC	60.2	178	86	HD domain containing protein 2 putative expressed
OsISM00304	LOC_Os01g01925	506288	AACACTGTTGCCGTGCTG	AAGAATCGTACCTTCAGGCG	59.4	1466	87	expressed protein
OsISM00305	LOC_Os01g02000	537030	TTTGGCTCCAATAATGCTGA	TCACCTCCGTCTCGTAAACC	59.7	209	83	phosphate transporter 1 putative expressed
OsISM00306	LOC_Os01g02000	537452	ACGACCCCTCCATTTCTTCT	TCACGTACCTCGTGGACTCA	60.1	1420	64	phosphate transporter 1 putative expressed
OsISM00307	LOC_Os01g02000	539172	ACGCCGAGAAGATGATCAAG	ATCTTCACGAACGCCATCAT	60.4	203	98	phosphate transporter 1 putative expressed
OsISM00308	LOC_Os01g02000	539559	CAACGGAAGGAGTCACACAC	CATGATGCAGTAGCCCAAGA	59.5	169	85	phosphate transporter 1 putative expressed
OsISM00309	LOC_Os01g02000	539774	TGGAGACCGTCTACCCTGTT	TGCAGGAACATTAGGCTGAA	59.5	143	47	phosphate transporter 1 putative expressed

ISM IDs	Rice gene locus IDs	Physical positions (bp) on chromosomes	Forward primers (5'-3')	Reverse primers (5'-3')	Melting tempearature (T˚C)	Expected product size (bp) in PCR	Expected product size (bp) in Q-PCR	Putative Functions
OsISM00310	LOC_Os01g02000	540112	CATCCCCGGATTCTTGCT	GAACTGAAACCGAGTGGAGC	60.4	292	83	phosphate transporter 1 putative expressed
OsISM00311	LOC_Os01g02000	540384	GCTCCACTCGGTTTCAGTTC	CAGAGCTGATCTGCCATGAA	60.0	225	98	phosphate transporter 1 putative expressed
OsISM00312	LOC_Os01g02000	540552	GGTTTCTGAATGTCTGCAGGT	CGACGTACTCGAGGCTTCTT	59.4	217	93	phosphate transporter 1 putative expressed
OsISM00313	LOC_Os01g02000	540844	TGGCCTATGCAGTTTCCTTC	CTCTCATCGAACCACCGTCT	60.2	314	71	phosphate transporter 1 putative expressed
OsISM00314	LOC_Os01g02000	541348	AAGAATCCATGGCTGAGGAA	CATGCAAGCCTTAGCACAAG	59.6	386	90	phosphate transporter 1 putative expressed
OsISM00315	LOC_Os01g02000	541794	TCTAGCTGCTCTCGAGGTGA	TTTTTACCGCCCTGAATTTG	59.5	231	96	phosphate transporter 1 putative expressed
OsISM00316	LOC_Os01g02020	548350	CTCCCTCTCCTCCCTCCC	GCTCCACATTTGCTCTCCTG	61.0	155	75	acetyl-CoA acetyltransferase cytosolic putative expressed
OsISM00317	LOC_Os01g02020	548619	CCGTCAACAAAGTTTGCTCC	AACAACATCATGCATCCCAA	60.2	218	81	acetyl-CoA acetyltransferase cytosolic putative expressed
OsISM00318	LOC_Os01g02020	548862	CCAATGCCCCCAAATATGTC	ATACACATCCCACAGCCCAT	61.1	200	99	acetyl-CoA acetyltransferase cytosolic putative expressed
OsISM00319	LOC_Os01g02020	549078	GAATGTGTGCCGAGTTGTGT	ATTGCACGTTCATTGCTCTG	59.7	173	86	acetyl-CoA acetyltransferase cytosolic putative expressed
OsISM00320	LOC_Os01g02020	549262	ATAGCGGCACATTTTCTTGG	GTGGTTTCCCTCTACCGGA	60.0	164	61	acetyl-CoA acetyltransferase cytosolic putative expressed
OsISM00321	LOC_Os01g02020	549404	TCTTCCGGTAGAGGGAAACC	TTTCCTCAGCTTCGCTGAAT	60.3	310	79	acetyl-CoA acetyltransferase cytosolic putative expressed
OsISM00322	LOC_Os01g02020	549748	CTGCCGGCAATTCTTCTAGT	TGCAATAACTTGAAGGCCAA	59.4	424	93	acetyl-CoA acetyltransferase cytosolic putative expressed
OsISM00323	LOC_Os01g02020	550169	GCAAGGATCAGAGGGTATGC	CCGCATTTGATACAGCCTTT	59.9	309	92	acetyl-CoA acetyltransferase cytosolic putative expressed
OsISM00324	LOC_Os01g02020	550458	AAAGGCTGTATCAAATGCGG	GCTTCTGGTTGGCTAAAGCA	60.3	181	93	acetyl-CoA acetyltransferase cytosolic putative expressed
OsISM00325	LOC_Os01g02020	550609	GACAGGTCGTTGCTTTAGCC	CAATAGGATGGCCCAGAGAA	60.0	179	94	acetyl-CoA acetyltransferase cytosolic putative expressed
OsISM00326	LOC_Os01g02020	550791	GCAGTGGTGCGAGAATCATA	ACCACCTCCATTGCAAACTC	59.9	299	90	acetyl-CoA acetyltransferase cytosolic putative expressed
OsISM00327	LOC_Os01g02020	551070	GAGTTTGCAATGGAGGTGGT	CAACTTCACAACGAGGAACG	59.7	302	86	acetyl-CoA acetyltransferase cytosolic putative expressed

ISM IDs	Rice gene locus IDs	Physical positions (bp) on chromosomes	Forward primers (5'-3')	Reverse primers (5'-3')	Melting tempearature (T°C)	Expected product size (bp) in PCR	Expected product size (bp) in Q-PCR	Putative Functions
OsISM00328	LOC_Os01g02090	588143	ATCAACACCGAGGAGTCCAG	TTGGTGCTAGCAGGAATTTG	59.5	424	96	expressed protein
OsISM00329	LOC_Os01g02090	588539	ATGGAGTGCAAATTCCTGCT	GGTGGAGGTGGTGATGACTT	59.8	580	98	expressed protein
OsISM00330	LOC_Os01g02090	589143	GCACAATGATCCTGCCATAA	TTCCAGCAGAAGGCAAACTT	59.7	149	75	expressed protein
OsISM00331	LOC_Os01g02090	590154	ATTGCCGAAACAAACACCTC	CTGCCTCTACCTCTTCCACG	60.0	745	85	expressed protein
OsISM00332	LOC_Os01g02090	590879	CGTGGAAGAGGTAGAGGCAG	TGGCCATGAAATCAAATTCT	59.0	829	71	expressed protein
OsISM00333	LOC_Os01g02090	591813	TGGAGGATGAGGAAACAGAAA	CCACGTCCAAATGTTCCACT	60.2	185	98	expressed protein
OsISM00334	LOC_Os01g02090	592006	ACGGGAGGTCAAGATTTTCC	GATGCCTTGGGAAATCACC	60.3	135	67	expressed protein
OsISM00335	LOC_Os01g02100	597896	TCTGCAAGCTCTGCAAGAAG	AGTTGGCAGTAACCAGGGTG	59.8	382	92	transposon protein putative unclassified expressed
OsISM00336	LOC_Os01g02100	599143	TGGAAACTTCCATTTGACCA	TCTTGATGGTGTTATCCATTGC	59.4	194	98	transposon protein putative unclassified expressed
OsISM00337	LOC_Os01g02120	611889	GCCGCAACGACCTCTACAC	CCAGTGGAGAAACTCCCTCA	61.0	191	84	osMFT2 MFT-Like2 homologous to Mother of FT and TFL1 gene; contains Pfam profile PF01161: Phosphatidylethanolamine-binding protein expressed
OsISM00338	LOC_Os01g02120	612051	ACCCATCCATGAGGGAGTTT	ACACGAGAGAACGCACCTTT	60.2	429	83	osMFT2 MFT-Like2 homologous to Mother of FT and TFL1 gene; contains Pfam profile PF01161: Phosphatidylethanolamine-binding protein expressed
OsISM00339	LOC_Os01g02120	612424	ATCGTGGTTAACATACCGGG	CGTACCTGTGTATCCCCACC	59.8	1680	95	osMFT2 MFT-Like2 homologous to Mother of FT and TFL1 gene; contains Pfam profile PF01161: Phosphatidylethanolamine-binding protein expressed
OsISM00340	LOC_Os01g02210	662142	ATAATGCATTCAATCCGCCT	AGTCTGACCTGATTGTCGGC	59.8	212	85	retrotransposon protein putative Ty3-gypsy subclass
OsISM00341	LOC_Os01g02210	662480	AGCATACCATGCCCAGAAAA	GTTGGATCTATGTCGGACGG	60.4	169	86	retrotransposon protein putative Ty3-gypsy subclass
OsISM00342	LOC_Os01g02210	662985	AGGACCACCTCATGTTCAGC	ATTGTTTCTTCGTCATCGCC	60.1	137	61	retrotransposon protein putative Ty3-gypsy subclass
OsISM00343	LOC_Os01g02210	663248	GAAAGCACTTGGAGAAGCCA	GTTCCCAAATGAGGAACCAG	59.9	174	97	retrotransposon protein putative Ty3-gypsy subclass
OsISM00344	LOC_Os01g02250	691415	TCCACTTGGATACCCTCCAC	GGCAACTCATTAAGCTTGGG	59.7	263	74	RGA-1 putative
OsISM00345	LOC_Os01g02280	707272	AAGTGGTCCTGTGACTGTGCT	TCGCTTTCGGATTTTGACTT	59.8	139	64	Leucine Rich Repeat family protein

ISM IDs	Rice gene locus IDs	Physical positions (bp) on chromosomes	Forward primers (5'-3')	Reverse primers (5'-3')	Melting tempearature (T˚C)	Expected product size (bp) in PCR	Expected product size (bp) in Q-PCR	Putative Functions
OsISM00346	LOC_Os01g02280	707478	ATGCAAAAGGATGGCAAGAG	ACAGGTCTCTTCGGTTGTGC	60.3	142	58	Leucine Rich Repeat family protein
OsISM00347	LOC_Os01g02280	707643	TGAGAAGGTGAAGGTGCAGA	TTATCCCCTTTTTCGCCTTT	59.7	178	88	Leucine Rich Repeat family protein
OsISM00348	LOC_Os01g02280	708079	CTCGAAAATGGAAAGCTTGG	CCCAATCGGCTTTTGACATA	60.3	439	81	Leucine Rich Repeat family protein
OsISM00349	LOC_Os01g02280	708652	AGGGAGGAGGAAGAATGGAA	CCTTTTCCACATAGCCTCTGA	59.7	177	95	Leucine Rich Repeat family protein
OsISM00350	LOC_Os01g02280	708809	CAGAGGCTATGTGGAAAAGGA	AGAACCATCCAAAGCTCGAA	59.6	162	86	Leucine Rich Repeat family protein
OsISM00351	LOC_Os01g02280	708993	TGGTGGACATTGAGAATGGA	TCGTTCCATATACAGCAGCA	58.9	498	63	Leucine Rich Repeat family protein
OsISM00352	LOC_Os01g02280	709483	TGGAACGATTTGGTAGAATGAA	GGATTCTCATATATGTCCTTCACAA	59.0	207	92	Leucine Rich Repeat family protein
OsISM00353	LOC_Os01g02280	709933	TGACAGATCCAGTGTGGGAA	TGCAGCCTTACTAAAGTCGTG	59.2	230	98	Leucine Rich Repeat family protein
OsISM00354	LOC_Os01g02280	711724	TCCACTTGGATACCCTCCAC	GGCAACTCATTAAGCTTGGG	59.7	263	74	Leucine Rich Repeat family protein
OsISM00355	LOC_Os01g02300	723433	CAAAGCCTGTGCGAGAGATA	TGGCAGAAGTCCAGTTTTGA	59.3	162	78	receptor kinase ORK10 putative expressed
OsISM00356	LOC_Os01g02300	723669	CTCGTTCTGGTATTTGCCGT	AACCGAGGAAGGTCAAAACA	59.9	174	92	receptor kinase ORK10 putative expressed
OsISM00357	LOC_Os01g02310	729686	TGACATGCAAGTACACGAGC	GGTGCCTTCCAATGCTTTTA	59.3	121	80	expressed protein
OsISM00358	LOC_Os01g02310	729787	TAAAAGCATTGGAAGGCACC	GTCCGGATAGATCTGAGGCA	60.1	132	99	expressed protein
OsISM00359	LOC_Os01g02360	745338	GCGTTCAGCTCACAAAGGA	CACACAGAGGTTTGGATTGC	59.6	821	93	resistance-related receptor-like kinase putative expressed
OsISM00360	LOC_Os01g02360	746094	TTGGTATTTGCAGGTTCACG	CACAGTAGCCACCACCAATG	59.8	456	85	resistance-related receptor-like kinase putative expressed
OsISM00361	LOC_Os01g02400	770144	CAATGTGAACGTGAAGGTCG	CCTGCAATGATTGTGACACG	60.7	1221	93	protein kinase domain containing protein expressed
OsISM00362	LOC_Os01g02400	771326	CTTCATATGTCCAGGTCCGC	GAGTGCAGTGGCCACAATTA	60.1	200	92	protein kinase domain containing protein expressed
OsISM00363	LOC_Os01g02420	781239	TCAAACATTCTGCACAAGCC	AAACAACAAATGCGGCTACC	59.9	1305	63	protein kinase domain containing protein

ISM IDs	Rice gene locus IDs	Physical positions (bp) on chromosomes	Forward primers (5'-3')	Reverse primers (5'-3')	Melting tempearature (T˚C)	Expected product size (bp) in PCR	Expected product size (bp) in Q-PCR	Putative Functions
OsISM00364	LOC_Os01g02440	789517	TTAGTTCACAAAGGGGCCAG	TGCATTACCTGCAATGAGGA	60.2	654	69	Ser/Thr receptor-like kinase putative expressed
OsISM00365	LOC_Os01g02440	790126	TTCCTTCCTTCAGGTTCACAA	TAGTGTGGCTGCTGAGGATG	59.9	236	58	Ser/Thr receptor-like kinase putative expressed
OsISM00367	LOC_Os01g02470	796272	TGCTCAAGGTCATCGTCCTA	TGGTTCATCAGGGACATCTTC	59.7	235	56	conserved hypothetical protein
OsISM00368	LOC_Os01g02480	800090	AATGGGTTCAGCCTCATCTC	CTCCGTGGGTTGTTGATCTT	59.5	444	99	retrotransposon protein putative unclassified
OsISM00369	LOC_Os01g02500	806893	TACATGCCGTTGTGCCTAGA	TTGATGGAAATCTGACAGGG	59.4	345	69	glutamyl-tRNA amidotransferase putative
OsISM00370	LOC_Os01g02500	807215	CCACCCTGTCAGATTTCCAT	GTTGAGGCGGGTGATCTT	59.2	339	96	glutamyl-tRNA amidotransferase putative
OsISM00371	LOC_Os01g02500	807833	AACGGTTGGAGCGCTAGAC	TCCATGCTGCAGCTTCTAGT	59.6	521	84	glutamyl-tRNA amidotransferase putative
OsISM00372	LOC_Os01g02500	808336	TAGAAGCTGCAGCATGGACA	TGGATGATGACATGAATTTGC	60.0	191	97	glutamyl-tRNA amidotransferase putative
OsISM00374	LOC_Os01g02520	820925	CCCTCCAAGGTGGTCATTTA	GATCTTCGGCCTCGGATT	60.0	194	93	retrotransposon protein putative unclassified
OsISM00375	LOC_Os01g02540	834965	GAAGTTGAGGCCATTGGAGA	CTTGAAATATGGCCCAAACG	60.3	347	96	retrotransposon protein putative unclassified
OsISM00376	LOC_Os01g02540	835430	GGTGCAAATCGAAGTATGGG	CCATAGTATTCACCACCACGG	60.2	254	93	retrotransposon protein putative unclassified
OsISM00377	LOC_Os01g02560	843263	CCCTGTGGTTTCAGTTCACA	ACCTGCAACAAGAGTGACCC	59.9	1727	73	Ser/Thr receptor-like kinase putative expressed
OsISM00378	LOC_Os01g02560	844950	CTTTCTACATGCAGGACCGC	GCAGTGGCCACAACTAATGA	60.3	304	88	Ser/Thr receptor-like kinase putative expressed
OsISM00379	LOC_Os01g02600	865609	TCATCAGCACTGGTGAGACTG	TGCAATGATTGTGACACGC	60.2	1274	99	Ser/Thr receptor-like kinase putative expressed
OsISM00380	LOC_Os01g02600	866845	TCTTCCTATGTCCAGGTCCG	GAGTGCAGTGGCCACAATTA	59.9	202	78	Ser/Thr receptor-like kinase putative expressed
OsISM00381	LOC_Os01g02650	893426	ATTGCCACATGCGACGAG	CATGCGAAGACGTCAGTGTT	60.9	146	72	retrotransposon protein putative unclassified
OsISM00382	LOC_Os01g02670	903415	CTGTAGACGGAGGCGAAAAG	TTAGCTGCTGCTTGCTGTTG	60.2	547	96	retrotransposon protein putative Ty1-copia subclass expressed
OsISM00383	LOC_Os01g02670	904296	GTGGGCGTCCACTAGAACAC	ATCTTGTGTCTCGGTCCAGG	60.3	939	90	retrotransposon protein putative Ty1-copia subclass expressed

ISM IDs	Rice gene locus IDs	Physical positions (bp) on chromosomes	Forward primers (5'-3')	Reverse primers (5'-3')	Melting tempearature (T˚C)	Expected product size (bp) in PCR	Expected product size (bp) in Q-PCR	Putative Functions
OsISM00384	LOC_Os01g02670	905285	GATGATGCGCTTTCTGGATT	CAACGGCTGTCTCCTCCTTA	60.3	145	98	retrotransposon protein putative Ty1-copia subclass expressed
OsISM00385	LOC_Os01g02670	905848	AGCTACCGTGGGAGATCAAA	TCATCAGTAACAACTGCGGC	59.8	176	73	retrotransposon protein putative Ty1-copia subclass expressed
OsISM00386	LOC_Os01g02670	906951	ACCTCAACAAAACGGCGTAG	GCATAACTGGGTGATCGATTG	60.3	150	97	retrotransposon protein putative Ty1-copia subclass expressed
OsISM00387	LOC_Os01g02670	907129	TGGTCTGGTTCCCCAGTAAA	TTGGAGACTGCTGAACAGTAGAA	59.8	379	98	retrotransposon protein putative Ty1-copia subclass expressed
OsISM00388	LOC_Os01g02670	907590	TGCTTTGAGTGTGACAGAAGAAA	TGTCCTCTCCTAACTCCCCA	59.9	419	78	retrotransposon protein putative Ty1-copia subclass expressed
OsISM00389	LOC_Os01g02670	908527	CCTTATTCAAGTTCAGTTGGTTCA	CCAAATCTCCAGCAAAATCTG	59.6	235	98	retrotransposon protein putative Ty1-copia subclass expressed
OsISM00390	LOC_Os01g02670	908757	TTTGGATAGGAGAAAATCGCTC	GCAGGAACTGGCTTTGTCAT	60.0	340	94	retrotransposon protein putative Ty1-copia subclass expressed
OsISM00391	LOC_Os01g02690	915416	GCATTCAGCTCACAAAGGAA	CCTGAAATGACTTTGACACTCG	59.4	1956	75	resistance-related receptor-like kinase putative
OsISM00392	LOC_Os01g02690	917342	TGCAGGTTCGAGTGTCAAAG	TGAAAGAACAACAAATGCAGC	59.5	295	62	resistance-related receptor-like kinase putative
OsISM00393	LOC_Os01g02710	923816	TCAAAAATTGGAAGCTGCAAG	TAAAAGTTGAGGGTGCCTGG	60.2	196	72	LRk-type protein putative
OsISM00394	LOC_Os01g02750	953138	TTACCTGCTATCACAGATGCAA	GGATTGAGCAGTACCTGCAA	58.7	1111	88	protein kinase domain containing protein expressed
OsISM00395	LOC_Os01g02750	954197	TTTGGTATTTGCAGGTTCACA	AAATGCGGTAACCGATGATG	59.9	393	59	protein kinase domain containing protein expressed
OsISM00396	LOC_Os01g02770	961533	GCATTCAGCTCACAAAGGAA	TGGATTATTGAACCAATACCTGC	59.5	1230	84	resistance-related receptor-like kinase putative expressed
OsISM00397	LOC_Os01g02770	962707	TTGGTTTTTGCAGGTTCACA	ATAACAAATGCAGCAACCGA	59.7	400	63	resistance-related receptor-like kinase putative expressed
OsISM00398	LOC_Os01g02790	976114	ATCTCCGAAGGAGCACTTGA	CTGACAATACCGCCCTCCTA	60.0	1338	98	protein kinase domain containing protein expressed
OsISM00399	LOC_Os01g02790	977396	CAGGCTGCTTAAGTCCATCA	CCTGCAATGATTCTGATACGTG	59.8	678	69	protein kinase domain containing protein expressed
OsISM00400	LOC_Os01g02790	978036	TTGGTATTTGCAGGTTCACG	TTGAGAAACATTTCGACCTTCA	59.7	441	82	protein kinase domain containing protein expressed
OsISM00401	LOC_Os01g02810	987378	CATTCAGCTCGCAAAGGAAT	GAATTATTGAACCAGTACCTGCAA	59.9	876	82	resistance-related receptor-like kinase putative

ISM IDs	Rice gene locus IDs	Physical positions (bp) on chromosomes	Forward primers (5'-3')	Reverse primers (5'-3')	Melting tempearature (T˚C)	Expected product size (bp) in PCR	Expected product size (bp) in Q-PCR	Putative Functions
OsISM00402	LOC_Os01g02810	988198	TTTGGTATTTGCAGGTTCACA	AAAAGAACAACAAATGCGGC	59.6	411	70	resistance-related receptor-like kinase putative
OsISM00403	LOC_Os01g02840	997713	AGCTCGCAAAGGAATCAAAC	CCTGCAATTACTTTGGCATGT	59.7	844	60	resistance-related receptor-like kinase putative
OsISM00404	LOC_Os01g02840	998515	ATGTTTGGCATTTGCAGGTT	TGGCAACCATCAACAAAAGA	60.2	431	87	resistance-related receptor-like kinase putative
OsISM00405	LOC_Os01g02870	1009514	TAGGAACCTCATCTCCCTCG	CTCCACCAGCAAAGAGAAGG	59.6	212	92	auxin-induced protein 5NG4 putative expressed
OsISM00406	LOC_Os01g02870	1009706	CCTTCTCTTTGCTGGTGGAG	GGTGAAAGGTGATACAGCCC	59.7	188	92	auxin-induced protein 5NG4 putative expressed
OsISM00407	LOC_Os01g02870	1009920	CAGTTCCTGCCATCACCTTT	CATGCCTCTTGCCTAGGTCT	59.8	201	70	auxin-induced protein 5NG4 putative expressed
OsISM00408	LOC_Os01g02870	1010294	TGGTCTGGATGGATGGTTCT	AACTGGAGGAGCCCAAAGAT	60.2	207	96	auxin-induced protein 5NG4 putative expressed
OsISM00409	LOC_Os01g02870	1010559	AGCGAGCTGTTCACAATCCT	GCACCATATCTGCAGAGCAA	60.0	166	70	auxin-induced protein 5NG4 putative expressed
OsISM00410	LOC_Os01g02870	1010799	CATCATTCTCGGAGATCAGC	TCACAATCAGAACTGCTCCG	59.2	164	57	auxin-induced protein 5NG4 putative expressed
OsISM00411	LOC_Os01g02880	1015245	TCTCCTACGCCGACGAGC	ATGTGGCATTCGACTCATCA	61.4	1253	88	fructose-bisphospate aldolase isozyme putative expressed
OsISM00412	LOC_Os01g02880	1016673	GGATCAGAATATCATGCCCG	CCTTGGCACCAAGATTCATT	60.1	181	82	fructose-bisphospate aldolase isozyme putative expressed
OsISM00413	LOC_Os01g02880	1016850	AAGGCCTAGATGGTTTGGCT	GGACCACAAGGGATGCTAAC	59.8	181	95	fructose-bisphospate aldolase isozyme putative expressed
OsISM00414	LOC_Os01g02880	1017058	ATGGGGACTTGCTCGATATG	TGGCTCAACAATTGGCACTA	60.1	563	62	fructose-bisphospate aldolase isozyme putative expressed
OsISM00415	LOC_Os01g02880	1017798	ATTGCGAAGCACACCCTTAC	GTTGCCTCCACCTCAGATTG	60.4	205	96	fructose-bisphospate aldolase isozyme putative expressed
OsISM00416	LOC_Os01g02884	1023306	TGATGAAATCCATGAGCGTG	ACAAGTCTTAGCTCAGGCCG	60.1	192	88	ATP-dependent RNA helicase DHX36 putative expressed
OsISM00417	LOC_Os01g02884	1023502	ATGAGTGCAACCCTTAACGC	GAAATCGACTCCGAACAGGA	60.2	859	98	ATP-dependent RNA helicase DHX36 putative expressed
OsISM00418	LOC_Os01g02884	1024472	AGAGCCAAATTGCTTCGGTT	TCACGAGTTCGTGCACTGTAG	60.6	156	74	ATP-dependent RNA helicase DHX36 putative expressed
OsISM00419	LOC_Os01g02884	1024812	CTGCTTGCATGTCATGGTTC	TTCCTTACACCAGGCTCAGG	60.3	583	75	ATP-dependent RNA helicase DHX36 putative expressed

ISM IDs	Rice gene locus IDs	Physical positions (bp) on chromosomes	Forward primers (5'-3')	Reverse primers (5'-3')	Melting tempearature (T°C)	Expected product size (bp) in PCR	Expected product size (bp) in Q-PCR	Putative Functions
OsISM00420	LOC_Os01g02884	1025524	CTGGATCTCCAAGGCTTCTG	GCTTCATACACGCATTGTGG	60.0	269	97	ATP-dependent RNA helicase DHX36 putative expressed
OsISM00421	LOC_Os01g02884	1025894	CCAGAGCTTTGCAATCACCT	CATTACGATCAAACGCTCCA	60.0	170	82	ATP-dependent RNA helicase DHX36 putative expressed
OsISM00422	LOC_Os01g02884	1026044	TGGAGCGTTTGATCGTAATG	GCTCAACTGGAAGCATGGAT	60.0	171	66	ATP-dependent RNA helicase DHX36 putative expressed
OsISM00423	LOC_Os01g02884	1026304	CCATTCTTGACTCCATTTGACA	CAAGTGCAAGGTGATCGCTA	60.0	308	89	ATP-dependent RNA helicase DHX36 putative expressed
OsISM00424	LOC_Os01g02884	1026801	AAAATCTAGTTCGGGCGGTC	CCCATCCTCCATTGTCTTGA	60.7	410	99	ATP-dependent RNA helicase DHX36 putative expressed
OsISM00425	LOC_Os01g02884	1027191	TCAAGACAATGGAGGATGGG	TCTCATTGAACACCAGCCAG	60.3	206	94	ATP-dependent RNA helicase DHX36 putative expressed
OsISM00426	LOC_Os01g02884	1027464	TGGAGGCAACATAAAACAAG G	TGATGCAAGATCACGACTCAT	59.6	168	89	ATP-dependent RNA helicase DHX36 putative expressed
OsISM00427	LOC_Os01g02884	1027610	CATGAGTCGTGATCTTGCATC	GATGTCCATCCTCGGGTTTT	60.2	657	92	ATP-dependent RNA helicase DHX36 putative expressed
OsISM00428	LOC_Os01g02890	1047109	TGCTACTGATGGGAACATGC	AAACGCGGTCAACAGAGAAG	60.1	302	79	phosphatidylserine synthase putative
OsISM00429	LOC_Os01g02890	1047387	GAAGCTTCTCTGTTGACCGC	TCACGTAGAAGGTGCCTGAA	59.8	413	99	phosphatidylserine synthase putative
OsISM00430	LOC_Os01g02890	1047728	ATTGACAGGGGTGTCTTTGC	CAAATGGCAGGATGTGGTCT	60.4	461	96	phosphatidylserine synthase putative
OsISM00431	LOC_Os01g02890	1048200	ACGGGATGGCAGTTGTTTAC	AAATTGCCTAGCATCATCACG	60.0	987	72	phosphatidylserine synthase putative
OsISM00432	LOC_Os01g02890	1049161	AGACCCGTGATGATGCTAGG	ATATATGCGGCAGTCGGTTC	60.0	188	96	phosphatidylserine synthase putative
OsISM00433	LOC_Os01g02890	1049354	TGATCATCCGAAAAGCAGGT	CATCCAAGGATATGGGCAAT	60.1	198	73	phosphatidylserine synthase putative
OsISM00434	LOC_Os01g02890	1049551	GGTGGGGGAAGGCTATAATG	GCAGCATATGGCGAAAGGT	60.8	183	94	phosphatidylserine synthase putative
OsISM00435	LOC_Os01g02890	1049744	TGAGTGTTGGTGGGATAGCA	AGTCTTCATTCCAGCCCAAA	59.9	654	74	phosphatidylserine synthase putative
OsISM00436	LOC_Os01g02890	1050427	ATGGGTTGGTTTGAGTCGTC	ACTGTGCTGGTGTGAACTGG	59.8	557	75	phosphatidylserine synthase putative
OsISM00437	LOC_Os01g02890	1051133	CGACTTGTCCTTTGGTGGTT	CAACAGAAAGACCCCACCTT	59.5	492	99	phosphatidylserine synthase putative

ISM IDs	Rice gene locus IDs	Physical positions (bp) on chromosomes	Forward primers (5'-3')	Reverse primers (5'-3')	Melting tempearature (T˚C)	Expected product size (bp) in PCR	Expected product size (bp) in Q-PCR	Putative Functions
OsISM00438	LOC_Os01g02890	1051610	GGGGTCTTTCTGTTGGCTTT	ACCATGACGGCATCGACT	60.3	188	99	phosphatidylserine synthase putative
OsISM00439	LOC_Os01g02960	1104134	TGCTACCGACTTCAGTGCAT	CAACCTTGCCTGCTGCTT	59.8	1310	60	expressed protein
OsISM00441	LOC_Os01g02980	1120562	TAGGCTGATCCAACAGGGTC	GGGAGTCTCACAAACCCAAG	59.8	1145	94	hypothetical protein
OsISM00442	LOC_Os01g02980	1121899	GCATGGATGGAGGAGTTTTC	CAGAATCCCCTCTCAAGCAA	59.9	587	84	hypothetical protein
OsISM00443	LOC_Os01g02980	1122466	TTGCTTGAGAGGGGATTCTG	TGCTGTAAAAGTTGATGCGG	60.1	375	90	hypothetical protein
OsISM00444	LOC_Os01g02980	1122866	CAAGCTGCTCACACTCCAAA	ATGGACCTCCTTCACCTCCT	60.1	493	73	hypothetical protein
OsISM00445	LOC_Os01g02980	1123362	AAACAGAATGTCCCTGAACCA	GGACTTCCTTCACCTCCTCC	59.7	150	71	hypothetical protein
OsISM00446	LOC_Os01g02980	1123523	AATGTCCCTGCACCATCTTC	AACACCAGAAGCCTTCACAAA	59.9	597	73	hypothetical protein
OsISM00447	LOC_Os01g02980	1124283	CAACCGTTGAAATCCGCTAT	AACATTGAATTTTTGATCCCAAT	59.3	144	63	hypothetical protein
OsISM00448	LOC_Os01g02980	1124503	CACGGTTTTTCTCGGATTTT	TGAAGATGTGCAGGGTGTCT	59.2	418	98	hypothetical protein
OsISM00449	LOC_Os01g02980	1125103	AAGGTTGTCTTCATTTTGCCA	AGAGCAAATGCTGCCTCAAT	59.8	163	83	hypothetical protein
OsISM00450	LOC_Os01g02980	1125299	GGGACTAGCTGAACTTGCTGA	TCTTTGCATTCCAGCATGAC	59.7	448	86	hypothetical protein
OsISM00451	LOC_Os01g03050	1165313	ATGTCCTCGTCTTCGCCAC	TTTTCGCCAAAACCTGACTC	60.7	561	71	fruit protein PKIWI502 putative expressed
OsISM00452	LOC_Os01g03050	1165865	TGGCGAAAATGAGAACATTG	TTCCACTGGATTCCCAATTA	58.8	173	99	fruit protein PKIWI502 putative expressed
OsISM00453	LOC_Os01g03050	1166063	ACCTGATGATCAATGGACCG	CCCCATGGATGAAGGATTTA	60.2	170	86	fruit protein PKIWI502 putative expressed
OsISM00454	LOC_Os01g03050	1166213	TAAATCCTTCATCCATGGGG	TATCTTTTGGCACACCGTCA	59.8	639	94	fruit protein PKIWI502 putative expressed
OsISM00455	LOC_Os01g03090	1182796	CCAACCCCTCCGGAGATT	GAGTCTGGCACGTCCTTGTC	61.5	252	44	methyltransferase putative expressed
OsISM00456	LOC_Os01g03090	1183128	AAGAACGGAGCCACACAGAT	CGTCCAGCTTACGAGGACAT	60.0	494	63	methyltransferase putative expressed

ISM IDs	Rice gene locus IDs	Physical positions (bp) on chromosomes	Forward primers (5'-3')	Reverse primers (5'-3')	Melting tempearature (T˚C)	Expected product size (bp) in PCR	Expected product size (bp) in Q-PCR	Putative Functions
OsISM00457	LOC_Os01g03090	1183648	GCGTAGCTGCAGTGTACTCG	GTGGTCGATCCCAGTCAGAT	59.9	676	95	methyltransferase putative expressed
OsISM00458	LOC_Os01g03090	1184359	TATCCGTGATGGGTTTGAGC	CCAGAGTTCCTTCATCCATCA	60.5	341	99	methyltransferase putative expressed
OsISM00459	LOC_Os01g03090	1184691	GAACTCTGGACACGATTGGG	ACCAGTATGCCACCAGGAGA	60.5	287	98	methyltransferase putative expressed
OsISM00460	LOC_Os01g03090	1184926	TGATGTATTGGCAGTCGGTT	TCCTGGACCAACTCATCCTT	59.3	379	92	methyltransferase putative expressed
OsISM00461	LOC_Os01g03160	1232131	GAGTGGGACTGGTTCGTCAC	TTGGCACTGAGGAGAAGACA	60.1	2388	86	xylosyltransferase putative expressed
OsISM00462	LOC_Os01g03160	1234511	AGTGCCAAGGCATCTCAACT	TGATTGGTTGCACTCTCTGA	58.9	158	75	xylosyltransferase putative expressed
OsISM00463	LOC_Os01g03160	1234713	CAAGCCACAGAGAAACGTGA	CGATTTAGAATGACCCACGG	60.2	153	72	xylosyltransferase putative expressed
OsISM00464	LOC_Os01g03170	1237797	CCTCTCACTCCCCCACTTTA	GTGATGTGCCCTCTCCTCAT	59.6	539	45	seven in absentia protein family protein expressed
OsISM00465	LOC_Os01g03170	1238973	GAGGGCCTCCTCGTCTTC	TAGATGCTTTTGGCTTCGGT	59.9	1030	52	seven in absentia protein family protein expressed
OsISM00466	LOC_Os01g03170	1240274	CTTGCCTCCAACCTCTCAAG	CACACGATATGCGCCTCA	60.2	363	56	seven in absentia protein family protein expressed
OsISM00467	LOC_Os01g03190	1253499	CTCTGCCTCCTCCCCCTC	GTGTGGCACTTCTTGCCC	61.3	191	99	expressed protein
OsISM00468	LOC_Os01g03220	1300792	CCATTGCAGCCCAGACAA	GCTCCTCCTAATCACACATTCTT T	60.9	131	98	hypothetical protein
OsISM00469	LOC_Os01g03220	1300840	ATCAGGGCGATGCATAAAAG	CCATGTCATATCGAACATTTGAA	59.9	1077	56	hypothetical protein
OsISM00470	LOC_Os01g03240	1312558	CCATGGGAGTCGAACAATCT	GGATACCGATTCTCCAGACG	59.7	619	79	conserved hypothetical protein
OsISM00471	LOC_Os01g03270	1327317	AGGAGACGACCAAGATGACC	CCTGTCCATCCATGACGAC	59.5	229	99	conserved hypothetical protein
OsISM00472	LOC_Os01g03300	1335316	CATGCTCTCCTCTCCCATCT	TCGTCGTAGCTGCATCTCTC	59.3	452	67	seven in absentia protein family protein
OsISM00473	LOC_Os01g03400	1367902	GCAATTGCCACAATCACTTA	TATGTCCCATTGACACGCTC	58.4	282	84	E3 ubiquitin-protein ligase SINA-like 2 putative
OsISM00474	LOC_Os01g03400	1368202	TTGTCACAATTGGCATGGAC	AGAGGAGCAGATGAAATGCC	59.9	420	99	E3 ubiquitin-protein ligase SINA-like 2 putative

ISM IDs	Rice gene locus IDs	Physical positions (bp) on chromosomes	Forward primers (5'-3')	Reverse primers (5'-3')	Melting tempearature (T˚C)	Expected product size (bp) in PCR	Expected product size (bp) in Q-PCR	Putative Functions
OsISM00476	LOC_Os01g03420	1374259	AGCATGGACATGGAGGTCTT	AAGGCGAACACACCAAGTG	59.6	236	95	seven in absentia protein family protein putative
OsISM00477	LOC_Os01g03420	1375270	TGACGAAGACGAGGATGATG	GCCGCTCAAAACAAGAAAAT	59.6	739	56	seven in absentia protein family protein putative
OsISM00479	LOC_Os01g03440	1383136	AATGGAGAGGGAAGATTCGG	GCACCCTCAATTACCTCGTT	59.7	171	77	transposon protein putative unclassified
OsISM00480	LOC_Os01g03440	1383366	CGGTGGTCAAGTCAACCAAT	TGTTGCGGTGATACTTCTCG	60.3	165	91	transposon protein putative unclassified
OsISM00481	LOC_Os01g03440	1383650	AAGGCTTGGAGAGCAAAACA	AGTTCTCCGCACTCTGTCCA	60.5	389	75	transposon protein putative unclassified
OsISM00482	LOC_Os01g03490	1403949	GTGCTCTGCAGAATGACCAA	CCTTGTGCTTACAGCCATCA	59.9	203	98	heavy-metal-associated domain-containing protein putative expressed
OsISM00483	LOC_Os01g03490	1404132	TGATGGCTGTAAGCACAAGG	CATCTAAGGCCACCGAGTACA	60.0	572	69	heavy-metal-associated domain-containing protein putative expressed
OsISM00484	LOC_Os01g03510	1413279	GCAAGGGGAAGTCCTTCGT	GAACTCTCTTGAGTGCCCG	60.8	619	82	WD domain G-beta repeat domain containing protein expressed
OsISM00485	LOC_Os01g03510	1413975	GGACCACCATTTAAATTCAAG C	CTCCCATCAGGAGCATATCG	60.2	607	78	WD domain G-beta repeat domain containing protein expressed
OsISM00486	LOC_Os01g03510	1414665	AAGGCAGTCACACAGGGAGT	TCCCAGACTTTGGCAGTTTT	59.7	597	95	WD domain G-beta repeat domain containing protein expressed
OsISM00487	LOC_Os01g03510	1415615	AGGAACTCGTCACCTCTGGA	TGGCTGACCTCCTACATCAA	59.5	815	99	WD domain G-beta repeat domain containing protein expressed
OsISM00488	LOC_Os01g03510	1416705	TTCCATGTTTGCTTCTGCTG	TCCGAGCTGAGTGGAACAAC	60.7	729	99	WD domain G-beta repeat domain containing protein expressed
OsISM00489	LOC_Os01g03520	1424226	TTATAGTCATTTGGGGCGATG	TGAGGCACCATCATCTTCAC	59.7	163	84	ubiquitin conjugating enzyme protein putative expressed
OsISM00490	LOC_Os01g03520	1424447	CGGATTTGCCTGATGATTCT	AGCTTCCATCCTGCGAATTA	59.9	194	97	ubiquitin conjugating enzyme protein putative expressed
OsISM00491	LOC_Os01g03520	1424663	TGTTGCTTTTGGCTTTGTGA	ATTGTTCTCCCCCTCAGCTT	60.2	166	89	ubiquitin conjugating enzyme protein putative expressed
OsISM00492	LOC_Os01g03520	1424953	GCCCTCCAGACCATCATTAC	TGTTCCATTCCTGCTGTACCT	59.5	279	85	ubiquitin conjugating enzyme protein putative expressed
OsISM00493	LOC_Os01g03520	1425211	AGGTACAGCAGGAATGGAAC A	AGGTCCATTCGATCCTCAAA	59.5	185	80	ubiquitin conjugating enzyme protein putative expressed
OsISM00494	LOC_Os01g03520	1425457	GACTTCCACCTTCCACCTGA	CCTTCCCATCCACATACAGG	60.1	224	98	ubiquitin conjugating enzyme protein putative expressed

ISM IDs	Rice gene locus IDs	Physical positions (bp) on chromosomes	Forward primers (5'-3')	Reverse primers (5'-3')	Melting tempearature (T˚C)	Expected product size (bp) in PCR	Expected product size (bp) in Q-PCR	Putative Functions
OsISM00495	LOC_Os01g03520	1425883	TCTGCTGAGCTTGAAATCCA	TTGGAGAAGTGGCTCTTTGC	60.1	424	82	ubiquitin conjugating enzyme protein putative expressed
OsISM00496	LOC_Os01g03530	1432567	TGGCGAGATAGAAGCACATC	TGCATCTGCATGTATGTAAGAAAA	59.3	174	61	multicopper oxidase domain containing protein expressed
OsISM00497	LOC_Os01g03530	1434116	AACCTCTCCCTCACCAACG	GTTCTTCCACCCCTTCTCGT	60.3	654	99	multicopper oxidase domain containing protein expressed
OsISM00498	LOC_Os01g03549	1436556	ACCATCGGCATGTACGAGAC	GTCCCGTAGGCGAAGACTC	60.4	6196	72	multi-copper oxidase type I family protein putative expressed
OsISM00499	LOC_Os01g03549	1444187	CCCGGCTACGTCTACCACT	GGACACGTACTAGCGCACAA	60.0	188	85	multi-copper oxidase type I family protein putative expressed
OsISM00500	LOC_Os01g03570	1446845	GCGTCACTTGAAAGGGTGAT	AATGTCTGAGCGCAAGGTCT	60.1	231	92	transcription factor X1 putative expressed
OsISM00501	LOC_Os01g03570	1447196	TGAACCTTGAGAAAGAGAAGGA	TCAGCACACTTTCATCTGCC	59.1	404	94	transcription factor X1 putative expressed
OsISM00502	LOC_Os01g03570	1447580	GGCAGATGAAAGTGTGCTGA	GCTTAGCATTCAACTGCTGC	59.4	175	99	transcription factor X1 putative expressed
OsISM00503	LOC_Os01g03570	1447938	AAAGCAATGATGAGCTGCAA	ACACCCATTCTCTTGATGCC	59.8	239	98	transcription factor X1 putative expressed
OsISM00504	LOC_Os01g03570	1448294	GCACCCTTTTAGGGTTGTCA	CGCAAAGTTTCCCATCATCT	60.0	600	72	transcription factor X1 putative expressed
OsISM00505	LOC_Os01g03580	1451457	TGGAAAGGACTCACGAAACA	TGTCGTTACCAGGTTGCTTG	59.5	193	51	transposon protein putative CACTA En/Spm sub-class
OsISM00506	LOC_Os01g03580	1453278	CAAAACAGCGTTGTTCGTGT	AATCATTACGCCTCCACCAG	59.9	159	76	transposon protein putative CACTA En/Spm sub-class
OsISM00507	LOC_Os01g03590	1457763	CCATGTGAGCTGCATATTCC	CGGCCTGCAGTGGTAAGTA	59.5	194	97	transposon protein putative CACTA En/Spm sub-class
OsISM00508	LOC_Os01g03590	1458013	CCTCGAGCTAGATTACCCTGG	GATCCTGAGGAGGAGTCGGT	60.4	233	72	transposon protein putative CACTA En/Spm sub-class
OsISM00509	LOC_Os01g03620	1485846	CATCGGCATGTACCAGAAGA	ACTGCCCGTACACGAACAC	59.6	553	72	multicopper oxidase domain containing protein expressed
OsISM00511	LOC_Os01g03690	1519903	TCGATAAACATCTCGACGCC	ATCTCCATCTGCCTCCCTTC	60.9	184	80	TKL IRAK DUF26-lg.1 - DUF26 kinases have homology to DUF26 containing loci expressed
OsISM00512	LOC_Os01g03710	1533514	GCGACCTCCCATTCCTCT	GAGGTCCTTGTCCGGGTG	60.8	198	75	mannose-6-phosphate isomerase putative expressed
OsISM00513	LOC_Os01g03710	1533790	GAGTTCCGCGTCCTCTGC	CAGCATCTTCAGGTCCAACA	61.0	2502	98	mannose-6-phosphate isomerase putative expressed

ISM IDs	Rice gene locus IDs	Physical positions (bp) on chromosomes	Forward primers (5'-3')	Reverse primers (5'-3')	Melting tempearature (T°C)	Expected product size (bp) in PCR	Expected product size (bp) in Q-PCR	Putative Functions
OsISM00514	LOC_Os01g03710	1536412	TGATTTTTCGCCTGAATGCT	GGGTATTGTTTCTCCAGCGA	60.4	1078	83	mannose-6-phosphate isomerase putative expressed
OsISM00515	LOC_Os01g03710	1537666	TGCAAACTCTTTGCTCTATGC	ACGGAGGGGTGTACCTTCTT	59.1	345	97	mannose-6-phosphate isomerase putative expressed
OsISM00516	LOC_Os01g03730	1565283	ATCATCGTCTGCAAGATCGC	CGCCTTCTCCGACAGGTA	60.6	146	43	nuclease PA3 putative expressed
OsISM00517	LOC_Os01g03730	1565529	GTCCTCTCCACTACGCCAAC	CACATCCCTTGCTGGTGAC	59.9	1531	86	nuclease PA3 putative expressed
OsISM00518	LOC_Os01g03730	1567063	TGTCGGGGCTATCAACAACT	AACATCAGGCTCTCCGTCAG	60.5	196	85	nuclease PA3 putative expressed
OsISM00519	LOC_Os01g03730	1567347	TACCGGAGGAAGGAAAACCT	GTCCTTCATGGCCGTCTCTA	60.1	170	64	nuclease PA3 putative expressed
OsISM00520	LOC_Os01g03730	1567531	CTTGGATACCATGGTGGAGG	GTTGCCACAATTTTCCCAGT	60.0	162	83	nuclease PA3 putative expressed
OsISM00521	LOC_Os01g03730	1567670	GTCACTGGGAAAATTGTGGC	GGCATAATTGCAGGACAGATG	60.4	176	84	nuclease PA3 putative expressed
OsISM00522	LOC_Os01g03730	1567825	CATCTGTCCTGCAATTATGCC	CACGATTGGGTACCGAGAGT	60.2	194	85	nuclease PA3 putative expressed
OsISM00523	LOC_Os01g03780	1587278	ACGAGAGGCTCACCACCTC	CCGCAGGAAGGAGACGAG	61.3	167	93	retrotransposon protein putative unclassified
OsISM00524	LOC_Os01g03780	1588096	CTGAACCCCAATAAGTGCGT	ACGCACTGCACATCCCTAAG	60.6	150	72	retrotransposon protein putative unclassified
OsISM00525	LOC_Os01g03780	1588757	GAAGAGCGCCCTGGAGAT	TAAGACGTGACCACCGTGAC	60.0	206	81	retrotransposon protein putative unclassified
OsISM00526	LOC_Os01g03800	1592912	TCTTCTCAGCCTCCTGTTGC	CAAACCAGCAAACACAAAACC	60.6	195	91	transposon protein putative Mutator sub-class
OsISM00527	LOC_Os01g03800	1593168	ATGCATCAAAGCTACCTGGG	TGTGTGCTCAAAGGGAAGAA	59.8	153	60	transposon protein putative Mutator sub-class
OsISM00528	LOC_Os01g03800	1593468	TATTCGAAGGCATGGTCTGG	GGCAGCAGCTAGTTGACCA	60.6	285	88	transposon protein putative Mutator sub-class
OsISM00529	LOC_Os01g03800	1594442	AAGGCACACAGTTGATTTACAGAA	GGTTGTTGGCTGGATTGTTT	60.0	408	98	transposon protein putative Mutator sub-class
OsISM00530	LOC_Os01g03800	1594830	AAACAATCCAGCCAACAACC	TCTTCTTGACTGGTGCAGCTT	60.0	160	82	transposon protein putative Mutator sub-class
OsISM00531	LOC_Os01g03820	1598181	GTGCCATTCTCAAACTGGGT	TGGCTGACCTCCTTTTAAACC	60.2	373	80	C2 domain containing protein putative expressed

ISM IDs	Rice gene locus IDs	Physical positions (bp) on chromosomes	Forward primers (5'-3')	Reverse primers (5'-3')	Melting tempearature (T°C)	Expected product size (bp) in PCR	Expected product size (bp) in Q-PCR	Putative Functions
OsISM00532	LOC_Os01g03820	1598562	TTGAACAAACGATGGAAGCA	TGGGCCAAACTTCTACCAAA	60.4	180	98	C2 domain containing protein putative expressed
OsISM00533	LOC_Os01g03820	1598735	TGGCCCAATTATATGGAACC	AGTTTTGGTTTTCGATGCTTT	58.7	539	81	C2 domain containing protein putative expressed
OsISM00534	LOC_Os01g03820	1599241	GCAGAAACGTTTAAAGCATCG	GGTGGACAACAACCAAGTGA	59.7	162	79	C2 domain containing protein putative expressed
OsISM00535	LOC_Os01g03820	1599392	TGTTGTCCACCTACCTTGGG	TCATTGCTGTCCCAATCAAA	60.4	614	93	C2 domain containing protein putative expressed
OsISM00536	LOC_Os01g03820	1600023	TGGCAAAATTAGCAAAACCA	TCACCATCAAGGATGGGTAAC	59.4	589	98	C2 domain containing protein putative expressed
OsISM00537	LOC_Os01g03820	1600702	GCTACCCGGTGTCTCAACAT	AAAACACAATCTGCGAGGCT	59.9	203	83	C2 domain containing protein putative expressed
OsISM00538	LOC_Os01g03820	1601228	CACCTACTTGACAAGCTGCG	GTTTTGCAACAGCACCAGAA	59.8	202	96	C2 domain containing protein putative expressed
OsISM00539	LOC_Os01g03820	1601455	TGGAATGGTTGTTCCATTTG	TGCTCAACGTAACTGAGCCA	59.9	856	96	C2 domain containing protein putative expressed
OsISM00540	LOC_Os01g03820	1602421	AACGGTAAATCTGGAAAGTGTGA	CCAAACCGGTCGAGTTGTAT	59.9	179	97	C2 domain containing protein putative expressed
OsISM00541	LOC_Os01g03820	1602778	TCAGGCTTCAAATAGAGCCAA	GCTCAATCCAGGTAGCTTCAA	59.7	375	85	C2 domain containing protein putative expressed
OsISM00542	LOC_Os01g03830	1608028	AACACCGTGTGAGGAGGTCT	ATCTTGAGCCATGAGGAAGG	59.4	292	77	retrotransposon protein putative unclassified
OsISM00543	LOC_Os01g03830	1608575	GTGGAGGCTTTGGACTGGAT	ATTCGTGCCTTCCAAATGAC	60.7	937	99	retrotransposon protein putative unclassified
OsISM00544	LOC_Os01g03830	1613691	TGAGTTCTTGCGGAGTGAGTT	CAAAAGAGACAAAAATGCAAGA	58.7	1080	99	retrotransposon protein putative unclassified
OsISM00545	LOC_Os01g03830	1614669	CCATGTCTACCTTTTGATGCC	GTTGACCAAGATCGACCCC	59.9	554	82	retrotransposon protein putative unclassified
OsISM00546	LOC_Os01g03830	1615225	CGGATCGGGACTAGACTCAG	ATCTTGAGCCATGAGGAAGG	59.5	382	94	retrotransposon protein putative unclassified
OsISM00547	LOC_Os01g03830	1615862	GTGGAGGCTTTGGACTGGAT	GCGGTCATTATAACGATAGTGGA	60.8	1014	88	retrotransposon protein putative unclassified
OsISM00548	LOC_Os01g03840	1624234	GTGGAGAGCTCAACGACCAC	AGCTTCATCCTTCCCTCCAT	60.5	337	85	ZOS1-02 - C2H2 zinc finger protein expressed
OsISM00549	LOC_Os01g03840	1624582	CGAGTGTAGGTTCTGCTCCC	GGTTGAGGGTTTCGGTCTC	59.7	343	96	ZOS1-02 - C2H2 zinc finger protein expressed

ISM IDs	Rice gene locus IDs	Physical positions (bp) on chromosomes	Forward primers (5'-3')	Reverse primers (5'-3')	Melting tempearature (T˚C)	Expected product size (bp) in PCR	Expected product size (bp) in Q-PCR	Putative Functions
OsISM00550	LOC_Os01g03840	1624906	GAGACCGAAACCCTCAACC	TCCCATGTTCACATCCCTG	59.9	228	97	ZOS1-02 - C2H2 zinc finger protein expressed
OsISM00551	LOC_Os01g03890	1656492	CTTCCTCGCCAAGTTCTACG	GTACAGCAGCGACCGGAATA	60.6	186	95	DUF260 domain containing protein putative expressed
OsISM00552	LOC_Os01g03950	1697664	ACATCCCTCTCCTCAGGCTC	TCCGTGATCTGGACTCGTATC	60.4	525	65	glycosyl hydrolase family 31 putative expressed
OsISM00553	LOC_Os01g03950	1698757	CAGTACACGTCCATGATCGG	CCCTCGACCACTGACAAGTT	60.1	1046	99	glycosyl hydrolase family 31 putative expressed
OsISM00554	LOC_Os01g03960	1706624	CTGTGACCACCACCACCAC	TTCGTACAGCGAGACGGAG	60.5	142	99	hypothetical protein
OsISM00555	LOC_Os01g03989	1718081	CGCCGGTGGATCTAGAGTTA	GCACGACAGCCTCCACAT	60.6	122	83	hypothetical protein
OsISM00557	LOC_Os01g04030	1754113	GATAAACCGATTCCACAAGCA	ATTTGCGGACAAGTTGAAGG	60.0	857	68	signal peptidase complex subunit 3 putative expressed
OsISM00558	LOC_Os01g04030	1754950	CCTTCAACTTGTCCGCAAAT	AGCGAATTCTTCGAGTTCTCA	59.7	557	98	signal peptidase complex subunit 3 putative expressed
OsISM00559	LOC_Os01g04030	1755459	GGTGTTCGTTTTTCTGACTGC	AATTCGCTTTGTCCTTGTCG	60.0	660	99	signal peptidase complex subunit 3 putative expressed
OsISM00560	LOC_Os01g04030	1756110	AAGCGAATTTGCAAGTGGAG	CAAGCTGAACTTTCTTCCCG	60.2	177	82	signal peptidase complex subunit 3 putative expressed
OsISM00562	LOC_Os01g04100	1780765	AGGACGTCTACGCTGTCACC	CCATGCACCGGGAAATAG	60.1	205	99	expressed protein
OsISM00563	LOC_Os01g04160	1835025	CCAATGATCAGTCGCATGAC	GTTGGTTGGGCTCATTGTTC	60.2	459	91	expressed protein
OsISM00564	LOC_Os01g04160	1835532	CGTTAGGAAGTCCAACCGAA	TCCACAATGTCAGACTCCAAA	59.6	267	98	expressed protein
OsISM00566	LOC_Os01g04190	1844600	CCATTGGAGAAGGTTCAAGC	CCACTCTGCAGAACACAAATTC	59.7	246	98	transporter family protein putative expressed
OsISM00567	LOC_Os01g04190	1844829	TGTGTTCTGCAGAGTGGTCA	TTGTGCTAACCACCCATCCT	59.7	354	96	transporter family protein putative expressed
OsISM00568	LOC_Os01g04190	1845193	TGCGACAGCAGGTTCTTTTA	GGTTTCCGTTGGATGACAAT	59.6	190	82	transporter family protein putative expressed
OsISM00569	LOC_Os01g04190	1845407	AACAGCTCATGATGTTCGCA	AGCTCAGTTGTTGGGCATGT	60.6	183	75	transporter family protein putative expressed
OsISM00570	LOC_Os01g04190	1845604	CTGCAGATTTCACCCACTGA	TGAAAACAATGTGAATATAACA ACCTG	60.1	466	92	transporter family protein putative expressed

ISM IDs	Rice gene locus IDs	Physical positions (bp) on chromosomes	Forward primers (5'-3')	Reverse primers (5'-3')	Melting tempearature (T°C)	Expected product size (bp) in PCR	Expected product size (bp) in Q-PCR	Putative Functions
OsISM00571	LOC_Os01g04190	1846092	AAGGTGGAGGACAATGTTCG	TGTCGGGCTTAAGTTTGAGG	60.1	152	66	transporter family protein putative expressed
OsISM00572	LOC_Os01g04190	1846349	ACCTAAAAGCTGCCAGCCA	ATAGCTGCGCCCACACTAAC	60.6	270	98	transporter family protein putative expressed
OsISM00573	LOC_Os01g04190	1846663	ACTCTACATCGGTGTTCCGC	AAGGCCATTTCAAAATTGATTC	59.7	130	53	transporter family protein putative expressed
OsISM00574	LOC_Os01g04190	1846782	GAAATGGCCTTTCTGATGGT	GAGAAGCAGTGAACTTTTAAGCAA	59.1	193	99	transporter family protein putative expressed
OsISM00575	LOC_Os01g04190	1846968	GCTTCTCCCTTTTGAAGGCT	AACAGTTAACAATGACAATCAGCA	59.4	165	76	transporter family protein putative expressed
OsISM00576	LOC_Os01g04190	1847193	CAGTTCCAGCATTGCTCCTT	AAGAAGTTCGAAACCTGCCC	60.5	390	95	transporter family protein putative expressed
OsISM00578	LOC_Os01g04200	1849528	TCATCGGTTCATTGGAGTCA	CATTCCTTGGACATCCACCT	59.9	138	65	metallo-beta-lactamase family protein putative expressed
OsISM00579	LOC_Os01g04200	1849752	CATCAAACTGGACAGTTGCC	CGGAAGGGCTGTAATGTGTT	59.6	453	95	metallo-beta-lactamase family protein putative expressed
OsISM00580	LOC_Os01g04200	1850302	GGATGCTGCTCACAAGTTCA	CCACTAACTTCTTTGGAAGGGA	59.8	428	65	metallo-beta-lactamase family protein putative expressed
OsISM00581	LOC_Os01g04200	1850708	TCCCTTCCAAAGAAGTTAGTGG	CAGGGTTGCATCTCTGAACA	59.7	278	86	metallo-beta-lactamase family protein putative expressed
OsISM00582	LOC_Os01g04200	1850977	GCAACCCTGATGCTGTTCTT	TTTCTCCCCCAGCTACTGC	60.3	649	97	metallo-beta-lactamase family protein putative expressed
OsISM00583	LOC_Os01g04200	1851636	AGGTGACCAACAACTGCAAG	TAAAACCCCCATGTGACCAT	59.3	1074	62	metallo-beta-lactamase family protein putative expressed
OsISM00584	LOC_Os01g04200	1852690	ATGGTCACATGGGGGTTTTA	TTGTCCAATGTTGCACTTCC	59.7	254	86	metallo-beta-lactamase family protein putative expressed
OsISM00585	LOC_Os01g04200	1852918	AGGCAAGGAAGTGCAACATT	CGGTGACATGTCCAAGAAAT	59.1	335	88	metallo-beta-lactamase family protein putative expressed
OsISM00586	LOC_Os01g04200	1853280	ATCAACCTATGGCCCAAACA	GTCCGAGCCCCATTCTCTAT	60.3	377	96	metallo-beta-lactamase family protein putative expressed
OsISM00587	LOC_Os01g04200	1853723	CAATGTTCGCCTTCATGTTG	TTTGTTCCACAGCTCACTTCA	59.8	897	91	metallo-beta-lactamase family protein putative expressed
OsISM00588	LOC_Os01g04210	1856663	CTGACTCCGTCTTCTACGGC	CTCTACCAGGTTCGAGTCGC	60.0	184	69	DNA binding protein putative
OsISM00589	LOC_Os01g04220	1864012	GTCTTCTATGGCCGCATGAT	CTCCGCTTCCACCAGGTT	60.6	486	67	DNA binding protein putative

ISM IDs	Rice gene locus IDs	Physical positions (bp) on chromosomes	Forward primers (5'-3')	Reverse primers (5'-3')	Melting tempearature (T°C)	Expected product size (bp) in PCR	Expected product size (bp) in Q-PCR	Putative Functions
OsISM00591	LOC_Os01g04260	1887804	CTACTACTGGAAGCGCCACG	AGAGGCATAGCCTTTGATGC	60.2	2218	97	fatty acid hydroxylase putative expressed
OsISM00592	LOC_Os01g04260	1890207	ACCTGCACACATACCACCAT	CTTGCAAAATCCCATCCAGT	59.3	460	91	fatty acid hydroxylase putative expressed
OsISM00593	LOC_Os01g04280	1901924	CTACGGACAGGAGGACCAGC	TCTGCATCTGCTTCACCATC	61.1	193	96	calmodulin binding protein putative expressed
OsISM00594	LOC_Os01g04280	1902130	ACTGGAGCCACTTCTCCGTA	TCTCACCTCTCGATGTAACGC	60.1	185	82	calmodulin binding protein putative expressed
OsISM00595	LOC_Os01g04280	1902263	AGGAGCTCCAGGCAGGTCT	AACGCCAGCTTCCACATC	60.6	188	95	calmodulin binding protein putative expressed
OsISM00596	LOC_Os01g04280	1902820	CTACGACGGCCCAAGAATAG	AGAACGGGAGGGTAGTGCTT	59.9	191	94	calmodulin binding protein putative expressed
OsISM00597	LOC_Os01g04280	1903084	GTCAGGAACGTGCAGGAGTT	GTCACCTCCCACATGCGAT	61.1	191	99	calmodulin binding protein putative expressed
OsISM00598	LOC_Os01g04280	1903466	AGCGAAACAACCTCCAAGAG	CGGGATAGTTATCTCCGCTG	59.6	176	87	calmodulin binding protein putative expressed
OsISM00599	LOC_Os01g04320	1924712	AAAGAGAGGGGGAGGTTGAA	AGCAGACGATCAACTGAAGGA	60.0	611	90	transposon protein putative unclassified
OsISM00600	LOC_Os01g04460	1983940	GGTACGATGCCAGCACATC	CCAACTTGCCCCTGTTCTTA	60.1	232	91	Ser/Thr protein kinase putative
OsISM00601	LOC_Os01g04460	1984147	GATGGTAAGAACAGGGGCAA	CAAGCAAGAAAGGCGAAAAC	60.0	179	78	Ser/Thr protein kinase putative
OsISM00602	LOC_Os01g04470	1988375	ATTCCTCTTGACATGGCAGC	GCGTTCGTTTTTCCAATGA	59.9	1645	49	hypothetical protein
OsISM00603	LOC_Os01g04480	1993191	CGACGGCTTCCTCTTGAC	CTCGGAGGTGTGCAGAGTTT	60.0	682	92	expressed protein
OsISM00604	LOC_Os01g04480	1993849	TCTCAAACTCTGCACACCTCC	TTGAGCTTTGACCTCCTGCT	60.3	787	93	expressed protein
OsISM00605	LOC_Os01g04480	1994616	AGCAGGAGGTCAAAGCTCAA	TCAAGCTGGTTGTAGCAGACA	59.9	217	59	expressed protein
OsISM00607	LOC_Os01g04490	1998520	ATGGGGTTCTCGTGCAAAT	TTCTTCCTGCCATTGCTCTT	60.1	1704	84	Ser/Thr protein kinase putative expressed
OsISM00608	LOC_Os01g04490	2000205	AGAGCAATGGCAGGAAGAAA	AAGCACATGGAAAGAGCAGG	60.2	623	70	Ser/Thr protein kinase putative expressed
OsISM00609	LOC_Os01g04490	2001071	TCTTGCATGACTCCAAAGGA	ATCCAGAGAACTGTTGGGCA	60.0	158	93	Ser/Thr protein kinase putative expressed

ISM IDs	Rice gene locus IDs	Physical positions (bp) on chromosomes	Forward primers (5'-3')	Reverse primers (5'-3')	Melting tempearature (T˚C)	Expected product size (bp) in PCR	Expected product size (bp) in Q-PCR	Putative Functions
OsISM00610	LOC_Os01g04490	2001812	AACTGGACATGCCTCCAAAG	CGCATTTAACTTGTGAACCG	59.7	156	66	Ser/Thr protein kinase putative expressed
OsISM00611	LOC_Os01g04520	2014117	AGCCGTGCAGACCACCTT	CCACCGATCGGAGATGTAAG	61.2	194	86	Ser/Thr protein kinase putative
OsISM00612	LOC_Os01g04520	2014615	TTATGTTCCAGTGCGTCAGC	GAAAAGGCTTTGACACAGTCG	59.9	577	89	Ser/Thr protein kinase putative
OsISM00613	LOC_Os01g04520	2015262	TGGCAGCCAATGACACTTAG	CCTGACATTCAGCTTCCGAT	60.0	514	55	Ser/Thr protein kinase putative
OsISM00614	LOC_Os01g04520	2015763	GCTGAATGTCAGGGCAATTT	AAACCAAGTGTTTGCTTCCG	60.1	394	85	Ser/Thr protein kinase putative
OsISM00615	LOC_Os01g04530	2021064	TCCTCATAACATGGGGGAAC	ACTTACCAGACTTCTGCGGC	59.6	797	95	expressed protein
OsISM00616	LOC_Os01g04530	2021810	ATGGTTTCCTCTTGACATGG	ATCATGCTTGACTGTGCTGC	58.7	472	79	expressed protein
OsISM00618	LOC_Os01g04540	2023778	ATAACGTCCTCCGGGTTCAT	TCTGAATGGGCAGTATGCAA	60.4	187	94	serine/threonine-protein kinase At1g18390 precursor putative expressed
OsISM00619	LOC_Os01g04550	2036710	GGAAATGCCACCAAAACAAA	TGCAGCTTTGGTATTTCCAG	59.8	181	98	expressed protein
OsISM00620	LOC_Os01g04560	2040448	ATGGAGCCCACGGGATAG	CTCGGTTCCCGAATTGGAT	61.5	324	90	conserved hypothetical protein
OsISM00622	LOC_Os01g04570	2048486	CGGTACAACAACGATTCCG	TCTCCTGCCAGTTTTCTTGC	60.2	1398	97	Ser/Thr protein kinase putative expressed
OsISM00623	LOC_Os01g04570	2049868	GAAAACTGGCAGGAGAACCA	TCCCATGCCATATTAGCACA	60.1	263	90	Ser/Thr protein kinase putative expressed
OsISM00624	LOC_Os01g04570	2051104	GCCACCAAAACAAAATTTCA	GAGCTTGTGCAGCTTTGGTA	58.9	182	99	Ser/Thr protein kinase putative expressed
OsISM00625	LOC_Os01g04580	2053312	GTTCGCCTGCATCTGCTC	ACCTTGTTCCCGGACTTCTT	60.3	1526	70	Ser/Thr protein kinase putative expressed
OsISM00626	LOC_Os01g04580	2054818	AAGAAGTCCGGGAACAAGGT	CCAATAGCACCAACCCAGTC	60.2	268	68	Ser/Thr protein kinase putative expressed
OsISM00627	LOC_Os01g04580	2056242	CTCCTCTGCCTCGGATTTC	ATTCCATCGCCACTTTTCAC	59.9	189	75	Ser/Thr protein kinase putative expressed
OsISM00628	LOC_Os01g04590	2058497	CGGTGTAATCTGGAGCTGTTC	CATCAGATTGCTTCCAACCAT	59.8	404	98	expressed protein
OsISM00629	LOC_Os01g04590	2059039	CTGCAATCCAGCTTTATGGA	TGTCTTGACAGTAGTCGCCG	59.5	964	88	expressed protein

ISM IDs	Rice gene locus IDs	Physical positions (bp) on chromosomes	Forward primers (5'-3')	Reverse primers (5'-3')	Melting tempearature (T°C)	Expected product size (bp) in PCR	Expected product size (bp) in Q-PCR	Putative Functions
OsISM00630	LOC_Os01g04590	2060142	CGAGCAGCTCATCTTCGAGT	AATCCCCTGGCAAGACTACA	60.2	184	88	expressed protein
OsISM00631	LOC_Os01g04590	2060343	TCAGGAGCTGTGATTTGTCG	TCCAGATCGCATAGTGTTGG	59.8	204	92	expressed protein
OsISM00632	LOC_Os01g04590	2060561	CATATCATCCCCTCTCCACG	CTGTTGTTGCCTTTTGGCTC	60.6	170	62	expressed protein
OsISM00634	LOC_Os01g04620	2070972	GTCTCCCTCCCCTGATCTC	GGTACTCCCCTGCGAACAT	59.2	205	99	transposon protein putative unclassified expressed
OsISM00635	LOC_Os01g04620	2072027	CGCCAAGAACCTGTACGTCT	GTTCGACTGCTGCACCACA	61.2	199	98	transposon protein putative unclassified expressed
OsISM00636	LOC_Os01g04640	2079127	CGAACTCACAGGACAACCAA	CCCTCCACAGGATGATGTCT	59.8	231	92	reticulon domain containing protein putative expressed
OsISM00637	LOC_Os01g04640	2079479	CTTCATATGGGCCAATGCTT	CACTTGCCTGGTGGTTCTCT	60.1	288	89	reticulon domain containing protein putative expressed
OsISM00638	LOC_Os01g04640	2079820	TGCTTGTGGAAAGGACATCA	GCGATCACTGACGCTATGAA	60.1	149	67	reticulon domain containing protein putative expressed
OsISM00639	LOC_Os01g04640	2079973	TGCTTCAGCTCCCTGACTCT	TCTCGTACATCACAGGCAGC	60.2	206	68	reticulon domain containing protein putative expressed
OsISM00640	LOC_Os01g04660	2096661	TGTTCTGCAGTCCTCAATGC	GTGTCTGAATCGTGTGAGGG	59.5	961	78	lipid phosphatase protein putative expressed
OsISM00641	LOC_Os01g04660	2097774	ACTGTACCAGCATGGGGTGT	TCTCTCCTAGCGATATAAACGGA	59.9	174	81	lipid phosphatase protein putative expressed
OsISM00642	LOC_Os01g04660	2097953	ATGATCTTCATCACGCAACA	CTGCAGTTATCAGCACCGAG	58.6	157	52	lipid phosphatase protein putative expressed
OsISM00643	LOC_Os01g04660	2098156	CTTCTTTTGGCGGTGTTTTC	GCGTCCATCCTTTAAGAAGC	59.3	209	98	lipid phosphatase protein putative expressed
OsISM00644	LOC_Os01g04660	2098355	GGATGGACGCAAGAGTTTTC	CAGAAATCCAAGTCCAGCAA	59.3	457	62	lipid phosphatase protein putative expressed
OsISM00645	LOC_Os01g04660	2098928	TGACTATCGACACCACTGGG	CAAGTAACAGAGCATCGCCA	59.8	162	74	lipid phosphatase protein putative expressed
OsISM00646	LOC_Os01g04660	2099087	TTGCACTTCTTTCCACCTCC	GAGTTGGCCACTTGAAGCTC	60.1	199	87	lipid phosphatase protein putative expressed
OsISM00647	LOC_Os01g04680	2104308	GTGTCTCGTTGCAATCACCA	AGGAGGAATACTTGCTGCGA	60.4	1413	87	retrotransposon protein putative unclassified
OsISM00648	LOC_Os01g04680	2105741	AGGAGCCCCTAGAAGCTTTG	AGGCTCTTGCGAATTTCTCC	60.4	161	94	retrotransposon protein putative unclassified

ISM IDs	Rice gene locus IDs	Physical positions (bp) on chromosomes	Forward primers (5'-3')	Reverse primers (5'-3')	Melting tempearature (T˚C)	Expected product size (bp) in PCR	Expected product size (bp) in Q-PCR	Putative Functions
OsISM00649	LOC_Os01g04720	2130553	GACGACCCCTTCCTCTCC	CGCAGTCGATGTTGTAGCTG	60.1	154	53	leucine-rich repeat protein-related putative expressed
OsISM00650	LOC_Os01g04720	2131721	AGACGAGGACAGTGCCAAAT	TTCAGGGAATCCTTCAATGC	59.9	160	56	leucine-rich repeat protein-related putative expressed
OsISM00651	LOC_Os01g04720	2131936	GCATGGGAGGGAGTCACTT	TGTAACCTTTTAGTCCTTGACTTGC	60.1	969	86	leucine-rich repeat protein-related putative expressed
OsISM00652	LOC_Os01g04720	2132913	GAAATAAGTCATCTGACAGACTTGG	TAGCAAGTGAAGGTTGCCCT	59.1	482	95	leucine-rich repeat protein-related putative expressed
OsISM00653	LOC_Os01g04720	2133374	TAGGGCAACCTTCACTTGCT	ATGAACCTATGGTGCCAGGA	60.1	852	76	leucine-rich repeat protein-related putative expressed
OsISM00654	LOC_Os01g04720	2134207	CCTGGCACCATAGGTTCATC	GTCTTTCTGGAACTTGCCCA	60.3	159	77	leucine-rich repeat protein-related putative expressed
OsISM00655	LOC_Os01g04720	2134369	ACTCGATTGGCGTACATGGT	CCCAAAATAATGCACAAGCA	60.0	801	91	leucine-rich repeat protein-related putative expressed
OsISM00656	LOC_Os01g04720	2135288	TGATTTTGAGTTCCCCCAAG	GCATTACAGACTTCGCCCTC	59.9	421	84	leucine-rich repeat protein-related putative expressed
OsISM00657	LOC_Os01g04770	2180360	ACTGGTCGCGTCAGCAAC	GGCCTTCATGCCTCTTTTT	60.2	578	99	retrotransposon protein putative unclassified
OsISM00658	LOC_Os01g04860	2244885	GGTGGAGGAGGAGGAGACTG	CCCATCCTGGTATCTCTCCA	60.5	166	70	DUF647 domain containing protein putative expressed
OsISM00659	LOC_Os01g04860	2245037	GATACCAGGATGGGGTTTCC	CTTCCAATGTGTTCGCCATA	60.0	211	97	DUF647 domain containing protein putative expressed
OsISM00660	LOC_Os01g04860	2245264	AGCAAATTCTTCGATTCCCC	TCCAGGTAATCGTCTGACACA	59.8	631	82	DUF647 domain containing protein putative expressed
OsISM00661	LOC_Os01g04860	2245945	CGCTGGTTACATCAACCCTT	CCTGTAAAGGACCCAACACC	59.6	408	50	DUF647 domain containing protein putative expressed
OsISM00662	LOC_Os01g04860	2246316	GATTGACGCAGGCTGTAGGT	AAAGCCCCTATGCCATCTTT	60.1	477	98	DUF647 domain containing protein putative expressed
OsISM00663	LOC_Os01g04860	2246774	AAGATGGCATAGGGGCTTTT	GCATACATCCGCCACTTCTT	60.0	233	89	DUF647 domain containing protein putative expressed
OsISM00664	LOC_Os01g04860	2247006	CCGATTTCATTGGAAGTGCT	GACGCAAGTGGGAGGAAGTA	60.2	187	77	DUF647 domain containing protein putative expressed
OsISM00665	LOC_Os01g04860	2247173	TACTTCCTCCCACTTGCGTC	GGATAACACGGAAGGATGGA	60.0	194	80	DUF647 domain containing protein putative expressed
OsISM00666	LOC_Os01g04860	2247368	AAATCACTTCGCGGAATCTG	GGCCTAACAACTGAGCTCCT	59.4	354	81	DUF647 domain containing protein putative expressed

ISM IDs	Rice gene locus IDs	Physical positions (bp) on chromosomes	Forward primers (5'-3')	Reverse primers (5'-3')	Melting tempearature (T˚C)	Expected product size (bp) in PCR	Expected product size (bp) in Q-PCR	Putative Functions
OsISM00667	LOC_Os01g04860	2247713	TGTTAGGCCTTTCTATCGGTG	TAGAAGACGGACACCAAGCC	59.7	234	96	DUF647 domain containing protein putative expressed
OsISM00668	LOC_Os01g04860	2247952	TTGGTTTCGCTATCAGTCCC	GATCTCACGAGAATCCGAGC	60.0	211	81	DUF647 domain containing protein putative expressed
OsISM00669	LOC_Os01g04880	2260943	GATGCTTCTCGTCGACCAAC	CCGGTCATCCTGTACGCC	61.7	199	76	retrotransposon protein putative unclassified expressed
OsISM00670	LOC_Os01g04880	2261171	ACGACTTCCTCAATTCGGAC	GGAGTTCCTGGTGGAGTCAG	59.4	1599	68	retrotransposon protein putative unclassified expressed
OsISM00671	LOC_Os01g04880	2262813	CAAACTGGAACCCCAAAGAC	GGGTTGTTCTTGATGGAGGA	59.7	520	80	retrotransposon protein putative unclassified expressed
OsISM00672	LOC_Os01g04880	2264370	TCACTTGACATGGCTCTTCG	GAGGCCGTAAATTGTTTGGA	60.0	150	62	retrotransposon protein putative unclassified expressed
OsISM00673	LOC_Os01g04910	2270290	TCTTCTCCGACGTCTCCAAC	CAGTGATGCGTTGAGGAATG	60.3	214	90	TP53RK-binding protein putative expressed
OsISM00674	LOC_Os01g04910	2270448	AGCTGATGCAGTCAGGGAAG	GAGAACCGGGAACACATCC	60.4	189	78	TP53RK-binding protein putative expressed
OsISM00675	LOC_Os01g04910	2270668	GGAGGGAGTCTCTGACCACA	GATTCCGCATCGTTTCAAAG	60.4	206	96	TP53RK-binding protein putative expressed
OsISM00676	LOC_Os01g04910	2270870	AATCTCCGACGACACGAGTT	TCTCAGTGCCACAGATGAGC	59.9	900	93	TP53RK-binding protein putative expressed
OsISM00677	LOC_Os01g04910	2271793	AGAGCAAACCAGCCACAGAT	TGCATCGGGCAGTGTAGATA	60.1	171	76	TP53RK-binding protein putative expressed
OsISM00678	LOC_Os01g04920	2274439	GCTCTTCGTCGAGCCGTC	CCGGTTCTTGTACCCAGAGA	61.2	475	50	glycosyl transferase group 1 domain containing protein expressed
OsISM00679	LOC_Os01g04920	2274920	AACTTCATCAAGCACTTGCG	CTTGGCACCATGGAATTCTT	59.5	174	88	glycosyl transferase group 1 domain containing protein expressed
OsISM00680	LOC_Os01g04920	2275084	TGGTGCCAAGGTTATTGGTT	TGCAACCTCCGAAATAATCC	60.1	539	98	glycosyl transferase group 1 domain containing protein expressed
OsISM00681	LOC_Os01g04920	2275640	CATTCATGCATCCTCACCTG	TAGAGGGACACCGAGCAGTT	60.0	160	71	glycosyl transferase group 1 domain containing protein expressed
OsISM00682	LOC_Os01g04920	2275780	AACTGCTCGGTGTCCCTCTA	TGCCACATTGGCTCTACAAG	59.9	362	95	glycosyl transferase group 1 domain containing protein expressed
OsISM00683	LOC_Os01g04920	2276124	TGTAGAGCCAATGTGGCAAG	GCTGATAGCAACAGATGGCA	59.9	166	77	glycosyl transferase group 1 domain containing protein expressed
OsISM00684	LOC_Os01g04920	2276289	CAAAGATTTTGAAACTGCCCA	GGATGGAAACTGGCTGAATC	59.8	185	82	glycosyl transferase group 1 domain containing protein expressed

ISM IDs	Rice gene locus IDs	Physical positions (bp) on chromosomes	Forward primers (5'-3')	Reverse primers (5'-3')	Melting tempearature (T˚C)	Expected product size (bp) in PCR	Expected product size (bp) in Q-PCR	Putative Functions
OsISM00685	LOC_Os01g04920	2276475	AAGTTCCGCAGTCACGAAAT	CCAAAGCGTCCTACATGGAT	59.8	175	81	glycosyl transferase group 1 domain containing protein expressed
OsISM00686	LOC_Os01g04920	2276630	ATCCATGTAGGACGCTTTGG	TGCAATTCTTACTCCAGGCA	59.7	218	82	glycosyl transferase group 1 domain containing protein expressed
OsISM00687	LOC_Os01g04920	2276810	GCAGGGTAATGGATAGGCTG	GCATCCCCTCAAACATCTTC	59.5	296	91	glycosyl transferase group 1 domain containing protein expressed
OsISM00688	LOC_Os01g04950	2292695	CACATCTCACCGGTACACCA	ACTCCAATGGCGACTTGCT	60.6	204	99	peptide transporter PTR2 putative expressed
OsISM00689	LOC_Os01g04950	2293035	TGTAATTGACCAGCTGATTAC TAGG	AAAACCGTGGAAACGAAGTG	59.4	183	99	peptide transporter PTR2 putative expressed
OsISM00690	LOC_Os01g04960	2300582	AACAACATAGTGTTTGGCAAC G	TGTAGGATCCTGAGATCCCG	60.0	367	95	transposon protein putative unclassified
OsISM00691	LOC_Os01g04960	2301156	GTCCATCTGGACAGGCAAGT	GTGGTTCCTGTCAATCCCAC	60.2	453	75	transposon protein putative unclassified
OsISM00693	LOC_Os01g04960	2302120	CTAGCAAGGCTTGGTTGCTC	AACCACAACAGGTACGCCTC	60.1	184	98	transposon protein putative unclassified
OsISM00694	LOC_Os01g04960	2302615	CGATGGGGACTTACTGGTGT	GGGTAACCACGTTTCACCTG	60.1	160	65	transposon protein putative unclassified
OsISM00695	LOC_Os01g04960	2302976	CGGACTTAGGCAGGTGTTTC	CTAGCTGTCCCCGTCGAGT	60.1	159	81	transposon protein putative unclassified
OsISM00696	LOC_Os01g04960	2303343	GCACCGTGACCGAGACTACT	GCAAACCTCTGCTCAACCTC	60.2	187	93	transposon protein putative unclassified
OsISM00697	LOC_Os01g04960	2303677	GAGCAGGACCTCGTTTGTCT	AACGCGCTCGAAGGCATA	61.2	187	95	transposon protein putative unclassified
OsISM00698	LOC_Os01g04960	2303931	AGCTCTTCCTCTCACGGAGC	AGACGAAGAGGCCCCAGTA	60.5	144	82	transposon protein putative unclassified
OsISM00699	LOC_Os01g04970	2327657	AGGCTCTGTCGGAGGAGG	ATCTTCTCGCCATGAATGCT	60.2	134	70	hypothetical protein
OsISM00700	LOC_Os01g04980	2337226	GAGGGTCATGTCCTCGCC	TTTTCTTTCTTCTTCCGAGGC	60.8	965	96	transposon protein putative unclassified
OsISM00701	LOC_Os01g05000	2355220	TGATGAAGAGGAAGAGGTTGT C	TATCTAGCCTCTCCAGCGCA	59.8	305	94	hypothetical protein
OsISM00702	LOC_Os01g05030	2365391	AGTTCCACTATTCCAGGCCC	CTGAACTGGCACCAGCATTA	60.1	139	65	transposon protein putative unclassified
OsISM00703	LOC_Os01g05030	2365948	TGTGGAGCTTCAAAATGCTT	GGTAAAAACACACACCTCGTGA	59.2	154	79	transposon protein putative unclassified

ISM IDs	Rice gene locus IDs	Physical positions (bp) on chromosomes	Forward primers (5'-3')	Reverse primers (5'-3')	Melting tempearature (T˚C)	Expected product size (bp) in PCR	Expected product size (bp) in Q-PCR	Putative Functions
OsISM00704	LOC_Os01g05030	2366048	TGGGCTGTACTCTTTTCCTTTC	CTAGCCCATTGTGCCCGT	60.9	482	72	transposon protein putative unclassified
OsISM00705	LOC_Os01g05090	2385461	AGGATGCCAGTGAGGGTG	CAGCTAGGAGGAGACGGATG	59.8	589	93	expressed protein
OsISM00706	LOC_Os01g05090	2386005	TGCAGGTCAAGATGCAAATG	GAGACATTCTGGGTTCCAGC	60.2	198	91	expressed protein
OsISM00707	LOC_Os01g05090	2386276	TGTGGAACAATGGAAGCAGT	AGCATCCACTATCTCCCCCT	59.5	256	79	expressed protein
OsISM00708	LOC_Os01g05090	2386512	AGGGGGAGATAGTGGATGCT	ATGATGGGAACTTGGACCAG	59.8	227	97	expressed protein
OsISM00709	LOC_Os01g05090	2386720	TGGTCCAAGTTCCCATCATT	AAGTCCACAGACTTGCCACC	60.2	148	69	expressed protein
OsISM00710	LOC_Os01g05090	2386843	CAGATGGTGGCAAGTCTGTG	AACCCACAGTCCACTCCAAC	60.1	187	95	expressed protein
OsISM00711	LOC_Os01g05090	2387070	GATGGTTCCATTAGCGGGTA	TCCAGAATCTTTCTCATTGGTACA	59.9	190	88	expressed protein
OsISM00712	LOC_Os01g05130	2402415	AGCTCTCGAGCACCCTCAG	CTTCTCCTCAACCTTCTGCG	60.5	175	69	expressed protein
OsISM00713	LOC_Os01g05130	2402658	CTCGTCGGTTAAAGTCGCTC	AATGACATTCGACCCTTTGC	60.0	655	82	expressed protein
OsISM00714	LOC_Os01g05130	2403265	CATGGAGGGTAATCCACACC	ACCCGTTGCCTCTTGAGTTA	59.9	176	95	expressed protein
OsISM00715	LOC_Os01g05130	2403444	CGCCAATCACAGTTAGCATC	TGTTGAAGTCACTAAATCTTGTTCTTG	59.7	1032	98	expressed protein
OsISM00716	LOC_Os01g05140	2405623	GAACTCCTCGTCGCGCTC	TCGTACAAGTTGATAGAGAGACAACA	60.9	1074	99	expressed protein
OsISM00717	LOC_Os01g05180	2434143	CCGTCGTTCTATCGGTGG	TGCCTCTAAAATCAGAGCAGA	59.0	2944	82	expressed protein
OsISM00718	LOC_Os01g05200	2440938	CAAAGACAAAAGAACCGGAAA	TGATAGGTGCATGCGATAGG	59.5	178	82	retrotransposon protein putative Ty1-copia subclass
OsISM00719	LOC_Os01g05200	2441329	CAAATATTCCGGTGGAGACG	TCCATCTAGCAATCCTCGCT	60.1	238	98	retrotransposon protein putative Ty1-copia subclass
OsISM00720	LOC_Os01g05200	2441605	CTTTGGCAAGCATAAGAGGG	AACAAGTAGTGGAAACGGCTT	58.9	474	96	retrotransposon protein putative Ty1-copia subclass
OsISM00721	LOC_Os01g05200	2442596	TGCTTTGAGTGTGGCAGAAG	CTGAGTACGGTGCGAATTGA	60.0	338	91	retrotransposon protein putative Ty1-copia subclass

ISM IDs	Rice gene locus IDs	Physical positions (bp) on chromosomes	Forward primers (5'-3')	Reverse primers (5'-3')	Melting tempearature (T˚C)	Expected product size (bp) in PCR	Expected product size (bp) in Q-PCR	Putative Functions
OsISM00722	LOC_Os01g05220	2451874	CGGTCGTTCTATCGGTGG	TTGGGAGATATGAACTCATAATTTTT	59.3	583	89	hypothetical protein
OsISM00724	LOC_Os01g05240	2464480	TTTTATACAGGTTCGGACCCC	GATGCAGATTCGAGGAGGAA	60.2	196	67	retrotransposon protein putative unclassified
OsISM00725	LOC_Os01g05250	2473836	CCTCTATTGCACCCCTCCTC	TCAAACTTTCATCTCATATTCATCA	59.4	321	87	expressed protein
OsISM00727	LOC_Os01g05289	2487683	CAGGATCTGGTCGGGGTC	CATGCCACTAAAATCAGAGCA	60.2	2172	78	expressed protein
OsISM00728	LOC_Os01g05330	2513748	CAAGCAACTCGTCGTTGATG	GGGGCACTGTTTAATTTCCA	60.1	353	60	expressed protein
OsISM00729	LOC_Os01g05340	2519692	CCTATCCTTGTGGCAGCTCT	ATGAAAACCTATCACCCGGC	60.3	681	56	hypothetical protein
OsISM00730	LOC_Os01g05340	2520609	GGCCTTGGAGAAGAGAGAAGA	AGTGAATGAGCAGAGGAGCC	59.8	367	98	hypothetical protein
OsISM00731	LOC_Os01g05350	2528513	GTGTAGAGGGGAGAGGGGAG	GCAGGCGAACATGTCCTTTA	60.6	288	96	hypothetical protein
OsISM00732	LOC_Os01g05350	2528795	GCCTGCGAAAATAAGACACC	TGCAGCATCTCCAGATTGTC	59.8	1238	93	hypothetical protein
OsISM00734	LOC_Os01g05390	2547037	GGTGATGGCGCACTCTTC	TCGTCAGCGTCAAGACCATA	60.4	198	52	hypothetical protein
OsISM00735	LOC_Os01g05400	2550305	GTCAATCCGGTCTCTGCTCC	TCCAACGATTCAATTCGACA	61.1	717	94	conserved hypothetical protein
OsISM00736	LOC_Os01g05420	2559951	CTCGCCTAGGGTTTCGATTC	GTGTCGTAGGGGTCCATGAG	60.7	196	93	IWS1 C-terminus family protein putative expressed
OsISM00737	LOC_Os01g05420	2560643	CTATGGGATACCATCGCCG	GCTGGATCAACCCCAGTATC	60.3	1055	99	IWS1 C-terminus family protein putative expressed
OsISM00738	LOC_Os01g05420	2561689	TGATCCAGCTGACCGTTATG	CCGCTCAATTTCATCATCCT	59.9	571	95	IWS1 C-terminus family protein putative expressed
OsISM00740	LOC_Os01g05420	2562628	CCTTTGCCTGATGGTAGCTT	CCCTTCTATCGTATTGCTCCA	59.3	245	98	IWS1 C-terminus family protein putative expressed
OsISM00741	LOC_Os01g05420	2562862	CGATAGAAGGGAGCAGCTTAAA	CCTTGGCCAGCTTTCTATTA	58.6	196	99	IWS1 C-terminus family protein putative expressed
OsISM00742	LOC_Os01g05420	2563042	AGAAAGCTGGCCAAGGAACT	ATCCTCGAATCGTGTGCTCT	60.1	173	70	IWS1 C-terminus family protein putative expressed
OsISM00743	LOC_Os01g05420	2563242	CCTTACAGGCGACCACAAAT	AGCATCAAGATCATCGTCCC	60.0	187	76	IWS1 C-terminus family protein putative expressed

ISM IDs	Rice gene locus IDs	Physical positions (bp) on chromosomes	Forward primers (5'-3')	Reverse primers (5'-3')	Melting tempearature (T°C)	Expected product size (bp) in PCR	Expected product size (bp) in Q-PCR	Putative Functions
OsISM00744	LOC_Os01g05420	2563409	GGGACGATGATCTTGATGCT	ACAAAGTCCAAGGGTGATGC	60.0	187	98	IWS1 C-terminus family protein putative expressed
OsISM00745	LOC_Os01g05420	2563646	AGCAGGTTGTCCAAGACCAG	TGACGCCTGAAGGTTTTTCT	60.1	199	87	IWS1 C-terminus family protein putative expressed
OsISM00746	LOC_Os01g05430	2567109	TGCTGTCAGAGCTGATAACAAA	AATCCCAACAGCCAAGTTGA	59.6	185	99	OsRhmbd1 - Putative Rhomboid homologue expressed
OsISM00747	LOC_Os01g05430	2567452	CCAAATCATACTTTGGGTTATTGC	CTGGCATTGAACCCTTTCAG	60.7	187	88	OsRhmbd1 - Putative Rhomboid homologue expressed
OsISM00748	LOC_Os01g05430	2567653	GGTGCCATTACCTGAGCTGT	TAATTGATTTCCAAGCTGCG	59.5	596	90	OsRhmbd1 - Putative Rhomboid homologue expressed
OsISM00749	LOC_Os01g05500	2598896	AGTTGTCCACGCAGGAGC	CCTGAAGCTGTTCTTGTGGA	59.5	900	67	zinc finger protein putative expressed
OsISM00750	LOC_Os01g05500	2600814	TTCTTGCATTGGTGAGGATG	AAGGACATAACGTGTATCTTCCAA	59.3	604	95	zinc finger protein putative expressed
OsISM00751	LOC_Os01g05500	2602533	GCGTAAGAATGGTGATGCAG	TCAAGCCTGGAAAGACGAGT	59.6	143	94	zinc finger protein putative expressed
OsISM00752	LOC_Os01g05500	2602784	GTCTTTGGATCTTGCGGAAC	CAAAAGAAAATGGTTGCGGT	59.8	301	78	zinc finger protein putative expressed
OsISM00753	LOC_Os01g05500	2603066	CCGCAACCATTTTCTTTTGT	ACCTGTAAAAGGGTATGCTGTC	58.6	694	56	zinc finger protein putative expressed
OsISM00754	LOC_Os01g05500	2603734	GGCTGACAGCATACCCTTTT	ACGGGGAAGAGACTTCACAA	59.4	190	47	zinc finger protein putative expressed
OsISM00755	LOC_Os01g05500	2603953	TGTTCTGGATTCACTTGCAGTT	TCCAGCTCCTTCCAGAATGT	59.8	175	88	zinc finger protein putative expressed
OsISM00756	LOC_Os01g05500	2604804	ATGGACAAGAAGGCAAAGGC	GCAAACTTCGACTGTTCTGCT	60.6	326	75	zinc finger protein putative expressed
OsISM00757	LOC_Os01g05500	2605238	CCTGTGCAGGGATTCAGATT	TCAACAAAGGTTGCAAGTCG	60.0	284	79	zinc finger protein putative expressed
OsISM00758	LOC_Os01g05500	2606069	GTACACCAGGAATGCCATGA	CCGGATCAACAATATGACCA	59.3	407	92	zinc finger protein putative expressed
OsISM00759	LOC_Os01g05500	2606437	GGAGACTTGGTTTTGAAACTGG	CAAATCTGCGACAGACTGGA	60.0	155	76	zinc finger protein putative expressed
OsISM00760	LOC_Os01g05500	2607135	CGAGCTCTATTTGCTCTGGC	TCACTAGTGGGGTCACCCAT	60.2	1265	95	zinc finger protein putative expressed
OsISM00761	LOC_Os01g05500	2608534	TCCCTTTCGTCCACCTATTC	AGGCAGTTGCTGAAACTTGA	58.8	185	98	zinc finger protein putative expressed

ISM IDs	Rice gene locus IDs	Physical positions (bp) on chromosomes	Forward primers (5'-3')	Reverse primers (5'-3')	Melting tempearature (T˚C)	Expected product size (bp) in PCR	Expected product size (bp) in Q-PCR	Putative Functions
OsISM00762	LOC_Os01g05500	2609144	TTAGGCTGATGGAGTTGCCT	AATCAGGGCATTGCATCTTG	60.4	184	70	zinc finger protein putative expressed
OsISM00763	LOC_Os01g05500	2609378	TGTTCGCCTAGTTGGAAACC	AAGAAATATGCCAACGCCAG	60.1	180	85	zinc finger protein putative expressed
OsISM00764	LOC_Os01g05500	2609538	CTGGCGTTGGCATATTTCTT	AGGCGTCAAGGTAGGGAGAT	60.1	180	94	zinc finger protein putative expressed
OsISM00765	LOC_Os01g05500	2609698	ATCTCCCTACCTTGACGCCT	TGAGGGCTGCGTATCTTTCT	60.0	778	90	zinc finger protein putative expressed
OsISM00766	LOC_Os01g05500	2610420	TGACATGCACAGAGGAAAGC	CTGTCAAGGCTGTGAGATGC	59.8	172	85	zinc finger protein putative expressed
OsISM00767	LOC_Os01g05530	2631849	AAGAGCTCGTGCCTTCAGAC	GGCACGGGGTCTATTATTTG	59.5	899	65	expressed protein
OsISM00768	LOC_Os01g05530	2632742	CGTGCCTCAAATGTGGTTATT	TGACCATCATAAGGAAGCAGAA	59.8	183	96	expressed protein
OsISM00769	LOC_Os01g05530	2632974	TGGAGCAGATGCAACTTTTG	TCATCATCTGATCACCTTCTGC	60.1	142	56	expressed protein
OsISM00770	LOC_Os01g05530	2633133	ACATTGAAAGAGATTCACATATCG	TTGGATGAAAATGAAAGTAAAGGA	58.2	399	99	expressed protein
OsISM00771	LOC_Os01g05530	2633549	CTTGGGCTCTTGTGAAGGAG	AAGCTGCACGCTCATCTTCT	60.1	147	67	expressed protein
OsISM00772	LOC_Os01g05530	2633684	AGCGTGCAGCTTCAGATCTAT	TCTCGCCTGCAGGGATACTA	60.1	195	91	expressed protein
OsISM00773	LOC_Os01g05530	2633867	CTGCAGGCGAGAGTGAAAAT	AACAAAAGCACCGTGGACAT	60.5	197	87	expressed protein
OsISM00774	LOC_Os01g05530	2634073	TGTGATCAAGCTCTGGATGG	TCCAAGTTTATCATTTGGCACA	60.1	1247	94	expressed protein
OsISM00775	LOC_Os01g05530	2636088	ATGAGGAATTCCGATGTTCA	GTCCATGACAAACGAAAGGG	59.1	148	72	expressed protein
OsISM00776	LOC_Os01g05530	2636267	TACTTGCGTGCCATTCAAAA	GCCCTCTTTGTGTGTGAGGT	60.2	206	85	expressed protein
OsISM00777	LOC_Os01g05560	2652797	ACTCGTCATTTGGCTCATCC	CTTGCCACTGTTCGTTCAAA	60.0	1530	89	expressed protein
OsISM00778	LOC_Os01g05590	2666002	AGGACAGGAAGAGATCAGCG	CTTCTGCTCCAAGGATTTCG	59.8	151	88	retrotransposon protein putative unclassified
OsISM00779	LOC_Os01g05680	2712505	CAGGAGGTCAACCACCTCAC	TCCATTTGGCTATTTGATCCTT	60.2	1186	98	geranylgeranyl transferase type-1 subunit beta putative

ISM IDs	Rice gene locus IDs	Physical positions (bp) on chromosomes	Forward primers (5'-3')	Reverse primers (5'-3')	Melting tempearature (T˚C)	Expected product size (bp) in PCR	Expected product size (bp) in Q-PCR	Putative Functions
OsISM00780	LOC_Os01g05680	2713671	GGATCAAATAGCCAAATGGA	TTGTTCTAGAGCCACAGAATCC	58.0	202	99	geranylgeranyl transferase type-1 subunit beta putative
OsISM00781	LOC_Os01g05680	2713842	CAATTTTATGGATTCTGTGGCTC	TGACTACCATTGTGGCAAGG	59.7	190	78	geranylgeranyl transferase type-1 subunit beta putative
OsISM00782	LOC_Os01g05680	2714125	AAACCTCCAACAGCCAGATG	CGCACCAATATGAGTAGGCA	59.9	590	47	geranylgeranyl transferase type-1 subunit beta putative
OsISM00783	LOC_Os01g05680	2714695	TGCCTACTCATATTGGTGCG	TTCCTTGTCCATTCCTGTCC	59.8	184	96	geranylgeranyl transferase type-1 subunit beta putative
OsISM00784	LOC_Os01g05680	2714875	GGAAAAGGCCAAGCAATACA	TTCTGAACCAGGAACCAAGC	60.2	171	71	geranylgeranyl transferase type-1 subunit beta putative
OsISM00785	LOC_Os01g05694	2718220	GCTGGTGGCTCAGATTCTTC	TGCCCCAGGTAAGAAACAAC	60.0	368	99	hypothetical protein
OsISM00786	LOC_Os01g05694	2718568	GTTGTTTCTTACCTGGGGCA	CCTCAGTGATGGACCAGCTT	60.1	484	93	hypothetical protein
OsISM00787	LOC_Os01g05694	2719022	CCTTGGTCATAAGCTGGTCC	CAGCCATAAGAGCCAGGAAG	59.8	248	96	hypothetical protein
OsISM00788	LOC_Os01g05694	2719228	AAGGAGACATTCGGATTTGC	AAACCGACCTCACTGCTGAT	59.4	201	96	hypothetical protein
OsISM00789	LOC_Os01g05694	2719405	TGGTATCAGCAGTGAGGTCG	AGTTCCATTTGTTGGGCATC	59.8	199	93	hypothetical protein
OsISM00790	LOC_Os01g05694	2719624	GCATCCATTCTTTCTCTCGC	CTGCAACGCAGTGTTCTTGT	60.0	154	58	hypothetical protein
OsISM00791	LOC_Os01g05710	2721904	CATCTGCTGTTTGCACAGACT	ACAAGATGCCCCAGGTAATG	59.5	549	99	expressed protein
OsISM00792	LOC_Os01g05710	2722434	ATTACCTGGGGCATCTTGTG	ATACCTCGGTGATGGACCAG	59.8	438	89	expressed protein
OsISM00793	LOC_Os01g05710	2722837	CCCCTTGATCATAAGCTGGT	GCCAGGAAGGTGTAAATCCA	59.5	337	87	expressed protein
OsISM00794	LOC_Os01g05710	2723154	TGGATTTACACCTTCCTGGC	TTAGGCCAACCTCACTAACCA	59.8	215	87	expressed protein
OsISM00795	LOC_Os01g05710	2723347	ATGGTTAGTGAGGTTGGCCT	GGCATCCCAATGCAGTATTT	59.2	175	75	expressed protein
OsISM00796	LOC_Os01g05710	2723520	CCCAACAAATGGAATTTCTCC	TATGGCGCAACAAGTATGGA	60.3	231	92	expressed protein
OsISM00797	LOC_Os01g05720	2727178	TCTACAATTGGGTCGCCTTC	GCCAAGGTCATGTAGCACAA	59.9	322	56	conserved hypothetical protein

ISM IDs	Rice gene locus IDs	Physical positions (bp) on chromosomes	Forward primers (5'-3')	Reverse primers (5'-3')	Melting tempearature (T˚C)	Expected product size (bp) in PCR	Expected product size (bp) in Q-PCR	Putative Functions
OsISM00798	LOC_Os01g05720	2727616	TCAGGGAGAATTGGAGATTG	ACAAGATGCCCCAGGTAATG	58.7	877	97	conserved hypothetical protein
OsISM00799	LOC_Os01g05720	2728473	CATTACCTGGGGCATCTTGT	CACCAGCATATGAGCAAGGA	59.8	588	76	conserved hypothetical protein
OsISM00800	LOC_Os01g05720	2729041	TCCTTGCTCATATGCTGGTG	GCCAGGAAGGTGTAAATCCA	59.9	327	86	conserved hypothetical protein
OsISM00801	LOC_Os01g05720	2729348	TGGATTTACACCTTCCTGGC	AACCACACCAACAGGAAAGC	60.0	196	71	conserved hypothetical protein
OsISM00802	LOC_Os01g05720	2729524	GCTTTCCTGTTGGTGTGGTT	TGGGCATCCTAAGGCAGTAT	59.8	185	91	conserved hypothetical protein
OsISM00803	LOC_Os01g05720	2729741	GCCAATGTCTTTTTCATGGC	GGGCGATCATATGGTGAAAC	60.3	194	65	conserved hypothetical protein
OsISM00804	LOC_Os01g05744	2741828	TCAAATCCCATAGCCCCATA	GTAGAGCCTCCGCAAGACTG	60.1	223	76	expressed protein
OsISM00805	LOC_Os01g05744	2742031	CAGTCTTGCGGAGGCTCTAC	TCTCTTCTCCAACAATGCCA	59.8	236	99	expressed protein
OsISM00806	LOC_Os01g05744	2742266	ATATGAGGCCGTCTACGCTG	GGATATCTCACCTACAATGGAAT G	59.5	250	95	expressed protein
OsISM00807	LOC_Os01g05744	2742457	GCCTGGTGATGGTGATACTG	CAACCTTGCAGTAATTTGTGG	58.6	2393	99	expressed protein
OsISM00808	LOC_Os01g05744	2744849	GTTCATGATCTGGGGCATCT	CCAGCTTATGAGCAAGGAGG	59.9	431	77	expressed protein
OsISM00809	LOC_Os01g05744	2745265	TTGCTCATAAGCTGGTGCAT	GCCAGGAAGGTGTAAATCCA	59.7	310	83	expressed protein
OsISM00810	LOC_Os01g05744	2745555	TGGATTTACACCTTCCTGGC	AGGCCAACCTCACTAACCAT	59.2	203	85	expressed protein
OsISM00811	LOC_Os01g05744	2745738	ATGGTTAGTGAGGTTGGCCT	AATTCCATTTGTTGGGCATC	59.1	185	89	expressed protein
OsISM00812	LOC_Os01g05744	2745943	GCCTCTGTCTTTTTCATGGC	AATAGTGGAACAAGTGCGGG	59.9	215	56	expressed protein
OsISM00813	LOC_Os01g05760	2751241	GCGGAGGCTCTACCTCTCTC	TGCCAATATCATGAACCACG	60.5	583	77	PAS2 putative
OsISM00814	LOC_Os01g05760	2751833	AACGGATATGAGGCCGTGTA	GAGCAATGAAACAAAATGGTTG	59.9	159	97	PAS2 putative
OsISM00816	LOC_Os01g05770	2754041	GTTGCCGCTTTTCTTGTCTC	TTTTGGTGGTGTTTGGATCA	59.9	1790	73	expressed protein

ISM IDs	Rice gene locus IDs	Physical positions (bp) on chromosomes	Forward primers (5'-3')	Reverse primers (5'-3')	Melting tempearature (T°C)	Expected product size (bp) in PCR	Expected product size (bp) in Q-PCR	Putative Functions
OsISM00817	LOC_Os01g05780	2758463	CGCAACTTCATCCAACAACT	GTGACCACCGTGTCAAAACC	60.0	769	83	expressed protein
OsISM00818	LOC_Os01g05780	2759382	GGACTTCGGTAGCCAAAACA	AACCATACCAACAGGAAAGCA	59.8	476	62	expressed protein
OsISM00819	LOC_Os01g05780	2759878	CATTGCTCTTCCTTTCATGG	GGAGAAACTCCATTTGTCGG	58.9	168	74	expressed protein
OsISM00820	LOC_Os01g05780	2760061	GCCAATCTCTTTTTCATGGC	GAAACGGTAGCAACCTGAGC	59.8	317	57	expressed protein
OsISM00821	LOC_Os01g05800	2769618	GTGGTTCGGGTTCATCTCC	CACATGAACAGCTGACAGGC	60.4	497	74	inner membrane protein putative expressed
OsISM00822	LOC_Os01g05800	2770158	TGAAGGCTGCAACATTACCTT	TTGCTTTGATCTGTGGCTGT	59.6	175	82	inner membrane protein putative expressed
OsISM00823	LOC_Os01g05800	2770313	ACAGCCACAGATCAAAGCAA	ACCGAGCAGTCTCAAGCTGT	59.8	281	72	inner membrane protein putative expressed
OsISM00824	LOC_Os01g05800	2770574	ACAGCTTGAGACTGCTCGGT	GGTAGAGGCCAATCCAGACA	60.1	327	99	inner membrane protein putative expressed
OsISM00825	LOC_Os01g05800	2770884	CTGGATTGGCCTCTACCAAG	CCCAAAGATGGAATCCAGAA	59.8	161	82	inner membrane protein putative expressed
OsISM00826	LOC_Os01g05800	2771071	AAAGTGGTGCTGGCATTTCT	GTCATGCCAGCCTAATGGAG	60.2	434	66	inner membrane protein putative expressed
OsISM00827	LOC_Os01g05800	2771559	ATGGAAATCATGAAGCCACC	GTGTGTTCTTCTGCGATGGA	59.8	150	50	inner membrane protein putative expressed
OsISM00828	LOC_Os01g05800	2771749	CTCTTTGTCAGTGCCGTCAG	CTCCCAGTTTCCGTAACCAA	59.8	246	90	inner membrane protein putative expressed
OsISM00829	LOC_Os01g05800	2772048	GCAAAACGTACGAGTGCTCA	TGCCTTTTCTCTTGCTCTCC	59.9	156	80	inner membrane protein putative expressed
OsISM00830	LOC_Os01g05800	2772212	CTTGCTGCAGGAGATTCAGA	TGTATCTTTCCTCAGGTCCTCC	59.4	248	98	inner membrane protein putative expressed
OsISM00832	LOC_Os01g05810	2778665	ACGCCATGGGATACACTTTC	CGAGCGATTTGTATCCATCC	60.1	182	97	gamma-glutamyltranspeptidase 1 precursor putative expressed
OsISM00833	LOC_Os01g05810	2779007	CGTCGACAACACTACGTTCC	GTGGTCATTCAGCTGGCTC	59.3	150	62	gamma-glutamyltranspeptidase 1 precursor putative expressed
OsISM00834	LOC_Os01g05810	2779376	ATGACGCCGACGATCATACT	ACTACTCCGGCGAGCTGTC	60.5	131	45	gamma-glutamyltranspeptidase 1 precursor putative expressed
OsISM00835	LOC_Os01g05810	2779597	CCAGCATCCCAGAGTCTACC	CGTCTCGTTCTCGTACACCA	59.8	497	59	gamma-glutamyltranspeptidase 1 precursor putative expressed

ISM IDs	Rice gene locus IDs	Physical positions (bp) on chromosomes	Forward primers (5'-3')	Reverse primers (5'-3')	Melting tempearature (T˚C)	Expected product size (bp) in PCR	Expected product size (bp) in Q-PCR	Putative Functions
OsISM00837	LOC_Os01g05820	2783760	TGTGATGGCATTTGACAGCTA	TTGTACTTGGAGGTCGGGTT	59.9	3447	72	gamma-glutamyltranspeptidase 1 precursor putative expressed
OsISM00838	LOC_Os01g05820	2787629	CCATGGGCTACACCTTCCT	TCGAGTGATTTGTATCCGCTC	60.1	186	95	gamma-glutamyltranspeptidase 1 precursor putative expressed
OsISM00839	LOC_Os01g05820	2787969	AGGATCGTCGACAACACCAC	CTCGTCCCGTGGTCATCC	61.8	171	75	gamma-glutamyltranspeptidase 1 precursor putative expressed
OsISM00840	LOC_Os01g05820	2788194	GAACCACCTCTTCGGGGC	CTATGATGAACGGCCCGC	62.8	340	99	gamma-glutamyltranspeptidase 1 precursor putative expressed
OsISM00841	LOC_Os01g05820	2788522	CGTTCATCATAGCGACGGT	CGTTCTCGTACACCACCTCA	59.7	709	55	gamma-glutamyltranspeptidase 1 precursor putative expressed
OsISM00842	LOC_Os01g05830	2790511	GAGATCGAGCGCAAGCTG	AAGACGTTGCGAATTGGAGT	60.4	605	49	expressed protein
OsISM00843	LOC_Os01g05830	2791138	CTGACATCGCATTGAAGGTG	CGGGTATCTTCTCGCTCATC	60.0	844	95	expressed protein
OsISM00844	LOC_Os01g05830	2792064	CTCTGGAGCCAGTCTTTTGC	CCTTCCCATCAAGAGGGACT	60.3	565	80	expressed protein
OsISM00845	LOC_Os01g05830	2792609	AGTCCCTCTTGATGGGAAGG	TCTTGTATTGGTTTGGATGTCA	59.2	245	98	expressed protein
OsISM00846	LOC_Os01g05830	2792826	CAGGATTGACATCCAAACCA	TGATCTGGGACTAAAGCAACC	59.3	178	82	expressed protein
OsISM00847	LOC_Os01g05830	2792983	GGTTGCTTTAGTCCCAGATCA	TCGAGAGGAGGAGGAAAAGA	59.1	166	72	expressed protein
OsISM00848	LOC_Os01g05830	2793103	TGATGTTTTGAACAGGCTGG	AAATGCTCAAGGAAACTTGC	58.4	172	99	expressed protein
OsISM00849	LOC_Os01g05830	2793303	GGTGGCCCTGCAGATTACTA	CGGGAGAGGTAATGCAGAAG	60.0	176	87	expressed protein
OsISM00850	LOC_Os01g05830	2793459	CTTCTGCATTACCTCTCCCG	TGTAAAGGCAAGCCTTCAGTC	59.7	196	86	expressed protein
OsISM00851	LOC_Os01g05830	2793683	CCCTACACGAGCAGTAAGGC	AGGCTTATCACATGCCTTGG	60.0	507	85	expressed protein
OsISM00852	LOC_Os01g05840	2799742	TTTGAGATGGGCATCTTGTC	TCCGTAAATTTGCGAGGATA	58.4	1122	96	oxidoreductase short chain dehydrogenase/reductase family domain containing family expressed
OsISM00853	LOC_Os01g05840	2801008	GGAAAACCCAAAAGCTCACA	AACTGAAATTTAAGCAGAACCA A	58.7	192	70	oxidoreductase short chain dehydrogenase/reductase family domain containing family expressed
OsISM00854	LOC_Os01g05840	2801228	TTTCCGTCATGCAGAAATAAT G	CCAAGCACTTTTTGCATGTTTA	60.1	150	53	oxidoreductase short chain dehydrogenase/reductase family domain containing family expressed

ISM IDs	Rice gene locus IDs	Physical positions (bp) on chromosomes	Forward primers (5'-3')	Reverse primers (5'-3')	Melting tempearature (T˚C)	Expected product size (bp) in PCR	Expected product size (bp) in Q-PCR	Putative Functions
OsISM00855	LOC_Os01g05840	2801450	ATATGAAAGCAACCGCGAAG	TCCACATAAATTTTCCAACAGAAA	60.0	172	86	oxidoreductase short chain dehydrogenase/reductase family domain containing family expressed
OsISM00856	LOC_Os01g05840	2801630	TGAAATAAGAATTTTGTATTGGCAGA	TCATCAATGGCACAGATCAAA	60.2	265	97	oxidoreductase short chain dehydrogenase/reductase family domain containing family expressed
OsISM00857	LOC_Os01g05840	2801893	GAAACAGGAAGAAGGAGCGA	ACCCCTGCAAGTGAAAACAA	60.0	232	94	oxidoreductase short chain dehydrogenase/reductase family domain containing family expressed
OsISM00858	LOC_Os01g05840	2802052	TCGATTGAAAAGAGAATGCTG	TTTGTTAAGTGTGTTGTTTCAGATG	58.2	234	99	oxidoreductase short chain dehydrogenase/reductase family domain containing family expressed
OsISM00859	LOC_Os01g05860	2804627	CATCTCCGGTACGATGCC	AGGAAGCCGAGCCTCCTAA	60.7	306	97	PPR repeat domain containing protein putative expressed
OsISM00860	LOC_Os01g05870	2813063	TTCAAGCAACACAATCGGAA	AAGAGTTGAGACCGCAGAGC	60.0	182	80	receptor kinase putative expressed
OsISM00861	LOC_Os01g05940	2832035	GCTCAATTGGAATCAGAGGTTC	TCACCTAGTTTGGAGGCAGC	60.2	225	77	receptor kinase putative expressed
OsISM00862	LOC_Os01g05940	2832538	TAGCAGATGCCGTGTCAAAG	GTTCCATGACCATGAAGCG	60.0	827	84	receptor kinase putative expressed
OsISM00863	LOC_Os01g05940	2833648	TGACAACCGTGTACCTCCAA	GGATCATCCCCTCCAGGT	59.8	190	85	receptor kinase putative expressed
OsISM00864	LOC_Os01g05940	2833807	ATCAACGGCAACCACCTG	AACTTGTCATCCCCAGCAAT	60.0	253	99	receptor kinase putative expressed
OsISM00865	LOC_Os01g05940	2834069	AATGCCTCAGCTTCACTTGG	TCCTTTGCCAAGCAGGTTAG	60.4	279	61	receptor kinase putative expressed
OsISM00866	LOC_Os01g05940	2834548	TCAAGGCACTGGTGTTTGAG	GTTGTGCTGTTTGGGAGGAT	59.9	271	92	receptor kinase putative expressed
OsISM00867	LOC_Os01g05940	2834854	TCAGAGGCTCCATTGGCTAT	TTTTGTGCTTGCATTTGTCA	59.3	401	66	receptor kinase putative expressed
OsISM00868	LOC_Os01g05960	2841712	GGAATCAGAGGTTCCATTGGT	TCAAGCAATAATATGCCTAAGCTG	60.0	252	99	receptor kinase putative expressed
OsISM00869	LOC_Os01g05980	2853630	AAAAGCTCAATTGGAATCCG	TCACCTGCTCTGGTTACTGC	59.1	226	81	receptor kinase putative
OsISM00870	LOC_Os01g06020	2876656	TTGATGGTCTCTGGCTTTGA	TGCACGCAGATCTTCATCTC	59.7	488	67	aspartyl-tRNA synthetase putative expressed
OsISM00871	LOC_Os01g06020	2877207	TTGAAGTTGAACGAAGAATTAATGA	AAAAGGATTTGGCAGCTTGA	59.5	547	70	aspartyl-tRNA synthetase putative expressed
OsISM00872	LOC_Os01g06020	2877806	ACCTTCGTTTTGATTGGCAG	TACTGCCCAAAAATGCATCA	60.1	154	52	aspartyl-tRNA synthetase putative expressed

ISM IDs	Rice gene locus IDs	Physical positions (bp) on chromosomes	Forward primers (5'-3')	Reverse primers (5'-3')	Melting tempearature (T°C)	Expected product size (bp) in PCR	Expected product size (bp) in Q-PCR	Putative Functions
OsISM00873	LOC_Os01g06020	2878073	ACTGAAGCTTCCAAAGCTGG	CACGAGTGGACCAATTCCTT	59.8	177	85	aspartyl-tRNA synthetase putative expressed
OsISM00874	LOC_Os01g06020	2878345	GGCTAATCGAATTCTTGGCA	GAAAATCCGTCACCCAAAGA	60.0	242	96	aspartyl-tRNA synthetase putative expressed
OsISM00875	LOC_Os01g06020	2878567	TCTTTGGGTGACGGATTTTC	TTCAGGATTTGGTGCAGTGA	60.1	805	92	aspartyl-tRNA synthetase putative expressed
OsISM00876	LOC_Os01g06020	2879394	CTCGAGCCTTGGCTTATGAC	ATCCTCAAACTGCCTCCTCC	60.3	656	62	aspartyl-tRNA synthetase putative expressed
OsISM00877	LOC_Os01g06020	2880084	ATCATCGGTATCTCACCCGA	AGGAGCACCCATGTCAAAAC	60.1	697	73	aspartyl-tRNA synthetase putative expressed
OsISM00878	LOC_Os01g06020	2880761	GTTTTGACATGGGTGCTCCT	AGCCAAAAGCATCACCAAAC	60.0	464	69	aspartyl-tRNA synthetase putative expressed
OsISM00879	LOC_Os01g06030	2885037	AAGGAGCAGACCGTGCAG	AAGGAAAACGTCGCTGAGAA	60.1	238	91	conserved hypothetical protein
OsISM00881	LOC_Os01g06130	2923573	GAAAGGACCGTGAAGATTGG	TAGTCCTCAGGGATGCAAGG	59.9	162	62	expressed protein
OsISM00882	LOC_Os01g06140	2927007	GCAGGAAAGGACCATGAAGA	GACTTCAGGGAAGCAAGGAG	59.6	175	64	expressed protein
OsISM00883	LOC_Os01g06150	2931499	AGGAACGGACCGTGAAGATT	CGGGAGAAACACATCAGTCA	60.3	210	93	expressed protein
OsISM00884	LOC_Os01g06170	2941368	CCCGAGGTGGTCGTTTAG	GTCTCCTCGGCTTCAGACC	59.5	202	97	retrotransposon protein putative unclassified
OsISM00885	LOC_Os01g06170	2942600	GAGCGAGCTCTTGAGGAGG	CGGTCTAGACGGGAGACG	59.9	361	99	retrotransposon protein putative unclassified
OsISM00886	LOC_Os01g06260	2968167	TGGAGGAGATCAGAGCACAA	ATTGGTCACGGTAGCAAAGC	59.8	1278	97	hypothetical protein
OsISM00887	LOC_Os01g06310	2999453	TACGTGCCGGGAGCCTAC	GGTGTGGTACGATCCCATGT	61.3	404	64	glycine-rich cell wall structural protein precursor putative expressed
OsISM00888	LOC_Os01g06330	3009899	GGAGGGCTCGGGTTTTTAT	CTCTCTCCCTTGTCCTGGTG	60.1	305	89	retrotransposon protein putative unclassified
OsISM00889	LOC_Os01g06330	3010681	CTGCCGGCGAATTCAAAC	TCCCATGACTTCTCCTTGAA	60.4	339	84	retrotransposon protein putative unclassified
OsISM00890	LOC_Os01g06360	3016155	TTCATCGATAAAGGCGCTG	TTGCGTGGTTGGTTCAGTAA	60.0	807	52	transposon protein putative unclassified
OsISM00891	LOC_Os01g06360	3017716	TGCAGAGGCTACGCTTCAAT	TCTCATGTGGAAAACACGGA	60.6	161	75	transposon protein putative unclassified

ISM IDs	Rice gene locus IDs	Physical positions (bp) on chromosomes	Forward primers (5'-3')	Reverse primers (5'-3')	Melting tempearature (T˚C)	Expected product size (bp) in PCR	Expected product size (bp) in Q-PCR	Putative Functions
OsISM00892	LOC_Os01g06360	3018075	TGCAGATTCCTTCAATTTCTCA	AGCCTGGAGTTGAAAACATCA	59.8	488	89	transposon protein putative unclassified
OsISM00893	LOC_Os01g06360	3018721	CGGTGCAAATTTCTCAAACA	TGCTGACCTCTGTAGGTCCTT	59.3	454	71	transposon protein putative unclassified
OsISM00894	LOC_Os01g06360	3019480	GTACTCAGCAAGCCAAGGGA	CTGGGTGATCCGGGTTAGTA	60.1	468	88	transposon protein putative unclassified
OsISM00895	LOC_Os01g06360	3019986	TCATGACAAGGCATTTCCAA	GCTCTTGCACTTAAGGACCG	60.0	272	95	transposon protein putative unclassified
OsISM00896	LOC_Os01g06360	3020245	TAAGTGCAAGAGCGGGAAAC	CGTGCACTCTGTGCGAGTAT	60.2	189	95	transposon protein putative unclassified
OsISM00897	LOC_Os01g06380	3026372	TCGTTGCTCTGGGGTACTCT	CAGCTTGACGTCTTCCATCC	60.3	146	51	hypothetical protein
OsISM00898	LOC_Os01g06450	3038837	CTTCTTCCAACAGCTCAGGC	TAGCTATTGGCCATGCTGC	60.0	246	51	glycosyltransferase family 43 protein putative expressed
OsISM00899	LOC_Os01g06450	3039378	CCGCAGGGATAACTCATCAA	GGATTCCCCTTTTCTTGTCC	60.4	187	93	glycosyltransferase family 43 protein putative expressed
OsISM00900	LOC_Os01g06460	3043834	GAACTCGGGGGTGCTCTC	GCACACATGGTGCTATGCTT	60.3	1398	93	uncharacterized Cys-rich domain containing protein putative expressed
OsISM00901	LOC_Os01g06460	3045346	TAGCCCCATGGTTCTCTCAT	GCACCCACATTGCTTAGTGA	59.6	300	93	uncharacterized Cys-rich domain containing protein putative expressed
OsISM00903	LOC_Os01g06470	3047436	AGGCAAAGACTGCTCATGCT	CACGAAGCGCCTCTATCTTC	60.1	559	91	expressed protein
OsISM00904	LOC_Os01g06470	3048051	GCTGGAGCAGTCCCTCTTTT	TCCCCAGCTGCTATGTTTGT	60.8	246	99	expressed protein
OsISM00905	LOC_Os01g06470	3048290	CTGGGGAACCAGAAGAAACA	CTCGGCAACAAGTGTGGAG	60.3	290	90	expressed protein
OsISM00906	LOC_Os01g06470	3048705	AACTGCTGCTTGGGCTATGT	TAGCACGCCATCAATGGTAA	60.0	318	83	expressed protein
OsISM00907	LOC_Os01g06470	3049004	TACCATTGATGGCGTGCTAA	CATGCCACCTCAGTTGCTAA	60.0	459	76	expressed protein
OsISM00908	LOC_Os01g06470	3049457	GGCATGGGTTGTGGTCTATC	CGGCAATAAGATTTCCCAAA	60.1	589	84	expressed protein
OsISM00909	LOC_Os01g06470	3050026	TTTGGGAAATCTTATTGCCG	CATTTTATAAGACCTGACAATGCTT	58.9	223	97	expressed protein
OsISM00910	LOC_Os01g06470	3050247	TGTTTGAAGAGTGATAACAGGGTT	CTTGTGCTCAAAACTGCCTG	59.4	183	82	expressed protein

ISM IDs	Rice gene locus IDs	Physical positions (bp) on chromosomes	Forward primers (5'-3')	Reverse primers (5'-3')	Melting tempearature (T°C)	Expected product size (bp) in PCR	Expected product size (bp) in Q-PCR	Putative Functions
OsISM00911	LOC_Os01g06470	3050656	CAGGGCTTGGTCTTCAGTTC	AGCTTTGGGCCTTGCTTATT	60.0	207	65	expressed protein
OsISM00912	LOC_Os01g06490	3053900	GGGAGGTCGCTCTACTTGAA	TGGAGTAGGAGTAGCCGACG	59.9	182	92	OsSCP1 - Putative Serine Carboxypeptidase homologue expressed
OsISM00913	LOC_Os01g06490	3054084	ACCACCTCCGATCTGTTCAC	AAACCGTTCCAACCAATTCA	60.1	177	82	OsSCP1 - Putative Serine Carboxypeptidase homologue expressed
OsISM00914	LOC_Os01g06490	3054253	AACGGTTTCCGCAGTACAAG	GCTGAGACAACTGAGGGACA	59.6	206	82	OsSCP1 - Putative Serine Carboxypeptidase homologue expressed
OsISM00915	LOC_Os01g06490	3054466	CCGGAATAACAAAGACGTTGA	TGTGCCAACGTAGTCATGGT	60.0	212	95	OsSCP1 - Putative Serine Carboxypeptidase homologue expressed
OsISM00916	LOC_Os01g06490	3054860	TTCATTTCTCAAACGCAGGTT	ACTTTTCAGTGCAGGGATCA	59.0	256	75	OsSCP1 - Putative Serine Carboxypeptidase homologue expressed
OsISM00917	LOC_Os01g06490	3055154	GCATTTCATGCCAATGTCAC	GCATGGACCTTGGTGAATCT	59.9	196	92	OsSCP1 - Putative Serine Carboxypeptidase homologue expressed
OsISM00918	LOC_Os01g06490	3055369	GATTGCGGCTGGTCTAAGAA	GCAGTGAGGGGGACTACAGA	60.3	244	64	OsSCP1 - Putative Serine Carboxypeptidase homologue expressed
OsISM00919	LOC_Os01g06490	3055659	TGGTATCCTTGGTATGATGATGA	TCGTCACCAGTGTCAAACCT	59.2	165	71	OsSCP1 - Putative Serine Carboxypeptidase homologue expressed
OsISM00920	LOC_Os01g06510	3060401	CAGTAACAAGAACCGACGGC	TGCTCGACGCTCTGTACACT	60.2	362	63	arginyl-tRNA synthetase putative expressed
OsISM00921	LOC_Os01g06510	3060844	ACCAGGTTTTGGGGATTACC	CATGGGGTTACGGAAGTTTG	60.1	199	86	arginyl-tRNA synthetase putative expressed
OsISM00922	LOC_Os01g06510	3061015	ATCCGCAACAAACTTCCGTA	TGACTGGGGGAGGTTATTTG	60.1	154	65	arginyl-tRNA synthetase putative expressed
OsISM00923	LOC_Os01g06510	3061189	TGTTGCTGGACCTGGTTACA	GGTGCCCATGTTTTGATACC	60.1	182	97	arginyl-tRNA synthetase putative expressed
OsISM00924	LOC_Os01g06510	3061515	TAACCATGTGGGAGACTGGG	TGGGAATTGCTCAAACAGAA	60.0	139	65	arginyl-tRNA synthetase putative expressed
OsISM00925	LOC_Os01g06510	3061659	GGAGGATGTTGGGAACCAG	CCGAGCCCTCTCCTTAAAAT	60.0	623	98	arginyl-tRNA synthetase putative expressed
OsISM00926	LOC_Os01g06510	3062262	ATTTTAAGGAGAGGGCTCGG	AATCAAACTCCATTCGGCTG	59.9	193	91	arginyl-tRNA synthetase putative expressed
OsISM00927	LOC_Os01g06510	3062432	AATCAGCCGAATGGAGTTTG	CAGGTGGAATGTAGGGGTTG	60.1	391	93	arginyl-tRNA synthetase putative expressed
OsISM00928	LOC_Os01g06510	3062917	AGATGGCGGCTTCAACTATG	CCATTCTGCCTTCTCCACAT	60.2	212	74	arginyl-tRNA synthetase putative expressed

ISM IDs	Rice gene locus IDs	Physical positions (bp) on chromosomes	Forward primers (5'-3')	Reverse primers (5'-3')	Melting tempearature (T˚C)	Expected product size (bp) in PCR	Expected product size (bp) in Q-PCR	Putative Functions
OsISM00929	LOC_Os01g06510	3063150	GGTCAGCAACGACACTTTCA	TTCCATTTTGTTCTGGGAGC	60.0	180	71	arginyl-tRNA synthetase putative expressed
OsISM00930	LOC_Os01g06510	3063435	TTGATGAGGCTAAAGCTCGG	TCGAGCTCATCATCTGTCCA	60.5	176	89	arginyl-tRNA synthetase putative expressed
OsISM00931	LOC_Os01g06510	3063583	TTGCTGACTGGACAGATGATG	TCTGTCAGCCGATTGTTTTT	59.1	406	95	arginyl-tRNA synthetase putative expressed
OsISM00932	LOC_Os01g06510	3063969	AAAAACAATCGGCTGACAGA	TACGGGCATGTGCATACTGA	59.7	209	95	arginyl-tRNA synthetase putative expressed
OsISM00933	LOC_Os01g06510	3064193	GGAAAGCCAGCAAGGATGTA	AGGAAACGCTCGTATGGATG	60.2	169	74	arginyl-tRNA synthetase putative expressed
OsISM00934	LOC_Os01g06510	3064342	CATCCATACGAGCGTTTCCT	CAAACGATGGGGCTGTAAAT	60.0	266	88	arginyl-tRNA synthetase putative expressed
OsISM00935	LOC_Os01g06510	3064598	CCATCGTTTGTGCGACTACTT	CAGACGGCTCGTTTCTTCAG	60.6	249	98	arginyl-tRNA synthetase putative expressed
OsISM00937	LOC_Os01g06570	3094274	GGTGCGTGGAGACTGAAGAC	TGGTGAAACCCTACCCATGT	60.5	558	87	hypothetical protein
OsISM00938	LOC_Os01g06640	3128542	CACTGAGGAGAATGATCCCTG	CCTTCACTTGGTCCACCACT	59.8	471	76	basic helix-loop-helix putative expressed
OsISM00939	LOC_Os01g06650	3133473	GTGGTTCGGCAACAATGG	ACCACCAAGGTCGGTTCTC	60.5	128	69	retrotransposon protein putative unclassified
OsISM00940	LOC_Os01g06650	3134114	AGAGAGGAAGCAGAGAGGGC	CCTTTCCTTGAGTCTCCCTTG	60.2	277	51	retrotransposon protein putative unclassified
OsISM00941	LOC_Os01g06690	3156702	TTGATGCTGTCCAGTTGAGC	GCATTTGCCTGCTCTATCGT	60.2	173	98	retrotransposon protein putative unclassified
OsISM00942	LOC_Os01g06690	3156849	CAGGATACGATAGAGCAGGC A	AAGAGCGGACAGGAGCATTA	60.2	128	62	retrotransposon protein putative unclassified
OsISM00943	LOC_Os01g06690	3157017	ACCCTCGACTCAAAAGCATC	GTAACGGGACGTCTTCTCCA	59.7	203	92	retrotransposon protein putative unclassified
OsISM00944	LOC_Os01g06720	3176648	CTAGACCTCGGTGCGTCTG	GCACAATTTGGGGAAGAGAA	59.8	438	98	disease resistance protein SlVe2 precursor putative
OsISM00945	LOC_Os01g06750	3196203	TGTTGCATAATCTCCATCGC	GCCGAGAAGCGTCCTCTAC	59.9	509	61	verticillium wilt disease resistance protein precursor putative
OsISM00946	LOC_Os01g06750	3196817	TGGGCTTTTACTTCTGGACG	AAAGGACTCTGGAACTGGACC	59.9	446	87	verticillium wilt disease resistance protein precursor putative
OsISM00947	LOC_Os01g06750	3197240	CTGGTCCAGTTCCAGAGTCC	GGTGTGACCAACAAGCAAAC	59.4	209	87	verticillium wilt disease resistance protein precursor putative

ISM IDs	Rice gene locus IDs	Physical positions (bp) on chromosomes	Forward primers (5'-3')	Reverse primers (5'-3')	Melting tempearature (T°C)	Expected product size (bp) in PCR	Expected product size (bp) in Q-PCR	Putative Functions
OsISM00948	LOC_Os01g06750	3197508	CCTTGATGCAAGTGGGTTTT	GGTAGAGGTATTGGCCCCTC	59.9	701	82	verticillium wilt disease resistance protein precursor putative
OsISM00949	LOC_Os01g06760	3200573	CAACCTGGGTGGAAATGACT	GGCCAATACTTTGTACAGGCA	59.9	131	87	verticillium wilt disease resistance protein Ve2 putative
OsISM00950	LOC_Os01g06760	3200778	ATTAACCCATGGCAAATGGA	GGTCAATCACATTTAGTGAGCG	59.8	235	83	verticillium wilt disease resistance protein Ve2 putative
OsISM00951	LOC_Os01g06760	3201301	TTGGTGCAAGTGGCTTTTCT	TGGGGTAGAGGTATTGTCCC	59.8	709	92	verticillium wilt disease resistance protein Ve2 putative
OsISM00952	LOC_Os01g06770	3208927	TACCCAGCTCCTCCACCG	AACCAAAGCGAGCTGGTG	61.1	503	98	retrotransposon protein putative Ty3-gypsy subclass
OsISM00953	LOC_Os01g06770	3210156	CCCCTGAAGAAGTGGACACA	ACTGGTGGTATTGCATGGGT	60.6	252	94	retrotransposon protein putative Ty3-gypsy subclass
OsISM00954	LOC_Os01g06790	3222539	CCATCATGGATTGGGAACTT	CGAAAAGAAGCTTCGGGATT	60.1	195	86	disease resistance protein putative expressed
OsISM00955	LOC_Os01g06790	3222901	CTCGTGCTTCAATCAAATCAA	GGTTGCAAGATGCTAGTCCC	59.3	178	95	disease resistance protein putative expressed
OsISM00956	LOC_Os01g06790	3223059	GGGACTAGCATCTTGCAACC	CCATGCAGTCTGTTGGAACT	59.2	249	88	disease resistance protein putative expressed
OsISM00957	LOC_Os01g06790	3223337	GAGATGGTGGTGTTCTCTTGG	TTTGTATTAAACAGGATGGCACC	59.8	216	94	disease resistance protein putative expressed
OsISM00958	LOC_Os01g06790	3224432	AAGCTGCAAGATCACCATCA	GCTTCTGCCTGGTACGTGTT	59.9	572	64	disease resistance protein putative expressed
OsISM00959	LOC_Os01g06820	3235882	GGAATCTGACCCAACTCGAA	AATTCACCGCGGGAAAAC	60.4	908	98	hcr2-0B putative
OsISM00960	LOC_Os01g06836	3240211	CTAGCCAGCTCCGAGACG	TTCAACATATTTCAGCCCCG	60.3	710	82	disease resistance protein SlVe2 precursor putative
OsISM00961	LOC_Os01g06890	3259135	GCAACGCTCTGGTTAATTCC	GCTACTTTGGGAGAACGCAC	59.8	828	98	leucine-rich repeat family protein putative
OsISM00962	LOC_Os01g06900	3273121	GGCCCATACCTTCTTGGATT	AGTGCGAAGATGGACTTTGG	60.2	388	98	verticillium wilt disease resistance protein Ve2 putative expressed
OsISM00963	LOC_Os01g06900	3275114	CCAGTTCTTGTCGTTTTCCAA	TTCCAAAGCCAAAGCAAAGT	60.0	298	99	verticillium wilt disease resistance protein Ve2 putative expressed
OsISM00964	LOC_Os01g07070	3321843	GTACGGATCCGACGACTACC	ATATCCTCCCCCTCCTCCTC	60.0	175	95	transposon protein putative CACTA En/Spm sub-class expressed
OsISM00965	LOC_Os01g07070	3322202	GTGACGGTGACTGGGTTTG	GTGCTCCACACTTGTTGCAT	59.9	732	82	transposon protein putative CACTA En/Spm sub-class expressed

ISM IDs	Rice gene locus IDs	Physical positions (bp) on chromosomes	Forward primers (5'-3')	Reverse primers (5'-3')	Melting tempearature (T°C)	Expected product size (bp) in PCR	Expected product size (bp) in Q-PCR	Putative Functions
OsISM00966	LOC_Os01g07070	3323697	TCCTGATGTGACTGTTGAGGA	TGTAGCCACGTTTCTGCTTG	59.6	838	81	transposon protein putative CACTA En/Spm sub-class expressed
OsISM00967	LOC_Os01g07070	3324600	TGCCTTGCCTATGAAGATCC	CTCAGCCATCGCAACACTAA	60.1	184	97	transposon protein putative CACTA En/Spm sub-class expressed
OsISM00968	LOC_Os01g07070	3324787	TCAGCTCCTAGAGCTCCTGC	CTGAAGTTATCCCTGCGACC	59.8	1084	75	transposon protein putative CACTA En/Spm sub-class expressed
OsISM00969	LOC_Os01g07100	3335433	CGTCGATGAGCATGAGGTC	TCAGCCACCATATTCTCAAGC	60.3	909	77	expressed protein
OsISM00970	LOC_Os01g07100	3336354	TGAAGCCCAACAAATGTACAAG	TGAAGGATGTCCAAGGCTTC	60.1	602	79	expressed protein
OsISM00971	LOC_Os01g07100	3336936	GAAGCCTTGGACATCCTTCA	AGCAAGTTCACCACCACAAG	59.5	430	76	expressed protein
OsISM00972	LOC_Os01g07100	3337371	GTTTGTGGACACCCTTGTCA	TGGAACAGATATCCTGGGGA	59.8	496	98	expressed protein
OsISM00973	LOC_Os01g07100	3337925	CAGCAAAAGTGCGTTCTGAG	ACTTCTTGATGCCCCAACCT	60.3	616	81	expressed protein
OsISM00974	LOC_Os01g07100	3338541	GGATCTCCTGAGCTGCATGT	ATCCAACGGCAGTCAACAC	60.0	200	81	expressed protein
OsISM00975	LOC_Os01g07100	3338769	TTTGTGAGGGGAAATGATCC	GCAGTATCATCTTCACCAGGG	59.6	468	84	expressed protein
OsISM00976	LOC_Os01g07100	3339206	CACAGTGCTACCCTGGTGAA	TTTGCATCCCTAAGATTCCC	59.4	647	83	expressed protein
OsISM00977	LOC_Os01g07100	3339887	TCTGCTGACTTGGAAATCCC	GCAGTGTCCTGAAAAGAGGG	60.0	901	98	expressed protein
OsISM00978	LOC_Os01g07100	3340780	GACACTGCGGCAGAAGTACA	TCTGGCTCCGTATTCCATAAA	59.8	530	99	expressed protein
OsISM00979	LOC_Os01g07100	3341311	CGCTTTGGAAGGACTTTTTG	GGGAGCTTTGTGCTGGAATA	60.0	622	84	expressed protein
OsISM00980	LOC_Os01g07200	3396606	GGACATCCAGCAGTTCGAG	CAATTAGCCTGCGCTTTCTT	59.5	202	85	staphylococcal nuclease homologue putative expressed
OsISM00981	LOC_Os01g07200	3396744	TTTTTAAGGTTCCGGATGGG	CCTGGCTTTCTTCCATGTCA	61.1	744	99	staphylococcal nuclease homologue putative expressed
OsISM00982	LOC_Os01g07200	3397527	GTCACCACGCTGAAGAATCA	TTGGAATTGGAAGGCCATAG	59.9	178	71	staphylococcal nuclease homologue putative expressed
OsISM00983	LOC_Os01g07200	3397739	ACACGTCCCAAAACCTCAAG	TGTCAATGTACACGGTGATCG	60.2	221	99	staphylococcal nuclease homologue putative expressed

ISM IDs	Rice gene locus IDs	Physical positions (bp) on chromosomes	Forward primers (5'-3')	Reverse primers (5'-3')	Melting tempearature (T˚C)	Expected product size (bp) in PCR	Expected product size (bp) in Q-PCR	Putative Functions
OsISM00984	LOC_Os01g07200	3398058	GACGGGTTGCAGAAGATGAT	CTTCTTTGCGAGCACTTCCT	59.9	183	79	staphylococcal nuclease homologue putative expressed
OsISM00985	LOC_Os01g07200	3398221	AGGAAGTGCTCGCAAAGAAG	CTTTTGCCTCCTTGCCGTA	60.5	194	85	staphylococcal nuclease homologue putative expressed
OsISM00986	LOC_Os01g07200	3398471	GACCGGTATGGTAGATGCGT	CCATGCACATCCTTTCTTCA	59.7	462	82	staphylococcal nuclease homologue putative expressed
OsISM00987	LOC_Os01g07200	3398939	ACTGCCTATGACCAACGACC	CAGATTGCGCCTGTTTCTC	59.8	168	53	staphylococcal nuclease homologue putative expressed
OsISM00988	LOC_Os01g07212	3400702	GGAGGAGGAGGAGCTAAGGA	TTCTCAATACCTGCGGGAAC	60.0	115	83	staphylococcal nuclease homologue putative expressed
OsISM00989	LOC_Os01g07212	3400752	CTTTGTAGCCGCTGCTGTC	GCGTCAACGACATTATCCAA	59.7	349	99	staphylococcal nuclease homologue putative expressed
OsISM00990	LOC_Os01g07212	3401085	ATAATGTCGTTGACGCCCA	TGAACAGTAATCGTGTCGCC	59.8	189	99	staphylococcal nuclease homologue putative expressed
OsISM00991	LOC_Os01g07212	3401510	CTGACTCAAGAGGTGGACAGG	CTGCCATAAGGCATTGAGGT	60.0	177	89	staphylococcal nuclease homologue putative expressed
OsISM00992	LOC_Os01g07212	3401784	GATTGCAATGGGGTATTCGT	TAGGCAATGTAGTGCCATGC	59.7	415	66	staphylococcal nuclease homologue putative expressed
OsISM00993	LOC_Os01g07212	3402195	CCTATGATCAACGACCGGAG	TGTGAGTATCACTGACAAGGCA	59.9	3061	85	staphylococcal nuclease homologue putative expressed
OsISM00994	LOC_Os01g07212	3405256	TGATGTTGATTTTGTTTGGGTA	AACGGCAAAAGCAAATGAAA	59.3	158	96	staphylococcal nuclease homologue putative expressed
OsISM00995	LOC_Os01g07212	3405393	ATTTCATTTGCTTTTGCCGT	GGTTGGCATGATCAACAATG	59.7	256	76	staphylococcal nuclease homologue putative expressed
OsISM00996	LOC_Os01g07212	3405637	ATCATGCCAACCTTTGGTGT	CACTGGCTTTAGGAGCTTGC	60.2	413	94	staphylococcal nuclease homologue putative expressed
OsISM00997	LOC_Os01g07212	3406104	CACAGCGAAGACAAACTCGT	CCATCATTTTGACTGGTGGA	59.2	139	59	staphylococcal nuclease homologue putative expressed
OsISM00998	LOC_Os01g07250	3422409	GCGGAGAGCAGAGATTCATT	GACACAGCTTTCCATTGGCT	59.9	714	95	saposin-like type B region 1 family protein putative expressed
OsISM00999	LOC_Os01g07250	3423214	CGCAAACGTTGGACCTCTA	CCATCGAAACCTCCAAGAAG	59.8	174	81	saposin-like type B region 1 family protein putative expressed
OsISM01000	LOC_Os01g07250	3423477	TGCCATCATGTTCTTGTTGAA	CTTGCTGCATGCTTTGAGAA	60.2	179	91	saposin-like type B region 1 family protein putative expressed
OsISM01001	LOC_Os01g07250	3423645	CATGCAGCAAGGCAGATAAT	TTTCACCAGAATCAAAGGGG	59.4	422	75	saposin-like type B region 1 family protein putative expressed

ISM IDs	Rice gene locus IDs	Physical positions (bp) on chromosomes	Forward primers (5'-3')	Reverse primers (5'-3')	Melting tempearature (T°C)	Expected product size (bp) in PCR	Expected product size (bp) in Q-PCR	Putative Functions
OsISM01002	LOC_Os01g07300	3444255	TTTGCAAAGCAAAAATTCCA	CGAACGTCACGGTGAAGAG	59.9	223	80	uncharacterized 50.6 kDa protein in the 5region of gyrA and gyrB putative expressed
OsISM01004	LOC_Os01g07320	3454793	GTCGTGATGGCTTCCACC	CCAACCGGTTCGTAGTATCC	59.7	149	69	hypothetical protein
OsISM01005	LOC_Os01g07390	3500857	TCTTCATCAGGCAGCACTTG	TAGACTTGAACATCACGGCG	60.0	266	67	zinc finger C3HC4 type family protein expressed
OsISM01006	LOC_Os01g07390	3501126	ATATCCTGCGCAAGCAAACT	TGTGATCCCAACAAGGACTG	59.7	761	63	zinc finger C3HC4 type family protein expressed
OsISM01007	LOC_Os01g07390	3501958	GGCTCTTGCTCCAAAAGAAA	ATCATAGCAGTTTGGCGCAC	60.4	455	88	zinc finger C3HC4 type family protein expressed
OsISM01008	LOC_Os01g07390	3502441	AAGAACAGCAAAGGGCGTAG	GCAGGCAATAATGCTCGGTA	60.3	219	90	zinc finger C3HC4 type family protein expressed
OsISM01009	LOC_Os01g07390	3502740	TCAAGTTGACATCAGTTGTGGA	TGCGTATGAACCGTAATGGA	59.6	167	90	zinc finger C3HC4 type family protein expressed
OsISM01010	LOC_Os01g07390	3502887	TCCATTACGGTTCATACGCA	AGCGCAAAAGAATGGGAGTA	59.9	267	97	zinc finger C3HC4 type family protein expressed
OsISM01011	LOC_Os01g07390	3503167	TTGCGAGGATTGTGTCTCTG	AGGTCTGCTGGTTTCACCAA	60.3	207	79	zinc finger C3HC4 type family protein expressed
OsISM01012	LOC_Os01g07400	3506453	ACAATGGATGGTACTCGCCT	GTCTGCTTTGTCAAGCCCTC	59.7	213	52	WD40-like domain containing protein putative expressed
OsISM01013	LOC_Os01g07400	3506859	CTGGATCCAATGCTAGCTCA	TGAGCAAATGACCACTCTGAA	59.2	1611	80	WD40-like domain containing protein putative expressed
OsISM01014	LOC_Os01g07400	3508522	ACTGTCATGATGGTTGGCCT	TAACCTGGTCAAAGCTGCAC	59.7	429	56	WD40-like domain containing protein putative expressed
OsISM01016	LOC_Os01g07410	3512166	TGCAATGGAGGACTCAATCA	TCCAGTTCAGGCACCACTAA	59.7	181	93	growth regulator related protein putative expressed
OsISM01017	LOC_Os01g07410	3512348	AAGAGGTCTTTTTGGGCTGA	TCATCAGCTCATCTCTTAACGAA	59.0	321	86	growth regulator related protein putative expressed
OsISM01018	LOC_Os01g07410	3512736	GAGCTGGTCCAACGAGACAT	AGGCGGGCATCTGTTTTATT	60.5	169	91	growth regulator related protein putative expressed
OsISM01019	LOC_Os01g07410	3513106	TGAAGAAACCGAAGAGCTCAC	GGGACAAAGTCCCTGAAACC	60.2	195	98	growth regulator related protein putative expressed
OsISM01020	LOC_Os01g07410	3513409	GCTTTGAAGGCTGCATATCCT	CCATCTGGGTTGAGTGGTTC	60.5	176	92	growth regulator related protein putative expressed
OsISM01021	LOC_Os01g07410	3513616	CGATGTTTTCATCCCCAGTT	CCCTGTCCAATAGGATGGTC	59.5	513	96	growth regulator related protein putative expressed

ISM IDs	Rice gene locus IDs	Physical positions (bp) on chromosomes	Forward primers (5'-3')	Reverse primers (5'-3')	Melting tempearature (T˚C)	Expected product size (bp) in PCR	Expected product size (bp) in Q-PCR	Putative Functions
OsISM01022	LOC_Os01g07450	3532113	CATCTTGGCTCTCCATGACA	TTCTTGATCTCGTTGTCCGA	59.6	195	85	MYB family transcription factor putative
OsISM01023	LOC_Os01g07460	3535171	CCTTGGATGCTAGCGCATTA	TCTGCGTGCTGAGTAGCCT	60.6	205	97	conserved hypothetical protein
OsISM01024	LOC_Os01g07470	3540000	TGGAGAGAGATGGGGAAGAA	TCCTCACCATTAACCAAGGC	59.8	239	60	hypothetical protein
OsISM01025	LOC_Os01g07540	3609395	TGACAATGGAAATTACGTGCC	CCGTTGTGTTTCATTCTGGTT	60.3	557	79	G-patch domain containing protein expressed
OsISM01026	LOC_Os01g07540	3609931	AACCAGAATGAAACACAACGG	TCAGCATTGACCTCTGCATT	59.6	185	96	G-patch domain containing protein expressed
OsISM01027	LOC_Os01g07540	3610096	AATGCAGAGGTCAATGCTGA	CGAGGCGAAGTTACCAGGTT	60.5	190	97	G-patch domain containing protein expressed
OsISM01028	LOC_Os01g07540	3610345	AAATGAGAATTCGACAGCCG	TGACCATACTGAGCAAGCCA	60.3	223	70	G-patch domain containing protein expressed
OsISM01029	LOC_Os01g07540	3610732	GCCTTCTAAGAACTGCCCCT	TGCTAGGGTTCCTTAGCCTG	59.7	358	88	G-patch domain containing protein expressed
OsISM01030	LOC_Os01g07540	3611242	GGTGTTTCAGCTGCTAGTGC	CTCTCAGCAGCTCTGTCCCT	59.3	268	63	G-patch domain containing protein expressed
OsISM01031	LOC_Os01g07540	3611668	AAATGATGGAGAACATGGGC	TACCCTTTGTGCTCTTCCCA	60.2	524	58	G-patch domain containing protein expressed
OsISM01032	LOC_Os01g07560	3623379	GGATCGTACGGATACATCGC	TGTACACGTCGCTCTTGGTC	60.1	190	68	receptor-like protein kinase 2 precursor putative expressed
OsISM01033	LOC_Os01g07590	3644882	GGAGAGTGGTTCGTGGATTC	TTCTGTGAACAAAACGTCGC	59.7	332	98	universal stress protein domain containing protein putative expressed
OsISM01034	LOC_Os01g07590	3645192	CTGCGACGTTTTGTTCACAG	CTGATGTTTTGTTGAGGGGC	60.5	442	90	universal stress protein domain containing protein putative expressed
OsISM01035	LOC_Os01g07590	3645759	TAGGCCTAGCCCATATTCAA	CTCCTTCCAAAATGCACGAT	58.7	261	83	universal stress protein domain containing protein putative expressed
OsISM01036	LOC_Os01g07590	3645998	AAATCGTGCATTTTGGAAGG	TCTGCTGATTCAACTGTTCCA	59.7	1031	94	universal stress protein domain containing protein putative expressed
OsISM01037	LOC_Os01g07590	3647058	TGGGCAACTGGTGGATCTAT	CCAGTACAACCCCCTTGAGA	60.1	246	72	universal stress protein domain containing protein putative expressed
OsISM01038	LOC_Os01g07590	3647284	TCTCAAGGGGGTTGTACTGG	TGGAGCTATTGGTCAGGTCC	60.0	929	88	universal stress protein domain containing protein putative expressed
OsISM01039	LOC_Os01g07590	3648107	TTCAATCTTGTCTCCTCATTTCC	ACTCCAATCTTCCTCCCACC	59.9	190	99	universal stress protein domain containing protein putative expressed

ISM IDs	Rice gene locus IDs	Physical positions (bp) on chromosomes	Forward primers (5'-3')	Reverse primers (5'-3')	Melting tempearature (T°C)	Expected product size (bp) in PCR	Expected product size (bp) in Q-PCR	Putative Functions
OsISM01040	LOC_Os01g07590	3648407	ATGAGCAAGGTGCAGTTCAA	CATATGTCTTGGCGACATGG	59.7	263	91	universal stress protein domain containing protein putative expressed
OsISM01041	LOC_Os01g07590	3648674	GTCACCCGACAAGGAAACAC	TGCAGGGTCACCGTAGAGTA	59.9	206	92	universal stress protein domain containing protein putative expressed
OsISM01042	LOC_Os01g07590	3648917	ACTGCTTGGTGGTTGGAAAC	TCACGATGTAGGAGCTGACG	60.0	402	76	universal stress protein domain containing protein putative expressed
OsISM01043	LOC_Os01g07600	3652057	CCCCATTCAGGATTCTGCT	ACAATCTTGTTCCCATTGGC	59.9	676	77	expressed protein
OsISM01044	LOC_Os01g07600	3652811	TGCTGTGAGATCTGTTTGGG	ACAGCTGATGCGGGTAGTCT	59.9	1055	88	expressed protein
OsISM01045	LOC_Os01g07600	3653885	CGGGGTGTCATTAGCACTTT	TCAAACTCGAGCCTGTGTTG	60.0	204	99	expressed protein
OsISM01046	LOC_Os01g07600	3654652	TCATGCCCTTAGATGCTTCA	TGCTCGGTCTTTGCCTATCT	59.7	215	90	expressed protein
OsISM01047	LOC_Os01g07710	3701542	TGCAAGACTCCAACACAAGG	CTGGTGAGGTGGTGTCCTTT	59.9	594	87	hypothetical protein
OsISM01048	LOC_Os01g07710	3702360	CCCAGATGCATCAACTCCTT	CTCTGTGGTGTTGTCATGGG	60.0	566	71	hypothetical protein
OsISM01049	LOC_Os01g07710	3702958	TGGTGGATCAAATCCAAGAA	GGTTTGTGTTGGGAAGAGGA	59.4	194	87	hypothetical protein
OsISM01050	LOC_Os01g07730	3710735	GCGTGGTACCTCCTCAACAT	GTGTAGGGGAACGGCAATG	60.7	152	72	phosphate/phosphoenolpyruvate translocator-related protein putative expressed
OsISM01051	LOC_Os01g07730	3710958	CGCCCAGAATCTCCATTTC	ACCTTGCTCAGGCTCATGTT	60.2	202	95	phosphate/phosphoenolpyruvate translocator-related protein putative expressed
OsISM01052	LOC_Os01g07730	3711214	CCTCTCCGCCTTCTTCCTC	GAGTTCAGTCAACGATGCCA	60.6	184	98	phosphate/phosphoenolpyruvate translocator-related protein putative expressed
OsISM01053	LOC_Os01g07730	3711378	TGGCATCGTTGACTGAACTC	GCTTCTTGCTGAGCACATTTC	60.0	223	97	phosphate/phosphoenolpyruvate translocator-related protein putative expressed
OsISM01054	LOC_Os01g07730	3711580	GAAATGTGCTCAGCAAGAAGC	GAGGTTTATGTCATCCAACGC	59.8	149	60	phosphate/phosphoenolpyruvate translocator-related protein putative expressed
OsISM01055	LOC_Os01g07730	3711784	CAGAAGGGGTCAAGTTCAGC	ATGGAAGCAAAAGCCAGCTA	59.9	179	96	phosphate/phosphoenolpyruvate translocator-related protein putative expressed
OsISM01056	LOC_Os01g07730	3711943	TAGCTGGCTTTTGCTTCCAT	TCTGGCCAGGATCAAGTAGG	60.1	169	54	phosphate/phosphoenolpyruvate translocator-related protein putative expressed
OsISM01057	LOC_Os01g07730	3712163	ATCGTGGCGTCTGTTCTCTT	AACTCCTCCCAATGCAACAC	59.9	199	79	phosphate/phosphoenolpyruvate translocator-related protein putative expressed

ISM IDs	Rice gene locus IDs	Physical positions (bp) on chromosomes	Forward primers (5'-3')	Reverse primers (5'-3')	Melting tempearature (T˚C)	Expected product size (bp) in PCR	Expected product size (bp) in Q-PCR	Putative Functions
OsISM01058	LOC_Os01g07770	3731327	GGCTTGCTCAGGCTACATTT	CTGTGGTGCAGTCCTCTCAG	59.5	175	94	peroxidase precursor putative
OsISM01059	LOC_Os01g07770	3731569	GGGTGGTATCCTGTGCTGAT	TCCATCTCTCCGTCCCAGT	60.2	207	99	peroxidase precursor putative
OsISM01060	LOC_Os01g07770	3731843	AAAAATTTGCAGCTCAAGGC	CGTGAAGTTGTAGAGCCGGT	59.9	728	90	peroxidase precursor putative
OsISM01061	LOC_Os01g07790	3737010	AGCACCGTGAATCTTTTGGA	CAGCACCTGTGGAACAACTG	60.5	337	85	polygalacturonase putative
OsISM01062	LOC_Os01g07790	3737313	TGACACCGGTAATCCAGTTG	TCAACACGAGCAACAACACA	59.7	367	75	polygalacturonase putative
OsISM01063	LOC_Os01g07790	3737731	ATGCCACTGGAGATGGAAAC	GCCTTCATTGTTGCAGGTCT	60.1	190	75	polygalacturonase putative
OsISM01064	LOC_Os01g07790	3737974	ATTCAACGGTTCCTGCAAGT	CCCAAATGTAGTTTGGTGCC	59.9	192	72	polygalacturonase putative
OsISM01065	LOC_Os01g07790	3738230	TCAGATTGATGGCAAAGGTG	TACAACAGGCCGATAGACGC	60.4	147	71	polygalacturonase putative
OsISM01066	LOC_Os01g07790	3738349	ACAGAAATGCGTCTATCGGC	ATGTGTTTGTCTGCGCTGTT	59.8	193	97	polygalacturonase putative
OsISM01067	LOC_Os01g07790	3738605	CCCCAATACAGACGGCATAA	ATGATAGCATCGACACGCAG	60.3	218	96	polygalacturonase putative
OsISM01068	LOC_Os01g07790	3738842	CACTGACATCACATGTGGGC	TAATTCGTTCCACCAGTGCC	61.0	188	81	polygalacturonase putative
OsISM01069	LOC_Os01g07790	3739053	GGAACAAAGAATGGTGTGAG G	TTCAAAAATGAACCCCGTTG	60.1	228	70	polygalacturonase putative
OsISM01070	LOC_Os01g07790	3739331	TTGTCCCCAAGGAAACTGTC	AGGATGTCCCACGGATATTG	59.8	182	81	polygalacturonase putative
OsISM01071	LOC_Os01g07840	3764889	CAGCCAGCCTATAGAGAGCG	CCGCATTGTTCCATATGCTT	60.6	226	91	transposon protein putative CACTA En/Spm sub-class
OsISM01072	LOC_Os01g07840	3765853	ATTGGTGGCTCCTTACATCG	AAGGTCAACCGTTGTCATCC	59.9	447	95	transposon protein putative CACTA En/Spm sub-class
OsISM01073	LOC_Os01g07840	3766562	CACGTAGCGATCACAAGGAA	AGCTACCACTTCGGCTTGAG	59.8	1871	81	transposon protein putative CACTA En/Spm sub-class
OsISM01074	LOC_Os01g07840	3768378	TGCATGTTGCAGAGAAGACG	TCCCAATGCGATTGTATCGT	61.2	835	98	transposon protein putative CACTA En/Spm sub-class
OsISM01075	LOC_Os01g07840	3769337	GGAACGAGGAGGAGGCTAGT	ACGTTATCCCTCACAACCCA	60.0	193	98	transposon protein putative CACTA En/Spm sub-class

ISM IDs	Rice gene locus IDs	Physical positions (bp) on chromosomes	Forward primers (5'-3')	Reverse primers (5'-3')	Melting tempearature (T˚C)	Expected product size (bp) in PCR	Expected product size (bp) in Q-PCR	Putative Functions
OsISM01076	LOC_Os01g07840	3770540	CATGTGAGCTGCATATTCCC	AGTCCCTGAAATGTCCGTTG	59.5	176	72	transposon protein putative CACTA En/Spm sub-class
OsISM01077	LOC_Os01g07840	3771767	CCCTCGACGTCTCTCTTCTG	ACCCAGTATCGTAAACCCCC	60.0	169	79	transposon protein putative CACTA En/Spm sub-class
OsISM01078	LOC_Os01g07840	3772021	GCAGCATTTCAAGACGTTCA	ATGCGTCCAAGTCGAAAAAG	60.1	169	78	transposon protein putative CACTA En/Spm sub-class
OsISM01079	LOC_Os01g07840	3772234	TTTGACAAGGTCTTCCAACTGA	GAAAATGAAACCTCCGTCCA	59.8	200	98	transposon protein putative CACTA En/Spm sub-class
OsISM01080	LOC_Os01g07840	3772409	AAACTTGGACGGAGGTTTCA	ATAGGCAGTGGGCATACTCG	59.8	189	92	transposon protein putative CACTA En/Spm sub-class
OsISM01081	LOC_Os01g07840	3772578	CGAGTATGCCCACTGCCTAT	TGTGTGGGAGTTTTTCCCTC	60.0	182	81	transposon protein putative CACTA En/Spm sub-class
OsISM01082	LOC_Os01g07840	3772861	AACGTCGGTCCTTCCTCTTC	CCACTCCTTACCGCATTGTC	60.6	696	96	transposon protein putative CACTA En/Spm sub-class
OsISM01083	LOC_Os01g07850	3778999	ACCTTCTACCGCGACTTCCT	GATGCCGATGCCGTAGTT	60.0	462	94	glyoxalase family protein putative expressed
OsISM01084	LOC_Os01g07850	3779504	AGGAGATCAACCCCAAGGAT	GACGCCCATCTCCTTCAG	59.5	1070	84	glyoxalase family protein putative expressed
OsISM01085	LOC_Os01g07870	3806058	TGATCCTTTCAGTGCAGTCG	GAAGCCAATTCTCCAAGCAA	60.2	194	73	ABC transporter family protein putative expressed
OsISM01086	LOC_Os01g07870	3806282	TGAATTCCTACCTGCTGCTG	ATTTGCCTGCCTGTGCTATT	59.4	152	66	ABC transporter family protein putative expressed
OsISM01087	LOC_Os01g07870	3807002	TGATTCCACTCCTAGTGGGC	GGTGTCCACTTCGCTTTGAT	60.1	146	62	ABC transporter family protein putative expressed
OsISM01088	LOC_Os01g07870	3807247	TTTATTCCTGTGCTTGCTGC	TCTTTGCAACTCTCTGGCTG	59.2	152	70	ABC transporter family protein putative expressed
OsISM01089	LOC_Os01g07870	3807625	TGGTCAATCTTCCGACTGGT	GCTCCATACAACCCATGCTT	60.2	182	99	ABC transporter family protein putative expressed
OsISM01090	LOC_Os01g07870	3807913	ACTGGCCATCAGAAGGAGAA	GCCCTTAAGAACAAATGGCA	59.9	251	78	ABC transporter family protein putative expressed
OsISM01091	LOC_Os01g07870	3808368	GAGGGTACCGTGAGAACCAA	CTCCTAGCTGACAGCGATCC	60.0	196	86	ABC transporter family protein putative expressed
OsISM01092	LOC_Os01g07870	3808544	GGATCGCTGTCAGCTAGGAG	CCAGTTCTCGCCGTTCTCTA	60.3	214	80	ABC transporter family protein putative expressed
OsISM01093	LOC_Os01g07870	3808929	ATCACCTCCGTCCTTGACAG	CGACTTGTCCTCCAGTAGCC	60.0	271	94	ABC transporter family protein putative expressed

ISM IDs	Rice gene locus IDs	Physical positions (bp) on chromosomes	Forward primers (5'-3')	Reverse primers (5'-3')	Melting tempearature (T˚C)	Expected product size (bp) in PCR	Expected product size (bp) in Q-PCR	Putative Functions
OsISM01094	LOC_Os01g07950	3842073	CATGAGAGGGCTCGTGAGAT	TGCTGGATAGCTGGTTGTGA	60.4	858	67	OsGrx S15.2 - glutaredoxin subgroup II expressed
OsISM01095	LOC_Os01g07950	3843033	GCCTACAAGCAAAAGTTCCAA	GACTCAGGAAAACCCTTCATGT	59.4	238	97	OsGrx S15.2 - glutaredoxin subgroup II expressed
OsISM01096	LOC_Os01g07950	3843279	GTGGATTCAGTGCATTGGC	GAGCTTGAGGTCTCCCAAAA	59.7	181	84	OsGrx S15.2 - glutaredoxin subgroup II expressed
OsISM01097	LOC_Os01g07950	3843441	TTTGGGAGACCTCAAGCTCA	CCCCCAACAAACTCTCCTTT	60.6	584	91	OsGrx S15.2 - glutaredoxin subgroup II expressed
OsISM01098	LOC_Os01g07950	3844005	AAAGGAGAGTTTGTTGGGGG	TTTTGCTCATGCTTTTGTGC	60.2	204	99	OsGrx S15.2 - glutaredoxin subgroup II expressed
OsISM01099	LOC_Os01g08010	3876262	CCTGTCTGGACAAACCATGA	AAGACAATTCCTCGATCTCCA	59.1	431	68	expressed protein
OsISM01100	LOC_Os01g08060	3903226	AAGAATTTGGAAAGGACGCA	TGTCGTTACCAGGTTGCTTG	59.7	200	58	transposon protein putative CACTA En/Spm sub-class
OsISM01101	LOC_Os01g08070	3904296	TCCAGCAGGATACTCCAAGG	GAGGAGATGGTGCACGAGAC	60.5	185	96	transposon protein putative CACTA En/Spm sub-class
OsISM01102	LOC_Os01g08070	3905295	GGACGCTCTCGACGTCTCTA	ATCCAGTATCGAAAACCCCC	60.4	193	84	transposon protein putative CACTA En/Spm sub-class
OsISM01103	LOC_Os01g08070	3905538	GCCACAGAGGACAATCTCGT	AATGACGGAACCGATACCAA	60.2	390	98	transposon protein putative CACTA En/Spm sub-class
OsISM01104	LOC_Os01g08070	3905935	CGGCAAATGGAGAGAAAGAC	GCAGCACAAGTTAGTTCCCTG	59.9	173	77	transposon protein putative CACTA En/Spm sub-class
OsISM01105	LOC_Os01g08070	3906130	ACTGCCTTGCAGACCAAATC	CTTGAACCGCCACGATAAAT	60.1	178	97	transposon protein putative CACTA En/Spm sub-class
OsISM01106	LOC_Os01g08090	3917580	CTGCACCTTCTCCTCCTTTG	CATCCGTGAGGTTCTCCAAT	60.0	776	66	flavonol-3-O-glycoside-7-O-glucosyltransferase 1 putative expressed
OsISM01107	LOC_Os01g08100	3920838	TGGCTTCTGTACCTTTGCCT	CTCCTCATCAGTGAGGTCCC	59.8	352	77	flavonol-3-O-glycoside-7-O-glucosyltransferase 1 putative expressed
OsISM01108	LOC_Os01g08110	3923901	GATGGCTTCTGTACCTTCGC	TCTCCTCATCTGCGACATTG	59.9	254	80	flavonol-3-O-glycoside-7-O-glucosyltransferase 1 putative expressed
OsISM01111	LOC_Os01g08160	3952147	CTCGAGGAAGAGAGGCTCAA	CTGCTGCCTGTACGCCTC	60.3	171	94	MYB family transcription factor putative expressed
OsISM01112	LOC_Os01g08160	3952954	AATGCTCTTCAGATCCTCGG	ATCAACCTTCATCAGCTCGC	59.9	202	70	MYB family transcription factor putative expressed
OsISM01113	LOC_Os01g08160	3953137	CGAGCTGATGAAGGTTGATG	CGTCTTGTGTGCAACCTGTA	58.9	242	76	MYB family transcription factor putative expressed

ISM IDs	Rice gene locus IDs	Physical positions (bp) on chromosomes	Forward primers (5'-3')	Reverse primers (5'-3')	Melting tempearature (T˚C)	Expected product size (bp) in PCR	Expected product size (bp) in Q-PCR	Putative Functions
OsISM01114	LOC_Os01g08190	3974988	TCACAAATTCACAATCCCACA	CGATCTAAACCGCCAAGAAA	60.0	821	93	transcriptional corepressor LEUNIG putative expressed
OsISM01115	LOC_Os01g08190	3975785	TTCGTTTCTTGGCGGTTTAG	AAAAAGGAAACATGGGGGTT	59.9	435	83	transcriptional corepressor LEUNIG putative expressed
OsISM01116	LOC_Os01g08190	3976259	GTTCTTGGAAGCAGGTTGGA	TGGTTTTCAATGTTTGTGTGG	59.8	777	88	transcriptional corepressor LEUNIG putative expressed
OsISM01117	LOC_Os01g08190	3977297	TTCAAGTAAAATTTCAAAGTCAACA	TTTTGCAATAAGATTGACGCA	58.3	172	89	transcriptional corepressor LEUNIG putative expressed
OsISM01118	LOC_Os01g08190	3977469	TGCTATTTTCATGTTCCTGATGTT	TGCAACGGACTTCAGCATAG	60.0	553	91	transcriptional corepressor LEUNIG putative expressed
OsISM01119	LOC_Os01g08190	3978025	ACAGCTCAGGCTTCAGGGTA	TGGTATTTATTTTGCGCTCCA	60.2	130	59	transcriptional corepressor LEUNIG putative expressed
OsISM01120	LOC_Os01g08190	3978170	CAGCCTATAATGTGTGATCTAATAGGA	CAAACAGCTACTACATGCTGGA	58.6	204	79	transcriptional corepressor LEUNIG putative expressed
OsISM01121	LOC_Os01g08190	3978353	CCAGCATGTAGTAGCTGTTTGA	AAAGTACCTGACACGCCACC	59.1	191	65	transcriptional corepressor LEUNIG putative expressed
OsISM01122	LOC_Os01g08190	3978767	TGTTTCTGGCTGCTAACTGG	GCTGCACAGGTGAGCACATA	60.1	548	84	transcriptional corepressor LEUNIG putative expressed
OsISM01123	LOC_Os01g08190	3979386	TTGCGAGGATACATTGGATG	GGTACTGTGTTATGCAAACTTGG	59.1	198	93	transcriptional corepressor LEUNIG putative expressed
OsISM01124	LOC_Os01g08190	3979770	TCGACCTTAACACCTCGTCA	AAACCTGTTCACTTCCACGG	59.6	217	70	transcriptional corepressor LEUNIG putative expressed
OsISM01125	LOC_Os01g08190	3980015	CCACCTTGATTTGTTACTGTGC	GTCAGGCATCAAACAGACCA	59.6	427	77	transcriptional corepressor LEUNIG putative expressed
OsISM01126	LOC_Os01g08190	3980459	CTCACACTGCAGGCTGACAT	AGGGTAACCACTGCCAATGA	60.2	215	92	transcriptional corepressor LEUNIG putative expressed
OsISM01127	LOC_Os01g08190	3980752	TGGAAATTGAGCAGTTGGTG	CATCCCAGACCCTGACAGTT	59.8	552	85	transcriptional corepressor LEUNIG putative expressed
OsISM01128	LOC_Os01g08190	3981344	ACCATCTATTTGCCTCGGTG	TGAATAGCCTTGCTGCAAAA	59.8	445	87	transcriptional corepressor LEUNIG putative expressed
OsISM01129	LOC_Os01g08190	3981837	CACCCAAACAAGGATGACCT	CCCTACAAAGGGATTGGAAA	59.3	454	99	transcriptional corepressor LEUNIG putative expressed
OsISM01130	LOC_Os01g08190	3982374	TGGAGACTCAAGCTTGCCTTA	ACCACACCTTCACCGTGTCT	60.3	473	99	transcriptional corepressor LEUNIG putative expressed
OsISM01131	LOC_Os01g08200	3985826	GCATCCACAAGGACGAGTG	AGGTCATGTCGACGTACAGC	59.5	173	73	ubiquitin carboxyl-terminal hydrolase 14 putative expressed

ISM IDs	Rice gene locus IDs	Physical positions (bp) on chromosomes	Forward primers (5'-3')	Reverse primers (5'-3')	Melting tempearature (T˚C)	Expected product size (bp) in PCR	Expected product size (bp) in Q-PCR	Putative Functions
OsISM01132	LOC_Os01g08200	3986119	TGAAGAAGCCCACGCTACTC	ATCAGTGAAACCTCCCTCCA	60.0	572	48	ubiquitin carboxyl-terminal hydrolase 14 putative expressed
OsISM01133	LOC_Os01g08200	3986755	TTCCATCAATTGACTTGCCA	TTTCGATCAGCACTTTCGG	60.0	149	74	ubiquitin carboxyl-terminal hydrolase 14 putative expressed
OsISM01134	LOC_Os01g08200	3987145	CTTGCGGTAAAGCTTGGAAC	TGCTGCGCTAATAGTGGATCT	60.0	932	99	ubiquitin carboxyl-terminal hydrolase 14 putative expressed
OsISM01135	LOC_Os01g08200	3988070	CGCAGCATTTGTCACATTTT	TGATCAAGCTCTCTTTCAGCAG	59.8	295	80	ubiquitin carboxyl-terminal hydrolase 14 putative expressed
OsISM01136	LOC_Os01g08200	3988423	TACGGCCCTGGTTATACTGG	AACTTGCATTACTGAGGCCA	59.1	153	67	ubiquitin carboxyl-terminal hydrolase 14 putative expressed
OsISM01137	LOC_Os01g08200	3988544	GCAGTTGCTATATGGCCTCA	ATGCAGCTTTCAAGCTCTGC	59.9	199	91	ubiquitin carboxyl-terminal hydrolase 14 putative expressed
OsISM01138	LOC_Os01g08200	3988726	GAGCTTGAAAGCTGCATTTG	GCATTCCATGAGCCAACTTT	59.4	630	84	ubiquitin carboxyl-terminal hydrolase 14 putative expressed
OsISM01139	LOC_Os01g08200	3989361	GGCAAATACTCTGTGCCAAA	TACTGGAAAATTCGGGATGC	59.3	868	95	ubiquitin carboxyl-terminal hydrolase 14 putative expressed
OsISM01140	LOC_Os01g08200	3990209	GCATCCCGAATTTTCCAGTA	TGTTTGCCTGATCAACTTGG	59.8	212	81	ubiquitin carboxyl-terminal hydrolase 14 putative expressed
OsISM01141	LOC_Os01g08200	3990529	TTTCTTTGAACATACCGTTACA TGA	TTCCTTTCCATCCAAATCCA	60.0	169	93	ubiquitin carboxyl-terminal hydrolase 14 putative expressed
OsISM01142	LOC_Os01g08200	3990669	AAGCTGCAATGGATTTGGAT	ATGCCTCCAGTGGGACTCTA	59.6	145	73	ubiquitin carboxyl-terminal hydrolase 14 putative expressed
OsISM01143	LOC_Os01g08200	3990836	GGAGGAAGTGCCTGAATTTT	TGCAACACCAGGTAATCAGG	58.9	646	97	ubiquitin carboxyl-terminal hydrolase 14 putative expressed
OsISM01144	LOC_Os01g08200	3991479	GCAGATGCGCAAGTTTGTAA	CGCATGTGCGAGATATCAAT	59.8	1017	94	ubiquitin carboxyl-terminal hydrolase 14 putative expressed
OsISM01145	LOC_Os01g08200	3992501	AAGGCATACAGCCTGGTGAA	GTCCTCACTGGCAACAGGAT	60.4	168	87	ubiquitin carboxyl-terminal hydrolase 14 putative expressed
OsISM01146	LOC_Os01g08200	3992756	GCGATGAATTGGCTTCTCTC	CAACAGTTTGTTCAGCAGCC	59.7	206	86	ubiquitin carboxyl-terminal hydrolase 14 putative expressed
OsISM01147	LOC_Os01g08200	3993003	GAAGATGTTGCTCGAAAGGC	TAAAAGCCTCAGGGTGGCTA	59.9	298	86	ubiquitin carboxyl-terminal hydrolase 14 putative expressed
OsISM01148	LOC_Os01g08200	3993347	TGCACACATACCAGATGGAAG	GAGGACATGGGCAACGTAGT	59.8	553	98	ubiquitin carboxyl-terminal hydrolase 14 putative expressed
OsISM01149	LOC_Os01g08280	4058839	GAGGCGGCTTTCCTACCTC	GGTTCTTAAAGGACGCATCAA	60.2	701	93	expressed protein

ISM IDs	Rice gene locus IDs	Physical positions (bp) on chromosomes	Forward primers (5'-3')	Reverse primers (5'-3')	Melting tempearature (T˚C)	Expected product size (bp) in PCR	Expected product size (bp) in Q-PCR	Putative Functions
OsISM01150	LOC_Os01g08280	4059610	CCCAATCCCGTTTATCATTG	CGCACTTAAGCACAATATGCC	60.3	750	95	expressed protein
OsISM01151	LOC_Os01g08280	4060480	GGACCTCCACCATTTGAGAA	GACTTCCGGTTTCCTAGCCT	59.8	675	97	expressed protein
OsISM01152	LOC_Os01g08280	4061228	CTGCAGTTGAACCCAAACAA	GGCTTGGTTTTGAACAGGAA	59.9	159	78	expressed protein
OsISM01153	LOC_Os01g08280	4061562	ATACCAGCCGAGACGAGGAG	CAAACGATCTGCGTCTGAAC	60.6	198	96	expressed protein
OsISM01154	LOC_Os01g08290	4063410	CTTGATCCCGACTTCAAGGA	CACTTGCAATGCCACTGTTT	60.0	843	71	transmembrane protein putative expressed
OsISM01155	LOC_Os01g08290	4064411	GCGGAACCACGAATTATCAC	GCTGCGAGAAGTCCATCAAT	60.4	168	81	transmembrane protein putative expressed
OsISM01156	LOC_Os01g08290	4064710	GTCTGATCGATTCGTTGCTG	GTCGGACCGTCTACTCCAAC	59.5	196	86	transmembrane protein putative expressed
OsISM01157	LOC_Os01g08340	4092001	TGGTGGAGTAGCAAGGATTG	CCAACAACAAACCACACAGC	59.4	476	97	zinc finger family protein putative expressed
OsISM01158	LOC_Os01g08340	4092476	GGAATGTGTGGATATTCGGG	GAAGCCGAATGCAAGGAATA	60.1	174	87	zinc finger family protein putative expressed
OsISM01159	LOC_Os01g08340	4092836	AATGGTGGAGTATTGGCAGC	GAACTTTGACAAGCAGATGCAG	60.0	212	85	zinc finger family protein putative expressed
OsISM01160	LOC_Os01g08350	4095059	GGCTCTAAGCGATCACGAAC	TGGATGGTTGTCCCTTCTTC	59.9	141	96	ATP synthase subunit alpha mitochondrial putative
OsISM01161	LOC_Os01g08360	4098479	GAGCACAAGACGAGGCTGTA	CCATTCGAACAAGGGACAAG	59.8	2027	74	hypothetical protein
OsISM01162	LOC_Os01g08360	4100533	TTCGCGGAATAATCGAAAAC	GGAAGGATTGAGCACCAAAA	60.0	265	71	hypothetical protein
OsISM01163	LOC_Os01g08390	4115458	CGTTGCGCAAAGAGTTCAT	GCCCATCTAAATTCAGCACC	59.8	257	86	hypothetical protein
OsISM01164	LOC_Os01g08400	4117423	ACCAGACCTCGCAGAAGCTC	CCGTAGGGTCATCAAAAACAG	60.8	1972	70	syntaxin putative expressed
OsISM01165	LOC_Os01g08400	4119585	GGAAGTTCTTACCATGCGGA	GTTCGCAGCTGATGATGAGA	60.1	160	77	syntaxin putative expressed
OsISM01166	LOC_Os01g08400	4119825	GGCCAGTGATTCTGCCACTA	CTGCATGAATGGCTGAGAAG	60.4	421	92	syntaxin putative expressed
OsISM01167	LOC_Os01g08400	4120355	TGGTTTCTCAGCAGGGAGAG	CTTCAGTAGCTGGCCCTGAG	60.3	631	93	syntaxin putative expressed

ISM IDs	Rice gene locus IDs	Physical positions (bp) on chromosomes	Forward primers (5'-3')	Reverse primers (5'-3')	Melting tempearature (T˚C)	Expected product size (bp) in PCR	Expected product size (bp) in Q-PCR	Putative Functions
OsISM01168	LOC_Os01g08410	4123094	AGGATGAGGAGGACGACGAC	CACAGCACCATCATCAACCT	60.6	1186	99	early fruit mRNA putative expressed
OsISM01169	LOC_Os01g08410	4124436	TTGGGAATTTCTGGTGGTTC	CCATCACCAGTTTGGTTTGA	59.6	237	81	early fruit mRNA putative expressed
OsISM01170	LOC_Os01g08420	4132715	AACCAACTTATGCTGGCACC	AATTTTCATCGGGTCTGCCT	60.4	663	92	EMB2410 putative expressed
OsISM01171	LOC_Os01g08420	4133449	ATATTCCTCCAAAAGGGGCA	ACTCCAGCTCATCAAGTGGC	60.5	223	98	EMB2410 putative expressed
OsISM01172	LOC_Os01g08420	4133673	TGCTTCATTACGTGGGTTTG	TGACAGCAAACCATGACCTC	59.6	282	77	EMB2410 putative expressed
OsISM01173	LOC_Os01g08420	4134001	TAGCTGCTCGATGGAGTGGT	TCCAGGAAAAACATACTCCCC	60.6	980	99	EMB2410 putative expressed
OsISM01174	LOC_Os01g08420	4135154	AGACCCAGCTATTCGTTCCA	GGTCACGAAGACTTTCAGCA	59.4	186	84	EMB2410 putative expressed
OsISM01175	LOC_Os01g08420	4135323	TGAAAGTCTTCGTGACCAGC	TCATCATCCAACCAATCAGG	59.2	212	58	EMB2410 putative expressed
OsISM01176	LOC_Os01g08420	4135589	TGGTTCTCTTGATGCCAGTG	TCTTGTAAGTGCCCCACTCC	60.0	437	90	EMB2410 putative expressed
OsISM01177	LOC_Os01g08420	4136759	CTCAAACTGGCTTCAAGGCT	GGGTGATCAACAGTGCCTTT	59.8	290	61	EMB2410 putative expressed
OsISM01178	LOC_Os01g08420	4137269	TGCGAAGTTCTGGACATACG	TGCAGCTGGCTGTCTACTTG	60.1	319	60	EMB2410 putative expressed
OsISM01179	LOC_Os01g08420	4137746	TCTCACGAGATGTCTCCCACT	CCATGTTCAGGAGTCCTTTCA	60.0	313	86	EMB2410 putative expressed
OsISM01180	LOC_Os01g08420	4138234	GGTGAGGTCAATTTGGTTGC	TCCAGAATAGGATCCAAACCA	59.9	204	96	EMB2410 putative expressed
OsISM01181	LOC_Os01g08420	4138524	GCTCTGTGGACCAGGATGTT	GCTGTCCATCACCTTCCAAT	60.0	342	91	EMB2410 putative expressed
OsISM01182	LOC_Os01g08420	4139055	AAGTGCAACTTGGAAAACGC	TGCATTGCCATTTCTGAATC	60.0	175	68	EMB2410 putative expressed
OsISM01183	LOC_Os01g08430	4144436	GCATGTGCTGACATAAAGCAA	GAGTCCTGAGCATCGAGTGA	59.5	1200	99	expressed protein
OsISM01184	LOC_Os01g08430	4147226	GAAAAAGGGAATGCCACTCA	ATTCCTGTAGCTGCTGCTCG	60.4	756	72	expressed protein
OsISM01185	LOC_Os01g08430	4148162	AGCAAGATGGCTTTTACCGA	CCCCCACATATTCGCTGTAT	59.8	145	49	expressed protein

ISM IDs	Rice gene locus IDs	Physical positions (bp) on chromosomes	Forward primers (5'-3')	Reverse primers (5'-3')	Melting tempearature (T˚C)	Expected product size (bp) in PCR	Expected product size (bp) in Q-PCR	Putative Functions
OsISM01186	LOC_Os01g08490	4195937	ATGGATCAGGCATCGGTAGA	AATCGTTGCCCTCATTCATC	60.2	796	83	conserved hypothetical protein
OsISM01187	LOC_Os01g08490	4196909	ATACTTGTGTCGGCGTGGTT	GAAAGGGCTTTCCTTCCAAC	60.2	386	71	conserved hypothetical protein
OsISM01188	LOC_Os01g08560	4254604	CGCTGGAGTGTGCTATCCTT	TCTGGTTTCCATGACATTGC	60.0	969	95	DnaK family protein putative expressed
OsISM01189	LOC_Os01g08560	4255705	GCAAATATCACCAAAGATCAGC	CATGGATGTTAAGGCGGACT	59.3	207	99	DnaK family protein putative expressed
OsISM01190	LOC_Os01g08560	4255911	GGGGTGGTCACAGTTGAATC	TCTTTCGGAGGCTCAGTTGT	60.1	182	84	DnaK family protein putative expressed
OsISM01191	LOC_Os01g08560	4256216	TTCTGAGGAAAAATCAGTCCC	CCAACTCAGCAACTGGAACA	59.1	668	93	DnaK family protein putative expressed
OsISM01192	LOC_Os01g08560	4256979	AAGAAGAATGCCGTGGAGTC	TGCTTCCTTGTCTTCAGCAG	59.3	166	91	DnaK family protein putative expressed
OsISM01193	LOC_Os01g08560	4257190	GGTGAGGATGAGACCAAAGG	TCCATTCCTTGTACCTTGCC	59.7	170	89	DnaK family protein putative expressed
OsISM01194	LOC_Os01g08560	4257414	ATGCTGCCTTGTCCAAAGAT	CCAGACTTCTGCTTCGGAAC	59.8	381	90	DnaK family protein putative expressed
OsISM01195	LOC_Os01g08560	4257830	GAAGCATGCTAACCCTGTCC	CTGGTTTCGGCTTCATCATT	59.9	183	96	DnaK family protein putative expressed
OsISM01196	LOC_Os01g08570	4263549	GGTGAAGCGCTACTTCATGC	CCTTTCCCTGTACTCCCTCTG	60.5	426	95	2-oxoglutarate and iron-dependent oxygenase domain-containing protein2 putative expressed
OsISM01197	LOC_Os01g08570	4263939	TTGCAGACGCAGAATCAGAG	CCGGATGTAATGGCTTGAAA	60.6	412	95	2-oxoglutarate and iron-dependent oxygenase domain-containing protein2 putative expressed
OsISM01198	LOC_Os01g08570	4264485	CCCTCATTCTGCCAGATGTT	CTTTGATTCGCTGATTGTGC	59.7	226	69	2-oxoglutarate and iron-dependent oxygenase domain-containing protein2 putative expressed
OsISM01199	LOC_Os01g08570	4264765	TTTTGGCTTGCAAGAGATGTT	CAGTGTATTGCCGCCAACTT	60.5	225	92	2-oxoglutarate and iron-dependent oxygenase domain-containing protein2 putative expressed
OsISM01200	LOC_Os01g08570	4265016	GTATGGTGAGGCTGATGGTG	AGTACATGTCAGCGCCAGTG	59.4	844	99	2-oxoglutarate and iron-dependent oxygenase domain-containing protein2 putative expressed
OsISM01201	LOC_Os01g08570	4265860	TCCGTGGTATACGTTGTGGA	CATGATGCAGCAGGACTTGT	59.9	2289	97	2-oxoglutarate and iron-dependent oxygenase domain-containing protein2 putative expressed
OsISM01202	LOC_Os01g08570	4268177	AACATCTGGACGGAGGGTTA	ACTCGCAACAAAACCCAGAA	60.0	417	99	2-oxoglutarate and iron-dependent oxygenase domain-containing protein2 putative expressed
OsISM01203	LOC_Os01g08570	4268597	AGTTTCAGAGGACGGCAAGA	GTCTTCTGACTCCTGTCCGC	60.0	526	81	2-oxoglutarate and iron-dependent oxygenase domain-containing protein2 putative expressed

ISM IDs	Rice gene locus IDs	Physical positions (bp) on chromosomes	Forward primers (5'-3')	Reverse primers (5'-3')	Melting tempearature (T˚C)	Expected product size (bp) in PCR	Expected product size (bp) in Q-PCR	Putative Functions
OsISM01204	LOC_Os01g08610	4283721	CCTAACAGGTGTCACCGGAC	GGGCAACATCTTCTCCATGT	60.2	1024	66	hAT dimerisation domain-containing protein putative
OsISM01205	LOC_Os01g08640	4303839	GGCGATAAGCACGGTTACTG	GTACAACCGCACGGTTACTG	59.9	174	94	transposon protein putative unclassified
OsISM01206	LOC_Os01g08640	4304013	CTCGCGGTAAGATCGGTAAC	CACTGATCAGGCAGCCAAT	59.8	150	95	transposon protein putative unclassified
OsISM01207	LOC_Os01g08650	4307246	TGCCACTGACTTCACTGCAT	GTACTCCATCTGCTTGCGCT	60.5	1427	98	expressed protein
OsISM01208	LOC_Os01g08690	4325802	GCGCAGGAGAAGTCGTTC	CCTCCAAACCTTCTTGATGC	59.7	503	89	holocarboxylase synthetase putative expressed
OsISM01209	LOC_Os01g08690	4326312	TGCCGATCTAGTTGCTTTCC	AAATTGACATGCTGGCTGTG	60.0	639	98	holocarboxylase synthetase putative expressed
OsISM01210	LOC_Os01g08720	4357697	AAGAATTTGGAAAGGACGCA	ACACGTCGATGTCGTTACCA	59.9	209	67	transposon protein putative CACTA En/Spm sub-class
OsISM01211	LOC_Os01g08730	4363495	CACTCAGCGGACACCATGT	GGTAAGTACCTGAAGGGTCCG	60.3	175	99	transposon protein putative CACTA En/Spm sub-class
OsISM01212	LOC_Os01g08730	4363736	ACCTCGAGCTGGATTACCCT	GAGGAGATGGTGCACGAGAC	60.5	132	81	transposon protein putative CACTA En/Spm sub-class
OsISM01213	LOC_Os01g08730	4364686	CGCCCTCGACGTCTCTATTA	TCCAGTATCGAAAACCCTCC	59.7	191	80	transposon protein putative CACTA En/Spm sub-class
OsISM01214	LOC_Os01g08730	4364958	AAGGCGCAGCATTACAAGAC	GCCTCCAGGTCGATGAGTAA	60.3	179	81	transposon protein putative CACTA En/Spm sub-class
OsISM01215	LOC_Os01g08730	4365137	CTGCACCGTCAACGTATATGA	AATGACGGAACCGATACCAA	59.9	183	96	transposon protein putative CACTA En/Spm sub-class
OsISM01216	LOC_Os01g08730	4365299	CTTGGTATCGGTTCCGTCAT	AGTTCCCTGCTTTTGCTTTG	59.7	189	93	transposon protein putative CACTA En/Spm sub-class
OsISM01217	LOC_Os01g08730	4365522	ACTGCCTTGCAGACCAAATC	CCGCGATAAATTCCTTGTGT	60.1	169	88	transposon protein putative CACTA En/Spm sub-class
OsISM01218	LOC_Os01g08750	4374186	GCCTATTTGGGCTGCTCTC	CTGAGAGGGAAGGTGATTCAA	59.6	205	88	expressed protein
OsISM01219	LOC_Os01g08750	4376230	CTGCATTTGCAGACAGGCT	CTTTGTGTAGTGCAAGGCCA	60.0	769	90	expressed protein
OsISM01220	LOC_Os01g08750	4376979	TGGCCTTGCACTACACAAAG	TGATCATTCTCGTCGCAAAG	59.9	1962	76	expressed protein
OsISM01221	LOC_Os01g08750	4378923	TTGCGACGAGAATGATCAAA	CCAAAAGGAAAGCCATTTCA	60.2	163	94	expressed protein

ISM IDs	Rice gene locus IDs	Physical positions (bp) on chromosomes	Forward primers (5'-3')	Reverse primers (5'-3')	Melting tempearature (T˚C)	Expected product size (bp) in PCR	Expected product size (bp) in Q-PCR	Putative Functions
OsISM01222	LOC_Os01g08750	4379103	CAGAGCCGAATCTCTGCAA	TTCTGCACCTATTTTCGATGA	59.3	174	76	expressed protein
OsISM01223	LOC_Os01g08750	4379635	TTTCCAACGATCCAGAAGATG	CGACAGCCAATCCAGTTTC	59.6	178	97	expressed protein
OsISM01224	LOC_Os01g08750	4379940	TGAGTGGAAGATGCTGGAGA	ATGACATTCTCCCTGCCAAG	59.8	248	91	expressed protein
OsISM01225	LOC_Os01g08750	4380361	CTGATGGTTATTGGGCCTGT	ATACGCATGTGCGTGACAAG	60.3	495	99	expressed protein
OsISM01226	LOC_Os01g08750	4381412	TTCCTTTGCTCAAGGAAGC	AGAAGAGTAACCACTGCGCC	58.8	841	55	expressed protein
OsISM01227	LOC_Os01g08750	4382574	GGCAAGATGGGTATGGTTGT	GAACCAACATGGGAGCATTT	59.7	238	84	expressed protein
OsISM01228	LOC_Os01g08750	4382800	CCATGTTGGTTCCCAAAGTC	AACTCTTCGGATGATGGTGC	60.1	647	99	expressed protein
OsISM01229	LOC_Os01g08750	4383494	GCGAAGCTAATGCTTTGTCC	TGATGTTCCACCCTCAAGTCT	59.8	578	98	expressed protein
OsISM01230	LOC_Os01g08750	4384208	CCTTTAAGTCAAACTGAACGACC	AATGTGCACGAAGCAACAAG	59.6	605	93	expressed protein
OsISM01231	LOC_Os01g08750	4385255	GCACACCATCTGCCATTAAG	GGAAAACCTGACACCTGAGC	59.4	184	96	expressed protein
OsISM01232	LOC_Os01g08750	4385419	GCTCAGGTGTCAGGTTTTCC	TTGAATCCCTTATCTCTTTGGC	59.6	159	65	expressed protein
OsISM01233	LOC_Os01g08750	4385843	TGTTTGGCTTGACATTACAGC	ACAGCGGATCCCACATTTT	59.6	203	91	expressed protein
OsISM01234	LOC_Os01g08750	4386373	CTCCCTTCTCCCCTTCATTC	ATGATGCCAAATGTGCAGAA	60.0	215	78	expressed protein
OsISM01235	LOC_Os01g08750	4386956	TCGAGTGATGTAGCTTGGGA	GGAACTCCTTCCATTCAGCA	59.8	171	83	expressed protein
OsISM01236	LOC_Os01g08750	4387162	AGCCACTGTCAATCGCTTTT	CCGGTATCTCTTCTCCTACCG	60.0	196	98	expressed protein
OsISM01237	LOC_Os01g08750	4387392	GCAATGGCTAAGCGGAACTA	GGAGCCAAACCCTAGTCTCA	59.8	164	84	expressed protein
OsISM01238	LOC_Os01g08750	4388221	GGAAGCAATACGACTGATCCA	CCATGGAAAAGGCAGTAACC	59.8	301	94	expressed protein
OsISM01239	LOC_Os01g08750	4388419	TTGGTATTGAATGCTACTGTTGG	CGCCACATTTCATGGCTAGT	60.1	237	96	expressed protein

ISM IDs	Rice gene locus IDs	Physical positions (bp) on chromosomes	Forward primers (5'-3')	Reverse primers (5'-3')	Melting tempearature (T˚C)	Expected product size (bp) in PCR	Expected product size (bp) in Q-PCR	Putative Functions
OsISM01240	LOC_Os01g08750	4388825	ACACACCATGCCTGCATCTA	CTCCACGATGTGCAGCTAAA	60.1	175	78	expressed protein
OsISM01241	LOC_Os01g08750	4389126	TGACTGGCCATGAAGTACCA	CATCCACTCCATCGAGACAA	59.9	1053	97	expressed protein
OsISM01242	LOC_Os01g08750	4390423	TATCCCAAGTGATGACGACG	TGCACGTGACTCAAGGAGAG	59.9	927	86	expressed protein
OsISM01243	LOC_Os01g08760	4394487	CGCATCGAGGAGTTCATCA	GAAGCTTCTTGGCGATCAG	59.8	215	86	choline/ethanolamine kinase putative
OsISM01244	LOC_Os01g08760	4394728	GGACCCAAGAACGTGTCACT	CTGAACTGCCTAGCCTCCTC	59.6	191	90	choline/ethanolamine kinase putative
OsISM01245	LOC_Os01g08760	4394991	TTGCCACAATGATCTGCAATA	GCCACAGGATTAAAACTCGC	59.9	316	97	choline/ethanolamine kinase putative
OsISM01246	LOC_Os01g08760	4395358	CACACCTCATGTGCTGGACT	AGGTGCATCCTGCTTACCTG	60.0	206	98	choline/ethanolamine kinase putative
OsISM01247	LOC_Os01g08760	4395493	TTTCAGGCATTGACGAACAA	TGTTCAACTTCCGCATCTGA	60.3	229	83	choline/ethanolamine kinase putative
OsISM01248	LOC_Os01g08760	4395768	TGGGGTCTTTGGGGAATAAT	CTGCCTTGCATACTCCTGGT	60.3	176	73	choline/ethanolamine kinase putative
OsISM01249	LOC_Os01g08770	4397090	GAGGCTCTGGATGAAATGGA	CCCTTTCAATTTCTTGTCGC	59.9	183	91	WD domain G-beta repeat domain containing protein expressed
OsISM01250	LOC_Os01g08770	4397279	GCCGCAAGAGAGAAGCTCTA	CTCTAAATAGCCGCCCTCG	60.0	199	88	WD domain G-beta repeat domain containing protein expressed
OsISM01251	LOC_Os01g08770	4397531	GGAGGACGTGGATCTTTTGA	GCCATTTCCCGTGTATCCTA	59.9	632	89	WD domain G-beta repeat domain containing protein expressed
OsISM01252	LOC_Os01g08770	4398215	TGAATTTGGAACTCGTCAAGG	TACTTTTTCTGGGCGACAGC	60.2	348	98	WD domain G-beta repeat domain containing protein expressed
OsISM01253	LOC_Os01g08770	4398535	AACTCTATGCTGTCGCCCAG	TGAATTTCTGTGCCATGTCG	60.5	913	62	WD domain G-beta repeat domain containing protein expressed
OsISM01254	LOC_Os01g08770	4399428	CGACATGGCACAGAAATTCA	GCTTTTCCAGAAATTGGAGC	59.8	325	68	WD domain G-beta repeat domain containing protein expressed
OsISM01255	LOC_Os01g08800	4417361	GGTCTGCCATCTACCTCCAA	GGAGAACATCGTCAACGAGC	60.4	211	97	cytochrome P450 putative expressed
OsISM01256	LOC_Os01g08830	4435019	ATAAGTGGCATGTCTGCGAA	TCTGAAGACCAGCCCAATCT	59.6	649	77	OsFBDUF1 - F-box and DUF domain containing protein expressed
OsISM01257	LOC_Os01g08850	4443923	CCTCCTCCTCAACTCGCTG	ACATCCGTGTCCAGATACCC	60.4	163	58	expressed protein

ISM IDs	Rice gene locus IDs	Physical positions (bp) on chromosomes	Forward primers (5'-3')	Reverse primers (5'-3')	Melting tempearature (T°C)	Expected product size (bp) in PCR	Expected product size (bp) in Q-PCR	Putative Functions
OsISM01258	LOC_Os01g08850	4444113	ATGCCTCTCGTTCCACGTC	CAGTCACAAAAGGGGGAAAA	60.6	224	98	expressed protein
OsISM01259	LOC_Os01g08850	4444252	ACGTGTTGTTGGCTTTTGTG	TCTGGATCTCGCTTTGAAGAA	59.9	419	97	expressed protein
OsISM01260	LOC_Os01g08850	4444754	TTGTCAACACTCCTGGATGG	ACAACAATTGTGGGGCAGAT	59.9	175	83	expressed protein
OsISM01261	LOC_Os01g08850	4445022	TGCTCCATTTCATGATGCTT	TACTCTATAAGCCGGCGCTC	59.4	739	82	expressed protein
OsISM01262	LOC_Os01g08850	4445820	GCCTAACTTCACTTCCACCC	TTTAGACTATGCCATACCTCACT AGG	58.9	246	98	expressed protein
OsISM01263	LOC_Os01g08850	4446116	GTTCAATCCCTTGGTGTGCT	AAATGCCTCTTTGCACATCG	60.6	244	61	expressed protein
OsISM01264	LOC_Os01g08850	4446406	TCTCTTGCTTCAGGGACTCA	AGCACATTCGTTGGCATGTA	59.4	393	85	expressed protein
OsISM01266	LOC_Os01g08970	4496930	ACGGCCATCTCTTCAACAAC	CCATGCAAGACCTCCAGAAT	60.1	831	87	SSRP1-like FACT complex subunit putative expressed
OsISM01267	LOC_Os01g08970	4497852	GAGTCAGGACCAAAGATGGC	GAGAGGCCCATATTCTTTTGC	59.9	1093	98	SSRP1-like FACT complex subunit putative expressed
OsISM01268	LOC_Os01g08970	4498966	TGTTAGTGGTCAAAACTGGGG	GCTTGCTTTGAACCAACCAT	60.0	151	73	SSRP1-like FACT complex subunit putative expressed
OsISM01269	LOC_Os01g08970	4499192	TGATGATACTACTGGGGGCA	CAGTGCGATTCTCATCTCCA	59.4	225	99	SSRP1-like FACT complex subunit putative expressed
OsISM01270	LOC_Os01g08970	4499397	TGGAGATGAGAATCGCACTG	ATCAACATCAGCAACACCCA	60.0	140	65	SSRP1-like FACT complex subunit putative expressed
OsISM01271	LOC_Os01g08970	4499540	TCTGAAGAGGCAGTTGTCACC	TGGAGACGCAAAAATGACAG	60.1	234	93	SSRP1-like FACT complex subunit putative expressed
OsISM01272	LOC_Os01g08970	4499803	ATAGCAGCATTGTTCGCCTT	CGGATTGGTGGATCAAGAGT	59.9	191	83	SSRP1-like FACT complex subunit putative expressed
OsISM01273	LOC_Os01g08970	4499975	CTCTTGATCCACCAATCCGT	GCCAGATTTCTTTCAACTACTGC	59.7	213	89	SSRP1-like FACT complex subunit putative expressed
OsISM01274	LOC_Os01g08970	4500220	AGGACAGGCTGGAGGAATCT	GACCACGAAGAACTTTGGTGA	60.2	964	64	SSRP1-like FACT complex subunit putative expressed
OsISM01275	LOC_Os01g08970	4501291	AAGGGCTTCTTCTTTCTGCC	CAGCACCATGACGTTCAAAT	59.8	224	83	SSRP1-like FACT complex subunit putative expressed
OsISM01276	LOC_Os01g08970	4501599	GGAGTGAATACCATAACCTCT TCAA	AACAGCTGTAACCCCACCAG	59.9	192	99	SSRP1-like FACT complex subunit putative expressed

ISM IDs	Rice gene locus IDs	Physical positions (bp) on chromosomes	Forward primers (5'-3')	Reverse primers (5'-3')	Melting tempearature (T˚C)	Expected product size (bp) in PCR	Expected product size (bp) in Q-PCR	Putative Functions
OsISM01277	LOC_Os01g08970	4501838	TAAAAATCAGGCTGGGGATG	TCCCCACCAGAATCATCAGT	60.1	199	97	SSRP1-like FACT complex subunit putative expressed
OsISM01278	LOC_Os01g08970	4502047	GCTAGTGAAAGTGGGGGTGA	CATCCCTTCCCTTAGGCTTC	60.1	179	92	SSRP1-like FACT complex subunit putative expressed
OsISM01279	LOC_Os01g08970	4502303	TACTTCTCAATGGCTGAGCG	GAGCTTCTTTGCAATCTCGG	59.6	375	76	SSRP1-like FACT complex subunit putative expressed
OsISM01280	LOC_Os01g08970	4502660	GAGATTGCAAAGAAGCTCGG	TGTCGACTTGAGACTGTTGGA	59.8	252	83	SSRP1-like FACT complex subunit putative expressed
OsISM01283	LOC_Os01g08990	4508586	TCGATCAAATCGAAGAAGCC	CCTGCAGGATCTACAGTAATGACA	60.4	409	92	conserved hypothetical protein
OsISM01284	LOC_Os01g08990	4508925	TTCAGGCGACTATGGAGGTT	GCAGCAGGTGCTATCAACG	60.1	853	95	conserved hypothetical protein
OsISM01285	LOC_Os01g08990	4509786	GGGCGTTGACCTATGGTTAC	ATGCTTGCAACAGCATTAGC	59.2	556	95	conserved hypothetical protein
OsISM01286	LOC_Os01g08990	4510325	AATGCTGTTGCAAGCATGAC	ATCCAAATGTTGAATGCGCT	60.2	242	94	conserved hypothetical protein
OsISM01287	LOC_Os01g08990	4510588	CAGATTTCCGGGCAAATAAA	TCGGAACCAATATCCTGGAG	59.9	212	66	conserved hypothetical protein
OsISM01288	LOC_Os01g08990	4510780	CTCCAGGATATTGGTTCCGA	GAAGGAGGCTACCTGTTTTGC	60.1	200	97	conserved hypothetical protein
OsISM01289	LOC_Os01g08990	4510922	GTTGGTTCTTCAGGGTGGAA	TCGACGAGGTACATCACACC	59.7	657	79	conserved hypothetical protein
OsISM01290	LOC_Os01g08990	4511583	ATAGAGCAAAACGGTGGGAG	AGCTGCTTTTGGTCTCCAGT	59.1	257	90	conserved hypothetical protein
OsISM01291	LOC_Os01g08990	4511801	TGCCAGGCTCTCTGGAATTA	CACTTCCTACAGGAGTCGCC	60.4	254	90	conserved hypothetical protein
OsISM01292	LOC_Os01g09010	4527667	AGGAGGACCTCGTCCCCTAC	GCCGCAGGTGAATTTGGT	61.9	3130	93	transferase family protein putative expressed
OsISM01293	LOC_Os01g09020	4533289	CCGTCTGGTACCGCCTCT	CTTCAGTGTGCCTGCGAATA	60.6	1677	97	WD domain G-beta repeat domain containing protein expressed
OsISM01294	LOC_Os01g09020	4535006	TTATCCAAATCCCACGGAAG	CAACCCTCAATCTTCTGACCA	59.9	254	96	WD domain G-beta repeat domain containing protein expressed
OsISM01295	LOC_Os01g09020	4535291	CAAGTCTCATGGTGCAAGGA	AATCACCTCGAAACAATGGG	59.8	953	72	WD domain G-beta repeat domain containing protein expressed
OsISM01296	LOC_Os01g09020	4536285	AGGACAGTCCGTCTTTGTTG	GTCAGACAGAGCAGTCACGG	58.7	210	76	WD domain G-beta repeat domain containing protein expressed

ISM IDs	Rice gene locus IDs	Physical positions (bp) on chromosomes	Forward primers (5'-3')	Reverse primers (5'-3')	Melting tempearature (T°C)	Expected product size (bp) in PCR	Expected product size (bp) in Q-PCR	Putative Functions
OsISM01297	LOC_Os01g09020	4536612	GGCCATGAAAAAGCACTGTC	GACCAGCAGGGAGGGTTT	60.3	459	58	WD domain G-beta repeat domain containing protein expressed
OsISM01298	LOC_Os01g09020	4537067	GGTCAGCTGCTCCAAAGATT	CCAACACACGATGATGAACC	59.6	402	79	WD domain G-beta repeat domain containing protein expressed
OsISM01299	LOC_Os01g09020	4537596	GCGCTTCGTGACCATAGAAT	TGCGTATGTCCCACAGAAGA	60.3	189	98	WD domain G-beta repeat domain containing protein expressed
OsISM01300	LOC_Os01g09030	4543548	ATTTCCGGCGTGTTAATTCC	CCTTGGAGGCAAACAAAAGA	60.6	207	99	2-aminoethanethiol dioxygenase putative expressed
OsISM01301	LOC_Os01g09030	4543832	CTATGATTGGGCTGATGCCT	GTGCCATCAACCTTCACCTT	60.0	174	82	2-aminoethanethiol dioxygenase putative expressed
OsISM01302	LOC_Os01g09030	4544139	TGCCAGCACTACAACGTCTC	GTTCACACTCCTCCAGCCAT	60.1	1012	95	2-aminoethanethiol dioxygenase putative expressed
OsISM01303	LOC_Os01g09080	4565451	GAGGGACGAGAAGAAAGTCG	CGATGGTGCAAAACTCTCAA	59.4	191	74	WRKY DNA-binding domain containing protein expressed
OsISM01304	LOC_Os01g09080	4565637	CATCGACGCAGAGAGATTCA	CTCGTTCTCCTCCCTCACCT	60.4	180	90	WRKY DNA-binding domain containing protein expressed
OsISM01305	LOC_Os01g09080	4566307	TCAGAGTCAAGTGCGACACC	CAAGGGTTCCCCTTTGAGAT	60.2	190	83	WRKY DNA-binding domain containing protein expressed
OsISM01306	LOC_Os01g09080	4566527	ACCCAACTGCCCTGTACG	GTCGTGATCAGGATCGACAT	59.0	190	64	WRKY DNA-binding domain containing protein expressed
OsISM01308	LOC_Os01g09110	4574827	TACTTGGACAAGGGGACACC	CTTGCTCCTTGAATCCGTGT	60.0	270	76	retrotransposon protein putative unclassified
OsISM01309	LOC_Os01g09160	4600009	AAGGTGGTCATTCAACCGAC	GATCTTCGGCCTCGGACT	60.1	196	87	transposon protein putative unclassified
OsISM01310	LOC_Os01g09230	4649663	GTACATCTCTTCCGCCGCT	AGGCCCAGTCAATATCAACG	60.2	508	79	expressed protein
OsISM01311	LOC_Os01g09246	4673376	GGTTCTGGGGGAAGCATCTA	CACTTATCTTGCCAGCACCA	60.6	921	59	glycine-rich protein putative expressed
OsISM01312	LOC_Os01g09246	4674327	GTGGAGGACGCGTTTCTATT	TATCTGGACAGCCCGAACTC	59.7	394	88	glycine-rich protein putative expressed
OsISM01313	LOC_Os01g09246	4674879	CCCTTGCTATGGAGTCGTGT	ATGGATAACGGGTCAAACCA	60.1	1138	86	glycine-rich protein putative expressed
OsISM01314	LOC_Os01g09246	4675997	TGGTTTGACCCGTTATCCAT	CAGACATTCTCAAGGCACCA	59.9	213	99	glycine-rich protein putative expressed
OsISM01315	LOC_Os01g09246	4676271	TGCAACATCGTTACTTGATGCT	CAACAGACCTTGACCACGAA	60.2	180	98	glycine-rich protein putative expressed

ISM IDs	Rice gene locus IDs	Physical positions (bp) on chromosomes	Forward primers (5'-3')	Reverse primers (5'-3')	Melting tempearature (T˚C)	Expected product size (bp) in PCR	Expected product size (bp) in Q-PCR	Putative Functions
OsISM01316	LOC_Os01g09246	4676467	GAGACATAATTGAAGCACAACGA	CAAGTGGACCCCGTAAAATG	59.8	1021	85	glycine-rich protein putative expressed
OsISM01317	LOC_Os01g09246	4677468	CATTTTACGGGGTCCACTTG	TGGTGGGTGTATTATTTCCACA	60.1	198	98	glycine-rich protein putative expressed
OsISM01318	LOC_Os01g09246	4677655	TACACCCACCAGAAGATTGC	GTTCCTTGTACGAGACCCCA	59.3	190	98	glycine-rich protein putative expressed
OsISM01319	LOC_Os01g09246	4677892	GTACCATCAGTGCAACAGGC	CCTCTACCACCATGACCACC	59.7	516	98	glycine-rich protein putative expressed
OsISM01320	LOC_Os01g09246	4678503	ACACCACAAAGCTTTCCACC	AGAGAATACTCCCATGAGCCC	59.8	152	62	glycine-rich protein putative expressed
OsISM01321	LOC_Os01g09246	4678870	TCCACTGGTCAAACATTCCA	TCCTTTCCCTTTGCTGATTC	59.6	694	96	glycine-rich protein putative expressed
OsISM01322	LOC_Os01g09246	4679559	AAGGATTTCCTGGCGAGAAC	CTTATACGTTCCCAAGGGGC	60.6	319	96	glycine-rich protein putative expressed
OsISM01323	LOC_Os01g09246	4679911	CAATGCCCTCCAGATGAACT	GCATGGGGTTTCATAAGCAC	60.2	355	73	glycine-rich protein putative expressed
OsISM01324	LOC_Os01g09246	4680455	CCAACTCAACAAGGATCCCA	GCGATTCCTCAGCCCTATTA	60.1	173	89	glycine-rich protein putative expressed
OsISM01325	LOC_Os01g09246	4680695	CACCCCACCAGAACAGATTT	CACCACTGGTAAGCTGCAAG	59.7	200	91	glycine-rich protein putative expressed
OsISM01326	LOC_Os01g09246	4681021	GTGCTTACGTTCTTGCCGTT	GCCCAGCATAAGATCAGGAG	60.1	579	74	glycine-rich protein putative expressed
OsISM01327	LOC_Os01g09246	4681705	ATGGCTCCATTTTCACTCCA	ATCCAGCTACAAGACGGTGC	60.4	180	92	glycine-rich protein putative expressed
OsISM01328	LOC_Os01g09246	4682073	CCAATGAGTGCAGAGCTTGA	TGGCCTAGTTGAGTATCAGCA	59.3	181	90	glycine-rich protein putative expressed
OsISM01329	LOC_Os01g09246	4682353	CCCGTTTTCTCTCATCTTGC	GCTAAAATCTGCGAGAAGCA	59.1	181	92	glycine-rich protein putative expressed
OsISM01330	LOC_Os01g09246	4682677	AGGCCTTGCTCGTGTGTATG	TGTAGTGTACAAGGCCGCAA	60.8	261	84	glycine-rich protein putative expressed
OsISM01331	LOC_Os01g09246	4682960	TAGTACGCAGCCATGGAACC	GACAAGCCTAGCTTGGATGC	60.5	229	95	glycine-rich protein putative expressed
OsISM01332	LOC_Os01g09252	4688727	GCCCATGGATATTTGTTGGA	ACCAGACCAGAAGGTTCACG	60.3	210	92	WD domain G-beta repeat domain containing protein expressed
OsISM01333	LOC_Os01g09252	4689105	TGGCTCACTTGACAAGACCA	GCAGCTTTGTGCTCAGAGAGT	60.2	184	79	WD domain G-beta repeat domain containing protein expressed

ISM IDs	Rice gene locus IDs	Physical positions (bp) on chromosomes	Forward primers (5'-3')	Reverse primers (5'-3')	Melting tempearature (T˚C)	Expected product size (bp) in PCR	Expected product size (bp) in Q-PCR	Putative Functions
OsISM01334	LOC_Os01g09252	4689354	TCTGGGCTGCTTCAAAGTCT	GTCTTTCCAACACGGTGCAT	60.6	2292	98	WD domain G-beta repeat domain containing protein expressed
OsISM01335	LOC_Os01g09252	4691665	CTCCACAACAGCAACTGCAT	CGATGGTCCTCACTTCCTTC	59.8	179	89	WD domain G-beta repeat domain containing protein expressed
OsISM01336	LOC_Os01g09270	4712659	TCCACGTCGTCCTCCAGT	GTTCGTGCATACGCTCTTCC	60.5	240	64	hypothetical protein
OsISM01337	LOC_Os01g09300	4725913	CGAATCTGAATTGCATCAGC	CCACCACATAGAAGAAGCCTG	59.6	1510	97	oxidoreductase putative expressed
OsISM01338	LOC_Os01g09300	4727542	CCCATGCTAGATGAGATTCTTG	GCTTGTGGATCATCTTCTGG	58.5	352	99	oxidoreductase putative expressed
OsISM01339	LOC_Os01g09300	4727884	ATCCACAAGCAAACAGACCA	TTCCATCACCTTCCTCCATC	59.5	540	81	oxidoreductase putative expressed
OsISM01340	LOC_Os01g09300	4728404	GATGGAGGAAGGTGATGGAA	CTTGCAATTGATTTTGCCAC	59.5	282	62	oxidoreductase putative expressed
OsISM01341	LOC_Os01g09300	4728919	GTGCCCATTCTGATTATGGC	TTGAGGCTGAGCATTCCTGT	60.6	183	90	oxidoreductase putative expressed
OsISM01342	LOC_Os01g09300	4729067	TGCAGATATGCAAGGACAGG	GCATATCGCCAAGATTGACA	59.7	247	91	oxidoreductase putative expressed
OsISM01343	LOC_Os01g09300	4729296	TCAATCTTGGCGATATGCTC	CAGAATGACGTTCTCGTCCA	59.3	227	91	oxidoreductase putative expressed
OsISM01344	LOC_Os01g09300	4729453	TACGGCTGTCTGCATACCTG	GGCTTGGTTCCACAAAGAAC	59.7	222	92	oxidoreductase putative expressed
OsISM01345	LOC_Os01g09300	4729697	GCCAACCTGCAAATCTGAAT	AGGTATGCCGAGCAAGTGAT	59.9	168	61	oxidoreductase putative expressed
OsISM01346	LOC_Os01g09330	4746314	GTGCCAGTGCTGTTGGCTAT	TCGTCTACCTTTCCTTGGCT	60.1	1024	86	conserved hypothetical protein
OsISM01347	LOC_Os01g09330	4747612	GCAGCTGAGTGGGTGAAAA	GGGCTACTCTATTGCATTCCC	60.0	186	83	conserved hypothetical protein
OsISM01348	LOC_Os01g09410	4792102	TCTCATGCAAAGGTCAACCA	ATCTGCATGCGACTTCAAAA	59.8	1102	79	expressed protein
OsISM01349	LOC_Os01g09410	4793203	TTTGGCATTTAGGCAGAAGAA	ACCATCACCCATGAGCAATC	60.3	413	97	expressed protein
OsISM01350	LOC_Os01g09430	4799609	AGAAGATCCCCTTCCACCAC	CCACCTCGCAGAAGAAGAAC	60.2	192	73	expressed protein
OsISM01351	LOC_Os01g09430	4799943	GTCTTCTCCTATTGGCCGTG	ATTCCAGCTTCATCCCGAG	59.9	732	96	expressed protein

ISM IDs	Rice gene locus IDs	Physical positions (bp) on chromosomes	Forward primers (5'-3')	Reverse primers (5'-3')	Melting tempearature (T˚C)	Expected product size (bp) in PCR	Expected product size (bp) in Q-PCR	Putative Functions
OsISM01352	LOC_Os01g09450	4816626	GAACCAATGCCTCCAAGC	GACGACGAGGCGAGGTTC	60.6	186	95	OsIAA2 - Auxin-responsive Aux/IAA gene family member expressed
OsISM01355	LOC_Os01g09450	4817366	TCAAGAGCTCCGACCTGC	AGCTGCTGCCCTCTTCCT	60.2	140	66	OsIAA2 - Auxin-responsive Aux/IAA gene family member expressed
OsISM01356	LOC_Os01g09470	4826125	CAAGATCCAGACAGCCTTCC	CTTCACCAGCGCCTTGAG	60.3	258	68	IQ calmodulin-binding motif family protein expressed
OsISM01357	LOC_Os01g09470	4826519	ACCTCCACCACCACCCTC	CGTGTCGTCCATATACCGCT	60.9	188	63	IQ calmodulin-binding motif family protein expressed
OsISM01358	LOC_Os01g09490	4841959	GTCCATCTGGACAGGCAAGT	CTGTCAAAACCACGGTTCAA	59.9	453	68	transposon protein putative unclassified expressed
OsISM01359	LOC_Os01g09490	4842396	ACCGTGGTTTTGACAGGAAC	ACTGCTGTACACGTGGAGCC	60.6	392	88	transposon protein putative unclassified expressed
OsISM01360	LOC_Os01g09490	4842963	GCACCGTGACCGAGACTACT	TCATTTGGACTGCGGTGATA	60.2	169	75	transposon protein putative unclassified expressed
OsISM01362	LOC_Os01g09490	4843671	AGCTCTTCCTCTCACGGAGC	AGACGAAGAGGCCCCAGTA	60.5	144	82	transposon protein putative unclassified expressed
OsISM01363	LOC_Os01g09490	4844001	TACTCCCAGGGGCACATTAG	CCGAAAGTGTGTTGTCGTGA	60.4	234	97	transposon protein putative unclassified expressed
OsISM01364	LOC_Os01g09510	4852276	CGGAGGATTTGACAACGATT	TGATGTCCTTGCCACTCACT	59.6	445	94	60S acidic ribosomal protein putative expressed
OsISM01365	LOC_Os01g09510	4852834	GCTGAGGCGAAGAAAGAAGA	GACCGCATCACTCAGTCAAA	59.8	307	87	60S acidic ribosomal protein putative expressed
OsISM01366	LOC_Os01g09520	4858368	GTGGTGACGAGAGGCTTGG	CCGCAGGAAGGAGACGAG	62.0	173	99	transposon protein putative unclassified
OsISM01367	LOC_Os01g09520	4858849	CCTGCCTCGCATAGATCAGA	AGGTTCCTACGGGGGTAATG	60.8	144	96	transposon protein putative unclassified
OsISM01368	LOC_Os01g09520	4862133	TATCGAGTGATCGGTGTCCC	AGAAGGAAAAACGGCACAGA	60.4	284	90	transposon protein putative unclassified
OsISM01369	LOC_Os01g09560	4890606	ACGACTCCGACGAGCGAC	CATTTGACCATCCAGTCCAT	60.4	1102	99	mitochondrial-processing peptidase subunit alpha mitochondrial precursor putative expressed
OsISM01370	LOC_Os01g09560	4891894	GCAAATGGTGTAAAAATTGCTTC	AGTGTTTCAGGTGCTTCATGT	58.6	192	99	mitochondrial-processing peptidase subunit alpha mitochondrial precursor putative expressed
OsISM01371	LOC_Os01g09560	4892246	CCTGAAATGGTTGAGGTGCT	GCAAGCTCTGCCTTCAGTTT	59.9	187	96	mitochondrial-processing peptidase subunit alpha mitochondrial precursor putative expressed
OsISM01372	LOC_Os01g09560	4892508	CCCTCTGATTGCCTCAGAAT	CAGCTAGAACAATTCGGGGA	59.7	200	99	mitochondrial-processing peptidase subunit alpha mitochondrial precursor putative expressed

ISM IDs	Rice gene locus IDs	Physical positions (bp) on chromosomes	Forward primers (5'-3')	Reverse primers (5'-3')	Melting tempearature (T˚C)	Expected product size (bp) in PCR	Expected product size (bp) in Q-PCR	Putative Functions
OsISM01373	LOC_Os01g09560	4892773	GTAACTGGACCAGCAAAGCC	ACCTCAAATGCCAAAGCAAC	59.9	180	96	mitochondrial-processing peptidase subunit alpha mitochondrial precursor putative expressed
OsISM01374	LOC_Os01g09560	4892958	GTGGGTGGCTCAAAGAAAAA	ATGCCTTCCCCAGGAATATG	60.6	189	87	mitochondrial-processing peptidase subunit alpha mitochondrial precursor putative expressed
OsISM01375	LOC_Os01g09560	4893144	CATGGAAAAGGCTTGCATTC	GGTCGAATTCATTTGCGAGA	60.9	537	50	mitochondrial-processing peptidase subunit alpha mitochondrial precursor putative expressed
OsISM01376	LOC_Os01g09560	4893695	GCTGCTTTCAAGGATGTTCA	AGCCTTAGGAACGAATGCTG	59.2	356	79	mitochondrial-processing peptidase subunit alpha mitochondrial precursor putative expressed
OsISM01377	LOC_Os01g09560	4894075	ACTTCTCTTGCCACTCCTGG	CGCTTTAGCACGGTCAAGTT	59.9	134	55	mitochondrial-processing peptidase subunit alpha mitochondrial precursor putative expressed
OsISM01378	LOC_Os01g09560	4894189	AACTTGACCGTGCTAAAGCG	TCCCATGTCTTCAGTTGCTG	60.1	160	81	mitochondrial-processing peptidase subunit alpha mitochondrial precursor putative expressed
OsISM01379	LOC_Os01g09560	4894324	GGCATCAGCAACTGAAGACA	CACCATCAACAGCCTTGAGA	59.9	977	87	mitochondrial-processing peptidase subunit alpha mitochondrial precursor putative expressed
OsISM01380	LOC_Os01g09560	4895319	CAGCCCTTGCTGAGAAAATC	CGAAACTTGCCACTGACTGA	60.0	185	98	mitochondrial-processing peptidase subunit alpha mitochondrial precursor putative expressed
OsISM01381	LOC_Os01g09570	4904965	CCCACCTCACGGACTACCT	GAATAAGCCGGGTTGTCTTG	59.8	510	68	6-phosphofructokinase putative expressed
OsISM01382	LOC_Os01g09570	4905438	CTTACCCGAATCCTCTGCAA	GTGACGGTGTCGTCAGTGTT	59.9	218	77	6-phosphofructokinase putative expressed
OsISM01383	LOC_Os01g09570	4905605	TCCTTCTTCTTCTTGCAGGC	TGCCCCTAGCACTGGTCTTA	60.2	196	87	6-phosphofructokinase putative expressed
OsISM01384	LOC_Os01g09570	4905918	TTGTGTGCGGCTTGTATGAT	TCAGTGCGACGGTGTTTCTA	60.3	385	94	6-phosphofructokinase putative expressed
OsISM01385	LOC_Os01g09570	4906439	TGGTACCCAGAAAGGTGCTT	ATGGTCTTTGGGACACCAAC	59.6	175	85	6-phosphofructokinase putative expressed
OsISM01386	LOC_Os01g09570	4906576	GGCCTCAAATGTTCTGTGGT	ACCGCAGTGTCAAAACCAA	60.1	170	87	6-phosphofructokinase putative expressed
OsISM01387	LOC_Os01g09570	4906782	TGAGGCTGAGAGTGCAGAGA	ACATCGCGACTAGCCAGAGT	60.0	192	97	6-phosphofructokinase putative expressed
OsISM01388	LOC_Os01g09570	4906924	ACTGCTACAGGGTTCATCGC	TGGAGACTCTGGGATTAAGCA	60.1	166	76	6-phosphofructokinase putative expressed
OsISM01389	LOC_Os01g09570	4907249	CTTCTGGATGTTGGCCTTTG	GGAAGTTGCGCTTCTTCTTG	60.4	354	65	6-phosphofructokinase putative expressed
OsISM01390	LOC_Os01g09570	4907754	CAGGCACGCGTATATCCC	CCACCTTGTTCTGCTTCTCC	60.0	175	51	6-phosphofructokinase putative expressed

ISM IDs	Rice gene locus IDs	Physical positions (bp) on chromosomes	Forward primers (5'-3')	Reverse primers (5'-3')	Melting tempearature (T˚C)	Expected product size (bp) in PCR	Expected product size (bp) in Q-PCR	Putative Functions
OsISM01391	LOC_Os01g09600	4934349	AAAGTGGAGAAAGCGAAGCC	GATGGTTTCCACGCAAAGAT	60.4	157	98	retrotransposon protein putative Ty3-gypsy subclass
OsISM01392	LOC_Os01g09600	4934769	AAGATCCTTTTGGGTTGCCT	CATCCACATAGGCTTCAACG	59.5	256	61	retrotransposon protein putative Ty3-gypsy subclass
OsISM01393	LOC_Os01g09600	4935393	AAGAAACTCCTCACCGAGCC	ACCGTTACCGAGTGACCTTG	60.4	278	99	retrotransposon protein putative Ty3-gypsy subclass
OsISM01394	LOC_Os01g09690	4990573	TCATTGAAGGCGAAAGAACC	GCCCTTGCTAATCAACAACC	59.9	1301	91	hypothetical protein
OsISM01395	LOC_Os01g09690	4991840	AAGCAGCCAAGACTGGTTGT	GCCCACTATTCAATGTGCCT	59.9	172	90	hypothetical protein
OsISM01396	LOC_Os01g09690	4992010	GCCAAAGATAATTGGACCGA	TCCAGTTTTGGTTCGTTTCC	59.9	750	88	hypothetical protein
OsISM01397	LOC_Os01g09710	5009105	ATTGGAGGGATATTGGGAGG	CATCCAGGTTTGCAAGACAA	59.8	2055	74	hypothetical protein
OsISM01398	LOC_Os01g09730	5016238	CTCGAGCAACTTCTCCTTCG	TCCTTCTTCAGCGGATGGTA	60.5	183	97	expressed protein
OsISM01399	LOC_Os01g09760	5029134	AGCATGACGAGGATCCAAAT	AGCTCATCCTTGCTCCATTG	59.9	216	89	DIRP family protein putative expressed
OsISM01400	LOC_Os01g09760	5029354	CGCTTTTATGAAGCCTACCG	GATTTTCCACCCACAGATGC	60.1	166	69	DIRP family protein putative expressed
OsISM01401	LOC_Os01g09760	5029500	GCATCTGTGGGTGGAAAATC	ATCCCATGGCAGTACCTTCA	60.3	379	98	DIRP family protein putative expressed
OsISM01402	LOC_Os01g09760	5029880	TGTTGCATTGGTAACTGGTCA	TTTCCGTACCTTCCCAGATG	60.0	629	98	DIRP family protein putative expressed
OsISM01403	LOC_Os01g09760	5030546	ATGCTCACCGCTCCTATCAT	GGATGTCTTGGAATGTTCTTGA	59.4	279	95	DIRP family protein putative expressed
OsISM01404	LOC_Os01g09760	5031114	GCAATGGTGGCTAAAGAAGG	TCTCCAAGACATCTAAGACCAAC A	59.9	875	95	DIRP family protein putative expressed
OsISM01405	LOC_Os01g09760	5031983	TGGAGAGTTTAGTGACTGTAC CCA	ATGCAGAATCATCCCTTCCA	60.3	187	85	DIRP family protein putative expressed
OsISM01406	LOC_Os01g09760	5032238	AGTGCCTCCAAGACAAGGAA	CGCTCTTCTGGCAAATCTAAA	59.7	622	99	DIRP family protein putative expressed
OsISM01407	LOC_Os01g09760	5033043	CGTTTGCAATGGAGCAGATA	CAGATGACAAGCAATGCTGC	60.2	135	57	DIRP family protein putative expressed
OsISM01408	LOC_Os01g09760	5033274	CGAATTTGAGCCATCTGTCA	ATGATCAGAAAAACGTCGGG	59.9	181	87	DIRP family protein putative expressed

ISM IDs	Rice gene locus IDs	Physical positions (bp) on chromosomes	Forward primers (5'-3')	Reverse primers (5'-3')	Melting tempearature (T˚C)	Expected product size (bp) in PCR	Expected product size (bp) in Q-PCR	Putative Functions
OsISM01409	LOC_Os01g09760	5033668	TTTGATAGGCCTGACCTTGG	TTTTGAGGTCATCAGGCAGA	59.7	1089	89	DIRP family protein putative expressed
OsISM01410	LOC_Os01g09760	5034862	CATCCAAAACCATGCACATAA	TGGTCCACAATTACCAGATGT	58.5	289	96	DIRP family protein putative expressed
OsISM01411	LOC_Os01g09760	5035276	CAAGCAGATGGTTGACGAAG	CTGTACAATCCGTCGCTTCA	59.7	182	96	DIRP family protein putative expressed
OsISM01412	LOC_Os01g09760	5035506	TATTCAACTGCATTGCCACG	GGACGCCTGCTATGTTAGGA	60.5	170	79	DIRP family protein putative expressed
OsISM01413	LOC_Os01g09840	5090951	AATGTGTACCCCCAAGCAAA	CGAGTGCATTCTGTTTCACC	59.8	163	74	hypothetical protein
OsISM01415	LOC_Os01g09850	5099157	CAAGAAGAAGCGCAGCCT	ACACGTACCTGTTCGTCGC	59.6	168	93	ZOS1-04 - C2H2 zinc finger protein expressed
OsISM01416	LOC_Os01g09850	5099597	AAGGCCCACTCCAAGGTCT	GTGCGTGATGAAGCTGTCC	60.7	173	85	ZOS1-04 - C2H2 zinc finger protein expressed
OsISM01417	LOC_Os01g09900	5147949	GCTTCACCTGTGGCTATGCT	TTGAGACGGTGCTGAATCTG	60.2	225	95	helix-loop-helix DNA-binding domain containing protein expressed
OsISM01418	LOC_Os01g09900	5148143	TCATCAGCTCGCAGATTCAG	CATCGTCATCATCCATGCTC	60.1	220	67	helix-loop-helix DNA-binding domain containing protein expressed
OsISM01419	LOC_Os01g09900	5148916	GGAGAAGCTCAACGACAGC	TCACGTACTCCCTTGCTCTG	58.9	238	99	helix-loop-helix DNA-binding domain containing protein expressed
OsISM01420	LOC_Os01g09930	5162306	TGGATCCATGGCTACACAAA	GCTGCTGCGGAAACTCTACT	59.9	247	92	helix-loop-helix DNA-binding domain containing protein expressed
OsISM01421	LOC_Os01g09930	5162523	GCTGCAGCCCAGTAGAGTTT	GACTCGTCATCCATGCCC	59.8	198	68	helix-loop-helix DNA-binding domain containing protein expressed
OsISM01422	LOC_Os01g09930	5163150	GGAGAAGCTCAACGACAGC	TGATCAGTATCGACGCCTTG	59.3	2436	81	helix-loop-helix DNA-binding domain containing protein expressed
OsISM01423	LOC_Os01g09930	5165976	GCCCTCCTAGTGCAAACCTA	ACTTGTAGGAGCATCTGCCA	58.9	596	64	helix-loop-helix DNA-binding domain containing protein expressed
OsISM01424	LOC_Os01g09930	5166601	CAAAAGCCAACCATCAAACA	CGGACTGATCATCGTCACAT	59.5	818	85	helix-loop-helix DNA-binding domain containing protein expressed
OsISM01425	LOC_Os01g09940	5170254	CTCAACGGCAGCTTCGTC	CCCTTGCTCCGATCAGTATC	60.2	485	99	hypothetical protein
OsISM01426	LOC_Os01g09960	5175286	GTGGCACTAGGGCTGTCGT	TGCACCAACATAAGGTTCCA	60.6	948	81	transposon protein putative unclassified
OsISM01427	LOC_Os01g09960	5176167	GGTGTAGATGGATAGACTTGT TCG	TCAAAGTAAACTTCCCATTCCAA	59.5	272	64	transposon protein putative unclassified

ISM IDs	Rice gene locus IDs	Physical positions (bp) on chromosomes	Forward primers (5'-3')	Reverse primers (5'-3')	Melting tempearature (T˚C)	Expected product size (bp) in PCR	Expected product size (bp) in Q-PCR	Putative Functions
OsISM01428	LOC_Os01g09960	5177236	ACCATCGTCCATACCGTGTT	TCCAAGCCTTGCCATACTTC	60.2	312	64	transposon protein putative unclassified
OsISM01429	LOC_Os01g09960	5177895	AATTCACGAGACTGGTGCTG	GAATACGCTGCCTTAGTGCC	59.4	291	95	transposon protein putative unclassified
OsISM01430	LOC_Os01g09960	5178970	CCAGGAGGGTCACAAGAAAA	TAACTCGGAGCGGTTGAAG	59.5	318	99	transposon protein putative unclassified
OsISM01431	LOC_Os01g09960	5179269	CTTCAACCGCTCCGAGTTAG	CCCCAGCATTAGGAAACAGA	60.0	495	71	transposon protein putative unclassified
OsISM01432	LOC_Os01g09960	5180098	GATGGACTGCTTACAGCCTCA	GCCCAGAAACAAATCAATGG	60.4	223	46	transposon protein putative unclassified
OsISM01433	LOC_Os01g09960	5180359	TCGGGAAGAAACAAGACTGG	CTTCTTTGTTCTCACCCTGTCA	59.8	188	81	transposon protein putative unclassified
OsISM01434	LOC_Os01g09960	5180483	CGATGCTCACTATTGTTTCATCA	CCCCATTTCATTCACACTCC	60.2	446	98	transposon protein putative unclassified
OsISM01435	LOC_Os01g09960	5180955	TGAGGACACGGAGAACACAC	CAACCGACCTGCAACCTAAT	59.9	162	90	transposon protein putative unclassified
OsISM01438	LOC_Os01g09970	5189962	CAAGGTGTACAATGGGACGA	CAGGCCTCGTGTTTTTCATC	60.0	384	67	hypothetical protein
OsISM01439	LOC_Os01g09980	5194894	TTTAGCAAAGTTAGCAGGACCA	CCCCAAAAGTGAAGAATTCAA	59.1	577	96	conserved hypothetical protein
OsISM01440	LOC_Os01g09990	5201144	GCAACAACGGCTTCATGTG	TTGAGGTGGTGCTCAATCTG	60.6	247	98	helix-loop-helix DNA-binding domain containing protein expressed
OsISM01441	LOC_Os01g09990	5201326	TGCAGCCCAGTAGAGTTTCA	ACTCGTCATCCATCCCCAT	59.9	202	98	helix-loop-helix DNA-binding domain containing protein expressed
OsISM01442	LOC_Os01g09990	5202131	AGCTCAACGACAGCTTCCTC	TTGCTCTGATCAGTATCGATGTC	59.6	221	82	helix-loop-helix DNA-binding domain containing protein expressed
OsISM01443	LOC_Os01g10040	5235808	GCTGGAGTTCGTGTCGTG	AACAGGTGCGACCTGAACA	59.6	183	97	cytochrome P450 putative expressed
OsISM01444	LOC_Os01g10040	5236259	CTCCACGTCCAGCACCTC	CTTCAGCTGCTGCATCTCC	60.1	596	94	cytochrome P450 putative expressed
OsISM01445	LOC_Os01g10040	5236862	TCCAGGAATTTATTGTCGGC	TATCAGCCTCGCCATCTTCT	59.9	420	99	cytochrome P450 putative expressed
OsISM01446	LOC_Os01g10040	5237458	CGTCAAGTTCCTCAGCGAGT	TTTTTCGCCTCTTGAGCTGT	60.4	1154	75	cytochrome P450 putative expressed
OsISM01447	LOC_Os01g10040	5238622	TGAGACCTTGCAATGGACAG	ATGATCCCACCGATGATGTT	59.9	206	94	cytochrome P450 putative expressed

ISM IDs	Rice gene locus IDs	Physical positions (bp) on chromosomes	Forward primers (5'-3')	Reverse primers (5'-3')	Melting tempearature (T˚C)	Expected product size (bp) in PCR	Expected product size (bp) in Q-PCR	Putative Functions
OsISM01448	LOC_Os01g10040	5238923	CGCTCTACGATGAGCCCTAC	AAGGAGTGAAGCTGCCATTG	60.2	588	76	cytochrome P450 putative expressed
OsISM01449	LOC_Os01g10040	5239561	GCTTCCATCTTCCTTCACCA	AGTTGACGATGTGGTCCTCC	60.1	3386	68	cytochrome P450 putative expressed
OsISM01450	LOC_Os01g10060	5251059	GAGGAGGGTGTTGGAGGAC	ATTTGCGCCCATAGATGTCA	60.4	1004	83	hypothetical protein
OsISM01451	LOC_Os01g10070	5256008	CCACTCCCTCACCATCCA	CCGCACTAGTGAAAATGTACCA	60.3	292	98	expressed protein
OsISM01452	LOC_Os01g10170	5338454	TCGATAGGGAGGAGATCGGT	TCGATTATCCTGCCATCCTC	60.5	137	77	hypothetical protein
OsISM01454	LOC_Os01g10190	5356111	AAAGAAAGGTTTGCTGGTCG	GTGCCCCTACTCCATTCTCC	60.1	519	52	hypothetical protein
OsISM01455	LOC_Os01g10200	5368449	TGAGGTCGATCTCGGTGG	TGCACTGCTCTGCTCCTTT	60.3	317	99	hypothetical protein
OsISM01457	LOC_Os01g10240	5386186	GATCACGGCCTTCTCACC	AGCTCCGTGGATAGGACCAT	60.0	108	73	urate anion exchanger putative expressed
OsISM01458	LOC_Os01g10240	5386274	ATGGTCCTATCCACGGAGC	TCGATGGCCTCTTGCTTC	60.0	248	93	urate anion exchanger putative expressed
OsISM01459	LOC_Os01g10280	5405330	CATGCATCCTTCTCCTCCTC	GACGACCGTGTTGGTGAC	59.1	191	68	phosphoribosylaminoimidazole carboxylase chloroplast precursor putative expressed
OsISM01460	LOC_Os01g10280	5405642	ATGTCGTCGGGAGCTTCAAT	CAATTTCCACCGTCAAAACC	61.1	192	76	phosphoribosylaminoimidazole carboxylase chloroplast precursor putative expressed
OsISM01461	LOC_Os01g10280	5405882	ATTGTGAGCCCAAAGCCTC	TTCCACAAAGTCAGGTAACGG	60.1	516	99	phosphoribosylaminoimidazole carboxylase chloroplast precursor putative expressed
OsISM01462	LOC_Os01g10280	5406368	TTTGGAATTCCGTTACCTGAC	TAACCAAACATTTCCCCAGC	59.4	147	75	phosphoribosylaminoimidazole carboxylase chloroplast precursor putative expressed
OsISM01463	LOC_Os01g10280	5406564	GCTGTTGCTCACGACAAAAA	TACAAGCCATGCTCAAACCC	60.5	286	69	phosphoribosylaminoimidazole carboxylase chloroplast precursor putative expressed
OsISM01464	LOC_Os01g10280	5406822	CTCTTGGTGGGTTTGAGCAT	CGTAGAACCGTCTCTGCTCC	60.1	1308	96	phosphoribosylaminoimidazole carboxylase chloroplast precursor putative expressed
OsISM01465	LOC_Os01g10280	5408111	GAGCAGAGACGGTTCTACGG	CCTCAACAACATGGCAGATG	60.1	185	75	phosphoribosylaminoimidazole carboxylase chloroplast precursor putative expressed
OsISM01466	LOC_Os01g10280	5408372	TGCTGGTGTTTTCGCTGTAG	TGTGAGGCCTTGGAGCTACT	60.0	167	78	phosphoribosylaminoimidazole carboxylase chloroplast precursor putative expressed
OsISM01467	LOC_Os01g10280	5408655	TACAACATCCTGGGTGAGGA	TGCACCTGAAATGTCCAAAG	59.3	217	85	phosphoribosylaminoimidazole carboxylase chloroplast precursor putative expressed

ISM IDs	Rice gene locus IDs	Physical positions (bp) on chromosomes	Forward primers (5'-3')	Reverse primers (5'-3')	Melting tempearature (T˚C)	Expected product size (bp) in PCR	Expected product size (bp) in Q-PCR	Putative Functions
OsISM01468	LOC_Os01g10280	5408865	AGGTGCATCTGTCCATTGGT	TATGGCCCATCTTTCTCTGC	60.3	161	60	phosphoribosylaminoimidazole carboxylase chloroplast precursor putative expressed
OsISM01469	LOC_Os01g10280	5409034	GTGGGGCCTTCAAAGTACAG	TGCAGCACGAGGTTTAACTG	59.8	711	#VALUE!	phosphoribosylaminoimidazole carboxylase chloroplast precursor putative expressed
OsISM01470	LOC_Os01g10280	5409772	CCTGTCATGAAAGATGCTGC	TGTACGATGAGCCGAAACAA	59.8	182	79	phosphoribosylaminoimidazole carboxylase chloroplast precursor putative expressed
OsISM01471	LOC_Os01g10280	5410005	GTCATAATTGCAGGTGCAGG	GGACAGAAGTCAATGAAGCCA	59.7	462	65	phosphoribosylaminoimidazole carboxylase chloroplast precursor putative expressed
OsISM01472	LOC_Os01g10280	5410502	CATGGAACTGATTCCCTCCT	TAAACCTGCATTTTCCGCAT	59.7	230	91	phosphoribosylaminoimidazole carboxylase chloroplast precursor putative expressed
OsISM01473	LOC_Os01g10280	5410738	GTTAGGATGCTGGCCTCAAG	TCGAGCCTTTTTGCTTTCTC	59.8	598	99	phosphoribosylaminoimidazole carboxylase chloroplast precursor putative expressed
OsISM01474	LOC_Os01g10300	5416332	CGCTCGACTTCGAGACATC	GTGAATCCCTCAGGTTCTGG	59.6	541	97	expressed protein
OsISM01475	LOC_Os01g10350	5442460	GAGGAGTGGGTCACCGTTT	CAAGTTGGTATCGCCAGGTAA	60.0	690	46	MPPN domain containing protein expressed
OsISM01476	LOC_Os01g10350	5443210	GCGAACTGGATGCATATCCT	TAACTGTTGACCGTGCTTGG	59.9	1361	73	MPPN domain containing protein expressed
OsISM01477	LOC_Os01g10380	5463466	TCTTGGTCAAATGTTGCAGG	GCCTTCCATTGCAAGAACTC	59.8	162	97	transposon protein putative CACTA En/Spm sub-class
OsISM01478	LOC_Os01g10380	5464054	GCAGTGGCGAAACATAGACA	TGTCGTTACCAGGTTGCTTG	59.8	230	87	transposon protein putative CACTA En/Spm sub-class
OsISM01479	LOC_Os01g10380	5464723	GGCGATGTGGAAAAAGAACT	TCTCCTTTGGTTCCGGGT	59.8	218	99	transposon protein putative CACTA En/Spm sub-class
OsISM01480	LOC_Os01g10390	5469586	GCTCGAGACATTCACCCTTC	TACTTCTTCTTGGCGGCATT	59.8	251	67	transposon protein putative CACTA En/Spm sub-class
OsISM01481	LOC_Os01g10390	5470478	ATGACATCACTCAGCGGACA	CTGAAGGGTCCATTGGGAT	60.0	194	97	transposon protein putative CACTA En/Spm sub-class
OsISM01482	LOC_Os01g10390	5471696	CGCCCTCGACGTCTCTATTA	ATCCAGTATCGAAAACCCCC	60.2	190	81	transposon protein putative CACTA En/Spm sub-class
OsISM01483	LOC_Os01g10430	5492719	GCCGAGTCGTTCGAGAAG	CAAGATCGCGAGCTCTGTAA	59.5	991	71	protein kinase family protein putative expressed
OsISM01484	LOC_Os01g10430	5493890	TGGAACATGACCTAGCTGGA	GCTCAAGGCCACAGAGTAGC	59.7	271	97	protein kinase family protein putative expressed
OsISM01485	LOC_Os01g10430	5494393	TGCTGAGTTATATGCTGGCAA	CTGATGGTGAACCACAGAGTTT	59.3	553	90	protein kinase family protein putative expressed

ISM IDs	Rice gene locus IDs	Physical positions (bp) on chromosomes	Forward primers (5'-3')	Reverse primers (5'-3')	Melting tempearature (T°C)	Expected product size (bp) in PCR	Expected product size (bp) in Q-PCR	Putative Functions
OsISM01486	LOC_Os01g10430	5495088	AGCAGATCGAGGAACAGCAT	ATCTCGGCAGACTTGAAGGA	60.0	845	87	protein kinase family protein putative expressed
OsISM01487	LOC_Os01g10430	5495913	TCCTTCAAGTCTGCCGAGAT	TGCTTTCCTCCAGCAATACC	60.1	604	94	protein kinase family protein putative expressed
OsISM01488	LOC_Os01g10430	5496569	CCTGACGCAAATGCAGAAT	GCCAGTGATTACTATGTATGTGA ATG	59.4	150	99	protein kinase family protein putative expressed
OsISM01489	LOC_Os01g10430	5496604	GGTAATACCTTGTTGACTGTT ATTGAA	GCTCTTGGCATTGACTTGGT	59.6	323	87	protein kinase family protein putative expressed
OsISM01490	LOC_Os01g10430	5497311	GACAGGAGTAGCAGGGTGGA	CCCAGAATGGTGGATCTTGT	60.0	377	67	protein kinase family protein putative expressed
OsISM01491	LOC_Os01g10440	5507303	CGACTACCCTCTCATGACCC	ATGAAGTTGGTGTTCCTCGG	59.7	153	70	xylosyltransferase putative expressed
OsISM01492	LOC_Os01g10440	5507462	CACCGGCTATCTAGGATGGA	CCACGTAGAAGATGTCCTGCT	59.7	192	96	xylosyltransferase putative expressed
OsISM01493	LOC_Os01g10460	5530065	AGGAGAGAAGAGTGAGGGGG	GGTCATCCAGCACAACAAGA	59.7	269	78	conserved hypothetical protein
OsISM01494	LOC_Os01g10460	5530314	TCTTGTTGTGCTGGATGACC	ACAATGCTTTGAAAATCGCC	59.9	344	88	conserved hypothetical protein
OsISM01495	LOC_Os01g10460	5530639	GCGATTTTCAAAGCATTGTG	CTTGACTTCTCGGCCTTACC	59.1	1964	60	conserved hypothetical protein
OsISM01496	LOC_Os01g10550	5599071	TTTTGTTGTTGTTGTTGTGGTG	GTACAGGAGCTCCGCCTCC	60.9	696	74	DEFL35 - Defensin and Defensin-like DEFL family
OsISM01497	LOC_Os01g10580	5639496	CTCAACGAGTTCTTCGGCTT	GTCCATGCTCCCGATCTT	59.1	300	97	B-box zinc finger family protein putative expressed
OsISM01499	LOC_Os01g10590	5651925	AAGCAATCCGACGTTGAGAG	ACGAATGTTTCCCCATACCA	60.2	264	90	osFTL8 FT-Like8 homologous to Flowering Locus T gene; contains Pfam profile PF01161: Phosphatidylethanolamine-binding protein expressed
OsISM01500	LOC_Os01g10590	5652123	TGCACAGGTTGGTGACAGAT	GCTCTCGTAGCACACCACCT	60.3	2008	72	osFTL8 FT-Like8 homologous to Flowering Locus T gene; contains Pfam profile PF01161: Phosphatidylethanolamine-binding protein expressed
OsISM01502	LOC_Os01g10700	5727477	CGATAGCTCCAGAGTTTCAGA AC	GGACTTGTGATACCAAGGTACA GTC	59.9	140	69	D-mannose binding lectin family protein
OsISM01503	LOC_Os01g10700	5727636	TCCAGGATACGATTCTTTAGC A	CTCGTCTTCGCCATCTTTGT	59.6	185	74	D-mannose binding lectin family protein
OsISM01505	LOC_Os01g10790	5760551	GAGGCGACAAATCAACCAAT	TCCAGCAGAGGCATACACAA	60.2	175	73	WD domain G-beta repeat domain containing protein expressed
OsISM01506	LOC_Os01g10790	5760707	TGTGTATGCCTCTGCTGGAA	AGTGTCCAATGGCTTTGCTT	60.1	162	74	WD domain G-beta repeat domain containing protein expressed

ISM IDs	Rice gene locus IDs	Physical positions (bp) on chromosomes	Forward primers (5'-3')	Reverse primers (5'-3')	Melting tempearature (T˚C)	Expected product size (bp) in PCR	Expected product size (bp) in Q-PCR	Putative Functions
OsISM01507	LOC_Os01g10790	5760850	AGCAAAGCCATTGGACACTT	CATCTGCAGCAGCAAGAAAA	60.0	252	87	WD domain G-beta repeat domain containing protein expressed
OsISM01508	LOC_Os01g10790	5761087	TTGCTGCTGCAGATGACAGT	GTGGGCTTCCCTCAATCTTT	60.6	564	81	WD domain G-beta repeat domain containing protein expressed
OsISM01509	LOC_Os01g10790	5761631	AAAGATTGAGGGAAGCCCAC	CCAAGGTCTCCAAGGAATGA	60.2	188	63	WD domain G-beta repeat domain containing protein expressed
OsISM01510	LOC_Os01g10790	5761799	TCATTCCTTGGAGACCTTGG	AAAACAGTGTTCGCCCTTTG	60.1	236	88	WD domain G-beta repeat domain containing protein expressed
OsISM01511	LOC_Os01g10790	5761994	GCTTGCTGTGTGGGATTTCT	GATTGAAACATTGCCCACCT	60.0	366	99	WD domain G-beta repeat domain containing protein expressed
OsISM01512	LOC_Os01g10790	5762573	AAGGGAACAGGATCCACCTT	AAGAAACTTCCCCTTCTCGC	59.8	174	96	WD domain G-beta repeat domain containing protein expressed
OsISM01513	LOC_Os01g10790	5762818	CAGTGCTGAATTGGTGTTGG	TCAGGTTATCTGAATCAGTCGG	59.7	256	81	WD domain G-beta repeat domain containing protein expressed
OsISM01514	LOC_Os01g10810	5767358	TCCCAGGATCATTTGCTCTC	TATTCCGTGGAGGACAAAGC	60.1	281	64	rho termination factor N-terminal domain containing protein expressed
OsISM01515	LOC_Os01g10810	5767815	CCGTTCGAAGAGGTTGAGAG	CATGTTCCTCCGAAACATCA	59.7	252	62	rho termination factor N-terminal domain containing protein expressed
OsISM01516	LOC_Os01g10820	5769078	CGAGCAACACTAAACCCCC	GTTCGCCTGCTTCTTCTCC	60.3	214	97	ribosomal protein L5 putative expressed
OsISM01517	LOC_Os01g10820	5769325	CTCAACATCTCCGTCGGC	TTTACCCTTGGAGAACACCG	60.2	535	97	ribosomal protein L5 putative expressed
OsISM01518	LOC_Os01g10820	5769809	TAGGTGCTGGAGCAGCTGAG	GATCTTCTCATTACGCCGGA	61.0	183	91	ribosomal protein L5 putative expressed
OsISM01519	LOC_Os01g10820	5770103	GGTTTTGGTATCCAGGAGCA	CGCTCAAGAACGACGTAGAA	59.6	787	90	ribosomal protein L5 putative expressed
OsISM01520	LOC_Os01g10850	5788721	CTTCTTCCACGACTGCTTCG	GTGTCGTCGAGCAAGATGG	60.8	136	55	peroxidase precursor putative expressed
OsISM01522	LOC_Os01g10880	5807075	AAGCTCCTCCGCTTCCTCT	GTAGTCGCGGTACGTGTTGA	60.2	173	73	expressed protein
OsISM01523	LOC_Os01g10910	5820350	AAAAATGTTTGCATTGCTGC	ATGGGACCAACAACATTTGC	59.7	355	96	transposon protein putative unclassified
OsISM01524	LOC_Os01g10910	5820925	AGTTCCACTATTCCAGGCCC	TGGATAATGAACCAGCACCA	60.1	169	95	transposon protein putative unclassified
OsISM01525	LOC_Os01g10970	5843850	TGCCTCTCGCCATTATTCTC	AGAAAAGCTTGGCAAACAGC	60.0	218	70	auxin-induced protein 5NG4 putative

ISM IDs	Rice gene locus IDs	Physical positions (bp) on chromosomes	Forward primers (5'-3')	Reverse primers (5'-3')	Melting tempearature (T°C)	Expected product size (bp) in PCR	Expected product size (bp) in Q-PCR	Putative Functions
OsISM01526	LOC_Os01g10970	5844048	GCTGTTTGCCAAGCTTTTCT	GTACTTGAGGCCCGTGTTGT	59.8	169	77	auxin-induced protein 5NG4 putative
OsISM01527	LOC_Os01g10970	5844254	TGCCCGTACTGACCTTCTTC	CTCAGCCGGATCACTTCC	59.8	2947	56	auxin-induced protein 5NG4 putative
OsISM01528	LOC_Os01g10980	5855603	CTCTCTTGCTGCTGCCTCTC	GCTTAGTGAACAACCGGAGC	60.2	191	76	auxin-induced protein 5NG4 putative
OsISM01529	LOC_Os01g10980	5855774	GCTCCGGTTGTTCACTAAGC	GAGGTGTACTTGAGGCCCAG	59.8	188	88	auxin-induced protein 5NG4 putative
OsISM01530	LOC_Os01g10980	5856029	AGGCGAGCTCAATCAATCAT	CTCTTCAGCCGGATCACTTC	59.9	141	51	auxin-induced protein 5NG4 putative
OsISM01531	LOC_Os01g10990	5861000	CTTCATCTTCTACCGCCAGG	ACAATCTGAGCGACATCGG	59.8	200	96	integral membrane protein DUF6 containing protein expressed
OsISM01532	LOC_Os01g10990	5861182	CGATGTCGCTCAGATTGTTC	GTGGTGTACTTGAGGCCCAT	59.6	415	95	integral membrane protein DUF6 containing protein expressed
OsISM01534	LOC_Os01g11040	5891201	GAGGCAGCACTTCCATCTCT	AAGTTGTGCATGCCAATCAG	59.6	577	94	SCAR-like protein 2 putative expressed
OsISM01535	LOC_Os01g11040	5891840	AGAATGTCGAGACCCACCAC	AATGTCTCCTCGAACAAGCC	59.6	142	78	SCAR-like protein 2 putative expressed
OsISM01536	LOC_Os01g11040	5892005	GCATGGGATATGATGAGAGC	TGATACGGTTCTCTCAAGCG	58.5	656	99	SCAR-like protein 2 putative expressed
OsISM01537	LOC_Os01g11040	5892676	CAGGCAGAGGAATGGTGAAT	CCGGCCATCAGTACTAGGAG	59.9	222	86	SCAR-like protein 2 putative expressed
OsISM01538	LOC_Os01g11040	5895752	CACCAGATCAACAAGGGCAT	TCAATGTCGAGAGGATTGCTT	60.4	177	80	SCAR-like protein 2 putative expressed
OsISM01539	LOC_Os01g11040	5895941	CTTGCTTCAGCAAATCAGGG	CATGTCCATTCAGCTTCTGC	60.2	338	51	SCAR-like protein 2 putative expressed
OsISM01540	LOC_Os01g11040	5896315	TGGATGAAAGGGAGGAGTTG	CAGTGGTTGGTGATGAGGTG	60.0	376	97	SCAR-like protein 2 putative expressed
OsISM01541	LOC_Os01g11040	5896707	AGCAATCTTGGAAAAGGCAA	ATCACCTCCCTCATCACTGG	59.9	146	59	SCAR-like protein 2 putative expressed
OsISM01542	LOC_Os01g11054	5898945	TCGACTACGAGACCCTCCTC	ACAGCCTCAGGCAATCCTC	59.9	201	97	phosphoenolpyruvate carboxylase putative expressed
OsISM01543	LOC_Os01g11054	5899496	TCACTCCTCCAGAAGCACG	ATCTCCTGCTCCTCGTTGTC	59.8	186	84	phosphoenolpyruvate carboxylase putative expressed
OsISM01544	LOC_Os01g11054	5899662	GACAACGAGGAGCAGGAGAT	GGATCTCGTCCGTCTTGAAC	59.5	179	71	phosphoenolpyruvate carboxylase putative expressed

ISM IDs	Rice gene locus IDs	Physical positions (bp) on chromosomes	Forward primers (5'-3')	Reverse primers (5'-3')	Melting tempearature (T˚C)	Expected product size (bp) in PCR	Expected product size (bp) in Q-PCR	Putative Functions
OsISM01545	LOC_Os01g11054	5899991	CCCTCATCCAGTTCTCCTCA	CACACGTCCCTGGTAACCTC	60.3	180	80	phosphoenolpyruvate carboxylase putative expressed
OsISM01546	LOC_Os01g11054	5900211	AAGATGGCCGATTTGATGTT	CTTCCTTGAGGACTTCCGGT	60.0	392	94	phosphoenolpyruvate carboxylase putative expressed
OsISM01547	LOC_Os01g11054	5900592	CCTCAAGGAAGTACGCCAAA	AATGCGGTAAGGTTCTCCTG	59.7	5363	69	phosphoenolpyruvate carboxylase putative expressed
OsISM01548	LOC_Os01g11054	5906005	CCAGATCTTGTCGAAGGGAA	ACAGCGATCGGTAGCACAG	60.1	273	99	phosphoenolpyruvate carboxylase putative expressed
OsISM01549	LOC_Os01g11054	5907597	GCCAACTACGTCGAGACACA	GCCTCAGGTACGGATCACTC	59.8	422	80	phosphoenolpyruvate carboxylase putative expressed
OsISM01550	LOC_Os01g11070	5910919	ACTTCCTCCTCTACCGCTCC	CCCAGCTACAAGAGCGTGA	60.0	1688	99	Alg9-like mannosyltransferase protein putative expressed
OsISM01551	LOC_Os01g11070	5912610	GCTTCAATGATTTTTGGGGA	GGCCAAGAAAGATTCTCACAG	59.4	177	62	Alg9-like mannosyltransferase protein putative expressed
OsISM01552	LOC_Os01g11070	5912877	GCTTAACCAGTGGCTGCTTC	CATGGAGAATGAACTTGGCA	59.8	166	57	Alg9-like mannosyltransferase protein putative expressed
OsISM01553	LOC_Os01g11070	5913211	GGATTTCTGACTTCCATGTTCC	CACTGATGCTGTCCATTTGC	60.0	158	79	Alg9-like mannosyltransferase protein putative expressed
OsISM01554	LOC_Os01g11070	5913587	CTTGCAGGCACACAAAGAAG	GCAGCTGCAACACATATCAA	59.0	631	64	Alg9-like mannosyltransferase protein putative expressed
OsISM01555	LOC_Os01g11070	5914219	GCTGTTATTGACACCTTTCCA G	AAGGATCAAAGGCCGCAG	60.0	187	97	Alg9-like mannosyltransferase protein putative expressed
OsISM01556	LOC_Os01g11070	5914447	GTTGAATGGATATGGTGCCC	CCATTCACTCCCAACACAGA	59.8	432	95	Alg9-like mannosyltransferase protein putative expressed
OsISM01557	LOC_Os01g11070	5914989	CACCACTGCTGCTCCATCT	GCTACATGCCCCAATGTCTT	60.0	169	80	Alg9-like mannosyltransferase protein putative expressed
OsISM01558	LOC_Os01g11070	5915194	TCCCGAGGAAGTGATTTGTC	CTCTATCCAGAAAGGGCAGC	59.5	149	59	Alg9-like mannosyltransferase protein putative expressed
OsISM01559	LOC_Os01g11110	5920900	AGCGACGACCTCGTCTACAT	CCCTCGTCTAACTTTGCCAC	59.8	10172	96	14-3-3 protein putative expressed
OsISM01560	LOC_Os01g11110	5931292	CCACATTCCTCTGGAGCTGA	CCCAGTCTTGAACTCTGCAA	60.2	165	85	14-3-3 protein putative expressed
OsISM01561	LOC_Os01g11110	5931430	CGGTACCTTGCAGAGTTCAA	GATCTGTCTGCGCAGTTTTG	59.3	618	98	14-3-3 protein putative expressed
OsISM01562	LOC_Os01g11110	5932054	CAACTGACCCTATCAGACTTG G	GCGTTCTTAGCAAGTTGGCA	60.1	182	98	14-3-3 protein putative expressed

ISM IDs	Rice gene locus IDs	Physical positions (bp) on chromosomes	Forward primers (5'-3')	Reverse primers (5'-3')	Melting tempearature (T°C)	Expected product size (bp) in PCR	Expected product size (bp) in Q-PCR	Putative Functions
OsISM01563	LOC_Os01g11110	5932333	TGGAATTCTGATATGGCTGATG	GGGTCACCTTTAGCACCAGT	59.5	399	72	14-3-3 protein putative expressed
OsISM01564	LOC_Os01g11110	5932712	ACTGGTGCTAAAGGTGACCC	CCACACCCTTGTTGTTGTTG	59.5	182	86	14-3-3 protein putative expressed
OsISM01565	LOC_Os01g11140	5961032	CATTGAAAGCCTCTTTGCGT	GAGATCCTCCTCGTCCAGTG	60.1	168	47	retrotransposon protein putative unclassified
OsISM01566	LOC_Os01g11140	5962035	TCGATCGAGTTGTTAGCACA	CCCAATCTGTTTGCATGGTT	59.4	345	78	retrotransposon protein putative unclassified
OsISM01567	LOC_Os01g11170	5990130	ATCAACCTCGAGCTGCTCTC	CCACCGCAGTCTACCACC	59.9	545	87	hypothetical protein
OsISM01568	LOC_Os01g11190	5993790	CGGTGATGAGGAAGATGGAG	AGCCACCACATCCACGAC	60.6	396	81	hypothetical protein
OsISM01569	LOC_Os01g11210	6003039	GGACCTCGTCTTTGAGCAAG	ACTCCTTCTGCAGCAAGCTC	59.9	190	75	expressed protein
OsISM01570	LOC_Os01g11210	6003268	GCTTGCTGCTCACTGACGTA	GCCTCGAGTCACGTTGGTAT	60.2	246	97	expressed protein
OsISM01571	LOC_Os01g11210	6003478	AGCAATTGACTGGCCGATAC	AGGAGAAAGCATGGTCTCCA	60.0	174	93	expressed protein
OsISM01572	LOC_Os01g11260	6025747	TGCAATCGTAAAATACCGCA	CCAGATAGATAAGTAAATCCCAGCA	60.0	180	70	phospho-N-acetylmuramoyl-pentapeptide-transferase putative expressed
OsISM01573	LOC_Os01g11260	6025893	GCAGATTGTTGCTGGGATTT	CAAGAGGAGGAGGAAGAGGAA	60.0	709	99	phospho-N-acetylmuramoyl-pentapeptide-transferase putative expressed
OsISM01574	LOC_Os01g11260	6026725	TTGCTGGATTGTCTGTTGCT	CCTGACATTGATGCTCCAAA	59.5	539	68	phospho-N-acetylmuramoyl-pentapeptide-transferase putative expressed
OsISM01575	LOC_Os01g11260	6027410	TTCTTCTTGAGCTGCTCTTGG	GGGAAGAATACGCAAGGGAT	60.1	803	96	phospho-N-acetylmuramoyl-pentapeptide-transferase putative expressed
OsISM01576	LOC_Os01g11330	6068513	GGAGTCCTCTTCGGCTCCT	CTCCCAAACCGCCTCTTT	60.5	186	89	retrotransposon protein putative unclassified
OsISM01577	LOC_Os01g11330	6069097	GGCTTTACCATGCCGGTT	AGTAGGTCATGTGGTGCGGT	60.7	1179	97	retrotransposon protein putative unclassified
OsISM01578	LOC_Os01g11370	6111979	CACCAGCTCCTCCTCCTCAT	GGTACTTGTACACCGGCAGC	61.2	198	78	ATP-dependent RNA helicase putative expressed
OsISM01579	LOC_Os01g11370	6112243	GAAAGTCCACGCAGATCCC	TATAAGTCGACCACCCTCGG	60.3	811	63	ATP-dependent RNA helicase putative expressed
OsISM01580	LOC_Os01g11370	6113060	ACTCAACCAAGACGATTGGC	TTCTTCCGCTACTCTTGATGC	59.9	869	52	ATP-dependent RNA helicase putative expressed

ISM IDs	Rice gene locus IDs	Physical positions (bp) on chromosomes	Forward primers (5'-3')	Reverse primers (5'-3')	Melting tempearature (T˚C)	Expected product size (bp) in PCR	Expected product size (bp) in Q-PCR	Putative Functions
OsISM01581	LOC_Os01g11370	6113966	TCCGATTTGAGGATCAAACA	TGGTCAGAAGAGGATCTTCCA	59.4	278	97	ATP-dependent RNA helicase putative expressed
OsISM01582	LOC_Os01g11370	6114223	TGGAAGATCCTCTTCTGACCA	GAAGACCCAGCAACATGTCA	59.7	349	88	ATP-dependent RNA helicase putative expressed
OsISM01583	LOC_Os01g11370	6114555	CATGTTGCTGGGTCTTCTGA	TGCTTCAATTGTTGCAGAGG	59.9	193	80	ATP-dependent RNA helicase putative expressed
OsISM01584	LOC_Os01g11370	6114733	GCAACAATTGAAGCAAGATCC	GGGCAAATGATCAGCAGACT	60.0	807	82	ATP-dependent RNA helicase putative expressed
OsISM01585	LOC_Os01g11370	6115520	AGTCTGCTGATCATTTGCCC	TGGATTTCCACTGTGTAACCTTT	60.0	170	74	ATP-dependent RNA helicase putative expressed
OsISM01586	LOC_Os01g11370	6115720	ACAGGCTGCTGTGAATACTGT	GCTGCGTCTATGTCATCCTG	58.5	969	94	ATP-dependent RNA helicase putative expressed
OsISM01587	LOC_Os01g11370	6116669	CAGGATGACATAGACGCAGC	GCCAGAGTACAAGGGCAGAA	59.9	281	97	ATP-dependent RNA helicase putative expressed
OsISM01588	LOC_Os01g11370	6116939	TGTACTCTGGCCTTCCGC	TGACGTCTCAGCAATGTTTG	59.2	177	99	ATP-dependent RNA helicase putative expressed
OsISM01589	LOC_Os01g11370	6117092	TCAACAAACATTGCTGAGACG	CTTCTGCTTGGAAAACCCAC	59.8	149	76	ATP-dependent RNA helicase putative expressed
OsISM01590	LOC_Os01g11370	6117223	GGGTTTTCCAAGCAGAAGTG	GGATGCCTTGGATATTGGTG	59.9	276	76	ATP-dependent RNA helicase putative expressed
OsISM01591	LOC_Os01g11370	6117528	GGTGCGACCAGGAAAGTG	TGCATTTCAGGAATCCCTTC	60.1	1276	82	ATP-dependent RNA helicase putative expressed
OsISM01592	LOC_Os01g11370	6118788	GGATTCCTGAAATGCAAAGG	GCCAATCAAATCCCAAAATG	59.8	1244	88	ATP-dependent RNA helicase putative expressed
OsISM01593	LOC_Os01g11370	6120114	AACAGTACCCCTTGGCTTCC	GAGCATCCGAAATTATTGGC	59.9	1085	88	ATP-dependent RNA helicase putative expressed
OsISM01594	LOC_Os01g11370	6121177	CAGCCAATAATTTCGGATGC	CCTCTCATGGAAACCCACAC	60.4	187	83	ATP-dependent RNA helicase putative expressed
OsISM01595	LOC_Os01g11370	6121387	CTAAGCTCCGTTTTGCTGCT	TCCAGATTGATGAAATCCTTTG	59.4	464	75	ATP-dependent RNA helicase putative expressed
OsISM01596	LOC_Os01g11370	6121874	GAACTTTTTGAACCACCAAGC	AGCTGCTCCCTGATGTCAAT	59.3	180	52	ATP-dependent RNA helicase putative expressed
OsISM01597	LOC_Os01g11370	6122059	GGATTATCAAGAGGTTTGGCA	ATGCATAAGCAAATGCACCA	59.6	883	94	ATP-dependent RNA helicase putative expressed
OsISM01598	LOC_Os01g11370	6122916	TATTGCTGGTGCATTTGCTT	TGTACATCCCATTCTGGCTG	59.4	818	60	ATP-dependent RNA helicase putative expressed

ISM IDs	Rice gene locus IDs	Physical positions (bp) on chromosomes	Forward primers (5'-3')	Reverse primers (5'-3')	Melting tempearature (T˚C)	Expected product size (bp) in PCR	Expected product size (bp) in Q-PCR	Putative Functions
OsISM01599	LOC_Os01g11370	6123716	GCCAGAATGGGATGTACAAAA	TGGTAGATGACCCACTTTGGA	60.1	193	98	ATP-dependent RNA helicase putative expressed
OsISM01600	LOC_Os01g11414	6133608	CTCCTCCTCCCCATCCTC	CTCTACTCCCGGACAGACCA	59.9	2273	71	sodium/calcium exchanger protein putative expressed
OsISM01601	LOC_Os01g11414	6135999	TGGCGTTGCAGTTGATTTAC	CTGCATAGCTGGTTTGGACA	59.8	356	84	sodium/calcium exchanger protein putative expressed
OsISM01602	LOC_Os01g11414	6136440	TGTCTTGCTGGCACTTATCG	GCGTATGCAAGCTTCCTCTT	59.8	205	97	sodium/calcium exchanger protein putative expressed
OsISM01603	LOC_Os01g11414	6136719	ATGGCACACCCAATGAAGAT	GGTTTGGCTCTCATCCATGT	60.1	154	66	sodium/calcium exchanger protein putative expressed
OsISM01604	LOC_Os01g11414	6137053	GTCCCTACCAGTGGTGCCTA	GTCCACCAGAAGGTCGTGTT	60.0	236	82	sodium/calcium exchanger protein putative expressed
OsISM01605	LOC_Os01g11414	6137527	AGCTACGGACCAGTTCCCTT	TGATGTAGATGAGCGCCAAG	60.1	197	97	sodium/calcium exchanger protein putative expressed
OsISM01607	LOC_Os01g11530	6208859	TTTCTAAGAATCAACCCGCC	AACCTAGCTACCCCAGCCAT	59.6	418	86	hypothetical protein
OsISM01608	LOC_Os01g11580	6247512	CAGGATCAGATGCCTCCAGT	AGTCGCCTACCTGCTGTGTT	60.1	181	96	bromodomain containing protein expressed
OsISM01609	LOC_Os01g11580	6247657	CGAACCACCTAAAGAAAACAC A	TGAACAGGTGATGATGAGCC	59.4	189	64	bromodomain containing protein expressed
OsISM01610	LOC_Os01g11580	6247903	TAGCGATTCAGGCTCTTCGT	TACGTTCTGGGTGACTGTGC	59.9	212	94	bromodomain containing protein expressed
OsISM01611	LOC_Os01g11600	6256984	GTCGTCGTCTCCTTCATTGG	CTCCAGCAGCACGGTCAC	61.2	212	94	conserved hypothetical protein
OsISM01612	LOC_Os01g11620	6266787	CCGCGTCATCATCGACTT	CTTCTTGAAGTCGCCGCC	61.4	178	83	GDSL-like lipase/acylhydrolase putative expressed
OsISM01613	LOC_Os01g11620	6267068	AAATCCAGTGGTTCCAGCAG	GCGACTTGCTCAGGTAGCTC	60.2	157	70	GDSL-like lipase/acylhydrolase putative expressed
OsISM01614	LOC_Os01g11620	6267325	CAAGATCACAAGCGGCGT	GTCAGGTACAGCGGGAAGC	60.9	194	97	GDSL-like lipase/acylhydrolase putative expressed
OsISM01615	LOC_Os01g11620	6267680	AGGTCACCCAGATGGTTCAG	CAGCACACTTTCAGGCCATA	59.9	1659	62	GDSL-like lipase/acylhydrolase putative expressed
OsISM01617	LOC_Os01g11650	6278213	CTCCATCTGCAAGTCCCCT	AACAGCGGCGAGTTGTAGTC	60.3	192	97	GDSL-like lipase/acylhydrolase putative expressed
OsISM01618	LOC_Os01g11650	6278452	TTGACTCCATCGGCAAGG	GAGGTACACCGGGAAGCAC	60.6	193	99	GDSL-like lipase/acylhydrolase putative expressed

ISM IDs	Rice gene locus IDs	Physical positions (bp) on chromosomes	Forward primers (5'-3')	Reverse primers (5'-3')	Melting tempearature (T˚C)	Expected product size (bp) in PCR	Expected product size (bp) in Q-PCR	Putative Functions
OsISM01619	LOC_Os01g11650	6278813	ATCCAGTTCGTCCTCCACG	TGAAGTTGTACTCCCCGACC	60.5	3265	92	GDSL-like lipase/acylhydrolase putative expressed
OsISM01621	LOC_Os01g11660	6287965	CAGCTAGGCTGGTTCGACTC	TCTCCCATGAAGAAGAGGGA	59.9	243	93	GDSL-like lipase/acylhydrolase putative expressed
OsISM01622	LOC_Os01g11660	6288282	TGGTGAAAACCATCTCGTCA	ATCCCAATGGTGGTATCCCT	60.2	2084	88	GDSL-like lipase/acylhydrolase putative expressed
OsISM01623	LOC_Os01g11660	6290550	GGTCATTCGAATCGTCCAGT	TTGTACTTCCCACCTCCACC	59.9	471	85	GDSL-like lipase/acylhydrolase putative expressed
OsISM01624	LOC_Os01g11670	6295021	GTCGTGCTCCTGCTCGTC	GCCTCGTAGTAGGCGAAGAA	60.2	155	99	OsSCP2 - Putative Serine Carboxypeptidase homologue expressed
OsISM01625	LOC_Os01g11700	6304107	CCTCTCCGATGGAAGATTGA	ATGCTCGAAGCTCCGGTT	60.5	209	95	GDSL-like lipase/acylhydrolase putative expressed
OsISM01626	LOC_Os01g11700	6304575	GGTCTCTCACACCCCGAGT	TGATTCCCGACACGAACAC	60.3	163	91	GDSL-like lipase/acylhydrolase putative expressed
OsISM01629	LOC_Os01g11710	6319008	CAGCTCAAGCCCTCACTCTG	GTTGTAGTCGTTTCCCCCAA	60.6	204	97	GDSL-like lipase/acylhydrolase putative expressed
OsISM01631	LOC_Os01g11710	6319634	CATGTCCTTCGCCCAGAA	GCCTTCGGGTTGAAGTTGTA	60.4	1286	97	GDSL-like lipase/acylhydrolase putative expressed
OsISM01632	LOC_Os01g11730	6340102	CCTACGGCGAGACCTTCTTC	AGCAAGGGGACTCCAAGG	60.7	209	95	GDSL-like lipase/acylhydrolase putative expressed
OsISM01633	LOC_Os01g11730	6340470	GCTCAAGCCTACCTTGTGCT	CCTATCTCGCCCATGAAGAA	59.9	1487	79	GDSL-like lipase/acylhydrolase putative expressed
OsISM01634	LOC_Os01g11730	6342007	CCATGTCATACGTGCCAAAG	TACCTGGCTCCCTCCTTGAT	60.5	176	74	GDSL-like lipase/acylhydrolase putative expressed
OsISM01635	LOC_Os01g11730	6342403	ATCAAGTTCGTGCAAAACCC	CACGTCGTAGTTGTACGCCC	60.8	401	97	GDSL-like lipase/acylhydrolase putative expressed
OsISM01636	LOC_Os01g11740	6352887	GCCGACTCATCATCGACTTC	GGAGGAAAGGGACTCCAAAG	60.4	145	49	GDSL-like lipase/acylhydrolase putative expressed
OsISM01637	LOC_Os01g11740	6353191	TCAGACCTACCTTGTGCTCG	CTCTCCCATGAAGAAGAGCG	59.6	208	72	GDSL-like lipase/acylhydrolase putative expressed
OsISM01638	LOC_Os01g11740	6353466	ACGTACCAAAGGTCATCGGA	ACGTACCTGGCTCCCTCCT	60.7	2276	68	GDSL-like lipase/acylhydrolase putative expressed
OsISM01639	LOC_Os01g11740	6355935	TTTGCCGACTACTACGACCC	GGAGCTTGGACGAGTCACTG	60.6	849	77	GDSL-like lipase/acylhydrolase putative expressed
OsISM01640	LOC_Os01g11750	6360731	CCTACGGCGAGACCTTCTTC	GAGCAAGGGGACTCCAAAGT	61.0	197	96	GDSL-like lipase/acylhydrolase putative expressed

ISM IDs	Rice gene locus IDs	Physical positions (bp) on chromosomes	Forward primers (5'-3')	Reverse primers (5'-3')	Melting tempearature (T˚C)	Expected product size (bp) in PCR	Expected product size (bp) in Q-PCR	Putative Functions
OsISM01641	LOC_Os01g11750	6361086	GCTCAAGCCTACCTTGTGCT	GAACTCGCCCATGAAGAAGA	60.0	192	77	GDSL-like lipase/acylhydrolase putative expressed
OsISM01642	LOC_Os01g11750	6361332	ATGTCGTACGTGCCAAAGGT	ACGTACCTGGCTCCCTCCT	60.7	171	75	GDSL-like lipase/acylhydrolase putative expressed
OsISM01643	LOC_Os01g11750	6361719	GATCGAGTTCGTGCGAATG	GTCGCGTTGTAGTTGTACCG	59.8	576	97	GDSL-like lipase/acylhydrolase putative expressed
OsISM01644	LOC_Os01g11760	6364302	CCGCATCATCATGGATTTC	AAGAGATGGTGGGACGAATG	59.9	158	59	GDSL-like lipase/acylhydrolase putative expressed
OsISM01645	LOC_Os01g11760	6364587	AGCTGGAGTGGTTCCACAAG	CCGAACTCTCCCATGAAGAA	60.2	204	98	GDSL-like lipase/acylhydrolase putative expressed
OsISM01646	LOC_Os01g11760	6365249	TCATCAAGAAACCGTCCAGA	CGTCGCGTTGTAGTTGTACG	59.8	647	96	GDSL-like lipase/acylhydrolase putative expressed
OsISM01647	LOC_Os01g11790	6376515	CCGCATCATCATGGACTTC	TACGAACGGCACATGAAACT	59.6	142	47	GDSL-like lipase/acylhydrolase putative expressed
OsISM01648	LOC_Os01g11790	6376816	AAGCTCAAGCCAACCTTGTG	AAGACGTAGTCGTTGCCACC	60.3	193	99	GDSL-like lipase/acylhydrolase putative expressed
OsISM01649	LOC_Os01g11790	6377060	AGTCGTCCAAGCCATCTCTG	ATTATTCGTACGCGACAGCG	60.8	168	95	GDSL-like lipase/acylhydrolase putative expressed
OsISM01650	LOC_Os01g11790	6377486	GACTTCATCAAGAAACCGGC	GTCGCGTCGTAGTTGTACGG	60.7	3189	96	GDSL-like lipase/acylhydrolase putative expressed
OsISM01651	LOC_Os01g11820	6393585	CAAGAGAAGGTCGTCGTCGT	AGACGTGCCCTTGTCAGACT	60.2	458	79	expressed protein
OsISM01652	LOC_Os01g11840	6400367	GTCACCGGCGTCAAGTTC	CTGATCTTGTTCAGCCCCTC	60.0	162	84	xylem cysteine proteinase 2 precursor putative expressed
OsISM01653	LOC_Os01g11860	6411909	AGGCGTCTCCTCCAACGA	GAGGACGTCGATGGGCAC	62.4	155	84	DJ-1 family protein putative expressed
OsISM01654	LOC_Os01g11860	6412187	CACTTCGACCTCATCGTCCT	CCTCACCATGGCTTCCAG	60.2	175	82	DJ-1 family protein putative expressed
OsISM01655	LOC_Os01g11860	6412374	ACCGGAGGTCTCTACGCC	ACGAACAGCGGATGAGCAG	61.4	1436	99	DJ-1 family protein putative expressed
OsISM01656	LOC_Os01g11860	6413938	AGAACAAGGCCGAGCAGAT	CGTGCCGTTACATTTCCATT	60.4	3836	99	DJ-1 family protein putative expressed
OsISM01657	LOC_Os01g11860	6417752	TCAATGGAAATGTAACGGCA	TATCAGCTCCATCTCCTCGG	60.1	252	71	DJ-1 family protein putative expressed
OsISM01658	LOC_Os01g11860	6418150	CAGCTTCGACCTGATCATCC	AGCTTCTCGTTGCTGCTCAT	60.5	172	67	DJ-1 family protein putative expressed

ISM IDs	Rice gene locus IDs	Physical positions (bp) on chromosomes	Forward primers (5'-3')	Reverse primers (5'-3')	Melting tempearature (T°C)	Expected product size (bp) in PCR	Expected product size (bp) in Q-PCR	Putative Functions
OsISM01660	LOC_Os01g11870	6424106	AGCGGCCTTCGTTTCTCT	TGTGCCTAATTCCATCCTCC	60.0	1343	80	hypothetical protein
OsISM01661	LOC_Os01g11870	6425435	TGGAATTAGGCACAACGTGA	GCGACTTCATCAAAAGCACA	60.1	744	97	hypothetical protein
OsISM01662	LOC_Os01g11870	6426209	TGAAACTCGACGTGGCATAG	GTCATTGCAGCTATGGTGGA	59.8	167	82	hypothetical protein
OsISM01663	LOC_Os01g11870	6426389	CACAATCGTGACTACCAGCG	GTCATGCAGGGTTGGTTCTT	60.1	160	76	hypothetical protein
OsISM01665	LOC_Os01g11880	6431890	CGAGGCCTTTGACCTCATC	CAGCACCTTGCAATCTCTGA	60.4	511	71	DJ-1 family protein putative expressed
OsISM01666	LOC_Os01g11880	6432479	CACTGGGGCTTGCTCAAAG	CACAGGGATAATTTCAGCGG	61.2	189	79	DJ-1 family protein putative expressed
OsISM01667	LOC_Os01g11880	6432767	GAGAAGTCGGAGGAGGTTGC	CCACTGCATTTCCACTCAAC	60.2	609	99	DJ-1 family protein putative expressed
OsISM01668	LOC_Os01g11880	6433356	GTTGAGTGGAAATGCAGTGG	TCCTCCGAACCATTAGCAAC	59.6	322	60	DJ-1 family protein putative expressed
OsISM01669	LOC_Os01g11880	6433810	AGAAGCTGCCAAAAGGGAAT	GAAAGCTTTTGAGCACCTGG	60.1	152	67	DJ-1 family protein putative expressed
OsISM01670	LOC_Os01g11880	6434038	CCTGCTTATGTTCTCGAGCC	GGTGGAAATGATGTTGCCTT	59.9	258	60	DJ-1 family protein putative expressed
OsISM01671	LOC_Os01g11900	6446033	AATATTCCAAGGGCGTGTTG	CCTTTCCAAGGAGAAAAACG	59.3	312	79	hhH-GPD superfamily base excision DNA repair protein expressed
OsISM01672	LOC_Os01g11900	6446332	CTCCTTGGAAAGGATCAGTTG	GCAGCAAGAGCCATAAATGC	59.8	194	98	hhH-GPD superfamily base excision DNA repair protein expressed
OsISM01673	LOC_Os01g11900	6447992	ATAACATGCTGGCAGAACGG	CATAGCGCAACCACTCAAGA	60.5	233	85	hhH-GPD superfamily base excision DNA repair protein expressed
OsISM01674	LOC_Os01g11900	6448215	TTGCGCTATGTCGATTCAGA	GGAGTGTCAAAAGACGCACA	60.2	898	95	hhH-GPD superfamily base excision DNA repair protein expressed
OsISM01675	LOC_Os01g11900	6449093	TGTGCGTCTTTTGACACTCC	CCATCCAAGCCTCACACATA	59.7	170	77	hhH-GPD superfamily base excision DNA repair protein expressed
OsISM01676	LOC_Os01g11900	6449280	ACCCGAGTCTCTTCAGTTGC	GATCAAGCTTGCATAACCTCG	59.7	167	90	hhH-GPD superfamily base excision DNA repair protein expressed
OsISM01677	LOC_Os01g11900	6449433	ATGCAAGCTTGATCAACGGA	TTGTTGTCTCATACCTTTCCAAA A	60.8	162	73	hhH-GPD superfamily base excision DNA repair protein expressed
OsISM01678	LOC_Os01g11900	6449539	TGTAGGTATGAGCTTCACTAT CAAATG	CGTTGCAATTGGGCTTACTT	60.1	157	74	hhH-GPD superfamily base excision DNA repair protein expressed

ISM IDs	Rice gene locus IDs	Physical positions (bp) on chromosomes	Forward primers (5'-3')	Reverse primers (5'-3')	Melting tempearature (T°C)	Expected product size (bp) in PCR	Expected product size (bp) in Q-PCR	Putative Functions
OsISM01679	LOC_Os01g11900	6449676	AAGTAAGCCCAATTGCAACG	TCTTCAGGTCCAGGAAGAGC	59.8	781	97	hhH-GPD superfamily base excision DNA repair protein expressed
OsISM01680	LOC_Os01g11900	6450849	GTCGCCTAAGGACAGAGCAC	AAGTGGATGTGAATCTGGCA	59.6	157	51	hhH-GPD superfamily base excision DNA repair protein expressed
OsISM01681	LOC_Os01g11900	6450986	TGCCAGATTCACATCCACTT	CACTTCTTACCTGGGGTCCA	59.5	183	95	hhH-GPD superfamily base excision DNA repair protein expressed
OsISM01682	LOC_Os01g11900	6451116	CCAGATGATCCTTGCCCATA	GCAGACCGACTTAGGTGCAT	60.6	168	82	hhH-GPD superfamily base excision DNA repair protein expressed
OsISM01683	LOC_Os01g11900	6451358	CCAAAAAGTTCGAGGGACAC	GGAAAGCTTCCTCTCATTGC	59.3	287	58	hhH-GPD superfamily base excision DNA repair protein expressed
OsISM01684	LOC_Os01g11900	6451618	CCGAACAGCAATGAGAGGA	GAACATCAATCGGGTTCCG	60.6	219	99	hhH-GPD superfamily base excision DNA repair protein expressed
OsISM01685	LOC_Os01g11900	6451880	TGGAACTTCAATTCCGACAAT	CCTCTCCAAAAGCAATGTTGT	59.3	1421	67	hhH-GPD superfamily base excision DNA repair protein expressed
OsISM01686	LOC_Os01g11900	6453243	CCCATGTTTCTATCGCAGGT	CCTATCAAAGCCTCTCACGC	60.0	1111	84	hhH-GPD superfamily base excision DNA repair protein expressed
OsISM01687	LOC_Os01g11910	6462972	GAGAGGATCAACGCCCAC	CACCACCTCAGCCAGCAG	60.4	268	88	basic helix-loop-helix putative expressed
OsISM01688	LOC_Os01g11920	6471817	GATTGATCCCTCGTACGGC	GAGCATCCTCGATGTGTCCT	60.3	696	75	retrotransposon protein putative unclassified expressed
OsISM01689	LOC_Os01g11920	6472636	TGAGGGAAAGTGACGCTTCT	CAAGCTGATTGAGCCGTAGA	59.6	182	94	retrotransposon protein putative unclassified expressed
OsISM01690	LOC_Os01g11920	6472897	GTTCCTTGCCAATGCATACC	ACAGAAGAGACAGCATGGTCA	59.4	333	82	retrotransposon protein putative unclassified expressed
OsISM01691	LOC_Os01g11920	6473489	CAACCCAACTATCCAAAGGC	TCTTTCACAGATTCTGCCCA	59.4	169	78	retrotransposon protein putative unclassified expressed
OsISM01692	LOC_Os01g11920	6473800	ATGGAACAATTCCAGATGGC	CAAATGGGGAAATTTTGCAG	60.0	1190	95	retrotransposon protein putative unclassified expressed
OsISM01693	LOC_Os01g11920	6474981	CCCCATTTGTGAATACCCTG	GCAGGGCTCTCGCTTATATC	59.5	159	74	retrotransposon protein putative unclassified expressed
OsISM01694	LOC_Os01g11920	6475159	GTGAGGACGGTTCCAACATT	TCAGGATAAACTTCACCGGC	60.0	206	99	retrotransposon protein putative unclassified expressed
OsISM01695	LOC_Os01g11920	6475288	GATGTTTCCTCAGGCCTCC	CAGCAACATTTGTGTCCTGC	60.0	840	95	retrotransposon protein putative unclassified expressed
OsISM01696	LOC_Os01g11920	6476090	AGTGACTGAACACAGCACGC	TGGCTTTGTTTGCTTTGATG	60.0	1517	98	retrotransposon protein putative unclassified expressed

ISM IDs	Rice gene locus IDs	Physical positions (bp) on chromosomes	Forward primers (5'-3')	Reverse primers (5'-3')	Melting tempearature (T˚C)	Expected product size (bp) in PCR	Expected product size (bp) in Q-PCR	Putative Functions
OsISM01697	LOC_Os01g11920	6477655	CCAAAATGTGGTGTGGTTGA	CGTAAGATCCCGAGACGAAA	60.2	761	97	retrotransposon protein putative unclassified expressed
OsISM01698	LOC_Os01g11920	6478422	GGACAGGTGATTGGTTCTCG	TACAGGGTGCAGATCCTTGC	60.9	236	98	retrotransposon protein putative unclassified expressed
OsISM01699	LOC_Os01g11920	6479663	CCTTAAAACCGTTTGAGCGA	ATGGCTTCATCCCACTGTTC	60.1	145	59	retrotransposon protein putative unclassified expressed
OsISM01700	LOC_Os01g11920	6479887	CTGCAAGCCAATCTGATGAA	GTTCTCTTCCTATTCCCGCC	60.0	230	96	retrotransposon protein putative unclassified expressed
OsISM01701	LOC_Os01g11920	6480264	TTCGGAGGTGTCTAATTCGG	GGAAAACCACCTGGGAATTT	60.0	184	93	retrotransposon protein putative unclassified expressed
OsISM01702	LOC_Os01g11920	6481355	AGAAGAGATGCTTTGGCTGC	ATTCATCTCCGGGAAACCTT	59.7	270	71	retrotransposon protein putative unclassified expressed
OsISM01703	LOC_Os01g11920	6482748	TGATCCAAGCTATTCCGGTT	TTCTGAACGTCCCAAGATCC	59.8	535	74	retrotransposon protein putative unclassified expressed
OsISM01704	LOC_Os01g11940	6494077	GACATGCGCACCTTCTACAC	TGCAGGTACTCCCTGAGGTT	59.5	177	81	osFTL1 FT-Like1 homologous to Flowering Locus T gene; contains Pfam profile PF01161: Phosphatidylethanolamine-binding protein expressed
OsISM01705	LOC_Os01g11940	6494230	TCCAAACCTCAGGGAGTACC	AGAGACTCCTGTGGTAGCCG	59.2	3854	62	osFTL1 FT-Like1 homologous to Flowering Locus T gene; contains Pfam profile PF01161: Phosphatidylethanolamine-binding protein expressed
OsISM01706	LOC_Os01g11940	6498035	CTATCAATGCAGGCTGGTCA	GCTCTCGTAGCACACCACCT	60.1	209	77	osFTL1 FT-Like1 homologous to Flowering Locus T gene; contains Pfam profile PF01161: Phosphatidylethanolamine-binding protein expressed
OsISM01707	LOC_Os01g11980	6524623	GCATATGCTCCCTATCCCCT	CCAGGATCATCAGAGCCAGT	60.2	180	79	SFT2 putative expressed
OsISM01708	LOC_Os01g11980	6524738	GCTTCTCCAGCGGGTCTAC	TGCAAATTTGATGGGTCTGA	60.0	826	98	SFT2 putative expressed
OsISM01709	LOC_Os01g11980	6525538	TTTTCGTCAGACCCATCAAA	CAACTGTTGACTTGGCCCTA	58.9	291	96	SFT2 putative expressed
OsISM01710	LOC_Os01g11980	6525873	CGGTGGATTTGTTTTTCTGG	AACAGAGCACAGATCTCGCA	60.0	191	94	SFT2 putative expressed
OsISM01711	LOC_Os01g11980	6526002	GTGCAGATCCATTCCAAGGT	GCGAAAGGGATGTAGCTCAA	60.1	961	90	SFT2 putative expressed
OsISM01713	LOC_Os01g11990	6529469	CTCCTGGCGCCTCCTCTAC	TACCAATGTGCCTGCTGCT	61.4	167	78	expressed protein
OsISM01714	LOC_Os01g11990	6529568	CCCCTCTCTTCTTCCTCCAG	GAGCAGGAACCCACCAATC	60.4	183	92	expressed protein
OsISM01716	LOC_Os01g11990	6530028	ATTGCACCCTCAACAAGTGC	CCATTCCAGAATCCCTGCT	60.6	178	86	expressed protein

ISM IDs	Rice gene locus IDs	Physical positions (bp) on chromosomes	Forward primers (5'-3')	Reverse primers (5'-3')	Melting tempearature (T˚C)	Expected product size (bp) in PCR	Expected product size (bp) in Q-PCR	Putative Functions
OsISM01717	LOC_Os01g11990	6530236	CTACAACGACGAGGAGACCC	CCCAAGACTTCGAGTGGAGA	60.0	223	99	expressed protein
OsISM01718	LOC_Os01g11990	6530521	TCCAACCTCCTGTCCAACTC	AGACACTATCTGCCCGTTCC	59.6	176	85	expressed protein
OsISM01719	LOC_Os01g12020	6540351	CTCTCCATCAGCTTCACCATC	GAACTCGATTCCTCACGCTC	59.9	192	87	LTPL18 - Protease inhibitor/seed storage/LTP family protein precursor expressed
OsISM01720	LOC_Os01g12040	6547599	TGACAACCAATTTGGCAGAG	AGATCCTGAGATCCCGAACC	60.1	509	96	transposon protein putative unclassified
OsISM01721	LOC_Os01g12040	6548287	GAATGCAGAAGTTCCAAGCG	CAATCTCACGGTTCAACAGC	60.1	537	96	transposon protein putative unclassified
OsISM01723	LOC_Os01g12040	6549346	CTAGCAAGGCTTGGTTGCTC	AACCACAACAGGTACGCCTC	60.1	184	98	transposon protein putative unclassified
OsISM01724	LOC_Os01g12040	6549840	ACGATGGGGACTTACTGGTG	GGAGACGGTCAAACTCGAAC	59.8	188	95	transposon protein putative unclassified
OsISM01725	LOC_Os01g12040	6550200	CGGACTTAGGCAGGTGTTTC	CTAGCTGTCCCCGTCGAGT	60.1	159	81	transposon protein putative unclassified
OsISM01726	LOC_Os01g12040	6550567	GCACCGTGACCGAGACTACT	ATATCGGCACTGATATCCCG	60.0	152	58	transposon protein putative unclassified
OsISM01728	LOC_Os01g12040	6551275	AGCTCTTCCTCTCACGGAGC	AGACGAAGAGGCCCCAGTA	60.5	144	82	transposon protein putative unclassified
OsISM01729	LOC_Os01g12040	6551605	TACTCCCAGGGGCACATTAG	CCGAAAGTGTGTTGTCGTGA	60.4	234	97	transposon protein putative unclassified
OsISM01730	LOC_Os01g12040	6551819	TCACGACAACACACTTTCGG	GGGTTCTCCATGTCTACCCA	60.3	586	77	transposon protein putative unclassified
OsISM01731	LOC_Os01g12040	6552450	GGACTACCTAAGGCGGAAGG	TCCATCGAGTAAATCCACCC	59.9	693	96	transposon protein putative unclassified
OsISM01732	LOC_Os01g12090	6582967	GCTACGGCATCAAGAAGCTC	GAGCCACCCAACCAGAATAA	60.0	430	67	ribosomal protein S6 family protein putative
OsISM01733	LOC_Os01g12090	6583429	TTTGCTTTGAATAATATAACCCTTGA	CAAGTGCCGTGTCACAAAGT	59.6	205	97	ribosomal protein S6 family protein putative
OsISM01734	LOC_Os01g12090	6583620	TGACACGGCACTTGAATGTT	GTTCTTGGGTGAAGGAGGGT	60.3	2064	82	ribosomal protein S6 family protein putative
OsISM01735	LOC_Os01g12200	6652540	ATGTCTTCTCCAAGGAGCCC	TTATCAATGCCCTCGAATTT	58.9	1177	86	expressed protein
OsISM01736	LOC_Os01g12210	6658490	GGTCCATCCTCACTGTTGCT	CAAATACACTTCCGACGGCT	60.1	241	88	aluminum-activated malate transporter putative expressed

ISM IDs	Rice gene locus IDs	Physical positions (bp) on chromosomes	Forward primers (5'-3')	Reverse primers (5'-3')	Melting tempearature (T˚C)	Expected product size (bp) in PCR	Expected product size (bp) in Q-PCR	Putative Functions
OsISM01737	LOC_Os01g12210	6658772	AGTGGCCATATTGCAGAACC	AGTGATGGCCATAGCTTCAT	58.6	234	87	aluminum-activated malate transporter putative expressed
OsISM01738	LOC_Os01g12210	6659207	CAGCTTCAACTCTCTCGCAG	TGTTCGGAACCATCATCACT	59.0	157	70	aluminum-activated malate transporter putative expressed
OsISM01739	LOC_Os01g12210	6659412	ATTCAGGAAGTGCCGAGCTA	GCTTGAATCTCCCATGTGGT	60.0	194	93	aluminum-activated malate transporter putative expressed
OsISM01740	LOC_Os01g12210	6659663	CCTACGAGGTGATGGCTTTG	AGATCTCGGACTTGAAGGCA	60.3	1116	85	aluminum-activated malate transporter putative expressed
OsISM01742	LOC_Os01g12220	6662962	GGGATCTCGGTGACATCAGT	ACCGCGATGAAAAATGTTCT	59.8	212	89	uncharacterized protein family UPF0016 domain containing protein expressed
OsISM01743	LOC_Os01g12220	6663139	TCTGAGCTAGGGGACAGAACA	TGTGCCAAGAAAAATGATCG	59.8	632	79	uncharacterized protein family UPF0016 domain containing protein expressed
OsISM01744	LOC_Os01g12220	6663759	TTTCTTGGCACATTTGGAGC	TGAAATGCTCGTCCAAGAACT	60.5	223	66	uncharacterized protein family UPF0016 domain containing protein expressed
OsISM01745	LOC_Os01g12220	6663963	TTCTTGGACGAGCATTTCACT	ACCAAAAGACAGGCAGCAAG	60.1	173	98	uncharacterized protein family UPF0016 domain containing protein expressed
OsISM01746	LOC_Os01g12220	6664087	GTGGCACAGATTTCCCAGTT	CTGAAGCTGCATCAAGCAAT	59.6	240	85	uncharacterized protein family UPF0016 domain containing protein expressed
OsISM01747	LOC_Os01g12240	6688740	TCTCCTCAACCGTTCTAGCC	TTTCCTGTGAGGACATTAGGA	58.3	1115	92	hypothetical protein
OsISM01748	LOC_Os01g12240	6689831	AAATCCTAATGTCCTCACAGGAA	GAGGTATCGTTCCATCCGAA	59.5	1111	85	hypothetical protein
OsISM01749	LOC_Os01g12240	6690920	CATTCGGATGGAACGATACC	TCGAATCCTCCTGTTCAAGC	60.2	743	80	hypothetical protein
OsISM01750	LOC_Os01g12240	6691606	GGGATTTGATTTCTCCAGCTC	CTCCCATGCAACCTTCATTT	60.0	853	71	hypothetical protein
OsISM01751	LOC_Os01g12240	6692439	AAATGAAGGTTGCATGGGAG	TTTGAGTACCAGAGTGAAATTCTGA	59.6	282	99	hypothetical protein
OsISM01752	LOC_Os01g12240	6692643	TTAGGAAAAGAGATCCCATATTACTCT	GGAGTGGCTCATTCACGATA	58.5	207	98	hypothetical protein
OsISM01753	LOC_Os01g12350	6747699	CCCTCCAAAGTGGTCGTTTA	GAATCCTCGGCCTCAGACT	59.7	187	94	retrotransposon protein putative unclassified
OsISM01754	LOC_Os01g12350	6748383	CGGTGTTGGATGGGTTAAGT	GTCGCCCCTCTTCGACAC	60.8	93	46	retrotransposon protein putative unclassified
OsISM01755	LOC_Os01g12464	6832360	AGCTACGACCGCGACAAGT	AGCCAGGGAAAATTTCTTCA	59.9	1582	85	CHCH domain containing protein expressed

ISM IDs	Rice gene locus IDs	Physical positions (bp) on chromosomes	Forward primers (5'-3')	Reverse primers (5'-3')	Melting tempearature (T°C)	Expected product size (bp) in PCR	Expected product size (bp) in Q-PCR	Putative Functions
OsISM01756	LOC_Os01g12470	6836174	GTCCTCGTCAACCCGCTC	GTGGAAGAGAAATGGCAAGC	60.8	298	94	mitochondrial carrier protein putative expressed
OsISM01757	LOC_Os01g12470	6836399	CCTGCAGACAAGGTTACAAGC	AACCATTCATTCCAGGGTTG	59.8	303	99	mitochondrial carrier protein putative expressed
OsISM01758	LOC_Os01g12470	6836736	CAGAGGGACAATGGATGTGTT	GGCTTGCAGCAGTACCTCTC	60.0	163	78	mitochondrial carrier protein putative expressed
OsISM01759	LOC_Os01g12470	6836879	GAGAGGTACTGCTGCAAGCC	GCAATAGATCGTATGAGGGCA	60.1	884	72	mitochondrial carrier protein putative expressed
OsISM01760	LOC_Os01g12470	6837868	CCCCTATTGAGTTGGCAAGA	GGGGTTTTCCTCCAACATTT	60.0	474	64	mitochondrial carrier protein putative expressed
OsISM01761	LOC_Os01g12470	6838370	GCTTGCATTGAGACAGAGCA	TGCAAGTTGTGCTCCAAGAC	60.2	296	83	mitochondrial carrier protein putative expressed
OsISM01762	LOC_Os01g12470	6838668	TGACGTCCCCTTCTCAGCTA	GCTTTGCTCGCCAACTATTC	60.5	196	80	mitochondrial carrier protein putative expressed
OsISM01763	LOC_Os01g12470	6838940	CTCTCGATGTTGCCAAGACA	TATGCGCCTTGTGTTCATGT	60.1	283	75	mitochondrial carrier protein putative expressed
OsISM01764	LOC_Os01g12470	6839170	CCTCATTTCAGAAGGACCCA	ACCTCTGAATATCCCGCTGA	59.8	604	99	mitochondrial carrier protein putative expressed
OsISM01765	LOC_Os01g12490	6857137	AGGTGTCCCTCGACCTCTG	AGTGGAGAACCCCAGGATTT	60.0	1572	96	flavin monooxygenase putative expressed
OsISM01766	LOC_Os01g12490	6858888	CCTAGCCAAGATCAAGTCCG	CAAATTCTTCAGTGCTGCCA	59.9	207	99	flavin monooxygenase putative expressed
OsISM01767	LOC_Os01g12490	6859091	TTTGATGTTGTCATCCTGGC	TGGGAAGCCATCTTTCTCTG	59.9	233	91	flavin monooxygenase putative expressed
OsISM01768	LOC_Os01g12500	6862367	CAGATCTGGCCGGAGGAG	GAGGACGACGAGGATGCC	61.9	141	96	hypothetical protein
OsISM01769	LOC_Os01g12530	6889169	GTGCTCTATGCTGGGGGATA	ACCTTCTCAACATCGCATCC	60.1	1357	85	smr domain containing protein expressed
OsISM01770	LOC_Os01g12530	6890493	TTCAGGTCAATATGGATGCG	TTGAAGGGAAGACATACCCG	59.7	1231	74	smr domain containing protein expressed
OsISM01771	LOC_Os01g12530	6891812	CAACTGCAGGCATTGAAAAA	CCAAAAGAATCCGTGCTCAT	60.0	666	93	smr domain containing protein expressed
OsISM01772	LOC_Os01g12530	6892458	ATGAGCACGGATTCTTTTGG	CTCCTCACATGCAACCTTCA	60.0	568	60	smr domain containing protein expressed
OsISM01773	LOC_Os01g12530	6893182	TCTCCCATCACATCGAACAA	ATAATGCCTTCCAGCGACAG	60.1	508	82	smr domain containing protein expressed

ISM IDs	Rice gene locus IDs	Physical positions (bp) on chromosomes	Forward primers (5'-3')	Reverse primers (5'-3')	Melting tempearature (T°C)	Expected product size (bp) in PCR	Expected product size (bp) in Q-PCR	Putative Functions
OsISM01774	LOC_Os01g12530	6893670	CTGTCGCTGGAAGGCATTAT	CTCTGCGAGATAGGAGGCAT	59.9	192	99	smr domain containing protein expressed
OsISM01775	LOC_Os01g12530	6893812	CCATGGCATATTCCAAAGGA	TGCGAGCTAATTCACGGTAA	60.1	319	79	smr domain containing protein expressed
OsISM01776	LOC_Os01g12530	6894122	TAGCTCGCATGGAAGATGAG	CCCATGCAGGTCAATAGTCA	59.3	159	90	smr domain containing protein expressed
OsISM01777	LOC_Os01g12530	6894297	CCATGAGGCTTCTCAAGGTT	TTCCCTGTGCCTTCTACACC	59.7	227	95	smr domain containing protein expressed
OsISM01778	LOC_Os01g12530	6894504	GGTGTAGAAGGCACAGGGAA	CACTCAACGCCTTCCTTCTC	60.1	416	72	smr domain containing protein expressed
OsISM01779	LOC_Os01g12540	6898067	GGGATGGCAGAGCTTTATGA	GGGTCATCTTGGTTGAGTCC	59.8	551	87	GTPase of unknown function domain containing protein putative expressed
OsISM01780	LOC_Os01g12540	6898762	GGCAGGCTTAACAAGGGACT	TCCTGACCTTTCCATCCAAC	60.3	393	95	GTPase of unknown function domain containing protein putative expressed
OsISM01781	LOC_Os01g12540	6899181	CGTGGTGCAGTCAAGAAAGA	CAGGGTGATTCATGCTACTTTT	59.1	1048	99	GTPase of unknown function domain containing protein putative expressed
OsISM01782	LOC_Os01g12540	6900301	TTAGGGAAAACCGACCATTG	TGGTATTCCTGTAACCTGCCA	60.1	793	87	GTPase of unknown function domain containing protein putative expressed
OsISM01783	LOC_Os01g12540	6901176	TAAGGTTGTCGACCTCACGG	CTTTTGGCTGGGTAGCTGAG	60.5	1279	85	GTPase of unknown function domain containing protein putative expressed
OsISM01784	LOC_Os01g12550	6903907	GAGCTCCCCCAATTCCTC	TCACTCTTCTTCAATGGCGA	59.5	1072	72	expressed protein
OsISM01785	LOC_Os01g12550	6904959	TCGCCATTGAAGAAGAGTGA	ACCACTTGGCACATTTAGGC	59.8	677	97	expressed protein
OsISM01786	LOC_Os01g12550	6905656	TGATGTGGCATACGAGCATT	CAGCACAAACCTGCTTTTCA	60.1	281	75	expressed protein
OsISM01787	LOC_Os01g12550	6905901	TCCAGTTTGTTCCAAGTGAAA A	ACGGGGACACCATCAAGTT	59.9	174	98	expressed protein
OsISM01788	LOC_Os01g12550	6906103	GTGGCAACGAATGATGGAAT	GAAAATGTTGATCCATTGAAGC	59.6	498	95	expressed protein
OsISM01789	LOC_Os01g12550	6906684	GCAGACAGTTTCCTTGAGCC	TAGCGGTATCCCAACTGGAC	60.0	162	70	expressed protein
OsISM01790	LOC_Os01g12550	6906927	GCTACTGGCATACAGCCACA	GCAGTAGCAGCCCTGTCAAT	60.2	575	84	expressed protein
OsISM01791	LOC_Os01g12550	6907689	GCACAAGGAAGAGACAGAGC A	GATTTTGGGGAGAAGTGGGT	60.4	316	82	expressed protein

ISM IDs	Rice gene locus IDs	Physical positions (bp) on chromosomes	Forward primers (5'-3')	Reverse primers (5'-3')	Melting tempearature (T˚C)	Expected product size (bp) in PCR	Expected product size (bp) in Q-PCR	Putative Functions
OsISM01792	LOC_Os01g12590	6929333	TAGTCACATGCGACGAGGTC	AAGACGTCAGCGTTTGCC	59.9	137	92	retrotransposon protein putative unclassified
OsISM01793	LOC_Os01g12650	6966951	CATCCACAAGGTCCTCGGT	AGTGGCACCGCCTAGTACAC	60.6	1041	89	reticulon domain containing protein putative expressed
OsISM01794	LOC_Os01g12650	6968044	GCCATGGGCTCATATTCTCT	TGGAATAATCACCTCTGGGA	58.5	214	99	reticulon domain containing protein putative expressed
OsISM01795	LOC_Os01g12650	6968310	CCTTTGCCAATCTTCGTCAG	AACAGAAAGCAACCAAAGGC	60.1	168	84	reticulon domain containing protein putative expressed
OsISM01796	LOC_Os01g12650	6968458	GCCTTTGGTTGCTTTCTGTT	AGAATTGGGACTGTGTGCAG	59.0	190	89	reticulon domain containing protein putative expressed
OsISM01797	LOC_Os01g12670	6979027	GGAAACCATCGAATTAGCCA	GCATCGACCGAGTTTCTAGC	59.9	204	73	expressed protein
OsISM01798	LOC_Os01g12670	6979534	GCATGTTGCAGAGCTTTTCC	CTAGCCCTCCATTCATCAGC	60.4	170	76	expressed protein
OsISM01799	LOC_Os01g12670	6979683	AGCTGATGAATGGAGGGCTA	TTTCTTGGCAATCTGCTTCA	59.7	185	80	expressed protein
OsISM01800	LOC_Os01g12670	6979871	GCAAATGAGGAAAGAAAGCG	GTTTCTCGACCATTAGGGCA	60.0	560	59	expressed protein
OsISM01801	LOC_Os01g12670	6980432	ACAGGAGCTTCGCTCTATGA	AACTGCAGCCTTAACACGCT	58.9	957	98	expressed protein
OsISM01802	LOC_Os01g12670	6981689	AGCAGCTACGACATGGGAAC	AAGGAATCCCACATCCTGC	60.1	448	51	expressed protein
OsISM01803	LOC_Os01g12670	6982095	ATTCCACCTTTGATTGCAGG	GAACCCAGTGCCCAGGTATT	60.5	589	81	expressed protein
OsISM01805	LOC_Os01g12680	6988848	TGCCAAGATTAAAACTTAGGTGC	TGGCACTACCATTACAGGCA	59.9	187	78	C4-dicarboxylate transporter/malic acid transport protein expressed
OsISM01808	LOC_Os01g12700	7015671	TTGTGCAGAAGAACACTGGC	ATTAATATCCCCCGCACCTC	60.0	322	88	MYB family transcription factor putative
OsISM01809	LOC_Os01g12700	7015915	TGTTGTTCTTTAATAGTTTTTGCATC	AAGCCAACGATTGTTCTTGC	59.2	725	99	MYB family transcription factor putative
OsISM01810	LOC_Os01g12700	7016849	CCGAATAGAAGTTGCAGCAG	GCCATGGTTGGCAAAGATA	59.1	207	98	MYB family transcription factor putative
OsISM01811	LOC_Os01g12710	7023673	CGAGAAGGCCGAGGAGAT	TATCGGCCTACTTGCGTAGC	60.4	977	49	oxidoreductase short chain dehydrogenase/reductase family domain containing protein expressed
OsISM01812	LOC_Os01g12710	7024664	TGAGCAGCGGTGTTGTACTT	CCCGAGCCAACTATACCAAA	59.7	199	87	oxidoreductase short chain dehydrogenase/reductase family domain containing protein expressed

ISM IDs	Rice gene locus IDs	Physical positions (bp) on chromosomes	Forward primers (5'-3')	Reverse primers (5'-3')	Melting tempearature (T°C)	Expected product size (bp) in PCR	Expected product size (bp) in Q-PCR	Putative Functions
OsISM01813	LOC_Os01g12710	7024909	AACACTGCAAAGCTGGACCT	GAAAGAAGGAACTCCCGAGC	60.1	460	86	oxidoreductase short chain dehydrogenase/reductase family domain containing protein expressed
OsISM01814	LOC_Os01g12710	7025349	GCTCGGGAGTTCCTTCTTTC	TCAAGCTCGTTGATGGTCTG	60.2	200	84	oxidoreductase short chain dehydrogenase/reductase family domain containing protein expressed
OsISM01815	LOC_Os01g12710	7025669	GAACTTTGCCAAGGATGAGC	CTTTGTTTGTGCCAGCGTTA	59.9	251	63	oxidoreductase short chain dehydrogenase/reductase family domain containing protein expressed
OsISM01816	LOC_Os01g12710	7025915	CAAAGGTTTTAGGCCACTGGT	TGGTGCACAGTAAAGAGCCA	60.4	240	87	oxidoreductase short chain dehydrogenase/reductase family domain containing protein expressed
OsISM01817	LOC_Os01g12710	7026216	AGCAGGTTCTGGAGGATCAA	GCTTGAAACTGTCTAAGGCCA	59.7	248	76	oxidoreductase short chain dehydrogenase/reductase family domain containing protein expressed
OsISM01818	LOC_Os01g12710	7026514	CTCCTGGAATGGTCCTTACG	CCTTGCAACTGTTTCTGGAAG	59.7	943	99	oxidoreductase short chain dehydrogenase/reductase family domain containing protein expressed
OsISM01819	LOC_Os01g12710	7027536	CCTTGGTTACTGCATGGGTT	CGGATTCTATCAGCCTCTGC	59.9	1374	83	oxidoreductase short chain dehydrogenase/reductase family domain containing protein expressed
OsISM01820	LOC_Os01g12720	7037736	ACCTCCTGCACTACTACGGC	AATTTTGGTACACCCCACCA	59.7	741	88	protein kinase domain containing protein expressed
OsISM01821	LOC_Os01g12720	7038931	TCCAAACAACACATTGGAGC	ACCCAAAGCAATGCGTAATC	59.8	629	83	protein kinase domain containing protein expressed
OsISM01822	LOC_Os01g12720	7039564	CGAAGGGATTGGCATATCTT	TCAAATCTCGCGTCAAGAAG	59.1	198	92	protein kinase domain containing protein expressed
OsISM01823	LOC_Os01g12720	7039743	TTCTTGACGCGAGATTTGAG	TGTGCCCATTACTCTGGTTG	59.4	477	96	protein kinase domain containing protein expressed
OsISM01824	LOC_Os01g12720	7040156	TTTCGGACTTGCAAAATTGA	AGACGCATACTCTGGTGCAA	59.4	249	92	protein kinase domain containing protein expressed
OsISM01825	LOC_Os01g12720	7040489	TCGAACCAATCACAGATGGA	ATGCTCGCATCATTAAAGGC	60.1	509	62	protein kinase domain containing protein expressed
OsISM01826	LOC_Os01g12720	7041092	TTGTGTACGCCATTCTGCAC	ATCCAATGACACATCGCCTT	60.5	318	77	protein kinase domain containing protein expressed
OsISM01827	LOC_Os01g12730	7043158	AGGTCATCATCCTCGGTGAC	GAATCGGACAACGCAAGATT	60.0	216	88	ras-related protein putative expressed
OsISM01828	LOC_Os01g12730	7043310	GTCGGGAAGACGTCTCTGAT	CGATCGTCGCTTTGTACTGA	59.6	195	68	ras-related protein putative expressed
OsISM01829	LOC_Os01g12730	7043516	CTGACCAAGGAGGTGCAGAT	CTCTGAAATCGCTCCTGTCC	60.1	1629	78	ras-related protein putative expressed
OsISM01830	LOC_Os01g12730	7045207	CCAAGTCATTTGAAAGGCTCA	TTCTCTGGATCTGATGGGCT	60.0	158	71	ras-related protein putative expressed

**ISM IDs**	**Rice gene locus IDs**	**Physical positions (bp) on chromosomes**	**Forward primers (5'-3')**	**Reverse primers (5'-3')**	**Melting tempearature (T˚C)**	**Expected product size (bp) in PCR**	**Expected product size (bp) in Q-PCR**	**Putative Functions**
OsISM01831	LOC_Os01g12730	7045398	TGTTGATGGTGGTAATAGCCG	CCCTTAGAAGCACACCAAGC	60.3	152	64	ras-related protein putative expressed
OsISM01832	LOC_Os01g12730	7045631	CAAGAATGAACCGGAAGAGG	CTAGCATTCACAGCCTGACG	59.6	469	92	ras-related protein putative expressed
OsISM01833	LOC_Os01g12740	7048231	GAGAAGTGCTGCTGTTCGAC	AAGGTCGTGAAGGTGGTGTC	59.4	688	86	cytochrome P450 putative expressed
OsISM01834	LOC_Os01g12750	7053081	GACACTGTCGCCATCAAGG	TGGTGAAGCTGGAGTCTGTG	60.1	1453	65	cytochrome P450 putative expressed
OsISM01835	LOC_Os01g12760	7061337	GACATGGACGCTGGTGTTC	ATCACTGTCGTCGTGGTGTC	59.8	2266	93	cytochrome P450 putative expressed
OsISM01836	LOC_Os01g12770	7071497	CCGTCGAAATCAAGGCTATT	CCATCATCGTGGTCGTAGTG	59.6	528	67	cytochrome P450 putative expressed
OsISM01837	LOC_Os01g12790	7079037	TGACACCTCCAAGATCACCA	CGAAGCTTCAGTTTACCCCT	59.3	1255	64	receptor protein kinase putative
OsISM01838	LOC_Os01g12790	7080264	AACAGAATAGGGGTAAACTG AAGC	CAAACGATCGATCCGTAGGT	59.6	252	72	receptor protein kinase putative
OsISM01839	LOC_Os01g12790	7080625	TGAATCACCTTCTTTTGCCA	GCCAACCAATGATTGCCTAC	59.8	265	51	receptor protein kinase putative
OsISM01840	LOC_Os01g12790	7083866	GGAACATTTGGGTACATGGC	CATGATTCCGAAGCTGAACA	59.9	446	82	receptor protein kinase putative
OsISM01841	LOC_Os01g12810	7092008	TGTCAAGACCAATGGCAAGA	CATTGCTGCCAGCTTCGT	60.7	192	84	retrotransposon protein putative unclassified expressed
OsISM01842	LOC_Os01g12810	7093069	ATCGTCTTCAACACCCTGCT	GATCCCGAACTGCTCCATC	60.2	171	91	retrotransposon protein putative unclassified expressed
OsISM01843	LOC_Os01g12860	7116192	CCTCTCAATGGGTAAAAGGC	TTTTCCAGTTCTTCCCGTTG	59.4	808	90	MYB family transcription factor putative expressed
OsISM01844	LOC_Os01g12860	7116980	CAACGGGAAGAACTGGAAAA	GTGCAAGCATTGTACATCGG	60.1	698	65	MYB family transcription factor putative expressed
OsISM01845	LOC_Os01g12860	7117675	CACAGGTGGCAAAAGGTTCT	TTTGGTCCAAGTTTGTTTACCA	59.8	192	99	MYB family transcription factor putative expressed
OsISM01846	LOC_Os01g12860	7117886	CAAGCTTTGCCTGGACGTAT	GTGTCCATGCCTCCTTGTTT	60.1	471	86	MYB family transcription factor putative expressed
OsISM01847	LOC_Os01g12860	7118394	GTATGGAAATAAATGGGCCG	CCAGTGATTTTTAATTGAATTGT CC	59.6	479	71	MYB family transcription factor putative expressed
OsISM01848	LOC_Os01g12860	7120580	GAACGCAGTTCTGCAACAAA	GCCGCTGTGTTCAATAAGAA	59.5	302	85	MYB family transcription factor putative expressed

ISM IDs	Rice gene locus IDs	Physical positions (bp) on chromosomes	Forward primers (5'-3')	Reverse primers (5'-3')	Melting tempearature (T˚C)	Expected product size (bp) in PCR	Expected product size (bp) in Q-PCR	Putative Functions
OsISM01849	LOC_Os01g12860	7120986	AAAGCCCACTGAACTCATCG	TCCTCTTTTGATACCTGGGG	59.6	910	98	MYB family transcription factor putative expressed
OsISM01850	LOC_Os01g12860	7121876	CCCCAGGTATCAAAAGAGGA	AGCTGGGCTGGTGAAGTATG	59.6	331	96	MYB family transcription factor putative expressed
OsISM01851	LOC_Os01g12860	7122363	GGGACAACCAAATTGCAAAC	CTGCCAGCATTTTTGTCTGA	60.1	337	96	MYB family transcription factor putative expressed
OsISM01852	LOC_Os01g12880	7128466	GAGATCTGCGACATCTGCAA	GTGCCCAATCCTTTTCTTGA	60.1	1524	76	VHS and GAT domain containing protein expressed
OsISM01853	LOC_Os01g12880	7129970	TCAAGAAAAGGATTGGGCAC	CCCACAGTTCTTGATTGCAG	59.7	530	81	VHS and GAT domain containing protein expressed
OsISM01854	LOC_Os01g12880	7130532	TGTGTTGCATGAGATGGTGA	CGAATGCTTCTTGCCATGTA	59.7	183	96	VHS and GAT domain containing protein expressed
OsISM01855	LOC_Os01g12880	7130718	GACCACGTGCAAGATATCCA	AAACAATGGTGCAGGTCGAT	60.0	293	99	VHS and GAT domain containing protein expressed
OsISM01856	LOC_Os01g12880	7131067	TGAATCGTCTGCTGGAAATG	ATCTCGGCTAGCACATCCAT	59.7	700	85	VHS and GAT domain containing protein expressed
OsISM01857	LOC_Os01g12880	7131752	TGTGCTAGCCGAGATGCTAA	GCTCCACAATTACCTCCTGC	59.7	701	72	VHS and GAT domain containing protein expressed
OsISM01858	LOC_Os01g12880	7132450	AGCTTGTTGATCAGTGTCGC	GTGCAAGCCCTTGTGACATA	59.4	461	91	VHS and GAT domain containing protein expressed
OsISM01859	LOC_Os01g12880	7133068	TGAGGATCCTACAACCCAAGA	GCGCTAGTTGCTCAAATGGT	60.0	1651	78	VHS and GAT domain containing protein expressed
OsISM01860	LOC_Os01g12880	7135110	TGGTCAAATTGCGAACAATG	ACTGAGCTTCCCAAGGAGGT	60.4	244	96	VHS and GAT domain containing protein expressed
OsISM01861	LOC_Os01g12890	7158326	CACAACACCAATGCGAGC	CTGGTCATGGAAAATCCGAG	60.1	1180	99	expressed protein
OsISM01862	LOC_Os01g12890	7159831	GAGCTGCTGAGGCCAATACT	TCAGAGACATTTGCTGCCAC	59.8	171	83	expressed protein
OsISM01863	LOC_Os01g12890	7161019	CCAGATGGAAACCGAAAACT	TCTGCTCATGAAGGTCAGACA	59.3	405	93	expressed protein
OsISM01865	LOC_Os01g12910	7173118	AAGGTCTCCGTCCACTACGA	CATCTTCCCTTTCTGCTCCA	60.0	1355	97	thioesterase family protein putative expressed
OsISM01867	LOC_Os01g12920	7175532	GTGCACAGGTTCACCATCAG	CTCCCATCCATCTCCACCT	60.0	187	78	thioesterase family protein putative expressed
OsISM01869	LOC_Os01g12950	7198109	TGCTCGAGCTATTTTGTGTGA	AAGGAGGAACAAGTTGCATGA	59.7	711	99	ubiquitin-conjugating enzyme putative expressed

ISM IDs	Rice gene locus IDs	Physical positions (bp) on chromosomes	Forward primers (5'-3')	Reverse primers (5'-3')	Melting tempearature (T˚C)	Expected product size (bp) in PCR	Expected product size (bp) in Q-PCR	Putative Functions
OsISM01870	LOC_Os01g12950	7198718	TGCTCTTCTGCCTGGAGTTT	ACATTTGCAGTGGGGTGTG	60.3	774	99	ubiquitin-conjugating enzyme putative expressed
OsISM01871	LOC_Os01g12950	7199624	TGGTTGCAATTCGTTTAGCC	TGTCTTCACCCAATCTTTGGA	60.7	187	98	ubiquitin-conjugating enzyme putative expressed
OsISM01872	LOC_Os01g12950	7199812	TCCAGAATGAATGGAGGCTT	AGCCCTCAGCAGATCAATCC	60.7	743	87	ubiquitin-conjugating enzyme putative expressed
OsISM01873	LOC_Os01g12950	7200617	TCCTTCTGAATACCCACAATCC	GGGCTCTCGTAGCTCATCAA	60.3	1983	70	ubiquitin-conjugating enzyme putative expressed
OsISM01874	LOC_Os01g13020	7239045	CAAGTAGGGGAGTACGAGCG	CCAAGGTCCTAGTTGGTGGA	59.9	596	83	hypothetical protein
OsISM01875	LOC_Os01g13030	7249218	GCTGAGGAGGAGGACAAGAA	CGTGTTCTTGCGGTAGCTG	60.1	873	97	OsIAA3 - Auxin-responsive Aux/IAA gene family member expressed
OsISM01876	LOC_Os01g13030	7250228	GGACATGTCTTTGGGTCTCG	TCTGGTTCTCAGCACCTTCC	60.4	924	69	OsIAA3 - Auxin-responsive Aux/IAA gene family member expressed
OsISM01877	LOC_Os01g13030	7251188	ATGGTGACTGGATGCTGGTT	CCAATTGCATCTGAGCCTTT	60.3	214	92	OsIAA3 - Auxin-responsive Aux/IAA gene family member expressed
OsISM01878	LOC_Os01g13030	7251378	CATGAAAGGCTCAGATGCAA	TCAACTGTGGGCAATAACCA	60.0	969	87	OsIAA3 - Auxin-responsive Aux/IAA gene family member expressed
OsISM01879	LOC_Os01g13050	7262146	TCTGCCTGTTCAGCAACCT	GTAGTCGTAGCACCCGACG	59.4	257	99	long cell-linked locus protein putative
OsISM01880	LOC_Os01g13050	7262415	GTGCTCAAGACGGAGCAGAT	CCCCGGTACTGCATATTGTT	60.1	192	96	long cell-linked locus protein putative
OsISM01881	LOC_Os01g13050	7262552	GAGGGGATGTGACCATCTTG	CGCCATTGTAGTCGTAGCAA	60.1	176	79	long cell-linked locus protein putative
OsISM01882	LOC_Os01g13060	7264513	GGCGGCAAGTTCAAGCTC	GCGATCTCGTAGGTGTCCA	60.9	220	87	CK1 CaseinKinase 1.1 - CK1 includes the casein kinase 1 kinases expressed
OsISM01883	LOC_Os01g13060	7264709	GCACGTGGACACCTACGAG	GCTTCGCCTCATAAAACAGC	60.2	676	81	CK1 CaseinKinase 1.1 - CK1 includes the casein kinase 1 kinases expressed
OsISM01884	LOC_Os01g13060	7265378	GCGAAGCTCTACAACGCTCT	AGCAAATCGATGACGAGGAC	60.1	189	96	CK1 CaseinKinase 1.1 - CK1 includes the casein kinase 1 kinases expressed
OsISM01885	LOC_Os01g13060	7265591	TGTCTATTGCGGCAGGAAAT	TGCAAGTATCCTTTGGAATGC	60.3	593	97	CK1 CaseinKinase 1.1 - CK1 includes the casein kinase 1 kinases expressed
OsISM01886	LOC_Os01g13060	7266200	GACAACTTTCTGATGGGCCT	CGATTTGTAGTGGAATCCCG	59.7	182	93	CK1 CaseinKinase 1.1 - CK1 includes the casein kinase 1 kinases expressed
OsISM01887	LOC_Os01g13060	7266362	CGGGATTCCACTACAAATCG	AACCCCAAGATGAGTGTTGC	60.1	209	94	CK1 CaseinKinase 1.1 - CK1 includes the casein kinase 1 kinases expressed

ISM IDs	Rice gene locus IDs	Physical positions (bp) on chromosomes	Forward primers (5'-3')	Reverse primers (5'-3')	Melting tempearature (T˚C)	Expected product size (bp) in PCR	Expected product size (bp) in Q-PCR	Putative Functions
OsISM01888	LOC_Os01g13060	7266531	CACTGCACGCTATGCAAGTT	CTAAATCATCCCGCCGACT	60.1	144	63	CK1 CaseinKinase 1.1 - CK1 includes the casein kinase 1 kinases expressed
OsISM01889	LOC_Os01g13060	7266656	AGTCGGCGGGATGATTTAG	CAGCTTTAAGTCCCTGCCAC	60.0	978	83	CK1 CaseinKinase 1.1 - CK1 includes the casein kinase 1 kinases expressed
OsISM01890	LOC_Os01g13060	7267630	GCTGCAACAAAGAAGCAGAA	AGCAAATTCCACTGGACAGG	59.7	180	94	CK1 CaseinKinase 1.1 - CK1 includes the casein kinase 1 kinases expressed
OsISM01891	LOC_Os01g13060	7267874	GCCTTCTCCGTGACTTGTTT	TGAACCTTTTGGCCTTGTTT	59.5	206	92	CK1 CaseinKinase 1.1 - CK1 includes the casein kinase 1 kinases expressed
OsISM01892	LOC_Os01g13060	7268036	TTCGACTGGACTGTGTTGAGA	TGTTTTGTCAAGGTCCCCAG	60.2	757	97	CK1 CaseinKinase 1.1 - CK1 includes the casein kinase 1 kinases expressed
OsISM01893	LOC_Os01g13060	7268726	GGCCTGTTTGTAGCATGGTT	CATTATGAGGGAAAGCACCA	59.3	228	96	CK1 CaseinKinase 1.1 - CK1 includes the casein kinase 1 kinases expressed
OsISM01894	LOC_Os01g13060	7269030	AGGTCCCCACCCTAACATTC	ACCAGGTAAAACAGATGCGG	60.0	645	86	CK1 CaseinKinase 1.1 - CK1 includes the casein kinase 1 kinases expressed
OsISM01895	LOC_Os01g13070	7275340	GAGGAGAACGAGGCAGCAT	GTTGGCACGGGGAGAGAT	60.8	157	97	expressed protein
OsISM01896	LOC_Os01g13070	7276117	TCTCAAGCGCACAAGACAAG	TTGGTCTTGATGAAGGTGCC	60.7	225	82	expressed protein
OsISM01897	LOC_Os01g13070	7276282	CCTGCAGGTGATCATCAAGA	GATCTCCGAGCAGATGTCAAG	59.9	520	92	expressed protein
OsISM01898	LOC_Os01g13090	7281497	TTGGGTATCCACCCACAACT	ACTCGTCGATGCGGAAAC	59.9	1016	98	nucleic acid binding protein putative expressed
OsISM01899	LOC_Os01g13090	7282581	GCTAAGGCTTCCAGTGTGCT	GGCTTCCATTAGTTTGTCCG	59.6	129	49	nucleic acid binding protein putative expressed
OsISM01900	LOC_Os01g13090	7282674	TGGATGCAGTCAGTATCGGA	ATCTGGTGAGCCTTTCATGC	60.2	507	97	nucleic acid binding protein putative expressed
OsISM01901	LOC_Os01g13090	7283197	ATGCTCCTGTGCTGGAAGTT	GGTTTGAGCTGGTAAAGGCA	60.1	370	82	nucleic acid binding protein putative expressed
OsISM01902	LOC_Os01g13090	7283547	TGCCTTTACCAGCTCAAACC	CCTTCCTGAACCTTGCATTC	60.0	815	90	nucleic acid binding protein putative expressed
OsISM01903	LOC_Os01g13090	7284338	TGATGAATGCAAGGTTCAGG	GATATTCGCCCCATTCATGT	59.6	184	97	nucleic acid binding protein putative expressed
OsISM01904	LOC_Os01g13100	7287000	CACCACCCATGTTGGCTT	GAATCGGACCTGGAGGAAG	60.0	262	99	KH domain containing protein putative expressed
OsISM01905	LOC_Os01g13100	7287205	CTCGCTCGCCCTCTATGC	CCTTGTGCACCTTTCATTACAA	61.1	710	99	KH domain containing protein putative expressed

ISM IDs	Rice gene locus IDs	Physical positions (bp) on chromosomes	Forward primers (5'-3')	Reverse primers (5'-3')	Melting tempearature (T˚C)	Expected product size (bp) in PCR	Expected product size (bp) in Q-PCR	Putative Functions
OsISM01906	LOC_Os01g13100	7287969	GAGGCCAATCTCCACAGAAG	CTGAGGCTGAATTCTCTTCCA	59.7	306	93	KH domain containing protein putative expressed
OsISM01907	LOC_Os01g13100	7288356	TGGAGGATGCTAGCTTCAATAA	TTTCACAAAGCGCATCAGAG	59.6	218	99	KH domain containing protein putative expressed
OsISM01908	LOC_Os01g13100	7288554	CTCTGATGCGCTTTGTGAAA	TTCAATCTGCTTCTGCATGG	60.0	170	51	KH domain containing protein putative expressed
OsISM01909	LOC_Os01g13100	7288749	TTCCATCTTCTAAGGAGGAAACA	TTTGCAATTTTCTCTGAAGCC	59.4	180	83	KH domain containing protein putative expressed
OsISM01910	LOC_Os01g13100	7288909	GCTTCAGAGAAAATTGCAAAGG	GATAGCCAATGGAAGCGAAA	60.3	1477	88	KH domain containing protein putative expressed
OsISM01911	LOC_Os01g13100	7290577	AATGGATATTAAGGGCTCCCA	CCGTTAGGTGAAAGGTTTTGG	60.2	614	78	KH domain containing protein putative expressed
OsISM01912	LOC_Os01g13100	7291217	GATCGCATTGCTAAGGCTTC	GCTTCCATTACTTGGGACGA	60.0	139	57	KH domain containing protein putative expressed
OsISM01913	LOC_Os01g13100	7291357	TTGGAAAACCGACCAATCTC	AACTTCCAGCACAGGAGCAT	59.9	316	97	KH domain containing protein putative expressed
OsISM01914	LOC_Os01g13100	7291653	ATGCTCCTGTGCTGGAAGTT	CTAGGCCCGACTTGACAAAG	59.9	553	85	KH domain containing protein putative expressed
OsISM01915	LOC_Os01g13100	7292187	TTTGTCAAGTCGGGCCTAGT	CTTCCTGTGCCTCACATTCA	59.8	434	85	KH domain containing protein putative expressed
OsISM01916	LOC_Os01g13100	7292579	CTTCAGTTGCACGTGACCAT	ATTCCGAGCATCGAATGAGT	59.7	163	85	KH domain containing protein putative expressed
OsISM01917	LOC_Os01g13120	7296852	GCTCCTCCTCACCTTCCTCT	GTCAGCCCCATTATCGTGTC	60.2	179	94	aquaporin protein putative expressed
OsISM01919	LOC_Os01g13150	7324526	AAGCTCAACCCCATCAACAC	CGCCATCAAAGGATTGTAGC	60.3	283	70	metallo-beta-lactamase family protein putative expressed
OsISM01920	LOC_Os01g13150	7324720	GAACGATGTCGAGCAACAAA	TCAACAGGTAAAGGAAACCCA	59.7	567	89	metallo-beta-lactamase family protein putative expressed
OsISM01921	LOC_Os01g13150	7325266	TGGGTTTCCTTTACCTGTTGA	TGTGTGTCCATTCCTGTACCA	59.7	332	70	metallo-beta-lactamase family protein putative expressed
OsISM01922	LOC_Os01g13150	7325631	CAAACAGAGGTTTATATTTAATGCTGG	TGGCGAGAAGGACCTCATAC	60.3	372	94	metallo-beta-lactamase family protein putative expressed
OsISM01923	LOC_Os01g13150	7325925	TGCTGTCTGATAGGGTCTTCAA	TGCAGTTTCTGAGCATACACG	60.0	205	98	metallo-beta-lactamase family protein putative expressed
OsISM01924	LOC_Os01g13150	7326074	TCTCCACCTCCCAGATTGAC	TCTCCTATGCCTGCCAGAGT	60.0	202	98	metallo-beta-lactamase family protein putative expressed

ISM IDs	Rice gene locus IDs	Physical positions (bp) on chromosomes	Forward primers (5'-3')	Reverse primers (5'-3')	Melting tempearature (T°C)	Expected product size (bp) in PCR	Expected product size (bp) in Q-PCR	Putative Functions
OsISM01925	LOC_Os01g13150	7326255	GACTCTGGCAGGCATAGGAG	TAGGTCAGAAGGGCCCCATA	60.8	173	59	metallo-beta-lactamase family protein putative expressed
OsISM01926	LOC_Os01g13150	7326741	GTGAACTTCAGCTCGGGAAC	AGTAGGTCCAGGAATCGACG	59.5	580	93	metallo-beta-lactamase family protein putative expressed
OsISM01927	LOC_Os01g13150	7327503	ATTTGGTGCCACACAGCATA	CCTTGCACTACCCTTCAGGA	60.1	829	77	metallo-beta-lactamase family protein putative expressed
OsISM01928	LOC_Os01g13150	7328368	TTCCCATCCTCTGGATTTTG	CTTTCACAGGCTTGCAATGA	59.9	172	87	metallo-beta-lactamase family protein putative expressed
OsISM01929	LOC_Os01g13150	7328520	TCATTGCAAGCCTGTGAAAG	GTTGAGCATATGGACGCAGA	59.9	663	68	metallo-beta-lactamase family protein putative expressed
OsISM01930	LOC_Os01g13150	7329607	TTGCGGTGAAGGAACCTTAG	CAGCATCATCAGCACCACTT	60.1	146	63	metallo-beta-lactamase family protein putative expressed
OsISM01931	LOC_Os01g13150	7330296	CGTCCAGAGATGCCACTGTA	CGATCGCCTCATCCTTCATA	60.5	458	67	metallo-beta-lactamase family protein putative expressed
OsISM01932	LOC_Os01g13160	7333147	CAAGCGACTCGACAACAAGA	TCCCGCTTGAAATCAGTACC	60.1	1420	94	expressed protein
OsISM01933	LOC_Os01g13160	7335207	AAATTGGACGCAGTTGCTTT	GCTTCCAATACACAAATTGCC	59.6	1112	93	expressed protein
OsISM01934	LOC_Os01g13160	7336393	TTAGATGCTGTGAGCTTCCG	TTCCAGTAGGTTGTTCCCCA	59.8	259	85	expressed protein
OsISM01935	LOC_Os01g13160	7337484	TGCTGTGATGAGCAACTCAA	TTTTTGAACCACGAGCTGC	59.5	276	84	expressed protein
OsISM01937	LOC_Os01g13170	7340008	ACCCAGCAGCAGCAGAAG	TGCTCAATCCAACTTGGTCA	60.3	1253	62	ubiquitin-conjugating enzyme E2 putative expressed
OsISM01938	LOC_Os01g13170	7341612	ATTATGCAGCAACATCAGGG	GCTGAATCCTCTTCACCCAG	59.2	145	55	ubiquitin-conjugating enzyme E2 putative expressed
OsISM01939	LOC_Os01g13170	7341755	GCATGACTGGAAACTTTTGGA	GGTCCGATAATAGCAGCCCT	60.3	963	97	ubiquitin-conjugating enzyme E2 putative expressed
OsISM01940	LOC_Os01g13170	7342736	CGATGGGCTCTTCTTCTTTG	TTGGGTTAATCCGTAGTCCG	59.9	182	96	ubiquitin-conjugating enzyme E2 putative expressed
OsISM01941	LOC_Os01g13170	7343148	TCATGCAGGACGATGCTCTA	TCCCTGAAGTGGCTGGTAAC	60.3	620	75	ubiquitin-conjugating enzyme E2 putative expressed
OsISM01942	LOC_Os01g13190	7347862	CGACTTCTCCTCCATTTTCG	ACTTACTCCTTGACCGCAGC	59.7	227	82	histidinol dehydrogenase chloroplast precursor putative expressed
OsISM01943	LOC_Os01g13190	7348016	TTGGGTGCGAGTAGGTCAAT	TGCAACCTTGTCGAACTTCA	60.5	187	93	histidinol dehydrogenase chloroplast precursor putative expressed

ISM IDs	Rice gene locus IDs	Physical positions (bp) on chromosomes	Forward primers (5'-3')	Reverse primers (5'-3')	Melting tempearature (T˚C)	Expected product size (bp) in PCR	Expected product size (bp) in Q-PCR	Putative Functions
OsISM01944	LOC_Os01g13190	7348217	TGTGCGTGTTAGCGATCTTC	CCACATCAAAGGCTTCCTTC	59.8	310	63	histidinol dehydrogenase chloroplast precursor putative expressed
OsISM01945	LOC_Os01g13190	7348557	CGCAAAAGTTGCCTGAAAAG	AAAGGCCAACAGAACCAATG	60.4	189	85	histidinol dehydrogenase chloroplast precursor putative expressed
OsISM01946	LOC_Os01g13190	7348762	TGCAGTATTGCCTTCCACTG	GCTACCATCACGACTAGGGG	59.7	189	98	histidinol dehydrogenase chloroplast precursor putative expressed
OsISM01947	LOC_Os01g13190	7348931	CCCCTAGTCGTGATGGTAGC	TCCTACCTGAGCTCCTCCAG	59.6	205	96	histidinol dehydrogenase chloroplast precursor putative expressed
OsISM01948	LOC_Os01g13190	7349105	TGTATTGAAAGCTGGAGGAGC	CCTTTGGGCATGAGACTGTT	59.8	148	66	histidinol dehydrogenase chloroplast precursor putative expressed
OsISM01949	LOC_Os01g13190	7349201	TTCTTTCAGGCCATTTCAGC	TTTTTGCAGCCGTGACATAC	60.0	468	98	histidinol dehydrogenase chloroplast precursor putative expressed
OsISM01950	LOC_Os01g13190	7349649	GTATGTCACGGCTGCAAAAA	ATACTTCTGAAGGGCCAGCA	59.8	158	81	histidinol dehydrogenase chloroplast precursor putative expressed
OsISM01951	LOC_Os01g13190	7349815	GCGGATAAATATGCAAACCC	AACAACCTGACTGTCTGGGC	59.7	259	79	histidinol dehydrogenase chloroplast precursor putative expressed
OsISM01952	LOC_Os01g13190	7350190	TGTTTTTGCGAAAGACATGG	CCCACTGTTCAGCATCCTTT	59.9	272	93	histidinol dehydrogenase chloroplast precursor putative expressed
OsISM01953	LOC_Os01g13190	7350442	AAAGGATGCTGAACAGTGGG	GGTCCATTGTCCCAAGAAGA	60.0	155	65	histidinol dehydrogenase chloroplast precursor putative expressed
OsISM01954	LOC_Os01g13250	7385021	GAGGAGCTCCGCTCCATAG	ACCCTTTAGCACACTTCCGA	59.9	873	88	sarcoma antigen NY-SAR-91 putative expressed
OsISM01955	LOC_Os01g13250	7385874	TCGGAAGTGTGCTAAAGGGT	GAACAGCCTCTCATCAGCCT	59.6	668	99	sarcoma antigen NY-SAR-91 putative expressed
OsISM01956	LOC_Os01g13250	7386529	TGAGAGGCTGTTCTCCCTGT	CTCGTCCAGCCATATGTTCA	59.8	221	63	sarcoma antigen NY-SAR-91 putative expressed
OsISM01957	LOC_Os01g13250	7386726	TCGATGAACATATGGCTGGA	CAGGATGCAGCTGGTATTGA	59.9	105	65	sarcoma antigen NY-SAR-91 putative expressed
OsISM01958	LOC_Os01g13250	7386825	ATCCTGGCAGATGGAAGAGA	GCGACCTCCTTTCTTTGGTT	60.2	268	89	sarcoma antigen NY-SAR-91 putative expressed
OsISM01959	LOC_Os01g13270	7401496	CAAGCATTTCAACGAGCAAG	AGGTCCCTGTACACAATGCC	59.7	765	94	AGC PVPK like kin82y.4 - ACG kinases include homologs to PKA PKG and PKC expressed
OsISM01960	LOC_Os01g13300	7416398	GGAGTTCGGAGAGATCAACC	GTGTTCTTGGAACCTGGTGG	59.5	627	90	B3 DNA binding domain containing protein expressed
OsISM01961	LOC_Os01g13300	7417096	GCGACAGGGATGACGATATT	AAGGAACAACCTCCATCACG	59.9	951	85	B3 DNA binding domain containing protein expressed

ISM IDs	Rice gene locus IDs	Physical positions (bp) on chromosomes	Forward primers (5'-3')	Reverse primers (5'-3')	Melting tempearature (T˚C)	Expected product size (bp) in PCR	Expected product size (bp) in Q-PCR	Putative Functions
OsISM01962	LOC_Os01g13300	7418027	CGTGATGGAGGTTGTTCCTT	TTTGCTCATTTTCCACCTCC	60.0	639	57	B3 DNA binding domain containing protein expressed
OsISM01963	LOC_Os01g13300	7418703	GAACACTAGCAATGGACGCA	TGCACATTTATTTTGGCCCT	60.1	393	62	B3 DNA binding domain containing protein expressed
OsISM01964	LOC_Os01g13300	7419068	GCCAGTTAAGGGCCAAAATA	GATCCACAGAACGCCTGAAC	59.9	147	70	B3 DNA binding domain containing protein expressed
OsISM01965	LOC_Os01g13300	7419294	CATGTTGTCAGCGGATTTTG	CAGTGTCATGCTTTGGGAGA	60.0	515	71	B3 DNA binding domain containing protein expressed
OsISM01966	LOC_Os01g13300	7419913	ATCAAGGTTGGCGATGTTGT	GCTCTGTCAGTTGGGCTGAT	60.4	265	96	B3 DNA binding domain containing protein expressed
OsISM01967	LOC_Os01g13300	7420158	ATCAGCCCAACTGACAGAGC	CCTTAGGCTTGGTGGCATTA	60.3	208	98	B3 DNA binding domain containing protein expressed
OsISM01968	LOC_Os01g13320	7429498	TCCACGACAACTACACCACC	CATGTCCCGCATGATGAA	59.7	160	72	pectinesterase putative expressed
OsISM01969	LOC_Os01g13340	7437587	GTGCTGACGCACCACGTC	TCAACAGATGCTTTGATGGC	61.2	912	95	harpin-induced protein 1 domain containing protein expressed
OsISM01970	LOC_Os01g13390	7457193	GTCAGCTACAGGGATGACCC	AACCCCTCTCCACCTCCTC	60.0	674	68	expressed protein
OsISM01971	LOC_Os01g13390	7458031	GTCCCCTCCAACAACAAAAA	GGACTGGATCACCTCTCAGC	59.8	495	54	expressed protein
OsISM01972	LOC_Os01g13420	7469978	ACGACATCTCCTTCCACGTC	CTGGAGGAGTTAAGGTTCGC	59.5	1077	83	SOUL heme-binding protein putative expressed
OsISM01973	LOC_Os01g13470	7506693	ATGTGCTTTACCCCGAGATG	TTCAGACCTTTCCTGACTGAG	58.5	186	67	KH domain containing protein putative expressed
OsISM01974	LOC_Os01g13470	7507218	TCCAAAATCTGAAGATCGCA	CAGGGGAAATTCCATCACC	59.7	898	66	KH domain containing protein putative expressed
OsISM01975	LOC_Os01g13470	7508312	GGGTGTCCCAGAAAATGATG	AAGATGGTTCCGCAGTCTTG	60.2	453	98	KH domain containing protein putative expressed
OsISM01976	LOC_Os01g13470	7508912	GGACGACCATTGGATGAGAT	CAGGCAATGAAATTGGATCA	59.6	923	77	KH domain containing protein putative expressed
OsISM01977	LOC_Os01g13470	7509844	GAATGGCGCATAGAGGAATG	TTTTGGGACCATGACATCTG	60.0	170	87	KH domain containing protein putative expressed
OsISM01978	LOC_Os01g13470	7510029	CTATGTGGGGAAGATGGAGG	CATCGGATCTGCGACAGTTA	59.6	160	76	KH domain containing protein putative expressed
OsISM01979	LOC_Os01g13500	7527787	GGGATTCAGCTGCATTGG	GAGACGGCACAGGGAGATAC	59.9	1255	53	hypothetical protein

ISM IDs	Rice gene locus IDs	Physical positions (bp) on chromosomes	Forward primers (5'-3')	Reverse primers (5'-3')	Melting tempearature (T°C)	Expected product size (bp) in PCR	Expected product size (bp) in Q-PCR	Putative Functions
OsISM01980	LOC_Os01g13510	7542720	CGGGAGTTGAGGACTACACA	TGCTTCTAATGGTTTCACTTTCG	59.7	299	75	hypothetical protein
OsISM01981	LOC_Os01g13510	7543031	TCGATAGGGAAAGGTTTGACA	GTGATAGCACGCCTACGTGA	59.7	458	78	hypothetical protein
OsISM01982	LOC_Os01g13520	7546017	GCCGCCTCTCTTCTTCTTCT	GTCCCCTACCCCTCCTCCT	60.9	200	98	auxin response factor 1 putative expressed
OsISM01983	LOC_Os01g13520	7546286	CGGGTGTTCTACTTCCTCCA	GTACGGCACCTGGAACATCT	60.1	216	97	auxin response factor 1 putative expressed
OsISM01984	LOC_Os01g13520	7546478	ATCAAGATGTTCCAGGTGCC	GTAATCTGCGCGAACACCTC	60.4	210	96	auxin response factor 1 putative expressed
OsISM01985	LOC_Os01g13520	7546668	GAGGTGTTCGCGCAGATTA	GGGTCAGGTAATGTTGGCAG	60.2	467	66	auxin response factor 1 putative expressed
OsISM01986	LOC_Os01g13520	7547215	GGTTCTCCGTGCTCCGAC	TGTGATGAGCTCTTGGGTTG	60.8	174	84	auxin response factor 1 putative expressed
OsISM01987	LOC_Os01g13520	7547369	CAACCCAAGAGCTCATCACA	AGTCAGAAGATGCCTACGGG	59.6	229	90	auxin response factor 1 putative expressed
OsISM01988	LOC_Os01g13520	7547625	AAGAAGCTGATTTCTGGCGA	TCTGTTTCTGCACAAGACGC	60.1	204	89	auxin response factor 1 putative expressed
OsISM01989	LOC_Os01g13520	7547888	GCATCTCATGCTATCAAGACT AATTC	GCCGCAAGGTACTTATTCACA	59.9	185	99	auxin response factor 1 putative expressed
OsISM01990	LOC_Os01g13520	7548092	GTGGGCATGAGGTTCAAGAT	CCACTGCAGAGAAAGATCACC	59.9	197	97	auxin response factor 1 putative expressed
OsISM01991	LOC_Os01g13520	7548279	TCTGCAGTGGTCAGGTTCTG	ACCCTCTCAGGGCCATTAAC	60.2	162	79	auxin response factor 1 putative expressed
OsISM01992	LOC_Os01g13520	7548498	GCAGTCAGCAACGAAAAACA	GGCATTCCAGATAGCCAAAA	60.0	183	97	auxin response factor 1 putative expressed
OsISM01993	LOC_Os01g13520	7549256	GCCATCAGAATTACACCGCT	CTAGATCCACAGCTCTGCCC	60.0	867	76	auxin response factor 1 putative expressed
OsISM01994	LOC_Os01g13520	7550196	ATGGAAGGTGGCTTTCACTG	TCTTTCTGACCATCTGGCAA	59.7	176	87	auxin response factor 1 putative expressed
OsISM01995	LOC_Os01g13530	7556814	CAGGAAGGCCTCCTCTTCTC	CTGTGAACGCAGATTCCTCA	60.2	918	55	ABIL2 putative expressed
OsISM01996	LOC_Os01g13530	7557741	GCGGCGGAATATTTTGAAGT	AGTGCTAACAAGGGCCTCAA	60.6	195	97	ABIL2 putative expressed
OsISM01997	LOC_Os01g13530	7557980	CAAGGAAAGATCCGATGAGG	ATCTTCCCTCCTGGTCGATT	59.8	296	99	ABIL2 putative expressed

ISM IDs	Rice gene locus IDs	Physical positions (bp) on chromosomes	Forward primers (5'-3')	Reverse primers (5'-3')	Melting tempearature (T˚C)	Expected product size (bp) in PCR	Expected product size (bp) in Q-PCR	Putative Functions
OsISM01998	LOC_Os01g13530	7558298	AGCACCAAAATACCACAGGC	GGATGAATGGCTGATTCCAC	60.1	148	64	ABIL2 putative expressed
OsISM01999	LOC_Os01g13530	7558481	TGAGCCGTAAAATGCACAAG	TGTAGATTGCCTGCCAACTG	59.9	132	57	ABIL2 putative expressed
OsISM02000	LOC_Os01g13530	7558593	CAGTTGGCAGGCAATCTACA	TCGAGAAGGTGACAAGGACC	60.1	228	93	ABIL2 putative expressed
OsISM02001	LOC_Os01g13530	7558828	GAGCTAAATCCCCATCTCCC	TCGGTATTGGTGAACCTGCT	60.2	194	70	ABIL2 putative expressed
OsISM02002	LOC_Os01g13530	7559055	CAAGAAGGCCACCTGTTCAT	TGCTCTTTGTGATTGCTCCA	60.3	237	80	ABIL2 putative expressed
OsISM02003	LOC_Os01g13540	7570765	CGTGGTCGAGGAAGTCCAT	AGGTGAAACTGCCATTTGCT	60.4	1280	91	NIN putative expressed
OsISM02004	LOC_Os01g13540	7572504	TGCTGGTGAGGTTGATAAAGTC	ATTGAATGCACACCTGGACA	59.6	259	96	NIN putative expressed
OsISM02005	LOC_Os01g13540	7572721	CTGAGATATTGGACCATCCGA	CGACCGTCAAGATCTCCAGT	60.1	178	85	NIN putative expressed
OsISM02006	LOC_Os01g13540	7573765	AAAAATGCAGCAAAAAGCCT	AACGAGAAATCCCATGTTGC	59.5	859	74	NIN putative expressed
OsISM02007	LOC_Os01g13560	7587505	AGTTCCGCTACACTCCATCG	AGGACGACCAAGCTGCAC	60.1	403	74	membrane associated DUF588 domain containing protein putative expressed
OsISM02008	LOC_Os01g13620	7634951	AACTGGGTCATGGCGAATTA	ACTCATCGTCCTTCCACTGC	60.3	193	83	retrotransposon protein putative unclassified
OsISM02009	LOC_Os01g13620	7635522	ATAAGCCGAGCTCCACTGC	GAGCTTCACCTCTGACTAGCG	60.0	412	61	retrotransposon protein putative unclassified
OsISM02010	LOC_Os01g13620	7636523	CGATGATCCGTCCAAGACC	ATCTTCTCTAGCCATGCGGA	60.7	485	83	retrotransposon protein putative unclassified
OsISM02011	LOC_Os01g13620	7637186	ACACGTTGTTGCAGGACTTG	ATGCGCTTGGAAGTAGTGGT	59.8	853	99	retrotransposon protein putative unclassified
OsISM02012	LOC_Os01g13620	7638960	GCCAACGGGATACTCTTGAA	GCGACGTACAGGTGCTGATA	60.0	316	93	retrotransposon protein putative unclassified
OsISM02013	LOC_Os01g13640	7647807	AGTGGCGGCCTTAGGTTC	GTGTCCTCTCTCGTGCTGCT	60.5	796	76	retrotransposon putative centromere-specific
OsISM02014	LOC_Os01g13690	7666484	AGTGGGACGACCAGCTCAT	ATTTCATGTCCTCCCAGAGC	59.9	181	82	ligA putative expressed
OsISM02015	LOC_Os01g13700	7669383	ATCGCGGAGTGCCTCATC	AGCACCAGGCTTCAACAAGT	61.1	1899	85	DNA-binding protein-related putative expressed

ISM IDs	Rice gene locus IDs	Physical positions (bp) on chromosomes	Forward primers (5'-3')	Reverse primers (5'-3')	Melting tempearature (T˚C)	Expected product size (bp) in PCR	Expected product size (bp) in Q-PCR	Putative Functions
OsISM02016	LOC_Os01g13710	7672322	CCTTCACAAGGCAGGAGAAC	CCAGCAGGTAGCTCGTGAA	60.0	248	91	transposon protein putative unclassified expressed
OsISM02017	LOC_Os01g13710	7672679	CAGCTTGTTCTGCACTCTGC	TCAGCTTGTAGTCCACGATCA	59.7	210	54	transposon protein putative unclassified expressed
OsISM02018	LOC_Os01g13710	7672920	CGTCAACAGCTTCCACACAC	ACGGATTTGAACATCGCATT	60.3	150	70	transposon protein putative unclassified expressed
OsISM02019	LOC_Os01g13710	7673331	GACCAGCTTCAAGGGAATCA	CCTAGCTGGAACAGGCAATC	60.0	740	79	transposon protein putative unclassified expressed
OsISM02020	LOC_Os01g13730	7678208	GGGATGAGGGTGAAGGTGAT	AAGTGCTGGATCATAATTGCG	60.6	796	97	WD domain G-beta repeat domain containing protein expressed
OsISM02021	LOC_Os01g13730	7679017	AAGCGGTTGAATACACCAGG	CCATTGCTCCAATAAATGGC	60.1	158	76	WD domain G-beta repeat domain containing protein expressed
OsISM02022	LOC_Os01g13730	7679193	TATCATGCATGGCGAAAAAC	AGCAATGTCCCACAGACGA	59.9	789	84	WD domain G-beta repeat domain containing protein expressed
OsISM02023	LOC_Os01g13730	7679950	TGCTGGCAGATATTCGTCTG	CAGTAGATGTCGCCAAACCC	60.2	275	97	WD domain G-beta repeat domain containing protein expressed
OsISM02024	LOC_Os01g13730	7680209	TTGGCGACATCTACTGATGG	CTTGAGCATAGGGACTTTCCA	59.5	436	82	WD domain G-beta repeat domain containing protein expressed
OsISM02025	LOC_Os01g13730	7680624	TGGAAAGTCCCTATGCTCAAG	TTACCAAAACGCATGCTTCC	60.2	180	97	WD domain G-beta repeat domain containing protein expressed
OsISM02026	LOC_Os01g13730	7680772	CGGTCTACACCTGGAAGCAT	AAAAGATTGCCATCCCACTG	60.0	156	59	WD domain G-beta repeat domain containing protein expressed
OsISM02027	LOC_Os01g13730	7680928	TGCAACTGTAGGTGCTCAGG	TGTATCTTTACCCCATTCAAAGC	59.7	166	80	WD domain G-beta repeat domain containing protein expressed
OsISM02028	LOC_Os01g13730	7681101	CCGTGAGATTTAATCCTGGAG	AGGGGATGACATGCGTAGAT	59.0	318	90	WD domain G-beta repeat domain containing protein expressed
OsISM02029	LOC_Os01g13730	7681398	GATCTACGCATGTCATCCCC	CTCCCTAGGGTTCCAGCATA	59.7	682	76	WD domain G-beta repeat domain containing protein expressed
OsISM02030	LOC_Os01g13730	7682065	TGGAACCCTAGGGAGCCTAT	TGAACAACTTTGGCTTCATCA	59.6	273	96	WD domain G-beta repeat domain containing protein expressed
OsISM02031	LOC_Os01g13730	7682333	GTTCACAAGGGCCATGTTTC	AACCCGTAACAAATTCACGC	60.1	514	71	WD domain G-beta repeat domain containing protein expressed
OsISM02032	LOC_Os01g13730	7682827	GCGTGAATTTGTTACGGGTT	TCTCCCTGCTATGATCACCA	59.5	169	72	WD domain G-beta repeat domain containing protein expressed
OsISM02033	LOC_Os01g13730	7682976	TGGTGATCATAGCAGGGAGAT	CATCACTGCCAGAAACCAAA	59.6	335	99	WD domain G-beta repeat domain containing protein expressed

ISM IDs	Rice gene locus IDs	Physical positions (bp) on chromosomes	Forward primers (5'-3')	Reverse primers (5'-3')	Melting tempearature (T˚C)	Expected product size (bp) in PCR	Expected product size (bp) in Q-PCR	Putative Functions
OsISM02034	LOC_Os01g13730	7683326	TGTGGAAGTCCAAAGCTTCA	GGTGCTTATACCGCTCCTTG	59.6	880	97	WD domain G-beta repeat domain containing protein expressed
OsISM02035	LOC_Os01g13730	7684179	ATGCTGTCAAGGAGCGGTAT	CCGCCTTGTAGATTGGCTTA	60.0	209	85	WD domain G-beta repeat domain containing protein expressed
OsISM02036	LOC_Os01g13760	7711774	GTTCAAGCAGATCTCCGAGG	GCGTTTCTGGGGATCACTAA	60.0	991	50	dnaJ domain containing protein expressed
OsISM02037	LOC_Os01g13760	7713116	CTTGCAGATCTGTACAAGGGTG	TCTGCAAAATTTCCTCCTGC	60.1	2729	98	dnaJ domain containing protein expressed
OsISM02038	LOC_Os01g13800	7740776	GTGGTAGAAGCCACTGTCCG	ATTGACGCGGAGTGTGTATG	60.2	179	97	receptor-like protein kinase 5 precursor putative expressed
OsISM02039	LOC_Os01g13810	7744000	ACTACTGCGCCGAGAGAGC	TACTCAAGCGTCCACACCTT	59.6	174	95	expressed protein
OsISM02040	LOC_Os01g13810	7744271	CCCGACGTGGTGTACTTCTT	ACTGAAGTCTTACGCTCCCG	59.8	1006	87	expressed protein
OsISM02041	LOC_Os01g13810	7745373	TGTGTTTCCTTACGGCCTTC	TCCAACTGGTTATCACACGC	59.8	1313	97	expressed protein
OsISM02042	LOC_Os01g13830	7754917	AAGCAGAGCCACCAAGCTC	CGAGGGAAACGTCAGAAAAG	60.3	2175	97	expressed protein
OsISM02043	LOC_Os01g13830	7757278	ATCTTCGTGTGATTGGGGAT	TCCCCTTGCCCTTCTCTACT	59.7	185	79	expressed protein
OsISM02044	LOC_Os01g13830	7757600	CAGGACCTCCTACTGGACGA	GAACCTCGAGTTCTTGCTGC	60.2	136	88	expressed protein
OsISM02045	LOC_Os01g13830	7757804	GCGCTGTAGGGGAGTCTGTA	CGACTGCCTCGTCATCCAT	61.3	1130	96	expressed protein
OsISM02046	LOC_Os01g13830	7759074	GTGGATACTTACGGGCCTGA	CGTGATGATACGGTGACCTG	60.0	169	74	expressed protein
OsISM02048	LOC_Os01g13860	7772054	ACGCCCGTGTGTTTTCTTAC	CACTCGAATTATTGGCAGCA	59.9	1176	94	hypothetical protein
OsISM02049	LOC_Os01g13880	7779254	GAAAAGGGAGAGAGTGGCG	GTCAAGCGGAGGAGATCAAG	59.9	164	90	hypothetical protein
OsISM02051	LOC_Os01g13890	7785162	CATGTCGCCTTCTGACCC	CTTCTGTGCCGACGACCT	60.1	305	90	retrotransposon protein putative unclassified
OsISM02052	LOC_Os01g13890	7785994	GTCCTCAAATACCTGGTCCG	CGGTCCTCGGGTAGAGTCTT	60.0	519	87	retrotransposon protein putative unclassified
OsISM02053	LOC_Os01g13910	7791086	GAAAGGACGGGGAAAAAGAG	GGAAGTTGGCAACCTTAGCA	60.1	438	77	hypothetical protein

ISM IDs	Rice gene locus IDs	Physical positions (bp) on chromosomes	Forward primers (5'-3')	Reverse primers (5'-3')	Melting tempearature (T°C)	Expected product size (bp) in PCR	Expected product size (bp) in Q-PCR	Putative Functions
OsISM02054	LOC_Os01g13910	7791565	GCCGTCGACCTTGATAACAT	CCATGATCACTGGTTGTTGG	59.9	240	90	hypothetical protein
OsISM02055	LOC_Os01g13910	7791847	GCTAGAGGATGGCTTGGCTA	AGACCATGCGAATGAAAAGC	59.9	654	87	hypothetical protein
OsISM02056	LOC_Os01g13910	7792444	GGAGAATAGAGAATCATATGC GTTG	ACATGGGGACGTTGAGGTT	60.3	138	67	hypothetical protein
OsISM02057	LOC_Os01g13910	7792975	TCACCTCCCTCTCCATCTGT	TGGAAGTTATCGAGGATGGC	59.8	172	95	hypothetical protein
OsISM02058	LOC_Os01g13910	7793269	CCAGCAAACCCACTCTGAAT	GAGCCAGGGAAATCCTCTTC	60.1	171	95	hypothetical protein
OsISM02060	LOC_Os01g13960	7822369	GCTTAGCAGGCGACAGAGTC	CTGATCATCACTTGGCCGAC	61.0	2020	86	retrotransposon protein putative unclassified
OsISM02061	LOC_Os01g13960	7824423	CTTTGTGAAAAGGGGGCAG	AAAGATGACGCCACCTGATG	60.8	168	99	retrotransposon protein putative unclassified
OsISM02062	LOC_Os01g13960	7824643	AATGTTCCGATAAAGCCGAG	TTACTTCTCGCCAATCGTCA	59.3	202	94	retrotransposon protein putative unclassified
OsISM02063	LOC_Os01g13960	7824813	GACAACGCATTGTGACGATT	ACGCACTAGAAGCTCTTCCG	59.7	151	79	retrotransposon protein putative unclassified
OsISM02064	LOC_Os01g13960	7825133	CATTGGCTGCGAGATTGC	TCCCTCATCTTCGTGAGGTC	60.9	110	76	retrotransposon protein putative unclassified
OsISM02065	LOC_Os01g13960	7825625	GAAATCGGTGATCTGGGAGA	ATTACTGCCAGTGACTCGGG	60.1	223	99	retrotransposon protein putative unclassified
OsISM02066	LOC_Os01g13960	7825905	TATAGAGGGAAGGATGGCGA	CGCCACTTCAAACTTCGTCT	60.0	198	98	retrotransposon protein putative unclassified
OsISM02068	LOC_Os01g14010	7846962	CCAAGAAGAAGCGCAATCTC	AACCTGTTCGTCGCCATC	59.9	178	89	ZOS1-05 - C2H2 zinc finger protein expressed
OsISM02069	LOC_Os01g14010	7847414	AAGGCCCACTCCAAGACCT	GTGCGTGATGAAGCTGTCC	60.7	321	85	ZOS1-05 - C2H2 zinc finger protein expressed
OsISM02070	LOC_Os01g14020	7851893	CGCAACTTCATCATCTGCAT	AATGCGCCTTTGTATCCTTG	60.0	916	64	expressed protein
OsISM02071	LOC_Os01g14020	7852866	GCTGCTACGGATCTCATTGG	ACCTTGCATTTCTTTGCACC	60.4	244	83	expressed protein
OsISM02072	LOC_Os01g14020	7853123	GGAGGAAGCTCGTAAACAGG	GCTCAATTTCAGCAAGCCTC	59.5	1206	99	expressed protein
OsISM02074	LOC_Os01g14070	7875448	AGGGCGATCCATACTACCG	CACGAGCACCTGCTTCATAA	60.0	775	73	60S ribosomal protein L18a-1 putative expressed

ISM IDs	Rice gene locus IDs	Physical positions (bp) on chromosomes	Forward primers (5'-3')	Reverse primers (5'-3')	Melting tempearature (T˚C)	Expected product size (bp) in PCR	Expected product size (bp) in Q-PCR	Putative Functions
OsISM02075	LOC_Os01g14070	7876239	CCTTCCTTGCTGTGGGATT	AATGCTCCGACATACCAAGG	60.0	234	85	60S ribosomal protein L18a-1 putative expressed
OsISM02076	LOC_Os01g14070	7876464	CGGAGCATTCCTACTGTGGT	ATAGCAATGGTGGCAAGGAC	60.0	1069	94	60S ribosomal protein L18a-1 putative expressed
OsISM02077	LOC_Os01g14090	7890654	TCCAGTCCAACAGCAATGAA	CCAGACATGTCCTTCCCATT	60.0	183	81	kinesin motor domain containing protein putative expressed
OsISM02078	LOC_Os01g14090	7891015	AGCAAACTCACCCAATTGCT	AACATTAGCGTCTTCGCGTT	59.8	245	57	kinesin motor domain containing protein putative expressed
OsISM02079	LOC_Os01g14090	7891332	TTGCGTTGAACTTGGAACTG	GCAAGTGCCCTCTTGAGATT	59.7	383	91	kinesin motor domain containing protein putative expressed
OsISM02080	LOC_Os01g14090	7891933	TTGATGGCTTCAATCACCAC	TGGATCTTCAGAGGATGTTGC	59.9	161	72	kinesin motor domain containing protein putative expressed
OsISM02081	LOC_Os01g14090	7892353	AATTGATCCATGGCTCTGAAA	TGGGACATCATTGTTTGTGC	59.9	191	90	kinesin motor domain containing protein putative expressed
OsISM02082	LOC_Os01g14090	7892607	CCATCGACCATGTCACGTAG	CTTGGCACCTCTTTTATCGC	59.9	647	70	kinesin motor domain containing protein putative expressed
OsISM02083	LOC_Os01g14120	7923564	TGATCTCTTGGATTGCGATG	TATGTCTGGCACATTGGCAC	60.2	146	99	hypothetical protein
OsISM02084	LOC_Os01g14160	7935593	CTCGTCCTTGCAGAGCTGA	GTTTCCACCGACGGCTTC	61.0	355	84	retrotransposon putative centromere-specific
OsISM02085	LOC_Os01g14190	7946462	GATCTCCTTGGCTCCTTGCT	CTACAATCACGGTGGTGACG	60.4	129	86	transposon protein putative unclassified
OsISM02086	LOC_Os01g14190	7947322	ATTTGCGATGGCATCTTCTT	GGCACACTTCTGAAATGCCT	60.0	165	46	transposon protein putative unclassified
OsISM02087	LOC_Os01g14190	7948440	GCAAGCTTTAGTTTGCACCA	CGCTTTCAATGTCCAATTCA	59.4	193	90	transposon protein putative unclassified
OsISM02088	LOC_Os01g14190	7948628	AAGCGTAGAGAGGCATCTGG	TCATCCTGTTCCATGTCATCA	59.8	148	73	transposon protein putative unclassified
OsISM02089	LOC_Os01g14200	7952720	TACCGGAGAATAGCAGCGTC	ATCTATTCCAGCATTTGGCG	60.2	654	94	retrotransposon protein putative unclassified expressed
OsISM02090	LOC_Os01g14200	7953463	CCAGAATTCCATAACCACCG	CTCTTGAGGCGTCGTTTTTC	60.1	185	94	retrotransposon protein putative unclassified expressed
OsISM02091	LOC_Os01g14200	7953644	AGAGGTCTGGTTCTCAGCCA	AGTATGATCGCTCTGGGCAC	60.1	325	76	retrotransposon protein putative unclassified expressed
OsISM02092	LOC_Os01g14200	7955291	TCCGATATTAAGGAGGGAAGG	GGAGACCACGCTTTTGGAAT	60.4	162	77	retrotransposon protein putative unclassified expressed

ISM IDs	Rice gene locus IDs	Physical positions (bp) on chromosomes	Forward primers (5'-3')	Reverse primers (5'-3')	Melting tempearature (T˚C)	Expected product size (bp) in PCR	Expected product size (bp) in Q-PCR	Putative Functions
OsISM02093	LOC_Os01g14200	7958295	ATACCATGAGGTGCTGGAGG	CATTTTTGCACAAGAACACGA	59.9	235	61	retrotransposon protein putative unclassified expressed
OsISM02094	LOC_Os01g14200	7958509	TCGTGTTCTTGTGCAAAAATG	TGGGTTGTCAGCACCTAACTC	60.0	181	95	retrotransposon protein putative unclassified expressed
OsISM02095	LOC_Os01g14200	7958748	AATTGCTCCACCTTGGTTTG	TCGCAGGGAGGGTATATCTG	60.0	150	74	retrotransposon protein putative unclassified expressed
OsISM02096	LOC_Os01g14200	7959087	ATATGGTGGACTCCGTCAGC	ATTTGCAAGCTGAGTTGCCT	60.0	746	81	retrotransposon protein putative unclassified expressed
OsISM02097	LOC_Os01g14200	7959852	TCCTGAGGTCAACTCCTTCAA	ACAGCTGGGAGCTCAATCAT	59.8	162	73	retrotransposon protein putative unclassified expressed
OsISM02098	LOC_Os01g14200	7960054	CAGATCAAGGACCTCAACCC	CAGGGTACCACCAGGATGAT	59.6	190	98	retrotransposon protein putative unclassified expressed
OsISM02099	LOC_Os01g14200	7960300	AATACATCCTGCCCTGCTGT	CCTGCAATGAGGCTGAGTTT	60.0	135	60	retrotransposon protein putative unclassified expressed
OsISM02100	LOC_Os01g14200	7960887	AACCTCCAGCTGAGCTTCCT	TCGTCAGCAACTATCTGGCTT	60.3	164	74	retrotransposon protein putative unclassified expressed
OsISM02101	LOC_Os01g14200	7961065	GCACGTCCAAGTCCTACAAGA	TCAGCAACACCATCACCATT	60.1	177	92	retrotransposon protein putative unclassified expressed
OsISM02102	LOC_Os01g14200	7961222	AATGGTGATGGTGTTGCTGA	CATGGAACGCATTGTTGTTT	59.7	548	69	retrotransposon protein putative unclassified expressed
OsISM02103	LOC_Os01g14200	7961781	GCGCGTACAACGTATCACAT	GCTGTGGTGGAAGAAGAGGA	60.0	248	51	retrotransposon protein putative unclassified expressed
OsISM02104	LOC_Os01g14200	7962072	CCTGCTCGAGGAAGAACAAC	CTCAGCGCTCCAATCAAACT	60.3	344	69	retrotransposon protein putative unclassified expressed
OsISM02105	LOC_Os01g14260	7992259	AATCCCTCCGTTTTGAAGGT	CCCACACAGGGTCCCTACTA	59.8	523	62	transposon protein putative Ac/Ds sub-class
OsISM02106	LOC_Os01g14260	7993101	TAGGAGGGATGTTGAGGAGC	CCTAGCTTGCTTCCCCTTCT	59.6	232	74	transposon protein putative Ac/Ds sub-class
OsISM02107	LOC_Os01g14260	7993381	CCTGGTAGAAACGCCGATAA	CTCGTTACCAGTTGGAGAAGG	59.5	168	91	transposon protein putative Ac/Ds sub-class
OsISM02108	LOC_Os01g14260	7993717	TTCCACAAGTCCATTGATGC	TATTTCGCGTACCACCTTCC	59.7	178	82	transposon protein putative Ac/Ds sub-class
OsISM02109	LOC_Os01g14260	7994138	GGTGGTGAACTGGTTAAGTGG	GCCTCTAAGTTCTCCCACCA	59.3	231	57	transposon protein putative Ac/Ds sub-class
OsISM02110	LOC_Os01g14260	7994542	GGATGCACGACCTAACCAAT	CGTAGTGCGTCCTAGGGTTC	59.8	142	67	transposon protein putative Ac/Ds sub-class

ISM IDs	Rice gene locus IDs	Physical positions (bp) on chromosomes	Forward primers (5'-3')	Reverse primers (5'-3')	Melting tempearature (T˚C)	Expected product size (bp) in PCR	Expected product size (bp) in Q-PCR	Putative Functions
OsISM02111	LOC_Os01g14260	7994815	GCACTAGCAAGAAGGATGGC	GGCGAACATAGACCACCATT	59.9	154	64	transposon protein putative Ac/Ds sub-class
OsISM02112	LOC_Os01g14260	7995631	TAGGGGTCACACCCAACAAT	CCATGATCCTGGTCCTGAGT	60.0	176	91	transposon protein putative Ac/Ds sub-class
OsISM02113	LOC_Os01g14290	8006355	AGCTCTTCCTCTCACGGAGC	TACCGAAGATCCCGGCTAC	60.6	118	65	transposon protein putative unclassified
OsISM02114	LOC_Os01g14290	8006676	TACTCCCAGGGGCACATTAG	GCTTCCATTTTCGTGTGTTG	59.6	508	95	transposon protein putative unclassified
OsISM02115	LOC_Os01g14290	8007190	CCTTGAGCGTCTCAACTGGT	GGGTTCTCCATGTCTACCCA	60.1	286	86	transposon protein putative unclassified
OsISM02116	LOC_Os01g14290	8007521	GGACTACCTAAGGCGGAAGG	TCCATCGAGTAAATCCACCC	59.9	693	96	transposon protein putative unclassified
OsISM02117	LOC_Os01g14300	8011187	ATTGGGTTTCTCGAATGGC	TCAGCGTACATCCATTGCC	60.3	1017	99	transposon protein putative CACTA En/Spm sub-class
OsISM02118	LOC_Os01g14330	8030651	AAGGAAGCCAATGAAACAGG	AAAGGCACCAAACAGTGTCC	59.6	183	77	conserved hypothetical protein
OsISM02119	LOC_Os01g14410	8061080	GTGGTGTCGTCTCCGCTG	GTCTCCTCCTTCGTCGGCT	61.7	193	98	early light-induced protein chloroplast precursor putative expressed
OsISM02120	LOC_Os01g14440	8083732	GAGGAGAAGAGCCGGAGC	CTGGTTCTCCTCGTTCATGC	60.5	286	73	OsWRKY1v2 - Superfamily of TFs having WRKY and zinc finger domains expressed
OsISM02121	LOC_Os01g14440	8084100	CTCAAATGATGCAGCCTCC	GCCCAGATCGAGGAATTG	59.2	212	99	OsWRKY1v2 - Superfamily of TFs having WRKY and zinc finger domains expressed
OsISM02122	LOC_Os01g14440	8084535	ATGCCAACATGAGGAAGGC	CGCCATCTTCTGACCGTACT	60.7	189	99	OsWRKY1v2 - Superfamily of TFs having WRKY and zinc finger domains expressed
OsISM02123	LOC_Os01g14440	8084746	CGTACTACCGCTGCACCAT	ACGTGGTGATCAGGATGGAC	60.3	239	88	OsWRKY1v2 - Superfamily of TFs having WRKY and zinc finger domains expressed
OsISM02124	LOC_Os01g14550	8159016	TCGTCAACAAGCTCTTCGAG	TCTCTGGACCTTCTCCTCCA	59.6	201	72	pathogen-related protein putative expressed
OsISM02125	LOC_Os01g14550	8159256	CGACCAGAGGACCAGAAGAG	CCATCAAATCCTTCCGATTC	59.6	187	86	pathogen-related protein putative expressed
OsISM02126	LOC_Os01g14550	8159684	GTTTTTCGGCATTTGCATTT	AATTCCGCCTTCTCAACCTT	60.0	498	58	pathogen-related protein putative expressed
OsISM02127	LOC_Os01g14580	8169503	CCATGGCGTTCGAGAAGAT	TGATCCATTTCCAAATAACTCG	60.1	928	85	dehydrogenase putative expressed
OsISM02128	LOC_Os01g14580	8170409	CGAGTTATTTGGAAATGGATCA	TCCAAGTCCAAGAAAGGGAA	59.3	250	54	dehydrogenase putative expressed

ISM IDs	Rice gene locus IDs	Physical positions (bp) on chromosomes	Forward primers (5'-3')	Reverse primers (5'-3')	Melting tempearature (T˚C)	Expected product size (bp) in PCR	Expected product size (bp) in Q-PCR	Putative Functions
OsISM02129	LOC_Os01g14580	8170693	GACGCTACTGGGGACAAAGT	TGCTGAACATCACCTGGAGT	59.2	318	99	dehydrogenase putative expressed
OsISM02130	LOC_Os01g14580	8170966	TGTAGCCATCAAATGTGCAA	TGTCCCATTTGGACTCTTCC	59.3	202	89	dehydrogenase putative expressed
OsISM02131	LOC_Os01g14580	8171140	TGCAATGTGGAAGAGTCCAA	TTGCAGATGATTGGTTCTCG	60.0	189	76	dehydrogenase putative expressed
OsISM02132	LOC_Os01g14580	8171324	TGCAAGAATATTCCTCGGCT	CAAATGCATGTCGTCCAATG	60.4	139	65	dehydrogenase putative expressed
OsISM02133	LOC_Os01g14580	8171495	AAAGGTCCTGGGAAGTTGAA	GACAAGGCTACTCCACCAGC	59.2	1251	96	dehydrogenase putative expressed
OsISM02134	LOC_Os01g14580	8172726	GCTGGTGGAGTAGCCTTGTC	TCATGGAAGCTTCAGCGAAT	60.4	175	68	dehydrogenase putative expressed
OsISM02135	LOC_Os01g14580	8172938	CACCAAAAACACGATCCTCA	TGCATCATCAAACTTGGCTC	59.7	179	95	dehydrogenase putative expressed
OsISM02136	LOC_Os01g14580	8173100	CCAAGTTTGATGATGCAGGA	TAAGGGCATAGGCCACCATA	60.0	154	67	dehydrogenase putative expressed
OsISM02137	LOC_Os01g14580	8173293	ATGGAGATGTGCAGAGCGAT	TTCAGCAGGGAGAACACTCA	60.2	183	87	dehydrogenase putative expressed
OsISM02138	LOC_Os01g14580	8173417	CAGGTTTTGGATCGCTAGGT	AATGGCGTGTGACTGTACCA	59.6	281	97	dehydrogenase putative expressed
OsISM02139	LOC_Os01g14580	8173756	TTGGACAACCGGACTAGGAC	CACTGTTCCCACACAAGCAG	60.2	422	95	dehydrogenase putative expressed
OsISM02140	LOC_Os01g14580	8174204	AGCTCTTCTTGTACATGGGC	CAGCAACAGCATCGATGAAC	58.7	154	75	dehydrogenase putative expressed
OsISM02142	LOC_Os01g14700	8228391	GGCAGAAGCTGCAAGAAAGA	GCACTTCTCCTCGTCGATGT	60.6	171	82	conserved hypothetical protein
OsISM02144	LOC_Os01g14710	8232119	GCAGAGCATGCAAGAGTGAG	ACTTCTCCGCATCAATGTCC	60.0	337	79	conserved hypothetical protein
OsISM02145	LOC_Os01g14730	8238002	GCCTACGTCTTCACCGACC	AGACGTAGACGCCCAACAAC	60.4	189	75	retrotransposon protein putative unclassified
OsISM02147	LOC_Os01g14780	8267826	CCCAAATCCAGGTCAAGTGT	TGGACTGGACTTGGTAGAATGA	59.7	134	99	hypothetical protein
OsISM02148	LOC_Os01g14790	8271010	ACTGCTGGCGTCTTCTCTGT	CCACCTGAGACTCCTTGGAA	60.2	200	97	expressed protein
OsISM02149	LOC_Os01g14810	8274830	TACCTAGGGGAGAGCACCAA	TCCTCTATAGGGCCATGCAA	60.1	359	99	5-nucleotidase domain-containing protein putative expressed

ISM IDs	Rice gene locus IDs	Physical positions (bp) on chromosomes	Forward primers (5'-3')	Reverse primers (5'-3')	Melting tempearature (T˚C)	Expected product size (bp) in PCR	Expected product size (bp) in Q-PCR	Putative Functions
OsISM02150	LOC_Os01g14810	8275203	AAGCGAGAGAAGCTGAATCG	CACGAGGAGAGTGTCTTACGG	59.9	1016	73	5-nucleotidase domain-containing protein putative expressed
OsISM02151	LOC_Os01g14810	8276248	CGATCCATCAGTGTCATTGG	CCATAATCATAAGCACGCCC	60.1	326	87	5-nucleotidase domain-containing protein putative expressed
OsISM02152	LOC_Os01g14810	8276591	GCATGGGTTTCCCTGTAGAT	TCAGCTTTGACCAAATTCCC	59.5	183	95	5-nucleotidase domain-containing protein putative expressed
OsISM02153	LOC_Os01g14810	8276805	GGCACCCAGATGCTTTCTAC	TTTCTTTCCGTAGATCCACCA	59.6	154	71	5-nucleotidase domain-containing protein putative expressed
OsISM02154	LOC_Os01g14810	8276980	CCCTTTTCTCTGTGTCAGAAGC	TAATCCAGTGGCCCAAGTTC	60.2	884	92	5-nucleotidase domain-containing protein putative expressed
OsISM02155	LOC_Os01g14810	8277851	GGCCACTGGATTACAAAGGA	TACATGCGCTCGAAAAAGTG	60.0	136	60	5-nucleotidase domain-containing protein putative expressed
OsISM02156	LOC_Os01g14810	8277967	CACTTTTTCGAGCGCATGTA	TCTACAAATCGTTCCGGCTC	60.1	161	68	5-nucleotidase domain-containing protein putative expressed
OsISM02157	LOC_Os01g14810	8278143	GCCTTTAGCTCTTCTGGATCA	GCATGATTCATCATTTTGTTCG	59.5	197	94	5-nucleotidase domain-containing protein putative expressed
OsISM02158	LOC_Os01g14810	8278336	ATGCATTCAATCGCTTCCTT	AACTCTGGCTTCCTTGCAGA	59.9	189	83	5-nucleotidase domain-containing protein putative expressed
OsISM02159	LOC_Os01g14810	8278565	GATGGATTAATGCGTCCCTG	GCACTGCCACCAGAGTACAA	60.1	1034	60	5-nucleotidase domain-containing protein putative expressed
OsISM02160	LOC_Os01g14810	8279689	TCATTTACGATGGAGGACAGC	TCTTTCTGAACATGGCTTCG	59.5	749	82	5-nucleotidase domain-containing protein putative expressed
OsISM02161	LOC_Os01g14810	8280480	TCTTTTTAACCAGCTTCGGC	TCTGTAAGCTCCTGATCATCCAT	59.8	327	97	5-nucleotidase domain-containing protein putative expressed
OsISM02162	LOC_Os01g14810	8280855	TGCACCAATGCTAGAATCAGA	ACTCTTATCCCATAGGCCCG	59.9	1049	80	5-nucleotidase domain-containing protein putative expressed
OsISM02163	LOC_Os01g14810	8281864	ATGGGGTTGGCTATCACGTA	GAAAATTTGAAACCCTTGATGTG	60.0	190	99	5-nucleotidase domain-containing protein putative expressed
OsISM02164	LOC_Os01g14810	8282063	CTCCATTCATGTATTTCCAATC A	CGAATGTCAGTGTTGTTGCC	59.5	460	84	5-nucleotidase domain-containing protein putative expressed
OsISM02166	LOC_Os01g14820	8284738	AATCTGAAGAAGGGGGATCG	CCAGGCCAAGATGCTTCTAA	60.4	335	84	expressed protein
OsISM02167	LOC_Os01g14820	8285361	ACCTCATGTTTCGTTCACCG	TGAGCAATCCCCAAAAGAAC	60.5	534	62	expressed protein
OsISM02168	LOC_Os01g14820	8286130	CTCGACGCCCTGATTTAGAG	CAACACGAAAGCTGAAGGGT	60.1	1297	94	expressed protein

ISM IDs	Rice gene locus IDs	Physical positions (bp) on chromosomes	Forward primers (5'-3')	Reverse primers (5'-3')	Melting tempearature (T˚C)	Expected product size (bp) in PCR	Expected product size (bp) in Q-PCR	Putative Functions
OsISM02169	LOC_Os01g14830	8290735	AGAAGGAGGGGTTCTTCGC	AGGGACTCCTTGTGCTCTGA	60.4	951	99	ribosomal protein L3 putative expressed
OsISM02170	LOC_Os01g14830	8291791	ACTGGAATCACAAAGGGCAA	ATGCAGGCATCCCACTAAAA	60.5	1123	68	ribosomal protein L3 putative expressed
OsISM02171	LOC_Os01g14830	8292927	TCTATCACATCGGAGCATCG	CCATACGTCCAGGCATCTTT	59.9	1533	84	ribosomal protein L3 putative expressed
OsISM02172	LOC_Os01g14830	8294471	CGCACCGTGAAAAATGTATG	AAACAAAGCTGCCTTGAGGA	60.0	189	95	ribosomal protein L3 putative expressed
OsISM02173	LOC_Os01g14840	8296934	CTGCTGCCTCTGCATCAC	AGAAATCGGGGATCACCTTC	59.7	205	81	ZOS1-06 - C2H2 zinc finger protein expressed
OsISM02174	LOC_Os01g14840	8297396	TGTGACCTTTGCTTGGAAGG	CCTCCGAATCACCAGATTTT	60.1	1655	98	ZOS1-06 - C2H2 zinc finger protein expressed
OsISM02175	LOC_Os01g14840	8299153	CAAGAGAACATTTTTCCTGCC	GCATACCTCTAAGTCATCATAGT TCC	58.8	164	87	ZOS1-06 - C2H2 zinc finger protein expressed
OsISM02176	LOC_Os01g14840	8299258	ACAGGCAACATTCTGGACAA	CAAGCACCCTTTATCCTCACA	59.6	413	99	ZOS1-06 - C2H2 zinc finger protein expressed
OsISM02177	LOC_Os01g14840	8299652	TGAGGATAAAGGGTGCTTGG	CCCGTGTTCCACACCATTA	60.1	192	83	ZOS1-06 - C2H2 zinc finger protein expressed
OsISM02178	LOC_Os01g14860	8330078	AGCATGGGTGCTGAGAAGTT	TCTGTTCCCTTGCCGTTAAT	59.7	189	96	CGMC GSK.1 - CGMC includes CDA MAPK GSK3 and CLKC kinases expressed
OsISM02179	LOC_Os01g14860	8330264	AGAAACCGGCCACATAATTG	CCTTGCCCTACAATACGCTC	59.8	219	94	CGMC GSK.1 - CGMC includes CDA MAPK GSK3 and CLKC kinases expressed
OsISM02180	LOC_Os01g14860	8330463	GAGCGTATTGTAGGGCAAGG	GCAACTGTCTCACCTGTCTCC	59.8	272	72	CGMC GSK.1 - CGMC includes CDA MAPK GSK3 and CLKC kinases expressed
OsISM02181	LOC_Os01g14860	8330930	GAACCAGCGTATGCCACTTA	GTGGCAAACTCCGATGCTAT	59.5	225	92	CGMC GSK.1 - CGMC includes CDA MAPK GSK3 and CLKC kinases expressed
OsISM02182	LOC_Os01g14860	8331135	ATAGCATCGGAGTTTGCCAC	TTTGCACTCCCAAAGTCACA	60.2	184	95	CGMC GSK.1 - CGMC includes CDA MAPK GSK3 and CLKC kinases expressed
OsISM02183	LOC_Os01g14860	8331299	TGTGACTTTGGGAGTGCAAA	TCGGGAGCAAATGTACGATA	59.7	149	64	CGMC GSK.1 - CGMC includes CDA MAPK GSK3 and CLKC kinases expressed
OsISM02184	LOC_Os01g14860	8331511	TCTGCTGGATGTGTTCTTGC	CCACTTTCACCAGGAAACAG	59.1	168	60	CGMC GSK.1 - CGMC includes CDA MAPK GSK3 and CLKC kinases expressed
OsISM02185	LOC_Os01g14860	8331648	TTCGACAGCCTCTGTTTCCT	CCTCCCTTGTAGGTGTTCCA	60.0	172	82	CGMC GSK.1 - CGMC includes CDA MAPK GSK3 and CLKC kinases expressed
OsISM02186	LOC_Os01g14860	8331860	TCCACAGATTAAAGCACACCC	AGCTTCTGGAGGCAACCTTT	60.2	144	59	CGMC GSK.1 - CGMC includes CDA MAPK GSK3 and CLKC kinases expressed

ISM IDs	Rice gene locus IDs	Physical positions (bp) on chromosomes	Forward primers (5'-3')	Reverse primers (5'-3')	Melting tempearature (T˚C)	Expected product size (bp) in PCR	Expected product size (bp) in Q-PCR	Putative Functions
OsISM02187	LOC_Os01g14860	8332024	GCTCCAGTACTCACCCAACC	CGAGCATTAGGGTCTCGAAG	59.8	200	85	CGMC GSK.1 - CGMC includes CDA MAPK GSK3 and CLKC kinases expressed
OsISM02188	LOC_Os01g14860	8332237	CGCTTTTTGCCTCCTCTCTT	CTGGGATCAATTTCGCCATA	60.9	259	80	CGMC GSK.1 - CGMC includes CDA MAPK GSK3 and CLKC kinases expressed
OsISM02190	LOC_Os01g14870	8333875	CGTCTGCCAACACTTCGTC	ACATGAATCCCCAAACTTGC	60.1	152	50	zinc finger matrin-type protein 5 putative expressed
OsISM02191	LOC_Os01g14870	8334049	CTCCTGCTAATCCAGGACCA	ATTAGACTGCTGCGCCATTG	60.8	1109	63	zinc finger matrin-type protein 5 putative expressed
OsISM02192	LOC_Os01g14870	8335189	ATCAGGCAGCAGATGGAAGT	TGAGGTGTGACCCCCTAGAA	60.2	1162	54	zinc finger matrin-type protein 5 putative expressed
OsISM02193	LOC_Os01g14890	8342781	GTTTGGAGCGAGGCACAAC	GTGCTCAGCTCCGGTACAGT	61.1	617	64	expressed protein
OsISM02194	LOC_Os01g14890	8343733	GCCTCCAGCTCTACCACAAC	ACGGTGGAGTAGAACGCTCC	60.7	200	92	expressed protein
OsISM02196	LOC_Os01g14890	8344302	CTCGCCGAGATCCCACTG	GGTAGCTCCAGCAGTCGAAC	61.5	179	44	expressed protein
OsISM02197	LOC_Os01g14890	8344454	GCAGGTTGTTCGACTGCTG	AGTAGCACGTCGTCGTCCAG	61.0	165	85	expressed protein
OsISM02198	LOC_Os01g14900	8347657	GGTGCACTACCTCTTCTCGC	TGTCAGCCTCAGTCGCAGT	60.4	1284	60	glycerol-3-phosphate acyltransferase putative expressed
OsISM02199	LOC_Os01g14910	8353481	TCGTGTGGAGGTCGATGTTA	CTTGACCTCTGCCGTGTCTC	60.6	484	92	hypothetical protein
OsISM02201	LOC_Os01g14950	8382424	ACAGTTCCGCAAGTTGCTTT	AGGAACAACGCCTGATTGAA	60.3	169	71	importin subunit alpha putative expressed
OsISM02202	LOC_Os01g14950	8382572	ATTCAATCAGGCGTTGTTCC	GCAATGTTTGTAAGTGCCCA	59.8	189	96	importin subunit alpha putative expressed
OsISM02203	LOC_Os01g14950	8382790	TCATTGATCATGGGGCTGTA	ACATTGCCCAATGCCCATAC	61.3	208	92	importin subunit alpha putative expressed
OsISM02204	LOC_Os01g14950	8383130	CAGAGGAAAACCACAGCCAT	AGTGAATAAGCCGTGCCAGT	59.9	154	69	importin subunit alpha putative expressed
OsISM02205	LOC_Os01g14950	8383365	TGATTGAAGCTGGTGTTTGC	TTCCAACAGTTCGTAGTGCG	59.9	196	88	importin subunit alpha putative expressed
OsISM02206	LOC_Os01g14950	8383524	CCTTCAGTGCTTATACCCGC	CAAGGGAGGGCTTGATGAT	59.9	989	96	importin subunit alpha putative expressed
OsISM02207	LOC_Os01g14950	8384548	GCATCAAGAAAGAGGCTTGC	CCAGAGGACCAATAATGCCA	60.5	588	97	importin subunit alpha putative expressed

ISM IDs	Rice gene locus IDs	Physical positions (bp) on chromosomes	Forward primers (5'-3')	Reverse primers (5'-3')	Melting tempearature (T˚C)	Expected product size (bp) in PCR	Expected product size (bp) in Q-PCR	Putative Functions
OsISM02208	LOC_Os01g14950	8385193	CAAATGCTACTTCAGGCGGT	ATCACACAATGGCTTGATGC	59.9	195	75	importin subunit alpha putative expressed
OsISM02209	LOC_Os01g15000	8404163	TGCAACATCGCCTACATGAT	GATGAAGTCACAAACCGCAA	59.9	718	75	lipase putative expressed
OsISM02210	LOC_Os01g15000	8405309	ATGATGATCAAGGCAGCACA	TCAAATAGGAGGTTGGCCTG	60.1	169	80	lipase putative expressed
OsISM02211	LOC_Os01g15000	8405458	CAGGCCAACCTCCTATTTGA	TACATTCCCTTGGCTGCTTC	60.1	316	87	lipase putative expressed
OsISM02212	LOC_Os01g15000	8406114	AGTCACCCATGCCTCACAA	TTGTCAGGTTGCAGGATGAA	60.2	512	72	lipase putative expressed
OsISM02213	LOC_Os01g15080	8456966	CTCCGACAACAAGCTACTCG	GTTGTCGATGACGATGATGG	59.3	100	57	hypothetical protein
OsISM02214	LOC_Os01g15080	8457129	GATGTCGAACGAGTACGCAA	CCGCATTTGTCGAGAATGTT	60.5	488	93	hypothetical protein
OsISM02215	LOC_Os01g15120	8486109	ACGGCCACTACATCTGCTTC	CTACCAAATTCAGCTTCGGC	60.1	1648	56	hydrolase alpha/beta fold family domain containing protein expressed
OsISM02216	LOC_Os01g15120	8487904	TGCCGAGAAATTTGAGAAGA	ACTCCAGTCCATGGTGCAGT	59.6	383	94	hydrolase alpha/beta fold family domain containing protein expressed
OsISM02218	LOC_Os01g15130	8490439	GATCACTACGTGTGCCGCT	GAGCAAATTCAGCTTCAGCC	60.0	259	53	hydrolase alpha/beta fold family domain containing protein expressed
OsISM02219	LOC_Os01g15130	8490868	GGGTTCACAGGAGGATTCAA	CGGAACATTTATCAGAGCCC	59.7	169	88	hydrolase alpha/beta fold family domain containing protein expressed
OsISM02220	LOC_Os01g15170	8504401	CTAGAGCAGGGCCATAAGGG	CAATGAGCTGGAGCCAAACT	60.7	184	98	hypothetical protein
OsISM02221	LOC_Os01g15180	8508674	CGGTGTTTTCATGGCTGATA	GACGCAGGTCACTTGTGAAA	59.7	716	99	retrotransposon protein putative Ty3-gypsy subclass
OsISM02222	LOC_Os01g15180	8509803	CTCTTAGTCGGCAAATTCGG	TATTGCTCTGCGAACTGCTG	60.1	346	99	retrotransposon protein putative Ty3-gypsy subclass
OsISM02223	LOC_Os01g15180	8510995	TCCCTCTGATCCTTCCAAAA	GGCGATCAGATCATCCTTCT	59.4	648	95	retrotransposon protein putative Ty3-gypsy subclass
OsISM02224	LOC_Os01g15260	8539975	CCGACATGAAGGAGGAGATG	GTCCTTCTCCATGGTGTGCT	60.4	187	78	dynein light chain type 1 domain containing protein expressed
OsISM02225	LOC_Os01g15260	8540205	CTACCTGGCACTGCATCGT	GCAAGACGGCTTTAGAATCG	59.9	2979	97	dynein light chain type 1 domain containing protein expressed
OsISM02226	LOC_Os01g15370	8611724	GGACAAGAGATAGGGGAGGC	ATTCTTCTCCTGCGAAAACG	59.7	2669	90	retrotransposon protein putative Ty3-gypsy subclass

ISM IDs	Rice gene locus IDs	Physical positions (bp) on chromosomes	Forward primers (5'-3')	Reverse primers (5'-3')	Melting tempearature (T˚C)	Expected product size (bp) in PCR	Expected product size (bp) in Q-PCR	Putative Functions
OsISM02227	LOC_Os01g15448	8656657	ATGCTGCTTGAGGTTTGGAT	ATTTCCCAGACAATGGCACT	59.6	466	60	expressed protein
OsISM02228	LOC_Os01g15470	8682302	ATGAGGAACCAAGAAGCGG	ACCCTTCAGTCTTCAGTGGG	59.7	760	82	protein kinase putative expressed
OsISM02229	LOC_Os01g15470	8683178	GGAGGATTCGGCAGTGTGTA	TTCCCTGAAGCCCATTTAGA	60.4	333	86	protein kinase putative expressed
OsISM02230	LOC_Os01g15470	8683631	TGGGATCACTAGAAGACCGC	TTCATCCGTGTTGTCCAATC	59.8	1034	71	protein kinase putative expressed
OsISM02231	LOC_Os01g15470	8684979	GGCCACCTGATGAACAAGAC	TGGTCTTTGAACAATGGACG	60.0	654	53	protein kinase putative expressed
OsISM02233	LOC_Os01g15520	8716805	CGACACCGCTCCTCAAAA	TTCTCCTCAAATGCTGGTCC	60.6	964	84	expressed protein
OsISM02234	LOC_Os01g15520	8717771	GCGAAGAGGCTATGCTTGAC	GACCATGAAAATCAGACGCA	59.9	185	97	expressed protein
OsISM02235	LOC_Os01g15520	8717977	ACCGTGATGGAAAATTGAGC	ACAGTTGCATCTGGTTGCTG	59.9	707	89	expressed protein
OsISM02236	LOC_Os01g15520	8718646	CTTGTCAGCTTTGCTGCTCA	AACTCGACGTGCAATCTTCC	60.4	346	91	expressed protein
OsISM02237	LOC_Os01g15520	8719007	CCTGACCCTGAAGAACTGGA	CCGAGACCTCGTCTTCTTTG	60.1	640	91	expressed protein
OsISM02238	LOC_Os01g15600	8758751	TCGACTACTACATCCGCACC	GCCTCTCTTGTTCTCCCCA	59.5	198	99	PUR ALPHA-1 putative expressed
OsISM02239	LOC_Os01g15600	8758930	TGGGGAGAACAAGAGAGGC	AACTACCAGCCGGAACAATG	60.2	2640	87	PUR ALPHA-1 putative expressed
OsISM02240	LOC_Os01g15600	8761622	TGAGGCTTCCCGACTTTATG	ATCCAGCACCAACATCATCA	60.1	185	99	PUR ALPHA-1 putative expressed
OsISM02241	LOC_Os01g15600	8761954	GAAGCAACAACAGGGGTCAT	GGCAAGATTATCGACGAACG	60.3	159	71	PUR ALPHA-1 putative expressed
OsISM02242	LOC_Os01g15610	8769916	GTCTTCGCTTTGCTCCTCCT	TTGCACTTGATGCTTGTTGA	60.0	323	73	conserved hypothetical protein
OsISM02243	LOC_Os01g15610	8770219	TCAACAAGCATCAAGTGCAA	CAGCATTTCGTCGTCTTCCT	59.7	1941	78	conserved hypothetical protein
OsISM02244	LOC_Os01g15620	8776532	CTAGCAAGGCTTGGTTGCTC	AACCACAACAGGTACGCCTC	60.1	184	98	transposon protein putative unclassified
OsISM02245	LOC_Os01g15620	8777027	CGATGGGGACTTACTGGTGT	CTCACCTGGACGTGAGCATA	59.8	146	53	transposon protein putative unclassified

ISM IDs	Rice gene locus IDs	Physical positions (bp) on chromosomes	Forward primers (5'-3')	Reverse primers (5'-3')	Melting tempearature (T˚C)	Expected product size (bp) in PCR	Expected product size (bp) in Q-PCR	Putative Functions
OsISM02246	LOC_Os01g15620	8777386	CGGACTTAGGCAGGTGTTTC	CTAGCTGTCCCCGTCGAGT	60.1	159	81	transposon protein putative unclassified
OsISM02247	LOC_Os01g15620	8777753	GCACCGTGACCGAGACTACT	ATATCGGCACTGATATCCCG	60.0	152	58	transposon protein putative unclassified
OsISM02248	LOC_Os01g15620	8778030	TGGACATCGTACCTCCAGC	GAACTGTACCGTGCGAAACC	60.1	258	99	transposon protein putative unclassified
OsISM02249	LOC_Os01g15620	8778246	GAGCAGTGCGACCTACAGC	GCATTGGAAGAATCTCCTGC	59.8	378	91	transposon protein putative unclassified
OsISM02250	LOC_Os01g15620	8778604	GCAGGAGATTCTTCCAATGC	TGGGTTCTCCATGTCTACCC	59.8	162	84	transposon protein putative unclassified
OsISM02251	LOC_Os01g15620	8778838	GAATGTCGAATTGCTGCTGA	TCGAGTAAATCCACCCATCC	59.9	661	64	transposon protein putative unclassified
OsISM02252	LOC_Os01g15630	8787649	TCAACAAGCTCAAGTGCCAT	GGAAGAATTGCTTGTGGTGG	60.0	1907	99	zinc finger C3HC4 type domain containing protein expressed
OsISM02253	LOC_Os01g15630	8789524	CAGCAGGTAGATCCACCACA	TCTTCGAATCTGAAGCCACA	59.6	218	91	zinc finger C3HC4 type domain containing protein expressed
OsISM02254	LOC_Os01g15660	8808475	CACCGTTTAATCCTCGCTTC	AGGCGCTGGGAGACGTAG	60.6	206	82	NAD dependent epimerase/dehydratase family protein putative expressed
OsISM02255	LOC_Os01g15660	8808752	CGCCGTACGTTTCTCCTAAC	CAAGAGTTGTGCATCGAACC	59.5	428	86	NAD dependent epimerase/dehydratase family protein putative expressed
OsISM02256	LOC_Os01g15660	8809135	CCGGTTCTTGTTCTTGTTCC	TCTTGCAAAGACCGGAGATT	59.7	194	96	NAD dependent epimerase/dehydratase family protein putative expressed
OsISM02257	LOC_Os01g15660	8809342	CTCATCTCCATTCCACCCAT	ATGCATTGTTTGCCATGAAA	59.8	847	63	NAD dependent epimerase/dehydratase family protein putative expressed
OsISM02258	LOC_Os01g15660	8810172	CATGGCAAACAATGCATCA	CACCATACACACCTGAAAAATGA	59.9	178	99	NAD dependent epimerase/dehydratase family protein putative expressed
OsISM02259	LOC_Os01g15660	8810361	GCATGGGTTGATGAGGAGTAA	CACCGTATTGGTTGAAATCAGA	59.9	403	90	NAD dependent epimerase/dehydratase family protein putative expressed
OsISM02260	LOC_Os01g15660	8810815	TTTTTCATGGGAGGCTTTTG	GGTTACTGGGATTACAAAGGGA	59.6	633	74	NAD dependent epimerase/dehydratase family protein putative expressed
OsISM02261	LOC_Os01g15700	8828572	CTATGGCTGCCAAGACCAAC	GATTAACGAACCCACCAGGA	60.2	1074	85	transposon protein putative Pong sub-class
OsISM02262	LOC_Os01g15700	8829744	CGCTTATTATGGACGAACGA	CATTTGAGTTGTTCAGCCAGG	59.7	157	62	transposon protein putative Pong sub-class
OsISM02263	LOC_Os01g15700	8829984	CGGGTTAGGCAAGTTAAGCA	CGTTGTTAGAACCAGCGACA	60.1	296	86	transposon protein putative Pong sub-class

ISM IDs	Rice gene locus IDs	Physical positions (bp) on chromosomes	Forward primers (5'-3')	Reverse primers (5'-3')	Melting tempearature (T˚C)	Expected product size (bp) in PCR	Expected product size (bp) in Q-PCR	Putative Functions
OsISM02264	LOC_Os01g15700	8830563	GAAACGGAAGAGTGTTGGGA	CTTTGAGAAGTGCAGCACCA	60.1	421	75	transposon protein putative Pong sub-class
OsISM02265	LOC_Os01g15700	8830957	CAAATCATGGTGCTGCACTT	TCCTCAACGATCCCTTCATC	59.9	1429	62	transposon protein putative Pong sub-class
OsISM02266	LOC_Os01g15700	8832366	GATGAAGGGATCGTTGAGGA	CCTCACCAGTGAGCAGGTCT	60.2	690	62	transposon protein putative Pong sub-class
OsISM02268	LOC_Os01g15720	8840001	CCAACTGGAAGATTCCTAAGGTT	TGAAGGTAGTGATATTGATGTTTAGC	58.9	190	95	tobamovirus movement protein containing protein
OsISM02269	LOC_Os01g15720	8842029	CATTGCCCGATAGAGATGGT	TCTTGATTAGGTTCTGGTAGGG	58.6	227	98	tobamovirus movement protein containing protein
OsISM02270	LOC_Os01g15720	8842280	TGGTTTACCTCCTTTATTTGCTG	GCAATTGACATCAGCACCAG	59.9	1099	65	tobamovirus movement protein containing protein
OsISM02271	LOC_Os01g15720	8843729	TGACAATTCTTTCCCAAAATCT	TCCTTGTGATCTTGTTTTGGA	58.0	1524	99	tobamovirus movement protein containing protein
OsISM02272	LOC_Os01g15740	8852856	CCACCAGGCTACTGATTTGC	GCCTCTGCCGCTCCTATT	60.6	1071	83	expressed protein
OsISM02273	LOC_Os01g15740	8853976	CGGAGCATTCTCCCTGTAAA	GGTAGACGCAGAGCATGGA	60.1	572	63	expressed protein
OsISM02275	LOC_Os01g15750	8863844	GATCTGGGAGATGGCTACGA	GAGCCCAGTCCCATCTGA	59.9	198	95	retrotransposon protein putative unclassified
OsISM02276	LOC_Os01g15750	8864496	CCCTCCAAGGTGGTCATTTA	GGTCCTCGGCCTCAGACT	60.1	189	93	retrotransposon protein putative unclassified
OsISM02278	LOC_Os01g15770	8882884	ACCACCTCGTCAGCATCGT	AGATCTCCGTGACGAACAGG	61.0	183	91	transmembrane protein 136 putative expressed
OsISM02279	LOC_Os01g15770	8883111	ATCAAGGACACCGACCTCAA	GTACGTGAGGTATGGCCCAA	60.6	738	91	transmembrane protein 136 putative expressed
OsISM02280	LOC_Os01g15770	8883851	CACCGTTACGGCAGACAAC	CCTCAGGAACCAGTAGGCAC	59.9	273	80	transmembrane protein 136 putative expressed
OsISM02281	LOC_Os01g15790	8892221	TGCGAGTGTCATTCATTCGT	TCGGAACAACAACGATAGCA	60.3	608	84	expressed protein
OsISM02282	LOC_Os01g15790	8892843	ACCTTCGACAACAATAGCCG	TTCAAAGTTCTTCCCGATTAGA	59.1	157	58	expressed protein
OsISM02283	LOC_Os01g15790	8892970	CAGGCAGATCTAATCGGGAA	TGCAGAACTGGACAACCAAA	60.2	204	77	expressed protein
OsISM02284	LOC_Os01g15790	8893155	TTGGTTGTCCAGTTCTGCAA	TGATAAAACGTCCAAAGTCCG	60.1	529	71	expressed protein

ISM IDs	Rice gene locus IDs	Physical positions (bp) on chromosomes	Forward primers (5'-3')	Reverse primers (5'-3')	Melting tempearature (T°C)	Expected product size (bp) in PCR	Expected product size (bp) in Q-PCR	Putative Functions
OsISM02285	LOC_Os01g15790	8893663	CGGACTTTGGACGTTTTATCA	TTCACAGTGCACTAGATCTCCA	59.3	1118	99	expressed protein
OsISM02286	LOC_Os01g15790	8894784	ACTTTTGAGAAGATGGGCCG	CTTTATTTGGGCATTGACCG	60.9	167	62	expressed protein
OsISM02287	LOC_Os01g15790	8894931	CGGTCAATGCCCAAATAAAG	CTGGTTACCTGCACATCAGTTC	60.0	228	84	expressed protein
OsISM02288	LOC_Os01g15790	8895092	TTTATTATAGGCAAACAAGGA TGC	GGACATGGGATAGCAAGTTCA	59.3	837	87	expressed protein
OsISM02289	LOC_Os01g15790	8895954	GGTTTCACTGTCGGATTCCA	TGAGATTGCTCAAATCCCAG	59.9	165	78	expressed protein
OsISM02290	LOC_Os01g15790	8896100	TGGGATTTGAGCAATCTCATC	CATGACTCTGCTCACCCTGA	60.0	844	52	expressed protein
OsISM02291	LOC_Os01g15790	8896976	TCAGGACTTGTTGGAAAACCT T	AGTAGGCATAGCACCGTTGG	60.1	332	94	expressed protein
OsISM02292	LOC_Os01g15790	8897287	ACCAACGGTGCTATGCCTAC	ACCTCATAAGGACTGCTCCG	59.7	165	87	expressed protein
OsISM02293	LOC_Os01g15790	8897483	CCAATTGAATTTACGCTCACC	ATAATCCCAAATGTTGCCCA	59.7	560	87	expressed protein
OsISM02294	LOC_Os01g15790	8898047	TGCATGTATGTGTGCCTTGA	CGTGTTTTGTAGATGAAACCTCC	59.8	316	99	expressed protein
OsISM02295	LOC_Os01g15790	8898301	GCATGCAGCTTCATTGTGTT	CAGGCAGCGCATATAAACC	59.6	184	95	expressed protein
OsISM02296	LOC_Os01g15790	8898464	CTGGTTTATATGCGCTGCCT	GCCATGATCATCTGCTCTCA	60.1	207	90	expressed protein
OsISM02297	LOC_Os01g15800	8900011	GCCATTCCGATCCCTCTTAG	CAGAGATGGTCGATGAAGCA	60.4	231	82	conserved hypothetical protein
OsISM02298	LOC_Os01g15800	8900222	TGCTTCATCGACCATCTCTG	CGAGCTTCCACATCACCAG	60.2	218	92	conserved hypothetical protein
OsISM02299	LOC_Os01g15830	8917054	CTTGGTCAGGCTGCATTTTC	TCACTGATGATGGTGGTGCT	60.4	741	85	peroxidase precursor putative expressed
OsISM02300	LOC_Os01g15830	8917899	ACATACTCGCCCTCGCTG	AAGTGGCACATCCCAGTACG	60.5	246	66	peroxidase precursor putative expressed
OsISM02301	LOC_Os01g15830	8918217	CCCACCATCATCACCAAGTT	CGAGACATGCCAATGGTGT	60.6	181	87	peroxidase precursor putative expressed
OsISM02302	LOC_Os01g15850	8924378	GCCACTTCGACGACCTCC	CATGAATTGCCTCTGGCTTT	61.0	1861	86	expressed protein

ISM IDs	Rice gene locus IDs	Physical positions (bp) on chromosomes	Forward primers (5'-3')	Reverse primers (5'-3')	Melting tempearature (T˚C)	Expected product size (bp) in PCR	Expected product size (bp) in Q-PCR	Putative Functions
OsISM02303	LOC_Os01g15850	8926284	ATATGCTTGGAAGCGTCAGC	TCTTTTCTGGTCAGTGAAGGC	59.9	885	92	expressed protein
OsISM02304	LOC_Os01g15850	8927550	GATCCTGATGCTGTGGCTTT	AACATGATGCACCGATAGGC	60.6	177	79	expressed protein
OsISM02305	LOC_Os01g15850	8927708	CCTATCGGTGCATCATGTTTT	AGTTGGTTCCACACTGACAAAG	59.5	172	80	expressed protein
OsISM02306	LOC_Os01g15850	8927830	AGGAGCATGCAGATAAAGCA	AAATAGAGTCTGATAGCTGGCA A	58.0	267	99	expressed protein
OsISM02307	LOC_Os01g15850	8928056	ACTTCTTGCAGCGTTGGATT	GCAGAGCCAAAGACTTGTCC	59.9	199	97	expressed protein
OsISM02308	LOC_Os01g15860	8932656	CGAGGAAAGAAAGCTGTTGG	TCTTGCCATGGTGAGAACAG	59.9	209	77	pre-mRNA-processing factor 6 putative expressed
OsISM02309	LOC_Os01g15880	8939070	CGACACTATGGTCTTCGGTG	TGGTGTACCCCGAGAAGAGA	59.9	761	98	transposon protein putative unclassified
OsISM02310	LOC_Os01g15880	8940289	TTTTCCTCACCGACTCCAAC	GAGCTTGGCGTTCTGGTC	59.8	1137	81	transposon protein putative unclassified
OsISM02312	LOC_Os01g15910	8958710	GACGCTGTCGGAGATGGT	GACGAGGTGCATGAAGGAGT	60.0	1912	74	UTP--glucose-1-phosphate uridylyltransferase putative
OsISM02313	LOC_Os01g15910	8960602	ACTCCTTCATGCACCTCGTC	GTGGGTCGCTCAACTTTGTT	60.2	179	80	UTP--glucose-1-phosphate uridylyltransferase putative
OsISM02314	LOC_Os01g15910	8960799	GTACGACTCACTGGTGCAGG	TCGTCCCAAGTCCTCCATTA	59.9	234	96	UTP--glucose-1-phosphate uridylyltransferase putative
OsISM02315	LOC_Os01g15910	8961012	TTAATGGAGGACTTGGGACG	GGTCAAGAAAGGTAAGGCCG	60.5	906	82	UTP--glucose-1-phosphate uridylyltransferase putative
OsISM02316	LOC_Os01g15910	8961878	TGTACGATTGAAGTCCGCAG	GAAAGTGTGAATTTCAATGTTTG TG	59.9	333	97	UTP--glucose-1-phosphate uridylyltransferase putative
OsISM02317	LOC_Os01g15910	8962174	CGTGGAGAAGTACACAAACAT TG	CTTCCTTCGCTTGAAAGTGG	59.8	200	97	UTP--glucose-1-phosphate uridylyltransferase putative
OsISM02318	LOC_Os01g15910	8962354	CCACTTTCAAGCGAAGGAAG	GAAAAACACATCACCGTGGC	60.7	242	67	UTP--glucose-1-phosphate uridylyltransferase putative
OsISM02319	LOC_Os01g15910	8962594	TCTCATTGTGCAAAAGTGGAA T	CTGTAGCGCCCAAGTTCTCT	59.6	743	91	UTP--glucose-1-phosphate uridylyltransferase putative
OsISM02320	LOC_Os01g15910	8963311	GAGTTCAGAGAACTTGGGCG	TTCTGTGCTGGTTAAGGATTCA	59.9	113	59	UTP--glucose-1-phosphate uridylyltransferase putative
OsISM02321	LOC_Os01g15910	8963402	TGAATCCTTAACCAGCACAGA A	GGGACCTGAAAAATCTCCAA	59.4	422	88	UTP--glucose-1-phosphate uridylyltransferase putative

ISM IDs	Rice gene locus IDs	Physical positions (bp) on chromosomes	Forward primers (5'-3')	Reverse primers (5'-3')	Melting tempearature (T°C)	Expected product size (bp) in PCR	Expected product size (bp) in Q-PCR	Putative Functions
OsISM02322	LOC_Os01g15910	8963804	TTGGAGATTTTTCAGGTCCC	GTTCACCCGCCTATATCCAA	59.4	259	55	UTP--glucose-1-phosphate uridylyltransferase putative
OsISM02323	LOC_Os01g15910	8964079	GGCTGGTAAAAGCTGAAGCA	TGCTGAACCAGCTTCCTTTT	60.3	179	99	UTP--glucose-1-phosphate uridylyltransferase putative
OsISM02324	LOC_Os01g15910	8964239	AAAGGAAGCTGGTTCAGCAA	GAACCTTGAGCGAGGAACAG	60.0	172	68	UTP--glucose-1-phosphate uridylyltransferase putative
OsISM02325	LOC_Os01g15910	8964391	CTGTTCCTCGCTCAAGGTTC	GGGTTACAGTGCCATCCATC	60.1	175	94	UTP--glucose-1-phosphate uridylyltransferase putative
OsISM02326	LOC_Os01g15910	8964595	CCCTTGATTGATCTTGGACC	TTTCAAGCTATCAAGCGCAA	59.5	233	94	UTP--glucose-1-phosphate uridylyltransferase putative
OsISM02327	LOC_Os01g15910	8964829	TCTCTGGGGATGTTTGGTTC	TTAACCCTAACTGGGCAGCA	60.3	350	73	UTP--glucose-1-phosphate uridylyltransferase putative
OsISM02328	LOC_Os01g15910	8965189	TCTTGATGGCAGTGTTTTTGA	ATAGATGCACAGTCCGCTCC	59.8	190	97	UTP--glucose-1-phosphate uridylyltransferase putative
OsISM02329	LOC_Os01g15910	8965474	GGGCAATGTTCCATTCAGA	ACAGCTTTCCTTGCACCAGT	59.7	399	85	UTP--glucose-1-phosphate uridylyltransferase putative
OsISM02330	LOC_Os01g15970	8985458	GCTCTCCCTCTCCCTCCTC	CACCTCTCCCTCATTCCAATC	60.6	209	80	expressed protein
OsISM02332	LOC_Os01g15979	8988027	GAGATCCCCACGAGGTGC	TATCTCGAACCAGTGGAGGC	60.9	493	89	gb protein putative expressed
OsISM02333	LOC_Os01g15979	8988500	GCCTCCACTGGTTCGAGATA	TCCAGCTGTACCCCCAATAG	60.1	351	59	gb protein putative expressed
OsISM02334	LOC_Os01g15979	8988827	TCTGCTATTGGGGGTACAGC	TGGCCCTTTTATGTACCGTC	60.0	659	79	gb protein putative expressed
OsISM02335	LOC_Os01g15979	8989464	TCGACGGTACATAAAAGGGC	TAACCCAGCACATGCAGAAG	59.9	907	86	gb protein putative expressed
OsISM02336	LOC_Os01g15979	8990351	CTTCTGCATGTGCTGGGTTA	GACGCTGTGGCTGAGTGATA	59.9	862	83	gb protein putative expressed
OsISM02337	LOC_Os01g16010	9012273	GACTTCCTCCGCGACCTC	TTTCCCAGCTCTAACACGCT	60.5	1322	91	BCAS2 protein putative expressed
OsISM02338	LOC_Os01g16010	9013684	AAGAAATGCTCAGAGCCAGC	AGCTTCCAAACCTCAACACC	59.5	709	88	BCAS2 protein putative expressed
OsISM02339	LOC_Os01g16010	9014372	TGGTGTTGAGGTTTGGAAGC	ATTCGACAGCCATCTTTTGC	60.6	408	75	BCAS2 protein putative expressed
OsISM02340	LOC_Os01g16010	9014760	GCAAAAGATGGCTGTCGAAT	GCATGGAGTTGACCACCAGT	60.6	170	91	BCAS2 protein putative expressed

ISM IDs	Rice gene locus IDs	Physical positions (bp) on chromosomes	Forward primers (5'-3')	Reverse primers (5'-3')	Melting tempearature (T°C)	Expected product size (bp) in PCR	Expected product size (bp) in Q-PCR	Putative Functions
OsISM02341	LOC_Os01g16010	9014978	AGCTGCATGTGTTGATCTGC	GCTGATTAGTTCCGGTGCTG	60.4	168	96	BCAS2 protein putative expressed
OsISM02344	LOC_Os01g16110	9075955	TCTGCCTCACACTCTGGGAT	TGGGTACAGCAGGCATGTAAT	60.6	175	84	la domain containing protein putative expressed
OsISM02345	LOC_Os01g16110	9076205	GAGCTAAAATACTGACTCAAGTGGA	GCCATGTTCGTCCATTAAAGA	59.1	1325	90	la domain containing protein putative expressed
OsISM02346	LOC_Os01g16110	9077529	CTGGGTTCCAATTTCCAAAG	CTTGAGTTTGCCAATGCTTCT	59.5	249	91	la domain containing protein putative expressed
OsISM02347	LOC_Os01g16110	9077757	AGAAGCATTGGCAAACTCAAG	CAAGGAAACCCACTTTGACC	59.5	170	83	la domain containing protein putative expressed
OsISM02348	LOC_Os01g16110	9078345	AATTGGAGCATGCAGAGCTT	GCCTGTTCACATCTTGGTCA	59.8	562	97	la domain containing protein putative expressed
OsISM02349	LOC_Os01g16110	9078887	TGACCAAGATGTGAACAGGC	TTTTGAAAATGCTTGAGGTCC	59.4	651	59	la domain containing protein putative expressed
OsISM02350	LOC_Os01g16110	9079514	GACGGACCTCAAGCATTTTC	CCAGCTTGGGAACTTCTTTG	59.8	787	99	la domain containing protein putative expressed
OsISM02351	LOC_Os01g16110	9080546	ACGGATCTACTCCAGAAAACC	AGAACCAACAGGCGAGCTAC	58.4	387	90	la domain containing protein putative expressed
OsISM02352	LOC_Os01g16110	9080956	TCAACACCCAAGTCATCAGC	AACTTCTTGCGTTCAGCGAT	59.9	230	94	la domain containing protein putative expressed
OsISM02353	LOC_Os01g16110	9081166	ATCGCTGAACGCAAGAAGTT	CGACCAAAAACGATATAACGAA	59.5	293	67	la domain containing protein putative expressed
OsISM02354	LOC_Os01g16110	9081525	GCGGCAGCAAATTACAGATA	TGCGTTGGAAGTTTTTCTCC	59.6	173	77	la domain containing protein putative expressed
OsISM02355	LOC_Os01g16110	9081728	TCGAATTCCATCACAAGGGT	GGCTGCTATCTTGATTTCGG	60.1	617	79	la domain containing protein putative expressed
OsISM02356	LOC_Os01g16124	9097636	TGCATCTCTTCATGGACGAC	ATGTGGAAGTCCACGCTTCT	59.8	202	96	retrotransposon protein putative unclassified
OsISM02357	LOC_Os01g16180	9162687	TGGAATGCTGAGGAGATTATGA	TCACCAACCCAAAGGAAGAC	59.8	194	78	MT-A70 domain containing protein expressed
OsISM02358	LOC_Os01g16180	9162861	GTCTTCCTTTGGGTTGGTGA	TTACCCAGCAAACATCCTCA	59.5	271	95	MT-A70 domain containing protein expressed
OsISM02359	LOC_Os01g16180	9163163	GCGGCATGACTCTCATACAA	GCTACGTCGCACTGTTCCTT	60.1	189	77	MT-A70 domain containing protein expressed
OsISM02360	LOC_Os01g16180	9163355	GATGGACATGTTATCCACGCT	TCTGTACATGTCTTCAGGCTTTTT	59.6	633	94	MT-A70 domain containing protein expressed

ISM IDs	Rice gene locus IDs	Physical positions (bp) on chromosomes	Forward primers (5'-3')	Reverse primers (5'-3')	Melting tempearature (T˚C)	Expected product size (bp) in PCR	Expected product size (bp) in Q-PCR	Putative Functions
OsISM02361	LOC_Os01g16180	9164050	TCGTCCTGGTTGGCTTACTC	CCCATCCTTGTCTGCAAAGT	60.2	193	89	MT-A70 domain containing protein expressed
OsISM02362	LOC_Os01g16190	9165557	CCCTCCTCAAGATGCTCAAG	ACCCTGACGGTGTAGTCGTC	60.0	174	95	26S proteasome non-ATPase regulatory subunit 14 putative expressed
OsISM02363	LOC_Os01g16190	9165787	ACCACGTCTTCCAGACCAAC	TCCAGGATGGGAGTGATACC	59.9	1509	87	26S proteasome non-ATPase regulatory subunit 14 putative expressed
OsISM02364	LOC_Os01g16190	9167291	CTGGATTTGGATGTTGGCTT	TATCACAACTGCAACAGCCC	59.8	658	87	26S proteasome non-ATPase regulatory subunit 14 putative expressed
OsISM02365	LOC_Os01g16190	9168034	CGCGACAGACAACATCAAAT	TAGTGTCTGTTCAGCCCGTG	59.8	169	77	26S proteasome non-ATPase regulatory subunit 14 putative expressed
OsISM02366	LOC_Os01g16190	9168276	TGGACCGATGGATTGATTTT	TTTGATGGCTAGGTTCAGCA	59.8	214	88	26S proteasome non-ATPase regulatory subunit 14 putative expressed
OsISM02368	LOC_Os01g16290	9224674	GAGGCGATGCAGGAGAAG	TGTGCGAACTCCATCCAAT	59.8	1209	82	DNA gyrase subunit B chloroplast/mitochondrial precursor putative expressed
OsISM02369	LOC_Os01g16290	9225897	CATTGGAAGCACAGGATCAC	CTTCATCCACGGCATTATCC	59.7	168	69	DNA gyrase subunit B chloroplast/mitochondrial precursor putative expressed
OsISM02370	LOC_Os01g16290	9226097	ATCCTTCATGGCGACAATTC	CGCAAGATTTCTTCGTCTGAG	60.0	713	86	DNA gyrase subunit B chloroplast/mitochondrial precursor putative expressed
OsISM02371	LOC_Os01g16290	9226798	AGAAATCTTGCGTGGAAACC	AGCCACTCTTTGAACCTCCA	59.5	454	70	DNA gyrase subunit B chloroplast/mitochondrial precursor putative expressed
OsISM02372	LOC_Os01g16290	9227274	TGGTGTTGGCCTATCAGTTG	TCCATCACGCCAAACAGTAA	59.8	373	65	DNA gyrase subunit B chloroplast/mitochondrial precursor putative expressed
OsISM02373	LOC_Os01g16290	9227720	CAAGTTCTCGTCAAGGCACA	TGCGACCAGCTATCGTATTG	59.9	179	94	DNA gyrase subunit B chloroplast/mitochondrial precursor putative expressed
OsISM02374	LOC_Os01g16290	9227878	TCAATACGATAGCTGGTCGC	CATCTTCTTCCTTTGTCAATGC	58.9	874	79	DNA gyrase subunit B chloroplast/mitochondrial precursor putative expressed
OsISM02375	LOC_Os01g16290	9228781	TATGCTGGTGGTCTGGTTGA	ATGCGATTGGATCATGAAGG	60.5	928	71	DNA gyrase subunit B chloroplast/mitochondrial precursor putative expressed
OsISM02376	LOC_Os01g16290	9229693	CATGATCCAATCGCATTCAG	GCATATCCTAGCACCGTATCAG	59.4	892	99	DNA gyrase subunit B chloroplast/mitochondrial precursor putative expressed
OsISM02377	LOC_Os01g16290	9230662	TCGCAAAGAAGTCGAAGACTC	ATGCTCCCCACTCAAGCTAA	59.8	271	54	DNA gyrase subunit B chloroplast/mitochondrial precursor putative expressed
OsISM02378	LOC_Os01g16290	9230975	AATCCAGAGTTTGAAGGGCA	TCGGACTTCTGGATTTCCC	59.8	164	52	DNA gyrase subunit B chloroplast/mitochondrial precursor putative expressed
OsISM02379	LOC_Os01g16290	9231205	GATTCGATTCTGTCAAAGTCC C	GCTCTCTTAGCTGCCAATGC	60.1	263	57	DNA gyrase subunit B chloroplast/mitochondrial precursor putative expressed

ISM IDs	Rice gene locus IDs	Physical positions (bp) on chromosomes	Forward primers (5'-3')	Reverse primers (5'-3')	Melting tempearature (T˚C)	Expected product size (bp) in PCR	Expected product size (bp) in Q-PCR	Putative Functions
OsISM02380	LOC_Os01g16290	9231516	TGGGAAATTAGCTGACTGTGC	TCCTTGTTTTGCACTACCCC	60.1	320	92	DNA gyrase subunit B chloroplast/mitochondrial precursor putative expressed
OsISM02381	LOC_Os01g16290	9231816	GGGGTAGTGCAAAACAAGGA	CTGCCTCGTCTCTCCTTTCA	60.3	1649	91	DNA gyrase subunit B chloroplast/mitochondrial precursor putative expressed
OsISM02382	LOC_Os01g16290	9233498	TTGTCGCTCTTGGATTAGGAG	TGCACCATCTACATCAGCATC	59.6	164	99	DNA gyrase subunit B chloroplast/mitochondrial precursor putative expressed
OsISM02383	LOC_Os01g16290	9233658	TGCACATATCCGGACTCTTCT	AAGAGGAGGCACACCAACAT	59.6	469	92	DNA gyrase subunit B chloroplast/mitochondrial precursor putative expressed
OsISM02384	LOC_Os01g16290	9234100	TGCATTTATGTTGGTGTGCC	GTAGTGCGCTTGTTTCCCAC	60.5	148	58	DNA gyrase subunit B chloroplast/mitochondrial precursor putative expressed
OsISM02385	LOC_Os01g16290	9234283	CACCTTCCCAACAAATGCTT	CAGGGTCCATTGTTGTCTCC	60.2	1040	93	DNA gyrase subunit B chloroplast/mitochondrial precursor putative expressed
OsISM02386	LOC_Os01g16290	9235367	GCCAACGTTGTGTTCTCATC	TAGGTTGACCATGCTTGCAG	59.5	173	85	DNA gyrase subunit B chloroplast/mitochondrial precursor putative expressed
OsISM02387	LOC_Os01g16310	9245708	GACCAGCCCATCACGTTC	AGAACGGAGACGGAGCATAC	59.7	2032	83	glycosyl hydrolase family 31 putative expressed
OsISM02388	LOC_Os01g16310	9247793	TTTGACCTGACAAAGCCTGA	ATCCTCCCCTGGACTGAACT	59.7	469	63	glycosyl hydrolase family 31 putative expressed
OsISM02389	LOC_Os01g16310	9248422	GTCCCGGTCTCTGCATTCT	CGTCGTCTACCTCCCAGTTC	60.0	1447	92	glycosyl hydrolase family 31 putative expressed
OsISM02390	LOC_Os01g16310	9249925	ACCATGACCTACTGCAACCC	CTTCTTGGCCTCCTCGAAC	59.9	422	82	glycosyl hydrolase family 31 putative expressed
OsISM02391	LOC_Os01g16310	9250952	CTTCACCGTCGTCTTCCG	CAGTTGGATCCTGGCTTGTT	60.3	189	49	glycosyl hydrolase family 31 putative expressed
OsISM02392	LOC_Os01g16310	9251199	AAAGCCTGGGAGTTCTACCG	CGGGTAGTGGAGGAAGAGGT	60.6	677	88	glycosyl hydrolase family 31 putative expressed
OsISM02393	LOC_Os01g16330	9258415	ACGAGAGGAGCGATGCAG	CTGAAGACGACGGAGAGGAG	60.2	227	96	OsRhmbd2 - Putative Rhomboid homologue expressed
OsISM02394	LOC_Os01g16330	9258622	CTCCTCTCCGTCGTCTTCAG	GCAAACAACTTCCATAGACGG	59.9	1041	96	OsRhmbd2 - Putative Rhomboid homologue expressed
OsISM02395	LOC_Os01g16330	9259733	TTCGAGAGGCAGATAGGCTC	CCTTTCAGGAGAACCAAAGAT	58.5	598	99	OsRhmbd2 - Putative Rhomboid homologue expressed
OsISM02396	LOC_Os01g16330	9260302	GAGATCCTATCTTTGGTTCTCCTG	GTCCACATGCAAGAGTGCTG	60.1	770	59	OsRhmbd2 - Putative Rhomboid homologue expressed
OsISM02397	LOC_Os01g16330	9261128	TCACGATTTCGTATTTTTGGTG	AGAGAACGCTTCCCAGAAGA	59.5	186	93	OsRhmbd2 - Putative Rhomboid homologue expressed

ISM IDs	Rice gene locus IDs	Physical positions (bp) on chromosomes	Forward primers (5'-3')	Reverse primers (5'-3')	Melting tempearature (T˚C)	Expected product size (bp) in PCR	Expected product size (bp) in Q-PCR	Putative Functions
OsISM02398	LOC_Os01g16330	9261351	TATCGTCTGAACGTGCTTGG	TCTTGCGAAAAATGATGCAA	60.1	704	67	OsRhmbd2 - Putative Rhomboid homologue expressed
OsISM02399	LOC_Os01g16330	9262073	GGAGGTTCATCTAGGCCCTC	GACAGGAGCGAATCCAGATG	60.4	645	97	OsRhmbd2 - Putative Rhomboid homologue expressed
OsISM02400	LOC_Os01g16340	9264347	ATTGGGGTGGATCCCGATA	GACCACTATGTGCACGAACG	61.2	184	94	expressed protein
OsISM02401	LOC_Os01g16340	9264550	GCTTGGACACCAAATCCATC	TTGGAAATGGGCTTGACTTC	60.2	314	91	expressed protein
OsISM02402	LOC_Os01g16340	9264844	GAAGTCAAGCCCATTTCCAA	ATCCACAGGCCATACGAAAA	60.2	443	70	expressed protein
OsISM02403	LOC_Os01g16340	9265328	CATCACAGACCTGGAAGGCT	GGGAACAAGATTGAAGCAGC	60.0	957	86	expressed protein
OsISM02404	LOC_Os01g16340	9266289	AAGGCCCTGTCCCTGAATAG	GATGGCAGCACAAGACCTTT	60.4	841	87	expressed protein
OsISM02405	LOC_Os01g16370	9293646	CCTGGGACATATGCCAACTC	GCCTCCAAAGGTCATGAAGA	60.3	1600	85	NBS-LRR type disease resistance protein putative expressed
OsISM02406	LOC_Os01g16370	9297111	GCTGCCAAGATTCATCCAAA	GGCTCCTGTCATTCAAAAGG	60.4	1224	79	NBS-LRR type disease resistance protein putative expressed
OsISM02407	LOC_Os01g16400	9313262	ACGACCTCGAGAGCATGAAC	CTCACCTGCTTCATCCACG	60.4	260	95	NB-ARC domain containing protein expressed
OsISM02408	LOC_Os01g16400	9314177	AAGGACATTCCCCAACTCCT	CCCAGTCCTCCAGACTCCAT	60.6	800	95	NB-ARC domain containing protein expressed
OsISM02409	LOC_Os01g16450	9339369	CCTCCACTTCCATGACTGCT	GTTGTCCAGCAAGATCGACG	61.0	869	56	peroxidase precursor putative expressed
OsISM02410	LOC_Os01g16480	9351749	TATGAGTTGCGTTGGATGGA	AACAACAAAGACTGGGTCCG	60.0	171	58	retrotransposon protein putative unclassified
OsISM02411	LOC_Os01g16480	9352027	GTCGGGATATGAAGTCGCTG	GCTGATCATCACTTGACCGA	60.2	248	80	retrotransposon protein putative unclassified
OsISM02412	LOC_Os01g16480	9352303	TTCCTCTCTGCGAGAATTGG	ACAGAGGGAAGTCGACAAGC	60.0	1577	87	retrotransposon protein putative unclassified
OsISM02413	LOC_Os01g16480	9353866	CGACTTCCCTCTGTCGAGTC	CGTACCCACACCCATTGAA	60.1	2449	99	retrotransposon protein putative unclassified
OsISM02414	LOC_Os01g16480	9356500	GAATCGTCGGAAGACACCC	CCACTTACTTCTCGCCAAGC	60.2	165	93	retrotransposon protein putative unclassified
OsISM02415	LOC_Os01g16480	9356631	CAACGGCTTGTAACGCTTG	TTCCCTCCAGATCTGTCCTG	60.3	141	66	retrotransposon protein putative unclassified

ISM IDs	Rice gene locus IDs	Physical positions (bp) on chromosomes	Forward primers (5'-3')	Reverse primers (5'-3')	Melting tempearature (T˚C)	Expected product size (bp) in PCR	Expected product size (bp) in Q-PCR	Putative Functions
OsISM02416	LOC_Os01g16480	9356960	ATGAGATTGCGGAGGCAAG	GACACCACACTCCCTGGC	60.7	248	99	retrotransposon protein putative unclassified
OsISM02417	LOC_Os01g16480	9357440	ATGTGATGGAAGAAATCGGC	TGCTGGACATCCTCGACAT	60.1	223	80	retrotransposon protein putative unclassified
OsISM02418	LOC_Os01g16480	9357733	ATAGACCAGGATCTGCCCG	CAGTACTGGATGCGACGGT	59.9	172	93	retrotransposon protein putative unclassified
OsISM02419	LOC_Os01g16510	9369830	GAATCCACCGGCTTCGAC	GAAGTTCTCGGTGGCGTG	61.0	146	70	expressed protein
OsISM02420	LOC_Os01g16510	9370034	CTCTACGGCGGGAGCAAG	ACTGCTCACGCTTGTCTGTG	61.1	520	92	expressed protein
OsISM02421	LOC_Os01g16510	9370713	GAGCTGGCTCGGCTACTG	ATACATTCCTTGCACGCCAC	60.4	876	73	expressed protein
OsISM02422	LOC_Os01g16510	9371596	ATCAGCTCTTGGTGCTGGAG	GTTGGTTTCGAACTTGCCAT	60.3	670	94	expressed protein
OsISM02423	LOC_Os01g16540	9385345	GTCCACGCCGACTTCAAC	CACCACCAGCACCTCCAC	60.7	154	70	NEDD8-activating enzyme E1 catalytic subunit putative expressed
OsISM02424	LOC_Os01g16540	9385583	ATCGACGTCTCCAACCTCAA	TCGCTCCATAACCCTCTTTG	60.4	726	94	NEDD8-activating enzyme E1 catalytic subunit putative expressed
OsISM02425	LOC_Os01g16540	9386424	ATATCAATTCCGTGGCGTGT	CAGGGATTGGCTTGTCATCT	60.1	736	58	NEDD8-activating enzyme E1 catalytic subunit putative expressed
OsISM02426	LOC_Os01g16540	9387300	GCTGAGACTCCGAGAACTGC	TCAGCATCAAAAGGTTTCCC	60.2	175	90	NEDD8-activating enzyme E1 catalytic subunit putative expressed
OsISM02427	LOC_Os01g16540	9387476	GATGCAGAACATATGCAGTGG	GAGAGACACCTGGGTAAACGA	59.2	174	97	NEDD8-activating enzyme E1 catalytic subunit putative expressed
OsISM02428	LOC_Os01g16540	9387596	GCAGAACTTTTTGGTATTTCTG G	GCAATGGCTGGGATGATATT	59.5	556	75	NEDD8-activating enzyme E1 catalytic subunit putative expressed
OsISM02429	LOC_Os01g16540	9388204	ATCTCTGGGTGCAGCAAAAC	AATGTGTGTTCCATCAAGGC	59.3	250	64	NEDD8-activating enzyme E1 catalytic subunit putative expressed
OsISM02430	LOC_Os01g16540	9388518	TTGGGACATCGACTACCCTC	GGTTATCGCCTTCGTGTGTT	60.0	560	97	NEDD8-activating enzyme E1 catalytic subunit putative expressed
OsISM02431	LOC_Os01g16580	9413967	TTGCAAAGATCATTGTATGGG	TGGTCCCGATGATATTCAGA	58.7	182	89	retrotransposon protein putative unclassified
OsISM02432	LOC_Os01g16580	9414131	TGAATATCATCGGGACCACA	AATGCCGTTTGGTAGGAGC	59.9	405	99	retrotransposon protein putative unclassified
OsISM02433	LOC_Os01g16600	9423995	AGATCCCCACCTTCGTCGT	ATGCCCAGGGCCTATTTAGT	60.8	690	76	expressed protein

ISM IDs	Rice gene locus IDs	Physical positions (bp) on chromosomes	Forward primers (5'-3')	Reverse primers (5'-3')	Melting tempearature (T˚C)	Expected product size (bp) in PCR	Expected product size (bp) in Q-PCR	Putative Functions
OsISM02434	LOC_Os01g16600	9424693	TGGCTGGAGAAAGCAAAGAT	TGATCTGGTTCTGCCATCAA	60.1	313	54	expressed protein
OsISM02435	LOC_Os01g16600	9425123	AGGCCCTGTCACAAATATCG	TCCATGTAGGGGCAATCTTC	59.9	257	84	expressed protein
OsISM02436	LOC_Os01g16600	9425422	AAGCTTTTGGATGGGTCTTG	TGGAGTGCACTAGCAACAGC	59.7	147	53	expressed protein
OsISM02437	LOC_Os01g16600	9425566	CCATGGGGTTCAACATATCC	TCCCAAGTTTGGTATCAAAAGG	60.0	186	66	expressed protein
OsISM02438	LOC_Os01g16600	9425765	CCACTTCACTTACGGATGTGACTA	GTGGCCTGTCTTGGTATGCT	60.3	250	99	expressed protein
OsISM02439	LOC_Os01g16600	9426023	AATCTTACATTGCCCCCTCC	TTTGCGGTAGCCTCGTTTAC	60.2	1701	75	expressed protein
OsISM02440	LOC_Os01g16630	9437612	GAGAGGGAAGTCGGTGTTTG	CCAAAGGTTTTTGGAAGCAA	59.9	220	97	hypothetical protein
OsISM02441	LOC_Os01g16630	9437857	GTGTAAGGAAGGATGAGGCG	TGTTGTTGGTGGCCGTAGTA	59.9	316	96	hypothetical protein
OsISM02442	LOC_Os01g16630	9438114	TGTTCAGAAGCAACGACAGC	GTGCACCCTGTCGATGAAC	60.1	208	90	hypothetical protein
OsISM02443	LOC_Os01g16680	9469821	TGCTCACTAGCCATAGCCCT	TCCCTGCCTGATCATAGTCC	60.0	521	95	heat shock protein DnaJ putative expressed
OsISM02444	LOC_Os01g16680	9470477	CCAAGAAGGGGATCTCACTG	TAGTGCTGCTTTGCGGTATG	59.8	1119	96	heat shock protein DnaJ putative expressed
OsISM02445	LOC_Os01g16680	9471576	CATACCGCAAAGCAGCACTA	TCTCTCCACACTGCATCGTT	59.7	166	89	heat shock protein DnaJ putative expressed
OsISM02446	LOC_Os01g16680	9471802	TGCTATACTTTCGGACCCTACAA	CATCCCCGCAGAAAAAATAAA	60.0	1613	73	heat shock protein DnaJ putative expressed
OsISM02447	LOC_Os01g16714	9488405	ATGGAGATCGCCTACGACC	TCGCCAAGTTCCATATCTCC	60.0	3238	98	flavin monooxygenase putative expressed
OsISM02448	LOC_Os01g16714	9491940	GGAGCACCACCAAGCACT	GTTCTCCCCCTTCCAGTGA	59.9	716	96	flavin monooxygenase putative expressed
OsISM02449	LOC_Os01g16730	9495817	CTGAAAATGATGATGTTATTTCTGA	TTCAGCTTCAAAGATCGCAA	58.6	209	92	ulp1 protease family C-terminal catalytic domain containing protein expressed
OsISM02450	LOC_Os01g16730	9496006	TTGCGATCTTTGAAGCTGAA	TTTTGAAATCAACTTCTGCTGTTT	59.5	217	95	ulp1 protease family C-terminal catalytic domain containing protein expressed
OsISM02451	LOC_Os01g16730	9496278	GAAACGAAAACTCCACTGCC	TTTCTTCTGTTGCATCAATCAT	58.5	170	63	ulp1 protease family C-terminal catalytic domain containing protein expressed

ISM IDs	Rice gene locus IDs	Physical positions (bp) on chromosomes	Forward primers (5'-3')	Reverse primers (5'-3')	Melting tempearature (T˚C)	Expected product size (bp) in PCR	Expected product size (bp) in Q-PCR	Putative Functions
OsISM02452	LOC_Os01g16730	9496991	GCAAGCTTGAGAAGTCCAAAA	TGCAACACTTTTCGCATACC	59.7	184	95	ulp1 protease family C-terminal catalytic domain containing protein expressed
OsISM02453	LOC_Os01g16730	9497256	TGGCAGAAGACCGATAAAAGA	TGCAGAATCAAGCCTTTGAA	59.7	178	95	ulp1 protease family C-terminal catalytic domain containing protein expressed
OsISM02454	LOC_Os01g16730	9497569	GCTAGCAAGCCAAGTGTTCC	ATGCTGCCATCATTCAACTT	59.1	210	78	ulp1 protease family C-terminal catalytic domain containing protein expressed
OsISM02455	LOC_Os01g16730	9497787	AAGCTAACATTTGTGAAACCCC	GCATTGGGGACTGTTCATTT	59.6	161	76	ulp1 protease family C-terminal catalytic domain containing protein expressed
OsISM02456	LOC_Os01g16730	9497940	CCCAATGCTGTGCAAAGATAC	CAGGCTTACAAAGCTTTCTCATTA	59.8	203	99	ulp1 protease family C-terminal catalytic domain containing protein expressed
OsISM02457	LOC_Os01g16730	9498093	GGATAAAAATTCTGATGTTGATGG	TCTTGCTTTAGTTATCTTTGGAGGA	59.3	213	86	ulp1 protease family C-terminal catalytic domain containing protein expressed
OsISM02458	LOC_Os01g16730	9498475	CAGCATGCCCTACTGAATTG	TTGTGACAGGTCCAATGGAAT	59.8	550	54	ulp1 protease family C-terminal catalytic domain containing protein expressed
OsISM02459	LOC_Os01g16730	9499007	CCATTGGACCTGTCACAAAA	TGAATCTTCCAAAGTTACCATTATCA	59.8	174	97	ulp1 protease family C-terminal catalytic domain containing protein expressed
OsISM02460	LOC_Os01g16730	9499214	TTGCCTGTTCAACAACAGTACAC	CTGTAGATTGCCTTTCCCCA	60.1	214	86	ulp1 protease family C-terminal catalytic domain containing protein expressed
OsISM02461	LOC_Os01g16730	9499661	CAATCTTTTTGTGCCACAGC	CGTCAATGTCAACAGCAACC	59.7	194	98	ulp1 protease family C-terminal catalytic domain containing protein expressed
OsISM02462	LOC_Os01g16730	9499958	CCATCCCTCAAAATCCAAAA	GGGATGCTCCATCAAAACAC	60.0	249	99	ulp1 protease family C-terminal catalytic domain containing protein expressed
OsISM02463	LOC_Os01g16730	9500201	CATCCCTTGCAAGACCTGTT	AACCAGTGCCGATTCTTGAC	60.1	141	68	ulp1 protease family C-terminal catalytic domain containing protein expressed
OsISM02464	LOC_Os01g16730	9500412	TGTCATCAACAGATTAGGCCA	TGACATAAAGGTTCCAGGCA	59.1	190	62	ulp1 protease family C-terminal catalytic domain containing protein expressed
OsISM02465	LOC_Os01g16730	9500635	TCATTTATCCTCCAGTGCCC	CCCAAACCTCCATGAACTTG	60.1	1042	76	ulp1 protease family C-terminal catalytic domain containing protein expressed
OsISM02466	LOC_Os01g16750	9512952	GGAGCACCACCAAGCAGT	GTTCTCCCCCTTCCAGTGAT	60.1	980	96	flavin monooxygenase putative
OsISM02468	LOC_Os01g16780	9533363	CCCCTCTAAGGTGGTCGTTT	GTTCTCGGCCTCAGACTGG	60.7	189	93	retrotransposon protein putative unclassified
OsISM02469	LOC_Os01g16820	9577611	TGCCGGCTATCCTTATGAAC	CCGGAGTTTCTTAATTTCCTGA	59.8	130	81	retrotransposon protein putative Ty1-copia subclass
OsISM02471	LOC_Os01g16830	9598641	TTTGATCTCAACATGGAGGC	ACTCAAATTGCAATGCGTCA	60.2	1286	72	transposon protein putative Mariner sub-class

ISM IDs	Rice gene locus IDs	Physical positions (bp) on chromosomes	Forward primers (5'-3')	Reverse primers (5'-3')	Melting tempearature (T˚C)	Expected product size (bp) in PCR	Expected product size (bp) in Q-PCR	Putative Functions
OsISM02472	LOC_Os01g16830	9600008	CCGACTCAGATGACGATCAA	ACGTTGGACTGTACGGATGC	60.4	191	87	transposon protein putative Mariner sub-class
OsISM02473	LOC_Os01g16830	9600720	TGGAAAGATAGGCATTTGGC	TCGATTAGCTCATCCATGTTTC	59.6	485	79	transposon protein putative Mariner sub-class
OsISM02475	LOC_Os01g16900	9667309	CGTGAAAATACCGAGGGAGA	TAAGCTTTCCACAACACCCC	60.0	147	67	dehydrogenase putative expressed
OsISM02476	LOC_Os01g16900	9667558	ATCATGAGGAAGACCGATGG	TGTCAATGATGACCTCCTCG	59.8	192	92	dehydrogenase putative expressed
OsISM02477	LOC_Os01g16900	9667730	CGAGGAGGTCATCATTGACA	TGGCATCACCAGTACATCAAA	59.8	269	74	dehydrogenase putative expressed
OsISM02478	LOC_Os01g16900	9668028	TTGTGCTGGTCTGATTGGAG	AGACAAATGCCACCTTCACC	59.9	147	67	dehydrogenase putative expressed
OsISM02479	LOC_Os01g16900	9668155	GGTGAAGGTGGCATTTGTCT	CAGCACTCAGCATAAGAGCG	59.9	485	98	dehydrogenase putative expressed
OsISM02481	LOC_Os01g17000	9733061	ACCATCCCCCTCCTCCTC	TTGGTTTTTACACCATTTCCTAAA	60.1	2228	87	expressed protein
OsISM02482	LOC_Os01g17020	9749293	ACTGGCTCCCCTTCTCCAT	GAAACAACATGAAGGACGCC	60.8	1521	95	glycosyl hydrolases family 17 protein putative expressed
OsISM02483	LOC_Os01g17020	9751058	GTTGGCAATTCATCCTCCAT	GTAGAATGAGAGGGCCACCA	59.9	1156	94	glycosyl hydrolases family 17 protein putative expressed
OsISM02484	LOC_Os01g17030	9753871	GTTCTTTTCGCTCGTAACCG	CTTGACAAAGCCACAAGCAG	59.8	418	46	heat shock protein DnaJ putative
OsISM02485	LOC_Os01g17030	9754269	CTGCTTGTGGCTTTGTCAAG	AATTTCCCTGTGCCATCGT	60.0	503	68	heat shock protein DnaJ putative
OsISM02486	LOC_Os01g17040	9764389	CCTCATCAATCTGGGTTTCG	CCATCAAGAAAAGCTGCTCC	60.2	827	55	heat shock protein DnaJ putative
OsISM02487	LOC_Os01g17040	9765196	GGAGCAGCTTTTCTTGATGG	AAGTTCAAAAGTGTAGAGGGTCAA	59.2	193	88	heat shock protein DnaJ putative
OsISM02488	LOC_Os01g17070	9816180	CTGTCGAGGCAGAGAGAAGG	GTGAGAATTGGACCCTCAGC	60.0	623	68	OsDegp1 - Putative Deg protease homologue expressed
OsISM02489	LOC_Os01g17070	9816865	TTGCTTTGGGTGTTTCACAG	TCGTTCCACCGTATCACAAA	59.8	172	95	OsDegp1 - Putative Deg protease homologue expressed
OsISM02490	LOC_Os01g17070	9817077	GTTAGACCTCCATGCGGAGA	TACCATGGAAATCTGGCAAA	59.6	899	82	OsDegp1 - Putative Deg protease homologue expressed
OsISM02491	LOC_Os01g17070	9818157	CAGAACTGTTAGTATCAAGTTGCGA	CAAAGAAGTTAATACCTACAACATGGC	60.2	1483	94	OsDegp1 - Putative Deg protease homologue expressed

ISM IDs	Rice gene locus IDs	Physical positions (bp) on chromosomes	Forward primers (5'-3')	Reverse primers (5'-3')	Melting tempearature (T˚C)	Expected product size (bp) in PCR	Expected product size (bp) in Q-PCR	Putative Functions
OsISM02492	LOC_Os01g17070	9819662	TTCGATGGCAATTGTTTTCA	GAATCCAATCCAAGGTCGAA	60.0	248	74	OsDegp1 - Putative Deg protease homologue expressed
OsISM02493	LOC_Os01g17070	9819970	TCGGATGTTGATAATGGCCT	CACCCTGACATATTCCAGCA	59.9	541	77	OsDegp1 - Putative Deg protease homologue expressed
OsISM02494	LOC_Os01g17070	9820524	ATGTGGTGGGAAATTCCTCT	CACCATTGGTTTCTTGGTCA	58.8	218	99	OsDegp1 - Putative Deg protease homologue expressed
OsISM02495	LOC_Os01g17070	9820734	CAATGGTGATTTTTCAGCGA	AGTGCTTCCATCATTTTCTCG	59.5	176	68	OsDegp1 - Putative Deg protease homologue expressed
OsISM02496	LOC_Os01g17070	9820887	AGCGAGAAAATGATGGAAGC	AGCTAGGGATCGGTGTAGGC	60.0	199	94	OsDegp1 - Putative Deg protease homologue expressed
OsISM02497	LOC_Os01g17110	9834444	CAGTCAGACCGGACAGCATA	GTCGTTTGTGTACCCTGGCT	59.9	479	82	retrotransposon protein putative Ty3-gypsy subclass
OsISM02498	LOC_Os01g17130	9845165	CCCTAGGAGGGAGCAGAGAT	TGCAGACCAGTGTCTTCTGG	59.9	1232	97	retrotransposon protein putative Ty3-gypsy subclass
OsISM02499	LOC_Os01g17150	9858461	CAATGTCGCCTCCACCAT	GAGATTCTTGCACTGGAGCC	60.2	749	47	plastid-specific 50S ribosomal protein 5 chloroplast precursor putative expressed
OsISM02500	LOC_Os01g17160	9867499	AACAAGTTCTTGGCCTCCCT	CTCGATGTCATCAGCAGGAA	60.0	285	80	OsSubt1 - Putative Subtilisin homologue expressed
OsISM02501	LOC_Os01g17160	9868039	CAAGTTCGACCACCACTGC	CGTCGAGATGAACAGGAAGA	59.6	636	80	OsSubt1 - Putative Subtilisin homologue expressed
OsISM02502	LOC_Os01g17160	9868836	CACCGTGTTCCACCTCTACC	CACCCCTGTTATACGGGTTC	59.8	528	96	OsSubt1 - Putative Subtilisin homologue expressed
OsISM02503	LOC_Os01g17170	9873826	TCTCCGGGTTCCTCCTCTAC	AGCGAGAAGATCTCAGCCAC	60.1	178	77	magnesium-protoporphyrin IX monomethyl ester cyclase chloroplast precursor putative expressed
OsISM02504	LOC_Os01g17170	9873968	GAAAACAAACCCAGTGGTGG	TTGAAATCAGACAGCCCCTT	60.0	288	94	magnesium-protoporphyrin IX monomethyl ester cyclase chloroplast precursor putative expressed
OsISM02505	LOC_Os01g17170	9874523	CAAGCTCTGGTCACGGTTCT	GTGTCCAGACCAATCCCTTC	59.9	201	97	magnesium-protoporphyrin IX monomethyl ester cyclase chloroplast precursor putative expressed
OsISM02506	LOC_Os01g17170	9874704	GAAGGGATTGGTCTGGACAC	GGGAAAATCCTTGCTGTTGT	59.2	302	78	magnesium-protoporphyrin IX monomethyl ester cyclase chloroplast precursor putative expressed
OsISM02507	LOC_Os01g17180	9877462	GGTCGCCTCCTCCTCTCC	CGGAGTACTCGAACCCGTAA	61.2	173	83	proteasome subunit putative expressed
OsISM02508	LOC_Os01g17180	9877626	AGTACTCCGACGACGAGCC	CCTCAGGGTCTGTCTCAACC	60.1	2468	88	proteasome subunit putative expressed
OsISM02509	LOC_Os01g17180	9880122	ACGTATGGAGCCTGAAAAGG	AGGCATCCATCATTTCTTTG	58.4	1423	99	proteasome subunit putative expressed

ISM IDs	Rice gene locus IDs	Physical positions (bp) on chromosomes	Forward primers (5'-3')	Reverse primers (5'-3')	Melting tempearature (T˚C)	Expected product size (bp) in PCR	Expected product size (bp) in Q-PCR	Putative Functions
OsISM02510	LOC_Os01g17180	9881683	TGAAAGCACTTGAAGAGGCA	CCATACTCCCCCATGTCAAA	60.1	173	89	proteasome subunit putative expressed
OsISM02511	LOC_Os01g17180	9881828	AAATTTGGTTTGACATGGGG	TGACAAGATTTATGCAGCTCCT	59.3	161	68	proteasome subunit putative expressed
OsISM02512	LOC_Os01g17180	9882054	TCCAAATGTACACTGAAACAAAGAA	CCCATGATTCTTGGATGAGG	60.1	639	98	proteasome subunit putative expressed
OsISM02513	LOC_Os01g17180	9882797	ATCCGCGGAGAATTCAATG	TAGCCTCTTGCCCATCAAAG	60.7	183	88	proteasome subunit putative expressed
OsISM02514	LOC_Os01g17180	9882960	CTTTGATGGGCAAGAGGCTA	GTTTGTCATGGCGAGGATCT	60.2	137	59	proteasome subunit putative expressed
OsISM02515	LOC_Os01g17180	9883085	GCCATGACAAACTTGATTGC	TGAAAGGATCGTCCATTATTGTT	59.4	211	98	proteasome subunit putative expressed
OsISM02516	LOC_Os01g17180	9883371	GAATCAGGATTCCATTCATTTC	CTCGACATCCTTTTCTGGGA	58.8	482	54	proteasome subunit putative expressed
OsISM02517	LOC_Os01g17180	9883886	ATCCAGGGCCACATAGATCA	GCTTGGTATTTCCTCATTCCC	60.0	161	75	proteasome subunit putative expressed
OsISM02518	LOC_Os01g17190	9886742	GAGTTCAAGGAGGCCTTCAG	GCATGACAGTTCCCAACTCC	59.8	860	77	OsCam3 - Calmodulin expressed
OsISM02519	LOC_Os01g17240	9913032	ATTCCTCCTCTGCAACATGG	ACAGTTTGTGGGTTCCATCC	59.9	336	85	transporter major facilitator family putative expressed
OsISM02520	LOC_Os01g17240	9913388	TCTTCTGGGGCTACCTGCTA	CGAAGCCAAGGACAGTCTTC	60.0	184	79	transporter major facilitator family putative expressed
OsISM02521	LOC_Os01g17240	9913649	CGTGCTTTCATGGGAGTTG	GCCAATGATCTGCTCCTCTC	60.1	535	96	transporter major facilitator family putative expressed
OsISM02522	LOC_Os01g17240	9914276	ACTCCTTTGGGTCTCTGGGT	TCCTGGATCCTCAAGTGGAC	60.0	190	78	transporter major facilitator family putative expressed
OsISM02523	LOC_Os01g17240	9914598	TGGGGAACTTTCATCTTGCT	CCAGGGGAGAACACAGAAAA	59.9	175	97	transporter major facilitator family putative expressed
OsISM02524	LOC_Os01g17240	9914788	TCTGCAAATTTTGGTGGTTG	GGCCCTAAGAATCCAATTGAC	59.7	317	99	transporter major facilitator family putative expressed
OsISM02525	LOC_Os01g17240	9915125	TGAGCCATATCGATTCACCA	AGACCGGACTGTGAGAATGC	60.2	243	80	transporter major facilitator family putative expressed
OsISM02526	LOC_Os01g17240	9915378	CACCAAGATATCGGTCCTCG	TAAAACCCCAGCCGTGTTAG	60.2	169	64	transporter major facilitator family putative expressed
OsISM02527	LOC_Os01g17240	9915564	AGCGGCAACAGGATACATCT	TTCCAGACCAAAGTCCCAAC	59.8	188	91	transporter major facilitator family putative expressed

ISM IDs	Rice gene locus IDs	Physical positions (bp) on chromosomes	Forward primers (5'-3')	Reverse primers (5'-3')	Melting tempearature (T˚C)	Expected product size (bp) in PCR	Expected product size (bp) in Q-PCR	Putative Functions
OsISM02528	LOC_Os01g17260	9929466	TGGGATTCTTTACAATGGCAG	AGCAAGAGCAAGCTGACCTG	60.4	1377	99	transcription factor putative expressed
OsISM02529	LOC_Os01g17260	9930840	GCTGCTGCTTCTGACTCTGAC	AAACGACTCTTCCTTGCAGC	60.1	181	99	transcription factor putative expressed
OsISM02530	LOC_Os01g17260	9931001	GCTGCAAGGAAGAGTCGTTT	GCTCGTTGCAATTCTTGTTCT	59.6	2085	96	transcription factor putative expressed
OsISM02531	LOC_Os01g17260	9933065	AGAACAAGAATTGCAACGAG C	GGAATGAGTCTGGTCCACTGA	59.8	151	68	transcription factor putative expressed
OsISM02532	LOC_Os01g17260	9933190	CCAGCTCAGTGGACCAGACT	TCTTCCAACCAACGTGCATA	60.3	230	80	transcription factor putative expressed
OsISM02533	LOC_Os01g17260	9933615	GCTAGGAGGATTCCGACCAT	CTGAAGGTTGGCTATCCCTG	60.1	449	95	transcription factor putative expressed
OsISM02534	LOC_Os01g17260	9934215	AGCTTGGGACCCTTGAGAAT	CTGTTGAAGAGTCTGCTGCC	59.4	226	63	transcription factor putative expressed
OsISM02535	LOC_Os01g17300	9953512	ATCTCCCTCACATCCACCAC	GCTCGCTCCTCGTATCGTC	60.4	183	82	expressed protein
OsISM02536	LOC_Os01g17320	9961738	GAATCCCTCCATCGCCAC	AAAGCTTGGTAGGCACGG	60.2	895	99	PPR repeat domain containing protein putative expressed
OsISM02537	LOC_Os01g17320	9963135	CGGAAGCTCTACACCAAAGC	ACCAAATCCACACGTGAAGC	60.5	1380	99	PPR repeat domain containing protein putative expressed
OsISM02538	LOC_Os01g17360	9989820	ATTCCCTCGTCAACGCCT	GTATTTTCGGTGATGGTGGG	60.3	259	58	retrotransposon protein putative unclassified
OsISM02540	LOC_Os01g17360	9991429	GACCTCAGTCTGCCCTTCAA	CGACTTTGAGGTCGCCAT	60.1	138	98	retrotransposon protein putative unclassified
OsISM02541	LOC_Os01g17360	9991673	GATGATCACGTGCGACGA	CGGTCTCCAGGCAAAGAC	59.9	154	80	retrotransposon protein putative unclassified
OsISM02542	LOC_Os01g17360	9992120	TCAGTTTGTTGACTCCACCG	GCTTAAATAGGGGTAGCGCC	59.9	549	54	retrotransposon protein putative unclassified
OsISM02543	LOC_Os01g17360	9993440	ACTTTGTGGCAGAGTGGACC	GCGAACCAGGTATCTGAGGA	60.2	188	96	retrotransposon protein putative unclassified
OsISM02544	LOC_Os01g17390	10004294	TGATTCGATCATGTCGTGGT	CATCATGAATCCAACATGCC	59.8	502	98	OsFBX5 - F-box domain containing protein expressed
OsISM02545	LOC_Os01g17390	10004795	GTCATGCTACGATGCAGAGC	CGTCCTCCAAGACTACGGTC	59.7	258	96	OsFBX5 - F-box domain containing protein expressed
OsISM02546	LOC_Os01g17390	10005159	CCAATGGAGAAGGAACAAGG	GACACCGACAAAAGTGCTCA	59.7	254	96	OsFBX5 - F-box domain containing protein expressed

ISM IDs	Rice gene locus IDs	Physical positions (bp) on chromosomes	Forward primers (5'-3')	Reverse primers (5'-3')	Melting tempearature (T˚C)	Expected product size (bp) in PCR	Expected product size (bp) in Q-PCR	Putative Functions
OsISM02547	LOC_Os01g17390	10005393	TGAGCACTTTTGTCGGTGTC	TCCATCTTGAACCAGGTGTG	59.7	803	75	OsFBX5 - F-box domain containing protein expressed
OsISM02548	LOC_Os01g17440	10028024	GGATGCGGACGAGGACAG	TATGTCCGACAGATGGGC	60.4	379	93	retrotransposon protein putative unclassified expressed
OsISM02549	LOC_Os01g17440	10028497	GGAGAAGAGTCACTGCCGAC	TGTGCTCTGCTTGTCAAAGG	60.1	1258	74	retrotransposon protein putative unclassified expressed
OsISM02551	LOC_Os01g17450	10031259	GCACCTGTGCGAGATGTTTA	ACGAAGGTAGTCGCCGAAC	60.1	366	61	retrotransposon protein putative unclassified
OsISM02552	LOC_Os01g17450	10032193	GTCGTTTAGCCGACCTCCC	CTGTCTCCTCGGCCTCAG	60.8	199	94	retrotransposon protein putative unclassified
OsISM02553	LOC_Os01g17970	10056161	CAATGTGCTAGTACTGATGAG GATG	AAAGCATCAGCTATGCCCCT	60.6	212	99	retrotransposon protein putative Ty1-copia subclass
OsISM02554	LOC_Os01g17970	10056427	TGCTGCAGATTTGGATAGGA	TGATGTCAATGTGCTTTGTCC	59.5	287	87	retrotransposon protein putative Ty1-copia subclass
OsISM02555	LOC_Os01g17970	10056731	GATGTAGTCGCGCAAGGTAA	CAACAAACTCCACCTTGAAACA	59.5	239	81	retrotransposon protein putative Ty1-copia subclass
OsISM02556	LOC_Os01g17990	10060787	GATACGCAGGTGATCAACGA	TCAGACCGTCGAACAAAGTG	59.8	275	89	conserved hypothetical protein
OsISM02557	LOC_Os01g17990	10061072	GCTGATGCGGCTGTCTATCT	GTTCAACGGAGCAACAGCTT	60.5	208	65	conserved hypothetical protein
OsISM02558	LOC_Os01g17990	10061275	TGAACAAGCATAGGTCACCG	AAGGTCCTACCCAGTCAGCA	59.7	1505	88	conserved hypothetical protein
OsISM02559	LOC_Os01g17990	10062767	TGGGTAGGACCTTCTTCTGC	ATCCATTGGTGGAGTTGGAA	59.7	260	97	conserved hypothetical protein
OsISM02560	LOC_Os01g17990	10063025	ATGTGGGAGCGTTCTTTTTG	TTCGTCATCATCATCATCCG	60.3	262	84	conserved hypothetical protein
OsISM02561	LOC_Os01g17990	10063263	AAAGCGGATGATGATGATGAC	CATCTTGTACCCGCCAAAAC	60.1	159	73	conserved hypothetical protein
OsISM02562	LOC_Os01g17990	10063500	CCAATGAGGCAAGCAAATCT	GTGGCTTTCTGACAACAGCA	60.1	340	92	conserved hypothetical protein
OsISM02563	LOC_Os01g17990	10063825	TTGTCAGAAAGCCACTCCCT	TCGGTGTTGTTTCTCTTCCC	60.0	181	98	conserved hypothetical protein
OsISM02565	LOC_Os01g18030	10087413	GAGTGCGGGGAAGCTTTATT	TCGAATAGTGAGCCGCAAG	60.3	334	99	retrotransposon protein putative unclassified
OsISM02566	LOC_Os01g18070	10118448	CGCCTCCAGGGGAACTACTA	CATAAGTGTGCGATGCCTTG	60.9	1858	55	bacterial transferase hexapeptide domain containing protein expressed

ISM IDs	Rice gene locus IDs	Physical positions (bp) on chromosomes	Forward primers (5'-3')	Reverse primers (5'-3')	Melting tempearature (T˚C)	Expected product size (bp) in PCR	Expected product size (bp) in Q-PCR	Putative Functions
OsISM02567	LOC_Os01g18070	10120398	TTCAATTTGGTATGGATGCG	TTGCCACATGGACAAGAGAA	59.8	1105	96	bacterial transferase hexapeptide domain containing protein expressed
OsISM02568	LOC_Os01g18070	10121533	CCATCATTGGGGATAACGTC	CAAATGCTTCGTCCTCAACA	59.9	300	73	bacterial transferase hexapeptide domain containing protein expressed
OsISM02569	LOC_Os01g18070	10121891	TGCTGGAGCCCTTGTAAGAC	GAATTTGGCAGGGTTTCCTC	60.6	391	71	bacterial transferase hexapeptide domain containing protein expressed
OsISM02570	LOC_Os01g18110	10143406	GTCATGGCTGGTGAAGGAG	TCTCCAGAGCATGCAAATGA	59.9	341	99	cinnamoyl CoA reductase putative expressed
OsISM02571	LOC_Os01g18110	10143818	TTATGCGATGGTGTTTTCCA	GCAGCAGCATTGATGACATT	59.9	430	99	cinnamoyl CoA reductase putative expressed
OsISM02572	LOC_Os01g18110	10144345	TGGAGTGACCTTGAGTTCTGC	ACACCTCTCTTCGACGCCT	60.2	314	99	cinnamoyl CoA reductase putative expressed
OsISM02573	LOC_Os01g18110	10144893	GTTTGTTCGCTTGCTTAGGG	GGTAAGGTTTCACCATTGGC	59.6	265	87	cinnamoyl CoA reductase putative expressed
OsISM02574	LOC_Os01g18120	10147935	GATACTTCGTGAGGGGAGCC	AGGGACAGGCCTTCTCTAGC	60.5	894	89	cinnamoyl CoA reductase putative expressed
OsISM02575	LOC_Os01g18120	10148902	GCATCGCCAGTCTCAAATG	CTTGGTTCCCTCGATAGCAG	60.1	168	55	cinnamoyl CoA reductase putative expressed
OsISM02576	LOC_Os01g18120	10149177	TGAGAGCTGTTGGAGTGACC	CAACATCTTCGCGTAGCAAT	59.0	1198	68	cinnamoyl CoA reductase putative expressed
OsISM02577	LOC_Os01g18120	10150677	CCCCCAGTACCCTATCACCT	GCATGCCTAAAGTCTCCAGC	60.0	333	96	cinnamoyl CoA reductase putative expressed
OsISM02578	LOC_Os01g18130	10155486	GCCCAGAAAGGTTGGTGTT	CCTTGCAAATTGACCAAAAA	59.3	718	83	hypothetical protein
OsISM02579	LOC_Os01g18130	10156161	TGCTGCAAGCTGTAATCTGC	TTGTTGTTGCCTTGTTGAGC	60.1	1241	96	hypothetical protein
OsISM02580	LOC_Os01g18130	10157382	GCTCAACAAGGCAACAACAA	CGTTCACACTCCAGAGTCCA	59.9	224	57	hypothetical protein
OsISM02581	LOC_Os01g18140	10159042	CAGGCCAACACCCATCTC	TTAGTGATTATCCCCCACGC	59.9	175	74	expressed protein
OsISM02583	LOC_Os01g18180	10175244	TGTTTGAATGCTTGGAGAGC	AGCTTTGTTATTGTTGCCCG	59.6	190	86	transposon protein putative CACTA En/Spm sub-class
OsISM02584	LOC_Os01g18180	10175793	AAGGGAGACTACGGGGAAAG	GGCCCTCGTGTAGAATGTGT	59.8	341	87	transposon protein putative CACTA En/Spm sub-class
OsISM02585	LOC_Os01g18190	10179780	TTTGATCGAGAGGGGTATCG	GTTAGTCGACTGGCAGCCTC	60.0	147	91	transposon protein putative CACTA En/Spm sub-class

ISM IDs	Rice gene locus IDs	Physical positions (bp) on chromosomes	Forward primers (5'-3')	Reverse primers (5'-3')	Melting tempearature (T˚C)	Expected product size (bp) in PCR	Expected product size (bp) in Q-PCR	Putative Functions
OsISM02586	LOC_Os01g18190	10180376	CATGTGAGCTGCATATTCCC	GGTAAGTCTCGGAAGGGTCC	59.5	178	85	transposon protein putative CACTA En/Spm sub-class
OsISM02588	LOC_Os01g18200	10184965	TAACGCAGCAGCATTTCAAG	GACCTTGTCAAAAATCTTCTCCTC	59.9	236	99	transposon protein putative CACTA En/Spm sub-class
OsISM02589	LOC_Os01g18200	10185177	GAGGAGAAGATTTTTGACAAGGTC	TTTTCTTTCCAAGTCCTGCG	60.0	190	87	transposon protein putative CACTA En/Spm sub-class
OsISM02590	LOC_Os01g18200	10185365	AAACTTGGACGGAGGTTTCA	GTTTGATAGGCAGTGGGCAT	59.8	194	97	transposon protein putative CACTA En/Spm sub-class
OsISM02591	LOC_Os01g18200	10185539	ATGCCCACTGCCTATCAAAC	TGATGTGGCAAGAGAATAAATGA	59.8	1708	96	transposon protein putative CACTA En/Spm sub-class
OsISM02593	LOC_Os01g18210	10193095	CAAGACGGTGGACAACTTCC	AGTCCTTGATGATTCGGTGG	60.2	190	87	peptidyl-prolyl cis-trans isomerase putative expressed
OsISM02594	LOC_Os01g18210	10193265	CCACCGAATCATCAAGGACT	TCATCGTCAAAACATTCACCA	59.9	688	97	peptidyl-prolyl cis-trans isomerase putative expressed
OsISM02595	LOC_Os01g18210	10193932	TGGTGAATGTTTTGACGATGA	TTGGCTTCCATTTGTGTCAG	59.8	640	95	peptidyl-prolyl cis-trans isomerase putative expressed
OsISM02596	LOC_Os01g18210	10194552	CTGACACAAATGGAAGCCAA	CAACGTGACGATTGTCCAAC	59.8	696	67	peptidyl-prolyl cis-trans isomerase putative expressed
OsISM02597	LOC_Os01g18280	10262285	GAGGACTTCCTTGTCGGGTC	CCTCCCACTCTCCCCTTTC	61.0	1337	83	SNF7 domain containing protein putative expressed
OsISM02599	LOC_Os01g18280	10263912	AGGTGCTGGGTGTTTGGTT	TTGAGCAGAATCTCCCCATC	60.3	178	98	SNF7 domain containing protein putative expressed
OsISM02600	LOC_Os01g18280	10264152	TGGCTGTCATCTCAAAGCAG	ATCAACAATTTCAGCAGCCC	60.1	179	87	SNF7 domain containing protein putative expressed
OsISM02601	LOC_Os01g18280	10264433	TTGACACAATGTGGGAAAGC	GACAGCAGGAACTTGTGCAG	59.6	185	97	SNF7 domain containing protein putative expressed
OsISM02602	LOC_Os01g18280	10264652	TGACGGAAGAAAAGTTGCAG	TGAAGGATACCAATGCCCTC	59.5	528	82	SNF7 domain containing protein putative expressed
OsISM02603	LOC_Os01g18280	10265262	GCTGGAGAGGGTTGAAGAAG	GCTCATTCATCGCCTGAATAC	59.4	205	99	SNF7 domain containing protein putative expressed
OsISM02604	LOC_Os01g18280	10265529	CAAAGAGAAATTGATGCTGCC	GCAATTCTGCTTCCAGCTTC	60.0	1148	98	SNF7 domain containing protein putative expressed
OsISM02605	LOC_Os01g18320	10291268	CAACAGCTTCCAGCCTTCC	ACACGAGATCGTCCTTGAGC	60.7	160	69	protoporphyrinogen oxidase chloroplast precursor putative expressed
OsISM02606	LOC_Os01g18320	10291513	CGACCTCATGAGCATCCC	GAAGTCCTCCACCGACTCCT	60.4	178	95	protoporphyrinogen oxidase chloroplast precursor putative expressed

ISM IDs	Rice gene locus IDs	Physical positions (bp) on chromosomes	Forward primers (5'-3')	Reverse primers (5'-3')	Melting tempearature (T˚C)	Expected product size (bp) in PCR	Expected product size (bp) in Q-PCR	Putative Functions
OsISM02607	LOC_Os01g18320	10291724	CGCCTCATTGAGCCTTTCT	CAGTATCCTCCAGCCTCCAC	60.1	174	98	protoporphyrinogen oxidase chloroplast precursor putative expressed
OsISM02608	LOC_Os01g18320	10291911	TGGTGGAACCATCAAAACAA	AAAGATGCAACTGTCTGCCC	60.0	173	97	protoporphyrinogen oxidase chloroplast precursor putative expressed
OsISM02609	LOC_Os01g18320	10292098	CTATGCTCCCGGATGCTATT	AATGCTTGTCAACTTCCATGA	58.7	496	69	protoporphyrinogen oxidase chloroplast precursor putative expressed
OsISM02610	LOC_Os01g18320	10292699	AGTGATATCTTGCGGCCACT	CAGCAGCAACTGGTGGATAA	59.8	637	74	protoporphyrinogen oxidase chloroplast precursor putative expressed
OsISM02611	LOC_Os01g18320	10293417	CGCGTAGTCAGGGAGTTGAG	GGAGCACGATTTGGAAAGAG	60.4	199	65	protoporphyrinogen oxidase chloroplast precursor putative expressed
OsISM02612	LOC_Os01g18320	10293614	CCAGCTGGAAGGGTGTTACT	TCACGGTCAACTGCTTCTACC	59.8	300	99	protoporphyrinogen oxidase chloroplast precursor putative expressed
OsISM02613	LOC_Os01g18330	10296828	GAGTCGGTACATGGGAGGAC	ATACTACCCTGCCTCCGCTT	59.8	150	97	hypothetical protein
OsISM02615	LOC_Os01g18360	10311585	TGCTGATGATAGCATCCCTTC	CTCCTGAGGAACGGCTTGAT	60.9	1080	79	OsIAA4 - Auxin-responsive Aux/IAA gene family member expressed
OsISM02616	LOC_Os01g18360	10312805	TCAAGCTCTGCCACATGTTC	GGCACTCCATAACATCAGCA	59.8	458	55	OsIAA4 - Auxin-responsive Aux/IAA gene family member expressed
OsISM02617	LOC_Os01g18360	10313287	CTGCCCATGTCCTCACCTAT	GCTTCTTTACGCTGCTCAGG	60.1	182	91	OsIAA4 - Auxin-responsive Aux/IAA gene family member expressed
OsISM02618	LOC_Os01g18375	10318496	CTGCAACATCTTGCTTTCCA	GTGAGGAGAAGGCAGATCCA	60.2	297	91	hydrophobic protein putative
OsISM02619	LOC_Os01g18480	10390966	GTCCATCTGGACAGGCAAGT	GTGGTTCCTGTCAATCCCAC	60.2	462	75	transposon protein putative unclassified
OsISM02621	LOC_Os01g18480	10391939	CTAGCAAGGCTTGGTTGCTC	AACCACAACAGGTACGCCTC	60.1	184	98	transposon protein putative unclassified
OsISM02622	LOC_Os01g18480	10392434	CGATGGGGACTTACTGGTGT	CGGGTAACCACGTCTCACTT	59.9	158	66	transposon protein putative unclassified
OsISM02623	LOC_Os01g18480	10392794	GACTTAGGCAGCTGTTTCCG	CTAGCTGTCCCCGTCGAGT	60.2	157	79	transposon protein putative unclassified
OsISM02624	LOC_Os01g18480	10393159	GCACCGTGACCGAGACTACT	ATATCGGCACTGATATCCCG	60.0	152	58	transposon protein putative unclassified
OsISM02626	LOC_Os01g18480	10393867	AGCTCTTCCTCTCACGGAGC	AGACGAAGAGGCCCCAGTA	60.5	144	82	transposon protein putative unclassified
OsISM02627	LOC_Os01g18480	10394186	ACCGACTCACGTACTCCCAC	GCAAGGGCGATTTTGTACTG	60.3	854	76	transposon protein putative unclassified

ISM IDs	Rice gene locus IDs	Physical positions (bp) on chromosomes	Forward primers (5'-3')	Reverse primers (5'-3')	Melting tempearature (T°C)	Expected product size (bp) in PCR	Expected product size (bp) in Q-PCR	Putative Functions
OsISM02631	LOC_Os01g18584	10469987	AGGACACGGGGAAGAATCA	CGGTTCTCCTCCACCACTC	60.6	219	80	OsWRKY9 - Superfamily of TFs having WRKY and zinc finger domains expressed
OsISM02632	LOC_Os01g18584	10470328	ACAACAACCGGCCTTCACTA	AGGAATTGCTGCGTCGTC	60.3	1381	71	OsWRKY9 - Superfamily of TFs having WRKY and zinc finger domains expressed
OsISM02633	LOC_Os01g18584	10471955	GAAACCGAGGGTGTCCGT	ATTCCCCTTTGCCATTTTCT	60.4	468	95	OsWRKY9 - Superfamily of TFs having WRKY and zinc finger domains expressed
OsISM02634	LOC_Os01g18584	10472433	GAGCGTACTACCGATGCACA	ATCAGCACGGTCTTGTCCTC	60.1	4623	83	OsWRKY9 - Superfamily of TFs having WRKY and zinc finger domains expressed
OsISM02635	LOC_Os01g18610	10479884	TGATCCCTCTGGAAGTGGTC	AAGAAGGGGTTCTGCTTGTG	59.7	706	96	retrotransposon protein putative LINE subclass
OsISM02636	LOC_Os01g18610	10480820	CGTCTTGAACCAGGAAGGTC	AAAATTGGCTAGCATCCATGA	59.6	1279	89	retrotransposon protein putative LINE subclass
OsISM02637	LOC_Os01g18610	10482005	GCTCGTTGGTCTGTGTTTTG	TCAATTCTCAGAGACCATTAGAACA	59.3	489	95	retrotransposon protein putative LINE subclass
OsISM02638	LOC_Os01g18660	10509436	CTGCAGAAAGTTACGGCCTC	AGAGCTGGCTAGCTGGAATG	59.9	2672	79	aminotransferase putative expressed
OsISM02639	LOC_Os01g18680	10539409	CCAAAGTGGACCTTCAAACG	GCTGTGGCGGATTCAGAG	60.5	304	86	retrotransposon protein putative unclassified
OsISM02640	LOC_Os01g18720	10551101	GAGAGGGGAGGAAGAGGAGA	AATCAACCTCCATTTTCCCA	59.6	244	66	hypothetical protein
OsISM02642	LOC_Os01g18790	10596907	GAGCTTGGCGAGGTTGAG	CCTGGACTGTCTTCCTCCAG	59.7	209	78	conserved hypothetical protein
OsISM02643	LOC_Os01g18800	10622290	AACAAGATCCTCTCCCTCCG	CTTGAGCAGCTTGAGCGTC	60.3	158	67	CAMK KIN1/SNF1/Nim1 like.9 - CAMK includes calcium/calmodulin depedent protein kinases expressed
OsISM02644	LOC_Os01g18800	10622442	CTCAAGCACCCCAACGTC	AGCTCGCCTCCATTGACATA	60.5	1680	90	CAMK KIN1/SNF1/Nim1 like.9 - CAMK includes calcium/calmodulin depedent protein kinases expressed
OsISM02645	LOC_Os01g18800	10624101	ATATGTCAATGGAGGCGAGC	GCATCGATTAACTGCTGGAA	59.5	196	94	CAMK KIN1/SNF1/Nim1 like.9 - CAMK includes calcium/calmodulin depedent protein kinases expressed
OsISM02646	LOC_Os01g18800	10624295	GCTGTGAGCTATTGCCATGA	GCCTCTTCGATCCACAAGAA	60.2	250	76	CAMK KIN1/SNF1/Nim1 like.9 - CAMK includes calcium/calmodulin depedent protein kinases expressed
OsISM02647	LOC_Os01g18800	10624562	CTTTGGCCTAAGTGCTTTGC	GTTGTGTGCAGCAATCCATC	60.1	141	55	CAMK KIN1/SNF1/Nim1 like.9 - CAMK includes calcium/calmodulin depedent protein kinases expressed
OsISM02648	LOC_Os01g18800	10624683	GATGGATTGCTGCACACAAC	AATGAGCCATCGTAACCTCG	60.1	170	81	CAMK KIN1/SNF1/Nim1 like.9 - CAMK includes calcium/calmodulin depedent protein kinases expressed
OsISM02649	LOC_Os01g18800	10624905	CTTCCCTTTGATGACCGAAA	GATTTGAGTGTCCCCCTTGA	60.0	158	64	CAMK KIN1/SNF1/Nim1 like.9 - CAMK includes calcium/calmodulin depedent protein kinases expressed

ISM IDs	Rice gene locus IDs	Physical positions (bp) on chromosomes	Forward primers (5'-3')	Reverse primers (5'-3')	Melting tempearature (T˚C)	Expected product size (bp) in PCR	Expected product size (bp) in Q-PCR	Putative Functions
OsISM02650	LOC_Os01g18800	10625185	TGTTGTTCCATATGACGACGA	TTTCCTGCTTTGCTTCATCA	59.8	197	84	CAMK KIN1/SNF1/Nim1 like.9 - CAMK includes calcium/calmodulin depedent protein kinases expressed
OsISM02651	LOC_Os01g18800	10625425	TGCCCTTGATCTTTCAGGTT	TTTGGTGAATGTGTTGATGTGA	59.8	233	79	CAMK KIN1/SNF1/Nim1 like.9 - CAMK includes calcium/calmodulin depedent protein kinases expressed
OsISM02652	LOC_Os01g18800	10625694	GATGGGATTCCAAGTCCAGA	CCTGCCATTTTTCATGACCT	59.9	160	56	CAMK KIN1/SNF1/Nim1 like.9 - CAMK includes calcium/calmodulin depedent protein kinases expressed
OsISM02653	LOC_Os01g18800	10625834	AGGTCATGAAAAATGGCAGG	CAACATATAAGGATGGCCCG	60.1	193	97	CAMK KIN1/SNF1/Nim1 like.9 - CAMK includes calcium/calmodulin depedent protein kinases expressed
OsISM02654	LOC_Os01g18800	10626025	TGTCGAATTGAAGAAATCCCA	TGCTCTGTCTTGCACACTCC	60.3	219	91	CAMK KIN1/SNF1/Nim1 like.9 - CAMK includes calcium/calmodulin depedent protein kinases expressed
OsISM02655	LOC_Os01g18840	10642409	GATCAAGCTTGCTTCCAAGG	ACCTCCTCCTCCATGCTTCT	60.1	172	94	IF putative expressed
OsISM02656	LOC_Os01g18840	10642616	AGCAGCTGCGAACATCAATA	GGCTTGTGCCACTGAAATCT	59.9	237	93	IF putative expressed
OsISM02657	LOC_Os01g18840	10642996	GTTCCTTGAAGCGAAAGAGC	TCAGCTCATGAACCGTTGTC	59.5	194	63	IF putative expressed
OsISM02658	LOC_Os01g18840	10643227	ATCAAGAGCTCAGCTGCGAA	GGCAATCGGCTTTGTAGTTT	60.3	269	79	IF putative expressed
OsISM02659	LOC_Os01g18850	10650607	CACCGGTTCTGTCAACAGTG	GAGCGACAGCTCTTCTTTCC	59.8	1376	72	OsSPL1 - SBP-box gene family member expressed
OsISM02660	LOC_Os01g18850	10652092	TTGAAACAACTTTCTGGCCT	AGGACCATTGATTTGCTCAGA	58.6	158	55	OsSPL1 - SBP-box gene family member expressed
OsISM02661	LOC_Os01g18850	10652405	TCCTGTAGGCACTGAATCCAC	AAATTTTGCACACGCCTTTC	60.1	150	58	OsSPL1 - SBP-box gene family member expressed
OsISM02662	LOC_Os01g18850	10652534	GGAAAGGCGTGTGCAAAAT	GGCTCTTTCCCGAAGAGTTT	60.4	182	97	OsSPL1 - SBP-box gene family member expressed
OsISM02663	LOC_Os01g18850	10652696	AAACTCTTCGGGAAAGAGCC	TATCACTTGGGCAATTCGAC	59.2	199	83	OsSPL1 - SBP-box gene family member expressed
OsISM02664	LOC_Os01g18850	10652910	GGCCTGGCTGTATCATCCTA	GTATCCAGTGAGCTGGGTCG	60.4	431	85	OsSPL1 - SBP-box gene family member expressed
OsISM02665	LOC_Os01g18850	10653392	ATGCAAGGGTGCAGGACTAC	TGATGCTTGTTGCCTATTGC	60.0	212	89	OsSPL1 - SBP-box gene family member expressed
OsISM02666	LOC_Os01g18850	10653619	CCCTATTGCAGTTGCTTGTTC	TCCCACCAAAAATGCAAAGT	60.1	171	96	OsSPL1 - SBP-box gene family member expressed
OsISM02667	LOC_Os01g18850	10653847	GCCCTCAGTGTCTGACCTTC	GGAAATGAAAGGCTGCTTTG	59.8	172	95	OsSPL1 - SBP-box gene family member expressed

ISM IDs	Rice gene locus IDs	Physical positions (bp) on chromosomes	Forward primers (5'-3')	Reverse primers (5'-3')	Melting tempearature (T˚C)	Expected product size (bp) in PCR	Expected product size (bp) in Q-PCR	Putative Functions
OsISM02668	LOC_Os01g18850	10654530	CCTCTTCATATTGCAGCCAC	CGAGCATTCTTCCATGCTTT	59.3	463	99	OsSPL1 - SBP-box gene family member expressed
OsISM02669	LOC_Os01g18960	10714838	GGTTCTGTTCGATTGACTTGC	TTTGTGTGTATGGATGTGAAACA	59.3	375	95	hypothetical protein
OsISM02670	LOC_Os01g18990	10731607	ATGAAGGCTACACGAGCTGC	CAAAACGAAGCCAGTCAGGT	60.4	1178	75	retrotransposon protein putative Ty3-gypsy subclass
OsISM02671	LOC_Os01g19000	10734536	ACAAGGAACTGGAGTCGCAC	GAGGTTGCTGCTGCTGTTG	60.5	132	88	retrotransposon protein putative Ty3-gypsy subclass
OsISM02672	LOC_Os01g19000	10734952	GATGTGCTGACGACTCCAGA	ATCCCAAGGATCACATCCAA	60.1	290	60	retrotransposon protein putative Ty3-gypsy subclass
OsISM02673	LOC_Os01g19000	10736610	TCAGTCGGATCTGAATCTTCG	GAGTGTTGCAACGGCTTTTT	60.3	134	54	retrotransposon protein putative Ty3-gypsy subclass
OsISM02674	LOC_Os01g19000	10737631	AACAGCTACCAAGCCAGCAT	GAGCTTCCCTTTCGTCTGG	59.9	297	94	retrotransposon protein putative Ty3-gypsy subclass
OsISM02676	LOC_Os01g19040	10765121	AGCTATCCAAAATTACATGAACG	CCTTGGACTGTGGTGACAAA	58.5	1804	96	conserved hypothetical protein
OsISM02677	LOC_Os01g19040	10766868	AATTTTCTTCTTTGCCGCAG	TTCTTCGTAACAAGGTCCAGTATG	59.5	704	99	conserved hypothetical protein
OsISM02678	LOC_Os01g19040	10767524	CAGAGGCTTTTCCATAGTGGTT	CCCTTGTGCCAGAAACCTT	59.9	360	98	conserved hypothetical protein
OsISM02679	LOC_Os01g19040	10767855	GCAGGCTTTGAAGGTTTCTG	ATTCACTACGCATTTGGGCT	59.8	157	89	conserved hypothetical protein
OsISM02680	LOC_Os01g19040	10768558	TCAATGCCCACCTACTTTCC	ACCTTCCGGAACTGGAGATT	59.9	176	99	conserved hypothetical protein
OsISM02681	LOC_Os01g19040	10768822	CATAACCCAGGATTCCCCTT	TAGGGGAGATAGCTCTGGCA	60.0	175	94	conserved hypothetical protein
OsISM02682	LOC_Os01g19040	10769090	GTACGTGGAACTGTCGGGTC	TCGAATCCATCCTGAACGTA	59.8	179	63	conserved hypothetical protein
OsISM02683	LOC_Os01g19040	10769496	CCACAAACTACTCCACACATGC	GTATTTGCGCTTCTTCTGCC	60.2	402	90	conserved hypothetical protein
OsISM02684	LOC_Os01g19040	10770010	TGGAATCTAGGATTGCTGACG	CTGAAAGGATGGGAGCTCTG	60.1	183	98	conserved hypothetical protein
OsISM02685	LOC_Os01g19040	10770323	CTTGGTTGGAGGCATTTCAT	GGCTAGCTTCCCAAAATTCC	60.0	154	74	conserved hypothetical protein
OsISM02686	LOC_Os01g19040	10770478	TCAACTTGCACACTTTTCGG	CGTACAGCACGTAATGATTGAA	59.3	191	99	conserved hypothetical protein

ISM IDs	Rice gene locus IDs	Physical positions (bp) on chromosomes	Forward primers (5'-3')	Reverse primers (5'-3')	Melting tempearature (T°C)	Expected product size (bp) in PCR	Expected product size (bp) in Q-PCR	Putative Functions
OsISM02687	LOC_Os01g19040	10770838	TTGGGACTTTGAGACAGTTGAA	CGACGACCATCGTAGTTCAC	59.5	175	97	conserved hypothetical protein
OsISM02688	LOC_Os01g19040	10771006	GTCGTCGTCTTCTGAACCCT	CGCAGAGTCAAAAGGTGGAT	59.8	168	77	conserved hypothetical protein
OsISM02689	LOC_Os01g19100	10790847	GCAAAGGCGATGGTTAGAAT	GGTTGAATGTCCAGGGTGTT	59.4	213	64	retrotransposon protein putative Ty3-gypsy subclass
OsISM02690	LOC_Os01g19110	10796103	CAGGAGGGAGATGAAGACGA	CAGTACCATTTCCCTGGATCA	60.1	261	75	transposon protein putative CACTA En/Spm sub-class
OsISM02691	LOC_Os01g19110	10796935	CGCCCTCGACGTCTCTATTA	ATCCAGTATCGAAAACCCCC	60.2	190	81	transposon protein putative CACTA En/Spm sub-class
OsISM02692	LOC_Os01g19110	10797205	AAGGCGCAGCATTACAAGAC	GCCGACAGGTCGAAGAGTAA	60.4	177	81	transposon protein putative CACTA En/Spm sub-class
OsISM02693	LOC_Os01g19110	10797422	TCTACGTTTGACAAGGTTTTCG	GTCTTTCTCTCCATTTGCCG	59.6	169	83	transposon protein putative CACTA En/Spm sub-class
OsISM02694	LOC_Os01g19110	10797571	CGGCAAATGGAGAGAAAGAC	GGCAATATTCGCACACGTAA	59.7	195	99	transposon protein putative CACTA En/Spm sub-class
OsISM02695	LOC_Os01g19110	10797764	CCACTGTCTTGCAGACCAAA	GTGCATGGTCAGGTTATCCC	60.0	155	74	transposon protein putative CACTA En/Spm sub-class
OsISM02696	LOC_Os01g19150	10823212	GAGCCACTGATCGTCTGACA	TTGCCACTGACCACTACAGC	59.9	169	80	CGMC GSK.3 - CGMC includes CDA MAPK GSK3 and CLKC kinases expressed
OsISM02697	LOC_Os01g19150	10823413	CTGTTGGTGGAAGAAATGGG	CCACCTGGAAGACAACTCCA	60.7	273	94	CGMC GSK.3 - CGMC includes CDA MAPK GSK3 and CLKC kinases expressed
OsISM02698	LOC_Os01g19150	10823655	CGTGGTTCATTTGGAGTTGTC	CCGGTTCTTGTATCTCGCAT	60.2	172	97	CGMC GSK.3 - CGMC includes CDA MAPK GSK3 and CLKC kinases expressed
OsISM02699	LOC_Os01g19150	10823973	CAATAAGATGAGCCAACGCA	ATGAATGTATGCCAAAGCCC	59.8	183	80	CGMC GSK.3 - CGMC includes CDA MAPK GSK3 and CLKC kinases expressed
OsISM02700	LOC_Os01g19150	10824161	TGTGGGAGTATGCCACAGAG	TTGCACTGCCAAAGTCACAC	60.3	207	90	CGMC GSK.3 - CGMC includes CDA MAPK GSK3 and CLKC kinases expressed
OsISM02701	LOC_Os01g19150	10824343	AAATTGTGTGACTTTGGCAGTG	CCAAATATGAGTTCTGGGGC	59.7	218	99	CGMC GSK.3 - CGMC includes CDA MAPK GSK3 and CLKC kinases expressed
OsISM02702	LOC_Os01g19150	10824594	GTCTGCTGGCTGTGTTCTTG	GCCACTGTCACCAGGAAAAA	60.3	143	62	CGMC GSK.3 - CGMC includes CDA MAPK GSK3 and CLKC kinases expressed
OsISM02703	LOC_Os01g19150	10824736	CGTCGACCAACTTGTTGAAA	TTGATTTCTTCCCTTGTTGGA	59.6	152	58	CGMC GSK.3 - CGMC includes CDA MAPK GSK3 and CLKC kinases expressed
OsISM02704	LOC_Os01g19150	10824924	CCAAATCAAAGCTCACCCAT	TGAGTACTGCAGAAGCCGAG	59.6	198	89	CGMC GSK.3 - CGMC includes CDA MAPK GSK3 and CLKC kinases expressed

ISM IDs	Rice gene locus IDs	Physical positions (bp) on chromosomes	Forward primers (5'-3')	Reverse primers (5'-3')	Melting tempearature (T˚C)	Expected product size (bp) in PCR	Expected product size (bp) in Q-PCR	Putative Functions
OsISM02705	LOC_Os01g19150	10825115	GTACTCACCACACCTCCGGT	CCATTTGGTAAGCGAGCATT	60.0	180	91	CGMC GSK.3 - CGMC includes CDA MAPK GSK3 and CLKC kinases expressed
OsISM02706	LOC_Os01g19150	10825269	GATCCAAATGCTCGCTTACC	TTCCATTGATGCTCCTTTCA	59.4	172	85	CGMC GSK.3 - CGMC includes CDA MAPK GSK3 and CLKC kinases expressed
OsISM02707	LOC_Os01g19170	10829671	GAAATGGTGGGATCTTCCCT	GGTTAAAAGGTCGTTGCGTT	59.6	208	99	polygalacturonase putative expressed
OsISM02708	LOC_Os01g19170	10829812	GGGGGAAACACTCATGGAC	CGTCACGTTGTTGCTGATG	60.0	163	67	polygalacturonase putative expressed
OsISM02709	LOC_Os01g19180	10835878	GGTGGTGGAAGAGGCGAC	ACCACCACTCAAACTCTGCC	60.9	129	51	hypothetical protein
OsISM02710	LOC_Os01g19180	10836015	GGTGCATCAGCCGTAGATTC	CACACGACCAGGAGAAGGTT	60.4	330	84	hypothetical protein
OsISM02712	LOC_Os01g19190	10854759	AAGGCAGCGGATGGTCAC	GGTCTCCAGGCGAAGACG	62.1	162	88	retrotransposon protein putative unclassified
OsISM02713	LOC_Os01g19220	10872248	CGTGCTCTTCGGAAAATACA	CGGGATCTTGCTCACGTACT	59.6	154	77	beta-D-xylosidase putative expressed
OsISM02715	LOC_Os01g19240	10876742	CTACCGGAATTTCATCGCAG	TTGAGGCCCACGAAGTAGAG	60.5	2779	94	integral membrane protein DUF6 containing protein
OsISM02716	LOC_Os01g19240	10879829	GGGCCATCTGGTTCATCAT	AAGCGAAGCTCTTGGACAGA	60.5	119	46	integral membrane protein DUF6 containing protein
OsISM02717	LOC_Os01g19240	10880049	CTTGACATCAGGCTCGTCG	ACATCACCGTGCACACCAT	60.7	160	68	integral membrane protein DUF6 containing protein
OsISM02718	LOC_Os01g19240	10880294	CATCCTCGGCGAGAAGATAC	GTGTACAGCCCCACGACAAT	60.3	207	67	integral membrane protein DUF6 containing protein
OsISM02719	LOC_Os01g19260	10891443	CGAGATCCTCTTCAGCAACC	CGATGTAGCTCCATGCTTTG	59.7	169	93	ATPase putative expressed
OsISM02721	LOC_Os01g19290	10910622	CCCCTTCGCCTACTACTTCG	CCCAAAAATGGAGGAGAAGAA	60.8	1012	86	integral membrane protein DUF6 containing protein expressed
OsISM02722	LOC_Os01g19290	10911568	AGGAAGAGTGGCATGGTGAT	CCACGAAGTAGAGCACCTGG	60.2	853	95	integral membrane protein DUF6 containing protein expressed
OsISM02723	LOC_Os01g19290	10912888	TTCGACATCCGCCTCTACTC	GGATGCACCACGACATGA	60.2	173	80	integral membrane protein DUF6 containing protein expressed
OsISM02724	LOC_Os01g19320	10930696	GGGAAGAAGAAGAGGGGAAA	TGTGACAACAACCCTTGCTC	59.7	1099	70	hypothetical protein
OsISM02725	LOC_Os01g19360	10953179	CACCGCGGGACTACCTAAG	CATCACGAGATTCCATGATTGT	60.2	319	99	retrotransposon protein putative Ty1-copia subclass

ISM IDs	Rice gene locus IDs	Physical positions (bp) on chromosomes	Forward primers (5'-3')	Reverse primers (5'-3')	Melting tempearature (T˚C)	Expected product size (bp) in PCR	Expected product size (bp) in Q-PCR	Putative Functions
OsISM02726	LOC_Os01g19360	10953742	TGCATCTCCTGATGCAGACT	CACCCATAGGGTCGTTCTGT	59.7	102	55	retrotransposon protein putative Ty1-copia subclass
OsISM02727	LOC_Os01g19360	10954073	AATGGAGAGCTGGATGAGGA	CCCCATCATGGCGATAGTA	59.5	219	86	retrotransposon protein putative Ty1-copia subclass
OsISM02728	LOC_Os01g19380	10964444	CTGCGGTTGTTCAAGACGAT	TGCATGGAGTGCAATAGAGC	60.6	392	73	pentatricopeptide putative
OsISM02729	LOC_Os01g19380	10965497	TGCAGCCTAACAAGGAGGTT	TGTAAAAGCATACTCGCCCC	60.0	278	81	pentatricopeptide putative
OsISM02730	LOC_Os01g19380	10965784	TTGAGAGGCATAGGGAGCAG	TGCACCATACTCAGCCTTCTT	60.2	1257	79	pentatricopeptide putative
OsISM02731	LOC_Os01g19400	10976259	GAGATCCTTCCACTCCCAGC	GTTGAGGAACTCCACCACG	60.1	140	56	expressed protein
OsISM02732	LOC_Os01g19400	10976493	TCAAGAAGCTCGGCATCC	GCTTCCAAAGACCAAGACGA	60.2	2053	79	expressed protein
OsISM02733	LOC_Os01g19440	10996778	GATCCGGATTCACATCGC	AGATGAAGCTCTTGTTCGCC	59.8	360	69	pectinesterase putative
OsISM02735	LOC_Os01g19440	10997713	GTGTTCTACCGGACAGCCAT	ACTCCACCATCTCCAGCGT	60.3	421	95	pectinesterase putative
OsISM02736	LOC_Os01g19460	11013326	CAAGCGAAAGGTCCCTATGT	AATTGTCCAGCACAAAAGGC	59.7	172	91	retrotransposon protein putative unclassified
OsISM02737	LOC_Os01g19460	11013670	ACTCTTGCTGCCGATGAAAC	GCCTTACGTTTTCTACGCGA	60.4	286	81	retrotransposon protein putative unclassified
OsISM02738	LOC_Os01g19460	11013936	TCGCGTAGAAAACGTAAGGC	CAGATCGAGATGAAGGGGAG	60.1	167	83	retrotransposon protein putative unclassified
OsISM02739	LOC_Os01g19460	11014139	GCCAACAATGCCATACACAG	GACCAGCAAATGGTTGCCTA	60.5	185	79	retrotransposon protein putative unclassified
OsISM02740	LOC_Os01g19460	11014303	CTAGGCAACCATTTGCTGGT	ATCACCATACATAAGCGCCA	59.6	332	94	retrotransposon protein putative unclassified
OsISM02741	LOC_Os01g19460	11014579	AATGATAATTGCCACGGCTC	CTGGAGGTGGCTTGTATGGT	60.0	312	90	retrotransposon protein putative unclassified
OsISM02742	LOC_Os01g19460	11015518	GGGAAGATGGCTTCCATAAA	TCATGCAAGGAGACGATCTG	59.5	932	52	retrotransposon protein putative unclassified
OsISM02743	LOC_Os01g19460	11016430	CAGATCGTCTCCTTGCATGA	ATGTTGTTATTTGGCCGAGC	60.0	120	69	retrotransposon protein putative unclassified
OsISM02744	LOC_Os01g19460	11016849	TCAACCAAAGGTCCTGGCTA	GCTTGGCCTGTAGCAATTTT	60.0	599	94	retrotransposon protein putative unclassified

ISM IDs	Rice gene locus IDs	Physical positions (bp) on chromosomes	Forward primers (5'-3')	Reverse primers (5'-3')	Melting tempearature (T˚C)	Expected product size (bp) in PCR	Expected product size (bp) in Q-PCR	Putative Functions
OsISM02745	LOC_Os01g19460	11017430	AATTGCTACAGGCCAAGCAC	TGCTGGTAAGCATGTGGGTA	60.2	167	99	retrotransposon protein putative unclassified
OsISM02746	LOC_Os01g19460	11017993	TGAGGACTTTGCGTATCGTG	GGCCAACAGGGTATTCCATA	59.8	370	68	retrotransposon protein putative unclassified
OsISM02747	LOC_Os01g19480	11058594	GAGGGCGTCGTCTACGTCT	GTCTCCAGGTTCAGCGTCAG	60.7	1357	75	kelch motif family protein putative expressed
OsISM02748	LOC_Os01g19480	11060392	GGTGGACGTGCTCGACAT	CAGGTGAGGCCCCTTTTACT	60.6	569	63	kelch motif family protein putative expressed
OsISM02749	LOC_Os01g19480	11060976	ACGCTGCTGTAGGTGTTGG	CCGATTTGCAACATCAAGAA	59.8	197	96	kelch motif family protein putative expressed
OsISM02750	LOC_Os01g19480	11061231	CGGTTGTCCTGAATACCGAT	AGCCCAACTGAAGAATGAGC	59.6	167	88	kelch motif family protein putative expressed
OsISM02751	LOC_Os01g19480	11061462	GGAGAGGCGAAAATTCTTGA	TTGTTCGATTTCTGCTTCCC	59.8	177	95	kelch motif family protein putative expressed
OsISM02752	LOC_Os01g19480	11061631	TCGAACAAAGAAACCCGTTC	GAACATCGCCAGTTCTCCTC	59.9	158	70	kelch motif family protein putative expressed
OsISM02753	LOC_Os01g19480	11062048	GTTCCTAGCAGCTGAACCCA	CCTGAATCGAAAGCTCCATC	60.1	183	97	kelch motif family protein putative expressed
OsISM02754	LOC_Os01g19480	11062263	TCTTCAGAGGCGTCCTTTTC	GTCTCGGAGCCGTTACTCC	59.7	152	49	kelch motif family protein putative expressed
OsISM02755	LOC_Os01g19580	11095281	TTGCTTAGTGGGAGCTCGAT	TGTCGAAGTTGTTTCCTCCC	60.0	1050	95	hypothetical protein
OsISM02756	LOC_Os01g19580	11096270	TTTGATAGGAGGATGGCGAC	CTTGCTGCTACCTTGGGAAG	60.0	140	90	hypothetical protein
OsISM02757	LOC_Os01g19580	11096364	TGTCCTATCGTGTTGCTTGC	CTAGCAACCTCCACTCTCCG	59.9	206	71	hypothetical protein
OsISM02758	LOC_Os01g19580	11096764	CATTGGCATTGCAACCTCTA	GGAGGTTGGGGATCACCTTA	60.4	636	70	hypothetical protein
OsISM02759	LOC_Os01g19620	11124999	GCGTGACAGGAGGAGGAAG	CCGCCAATCTTGAATAGCTT	60.1	1025	86	retrotransposon protein putative unclassified
OsISM02760	LOC_Os01g19650	11144016	TGCTGTAATAGGCGCTGAGTT	GGAATACCAGGCATATCGGA	59.9	111	64	retrotransposon protein putative Ty3-gypsy subclass
OsISM02761	LOC_Os01g19650	11144368	CCCAAAGATCCTTTTGGGTT	GGTCATCCTTCTGCTTGGTC	59.9	307	98	retrotransposon protein putative Ty3-gypsy subclass
OsISM02762	LOC_Os01g19680	11159194	GTCACCATGTGCATGAGCTT	GCGTACTTAGGAGTGCAGCC	59.9	543	66	retrotransposon protein putative unclassified

ISM IDs	Rice gene locus IDs	Physical positions (bp) on chromosomes	Forward primers (5'-3')	Reverse primers (5'-3')	Melting tempearature (T˚C)	Expected product size (bp) in PCR	Expected product size (bp) in Q-PCR	Putative Functions
OsISM02763	LOC_Os01g19680	11159718	GCTGCACTCCTAAGTACGCC	GATGTACCCAGAAAATGGCA	59.2	2814	74	retrotransposon protein putative unclassified
OsISM02764	LOC_Os01g19694	11166384	CCTCCTCTCCGCCTACATC	GATCTCCTTCAGCAGCGAC	59.2	231	71	Homeobox domain containing protein expressed
OsISM02766	LOC_Os01g19694	11166966	ACGCAGCTCAGCAACCTC	AGCACTGGTCTTCGTCGG	60.1	5387	98	Homeobox domain containing protein expressed
OsISM02767	LOC_Os01g19694	11172543	ACACTACCGCTGGCCATATC	TTGGGTCAAGACCTGTCCTC	60.0	326	69	Homeobox domain containing protein expressed
OsISM02768	LOC_Os01g19760	11211164	CTCCACACCAAGGAGGAATG	TTCGGATGCAGTAATCCACA	60.3	1394	50	expressed protein
OsISM02769	LOC_Os01g19760	11212582	TTGCTCGTAGAGTGTGCAGG	AGTCTGCAAGATGATACGGG	59.0	769	94	expressed protein
OsISM02770	LOC_Os01g19760	11213359	CGAGTTTCGAGGATCTCACC	GCAGCAGTGAAGTTTGGAAT	58.9	752	99	expressed protein
OsISM02771	LOC_Os01g19760	11214112	GATGCCTTGAGGCTTACTGG	GCAATTGACTTATTCAGCTTCCA	60.2	585	99	expressed protein
OsISM02772	LOC_Os01g19760	11214716	CAGCCTGTTGACTTCCCAAT	TGTTGCAGTCTCTTCCTCTGAA	60.1	266	97	expressed protein
OsISM02773	LOC_Os01g19760	11215074	TGATGTGCCTGTTTACCCTG	AAACAAAACCTTCGAGCCTG	59.5	432	57	expressed protein
OsISM02774	LOC_Os01g19760	11215481	GTTTCCAGGCTCGAAGGTTT	GCATTGGCACTTGACACAAC	60.4	206	99	expressed protein
OsISM02775	LOC_Os01g19760	11215972	TTCGTACTTGATGATGGGCTC	TCTGCAATGCAAGAATCACC	59.9	1527	94	expressed protein
OsISM02776	LOC_Os01g19760	11218918	CCAAACAGAAGTCGTGGGAT	TAAAGATTTGGTGCCAGCCT	59.8	200	86	expressed protein
OsISM02777	LOC_Os01g19820	11244588	CAACGTCGTCTCCTCCTCC	GTTGGCCTCGATGAATTAGG	60.2	169	64	universal stress protein domain containing protein putative expressed
OsISM02778	LOC_Os01g19820	11244778	AGGGTACATGATGACCTCGG	CGCATCGCATCTTTGAAGTA	59.9	160	83	universal stress protein domain containing protein putative expressed
OsISM02779	LOC_Os01g19820	11244968	GTGGAGACGATGGTGGAGAG	CCCACGTAAGTATACATGCCAA	60.2	401	97	universal stress protein domain containing protein putative expressed
OsISM02780	LOC_Os01g19870	11272153	CGTGCTCTTCTGCTTCCTCT	AGCTCCGGACTTGATCAGAA	59.9	1656	99	hydrolase acting on carbon-nitrogen putative expressed
OsISM02781	LOC_Os01g19870	11274011	TCCACCTATTGACAGGAAACG	CCTGCTCAACAGGCACATAA	59.9	148	66	hydrolase acting on carbon-nitrogen putative expressed

ISM IDs	Rice gene locus IDs	Physical positions (bp) on chromosomes	Forward primers (5'-3')	Reverse primers (5'-3')	Melting tempearature (T˚C)	Expected product size (bp) in PCR	Expected product size (bp) in Q-PCR	Putative Functions
OsISM02782	LOC_Os01g19870	11274281	TCATTGGAAGAGCGAGACAA	CCCAGTATTGACACCGGGTA	60.1	219	94	hydrolase acting on carbon-nitrogen putative expressed
OsISM02783	LOC_Os01g19870	11274538	GTGCCAGGGGATAGTTGTTG	GAAGCAAACTGTATCCTTTGAAAAA	60.2	173	98	hydrolase acting on carbon-nitrogen putative expressed
OsISM02784	LOC_Os01g19870	11274814	TTCCATATTCAGATGCAAGGC	TCCAAATTGAATACCGCACA	60.0	701	85	hydrolase acting on carbon-nitrogen putative expressed
OsISM02785	LOC_Os01g19870	11275505	TCAATTTGGATTGATGGGAAA	GCCTGGAAATAGCGAAGCTAA	60.5	304	98	hydrolase acting on carbon-nitrogen putative expressed
OsISM02786	LOC_Os01g19870	11275930	TTCAGCTAAGCTTCCTATCACG	AGGGATGTGCTCCCGTATAA	59.1	140	65	hydrolase acting on carbon-nitrogen putative expressed
OsISM02787	LOC_Os01g19870	11276190	ATGTTCCTGGATTGCGAAAG	GGAGGCGGACTTCGTAGTATT	59.9	220	84	hydrolase acting on carbon-nitrogen putative expressed
OsISM02788	LOC_Os01g19940	11315834	GTTGGACTACTCTCGCGCTC	TCCATATTTGTTCTGGGACGA	60.2	2016	96	expressed protein
OsISM02789	LOC_Os01g19940	11317812	TGAATTGTTTCCAGGGATCG	GAATCGAAATAGGCACGCTC	60.3	230	95	expressed protein
OsISM02790	LOC_Os01g19940	11318179	ACCTATTTCTGCAGCAAGGC	GTGGAGGAAGCTGGGGATA	59.8	536	97	expressed protein
OsISM02792	LOC_Os01g19960	11325867	CTCTAGTTCGCGCCAGAAAC	TGCCAGTTGTACCATCGTGT	60.1	279	91	transposon protein putative CACTA En/Spm sub-class
OsISM02793	LOC_Os01g19960	11326256	GACAGTGGGATGACAACGGT	CTTTTTCCTGGGAGAACCAC	59.7	158	99	transposon protein putative CACTA En/Spm sub-class
OsISM02794	LOC_Os01g19960	11326533	CACGTAGCGATCACAAGGAA	CAGTACTGGCTGCTTCCCTC	59.9	3154	92	transposon protein putative CACTA En/Spm sub-class
OsISM02795	LOC_Os01g19960	11330066	CTCGAGACGTTCACGCTACC	TCTCCCTTGAAGCTCTGGAA	60.5	95	48	transposon protein putative CACTA En/Spm sub-class
OsISM02796	LOC_Os01g19960	11330297	AATGCCGCCAAGAAGAAGTA	AGATCTGCAATCTTCGCCTC	59.7	440	81	transposon protein putative CACTA En/Spm sub-class
OsISM02797	LOC_Os01g19960	11330830	CCAGCCGTACATTCCTCCT	GAGTATCCTGCTGGAATCGG	59.9	355	97	transposon protein putative CACTA En/Spm sub-class
OsISM02798	LOC_Os01g19960	11332171	CCCTCGACGTCTCTCTTCTG	GAGGGTCGATGAACCCAGTA	60.0	181	91	transposon protein putative CACTA En/Spm sub-class
OsISM02799	LOC_Os01g19960	11332420	ATGCAGCAGCATTTCAAGAC	GACCTTGTCAAAAATCTTCTCCTC	59.3	230	97	transposon protein putative CACTA En/Spm sub-class
OsISM02800	LOC_Os01g19960	11332637	TTTTGACAAGGTCTTCAAACTGA	GAAAATGAAACCTCCGTCCA	59.4	202	99	transposon protein putative CACTA En/Spm sub-class

ISM IDs	Rice gene locus IDs	Physical positions (bp) on chromosomes	Forward primers (5'-3')	Reverse primers (5'-3')	Melting tempearature (T˚C)	Expected product size (bp) in PCR	Expected product size (bp) in Q-PCR	Putative Functions
OsISM02801	LOC_Os01g19960	11332814	AAACTTGGACGGAGGTTTCA	GATAGGCAGTGGGCAAACTC	59.6	190	93	transposon protein putative CACTA En/Spm sub-class
OsISM02803	LOC_Os01g19970	11349810	GGACATCATCCTCAACCTCC	TCTTGATCTCATTGTCCGTCC	59.7	5152	90	MYB family transcription factor putative expressed
OsISM02804	LOC_Os01g19980	11357164	ACAGATGGATTGATCGAGGC	TCGGGAACTCTGAAAACCAT	59.8	348	58	hypothetical protein
OsISM02805	LOC_Os01g19980	11357548	CCAAAAATGGTTTTCGGAAT	AAATGTGCTCTTTGGATGCC	59.4	1203	82	hypothetical protein
OsISM02806	LOC_Os01g20020	11371859	AGGAGGGAGGAGAGGGAAAC	GGCCAGAGCTAGCAGAGAGA	60.5	1022	94	hypothetical protein
OsISM02807	LOC_Os01g20020	11372809	AACAGCATGAAAGGGACTGG	AAGCGAATAATGCTATGTCCTTG	59.9	569	74	hypothetical protein
OsISM02808	LOC_Os01g20040	11382403	CACTTGTGCGAGATGTTCGT	AATCTCTCCCACTTCTTGCG	59.7	169	49	retrotransposon protein putative unclassified
OsISM02810	LOC_Os01g20110	11411868	ACCAAACATGCTTCATTCCC	CACCTTCAGTGCAGTCATCC	59.5	3595	96	expressed protein
OsISM02811	LOC_Os01g20110	11415642	TCAATGAAGCAAAAGTTGGTT G	GCGGAGCTGTGTGAAGAGTT	60.4	320	91	expressed protein
OsISM02812	LOC_Os01g20110	11416015	TGGAGGGTTCTTCCAACATC	TTGCAGACAGGCACTTCG	59.8	602	99	expressed protein
OsISM02813	LOC_Os01g20110	11416786	GGCTGCTGCAAAAGACTCTA	AGGCGAGATGCTCTTGAGAC	59.1	1751	98	expressed protein
OsISM02814	LOC_Os01g20110	11418600	CTTGCAACAGCCTTTTCTCC	GGTCCATTTGTGGAGTTCGT	59.9	901	88	expressed protein
OsISM02816	LOC_Os01g20130	11441107	CTTCCTCCAGCCTGCTACAC	TATTCCTTATTGCAACGGGC	60.0	199	95	transposon protein putative CACTA En/Spm sub-class
OsISM02817	LOC_Os01g20150	11445443	CCAGCCACAGAGGCAGTT	TCGACGTATGTTGTTCCTGC	59.9	182	92	transposon protein putative CACTA En/Spm sub-class
OsISM02818	LOC_Os01g20150	11445993	CATACGCCGTTCGATGAATA	CTCCTTGCCGATGTACCCT	59.8	168	88	transposon protein putative CACTA En/Spm sub-class
OsISM02819	LOC_Os01g20150	11446295	TGAGAACAAAAGTGATGCCG	CCTCGCTTTGTGAAGCTTGT	60.2	152	72	transposon protein putative CACTA En/Spm sub-class
OsISM02820	LOC_Os01g20150	11446833	CTCCCGATCGCACCACTA	AGTTGGGTGGACTCTCGCTA	60.3	162	79	transposon protein putative CACTA En/Spm sub-class
OsISM02821	LOC_Os01g20150	11447748	CGGAAGTTGGACACTGGTCT	GATCAAGGAACCCAACCTGA	60.0	173	89	transposon protein putative CACTA En/Spm sub-class

ISM IDs	Rice gene locus IDs	Physical positions (bp) on chromosomes	Forward primers (5'-3')	Reverse primers (5'-3')	Melting tempearature (T˚C)	Expected product size (bp) in PCR	Expected product size (bp) in Q-PCR	Putative Functions
OsISM02822	LOC_Os01g20150	11447994	GGCCCATCAAGACAAGGAGT	CGATGACTAGGAGAATCCAATG	60.1	150	69	transposon protein putative CACTA En/Spm sub-class
OsISM02823	LOC_Os01g20170	11471449	CCCAATCCATTTTACACCGA	TGATTGCAATTGATGGAAGG	60.0	1206	95	hypothetical protein
OsISM02824	LOC_Os01g20170	11472639	CCATCAATTGCAATCAAGACC	AGAGGAGGTCTCGGTGGTTT	60.2	389	90	hypothetical protein
OsISM02825	LOC_Os01g20170	11473139	AGTTGTGCCGGAAAATGAAC	ATTCCAAACTTGGTGCGACT	59.8	95	50	hypothetical protein
OsISM02826	LOC_Os01g20170	11473387	TGGATGGTATCCACCTTCAGA	GCGTCACGGTATGTTGTATTTG	60.3	489	92	hypothetical protein
OsISM02827	LOC_Os01g20208	11514317	GAAGCTTCGCGAGACTGG	AGCTTCCATATCCGAGCAAA	59.8	444	50	expressed protein
OsISM02828	LOC_Os01g20700	11516757	TGAAGCGTACAAGGAGGAGC	AGGCCTGTCTCATTCATTGAC	59.8	2705	88	hypothetical protein
OsISM02829	LOC_Os01g20700	11519438	CAAGTCAATGAATGAGACAGGC	TCAACATGTGAGGTTTGTCG	58.9	213	94	hypothetical protein
OsISM02830	LOC_Os01g20700	11520144	CCACCTGATCATCACATCCA	TGCCTTCTGCCGTGTACATA	60.3	238	92	hypothetical protein
OsISM02831	LOC_Os01g20750	11558284	GACAGCGAGTTTGACAAGCA	TCGACATCAAGGAGGTGACA	60.2	258	99	hypothetical protein
OsISM02832	LOC_Os01g20760	11561997	GCATGAAGGAAGATTGTCCG	AACCTGGTTGTTCCCATCAC	60.1	557	96	transposon protein putative unclassified
OsISM02833	LOC_Os01g20760	11563827	GTCCATCTGGACAGGCAAGT	GTGGTTCCTGTCAATCCCAC	60.2	515	75	transposon protein putative unclassified
OsISM02834	LOC_Os01g20760	11564322	GTGGGATTGACAGGAACCAC	CGCCTCAACAAGCCTACAA	60.1	236	75	transposon protein putative unclassified
OsISM02835	LOC_Os01g20760	11564853	CTAGCAAGGCTTGGTTGCTC	AACCACAACAGGTACGCCTC	60.1	184	98	transposon protein putative unclassified
OsISM02836	LOC_Os01g20760	11565347	ACGATGGGGACTTACTGGTG	GCTGGAGACGGTAAAACTCG	59.9	191	98	transposon protein putative unclassified
OsISM02837	LOC_Os01g20760	11565518	CGAGTTTTACCGTCTCCAGC	AACACCTGCCTAAGTCCGAA	59.8	205	88	transposon protein putative unclassified
OsISM02838	LOC_Os01g20760	11565705	CGGACTTAGGCAGGTGTTTC	CTAGCTGTCCCCGTCGAGT	60.1	159	81	transposon protein putative unclassified
OsISM02839	LOC_Os01g20760	11566075	CCGTGACCAAGACTACTTCG	ATATCGGCACTGATATCCCG	59.1	149	55	transposon protein putative unclassified

ISM IDs	Rice gene locus IDs	Physical positions (bp) on chromosomes	Forward primers (5'-3')	Reverse primers (5'-3')	Melting tempearature (T°C)	Expected product size (bp) in PCR	Expected product size (bp) in Q-PCR	Putative Functions
OsISM02841	LOC_Os01g20760	11566783	TCTTCCTCTTACGGAGCGTC	GACTAGTCCTACCGGCGTGA	59.9	160	98	transposon protein putative unclassified
OsISM02842	LOC_Os01g20760	11566982	ACCACCACGACGTACTAGGC	TCGAGTAAATCCACCCATCC	59.9	743	99	transposon protein putative unclassified
OsISM02843	LOC_Os01g20830	11610144	TCAACTTCAGTGATGGCTGC	TCTTCCTGATCTTGTGCTCG	59.6	219	95	heavy metal-associated domain containing protein expressed
OsISM02844	LOC_Os01g20830	11610315	TTCATGTGAGGATGGACTGC	TCGCTGCATCTACGTACACC	59.8	316	94	heavy metal-associated domain containing protein expressed
OsISM02845	LOC_Os01g20840	11617730	CGTTCGGGATCAAGTACCTC	TCAGGCCCTTTCAGTTCAAT	59.6	913	92	lipase class 3 family protein putative expressed
OsISM02846	LOC_Os01g20840	11618734	GGATTTTCCCCAGAGGATGT	CATCACGCAGGATTGTATGG	60.0	204	77	lipase class 3 family protein putative expressed
OsISM02847	LOC_Os01g20840	11619140	TCAAGCAGTCAGTCAATGCC	CAGTACCACCACCCAATGAA	59.6	479	66	lipase class 3 family protein putative expressed
OsISM02848	LOC_Os01g20840	11619667	CAACTTGTGTTGCCTTTGCT	CTCACAAAATGCTTGCCTGA	59.5	143	71	lipase class 3 family protein putative expressed
OsISM02849	LOC_Os01g20840	11619819	AAACGGTGCTGATCTGGTTC	CATTTAACCATGAAGATGCCG	60.2	339	84	lipase class 3 family protein putative expressed
OsISM02850	LOC_Os01g20840	11620276	TGCACTGCTACGACCTGTTT	ACTTCTAGCAACAGCCTGCG	60.1	310	80	lipase class 3 family protein putative expressed
OsISM02851	LOC_Os01g20880	11654526	TTATGAAGAAGGGGTGGCTG	CTTTGAAAAACCACATGCCC	60.2	449	70	OsWAK3 - OsWAK receptor-like cytoplasmic kinase OsWAK-RLCK
OsISM02852	LOC_Os01g20880	11656732	GTGCAGCGATATCTTTTGGC	AGGGGCTACATGTTGGTTTG	60.3	1256	78	OsWAK3 - OsWAK receptor-like cytoplasmic kinase OsWAK-RLCK
OsISM02853	LOC_Os01g20880	11658137	TGGGCAGAAATCTGCAATAA	TGGACAATGTTGTCATGCTTC	59.4	200	97	OsWAK3 - OsWAK receptor-like cytoplasmic kinase OsWAK-RLCK
OsISM02854	LOC_Os01g20880	11658394	AAAGGGTTCCTTGCACGATA	AGCAATCTTCACTCGCTGCT	59.9	227	89	OsWAK3 - OsWAK receptor-like cytoplasmic kinase OsWAK-RLCK
OsISM02855	LOC_Os01g20900	11677931	TGATGTTCTTTCCCGTCTCC	GCTAGGTCCATTATCGCCAC	59.8	1268	98	OsWAK4 - OsWAK receptor-like cytoplasmic kinase OsWAK-RLCK expressed
OsISM02856	LOC_Os01g20900	11679379	TCTGCAGTAAAGAAGCTCAATCC	GGACAACATTGTCATGCTTCA	59.8	188	86	OsWAK4 - OsWAK receptor-like cytoplasmic kinase OsWAK-RLCK expressed
OsISM02857	LOC_Os01g20900	11679612	ATGAGTATGCACCAAAGGGC	GCTGCACCCATGATAGAGGT	60.0	244	94	OsWAK4 - OsWAK receptor-like cytoplasmic kinase OsWAK-RLCK expressed
OsISM02858	LOC_Os01g20900	11680048	TTCCACAGATTTTTCGCTATGA	TACAACCCCAAAGGCGTAGA	60.1	201	87	OsWAK4 - OsWAK receptor-like cytoplasmic kinase OsWAK-RLCK expressed

ISM IDs	Rice gene locus IDs	Physical positions (bp) on chromosomes	Forward primers (5'-3')	Reverse primers (5'-3')	Melting tempearature (T˚C)	Expected product size (bp) in PCR	Expected product size (bp) in Q-PCR	Putative Functions
OsISM02859	LOC_Os01g20900	11680300	GGCCAGATGAGCCTTGTAAA	TCCAAGCTTTGGATCTACGC	60.3	163	79	OsWAK4 - OsWAK receptor-like cytoplasmic kinase OsWAK-RLCK expressed
OsISM02860	LOC_Os01g20930	11687950	GAGGAGTGTGGCGAAGTTG	CCCAAACTTCCAATTCCAAA	59.6	1393	87	RING-H2 finger protein putative expressed
OsISM02861	LOC_Os01g20940	11693147	AGAGGAGGAGGAGGAGGAGG	GGTTTCGATCTCTTTCCTCG	60.1	968	75	PHS1 putative expressed
OsISM02862	LOC_Os01g20940	11694084	CAGGAAAACGACGAGGAAAG	ATTTCTCGAATGTCTGCGCT	59.9	232	92	PHS1 putative expressed
OsISM02863	LOC_Os01g20940	11694382	GGATTGAAGTCATGCCGAGT	AGCTCTGCTGTAACCACTTGC	59.9	805	77	PHS1 putative expressed
OsISM02864	LOC_Os01g20940	11695341	TCGGACCACTCTGAAGATGA	AGCAAAGAACACTACACCACCA	59.5	308	73	PHS1 putative expressed
OsISM02865	LOC_Os01g20940	11695760	TTGCTTGGAGTTCAGACACC	GTGCATTTTCTGCAGCCTTT	59.6	180	86	PHS1 putative expressed
OsISM02866	LOC_Os01g20940	11696002	GCTCTTGAGCTAAGCCGTTG	CAAGAAGGGGAGATCCATGA	60.1	144	59	PHS1 putative expressed
OsISM02867	LOC_Os01g20940	11697127	TGACAATGAAGCTCCGTGAT	GACTTAAGCGCCTCATTCCA	59.8	341	89	PHS1 putative expressed
OsISM02868	LOC_Os01g20940	11697517	GATGGCAATGAGAATAACATGG	AGCGTCAGAGACCAAGGCTA	59.7	173	91	PHS1 putative expressed
OsISM02869	LOC_Os01g20940	11697828	GATTCCCAATTTCCCGATCT	GGCTTCTTCAAAAAGATCACTG	59.1	178	88	PHS1 putative expressed
OsISM02870	LOC_Os01g20940	11698088	TTGTGCTTGCCTACCTGATG	AAGCTTTCGCAAGAGTGAGG	59.8	154	49	PHS1 putative expressed
OsISM02871	LOC_Os01g20960	11709883	CCGAAGTGATCAATTATGGAGG	TTTGCTGCATGAGGCTAGTG	60.4	177	97	hypothetical protein
OsISM02872	LOC_Os01g20970	11712979	TCTACGACGACGACAGCATC	GTCCGTCAGCTGCTCCAC	60.3	225	99	invertase/pectin methylesterase inhibitor family protein putative expressed
OsISM02873	LOC_Os01g20990	11720261	CTCCGAGATCAAATCAACGG	GTCGTGTGTCCTTCGTGGA	60.7	378	71	retrotransposon protein putative unclassified
OsISM02874	LOC_Os01g20990	11721443	AAGTCGCTCGAGAAGCACAT	GGAGCCGTTATCGGTTATGA	60.0	617	76	retrotransposon protein putative unclassified
OsISM02875	LOC_Os01g21010	11726706	AAAAGGAGATTAAGGAGGCCA	ACCACCATGACCACCAATTT	59.6	512	84	conserved hypothetical protein
OsISM02876	LOC_Os01g21010	11727321	CGGACATGCTCGCTTTACTA	TGCCAAGGTACTTGACAGGA	59.2	312	63	conserved hypothetical protein

ISM IDs	Rice gene locus IDs	Physical positions (bp) on chromosomes	Forward primers (5'-3')	Reverse primers (5'-3')	Melting tempearature (T˚C)	Expected product size (bp) in PCR	Expected product size (bp) in Q-PCR	Putative Functions
OsISM02877	LOC_Os01g21010	11727667	TTGGAAAGTTATTAAACAAAGAATTGA	TTGTAGTTGAGCCCAGAGCC	59.6	1240	96	conserved hypothetical protein
OsISM02878	LOC_Os01g21020	11736960	ATTGGGAGGTAACTCGGAGC	GGCGCATATGTCCACCAC	60.7	151	57	hypothetical protein
OsISM02879	LOC_Os01g21034	11740916	ACCAGTACACCTGCCTCGAC	ACTTACCCAACCCAACGAGG	60.7	7355	63	pectinesterase putative expressed
OsISM02880	LOC_Os01g21060	11752900	TCCTGAATCGAACCAACTCA	TAACCTCAGGGGCCATACTG	59.6	1082	72	hypothetical protein
OsISM02882	LOC_Os01g21070	11756542	ACTACCTCCTCAAGGCCACC	GTCCCTGTTGGCATCACC	60.2	394	66	endoglucanase putative expressed
OsISM02883	LOC_Os01g21070	11757068	AAGTCCGACCCGGCCTAC	GAGACGGGTGCTGTACGTG	61.4	218	94	endoglucanase putative expressed
OsISM02884	LOC_Os01g21070	11757288	GCCATACGTCTGCCCATACT	GTTCTTGGTCGCCCTGTGTA	60.5	210	89	endoglucanase putative expressed
OsISM02885	LOC_Os01g21110	11778094	GTAGCAGCTGCCTGGGAGTA	TGACTAAGGCTGAACCCGTC	60.4	1858	87	hypothetical protein
OsISM02886	LOC_Os01g21110	11779956	TTGGTCACCGATGACTTTGA	TGGAGATGGGGACGAGAGTA	60.4	336	92	hypothetical protein
OsISM02888	LOC_Os01g21120	11783061	GTTGTGCGCCTCCGACTT	GGTTCTTCCGCTCCCTCTT	61.6	180	96	AP2 domain containing protein expressed
OsISM02889	LOC_Os01g21210	11831119	GTCAGACCGGGACTCAGG	TGCAGCAGTGGATACAGGAG	59.6	170	74	retrotransposon protein putative unclassified
OsISM02890	LOC_Os01g21210	11831306	CCCCACATGTTCCTAATGCT	CGGCTTTTTGTAAACCCTAGC	60.0	224	66	retrotransposon protein putative unclassified
OsISM02891	LOC_Os01g21210	11832206	ACCTAATGCCGATTCCTCG	CGGTGAACTTCAATCGTCCT	60.1	264	76	retrotransposon protein putative unclassified
OsISM02892	LOC_Os01g21210	11832593	GTGAACCAAGGCCAAAGATG	TTGCCTCCACCTTAATACGC	60.3	491	91	retrotransposon protein putative unclassified
OsISM02893	LOC_Os01g21210	11833121	TGATGTGCCGATACCTTCTG	GTCGGACCATCGTCGTGT	60.1	161	94	retrotransposon protein putative unclassified
OsISM02894	LOC_Os01g21210	11833558	AGAGAAAAAGGCCGAAGAGC	GAGCAACCTCTGCTTCATCC	60.0	224	87	retrotransposon protein putative unclassified
OsISM02895	LOC_Os01g21210	11834057	AACAAGACGTTACCAACCGC	TGCACCCTCTTTTGTAACCA	59.6	384	75	retrotransposon protein putative unclassified
OsISM02896	LOC_Os01g21210	11835417	CCGACATGCAAGAAGGAAGT	CCCCAAGTAAAATCGGCTTC	60.5	157	96	retrotransposon protein putative unclassified

ISM IDs	Rice gene locus IDs	Physical positions (bp) on chromosomes	Forward primers (5'-3')	Reverse primers (5'-3')	Melting tempearature (T˚C)	Expected product size (bp) in PCR	Expected product size (bp) in Q-PCR	Putative Functions
OsISM02897	LOC_Os01g21210	11835737	ATTACATATTTGAGCCGCCG	CTTGCCAATCCTCCCACTTA	60.0	195	67	retrotransposon protein putative unclassified
OsISM02898	LOC_Os01g21210	11837226	AAGCTGTGCCGTTGAAGAAT	CTTGACTCGGCTTGTCCATT	60.1	214	56	retrotransposon protein putative unclassified
OsISM02899	LOC_Os01g21210	11837583	GTCAAGAAGCCGTTTTACCG	CACCCTCTTGTTGTACGCCT	60.0	193	84	retrotransposon protein putative unclassified
OsISM02900	LOC_Os01g21210	11837828	TGGGTACTCGATCCAAGGAG	TATTTCCCGTTGATTGCTCG	60.5	142	66	retrotransposon protein putative unclassified
OsISM02902	LOC_Os01g21230	11845537	AAGATTTCAAATGATAAATTTAGGTTG	TCGTTGTTTTATTCAGTATTTTTGTTG	58.7	296	82	nodulin MtN3 family protein putative
OsISM02903	LOC_Os01g21230	11845782	GTGGCGATAGCTCATTTCCT	CCCACTGCCCATTTTATTGT	59.5	1427	77	nodulin MtN3 family protein putative
OsISM02904	LOC_Os01g21230	11847275	GAGAATCAGGGTGATCGGAA	CCACGTTCTCGTGTTGAATC	59.6	1593	99	nodulin MtN3 family protein putative
OsISM02905	LOC_Os01g21230	11848911	AGGCGTCTGGCTTGTCTATG	ACGATCAGTTGGATTGTCCC	60.1	1940	94	nodulin MtN3 family protein putative
OsISM02906	LOC_Os01g21240	11854578	GCGATCAACAAAAGGAGTGG	ACATGAGTCGGTGTAGCCGT	60.6	212	95	MLA6 protein putative expressed
OsISM02907	LOC_Os01g21240	11855093	TTTGCACCGTGAGTATGATCT	CCCATACCGATTCACTCCAA	59.5	787	99	MLA6 protein putative expressed
OsISM02908	LOC_Os01g21270	11878952	CATCTCACCACTGTCAAGCC	GCTAAACCATGACCCCTCAA	59.6	316	54	retrotransposon protein putative unclassified
OsISM02909	LOC_Os01g21270	11879623	CCAGCACACCAATTTCTTGA	TCTGGATGTGCCAAACCTCT	60.2	231	75	retrotransposon protein putative unclassified
OsISM02910	LOC_Os01g21270	11879827	GCTTCTCAGAGGTTTGGCAC	TGCGACTTTCAGTTCTGAGG	59.6	487	89	retrotransposon protein putative unclassified
OsISM02911	LOC_Os01g21290	11886282	ACTGGTGTCCCCGCCTATAC	TGGATCCTGCAGAACAAACA	60.7	905	60	retrotransposon protein putative unclassified
OsISM02912	LOC_Os01g21300	11896581	GGCCGAGATATCAATGGAAG	CTCTCGAGTTCCGCACGTA	59.8	161	54	esterase/lipase/thioesterase family protein putative expressed
OsISM02913	LOC_Os01g21300	11896789	AAGGCCCTCGTCTTCCTCT	CAAATGTGTGCCAGGTTCAT	59.9	262	78	esterase/lipase/thioesterase family protein putative expressed
OsISM02914	LOC_Os01g21300	11896985	TTGTTCATCAGGCTATGCCA	CTCGTAGTCCATGCCGAAC	59.7	3129	98	esterase/lipase/thioesterase family protein putative expressed
OsISM02915	LOC_Os01g21310	11904460	CATGAAGGACCCAACGTTCT	CAGGAGAGAGATCACCACCG	60.4	205	98	expressed protein

ISM IDs	Rice gene locus IDs	Physical positions (bp) on chromosomes	Forward primers (5'-3')	Reverse primers (5'-3')	Melting tempearature (T˚C)	Expected product size (bp) in PCR	Expected product size (bp) in Q-PCR	Putative Functions
OsISM02916	LOC_Os01g21310	11904730	CTTCAAGGACCCTGCCAAG	GAGCATCTCAAGTGCGGTCT	60.7	341	89	expressed protein
OsISM02917	LOC_Os01g21310	11905048	TCAAGACCGCACTTGAGATG	CACGAAGAACGGCAGCTT	60.1	239	78	expressed protein
OsISM02918	LOC_Os01g21420	11955903	TGATAGTGGGCAGTCGTCAA	AATTTTATGGTGTGCGGACG	60.5	292	90	pre-mRNA-splicing factor SF2 putative expressed
OsISM02919	LOC_Os01g21420	11956105	CCCCATAGTTCAAAGATGCAC	TTGAAGCAATCACGGTCAAG	59.6	150	93	pre-mRNA-splicing factor SF2 putative expressed
OsISM02920	LOC_Os01g21420	11956324	GCATTGCCTGGACTTACCAT	GACCAGCTACCAACCGACC	60.2	170	97	pre-mRNA-splicing factor SF2 putative expressed
OsISM02921	LOC_Os01g21420	11956482	TGGTAGCTGGTCGAAAATCC	GTACAAGCATTCTGGGTCCG	60.3	2371	67	pre-mRNA-splicing factor SF2 putative expressed
OsISM02922	LOC_Os01g21420	11958833	CGGACCCAGAATGCTTGTA	CCTACATAGATGGTGCGGCT	59.9	490	84	pre-mRNA-splicing factor SF2 putative expressed
OsISM02923	LOC_Os01g21420	11959339	ATTCGTGAGAGGGAGGTTGA	GCAAATTGCATCATCAGCAT	59.7	1186	70	pre-mRNA-splicing factor SF2 putative expressed
OsISM02924	LOC_Os01g21420	11960525	GGCCGTGATGGGTATAACTTT	TCTTGGGCGATCGTATGAGT	60.4	177	91	pre-mRNA-splicing factor SF2 putative expressed
OsISM02925	LOC_Os01g21420	11960715	GACGACGTGGAGGTGTTTCT	CATGATGCCGATGAAGGTAA	59.8	154	71	pre-mRNA-splicing factor SF2 putative expressed
OsISM02926	LOC_Os01g21420	11960842	CACTGGCTTACCTTCATCGG	AGAAGCAGACATCACCAGCC	60.5	143	72	pre-mRNA-splicing factor SF2 putative expressed
OsISM02927	LOC_Os01g21420	11960965	GGCTGGTGATGTCTGCTTCT	CCGCATACTTCATGTCTTCA	58.8	480	93	pre-mRNA-splicing factor SF2 putative expressed
OsISM02928	LOC_Os01g21420	11961394	CAACCGTTGGAATTGTGGAT	GAACTCTGAGTCATCAAGCTTCC	60.1	456	75	pre-mRNA-splicing factor SF2 putative expressed
OsISM02929	LOC_Os01g21420	11961854	ATGCATTTTCACGGGCATA	CGGCTTCTGCTTCTTGATCT	59.8	188	62	pre-mRNA-splicing factor SF2 putative expressed
OsISM02930	LOC_Os01g21420	11961968	TGATCTTTCGTCAACGTGTTCT	TTCATCCACAGGTGACGGT	59.9	181	76	pre-mRNA-splicing factor SF2 putative expressed
OsISM02931	LOC_Os01g21420	11962096	TGACTGCATTGTTCCACAGA	AACAGGAGTCCGTGATCTTGA	59.2	931	81	pre-mRNA-splicing factor SF2 putative expressed
OsISM02932	LOC_Os01g21420	11963007	CAAGATCACGGACTCCTGTTT	GAGAGGGAGAGCGGGATAAG	59.8	179	85	pre-mRNA-splicing factor SF2 putative expressed
OsISM02933	LOC_Os01g21420	11963151	AAGCCCAAGCAGGAGCTTAT	CGTCACAGTCTGGAGGGTTT	60.3	208	76	pre-mRNA-splicing factor SF2 putative expressed

ISM IDs	Rice gene locus IDs	Physical positions (bp) on chromosomes	Forward primers (5'-3')	Reverse primers (5'-3')	Melting tempearature (T˚C)	Expected product size (bp) in PCR	Expected product size (bp) in Q-PCR	Putative Functions
OsISM02934	LOC_Os01g21430	11966798	CTTGTTCGGACACGGAGATT	GGCAACTTCCTCTTGGTTCA	60.2	158	93	retrotransposon protein putative Ty3-gypsy subclass
OsISM02935	LOC_Os01g21430	11967105	TGTCTTCGTTACACCTCAACAAA	CGCAGTGTCCGTCGTAGTAG	59.6	382	96	retrotransposon protein putative Ty3-gypsy subclass
OsISM02936	LOC_Os01g21440	11969879	GGGGTTAATTTCTTGCGGTT	CAAGTGTATGTCATGTGTATTATTTGA	58.7	1065	82	expressed protein
OsISM02937	LOC_Os01g21450	11973613	GAGGTGCATCTACCAGCCAT	GAGCCATCAAGGTACGCAAT	60.1	2679	83	expressed protein
OsISM02938	LOC_Os01g21450	11976479	TTGTGTATGTAACACAATCAAAATTCA	CAGTGGGACTCAACTCTGGT	58.7	202	90	expressed protein
OsISM02939	LOC_Os01g21450	11976661	ACCAGAGTTGAGTCCCACTG	TGGGAAGAATAAGATGCCACA	59.1	1415	94	expressed protein
OsISM02940	LOC_Os01g21450	11978466	GTTGGCATTGCAAAATGAAA	CCATATGCAGAACGTAGAACCA	59.8	911	99	expressed protein
OsISM02941	LOC_Os01g21530	12034086	CAACATCGTCACCACTGCC	CTGGAGCTTCATCCCCGA	61.8	1668	98	retrotransposon protein putative unclassified
OsISM02943	LOC_Os01g21530	12036369	AAGCCTTCATCCGAGAGGAG	ACGAGTCATACGCTGAAAGG	59.4	380	75	retrotransposon protein putative unclassified
OsISM02944	LOC_Os01g21530	12037047	CCGCCCTAAGTCGATTCATA	GGCCTACTCTCGTTGCGTT	60.2	245	50	retrotransposon protein putative unclassified
OsISM02945	LOC_Os01g21530	12037619	ATTGCGCCATTATTTCCAAG	AGGTCGAACTCAGCAAGCTC	59.8	129	94	retrotransposon protein putative unclassified
OsISM02946	LOC_Os01g21530	12037976	CCTTAAGTACGTCGTTCGGC	GTCGGTGCACTGGTATTCCT	59.9	172	92	retrotransposon protein putative unclassified
OsISM02947	LOC_Os01g21600	12098075	CCGGGAGATAGTTACGAGGG	GATGGCCAATGTCATTACCG	61.0	180	87	retrotransposon protein putative unclassified
OsISM02948	LOC_Os01g21600	12098597	ACGAGGAGGACGATAACGG	GTCAGAGGGCGATAGGTGTG	60.4	164	96	retrotransposon protein putative unclassified
OsISM02949	LOC_Os01g21610	12108359	AAAGGAGTTGTTTGTCTTCCGA	CGAAAAGGAAGTTGCTCCAA	60.3	449	60	uncharacterized aarF domain-containing protein kinase 4 putative expressed
OsISM02950	LOC_Os01g21610	12108849	GCTCGTGATTTTCCAAAACG	CCAGCTCTATCCTTGTTCGC	60.3	192	75	uncharacterized aarF domain-containing protein kinase 4 putative expressed
OsISM02951	LOC_Os01g21620	12111991	CTGCCATTTCTCTTGCTTCC	TAGGCGAGCTTGTACCAGTG	59.5	215	57	conserved hypothetical protein
OsISM02954	LOC_Os01g21630	12119948	TCCTCCGACCACATGGAG	GATCTTGACGCGGTTCCA	60.7	189	97	pectinacetylesterase domain containing protein expressed

ISM IDs	Rice gene locus IDs	Physical positions (bp) on chromosomes	Forward primers (5'-3')	Reverse primers (5'-3')	Melting tempearature (T˚C)	Expected product size (bp) in PCR	Expected product size (bp) in Q-PCR	Putative Functions
OsISM02956	LOC_Os01g21630	12120429	CCACCGTCAAGTGCCTCT	GCCGTAGTATGACCTCAGGC	59.8	177	84	pectinacetylesterase domain containing protein expressed
OsISM02957	LOC_Os01g21630	12120646	AGACCTGCACCGACCATCT	GGTGTCTTTATGCCGTCGAT	60.3	216	71	pectinacetylesterase domain containing protein expressed
OsISM02958	LOC_Os01g21630	12120842	ATCGACGGCATAAAGACACC	CTGGTGTCAGCTGCGTTAGG	61.0	254	93	pectinacetylesterase domain containing protein expressed
OsISM02959	LOC_Os01g21630	12121105	GCGAGTCTGCAAGTTCAACC	GAGAAGACTCTCACCGCTGC	60.6	235	97	pectinacetylesterase domain containing protein expressed
OsISM02960	LOC_Os01g21630	12121414	ATGGTGGCTCTCCTGCTCT	CACCTCAGCTCGACCAAAGT	60.2	237	75	pectinacetylesterase domain containing protein expressed
OsISM02961	LOC_Os01g21640	12125400	GGTCACCGTACATGCTGAAG	GTCCGACCTGCAATAGGGTA	59.6	187	51	expressed protein
OsISM02962	LOC_Os01g21640	12125615	CGTACATTTCAGCAGCAGCC	TGTTCTCTCCGAGTGGCAG	61.0	176	99	expressed protein
OsISM02964	LOC_Os01g21660	12153759	GATGCTTTGGGGATGTTGAT	TGAACTTGTCCTCACTTGCC	59.3	1224	98	hypothetical protein
OsISM02965	LOC_Os01g21660	12154999	GATCGAGGAGTTCGAGTGGA	GAATTCACCCTCCGTGGTTA	60.1	537	59	hypothetical protein
OsISM02966	LOC_Os01g21690	12165765	CTTCATCGGAGCCCATTTAT	TCCAGCGGTTATGTCCAGAT	59.7	496	90	retrotransposon putative centromere-specific
OsISM02967	LOC_Os01g21690	12166562	AGAGGAAACAACCCGTTCCT	GCGAATGCAAAAGACTCAGA	59.6	247	98	retrotransposon putative centromere-specific
OsISM02968	LOC_Os01g21690	12167635	GTCGTTGCAAATGCTCAGAA	CTTTGGAAAGATGCAGGGG	60.3	306	99	retrotransposon putative centromere-specific
OsISM02969	LOC_Os01g21690	12168567	TCGCGGTTGTTTATGCTATC	AATCATTGATCGGCTGTTCC	59.3	275	85	retrotransposon putative centromere-specific
OsISM02970	LOC_Os01g21700	12175245	GTGCTCAGGCTTGGAGTGA	CCACGAAATCCAATTCTTTCA	60.0	305	98	hypothetical protein
OsISM02972	LOC_Os01g21710	12185213	TGTCTTCTTTCATTTCCACGAA	TTGACACCCATTTCATGGTC	59.5	215	48	plant protein of unknown function domain containing protein
OsISM02973	LOC_Os01g21710	12185400	ACTAGCCGGACCATGAAATG	CTCCCGAGAAGACTCCCAAT	60.3	289	92	plant protein of unknown function domain containing protein
OsISM02974	LOC_Os01g21760	12208571	CATTCTGCTTCCGAGGTAGG	CGTAGATCGAAAGTGGAGCC	59.8	812	77	retrotransposon protein putative unclassified
OsISM02975	LOC_Os01g21760	12209376	ATCTACGATGCTACCGCAGG	CAAATCCCCAATTCCCTTTT	60.1	177	99	retrotransposon protein putative unclassified

ISM IDs	Rice gene locus IDs	Physical positions (bp) on chromosomes	Forward primers (5'-3')	Reverse primers (5'-3')	Melting tempearature (T˚C)	Expected product size (bp) in PCR	Expected product size (bp) in Q-PCR	Putative Functions
OsISM02976	LOC_Os01g21780	12219436	GTGCTCAGGCTTGGAGTGA	TTCTTTCGTTCTCTCACGCA	59.9	269	85	hypothetical protein
OsISM02977	LOC_Os01g21780	12219765	TTTTTAGGCGAGGAGACAGG	TACTCGTTGCCTCCCTCATT	59.6	980	60	hypothetical protein
OsISM02978	LOC_Os01g21780	12220728	GAGGGAGGCAACGAGTACAG	TGGTCGTCCTTCTTACCGAC	60.0	166	99	hypothetical protein
OsISM02979	LOC_Os01g21780	12220878	GTAAGAAGGACGACCAACGG	TCGGATCAATCCTCAATTTCA	60.0	1297	97	hypothetical protein
OsISM02980	LOC_Os01g21780	12222167	TGATCCGATGAATGATCCAG	TTGTTGGTCTTTTTGTTCTTGTTT	59.3	213	98	hypothetical protein
OsISM02981	LOC_Os01g21780	12222713	ACAACACGTCGAGTTTCAGC	ATGGGGTGCATATCTTTCGT	59.1	221	84	hypothetical protein
OsISM02983	LOC_Os01g21800	12230205	GCGAGGCTTGGAGTGAACTT	TCATTCTCTCACGCAGCATC	61.0	204	77	hypothetical protein
OsISM02984	LOC_Os01g21810	12231675	TTAACCAGCTCTCGCTGACA	TTTTTCATAGTCCTCTTGGATCTT T	59.3	654	78	hypothetical protein
OsISM02985	LOC_Os01g21810	12232304	AAAGATCCAAGAGGACTATGA AAA	AAGTATTTTTGTGGGCACCG	58.7	419	49	hypothetical protein
OsISM02986	LOC_Os01g21810	12232731	TGAAATTGATGATTGATCCGA	ATTCTTTTTGAAGACGTTTCTTAT G	58.1	212	94	hypothetical protein
OsISM02987	LOC_Os01g21810	12233003	CCAATACCAGTTCTATCCAAC AAA	CAACGTGGGTTCCTTCAACT	59.7	137	93	hypothetical protein
OsISM02988	LOC_Os01g21810	12233296	CAGAGCCAACTAATGGCACA	CGTCATCGTTACTTCCCAGG	60.2	210	79	hypothetical protein
OsISM02989	LOC_Os01g21810	12233552	CAACACCTGTCGATTCCTCT	AGGATGCCAACCATATTTGC	58.8	171	98	hypothetical protein
OsISM02990	LOC_Os01g21840	12252380	GTCCATCTGGACAGGCAAGT	GTGGTTCCTGTCAATCCCAC	60.2	462	75	transposon protein putative unclassified expressed
OsISM02992	LOC_Os01g21840	12253353	CTAGCAAGGCTTGGTTGCTC	AACCACAACAGGTACGCCTC	60.1	184	98	transposon protein putative unclassified expressed
OsISM02993	LOC_Os01g21840	12253847	ACGATGGGGACTTACTGGTG	GGAGACGGTCAAACTCGAAC	59.8	187	95	transposon protein putative unclassified expressed
OsISM02994	LOC_Os01g21840	12254206	CGGACTTAGGCAGGTGTTTC	CTAGCTGTCCCCGTCGAGT	60.1	159	81	transposon protein putative unclassified expressed
OsISM02995	LOC_Os01g21840	12254573	GCACCGTGACCGAGACTACT	TCACTTGGACTGCGGTGATA	60.3	169	75	transposon protein putative unclassified expressed

ISM IDs	Rice gene locus IDs	Physical positions (bp) on chromosomes	Forward primers (5'-3')	Reverse primers (5'-3')	Melting tempearature (T˚C)	Expected product size (bp) in PCR	Expected product size (bp) in Q-PCR	Putative Functions
OsISM02997	LOC_Os01g21840	12255281	AGCTCTTCCTCTCACGGAGC	AGACGAAGAGGCCCCAGTA	60.5	144	82	transposon protein putative unclassified expressed
OsISM02998	LOC_Os01g21840	12255600	ACCGACTCACGTACTCCCAC	GCAAGGGCGATTTTGTACTG	60.3	873	76	transposon protein putative unclassified expressed
OsISM02999	LOC_Os01g21860	12264010	CGTCAACTTCATCTCCGAGG	CCTGGAAGAGCGGCTTGT	61.1	205	99	AGAP010768-PA putative expressed
OsISM03000	LOC_Os01g21860	12264151	AAGCGGACGAGAGGGGAG	CTCGGCATCCTTCTTCTCCT	61.9	203	97	AGAP010768-PA putative expressed
OsISM03001	LOC_Os01g21860	12264339	AAGAAGGATGCCGAGTTCAA	CATCTCATCTTCATCCAAGGC	59.7	1827	67	AGAP010768-PA putative expressed
OsISM03002	LOC_Os01g21860	12266139	CCAAAAGCCTTGGATGAAGA	AGCCACCTGCTGCTCATATT	60.0	1015	79	AGAP010768-PA putative expressed
OsISM03003	LOC_Os01g21860	12267134	AATATGAGCAGCAGGTGGCT	ATAATATTAGACCGGGCCGC	60.0	238	80	AGAP010768-PA putative expressed
OsISM03004	LOC_Os01g21860	12267328	TGCTTGTTCTTTCAGGAGGC	CTTTTTGCCTTAGGCTTGCT	59.7	260	99	AGAP010768-PA putative expressed
OsISM03005	LOC_Os01g21950	12315823	GAGCATGGACACAACAGCC	CCAGGTCTTTCCAATCTCCA	60.2	350	59	hypothetical protein
OsISM03006	LOC_Os01g21960	12327411	TGGCACTCCTGTTGCTGTTA	GCGAACATGACCAATAGCCT	60.3	214	92	serine/threonine-protein kinase putative expressed
OsISM03007	LOC_Os01g21960	12327640	CGGCTTCTGGGTTACTGTGT	GCCATTGCTCTAGGTTTCCA	60.2	257	80	serine/threonine-protein kinase putative expressed
OsISM03008	LOC_Os01g21960	12327910	GAGTCACCGTGGTTCCCTTA	CACTTTGGGTTCAATTGCCT	60.0	428	98	serine/threonine-protein kinase putative expressed
OsISM03009	LOC_Os01g21960	12328416	AAACTTCTTGGTGCTGGCAA	TGTTTGCATACTCTGGTGCC	60.3	177	80	serine/threonine-protein kinase putative expressed
OsISM03010	LOC_Os01g21960	12328657	AGGGAGGGATCCTGTTGACT	GCCTGCTAGCAACCATCATT	60.1	1186	81	serine/threonine-protein kinase putative expressed
OsISM03011	LOC_Os01g21960	12329984	AATCGGATGACCCAATTCCT	GTCCCTTTGGGAATCCATTT	60.3	290	81	serine/threonine-protein kinase putative expressed
OsISM03012	LOC_Os01g21990	12344147	CGTCAAGCCCCACATCTC	CATGTTGTGGGTCAATCCAT	59.6	1448	65	CRS2-associated factor 2 chloroplast precursor putative expressed
OsISM03013	LOC_Os01g21990	12345639	AGTGAGATGCCGAGGAGTTC	GGTGAATAACCTTTCCGCCT	59.9	733	84	CRS2-associated factor 2 chloroplast precursor putative expressed
OsISM03014	LOC_Os01g21990	12346541	GAAGGATCTGCTGCCAATTT	CTTCCCTCAAAAGCATCTCG	59.6	2250	85	CRS2-associated factor 2 chloroplast precursor putative expressed

ISM IDs	Rice gene locus IDs	Physical positions (bp) on chromosomes	Forward primers (5'-3')	Reverse primers (5'-3')	Melting tempearature (T˚C)	Expected product size (bp) in PCR	Expected product size (bp) in Q-PCR	Putative Functions
OsISM03015	LOC_Os01g21990	12348795	CTGGTGAAGATTGACTGCGA	CGACAAAAGAACACAAGGAACA	60.1	207	91	CRS2-associated factor 2 chloroplast precursor putative expressed
OsISM03016	LOC_Os01g21990	12349057	CAAAGCCTCTGACTCTTATTCCA	ACGGTCATCATCATCAGTGG	59.6	472	96	CRS2-associated factor 2 chloroplast precursor putative expressed
OsISM03017	LOC_Os01g22039	12375350	GTGCGCGTGAAGATCCAT	CGCCGACATAAAGTCCAGAT	60.5	816	86	hypothetical protein
OsISM03018	LOC_Os01g22039	12376329	CAATGTTCTTGGCCCTCCTA	TAGCTTCGATGCAGAGAGCA	60.0	553	75	hypothetical protein
OsISM03019	LOC_Os01g22070	12393013	GAGTACTGTGCAAGCAGCGA	CACCCTAAACTCTGCATCAGG	60.0	206	80	transposon protein putative Mutator sub-class
OsISM03020	LOC_Os01g22070	12394070	GTTCAGAGGCTTTTGCAAGG	CCTTCTCTGCCACCCACTTA	60.1	263	93	transposon protein putative Mutator sub-class
OsISM03021	LOC_Os01g22070	12394814	GGATCAATCACCCCTAGCAA	GGCATGAAAGACCTAGCTGC	59.9	180	73	transposon protein putative Mutator sub-class
OsISM03022	LOC_Os01g22070	12395760	AGGCCATCATCCTCAAAATG	TACTGGCCTTCCTTGTTTCG	60.1	142	76	transposon protein putative Mutator sub-class
OsISM03023	LOC_Os01g22070	12395933	CACCAAAACGACAAAAAGTCG	TCCTTGTTCACCAGCATTGA	60.4	174	99	transposon protein putative Mutator sub-class
OsISM03025	LOC_Os01g22080	12397707	GTTAGCACTTGGGTGTGGCT	CAACGAGTGCAAGGAGGTG	60.3	750	81	hypothetical protein
OsISM03026	LOC_Os01g22080	12398725	TCTTGACAACGGTCAGCTTG	ACCATAGGGGAAATCTGGGA	60.3	347	93	hypothetical protein
OsISM03027	LOC_Os01g22080	12399053	CCCAGATTTCCCCTATGGTC	AAAACCGGAGCCAATGAGTT	60.7	1425	66	hypothetical protein
OsISM03028	LOC_Os01g22100	12407792	GGACCAAGGTGGGAGGAC	AGTAGCATAGCGAAAGGATCG	59.5	529	76	retrotransposon protein putative unclassified
OsISM03029	LOC_Os01g22100	12408362	AAACAGAGATGAAGTCGCATTG	CGGTGATGTGTCCCTCTAGC	60.0	463	70	retrotransposon protein putative unclassified
OsISM03030	LOC_Os01g22110	12411399	CCAAGTTGCAGAGTGGGATT	TCCATTGCCTCTTTCCAGTT	59.9	136	77	retrotransposon protein putative unclassified
OsISM03031	LOC_Os01g22110	12412446	ATCATTGGGCTGCTACCAAG	GCGCACTCCATGACATAAGA	60.0	195	94	retrotransposon protein putative unclassified
OsISM03033	LOC_Os01g22120	12417093	GCTGCAGGACCCTATTAACG	CTTGGAAATTGAGTAGGGCG	59.7	869	78	retrotransposon protein putative unclassified
OsISM03034	LOC_Os01g22170	12456102	TATGGCTAGGATGGGTTGGA	GTCATGTCCAGACCACGATG	60.1	1027	91	retrotransposon protein putative Ty3-gypsy subclass

ISM IDs	Rice gene locus IDs	Physical positions (bp) on chromosomes	Forward primers (5'-3')	Reverse primers (5'-3')	Melting tempearature (T°C)	Expected product size (bp) in PCR	Expected product size (bp) in Q-PCR	Putative Functions
OsISM03035	LOC_Os01g22170	12457840	CGTTGGTAGCACGGAGGAT	GCCATCACCGGTATTAGCAC	60.7	349	80	retrotransposon protein putative Ty3-gypsy subclass
OsISM03036	LOC_Os01g22180	12465022	TATGGCTAGGATGGGTTGGA	GAGAGACACCACTCCCGATG	60.5	869	83	retrotransposon protein putative Ty3-gypsy subclass
OsISM03037	LOC_Os01g22190	12473415	TGCGACTTACACTGGTGGAG	TAAACTCTTTGGACCGCCAG	60.1	956	80	transposon protein putative CACTA En/Spm sub-class
OsISM03038	LOC_Os01g22190	12474351	CTGGCGGTCCAAAGAGTTTA	ATCCCGACTCAAACAAGTGG	60.1	161	88	transposon protein putative CACTA En/Spm sub-class
OsISM03039	LOC_Os01g22190	12475095	AGAAGTTGACGGGCAGCTAA	GCTTGGGGCCTTGAATAATA	59.5	326	84	transposon protein putative CACTA En/Spm sub-class
OsISM03040	LOC_Os01g22190	12475734	GCACTTTGAACATAAGGCGG	CGCGTTGTTCCATATGCTT	60.2	332	90	transposon protein putative CACTA En/Spm sub-class
OsISM03041	LOC_Os01g22190	12476556	CATGGGCGTTCTGAAGAAGT	TGCTTTCCTTCCGAGAGTTC	59.9	186	98	transposon protein putative CACTA En/Spm sub-class
OsISM03042	LOC_Os01g22190	12477517	GTAGCGATCACAAGGAGGGA	GCTAGCTACCACTCCGGCTT	60.6	2021	81	transposon protein putative CACTA En/Spm sub-class
OsISM03043	LOC_Os01g22190	12479486	TTGCAGAAAAGACGTTGTCG	CCTCCGCGATTGTATCGTAT	60.0	1044	90	transposon protein putative CACTA En/Spm sub-class
OsISM03045	LOC_Os01g22200	12482993	TCAGGGGTATGTCTGCACCT	TCCTGGTTTGGTTGCTCTCT	60.2	124	77	transposon protein putative CACTA En/Spm sub-class
OsISM03046	LOC_Os01g22200	12483441	CGCCCTCGACGTCTCTATTA	ATCCAGTATCGAAAACCCCC	60.2	187	81	transposon protein putative CACTA En/Spm sub-class
OsISM03047	LOC_Os01g22200	12483708	AAGGCGCAGCATTACAAGAC	GCCTCCAGGTCGAAGAGTAA	59.9	177	81	transposon protein putative CACTA En/Spm sub-class
OsISM03048	LOC_Os01g22200	12483882	GGCCTACACCGTCAACGTAT	AATGACGGAACCGATACCAA	60.0	184	99	transposon protein putative CACTA En/Spm sub-class
OsISM03049	LOC_Os01g22200	12484045	CTTGGTATCGGTTCCGTCAT	AGTTCCCTGCTTTTGCTTTG	59.7	192	93	transposon protein putative CACTA En/Spm sub-class
OsISM03050	LOC_Os01g22200	12484271	ACTGCCTTGCAGACCAAATC	CCGCGATAAATTCCTTGTGT	60.1	169	88	transposon protein putative CACTA En/Spm sub-class
OsISM03051	LOC_Os01g22230	12499180	CTCCACTTCCACGACTGCTT	GGGTTCTTGGTCAGGAGGAC	60.7	2856	60	peroxidase precursor putative expressed
OsISM03052	LOC_Os01g22230	12502412	GTCTGGAACGGCAACACC	GTCCCTTGTGGGGCACAG	61.3	180	85	peroxidase precursor putative expressed
OsISM03053	LOC_Os01g22249	12508776	TCTGCACTTCCATGACTGCT	CCCGTTGATCAAAACTGAGG	60.0	8227	56	peroxidase precursor putative expressed

ISM IDs	Rice gene locus IDs	Physical positions (bp) on chromosomes	Forward primers (5'-3')	Reverse primers (5'-3')	Melting tempearature (T°C)	Expected product size (bp) in PCR	Expected product size (bp) in Q-PCR	Putative Functions
OsISM03055	LOC_Os01g22270	12520036	CGATTTGTGTTTACCGGGTT	CACGACGAAGCTCTTTGATG	59.7	678	86	retrotransposon protein putative unclassified
OsISM03056	LOC_Os01g22270	12521454	AACGTCTCCGATGTTTGCTT	TGTTGAGCAATCCAAAACATT	58.9	462	70	retrotransposon protein putative unclassified
OsISM03057	LOC_Os01g22270	12522122	CCTCAACATGATTTTGGATCA	TCTCACCACTTCTAGCACCAA	58.5	125	50	retrotransposon protein putative unclassified
OsISM03058	LOC_Os01g22270	12522212	TGTTGTGTTAGGTGTTGGTGC	TCCACGATCCACACATTCTC	59.5	665	65	retrotransposon protein putative unclassified
OsISM03059	LOC_Os01g22270	12524305	TGAGGGTTCTGTTGATGCTG	TGCACAAACATCATGAGTCG	59.5	196	74	retrotransposon protein putative unclassified
OsISM03060	LOC_Os01g22270	12524806	TTTGTTGGCTTTTGCTGCTT	CCATGCCCTAGGAGATTGTT	60.0	193	59	retrotransposon protein putative unclassified
OsISM03061	LOC_Os01g22270	12525044	GGAAAGGTTGACAAAACGCT	CAAATTCCCTGCGCATAGTC	59.9	142	65	retrotransposon protein putative unclassified
OsISM03062	LOC_Os01g22280	12528296	GCGTGACAGGAGGAGGAAG	GCCCCCTTCTTGTTATAGGC	60.4	980	51	retrotransposon protein putative unclassified
OsISM03063	LOC_Os01g22320	12542576	CCCATAGAAAGGGTGGTTGA	CCTTGCTTCTCCAAAAATCC	59.3	480	99	transposon protein putative unclassified
OsISM03064	LOC_Os01g22320	12542999	CATTGTCAAAAGGGGGAGAA	TACTCTCCCGAGACATCGAG	58.9	674	99	transposon protein putative unclassified
OsISM03065	LOC_Os01g22336	12550563	CTCCATTTCCACGACTGCTT	GACGTCAGCAGCACCGAC	61.0	4491	54	peroxidase precursor putative expressed
OsISM03067	LOC_Os01g22352	12563528	CTCCATTTCCACGACTGCTT	GTTCGGCGAGAAGATCAGC	60.7	3902	61	peroxidase precursor putative expressed
OsISM03068	LOC_Os01g22352	12567778	TCCTTCCTCTTCAAGAACCG	CTGGTACGCCGGGCTAAT	60.2	481	58	peroxidase precursor putative expressed
OsISM03069	LOC_Os01g22370	12577391	TGCACTTCCATGACTGCTTT	GTTCCCGTCGATCAAAACC	59.9	2778	57	peroxidase precursor putative expressed
OsISM03070	LOC_Os01g22410	12599974	AAGGTGGTCATTCAACCGAC	GATCTTCGGCCTCGGACT	60.1	182	87	transposon protein putative unclassified
OsISM03071	LOC_Os01g22450	12627076	GTGGAGGCACGGATGAAG	CAGGTCTTGTTGGCTCTGAA	59.6	867	97	hypothetical protein
OsISM03072	LOC_Os01g22450	12627926	AGAGCCAACAAGACCTGTCC	ACCAAAATGTAAGGCGCGTA	59.9	972	77	hypothetical protein
OsISM03073	LOC_Os01g22500	12645671	TGGTACGGGATAGGGTAGGC	TGGTTCATCCAAAATCACATCT	60.2	1104	97	craniofacial development protein 1 putative expressed

ISM IDs	Rice gene locus IDs	Physical positions (bp) on chromosomes	Forward primers (5'-3')	Reverse primers (5'-3')	Melting tempearature (T˚C)	Expected product size (bp) in PCR	Expected product size (bp) in Q-PCR	Putative Functions
OsISM03074	LOC_Os01g22500	12646783	TTCAGGAAGCGCTGAAAGTT	CTGGCATTCCACTGTTCATTT	60.1	723	96	craniofacial development protein 1 putative expressed
OsISM03075	LOC_Os01g22500	12647528	GAATAAGCTCAGCACACCTGC	TTGGTGAAAGTCCCAGAACA	59.6	191	78	craniofacial development protein 1 putative expressed
OsISM03076	LOC_Os01g22500	12647797	AACTTGCAGCGAGTGCTCTT	CCCAGCAAAGTCCCTAACCT	60.4	564	96	craniofacial development protein 1 putative expressed
OsISM03077	LOC_Os01g22510	12649794	ATGTCGAGACCTGCGCTC	AGCCACTGAACAGGGAACAT	59.8	954	88	cyclase/dehydrase family protein expressed
OsISM03078	LOC_Os01g22510	12650771	GACCATTCCTCAATGGATGC	AGTCCAACGTGATTGATCTGG	60.1	1350	74	cyclase/dehydrase family protein expressed
OsISM03079	LOC_Os01g22510	12652157	TCGTGGCTAGCTCGAAACAT	AGACCTTCAAGAGATCGCCA	60.4	145	66	cyclase/dehydrase family protein expressed
OsISM03080	LOC_Os01g22510	12652282	TGGCGATCTCTTGAAGGTCT	TCTGCAAGAGGATGAGCTTTT	59.6	382	70	cyclase/dehydrase family protein expressed
OsISM03081	LOC_Os01g22510	12652614	CTTGTAGGGGTGCTGTTCGT	CAACAGGTGCCAAAATTTCA	59.9	194	97	cyclase/dehydrase family protein expressed
OsISM03082	LOC_Os01g22510	12652786	CCTGAAATTTTGGCACCTGT	CCTTCCAGAAATGGCTTCAG	59.9	151	48	cyclase/dehydrase family protein expressed
OsISM03085	LOC_Os01g22590	12702214	TGGAATCTCCCGTGTAAACC	CTTACTGCAGGGCTCTCACA	59.5	223	75	retrotransposon protein putative unclassified expressed
OsISM03086	LOC_Os01g22590	12702392	CAATGGATCGACCCTACCC	GCTCGCCACGAACTTGAT	60.0	162	59	retrotransposon protein putative unclassified expressed
OsISM03087	LOC_Os01g22630	12726109	GTATGTTCCTGCAACTGCGA	ACCATTCCGTAGGTGATCCA	60.0	167	66	ECT protein putative expressed
OsISM03088	LOC_Os01g22630	12726256	TGGATCACCTACGGAATGGT	ACTGTGTGCCTTCGGTTTG	60.0	176	93	ECT protein putative expressed
OsISM03089	LOC_Os01g22630	12726842	CCACTGAAGAAAGAGCTGGC	CGCCTCTCAGGAAAATTTGA	60.2	850	87	ECT protein putative expressed
OsISM03090	LOC_Os01g22630	12728162	CCAAGTGCCCTGTTTTTCTG	ACAGGACCAACCATCTCAGC	60.4	164	77	ECT protein putative expressed
OsISM03091	LOC_Os01g22630	12728456	AAGCCTGTCACACACAGCAG	AGCAAACACTCCTTCACTTTGA	59.6	321	99	ECT protein putative expressed
OsISM03092	LOC_Os01g22640	12733739	CGCCTCGTCATCGACTTC	ACTTCAGGTACGGGCTCAGA	60.2	937	62	GDSL-like lipase/acylhydrolase putative expressed
OsISM03093	LOC_Os01g22640	12734787	TCAGAACCAGATCCATCGAA	TGTAGATGGCGTTTCGGAAT	59.8	838	79	GDSL-like lipase/acylhydrolase putative expressed

ISM IDs	Rice gene locus IDs	Physical positions (bp) on chromosomes	Forward primers (5'-3')	Reverse primers (5'-3')	Melting tempearature (T˚C)	Expected product size (bp) in PCR	Expected product size (bp) in Q-PCR	Putative Functions
OsISM03094	LOC_Os01g22640	12735697	CCACCATTGTTGCTCACATC	CGTGCACCCAGAACTTCC	60.1	534	73	GDSL-like lipase/acylhydrolase putative expressed
OsISM03095	LOC_Os01g22660	12741412	CGCCTCACCATCGACTTC	ATGCCTTCAGGTACGGACTC	59.8	1745	65	GDSL-like lipase/acylhydrolase putative expressed
OsISM03096	LOC_Os01g22660	12743232	CATTGCACATACAGGTGAAAC A	AGCATCGACTGGACCAGGTA	60.1	237	87	GDSL-like lipase/acylhydrolase putative expressed
OsISM03097	LOC_Os01g22660	12743558	TGCAAGGTTCCCTCCAATAC	TCCATGAACCCAGAAGTTCC	59.9	180	86	GDSL-like lipase/acylhydrolase putative expressed
OsISM03098	LOC_Os01g22660	12743932	CAAGTACGACCTCATTGCGA	TATAGTTGTAAGGCGGCCCA	60.2	177	90	GDSL-like lipase/acylhydrolase putative expressed
OsISM03099	LOC_Os01g22680	12751430	GTCAAGCTCCAGGGCTCC	GCCGGTCTTGATCCTGTG	60.6	176	82	fruit bromelain precursor putative expressed
OsISM03100	LOC_Os01g22720	12764454	ACCACATCGCCCTGCTACT	GACTCCCCAGCTACCGCT	60.5	1011	52	hypothetical protein
OsISM03101	LOC_Os01g22720	12765652	CTCCAGCAACAAAGAGGGAG	GGAACATATGGAGCCCACTG	60.2	2263	59	hypothetical protein
OsISM03102	LOC_Os01g22720	12768143	ATGGCAAGCTTGATGTCTCC	AGAGGTTGGCCATGACATTC	60.1	381	82	hypothetical protein
OsISM03103	LOC_Os01g22740	12781580	TCAGAGGAGAGAGCCCCTAA	AGGTGTTCCTCCCAACTTCC	59.7	162	97	transposon protein putative unclassified
OsISM03104	LOC_Os01g22740	12781900	AAGGTGGTCATTCAACCGAC	GATCTTCGGCCTCGGACT	60.1	182	87	transposon protein putative unclassified
OsISM03106	LOC_Os01g22780	12807782	CTCTCCGAGATCAAGGATGC	CAGGTTCTTGGCTCCATTGT	60.0	546	55	GDSL-like lipase/acylhydrolase putative expressed
OsISM03107	LOC_Os01g22780	12808536	AGTACGACCTCATCGCCAAC	GCTCCTCGAACCCTACAAAA	59.7	171	65	GDSL-like lipase/acylhydrolase putative expressed
OsISM03108	LOC_Os01g22794	12813634	GATAACGACGCTGCAGATGA	TCGCCAAATGTCTTGTCACT	59.6	215	90	transposon protein putative CACTA En/Spm sub-class
OsISM03109	LOC_Os01g22794	12814208	AAGACGGGATGCTGAGACAC	GTCGCGTTGTTCCATATGCT	60.7	868	96	transposon protein putative CACTA En/Spm sub-class
OsISM03110	LOC_Os01g22794	12815107	GCCATTACTTGAGTCCAGCC	TTATTGTTGCCCGAACATTG	59.6	237	94	transposon protein putative CACTA En/Spm sub-class
OsISM03111	LOC_Os01g22794	12815702	ACGCCATGATGGGAGACTAC	ATCAATCGTTACGCCTCCAC	60.0	532	98	transposon protein putative CACTA En/Spm sub-class
OsISM03112	LOC_Os01g22820	12820536	GTCATCTGCCAGTCCATCTG	ATGCGATAGGCTTGGAATTG	59.6	182	83	retrotransposon protein putative Ty3-gypsy subclass

ISM IDs	Rice gene locus IDs	Physical positions (bp) on chromosomes	Forward primers (5'-3')	Reverse primers (5'-3')	Melting tempearature (T˚C)	Expected product size (bp) in PCR	Expected product size (bp) in Q-PCR	Putative Functions
OsISM03113	LOC_Os01g22830	12826518	GAGGAAGACCGGAGCGAC	ATTCCAAGGCCTCGGCTAC	61.2	1038	98	retrotransposon protein putative Ty3-gypsy subclass
OsISM03114	LOC_Os01g22830	12828303	AGTTGCATCTGTTCCACCAA	GTGGAGGCGAGGATTCTGT	59.7	172	88	retrotransposon protein putative Ty3-gypsy subclass
OsISM03115	LOC_Os01g22830	12828770	GGATGTTGATCAAGCTGCAA	TTTGAGAGATGGTGCTGTCG	59.9	229	71	retrotransposon protein putative Ty3-gypsy subclass
OsISM03116	LOC_Os01g22830	12828979	CGACAGCACCATCTCTCAAA	GTGACTCCCTACAGCCGATG	60.3	168	72	retrotransposon protein putative Ty3-gypsy subclass
OsISM03117	LOC_Os01g22830	12829165	CAACCAGCTGCTCCTGCTAT	TCAAAAGAAAACAGATCGGC	59.3	130	48	retrotransposon protein putative Ty3-gypsy subclass
OsISM03118	LOC_Os01g22830	12829268	GGCTTTAGCCGATCTGTTTTC	GCCACCAGATTATCCAGGAA	60.1	158	94	retrotransposon protein putative Ty3-gypsy subclass
OsISM03119	LOC_Os01g22860	12843805	TCCCTCGTCATCGCCACT	CCGCTGCTGTTTATTTTGATT	61.1	452	99	retrotransposon protein putative unclassified
OsISM03120	LOC_Os01g22870	12848004	GAAGAACTCAAGCGGAGTGG	GGATTTGCATAATTCTCGCC	59.8	475	77	expressed protein
OsISM03121	LOC_Os01g22870	12848460	GCGAGAATTATGCAAATCCAA	TATGTAGAACGCCAATGCCC	60.5	173	72	expressed protein
OsISM03122	LOC_Os01g22870	12848757	ATGAGATCGATGATGAGGGC	ACCAGAACAGCCATGAGTCC	60.1	180	97	expressed protein
OsISM03123	LOC_Os01g22870	12848923	ATGGCTGTTCTGGTGGACTC	ATCCCAAGAACAATCCATGC	59.9	2224	55	expressed protein
OsISM03124	LOC_Os01g22870	12851215	CAAACATTGTTGGCTTCACC	TGTTGACCTGACACCAGATGA	59.6	746	96	expressed protein
OsISM03125	LOC_Os01g22890	12860772	AGGGTCACTCTGTGGCTGAC	GCCAAGAACGCTAAGAATGC	60.2	141	97	retrotransposon protein putative Ty1-copia subclass
OsISM03126	LOC_Os01g22890	12864364	TGCCGGCTATCCTTATGAAC	CCGGAGTTTCTTAATTTCCTGA	59.8	130	81	retrotransposon protein putative Ty1-copia subclass
OsISM03127	LOC_Os01g22890	12864583	CGAAGGAGATGGGTTTGAGA	GACTGCGACCAACAGTCAAG	59.8	288	73	retrotransposon protein putative Ty1-copia subclass
OsISM03128	LOC_Os01g22900	12870014	AACTTCCTCCTCCACACCCT	CCCAGGGCTGTAACAATCAA	60.4	774	64	neutral/alkaline invertase putative expressed
OsISM03129	LOC_Os01g22900	12870862	TTTGGACCCTGACTTTGGAG	TGCCCTCAGCAAAATAATCC	60.1	169	83	neutral/alkaline invertase putative expressed
OsISM03130	LOC_Os01g22900	12871176	GGAGGATGGGAATACATGGG	CCCATCGTTCATAACAAGCA	60.4	348	93	neutral/alkaline invertase putative expressed

ISM IDs	Rice gene locus IDs	Physical positions (bp) on chromosomes	Forward primers (5'-3')	Reverse primers (5'-3')	Melting tempearature (T˚C)	Expected product size (bp) in PCR	Expected product size (bp) in Q-PCR	Putative Functions
OsISM03131	LOC_Os01g22900	12871896	ATATGTTACCCCGCAATGGA	GCCAGGATCCACCATTATGA	60.4	459	95	neutral/alkaline invertase putative expressed
OsISM03132	LOC_Os01g22900	12872335	TCATAATGGTGGATCCTGGC	CCCATTTTGATACAGGCCAA	60.7	153	61	neutral/alkaline invertase putative expressed
OsISM03133	LOC_Os01g22954	12895655	CCCTCTCAGAAGCCTCTCCT	TCCAACTGATGAGCAACCTG	60.0	152	58	serine carboxypeptidase putative expressed
OsISM03134	LOC_Os01g22954	12895822	GAACTAGGGCCCCTCAGAGT	GGGACTCCAAGAACAGCAAA	60.0	205	95	serine carboxypeptidase putative expressed
OsISM03135	LOC_Os01g22954	12896055	CACATCCTCTGACCTCACCA	TGTGGAAATCTGTCCAACCA	59.8	179	88	serine carboxypeptidase putative expressed
OsISM03136	LOC_Os01g22954	12896203	TCCTGGTAAATTGGTTGGACA	TGCAAGCTGTGGCACATAGT	60.3	776	96	serine carboxypeptidase putative expressed
OsISM03137	LOC_Os01g22954	12896999	CAAAGACAAGAAGGCCAACA G	TTCAACAAGACCTTTTGAGTCG	59.6	460	95	serine carboxypeptidase putative expressed
OsISM03138	LOC_Os01g22954	12897645	GTAGCTGCTTTCGATCACGC	CGCGTCATATCCTGAGAACA	60.5	151	82	serine carboxypeptidase putative expressed
OsISM03139	LOC_Os01g22954	12897876	CCCTGGAAAATGGAAGGTTT	CAAGACAGAAAGCACCGAAA	59.6	15533	68	serine carboxypeptidase putative expressed
OsISM03140	LOC_Os01g22954	12913433	GCAGGACTCAGAATTTGGCT	TACCTTGACCCGATCACTGG	60.2	289	66	serine carboxypeptidase putative expressed
OsISM03141	LOC_Os01g22954	12913757	ACTGGCAACCTTGGTACCTG	GGTACCAAATGTCCAGCACC	60.1	242	98	serine carboxypeptidase putative expressed
OsISM03142	LOC_Os01g23020	12949360	GTCGATGGACATTCTCCAGG	AAGCCAGTCATGTCCAAACC	60.2	1194	92	retrotransposon protein putative Ty3-gypsy subclass
OsISM03143	LOC_Os01g23020	12951253	CTCCGGCGTTGGTAGCAC	TGGTCGTCACCATCTGAAAA	61.4	331	87	retrotransposon protein putative Ty3-gypsy subclass
OsISM03144	LOC_Os01g23070	12981007	CGGCCTCATGGAGTCTGT	GATCTTGTCGGTGACGGTGT	60.4	203	50	hypothetical protein
OsISM03145	LOC_Os01g23090	12991313	GTGGGTTGATACGGGAGCTA	CAAGAACAGCAGCACCTGAA	60.1	119	81	retrotransposon protein putative Ty1-copia subclass
OsISM03146	LOC_Os01g23090	12991454	GCTCACTTCAGGGAAAATCAT C	CACCACGATCTGACCGTAGAC	60.3	644	99	retrotransposon protein putative Ty1-copia subclass
OsISM03147	LOC_Os01g23090	12992540	CATGGTAACGGAATCTCGTG	CTTGCAACCAAATGGTCGAT	59.9	360	98	retrotransposon protein putative Ty1-copia subclass
OsISM03148	LOC_Os01g23110	12998070	ATTCGAACCAGTCGGGAAG	ATCATCGGGGAGTTTCATCA	60.2	188	84	retrotransposon protein putative Ty3-gypsy subclass

ISM IDs	Rice gene locus IDs	Physical positions (bp) on chromosomes	Forward primers (5'-3')	Reverse primers (5'-3')	Melting tempearature (T˚C)	Expected product size (bp) in PCR	Expected product size (bp) in Q-PCR	Putative Functions
OsISM03149	LOC_Os01g23120	13000854	CCCCTAGATCCCTCTGATCC	GCATATCGGATGGTTTCCAC	60.0	128	83	retrotransposon protein putative Ty3-gypsy subclass
OsISM03150	LOC_Os01g23120	13001155	GCCAACCCAGTCCTAGTTCA	TTCCTTTAGTCGGATCTGATGAT	59.6	195	88	retrotransposon protein putative Ty3-gypsy subclass
OsISM03151	LOC_Os01g23160	13018002	GAAGCGGACGGAGGTGTC	CTGTTGCTGGACTGTAGGCA	60.9	1057	95	retrotransposon protein putative unclassified
OsISM03152	LOC_Os01g23210	13047640	CTGGCTCTGCCATCCTCTT	CTTCCCCTCTCTCTCAAGCC	60.5	255	98	retrotransposon protein putative unclassified
OsISM03153	LOC_Os01g23210	13048017	TGTAGGTCAGCGACAGCACT	GTCATCGGATCCATCATTCC	59.9	3884	77	retrotransposon protein putative unclassified
OsISM03154	LOC_Os01g23270	13075635	GACCACCGAATCCGATAAAA	CGAGCGGTCGAACTTAATTG	60.3	674	66	retrotransposon protein putative Ty3-gypsy subclass
OsISM03155	LOC_Os01g23270	13076776	CCAAACCCACGAGATCACTC	TTGAAGAAAGGTGATGAGCG	59.8	217	95	retrotransposon protein putative Ty3-gypsy subclass
OsISM03156	LOC_Os01g23300	13085080	TATGGCTAGGATGGGTTGGA	ACTCCTCTTCTGGGTTTGGG	60.4	555	90	retrotransposon protein putative unclassified
OsISM03157	LOC_Os01g23310	13089302	CTGCTCGTGACTTCCTGAGA	AGCCATCATCTTGCTGCTCT	59.7	1015	94	retrotransposon putative centromere-specific
OsISM03158	LOC_Os01g23310	13090297	AGAGCAGCAAGATGATGGCT	GCGGCTGTTAAATGTCGATT	60.1	1496	68	retrotransposon putative centromere-specific
OsISM03159	LOC_Os01g23310	13091810	CTTAGCACTTTGCTCGTCCC	CAGGTAAGCAGGTAAGCACG	59.3	1295	87	retrotransposon putative centromere-specific
OsISM03161	LOC_Os01g23350	13129492	TGCAGACTTTGTCCCATGCT	CGCTGATGACACCCTTATCA	60.7	494	90	hypothetical protein
OsISM03162	LOC_Os01g23370	13137091	GGACAAGCCGGATATTAGGA	GACGAGGCCATGTTAACGG	60.2	112	67	hypothetical protein
OsISM03163	LOC_Os01g23370	13137954	GTCCTAATCGTTGTCGGAGC	GCTTCCCTTCTTTGAATGGC	60.4	1020	77	hypothetical protein
OsISM03164	LOC_Os01g23370	13139628	CCACGTGGACCGAGAAAC	GAGCCGCTCGTACCTCTG	59.9	114	78	hypothetical protein
OsISM03165	LOC_Os01g23390	13144215	ACGGAGATGACGGAGATGAC	CCAATGCTAAGGCAAAGGTC	59.9	375	97	hypothetical protein
OsISM03166	LOC_Os01g23420	13155274	AAACATTTTGATGAGCTTGCAG	AAAGTTTGCCATTCCTAAACAAA	59.3	429	82	transposon protein putative unclassified
OsISM03167	LOC_Os01g23420	13156596	GCATGTGCCAAAGTACGAGT	CCACGCTTTGTGGTAGCTAAT	59.1	216	98	transposon protein putative unclassified

ISM IDs	Rice gene locus IDs	Physical positions (bp) on chromosomes	Forward primers (5'-3')	Reverse primers (5'-3')	Melting tempearature (T˚C)	Expected product size (bp) in PCR	Expected product size (bp) in Q-PCR	Putative Functions
OsISM03168	LOC_Os01g23420	13157485	AGAAAAATGACCAACCAGCC	GACGTATGCTGTGCTGTCCA	60.0	617	84	transposon protein putative unclassified
OsISM03171	LOC_Os01g23490	13188308	GCCAGTTCGGTCTGAATCAT	AGTCCAATGGCGTTTGGTAG	60.0	615	78	retrotransposon protein putative Ty1-copia subclass
OsISM03172	LOC_Os01g23510	13199703	ACTGGCAGCATCACGTCTC	CAACTAGATCCGCCCACG	60.1	1455	79	hypothetical protein
OsISM03173	LOC_Os01g23530	13211074	CTTCCTCAACTATGAGCCGC	TCAACACTTCAGCCCTTCCT	59.9	1631	66	terpene synthase putative
OsISM03174	LOC_Os01g23530	13212889	TCTTCTGCGAAAGAATGGCT	TGTCATCAACAATGAAATTCCC	59.9	1067	84	terpene synthase putative
OsISM03175	LOC_Os01g23530	13214205	TTGCGAAACTGAATTTCAACC	TGATTTGACTTTAAGCTCCTTCC	59.5	201	90	terpene synthase putative
OsISM03176	LOC_Os01g23530	13214560	CAGAAGAGAGTATGCTACTTG CTGA	TCTGGGAGCAGTCCTATTGC	59.9	1456	68	terpene synthase putative
OsISM03177	LOC_Os01g23530	13216067	GGAAGAGCTTGGACCTGAGA	TAATCCTCGTCACGCCATTT	60.0	781	85	terpene synthase putative
OsISM03178	LOC_Os01g23530	13217008	TTTGTGCGACTCTCCAATGA	TAGTGGTCCCCAGTTTGCTC	60.3	259	57	terpene synthase putative
OsISM03179	LOC_Os01g23540	13220306	AGACAAGGAACAAGGAGCGA	CGACATCGTCGATAGCCAA	60.4	151	66	retrotransposon protein putative unclassified
OsISM03180	LOC_Os01g23540	13221077	CGTCATCGTCATCATCCTTG	CCTTGGAAGTTGGCTACGAA	60.2	366	87	retrotransposon protein putative unclassified
OsISM03181	LOC_Os01g23540	13222230	CGATATCCGATCGTGGTTG	GCCGAAAGTCACAGTCAACA	59.9	219	88	retrotransposon protein putative unclassified
OsISM03182	LOC_Os01g23550	13223607	ATCGAGAACATGAAGTCGCC	GTGCAATGAATGCCTCTTCG	61.0	178	97	retrotransposon protein putative unclassified
OsISM03183	LOC_Os01g23570	13232288	CTTCCGTGATTAGTCCCACA	AAATCCTCGTCCAGGAATCA	59.0	533	84	hypothetical protein
OsISM03184	LOC_Os01g23580	13236571	GAGATCCAAAGCGCCATTC	TTGCGTGAACAAGAACGAGT	60.1	5363	49	inorganic H+ pyrophosphatase putative expressed
OsISM03185	LOC_Os01g23580	13242427	ATCTTGTCGGCAAGGTTGAG	TTGTCACCCACATTGTCAGC	60.4	470	80	inorganic H+ pyrophosphatase putative expressed
OsISM03186	LOC_Os01g23580	13243220	ACTTTGGAGAGGTGAAGCGA	CGATTGTGACGCAGAAGAAC	59.7	157	55	inorganic H+ pyrophosphatase putative expressed
OsISM03187	LOC_Os01g23580	13243408	CACCGAGTACTTCACCAGCA	GAGCCCGAAGATGACGTTC	60.3	220	92	inorganic H+ pyrophosphatase putative expressed

ISM IDs	Rice gene locus IDs	Physical positions (bp) on chromosomes	Forward primers (5'-3')	Reverse primers (5'-3')	Melting tempearature (T˚C)	Expected product size (bp) in PCR	Expected product size (bp) in Q-PCR	Putative Functions
OsISM03189	LOC_Os01g23580	13244323	ACCTTCTTCGGCGTCCAG	CTGTTGGAAGCAGATATGGC	59.8	168	84	inorganic H+ pyrophosphatase putative expressed
OsISM03190	LOC_Os01g23580	13244500	CTGGGACAATGCCAAGAAAT	CTTGGGACCAAGTGCCTTT	60.0	195	65	inorganic H+ pyrophosphatase putative expressed
OsISM03191	LOC_Os01g23610	13271984	TACGACCTCGTGATCATTGG	ACATCCCCCTCAATTATGGC	60.2	2526	96	dihydrolipoyl dehydrogenase putative expressed
OsISM03192	LOC_Os01g23610	13274572	GTCGGATGAGGGAACTTCAA	ATCTGCAACAGCCTGCCTAT	60.0	360	90	dihydrolipoyl dehydrogenase putative expressed
OsISM03193	LOC_Os01g23610	13275005	TGACTGGTTTTGGCACCAT	GGCAGTGATTTCATTGTCAGG	60.2	491	78	dihydrolipoyl dehydrogenase putative expressed
OsISM03194	LOC_Os01g23610	13275522	TGTTCCTTTTGTCCCCAATG	CGATAGCAATCCAGTCAGGAA	60.5	1564	99	dihydrolipoyl dehydrogenase putative expressed
OsISM03195	LOC_Os01g23610	13277090	AAGCGGTTACATTGGACTGG	TCAAAACCAGGCATCAACTG	59.8	456	97	dihydrolipoyl dehydrogenase putative expressed
OsISM03196	LOC_Os01g23610	13277602	ATCATACTGGGGTCTTTGCG	CACGGGTTTACCATCCTTTG	60.1	140	54	dihydrolipoyl dehydrogenase putative expressed
OsISM03197	LOC_Os01g23610	13277729	TGGTAAACCCGTGTTGATTG	GCCCTTCCGGTAGCTATGAG	60.2	586	94	dihydrolipoyl dehydrogenase putative expressed
OsISM03198	LOC_Os01g23610	13278288	TGCAGCTCTCATAGCTACCG	ATCAACAGGAACAAAACCGC	59.7	174	95	dihydrolipoyl dehydrogenase putative expressed
OsISM03199	LOC_Os01g23610	13278442	GCGGTTTTGTTCCTGTTGAT	GCCATTGGCATCTCCAATAC	60.1	1128	90	dihydrolipoyl dehydrogenase putative expressed
OsISM03200	LOC_Os01g23610	13279571	AACTCATGCTGGCTCATGCT	TGTCTTTCCCAGAGATTCGC	60.7	178	67	dihydrolipoyl dehydrogenase putative expressed
OsISM03201	LOC_Os01g23610	13279793	CCGGAGATTAGTATGGTTGGG	TTTGCCTTGAAGCTGGTCTT	60.3	325	99	dihydrolipoyl dehydrogenase putative expressed
OsISM03202	LOC_Os01g23610	13280125	GCTCTGGCTGAGAATGAAGG	TCAGCAGCATGCAAACCTAA	60.3	260	99	dihydrolipoyl dehydrogenase putative expressed
OsISM03203	LOC_Os01g23610	13280388	CATCCACGAGGCATCAAAC	ACAAAGTTGGGTGTGCATGA	60.0	150	81	dihydrolipoyl dehydrogenase putative expressed
OsISM03204	LOC_Os01g23610	13280519	CATGCACACCCAACTTTGTC	TTACGGAATGAGGAACACCTG	60.0	165	80	dihydrolipoyl dehydrogenase putative expressed
OsISM03205	LOC_Os01g23620	13285526	AAGACCACCCTCCTCCACAT	ACTTGATCTTCCCGATGCTG	60.5	233	98	miro putative expressed
OsISM03206	LOC_Os01g23620	13285796	CCGCGTCTGGAAGGACTACT	TTGACTCAGCAAAACGTTCCT	60.8	1061	84	miro putative expressed

ISM IDs	Rice gene locus IDs	Physical positions (bp) on chromosomes	Forward primers (5'-3')	Reverse primers (5'-3')	Melting tempearature (T˚C)	Expected product size (bp) in PCR	Expected product size (bp) in Q-PCR	Putative Functions
OsISM03207	LOC_Os01g23630	13290797	ACCACCCTCAAGCGCAAG	ATCCCCAAATCCGTACATCA	61.2	154	67	transcription initiation factor IID 18kD subunit family protein expressed
OsISM03208	LOC_Os01g23630	13290892	TTTATGCTGCATTTTTCCCC	CAAATTGCATCCTATCCCCA	60.3	145	99	transcription initiation factor IID 18kD subunit family protein expressed
OsISM03209	LOC_Os01g23630	13291002	AGGCTAGGAATTGTTTGGGG	GTCCTCCACAAGTGCAACAG	59.8	243	93	transcription initiation factor IID 18kD subunit family protein expressed
OsISM03210	LOC_Os01g23630	13291225	CTGTTGCACTTGTGGAGGAC	TCTTTGATGCAACGTTCTGG	59.6	1973	76	transcription initiation factor IID 18kD subunit family protein expressed
OsISM03211	LOC_Os01g23630	13293199	GGGCAAGCTCTTGACAGAAG	TGGAGAGTAGCTCAGTGGCA	59.9	169	81	transcription initiation factor IID 18kD subunit family protein expressed
OsISM03212	LOC_Os01g23640	13295531	AGCTCCCCTACTACTTCCGC	CGCAGGAGGATGACATACAG	59.6	342	98	mov34/MPN/PAD-1 family protein expressed
OsISM03213	LOC_Os01g23640	13295853	CTGTATGTCATCCTCCTGCG	AGTCACGGTGCTTTGGAATC	59.7	147	62	mov34/MPN/PAD-1 family protein expressed
OsISM03214	LOC_Os01g23640	13296024	TGGATTTCTTCAGGCTTGCT	GCACAACTGGCTTCAGTGAC	59.7	222	82	mov34/MPN/PAD-1 family protein expressed
OsISM03215	LOC_Os01g23640	13296304	AAATGGAACCATTGCTGTAAA	TCAAATGATCCATTGTTGCTTC	58.6	181	94	mov34/MPN/PAD-1 family protein expressed
OsISM03216	LOC_Os01g23640	13296536	ACCTGATAGACTGACCCGCA	CCCATGAAGCCCATTAGGT	60.2	967	98	mov34/MPN/PAD-1 family protein expressed
OsISM03217	LOC_Os01g23640	13297505	ATGGACTGGGCCTGTTACTG	TTCTCACCTAGACACATCGCTT	59.5	184	80	mov34/MPN/PAD-1 family protein expressed
OsISM03218	LOC_Os01g23640	13297631	GCAGGTTCAGTATCCAAGCA	TGGGACCATTCTGACCATCT	59.6	1937	93	mov34/MPN/PAD-1 family protein expressed
OsISM03219	LOC_Os01g23640	13299793	CACCAGGACTTGCTGTCTCA	GTTTGCCTCAGCAACCCTTA	60.1	264	93	mov34/MPN/PAD-1 family protein expressed
OsISM03220	LOC_Os01g23640	13300074	AACATGTGGAGTTCTTGCTGG	GCTTTGGAATTATCAAGGCA	59.0	159	69	mov34/MPN/PAD-1 family protein expressed
OsISM03221	LOC_Os01g23640	13300214	GCCTTGATAATTCCAAAGCA	GAGCCCTTGTCCTGAACATC	58.8	234	87	mov34/MPN/PAD-1 family protein expressed
OsISM03222	LOC_Os01g23640	13300428	GATGTTCAGGACAAGGGCTC	ATGGAAGACAGGAAGCAGGA	59.7	1081	78	mov34/MPN/PAD-1 family protein expressed
OsISM03223	LOC_Os01g23640	13301475	CACATCCAACACAGTCCTGC	TTGCAATTGCTTCAGGTAACA	59.8	1571	85	mov34/MPN/PAD-1 family protein expressed
OsISM03224	LOC_Os01g23640	13303018	CAGGTGATGTTACCTGAAGCA A	ATACCGCCAGGATCAGAGAG	59.7	803	93	mov34/MPN/PAD-1 family protein expressed

ISM IDs	Rice gene locus IDs	Physical positions (bp) on chromosomes	Forward primers (5'-3')	Reverse primers (5'-3')	Melting tempearature (T°C)	Expected product size (bp) in PCR	Expected product size (bp) in Q-PCR	Putative Functions
OsISM03225	LOC_Os01g23660	13310946	AGAGGAGAAGACGGGTTGCT	TTCAATCTCCTTCGATTCCG	60.3	335	87	hypothetical protein
OsISM03226	LOC_Os01g23670	13313536	TTTAGACAGAGCACCTGGGC	GGTTAAACCCCTTAGCCTGC	60.2	465	93	retrotransposon protein putative unclassified
OsISM03227	LOC_Os01g23690	13326431	CCTCATCTCCCCTCTCTTCA	ACCCTTATCCGTGCCCAT	59.7	332	97	hypothetical protein
OsISM03229	LOC_Os01g23740	13352844	ACCTTCGAGAAGGAGGTCG	CAAGCTTCTTGCAGTGACCA	59.8	1207	80	OsPDIL2-2 protein disulfide isomerase PDIL2-2 expressed
OsISM03230	LOC_Os01g23740	13354045	AGCTTGCCCCTGAGTATGAA	GCTGCACACACTCTTGTGCT	60.0	294	99	OsPDIL2-2 protein disulfide isomerase PDIL2-2 expressed
OsISM03231	LOC_Os01g23740	13354363	ACAATTCAATGGTTCCCCAA	CTCTGCACTGCGTTGTCCT	60.1	152	64	OsPDIL2-2 protein disulfide isomerase PDIL2-2 expressed
OsISM03232	LOC_Os01g23740	13354514	GGCCCTTGCTGAATTTGTTA	GGGCCAAGCACTACAACACT	60.1	297	82	OsPDIL2-2 protein disulfide isomerase PDIL2-2 expressed
OsISM03233	LOC_Os01g23740	13354803	CTTGGCCCAGACAACTTTGA	AGCAAGATGCTTGCAGTGG	60.7	206	97	OsPDIL2-2 protein disulfide isomerase PDIL2-2 expressed
OsISM03234	LOC_Os01g23740	13354971	TTTTGATCCATAGGTGTGGC	GCAATTACAACTCCATCGTCC	58.9	198	92	OsPDIL2-2 protein disulfide isomerase PDIL2-2 expressed
OsISM03235	LOC_Os01g23740	13355172	CCTTGATGCTGACAAACACAA	TCACCAGCTTTGTTTCCCTT	59.7	304	94	OsPDIL2-2 protein disulfide isomerase PDIL2-2 expressed
OsISM03236	LOC_Os01g23740	13355529	AGTGTGGTACCAGCCGTGAT	GAGGGCATCCAAACTTGCTA	60.3	165	72	OsPDIL2-2 protein disulfide isomerase PDIL2-2 expressed
OsISM03237	LOC_Os01g23740	13355750	GGAAGAGGAGGTTGTGAAGC	CGTGACCTTTGTCCAGGATT	59.4	183	90	OsPDIL2-2 protein disulfide isomerase PDIL2-2 expressed
OsISM03238	LOC_Os01g23740	13355952	AGAGAGGCTTGAACGCATGT	GAATTCATCGGCCTTTGATG	60.2	164	56	OsPDIL2-2 protein disulfide isomerase PDIL2-2 expressed
OsISM03239	LOC_Os01g23770	13365460	GGAAGGAGACCGTGATGAAA	AGGTACTCCCGGTGCGTG	61.1	182	98	OsMADS94 - MADS-box family gene with M-beta type-box
OsISM03240	LOC_Os01g23770	13366011	AGATGGCATCAGCTTCGC	GTTCGGTGCGTTGATGAAGT	60.6	338	95	OsMADS94 - MADS-box family gene with M-beta type-box
OsISM03241	LOC_Os01g23780	13372834	CGAGAGTGCCTCGAGTTGA	CTCCTCCGCCGTACTGATG	61.0	123	71	OsMADS95 - MADS-box family gene with M-beta type-box
OsISM03243	LOC_Os01g23830	13410003	TTGTCCTTCCGACAAGAACC	ACGTGCTAGTCCCACCGAT	60.3	458	99	retrotransposon protein putative unclassified
OsISM03244	LOC_Os01g23940	13467116	CTGTCGAACTCCCTCCGTT	GATGTCGGAGACGCGTAGAT	60.2	450	77	hypothetical protein

ISM IDs	Rice gene locus IDs	Physical positions (bp) on chromosomes	Forward primers (5'-3')	Reverse primers (5'-3')	Melting tempearature (T˚C)	Expected product size (bp) in PCR	Expected product size (bp) in Q-PCR	Putative Functions
OsISM03245	LOC_Os01g23940	13468308	CGTTCGTCCCGAGTTGTC	GATCGTGGAGGAAGATGACG	60.4	102	54	hypothetical protein
OsISM03246	LOC_Os01g23970	13487660	TCTTCAAGGAGACCAGCACC	CTCACGATTTCCCTTTCCAA	60.2	747	92	cysteine-rich receptor protein kinase putative
OsISM03247	LOC_Os01g23970	13488489	GGAGAAGGTGGATTTGGTCA	AATCCCTGTGCAGAATTTGG	59.9	246	93	cysteine-rich receptor protein kinase putative
OsISM03248	LOC_Os01g23970	13488851	CTAATGGAAGCTTGGATGGC	CTGTCACACTGGAGAGGCAA	59.8	375	67	cysteine-rich receptor protein kinase putative
OsISM03250	LOC_Os01g23980	13496021	CCGTGTTCCGTTTCCACTAC	CCCACCAATCTACAGCAGGT	60.2	1060	94	retrotransposon protein putative Ty3-gypsy subclass
OsISM03251	LOC_Os01g23980	13498447	CCGAATGGCAGCTAATGAGT	GTTGTCGATGAACACCACGA	60.4	448	89	retrotransposon protein putative Ty3-gypsy subclass
OsISM03252	LOC_Os01g24010	13516831	CTTCCTGCTCAAGCTCAAGG	CACCTCTGCCTCCACCTCTA	60.3	1850	95	pleiotropic drug resistance protein putative expressed
OsISM03253	LOC_Os01g24010	13518705	ACGCTCCTCAACTCCATCAT	CTTCCTGGTGGGGGAGAT	59.8	1838	70	pleiotropic drug resistance protein putative expressed
OsISM03254	LOC_Os01g24010	13520563	TGACGTCAGCGGAATAATCA	AGCAACGTTGTCTTGCCTG	60.1	192	76	pleiotropic drug resistance protein putative expressed
OsISM03255	LOC_Os01g24010	13520765	GCTGGGAAGCTTGAAGACAA	AGGCACAAACTCATCCATCC	60.2	233	76	pleiotropic drug resistance protein putative expressed
OsISM03256	LOC_Os01g24010	13521048	GACAGTGAGGGAGACCTTGG	TCTGCTTTTTCCCTCCTTGA	59.8	168	94	pleiotropic drug resistance protein putative expressed
OsISM03257	LOC_Os01g24010	13521196	TCAAGGAGGGAAAAAGCAGA	TCAGTGACAACGCTGGACTC	60.0	236	99	pleiotropic drug resistance protein putative expressed
OsISM03258	LOC_Os01g24010	13521406	GGACAAGAGTCCAGCGTTGT	CCCTACCACTGTGTCTGCAC	59.7	165	76	pleiotropic drug resistance protein putative expressed
OsISM03259	LOC_Os01g24010	13521583	AGGGGTGTATCTGGTGGACA	TGAACAGAGCTCTTGCTGGA	60.0	233	74	pleiotropic drug resistance protein putative expressed
OsISM03260	LOC_Os01g24010	13522019	TTCTTCGAGTTGATGGGCTT	TGTTCCTGGTCTTTCTTTGATG	59.5	186	87	pleiotropic drug resistance protein putative expressed
OsISM03261	LOC_Os01g24010	13522418	CATGAAGAGGAACTCGTTTGTG	TACGAGGAACGCGGTAAGAG	60.1	152	65	pleiotropic drug resistance protein putative expressed
OsISM03262	LOC_Os01g24010	13522834	GTTGGCTTTGATCCCAATGT	TGCCAGCAATAAATCGAAAC	59.3	255	98	pleiotropic drug resistance protein putative expressed
OsISM03263	LOC_Os01g24010	13523146	CAGCACTGGGTGGCTTTATT	TCCAGTAACCCCAAATCCAC	59.9	187	64	pleiotropic drug resistance protein putative expressed

ISM IDs	Rice gene locus IDs	Physical positions (bp) on chromosomes	Forward primers (5'-3')	Reverse primers (5'-3')	Melting tempearature (T°C)	Expected product size (bp) in PCR	Expected product size (bp) in Q-PCR	Putative Functions
OsISM03264	LOC_Os01g24010	13523374	GAATTTCTAGGGCGCAGTTG	GCCTCGAGATTTCAGAATGC	59.9	154	79	pleiotropic drug resistance protein putative expressed
OsISM03265	LOC_Os01g24010	13523593	CAACCTCCTTTACACTGTGGC	ACGCTTTTCCTTCAGTGCAT	59.8	207	89	pleiotropic drug resistance protein putative expressed
OsISM03266	LOC_Os01g24010	13523929	CTCCCCTTTGCACAACTGTC	GCGCTCTTCTGTTACTCCTTG	60.0	194	94	pleiotropic drug resistance protein putative expressed
OsISM03267	LOC_Os01g24010	13524370	TATTTTCTGCTTGGATGCGG	CAAGCTCCATGACCTCCTCT	60.2	181	79	pleiotropic drug resistance protein putative expressed
OsISM03268	LOC_Os01g24010	13524767	GCACAATTCACCAGCCAAGT	GGCCCAATGGACCAACATA	61.3	196	97	pleiotropic drug resistance protein putative expressed
OsISM03269	LOC_Os01g24010	13524958	GGGCCAGAATTCCTCAAAAC	CATCCATGTTGCTGGATTGT	60.1	192	89	pleiotropic drug resistance protein putative expressed
OsISM03270	LOC_Os01g24010	13525174	GAGGAGATGCTTGGGATTGA	CCAGGAGTAGGTGTGCTTAGG	59.5	206	99	pleiotropic drug resistance protein putative expressed
OsISM03271	LOC_Os01g24010	13525533	AACAATGTTTTGGGACCTCG	CTGCAGCATACATTGATCCC	59.5	172	78	pleiotropic drug resistance protein putative expressed
OsISM03272	LOC_Os01g24010	13525801	CAGGTTTCCCGTATGCATTT	GAACTAGGATGTATGGCAATTCG	59.9	863	55	pleiotropic drug resistance protein putative expressed
OsISM03273	LOC_Os01g24010	13526849	GCATGGAACCTCTTCTCAGG	CACCTCCACCAGACAGGAAT	59.9	160	60	pleiotropic drug resistance protein putative expressed
OsISM03275	LOC_Os01g24040	13542790	GATACAAACCATGGCTAAGGA A	TGATGGTTTTCTCCCAATCC	58.9	859	95	retrotransposon protein putative Ty1-copia subclass
OsISM03276	LOC_Os01g24040	13544283	GCGAAGATTTCTTTGACGCT	TCCTCATCCAGCTCTCCATT	59.7	143	77	retrotransposon protein putative Ty1-copia subclass
OsISM03277	LOC_Os01g24040	13544614	GGGGTGAGGGAGTTATTCTG	GGATAACATCAGCTACTCCCAA	58.3	138	88	retrotransposon protein putative Ty1-copia subclass
OsISM03278	LOC_Os01g24040	13545044	TGAGCGAGTAATGCGCTATC	GGTTGACCTCGTCAAAATGG	59.8	205	80	retrotransposon protein putative Ty1-copia subclass
OsISM03279	LOC_Os01g24040	13545343	TGCCGGCTATCCTTATGAAC	TCGTGATAGTCCCTTCGTGA	59.7	200	70	retrotransposon protein putative Ty1-copia subclass
OsISM03280	LOC_Os01g24040	13545562	CGAAGGAGATGGGTTTGAGA	AAACGAATCCGAATTTGCAG	60.1	932	53	retrotransposon protein putative Ty1-copia subclass
OsISM03281	LOC_Os01g24040	13546483	CGGATTCGTTTCTGTTTTGG	AGATGTTCCGGAGACCTGG	60.3	293	99	retrotransposon protein putative Ty1-copia subclass
OsISM03282	LOC_Os01g24040	13546782	GACTTCCTGCACCAGGAGAA	GGAGATTAAGAAATTCAGCCCA	60.0	885	58	retrotransposon protein putative Ty1-copia subclass

ISM IDs	Rice gene locus IDs	Physical positions (bp) on chromosomes	Forward primers (5'-3')	Reverse primers (5'-3')	Melting tempearature (T°C)	Expected product size (bp) in PCR	Expected product size (bp) in Q-PCR	Putative Functions
OsISM03283	LOC_Os01g24050	13549451	AAGTTTGCACATCCAGGTCC	ATTGCGAAGCTCAAGAGCAC	60.3	4204	95	expressed protein
OsISM03285	LOC_Os01g24060	13560317	AGTCGACCACGCAGTTCAG	GAGGAATTCCACAAAGCGAG	59.9	1459	99	importin subunit alpha putative expressed
OsISM03286	LOC_Os01g24060	13561772	CCTCACCAGGGAGGAGTACC	ATATTGGTGAGAGCCCATGC	60.4	280	58	importin subunit alpha putative expressed
OsISM03287	LOC_Os01g24060	13562130	ACCAAGAGAGGATGTCCGTG	AACCAGGTTACGGCATTTTG	60.0	192	80	importin subunit alpha putative expressed
OsISM03288	LOC_Os01g24060	13562393	CAGTGCCTGGACCCTTTCTA	GAGAGAGGATGAGCTCCCG	60.1	184	99	importin subunit alpha putative expressed
OsISM03289	LOC_Os01g24060	13562660	GAAACTGGCGTGTGTGATTG	GAATGAGCACCACAGGTGAA	59.9	152	62	importin subunit alpha putative expressed
OsISM03290	LOC_Os01g24060	13562822	CACGGTTGGCAACATTGTAA	AAAGACAGGGAAGTGCTTGG	59.9	1566	78	importin subunit alpha putative expressed
OsISM03291	LOC_Os01g24060	13564447	TCCAACATCACAGCTGGAAC	TGCAACAGATGCAATAGAGGA	59.6	255	81	importin subunit alpha putative expressed
OsISM03292	LOC_Os01g24060	13564729	GGCTGTTTGGGCAATTTCTA	TAAATCACAAAGCGGCTTGA	59.8	194	95	importin subunit alpha putative expressed
OsISM03293	LOC_Os01g24060	13565293	TTCAGTTTGGCAGTTTCGGT	TGAACAGACAGCTACGGTGC	60.4	154	62	importin subunit alpha putative expressed
OsISM03294	LOC_Os01g24070	13569768	TCTCTTCTCCAACCAACGCT	AGCTCCTCCTCCCTTCCTC	59.9	223	91	GATA zinc finger domain containing protein expressed
OsISM03295	LOC_Os01g24070	13569973	AGGAAGGGAGGAGGAGCTG	TCCGCAAGCGTTGCATAG	61.5	1403	96	GATA zinc finger domain containing protein expressed
OsISM03296	LOC_Os01g24080	13579018	ATTGTACATTGCCCCCTCTG	TTTCGGTGTAGTTGTTGGGA	59.4	716	92	hypothetical protein
OsISM03297	LOC_Os01g24100	13591295	GATTGCTGCATGGAAGAGGT	GCAGACAATCCCCTTAATTTACA	59.8	471	76	transposon protein putative unclassified
OsISM03298	LOC_Os01g24120	13594737	TGGGTGTTTTGTTGAGGAAA	ACCAATGAGCAGAACAACCC	59.5	231	82	hypothetical protein
OsISM03299	LOC_Os01g24120	13594935	TCTTTGAGGCTGAGGGTTGT	CCATTTCAATGAGGTCCGAG	60.1	229	72	hypothetical protein
OsISM03300	LOC_Os01g24120	13595199	ATCCCAGAACCTTGCTGTTG	CCTGTTCCACTAAGTGCTCG	59.3	555	97	hypothetical protein
OsISM03301	LOC_Os01g24120	13595718	CATGTTTTCAGTAGGACGAGCA	CCCTTACATGGAGCACCTTT	59.7	990	57	hypothetical protein

ISM IDs	Rice gene locus IDs	Physical positions (bp) on chromosomes	Forward primers (5'-3')	Reverse primers (5'-3')	Melting tempearature (T˚C)	Expected product size (bp) in PCR	Expected product size (bp) in Q-PCR	Putative Functions
OsISM03302	LOC_Os01g24120	13596746	TTGAAAGGATTTGTGCTTGC	AGCATGATTGCTCATGGAAC	58.8	180	64	hypothetical protein
OsISM03303	LOC_Os01g24120	13596931	AAATGTAAAAGAGACAGAGTGGTTC	CGGTTTTTGAAACCCTGGTA	58.5	580	84	hypothetical protein
OsISM03304	LOC_Os01g24140	13611812	TTGTACGATGAATCAAAGGGC	AGGCCTCAGGAGCATTTTCT	60.2	116	55	transposon protein putative CACTA En/Spm sub-class
OsISM03305	LOC_Os01g24140	13612615	AAGGCCATCATCTTCGTGAC	TTGTCCCCTTTGATGGTTTC	59.9	324	92	transposon protein putative CACTA En/Spm sub-class
OsISM03306	LOC_Os01g24150	13616676	TGAAAAAGGCGACAAGGACT	CTTGACCGTGATGGGATCTT	59.9	401	86	hypothetical protein
OsISM03307	LOC_Os01g24170	13623474	ACCTGAGCCAGATTACCCCT	TTCTCCCAGGGATAGCAATG	60.0	122	87	hypothetical protein
OsISM03308	LOC_Os01g24180	13625556	TATGGCTAGGATGGGTTGGA	TACCCGCTGACATTCTTTCC	60.2	1396	91	retrotransposon protein putative unclassified
OsISM03309	LOC_Os01g24180	13628246	CTTTGGATGTTCCAGCACAG	GAATGGTTGGTGCTGAAGGT	59.6	825	86	retrotransposon protein putative unclassified
OsISM03310	LOC_Os01g24200	13634423	TGAGCCACGACTCCAAGTCT	CGATGATATATGGCATGGAGA	59.7	355	95	hypothetical protein
OsISM03311	LOC_Os01g24200	13634774	ATCGGGACAAGATGAACGTC	TGGATCCAAGAAGCTGATAGGT	60.0	428	96	hypothetical protein
OsISM03312	LOC_Os01g24200	13635287	AATACAAGAAGGGCTTGCACA	TCCAAGACTACCACGGTTCC	59.9	250	84	hypothetical protein
OsISM03313	LOC_Os01g24200	13635530	TCTTGGACCCTCTCGATTACA	TACCCGCAAAAGACAGTTCC	59.9	345	97	hypothetical protein
OsISM03314	LOC_Os01g24200	13635890	CCTCAGGGTTAATGGGAGGT	TGGACATTCGTGATGCTTGT	60.2	173	94	hypothetical protein
OsISM03315	LOC_Os01g24210	13643426	AGACGAGGTGGAGGAGGC	CGTAGCCAATGTTTAGAACTCCA	60.4	830	96	retrotransposon protein putative Ty1-copia subclass
OsISM03316	LOC_Os01g24210	13644276	TTGCCTGGAGACCGTATTTT	CGTCGAACTCTTTTGTTTCCA	59.9	286	53	retrotransposon protein putative Ty1-copia subclass
OsISM03317	LOC_Os01g24210	13644981	GCTTCCGGAGGACAGGATAC	TCAGGTAGTGGAGCAGAGCC	60.8	156	99	retrotransposon protein putative Ty1-copia subclass
OsISM03318	LOC_Os01g24220	13647984	AAACGGAACGAAAACAACGA	GATTCCGCCAGGAACCTT	60.3	243	97	retrotransposon protein putative Ty1-copia subclass
OsISM03319	LOC_Os01g24220	13648202	TTACATGAAGGTTCCTGGCG	GTTGTACCACATCCTGCTCG	60.1	121	86	retrotransposon protein putative Ty1-copia subclass

ISM IDs	Rice gene locus IDs	Physical positions (bp) on chromosomes	Forward primers (5'-3')	Reverse primers (5'-3')	Melting tempearature (T°C)	Expected product size (bp) in PCR	Expected product size (bp) in Q-PCR	Putative Functions
OsISM03320	LOC_Os01g24240	13660998	TTGAAGCGCTACGTGACAAC	TCTCACATGTTTGAAGCTCTTACC	59.9	373	81	conserved hypothetical protein
OsISM03321	LOC_Os01g24240	13661272	GGTGGTATTTTTGGGACATTC	GATCTTGCAACCTTGTTGTGA	58.5	228	99	conserved hypothetical protein
OsISM03322	LOC_Os01g24240	13661827	GCCAATGGTTGGGATGAATA	ACCAAGGCCCAGTAATGTTG	60.2	188	98	conserved hypothetical protein
OsISM03323	LOC_Os01g24280	13677531	CATGTCCCAAGCTCGCTC	GCCGTTCCGTTGGTTTTATT	60.8	264	92	retrotransposon protein putative unclassified
OsISM03324	LOC_Os01g24350	13716346	TACCAGCGAGGATGGTGG	TCAACTCGTGAGTCCAGACG	60.3	976	75	expressed protein
OsISM03325	LOC_Os01g24410	13761091	TGGAACAAAGAGGAGGAGGA	CCAAGGTGATCTTCGGTGTT	59.9	112	77	transposon protein putative CACTA En/Spm sub-class
OsISM03326	LOC_Os01g24410	13761681	ATATGCAAAAGTGCAGCCG	AGGAGGTCTCGGTGGTTTTT	59.9	181	86	transposon protein putative CACTA En/Spm sub-class
OsISM03327	LOC_Os01g24420	13764302	CTCTTCTGACGGCGAGAGAC	TTGCAGGATTTGGGTCCTTA	60.4	801	61	expressed protein
OsISM03328	LOC_Os01g24420	13765171	AGAAGCCACGACTCCAAGAA	CTTGCTGGCTTCCATGTACC	60.3	257	97	expressed protein
OsISM03329	LOC_Os01g24420	13765525	GTCGGGAAAAGACGGACATA	GGTTCACCGATCTTCTTAGCA	59.6	147	77	expressed protein
OsISM03330	LOC_Os01g24420	13765834	AGCCTTCTTGCACTTCCAAA	GCATGCGTAGTACCCACAGA	59.9	540	83	expressed protein
OsISM03331	LOC_Os01g24420	13766375	AATACCTCCGGGTTACTGGG	GAATCCTTCTCGACGCTCAA	60.3	161	72	expressed protein
OsISM03332	LOC_Os01g24420	13766629	AACATCCTTGATGAGGACACAA	GGCCTCTTTTCTTTCGTTCA	59.4	185	98	expressed protein
OsISM03333	LOC_Os01g24420	13766761	TTTTGTGTGTTTTCGTTTGC	TACCATCTCAAAACCTCGGC	58.7	2625	83	expressed protein
OsISM03334	LOC_Os01g24420	13769357	GGATTAATCGCCGAGGTTTT	GCACACCATGATCACCACC	60.6	531	81	expressed protein
OsISM03335	LOC_Os01g24470	13793835	TATGGAAGAGGGATTCGTGG	ACTCCCATTGCGTTTCCTC	60.0	208	98	PHS1 putative expressed
OsISM03336	LOC_Os01g24470	13794087	ATTTGCTGCCCAATCTTCAG	CAATCGCATCGCTATAAGGG	60.4	454	66	PHS1 putative expressed
OsISM03337	LOC_Os01g24470	13794689	CTACGAGGATGACACCAGCA	GCAATGAAAACAACTCCTCCA	60.0	463	64	PHS1 putative expressed

ISM IDs	Rice gene locus IDs	Physical positions (bp) on chromosomes	Forward primers (5'-3')	Reverse primers (5'-3')	Melting tempearature (T˚C)	Expected product size (bp) in PCR	Expected product size (bp) in Q-PCR	Putative Functions
OsISM03338	LOC_Os01g24470	13795256	ATGAACTTGCGAAATGGCTT	AGCGTTCTCAACTGCATCCT	59.9	179	99	PHS1 putative expressed
OsISM03339	LOC_Os01g24470	13795507	TTGAGCCGATGCTTATTCCT	TAGCAGAGGGGAGCCATGTA	60.3	133	49	PHS1 putative expressed
OsISM03340	LOC_Os01g24470	13796571	TGCGGCTGACAATGAAACTA	TGTCCTGAGCATGTCATTCC	60.0	501	96	PHS1 putative expressed
OsISM03341	LOC_Os01g24470	13797136	GCATTGCAGTTGATGCTTATG	GTGCTTGCAGCATCAGAAAT	59.2	164	83	PHS1 putative expressed
OsISM03342	LOC_Os01g24470	13797431	AATCTCAGCAGCCTGATCGT	TGTCTGCATTTTCATCATCG	59.1	160	64	PHS1 putative expressed
OsISM03343	LOC_Os01g24470	13797685	GAGCGCAACTGTTGTTCTTG	GTCGGTGCACCTTCTTTAGC	59.8	267	87	PHS1 putative expressed
OsISM03344	LOC_Os01g24500	13814001	ACAGGGTGGCCAAACTCTTC	TGTTGCTAGCAGCTCACCAT	60.5	400	96	hypothetical protein
OsISM03345	LOC_Os01g24500	13814353	GGACTGCATTTGTTCTGTTCC	TGGGCATACCATGACCACTA	59.7	618	98	hypothetical protein
OsISM03346	LOC_Os01g24560	13841772	GTCACCGGCGTCAAGGAC	CTGATCTTCGTCAGCCCCT	61.6	172	84	fruit bromelain precursor putative expressed
OsISM03347	LOC_Os01g24600	13858707	CGTCAAGTTCCAAGGCAAT	GGTCTTGATCTTGTGCAGGC	59.8	174	80	fruit bromelain precursor putative expressed
OsISM03348	LOC_Os01g24610	13860720	CCACAGACGACATATGAAGCA	TTGTATCTTGATGCCCCACA	59.8	149	99	transposon protein putative CACTA En/Spm sub-class
OsISM03349	LOC_Os01g24620	13864823	AAGGTGGTCATTCAACCGAC	GATCTTCGGCCTCGGACT	60.1	182	87	transposon protein putative unclassified
OsISM03350	LOC_Os01g24660	13879664	TGATGCATTCGTCTATCAGGA	CGTGGACTCAACCTCGTTTT	59.7	150	71	transposon protein putative CACTA En/Spm sub-class
OsISM03351	LOC_Os01g24660	13880194	GGTCGCACCACTAGCTGC	GTATCGATGGACACCTTGGC	60.5	259	82	transposon protein putative CACTA En/Spm sub-class
OsISM03352	LOC_Os01g24660	13881089	AAGGATTCCGAAGTTGGACA	GATTACCAAGGCGACGACTC	59.6	162	78	transposon protein putative CACTA En/Spm sub-class
OsISM03353	LOC_Os01g24660	13881342	GGCCCATCAAGACAAGGAGT	CGATGACTAGGAGAATCCAATG	60.1	150	69	transposon protein putative CACTA En/Spm sub-class
OsISM03354	LOC_Os01g24660	13881691	TGTGTGCCACAACATGTCAA	ACCCCATGAAAGTCCTCAAA	60.0	540	99	transposon protein putative CACTA En/Spm sub-class
OsISM03355	LOC_Os01g24680	13886545	CGTCAACTCGCTCTCCATC	GCTTTCACATCATTCCTCCG	60.1	1014	79	3-hydroxyacyl-CoA dehydrogenase putative expressed

ISM IDs	Rice gene locus IDs	Physical positions (bp) on chromosomes	Forward primers (5'-3')	Reverse primers (5'-3')	Melting tempearature (T˚C)	Expected product size (bp) in PCR	Expected product size (bp) in Q-PCR	Putative Functions
OsISM03356	LOC_Os01g24680	13887539	CGGAGGAATGATGTGAAAGC	GCCACCCTGAACACTACCAA	60.8	221	94	3-hydroxyacyl-CoA dehydrogenase putative expressed
OsISM03357	LOC_Os01g24680	13887692	TGACAGGGAAAGGAGGAAAA	TGTAACCAACCTTTGGCTGTT	59.6	194	94	3-hydroxyacyl-CoA dehydrogenase putative expressed
OsISM03358	LOC_Os01g24680	13887862	TTGAACAGCCAAAGGTTGGT	GACCATCAATTGCAGCCAC	60.3	186	91	3-hydroxyacyl-CoA dehydrogenase putative expressed
OsISM03359	LOC_Os01g24680	13888052	TCTTGGTGGAGGATTGGAAG	AGCTGTGGGAGTTGAAATGC	60.2	144	59	3-hydroxyacyl-CoA dehydrogenase putative expressed
OsISM03360	LOC_Os01g24680	13888180	TTCAACTCCCACAGCTCAAT	TTGTCAATCCAACAAGACGC	59.0	396	99	3-hydroxyacyl-CoA dehydrogenase putative expressed
OsISM03361	LOC_Os01g24680	13888537	TAGGAACACAACGACTCCCG	TCTGCACCCTTAATTGGCTT	60.4	229	83	3-hydroxyacyl-CoA dehydrogenase putative expressed
OsISM03362	LOC_Os01g24680	13888966	ATCTTGAGCATCCGCTTGTT	AAGACCCTGGAAAGCATTTG	59.5	195	99	3-hydroxyacyl-CoA dehydrogenase putative expressed
OsISM03363	LOC_Os01g24680	13889199	CTTTTCTCAACGGGCAACAT	GGCATCAATCCCAAGTCTGT	60.0	127	55	3-hydroxyacyl-CoA dehydrogenase putative expressed
OsISM03364	LOC_Os01g24680	13889439	TTCTAAATGCTGGAATTGACAGA	TTTCCCTTTCCTCACACGAC	59.5	169	60	3-hydroxyacyl-CoA dehydrogenase putative expressed
OsISM03365	LOC_Os01g24680	13889664	TGAAAGGTTCAAAGACGTGG	TGCTGTTTCAATTTCACATTTTC	59.0	152	64	3-hydroxyacyl-CoA dehydrogenase putative expressed
OsISM03366	LOC_Os01g24680	13889916	AAAGACCGTATTGTCGGTGC	CGACAACTTGTGGTGAGGTG	60.1	169	95	3-hydroxyacyl-CoA dehydrogenase putative expressed
OsISM03367	LOC_Os01g24680	13890230	CGATCGTGCATGTACCAAAT	ACCAAAACCAACGAGGTCTG	59.7	202	68	3-hydroxyacyl-CoA dehydrogenase putative expressed
OsISM03368	LOC_Os01g24680	13890461	TGAGAACTTCCCAGAGAGGGT	TGTAAAAGCCCTTCCGAGAG	59.8	203	93	3-hydroxyacyl-CoA dehydrogenase putative expressed
OsISM03369	LOC_Os01g24680	13890717	GAAAAATCTAGGAGTATGGCTGGA	CTGGGAAGAAGACCATCTCAA	59.6	538	89	3-hydroxyacyl-CoA dehydrogenase putative expressed
OsISM03370	LOC_Os01g24680	13891315	ACATTGCCTCAATCTTTGGC	AATGGAATCTGCCCAGAACA	60.3	157	69	3-hydroxyacyl-CoA dehydrogenase putative expressed
OsISM03371	LOC_Os01g24680	13891534	GCCTTGTTCCTACCTTGCAG	GCCTTGACTTGCTTAGCTGG	60.0	302	70	3-hydroxyacyl-CoA dehydrogenase putative expressed
OsISM03373	LOC_Os01g24690	13896743	TGACATCCAGGCAAAGAAAG	TAAGCCTTCTTCTTGCCGTC	59.2	147	58	60S ribosomal protein L23A putative expressed
OsISM03374	LOC_Os01g24780	13940546	ACTCCCACGACTTCATCCC	CGCCAAGCCAGTAAAGAAAC	59.9	246	85	cytochrome P450 putative expressed

ISM IDs	Rice gene locus IDs	Physical positions (bp) on chromosomes	Forward primers (5'-3')	Reverse primers (5'-3')	Melting tempearature (T˚C)	Expected product size (bp) in PCR	Expected product size (bp) in Q-PCR	Putative Functions
OsISM03375	LOC_Os01g24780	13940964	AGCGTTCAACCACGACAAG	TGTATCTGTGATTGCCAGCG	60.4	678	88	cytochrome P450 putative expressed
OsISM03376	LOC_Os01g24780	13941804	AGATGTGCCAGCTCTGGTTT	GGTCCTCGATTTCTTGACGA	60.0	393	86	cytochrome P450 putative expressed
OsISM03377	LOC_Os01g24780	13942494	GACATGGCTAACAGGCTGAA	TCTGAACCAGCAATCCTCCT	59.3	613	96	cytochrome P450 putative expressed
OsISM03378	LOC_Os01g24800	13964607	CAAATTTGGAAAAAGAGGTTTC	TTCTCACCACTTCTAGCACCAA	58.5	193	80	retrotransposon protein putative unclassified
OsISM03379	LOC_Os01g24800	13964903	CCTGTTGTTGCTTCTGTTGC	TCCACACGTTCTCCTTCCTT	59.6	533	71	retrotransposon protein putative unclassified
OsISM03380	LOC_Os01g24800	13965499	AGGCATCCAAGACTGAATCG	AAAATACGACCATCCTCCCC	60.1	1244	98	retrotransposon protein putative unclassified
OsISM03381	LOC_Os01g24800	13966767	CGATGATGATGATGAGGTCG	CCTTAGACCTTGTAATCCGCT	58.8	270	90	retrotransposon protein putative unclassified
OsISM03382	LOC_Os01g24800	13967734	CAGGGAATTTGAGATGAGCA	TGGCCTAGAAGCAGTTAGATACAA	59.1	265	98	retrotransposon protein putative unclassified
OsISM03383	LOC_Os01g24800	13967986	TGCTTCTAGGCCAGACATACA	TCACAAAAGAGAGGTACTTTCTCAAA	59.2	402	94	retrotransposon protein putative unclassified
OsISM03384	LOC_Os01g24800	13968454	TCGCTTTCACTTTTTGAGGG	CGGTCCATCACCACAAATAC	59.5	591	86	retrotransposon protein putative unclassified
OsISM03385	LOC_Os01g24860	13998220	AATTGGCTCATGAGGGATCA	TGTCATGTCCTCACTCCCAA	60.3	167	99	retrotransposon protein putative unclassified
OsISM03386	LOC_Os01g24860	13999068	CAAAAGCCCTCTCACACCTC	TTTCATTGACAACCCTAAGGC	59.5	300	71	retrotransposon protein putative unclassified
OsISM03387	LOC_Os01g24860	13999949	CGGCCAGAGGGTCTTTACTA	TGCAGCTACTGTCCACAACC	59.6	244	74	retrotransposon protein putative unclassified
OsISM03388	LOC_Os01g24860	14000906	GCAAAGCAAGCTCAAAACCT	CGTGCTTGATTCCTTCTTCA	59.3	118	86	retrotransposon protein putative unclassified
OsISM03389	LOC_Os01g24860	14001693	GGACCTGTGGCAGTGAAGAC	TATCCTTGGATGGTGCACTG	60.1	113	60	retrotransposon protein putative unclassified
OsISM03390	LOC_Os01g24860	14002018	GACTTTGGTGGAGAGACCCA	CCAACTCCGAGATAGGTCCA	60.1	275	96	retrotransposon protein putative unclassified
OsISM03391	LOC_Os01g24860	14002738	TAATGGCCACCTAGACCTCG	AATCGGAATAGCCCAAAACC	60.1	263	81	retrotransposon protein putative unclassified
OsISM03392	LOC_Os01g24870	14004053	CGATCGATTTGTGTTTCCCT	GCTCGAGTCAGCAGGAACAT	60.2	389	88	retrotransposon protein putative unclassified

ISM IDs	Rice gene locus IDs	Physical positions (bp) on chromosomes	Forward primers (5'-3')	Reverse primers (5'-3')	Melting tempearature (T˚C)	Expected product size (bp) in PCR	Expected product size (bp) in Q-PCR	Putative Functions
OsISM03393	LOC_Os01g24870	14005332	CAACAAGCCAGATTTCAGCA	CCTGAGAGCCTTCTTTATGGG	60.1	292	92	retrotransposon protein putative unclassified
OsISM03395	LOC_Os01g24880	14015700	TGATTGGGGTCATGATGAAG	ATAGGACACCATTGGAAGCG	59.6	532	91	zinc finger C3HC4 type domain containing protein expressed
OsISM03396	LOC_Os01g24880	14016212	CGCTTCCAATGGTGTCCTAT	TACAGGTTACGGGTGGTGCT	60.2	533	83	zinc finger C3HC4 type domain containing protein expressed
OsISM03397	LOC_Os01g24880	14016714	GAAGGAGAGTCAGCACCACC	CAGGTGCTGATACAGGCAAA	59.9	180	83	zinc finger C3HC4 type domain containing protein expressed
OsISM03398	LOC_Os01g24880	14017187	AGTGATAGCCAACAGCGAGG	TGAATTGTGGTGCCATAGGA	60.2	530	98	zinc finger C3HC4 type domain containing protein expressed
OsISM03399	LOC_Os01g24900	14025275	GGTAGCGGTCAGTGGCAG	AGGGATCAATGGTTGAGGGT	60.5	140	97	hypothetical protein
OsISM03400	LOC_Os01g24920	14030427	AATCGGTGGAGGTTTGGAC	TTGATGGATCAACATCCCAA	59.7	744	80	poly synthetase 2-B putative expressed
OsISM03401	LOC_Os01g24920	14031193	AGAAGATCTATCCATGGCGG	CAGCAGCAGCAGCATTAGAA	59.8	2804	85	poly synthetase 2-B putative expressed
OsISM03402	LOC_Os01g24920	14034013	TGGTGATCAGGACAAGACGA	GCCGTGACAATCTTCTCCTT	59.8	222	82	poly synthetase 2-B putative expressed
OsISM03403	LOC_Os01g24920	14034283	CAAAACGGCCTACCATGTCT	TAGTCTGGTTCATGGTGGCA	60.1	552	63	poly synthetase 2-B putative expressed
OsISM03404	LOC_Os01g24920	14034815	TGCCACCATGAACCAGACTA	ACCATGAAGTTTCCACCAGC	60.0	330	91	poly synthetase 2-B putative expressed
OsISM03405	LOC_Os01g24920	14035125	GCTGGTGGAAACTTCATGGT	AGATCCCTTGCGATAGGACC	60.2	159	79	poly synthetase 2-B putative expressed
OsISM03406	LOC_Os01g24920	14035303	ACACCTGGCTTGAAATGGAC	CGAGTTTCAAGTTTCGTTTCTTC	59.7	190	98	poly synthetase 2-B putative expressed
OsISM03407	LOC_Os01g24920	14035529	CATGATGAAGCAGCAAATGG	TGCTTAGCTTTCCCAGAGGA	60.2	478	66	poly synthetase 2-B putative expressed
OsISM03408	LOC_Os01g24920	14035987	TCCTCTGGGAAAGCTAAGCA	CTGTGTCCTGTCCGTGTCTG	60.2	364	98	poly synthetase 2-B putative expressed
OsISM03409	LOC_Os01g24920	14036331	CAGACACGGACAGGACACAG	CGAAGTCATGAGGGATCACA	60.0	570	67	poly synthetase 2-B putative expressed
OsISM03410	LOC_Os01g24920	14036881	TGTGATCCCTCATGACTTCG	GCTTTTAGCTTCTGAGGAGTGTC	59.2	191	70	poly synthetase 2-B putative expressed
OsISM03411	LOC_Os01g24920	14037049	GACACTCCTCAGAAGCTAAAAGC	TCGTCCTCCAGAAGCTTGAT	59.4	400	84	poly synthetase 2-B putative expressed

ISM IDs	Rice gene locus IDs	Physical positions (bp) on chromosomes	Forward primers (5'-3')	Reverse primers (5'-3')	Melting tempearature (T˚C)	Expected product size (bp) in PCR	Expected product size (bp) in Q-PCR	Putative Functions
OsISM03412	LOC_Os01g24940	14046978	CTCCGGCAACAAGCCAGT	CTCCTCCTCCTCCTCTTTCC	60.9	191	65	poly synthetase 2-A putative expressed
OsISM03413	LOC_Os01g24940	14047412	CCTTCTGATACGGATGGTGG	CACTCCTCAACTCTGTCGTCAC	60.1	183	81	poly synthetase 2-A putative expressed
OsISM03414	LOC_Os01g24940	14047647	CAGCACATCCCTGACCATATAA	AGTCTGGTTCATGGTGGCAT	60.1	426	97	poly synthetase 2-A putative expressed
OsISM03415	LOC_Os01g24940	14048054	TGCCACCATGAACCAGACTA	GAAGCTTCCTCCAGCATCAG	60.1	523	86	poly synthetase 2-A putative expressed
OsISM03416	LOC_Os01g24940	14048743	ACACCTGGCTTGAGATGGAC	TCAGTGCTGGGACTGACTTTC	60.3	143	68	poly synthetase 2-A putative expressed
OsISM03417	LOC_Os01g24940	14048952	CATGATGAAGCAGCAAATGG	GCTTTCCAAGAGGAAGTTTATCA	59.6	495	60	poly synthetase 2-A putative expressed
OsISM03418	LOC_Os01g24940	14049424	TGATAAACTTCCTCTTGGAAAGC	GCCCTCGAAATAACATTGGA	59.4	345	88	poly synthetase 2-A putative expressed
OsISM03419	LOC_Os01g24940	14049749	TCCAATGTTATTTCGAGGGC	CGAAGTCATGAGGGATCACA	59.8	571	80	poly synthetase 2-A putative expressed
OsISM03420	LOC_Os01g24940	14050300	TGTGATCCCTCATGACTTCG	TCCAGCTTAGCTTTTAGCTTCTG	59.6	299	79	poly synthetase 2-A putative expressed
OsISM03421	LOC_Os01g24940	14050576	CAGAAGCTAAAAGCTAAGCTGGA	TCAATCTCACCAAGGGCTTC	59.8	402	48	poly synthetase 2-A putative expressed
OsISM03422	LOC_Os01g24940	14050994	CTGGAGGATGATTCAACCGA	TTCAGAACCAACTTCCAAAGG	60.1	330	91	poly synthetase 2-A putative expressed
OsISM03423	LOC_Os01g24940	14051301	CACCTTTGGAAGTTGGTTCTG	TGCGTTTTTCCATGGGTATT	59.9	349	71	poly synthetase 2-A putative expressed
OsISM03424	LOC_Os01g24940	14051692	AAGGCATGGTGAAATGGAAC	ACCATGCCATAAAAGCATCC	59.8	156	68	poly synthetase 2-A putative expressed
OsISM03425	LOC_Os01g24940	14051827	AGGATGCTTTTATGGCATGG	TGCTTCAGGAGGAGCAATTC	60.2	510	82	poly synthetase 2-A putative expressed
OsISM03426	LOC_Os01g24940	14052429	GTCCGGTGTGCTGCTTCTAT	CTTTGGCAGGTTATTGGCAT	60.1	504	86	poly synthetase 2-A putative expressed
OsISM03427	LOC_Os01g24940	14052913	ATGCCAATAACCTGCCAAAG	CGTGATCTTCGACTCTGCTG	59.8	538	81	poly synthetase 2-A putative expressed
OsISM03428	LOC_Os01g24940	14053481	CCTAAGGCAGAGCCTTCAAA	AAACATGGAGAACGTACCGC	59.8	179	95	poly synthetase 2-A putative expressed
OsISM03429	LOC_Os01g24980	14078110	GAGATGGCACGTCTTGACAG	CTTCTGGGACGCTGTGATTT	59.8	166	71	naringenin 2-oxoglutarate 3-dioxygenase putative expressed

ISM IDs	Rice gene locus IDs	Physical positions (bp) on chromosomes	Forward primers (5'-3')	Reverse primers (5'-3')	Melting tempearature (T°C)	Expected product size (bp) in PCR	Expected product size (bp) in Q-PCR	Putative Functions
OsISM03430	LOC_Os01g24980	14078470	TTCTGGCCTAACCAACTTGC	ATGCTCTTCACAGCAGCAGA	60.1	148	66	naringenin 2-oxoglutarate 3-dioxygenase putative expressed
OsISM03431	LOC_Os01g24980	14078877	TTCATTGTCAACGTGGGAGA	AGCCGCTCTTTCTTCATGTC	59.8	216	96	naringenin 2-oxoglutarate 3-dioxygenase putative expressed
OsISM03432	LOC_Os01g24990	14085382	GCCTGCAGCTCAAGAAGAGA	CGTCCTGATTTTTCGCTTTC	60.4	399	59	retrotransposon protein putative unclassified
OsISM03433	LOC_Os01g25000	14096696	ATAGAGCAAAAATGCCGTGG	GGTCCAGTTTGTTGTCCAGG	60.2	306	96	retrotransposon protein putative unclassified
OsISM03434	LOC_Os01g25010	14101215	CCAGGACTGGGGCTTCTT	TCCACCACATCCTCTGGAAC	60.6	309	55	flavonol synthase/flavanone 3-hydroxylase putative expressed
OsISM03435	LOC_Os01g25010	14101695	CAGGAACCTCAGATTCTGGC	CGCGCAGTATCTGTCCAGT	59.9	562	65	flavonol synthase/flavanone 3-hydroxylase putative expressed
OsISM03436	LOC_Os01g25010	14102530	ATCGTCAACGTGGGAGACAT	CTCGACGCTCTTGTACCTCC	60.2	3999	58	flavonol synthase/flavanone 3-hydroxylase putative expressed
OsISM03437	LOC_Os01g25020	14112876	GTATGTACATGAGGCAGGCG	AACGACGGCATCTTCATCAC	60.1	203	83	hypothetical protein
OsISM03438	LOC_Os01g25040	14121252	CAAGGAAGGCGTACTCATCG	ATGGAGGGACTTGAGTGGTG	60.4	188	76	transposon protein putative CACTA En/Spm sub-class
OsISM03439	LOC_Os01g25040	14121574	CCGTCTCTGTTCCAGACGTT	CCAGAGATGATGTCCAGGGT	60.1	258	96	transposon protein putative CACTA En/Spm sub-class
OsISM03440	LOC_Os01g25040	14121834	ACTACACTAGGTGGCGTGGG	GCTTTGGGATTAAGAGCGTG	59.9	262	95	transposon protein putative CACTA En/Spm sub-class
OsISM03441	LOC_Os01g25040	14122203	ATGCACCCTCACTCTTGCTT	GCTTCCTTCAAGAACGTTGG	59.9	984	89	transposon protein putative CACTA En/Spm sub-class
OsISM03442	LOC_Os01g25040	14123193	ATTTGTTGACCTCGCAAAGG	TACCTGAGCCACCGTATTCC	60.0	281	96	transposon protein putative CACTA En/Spm sub-class
OsISM03443	LOC_Os01g25040	14124804	CGAAAGTAACCAGCCCTTCA	GGACTTCAACCCATCTTTCG	59.9	175	95	transposon protein putative CACTA En/Spm sub-class
OsISM03444	LOC_Os01g25040	14125178	CCATCTCTGGGTACAATGCTC	GGGGCCTAACTCTTCAGGAT	59.6	167	99	transposon protein putative CACTA En/Spm sub-class
OsISM03445	LOC_Os01g25060	14130035	CCGGATTATATTCCTATCGAGC	TTTTCTTTGGACAAGCTCCC	59.1	228	91	transposon protein putative CACTA En/Spm sub-class
OsISM03446	LOC_Os01g25060	14130310	CATTCTTGCTCTCGACGATG	ATGGTGCCCAATTCTTCAAA	59.9	315	79	transposon protein putative CACTA En/Spm sub-class
OsISM03447	LOC_Os01g25060	14130924	TGACCACGTCTCTCCAACAA	GTGGAAACCCTACGTTCCAA	60.1	157	85	transposon protein putative CACTA En/Spm sub-class

ISM IDs	Rice gene locus IDs	Physical positions (bp) on chromosomes	Forward primers (5'-3')	Reverse primers (5'-3')	Melting tempearature (T˚C)	Expected product size (bp) in PCR	Expected product size (bp) in Q-PCR	Putative Functions
OsISM03448	LOC_Os01g25060	14131471	TTGACATCCCAACACCAGAG	GTTCGTCAAGGGGTAGAGCA	59.9	321	88	transposon protein putative CACTA En/Spm sub-class
OsISM03449	LOC_Os01g25060	14132231	GGACCTCCACGCATTCTTTA	CCCATTGTGACTTGATGGAA	59.7	155	93	transposon protein putative CACTA En/Spm sub-class
OsISM03450	LOC_Os01g25060	14132595	AAACGAGTCATCATGGTCCC	CTTTACGTACCCGCCATGTT	59.8	377	70	transposon protein putative CACTA En/Spm sub-class
OsISM03451	LOC_Os01g25060	14132952	AACATGGCGGGTACGTAAAG	AACCGTATAGGTTCGTCAAGC	59.1	174	97	transposon protein putative CACTA En/Spm sub-class
OsISM03452	LOC_Os01g25060	14133139	GATGCTCAGGGTTAATGGGA	AAGCGTTTTCTGATCGAACC	59.6	177	89	transposon protein putative CACTA En/Spm sub-class
OsISM03453	LOC_Os01g25100	14166258	TCTGTGTTGGACCAGCTCTC	GACGACCTCGTACTCGACG	59.2	423	86	glutathione S-transferase putative expressed
OsISM03454	LOC_Os01g25100	14166683	GTTGAGCTTGACCAACGGC	AGAGTGTCAGGTCACCGTCC	61.0	231	96	glutathione S-transferase putative expressed
OsISM03455	LOC_Os01g25100	14166868	CCATTCGGTCAGATTCCAGT	TTGCCGAGAACGTACCTAGC	60.2	172	81	glutathione S-transferase putative expressed
OsISM03456	LOC_Os01g25110	14170258	GCGCCTCCTCAAGTTCTACA	GAGTTCCTGATGCTTCTCGG	60.2	234	50	clathrin adaptor complex small chain domain containing protein
OsISM03457	LOC_Os01g25110	14170472	CCGAGAAGCATCAGGAACTC	AATTGCTTACACTGTCCGGC	60.0	147	70	clathrin adaptor complex small chain domain containing protein
OsISM03458	LOC_Os01g25110	14170618	TTCGTCAAGGTTGATGCAAT	CAATGTGGCCAAATGTTTGTA	59.2	727	64	clathrin adaptor complex small chain domain containing protein
OsISM03459	LOC_Os01g25110	14171379	AACTTGCGGTGCTTGATCTT	CATTCTTGAAGCATCTGTCCA	59.4	2415	61	clathrin adaptor complex small chain domain containing protein
OsISM03460	LOC_Os01g25110	14173770	CATTGGACAGATGCTTCAAGA	TGAGGCTGGTTGTGTTTGAC	59.3	695	89	clathrin adaptor complex small chain domain containing protein
OsISM03461	LOC_Os01g25220	14247052	TCGCTGAGGAGGAAGAAAAG	AGTTGAGGATGGTGCGGAT	60.1	216	89	hypothetical protein
OsISM03462	LOC_Os01g25230	14249237	ATGAGCTCGCTGCTGGAC	GATGTCCCTGGCCTCTGTTA	60.2	1502	70	conserved hypothetical protein
OsISM03463	LOC_Os01g25240	14252058	CCTCCTCCTCAACACCCTC	ATCCAGGTAGGCCACCTTCT	59.8	600	50	expressed protein
OsISM03464	LOC_Os01g25240	14252683	CACCTCCTGGGTTTGTATCG	CTTACCTCTTTGGTGTCCGC	60.1	622	85	expressed protein
OsISM03465	LOC_Os01g25240	14253272	AAATGTTGTACCTGCGGACAC	TTCTTTGCAGAGCAATCACC	59.5	587	59	expressed protein

ISM IDs	Rice gene locus IDs	Physical positions (bp) on chromosomes	Forward primers (5'-3')	Reverse primers (5'-3')	Melting tempearature (T˚C)	Expected product size (bp) in PCR	Expected product size (bp) in Q-PCR	Putative Functions
OsISM03466	LOC_Os01g25240	14253922	TCGCTTTGAAGACACCGATA	GTCAGCATATGCAGGCCA	59.4	255	94	expressed protein
OsISM03467	LOC_Os01g25240	14254307	ATTCGTGCTGCACAACACTC	CACGAATGATCCTTGCCTCT	60.1	141	92	expressed protein
OsISM03468	LOC_Os01g25240	14254543	CCGTTTCAGCTTCCTTTGTC	TGCCAACAATAGTACCTTTCAAGA	60.0	723	98	expressed protein
OsISM03469	LOC_Os01g25240	14255301	TGTTCCACACCTTGTTGCAT	CCAAAAGCTGACGAGAAACA	59.5	226	98	expressed protein
OsISM03470	LOC_Os01g25240	14255519	GCTTTTGGAAAATGCCGATA	GCCTGTCCGCTATATCACTTG	59.9	814	99	expressed protein
OsISM03471	LOC_Os01g25250	14258337	GGTTTCTGCTCCTCCTCCC	AAGAGAACCTTGCCGTCGT	60.5	404	75	hypothetical protein
OsISM03472	LOC_Os01g25250	14258730	AAGGTTCTCTTCGTGCCGT	GCTCCAGCAAGTGCAAAAA	60.0	946	71	hypothetical protein
OsISM03473	LOC_Os01g25280	14280076	GAATGGATGGACTGGAGCAT	ACGCTCTGTAGGGCCAATCT	60.5	719	84	jacalin-like lectin domain containing protein expressed
OsISM03474	LOC_Os01g25290	14284965	GCCGCCCATTTGACCTACT	GACACTCAGAGGGAGGACCA	61.3	759	73	hypothetical protein
OsISM03475	LOC_Os01g25330	14307390	TAAGCATCTCCCATCCATCC	ATCAGCCACTGGGGTGATAC	59.8	416	98	SAC9 putative expressed
OsISM03476	LOC_Os01g25330	14307782	CCTAGTATCACCCCAGTGGC	TTCAACATAATCAGCACCTTGC	59.8	198	97	SAC9 putative expressed
OsISM03477	LOC_Os01g25330	14308079	AGAGTTGGCTCCAGCGTAGA	GATCAGCAAATTCGTCCCAT	60.0	621	85	SAC9 putative expressed
OsISM03478	LOC_Os01g25330	14308867	CAGGGCAAGAACACCAATTA	TCCACCCATGGAAGAGAAAC	59.3	183	64	SAC9 putative expressed
OsISM03479	LOC_Os01g25330	14309409	GCCAGGTTGAAGAATTTGACA	CATTTGCTCGAAGTTAAGACCC	60.1	573	96	SAC9 putative expressed
OsISM03480	LOC_Os01g25330	14310681	GGTGAAGAATCCCTTGCAGA	CCAAATGGTGCCAGTAATGA	59.8	357	99	SAC9 putative expressed
OsISM03481	LOC_Os01g25330	14311157	GATACTCAACATCTGACTGCCC	TCGTGTACATCCCATTTTCCT	59.0	1949	95	SAC9 putative expressed
OsISM03482	LOC_Os01g25330	14313440	GATAGATCGCAACCACTGGG	AGCCTTTCAGCTTCAACTGG	60.0	133	51	SAC9 putative expressed
OsISM03483	LOC_Os01g25330	14313748	CCAGCAATTCTTTATTTACAAGTCA	TCCTCAACAACCAGCTTTCC	59.5	415	60	SAC9 putative expressed

ISM IDs	Rice gene locus IDs	Physical positions (bp) on chromosomes	Forward primers (5'-3')	Reverse primers (5'-3')	Melting tempearature (T˚C)	Expected product size (bp) in PCR	Expected product size (bp) in Q-PCR	Putative Functions
OsISM03485	LOC_Os01g25402	14365716	CAGCCAGCCTATAGAGAGCG	CCGGACATTGTCTAGGAGGA	60.2	485	59	transposon protein putative CACTA En/Spm sub-class
OsISM03486	LOC_Os01g25402	14367390	CACGTAGCGATCACAAGGAA	GGGCTAGCTACCACTTTGGC	60.5	1725	86	transposon protein putative CACTA En/Spm sub-class
OsISM03487	LOC_Os01g25402	14369077	GTCGTCGTCGTCCCTCAG	GTACTGGCTACTTCCCGGTG	60.0	630	88	transposon protein putative CACTA En/Spm sub-class
OsISM03488	LOC_Os01g25402	14371022	CATGTGAGCTGCATATTCCC	AGTCCCTGAAATGTCCGTTG	59.5	176	81	transposon protein putative CACTA En/Spm sub-class
OsISM03489	LOC_Os01g25402	14371777	GACCGAACACTTAGGAAAGCC	AACTTGTATGCCACTTCCGC	60.1	182	87	transposon protein putative CACTA En/Spm sub-class
OsISM03490	LOC_Os01g25402	14372162	CCCTCGACGTCTCTCTTCTG	GAGGGTCGATGAACCCAGTA	60.0	181	91	transposon protein putative CACTA En/Spm sub-class
OsISM03491	LOC_Os01g25402	14372410	AACGCAGCAGCATTACAAGA	TTTTCTTTCCAAGTCCCGC	59.9	396	94	transposon protein putative CACTA En/Spm sub-class
OsISM03492	LOC_Os01g25402	14372787	GCGGGACTTGGAAAGAAAA	AGTTCCCTTGTCCTGCTTTG	59.8	163	66	transposon protein putative CACTA En/Spm sub-class
OsISM03493	LOC_Os01g25402	14372974	GAGTTTGCCCACTGCCTATC	TGTGTGGGAGTTTTTCCCTC	59.8	181	80	transposon protein putative CACTA En/Spm sub-class
OsISM03494	LOC_Os01g25450	14428944	AAGGTGCTTCCCACTATCCA	TGAATTGATGCCTTTGGTTG	59.5	203	90	AIG1 family protein expressed
OsISM03495	LOC_Os01g25450	14429854	TTTCTGTTGGAGAAAGTCTAG CA	CGCAGAGTGTTTAGGCCAAT	59.3	253	89	AIG1 family protein expressed
OsISM03496	LOC_Os01g25484	14446744	TCTCCAAGGAGGACATCGAC	CTTCAGCCGCATCATGAAC	60.3	3937	90	ferredoxin--nitrite reductase putative expressed
OsISM03497	LOC_Os01g25484	14450946	ACACCAACCTCCTCTCCTCC	ATCACGCACACGTTCCACT	60.3	193	89	ferredoxin--nitrite reductase putative expressed
OsISM03498	LOC_Os01g25500	14452813	ATGATCGGCTTCGTCTACCA	CCCTTCTTGAGATCGACGAC	60.2	198	87	sterol carrier protein-2 putative expressed
OsISM03499	LOC_Os01g25500	14452991	GTCGTCGATCTCAAGAAGGG	CTGGAGATGGCCAGGAAAT	59.9	172	99	sterol carrier protein-2 putative expressed
OsISM03500	LOC_Os01g25500	14453141	ACGATTTCCTGGCCATCTC	ATGTCCGGCGTGAACTTCT	60.3	198	79	sterol carrier protein-2 putative expressed
OsISM03501	LOC_Os01g25510	14459004	GTCCCTTTCCGTGCTCATC	CATTCTCCTTCCACGACTCC	60.1	202	99	alpha-amylase isozyme C putative expressed
OsISM03502	LOC_Os01g25510	14459263	ATCACCCACGTCTGGCTC	ACGCGTCCAGATCGTACAG	59.7	174	83	alpha-amylase isozyme C putative expressed

ISM IDs	Rice gene locus IDs	Physical positions (bp) on chromosomes	Forward primers (5'-3')	Reverse primers (5'-3')	Melting tempearature (T˚C)	Expected product size (bp) in PCR	Expected product size (bp) in Q-PCR	Putative Functions
OsISM03503	LOC_Os01g25510	14459439	AATCATCGAGGCGTTCCAT	AAGCCGATGTCCATCTTGAG	60.3	263	88	alpha-amylase isozyme C putative expressed
OsISM03504	LOC_Os01g25510	14460086	TTCCCTCCGACAAGGATATG	TCATCCTTGAGACCCCAGTC	60.0	181	91	alpha-amylase isozyme C putative expressed
OsISM03505	LOC_Os01g25520	14461460	CGTCCTCCTCGACACCAG	ACAATGACATGGGCTCCTTC	60.2	162	69	ferredoxin-nitrite reductase putative
OsISM03506	LOC_Os01g25520	14461685	TCTCCAAGGAGGACATCGAC	GAGCTCAAGGCCACTGAATC	60.1	631	92	ferredoxin-nitrite reductase putative
OsISM03507	LOC_Os01g25520	14462363	GGTGGTGGGATCCTGTGA	TGAGATTACCTCCCTAGTCCC	58.8	390	96	ferredoxin-nitrite reductase putative
OsISM03508	LOC_Os01g25520	14462723	TGATAGGTAGGGACTAGGGAGG	TTGAAGCCGGTGCTATCAG	59.3	1085	75	ferredoxin-nitrite reductase putative
OsISM03509	LOC_Os01g25580	14492643	AATGATCACGTGCGACGA	CTCCATGCGAAGACGTCAG	60.2	150	76	retrotransposon protein putative unclassified
OsISM03510	LOC_Os01g25580	14494108	GAAGAGGATCGGCCCCAC	AAGACGTAACCACCGTGACC	61.4	175	50	retrotransposon protein putative unclassified
OsISM03511	LOC_Os01g25610	14512324	GGAAGAGAGCGACGCAGAC	GAGATGCTTCTTCAATCCCC	60.0	670	94	40S ribosomal protein S4 putative expressed
OsISM03512	LOC_Os01g25610	14512989	ATCTCAAGAGGCTCAATGCG	TTCCCTGGACTTGTGAGGTC	60.3	206	96	40S ribosomal protein S4 putative expressed
OsISM03513	LOC_Os01g25610	14513283	TCTTGGTTGATGGGAAGGTC	TCACCAGTCTTTGGAATGGA	59.5	188	80	40S ribosomal protein S4 putative expressed
OsISM03514	LOC_Os01g25610	14513516	GTCCGTCAAGGATGAGGATG	ATGCCCTTCTGTCCAAACTG	60.3	168	70	40S ribosomal protein S4 putative expressed
OsISM03515	LOC_Os01g25650	14535233	CATTTGATCCTCCTGTATCTACTGC	TAATTTCGAGGAAACGGCAC	60.2	310	97	retrotransposon protein putative unclassified
OsISM03516	LOC_Os01g25650	14535549	TCCCGAATTTGCTAAGTTTAGTG	CAAGACCGGCCAAATCTCTA	59.9	368	97	retrotransposon protein putative unclassified
OsISM03517	LOC_Os01g25650	14536904	ATCATATGCCTCCAATGCAA	GCGTTTTCCTTCCCTCTTTT	59.3	137	81	retrotransposon protein putative unclassified
OsISM03518	LOC_Os01g25650	14537179	GGTCTGACCAGGCCCTCT	TCAGGCATCCACCTAGGATT	59.9	294	69	retrotransposon protein putative unclassified
OsISM03519	LOC_Os01g25650	14537609	GAGAAGGCAAAACCGATTGA	GAGAATTGAGCAACTTCGGC	60.1	152	81	retrotransposon protein putative unclassified
OsISM03520	LOC_Os01g25650	14538025	GGGAAACAAGACGCTACCAA	CCGATATTCTTGCACCCTTT	59.6	398	98	retrotransposon protein putative unclassified

ISM IDs	Rice gene locus IDs	Physical positions (bp) on chromosomes	Forward primers (5'-3')	Reverse primers (5'-3')	Melting tempearature (T˚C)	Expected product size (bp) in PCR	Expected product size (bp) in Q-PCR	Putative Functions
OsISM03521	LOC_Os01g25650	14538613	ATGTTGATTGCTTTGCATGG	TCTTCTCACCGGGATTATGC	59.8	233	76	retrotransposon protein putative unclassified
OsISM03522	LOC_Os01g25650	14539688	ATTACATATTTGAGCCGCCG	TTGTGCCAAGTCATTTGCTC	59.9	699	85	retrotransposon protein putative unclassified
OsISM03523	LOC_Os01g25650	14540539	GTCTGACCGGCTGTATGCTG	ATTGAACATTGCCGAACCAT	61.0	496	81	retrotransposon protein putative unclassified
OsISM03524	LOC_Os01g25780	14606964	CAGGAACTCAAAGTACCCGAA	CGACTATTTTGTGGTACAGATTAACAA	59.5	801	99	retrotransposon protein putative unclassified
OsISM03525	LOC_Os01g25780	14607782	CAGGCTCAATCATACACAAGTGA	CTACCACTTGCCACCCAGTT	60.1	184	71	retrotransposon protein putative unclassified
OsISM03526	LOC_Os01g25890	14689828	CGGATGAGGAAGAGGATGAA	CCGTGCTTCTTGAGGATGTT	60.2	1273	98	retrotransposon protein putative unclassified
OsISM03527	LOC_Os01g25910	14698949	AGTGATTCCGTCAGAGCTGG	GCCGGAAGTGTGTTTCCTT	60.3	225	98	retrotransposon protein putative unclassified
OsISM03528	LOC_Os01g25910	14699155	AAGGAAACACACTTCCGGC	ACGGAATACCGCTGTAGCAC	60.1	1086	84	retrotransposon protein putative unclassified
OsISM03529	LOC_Os01g25910	14700688	CCAAGCTTTCCTTCAGCAAC	TCGTCACAACAGGCAGGTAG	59.9	324	76	retrotransposon protein putative unclassified
OsISM03530	LOC_Os01g25930	14707473	GTATCATTGACGGGTACGCA	CACTTCTTCGACAGCCATCA	59.7	257	87	retrotransposon protein putative unclassified
OsISM03531	LOC_Os01g25950	14714743	GTCACGTCTTCCGTCCGTAT	GTCATCGGATCCATCATTCC	60.1	1395	75	retrotransposon protein putative Ty3-gypsy subclass
OsISM03532	LOC_Os01g25950	14716247	TACAGAATCGGAACAACCCC	CACCATTCTGAAGCTGGTCC	60.2	241	65	retrotransposon protein putative Ty3-gypsy subclass
OsISM03533	LOC_Os01g26010	14748415	GCTCATTTATTTTCCTCCACG	ATGCTTCTCTCACCACCACC	59.4	388	75	hypothetical protein
OsISM03534	LOC_Os01g26020	14751244	GGCTTCTCTCCTCTCGCC	TAGGCGTCTTCCTTCTCCAA	60.1	198	97	expressed protein
OsISM03535	LOC_Os01g26020	14751507	GGAACGAGACCTCTCCCCT	AAAGGTGGGCCATTTCTCTT	60.3	1527	73	expressed protein
OsISM03536	LOC_Os01g26060	14766436	GGAGGTGAAGCTGGTTGAGA	GCCTGGTGAATACGGTAAGC	60.0	1330	97	hypothetical protein
OsISM03537	LOC_Os01g26080	14776895	GGGGAGGAGAGGAAGCAA	CTGTGAGGCACCTACCATCC	60.4	1143	91	hypothetical protein
OsISM03538	LOC_Os01g26090	14785187	GAAGAGGCTGGATGAGATCG	GACCTGGGCCTCATACTTCA	60.0	237	77	hypothetical protein

ISM IDs	Rice gene locus IDs	Physical positions (bp) on chromosomes	Forward primers (5'-3')	Reverse primers (5'-3')	Melting tempearature (T˚C)	Expected product size (bp) in PCR	Expected product size (bp) in Q-PCR	Putative Functions
OsISM03539	LOC_Os01g26090	14785404	TGAAGTATGAGGCCCAGGTC	TTGGAAAAATTGTGCATCCC	60.4	278	84	hypothetical protein
OsISM03540	LOC_Os01g26110	14791248	GCAAGATCCCACACCATCTT	GGCATGTACGAAATGAGGGA	60.4	360	65	hypothetical protein
OsISM03541	LOC_Os01g26120	14792531	CAAGTGGACCTACTCCTCCG	GCTGAACGCGAACACCAT	60.3	3087	65	membrane associated DUF588 domain containing protein putative expressed
OsISM03543	LOC_Os01g26160	14808987	GATGGACTGGCGCTCCTC	AAATGATGTGTTCGCCAATG	60.7	809	98	exportin 1 putative expressed
OsISM03544	LOC_Os01g26160	14809816	GTCTTCAGAGGTCCGACTGC	CCTCCCATCTCAATCTCACC	59.7	961	63	exportin 1 putative expressed
OsISM03545	LOC_Os01g26160	14810860	GCTTTGAAGAGTCAAACGGC	ACAAAGCAACTCCCTCCCTT	60.1	169	62	exportin 1 putative expressed
OsISM03546	LOC_Os01g26160	14811055	TCGTTGTCAAACAATGGTCC	AAGCCACCTCAGAATCATGG	59.7	151	58	exportin 1 putative expressed
OsISM03547	LOC_Os01g26160	14811190	GATTCTGAGGTGGCTTCCAG	AAGACCACGTAACAATGCCC	59.8	204	83	exportin 1 putative expressed
OsISM03548	LOC_Os01g26160	14811397	GAGTCGTTGCCACAAATCCT	GCCAACTCCATTTGCTGATT	60.1	187	96	exportin 1 putative expressed
OsISM03549	LOC_Os01g26160	14811632	ATATGCTGAATGGGCTCCTG	CACAAGCATGGAGACGAAAA	59.9	179	99	exportin 1 putative expressed
OsISM03550	LOC_Os01g26160	14811791	TTTTCGTCTCCATGCTTGTG	TACTCCACAATCGCGACATC	59.8	174	76	exportin 1 putative expressed
OsISM03551	LOC_Os01g26160	14812142	AGCAAGGACTTTGCACTTCC	AAGATTGGAAATGAAGCGTGA	59.6	451	75	exportin 1 putative expressed
OsISM03552	LOC_Os01g26160	14812572	TCACGCTTCATTTCCAATCTT	CAGCAGGGGTGTCACTAGAA	59.3	181	97	exportin 1 putative expressed
OsISM03553	LOC_Os01g26160	14812939	ACTTCTGCAACTACCGTGCC	GGCCTTTGAGATGCAATGAC	60.5	1049	59	exportin 1 putative expressed
OsISM03554	LOC_Os01g26160	14814034	GGAGATGCTAATGAAGCAACG	CTAACAACTGCTTCCAGCCC	59.9	210	81	exportin 1 putative expressed
OsISM03555	LOC_Os01g26160	14814279	TTGACCAGGACACAAAATTCC	CAGCAAGATTTGGTTGTGACC	60.2	182	91	exportin 1 putative expressed
OsISM03556	LOC_Os01g26160	14814516	TGCAGTTGCCTGTATTGTGA	GCAGTCTAGCTTGACGGAAA	58.6	356	96	exportin 1 putative expressed
OsISM03557	LOC_Os01g26160	14814906	TGCTGATAAAGCACTTCTGCC	TAAGCGACCCTCTCCTTGAA	60.2	2975	74	exportin 1 putative expressed

ISM IDs	Rice gene locus IDs	Physical positions (bp) on chromosomes	Forward primers (5'-3')	Reverse primers (5'-3')	Melting tempearature (T˚C)	Expected product size (bp) in PCR	Expected product size (bp) in Q-PCR	Putative Functions
OsISM03558	LOC_Os01g26160	14817907	TGAAGCGTTTCTTGTCATGG	TGCGTCCACATTTTGTTGAT	59.9	203	94	exportin 1 putative expressed
OsISM03559	LOC_Os01g26160	14818322	GGATTCTGCCCCCTCTGT	TTTTTGCCGCTTTGACTTCT	60.0	226	97	exportin 1 putative expressed
OsISM03560	LOC_Os01g26160	14818670	ATAAGGAACTGGTTACGCGG	CCCTCTATGCATCGGAAAAA	59.6	331	93	exportin 1 putative expressed
OsISM03561	LOC_Os01g26160	14819379	TGCCAACCGGATTAATTCTT	CAGGAACAGCAGTGGAGACA	59.7	173	96	exportin 1 putative expressed
OsISM03562	LOC_Os01g26160	14819785	TCAGATAGAGATCCCGCACC	ATGTGTGGGAGGGACAGAAG	60.1	322	48	exportin 1 putative expressed
OsISM03563	LOC_Os01g26160	14820191	ACTTTTGCTTGCTTCAGGGA	CAGCAGAGCTCCGGTTTC	59.8	507	96	exportin 1 putative expressed
OsISM03564	LOC_Os01g26174	14824913	TGCACTCTTGGGTATCATGG	CATGGGCTTTGATTTTGCTC	60.1	431	81	OsWAK5 - OsWAK receptor-like protein kinase expressed
OsISM03565	LOC_Os01g26174	14825400	TGGGAAGAAAGTTGGCAAAG	ACAATGGAGCCAATGCTGA	60.2	680	80	OsWAK5 - OsWAK receptor-like protein kinase expressed
OsISM03567	LOC_Os01g26224	14860071	TGGCCATATTACATTTGGAGC	CGGTCAGCGTACATCCATT	59.7	430	85	transposon protein putative CACTA En/Spm sub-class
OsISM03568	LOC_Os01g26224	14861100	TCTGCCCTCTAGGACTGGAA	CTCTTCTTCGTTAGCTGCCC	59.6	172	95	transposon protein putative CACTA En/Spm sub-class
OsISM03569	LOC_Os01g26224	14861495	GGCATGAATCCTTTTGGAGA	TGTCGTTACCAGGTTGCTTG	59.9	150	53	transposon protein putative CACTA En/Spm sub-class
OsISM03570	LOC_Os01g26224	14861961	GGACAACCGTACGAAGCCTA	GACGTCCAAGAATTCCCAAT	59.5	183	92	transposon protein putative CACTA En/Spm sub-class
OsISM03571	LOC_Os01g26224	14863045	CTCTAGTTCGCGCCAGAAAC	TGCCAGTTGTACCATCGTGT	60.1	279	97	transposon protein putative CACTA En/Spm sub-class
OsISM03572	LOC_Os01g26224	14863712	CACCCTTTACTCACGTAGCGA	AGCTACCACTTCGGCTTGAG	60.0	1853	92	transposon protein putative CACTA En/Spm sub-class
OsISM03573	LOC_Os01g26224	14865532	GTCGTCGTCGTCCCTCAA	GTTCCAGAACTGGCTGCTTC	60.4	526	94	transposon protein putative CACTA En/Spm sub-class
OsISM03574	LOC_Os01g26224	14866074	AGGAACCAAACCAGCACTTG	CCGGTACAGATCTCCCTTGA	60.1	449	96	transposon protein putative CACTA En/Spm sub-class
OsISM03575	LOC_Os01g26224	14867114	CTCAGCATGCAAGAGGAGGT	AGTCCCTGAAATGTCCGTTG	60.3	393	70	transposon protein putative CACTA En/Spm sub-class
OsISM03576	LOC_Os01g26224	14868485	GCCCTCGACGTCTCTCTTTT	GAGGGTCGATGAACCCAGTA	60.4	182	92	transposon protein putative CACTA En/Spm sub-class

ISM IDs	Rice gene locus IDs	Physical positions (bp) on chromosomes	Forward primers (5'-3')	Reverse primers (5'-3')	Melting tempearature (T˚C)	Expected product size (bp) in PCR	Expected product size (bp) in Q-PCR	Putative Functions
OsISM03577	LOC_Os01g26224	14868740	GCAGCATTTCAAGACGTTCA	TGGAAGACCTTGTCAAAAACC	59.5	230	97	transposon protein putative CACTA En/Spm sub-class
OsISM03578	LOC_Os01g26224	14868949	GGTTTTTGACAAGGTCTTCCA	TCCGTCCAAGTTTTGCTTTC	59.6	192	90	transposon protein putative CACTA En/Spm sub-class
OsISM03579	LOC_Os01g26250	14880963	TCCTCGATTTCATCTCCAGC	GGCTTGAGGGGACTCCAT	60.2	358	89	conserved hypothetical protein
OsISM03582	LOC_Os01g26280	14895659	ATGGAGATGAGGGGAGGAAG	ACTTGGATTCTGCTCGCATT	60.1	489	58	OsWAK8 - OsWAK receptor-like protein kinase expressed
OsISM03583	LOC_Os01g26280	14896251	GCAACCCAGACATCAACCTT	CTTCGCTCAAACATCAAACG	59.7	540	92	OsWAK8 - OsWAK receptor-like protein kinase expressed
OsISM03584	LOC_Os01g26290	14900275	CTCTTATCCCCAAACCAGCA	GCTGACATATGCATTATCAAACA A	59.6	156	83	hypothetical protein
OsISM03585	LOC_Os01g26290	14900412	TGATAATGCATATGTCAGCAC G	CTGCTCCACCACCACCAT	60.1	1020	82	hypothetical protein
OsISM03586	LOC_Os01g26290	14901445	TTTCTCAGCGAACTCTGCAA	CCAGTCCCAGGACACCATAC	60.1	642	74	hypothetical protein
OsISM03587	LOC_Os01g26320	14923986	AGATGCAAGAGCTGTCCGAT	TGATTGGGTGCAGCATGTAG	60.3	897	85	ZmEBE-1 protein putative expressed
OsISM03588	LOC_Os01g26320	14924946	CGACATTAATTCAGCGAAGG	CGCACGTAGCCCGTAGTAAT	59.3	383	80	ZmEBE-1 protein putative expressed
OsISM03589	LOC_Os01g26320	14925391	CAGTCATTTCGGTGACGATG	GAGGACGAGAGTCGCCATAG	60.0	235	93	ZmEBE-1 protein putative expressed
OsISM03590	LOC_Os01g26320	14925611	GCGACTCTCGTCCTCACTTT	CGTTTGCTCGTTCAAAACCT	59.9	215	97	ZmEBE-1 protein putative expressed
OsISM03591	LOC_Os01g26320	14925840	CCAGGTGCTTCTATTGACCC	CACCAATCTCCACTTGTCCC	60.0	724	91	ZmEBE-1 protein putative expressed
OsISM03592	LOC_Os01g26340	14941589	CACATCATTTAACTTGGTTTTC G	TCTGAAGCGTTGAACCATTG	59.2	279	54	dirigent putative
OsISM03593	LOC_Os01g26340	14942006	TCCATGCATACTACAGCCCA	AAACTTGCGATTTCGCCTAA	60.0	326	93	dirigent putative
OsISM03594	LOC_Os01g26390	14964418	ATCCCGGAACAGGCACAG	GCAACAATAGCAACGATCCA	60.9	153	63	TKL IRAK DUF26-lh.1 - DUF26 kinases have homology to DUF26 containing loci expressed
OsISM03595	LOC_Os01g26390	14964600	TGCTTTCCATTGTATGGATCA	TGCGTCTTCTTCCAGCCTAT	59.5	207	90	TKL IRAK DUF26-lh.1 - DUF26 kinases have homology to DUF26 containing loci expressed
OsISM03596	LOC_Os01g26390	14964877	CAGACAACTTCGCAGCAGAG	TTAACTGCAACTTCCACCCC	59.9	697	92	TKL IRAK DUF26-lh.1 - DUF26 kinases have homology to DUF26 containing loci expressed

ISM IDs	Rice gene locus IDs	Physical positions (bp) on chromosomes	Forward primers (5'-3')	Reverse primers (5'-3')	Melting tempearature (T˚C)	Expected product size (bp) in PCR	Expected product size (bp) in Q-PCR	Putative Functions
OsISM03597	LOC_Os01g26390	14965709	TGAATATTTGCCAAACAAAAGC	CGCAACGTTTATTCCAGTCA	59.4	190	81	TKL IRAK DUF26-lh.1 - DUF26 kinases have homology to DUF26 containing loci expressed
OsISM03598	LOC_Os01g26390	14966068	AAACACAAAGAGGGTGGTGG	CAGAGGCATACTCTGGTGACA	59.4	164	54	TKL IRAK DUF26-lh.1 - DUF26 kinases have homology to DUF26 containing loci expressed
OsISM03599	LOC_Os01g26390	14966312	GGTTTCCACCAATACGGAGA	TCAAGCCATCTCCCTTCTTC	59.6	189	75	TKL IRAK DUF26-lh.1 - DUF26 kinases have homology to DUF26 containing loci expressed
OsISM03600	LOC_Os01g26400	14982503	GTGATGTGGGCGGTGATG	CGCCGTTTCCGTTACTCC	61.8	1233	91	retrotransposon protein putative Ty3-gypsy subclass
OsISM03601	LOC_Os01g26410	14986584	CCCAGCCCAATTATTCAAAA	GCACGAGTCGCTGTAGACAA	60.0	565	84	hypothetical protein
OsISM03605	LOC_Os01g26980	15027428	TGGTTCAAAAGAAGAAGGCAA	ATGTTCTTTGCTACGGACGG	60.0	305	89	transposon protein putative unclassified
OsISM03606	LOC_Os01g26980	15027667	TGCAGTAGTAGTATTGTTTCGGATG	CGCCTACTCCCTTTTAACCA	59.4	296	97	transposon protein putative unclassified
OsISM03607	LOC_Os01g26980	15027934	CGAGCAAGCTGGTTAAAAGG	CCAGAATTTCACCTTCACCG	60.2	541	89	transposon protein putative unclassified
OsISM03608	LOC_Os01g26980	15028859	GGGATGTTGGTCCTGACATT	CTCTTCAGCAGCGAGGTTTT	59.7	170	93	transposon protein putative unclassified
OsISM03609	LOC_Os01g26980	15029449	GAAGGTGATGACCTGCCTGT	GGAGGACGAATTGACTCCC	59.8	227	70	transposon protein putative unclassified
OsISM03610	LOC_Os01g26980	15029697	AGCATTCTTTGCATTCGGAG	CATCCTCCTGGAGCTCTTTG	60.1	301	80	transposon protein putative unclassified
OsISM03611	LOC_Os01g26980	15031106	CGTGGAACCTAGGCAACAAC	GGGTAAGGCCACTCCTCTTC	60.3	468	73	transposon protein putative unclassified
OsISM03612	LOC_Os01g26980	15031645	TAGCTGTCCTGATACCGCGT	GTCGTAATGCATCCTTGCCT	60.5	202	90	transposon protein putative unclassified
OsISM03613	LOC_Os01g26980	15032160	AAAAAGAAACTCATGGCCCC	GTACTCCTGGACATCGTCGG	60.4	152	78	transposon protein putative unclassified
OsISM03614	LOC_Os01g26980	15032639	ACTGACGCAGTTGATGATCG	CACATTGTGCACCTGGATTC	59.9	168	85	transposon protein putative unclassified
OsISM03615	LOC_Os01g26980	15032948	CGAAAAATGCTGGTGTACGA	TATCCATGGGGTCATCCTTG	60.1	277	97	transposon protein putative unclassified
OsISM03616	LOC_Os01g26980	15033391	ATATGCAGCTAGCACCCCC	TCCCATAATGTTTTCGCCTC	60.0	169	77	transposon protein putative unclassified
OsISM03617	LOC_Os01g26980	15033875	CCTAGGAGGCTTTGGTTCAA	TGGGCGTAGAGCTATATGGG	59.7	135	63	transposon protein putative unclassified

ISM IDs	Rice gene locus IDs	Physical positions (bp) on chromosomes	Forward primers (5'-3')	Reverse primers (5'-3')	Melting tempearature (T˚C)	Expected product size (bp) in PCR	Expected product size (bp) in Q-PCR	Putative Functions
OsISM03618	LOC_Os01g26980	15033996	TAGCTCTACGCCCATCACCT	TAGCAGGAGGCATATGAGGC	60.1	182	96	transposon protein putative unclassified
OsISM03619	LOC_Os01g26980	15034228	CAAGGAGGATTTGCACCATT	AAATCTGCAGACATTTGGGG	59.9	151	69	transposon protein putative unclassified
OsISM03620	LOC_Os01g26980	15034384	GTCAAGGAGGGTTTACGTCG	GGGTTGGTGCCATATGTAGG	59.8	178	74	transposon protein putative unclassified
OsISM03621	LOC_Os01g26980	15034542	CCTACATATGGCACCAACCC	CCTGACATTAGTTCAGGGCG	60.4	382	75	transposon protein putative unclassified
OsISM03622	LOC_Os01g26990	15037984	GCCGGTAGATCTGGGAGAC	CCTCCACGGAGCTGAGATT	60.0	260	96	retrotransposon protein putative unclassified
OsISM03623	LOC_Os01g26990	15038656	GTCGTTTAGCCGACCTCCTC	ACTTGGAATGGCAGGAAGG	60.6	144	57	retrotransposon protein putative unclassified
OsISM03624	LOC_Os01g27020	15058880	CGACACCTTCGAGAAGCTCA	ACACGTTGCTATAGGTGCCC	60.8	178	51	transposon protein putative unclassified expressed
OsISM03625	LOC_Os01g27020	15059289	CGGACGTCAAGTTTACTCTGC	GCTCAAGCCCTGACAATAGC	60.0	152	64	transposon protein putative unclassified expressed
OsISM03626	LOC_Os01g27020	15059675	CAGAGCTCTTGCATGGGAA	GGGAGCCACATAGCTTGAAA	60.1	2471	82	transposon protein putative unclassified expressed
OsISM03627	LOC_Os01g27020	15062299	AGCGCCAAACTGCTACATCT	CATGCATAAGGCTCAGTTGC	59.7	918	59	transposon protein putative unclassified expressed
OsISM03628	LOC_Os01g27020	15063232	GCCGACATATCCACCAAGTAA	TTTTACCCCCTCTCCTTTGG	60.1	1774	98	transposon protein putative unclassified expressed
OsISM03629	LOC_Os01g27020	15065060	GCAGAGCTTCAAGCAAACCT	TTTTATTTCCACACCCACAGAA	59.6	634	91	transposon protein putative unclassified expressed
OsISM03630	LOC_Os01g27020	15065574	ATCAGTCGCGCTCCATAATC	ACTTTTGCTCTTGGCATTGG	60.2	338	94	transposon protein putative unclassified expressed
OsISM03631	LOC_Os01g27030	15069387	GGTGCATGGGAAAGCAAG	GCTAGTTCCCGGAGTTGACA	60.2	183	77	hypothetical protein
OsISM03632	LOC_Os01g27050	15085207	CGTCGATGTCCAAGATGCT	CCTTCCCCTCCGTAGATGAC	60.3	139	91	flavin-containing monooxygenase family protein putative expressed
OsISM03633	LOC_Os01g27060	15093077	AACACAATATTTGGTGCGGC	ATCGCCATATTCACACCCAC	60.7	209	97	retrotransposon putative centromere-specific
OsISM03634	LOC_Os01g27060	15093295	TGCGTTGTTTGAGAGTGAGG	TTAGAGGCCTAACTCCGAGG	59.3	724	87	retrotransposon putative centromere-specific
OsISM03635	LOC_Os01g27060	15093997	TACCTCGGAGTTAGGCCTCT	TCATCGTCATGATATAGAATAATTGC	58.3	1237	84	retrotransposon putative centromere-specific

ISM IDs	Rice gene locus IDs	Physical positions (bp) on chromosomes	Forward primers (5'-3')	Reverse primers (5'-3')	Melting tempearature (T˚C)	Expected product size (bp) in PCR	Expected product size (bp) in Q-PCR	Putative Functions
OsISM03636	LOC_Os01g27110	15115517	CGCCTGCATGGAATTATGTT	CGCTTCTTGTTGAGCAAGGT	60.7	226	95	hypothetical protein
OsISM03637	LOC_Os01g27160	15145242	AGCTCAAGTGCATCCTCGAT	GCGTGCACATGTTGTAAACC	60.0	221	91	cullin putative expressed
OsISM03638	LOC_Os01g27160	15145481	ACTCGCAGGTGCTCTACGAT	TGCTTCTCATTCAAGGAGGG	60.2	1113	83	cullin putative expressed
OsISM03639	LOC_Os01g27160	15146718	CTCAAAGATCTGGGCTGGAG	GCTCACCATGCCAATCATAA	59.7	208	88	cullin putative expressed
OsISM03640	LOC_Os01g27160	15146881	AAGCTCAAAAGTACTGTGGCA A	TTGCCTTCCCTCTCATCATC	59.6	143	60	cullin putative expressed
OsISM03641	LOC_Os01g27160	15147171	TCTTGAGAATTCTTGCCCTGA	TAATTTGCAACCCGGTCTTT	59.7	152	76	cullin putative expressed
OsISM03642	LOC_Os01g27160	15147303	AAAGACCGGGTTGCAAATTA	TCAGAATTTCTTCTGCACGC	59.3	214	98	cullin putative expressed
OsISM03643	LOC_Os01g27160	15147524	GAACTCCGGATGCAAAGTGT	GAAGCAGCCCGTCAGTTATC	60.0	196	93	cullin putative expressed
OsISM03644	LOC_Os01g27160	15147700	GATAACTGACGGGCTGCTTC	GCAGCATCTGTTGCATGTTT	59.9	172	91	cullin putative expressed
OsISM03645	LOC_Os01g27160	15147821	GCATGTCATTGCTGAGGGTA	ACCACCACCCCCTTCTTCT	60.2	991	85	cullin putative expressed
OsISM03646	LOC_Os01g27160	15148789	GATGAGAAGAAGGGGGTGGT	TGGAGCTCTATGACACTCCTCA	60.2	149	66	cullin putative expressed
OsISM03647	LOC_Os01g27160	15148959	CACCAACTGTTTCCAGAGCA	CAGCACTACTGCAACCAACAA	59.9	200	96	cullin putative expressed
OsISM03648	LOC_Os01g27160	15149191	AAAAGAGGTGGAAGTGAAAA GC	AAAGAGGTCCTTGTCGCTGA	59.5	177	97	cullin putative expressed
OsISM03649	LOC_Os01g27160	15149346	TCTCAGCGACAAGGACCTCT	CAAACAGCAATCTCCTTCCA	59.5	171	63	cullin putative expressed
OsISM03650	LOC_Os01g27160	15149713	GACAACAGGATACTGGCCAAC	CTTGAAAACCTCCACGCATT	59.8	295	80	cullin putative expressed
OsISM03651	LOC_Os01g27160	15150066	ACTGCGTCGTCATTGGAAAT	GCTTCATTGAATAGCAGCAACA	60.5	191	89	cullin putative expressed
OsISM03652	LOC_Os01g27160	15150421	TTACCGATAGGATGCGAAGG	ATCAATTGCGAACCTCCTGT	59.8	177	99	cullin putative expressed
OsISM03653	LOC_Os01g27160	15150644	AGCAACTTGTAGCGGAATGC	CCCTGCTGATAAGATCCTCG	60.1	268	94	cullin putative expressed

ISM IDs	Rice gene locus IDs	Physical positions (bp) on chromosomes	Forward primers (5'-3')	Reverse primers (5'-3')	Melting tempearature (T˚C)	Expected product size (bp) in PCR	Expected product size (bp) in Q-PCR	Putative Functions
OsISM03654	LOC_Os01g27200	15167147	GGGGTAAGGAAGGGAGAGAA	TGGCACTCCACTATCGACG	60.2	1002	99	retrotransposon protein putative unclassified
OsISM03655	LOC_Os01g27200	15168462	TTTTCTGCGAAACAGGCTCT	TACAGAACCGGATCGAAAGG	60.1	507	57	retrotransposon protein putative unclassified
OsISM03656	LOC_Os01g27270	15219524	TGGAAGATGAAGATGAGGGC	ACGGACATCGTCTTACCACC	60.0	1068	94	retrotransposon protein putative unclassified
OsISM03657	LOC_Os01g27280	15222151	GGAGAAACATCGGGAAACCT	TTAGCAGGGAATTGAATGGG	60.1	922	93	retrotransposon protein putative unclassified
OsISM03658	LOC_Os01g27280	15223104	ATTGCGCAGGCAATTGAA	ACGCACAGCAGTTTGCTTTT	61.2	639	94	retrotransposon protein putative unclassified
OsISM03659	LOC_Os01g27280	15224498	CCAGTTTGAGGGTTTTCGAC	AGGACAGGATCAGGGATAGGA	59.7	553	97	retrotransposon protein putative unclassified
OsISM03660	LOC_Os01g27280	15225138	GTCAGTGGTGGCTGGTTCTC	CACTGTCTTCAACCCCAACC	60.6	326	75	retrotransposon protein putative unclassified
OsISM03661	LOC_Os01g27280	15225416	TTGCTCTTCTAGCAGCAGTCC	CTTTTCTTCAAGGAAGGCCG	60.6	217	81	retrotransposon protein putative unclassified
OsISM03662	LOC_Os01g27280	15225596	AGAATTGGTCCTCTTGTCGG	TGTACCCCTCTGTGAGCTTG	59.0	123	87	retrotransposon protein putative unclassified
OsISM03663	LOC_Os01g27280	15225733	AATCTGGGGATTGGAAAACC	CAGGAATCAAAAGACCATCCA	60.0	411	89	retrotransposon protein putative unclassified
OsISM03664	LOC_Os01g27280	15226170	TTTTTAATCCTTCTCGTCCCTG	CCACAAACAAACCACCAGAA	59.5	267	59	retrotransposon protein putative unclassified
OsISM03665	LOC_Os01g27280	15226583	GTTCACTGGATGAATGGGCT	AAGGACTGCAGGGTGCTCTA	60.0	1418	97	retrotransposon protein putative unclassified
OsISM03666	LOC_Os01g27280	15228480	AGGGCTCCCTCTCAGTTGTT	CCACCGAAAATGCCTTCTTA	60.2	226	83	retrotransposon protein putative unclassified
OsISM03667	LOC_Os01g27280	15228765	GCCCAAGAAGAAAGGTGGTT	TGAACAATGTCCTTCCACCA	60.2	198	91	retrotransposon protein putative unclassified
OsISM03668	LOC_Os01g27380	15288271	GTACGAGCTGGTCGGCATAC	AAAGCTGGAACTTGACCGAA	60.3	881	88	glutathione S-transferase putative expressed
OsISM03669	LOC_Os01g27380	15289132	TTCGGTCAAGTTCCAGCTTT	ATTGCAGACTGCGAGAGGTT	59.9	794	81	glutathione S-transferase putative expressed
OsISM03670	LOC_Os01g27390	15290838	GCCCGCCTCATATCTCCC	CGATGCTATCTCACCGAACA	61.3	878	84	glutathione S-transferase putative expressed
OsISM03671	LOC_Os01g27390	15291678	AGGATGGCGACCTGACTCT	TCAGATCCATGTTTGCGAAG	59.8	1100	68	glutathione S-transferase putative expressed

ISM IDs	Rice gene locus IDs	Physical positions (bp) on chromosomes	Forward primers (5'-3')	Reverse primers (5'-3')	Melting tempearature (T˚C)	Expected product size (bp) in PCR	Expected product size (bp) in Q-PCR	Putative Functions
OsISM03672	LOC_Os01g27420	15301259	CACTGAGGCCGGATAAATTC	CGAATTGCTTCTGCTGTTCA	59.8	144	55	retrotransposon protein putative Ty1-copia subclass
OsISM03673	LOC_Os01g27420	15304901	TGCCGGCTATCCTTATGAAC	CCGGAGTTTCTTAATTTCCTGA	59.8	130	81	retrotransposon protein putative Ty1-copia subclass
OsISM03674	LOC_Os01g27420	15305119	TCGAAGGAGATGGGTTTGAG	TTTGAACCCCTTGCAATCTC	60.1	1607	61	retrotransposon protein putative Ty1-copia subclass
OsISM03675	LOC_Os01g27430	15308178	TTATATGCTGGGCAATTTGG	ATGCAACTTTCAGGAGGTGG	59.3	197	98	retrotransposon protein putative unclassified
OsISM03676	LOC_Os01g27430	15308762	GCGTCATGTGTGGCTTCTAA	GACTCGTCGTCGCTGAAAAC	60.4	1285	88	retrotransposon protein putative unclassified
OsISM03677	LOC_Os01g27430	15310435	GCCATCAAAAGTTTGGCAGT	CCTCAGCTTTGCTGACCTCT	59.9	445	65	retrotransposon protein putative unclassified
OsISM03678	LOC_Os01g27430	15310945	ATGGCGTGCCAAACACTT	GTACGCAGTGGCGCAGTC	60.9	144	82	retrotransposon protein putative unclassified
OsISM03679	LOC_Os01g27430	15311361	CGATGCCCAAGATCACCC	CTTCTTGTTCTTGCTTCGCC	61.3	399	83	retrotransposon protein putative unclassified
OsISM03680	LOC_Os01g27480	15343537	ACGTCGACTTCGGTGACAG	AGCATCAGGTCTCCATCCTG	60.1	209	95	glutathione S-transferase putative expressed
OsISM03681	LOC_Os01g27480	15343722	GTTCCAGGATGGAGACCTGA	AGTATGTACCGGCAAATCGC	60.0	154	55	glutathione S-transferase putative expressed
OsISM03682	LOC_Os01g27500	15349539	CACATGGACCACGAATGAAC	GCCTGGCAACCTTTATTGAA	59.9	130	91	hypothetical protein
OsISM03683	LOC_Os01g27500	15349630	ATACGTGCAGGCTAAGCGTT	GTATGCAGGTACCCTCTCCG	59.8	412	69	hypothetical protein
OsISM03684	LOC_Os01g27500	15350056	AGTGGGTGGTTGTGTTGTCC	CATTCTGTTGACGAAGCCAG	60.1	320	83	hypothetical protein
OsISM03685	LOC_Os01g27550	15371959	GTGCCACTCGTTTTGTCAAG	GGCATTCTTTTTACCACCGA	59.6	200	95	retrotransposon protein putative unclassified
OsISM03686	LOC_Os01g27550	15372088	GGAAGGCTCATGATGGTAGC	ACTATCAAGCACCCAATGGC	59.8	198	97	retrotransposon protein putative unclassified
OsISM03687	LOC_Os01g27550	15372380	GCAAAGGAAAGGTAATTGGG	GCTCAATTGATTCATGCCAA	59.1	331	90	retrotransposon protein putative unclassified
OsISM03688	LOC_Os01g27550	15373458	AAGGCATACCGGGTCTACAA	TTCCACTTCAATAGGCTTGACA	59.6	180	97	retrotransposon protein putative unclassified
OsISM03689	LOC_Os01g27550	15374054	AAACAAAGCAAGGTTGGTGG	TGTGAAAAGGGTGGTGTCAA	60.0	363	80	retrotransposon protein putative unclassified

ISM IDs	Rice gene locus IDs	Physical positions (bp) on chromosomes	Forward primers (5'-3')	Reverse primers (5'-3')	Melting tempearature (T˚C)	Expected product size (bp) in PCR	Expected product size (bp) in Q-PCR	Putative Functions
OsISM03690	LOC_Os01g27550	15374662	GCCCATCAAGACACCTATGG	TCCTTTTCACGGCCACTAAG	60.3	203	79	retrotransposon protein putative unclassified
OsISM03691	LOC_Os01g27550	15374845	CTTAGTGGCCGTGAAAAGGA	GGACCGGGTTATTGGCTATT	60.1	367	98	retrotransposon protein putative unclassified
OsISM03692	LOC_Os01g27560	15380188	TGTTGAGGGGGATAGTGAGG	TTGAAGAGATCCTGCAACCC	60.1	322	97	retrotransposon protein putative unclassified
OsISM03693	LOC_Os01g27620	15404304	ATTCGTCATTCGTGTGGGAT	CCACCCTTGGCTGGATAGTA	60.1	696	83	hypothetical protein
OsISM03694	LOC_Os01g27620	15404957	CCGGTGACTTTGATGTGTCC	TTCCCAGGAAAAAGCGACTA	60.6	253	84	hypothetical protein
OsISM03695	LOC_Os01g27630	15409918	CAATTGCTGAGCACAAGAGC	AGAGCTGGGACTTGACCAAA	59.8	208	62	glutathione S-transferase putative expressed
OsISM03696	LOC_Os01g27630	15410099	ACAGCCATTTGGTCAAGTCC	ACGTACTTCGAAATTGCACG	59.4	384	76	glutathione S-transferase putative expressed
OsISM03697	LOC_Os01g27640	15413463	GCAATCAAGACTGCGAGTGA	CACGGTGCTACCTGCTATGA	60.0	1008	49	retrotransposon protein putative unclassified
OsISM03698	LOC_Os01g27640	15414462	AGCACCGTGGTAGAGCAGAT	CCTCCGATGTGGTTTTCCTA	59.9	166	91	retrotransposon protein putative unclassified
OsISM03699	LOC_Os01g27700	15444514	GAGCCAAGACTCGCCACTAC	GCCTCGTGTAGTTGGTCTCC	59.9	159	52	laccase precursor protein putative expressed
OsISM03700	LOC_Os01g27700	15444770	CAACGGCAACAAAAACATCA	GCACTGTGTGATGAACTCCG	60.4	195	89	laccase precursor protein putative expressed
OsISM03701	LOC_Os01g27700	15444993	ACCAGGTGATCCTGTTCGAG	GATGGTTCAGATCAAAACACG	59.1	245	97	laccase precursor protein putative expressed
OsISM03702	LOC_Os01g27700	15445132	GATACCCGTCATCCTCGGTA	GCTCAACGTCGTCATTCCA	60.3	167	98	laccase precursor protein putative expressed
OsISM03703	LOC_Os01g27700	15446316	TCCAGAACACTGTCTCCGTG	CAAAATGGCAATGCATGAAC	59.9	188	91	laccase precursor protein putative expressed
OsISM03704	LOC_Os01g27720	15452958	CCTACTCCTCGCCTTCTCCT	CGGTCTTCAAGGCACACTTA	59.4	1847	90	E3 ubiquitin-protein ligase MGRN1 putative expressed
OsISM03705	LOC_Os01g27720	15455199	GGTTTGCCTTTCTGAACCAA	AACTTGAGCACACTCCCTGC	60.3	994	77	E3 ubiquitin-protein ligase MGRN1 putative expressed
OsISM03706	LOC_Os01g27750	15479296	TCTCCCTGCCCTCGTCAC	ATCACCATCGTACTGGCCTC	61.2	5049	56	bifunctional 3-dehydroquinate dehydratase/shikimate dehydrogenase chloroplast precursor putative expressed
OsISM03707	LOC_Os01g27750	15484362	AGCACTCCGTTTAGCAATGG	TCTGGCTTACTGCCAGAAATAAA	60.3	1266	97	bifunctional 3-dehydroquinate dehydratase/shikimate dehydrogenase chloroplast precursor putative expressed

ISM IDs	Rice gene locus IDs	Physical positions (bp) on chromosomes	Forward primers (5'-3')	Reverse primers (5'-3')	Melting tempearature (T˚C)	Expected product size (bp) in PCR	Expected product size (bp) in Q-PCR	Putative Functions
OsISM03708	LOC_Os01g27750	15485779	TTCCAAGTGATGGTGCACTG	CATTAAACCTTTTTCGCCCA	60.3	1084	64	bifunctional 3-dehydroquinate dehydratase/shikimate dehydrogenase chloroplast precursor putative expressed
OsISM03709	LOC_Os01g27750	15487142	TCATCTCCGGATTTTTCAGG	GCGTCCACTTTGAAAGGAAG	59.9	1245	51	bifunctional 3-dehydroquinate dehydratase/shikimate dehydrogenase chloroplast precursor putative expressed
OsISM03710	LOC_Os01g27750	15488400	CATGAACATGACCCAGTTGC	ATGGTGTTTATGGCACCGAT	60.0	137	45	bifunctional 3-dehydroquinate dehydratase/shikimate dehydrogenase chloroplast precursor putative expressed
OsISM03711	LOC_Os01g27750	15488594	TTCTGCTATTGAGGATGGCA	TCCACCAGCACCTACAACAA	59.8	181	95	bifunctional 3-dehydroquinate dehydratase/shikimate dehydrogenase chloroplast precursor putative expressed
OsISM03712	LOC_Os01g27750	15488804	GGAGAAGGGTGCAAGAGTTG	CAAGCTCGGCTAATCTCAGG	60.0	1870	99	bifunctional 3-dehydroquinate dehydratase/shikimate dehydrogenase chloroplast precursor putative expressed
OsISM03713	LOC_Os01g27750	15490721	TTGGGAATGTACCCCAATGT	GGGCATATACCGCATCAAAT	59.8	262	86	bifunctional 3-dehydroquinate dehydratase/shikimate dehydrogenase chloroplast precursor putative expressed
OsISM03714	LOC_Os01g27750	15491058	GACAAGCCATGGGTCAGTTT	ATTTGGCAGCTATCTCACGC	60.2	189	79	bifunctional 3-dehydroquinate dehydratase/shikimate dehydrogenase chloroplast precursor putative expressed
OsISM03716	LOC_Os01g27770	15507514	TTCGTCTCCGGATTTTTCAG	CATGACAACACTGTACGGCA	59.7	1311	69	expressed protein
OsISM03717	LOC_Os01g27770	15508801	TTGATGCCGTACAGTGTTGTC	GTAGTTTGCCATCTGGCCTC	59.7	193	91	expressed protein
OsISM03718	LOC_Os01g27790	15514318	ACCACATCGCGTCTGTCAGT	CTGGAGTCTCTGGATCGGG	61.3	2790	90	retrotransposon protein putative unclassified
OsISM03719	LOC_Os01g27790	15518376	GACCTGGGGTTCCAGGAG	GTCGCCTCATGGTAGGGG	61.0	130	84	retrotransposon protein putative unclassified
OsISM03720	LOC_Os01g27790	15519041	GACGAGAGGCTCAGCACC	CCGCAGGAAGGAGACGAG	60.9	168	94	retrotransposon protein putative unclassified
OsISM03721	LOC_Os01g27790	15519337	GGAACGTCAAGCCTTCATCC	TAGTCGATGCACATCCGAAG	60.9	141	94	retrotransposon protein putative unclassified
OsISM03722	LOC_Os01g27790	15520195	GTTGCTATTCTACCTGGCGG	GGGTCAGAGGGCGATAGG	60.2	276	96	retrotransposon protein putative unclassified
OsISM03724	LOC_Os01g27790	15521127	CTCCTGGTGTTAGGCGACTC	GAAGCTCTATCCCGTCGAAA	59.6	133	77	retrotransposon protein putative unclassified
OsISM03725	LOC_Os01g27790	15521990	TAAGCACTCCGCGCTTAAAT	TGTCTTCGCAGTAGTCACCG	60.0	122	86	retrotransposon protein putative unclassified
OsISM03726	LOC_Os01g27930	15611012	CCCATCCAGCCTTTGACTAC	ACACGATGCTCCACGGTT	59.8	168	86	retrotransposon protein putative unclassified
OsISM03727	LOC_Os01g27930	15611225	CACCCCTCTTTGCGAGAA	GATGATGTCAGCCAGGAGGA	60.6	139	71	retrotransposon protein putative unclassified

ISM IDs	Rice gene locus IDs	Physical positions (bp) on chromosomes	Forward primers (5'-3')	Reverse primers (5'-3')	Melting tempearature (T˚C)	Expected product size (bp) in PCR	Expected product size (bp) in Q-PCR	Putative Functions
OsISM03728	LOC_Os01g27930	15611326	TTCATGTTGTGCAGGCTCTC	TAGACCCATCTTGCGTAGCC	60.1	457	88	retrotransposon protein putative unclassified
OsISM03729	LOC_Os01g27930	15612034	AGGCTGACGCGTGATCTG	GTAGGTAACCCACAGCGAGC	60.5	156	94	retrotransposon protein putative unclassified
OsISM03730	LOC_Os01g27930	15612450	GCGTAAGGACGTGATGGAAG	TGCTGGACATCCTCGACAT	60.4	231	88	retrotransposon protein putative unclassified
OsISM03731	LOC_Os01g27930	15612774	GACATGCATGACGACGACTC	TCCAGTATTGGATGTGACGG	59.8	184	72	retrotransposon protein putative unclassified
OsISM03733	LOC_Os01g27960	15630729	CAGGCACTGAAGTCACTTGG	CGAGGTGATTGAACTCATGG	59.3	153	97	retrotransposon protein putative Ty3-gypsy subclass
OsISM03734	LOC_Os01g27990	15644950	TTCCTGGTGCCTGTCAAGAT	ATTGCTACAGGGTTTCCACG	60.3	3031	84	NPL4 putative expressed
OsISM03735	LOC_Os01g28000	15649624	GACCTGGAGAAGTTCACGGA	CCATGTACGTCTTCGCCTTC	60.4	393	98	retrotransposon protein putative Ty3-gypsy subclass
OsISM03736	LOC_Os01g28000	15650085	GTGGGGTGGCTGGATGTTA	CCAAAAGCGTGTTGATGAAA	60.7	1536	77	retrotransposon protein putative Ty3-gypsy subclass
OsISM03737	LOC_Os01g28000	15651876	GCAAGGGAACACTGGTTCAG	TCTGTTGCAAAGCACTACCG	60.4	1177	97	retrotransposon protein putative Ty3-gypsy subclass
OsISM03738	LOC_Os01g28000	15653036	TAGTGCTTTGCAACAGAGGC	TCCTTGAGAGCCATCACCTT	59.5	2156	92	retrotransposon protein putative Ty3-gypsy subclass
OsISM03739	LOC_Os01g28000	15655550	TGCCGGGTCAATTAGTGAAT	AATTGGTTGCAAAATCTGCC	60.1	266	84	retrotransposon protein putative Ty3-gypsy subclass
OsISM03740	LOC_Os01g28000	15655964	AACCGAATCAAACGGTGGTA	CAAATTGAGGTTGAGCCGAT	60.2	161	67	retrotransposon protein putative Ty3-gypsy subclass
OsISM03741	LOC_Os01g28000	15656249	TGGAAATTTGTACCGGCAAC	AAAATGCTTCTCCAAATCGG	59.9	277	56	retrotransposon protein putative Ty3-gypsy subclass
OsISM03742	LOC_Os01g28000	15656512	TGGAGAAGCATTTTCACAGC	AATCGGCTAACTGGGCATC	59.5	174	97	retrotransposon protein putative Ty3-gypsy subclass
OsISM03743	LOC_Os01g28000	15657398	TTGCTGGAATGAGGATGTGA	TGCTTTGGCTTCGTTTTCTT	60.1	315	76	retrotransposon protein putative Ty3-gypsy subclass
OsISM03744	LOC_Os01g28000	15658033	TGATGCCTTACGCTACATTCA	CACGACGCCCTCAAAATAAT	59.6	459	99	retrotransposon protein putative Ty3-gypsy subclass
OsISM03745	LOC_Os01g28000	15659008	GATGCTGCTTCAGGACACAA	GGCTCTTTGATATGTAGCCCC	60.0	183	73	retrotransposon protein putative Ty3-gypsy subclass
OsISM03746	LOC_Os01g28000	15659470	TTGCAAGAGATAATCGGCAA	AACAGTGCATTCGTTGGACA	59.8	410	97	retrotransposon protein putative Ty3-gypsy subclass

ISM IDs	Rice gene locus IDs	Physical positions (bp) on chromosomes	Forward primers (5'-3')	Reverse primers (5'-3')	Melting tempearature (T˚C)	Expected product size (bp) in PCR	Expected product size (bp) in Q-PCR	Putative Functions
OsISM03747	LOC_Os01g28000	15659928	AAAGGAAGGGTTGGGAAGTG	GCCACAACCATGAGTGCATA	60.4	189	73	retrotransposon protein putative Ty3-gypsy subclass
OsISM03748	LOC_Os01g28060	15703195	TATGGCTAGGATGGGTTGGA	CCCCCTTTTCACTTTCACAG	59.9	1572	96	transposon protein putative CACTA En/Spm sub-class
OsISM03749	LOC_Os01g28060	15705254	TGAAGAGCGTCAACAGGATG	TGTCGTTACCAGGTTGCTTG	59.9	243	66	transposon protein putative CACTA En/Spm sub-class
OsISM03750	LOC_Os01g28060	15706035	TTGCGTAAACCTTCTTGGCT	CTTTCCTTCTCTGCCTTGCT	59.3	177	94	transposon protein putative CACTA En/Spm sub-class
OsISM03751	LOC_Os01g28060	15707148	TGCCATGGGACACGATAGTA	CATCCCACTGTCATCAATCG	59.9	151	68	transposon protein putative CACTA En/Spm sub-class
OsISM03752	LOC_Os01g28060	15707576	CACGTAGCGATCACAAGGAA	GGGAATACGTCGAAGTTTGG	59.6	1638	95	transposon protein putative CACTA En/Spm sub-class
OsISM03753	LOC_Os01g28070	15710468	CAAGACAAGGTCTCGCCAAG	GGCTTGAGTTGCCTCTTCAC	60.5	156	84	transposon protein putative CACTA En/Spm sub-class
OsISM03754	LOC_Os01g28070	15710768	CCCTCAGCTTGGAGAACAAC	AACTTGTATGCCACTTCCGC	60.0	153	76	transposon protein putative CACTA En/Spm sub-class
OsISM03755	LOC_Os01g28070	15711123	GCCCTCGACGTCTCTCTTCT	GAGGGTCGATGAACCCAGTA	60.5	182	92	transposon protein putative CACTA En/Spm sub-class
OsISM03758	LOC_Os01g28140	15770335	GTGTCCGTGGACTGCCACTA	CTGCATGAAAGCTCCAAACA	61.1	134	81	transposon protein putative unclassified
OsISM03759	LOC_Os01g28140	15770893	ACGATATGGACGAGTCGGAG	CCGACACATCGGTAGGAGG	60.8	886	60	transposon protein putative unclassified
OsISM03760	LOC_Os01g28140	15772080	GACCTACGAAGAGGTGGCTG	GCAAATACGCCTCCAAACAT	59.9	181	94	transposon protein putative unclassified
OsISM03761	LOC_Os01g28140	15772318	GACTCGTGCACTACGCCAG	AAAGAAAGAGAGGGCACCCA	60.9	162	88	transposon protein putative unclassified
OsISM03762	LOC_Os01g28140	15772583	GATGGGTACTCTGTGGTGCC	TCAGGATACGACCGCCTAAC	60.2	149	67	transposon protein putative unclassified
OsISM03763	LOC_Os01g28140	15772941	AGCCCTTTCCAGTACCGTTT	AGCTGACTGCTGACCCTTG	59.6	181	75	transposon protein putative unclassified
OsISM03764	LOC_Os01g28140	15773296	CACGTTGCGAGCACTAGAGA	TGAACTGTCAACGACAACCC	60.0	188	94	transposon protein putative unclassified
OsISM03765	LOC_Os01g28140	15773670	CACGTTCCTAGGCCGACTC	GGGGCACAACAGAAGGACTA	60.5	166	89	transposon protein putative unclassified
OsISM03766	LOC_Os01g28140	15773816	TAGTCCTTCTGTTGTGCCCC	CTGCGTCATAGCGAACTGG	60.3	138	50	transposon protein putative unclassified

ISM IDs	Rice gene locus IDs	Physical positions (bp) on chromosomes	Forward primers (5'-3')	Reverse primers (5'-3')	Melting tempearature (T˚C)	Expected product size (bp) in PCR	Expected product size (bp) in Q-PCR	Putative Functions
OsISM03767	LOC_Os01g28150	15776570	AGTCCTGTGGACGGAATCTG	TCTTGGAATTCATCTCGCCT	59.9	1105	99	OsFBX6 - F-box domain containing protein expressed
OsISM03768	LOC_Os01g28160	15781058	GCAGAGGCCGATGTTGAG	TGGCTTAATTAGCACATTGACA	59.5	923	73	hypothetical protein
OsISM03769	LOC_Os01g28170	15785598	TCGTCTCTGTCCATGTTTCG	AACCCCGACTCAAACAAGTG	59.9	1164	75	transposon protein putative CACTA En/Spm sub-class expressed
OsISM03770	LOC_Os01g28170	15787547	AAGAATTTGGAAAGGACGCA	ACACGTCGATGTCGTTACCA	59.9	209	67	transposon protein putative CACTA En/Spm sub-class expressed
OsISM03771	LOC_Os01g28184	15791636	CGTGAATGAGAACAAGCGAA	ATCTGTTCCTCATTGCGGTC	60.0	752	91	transposon protein putative CACTA En/Spm sub-class
OsISM03772	LOC_Os01g28184	15793742	CCATGTGAGCTGCATATTCC	AGTACCTGAAGGGTCCGTTG	59.1	158	82	transposon protein putative CACTA En/Spm sub-class
OsISM03773	LOC_Os01g28184	15793970	ACCTCGAGCTGGATTACCCT	AGGATCCTGAGGAGGAGTCG	60.4	236	75	transposon protein putative CACTA En/Spm sub-class
OsISM03774	LOC_Os01g28184	15794816	CCGGTAGAGAGATGATCGGA	TCCAGTATCGAAAACCCTCC	59.6	297	90	transposon protein putative CACTA En/Spm sub-class
OsISM03775	LOC_Os01g28184	15795194	AAGGCGCAGCATTACAAGAC	GAGTCATATACGTTGACGGTGC	60.0	201	69	transposon protein putative CACTA En/Spm sub-class
OsISM03776	LOC_Os01g28184	15795371	CTGCACCGTCAACGTATATGA	AATGACGGAACCGATACCAA	59.9	182	96	transposon protein putative CACTA En/Spm sub-class
OsISM03777	LOC_Os01g28184	15795560	CGGCAAATGGAGAGAAAGAC	GGCAATACTCGCACACGTAA	59.8	195	99	transposon protein putative CACTA En/Spm sub-class
OsISM03778	LOC_Os01g28184	15795755	ACTGCCTTGCAGACCAAATC	CCGCGATAAATTCCTTGTGT	60.1	169	88	transposon protein putative CACTA En/Spm sub-class
OsISM03780	LOC_Os01g28200	15800073	AAGGCAGCGAAAGAACAAAA	GATTTGGTGTGCTTGTTCCA	59.8	147	69	transposon protein putative unclassified
OsISM03781	LOC_Os01g28200	15800255	TGCAGCTCCTTAGCTTGTGA	TCATGAAGTAGCCTGTTGCG	59.9	197	78	transposon protein putative unclassified
OsISM03782	LOC_Os01g28260	15834845	CTGTCAAGTACTCAGCGCCA	TGCAGTAGGGTGTGAGCAAG	60.1	251	92	hypothetical protein
OsISM03783	LOC_Os01g28260	15835208	TGCCGCCTCCAATTCTATAC	TCCCCTGTCCTGACAATAGG	60.0	218	86	hypothetical protein
OsISM03784	LOC_Os01g28260	15835406	CCTATTGTCAGGACAGGGGA	GAGCAATCGAGCAGACATCA	60.0	127	92	hypothetical protein
OsISM03785	LOC_Os01g28330	15865858	GGATTGTGTTATGCAAGGGG	CCGGCTGCACAGTTCTATCT	60.3	295	94	retrotransposon protein putative Ty3-gypsy subclass

ISM IDs	Rice gene locus IDs	Physical positions (bp) on chromosomes	Forward primers (5'-3')	Reverse primers (5'-3')	Melting tempearature (T˚C)	Expected product size (bp) in PCR	Expected product size (bp) in Q-PCR	Putative Functions
OsISM03786	LOC_Os01g28330	15866151	GGGATGGTTGTTCAGAATGG	CTCCTGGACAAATCCCTCAA	60.1	1063	90	retrotransposon protein putative Ty3-gypsy subclass
OsISM03787	LOC_Os01g28330	15867789	GAAAGAAGTTACCCCAGCCC	TGCTAGAGAAAATAGGCGGC	59.8	664	90	retrotransposon protein putative Ty3-gypsy subclass
OsISM03788	LOC_Os01g28330	15870333	GAGCATGAAGAACATCTTCGC	ACCTTCCCATCTTGCATCAG	60.0	458	93	retrotransposon protein putative Ty3-gypsy subclass
OsISM03789	LOC_Os01g28340	15874801	GGATTGTGTTATGCAAGGGG	CCGGCTGCACAGTTCTATCT	60.3	295	94	retrotransposon protein putative Ty3-gypsy subclass
OsISM03790	LOC_Os01g28340	15875094	GGGATGGTTGTTCAGAATGG	CTCGACCTCGATGGTGCT	60.1	1663	93	retrotransposon protein putative Ty3-gypsy subclass
OsISM03792	LOC_Os01g28400	15916862	GATTGCGAGGGAAAACAGAA	AAACCTCCATGGTGCAACTC	60.1	259	80	transposon protein putative unclassified
OsISM03793	LOC_Os01g28400	15917129	GGTGCGGTCATCTTAGGTCT	ACCCTCAGCCCATCACTAAG	59.2	182	96	transposon protein putative unclassified
OsISM03794	LOC_Os01g28400	15917290	TCTTAGTGATGGGCTGAGGG	CCTGGTCCAGCTTGGTCTAC	60.0	235	97	transposon protein putative unclassified
OsISM03795	LOC_Os01g28400	15918613	CATACCCTTGGACCATAATGA	CCTATGCTCTGTGTCAGGGA	58.1	163	81	transposon protein putative unclassified
OsISM03796	LOC_Os01g28460	15944757	CAAGGAATCGAACAATGGCT	CTCTGTCCCTCATCCTCAGC	60.0	635	96	retrotransposon putative centromere-specific
OsISM03798	LOC_Os01g28474	15948129	ATGAGCAGAGCTTTGTGTGC	GATGGCGGTGCAGAGGAT	60.7	2816	84	transferase family protein putative expressed
OsISM03799	LOC_Os01g28490	15954808	CGAGAACGACAACATCAACG	CCTTAGGAGAGAGAGGCGGT	60.1	414	99	retrotransposon protein putative Ty1-copia subclass
OsISM03800	LOC_Os01g28490	15955319	AGCGAAGGATATGTGGGATG	ACACCCAAAGAGCCAATGAG	60.0	300	91	retrotransposon protein putative Ty1-copia subclass
OsISM03802	LOC_Os01g28540	15975010	CAGCTGCATTCGAGCTTTCT	TCTCACTCCACCACCGAAAT	60.7	547	99	stress regulated protein putative expressed
OsISM03803	LOC_Os01g28540	15975573	CAATTGGTCACAATCTTAGCC A	GACCCCAAGCAAATACGCTA	60.0	1036	87	stress regulated protein putative expressed
OsISM03804	LOC_Os01g28540	15976674	AGCTGGGACAGCTTTTGTTG	TAGAAACCCTTACCGTGGCA	60.5	923	78	stress regulated protein putative expressed
OsISM03805	LOC_Os01g28540	15977553	CCAGGTCAATTCAGGCAAAC	TCTGTTGCTACTCCAGCCAA	60.0	1422	76	stress regulated protein putative expressed
OsISM03806	LOC_Os01g28540	15978955	TTGGCTGGAGTAGCAACAGA	CCTAGACCTTTGAGCAATCCA	59.5	204	93	stress regulated protein putative expressed

ISM IDs	Rice gene locus IDs	Physical positions (bp) on chromosomes	Forward primers (5'-3')	Reverse primers (5'-3')	Melting tempearature (T˚C)	Expected product size (bp) in PCR	Expected product size (bp) in Q-PCR	Putative Functions
OsISM03807	LOC_Os01g28580	15992267	ATCGCCACGGAGGTCTATC	CAACAGAGATGCACTCCCAA	59.9	1088	99	expressed protein
OsISM03808	LOC_Os01g28580	15993423	TCAACTGCCATCTGCTCATT	TTGCTGATTGCAAGAAGTGG	59.7	881	98	expressed protein
OsISM03809	LOC_Os01g28580	15994270	CTTTGTCGGATCTGCCACTT	AGCCCCCAACAATCTTAACC	60.2	856	62	expressed protein
OsISM03810	LOC_Os01g28610	16003694	TTCAAAGGCTGATGAAAGGG	ACGCTCCAGCAACAAAAATC	60.2	782	74	hypothetical protein
OsISM03811	LOC_Os01g28610	16004509	CGATTTTGGGGAACGTACAG	GCCACTCCACATCAGCATTA	60.0	635	98	hypothetical protein
OsISM03812	LOC_Os01g28610	16005196	CACCCAACGAAAATGGAGAG	ATTTGATTCGAACCCCATCA	60.3	177	93	hypothetical protein
OsISM03813	LOC_Os01g28610	16005402	TATGCTGAACCACAGATGGG	TCCGGATCTTCGTATAGCTCA	59.7	160	63	hypothetical protein
OsISM03814	LOC_Os01g28630	16019553	AAAAGGCTTATGGCAGGGTT	TTGTTTTTGCCTTTTTCAGGA	59.8	253	88	retrotransposon protein putative unclassified
OsISM03815	LOC_Os01g28630	16019820	GGTTTGATTCCTGAGCTAATT CC	TCCTCATCTTTCTCTACACCCA	59.6	524	96	retrotransposon protein putative unclassified
OsISM03817	LOC_Os01g28660	16033177	ATCTTGGCAATGACTCGGAC	GTTGGTTGGGGTAACCAGG	60.1	786	83	hypothetical protein
OsISM03819	LOC_Os01g28660	16034621	GCAGAGCTTGTGTGGTGGT	TCACTAAGCGTGACCAATCG	59.9	309	75	hypothetical protein
OsISM03820	LOC_Os01g28660	16034949	CTTCTCCTGGGCTTGTGTGT	GTTGCAAGTCATGCTCCAAA	60.1	389	65	hypothetical protein
OsISM03821	LOC_Os01g28660	16035327	TGACTTGCAACAGGCAAAGA	CTAAAACCGGAGCCAATGAG	60.1	1445	85	hypothetical protein
OsISM03822	LOC_Os01g28660	16036732	GCAGGTTATTTTCAGGGCAA	CTACTTCGTCCGTTCCGGT	60.1	562	88	hypothetical protein
OsISM03823	LOC_Os01g28660	16037392	GATCAGTGGCATAGGGGAGA	TCTGCTGATTTTTGCCTGAA	59.8	412	64	hypothetical protein
OsISM03825	LOC_Os01g28670	16040569	ATCATCCAAGCCTCAAGCTC	CGACCTACTCGAGTGCTCAAT	59.5	496	98	hypothetical protein
OsISM03826	LOC_Os01g28680	16047364	AGTAAAAGGCCCATGGTGTG	TGTTGGCATAAAGGATTGTCA	59.4	217	83	WD domain G-beta repeat domain containing protein expressed
OsISM03827	LOC_Os01g28680	16047613	TGCTGAGAAAAAGTGGACGA	AAGCCTGCAAAAAGAACGAG	59.6	1101	99	WD domain G-beta repeat domain containing protein expressed

ISM IDs	Rice gene locus IDs	Physical positions (bp) on chromosomes	Forward primers (5'-3')	Reverse primers (5'-3')	Melting tempearature (T˚C)	Expected product size (bp) in PCR	Expected product size (bp) in Q-PCR	Putative Functions
OsISM03828	LOC_Os01g28680	16048705	TGCAGGCTTATTCCAGTCTGT	CAAGCCTTCGTGAACTTGCT	60.2	1068	99	WD domain G-beta repeat domain containing protein expressed
OsISM03829	LOC_Os01g28680	16049850	TGTTGGAAACAACAATGAATCAA	TGACTGCATTGACATCAGCC	60.6	1008	96	WD domain G-beta repeat domain containing protein expressed
OsISM03830	LOC_Os01g28680	16050851	GCAGTCACCTTTGCTGATGA	TCCCAGACCTGAAGATTGGA	60.3	490	72	WD domain G-beta repeat domain containing protein expressed
OsISM03831	LOC_Os01g28680	16051417	CGTGGAGATGGGCATTATTT	GCAGCTGCAAAAACAAACTACA	60.1	339	91	WD domain G-beta repeat domain containing protein expressed
OsISM03832	LOC_Os01g28680	16051821	GCATCCATATGACCAGTCCC	CGTGCTGCAAAAAGAGAAGA	59.8	1993	89	WD domain G-beta repeat domain containing protein expressed
OsISM03833	LOC_Os01g28680	16053794	TCTTCTCTTTTTGCAGCACG	AACAATTTTTCCAGTAGCCTGC	59.5	882	93	WD domain G-beta repeat domain containing protein expressed
OsISM03834	LOC_Os01g28690	16061970	AGTCCCACGTTTTCTTTCCC	CATAGACCCTGCTGCCTTTC	60.1	1269	62	nucleoporin autopeptidase domain containing protein expressed
OsISM03835	LOC_Os01g28690	16063319	CATGAGGAGCTTCGACATGA	AATTTTCTGCAGGCCATCAC	60.0	607	64	nucleoporin autopeptidase domain containing protein expressed
OsISM03836	LOC_Os01g28690	16064389	CGGCTGCAAATACAGTCTCA	GACCAAGGGATGATGTGGTT	59.8	140	61	nucleoporin autopeptidase domain containing protein expressed
OsISM03837	LOC_Os01g28690	16064593	TGTATTCAGCAACTCAGCGG	TGGTATTCGTGGGAACATCC	60.3	248	60	nucleoporin autopeptidase domain containing protein expressed
OsISM03838	LOC_Os01g28690	16064899	TGATGGTAATGGAAGTGTTGCT	TGCACAAAGAAATGGGTGTT	59.3	301	78	nucleoporin autopeptidase domain containing protein expressed
OsISM03839	LOC_Os01g28690	16065277	AGTCCAACTGGTGGTCATCC	ACCAGCAGCACTGTTGCATA	60.1	617	64	nucleoporin autopeptidase domain containing protein expressed
OsISM03840	LOC_Os01g28720	16079669	GAATTAACGGTGTCGCTCGT	CAATGTATTTGGCGCTGACC	60.8	985	58	hypothetical protein
OsISM03841	LOC_Os01g28750	16103768	GCTGGGTCATGGGACAGATA	AGGATGGTTTCGCAATGAAC	60.4	1298	89	retrotransposon putative centromere-specific
OsISM03842	LOC_Os01g28750	16105260	GCCAAAAGAGACCACCAGTC	CGGGAAGTTCTGTCTCTTGG	59.8	295	59	retrotransposon putative centromere-specific
OsISM03843	LOC_Os01g28750	16105554	GGAGCCCATTTGGTCCTATT	TTGATGTTGAGGACTCCGGT	60.3	183	83	retrotransposon putative centromere-specific
OsISM03844	LOC_Os01g28760	16107472	AACACCAATGGAGGCATTTC	CTTGGCCTCAAGTACATCCC	59.7	578	83	retrotransposon protein putative Ty1-copia subclass
OsISM03845	LOC_Os01g28760	16108066	CCGGCAGTGAGCTGTATGT	AAACTTGTCCGGCAACTCAC	60.0	140	96	retrotransposon protein putative Ty1-copia subclass

ISM IDs	Rice gene locus IDs	Physical positions (bp) on chromosomes	Forward primers (5'-3')	Reverse primers (5'-3')	Melting tempearature (T˚C)	Expected product size (bp) in PCR	Expected product size (bp) in Q-PCR	Putative Functions
OsISM03846	LOC_Os01g28760	16108315	GTGTTGAAGAGAAGGCGAGG	TGTGAAATGCATGAGGGTTC	59.7	102	67	retrotransposon protein putative Ty1-copia subclass
OsISM03847	LOC_Os01g28760	16108545	GAGCGCAAAGACAGGAAGTC	GCAAGTGACCCGTTTACCAT	60.0	963	72	retrotransposon protein putative Ty1-copia subclass
OsISM03848	LOC_Os01g28760	16109488	ATGGTAAACGGGTCACTTGC	GCTTGCGAGGTTGTTTCAAT	60.1	443	80	retrotransposon protein putative Ty1-copia subclass
OsISM03849	LOC_Os01g28760	16110464	CTACGAACATGGGGCTGTTT	GAAGTGCTATGTGTATTTTTCATGTG	59.5	233	84	retrotransposon protein putative Ty1-copia subclass
OsISM03850	LOC_Os01g28770	16112358	ACCGCTACCTCGGCTTTTAT	CTTGAGGAGTTGAGGCCTTG	60.0	1205	94	hypothetical protein
OsISM03851	LOC_Os01g28820	16143902	GGATCGGGTATTTGGTCACA	GCTCATCCACAAGCACAAAA	60.2	125	84	retrotransposon putative centromere-specific
OsISM03852	LOC_Os01g28910	16196006	TCGGCTCCAATCCTTCAGTA	CCCTTCCACCACTGATTGTG	61.1	159	96	retrotransposon protein putative unclassified
OsISM03853	LOC_Os01g28989	16231475	CTTCCTTGCTGGAGAGCTG	CTCCCATTCGACAGGTTTCT	59.0	501	99	expressed protein
OsISM03854	LOC_Os01g29000	16234756	CTGGCTATGAAATTCGTCCC	CGATCTTCCAGCGGTTATGT	59.8	479	72	retrotransposon putative centromere-specific
OsISM03855	LOC_Os01g29000	16235968	TCAACACACCCCGATTAGGT	GGAGACTCATGAGGGGGAAC	60.5	151	98	retrotransposon putative centromere-specific
OsISM03856	LOC_Os01g29000	16236445	CCTCTAGGGAAGCTCAACGA	AGCGGGAAATGATCCTTCTT	59.8	268	74	retrotransposon putative centromere-specific
OsISM03857	LOC_Os01g29000	16236764	TTTCCTTGATGGGTATTCCG	ACACTCGATGCCCAAGAACT	59.7	350	78	retrotransposon putative centromere-specific
OsISM03858	LOC_Os01g29000	16237947	GTCTGCTCCGATGGCTTACT	CATAGTCGACTGCCACCAGA	59.7	340	89	retrotransposon putative centromere-specific
OsISM03859	LOC_Os01g29000	16238477	TCCTTACCATCCACAGACCAG	GCAGTCGACATGACTTTCCA	59.9	200	99	retrotransposon putative centromere-specific
OsISM03860	LOC_Os01g29000	16238985	ATCACACTCCGCGACGAT	ACCTTCATAGGTGGAGGGCT	60.1	1035	96	retrotransposon putative centromere-specific
OsISM03861	LOC_Os01g29050	16259447	TCAAGCCCTTGGACTCACAC	CTGACCTCCTTGTGGACGAT	60.7	137	81	retrotransposon protein putative unclassified
OsISM03862	LOC_Os01g29050	16260383	TGCCAAAATTGACGTCTTTG	GACCCCTTGAACAGACGAGA	60.0	1252	71	retrotransposon protein putative unclassified
OsISM03863	LOC_Os01g29050	16262215	CTGAGACCCATTAGGGGAGA	TATGTTCCACCTGCACGAGA	59.7	394	75	retrotransposon protein putative unclassified

ISM IDs	Rice gene locus IDs	Physical positions (bp) on chromosomes	Forward primers (5'-3')	Reverse primers (5'-3')	Melting tempearature (T˚C)	Expected product size (bp) in PCR	Expected product size (bp) in Q-PCR	Putative Functions
OsISM03864	LOC_Os01g29050	16262897	TAGGAGGATGGACACCCTTG	GCTCAGGGTCCTCGATAAAG	59.4	198	69	retrotransposon protein putative unclassified
OsISM03865	LOC_Os01g29050	16263075	CTTTATCGAGGACCCTGAGC	CCTCAACGTAAACGCTCTCC	59.4	2811	95	retrotransposon protein putative unclassified
OsISM03868	LOC_Os01g29080	16282795	GCGAATCTCAATTTGCCCTA	CAATCTTGACATCGCCAGAG	59.8	142	80	retrotransposon protein putative unclassified
OsISM03869	LOC_Os01g29080	16283030	CATCCAAGAAGCGCATCAC	AAGACGTTGGAGTTTGCCC	60.2	153	98	retrotransposon protein putative unclassified
OsISM03870	LOC_Os01g29160	16338330	AGGCTTTGCAGTCAATGAGG	CACCTCAAGGTTTGTCCCAA	60.7	117	74	retrotransposon protein putative Ty1-copia subclass
OsISM03872	LOC_Os01g29170	16341918	ACAACCGCGAGTAGTTCCTG	GACATGGGCTTCATCTCCA	59.9	239	79	retrotransposon protein putative Ty1-copia subclass
OsISM03873	LOC_Os01g29170	16342213	AAAGAGCATGCTGGAGGAGA	AAGGTTTCCATGGAGTCATCC	60.1	346	90	retrotransposon protein putative Ty1-copia subclass
OsISM03874	LOC_Os01g29190	16355926	CAAGCTTTCGACCAGGCTAC	GCCTCCACCCTTCATCATTA	60.0	738	62	retrotransposon protein putative unclassified
OsISM03875	LOC_Os01g29190	16356799	CAGCCCATGGTGATGAAAAT	GACATCACGAAGTTGCTCCA	60.3	125	54	retrotransposon protein putative unclassified
OsISM03877	LOC_Os01g29220	16365736	TGCAATTCGGTTTGTAGCTC	CTCCCTCAATGCACCAGATT	59.5	949	81	expressed protein
OsISM03878	LOC_Os01g29220	16366713	GGCAAAGTTGGTTGGTTTTG	CGGTACTAGCGGTGCATTTT	60.3	549	93	expressed protein
OsISM03879	LOC_Os01g29220	16367235	ATCGAGTAAAATGCACCGCT	TAATCTCCACCATCTTCGGA	58.9	993	99	expressed protein
OsISM03880	LOC_Os01g29220	16368349	CAGAATGGCCTGAGGTGTCT	TTTGCTACAACTTTAACTAGGTATTCA	58.7	479	83	expressed protein
OsISM03881	LOC_Os01g29230	16376690	ATGCCCCTCCTGTCCATACT	CAAAAGCACACAATTGAACCA	60.2	583	99	expressed protein
OsISM03882	LOC_Os01g29230	16377411	AAATTCGCGAAATGGTCAAG	TCGGGATCAAATCCCAGAT	60.2	769	88	expressed protein
OsISM03883	LOC_Os01g29230	16379326	AGTTCATTGTCGCGAATGCT	CATTCGAGGATCGTCAGCTA	59.9	191	92	expressed protein
OsISM03884	LOC_Os01g29230	16380905	GTCTGCAAACATGCCGATAG	TGGCCCATAGTTCACACAAA	59.6	178	95	expressed protein
OsISM03885	LOC_Os01g29230	16382001	CTGGAGGATCAGTTTCCCAA	ATGCGATCAACCACAATCAA	60.0	197	72	expressed protein

ISM IDs	Rice gene locus IDs	Physical positions (bp) on chromosomes	Forward primers (5'-3')	Reverse primers (5'-3')	Melting tempearature (T˚C)	Expected product size (bp) in PCR	Expected product size (bp) in Q-PCR	Putative Functions
OsISM03886	LOC_Os01g29230	16382503	GGTGTGTGCTTGATTGGTCA	GGTTGGCAATATTTGTTGGG	60.3	2013	94	expressed protein
OsISM03887	LOC_Os01g29230	16384966	CAGGTGAAGTGCGACGATTA	ATGACCACCAAGGAGGAGTG	59.9	172	85	expressed protein
OsISM03888	LOC_Os01g29230	16385154	ATCAGATTTGGCTGGCTGTC	TTTGCTCAGTGTTTCACCAA	59.0	1279	95	expressed protein
OsISM03889	LOC_Os01g29230	16386520	CCTTATGAGAATCTTCCATCTTGTG	TGCTGCATAAATGCCTTCAC	60.1	1860	61	expressed protein
OsISM03890	LOC_Os01g29260	16399938	CTGGATGGAATCCAAAAAGG	ACCGGATCCGAATCCTCTAT	59.6	150	76	retrotransposon protein putative unclassified
OsISM03891	LOC_Os01g29260	16400204	TGTCGGGGTATGAAGTTGCT	ATCATCAGTTGGCTGACACG	60.1	244	77	retrotransposon protein putative unclassified
OsISM03892	LOC_Os01g29260	16400484	CTTTTGCGAGAAAGGGGC	GCCACCTGATAGTCCACGAG	60.8	160	91	retrotransposon protein putative unclassified
OsISM03893	LOC_Os01g29260	16400751	TAGTCGGACATCGTCGGAAG	ACTTCTCGCTGAGCATGACA	60.5	166	94	retrotransposon protein putative unclassified
OsISM03894	LOC_Os01g29260	16400890	AGCGGCTTGTCATGCTCAG	CTCCAGACCTATCCTGCACC	61.2	128	61	retrotransposon protein putative unclassified
OsISM03896	LOC_Os01g29260	16401684	GCAAGGATGTGATGGAGGAG	CTGGACATCCTCGACATCAA	60.1	222	84	retrotransposon protein putative unclassified
OsISM03897	LOC_Os01g29260	16401972	GGCGATGTAGATCGGAGTTC	CGATACTGGATGCGACGAT	59.7	211	99	retrotransposon protein putative unclassified
OsISM03898	LOC_Os01g29290	16417634	GTACACTCTGCTCCGGGTTC	TTGTAGCAAACTCTTGCCCA	59.6	905	89	retrotransposon protein putative Ty3-gypsy subclass
OsISM03899	LOC_Os01g29310	16423603	ATGGCGACGAAAATACCAAG	GCGAATGCCGAGTAGATGTT	60.1	185	57	retrotransposon protein putative unclassified
OsISM03900	LOC_Os01g29320	16425411	GAAGTGCAAAAGCAGCTCAA	AAATCGTCCTTCGGGTACG	59.6	152	82	retrotransposon protein putative unclassified
OsISM03901	LOC_Os01g29320	16425857	GCGTCCGACTTACAGGAGAC	GGCTCTGCAGATATCGCTTT	59.7	113	74	retrotransposon protein putative unclassified
OsISM03902	LOC_Os01g29320	16426244	GGGCAAAGAAATTACTGGCT	AACTGTAGCCTTGCGGCATA	59.6	269	97	retrotransposon protein putative unclassified
OsISM03903	LOC_Os01g29320	16427023	TATAATTCCAAGCAATGGCG	TTTGTATCAACTTTTATGTACCTCGC	59.3	1920	61	retrotransposon protein putative unclassified
OsISM03904	LOC_Os01g29340	16436739	GAGAATCACCAGGGTCTTGC	ATAACTCCGCCCAACCTTCT	59.8	363	95	retrotransposon protein putative unclassified

ISM IDs	Rice gene locus IDs	Physical positions (bp) on chromosomes	Forward primers (5'-3')	Reverse primers (5'-3')	Melting tempearature (T˚C)	Expected product size (bp) in PCR	Expected product size (bp) in Q-PCR	Putative Functions
OsISM03905	LOC_Os01g29340	16437443	GCACCTGGGAAGATTGGATA	CTTCCACTGGTATGTTCAGCC	59.8	447	89	retrotransposon protein putative unclassified
OsISM03906	LOC_Os01g29340	16437869	GGCTGAACATACCAGTGGAAG	ATCAAATGCCCTGTGAGGAG	59.8	480	84	retrotransposon protein putative unclassified
OsISM03907	LOC_Os01g29340	16438368	CAGGGGTGAAACCAGTCAAT	ACCACTGGCATTGGGTACTT	59.6	232	80	retrotransposon protein putative unclassified
OsISM03908	LOC_Os01g29340	16438904	CAAGTCCTGGCTCAACATCA	TGTTGCACAGCTACCACCTT	59.6	137	75	retrotransposon protein putative unclassified
OsISM03909	LOC_Os01g29340	16439827	CCTACTAGCCTTGCACAGCA	CCCGGACTCTTACAGTGCTC	59.6	170	90	retrotransposon protein putative unclassified
OsISM03910	LOC_Os01g29340	16439930	TGCATGATTATGTGCAGGGT	TATACCAAAACTGCGCCTGG	60.5	506	80	retrotransposon protein putative unclassified
OsISM03911	LOC_Os01g29340	16440556	TACAGGAGCGCTCTCAGATG	TAGCTCCAATTCGACCCAAA	59.9	238	97	retrotransposon protein putative unclassified
OsISM03912	LOC_Os01g29340	16440841	GTGCATGTATCGCTCCTCAA	CATATGGCACAGTGGCACC	60.4	154	95	retrotransposon protein putative unclassified
OsISM03913	LOC_Os01g29350	16446209	GGATGCAAATTGACTGGGAC	GTAGCCATGTCCTTTTCCGA	60.2	458	76	hypothetical protein
OsISM03914	LOC_Os01g29370	16460869	TGGAGCGCATGATAAAGCTA	GACGATGATGGCATTTGAGA	59.6	215	99	retrotransposon protein putative unclassified
OsISM03915	LOC_Os01g29370	16461114	TTCAATGCCTTCAATCCTCC	GGATCCGACTTCATCAAAGC	59.8	139	51	retrotransposon protein putative unclassified
OsISM03916	LOC_Os01g29370	16461486	ATCCAGTGCCAAAGATCAGG	TGGTCGGGTCAGAGTCTTTT	59.9	154	76	retrotransposon protein putative unclassified
OsISM03917	LOC_Os01g29370	16461898	CTCCCGTTAAGACGCAAGAG	CAGAGCCTTGACCTGCTTCT	59.9	698	96	retrotransposon protein putative unclassified
OsISM03918	LOC_Os01g29370	16462610	CGAATTAGCAAAGCAGAGGG	CTGTGCCTGGGACTTGATTT	60.0	132	68	retrotransposon protein putative unclassified
OsISM03919	LOC_Os01g29409	16480173	AAGAGGGTTGATGACCTCCC	TTGCACAAGCTCGTAGGATG	60.2	577	88	tRNA methyltransferase putative expressed
OsISM03920	LOC_Os01g29409	16480732	TCCTACGAGCTTGTGCAATG	GGAACGATTATTCCTTCTGGG	59.9	1530	96	tRNA methyltransferase putative expressed
OsISM03921	LOC_Os01g29409	16482289	TGCACACTTGAACTTGAGGG	TGGATCTTTGGCTTGTTTTTG	60.0	157	85	tRNA methyltransferase putative expressed
OsISM03922	LOC_Os01g29409	16482542	TGAAAGTGGTCTTCGGTTCC	TGGTCAACAAGCCTCTGTCTT	60.0	7221	85	tRNA methyltransferase putative expressed

ISM IDs	Rice gene locus IDs	Physical positions (bp) on chromosomes	Forward primers (5'-3')	Reverse primers (5'-3')	Melting tempearature (T˚C)	Expected product size (bp) in PCR	Expected product size (bp) in Q-PCR	Putative Functions
OsISM03923	LOC_Os01g29409	16489742	AAGACAGAGGCTTGTTGACCA	TGGACCAACACCAGAGAACA	60.0	892	71	tRNA methyltransferase putative expressed
OsISM03924	LOC_Os01g29409	16490720	TGTTCTTAACAAGCTCGAGAG AAA	GATGCACATGTTGGCTTGAG	60.0	412	90	tRNA methyltransferase putative expressed
OsISM03925	LOC_Os01g29409	16491122	CATGTGCATCCAGTCACACA	TGGTGGTTCCATGAGATTCC	60.4	1139	90	tRNA methyltransferase putative expressed
OsISM03926	LOC_Os01g29409	16492310	TCCAAAGCAGAAGATCCTGAA	ATGCATTTCAACATCGGACA	59.9	181	82	tRNA methyltransferase putative expressed
OsISM03927	LOC_Os01g29450	16503638	CATACCCCCGTGGTTTTAGA	CTTGACCGATGTCAGGTGTG	59.9	290	90	retrotransposon protein putative unclassified
OsISM03928	LOC_Os01g29450	16504020	TGCTGAACTAGCACATTCTGG	CTTCATTGATGGCCGATTG	59.6	515	99	retrotransposon protein putative unclassified
OsISM03929	LOC_Os01g29450	16506204	TGGGAGCAAAACTCTCCCTA	TCAATAGCCTCCCAAATATCG	59.4	429	88	retrotransposon protein putative unclassified
OsISM03930	LOC_Os01g29450	16507563	AGCGCCGCAGTGTAAGTTAG	GAATCGGCTTTTGCAACATA	59.7	479	78	retrotransposon protein putative unclassified
OsISM03931	LOC_Os01g29450	16508566	CAAGCATCCGGTTATCGAGT	TGTACTTCACCCATAGCAACCTT	60.0	274	87	retrotransposon protein putative unclassified
OsISM03932	LOC_Os01g29450	16508810	CCAAAGTAAGGTTGCTATGGG T	GGTTTGTGGTATGCCGAATC	59.8	412	68	retrotransposon protein putative unclassified
OsISM03933	LOC_Os01g29488	16523924	TGAGGAATTGTGTTCATGCC	AATCTGCCGAAAACTGAGGA	59.7	226	79	retrotransposon protein putative unclassified
OsISM03934	LOC_Os01g29488	16524677	CAATCATGAGCAACGAATGG	TCCATGATGTGAGCAATGGT	60.0	177	70	retrotransposon protein putative unclassified
OsISM03935	LOC_Os01g29488	16524833	AACCATTGCTCACATCATGG	CTGTGGGTTGTTGATCTTGC	59.3	614	93	retrotransposon protein putative unclassified
OsISM03936	LOC_Os01g29488	16525427	GCAAGATCAACAACCCACAG	TGCGTGGAGAAGTCGTCATA	59.8	1653	91	retrotransposon protein putative unclassified
OsISM03937	LOC_Os01g29488	16527060	TATGACGACTTCTCCACGCA	TTGCTCTTCTCCTCATGGTG	59.7	413	64	retrotransposon protein putative unclassified
OsISM03938	LOC_Os01g29488	16527789	TGTGCTGATTTTAAGCATGTTG	TCCGAGACCTTTCCATCAAG	59.5	570	89	retrotransposon protein putative unclassified
OsISM03939	LOC_Os01g29488	16528454	TGGGCATGTAACTTCGTCTG	CTCCGTGGGTTGTTGATCTT	59.8	1851	91	retrotransposon protein putative unclassified
OsISM03940	LOC_Os01g29488	16530284	CAAGATCAACAACCCACGG	GACACTCGAGGCTGTTGTTG	59.7	151	69	retrotransposon protein putative unclassified

ISM IDs	Rice gene locus IDs	Physical positions (bp) on chromosomes	Forward primers (5'-3')	Reverse primers (5'-3')	Melting tempearature (T˚C)	Expected product size (bp) in PCR	Expected product size (bp) in Q-PCR	Putative Functions
OsISM03941	LOC_Os01g29488	16530653	ACATTGACGTCCTAATCGGG	CACTTCCTCCGCAAATTCAT	59.9	167	99	retrotransposon protein putative unclassified
OsISM03942	LOC_Os01g29488	16531235	TGAAACTCTTGTGTGCCTGG	GCGCCAGCTGAGTCTCTATC	60.1	273	85	retrotransposon protein putative unclassified
OsISM03943	LOC_Os01g29507	16539554	GTTCGGGAAGGAATGAGGAG	GTCATCTGGCCCAAAGAAAC	60.2	151	93	retrotransposon putative centromere-specific
OsISM03944	LOC_Os01g29507	16539683	CTGTTTCTTTGGGCCAGATG	GACTCATGGCGAGCATTCTT	60.5	991	47	retrotransposon putative centromere-specific
OsISM03945	LOC_Os01g29507	16540765	ATCGGCACCAAGAACTATCG	CGGATCCCTTGAATAGACGA	60.1	2018	74	retrotransposon putative centromere-specific
OsISM03946	LOC_Os01g29507	16542888	ACCTTGTCAGTGATCGGGAG	TCCTTATGGAAAAACAAAACGA A	60.0	242	89	retrotransposon putative centromere-specific
OsISM03947	LOC_Os01g29507	16543573	TGCAAGGGCATATTCTGAAA	CACCTGCACGAGGTTTTTCT	59.8	159	64	retrotransposon putative centromere-specific
OsISM03948	LOC_Os01g29507	16544028	AGGAGAATGGACACCCTTGA	GTCCTTGATGCGGATTGAA	59.6	190	91	retrotransposon putative centromere-specific
OsISM03949	LOC_Os01g29507	16544211	CAAGGACCCTGAGCGAAAG	GAGCACTTACCAAGAACCCA	59.4	301	99	retrotransposon putative centromere-specific
OsISM03950	LOC_Os01g29507	16544450	TTGAAATGATGAATAGTTGCT CTG	TGTGCATATATGTGGGTGTATGA	58.6	412	99	retrotransposon putative centromere-specific
OsISM03951	LOC_Os01g29580	16575562	AAGGGGTCCTCCCCATCT	ACCGGCTGGACTTGGAAT	60.6	549	97	retrotransposon protein putative unclassified
OsISM03953	LOC_Os01g29610	16589496	CACGACATCCGAAAACACAG	ACAACCAGCTATGGTGGAGG	60.1	515	84	hypothetical protein
OsISM03954	LOC_Os01g29610	16590025	CCATGCCATGACATCTGAAA	CATCATACCACACCATCCTCA	59.9	546	99	hypothetical protein
OsISM03955	LOC_Os01g29610	16590495	TTCATGCAGAGGTTTCAGTCA	TGGTATGCAATCCAAAGGTG	59.4	141	92	hypothetical protein
OsISM03956	LOC_Os01g29610	16590640	TTTCCATGTTCTTCAGCTCTCA	CGTCGCAATCTGTCAAATTCT	60.1	374	90	hypothetical protein
OsISM03957	LOC_Os01g29610	16590958	GAGGAGAAAGGGAAGTGAGG A	CCTGGACTCCTGAGAAACCA	60.0	867	68	hypothetical protein
OsISM03958	LOC_Os01g29610	16591859	TCGTCCTGAGCTTCCAGATT	CCGGATACACAATATCCTCATGT	60.0	1724	94	hypothetical protein
OsISM03959	LOC_Os01g29610	16593558	ATACATGAGGATATTGTGTAT CCG	TCCTTGACTGTTTCAAGGCTG	58.7	705	90	hypothetical protein

ISM IDs	Rice gene locus IDs	Physical positions (bp) on chromosomes	Forward primers (5'-3')	Reverse primers (5'-3')	Melting tempearature (T°C)	Expected product size (bp) in PCR	Expected product size (bp) in Q-PCR	Putative Functions
OsISM03960	LOC_Os01g29610	16594271	AAGCCATGGTGAGTGATGCT	TTTAGCCCACAGTGCACAAC	60.2	607	98	hypothetical protein
OsISM03961	LOC_Os01g29610	16594858	GTTGTGCACTGTGGGCTAAA	AAAGGGCTCGACTTTCCAAT	59.9	1514	83	hypothetical protein
OsISM03963	LOC_Os01g29710	16648617	AGAACGATCGAACGAACGAG	ATATCTGTTCCTCATCGCGG	60.2	1054	85	transposon protein putative CACTA En/Spm sub-class
OsISM03964	LOC_Os01g29710	16650885	ATTCCTCCTGCAATGGTGAG	GTACCTGAAGGGTCCGTTGG	60.9	297	99	transposon protein putative CACTA En/Spm sub-class
OsISM03965	LOC_Os01g29710	16652205	CGCCCTCGACGTCTCTATTA	TCCAGTATCGAAAACCCTCC	59.7	191	80	transposon protein putative CACTA En/Spm sub-class
OsISM03966	LOC_Os01g29710	16652477	AAGGCGCAGCATTACAAGAC	GCCTCCAGGTCGATGAGTAA	60.3	179	81	transposon protein putative CACTA En/Spm sub-class
OsISM03967	LOC_Os01g29710	16652653	GGCCTACACCGTCAACGTAT	AATGACGGAACCGATACCAA	60.0	185	99	transposon protein putative CACTA En/Spm sub-class
OsISM03968	LOC_Os01g29710	16652845	CGGCAAATGGAGAGAAAGAC	GGCAATACTCGCACACGTAA	59.8	195	99	transposon protein putative CACTA En/Spm sub-class
OsISM03969	LOC_Os01g29710	16653040	ACTGCCTTGCAGACCAAATC	CCGCGATAAATTCCTTGTGT	60.1	169	88	transposon protein putative CACTA En/Spm sub-class
OsISM03970	LOC_Os01g29720	16655036	CGACCGATCTATCCCAATCA	TGAACTAATGTTCCCTTGCG	59.8	617	65	hypothetical protein
OsISM03971	LOC_Os01g29720	16655609	TTCTTGAGCTCATTCACGGA	GCCATCACCTTGGTCTCATT	59.7	186	87	hypothetical protein
OsISM03972	LOC_Os01g29740	16658964	CTCTCCGCCCTCCTCTGA	CAGAACTGCAAAGAATATGCG	60.1	2865	98	expressed protein
OsISM03974	LOC_Os01g29804	16699019	AATGCACGAACTGACCATCA	GGTGCTAATGCAATCTGGCT	60.2	248	94	expressed protein
OsISM03975	LOC_Os01g29830	16716232	GCATGCAGCAAGACAACATC	TCACATGGTGGGCTCTACAA	60.3	506	75	expressed protein
OsISM03976	LOC_Os01g29830	16716899	CCTCTTGCAATGAAGATGAAA G	ATTGTTGCCTTGGCTACTGG	59.6	177	89	expressed protein
OsISM03977	LOC_Os01g29850	16726419	ACTAGCCGGGCCAACATACT	GGATCCAAGAAGCCAATAGGT	60.2	419	97	hypothetical protein
OsISM03978	LOC_Os01g29850	16726979	ACGAGCCTTCTTGCACTTTC	TTCCGTCTTTAGGGTGGATG	59.8	178	96	hypothetical protein
OsISM03979	LOC_Os01g29850	16727173	CCCTCTCGATTACAAACACCA	GGTCTGTTCTCCACCCTTGA	60.0	181	92	hypothetical protein

ISM IDs	Rice gene locus IDs	Physical positions (bp) on chromosomes	Forward primers (5'-3')	Reverse primers (5'-3')	Melting tempearature (T˚C)	Expected product size (bp) in PCR	Expected product size (bp) in Q-PCR	Putative Functions
OsISM03980	LOC_Os01g29850	16727334	TCAAGGGTGGAGAACAGACC	ACGCGTAATACTCGCAGAGG	60.3	188	85	hypothetical protein
OsISM03981	LOC_Os01g29850	16727508	CGAGTATTACGCGTGGGAAT	TCAAAGCTTGTTCGACGTTC	59.5	170	91	hypothetical protein
OsISM03982	LOC_Os01g29870	16731020	TTGTCACATTCCTGAGCAGC	GAACGTTGGAGCTGTACGTG	59.7	162	82	multidrug resistance protein 9 putative expressed
OsISM03983	LOC_Os01g29870	16731321	CATATTCGTCGTGCTCATCG	GTATACTGCAGCCTGGCCTC	60.1	819	86	multidrug resistance protein 9 putative expressed
OsISM03985	LOC_Os01g29900	16765438	GATTGATTTGCCTGCAGATTT	AGCAACCGATACACCGTCTC	59.7	912	98	retrotransposon protein putative unclassified
OsISM03986	LOC_Os01g29910	16832271	GCATGGAAGGTGGTGGGA	TTGCTTGCAGGAATAGGGTT	61.3	754	43	retrotransposon protein putative unclassified
OsISM03987	LOC_Os01g29910	16832994	CGGTGCAGATCAACCCTATT	AACGGCACTTTGATGTGGAT	60.2	2622	95	retrotransposon protein putative unclassified
OsISM03988	LOC_Os01g29910	16836136	GAGTTTCAACCAAGGACGGA	TCGTTGTTTCCTTCTCCAGG	60.2	148	56	retrotransposon protein putative unclassified
OsISM03989	LOC_Os01g29910	16836330	GGCCAAGAAGTCAGATCAGC	GGAAGGGATGTCCTCATCATT	60.1	880	98	retrotransposon protein putative unclassified
OsISM03990	LOC_Os01g29910	16837226	CTATTGCACAGCAAGGACCA	TCCACGTTGTTGCTGTTCTT	59.6	692	98	retrotransposon protein putative unclassified
OsISM03991	LOC_Os01g29910	16838036	CGTTGCTGCATCAAGTGGTA	ACAACAAGCACGATCCTGTG	60.3	971	92	retrotransposon protein putative unclassified
OsISM03992	LOC_Os01g29910	16839031	TTGCTGTTCTTGCTGTTTGC	GCGACCCTAACATCCTCATC	59.8	274	89	retrotransposon protein putative unclassified
OsISM03993	LOC_Os01g29910	16839556	TCGTCCACAAAGACAACAGC	TTTGGATTGGAACGAACAAA	59.4	279	83	retrotransposon protein putative unclassified
OsISM03994	LOC_Os01g29910	16840770	CACATCCGCAGCCCTACTAT	AGCCGCAATAAGATGCTCAC	60.3	338	78	retrotransposon protein putative unclassified
OsISM03995	LOC_Os01g29910	16841517	CTATTGCACAGCAAGGACCA	TCCACGTTGTTGCTGTTCTT	59.6	692	98	retrotransposon protein putative unclassified
OsISM03996	LOC_Os01g29910	16842154	ATTGCTTGCAGGTTCAAGGT	TTCGATTTGGCGAAGGATAC	59.9	138	93	retrotransposon protein putative unclassified
OsISM03997	LOC_Os01g30950	16888069	CGCCAAATCTACATACGTGC	CTCCGTGGGTTGTTGATCTT	59.6	514	75	retrotransposon protein putative unclassified
OsISM03998	LOC_Os01g30956	16908546	TACAACCACGCCTATTGCAC	TTGCGATTTGGTTCCTCTTC	59.9	177	92	retrotransposon protein putative unclassified expressed

ISM IDs	Rice gene locus IDs	Physical positions (bp) on chromosomes	Forward primers (5'-3')	Reverse primers (5'-3')	Melting tempearature (T˚C)	Expected product size (bp) in PCR	Expected product size (bp) in Q-PCR	Putative Functions
OsISM03999	LOC_Os01g30962	16915280	CTGCATACGACCTCCGTTTT	ATCGATACACCTCTCCGTGG	60.0	519	79	retrotransposon protein putative unclassified
OsISM04000	LOC_Os01g30970	16927923	CTCATCTTTCCGCTACTGGC	GGCTCCTCTCCAGCATATCA	60.2	215	81	PMR5 putative expressed
OsISM04001	LOC_Os01g31000	16953378	TGGCGTTTTTGAACTTTTTG	AACGGCACTTTGATGTGGAT	59.6	1517	77	retrotransposon protein putative unclassified
OsISM04002	LOC_Os01g31000	16955415	GAGTTTCAACCAAGGACGGA	TCGTTGTTTCCTTCTCCAGG	60.2	148	56	retrotransposon protein putative unclassified
OsISM04003	LOC_Os01g31020	16960277	GTTACAAGCCCAAAGGACCA	AGGTCAAGGCCTCCAAATG	60.0	233	66	retrotransposon protein putative unclassified
OsISM04004	LOC_Os01g31020	16960570	CATCACAAAGGCTAGTGGGC	AGTTGAGACAATCGCCTCCT	59.8	301	49	retrotransposon protein putative unclassified
OsISM04005	LOC_Os01g31020	16961435	TGCTGTGTCACCGTATTTGG	TTTACAACGGCCTCCGATAC	60.3	477	91	retrotransposon protein putative unclassified
OsISM04006	LOC_Os01g31020	16962004	CGTTGGAGACACCTTCCAAT	ACCCCCTCATCAGATAACCC	60.0	397	53	retrotransposon protein putative unclassified
OsISM04007	LOC_Os01g31020	16963099	GCCGTGATGACACAACTGAC	CGGTTGTTGATGACGAAGAA	59.9	124	80	retrotransposon protein putative unclassified
OsISM04008	LOC_Os01g31020	16963286	GAAGCTTGGCTTGACTACACG	GGAACCACATCACAATCAACA	59.7	147	99	retrotransposon protein putative unclassified
OsISM04009	LOC_Os01g31020	16963419	TGTGATGTGGTTCCTATGCAA	TCTGGGTTGGTTCGATAAGC	60.0	535	81	retrotransposon protein putative unclassified
OsISM04010	LOC_Os01g31020	16963934	GCTTATCGAACCAACCCAGA	GAAATCTTTGACGAAACGGC	59.9	729	95	retrotransposon protein putative unclassified
OsISM04011	LOC_Os01g31020	16964768	CACGCACCATTGCTACAACT	CGCTTCCGCTTGTTAAACTC	59.9	173	82	retrotransposon protein putative unclassified
OsISM04012	LOC_Os01g31020	16964872	TTATTCCTTGTCCCAGGAGG	TCTGTTGCAAAGCACTACCG	59.5	1030	86	retrotransposon protein putative unclassified
OsISM04013	LOC_Os01g31020	16965903	GCCCGAGCTTAATCCTGATA	TTCAAAACAGGACCATGCAA	59.7	2134	94	retrotransposon protein putative unclassified
OsISM04014	LOC_Os01g31020	16968017	TTGCATGGTCCTGTTTTGAA	CAAAATGTCCCATCAACCCT	59.9	502	86	retrotransposon protein putative unclassified
OsISM04015	LOC_Os01g31020	16968499	AGGGTTGATGGGACATTTTG	GAGCACGAGTGTTGAAACCA	59.8	1517	60	retrotransposon protein putative unclassified
OsISM04016	LOC_Os01g31020	16970410	AATTCAAGAGGGGGAGGATG	TATGTTCCACCTGCACGAGA	60.3	1816	94	retrotransposon protein putative unclassified

ISM IDs	Rice gene locus IDs	Physical positions (bp) on chromosomes	Forward primers (5'-3')	Reverse primers (5'-3')	Melting tempearature (T˚C)	Expected product size (bp) in PCR	Expected product size (bp) in Q-PCR	Putative Functions
OsISM04017	LOC_Os01g31020	16972424	GACAATCAACCTCCCAGATCA	TCAGCAGATTGTTGCCATTC	59.9	185	91	retrotransposon protein putative unclassified
OsISM04018	LOC_Os01g31030	16973854	CGCCAAATCTACATACGTGC	ATCGATACACCTCTCCGTGG	59.6	526	87	retrotransposon putative centromere-specific
OsISM04019	LOC_Os01g31040	16977466	CGCCAAATCTACATACGTGC	ATCGATACACCTCTCCGTGG	59.6	526	87	retrotransposon protein putative unclassified expressed
OsISM04020	LOC_Os01g31050	16981164	ATTGTGTCTAGAGGTGGCCG	CCCTCAAACCACTCCAAACT	59.6	674	90	hypothetical protein
OsISM04021	LOC_Os01g31070	16986681	AGCAACTTATGCAACAGCCC	TTTCTGGTTATTCCGCAAGG	60.2	214	82	retrotransposon protein putative Ty3-gypsy subclass
OsISM04022	LOC_Os01g31070	16986841	TGCAAGATAAGGTCTTTCCGA	ATTTGTGGCCATACAACGCT	60.1	487	77	retrotransposon protein putative Ty3-gypsy subclass
OsISM04023	LOC_Os01g31080	16988469	ATTTCAAAGAGCCGACATGC	TCCTTGCCATCTTCCTCTTG	60.3	304	89	retrotransposon protein putative unclassified
OsISM04024	LOC_Os01g31080	16988780	ATTACATATTTGAGCCGCCG	TGCTTGTTGCGCTAAATCAT	59.7	708	64	retrotransposon protein putative unclassified
OsISM04025	LOC_Os01g31080	16989785	GCCCAGGGAACAGAAGAAG	CCGTAGCAACTAGCATGAACC	59.8	471	92	retrotransposon protein putative unclassified
OsISM04026	LOC_Os01g31080	16990254	GGATTACTTCACCAAGTGGGC	TTACTCGACTCGGCTTGTCC	60.6	240	82	retrotransposon protein putative unclassified
OsISM04027	LOC_Os01g31100	17004716	AGCTTTCAGCAGATGAGGGA	ATCAGGTTGGTTGGCATCAC	60.4	200	81	retrotransposon putative centromere-specific
OsISM04028	LOC_Os01g31110	17012223	GTCAAGGCGACCCTCAAGAC	TGCGAATGAATGTTCTCCAA	61.2	703	84	CRS2-associated factor 1 chloroplast precursor putative expressed
OsISM04029	LOC_Os01g31110	17012960	AAAGGTGTATGCACGGTTGA	CACACCACCCTGATGATGAA	59.7	352	88	CRS2-associated factor 1 chloroplast precursor putative expressed
OsISM04030	LOC_Os01g31110	17013456	CGCAAGAGAGGACGTCAGTT	TGCTTCTCGAACTTGCTTCA	60.2	470	85	CRS2-associated factor 1 chloroplast precursor putative expressed
OsISM04031	LOC_Os01g31110	17013938	GATCTTGTCCGGGTTGATTG	TCAAAAGACAGTAAGGTACAGGGA	59.8	173	99	CRS2-associated factor 1 chloroplast precursor putative expressed
OsISM04032	LOC_Os01g31124	17023878	GACGGAGGAAAGCGGTCT	CTTCACTGCGTCGTCTCGTA	60.3	238	96	hypothetical protein
OsISM04033	LOC_Os01g31150	17034972	TGTGAGGCCACCTACCTTTT	CCCACCATTCTGAAGCAGAT	59.8	142	96	retrotransposon protein putative unclassified
OsISM04035	LOC_Os01g31240	17081841	TTCCATCCAAGTCATCCACA	GGTTGTTAATCTTGCCGGTG	60.1	396	68	retrotransposon protein putative unclassified

ISM IDs	Rice gene locus IDs	Physical positions (bp) on chromosomes	Forward primers (5'-3')	Reverse primers (5'-3')	Melting tempearature (T°C)	Expected product size (bp) in PCR	Expected product size (bp) in Q-PCR	Putative Functions
OsISM04036	LOC_Os01g31240	17082220	CGGCAAGATTAACAACCCAC	CAATTTCGTAGGTCCCGATG	60.3	448	99	retrotransposon protein putative unclassified
OsISM04037	LOC_Os01g31240	17082718	TCGGTTAGTTTTGAAGTTGCG	GCAGGTGCTTCATCCTCTTT	59.9	1832	93	retrotransposon protein putative unclassified
OsISM04038	LOC_Os01g31300	17120090	AGGAGGGATCAACATGACCA	TTTGTTAAGCCCACATAAAAACC	59.8	452	94	retrotransposon protein putative unclassified
OsISM04039	LOC_Os01g31300	17120665	ACACTCAAAGTCGAGGCACC	CTCCGTGGGTTGTTGATCTT	60.1	1303	87	retrotransposon protein putative unclassified
OsISM04040	LOC_Os01g31310	17134780	CAACGCTTTAGGCACGGT	CCTCGCAGGTGTCCTCTCT	60.2	138	75	hypothetical protein
OsISM04042	LOC_Os01g31350	17146023	AGACGTGCTCTCCTTGAGAAA	GCTCTGTTGGGTTCTCCAAA	59.7	176	98	retrotransposon protein putative unclassified
OsISM04043	LOC_Os01g31350	17146311	GGAGAAGGCTGATGCACCTA	TGGCACAACAACAGAGGGT	60.3	544	98	retrotransposon protein putative unclassified
OsISM04044	LOC_Os01g31350	17146902	AAGTTTGTTTTGCTTTTGTCCA	ATCTTCCATATGCAGGCCAA	59.6	354	65	retrotransposon protein putative unclassified
OsISM04045	LOC_Os01g31350	17147236	TTGGCCTGCATATGGAAGAT	CGATACACCTCTTCGTGGGT	60.2	626	87	retrotransposon protein putative unclassified
OsISM04046	LOC_Os01g31350	17147810	AATAGGGTTGATCTGCACCG	AGGTGCCACTGATCAAGACC	60.0	316	99	retrotransposon protein putative unclassified
OsISM04047	LOC_Os01g31350	17148172	CGAGAAAGCTGCTGCCAT	ATGAGCGGATGAGATCTTGG	60.2	196	80	retrotransposon protein putative unclassified
OsISM04048	LOC_Os01g31350	17148427	CGGTTCTCGGCAGAAGTC	TTGGAGGAGGAGCCAGACTA	59.7	136	94	retrotransposon protein putative unclassified
OsISM04049	LOC_Os01g31350	17148494	AAAGATACCGGAATGGGGTC	TCAGCTGCCATGGAGTAAAC	59.4	215	88	retrotransposon protein putative unclassified
OsISM04050	LOC_Os01g31350	17148994	CCATAGTTTTCCAAGGCTGC	CAAGCGTACCCAGAATCCAC	60.1	165	58	retrotransposon protein putative unclassified
OsISM04051	LOC_Os01g31350	17149386	CACAACTTCAGGCTCGACAG	GCTTTTCCTCCTTGCTCAGA	59.7	494	85	retrotransposon protein putative unclassified
OsISM04052	LOC_Os01g31410	17173547	GCAACAGTACATCCAACCCC	CCTTGCTCACGGCACCTT	61.3	533	84	retrotransposon protein putative unclassified
OsISM04053	LOC_Os01g31420	17180892	CTCAGTGTGGTGAAGCATCG	CTCCCTAATCGGAGCAATCA	60.3	335	60	retrotransposon protein putative Ty3-gypsy subclass
OsISM04054	LOC_Os01g31420	17181269	CAGATGGTAACCTTGCACGA	TTTACTCCTCACCCATTCGG	59.8	144	97	retrotransposon protein putative Ty3-gypsy subclass

ISM IDs	Rice gene locus IDs	Physical positions (bp) on chromosomes	Forward primers (5'-3')	Reverse primers (5'-3')	Melting tempearature (T°C)	Expected product size (bp) in PCR	Expected product size (bp) in Q-PCR	Putative Functions
OsISM04055	LOC_Os01g31420	17181654	TTGAGGGAGGCAAGATTAGG	ATTCACCTGAAAACCCTTCG	59.1	134	99	retrotransposon protein putative Ty3-gypsy subclass
OsISM04056	LOC_Os01g31420	17181860	GATGGTTTTGATCCTCATTGG	ACAGGTTCAAGCATATCGGC	59.7	598	81	retrotransposon protein putative Ty3-gypsy subclass
OsISM04057	LOC_Os01g31420	17182981	CAGGAGATGATCGGCAAGAT	GTATTTGACAACGTCGGCCT	60.1	435	97	retrotransposon protein putative Ty3-gypsy subclass
OsISM04058	LOC_Os01g31420	17183560	GGCTCAGTTGAAATAGTGCCA	CGAAGATCAAACTCGGTTAAGG	60.2	303	93	retrotransposon protein putative Ty3-gypsy subclass
OsISM04059	LOC_Os01g31440	17192417	ACATTGACATCTTCGCCTGG	TTGAGCAGCTTTTGCACTTC	60.2	144	71	retrotransposon protein putative unclassified
OsISM04060	LOC_Os01g31480	17222626	GGACAAAGTTGGTGGGATTG	TTGGGTTGAGACCCTGAAAC	60.1	198	99	conserved hypothetical protein
OsISM04061	LOC_Os01g31480	17223039	TGATACAACCGATGCTGCTC	TTCACCTTCTCTGCCCACTT	59.8	861	94	conserved hypothetical protein
OsISM04063	LOC_Os01g31550	17257063	TTGAAGAGGTTCAACATGCAA	GCTGATTGGTATCGGCTTGT	59.7	202	75	retrotransposon protein putative Ty1-copia subclass
OsISM04065	LOC_Os01g31650	17325923	CGCTGTGACCCTTCACTTTC	ACCCCGGACCAGATAAAAAG	60.5	940	70	retrotransposon protein putative Ty3-gypsy subclass
OsISM04066	LOC_Os01g31690	17348587	GCCACCTCAGCTCTGCTC	TCTCGTCGAAGGTAAGCCTC	59.7	187	68	oxygen-evolving enhancer protein 1 chloroplast precursor putative expressed
OsISM04067	LOC_Os01g31714	17358721	GCGTTCTTCCAGCAAAAGAG	GTCTCCCATTGGCAGTGAGT	60.1	503	81	retrotransposon protein putative unclassified
OsISM04068	LOC_Os01g31714	17359465	GCTACTCACACTTGCTGCCA	GGATCCGACTTCATCAAAGC	59.9	168	80	retrotransposon protein putative unclassified
OsISM04069	LOC_Os01g31714	17359852	GAAGAAAACATCCAATCGCC	CAGAGTCTTTCCCAGACGGA	59.9	162	84	retrotransposon protein putative unclassified
OsISM04070	LOC_Os01g31714	17360063	TGGGGATCATCTGAGTGACA	ACGGGGGATTTCTTCTTCAT	59.9	384	95	retrotransposon protein putative unclassified
OsISM04071	LOC_Os01g31714	17360564	AAAAAGGCACTCAACCAAGC	GCTCAGCCTTGTCATTTTCC	59.6	430	86	retrotransposon protein putative unclassified
OsISM04072	LOC_Os01g31714	17360993	CTGGCAAGACAGAGGGATTC	TGTGCTTGAGACTTGATCTTGC	60.2	138	63	retrotransposon protein putative unclassified
OsISM04073	LOC_Os01g31740	17369633	CTGCATACGACCTCCGTTTT	ATCGATACACCTCTCCGTGG	60.0	518	79	retrotransposon protein putative unclassified
OsISM04074	LOC_Os01g31750	17376573	CAATGCTTAGCACGGTTCCT	TTCGATCCCTGGCTAGTTTG	60.2	204	88	transposon protein putative unclassified

ISM IDs	Rice gene locus IDs	Physical positions (bp) on chromosomes	Forward primers (5'-3')	Reverse primers (5'-3')	Melting tempearature (T°C)	Expected product size (bp) in PCR	Expected product size (bp) in Q-PCR	Putative Functions
OsISM04075	LOC_Os01g31750	17377596	GCTGCAAATGGAGGATTCAT	GGTTTAACTTCTTGTATGGCTGC	59.6	159	83	transposon protein putative unclassified
OsISM04076	LOC_Os01g31750	17378462	GCAAAGAGGGTGATCCAGAG	TCGTTCTCTTCTTTGGTGGC	60.1	314	67	transposon protein putative unclassified
OsISM04077	LOC_Os01g31750	17378809	TCATGGCTGCTCCAATAGTT	TGTCCTATTGTCCTCGGTGA	58.7	154	78	transposon protein putative unclassified
OsISM04078	LOC_Os01g31750	17378943	TCACCGAGGACAATAGGACA	GGTTGGTTGGAGCCAGTAAG	59.3	163	83	transposon protein putative unclassified
OsISM04079	LOC_Os01g31760	17383912	CCATCTTCGTCCGAGCAA	AGGGCATGGTTTCCCTTACT	60.4	1710	77	acyl-ACP thioesterase putative
OsISM04080	LOC_Os01g31760	17385556	GATATACAGGCGGTTTTCGC	ACAGCCTCCTGGTGACCTTA	59.6	197	72	acyl-ACP thioesterase putative
OsISM04081	LOC_Os01g31760	17385845	CTCGACACCAATGCCACATA	TGACGTTGTTGACATGATGG	59.7	305	83	acyl-ACP thioesterase putative
OsISM04082	LOC_Os01g31760	17386130	CCATCATGTCAACAACGTCA	TGCTACTAAGCTGGTGCTGC	59.2	2122	84	acyl-ACP thioesterase putative
OsISM04083	LOC_Os01g31770	17393711	ACTATTGGCCCCATGGTGAG	TGTCAGCTATGCTGAAACGC	61.1	3358	99	oxidoreductase 2OG-Fe oxygenase family protein putative expressed
OsISM04084	LOC_Os01g31770	17396988	TTATTGCAGGTTCAAAGGCA	GGGCGCATTTTGTATAGCTC	59.5	1113	87	oxidoreductase 2OG-Fe oxygenase family protein putative expressed
OsISM04085	LOC_Os01g31770	17398174	ACTGAGCCAGTTCCTGGTGT	TGGACCCATTTTTCGAAGTT	59.6	189	96	oxidoreductase 2OG-Fe oxygenase family protein putative expressed
OsISM04086	LOC_Os01g31770	17398426	TTTTGGCCTGGAAGTTATGC	CCTCCACCAACCTCAGGATA	60.0	186	85	oxidoreductase 2OG-Fe oxygenase family protein putative expressed
OsISM04087	LOC_Os01g31770	17398649	GGGAAAGACAGGGATGTTGA	ACCACCAGAGAATTGCTTGC	60.1	1497	88	oxidoreductase 2OG-Fe oxygenase family protein putative expressed
OsISM04088	LOC_Os01g31770	17400151	ATACTTTCGTGGCATCCGTT	AGCTCGTCCAGGAACATGAG	59.9	338	89	oxidoreductase 2OG-Fe oxygenase family protein putative expressed
OsISM04089	LOC_Os01g31770	17400532	CATCTGGGCTCAGAATCAATC	GCCCGAAAAGTCCTTCTGAT	60.1	2330	84	oxidoreductase 2OG-Fe oxygenase family protein putative expressed
OsISM04090	LOC_Os01g31770	17402892	GAGAGGCAGATTCATGCTGTT	ATTTAAATCGTTGCCCCTCC	59.8	179	72	oxidoreductase 2OG-Fe oxygenase family protein putative expressed
OsISM04091	LOC_Os01g31780	17412654	CAAGGAAACAGCAGGAGCAC	GTCAAGAAGCCGGACCATT	60.5	108	51	hypothetical protein
OsISM04092	LOC_Os01g31800	17419686	GTCCTCGAGTACCTCGCC	TTCTTCTTGTTGTCCCTGGC	59.3	179	66	Core histone H2A/H2B/H3/H4 domain containing protein putative expressed

ISM IDs	Rice gene locus IDs	Physical positions (bp) on chromosomes	Forward primers (5'-3')	Reverse primers (5'-3')	Melting tempearature (T˚C)	Expected product size (bp) in PCR	Expected product size (bp) in Q-PCR	Putative Functions
OsISM04094	LOC_Os01g31860	17452180	TGGTGTTCCCTCCGATGTAT	CAAAACAAGGTTTTCACCCG	60.3	177	83	hypothetical protein
OsISM04096	LOC_Os01g31920	17481602	GCTTCGTCGAGCAAGTCC	AAACGGTTCACATGCCTTTC	59.8	1108	63	hypothetical protein
OsISM04097	LOC_Os01g31920	17482691	AAAGGCATGTGAACCGTTTG	ACACTCAGACTCGGACCTGC	60.7	1292	98	hypothetical protein
OsISM04098	LOC_Os01g31920	17483895	CGTGTGTTGGCAGGTCAG	GACCAAGAACCGGGTTAGAC	59.2	840	99	hypothetical protein
OsISM04099	LOC_Os01g31920	17484764	GATCTGGTTTCGCCTGTTGT	GCGCTAAGAAAGTACCACGC	60.1	148	65	hypothetical protein
OsISM04100	LOC_Os01g31930	17487108	TCATCGTCCCCGTATTTGAC	GTGGTGGCAGTAGAGCCTTC	60.3	341	88	hypothetical protein
OsISM04101	LOC_Os01g31970	17500240	GGCATTCTTGCCCTTGTG	CCGTGGTATTGTTAGAGATTTGG	60.0	211	98	hypothetical protein
OsISM04102	LOC_Os01g31970	17500391	TTTGTTCGAGAAAAACAATTGAA	CCAAACCACGAAGGTACTCA	58.7	829	97	hypothetical protein
OsISM04104	LOC_Os01g32020	17531685	GCCCTTCTGGAAGATGAGGT	TTCGATCATGTCCGAGAAAA	59.9	819	70	retrotransposon putative centromere-specific
OsISM04105	LOC_Os01g32030	17534523	TCATCGACACCTCTTTGCAC	GGAGTTTGCACTCCCTGGAT	60.6	981	95	retrotransposon putative centromere-specific
OsISM04106	LOC_Os01g32030	17535705	GAAACTCCGCAAGGAGAACA	TTCTACGAATGCACGCTTGA	60.5	135	77	retrotransposon putative centromere-specific
OsISM04107	LOC_Os01g32044	17539001	CAACTTTGACGTGGGAACG	CTCCACTTCGACCACTTGAA	59.5	148	99	retrotransposon protein putative unclassified
OsISM04108	LOC_Os01g32044	17539321	GGAGGGAGAAGCCAAAGATG	TGAACAGACGAGACAATCGC	60.5	722	72	retrotransposon protein putative unclassified
OsISM04109	LOC_Os01g32044	17540529	GTGCTCCAATTTGCAAGAAA	TTCTAACCATGGCAAACATGA	59.0	152	79	retrotransposon protein putative unclassified
OsISM04110	LOC_Os01g32060	17547712	AGTTTCCGGAGATCTGTTGC	GGACATGGGAGTGAGTCCA	59.4	348	66	retrotransposon protein putative unclassified
OsISM04111	LOC_Os01g32060	17548444	ATAACGGGTACCGTCCACAA	GCCGGGTTTCTATCATTTTG	59.8	258	92	retrotransposon protein putative unclassified
OsISM04112	LOC_Os01g32080	17561297	GGATTCAGTGCAATGGAGGT	CAACAGGCAGCTGGTACCTT	60.1	1981	50	thiamine pyrophosphate enzyme C-terminal TPP binding domain containing protein expressed
OsISM04113	LOC_Os01g32130	17582922	AGCTAGAGCCAGTGGTCGAA	ATCAACATCGTTACCGGAGC	60.1	173	69	expressed protein

ISM IDs	Rice gene locus IDs	Physical positions (bp) on chromosomes	Forward primers (5'-3')	Reverse primers (5'-3')	Melting tempearature (T˚C)	Expected product size (bp) in PCR	Expected product size (bp) in Q-PCR	Putative Functions
OsISM04114	LOC_Os01g32130	17583077	TCCGGTAACGATGTTGATGA	GAGGCTCTCTTAACAGACGAATT T	59.7	1016	99	expressed protein
OsISM04115	LOC_Os01g32130	17584222	AAATGAGGAGATGGTCAGGG	TCTCCTAACAATTTCTTTGATCG AG	59.5	164	67	expressed protein
OsISM04116	LOC_Os01g32140	17588228	GTCGAGGACTCCGACAGC	TTTTGTAATCCTTTATTTTGCATT TG	59.6	910	71	hypothetical protein
OsISM04117	LOC_Os01g32160	17599527	AGAGAGGCGATTCAATGCTG	TTGATCATACATATCCGCCA	58.9	1407	93	hypothetical protein
OsISM04119	LOC_Os01g32200	17628856	AGGAGAGGTGAGGGCAATCT	GCCAGGCCCACTTACATCT	60.2	746	86	hypothetical protein
OsISM04120	LOC_Os01g32230	17646845	AAGAATTTGGAAAGGACGCA	ACACGTCGATGTCGTTACCA	59.9	209	67	transposon protein putative CACTA En/Spm sub-class
OsISM04121	LOC_Os01g32230	17647038	AACGACATCGACGTGTACCT	CGAAGTGCTTGCCTTTCTTC	59.1	313	98	transposon protein putative CACTA En/Spm sub-class
OsISM04122	LOC_Os01g32230	17649110	AGACACCCCTTACTCACGCA	ATATCTGTTCCTCATCGCGG	60.4	1999	69	transposon protein putative CACTA En/Spm sub-class
OsISM04123	LOC_Os01g32230	17652323	ATTCCTCCTGCAATGGTGAG	GTACCTGAAGGGTCCGTTGG	60.9	295	99	transposon protein putative CACTA En/Spm sub-class
OsISM04124	LOC_Os01g32230	17653641	CGCCCTCGACGTCTCTATTA	AGTATTGAAAACCCTCCGCC	60.3	188	77	transposon protein putative CACTA En/Spm sub-class
OsISM04125	LOC_Os01g32230	17653913	AAGGCGCAGCATTACAAGAC	GCCTCCAGGTCGAAGAGTAA	59.9	179	81	transposon protein putative CACTA En/Spm sub-class
OsISM04126	LOC_Os01g32230	17654092	CTGCACCGTCAACGTATATGA	AATGACGGAACCGATACCAA	59.9	182	96	transposon protein putative CACTA En/Spm sub-class
OsISM04127	LOC_Os01g32230	17654244	CATGTAGGGCTTGGTATCGG	CGCAAAAGTTAGTTCCCTGC	60.1	208	64	transposon protein putative CACTA En/Spm sub-class
OsISM04128	LOC_Os01g32230	17654480	CCTTGCAGACCAAATCATCA	CCGCGATAAATTCCTTGTGT	59.8	165	84	transposon protein putative CACTA En/Spm sub-class
OsISM04129	LOC_Os01g32250	17666830	CATCTCCGATGTTTGCTTCA	TTGAGCAATCCAAAACATCA	58.7	449	93	retrotransposon protein putative unclassified
OsISM04130	LOC_Os01g32250	17667415	TTGTGCAAATTTAGAAAATGT CG	TCTCACCACTTCTAGCACCAA	58.9	156	81	retrotransposon protein putative unclassified
OsISM04131	LOC_Os01g32250	17667911	GACTGGTCAGACCGGCTACA	TCCACACGTTCTCCTTCCTT	60.5	278	96	retrotransposon protein putative unclassified
OsISM04132	LOC_Os01g32250	17668289	TTCTACAAGTGCTGTTCTCGCT	TCAACACAGCATCACCACAA	59.5	219	99	retrotransposon protein putative unclassified

ISM IDs	Rice gene locus IDs	Physical positions (bp) on chromosomes	Forward primers (5'-3')	Reverse primers (5'-3')	Melting tempearature (T°C)	Expected product size (bp) in PCR	Expected product size (bp) in Q-PCR	Putative Functions
OsISM04133	LOC_Os01g32250	17668954	TTGGAGCTTGAATTTCCTGG	AAACTACGACCATCCTCCCC	60.2	580	90	retrotransposon protein putative unclassified
OsISM04134	LOC_Os01g32250	17670863	ACATCGTCAAGCAGTCAAGC	ATATCAATGTGCTTCGTGCG	59.4	399	85	retrotransposon protein putative unclassified
OsISM04135	LOC_Os01g32259	17680499	TCGGAGTCAGAGAGACCAGG	GGGTCCATGTAGGGTTGATG	60.3	617	83	expressed protein
OsISM04136	LOC_Os01g32259	17681125	TGAAGGGAGCTCTGCATCTT	ATCACCACCATGCTCATACG	59.7	168	92	expressed protein
OsISM04137	LOC_Os01g32259	17681251	TTTTTCAGGATTGTGCTCCA	TCCGAGCGGAAAGTACAGAT	59.5	794	99	expressed protein
OsISM04138	LOC_Os01g32270	17690868	GTCCATGAAGTTCTGGGCTG	ATGGGTCAACAGGATCTCCA	60.5	631	81	hypothetical protein
OsISM04139	LOC_Os01g32270	17691492	GACCCATTAGAGTGGGACGA	GCCTCGATATGAAACATGGG	60.1	888	58	hypothetical protein
OsISM04140	LOC_Os01g32280	17696411	ATCGAGGACGACTTCAAGGA	TGTTGCCAATATGATGACCAA	59.8	790	94	transmembrane protein putative expressed
OsISM04141	LOC_Os01g32280	17697304	CTTGGTCATCCGAACTTGGT	TCCACCGTTTGTACCTTTCC	59.9	988	87	transmembrane protein putative expressed
OsISM04142	LOC_Os01g32280	17698439	TTGCTTGGCAGTCTGATTGA	ACTGTAGGGCCCCGTTTACT	60.2	6303	99	transmembrane protein putative expressed
OsISM04143	LOC_Os01g32300	17707967	GCCAAGTTCTGCACGCTC	CTTCTGCAGAGACCCAGCTT	60.2	1117	73	expressed protein
OsISM04144	LOC_Os01g32300	17709319	CCCCTCCATTACCGAAAGTT	CAATGTCAATGCGTTCAACC	60.1	1553	88	expressed protein
OsISM04145	LOC_Os01g32300	17710857	AACGCATTGACATTGGTGAA	GGATTTGTGGTAACAGGTTGG	59.6	738	89	expressed protein
OsISM04146	LOC_Os01g32330	17740194	AGCCTGACAGAGACCAAGGA	CTCGCAGTCCATCTTCACCT	60.2	257	85	heavy metal-associated domain containing protein expressed
OsISM04147	LOC_Os01g32350	17745622	ACACCACCAACATGCTCCTT	CAGCAAGAGTTGGCTGATGA	60.3	1400	93	OsClp2 - Putative Clp protease homologue expressed
OsISM04148	LOC_Os01g32350	17747020	TGCTGGATGCTGAGGATAAA	CATGGCATCATAAACTCCCA	59.4	2666	93	OsClp2 - Putative Clp protease homologue expressed
OsISM04149	LOC_Os01g32350	17749778	ATTTTGCATGCCTAATGCG	TCATCTCTCGTATCTGTAATCCC A	59.8	424	93	OsClp2 - Putative Clp protease homologue expressed
OsISM04150	LOC_Os01g32350	17750232	TGTCAAGAATCACCGGGAA	ATGCCATAATCCTTGGCTTC	59.5	179	92	OsClp2 - Putative Clp protease homologue expressed

ISM IDs	Rice gene locus IDs	Physical positions (bp) on chromosomes	Forward primers (5'-3')	Reverse primers (5'-3')	Melting tempearature (T˚C)	Expected product size (bp) in PCR	Expected product size (bp) in Q-PCR	Putative Functions
OsISM04151	LOC_Os01g32364	17755465	CTAGCATTTGGGACGCCTT	TCTGCCGTAGCATTATTTGC	59.6	929	59	Os1bglu1 - beta-mannosidase/glucosidase homologue expressed
OsISM04152	LOC_Os01g32364	17756374	GCAAATAATGCTACGGCAGA	CGCATCGAAACCCATACTTT	59.4	3388	88	Os1bglu1 - beta-mannosidase/glucosidase homologue expressed
OsISM04153	LOC_Os01g32364	17759759	GCGTACCGCTTCTCAATCTC	ATGCCACACCTTTCCAATTT	59.6	196	74	Os1bglu1 - beta-mannosidase/glucosidase homologue expressed
OsISM04154	LOC_Os01g32364	17759935	AAATTGGAAAGGTGTGGCAT	TGATACAAATTGGCATAAGGTG	58.4	212	82	Os1bglu1 - beta-mannosidase/glucosidase homologue expressed
OsISM04155	LOC_Os01g32364	17760158	CAGAGGCACTAGAGGTGCAA	CTGTCGCCAAATGTCTTGAA	59.5	531	98	Os1bglu1 - beta-mannosidase/glucosidase homologue expressed
OsISM04156	LOC_Os01g32364	17760832	TTCTTTCCCATGCTTCTGCT	TTCCAATCTTCCCCTTTTGT	59.2	1499	70	Os1bglu1 - beta-mannosidase/glucosidase homologue expressed
OsISM04157	LOC_Os01g32364	17762387	GCGGCTCAAAGATCAAGAGA	CACTTGCAGCGATTTAGGGT	60.4	311	82	Os1bglu1 - beta-mannosidase/glucosidase homologue expressed
OsISM04158	LOC_Os01g32364	17762816	ACGCCACGACTCTCCCTAGT	AGATGGCGTACCCTTGGTC	60.6	282	92	Os1bglu1 - beta-mannosidase/glucosidase homologue expressed
OsISM04159	LOC_Os01g32364	17763041	TCTTGGTGCTGCAGATGAAC	TGTACAAACCCCACGGTACA	59.9	185	87	Os1bglu1 - beta-mannosidase/glucosidase homologue expressed
OsISM04160	LOC_Os01g32364	17763254	TGGTAACCCCACAATGTTTCT	AATGGTCACATTGCCAGGAT	59.7	151	59	Os1bglu1 - beta-mannosidase/glucosidase homologue expressed
OsISM04161	LOC_Os01g32400	17775444	GAGAGGAGGCCAAGGACAAG	GAAGGTCACCGGAAGCTCTA	60.4	486	87	retrotransposon protein putative unclassified
OsISM04162	LOC_Os01g32430	17788918	GACGAGTTCCGGAGTAAACC	CTCAGGGGCTACACTATCCG	59.2	177	88	hypothetical protein
OsISM04163	LOC_Os01g32490	17816784	ACGAGAACGGCAGTTACCAG	ATGGAATCCCCTCAATCTCC	60.2	731	52	hypothetical protein
OsISM04164	LOC_Os01g32500	17819434	GGAAGCAAGAACGATGTGGT	TCGGCTTGTACGTCAAGAAA	59.8	251	87	retrotransposon protein putative unclassified
OsISM04165	LOC_Os01g32500	17820138	GACAGAACGACAACGGGAAT	AGCCATTTAGATGGTCACGG	60.0	184	87	retrotransposon protein putative unclassified
OsISM04166	LOC_Os01g32500	17821479	AAGGCACTTGCGAAACAAAC	AACCACTTCATCTTGGGTGC	60.1	231	48	retrotransposon protein putative unclassified
OsISM04167	LOC_Os01g32500	17822168	ACATGGTCGAGCATACTCCC	TTGTGCTCAATCACCTCCCT	60.3	265	95	retrotransposon protein putative unclassified
OsISM04168	LOC_Os01g32500	17822847	AACAGTGAAATTTGGCAAGC	AATATTGATGAGATGCGGGC	58.9	1337	61	retrotransposon protein putative unclassified

ISM IDs	Rice gene locus IDs	Physical positions (bp) on chromosomes	Forward primers (5'-3')	Reverse primers (5'-3')	Melting tempearature (T˚C)	Expected product size (bp) in PCR	Expected product size (bp) in Q-PCR	Putative Functions
OsISM04169	LOC_Os01g32500	17824196	GGAGCTAAGCAGATCTCCCA	GTAGGCCTCGACATTGTTCC	59.5	134	47	retrotransposon protein putative unclassified
OsISM04170	LOC_Os01g32530	17840190	CGAGACGACATACGAAGCAA	TTGCAGACAGGACAAGCATC	60.0	136	58	transposon protein putative CACTA En/Spm sub-class
OsISM04171	LOC_Os01g32530	17840446	CCCAAACGAAGCGAAGTTAG	ATCGATATCATTGCCGGGT	60.0	316	80	transposon protein putative CACTA En/Spm sub-class
OsISM04172	LOC_Os01g32530	17841024	TACGTCGCGCATTTAGAAGA	TCCTTGACCATATTGTGGACC	59.6	106	62	transposon protein putative CACTA En/Spm sub-class
OsISM04173	LOC_Os01g32530	17841146	GGGAAGAAAAGAAAACGTAG CA	ACATCAGGCCAACCAAACTC	59.9	160	71	transposon protein putative CACTA En/Spm sub-class
OsISM04174	LOC_Os01g32530	17842049	AAAAGAATTAGGCCCGATCAA	GTTTCATTTGGGACATCCGT	59.8	218	99	transposon protein putative CACTA En/Spm sub-class
OsISM04175	LOC_Os01g32550	17846001	AGGACATTGCTTGGCTAACG	AAGCGATATGCCTCCCTTCT	60.2	259	89	hypothetical protein
OsISM04176	LOC_Os01g32550	17846445	CAATCTTCATGCCAAATCCA	GTCGGCATCACCCTAACTGT	59.7	698	86	hypothetical protein
OsISM04177	LOC_Os01g32550	17847301	TCCTCTCACTTGGAAATGCC	GTCCTCCCCAAAATCCATGT	60.6	206	93	hypothetical protein
OsISM04178	LOC_Os01g32550	17847935	CGACCTCTACAGGCTCCAAG	TTCTTCTGACACCATTGCCA	60.1	159	79	hypothetical protein
OsISM04179	LOC_Os01g32550	17848197	CGCCGAGACAAGGAGTACAT	TGTTATGACCAGAAGGATCCAA	59.9	151	67	hypothetical protein
OsISM04180	LOC_Os01g32550	17848581	AAAGGTGAAGACTTTGGCTGA	CGGAAGATGAAGCTGGCTAT	59.2	184	97	hypothetical protein
OsISM04181	LOC_Os01g32640	17895066	CAGCGGCGAGACTTATCTTT	GTAGCGATCATCTGAAGCGG	60.3	1603	88	OsFBX9 - F-box domain containing protein expressed
OsISM04182	LOC_Os01g32640	17896695	TTGCTGCAGAGAGAACTGATG	TAGCTTGTTGTGCGTCCAGT	59.4	171	88	OsFBX9 - F-box domain containing protein expressed
OsISM04183	LOC_Os01g32640	17896923	CTGAAGCACAAAACCAGTCAG	TTCGAAACTGGGACTTCAGG	59.4	161	68	OsFBX9 - F-box domain containing protein expressed
OsISM04184	LOC_Os01g32660	17904051	AAGGAGCTCAAGCTCTCCG	ATTAAGCCGCAGTTCACCAT	59.7	595	94	STE MEK ste7 MAP2K.2 - STE kinases include homologs to sterile 7 sterile 11 and sterile 20 from yeast expressed
OsISM04185	LOC_Os01g32660	17904634	CTGCGGCTTAATCAAAGAGG	TCCACCTTCAGGTTTGTTGA	59.5	429	78	STE MEK ste7 MAP2K.2 - STE kinases include homologs to sterile 7 sterile 11 and sterile 20 from yeast expressed
OsISM04186	LOC_Os01g32660	17905146	ACAAGTGGGTGGGGACATTA	ATTTGTTTGCGAACTGCCTC	60.2	560	71	STE MEK ste7 MAP2K.2 - STE kinases include homologs to sterile 7 sterile 11 and sterile 20 from yeast expressed

ISM IDs	Rice gene locus IDs	Physical positions (bp) on chromosomes	Forward primers (5'-3')	Reverse primers (5'-3')	Melting tempearature (T˚C)	Expected product size (bp) in PCR	Expected product size (bp) in Q-PCR	Putative Functions
OsISM04187	LOC_Os01g32660	17905853	AATTCTGGAGCCATACCTGG	TGGCTTTATATCCCTGTGAATC	58.5	231	98	STE MEK ste7 MAP2K.2 - STE kinases include homologs to sterile 7 sterile 11 and sterile 20 from yeast expressed
OsISM04188	LOC_Os01g32660	17906163	GTCAGCGGGATACGTTTGTT	AAGCCCAAACTCCATATGTCA	59.7	218	98	STE MEK ste7 MAP2K.2 - STE kinases include homologs to sterile 7 sterile 11 and sterile 20 from yeast expressed
OsISM04189	LOC_Os01g32660	17906500	CGGACCAGTTCTCTCCAGAAT	GGCAATGCTGACCAAGAGTT	60.4	2043	99	STE MEK ste7 MAP2K.2 - STE kinases include homologs to sterile 7 sterile 11 and sterile 20 from yeast expressed
OsISM04190	LOC_Os01g32660	17908484	GCATACAAAAGGATCCTGCG	GAATGCGCAGGTCTAAATCC	60.1	197	97	STE MEK ste7 MAP2K.2 - STE kinases include homologs to sterile 7 sterile 11 and sterile 20 from yeast expressed
OsISM04191	LOC_Os01g32700	17926667	AGTCTGGTCATGACTGGGAA	ACCTCGCAAACACCGTAAAA	59.3	215	98	transposon protein putative unclassified
OsISM04192	LOC_Os01g32700	17926876	CGAGGTACTTGGAGGTGGAA	TTTTCTCTGATGGCTTACCGA	60.0	199	99	transposon protein putative unclassified
OsISM04193	LOC_Os01g32700	17927028	TTCAGATCGGTAAGTCAGTAT CCA	CTCCAACAATTCCTCAACCAA	60.0	731	60	transposon protein putative unclassified
OsISM04194	LOC_Os01g32700	17927864	GAAAGAAGAAATGCCGCAAA	GGGCAGCTAACGGATGATTA	60.2	221	92	transposon protein putative unclassified
OsISM04195	LOC_Os01g32710	17932196	GCCTGAAAAGAACCTGTGGA	TCTTAAGTCATCCTCTGAGGGTC T	60.0	158	77	EDM2 putative
OsISM04196	LOC_Os01g32710	17932384	GAGAAAGACTCGACCTTGGC	CACAGTCCCAACGTACCTCA	59.3	225	92	EDM2 putative
OsISM04197	LOC_Os01g32710	17932549	TGCAAGGCTTCACTCAAGAA	TCATCATCAGCAATGAATGGA	59.9	179	92	EDM2 putative
OsISM04199	LOC_Os01g32720	17934871	GGCGTGTTTTGTGTGGTAAA	TTTTTGCATGGAAGGACCTC	59.8	1332	73	expressed protein
OsISM04200	LOC_Os01g32720	17936233	CACTTGGGTACACTGAGGCA	TGCTTATGCTCACAATTTTTGC	60.0	159	68	expressed protein
OsISM04201	LOC_Os01g32720	17936410	TGGAGCTTTAGAACCTTCAGA TG	TCCTCTGTGTGTTTTGCTGC	60.0	423	66	expressed protein
OsISM04202	LOC_Os01g32720	17936804	GTCGCTACAGCAGCAAAACA	TGGTCTCTGATCGGTGTGTC	59.9	1229	73	expressed protein
OsISM04203	LOC_Os01g32720	17938389	CGAAGATACCCCAAAGTTCA	GAGGCTTCTCTTTCCACCAA	58.5	317	80	expressed protein
OsISM04204	LOC_Os01g32720	17938780	ACAGGATTATTGCGACTGGC	ACATTGCCTCCATTTTCCAG	60.0	167	83	expressed protein
OsISM04205	LOC_Os01g32720	17938970	AACCTGAAGTCTTGAAGCAGC	GCCGACCAAACGAACTGTAG	59.7	1737	97	expressed protein

ISM IDs	Rice gene locus IDs	Physical positions (bp) on chromosomes	Forward primers (5'-3')	Reverse primers (5'-3')	Melting tempearature (T˚C)	Expected product size (bp) in PCR	Expected product size (bp) in Q-PCR	Putative Functions
OsISM04206	LOC_Os01g32720	17940687	CTACAGTTCGTTTGGTCGGC	CAGGTTCCACGTAGCAATGA	60.2	1640	81	expressed protein
OsISM04207	LOC_Os01g32720	17942307	TCATTGCTACGTGGAACCTG	TTTGCACCACAGCAGAAGTC	59.9	656	52	expressed protein
OsISM04208	LOC_Os01g32720	17942992	TTGACAAAGTCCAGAAGAAGTGTC	GGCCGTACAGTCATCCAATC	60.1	343	98	expressed protein
OsISM04209	LOC_Os01g32720	17943333	CCAAAAGAATTGCCTCATGG	TTCGCAAGAGATGCTTTGAC	59.8	155	75	expressed protein
OsISM04210	LOC_Os01g32720	17943535	TGATTGTCCCAAAGGAAACA	GACGGTCTTTACCTTTCCCG	59.9	367	99	expressed protein
OsISM04211	LOC_Os01g32720	17943819	CCATCAGGTTGGATCAAAAGA	CATCAACAGAACCAGGCAAA	59.8	187	97	expressed protein
OsISM04212	LOC_Os01g32740	17955268	CAAAACCGACAACAAGAGCA	AGGAATACCGTGGTTCCCAT	60.2	251	80	hypothetical protein
OsISM04213	LOC_Os01g32840	18021080	GTGGTTCCCCCTTTCCACT	TGTAGGAGGATCAAATGCTGC	60.7	1103	78	expressed protein
OsISM04214	LOC_Os01g32840	18022220	CCCAGACCTTTCTCAGCTTT	GAGTAGTGGGCTGCAGCAAT	59.5	104	52	expressed protein
OsISM04215	LOC_Os01g32840	18022363	CTGAAGCATCACATAGGGCA	TTGCAAGGTGGTAGAAACAGAA	59.8	119	68	expressed protein
OsISM04216	LOC_Os01g32840	18022442	TGCAGTTTCAGTTTCATCTTCTG	CGACAACATGCTTACCAGTGA	59.7	239	99	expressed protein
OsISM04217	LOC_Os01g32840	18022620	GAAAACTAGAGCTTCACATAGACAGA	ACACCTGGCAAAGATGGTTC	58.8	771	90	expressed protein
OsISM04219	LOC_Os01g32870	18041157	CAGGAGATCAAGTCCGCCTA	GAAGAGTTCTGATGCTTCTGGG	60.4	1102	85	heat shock protein DnaJ putative expressed
OsISM04220	LOC_Os01g32870	18042290	CAGATCCTGAGAAACGGAGG	TCCATTCCTTCATTCTCCAA	58.9	807	65	heat shock protein DnaJ putative expressed
OsISM04221	LOC_Os01g32870	18043129	TGAACACAATGTTTGCTGCAC	GGTCTTGATTGGAACACCGA	60.8	142	51	heat shock protein DnaJ putative expressed
OsISM04222	LOC_Os01g32870	18043332	CAGTTGGATCATCAGCAACG	CAATGCCTGACTGAGCTTGT	59.7	164	91	heat shock protein DnaJ putative expressed
OsISM04223	LOC_Os01g32870	18043507	ACCTCAGCCGCACAAAGTAA	CGAAGGCTCGAAAGTACCTG	60.4	209	93	heat shock protein DnaJ putative expressed
OsISM04224	LOC_Os01g32870	18043655	TCTTTGAACAAGAAATTAATGGAGG	TGCAAGAAATACATGCCTGC	59.9	2846	95	heat shock protein DnaJ putative expressed

ISM IDs	Rice gene locus IDs	Physical positions (bp) on chromosomes	Forward primers (5'-3')	Reverse primers (5'-3')	Melting tempearature (T˚C)	Expected product size (bp) in PCR	Expected product size (bp) in Q-PCR	Putative Functions
OsISM04225	LOC_Os01g32870	18046469	AAGGTGACATCTGCAGGCAT	CTCAGGATCCTTGGCCATT	60.4	176	91	heat shock protein DnaJ putative expressed
OsISM04226	LOC_Os01g32870	18046689	GTCGGCTCTCAAATCTGGAA	TCTTTGCACACATTGCTTCA	59.7	169	93	heat shock protein DnaJ putative expressed
OsISM04227	LOC_Os01g32870	18046910	GAAGAGAAATGATTTGCGCC	ACCGCTTCTTTTTCTTGGGT	60.0	196	97	heat shock protein DnaJ putative expressed
OsISM04228	LOC_Os01g32870	18047053	TTAGCGCGGTTTCAAGAAGT	CGTGAAGGAGGAGTGGATGT	60.1	1651	97	heat shock protein DnaJ putative expressed
OsISM04229	LOC_Os01g32900	18064113	CTACCTCCCAGCATCGACAT	CCTTGGTGGAAGTTGTGGAA	60.5	569	94	retrotransposon protein putative unclassified
OsISM04230	LOC_Os01g32930	18076663	GATGCTGAGCGTCTCCGT	ATCTTCGATGGGAGGATGC	60.0	116	64	SGS domain containing protein expressed
OsISM04231	LOC_Os01g32990	18117093	GGGTGGACACTGGTGCTAAT	TCCCATCAACAAGGAGGAAC	59.9	92	54	retrotransposon protein putative Ty1-copia subclass
OsISM04232	LOC_Os01g32990	18117343	TGAGTCCAATAAATGTGCCG	GAGGCTTGCGAGTTTGTTTC	59.8	284	96	retrotransposon protein putative Ty1-copia subclass
OsISM04233	LOC_Os01g33000	18124339	GCCTGCTCAGTTCCCCTC	CTCTGAGGAAGAAGTGCGGG	61.7	203	99	expressed protein
OsISM04234	LOC_Os01g33000	18124673	GTCCTGCGACTGTCTTCTGG	ACTCCCCATCACTGTCATCC	60.4	1167	53	expressed protein
OsISM04235	LOC_Os01g33000	18125981	CTCCATTTTGTCGTGAACCA	GGGTCCTCAGTCCTGGAAAT	59.9	144	70	expressed protein
OsISM04236	LOC_Os01g33000	18126137	ATTTATCACCGCGAAGTTGG	TTAATGTTGGGCCAGTAGGG	59.9	162	70	expressed protein
OsISM04237	LOC_Os01g33000	18126304	TTGGATGCATGCTTTCTCAC	TGCTACCAGGACCCCAGTAT	59.6	734	89	expressed protein
OsISM04238	LOC_Os01g33040	18151463	GCTGCACCCACCTCTTACAC	TTCTGTCTGGGAAGCTGGTC	60.6	211	55	kinesin motor domain containing protein expressed
OsISM04239	LOC_Os01g33040	18151708	TGTCTGCATTGACAGGCATT	ACCACTGCTGGTTTGACCAT	60.4	144	57	kinesin motor domain containing protein expressed
OsISM04240	LOC_Os01g33040	18151863	CATGAGAGGCGTCACAGAGA	TTGATGATAAAGTCCCTCTCAGG	59.9	182	79	kinesin motor domain containing protein expressed
OsISM04241	LOC_Os01g33040	18152094	CCTGAGTCTACCAATCTTCGC	TTGGCTATCCTTGGCAATTT	59.2	183	88	kinesin motor domain containing protein expressed
OsISM04242	LOC_Os01g33040	18152256	GAAATTGCCAAGGATAGCCA	TTAGTGCAGTCTCCCCAACC	60.1	211	80	kinesin motor domain containing protein expressed

ISM IDs	Rice gene locus IDs	Physical positions (bp) on chromosomes	Forward primers (5'-3')	Reverse primers (5'-3')	Melting tempearature (T˚C)	Expected product size (bp) in PCR	Expected product size (bp) in Q-PCR	Putative Functions
OsISM04243	LOC_Os01g33040	18152447	GGTTGGGGAGACTGCACTAA	CACATCCTGACACTTCACGG	60.1	182	93	kinesin motor domain containing protein expressed
OsISM04244	LOC_Os01g33040	18152606	GCTCCGTGAAGTGTCAGGAT	TTTGTGCAGCTCGTTCACTT	60.0	164	84	kinesin motor domain containing protein expressed
OsISM04245	LOC_Os01g33040	18152787	AGACTGAAAGAAGGCTGCCA	CGATAGGGTATGTGTCCACTTC	59.3	754	99	kinesin motor domain containing protein expressed
OsISM04246	LOC_Os01g33040	18153670	TGCCAAGGAGGTTACGAATAA	GCTCAGCTTCGAGTCTAGCAA	59.8	181	99	kinesin motor domain containing protein expressed
OsISM04247	LOC_Os01g33040	18153860	CTGATCGTGCCTCCTCTTCT	TTTGCTTCTTGAGTTCCTCCA	59.8	171	91	kinesin motor domain containing protein expressed
OsISM04248	LOC_Os01g33040	18154023	GAAGCAAAGAGACAATGCACA	TGGTGAGTCAAAAGGATTCCA	59.8	206	89	kinesin motor domain containing protein expressed
OsISM04249	LOC_Os01g33040	18155017	CCGGGCTTATGTCACTGAAC	GCCTTCATTCGATTCCAACT	59.8	194	98	kinesin motor domain containing protein expressed
OsISM04250	LOC_Os01g33040	18155474	AGCTGGTGATGACTCCACAA	GGAATTCTCGCTCATTCCTC	59.0	406	66	kinesin motor domain containing protein expressed
OsISM04251	LOC_Os01g33060	18163021	CTCCTTCTTCTCCGACCCC	CAGCAAGCTCTGTGGATCAG	60.4	663	89	HEAT repeat family protein putative expressed
OsISM04252	LOC_Os01g33060	18163796	CTCCTGATGGCTCAAATGCT	TTTGGCATCCCTTTTGTCAT	60.3	1155	72	HEAT repeat family protein putative expressed
OsISM04253	LOC_Os01g33060	18165021	TTTTGGCTGGGTATCGACTT	TCAGGCACACAAGCTGAACT	59.6	655	95	HEAT repeat family protein putative expressed
OsISM04254	LOC_Os01g33060	18165673	TGATGCCCTTCGTCGTTACT	CAGTAGCGTCTCATTTTCGC	59.7	906	95	HEAT repeat family protein putative expressed
OsISM04255	LOC_Os01g33060	18166672	GAAGCTGCACACAAGGACAT	CCTTCCTCTGGACATCCAAT	58.9	158	77	HEAT repeat family protein putative expressed
OsISM04256	LOC_Os01g33060	18167271	TTCATACCGAGAAAGTAGAGAGTCC	TTGGAAGAGCATCCGAAACT	59.6	188	91	HEAT repeat family protein putative expressed
OsISM04257	LOC_Os01g33060	18167448	TGCTCTTCCAAAGATTGTTCC	GCACAGATGATCAACGGAAG	59.3	147	70	HEAT repeat family protein putative expressed
OsISM04258	LOC_Os01g33060	18167657	TCAAACGTCCTGATGGACAA	CACTCTTAGCAAGCTCCACACA	60.4	188	64	HEAT repeat family protein putative expressed
OsISM04259	LOC_Os01g33060	18167820	GCTTGTGTGGAGCTTGCTAA	CGCTCCTCATATTGGTGATT	58.4	177	96	HEAT repeat family protein putative expressed
OsISM04260	LOC_Os01g33060	18168134	ACCTCACATTGCTGCTTCCT	CGAAGGGTCACAGACCAACT	60.0	279	87	HEAT repeat family protein putative expressed

ISM IDs	Rice gene locus IDs	Physical positions (bp) on chromosomes	Forward primers (5'-3')	Reverse primers (5'-3')	Melting tempearature (T˚C)	Expected product size (bp) in PCR	Expected product size (bp) in Q-PCR	Putative Functions
OsISM04261	LOC_Os01g33060	18168499	ACTGGCACATATCCTGGCAT	TCTATTGCACCTTCAACCCC	60.2	147	64	HEAT repeat family protein putative expressed
OsISM04262	LOC_Os01g33060	18168763	TGCTGATCCTTCTACAGGCA	TCCATTCAAATGCTGACCAC	59.5	419	99	HEAT repeat family protein putative expressed
OsISM04263	LOC_Os01g33060	18169221	GCCTGTTGCCTGCTAAAGAG	CTATGCCATAACGTCCCGAT	60.0	176	79	HEAT repeat family protein putative expressed
OsISM04264	LOC_Os01g33060	18169468	TATTTTCCTCTGGCAACCCA	AATTCCAAGTTTTTCGGCAA	60.0	163	73	HEAT repeat family protein putative expressed
OsISM04265	LOC_Os01g33060	18169766	ATCATTGACGCAGTTCGGTT	GGCATCAGAGATCACAACCA	60.1	190	91	HEAT repeat family protein putative expressed
OsISM04266	LOC_Os01g33060	18169966	CCAATGCTGCAGCTCTACTG	GGCAAAACATGAGTTGATGC	59.4	191	71	HEAT repeat family protein putative expressed
OsISM04267	LOC_Os01g33060	18170213	AAGCATTTGGAGCTGTTGCT	GCTTCATGTGATCCATCTTCAA	60.1	174	98	HEAT repeat family protein putative expressed
OsISM04268	LOC_Os01g33060	18170417	GGCAGTGCCACATACAACAG	ACGCCGCTCAATATCATTTC	60.1	196	98	HEAT repeat family protein putative expressed
OsISM04269	LOC_Os01g33060	18170593	GAAATGATATTGAGCGGCGT	CACGTACGCTTGTAGCAGGA	60.1	1300	94	HEAT repeat family protein putative expressed
OsISM04270	LOC_Os01g33070	18175792	CTCTGCACCATGGCCCTC	CTCAATCCTCCCCAGAATCA	61.4	274	85	pentatricopeptide repeat protein PPR868-14 putative
OsISM04272	LOC_Os01g33080	18186768	ATCACCTCTTCCACCTCACC	CTTCTTGGTGTTGATGGCG	59.6	212	99	fimbrin-like protein 2 putative expressed
OsISM04273	LOC_Os01g33080	18187044	GCTGCACTGTCGTGAACATC	TGTGATATCAAACCCAAGATAAGG	59.9	814	74	fimbrin-like protein 2 putative expressed
OsISM04274	LOC_Os01g33080	18187821	GCTTGCAGCCTCACCTTATC	TGAGGTGTGCTCTTCAGGTTT	59.9	182	86	fimbrin-like protein 2 putative expressed
OsISM04275	LOC_Os01g33080	18187983	AACCTGAAGAGCACACCTCAA	GATAAGCTCATAAGCTCCTCCA	58.8	169	72	fimbrin-like protein 2 putative expressed
OsISM04276	LOC_Os01g33080	18188189	TTGAAGAAAGGAGGTTTCCAA	TCAGGTGCCAAGACATTCAG	59.3	542	96	fimbrin-like protein 2 putative expressed
OsISM04277	LOC_Os01g33080	18188842	CATTGTCGACGGTTTACCAA	TCATTTGTTTTGACAGCCCA	59.8	1111	81	fimbrin-like protein 2 putative expressed
OsISM04278	LOC_Os01g33080	18190008	GAGGTCATTCCGGTTATGGA	CAATCACCTCAAGAAGCACC	59.0	188	99	fimbrin-like protein 2 putative expressed
OsISM04279	LOC_Os01g33080	18190314	TTCCTTGGTGAATATTGCTGG	CCTCATCAATTGCCACAGAA	59.8	481	80	fimbrin-like protein 2 putative expressed

ISM IDs	Rice gene locus IDs	Physical positions (bp) on chromosomes	Forward primers (5'-3')	Reverse primers (5'-3')	Melting tempearature (T˚C)	Expected product size (bp) in PCR	Expected product size (bp) in Q-PCR	Putative Functions
OsISM04280	LOC_Os01g33080	18190859	ACGACATACTGATTTGGGCA	ATGCCACTTGAAAGGCTTCT	59.0	330	92	fimbrin-like protein 2 putative expressed
OsISM04281	LOC_Os01g33080	18191217	GAACCTCGGGTTGTGAATTG	CCAAGCTTCCTAGCCACAGA	60.4	421	99	fimbrin-like protein 2 putative expressed
OsISM04282	LOC_Os01g33080	18191620	TGTGGCTAGGAAGCTTGGTT	TGCTTGCTGTCAAGGTTAGC	59.5	280	90	fimbrin-like protein 2 putative expressed
OsISM04283	LOC_Os01g33090	18205620	TACATCCCTAATGGCAGCCT	CATGGCAATGGTGTGAATGT	59.9	496	72	expressed protein
OsISM04284	LOC_Os01g33110	18231241	ATTGGAGTGCATTAGCTGGG	CGTCACATTTCTCCGTGACT	59.4	199	79	receptor-like protein kinase 5 precursor putative expressed
OsISM04285	LOC_Os01g33130	18236372	CGTTCATCCTGTGGTTTGG	AGGGATTTATTCTCCGCGTT	59.9	602	79	retrotransposon protein putative unclassified
OsISM04286	LOC_Os01g33150	18252964	GTGCACCATCAGCCGAATAC	CTTCTCCTGCAAAATCGGAC	60.6	1473	63	retrotransposon protein putative Ty3-gypsy subclass
OsISM04287	LOC_Os01g33160	18258638	CCTCGTAAACCTCGTCCCTT	ACGTGTGTGAATCCATGGTG	60.4	7075	85	stress responsive A/B Barrel domain containing protein expressed
OsISM04288	LOC_Os01g33220	18289997	ATGCACGTGACTTGCTGAAT	ACCCACCCAAAAATCTAGCC	59.8	148	82	retrotransposon protein putative unclassified
OsISM04289	LOC_Os01g33220	18290212	TGCTTGTAAGGCTGAAAGGG	AACGGTGGCACTGAACATCT	60.5	260	93	retrotransposon protein putative unclassified
OsISM04290	LOC_Os01g33220	18291245	GCATCACCTTCAGTTGCCTA	TCCTTGGGAAACACATCAGA	59.0	128	51	retrotransposon protein putative unclassified
OsISM04291	LOC_Os01g33220	18291476	GAAACCAAGGAGATTCAGCG	TGACGATATCGAATGGTAATGTT	59.1	170	78	retrotransposon protein putative unclassified
OsISM04292	LOC_Os01g33220	18292780	GGATGGAAGAACATGGAACA A	AACGAACCTGCCAACATCTC	60.0	214	64	retrotransposon protein putative unclassified
OsISM04293	LOC_Os01g33220	18292998	GCTGCGCTACATGTCAAAAG	AAATCAATTGGAGCACGAGG	59.9	568	81	retrotransposon protein putative unclassified
OsISM04294	LOC_Os01g33220	18294038	GCTCGTGCACGTCAACTAAA	AACCGATACACCGTCTCCAA	60.2	718	97	retrotransposon protein putative unclassified
OsISM04295	LOC_Os01g33324	18358054	AATACCGCCTCAAACTCCAG	CACATGTCCTTCCCCGAA	59.8	548	96	retrotransposon protein putative Ty1-copia subclass
OsISM04296	LOC_Os01g33324	18359228	GGTGGGTTGATACTGGTGCT	ACACCATGAACAGCTGCAAG	59.9	124	86	retrotransposon protein putative Ty1-copia subclass
OsISM04297	LOC_Os01g33324	18359303	GTTCCTCCTTGTTGATGGGA	CAAGGAAAAGCGAAACAAGC	59.9	262	84	retrotransposon protein putative Ty1-copia subclass

ISM IDs	Rice gene locus IDs	Physical positions (bp) on chromosomes	Forward primers (5'-3')	Reverse primers (5'-3')	Melting tempearature (T°C)	Expected product size (bp) in PCR	Expected product size (bp) in Q-PCR	Putative Functions
OsISM04298	LOC_Os01g33324	18359546	CTTGTTTCGCTTTTCCTTGG	TTTGGTTTTCTACCTCAGCCTT	59.6	425	99	retrotransposon protein putative Ty1-copia subclass
OsISM04299	LOC_Os01g33324	18359972	TGGAAAGGAAAATCAAACGG	GAGTGCGGTTCTTTCTTTCG	60.0	156	99	retrotransposon protein putative Ty1-copia subclass
OsISM04300	LOC_Os01g33324	18360297	CACATGGGGCTATTTGGC	TCTTGGAGTATCATCCACAAGAT	58.8	406	92	retrotransposon protein putative Ty1-copia subclass
OsISM04301	LOC_Os01g33324	18360944	ACTTATTCACCGGTTGCTCG	TTGACCTCCCCAATCACATT	60.2	394	86	retrotransposon protein putative Ty1-copia subclass
OsISM04302	LOC_Os01g33324	18361625	CATTTGCTGTGAGCAAGTTGA	GCTTCCATTGTTGACCTCGT	60.1	276	83	retrotransposon protein putative Ty1-copia subclass
OsISM04303	LOC_Os01g33360	18369707	CTTCTTCGACGTCCTCGC	GAGCAGCGCCTCGATCTC	61.0	220	71	DnaK family protein putative expressed
OsISM04304	LOC_Os01g33360	18370217	CGACGACGACAAACCAACTA	CGTCTCGATGCTGATTGTGT	59.8	149	74	DnaK family protein putative expressed
OsISM04305	LOC_Os01g33380	18377672	GTTCGATGCAGGGTTTTGAT	TATGTTCCACCTGCACGAGA	60.1	338	86	retrotransposon protein putative unclassified
OsISM04306	LOC_Os01g33380	18378152	GGCTGGGAAAGTGCTAGTGA	GAGAAACTTGAGCGCCATTC	60.2	127	78	retrotransposon protein putative unclassified
OsISM04308	LOC_Os01g33440	18412441	TGAGGTGATGCAGAAGAAGA A	GCACCATCCAGAATAACTGACA	59.3	211	97	retrotransposon protein putative unclassified
OsISM04309	LOC_Os01g33440	18412929	GGTTTGCCTATGGGGGTT	GACAAAACCCAATTAGGGGC	60.1	649	54	retrotransposon protein putative unclassified
OsISM04310	LOC_Os01g33440	18413816	GCTGGGAGAGGGAGAATACC	CGCGATCTCTTTCCTTTGAC	60.0	400	89	retrotransposon protein putative unclassified
OsISM04311	LOC_Os01g33450	18416097	ACCAAGGGGATCTGACGAG	TGGGGAGTAGTTCTTGGCAC	60.1	728	99	expressed protein
OsISM04313	LOC_Os01g33460	18424817	AGGGGCAATTTGAGAGGTTT	CATCTTCTTGACCACCACCC	60.2	136	78	retrotransposon protein putative unclassified
OsISM04314	LOC_Os01g33460	18425807	AGAGCGGTCTCATGAACTCAA	GCCATTGTGAAGGAGTCTCA	59.4	352	95	retrotransposon protein putative unclassified
OsISM04315	LOC_Os01g33460	18426214	GGGACAACTTCAATCATGAGG	GAAATATTCTCGCCCCTTGG	60.1	232	86	retrotransposon protein putative unclassified
OsISM04316	LOC_Os01g33460	18426449	CAAGAGAGTACTTTGGGTGCC	CAACTTCTTCCACCGGAAAC	59.2	121	82	retrotransposon protein putative unclassified
OsISM04317	LOC_Os01g33460	18426676	GGAAAGGGAAAGGTTATGGG	TCCTCATACCAACATGGGCT	60.0	320	83	retrotransposon protein putative unclassified

ISM IDs	Rice gene locus IDs	Physical positions (bp) on chromosomes	Forward primers (5'-3')	Reverse primers (5'-3')	Melting tempearature (T˚C)	Expected product size (bp) in PCR	Expected product size (bp) in Q-PCR	Putative Functions
OsISM04318	LOC_Os01g33460	18428251	GTTGAACAACTTCACCCGGA	CAACCTCGCCTTGTTCCTTA	60.6	133	83	retrotransposon protein putative unclassified
OsISM04319	LOC_Os01g33470	18431382	GATCAACTGGGAGGAGTTGC	TGGTGGCATAAAACTGAGCA	60.0	142	78	retrotransposon protein putative unclassified
OsISM04321	LOC_Os01g33514	18446337	CCTTGATGTTCTTCGGTGGT	ATCACGCACAGTCCCAAAAT	60.2	173	80	expressed protein
OsISM04322	LOC_Os01g33530	18454878	TGGACTCTACGGCAAAAACA	CAATCTCCACAGGCAAGTCA	59.6	719	76	hypothetical protein
OsISM04323	LOC_Os01g33530	18455664	GGTCCAAGAACTCCAAATGC	TCTTGCGTGGATATCGTGAA	59.9	143	66	hypothetical protein
OsISM04324	LOC_Os01g33530	18456034	GGAGCAAATTTGGAGTGTCTG	GCCTGTAGTGTTCAACAAAAGC	59.3	173	98	hypothetical protein
OsISM04325	LOC_Os01g33530	18456324	ACGAATCAGCTGGCCATAGA	CCATCAACGATTGCTACATACC	60.1	178	93	hypothetical protein
OsISM04326	LOC_Os01g33530	18456438	GATGCAGGTCTTCCTTCCAA	GGCAAGCTTGATGCATTTTT	60.2	256	74	hypothetical protein
OsISM04327	LOC_Os01g33530	18456760	CGATACTTGGCCAGGAAGAA	AGTCAGACACACCCCATTCC	60.0	162	85	hypothetical protein
OsISM04328	LOC_Os01g33530	18456894	CGAGTACTGGAATGGGGTGT	GAATCGCTGCTATCTTTCTTCTG	59.8	156	81	hypothetical protein
OsISM04329	LOC_Os01g33550	18463304	GATCCTTGGCCATTCTGTCA	CCCTCCACTTGACTCTGAGC	60.5	223	90	MEGL19 - Maternally expressed gene MEG family protein precursor putative
OsISM04330	LOC_Os01g33580	18481073	TGGTTCGAGAGCAAACCTTC	TGCACCGAGAGATTCCTTCT	60.2	310	49	retrotransposon putative centromere-specific
OsISM04331	LOC_Os01g33580	18481905	CTGAAGCAACTCAGATCCGTT	GAGGACACTAGTTTCGGGCA	59.9	331	99	retrotransposon putative centromere-specific
OsISM04332	LOC_Os01g33590	18484322	GTACGATGAGTCGAAGGGCT	GCATGTTGGGCATTCATCTA	59.4	286	80	transposon protein putative CACTA En/Spm sub-class
OsISM04333	LOC_Os01g33590	18484913	TCCTTTTGGAGACATGAGCA	TCAATACCAGGCTGACAGGG	60.2	141	61	transposon protein putative CACTA En/Spm sub-class
OsISM04334	LOC_Os01g33590	18486030	TTGTGCTACTTTTTGAACGCA	TTGATGGAGGTACAATGGGC	60.1	195	64	transposon protein putative CACTA En/Spm sub-class
OsISM04335	LOC_Os01g33609	18495911	TGAAGAAGGCGACAAGGACT	TTTTTCTTTGGCTGCCATTT	59.8	166	79	transposon protein putative CACTA En/Spm sub-class
OsISM04336	LOC_Os01g33609	18496288	AGGGAAAAGATCCCCATCAC	AGTACCCAGCGCCGTACTTA	60.0	623	97	transposon protein putative CACTA En/Spm sub-class

ISM IDs	Rice gene locus IDs	Physical positions (bp) on chromosomes	Forward primers (5'-3')	Reverse primers (5'-3')	Melting tempearature (T˚C)	Expected product size (bp) in PCR	Expected product size (bp) in Q-PCR	Putative Functions
OsISM04337	LOC_Os01g33609	18497011	AACTTAGAGACGAAGGGGCTG	TAGCGGCCTCAAGAGTTTTC	59.7	189	88	transposon protein putative CACTA En/Spm sub-class
OsISM04338	LOC_Os01g33609	18497306	TGACTTCCCTAATGCAGATGG	GTTTTGGCACCACAGGTTTT	60.0	128	90	transposon protein putative CACTA En/Spm sub-class
OsISM04339	LOC_Os01g33609	18497442	GAAAAACCTCCGAGACCTCC	GTCCCATTCCAATCCTGGTA	59.8	217	94	transposon protein putative CACTA En/Spm sub-class
OsISM04340	LOC_Os01g33609	18497895	GCCAACATACTTGGCAACTG	TGGATCCAAGAAGCCGATAG	59.7	413	89	transposon protein putative CACTA En/Spm sub-class
OsISM04341	LOC_Os01g33609	18498472	TAAGAGAGTCGTCATGGCCG	TCGTCAAAGCTTGTTCGATG	60.7	747	76	transposon protein putative CACTA En/Spm sub-class
OsISM04342	LOC_Os01g33670	18529534	CAGCCATCATCAATCCGTC	ATGAACTTCTCCCCTTCGCT	60.1	361	72	hypothetical protein
OsISM04343	LOC_Os01g33710	18555312	CTACCCACTGGAAGAGCAGC	TCTCCATTTTGTCCAATGCTT	59.8	226	86	expressed protein
OsISM04345	LOC_Os01g33750	18573186	GAGCTGGGTGCAACTATTGA	CCATGCCGTACTCATCTCCT	59.5	258	82	retrotransposon protein putative unclassified
OsISM04346	LOC_Os01g33750	18573553	ACAAGGGGGATGCATTGATA	CCCAAAAACCTGTGGTCATC	60.2	132	83	retrotransposon protein putative unclassified
OsISM04347	LOC_Os01g33750	18573800	GGAAGAGGTATTATATCGTGCCC	CCTCTGGACACTCCCTTTCA	60.2	100	62	retrotransposon protein putative unclassified
OsISM04348	LOC_Os01g33750	18573878	GCTGAAAGGGAGTGTCCAGA	GTACCACCTCGAGCACGACT	60.4	221	63	retrotransposon protein putative unclassified
OsISM04349	LOC_Os01g33750	18574289	GGTCTATCGGATGATAAAGCTGA	GCATTGCATGTGCCTCACTA	60.2	289	74	retrotransposon protein putative unclassified
OsISM04350	LOC_Os01g33750	18574573	AATGCTGAAGGAAGCATTGG	AGCCTCCATCTCAGTTGCTG	60.4	215	64	retrotransposon protein putative unclassified
OsISM04351	LOC_Os01g33750	18575086	GCGAGAAGGAGACCAGAAAA	CAGCTTCACAGCCACTTCAC	59.6	387	85	retrotransposon protein putative unclassified
OsISM04352	LOC_Os01g33750	18577121	ATTTCTGGTGGGGTGATGAG	GCCTTTAAAACCCACACACA	59.2	190	65	retrotransposon protein putative unclassified
OsISM04353	LOC_Os01g33750	18577392	GCTGCAATTGCTGAAGACAG	CCCAGATTTTTCGTAGTGCC	59.7	288	89	retrotransposon protein putative unclassified
OsISM04354	LOC_Os01g33750	18577787	AATTTGGAGTACGCCTGTGC	GCAAGCCATATTCCTTCCCT	60.3	714	97	retrotransposon protein putative unclassified
OsISM04356	LOC_Os01g33760	18581674	AGTCCACCAGGCCACCAC	TGTTGCTCATCTTGCTTCTTG	60.6	474	91	hypothetical protein

ISM IDs	Rice gene locus IDs	Physical positions (bp) on chromosomes	Forward primers (5'-3')	Reverse primers (5'-3')	Melting tempearature (T˚C)	Expected product size (bp) in PCR	Expected product size (bp) in Q-PCR	Putative Functions
OsISM04357	LOC_Os01g33762	18584538	GCCACAGCAGAATTTTCAAC	AGCACCAAAGCCAGCATAAT	59.0	469	85	expressed protein
OsISM04358	LOC_Os01g33762	18585001	GGTGCTACCCAAGCCTGTT	GATTTCTTTTGGGCAGCATC	59.9	145	89	expressed protein
OsISM04359	LOC_Os01g33762	18585126	GATGCTGCCCAAAAGAAATC	TGATGAGCACATCCAAGACC	59.6	321	86	expressed protein
OsISM04360	LOC_Os01g33762	18585465	GGGGATGCTGTTAGTGATGG	AGCTCACCCTCTGCCTTTG	60.4	1114	89	expressed protein
OsISM04361	LOC_Os01g33784	18592993	GTGGTATTGCTGCAACATGG	AGCCAACAACACCATTCGAT	60.2	2160	65	lipase family protein putative expressed
OsISM04362	LOC_Os01g33784	18595113	CGTTTCTCTTGCAGTTGGGT	AAACCACGCAGATTTCCAAG	60.2	178	99	lipase family protein putative expressed
OsISM04363	LOC_Os01g33784	18595291	AGTCTCCAGGGAGCATGTTG	GCTGGCATATCTTTGGTTCC	59.9	220	88	lipase family protein putative expressed
OsISM04364	LOC_Os01g33800	18600445	ACATCCTCGGTGATTTCCC	TCCAGAAATATCAATGCCTTCAC	60.0	792	87	heat shock protein DnaJ putative expressed
OsISM04365	LOC_Os01g33800	18601501	GCTGATGAACAACCCCCTAA	GATGGCATAGCAGGACCAAT	59.9	1441	48	heat shock protein DnaJ putative expressed
OsISM04366	LOC_Os01g33800	18602958	GCAGCTGCTGAAATGACAGA	GGGGCCTATGAGAAAACCAT	60.2	255	76	heat shock protein DnaJ putative expressed
OsISM04367	LOC_Os01g33800	18603246	GCAAATGAAGCTGAGCGATT	CCACAACATCATATGGCGAG	60.2	1202	74	heat shock protein DnaJ putative expressed
OsISM04368	LOC_Os01g33800	18604446	GGGCATCAATTGGAAGATGT	CCTGAGCTGAAGGATGAGGA	60.1	1411	99	heat shock protein DnaJ putative expressed
OsISM04369	LOC_Os01g33800	18605879	TGCTTTCAAAGACTTACAGGACC	CCTCAAATTGTTCCATCTCCTC	59.9	1114	84	heat shock protein DnaJ putative expressed
OsISM04370	LOC_Os01g33800	18606925	CCCTTTGTTCCAGAGAGGTG	CGCGCATTGCTTTAAGCTC	60.9	213	87	heat shock protein DnaJ putative expressed
OsISM04371	LOC_Os01g33800	18607117	TCGAGCTTAAAGCAATGCG	CTGTGCCTGTTTTGGAACTG	59.8	1230	99	heat shock protein DnaJ putative expressed
OsISM04372	LOC_Os01g33800	18608371	ACCACATTGCCTCCTGAAAG	CACCACGTCCTTCTTTACCA	59.4	199	83	heat shock protein DnaJ putative expressed
OsISM04373	LOC_Os01g33800	18608563	CGTGGTGATACAAGTGTCTGG	GCCTTTGCTTTGTTGTAGGC	59.5	1312	90	heat shock protein DnaJ putative expressed
OsISM04374	LOC_Os01g33810	18612839	AGGGAGCTCAGGCTGATACA	AAGGGAGCGTGAAATGCTT	59.9	387	97	disease resistance protein RPM1 putative expressed

ISM IDs	Rice gene locus IDs	Physical positions (bp) on chromosomes	Forward primers (5'-3')	Reverse primers (5'-3')	Melting tempearature (T˚C)	Expected product size (bp) in PCR	Expected product size (bp) in Q-PCR	Putative Functions
OsISM04375	LOC_Os01g33820	18616513	CTTCAGGGAGGGGTCAGAT	CGACTTACTGCACACCTGGA	59.5	199	72	hypothetical protein
OsISM04376	LOC_Os01g33840	18621234	GAGGAGAGATGGGTGACCAG	TGCATCTGCAATTTCCTTTG	59.7	1416	78	heat shock protein binding protein putative
OsISM04377	LOC_Os01g33869	18625341	AAAGCCCAAGGACTTTTGGT	AGTTGATCCTCGGGTTTCTG	59.6	12824	88	expressed protein
OsISM04378	LOC_Os01g33869	18639738	TATTCAGGCCCAGGTTATGG	TCGAGCTTCTGAACTGCAAC	59.5	407	96	expressed protein
OsISM04379	LOC_Os01g33869	18640155	TTCCCTGATGGACAACCTTC	TGGCAAATCTGTGAGCTGAG	60.0	212	96	expressed protein
OsISM04380	LOC_Os01g33869	18640394	GGCTCTACAGAGTGCCAACC	CGCCTTGTCCATTTCATACC	60.1	182	86	expressed protein
OsISM04381	LOC_Os01g33910	18651384	TTTCTGGTTCGTGACCATCA	CAGAGGTGTGTCCGCTATCA	60.0	385	92	hypothetical protein
OsISM04382	LOC_Os01g33930	18658210	GGACCGCAAATTGAGAAGAG	CAGCCTTGATGATTGCTTCA	59.9	2521	95	expressed protein
OsISM04383	LOC_Os01g33930	18661389	GGATACAGACACAAGAGGGC A	TGTCCTGATCATCCACAAGC	59.9	492	90	expressed protein
OsISM04384	LOC_Os01g33930	18662039	TTACCGCGGTTACCGTCC	GCAGATCAGCTTGTTGGCTT	61.2	597	89	expressed protein
OsISM04385	LOC_Os01g33930	18662983	CTTAGGGCCTACCAAGGAGC	TTTTTGACTCTGCTAGGTCGAT	59.2	288	98	expressed protein
OsISM04386	LOC_Os01g33930	18663247	CGATCGACCTAGCAGAGTCA	GCCCTCTGCAGAATCTATTAGC	59.3	575	87	expressed protein
OsISM04387	LOC_Os01g33950	18672909	CAGCAGCACTGTTGGGATAA	CAAAGCTGTAGGCATCACCA	59.9	220	96	conserved hypothetical protein
OsISM04388	LOC_Os01g33950	18673363	TGGACATGAAACATGCTGCT	TGCATTACCTTACGATTGTGTCA	60.3	430	76	conserved hypothetical protein
OsISM04389	LOC_Os01g33950	18673727	CCATTGCTTCCGACAAATG	GGAGCCAGAAGGCTTAGGAA	60.4	839	99	conserved hypothetical protein
OsISM04390	LOC_Os01g33990	18702665	GGACACCACCGACGATAGAT	TGTGTACCATCCAATGCACC	60.0	165	61	transposon protein putative CACTA En/Spm sub-class
OsISM04391	LOC_Os01g33990	18702871	GGTCAAGAAAAGGAGACATT GTT	CCTCGGTAAGGGGCTAAAAA	59.6	306	98	transposon protein putative CACTA En/Spm sub-class
OsISM04392	LOC_Os01g33990	18703533	TTCGCAACACTCTTGCTCAT	CACAATGCTTGAGCTTCCAA	59.8	196	55	transposon protein putative CACTA En/Spm sub-class

ISM IDs	Rice gene locus IDs	Physical positions (bp) on chromosomes	Forward primers (5'-3')	Reverse primers (5'-3')	Melting tempearature (T˚C)	Expected product size (bp) in PCR	Expected product size (bp) in Q-PCR	Putative Functions
OsISM04393	LOC_Os01g33990	18703701	GGAGCATCTTGGAAGCTCAA	GCAGTAAGGCCAGAACTTGG	60.2	207	72	transposon protein putative CACTA En/Spm sub-class
OsISM04394	LOC_Os01g33990	18703977	CAAAAGTGGCTTTTCTTGGG	AGACCCTTTGCCTCATTGTT	59.2	388	97	transposon protein putative CACTA En/Spm sub-class
OsISM04397	LOC_Os01g34010	18719077	CGGATCTGCGAGTTCTTACC	GGATGAACTGATCCCTTGGA	59.8	709	80	conserved hypothetical protein
OsISM04398	LOC_Os01g34010	18721302	CATTTTACACGCCAACTCCA	CCACTTCTGACTTTATTGCTTTCA	59.7	193	99	conserved hypothetical protein
OsISM04399	LOC_Os01g34010	18721466	GCTGCTGAAAGCAATAAAGTCA	TTGGTCCTCCATTCTTGGAA	60.1	202	94	conserved hypothetical protein
OsISM04401	LOC_Os01g34030	18731996	AAGTGGACTTTCAACCGCC	CGACCCTGAGACTCCATGA	59.9	159	58	retrotransposon protein putative unclassified
OsISM04402	LOC_Os01g34030	18732243	GGACCCACAGGACACATACC	GCTTGGACTTGGACGTTGAG	60.5	334	83	retrotransposon protein putative unclassified
OsISM04403	LOC_Os01g34030	18732992	CAGGAGTTTGAAGCCACGAT	TCCCACTGATCTAACAGGCG	60.7	137	93	retrotransposon protein putative unclassified
OsISM04404	LOC_Os01g34060	18754784	CATGGACGGAGGAAGAGC	CCGCGAGATGACGAAGTT	59.6	219	96	MYB family transcription factor putative expressed
OsISM04406	LOC_Os01g34080	18763411	CGCCATGGAGGAAATTTTTA	CCAAATGGTACACAAAGACTGC	59.7	265	80	armadillo/beta-catenin-like repeat family protein expressed
OsISM04407	LOC_Os01g34080	18763829	GCTTTGGTTGAAGCAGGTGT	AGTCGTCCTCTTTGTTTTGC	58.7	2607	62	armadillo/beta-catenin-like repeat family protein expressed
OsISM04408	LOC_Os01g34080	18767022	CGCTGTTTATGCATTGGAAA	GCAAGCAGTTTTAGACCACCA	60.0	867	82	armadillo/beta-catenin-like repeat family protein expressed
OsISM04409	LOC_Os01g34080	18767896	GTGAAGGATCCCAATACCCC	GCTACCATGCTGATACCCGT	60.2	169	87	armadillo/beta-catenin-like repeat family protein expressed
OsISM04410	LOC_Os01g34080	18768044	CACGGGTATCAGCATGGTAG	GCATTGTATCCTCAGACCCA	58.8	146	63	armadillo/beta-catenin-like repeat family protein expressed
OsISM04411	LOC_Os01g34080	18768225	GATGGTGGAACCAATGGAAC	GCGTGAAATGACGTCGAAAT	60.5	1472	88	armadillo/beta-catenin-like repeat family protein expressed
OsISM04412	LOC_Os01g34080	18769823	GAAGCTGTTCTGGAAATGGG	TCAAAGTTTTCAGGATCGCC	59.9	987	57	armadillo/beta-catenin-like repeat family protein expressed
OsISM04413	LOC_Os01g34190	18848002	GACACGAGTTCTGCTCCTCC	CCAGCTCATCATCAACCTCA	59.9	2268	57	G-patch domain containing protein expressed
OsISM04414	LOC_Os01g34200	18852759	GAGGAAAGCTTCTACTCCGC	CTCCTCTTGCTCCTCCTCCT	59.2	206	97	AATF putative expressed

ISM IDs	Rice gene locus IDs	Physical positions (bp) on chromosomes	Forward primers (5'-3')	Reverse primers (5'-3')	Melting tempearature (T˚C)	Expected product size (bp) in PCR	Expected product size (bp) in Q-PCR	Putative Functions
OsISM04415	LOC_Os01g34200	18853058	CTCGAGAAGGAGTACCGCAC	CCCTTCGAAACATCGTCATC	60.2	179	87	AATF putative expressed
OsISM04416	LOC_Os01g34200	18853217	GATGACGATGTTTCGAAGGG	ATCTCGAGTGCCTTGTCCC	60.3	183	66	AATF putative expressed
OsISM04417	LOC_Os01g34200	18853412	GCAAAAGGCATTTTCCACTT	CAAAACATCGACCTGATGGG	60.3	185	58	AATF putative expressed
OsISM04418	LOC_Os01g34200	18853643	TCATCGAAGCAGACTCTTGGT	TAGCACGATTCCTCTCCAGC	60.3	174	65	AATF putative expressed
OsISM04419	LOC_Os01g34200	18853770	ACACTTTGAAAACAACCGGC	TCATCCTCTCCATTCAACTCTG	59.6	191	97	AATF putative expressed
OsISM04420	LOC_Os01g34200	18853949	TGGAGAGGATGATGAGTGGTC	TCACTTGTGTTTTCCTCTGCC	60.2	762	99	AATF putative expressed
OsISM04421	LOC_Os01g34200	18854722	GCTGCATTGAAGGGAAAGTT	CCTGCTTGGGTCTCTCATGT	59.8	167	79	AATF putative expressed
OsISM04422	LOC_Os01g34200	18854869	ACATGAGAGACCCAAGCAGG	TTGTTTTCATGCTCTCCCAC	59.5	185	92	AATF putative expressed
OsISM04423	LOC_Os01g34200	18855030	GGAAGTGGGAGAGCATGAAA	AAGTTCAGGATCACCTTCCG	59.7	279	59	AATF putative expressed
OsISM04424	LOC_Os01g34200	18855352	TTGAGTCATGTGATGCAGGC	AAGCACGACGATCAACCAGT	60.8	320	91	AATF putative expressed
OsISM04425	LOC_Os01g34200	18855652	ACTGGTTGATCGTCGTGCTT	TTGCAATCTTCTCGTGGACA	60.6	184	63	AATF putative expressed
OsISM04427	LOC_Os01g34240	18877845	TACTTCTTCCTTACCCCGCC	AATCTCTAGGGCAGGTGGCT	60.3	129	82	retrotransposon protein putative Ty3-gypsy subclass
OsISM04428	LOC_Os01g34250	18879662	GGACTCCGCAGACTAACAGG	CCAATCCATACCCAATATGACA	59.6	365	75	retrotransposon protein putative Ty3-gypsy subclass
OsISM04429	LOC_Os01g34250	18881356	TGCGATATCTACACTGACCAC A	TTTAAGCTCAGGAGCCATCC	59.3	210	97	retrotransposon protein putative Ty3-gypsy subclass
OsISM04430	LOC_Os01g34250	18882217	GGCACACAGTTTACTTCGCA	GAACCTTTTCTTCGGCCTCT	59.9	346	89	retrotransposon protein putative Ty3-gypsy subclass
OsISM04432	LOC_Os01g34290	18898620	TATACCTTCTCCATTGGGCG	ATCCAGCATACAAAGGCGTC	60.0	155	87	transposon protein putative CACTA En/Spm sub-class expressed
OsISM04433	LOC_Os01g34290	18898934	AGGTTATGTGTCCACTGGGG	GGACCACCCCTCTTTGACTT	60.0	172	74	transposon protein putative CACTA En/Spm sub-class expressed
OsISM04434	LOC_Os01g34290	18899151	AATCTTTGCCAACCTGAACG	ATCGATATCATTGCCGGGT	60.1	328	92	transposon protein putative CACTA En/Spm sub-class expressed

ISM IDs	Rice gene locus IDs	Physical positions (bp) on chromosomes	Forward primers (5'-3')	Reverse primers (5'-3')	Melting tempearature (T˚C)	Expected product size (bp) in PCR	Expected product size (bp) in Q-PCR	Putative Functions
OsISM04435	LOC_Os01g34290	18899540	GGGATGCATATGGTCAGGAG	CAATTCTTTGGGAGCAGGC	60.5	273	99	transposon protein putative CACTA En/Spm sub-class expressed
OsISM04436	LOC_Os01g34290	18899824	GGTCCACAATATGGTCAAGGA	TGCATCTCGTATGGGCTAGA	59.5	292	97	transposon protein putative CACTA En/Spm sub-class expressed
OsISM04437	LOC_Os01g34290	18900090	GCGAACTCTAGCCCATACGA	TGAATAGCCCGATGGAACTT	60.0	134	58	transposon protein putative CACTA En/Spm sub-class expressed
OsISM04438	LOC_Os01g34290	18901247	AAAGCCGACAAGGAAGGTTT	CCTCACGATGTGCATCTTCT	59.5	138	97	transposon protein putative CACTA En/Spm sub-class expressed
OsISM04439	LOC_Os01g34290	18901413	ACAACGTCGTCGATGAAGAA	TGAACGTTAGCCAAGCAATG	59.6	888	82	transposon protein putative CACTA En/Spm sub-class expressed
OsISM04441	LOC_Os01g34290	18902730	TGCCAAATGCAGAAGATCAA	CTTTGAGAGCAGGCCTCTTG	60.3	166	86	transposon protein putative CACTA En/Spm sub-class expressed
OsISM04442	LOC_Os01g34290	18903248	GCGGAAAATCCAACATCAGT	GTCGGCATCACCCTAACTGT	60.0	173	84	transposon protein putative CACTA En/Spm sub-class expressed
OsISM04443	LOC_Os01g34290	18903568	GAACGACGAGGTCCTCTCAC	CCCAAAATCCGTGTCGTACT	59.8	209	98	transposon protein putative CACTA En/Spm sub-class expressed
OsISM04444	LOC_Os01g34290	18904232	GGTGGACACAAGCCTCCTTA	CATGCTTCTGACACCATTGC	60.2	140	60	transposon protein putative CACTA En/Spm sub-class expressed
OsISM04445	LOC_Os01g34290	18904473	CGCCGAGACAAGGAGTACAT	TGTTATGACCAGAAGGATCCAA	59.9	151	67	transposon protein putative CACTA En/Spm sub-class expressed
OsISM04446	LOC_Os01g34290	18904826	TGTGCCACAATATGCGACTT	CCTCCTCAAAGCTGGTAGTCA	59.8	184	97	transposon protein putative CACTA En/Spm sub-class expressed
OsISM04447	LOC_Os01g34290	18904952	GGAATTATGACAATGTAGGGCA	CATCACAGTTGCATAATAAGTGTTCA	59.6	195	88	transposon protein putative CACTA En/Spm sub-class expressed
OsISM04448	LOC_Os01g34300	18906483	CTCATCGTCGATCTAGTGGG	GTAGGTGGAGGGCGACAAG	59.5	205	73	hypothetical protein
OsISM04449	LOC_Os01g34310	18909789	GGCTGCTTTTAAAGATCGGTT	CTAAACTTGGCAAATTCGGG	59.7	748	93	retrotransposon protein putative unclassified
OsISM04450	LOC_Os01g34310	18910516	TCCCGAATTTGCCAAGTTTA	GACCGGCCAAATCTCTATCA	60.2	365	94	retrotransposon protein putative unclassified
OsISM04452	LOC_Os01g34310	18912992	GGGAAACAAGACGCTACCAA	TGCACCCTTCTTTGTAACCA	59.6	385	79	retrotransposon protein putative unclassified
OsISM04453	LOC_Os01g34310	18914653	ATTACATATTTGAGCCGCCG	TAGCCAGATGCTTGTTGTGC	60.0	716	99	retrotransposon protein putative unclassified
OsISM04454	LOC_Os01g34310	18915436	ATTGGCAAAATTGGAGACCA	TTATAGGGTTCAACACGGCG	60.6	598	92	retrotransposon protein putative unclassified

ISM IDs	Rice gene locus IDs	Physical positions (bp) on chromosomes	Forward primers (5'-3')	Reverse primers (5'-3')	Melting tempearature (T˚C)	Expected product size (bp) in PCR	Expected product size (bp) in Q-PCR	Putative Functions
OsISM04455	LOC_Os01g34310	18916112	TCGTGCTAGTTGCTACGGATT	TTACTCGACTCGGCTTGTCC	60.2	256	98	retrotransposon protein putative unclassified
OsISM04456	LOC_Os01g34320	18920130	CCCATGAAGACCAGAGGAGA	TTTGCTACAACATCTGAAACATCA	59.9	308	89	hypothetical protein
OsISM04457	LOC_Os01g34330	18923063	CGCAAGCATTACACCTCCTT	TCTCCTTGCTATCCTCAGGC	59.9	203	86	BAH domain containing protein expressed
OsISM04458	LOC_Os01g34330	18923264	GAAACCTTATGTCGCCATCC	TTTTGTCAGCCTCTTCTGGTC	59.4	714	96	BAH domain containing protein expressed
OsISM04459	LOC_Os01g34330	18924557	ATCCTGGTGGTGCTACCAAA	GGCCATTATTGAAACAATCACA	60.0	1270	96	BAH domain containing protein expressed
OsISM04460	LOC_Os01g34330	18925851	GCTCTTGGTTCTGACTTCCAG	CATCAGCCGGTTCCGTAGTA	60.1	415	91	BAH domain containing protein expressed
OsISM04461	LOC_Os01g34330	18926267	ACAAGGAGCTTGATCCTCCA	TCTCTCTGCTGCTGTCAAGC	59.7	697	75	BAH domain containing protein expressed
OsISM04462	LOC_Os01g34330	18926944	GCTTGACAGCAGCAGAGAGA	CATCTTACACATCGTGCATCAG	59.4	217	77	BAH domain containing protein expressed
OsISM04463	LOC_Os01g34330	18927192	ATTCATGCTGGTCATGGAGA	CACTGTCAGGGTTGTGATGG	59.5	10368	94	BAH domain containing protein expressed
OsISM04465	LOC_Os01g34430	18978528	AGTGCCACCACTCTTCTCAA	GCTGTACAACTCCCCTTTGC	59.1	1005	99	expressed protein
OsISM04466	LOC_Os01g34430	18979499	TTGTCCAAAAGCATGCAAAG	TTGAAAGGAAGTGCAAGGGT	59.8	1239	78	expressed protein
OsISM04467	LOC_Os01g34430	18980716	CAACCCTTGCACTTCCTTTC	TTTCGAAATGTGGTCTTCCC	59.8	2164	69	expressed protein
OsISM04468	LOC_Os01g34430	18982983	TGCAGATCTCACAAAGCCAA	TTTGCCGAAAGGACCAGTAG	60.4	191	75	expressed protein
OsISM04469	LOC_Os01g34430	18983194	TGATCCTCGAAAAGACCCAG	AACCTACGCTCCTTCCTTGG	60.4	1724	94	expressed protein
OsISM04470	LOC_Os01g34430	18984878	CCCCTGAGCTGAGGAATCTT	CTTCGATGGCATCACTTCAA	60.3	638	84	expressed protein
OsISM04471	LOC_Os01g34430	18985562	AAGCGGAAATTCGAAAACCT	TCGGCTTACAAAACCAACCT	59.8	1009	67	expressed protein
OsISM04472	LOC_Os01g34430	18986679	AGAAGCAGGAAGCACTTTGC	CCAGCTTGATCCTCAGTTCC	59.8	482	86	expressed protein
OsISM04473	LOC_Os01g34430	18987187	CGGAGGATGATGAAGAGGAA	TCCAAGGTGAAATCAGGGAC	60.0	164	83	expressed protein

ISM IDs	Rice gene locus IDs	Physical positions (bp) on chromosomes	Forward primers (5'-3')	Reverse primers (5'-3')	Melting tempearature (T˚C)	Expected product size (bp) in PCR	Expected product size (bp) in Q-PCR	Putative Functions
OsISM04474	LOC_Os01g34430	18987331	GTCCCTGATTTCACCTTGGA	TTCATGCAGTGGAAAATTGC	59.8	847	94	expressed protein
OsISM04475	LOC_Os01g34450	18992327	GATCGATCAACAAGACTGATGC	TCCCAGATGCAATTCTGATG	59.7	1260	83	expressed protein
OsISM04476	LOC_Os01g34480	19008917	CTCGTCAGGCTGCTCCTC	GCACGCAGGTGTAGTGTTTT	59.3	482	87	NAD dependent epimerase/dehydratase family protein putative expressed
OsISM04477	LOC_Os01g34480	19009495	CCATACGGCTTCTCCCTTTT	GTTCCTTTGACTGCAGGGTC	60.1	790	73	NAD dependent epimerase/dehydratase family protein putative expressed
OsISM04478	LOC_Os01g34480	19010416	TTCCAGAGATTTGTGAAAAGCA	TGAAAACTTCCAAGCAGCCT	59.9	1086	78	NAD dependent epimerase/dehydratase family protein putative expressed
OsISM04479	LOC_Os01g34480	19011558	GCTGCAGCCTTCACTGAATA	TCCAGCCAAAGCTGAAATTA	58.8	852	90	NAD dependent epimerase/dehydratase family protein putative expressed
OsISM04480	LOC_Os01g34480	19012509	AGCTCGTCCAAATCATTCGT	TGGTAGATTGGCACTGATGG	59.6	321	86	NAD dependent epimerase/dehydratase family protein putative expressed
OsISM04481	LOC_Os01g34520	19031340	TACCTTGTTCTCCCGTGCTT	CTTGCTTCTTGCATGTCCG	60.1	128	75	hypothetical protein
OsISM04482	LOC_Os01g34520	19031487	CTATCGCTGCCTGAGAGGAC	ATATTCCCCTTGCCACATGA	60.1	167	65	hypothetical protein
OsISM04483	LOC_Os01g34550	19055330	GGGGTCCCTTTTATACCGTG	GAGACGTCGTTGCCTTTTTC	60.1	1542	69	retrotransposon protein putative unclassified
OsISM04484	LOC_Os01g34570	19061743	ACAACCCAAGGGATTTGTTG	CAACGATGTCTTAACGGATTCA	59.8	268	95	retrotransposon protein putative Ty1-copia subclass
OsISM04485	LOC_Os01g34610	19078320	CCGTGTCCTACTTGCAGGC	CTTCAACCCTCTCCAGCCAC	62.0	165	58	expressed protein
OsISM04486	LOC_Os01g34610	19078477	GGTTGAAGGCGAGGAAGG	CATGCCGTGTTTTAATGGAA	60.1	1125	99	expressed protein
OsISM04487	LOC_Os01g34610	19079672	GCATCTGAATGGATTTCCTGA	TTCATCTCTGCCATGTTTGC	59.9	506	97	expressed protein
OsISM04488	LOC_Os01g34610	19080935	CCCACAGGCCTTATTTCTCA	AACTAACCTTTACGGGGCGT	60.0	642	93	expressed protein
OsISM04489	LOC_Os01g34610	19081544	GCAAATACATGATACGCCCC	TCTTTCGCAGACGTCCTCTT	60.2	347	48	expressed protein
OsISM04490	LOC_Os01g34614	19084963	CTGAGCCCGTCAGCTATCTC	GAGGATGCGATAGCCACATT	60.1	524	96	DNA directed RNA polymerase 7 kDa subunit domain containing protein expressed
OsISM04491	LOC_Os01g34614	19085467	AATGTGGCTATCGCATCCTC	TTCCCTTATCTTCAGCGTGC	60.2	1554	74	DNA directed RNA polymerase 7 kDa subunit domain containing protein expressed

ISM IDs	Rice gene locus IDs	Physical positions (bp) on chromosomes	Forward primers (5'-3')	Reverse primers (5'-3')	Melting tempearature (T°C)	Expected product size (bp) in PCR	Expected product size (bp) in Q-PCR	Putative Functions
OsISM04492	LOC_Os01g34620	19089111	CAGGGTAGTCATGGCGAGAT	GGATAGTTGGTTGGGCCAC	60.1	999	99	OsGrx S15.1 - glutaredoxin subgroup II expressed
OsISM04493	LOC_Os01g34620	19090210	TTTCCAGCCTACAACCAAAA	CAGGACTGGGTTCTCCTTGA	59.2	212	77	OsGrx S15.1 - glutaredoxin subgroup II expressed
OsISM04494	LOC_Os01g34620	19090461	TTCAGTGCGTTGGCAGTTAG	GAGCTTGAGGTCTCCCAAAA	59.7	168	79	OsGrx S15.1 - glutaredoxin subgroup II expressed
OsISM04495	LOC_Os01g34620	19090612	TGGGAGACCTCAAGCTCAAA	TCAGATCCCCCAACAAACTC	60.4	605	95	OsGrx S15.1 - glutaredoxin subgroup II expressed
OsISM04496	LOC_Os01g34620	19091197	GAGTTTGTTGGGGGATCTGA	TTTTGTTCACGCTTTTGTGC	59.9	198	93	OsGrx S15.1 - glutaredoxin subgroup II expressed
OsISM04497	LOC_Os01g34700	19134975	GTTCCACATGGTCCAGATCC	CCACCACTCTTGCAAAACAA	60.0	1419	98	dienelactone hydrolase family protein expressed
OsISM04498	LOC_Os01g34700	19136419	ATGCTGTCCACATTTCCCA	ATGCTGAGCTTCCGCTACAT	60.0	728	96	dienelactone hydrolase family protein expressed
OsISM04499	LOC_Os01g34700	19137207	CTCAAGGCAAATGGATCACC	ACAGCATCAACCTCTGGGAC	60.3	838	99	dienelactone hydrolase family protein expressed
OsISM04500	LOC_Os01g34700	19138119	TTGGGGAGCTTGACAGTTTT	ACGCCAGATGACTTGAGCTT	59.9	361	80	dienelactone hydrolase family protein expressed
OsISM04501	LOC_Os01g34710	19141956	ATTCCAGGGCGGTCAAAC	GGTGCTAAGGCCTATCCTGG	60.9	680	71	transposon protein putative unclassified
OsISM04502	LOC_Os01g34710	19142721	TCTCTCCAGATACCATGCCT	TGATCTCGACTGGCTGAATG	58.6	1401	83	transposon protein putative unclassified
OsISM04503	LOC_Os01g34710	19144102	CATTCAGCCAGTCGAGATCA	TTTCATTGCAACCAACAACC	59.7	418	86	transposon protein putative unclassified
OsISM04504	LOC_Os01g34710	19144557	ACAACTAAAGGGTGGCCATT	TTTGATCCATCAAGAAGCTGAA	59.1	2145	88	transposon protein putative unclassified
OsISM04505	LOC_Os01g34720	19152733	GTGCGTAACATTCAGCCAAA	CAAACTGCTCCCTCATCACA	59.8	223	98	retrotransposon protein putative unclassified
OsISM04506	LOC_Os01g34730	19158303	AATGGATGGTGGACAGAACC	TTAACATGCCACAGGCAGTC	59.7	332	77	retrotransposon protein putative Ty3-gypsy subclass
OsISM04507	LOC_Os01g34730	19158677	TAGTTGTTGGCCTACCTGGC	TGCCTAACTCTTCAGCCGAT	60.1	257	82	retrotransposon protein putative Ty3-gypsy subclass
OsISM04508	LOC_Os01g34730	19159047	TTGAAGAAGCCGATGAAGGT	AGAACTCCCTGCATCATTGC	60.0	291	99	retrotransposon protein putative Ty3-gypsy subclass
OsISM04509	LOC_Os01g34730	19159226	TGGTTTTGATCATCATTGGG	TGAAAACGCTTCCTGTTCCT	59.5	303	98	retrotransposon protein putative Ty3-gypsy subclass

ISM IDs	Rice gene locus IDs	Physical positions (bp) on chromosomes	Forward primers (5'-3')	Reverse primers (5'-3')	Melting tempearature (T˚C)	Expected product size (bp) in PCR	Expected product size (bp) in Q-PCR	Putative Functions
OsISM04510	LOC_Os01g34730	19160160	TGGCAAGGTGACAAGATTGA	AGACATTTGAACCCTCCACG	60.1	215	98	retrotransposon protein putative Ty3-gypsy subclass
OsISM04511	LOC_Os01g34730	19160355	CGTGGAGGGTTCAAATGTCT	GAATGTTGGCCTTGGTCTGT	60.0	133	74	retrotransposon protein putative Ty3-gypsy subclass
OsISM04512	LOC_Os01g34750	19165070	GTCACGTCTTCCGTCCGTAT	CATGTCCATACCCCATCCTC	60.0	857	54	retrotransposon protein putative Ty3-gypsy subclass
OsISM04513	LOC_Os01g34800	19212817	GAGGCAGCGGATAGTCACAT	CTCCATGCGAAGACGTCAG	60.4	159	85	retrotransposon protein putative unclassified
OsISM04514	LOC_Os01g34810	19218910	ATCGATAAGCACTCCGCACT	AGAGCTTGATGCCCATGTCT	59.9	142	89	retrotransposon protein putative unclassified
OsISM04515	LOC_Os01g34850	19242085	GCCAGCGGCTTTAGTACTATTC	GCCGATTCGTTTGTGATCTT	59.8	174	89	OsHKT2;3 - Na+ transporter expressed
OsISM04516	LOC_Os01g34850	19242374	CACTCTCCACTGACCCTCTCA	CAGCTGTAGCCAGTGGACAA	60.2	198	95	OsHKT2;3 - Na+ transporter expressed
OsISM04517	LOC_Os01g34870	19251858	GAACACTGTGACCCAATGGA	TCAAGACCTTCAGCAGACTCAA	59.8	476	84	expressed protein
OsISM04518	LOC_Os01g34870	19252389	TCTGCAAAAACAAATGCCTG	GGCGTCTGAATCATCAGGTT	60.0	180	97	expressed protein
OsISM04519	LOC_Os01g34870	19252716	AAACTTCCAGAGGATTGCCA	TTTTCCTTCTCCGAGCCAG	60.1	166	89	expressed protein
OsISM04520	LOC_Os01g34870	19252855	TGTAGTGTCTGGCTCGGAGA	ATCACCATCCCAAGGTTCAG	59.7	1073	90	expressed protein
OsISM04521	LOC_Os01g34870	19253968	CCGATGTAGAGGATGCAGTAGA	GGCATGAACATCGACTTGTTT	59.7	1001	99	expressed protein
OsISM04522	LOC_Os01g34870	19254962	TCATGCCTTGAAAGAAGTGC	CAGGGGTGATTCTGTGTCCT	59.5	981	89	expressed protein
OsISM04523	LOC_Os01g34960	19344642	TCATCTGCAACAGTTCCTGG	CGTAACGTGTGAGTCCAAGG	59.5	613	94	retrotransposon putative centromere-specific
OsISM04524	LOC_Os01g34960	19345346	GAGGTCAAGGGTGGAATCAA	TTTTGCTTAGCGCATCAGTG	60.0	129	98	retrotransposon putative centromere-specific
OsISM04525	LOC_Os01g34970	19352091	GGATGTCAGGGGACACATTC	TGCAATAATGAAGCCTCCAA	59.7	841	99	MDR-like ABC transporter putative expressed
OsISM04526	LOC_Os01g34970	19353032	CCCGCATGCAAGAAAAGTAT	ATAGCTTGCTTCTCGCCATT	59.6	204	98	MDR-like ABC transporter putative expressed
OsISM04527	LOC_Os01g34970	19353381	AACCGTGGATACAATGGTGG	TGATGGTGTTGCCTGACCTA	60.3	589	85	MDR-like ABC transporter putative expressed

ISM IDs	Rice gene locus IDs	Physical positions (bp) on chromosomes	Forward primers (5'-3')	Reverse primers (5'-3')	Melting tempearature (T°C)	Expected product size (bp) in PCR	Expected product size (bp) in Q-PCR	Putative Functions
OsISM04528	LOC_Os01g34970	19354431	GCTTGCAAATGCAGCAAAAT	CAATTGAATTCCACGTTCCC	60.7	160	77	MDR-like ABC transporter putative expressed
OsISM04529	LOC_Os01g34970	19354800	ACAGCAGGGAAAGATGGTTG	GCTGAGCATAAGCACCTTCA	59.6	455	72	MDR-like ABC transporter putative expressed
OsISM04530	LOC_Os01g34970	19355819	GAAGTGTCATGCACCAGGAA	CAGAGCATCAACAGACAGCC	59.6	810	87	MDR-like ABC transporter putative expressed
OsISM04531	LOC_Os01g34970	19356796	GAAAGGTTTCAACAAAAACGC	CACAGTTCTGATGCCTCCAA	59.3	180	83	MDR-like ABC transporter putative expressed
OsISM04532	LOC_Os01g34970	19357156	GTGGCAACGTTTCCTGAAGT	GCCTATTGCACTTGTTCGTG	59.8	160	66	MDR-like ABC transporter putative expressed
OsISM04533	LOC_Os01g34990	19363161	AAGGGAGAGACCACAGCCTC	CGGTGAGGGTGGGAACAT	61.3	191	89	conserved hypothetical protein
OsISM04536	LOC_Os01g35030	19379777	GGAGCAGGACTTGTTTCAGC	ATACAACGCCCTAATCCGTG	59.9	286	82	MDR-like ABC transporter putative expressed
OsISM04537	LOC_Os01g35030	19380152	CCTCATTCAAGATGCCATTG	AACGCAATAATGAAGCCTCC	59.1	769	82	MDR-like ABC transporter putative expressed
OsISM04538	LOC_Os01g35030	19381048	GGAATCGTGGTCGAACAAAC	ATAGCTTTCTTCTCACCATTGAA	58.8	163	69	MDR-like ABC transporter putative expressed
OsISM04539	LOC_Os01g35030	19381345	GATTGATAGTCGAGCGAGGC	TTGATGATGTCGCATGACCT	60.0	727	97	MDR-like ABC transporter putative expressed
OsISM04540	LOC_Os01g35030	19382534	TTGCAAATGCAGCAAAGTTC	AGTTGAATTCCACGCTCACC	60.1	150	74	MDR-like ABC transporter putative expressed
OsISM04541	LOC_Os01g35030	19382860	GCACAGTAAAGAACGCCGAT	AACCTTCAGGCTTCTTCATCA	59.6	545	94	MDR-like ABC transporter putative expressed
OsISM04542	LOC_Os01g35030	19383985	TGTCATGTACCAGGAGATCAGC	GCTTCACGTTCAAAGCATCA	60.1	511	93	MDR-like ABC transporter putative expressed
OsISM04543	LOC_Os01g35030	19384635	TGCTCAAATGATGTTCCTGA	GGTTCTGATGCCTCCAACTG	59.2	191	98	MDR-like ABC transporter putative expressed
OsISM04544	LOC_Os01g35030	19384996	TGTTCACCAAGGAACAGCAA	TGATCCTATTGCACTTGTTCG	59.5	217	98	MDR-like ABC transporter putative expressed
OsISM04545	LOC_Os01g35030	19385317	CAGGGGTGACATTGAGTTCC	AGACTTCCCGCTTCCACTCT	60.4	231	62	MDR-like ABC transporter putative expressed
OsISM04547	LOC_Os01g35040	19390885	TCCATGAGCCACTACAATGC	GCAGGTTGAGGATTGGTGTT	59.8	424	75	ZOS1-09 - C2H2 zinc finger protein expressed
OsISM04548	LOC_Os01g35080	19423705	GAAGGAGATCATCGGGTGG	TCGCAGGCATGAATGAAAT	60.3	213	99	retrotransposon protein putative unclassified

ISM IDs	Rice gene locus IDs	Physical positions (bp) on chromosomes	Forward primers (5'-3')	Reverse primers (5'-3')	Melting tempearature (T˚C)	Expected product size (bp) in PCR	Expected product size (bp) in Q-PCR	Putative Functions
OsISM04549	LOC_Os01g35080	19424445	CATGTGCTGCCAGTTGTACC	ATTGGGGGATCCGACTTTAT	59.8	166	78	retrotransposon protein putative unclassified
OsISM04550	LOC_Os01g35080	19424685	CCAACACCTCTAATCCAGCC	CGGATCAGAGTCCTTTCCAG	59.7	304	99	retrotransposon protein putative unclassified
OsISM04551	LOC_Os01g35080	19425263	AGGCATCTGAGTCCACTCGT	TCCGGAGAATTTCCTCTTCA	59.8	155	76	retrotransposon protein putative unclassified
OsISM04552	LOC_Os01g35080	19425467	TGACGACTTGCAACCTCAAG	CAGTCTTCGCCTCGTTCCTA	60.3	207	84	retrotransposon protein putative unclassified
OsISM04553	LOC_Os01g35170	19465471	AGTACGCCGACCTCAACCT	CACCGAGAAAGAGGAGCTGA	60.2	199	96	tRNA methyltransferase putative expressed
OsISM04554	LOC_Os01g35170	19465644	ACCCGCTCAGCTCCTCTT	TCGAACACCTCCTTCCACTC	60.2	155	59	tRNA methyltransferase putative expressed
OsISM04555	LOC_Os01g35170	19465783	GGAAGGAGGTGTTCGAGGAT	AGCCAAATCAAGAACCTCCC	60.4	193	77	tRNA methyltransferase putative expressed
OsISM04556	LOC_Os01g35170	19466002	ACTACCTCGGCCTGGAGATT	CCAACTCGGTTACCCAGAAC	59.8	173	64	tRNA methyltransferase putative expressed
OsISM04557	LOC_Os01g35170	19466155	GTTCTGGGTAACCGAGTTGG	CGCAATCTGGTTGAAAGACA	59.6	172	77	tRNA methyltransferase putative expressed
OsISM04558	LOC_Os01g35170	19466337	CTGGTCCCCTGTCACTTGTT	CCACCAACTGCGATTGTAGA	59.9	212	91	tRNA methyltransferase putative expressed
OsISM04559	LOC_Os01g35170	19466530	CTACAATCGCAGTTGGTGGA	AGCCACTTCGAGTACATCCG	60.0	458	85	tRNA methyltransferase putative expressed
OsISM04560	LOC_Os01g35200	19479629	TGATTTTTATAGAGGTTCGGGC	GGAGTTTCGCATAAGGAAGG	59.1	502	66	transposon protein putative CACTA En/Spm sub-class
OsISM04561	LOC_Os01g35200	19480111	CCTTCCTTATGCGAAACTCC	CCCTGTTGTAGCCTTCTCACA	59.5	2035	84	transposon protein putative CACTA En/Spm sub-class
OsISM04562	LOC_Os01g35200	19482097	TGTTCTCGTCCTATCCAGGAG	TGTGCCAGTAGTGTGGGCTA	59.8	244	69	transposon protein putative CACTA En/Spm sub-class
OsISM04563	LOC_Os01g35200	19482361	ATGGCTGATACCCCCTACCT	CGTTCTCCGCATCTCATACA	59.7	843	76	transposon protein putative CACTA En/Spm sub-class
OsISM04565	LOC_Os01g35210	19488301	GCGATGATGATCCGGACTAC	TTGCTCTGTCCTGTTCCTCC	60.4	182	90	transposon protein putative CACTA En/Spm sub-class
OsISM04566	LOC_Os01g35210	19490639	TAATCTCGTTGCGGCTTTCT	CGACGTATCCAACCTTTGCT	60.1	159	72	transposon protein putative CACTA En/Spm sub-class
OsISM04567	LOC_Os01g35210	19490973	TGTCTTAATGGTCCCCTACCA	TGATCAATTGGTCTTTCGGA	59.2	154	72	transposon protein putative CACTA En/Spm sub-class

ISM IDs	Rice gene locus IDs	Physical positions (bp) on chromosomes	Forward primers (5'-3')	Reverse primers (5'-3')	Melting tempearature (T˚C)	Expected product size (bp) in PCR	Expected product size (bp) in Q-PCR	Putative Functions
OsISM04568	LOC_Os01g35210	19491122	GATCATATCATTGGACCCCG	TCCACCTCTTTTGCGGTAGT	59.9	189	98	transposon protein putative CACTA En/Spm sub-class
OsISM04569	LOC_Os01g35210	19491291	ACTACCGCAAAAGAGGTGGA	AAGTTGGTTCCTGGTGGTTG	59.8	194	98	transposon protein putative CACTA En/Spm sub-class
OsISM04570	LOC_Os01g35210	19491498	GTGTGCGAGATGCTCAGAGT	GTGTGGTTGAATCGTTGTGC	59.6	179	90	transposon protein putative CACTA En/Spm sub-class
OsISM04571	LOC_Os01g35240	19508962	GGAACCTTCGAAGTGCAGAG	GTCATGGCAACGAGTCTTCA	59.9	579	97	hypothetical protein
OsISM04572	LOC_Os01g35240	19509567	AAGCATCCTGAGGAAAGCAA	TCGTAGAGGACACGGTCGAT	60.3	1094	76	hypothetical protein
OsISM04573	LOC_Os01g35290	19536288	TGTTCCTGTTGAGAGTCAGGAG	TTTTTGGGCTTATCTTTTGC	58.3	180	73	retrotransposon protein putative Ty1-copia subclass
OsISM04574	LOC_Os01g35310	19541407	GGAGGAGGTTGGAGAGGAAG	TCTTGCAAAGAAGCTGTGGA	60.0	423	67	hypothetical protein
OsISM04575	LOC_Os01g35310	19541920	CTCGGCTATGAAGGTCTTGG	GAGTAGTGAACGTGCCCCTC	59.8	775	72	hypothetical protein
OsISM04576	LOC_Os01g35310	19543027	TCGACACTCTTCTTGAGGCA	TCCATCCGTATAATCGTCAGC	59.8	154	74	hypothetical protein
OsISM04577	LOC_Os01g35320	19546510	AGAGGAGAGGGGAGGGGTAG	AGTATGATTTTTGCAGGCGG	60.5	537	93	expressed protein
OsISM04578	LOC_Os01g35380	19569537	GTTATTCACATTTGCTGCCG	TGGGTCGACATTGTCAGTTG	59.9	633	99	retrotransposon protein putative Ty3-gypsy subclass
OsISM04579	LOC_Os01g35380	19570367	TCACGCTCAACCAGAGACTG	GCGCACTTCTTCGTCTTTCT	60.0	247	89	retrotransposon protein putative Ty3-gypsy subclass
OsISM04580	LOC_Os01g35380	19570612	GCCAGCTGAAGAAAGCACTC	GTTGTGGTTTTGGCCTCATT	60.1	154	77	retrotransposon protein putative Ty3-gypsy subclass
OsISM04581	LOC_Os01g35380	19570851	GAACACGATCTAAGGGACCG	CCAGTTGAGCTATCTTGGCA	59.3	168	95	retrotransposon protein putative Ty3-gypsy subclass
OsISM04582	LOC_Os01g35380	19571073	ACTCGGCTTTGAAGGAGGTT	ACTGACCAGGACGTCGAACT	60.0	139	66	retrotransposon protein putative Ty3-gypsy subclass
OsISM04583	LOC_Os01g35410	19587988	AGAGAAGAAGAGGCGAAGCC	ACGTTTCCTGAGCCATGGTA	60.4	358	93	transposon protein putative unclassified
OsISM04584	LOC_Os01g35410	19588452	AGGGGGTTGAGAGTTGATCC	ATCTTTCGGGTGAACAAACG	60.1	186	85	transposon protein putative unclassified
OsISM04585	LOC_Os01g35410	19589063	CAACCGGTCGCAATATGAAG	CACTCTTGCCAGGATGTTCA	60.6	438	84	transposon protein putative unclassified

ISM IDs	Rice gene locus IDs	Physical positions (bp) on chromosomes	Forward primers (5'-3')	Reverse primers (5'-3')	Melting tempearature (T˚C)	Expected product size (bp) in PCR	Expected product size (bp) in Q-PCR	Putative Functions
OsISM04586	LOC_Os01g35410	19589686	ACCGACAGCTGGTACTGGTT	TGTCCAAGAGCTTGAGGAGG	60.1	381	93	transposon protein putative unclassified
OsISM04587	LOC_Os01g35420	19592959	ACAACGACGACGACGAGAAC	TGTGAACACCCCAGCTATTG	60.4	1009	98	transposon protein putative unclassified
OsISM04588	LOC_Os01g35420	19594177	AGGATCAGCAGCAGATGGAG	CGTAAGTAGCTCACCGCACA	60.3	729	80	transposon protein putative unclassified
OsISM04589	LOC_Os01g35420	19594886	TGTGCGGTGAGCTACTTACG	AGCCGACACAACGGTAGG	59.9	153	77	transposon protein putative unclassified
OsISM04590	LOC_Os01g35420	19595242	CTGTGGACTGGAAGGACGAT	GAGTACTCGTCGGCCTCATC	60.0	243	74	transposon protein putative unclassified
OsISM04591	LOC_Os01g35420	19595836	GATGGGTACTCTGTGGTGCC	AAGGCGATCGAACTCCATAA	60.0	162	92	transposon protein putative unclassified
OsISM04592	LOC_Os01g35420	19596182	AGCCCTTTCCAGTACCGTTT	CAGTCGCCAACTAACTGCTG	59.8	189	83	transposon protein putative unclassified
OsISM04593	LOC_Os01g35420	19596566	CAGGTACCACGACCAGGACT	AACGACGACCCTGTTGATGT	60.2	149	56	transposon protein putative unclassified
OsISM04594	LOC_Os01g35430	19601060	ACTGGTGTCCCCGCCTATAC	GATCCATTCCTGGAGCGTAA	60.6	1673	94	retrotransposon protein putative unclassified
OsISM04595	LOC_Os01g35440	19610308	GGCTAGGATGGGTTGGAAAC	CGAAACCAGTCATGTCCAAA	60.1	1034	96	retrotransposon protein putative Ty3-gypsy subclass
OsISM04596	LOC_Os01g35450	19615318	GTTGCTCACCTTCCGTCCT	GCCATCACCGGTATTAGCAC	60.3	324	56	retrotransposon protein putative Ty3-gypsy subclass
OsISM04597	LOC_Os01g35450	19616210	AAGATGTGCGGGAAGATGAG	TAGGGATTGGTGTAGCGGAC	60.1	226	98	retrotransposon protein putative Ty3-gypsy subclass
OsISM04598	LOC_Os01g35460	19622483	TATGGCTAGGATGGGTTGGA	TCTAGGGGCAGAGGTTGATTT	60.2	375	77	retrotransposon protein putative unclassified
OsISM04599	LOC_Os01g35470	19624983	ATAATGCATTCAATCCGCCT	ACTTCCCTGAATGGTGTGCT	59.5	187	99	retrotransposon protein putative Ty3-gypsy subclass
OsISM04600	LOC_Os01g35470	19625837	ATGTTCAGCCAGGAACATCC	CGCCTTTATGCGAGGAATTA	60.1	142	66	retrotransposon protein putative Ty3-gypsy subclass
OsISM04601	LOC_Os01g35470	19626089	GAAAGCACTTGGAGAAGCCA	GTTCCCAAATGAGGAACCAG	59.9	174	97	retrotransposon protein putative Ty3-gypsy subclass
OsISM04602	LOC_Os01g35470	19626350	CCAGGATTCAAACTTCGAGC	TCCATCTGAGCCATTCTGGT	60.2	138	64	retrotransposon protein putative Ty3-gypsy subclass
OsISM04603	LOC_Os01g35470	19626532	CAGAAAGGGACTCAGCCTTG	GCCTCTTGATCTTACCAACCA	59.6	162	88	retrotransposon protein putative Ty3-gypsy subclass

ISM IDs	Rice gene locus IDs	Physical positions (bp) on chromosomes	Forward primers (5'-3')	Reverse primers (5'-3')	Melting tempearature (T˚C)	Expected product size (bp) in PCR	Expected product size (bp) in Q-PCR	Putative Functions
OsISM04604	LOC_Os01g35540	19661001	AAAACTGAGCCCCAACCTTT	AACTGATGAGTTGCGAAGCC	60.2	133	75	retrotransposon protein putative unclassified
OsISM04605	LOC_Os01g35580	19683456	TGCAACAAATGCTCTACCCA	CGCTTGATATGGGACTCGTT	60.2	159	87	expressed protein
OsISM04606	LOC_Os01g35590	19688468	AAGGAGGAGGGCGAGGAT	GATCGGCTGCACCAAATG	61.2	911	62	hypothetical protein
OsISM04607	LOC_Os01g35590	19689591	CGTCGCCATTTCTCTCTCTC	GAGGTCTTGGCGATGGAG	59.7	173	93	hypothetical protein
OsISM04608	LOC_Os01g35590	19689744	CTCTCCATCGCCAAGACCT	TTTACCACCATGATGACCCA	60.0	1788	90	hypothetical protein
OsISM04609	LOC_Os01g35590	19691498	CTATGGTCCTTGGGTGGGT	TTAATACTCCATAGTGATCTCATAGCA	58.7	412	63	hypothetical protein
OsISM04610	LOC_Os01g35610	19709043	TGAAGAAGACGGTAGCCGAT	ACATCGTCGTCGTTCCGA	60.6	150	85	hypothetical protein
OsISM04611	LOC_Os01g35640	19739802	GAAGACTCGGAGGAAGACCC	ATTGTTGGCAGACCCACTTC	60.1	423	89	retrotransposon protein putative Ty3-gypsy subclass
OsISM04612	LOC_Os01g35640	19740806	TTCCAGAAGTTGGTGGACAA	TGTCCATAGCTGTTGGTGAGTT	59.4	797	93	retrotransposon protein putative Ty3-gypsy subclass
OsISM04613	LOC_Os01g35640	19742168	CCCGCTTTCTTCATGAACTT	GCTCACATTCTGCTGACCAC	59.4	426	98	retrotransposon protein putative Ty3-gypsy subclass
OsISM04614	LOC_Os01g35650	19745234	CTATGGCTAGGATGGGTTGG	ACCAGTCAGGTCCAGACCAC	59.7	1033	96	retrotransposon protein putative Ty3-gypsy subclass
OsISM04616	LOC_Os01g35660	19750229	GACAGTGGGGCATGGTTTAT	ACAGCACTTCTCCTGGTCGT	59.8	220	48	retrotransposon protein putative Ty3-gypsy subclass
OsISM04617	LOC_Os01g35660	19752340	CCCTGGATCGATGATCTGTT	TACAATTGATGTTCCCGCAA	59.9	338	99	retrotransposon protein putative Ty3-gypsy subclass
OsISM04618	LOC_Os01g35680	19759936	TCTGAGTTGCTTGTTCGTGG	TTCGGAGTCAACCTCAGACA	59.7	422	98	retrotransposon protein putative unclassified
OsISM04619	LOC_Os01g35690	19762885	TCGAGATGTCTAGGCAAATGAA	GTTGGCCTTACCAGGATGAT	59.4	1212	96	retrotransposon protein putative Ty3-gypsy subclass
OsISM04620	LOC_Os01g35690	19764343	GAACACGGAACCCTATGGAA	TGGAGAGGTTGGAGAAGTCC	59.5	257	78	retrotransposon protein putative Ty3-gypsy subclass
OsISM04621	LOC_Os01g35730	19791690	ATAATGCATTCAATCCGCCT	ACTTCCCTGAATGGTGTGCT	59.5	187	99	retrotransposon protein putative Ty3-gypsy subclass
OsISM04622	LOC_Os01g35730	19792526	ATGTTCAGCCAGGAACATCC	CGCCTTTATGCGAGGAATTA	60.1	142	66	retrotransposon protein putative Ty3-gypsy subclass

ISM IDs	Rice gene locus IDs	Physical positions (bp) on chromosomes	Forward primers (5'-3')	Reverse primers (5'-3')	Melting tempearature (T°C)	Expected product size (bp) in PCR	Expected product size (bp) in Q-PCR	Putative Functions
OsISM04623	LOC_Os01g35730	19792778	GAAAGCACTTGGAGAAGCCA	GTTCCCAAATGAGGAACCAG	59.9	174	97	retrotransposon protein putative Ty3-gypsy subclass
OsISM04624	LOC_Os01g35730	19793039	CCAGGATTCAAACTTCGAGC	ATCTGTTTCTCCATCTGGGC	59.5	142	73	retrotransposon protein putative Ty3-gypsy subclass
OsISM04625	LOC_Os01g35730	19793203	GTAGAACTGGCTGCGGAAAG	CAACCAGGACGTCAAACTGA	59.9	160	86	retrotransposon protein putative Ty3-gypsy subclass
OsISM04627	LOC_Os01g35770	19811580	CGCCTTGCAATTAAAGAAGC	GACGTTACGAACGACTGGGT	60.0	316	92	retrotransposon protein putative Ty3-gypsy subclass
OsISM04628	LOC_Os01g35770	19812531	TGCGTGGAAACCATCTGATA	ACTGCCCTGTCTTTTTCCG	60.2	233	78	retrotransposon protein putative Ty3-gypsy subclass
OsISM04629	LOC_Os01g35770	19813264	GCCATCCTCAACATGAAACC	TATGTGACCACCGTAACCGA	60.1	469	84	retrotransposon protein putative Ty3-gypsy subclass
OsISM04630	LOC_Os01g35770	19814643	GTCAGCAAAGCATACCGACA	CCGAAGTGATGCACAATGTT	59.7	301	90	retrotransposon protein putative Ty3-gypsy subclass
OsISM04631	LOC_Os01g35770	19814942	GGCGTGCCAAATCGTATTAT	GCTCAACCTGGCCATTACTC	59.8	136	77	retrotransposon protein putative Ty3-gypsy subclass
OsISM04632	LOC_Os01g35770	19815135	CTTATGCTGGCAAATGGGTT	CTTCCGTAATCTGAGGCGAA	60.2	962	56	retrotransposon protein putative Ty3-gypsy subclass
OsISM04633	LOC_Os01g35770	19816161	CGTGTCGGTTTACGTATCTCC	TGTCGACGACTGATACTCGC	59.8	395	89	retrotransposon protein putative Ty3-gypsy subclass
OsISM04634	LOC_Os01g35770	19816728	CTACTGGCCCGATCATCATT	GAGGGTCCTGAGGAAGATCC	60.0	395	80	retrotransposon protein putative Ty3-gypsy subclass
OsISM04635	LOC_Os01g35789	19824004	GCGTGCAAGTCATCTCTGAA	AGCTGACTTCACCAACCGAT	59.9	499	74	expressed protein
OsISM04636	LOC_Os01g35800	19826706	GCCACCCCTTATTCTGTGAG	GAAGGAGGAGACGATGGTGA	59.9	201	97	retrotransposon protein putative unclassified
OsISM04637	LOC_Os01g35800	19827377	AAAACTGGAACGCAAGTTCG	TCGTCTCTATGATTGCAGCG	60.2	239	81	retrotransposon protein putative unclassified
OsISM04638	LOC_Os01g35800	19827795	GCTCTTCTCCTCGACATCCA	CTGGCAACTTGGTGTGTTTG	60.3	175	86	retrotransposon protein putative unclassified
OsISM04639	LOC_Os01g35840	19849545	ATTCTGCAGCAGTAGATGGC	ATCTGCCAGAGGACGAAGC	59.3	346	99	hypothetical protein
OsISM04641	LOC_Os01g35860	19855246	TGCAAGTGGAGACTTCATGC	CCTTGCCTCCTAGTGCTCTG	60.1	183	99	transposon protein putative Mutator sub-class
OsISM04642	LOC_Os01g35860	19855537	CCATAAAATTGTGGTGCCCT	TGTCCCCTAAACCTGCTTGT	59.6	291	94	transposon protein putative Mutator sub-class

ISM IDs	Rice gene locus IDs	Physical positions (bp) on chromosomes	Forward primers (5'-3')	Reverse primers (5'-3')	Melting tempearature (T˚C)	Expected product size (bp) in PCR	Expected product size (bp) in Q-PCR	Putative Functions
OsISM04643	LOC_Os01g35860	19856932	TATAGCTGCAATGGAGGCAG	GGGAGGGATTAGCCAACACTA	59.7	182	99	transposon protein putative Mutator sub-class
OsISM04644	LOC_Os01g35860	19857237	CAACCACAACCTCAGAACCA	AGGTGCCACACATTGCAGTA	59.9	181	90	transposon protein putative Mutator sub-class
OsISM04645	LOC_Os01g35890	19868497	CCTCAGCCATGAGTTCCACT	TACCGGCTCGCCATAATAAG	60.2	237	99	retrotransposon protein putative Ty1-copia subclass
OsISM04646	LOC_Os01g35890	19868705	TGCTGTTGACTTATTATGGCG	CGTAGGATCAGAAACGGGAG	59.3	115	50	retrotransposon protein putative Ty1-copia subclass
OsISM04648	LOC_Os01g35920	19875183	ATGTGGTCAAGGGAGGACTG	GCAGGAGCAGTTCCTTTGTG	60.5	147	92	conserved hypothetical protein
OsISM04649	LOC_Os01g35920	19875401	TAGCAACATGTCAAACCCCA	TCCCTGGAAGTATCAGCACC	60.0	795	64	conserved hypothetical protein
OsISM04650	LOC_Os01g35920	19876153	GGCCTGATCCACAGATGAAT	GTCCTGATCCACCTAGCAGC	59.9	199	86	conserved hypothetical protein
OsISM04651	LOC_Os01g35920	19876331	AGCTGCTAGGTGGATCAGGA	GCTTGCCATCTTGTATGTGAA	59.4	172	88	conserved hypothetical protein
OsISM04652	LOC_Os01g35920	19876453	CCCCCAAGAAAGGATGAACT	AACTTGAAAGCACGAGCATGT	60.1	465	87	conserved hypothetical protein
OsISM04653	LOC_Os01g35920	19876898	CATGCTCGTGCTTTCAAGTT	TACCTATTGAGTGCATGCGG	59.4	323	80	conserved hypothetical protein
OsISM04654	LOC_Os01g35920	19877194	GCAGTAACCGCATGCACTC	GTCCATGGAGAAGGTGCAAT	60.2	419	92	conserved hypothetical protein
OsISM04655	LOC_Os01g35920	19877580	GGCATCTGATGTTATTGCACC	CCACCATCTTTTGCCTCAGT	60.2	154	85	conserved hypothetical protein
OsISM04656	LOC_Os01g35920	19877714	ACTGAGGCAAAAGATGGTGG	CATGCTGAGAATCAGGCAAA	60.0	527	86	conserved hypothetical protein
OsISM04657	LOC_Os01g35930	19883371	GAACCGACCAGACAATCGTT	CAGCATTGGATAGGCGAAGT	60.1	232	75	retrotransposon protein putative unclassified
OsISM04658	LOC_Os01g35930	19883622	CATGATGGCACATCCACTTC	GCAGAAACACATTTCGCTGA	60.0	152	61	retrotransposon protein putative unclassified
OsISM04659	LOC_Os01g35930	19883874	CAACATTCATGAAGGAAGGGA	CGCGGTTTTACCTGCACTA	59.9	818	87	retrotransposon protein putative unclassified
OsISM04660	LOC_Os01g35930	19884611	CATGTCGTTCCTACACAGATGC	ATACGTCTGGTTGCCCATTT	59.9	371	95	retrotransposon protein putative unclassified
OsISM04661	LOC_Os01g35950	19892622	GAATCGTGGCGTGAGTACG	ATATTCGGCGTCGTCACATT	60.3	550	94	hypothetical protein

ISM IDs	Rice gene locus IDs	Physical positions (bp) on chromosomes	Forward primers (5'-3')	Reverse primers (5'-3')	Melting tempearature (T°C)	Expected product size (bp) in PCR	Expected product size (bp) in Q-PCR	Putative Functions
OsISM04662	LOC_Os01g35950	19893204	GTCGGGGACATATGGGAAG	TTCTTCGTAGCCGGTGTTCT	60.0	457	80	hypothetical protein
OsISM04663	LOC_Os01g35970	19902349	TATGCATCTGACCACCAAGG	GGAAAGGCATTATGGTTGTGA	59.7	963	96	transposon protein putative Mariner sub-class
OsISM04664	LOC_Os01g35970	19903326	TTGATGACGATGAGGAAGACC	GCATACACGCCGTACTCTTG	59.7	585	59	transposon protein putative Mariner sub-class
OsISM04665	LOC_Os01g35970	19904123	GAAGAGTGAAGCAATGCCGT	TTGGTGACTAGAGTCCCCCTT	60.2	689	92	transposon protein putative Mariner sub-class
OsISM04666	LOC_Os01g36000	19922153	TTCATGGCTGACAACGAAAC	AGGAGGGTGTCGAGGAGAGT	60.0	307	99	retrotransposon protein putative Ty3-gypsy subclass
OsISM04667	LOC_Os01g36000	19922964	CAAGGCCATGGCAAACTATC	GAGGTCAAACTGTGTGCCG	60.4	163	59	retrotransposon protein putative Ty3-gypsy subclass
OsISM04668	LOC_Os01g36010	19925631	CGAGGTTTTAGCCAACTCCA	CGTGACCGAGTGACTTTGAA	60.1	112	62	retrotransposon protein putative Ty3-gypsy subclass
OsISM04669	LOC_Os01g36010	19926047	TTTCTGCATCCCATAACGTG	CTCTTGTCGATAAGCGGTCA	59.3	173	84	retrotransposon protein putative Ty3-gypsy subclass
OsISM04670	LOC_Os01g36050	19949239	ATTTGGCTGTCCCAAGAAGA	CCTTTCTGTCTGACCATCGG	60.2	142	87	retrotransposon protein putative Ty3-gypsy subclass
OsISM04671	LOC_Os01g36070	19958827	ACGACGTCTGCTGCTATGG	CTTACAGCGGGGAGATGAAG	59.9	269	73	nodulin MtN3 family protein putative expressed
OsISM04672	LOC_Os01g36070	19959061	CTTCGCTCTTGTGCTCTTCA	CGAGTAAATGTACGGCATCG	59.3	224	98	nodulin MtN3 family protein putative expressed
OsISM04673	LOC_Os01g36070	19959375	CTCTTCCAGCTCGCCTACAC	GAACACCATAACCAGCAGGC	60.3	566	88	nodulin MtN3 family protein putative expressed
OsISM04674	LOC_Os01g36070	19960034	ATATTCATGTTTGCGTCCCC	TACTCCACGCTCTTCGTCCT	59.8	240	63	nodulin MtN3 family protein putative expressed
OsISM04675	LOC_Os01g36070	19960310	GAGCGTATCGTTCTTCGCAT	CACCAATTGAATGACACCCA	60.3	189	95	nodulin MtN3 family protein putative expressed
OsISM04677	LOC_Os01g36100	19977470	CTTCGGAAGAGTGGAGGATG	GCATAAGTCTTCCCCATTGC	59.7	706	58	hypothetical protein
OsISM04678	LOC_Os01g36140	19991344	GCTACTCACATCAGCTGCCA	GGGGGTCAGACTTCATCAAT	59.5	170	82	retrotransposon protein putative Ty3-gypsy subclass
OsISM04679	LOC_Os01g36150	19992705	CCAGGATTCAAACTTCGAGC	TTTCTCCATTTGAGCCATCC	59.9	141	68	retrotransposon protein putative Ty3-gypsy subclass
OsISM04680	LOC_Os01g36150	19992868	ACAAGGTAGAACTGGCTGCG	TTACCAACCAGGACGTCAAA	59.7	169	95	retrotransposon protein putative Ty3-gypsy subclass

ISM IDs	Rice gene locus IDs	Physical positions (bp) on chromosomes	Forward primers (5'-3')	Reverse primers (5'-3')	Melting tempearature (T˚C)	Expected product size (bp) in PCR	Expected product size (bp) in Q-PCR	Putative Functions
OsISM04681	LOC_Os01g36170	20000564	GGCTAGGATGGGTTGGAAAC	AGACGAAGCCAGTCATGTCC	60.5	1041	99	retrotransposon protein putative Ty3-gypsy subclass
OsISM04682	LOC_Os01g36170	20002299	CTCCGGCGTTGGTAGCTC	TGGTCGTCACCATCTGAAAA	61.0	329	72	retrotransposon protein putative Ty3-gypsy subclass
OsISM04683	LOC_Os01g36240	20061199	CTCCACTTCCACGACTGCTT	GATCGTCGCACTGCTGTCTA	60.3	211	67	peroxidase precursor putative expressed
OsISM04684	LOC_Os01g36240	20061509	GTCTCTTGCGCTGACATCCT	CTCCCCAGCGGCACTATC	61.2	1587	84	peroxidase precursor putative expressed
OsISM04685	LOC_Os01g36240	20063195	CCTCGACATCGTCGACCT	CGTTCCCCGTCTGGTTGTA	60.8	1562	99	peroxidase precursor putative expressed
OsISM04686	LOC_Os01g36270	20099748	CTGTCGAACTCCCTCCGTT	ATTAATCAGGGCCACCGTCT	60.5	248	65	hypothetical protein
OsISM04687	LOC_Os01g36270	20100099	ACCATCACCGAGCTCTCCTT	TTTGAAGGGTCATCTTCTCCA	60.4	1028	97	hypothetical protein
OsISM04688	LOC_Os01g36280	20109557	TCGAAGATCTTAAACTGTTGTGGA	GAATCGACGATGACCCATGT	60.5	239	87	transposon protein putative CACTA En/Spm sub-class
OsISM04689	LOC_Os01g36280	20109939	CAGCCAGCCTATAGAGAGCG	TGCTTCAGATCGTTACGTGC	60.1	209	76	transposon protein putative CACTA En/Spm sub-class
OsISM04690	LOC_Os01g36280	20111021	TCTCATGGGTAACGAGACGA	TCGTGCTCTTCATGTCTTGG	59.6	137	99	transposon protein putative CACTA En/Spm sub-class
OsISM04691	LOC_Os01g36280	20111611	CACGTAGCGATCACAAGGAA	GCTTGAGGGACGACTACGAC	59.9	1679	68	transposon protein putative CACTA En/Spm sub-class
OsISM04692	LOC_Os01g36280	20113254	TTGCAGAAAAGACATCGTCG	GTACTGGCTGCTTCCCTCTG	60.0	577	80	transposon protein putative CACTA En/Spm sub-class
OsISM04693	LOC_Os01g36280	20115158	CATGTGAGCTGCATATTCCC	AGTCCCTGAAATGTCCGTTG	59.5	174	81	transposon protein putative CACTA En/Spm sub-class
OsISM04694	LOC_Os01g36280	20115434	AGACACAAGCCATGCCATTA	CGTCAACTTTCGCCTTCTTC	59.6	356	98	transposon protein putative CACTA En/Spm sub-class
OsISM04695	LOC_Os01g36280	20116378	CCCTCGACGTCTCTCTTCTG	TCAGCTGGAAGACCTTGTCA	59.8	486	94	transposon protein putative CACTA En/Spm sub-class
OsISM04696	LOC_Os01g36280	20116844	TGACAAGGTCTTCCAGCTGAT	TTTTCTTTCCAAGTCCCACG	60.0	175	73	transposon protein putative CACTA En/Spm sub-class
OsISM04697	LOC_Os01g36280	20116999	CGTGGGACTTGGAAAGAAAA	TCGCATATGTAGTAGCCGCA	60.2	190	93	transposon protein putative CACTA En/Spm sub-class
OsISM04698	LOC_Os01g36294	20124909	AGGGCATCACCAGAGAACAC	CGGCCATGGCAAATACTAAG	60.3	182	94	cytochrome P450 putative expressed

ISM IDs	Rice gene locus IDs	Physical positions (bp) on chromosomes	Forward primers (5'-3')	Reverse primers (5'-3')	Melting tempearature (T˚C)	Expected product size (bp) in PCR	Expected product size (bp) in Q-PCR	Putative Functions
OsISM04699	LOC_Os01g36330	20145107	AGTGGCCTTTCTGGTAACGA	CATCAGAATGCCCTTCTTCC	59.7	142	99	hypothetical protein
OsISM04700	LOC_Os01g36330	20145280	GGCTGGGGTGCTAGATTGTA	AAACGATGGGAGTCGTGTTC	60.0	262	95	hypothetical protein
OsISM04701	LOC_Os01g36350	20152921	TGCTGACCAACAAGAAGGTG	TCCCATACCCTTGGTGGATA	59.9	1031	99	cytochrome P450 putative expressed
OsISM04702	LOC_Os01g36350	20154305	AGGCATCACCAGAGATCACC	CATGGCGAATACTAGCACCA	59.9	183	89	cytochrome P450 putative expressed
OsISM04703	LOC_Os01g36360	20159909	AACCCTGGGAGACTTACCGT	GGCAGTCATACCACTGACACA	59.7	1641	96	retrotransposon protein putative Ty3-gypsy subclass
OsISM04704	LOC_Os01g36360	20161466	TGGTTGCTTGCTAGTTTTCG	CGCGTGAACCATTCCTTATC	59.8	244	96	retrotransposon protein putative Ty3-gypsy subclass
OsISM04705	LOC_Os01g36370	20168491	AACCCTGGGAGACTTACCGT	CCCAGAAATTCTCGAACCAG	59.8	955	88	transposon protein putative unclassified
OsISM04706	LOC_Os01g36370	20169435	GAATTTCTGGGCGTGACAAG	TTTTTCCCCAATAGTGCAGG	60.3	545	60	transposon protein putative unclassified
OsISM04707	LOC_Os01g36370	20169960	CCTGCACTATTGGGGAAAAA	AGCTGGATCGATTTGTGTCG	60.6	790	76	transposon protein putative unclassified
OsISM04708	LOC_Os01g36370	20170836	CCGAGAAGGCTAATCCCTGT	AATGGTTTTGGTCTCACATCC	59.7	364	79	transposon protein putative unclassified
OsISM04709	LOC_Os01g36370	20171394	TCTCCTTGTCCCTTCCATTG	CTCCTGGTCTCGGGATGAT	60.0	209	75	transposon protein putative unclassified
OsISM04710	LOC_Os01g36370	20171869	GCCATCCTAGCTTGCCAAC	AGCTGAAGCTGAACTTGATCC	59.7	1170	82	transposon protein putative unclassified
OsISM04711	LOC_Os01g36370	20173488	AGAGAAATCTGACCCGGAGA G	ATGCCTGCGATGGTGTAGTT	60.2	255	99	transposon protein putative unclassified
OsISM04712	LOC_Os01g36370	20173780	TCCTAACATGGGTCCCAAAG	CCTCCCATCAACTTCCTTGA	59.9	218	95	transposon protein putative unclassified
OsISM04713	LOC_Os01g36370	20174032	CTTGCATTGAGGGGTTTGTT	GGATACATCCAATTGTGCCC	60.0	127	62	transposon protein putative unclassified
OsISM04714	LOC_Os01g36370	20174660	AAGGAGGATTGGAGAAAGGC	GCAGCGCTGGTATCTCTCTC	60.0	744	96	transposon protein putative unclassified
OsISM04715	LOC_Os01g36370	20175513	GACCGGTGGCAAGAGTAGTG	ACCTTCTTGTCACCATTCCG	60.3	330	91	transposon protein putative unclassified
OsISM04716	LOC_Os01g36370	20175804	CACTAATGCAAGAAAGCCCC	CAAATTGCCTCCTGCTTCTC	59.8	178	76	transposon protein putative unclassified

ISM IDs	Rice gene locus IDs	Physical positions (bp) on chromosomes	Forward primers (5'-3')	Reverse primers (5'-3')	Melting tempearature (T°C)	Expected product size (bp) in PCR	Expected product size (bp) in Q-PCR	Putative Functions
OsISM04718	LOC_Os01g36390	20191834	TCGGTTTGCTTGAGGAATCT	CCTTGAGCTCTGGAATCACC	59.8	183	98	MCM4 - Putative minichromosome maintenance MCM complex subunit 4 expressed
OsISM04719	LOC_Os01g36390	20192042	CGGGTTCTACTCTGAGCCTG	TCTTTCTGACAGATGTGCGG	60.0	694	67	MCM4 - Putative minichromosome maintenance MCM complex subunit 4 expressed
OsISM04720	LOC_Os01g36390	20192759	TGACTCTTGTGCATAACCGA	TCATCTGGTGTTTCCTGCAA	59.0	165	68	MCM4 - Putative minichromosome maintenance MCM complex subunit 4 expressed
OsISM04721	LOC_Os01g36390	20192979	GTAGATGCTGGAAAGCCTGG	CACTGTCCTCTGAGTTGGGC	60.3	192	88	MCM4 - Putative minichromosome maintenance MCM complex subunit 4 expressed
OsISM04722	LOC_Os01g36390	20193152	CCCAACTCAGAGGACAGTGA	CCATGGAGTCCTCAACATGA	59.1	211	99	MCM4 - Putative minichromosome maintenance MCM complex subunit 4 expressed
OsISM04723	LOC_Os01g36390	20193357	CCATGGAAACTGATAACCCC	TCTGGCAACTTTGACAACTCTT	59.1	174	95	MCM4 - Putative minichromosome maintenance MCM complex subunit 4 expressed
OsISM04724	LOC_Os01g36390	20193566	ACATATGGGAGCTGGACGAC	GAAGCCTCAAAGCATTTCCA	60.1	145	70	MCM4 - Putative minichromosome maintenance MCM complex subunit 4 expressed
OsISM04725	LOC_Os01g36390	20193834	GAGGAAGTTCAGCTGTTGGC	AAAACAAGCGCTCCACTCTC	59.8	181	86	MCM4 - Putative minichromosome maintenance MCM complex subunit 4 expressed
OsISM04726	LOC_Os01g36390	20194013	TTGAGTGACAAAGGTGTTTGTTG	TGGAGACAGTCTGTTGTTCCAT	59.9	185	98	MCM4 - Putative minichromosome maintenance MCM complex subunit 4 expressed
OsISM04727	LOC_Os01g36390	20194307	CCATCTTCCTCCAACACTGC	GCTCATCTGCCTTGTCCAAT	60.4	168	66	MCM4 - Putative minichromosome maintenance MCM complex subunit 4 expressed
OsISM04728	LOC_Os01g36390	20194486	CGCCTGGCTAAGCATATTGT	GTTGGCAAATCCAAGACCTC	59.9	219	75	MCM4 - Putative minichromosome maintenance MCM complex subunit 4 expressed
OsISM04729	LOC_Os01g36390	20194811	AACAGCCCTGGTAGCAGAAA	AGTGCTTCACTGAGCCGAAT	59.9	158	78	MCM4 - Putative minichromosome maintenance MCM complex subunit 4 expressed
OsISM04730	LOC_Os01g36390	20194940	GAGAGCTTGATTCGGCTCAG	GCCTGAAGGCCTCTACAACA	60.3	181	86	MCM4 - Putative minichromosome maintenance MCM complex subunit 4 expressed
OsISM04731	LOC_Os01g36390	20195114	TTCAGGCTTCTCGAAGTTGC	CTTTCGCTCGCAGATATTCC	60.3	194	99	MCM4 - Putative minichromosome maintenance MCM complex subunit 4 expressed
OsISM04732	LOC_Os01g36390	20195353	GGAGAAAATGCAGCTTGGAG	CATCATGCAGATGAACTTCCA	59.8	182	93	MCM4 - Putative minichromosome maintenance MCM complex subunit 4 expressed
OsISM04733	LOC_Os01g36390	20195469	GATTGATTTCGCAGTTGCTG	ATCAGAGTGCCAAGAGCACC	59.9	178	89	MCM4 - Putative minichromosome maintenance MCM complex subunit 4 expressed
OsISM04734	LOC_Os01g36410	20211954	TTACCCCGTGCTCTATTTGG	GAAGCGACAAGGCGAGAC	59.8	557	87	conserved hypothetical protein
OsISM04735	LOC_Os01g36430	20220356	ATCCCGTTCTGAACAACGAC	GGGGTAGCTTGTTGAGGTGA	60.0	175	56	retrotransposon protein putative unclassified

ISM IDs	Rice gene locus IDs	Physical positions (bp) on chromosomes	Forward primers (5'-3')	Reverse primers (5'-3')	Melting tempearature (T˚C)	Expected product size (bp) in PCR	Expected product size (bp) in Q-PCR	Putative Functions
OsISM04736	LOC_Os01g36430	20221057	ACAGTGCTTTATGCCGCTG	GTGGCGCTTGTATGTTCCTT	60.1	172	79	retrotransposon protein putative unclassified
OsISM04737	LOC_Os01g36430	20222089	TACCAGCACTCTGGATGCAA	TGGGATGTGGCCATAAACTT	60.3	243	88	retrotransposon protein putative unclassified
OsISM04738	LOC_Os01g36430	20222427	TCGACATGGTCGAGCAAAC	ATTATCGCGGAGGAAGGTG	60.4	216	87	retrotransposon protein putative unclassified
OsISM04739	LOC_Os01g36430	20223934	CGCGAACAGCCATTAAATCT	CCTTCGTATTCTGCCGTGTT	60.2	235	95	retrotransposon protein putative unclassified
OsISM04740	LOC_Os01g36450	20233748	CCAGACCAGGACGGAGATT	TACTTCGTAGGCCTGCTGCT	60.1	217	65	retrotransposon protein putative unclassified
OsISM04741	LOC_Os01g36450	20234073	ATGCAAGCGCCAGTGTACTT	CGAAGGAAGGCTTGAAGCTG	61.7	182	96	retrotransposon protein putative unclassified
OsISM04742	LOC_Os01g36510	20261344	CAAAGCTGATGAAGATGCCA	CACCCATTTCCCTTTCTTCA	59.9	103	68	hypothetical protein
OsISM04743	LOC_Os01g36510	20261522	GGGAAGGAAGAGGAGTTTGC	TCCTCTTTGTTCAGATCAGGG	59.7	147	96	hypothetical protein
OsISM04744	LOC_Os01g36510	20261685	GGAGATGGGATGCTAGGACA	GGCAATTGATGGAACACCTT	59.9	375	97	hypothetical protein
OsISM04745	LOC_Os01g36510	20262101	AGACCCTGTGGAAGTGGATG	CTGGTGGGAAGGACTGCTTA	60.1	167	96	hypothetical protein
OsISM04746	LOC_Os01g36510	20262254	GTCCTTCCCACCAGAAGACC	AAACTCTTCCTCCTCTTGGTTG	59.9	189	95	hypothetical protein
OsISM04747	LOC_Os01g36510	20262739	TCCAGGGGGAGATAAAGGTC	CGCAATCACTTCCAGTTGTC	59.8	142	92	hypothetical protein
OsISM04748	LOC_Os01g36520	20264829	GGGACCCTAGAGCTATGGAGA	TGGCTAGTTCTGAGGCCAAT	59.8	852	74	hypothetical protein
OsISM04749	LOC_Os01g36520	20265663	TGGCCTCAGAACTAGCCACT	GCATGGATCATTAGCTCCAGA	60.1	370	69	hypothetical protein
OsISM04750	LOC_Os01g36550	20284040	GGGAGTTGCTGGTCCTTCTA	ACAGGCCTCTCTGTTTGGAA	59.6	635	55	protein kinase family protein putative
OsISM04751	LOC_Os01g36550	20284734	TTGGTGAGGGAGGATATGGA	CCTTTGATTTGCTTGCTTTCA	60.3	186	88	protein kinase family protein putative
OsISM04752	LOC_Os01g36550	20284899	TGAAAGCAAGCAAATCAAAG G	CCAGCAGCATAACCAATCAA	60.0	221	95	protein kinase family protein putative
OsISM04753	LOC_Os01g36550	20285175	CATCTCCATGATCCTTTGCTG	ACGTGCAGAGTCCATTGCTA	60.0	270	79	protein kinase family protein putative

ISM IDs	Rice gene locus IDs	Physical positions (bp) on chromosomes	Forward primers (5'-3')	Reverse primers (5'-3')	Melting tempearature (T˚C)	Expected product size (bp) in PCR	Expected product size (bp) in Q-PCR	Putative Functions
OsISM04754	LOC_Os01g36550	20285487	CACCGTGACATCAAAACCAG	CATCACTACGCTGAACGAGC	59.8	177	95	protein kinase family protein putative
OsISM04755	LOC_Os01g36550	20285673	GTCTGGCAACTCGTTTGGTT	CGAATGCATACACATCGGATT	60.4	170	94	protein kinase family protein putative
OsISM04756	LOC_Os01g36550	20285887	CGGGACAACAAGGAAGCTAA	TTGTCTCCAGGGAACTCTCC	59.7	230	92	protein kinase family protein putative
OsISM04757	LOC_Os01g36550	20286130	CCTGAAGGACAATTACCCCA	CACCACATCGAAATGTTTGC	59.9	149	58	protein kinase family protein putative
OsISM04758	LOC_Os01g36630	20321736	ATGGTTCCCTGCTGTACCTG	TGTTGGTGTGGTGTATGCAA	59.7	594	75	jmjC domain-containing protein 4 putative expressed
OsISM04759	LOC_Os01g36630	20322432	CGGTTTGTTTACATGGGACC	TTTTCTGCCGCATACATTTG	59.9	254	94	jmjC domain-containing protein 4 putative expressed
OsISM04760	LOC_Os01g36630	20322689	TGGCTCTTTCTACCACCATCA	CTGCTTTTCAGAAACATCATCA	59.3	815	94	jmjC domain-containing protein 4 putative expressed
OsISM04761	LOC_Os01g36630	20323491	TTCTGAAAAGCAGTTTCCTGAAT	ACCACCCACTAGGCACAAAA	59.9	189	90	jmjC domain-containing protein 4 putative expressed
OsISM04762	LOC_Os01g36630	20323673	GGGTGGTATCACCAAGTCCA	CTCACCACCCAATGAAGGTT	60.2	820	87	jmjC domain-containing protein 4 putative expressed
OsISM04763	LOC_Os01g36630	20324457	TTGGTTTAATGGCTACAACCTTC	CGTCACATATGTCCCGAATG	59.6	174	99	jmjC domain-containing protein 4 putative expressed
OsISM04764	LOC_Os01g36630	20324646	GTCAACGCAACTTAGCAGCA	ATTGGCTAAAGCAAAACGTG	59.1	157	78	jmjC domain-containing protein 4 putative expressed
OsISM04765	LOC_Os01g36680	20368768	TGCCGGCTATCCTTATGAAC	CCGGAGTTTCTTAATTTCCTGA	59.8	130	81	retrotransposon protein putative Ty1-copia subclass
OsISM04766	LOC_Os01g36680	20368987	CGAAGGAGATGGGTTTGAGA	AATCTTCATGGACTGCGACC	60.1	298	83	retrotransposon protein putative Ty1-copia subclass
OsISM04767	LOC_Os01g36690	20372711	GATCTCGGCTCGTTGATGAT	CCATCCAGTTGCTACCATCC	60.3	603	98	hypothetical protein
OsISM04768	LOC_Os01g36700	20376898	ATACTTGTGTCGGCGTGGTT	ACAGTGCAGGAATTGGAAGG	60.3	401	85	hypothetical protein
OsISM04769	LOC_Os01g36750	20410975	AAACAAGGTGATAGAACTTTTAGGGA	ACCAAGCATGAATAGGTGCC	59.9	183	97	retrotransposon protein putative unclassified
OsISM04770	LOC_Os01g36800	20472068	TTCGGGAAAAATTAGAACATCTG	CTCCTTTTCTTGGATGTGCTG	59.7	386	93	retrotransposon protein putative unclassified
OsISM04771	LOC_Os01g36800	20473434	GGCTTGTAACTTCGTCTGGG	CTCCGTGGGTTGTTGATCTT	59.9	369	89	retrotransposon protein putative unclassified

ISM IDs	Rice gene locus IDs	Physical positions (bp) on chromosomes	Forward primers (5'-3')	Reverse primers (5'-3')	Melting tempearature (T˚C)	Expected product size (bp) in PCR	Expected product size (bp) in Q-PCR	Putative Functions
OsISM04772	LOC_Os01g36830	20485394	GTTCAGCGTGTCGTCGTTC	AGGAGCCAACACATCACCAT	60.4	650	77	polygalacturonase putative
OsISM04773	LOC_Os01g36830	20486127	CACTGGCACTGTTGATGGAC	ACCGTAGGCTTCACTCCTGA	60.0	251	94	polygalacturonase putative
OsISM04774	LOC_Os01g36830	20486349	TGGTATGTATCAGGAGTGAAGCC	GCACGCTGACATTGAAGCTA	60.3	173	62	polygalacturonase putative
OsISM04775	LOC_Os01g36830	20486647	CAGAACACCAGGGATGTTGA	ATTGGAGCATCCCGTCTGTA	60.0	188	82	polygalacturonase putative
OsISM04776	LOC_Os01g36830	20486815	TACAGACGGGATGCTCCAAT	GCAAGCCAAGCTGTTATCCT	60.0	195	99	polygalacturonase putative
OsISM04777	LOC_Os01g36830	20487028	GAGCACATCAACGTGGAGAA	ACCCTCACGTTGGAGAAGG	60.0	193	99	polygalacturonase putative
OsISM04778	LOC_Os01g36860	20521885	GATGATCCCTCCACCGAG	GGGAATCCACCAGTCTCAAA	59.4	223	99	DEAD-box ATP-dependent RNA helicase 40 putative expressed
OsISM04779	LOC_Os01g36860	20522088	TTTGAGACTGGTGGATTCCC	ATGACTGAGCTTGGATTGGC	60.1	1010	86	DEAD-box ATP-dependent RNA helicase 40 putative expressed
OsISM04780	LOC_Os01g36860	20523249	AAGGGAGCTTGCAACTCAGA	CCTTTTGGAGCACCACCATA	60.5	356	94	DEAD-box ATP-dependent RNA helicase 40 putative expressed
OsISM04781	LOC_Os01g36860	20523874	TCCAGGTGACCATTGGAAGT	GAAGCTTTTCGGACGGTGT	60.3	256	88	DEAD-box ATP-dependent RNA helicase 40 putative expressed
OsISM04782	LOC_Os01g36860	20524332	AACAGATGTTGCTGCACGAG	ATGCCAGTGGGGAAATCATA	60.1	1301	73	DEAD-box ATP-dependent RNA helicase 40 putative expressed
OsISM04783	LOC_Os01g36860	20525896	GTCTGTCAGGGTCTTCTGGC	AGACCGTCCATCATCACCAT	60.0	323	75	DEAD-box ATP-dependent RNA helicase 40 putative expressed
OsISM04784	LOC_Os01g36890	20540046	TACGAGGAGGAGGTGGAGAA	GCTGTGGATCCCCACGTA	60.1	201	94	DEAD-box ATP-dependent RNA helicase putative expressed
OsISM04785	LOC_Os01g36890	20540295	CAGGACTGCGGGTTTGAG	ATTCCAAGAATGGCTTGTGG	60.2	1698	66	DEAD-box ATP-dependent RNA helicase putative expressed
OsISM04786	LOC_Os01g36890	20542095	TGTGCTATGTCACACAAGGGA	CCGCAACTTTTACCTCAGGA	60.2	170	87	DEAD-box ATP-dependent RNA helicase putative expressed
OsISM04787	LOC_Os01g36890	20542381	TCCTTGAAGAATGTGAGGCAT	CTCCTGCACATCTCTACGCA	59.9	230	82	DEAD-box ATP-dependent RNA helicase putative expressed
OsISM04788	LOC_Os01g36890	20542659	ACCCTTAGCAAGGAGATCCG	GCCCATGAAGGGTCAGTTTA	60.3	715	95	DEAD-box ATP-dependent RNA helicase putative expressed
OsISM04789	LOC_Os01g36890	20543354	TAAACTGACCCTTCATGGGC	GGTTCTTCTCCGCCTCACTT	60.3	140	60	DEAD-box ATP-dependent RNA helicase putative expressed

ISM IDs	Rice gene locus IDs	Physical positions (bp) on chromosomes	Forward primers (5'-3')	Reverse primers (5'-3')	Melting tempearature (T˚C)	Expected product size (bp) in PCR	Expected product size (bp) in Q-PCR	Putative Functions
OsISM04790	LOC_Os01g36890	20543589	TGTGAATGCAACTTTCCTGC	TCCTCTTGTGTCCTTCCTTGA	59.8	1549	95	DEAD-box ATP-dependent RNA helicase putative expressed
OsISM04791	LOC_Os01g36890	20545202	CTATGACATGCCCGATTCTG	ATTGCAAGTCCTTTGGTTCC	59.1	147	79	DEAD-box ATP-dependent RNA helicase putative expressed
OsISM04792	LOC_Os01g36890	20545351	CATTTGTTTCCTCTGCCTCTG	TCTGCTCAGGAAGCTCCTTT	59.6	174	85	DEAD-box ATP-dependent RNA helicase putative expressed
OsISM04793	LOC_Os01g36890	20545508	GGAGCTTCCTGAGCAGATTG	CCACCCGGATCTTTAAGAAG	59.4	159	56	DEAD-box ATP-dependent RNA helicase putative expressed
OsISM04794	LOC_Os01g36920	20570158	TACGAGGAGGAGGTCGAGAA	GCTGTGGATCCCCACGTA	60.2	199	94	DEAD-box ATP-dependent RNA helicase putative expressed
OsISM04795	LOC_Os01g36920	20570405	CAGGACTGCGGGTTTGAG	ATTCCAAGAATGGCTTGTGG	60.2	1428	66	DEAD-box ATP-dependent RNA helicase putative expressed
OsISM04796	LOC_Os01g36920	20571905	TGATCCTGTTGCTGGTCAAG	AAACCTCTCAAATTCATGGCA	59.7	169	86	DEAD-box ATP-dependent RNA helicase putative expressed
OsISM04797	LOC_Os01g36920	20572221	TCCTTGAAGAATGTGAGGCAT	CTCCTGCACATCTCTACGCA	59.9	230	82	DEAD-box ATP-dependent RNA helicase putative expressed
OsISM04798	LOC_Os01g36920	20572499	ACCCTTAGCAAGGAGATCCG	CGTGAAGGGTCAGTTTAGCC	60.2	713	92	DEAD-box ATP-dependent RNA helicase putative expressed
OsISM04799	LOC_Os01g36920	20573193	GCTAAACTGACCCTTCACGG	GGTTCTTCTCCGCCTCACTT	60.3	141	62	DEAD-box ATP-dependent RNA helicase putative expressed
OsISM04800	LOC_Os01g36920	20573429	TGTGAATGCAACTTTCCTGC	TCCTCTTGTGTCCTTCCTTGA	59.8	873	95	DEAD-box ATP-dependent RNA helicase putative expressed
OsISM04801	LOC_Os01g36920	20574366	CTATGACATGCCCGATTCTG	TCCTTTGGTTCCAAAACGTC	59.5	142	71	DEAD-box ATP-dependent RNA helicase putative expressed
OsISM04802	LOC_Os01g36920	20574518	CATTTGTTTCCTCTGCCTCAG	TCTGCTCAGGAAGCTCCTTT	59.6	171	85	DEAD-box ATP-dependent RNA helicase putative expressed
OsISM04803	LOC_Os01g36920	20574672	GGAGCTTCCTGAGCAGATTG	TTTCCGGTTCGAATGTAAGC	60.1	189	87	DEAD-box ATP-dependent RNA helicase putative expressed
OsISM04804	LOC_Os01g36940	20594666	CTCTCCATGACCAACTCCG	ATGTTGTGCTGCTCCACTGT	59.3	901	96	OsFBX13 - F-box domain containing protein expressed
OsISM04805	LOC_Os01g36940	20595702	CAAATGCATTCATGCTACCG	CAGTTGAATAACAGGCCCCA	60.5	1631	76	OsFBX13 - F-box domain containing protein expressed
OsISM04806	LOC_Os01g36970	20628700	ACGAAGGATCCCACCACC	AACCACTCCTGCAAATCCTG	60.4	279	96	expressed protein
OsISM04808	LOC_Os01g37000	20640106	CCTCAACAAGCCTCCCCT	AAGAGGATGGTCGAATGCAG	60.2	195	95	carboxyl-terminal peptidase putative expressed

ISM IDs	Rice gene locus IDs	Physical positions (bp) on chromosomes	Forward primers (5'-3')	Reverse primers (5'-3')	Melting tempearature (T°C)	Expected product size (bp) in PCR	Expected product size (bp) in Q-PCR	Putative Functions
OsISM04809	LOC_Os01g37000	20640288	CGACCATCCTCTTCTCAAGG	AATGGACGTGTTGCTTCTCC	60.0	219	85	carboxyl-terminal peptidase putative expressed
OsISM04810	LOC_Os01g37000	20640645	TCACTCTCACTTGGGTGGTG	GATGGTCACTTTGGTTCCGT	59.8	271	92	carboxyl-terminal peptidase putative expressed
OsISM04811	LOC_Os01g37000	20640980	CGGGCTCCTATGAAAACAAG	TCATCCCCGTACATAGCTGG	60.3	734	74	carboxyl-terminal peptidase putative expressed
OsISM04812	LOC_Os01g37000	20641691	TACCCAGCTATGTACGGGGA	CCCTGAGCACGCTAGATTGT	60.4	230	97	carboxyl-terminal peptidase putative expressed
OsISM04813	LOC_Os01g37000	20641956	CAGCTTATCCCCTGTCTCCA	GCAGTTGCAACCACCAGTT	60.0	249	96	carboxyl-terminal peptidase putative expressed
OsISM04814	LOC_Os01g37020	20650644	GCCTCAACAAGCCTCCTCTC	AATGCAGGCTGTCTGGAGAT	60.6	235	83	carboxyl-terminal peptidase putative
OsISM04815	LOC_Os01g37020	20650871	CCTGCATTCGATCATCCTCT	ACATCCTCCTCCTTGGTCCT	60.1	280	99	carboxyl-terminal peptidase putative
OsISM04816	LOC_Os01g37020	20651199	GAGCCCTCACTCTCACTTGG	GATGGTCACTTTGGTTCCGT	59.9	252	98	carboxyl-terminal peptidase putative
OsISM04817	LOC_Os01g37020	20651541	AATACCATTGAAGCGGGATG	TCATCCCCATACATAGCTGG	59.1	707	48	carboxyl-terminal peptidase putative
OsISM04818	LOC_Os01g37020	20652227	CCCAGCTATGTATGGGGATG	CCCTGAGCACGCTAGATTGT	60.3	227	95	carboxyl-terminal peptidase putative
OsISM04819	LOC_Os01g37020	20652503	TCTCCATCTATGGTGGCACA	CAACCACCAATTACCCCTTG	60.1	983	66	carboxyl-terminal peptidase putative
OsISM04820	LOC_Os01g37040	20662827	AAGAGGAACAAGAGCCTCCC	TTGCAGGATTTCTAGCTCCA	59.2	807	99	retrotransposon protein putative Ty1-copia subclass expressed
OsISM04821	LOC_Os01g37070	20686600	GATAGTCACGTGCGATGAGG	CTCCATGCGAAGACGTCAG	59.9	150	76	retrotransposon protein putative unclassified
OsISM04823	LOC_Os01g37090	20699941	CGCTGACTCCTCCTGCTG	GGTGCAGCTCTCTGTCACAA	60.5	1912	67	expressed protein
OsISM04824	LOC_Os01g37110	20713380	ATGCAGGAGACGCTCATCTT	TGCGACTTCAGCATCTTGAG	60.1	405	57	ABIL2 putative expressed
OsISM04825	LOC_Os01g37110	20713815	AGCCTACACGCAGGAAGATG	GATCCCAGGTGATCGACAGT	60.2	210	94	ABIL2 putative expressed
OsISM04826	LOC_Os01g37110	20714058	AAGGTTCGATGAGGTTGACG	GAAAGGCCTTCCTTGTCCAT	60.3	200	94	ABIL2 putative expressed
OsISM04827	LOC_Os01g37110	20714270	GGTGATCACAGCTCCCAAGT	AAGTTGGGCACAGCATTAGG	60.1	204	79	ABIL2 putative expressed

ISM IDs	Rice gene locus IDs	Physical positions (bp) on chromosomes	Forward primers (5'-3')	Reverse primers (5'-3')	Melting tempearature (T˚C)	Expected product size (bp) in PCR	Expected product size (bp) in Q-PCR	Putative Functions
OsISM04828	LOC_Os01g37110	20714471	CTTCAGCGAGATGAGGAAGG	TCTCTTTGGCTTTTGCTTGG	60.3	298	90	ABIL2 putative expressed
OsISM04829	LOC_Os01g37110	20714749	CCAAGCAAAAGCCAAAGAGA	CTGTGCTGGTGACGACGA	60.6	181	95	ABIL2 putative expressed
OsISM04830	LOC_Os01g37110	20714930	CGCCCACCTTCTGAGAATAC	ATCCAGACCTTGTGAGTGGC	59.9	272	92	ABIL2 putative expressed
OsISM04831	LOC_Os01g37110	20715207	CCACAAAAGCCATCCTTGTT	TGCCAATTTCTTGTGCTCTG	60.0	166	73	ABIL2 putative expressed
OsISM04832	LOC_Os01g37130	20737854	CCTCGACGGCTTCTCCTT	GCGCATTCCTTGTACAGCTC	60.7	175	91	protein phosphatase 2C putative expressed
OsISM04833	LOC_Os01g37130	20738076	CCATCACCGACTCGATCC	CGGACTCATCTTCCTTGTCC	59.8	549	88	protein phosphatase 2C putative expressed
OsISM04834	LOC_Os01g37130	20738657	GATGTCCTTGTTGTCTCGCA	AGTTGGTCACAGCTTGAGGC	60.1	245	80	protein phosphatase 2C putative expressed
OsISM04835	LOC_Os01g37130	20738938	AAGAGGTGAAGCGGATCAGA	CTCCAAAAGCACGAGACACA	60.0	576	85	protein phosphatase 2C putative expressed
OsISM04836	LOC_Os01g37130	20739492	TCTGTGTCTCGTGCTTTTGG	TTTCAGTCCATCTCCCTTCTTT	59.6	200	89	protein phosphatase 2C putative expressed
OsISM04837	LOC_Os01g37130	20739671	AAGAAGGGAGATGGACTGAAAA	GACCAGGGATACGTCAGGTG	59.8	606	84	protein phosphatase 2C putative expressed
OsISM04838	LOC_Os01g37130	20740309	AGCAACAGATGGTCTTTGGG	TCATACCTGGACATCCCCAT	60.1	217	92	protein phosphatase 2C putative expressed
OsISM04839	LOC_Os01g37130	20740488	GGGATCAACTACGGCAACAT	TCTGACCAAGTGCCTCACAG	59.9	150	55	protein phosphatase 2C putative expressed
OsISM04840	LOC_Os01g37130	20740618	CTGTGAGGCACTTGGTCAGA	TAATGTTGTCTTGCGACCGA	60.1	188	51	protein phosphatase 2C putative expressed
OsISM04841	LOC_Os01g37130	20740786	TCGGTCGCAAGACAACATTA	GCAGCTCCTTCCAGTTTGTC	60.1	303	63	protein phosphatase 2C putative expressed
OsISM04842	LOC_Os01g37140	20743755	CAAATTGAGAGTGCAAGAAGAAGA	GTGTTTTTCCACCAAATGGG	60.1	500	89	retrotransposon protein putative unclassified
OsISM04843	LOC_Os01g37160	20754315	GCGGTACAGATGAGAAAGGG	TGTTGTCCACCAAAACCTTG	59.6	602	92	retrotransposon protein putative unclassified
OsISM04844	LOC_Os01g37170	20759605	ATCCATCCTGAATGGCCTG	TTGTCTGGCCAAAACTTTCC	60.5	340	91	retrotransposon protein putative unclassified
OsISM04845	LOC_Os01g37230	20794066	GCTATTCACACTTGCTGCCA	GGATCCGACTTCATCAAAGC	59.8	168	80	retrotransposon protein putative unclassified

ISM IDs	Rice gene locus IDs	Physical positions (bp) on chromosomes	Forward primers (5'-3')	Reverse primers (5'-3')	Melting tempearature (T˚C)	Expected product size (bp) in PCR	Expected product size (bp) in Q-PCR	Putative Functions
OsISM04846	LOC_Os01g37230	20794460	TCCAGTCGTCCTGTACCAAA	TCTGCTTCCATATTAGTCGGG	59.2	174	96	retrotransposon protein putative unclassified
OsISM04847	LOC_Os01g37230	20794894	CACAAGAGTCCACTCGTCCC	TCCGGAGGATCTCTTCTTCA	60.3	152	73	retrotransposon protein putative unclassified
OsISM04848	LOC_Os01g37230	20795157	TGCTGAAAAAGGCGATCAG	TTGCCGTTTTAGCTTCGTTC	60.2	149	73	retrotransposon protein putative unclassified
OsISM04849	LOC_Os01g37230	20795286	GAACGAAGCTAAAACGGCAA	TTGGCAGCTTGCACTACATT	59.9	260	52	retrotransposon protein putative unclassified
OsISM04850	LOC_Os01g37230	20795600	GGCAAGACAGAGGGACTCAG	CTGTGCCTGGGACTTGATCT	60.1	137	62	retrotransposon protein putative unclassified
OsISM04851	LOC_Os01g37260	20805957	GCGGATAAGATCACAATGGG	TGCTCATCTGAACAGCTTGC	60.3	181	99	OsFBX14 - F-box domain containing protein expressed
OsISM04852	LOC_Os01g37260	20806863	GTTTTCACTGGGCTTCCAAA	CCAGGTATCTGAAAGACGCC	59.9	182	78	OsFBX14 - F-box domain containing protein expressed
OsISM04853	LOC_Os01g37260	20807157	TTGGAAGCTGCTCCTTTGTT	CACACGTCATCTGCACACAA	60.2	1593	47	OsFBX14 - F-box domain containing protein expressed
OsISM04854	LOC_Os01g37300	20822468	CTGCATACGACCTCCGTTTT	CTCCGTGGGTTGTTGATCTT	60.0	506	67	retrotransposon protein putative unclassified
OsISM04855	LOC_Os01g37310	20825790	GTGATGTCGAGCAGCAGAAG	CCAACTTTGCAAGGTGTCAT	59.2	1127	84	SWIM zinc finger family protein putative
OsISM04856	LOC_Os01g37310	20826968	TGTGGATGCAAGCTTTTTGA	CGGTATTTCCGTGAAACCAT	60.0	159	85	SWIM zinc finger family protein putative
OsISM04857	LOC_Os01g37340	20845977	CAAGGACCACAAGGAGGAAA	CTTCAGCATCATTGGCTACG	59.8	889	98	retrotransposon protein putative unclassified
OsISM04859	LOC_Os01g37380	20868968	GACATTTCACAGTGGGGGAT	CTCACATGCTCATTGGATGG	59.9	174	97	transposon protein putative unclassified
OsISM04860	LOC_Os01g37380	20869461	AATGTGGGCTGTAGGTGGAG	CATGATCGTATGGGTTCCTTG	60.1	163	83	transposon protein putative unclassified
OsISM04861	LOC_Os01g37380	20869913	TCACCATACCATCAACAACAAAA	TGTGACCATCAACTGCACAA	59.9	170	63	transposon protein putative unclassified
OsISM04862	LOC_Os01g37380	20870174	CAACCCAGTTGGGCTTACAT	TGGTCCACAATTTCCTGTGA	59.9	275	78	transposon protein putative unclassified
OsISM04863	LOC_Os01g37380	20870492	TAACTTCGCTGAGGCATTCA	GCTAGCTCCTCTCAACTTCTGC	59.8	253	77	transposon protein putative unclassified
OsISM04864	LOC_Os01g37380	20871112	AATCAGCTTTGGAGGGAGGT	TGGCTTCTGTGTAGCACTGG	60.1	373	99	transposon protein putative unclassified

ISM IDs	Rice gene locus IDs	Physical positions (bp) on chromosomes	Forward primers (5'-3')	Reverse primers (5'-3')	Melting tempearature (T˚C)	Expected product size (bp) in PCR	Expected product size (bp) in Q-PCR	Putative Functions
OsISM04865	LOC_Os01g37380	20871517	GCCACCAACATCAGCAAGTA	ATTTGGAGAGCAAAGGGGTT	59.8	153	79	transposon protein putative unclassified
OsISM04866	LOC_Os01g37430	20899626	CCAGCCACAGAGGCAGTT	TCGACGTATGTTGTTCCTGC	59.9	182	92	transposon protein putative CACTA En/Spm sub-class
OsISM04867	LOC_Os01g37430	20899960	ATAGCGGCACGTGAGAACTT	TCCGTTTCCTGTATTGGGAG	59.9	192	71	transposon protein putative CACTA En/Spm sub-class
OsISM04868	LOC_Os01g37430	20900391	CTCCCGATCGCACCACTA	AGTTGGGTGGACTCTCGCTA	60.3	162	79	transposon protein putative CACTA En/Spm sub-class
OsISM04869	LOC_Os01g37430	20901306	CGGAAGTTGGACACTGGTCT	GATCAAGGAACCCAACCTGA	60.0	173	89	transposon protein putative CACTA En/Spm sub-class
OsISM04870	LOC_Os01g37430	20901552	GGCCCATCAAGACAAGGAGT	CGATGACTAGGAGAATCCAATG	60.1	150	69	transposon protein putative CACTA En/Spm sub-class
OsISM04872	LOC_Os01g37440	20919217	CGTCGAATTAGGGACAAAGA	CGTGACAAAGCAATCATCATC	58.5	377	99	retrotransposon protein putative unclassified
OsISM04873	LOC_Os01g37440	20920327	CCACATCCACATCCGTATCA	TGGCCATCCTTGGAGGTAT	60.2	592	98	retrotransposon protein putative unclassified
OsISM04874	LOC_Os01g37460	20935290	AAGAACGGCGACTGGATCT	GCACTCGGTTCTGTTAGCGT	60.1	674	67	zinc finger family protein putative expressed
OsISM04875	LOC_Os01g37460	20935944	ACGCTAACAGAACCGAGTGC	TTTCTTCTCAGTGCAAGGCA	60.1	206	92	zinc finger family protein putative expressed
OsISM04876	LOC_Os01g37500	20951166	TTCCATGACAAGCTCCTTCA	CACTTGTTTCAGAGGCACCA	59.6	728	56	hypothetical protein
OsISM04878	LOC_Os01g37540	20965836	GTGGTGACGAGAGGCTTGG	GCAAGAAGGAGACGAGCG	60.8	171	97	transposon protein putative unclassified
OsISM04879	LOC_Os01g37580	21009543	TTTTCCCTCTTCCTTATGCG	GTTCGGACGGAAAGAAGCC	60.7	685	99	retrotransposon protein putative unclassified
OsISM04880	LOC_Os01g37580	21010229	GGCTGAGGTCCTTTCTTTCC	GTATGACACCAAGGGCAACC	60.2	518	93	retrotransposon protein putative unclassified
OsISM04881	LOC_Os01g37580	21011044	GCTAGGCTCCAGTCACGCTA	CCAGGACAGAACACATGGAA	60.1	573	92	retrotransposon protein putative unclassified
OsISM04882	LOC_Os01g37580	21011679	CCCATTGACAGTGAGCCTTT	TTCCCTATTAACGGCAGCAG	60.2	186	88	retrotransposon protein putative unclassified
OsISM04883	LOC_Os01g37580	21011863	AAAGGCGGCGTATGAAAATA	AAGGCAAGATGTCATCAGCA	59.5	211	94	retrotransposon protein putative unclassified
OsISM04884	LOC_Os01g37580	21012057	TGATGACATCTTGCCTTTGC	ATTCATGAGATGGACCACCA	59.3	166	90	retrotransposon protein putative unclassified

ISM IDs	Rice gene locus IDs	Physical positions (bp) on chromosomes	Forward primers (5'-3')	Reverse primers (5'-3')	Melting tempearature (T°C)	Expected product size (bp) in PCR	Expected product size (bp) in Q-PCR	Putative Functions
OsISM04885	LOC_Os01g37580	21012286	AGCGCAAGATTCTGTACAACC	CGGTCCCATTGTTTATCGTC	59.8	210	70	retrotransposon protein putative unclassified
OsISM04886	LOC_Os01g37580	21012821	TTTGCCAACGAGTGGTGATA	GGTCGGTTAGCACATCAGGT	60.1	964	96	retrotransposon protein putative unclassified
OsISM04887	LOC_Os01g37580	21015680	GACCAAAACCGTGTCGATCT	CGTAGTAGCATAATCGTCACACC	59.4	457	94	retrotransposon protein putative unclassified
OsISM04888	LOC_Os01g37580	21016152	TATGGGTCTATGCAATGGGA	TCACCGAGCTCAAGGTTAGG	59.6	733	45	retrotransposon protein putative unclassified
OsISM04889	LOC_Os01g37580	21016966	TGGTCTTGTTTTGGTTGGTG	CCTTCGCTGATCAGTGGTTT	59.8	172	91	retrotransposon protein putative unclassified
OsISM04890	LOC_Os01g37580	21017302	AAGAGCTCCGCTCTCGTGT	CACGTGTCCGAACAGTTGTC	60.3	190	94	retrotransposon protein putative unclassified
OsISM04891	LOC_Os01g37580	21017480	TTCGGACACGTGTTAGAGACA	GCTCTTTCTCCCCATAACACA	59.3	346	98	retrotransposon protein putative unclassified
OsISM04892	LOC_Os01g37580	21017847	AATTCTCAGTGCTGGCAAGG	CAGTGTCCCACGCTATGAGA	60.1	144	96	retrotransposon protein putative unclassified
OsISM04893	LOC_Os01g37580	21017971	TCTCATAGCGTGGGACACTG	TTGATTGCCTCGTAATTCCC	59.9	226	91	retrotransposon protein putative unclassified
OsISM04894	LOC_Os01g37580	21018266	AGACCCGCACCCTTTTAAGT	CGCATGCAGTATACCACCAA	60.3	184	96	retrotransposon protein putative unclassified
OsISM04895	LOC_Os01g37580	21018515	CCAACTGGCTCAGCTTCAC	GCGCTAAATGCCCAAAGATA	59.9	168	92	retrotransposon protein putative unclassified
OsISM04896	LOC_Os01g37580	21019093	GCACAGGATGCTGGTGATAA	CTTAGTTGCAGGGGCAGAAG	59.8	166	85	retrotransposon protein putative unclassified
OsISM04897	LOC_Os01g37580	21019239	CTTCTGCCCCTGCAACTAAG	CAGTTCCAGGAGATGGCCTA	60.1	159	70	retrotransposon protein putative unclassified
OsISM04898	LOC_Os01g37580	21019413	TGTTCAAAGATGATGCTGCTG	TAATCTGCCTGTTGCTGTCG	60.0	1360	79	retrotransposon protein putative unclassified
OsISM04899	LOC_Os01g37580	21020900	GGTACTTGGAGCTGGCAATC	TGTCCATGCAGCCAATCTAA	60.0	408	98	retrotransposon protein putative unclassified
OsISM04900	LOC_Os01g37590	21037786	CCCAGCTTACAGGAGACCAG	TTCTTCTCCACCTCCTGCAC	60.1	250	63	peptide transporter PTR2 putative expressed
OsISM04901	LOC_Os01g37700	21077018	GGATACACAAGATGGGTCCG	CACCACCCTTCTCCTCAACT	59.7	149	90	hypothetical protein
OsISM04903	LOC_Os01g37710	21082421	GCATTTGACCGCTGTAGGAG	GGGCAGCCTTAGAATCATGT	60.0	434	96	conserved hypothetical protein

ISM IDs	Rice gene locus IDs	Physical positions (bp) on chromosomes	Forward primers (5'-3')	Reverse primers (5'-3')	Melting tempearature (T˚C)	Expected product size (bp) in PCR	Expected product size (bp) in Q-PCR	Putative Functions
OsISM04904	LOC_Os01g37710	21083383	AAGATGGTAAAAATATGGATGGGA	CACAAGCCCATCAAACTCCT	60.0	243	96	conserved hypothetical protein
OsISM04905	LOC_Os01g37710	21083783	CTGGGAGGAATTTGGAGGTT	TTTGCATCGATAAAACCCAG	59.5	122	80	conserved hypothetical protein
OsISM04906	LOC_Os01g37710	21084132	CCTCTGATTCTATGATATCTCCTTCG	TCAGAAATAATTTGGGTCCTTGA	60.3	139	86	conserved hypothetical protein
OsISM04907	LOC_Os01g37710	21084345	TTAAAAATCTTTGTGGCGGG	CAAAACCATCCCTTAACAGCC	60.3	630	88	conserved hypothetical protein
OsISM04908	LOC_Os01g37760	21118783	TCGAGAGGCCGGTATATGTC	TCAGCTTCATGAGGGTCCTT	59.9	597	86	glutamate dehydrogenase protein putative expressed
OsISM04909	LOC_Os01g37760	21119410	TTCCTTGGAACCAGTTTTGG	CTCAGGTTCCAACAAACGCT	60.1	155	68	glutamate dehydrogenase protein putative expressed
OsISM04910	LOC_Os01g37760	21119689	CCATGACTTTGAGCGTAGCA	AAAATCACTCCCTCCAGCAG	59.6	676	99	glutamate dehydrogenase protein putative expressed
OsISM04911	LOC_Os01g37760	21120352	AGGGAGTGATTTTGATCCCA	CTGATCAGGCCCCAAATATC	59.3	175	98	glutamate dehydrogenase protein putative expressed
OsISM04912	LOC_Os01g37760	21120479	TGTCAAAGTTTCATGGATGAGC	AACCCATTTCTCTTGGACCC	60.1	212	92	glutamate dehydrogenase protein putative expressed
OsISM04913	LOC_Os01g37760	21120685	TGGGTTATCTTTTTGGGCAG	CCAGAAGATTTTAGGCCCTG	59.4	641	75	glutamate dehydrogenase protein putative expressed
OsISM04914	LOC_Os01g37760	21121295	GGGGAATTTTACAGGGCCTA	GCAAGCACTACACGTGCAAA	60.6	251	97	glutamate dehydrogenase protein putative expressed
OsISM04915	LOC_Os01g37760	21121514	TTGCAGGTATTCTTTGCACG	GCATTGCTATCTTTCCAGAACC	60.0	178	95	glutamate dehydrogenase protein putative expressed
OsISM04916	LOC_Os01g37760	21121698	TGGAAAAGCTTCTGTCATGTG	TCATGTAATCAAACCCTTCCG	59.4	619	88	glutamate dehydrogenase protein putative expressed
OsISM04917	LOC_Os01g37760	21122337	ATCAAGGCTCAGCAAAGGAG	CTTCTCACTCCAGGGCTTTG	59.8	165	88	glutamate dehydrogenase protein putative expressed
OsISM04918	LOC_Os01g37760	21122530	AAAATGAGATTGACCAGGCG	CTTGAGCAGTGCAAGGCATA	60.1	169	91	glutamate dehydrogenase protein putative expressed
OsISM04919	LOC_Os01g37760	21122722	AAAGTAGTTGTTGCTCCCGC	CCACTGACCACTGCATCAAA	60.1	291	98	glutamate dehydrogenase protein putative expressed
OsISM04920	LOC_Os01g37760	21122999	GCAGTGGTCAGTGGAGGATT	CCATAATCTTGAGCAGCTTTGA	59.8	181	88	glutamate dehydrogenase protein putative expressed
OsISM04921	LOC_Os01g37760	21123126	GGATGCGGTGAAACAAACAT	GTTTGCACCATGCACCAAC	60.9	182	95	glutamate dehydrogenase protein putative expressed

ISM IDs	Rice gene locus IDs	Physical positions (bp) on chromosomes	Forward primers (5'-3')	Reverse primers (5'-3')	Melting tempearature (T˚C)	Expected product size (bp) in PCR	Expected product size (bp) in Q-PCR	Putative Functions
OsISM04922	LOC_Os01g37790	21138275	ACGCCCATCAAGCTCCTC	CTCTCCACATCGAAATGGCT	60.8	1090	91	expressed protein
OsISM04923	LOC_Os01g37790	21140330	TGTTATGGAGGAAGATGCCC	TCCTGGGTTTTGAGATCTGG	60.0	480	87	expressed protein
OsISM04924	LOC_Os01g37825	21158276	CTACTACGTCCGCTCCAACC	GTCCTCCTCCTCCACCACC	60.8	187	86	M16 domain containing zinc peptidase putative expressed
OsISM04925	LOC_Os01g37825	21158607	ATCTACGAGCTGCTTGTCCC	GACACACGAACCTTTCACCA	59.5	188	90	M16 domain containing zinc peptidase putative expressed
OsISM04926	LOC_Os01g37825	21158858	TACTGGGAGGATGCAGGATT	CAGGCGTTCTGCATACTGAG	59.6	293	65	M16 domain containing zinc peptidase putative expressed
OsISM04927	LOC_Os01g37825	21159231	CAACATGGCTGTCTTTGCTG	TGGCCAAAGTGCTCCTTTAT	60.1	162	76	M16 domain containing zinc peptidase putative expressed
OsISM04928	LOC_Os01g37825	21159447	GCATGTTGAACCTCGGTTTT	AACAGCTGACTGTTTTGGGG	60.1	163	56	M16 domain containing zinc peptidase putative expressed
OsISM04929	LOC_Os01g37825	21159773	TTGTACGACCAGTGAAGGCA	CCGAACCCTAGCAACCTTTT	60.4	491	99	M16 domain containing zinc peptidase putative expressed
OsISM04930	LOC_Os01g37825	21160337	TGGAACGTGATCAGATGCAG	ATTCAATCCCGACAACAGGA	60.6	568	85	M16 domain containing zinc peptidase putative expressed
OsISM04931	LOC_Os01g37825	21160878	ATGAAGATCCTGTTGTCGGG	GCAAACTTTACCACTTCCGC	59.8	265	89	M16 domain containing zinc peptidase putative expressed
OsISM04932	LOC_Os01g37825	21161270	CCATGGGATGAAGAGCAAAT	TGTAGCACCAATTCCTGGGT	60.1	232	97	M16 domain containing zinc peptidase putative expressed
OsISM04933	LOC_Os01g37825	21161521	GCATGCGAATTTGCTACAAG	CAGACAGACCACCATAGGCA	59.6	1205	79	M16 domain containing zinc peptidase putative expressed
OsISM04934	LOC_Os01g37825	21162875	TCATTTTCTGGGGACTGCTC	CTCTCTTGGCTCCACCTTTG	60.1	848	88	M16 domain containing zinc peptidase putative expressed
OsISM04935	LOC_Os01g37825	21163801	AATCGTGCACGGGAGATAAA	ATTGGCTGCAAAAGGAACTC	59.9	132	48	M16 domain containing zinc peptidase putative expressed
OsISM04936	LOC_Os01g37825	21163999	GGATCCATCAGCTTTTACTGTTG	TCCCTACGTTAGGTATTCCACC	59.6	176	99	M16 domain containing zinc peptidase putative expressed
OsISM04937	LOC_Os01g37825	21164172	GGAATGCAGTTCTGCCACTTA	TAGGGCTGCGAACAACTTCT	60.1	324	91	M16 domain containing zinc peptidase putative expressed
OsISM04938	LOC_Os01g37825	21164503	AGCTCAATGCTTTGTGCAAC	TCCAGAAGTTTACTTAGAAACCCAA	59.3	186	97	M16 domain containing zinc peptidase putative expressed
OsISM04939	LOC_Os01g37825	21164685	TGGAGACGAAAATCATGCAA	TTCTTGAAGGTTTGTTGCCA	59.7	192	94	M16 domain containing zinc peptidase putative expressed

ISM IDs	Rice gene locus IDs	Physical positions (bp) on chromosomes	Forward primers (5'-3')	Reverse primers (5'-3')	Melting tempearature (T˚C)	Expected product size (bp) in PCR	Expected product size (bp) in Q-PCR	Putative Functions
OsISM04940	LOC_Os01g37825	21164899	AAGTGTGAATTTTTCATGCGA	CCAGCACAAAGTCAACCTGA	59.1	141	57	M16 domain containing zinc peptidase putative expressed
OsISM04941	LOC_Os01g37825	21165066	GGCCCTTCTGAAGAAGACGTA	CAGAACTCGATCCAGCCAGTA	60.6	172	97	M16 domain containing zinc peptidase putative expressed
OsISM04942	LOC_Os01g37825	21165218	ACTGGCTGGATCGAGTTCTG	CGTCCCTCATCTTGGATCTG	60.5	342	86	M16 domain containing zinc peptidase putative expressed
OsISM04943	LOC_Os01g37825	21165670	CATCCTGCTGGAACTCGTTT	GCCAAAATCTGCAGGAAAGA	60.3	170	74	M16 domain containing zinc peptidase putative expressed
OsISM04944	LOC_Os01g37832	21167035	ACTGGTCGCTCTTCAACCAC	CCACAGATTCGTTCAGAGCC	60.6	330	58	thioredoxin putative expressed
OsISM04945	LOC_Os01g37832	21167372	CGTCAAGTCGGTCTTCAAGC	GGGTCACCCTCATTACTCTCC	60.4	1173	76	thioredoxin putative expressed
OsISM04946	LOC_Os01g37832	21168510	CACCAGGGTTTTCTGGAGAG	CCACCAACAACAGAGATGCT	59.2	1073	99	thioredoxin putative expressed
OsISM04947	LOC_Os01g37832	21169568	CTCTGTTGTTGGTGGTGCAG	AGTCAATGCCTTCTCGGTTG	60.3	182	72	thioredoxin putative expressed
OsISM04948	LOC_Os01g37832	21169728	ATCAACCGAGAAGGCATTGA	ACTCCCTGCAAATTCTCTGC	60.0	172	84	thioredoxin putative expressed
OsISM04949	LOC_Os01g37832	21169861	TGATTAGGAATGGGAGCTGG	TTAGGTTAGCCACACCCTGG	60.0	490	87	thioredoxin putative expressed
OsISM04950	LOC_Os01g37832	21170396	ATTACATTGGGTTGCGTGGT	GTGGCCACAACATCCCTTT	60.4	138	53	thioredoxin putative expressed
OsISM04951	LOC_Os01g37832	21170511	AACAAAAGGGATGTTGTGGC	AACATGCGAGAAACCACCTC	60.0	692	97	thioredoxin putative expressed
OsISM04952	LOC_Os01g37837	21175045	GAGGTCATCGCCCTCGAC	GCTGGTTTTGTTGAGCTCCT	60.6	179	79	seryl-tRNA synthetase putative expressed
OsISM04953	LOC_Os01g37837	21175214	CAAAACCAGCAAGGAAATCG	TGGCAGCCAACCTCTTCTTA	60.8	187	96	seryl-tRNA synthetase putative expressed
OsISM04954	LOC_Os01g37837	21175447	CGTGACGATTGGAAACATTG	TACCCTCCAGCCTCCTCTCT	60.2	442	99	seryl-tRNA synthetase putative expressed
OsISM04955	LOC_Os01g37837	21175875	GAGGCTGGAGGGTAATTTGA	AGTAACCCCTTCCACCTGCT	59.6	1242	99	seryl-tRNA synthetase putative expressed
OsISM04956	LOC_Os01g37837	21177226	GGAAACCATGGGAAAATGTG	ACTTTTCCTCTCCATCGCCT	60.1	331	78	seryl-tRNA synthetase putative expressed
OsISM04957	LOC_Os01g37837	21177611	TTTATCCTGCTGAATTGCCA	CCTTCCATGTGAACCAGCTT	59.7	154	75	seryl-tRNA synthetase putative expressed

ISM IDs	Rice gene locus IDs	Physical positions (bp) on chromosomes	Forward primers (5'-3')	Reverse primers (5'-3')	Melting tempearature (T˚C)	Expected product size (bp) in PCR	Expected product size (bp) in Q-PCR	Putative Functions
OsISM04958	LOC_Os01g37837	21177842	ATCCTGGGAGATGCATGAAG	AGCACCAGACACAATGGAGA	59.6	190	95	seryl-tRNA synthetase putative expressed
OsISM04959	LOC_Os01g37837	21178104	GTGTCCTGCTCAAATTGCAC	ACGAATTGCTTGGATTGCTC	59.8	175	90	seryl-tRNA synthetase putative expressed
OsISM04960	LOC_Os01g37842	21180858	GGACGAAGAACAAGGGGAAG	AAGCGCTTGCAAGTTTCATT	60.5	549	80	expressed protein
OsISM04961	LOC_Os01g37842	21181426	CTTGTTTGGAATGCTGTTGG	ATCTGGCCTCCACACTTCTG	59.7	639	90	expressed protein
OsISM04962	LOC_Os01g37842	21182144	AAGGAAATTATGGCGAAGGG	TTCATTTCTGCTTGTTTGCG	60.1	369	79	expressed protein
OsISM04963	LOC_Os01g37850	21184044	ACCTCATCCCGATGGACA	GACCGGACCATCTCCTCC	60.1	129	44	expressed protein
OsISM04964	LOC_Os01g37850	21184148	CAGACCAGGAGGAGATGGTC	GTAGCCGAGGAGGAACCC	59.6	232	93	expressed protein
OsISM04965	LOC_Os01g37850	21184392	GTCTACTCCGGCTTTCACCA	CGGTGATGACAAATCTTCCA	59.9	561	79	expressed protein
OsISM04966	LOC_Os01g37850	21184942	TGTCATCACCGATGGTCGTA	CCACATCCACTTCTTCCAGG	60.7	817	96	expressed protein
OsISM04967	LOC_Os01g37850	21185737	CTCCTGGAAGAAGTGGATGTG	ATCCCATCGAATTCTCGGT	59.5	259	98	expressed protein
OsISM04968	LOC_Os01g37850	21185988	GATGGGATGTAGCATGGCTC	TCTTGCATTGACTCCAGCAT	59.9	186	89	expressed protein
OsISM04970	LOC_Os01g37870	21194858	GACTGCAACATAATCCTGCG	CCCGAAGACCTGAAACAAAG	59.5	155	67	PPR repeat domain containing protein putative expressed
OsISM04971	LOC_Os01g37870	21195187	GGGAGGTTCGATAGCAGCTT	TTCATGCACCCTGACAGAAG	60.3	196	99	PPR repeat domain containing protein putative expressed
OsISM04972	LOC_Os01g37870	21195607	ATGAAAAGGCTGATCTGTTGA	CAAAGTGGTGAGTATAACCTGCAA	58.9	951	75	PPR repeat domain containing protein putative expressed
OsISM04973	LOC_Os01g37870	21196583	TCTTTGAGAAGGCAAGGGAA	TTTGACAAGGCCATCAATCA	60.0	214	96	PPR repeat domain containing protein putative expressed
OsISM04974	LOC_Os01g37870	21196777	TGATTGATGGCCTTGTCAAA	CTGAATGCATAGCCATCTGC	59.7	1698	98	PPR repeat domain containing protein putative expressed
OsISM04975	LOC_Os01g37870	21198704	TGCATGGCAAAGGTCTATCA	CAGAACAAAGCTCCTGGAGAA	59.9	337	86	PPR repeat domain containing protein putative expressed
OsISM04976	LOC_Os01g37870	21199131	AATCTCTTCATGAGAAGGTTTTATGT	TCGGAATTGTCCTGTTCAAA	58.5	157	83	PPR repeat domain containing protein putative expressed

ISM IDs	Rice gene locus IDs	Physical positions (bp) on chromosomes	Forward primers (5'-3')	Reverse primers (5'-3')	Melting tempearature (T˚C)	Expected product size (bp) in PCR	Expected product size (bp) in Q-PCR	Putative Functions
OsISM04977	LOC_Os01g37870	21199330	CATGGCATCAGGCAACATAA	CCCAAAGATATCCTGCGTGT	60.2	378	86	PPR repeat domain containing protein putative expressed
OsISM04978	LOC_Os01g37880	21207784	AAGGTGGTCATTCAACCGAC	GATCTTCGGCCTCGGACT	60.1	181	87	transposon protein putative unclassified
OsISM04979	LOC_Os01g37910	21229678	CGGCTACTACAACTACCGCC	GATGTTCTCGTCCTTGAGCC	59.8	850	83	vacuolar-processing enzyme precursor putative expressed
OsISM04980	LOC_Os01g37910	21230600	CAGGGTGGCGATGTCTATG	CGAGCAGAACAGCAAACAAG	59.9	480	83	vacuolar-processing enzyme precursor putative expressed
OsISM04981	LOC_Os01g37910	21231152	TTTACAGTGACCATGGGGGT	GCAGCATGCTTCTTCTTCAG	59.5	209	98	vacuolar-processing enzyme precursor putative expressed
OsISM04982	LOC_Os01g37910	21231356	GCTGCTGGGACGTACAAAAG	GACCCTCAAAGATGCTTCCA	60.5	164	68	vacuolar-processing enzyme precursor putative expressed
OsISM04983	LOC_Os01g37910	21231628	ACACATGCCTAGGGGACTTG	CGAGATTGTACTGCTGCTTGA	59.6	926	94	vacuolar-processing enzyme precursor putative expressed
OsISM04984	LOC_Os01g37910	21232504	CGATGTCCACAACCTGAGAA	TGCTGCACAGATGTCCTTTC	59.8	153	73	vacuolar-processing enzyme precursor putative expressed
OsISM04985	LOC_Os01g37910	21232800	CTGTTAATCAGCGGGATGCT	CGAGCTTCGTTTTTCTCAGG	60.2	195	86	vacuolar-processing enzyme precursor putative expressed
OsISM04986	LOC_Os01g37910	21233114	CTGGCGAACCTCTTGTTGAT	GCTTCATTCCATACTGCGCT	60.3	297	94	vacuolar-processing enzyme precursor putative expressed
OsISM04987	LOC_Os01g37930	21247089	CTCCAAGGTGGTCGTTCAA	GATCTTTGGCCTCGGATTG	60.1	192	91	retrotransposon protein putative unclassified
OsISM04989	LOC_Os01g37970	21272348	CGAGCTCAAGCTACCAACG	CTTGAGCAAGCTCGTCTGC	59.9	307	75	hypothetical protein
OsISM04990	LOC_Os01g37980	21274719	GAGATCTCCGCCTCCCTC	CGATGCCAGACTTGATCTCC	60.3	197	95	vesicle transport v-SNARE protein putative expressed
OsISM04991	LOC_Os01g37980	21274894	TCGGAGATCAAGTCTGGCAT	CACTTGGCTGTTGGTTCCTT	60.5	447	80	vesicle transport v-SNARE protein putative expressed
OsISM04992	LOC_Os01g37980	21275446	GGGAAGAGTTGCTGGAGTCA	TGCTTTTCAGTTATCCTGAGCA	60.2	1014	86	vesicle transport v-SNARE protein putative expressed
OsISM04993	LOC_Os01g37980	21276517	TCTTGGAGTCTCCCTCTTGC	TCTTCCCGATGTTGTCATCC	60.2	901	96	vesicle transport v-SNARE protein putative expressed
OsISM04994	LOC_Os01g37990	21281702	CGGACGTCTGATACATGCTC	CTGTCCATCCTCCTCACCAT	59.6	696	97	conserved hypothetical protein
OsISM04995	LOC_Os01g37990	21282322	TGCAGTTGCATAACGTGGAT	CCAATTATTCCCAACCCAAA	59.8	464	86	conserved hypothetical protein

ISM IDs	Rice gene locus IDs	Physical positions (bp) on chromosomes	Forward primers (5'-3')	Reverse primers (5'-3')	Melting tempearature (T˚C)	Expected product size (bp) in PCR	Expected product size (bp) in Q-PCR	Putative Functions
OsISM04996	LOC_Os01g37990	21282813	ATTTTTGGCAGGAAACATGG	CTCCTGAACCTTCTCGTCCA	60.1	192	83	conserved hypothetical protein
OsISM04997	LOC_Os01g37990	21283182	AACTCAAGGATGAAGCAGCC	GTCGGATCTCCGAGCTTGT	59.9	553	76	conserved hypothetical protein
OsISM04998	LOC_Os01g38020	21294785	TTGTTCGTGGGAGCTATTCC	TTCAAATCTTCGGAGTCAACC	59.6	480	96	retrotransposon protein putative unclassified
OsISM04999	LOC_Os01g38080	21329805	ATGGCGGCATAATAGGTCAG	CAAATCAGTCAGGGGGAAAC	59.7	783	90	retrotransposon protein putative unclassified
OsISM05002	LOC_Os01g38140	21353629	CGTGGAGTGGGGAATGATAA	ACATGGACACTGACATCCCA	60.3	251	89	retrotransposon protein putative unclassified
OsISM05003	LOC_Os01g38140	21353852	CTGGAGGATGGGATGTCAGT	GTGCAAAGGCAGGACAAAAG	60.4	173	80	retrotransposon protein putative unclassified
OsISM05004	LOC_Os01g38140	21354005	CTTTTGTCCTGCCTTTGCAC	GTCTACCCCGTTCACCAAAA	60.3	1497	99	retrotransposon protein putative unclassified
OsISM05005	LOC_Os01g38140	21355634	ACTTGGAAGGACAGCACACC	AGCCCCATCATGCAGTTTAC	60.1	173	72	retrotransposon protein putative unclassified
OsISM05006	LOC_Os01g38140	21355825	CACCAAGGAGATGAGCCATT	AGACCTCTCCCTCCCACATT	60.0	173	95	retrotransposon protein putative unclassified
OsISM05007	LOC_Os01g38140	21356051	CCTGGTTCTTCCGTCACCTA	CTCAACGCTCTCTGCTTGACT	60.0	186	88	retrotransposon protein putative unclassified
OsISM05008	LOC_Os01g38140	21356245	TGAGAAGGAAAAGGAGGGTG	TTTCCAGGGTCGTATTCCAA	59.8	1612	94	retrotransposon protein putative unclassified
OsISM05009	LOC_Os01g38140	21357892	CCGAGGCTTCGCTTGTAATA	AATGGAATGCTGTTCCTTCG	60.2	513	78	retrotransposon protein putative unclassified
OsISM05010	LOC_Os01g38140	21358574	CGACAAACATCGAGAACCAA	CTGGACGATGACGAAGACG	60.1	1003	89	retrotransposon protein putative unclassified
OsISM05011	LOC_Os01g38140	21359971	TCGCTGACATGTGCTACCTC	ACCTGCATTTCCGTTCGATA	60.2	1026	73	retrotransposon protein putative unclassified
OsISM05012	LOC_Os01g38140	21361349	GCCACGTAGCTGCTCAGG	GGACGTCCACCACCTTCTC	60.6	372	82	retrotransposon protein putative unclassified
OsISM05013	LOC_Os01g38140	21361832	CTCTCGATGACGAAGAAGGG	GTTGTCGGGCCACTGATTAT	59.9	283	89	retrotransposon protein putative unclassified
OsISM05014	LOC_Os01g38140	21362141	GTGAGAAAGGGGTAGCCGA	CCGATGATATCAGTCAGAGGG	59.9	131	62	retrotransposon protein putative unclassified
OsISM05015	LOC_Os01g38140	21362409	GACATCATCGGAAGACACCC	CGAAAAGCTGCAACTGAGAC	59.6	229	50	retrotransposon protein putative unclassified

ISM IDs	Rice gene locus IDs	Physical positions (bp) on chromosomes	Forward primers (5'-3')	Reverse primers (5'-3')	Melting tempearature (T°C)	Expected product size (bp) in PCR	Expected product size (bp) in Q-PCR	Putative Functions
OsISM05016	LOC_Os01g38140	21363381	AGTCCGTCCTCCCACTTTTT	GAACGATCACTGGCAACTCA	59.9	220	82	retrotransposon protein putative unclassified
OsISM05017	LOC_Os01g38140	21363641	AGATCAAAGTTCGCCTGTGG	AATGCGACGATCCTGGTTAG	60.2	195	83	retrotransposon protein putative unclassified
OsISM05018	LOC_Os01g38140	21363896	AAGGACCAGGCCGTAGAGAC	GATACGTTGACCGATGCCTT	60.3	195	58	retrotransposon protein putative unclassified
OsISM05019	LOC_Os01g38170	21375878	CATCAAGGGTTTGTTGCTGT	CCCAAGATGGCGTCATAAGT	59.3	155	84	retrotransposon protein putative unclassified
OsISM05020	LOC_Os01g38170	21376141	CGTCTCTCATTGTGCTGGAA	GCATGATCAAATTCACGAGC	59.6	207	94	retrotransposon protein putative unclassified
OsISM05021	LOC_Os01g38170	21376328	GCTCGTGAATTTGATCATGC	GCATTTGGAGCATTTGACCT	59.7	617	79	retrotransposon protein putative unclassified
OsISM05022	LOC_Os01g38170	21377297	TGGGAGCTGTTCTTATGCAA	TCACAAAAGTCTGCACTGCC	59.7	654	52	retrotransposon protein putative unclassified
OsISM05023	LOC_Os01g38170	21378206	TTCATGGACTTCCTCTGGCT	TGTACCAAAATTCAGGAAGCG	60.0	236	78	retrotransposon protein putative unclassified
OsISM05024	LOC_Os01g38170	21378464	GGGTTCTTCCCCATTTAAGG	ATCTTTCATTCGTTGCTGGG	59.9	169	81	retrotransposon protein putative unclassified
OsISM05025	LOC_Os01g38180	21382944	GGAGCGATAAGCCTTGTGAT	ACTGACATCCCATCCTCCAG	59.6	242	71	peptidyl-prolyl isomerase putative expressed
OsISM05026	LOC_Os01g38180	21383166	CTGGAGGATGGGATGTCAGT	TGCAAATGCAGGACAAAAGA	60.1	172	79	peptidyl-prolyl isomerase putative expressed
OsISM05027	LOC_Os01g38180	21383338	CGTGCTGTGGAGACTATGACA	GTCTACCCCGTTCACCAAAA	59.9	368	80	peptidyl-prolyl isomerase putative expressed
OsISM05028	LOC_Os01g38180	21383834	GGAAATTTGGAAGGACAGCA	AATACAGCCCCATCTTGCAG	60.1	193	81	peptidyl-prolyl isomerase putative expressed
OsISM05029	LOC_Os01g38180	21384007	CTGCAAGATGGGGCTGTATT	CCTCTAGGCCTTCGCTAACC	60.2	172	95	peptidyl-prolyl isomerase putative expressed
OsISM05030	LOC_Os01g38180	21384266	TTGGCTCTTCCGTGACCTAC	CAGCTTCGATGGTCTCTGCT	60.5	244	91	peptidyl-prolyl isomerase putative expressed
OsISM05031	LOC_Os01g38180	21384512	GCTGAAAAGGAAAAGGAGGG	CGCAGGAGTATGATCTGCC	59.8	247	96	peptidyl-prolyl isomerase putative expressed
OsISM05032	LOC_Os01g38180	21384851	ATGAACAAGCATACCACCGC	GCTTTCACATTTCCAGGGTC	60.2	201	59	peptidyl-prolyl isomerase putative expressed
OsISM05033	LOC_Os01g38180	21385089	CTTGGAATAGACACTGCCGC	GAGAACTCCGGGGTCATGTA	60.4	3134	99	peptidyl-prolyl isomerase putative expressed

ISM IDs	Rice gene locus IDs	Physical positions (bp) on chromosomes	Forward primers (5'-3')	Reverse primers (5'-3')	Melting tempearature (T˚C)	Expected product size (bp) in PCR	Expected product size (bp) in Q-PCR	Putative Functions
OsISM05034	LOC_Os01g38229	21414433	CGCCAAGGACAAGAACTTTTA	CTTGACACGAACTGCGTACC	59.4	8830	82	peptidyl-prolyl isomerase putative expressed
OsISM05035	LOC_Os01g38229	21423253	TCGTGTCAAGCCGAGAAAAT	CTGGTTGCATCGAACTGACA	60.8	265	97	peptidyl-prolyl isomerase putative expressed
OsISM05036	LOC_Os01g38229	21423690	TGTCAAGAACGCAGAACCC	AAGTTCCATCCATCAGGCAT	59.6	748	87	peptidyl-prolyl isomerase putative expressed
OsISM05037	LOC_Os01g38229	21424418	ATGCCTGATGGATGGAACTT	ATGTGCAAATGCAGGACAGA	59.8	189	86	peptidyl-prolyl isomerase putative expressed
OsISM05038	LOC_Os01g38229	21424620	TGAAAGAAGGAGAGGAAGCA G	AGGTCTTCCTTGTTCACCGA	59.2	368	66	peptidyl-prolyl isomerase putative expressed
OsISM05039	LOC_Os01g38229	21425130	CACAAAGTCAAGCTGTGGTG	AACTCGAACGGTTCATCGTC	59.0	5524	98	peptidyl-prolyl isomerase putative expressed
OsISM05040	LOC_Os01g38229	21430642	ACCGTTCGAGTTCGTGGTAG	ACAGATTCATCCAGGCCATC	60.0	161	55	peptidyl-prolyl isomerase putative expressed
OsISM05041	LOC_Os01g38229	21430878	TCAGCATCAGTTTGCCTTTG	GCTCGGAATGTACAACGGAT	60.0	1109	98	peptidyl-prolyl isomerase putative expressed
OsISM05042	LOC_Os01g38229	21432250	CATGCAAGCATCGTGCTATT	TTGCACATTAGCCACATCGT	60.0	4930	56	peptidyl-prolyl isomerase putative expressed
OsISM05043	LOC_Os01g38229	21437220	GTTCCTCAAGAGACCCGGAC	CGATACGGATGCTCACCTTT	60.6	2794	76	peptidyl-prolyl isomerase putative expressed
OsISM05044	LOC_Os01g38260	21463436	ACCAATCTCGCTTAGCTCCA	TTCCTCCGTCTCATTATCGG	60.0	247	79	retrotransposon protein putative Ty3-gypsy subclass
OsISM05045	LOC_Os01g38270	21467353	GCTTTAGCAGATTTCGTCGC	CGCGACGTTGTGAGATGTAG	60.3	198	85	retrotransposon protein putative Ty3-gypsy subclass
OsISM05047	LOC_Os01g38320	21494158	CGGTTCGAGGATGTAAACCTT	ATGGGGTCGATGAGTCTGTC	60.1	156	68	retrotransposon protein putative unclassified
OsISM05048	LOC_Os01g38359	21525053	CGAGGATGATGATGCCGTAT	GGGTTGAAACGAACTCGGTA	60.4	15186	84	peptidyl-prolyl cis-trans isomerase FKBP-type putative expressed
OsISM05049	LOC_Os01g38359	21540219	TACCGAGTTCGTTTCAACCC	CAAGCTTAAGCCATGCATCA	60.0	3834	83	peptidyl-prolyl cis-trans isomerase FKBP-type putative expressed
OsISM05050	LOC_Os01g38359	21544271	ATCTCCCTCCAATCATGCAG	TCCGTCTTCTAGACATGCGTT	60.0	435	59	peptidyl-prolyl cis-trans isomerase FKBP-type putative expressed
OsISM05051	LOC_Os01g38359	21544734	AATTGAACCTTGCCAGTCGT	GCTTCCTCTCCTTCCCTCAT	59.7	181	80	peptidyl-prolyl cis-trans isomerase FKBP-type putative expressed
OsISM05052	LOC_Os01g38359	21544895	ATGAGGGAAGGAGAGGAAGC	CCAGTAGAATCCCTGCCTTG	59.7	1034	75	peptidyl-prolyl cis-trans isomerase FKBP-type putative expressed

ISM IDs	Rice gene locus IDs	Physical positions (bp) on chromosomes	Forward primers (5'-3')	Reverse primers (5'-3')	Melting tempearature (T˚C)	Expected product size (bp) in PCR	Expected product size (bp) in Q-PCR	Putative Functions
OsISM05053	LOC_Os01g38359	21545973	TCCAGTTAGCTGAACGGAAGA	CATTCGGGCCATCTATGAAT	59.9	1515	73	peptidyl-prolyl cis-trans isomerase FKBP-type putative expressed
OsISM05054	LOC_Os01g38359	21547468	ATTCATAGATGGCCCGAATG	CTACAGGCAAGGAGAATCCC	59.2	865	87	peptidyl-prolyl cis-trans isomerase FKBP-type putative expressed
OsISM05055	LOC_Os01g38400	21569395	GGTGTTTTGGCGGATACATT	CTCTATCGAACCATTCGGGA	59.9	301	59	retrotransposon protein putative Ty3-gypsy subclass
OsISM05056	LOC_Os01g38410	21584838	CAGCTCTAGCTCTAGCCCCA	CTGCCATTCCTGGTGTTGAC	60.7	563	89	retrotransposon protein putative Ty3-gypsy subclass
OsISM05057	LOC_Os01g38420	21586532	CGCAAGTAGCACCAGAGGTC	ACTTGGTCAGCCAATCCATC	60.5	304	58	retrotransposon protein putative Ty3-gypsy subclass
OsISM05058	LOC_Os01g38420	21587492	AACGCACCTGCATTCTTCAT	GGCCGTCCATTTAAATTTCTC	60.2	423	94	retrotransposon protein putative Ty3-gypsy subclass
OsISM05059	LOC_Os01g38420	21588230	CCGGATCTAAATCTCCGACA	CTAGAGTGGCTACGAACCCG	60.0	220	98	retrotransposon protein putative Ty3-gypsy subclass
OsISM05060	LOC_Os01g38420	21588867	GTCTGCCCAGGACATCATCT	TGCGGATGGTAGGCTGTACT	60.4	262	92	retrotransposon protein putative Ty3-gypsy subclass
OsISM05061	LOC_Os01g38440	21605281	TGGTCTGGTCATGCTAAGGA	TGAGCTTAAGACCCAAGACCC	59.9	97	65	transposon protein putative unclassified
OsISM05062	LOC_Os01g38440	21605410	GAGCGTGTGATTGTTGTTGC	CACCAGACAAAACAACAGTTGA A	60.2	522	74	transposon protein putative unclassified
OsISM05063	LOC_Os01g38450	21608773	GAGAGTGGAAGAAGGCATGG	TGACCCAGGTTAGTAGCTTCG	59.6	1100	99	hypothetical protein
OsISM05064	LOC_Os01g38460	21614808	GTCTTGATCGTCCCAGAGGA	TCACTAGCACTTGACCCACG	60.0	365	77	retrotransposon protein putative unclassified
OsISM05065	LOC_Os01g38460	21615201	GACTCCCGTTTGCGAGTTT	ATCCACTTTTTCCCCCATTC	60.1	1308	71	retrotransposon protein putative unclassified
OsISM05066	LOC_Os01g38540	21646388	GAGAGGCTTGGCACCTCTG	CCGCAGGAAGGAGACGAG	61.6	165	91	transposon protein putative unclassified
OsISM05067	LOC_Os01g38600	21674515	GAGTGTGTCGACCACGGAG	TCCTCCACCACACGACACT	60.2	267	73	hypothetical protein
OsISM05068	LOC_Os01g38600	21674855	GTAGAGGTGGTTGCGGAGAA	CTCTCCTTCACGAGCCACAC	60.6	241	96	hypothetical protein
OsISM05069	LOC_Os01g38670	21718960	GACCATCTTCGTCTTCCTCG	ACGAGTCCATCGACGTCAC	59.7	184	99	transporter family protein putative expressed
OsISM05070	LOC_Os01g38670	21719383	GTCGCCATGCTCATCATC	GCCATCTCCGACAGGTACAG	59.6	380	87	transporter family protein putative expressed

ISM IDs	Rice gene locus IDs	Physical positions (bp) on chromosomes	Forward primers (5'-3')	Reverse primers (5'-3')	Melting tempearature (T˚C)	Expected product size (bp) in PCR	Expected product size (bp) in Q-PCR	Putative Functions
OsISM05071	LOC_Os01g38670	21720319	CGCAAGCTCCTCCTTCAG	CGATCAGCGTCCCAAGAAT	60.5	150	65	transporter family protein putative expressed
OsISM05072	LOC_Os01g38690	21729353	GGTATAGGCAGGTGGAGCAG	AGTGAGAGGAGGACACCCG	60.0	461	93	nucleic acid binding protein putative
OsISM05073	LOC_Os01g38690	21729965	TTGGCTTCATCATTGGTGTC	TTACATGGCTGTTTGATCCG	59.5	356	85	nucleic acid binding protein putative
OsISM05074	LOC_Os01g38690	21730265	TCTTTTAGTTTACAATTTTGCTCCTTT	GCTTCGACTCCTTGGTACTCC	59.8	1173	85	nucleic acid binding protein putative
OsISM05075	LOC_Os01g38690	21731417	GGAGTACCAAGGAGTCGAAGC	AGGAGACGTGTCGTACTCGC	60.4	787	98	nucleic acid binding protein putative
OsISM05076	LOC_Os01g38720	21741264	ATGGCAGCATGTTTACACCA	CCCAAAAAGCCCACATTCTA	60.0	379	88	nucleic acid binding protein putative
OsISM05077	LOC_Os01g38720	21741635	TTTTTGGGCTTTCAGCCTTA	TGTGATGCCCTGGTCAAATA	59.9	179	93	nucleic acid binding protein putative
OsISM05078	LOC_Os01g38720	21741794	TATTTGACCAGGGCATCACA	AAGCGAAATTCGAAGGTTGA	59.9	353	57	nucleic acid binding protein putative
OsISM05079	LOC_Os01g38730	21745469	CTCCTGGTGACGAGAAGGAC	CTGGGTGATCCGGGTTAGTA	59.8	1281	81	retrotransposon protein putative Ty3-gypsy subclass
OsISM05080	LOC_Os01g38740	21749132	GATGACGACGGCGATGAC	AATTTGGCCCAACATCCTTT	60.9	731	68	retrotransposon protein putative Ty1-copia subclass
OsISM05081	LOC_Os01g38740	21749899	AGCCCAAAAAGTCCAACAAA	ACAATGGGCACAAATGGAGT	59.9	521	83	retrotransposon protein putative Ty1-copia subclass
OsISM05082	LOC_Os01g38740	21750400	ACTCCATTTGTGCCCATTGT	GAAAGAGCCCTCTCCTTCAAA	60.1	823	87	retrotransposon protein putative Ty1-copia subclass
OsISM05083	LOC_Os01g38740	21751267	AAACCGAGGTCCGAAGAAAG	TGGAACTTTTGCAATGTTCTGT	60.1	248	94	retrotransposon protein putative Ty1-copia subclass
OsISM05084	LOC_Os01g38740	21751858	GCTCATCAAGGGTAACATGG	CACTCATGTGCCCAAGTCTC	58.6	147	99	retrotransposon protein putative Ty1-copia subclass
OsISM05085	LOC_Os01g38740	21752154	TTGGTGCACTCTGATGTATGC	ATGCCTAATCCCTTGCTCCT	59.9	611	97	retrotransposon protein putative Ty1-copia subclass
OsISM05086	LOC_Os01g38750	21754209	AGGGAGTCGCACTCGACG	GCGTCTTCATCACCTGCAA	61.8	386	68	hypothetical protein
OsISM05088	LOC_Os01g38880	21814648	CCCACCTACTCCTTTCGTGA	TCAGTGCTGGTGTTGAGCTT	59.9	120	75	hypothetical protein
OsISM05089	LOC_Os01g38880	21815256	CTTCTCTCGAAAGTCGCCAT	GACGATGTGGAGATCACCCT	59.8	732	84	hypothetical protein

ISM IDs	Rice gene locus IDs	Physical positions (bp) on chromosomes	Forward primers (5'-3')	Reverse primers (5'-3')	Melting tempearature (T˚C)	Expected product size (bp) in PCR	Expected product size (bp) in Q-PCR	Putative Functions
OsISM05090	LOC_Os01g38940	21875231	GGCAAAGGAATTGCTCATGT	GCCACTCTGTTAGGCTTTGC	60.1	216	98	hypothetical protein
OsISM05091	LOC_Os01g38950	21878233	GTGGGCAACAAGTACCGC	CTCGTTGGTCTGCACGTTAG	59.8	205	85	CK1 CaseinKinase 1.2 - CK1 includes the casein kinase 1 kinases expressed
OsISM05092	LOC_Os01g38950	21878418	CTAACGTGCAGACCAACGAG	CAGCAATTGTGGATGCTTTG	59.9	705	69	CK1 CaseinKinase 1.2 - CK1 includes the casein kinase 1 kinases expressed
OsISM05093	LOC_Os01g38950	21879117	TTGCTGTACGAGTCAAAGCTG	CCCTCAACACCAAACCACTT	59.6	175	78	CK1 CaseinKinase 1.2 - CK1 includes the casein kinase 1 kinases expressed
OsISM05094	LOC_Os01g38950	21879373	AAAAACTGTTCTAATGCTTGC TGA	TCGATGCAAGAAAGACTTTGA	59.1	188	74	CK1 CaseinKinase 1.2 - CK1 includes the casein kinase 1 kinases expressed
OsISM05095	LOC_Os01g38950	21879573	GACAATTTTCTCATGGGCCT	TGCCAGTCCAAAATCAATCA	59.5	162	64	CK1 CaseinKinase 1.2 - CK1 includes the casein kinase 1 kinases expressed
OsISM05096	LOC_Os01g38950	21879741	TACCGGGACACTTCAACACA	CGATTCCAAGATGGGTGTTT	59.9	372	98	CK1 CaseinKinase 1.2 - CK1 includes the casein kinase 1 kinases expressed
OsISM05097	LOC_Os01g38950	21880067	ACTGGGACAGCTCGATATGC	TTCAAGGTCATCCCTTCTGC	60.2	204	70	CK1 CaseinKinase 1.2 - CK1 includes the casein kinase 1 kinases expressed
OsISM05098	LOC_Os01g38950	21880251	GCAGAAGGGATGACCTTGAA	GGTCCCAGCTTTTAGACCCT	59.9	172	87	CK1 CaseinKinase 1.2 - CK1 includes the casein kinase 1 kinases expressed
OsISM05099	LOC_Os01g38950	21880445	TTAGCGAACGGAAGATTGCT	TCAAAACGTAGTGAACGGCA	60.1	178	98	CK1 CaseinKinase 1.2 - CK1 includes the casein kinase 1 kinases expressed
OsISM05100	LOC_Os01g38950	21880615	CGTTTTGAAGATTTGCCAGA	TGGTCCAGTCAAAAACGTAATC	58.9	176	98	CK1 CaseinKinase 1.2 - CK1 includes the casein kinase 1 kinases expressed
OsISM05101	LOC_Os01g38950	21880813	GATAACCAGTGTTCCACCACG	AGACATTCCAGAGCTTTGCC	59.9	144	59	CK1 CaseinKinase 1.2 - CK1 includes the casein kinase 1 kinases expressed
OsISM05102	LOC_Os01g38950	21880909	GCTCTTTTGTTTCAGGCTGC	CGCTTCTTCTTCCCTCTTCA	59.9	965	98	CK1 CaseinKinase 1.2 - CK1 includes the casein kinase 1 kinases expressed
OsISM05103	LOC_Os01g38950	21881957	AGGCCAGACATGTCCACTTC	CCTTAAGGATCCGCTTGACC	60.5	199	76	CK1 CaseinKinase 1.2 - CK1 includes the casein kinase 1 kinases expressed
OsISM05104	LOC_Os01g38970	21892992	GCTTTGAGCCTTCCATTGAC	CAGACCCAGGGAACTTTTCA	60.0	1179	70	carbamoyl-phosphate synthase large chain putative expressed
OsISM05105	LOC_Os01g38970	21894773	GGCCAGGGTATTGAGTTTGA	TGTCTCTGGGTTGGAGTTCA	59.6	281	97	carbamoyl-phosphate synthase large chain putative expressed
OsISM05106	LOC_Os01g38990	21917962	ATTCGCAAAGACTGCGGTAG	TGATTGGAGGATACCAAGGG	60.1	193	92	mitochondrial carrier protein putative expressed
OsISM05107	LOC_Os01g38990	21918154	ATTGAGGAAGCTGTGGCAGT	GGAACAATCCGAAGCACACT	60.0	170	76	mitochondrial carrier protein putative expressed

ISM IDs	Rice gene locus IDs	Physical positions (bp) on chromosomes	Forward primers (5'-3')	Reverse primers (5'-3')	Melting tempearature (T°C)	Expected product size (bp) in PCR	Expected product size (bp) in Q-PCR	Putative Functions
OsISM05108	LOC_Os01g38990	21918474	CCTTGCTAGGACAAAGCTGG	CCATATGCTGGCTGTCTACC	59.1	302	88	mitochondrial carrier protein putative expressed
OsISM05109	LOC_Os01g38990	21918814	GAGGCGCACGAGCTTTATAC	TCGCGATTTCAGGTCTTCAT	60.4	842	99	mitochondrial carrier protein putative expressed
OsISM05110	LOC_Os01g38990	21919723	GGCAAACCCTCACTTATCCA	GAAGGCATCGTTCGCATTAT	60.0	175	84	mitochondrial carrier protein putative expressed
OsISM05111	LOC_Os01g38990	21919955	CAACTCTTCGCTGGCCTAAG	TGTGAAACCAATAGCAACCG	59.9	249	64	mitochondrial carrier protein putative expressed
OsISM05112	LOC_Os01g39010	21932504	GGAGAGATCTCCTTCCCCTG	CTCCAGGGGTTCATTAGCAA	60.1	1775	85	50S ribosomal protein putative expressed
OsISM05113	LOC_Os01g39010	21934309	ACCATCAAGCGGAAAAGAAC	ATCACCTTACGCCCACCTTT	60.0	321	66	50S ribosomal protein putative expressed
OsISM05114	LOC_Os01g39020	21938234	CTTCAAGCACGCCAACTTCT	CACCGATCTGGAGTGACCTT	60.3	1484	76	HSF-type DNA-binding domain containing protein expressed
OsISM05115	LOC_Os01g39060	21957424	TGACTTTGCTCCATTGCTTG	CAACATCGCAATGATCTCCA	60.3	258	99	expressed protein
OsISM05116	LOC_Os01g39060	21957770	AATTTGCGTACATCCCTTGG	GCCTCATTGATGCGAAAACT	60.0	3115	71	expressed protein
OsISM05117	LOC_Os01g39080	21967303	GTCTTTGCCGTTGGTTACAA	CCAAAAGCACGGTAAGGCT	59.5	130	53	hypothetical protein
OsISM05118	LOC_Os01g39100	21973722	GCGCAAGCAGTCAGTTATGA	AGGTGCGCTTTCTTGTGTTT	60.0	464	95	zinc finger C-x8-C-x5-C-x3-H type family protein expressed
OsISM05119	LOC_Os01g39100	21974172	AAGAAAGCGCACCTTGACAG	TCAGTCTTTTCACCCCTTCG	60.4	219	99	zinc finger C-x8-C-x5-C-x3-H type family protein expressed
OsISM05120	LOC_Os01g39100	21974417	GTGGTGAAGGTTTGCCACTT	CATGGGAGAACTTGCACAAA	59.9	694	68	zinc finger C-x8-C-x5-C-x3-H type family protein expressed
OsISM05121	LOC_Os01g39100	21975092	TTGTGCAAGTTCTCCCATGA	GGGCAATCATCTCCTTTCAA	60.1	472	93	zinc finger C-x8-C-x5-C-x3-H type family protein expressed
OsISM05122	LOC_Os01g39100	21975613	GGAATGTGCATCAGAGGTGA	TTCTTTGCATCTGGTGTGGA	59.9	195	81	zinc finger C-x8-C-x5-C-x3-H type family protein expressed
OsISM05123	LOC_Os01g39100	21975935	AACCTTGCTGGCATATCAGG	ATGTGGACCACCAGTTGGAT	60.1	159	79	zinc finger C-x8-C-x5-C-x3-H type family protein expressed
OsISM05124	LOC_Os01g39100	21976232	AGTTTGCCTACCACTGCTGC	AATGCTGCACAATCTCATGG	60.1	249	85	zinc finger C-x8-C-x5-C-x3-H type family protein expressed
OsISM05125	LOC_Os01g39100	21976489	TCCGCACACCAGTTTTATTG	GCCCCAAGTTCTGCAAAATA	59.8	152	79	zinc finger C-x8-C-x5-C-x3-H type family protein expressed

ISM IDs	Rice gene locus IDs	Physical positions (bp) on chromosomes	Forward primers (5'-3')	Reverse primers (5'-3')	Melting tempearature (T˚C)	Expected product size (bp) in PCR	Expected product size (bp) in Q-PCR	Putative Functions
OsISM05126	LOC_Os01g39100	21976682	TGAATGCACTTCTGTTCAATCC	ACTGTTGGCTTCTTGCTGCT	60.2	2090	87	zinc finger C-x8-C-x5-C-x3-H type family protein expressed
OsISM05127	LOC_Os01g39100	21978767	ACAGTGCAGCAAAAGGGAGT	TCAACAGTCACCTTTATTGAGGAA	60.0	405	78	zinc finger C-x8-C-x5-C-x3-H type family protein expressed
OsISM05128	LOC_Os01g39100	21979128	TTTTACAGGTTTTAAGTATGTTCCTCA	ATGGCGCTTAGCATTTTCAT	59.2	301	93	zinc finger C-x8-C-x5-C-x3-H type family protein expressed
OsISM05129	LOC_Os01g39110	21992705	AACAGCAACAAGAGGAAGCG	AACCTGTTCGTCGCCATC	60.1	191	96	ZOS1-10 - C2H2 zinc finger protein expressed
OsISM05130	LOC_Os01g39110	21993200	CGCGAGTACAAGTGCGATT	GGTGCGTGATGAAGCTGT	59.2	1359	59	ZOS1-10 - C2H2 zinc finger protein expressed
OsISM05131	LOC_Os01g39170	22039639	GAGCCGTGGTGGTCATGT	CAAACGGTACCCACAGCAG	60.4	462	98	hypothetical protein
OsISM05132	LOC_Os01g39180	22041998	CGAGATCTCCAACCACACCT	ACGGATCGTCCACCATTG	60.2	1515	94	DUF260 domain containing protein putative
OsISM05133	LOC_Os01g39190	22045412	ACGTTACCTGGATACGGCAG	CAAGCACCACCAGATCAAGA	59.9	405	67	retrotransposon protein putative unclassified
OsISM05134	LOC_Os01g39190	22046230	GCTTGTAACTTCGTCTGGGA	CCGGTGCAGATCAACCTTA	58.8	323	99	retrotransposon protein putative unclassified
OsISM05135	LOC_Os01g39190	22046606	TCTCGTACGGTTGTAGTCGG	CACCTTGGATGTCACCTGG	59.4	1136	78	retrotransposon protein putative unclassified
OsISM05136	LOC_Os01g39190	22048492	CGTCACAATTGCTGCATGTT	GTTGGCCTTGGAACTCTTCA	60.5	2148	91	retrotransposon protein putative unclassified
OsISM05137	LOC_Os01g39200	22053213	GAAAGAAGTTACCCCAGCCC	CCGTTGCTGCGAGTATTCA	60.5	458	64	retrotransposon protein putative Ty3-gypsy subclass expressed
OsISM05138	LOC_Os01g39200	22054162	AGTTCAATCGTCTGGCTCGT	CTTGATTGCGGATGTTGTTG	60.0	332	79	retrotransposon protein putative Ty3-gypsy subclass expressed
OsISM05139	LOC_Os01g39250	22073439	GTTTGGGATCTCATCGAGGC	TAGATTGTGGATGCTGGTCG	60.8	928	99	OsFtsH4 FtsH protease homologue of AtFtsH4 expressed
OsISM05140	LOC_Os01g39250	22074390	CTTCGTGGCTGCTTAGAGGT	CACCCTGCGTGCTAAGCTA	59.9	431	99	OsFtsH4 FtsH protease homologue of AtFtsH4 expressed
OsISM05141	LOC_Os01g39250	22074975	GGCTGGATGATAGCACCTTG	CTCTCCTCCTGAAACAGCCA	60.6	818	60	OsFtsH4 FtsH protease homologue of AtFtsH4 expressed
OsISM05142	LOC_Os01g39250	22075955	CTTGTAATTTCGGGCATTGG	CCATGATTGGTTGAACTTCTTG	59.9	177	86	OsFtsH4 FtsH protease homologue of AtFtsH4 expressed
OsISM05143	LOC_Os01g39250	22076163	TGTTGATGAAGCCAAAGCTG	GGCAGTTTTCCACCAAGATG	60.2	804	85	OsFtsH4 FtsH protease homologue of AtFtsH4 expressed

ISM IDs	Rice gene locus IDs	Physical positions (bp) on chromosomes	Forward primers (5'-3')	Reverse primers (5'-3')	Melting tempearature (T˚C)	Expected product size (bp) in PCR	Expected product size (bp) in Q-PCR	Putative Functions
OsISM05144	LOC_Os01g39250	22077361	CCGAATCCTGATGTTGAAGG	GGCAATGATCATCAAATCCA	59.9	178	91	OsFtsH4 FtsH protease homologue of AtFtsH4 expressed
OsISM05145	LOC_Os01g39260	22081469	CGGCGGACTAATAAGGGATT	ATATGCGGATCGGTTCCTT	59.8	669	76	OsFtsH5 FtsH protease homologue of AtFtsH4 expressed
OsISM05146	LOC_Os01g39260	22082163	CCAACCCATCGAGGATTCTT	GCGTGCAAAGCTACCAACAT	61.2	413	99	OsFtsH5 FtsH protease homologue of AtFtsH4 expressed
OsISM05147	LOC_Os01g39260	22082737	GTTGGAGGATAGCACCTTGC	TTTTCTTCCTCCCTTGCTGA	59.8	832	67	OsFtsH5 FtsH protease homologue of AtFtsH4 expressed
OsISM05148	LOC_Os01g39260	22083731	AGCGCTAACATTCCTCCTGA	CCATGCTTGGTTGAACCTCT	60.0	186	99	OsFtsH5 FtsH protease homologue of AtFtsH4 expressed
OsISM05149	LOC_Os01g39260	22083957	AGCCAAAGCTGAACTTGAGG	GACCAACCAGCAAGACACCT	59.9	1315	99	OsFtsH5 FtsH protease homologue of AtFtsH4 expressed
OsISM05150	LOC_Os01g39260	22085642	TCCAAATCCAGATGTTGAAGG	GGCAATGATCATCAAATCCA	59.6	179	92	OsFtsH5 FtsH protease homologue of AtFtsH4 expressed
OsISM05151	LOC_Os01g39310	22120538	GTTAAAATGGGCGAATGTGG	GGTGCGGAAAAGGTGATTTA	60.1	208	97	SEY1 putative expressed
OsISM05152	LOC_Os01g39310	22120726	TAAATCACCTTTTCCGCACC	TCAATATTATGGGCCTTCGC	59.9	221	98	SEY1 putative expressed
OsISM05153	LOC_Os01g39310	22120961	TAATGGATTTGGAAGGCACG	TGAAACAGCCAAAGCAAACA	60.6	1089	90	SEY1 putative expressed
OsISM05154	LOC_Os01g39310	22122030	TGTTTGCTTTGGCTGTTTCA	TGTTCGCAGCCTGTTCTCTA	60.1	167	76	SEY1 putative expressed
OsISM05155	LOC_Os01g39310	22122177	TAGAGAACAGGCTGCGAACA	TCGTTTTACGTGGACTGAACA	59.5	183	78	SEY1 putative expressed
OsISM05156	LOC_Os01g39310	22122341	TTCAGTCCACGTAAAACGACA	TCGGTTCAAGATTTTCCAGAG	59.3	202	74	SEY1 putative expressed
OsISM05157	LOC_Os01g39310	22122493	ATGTGCCCATTTCGGTTTAC	TGGGGTTTAGGGACACCAT	59.9	183	97	SEY1 putative expressed
OsISM05158	LOC_Os01g39310	22122678	CCCACAAGGAAACTCCACTG	TGCCAACCTGCTCTTTGAAT	60.7	176	97	SEY1 putative expressed
OsISM05159	LOC_Os01g39310	22122790	GTCGAAGTTGTGGCCCTATC	GGAGCAATAGACTGTTGGAACC	59.8	186	96	SEY1 putative expressed
OsISM05160	LOC_Os01g39310	22123034	TCACAACAATTTTGGAAGGTCA	CGATTTCCTCACAGCGTACA	60.1	169	92	SEY1 putative expressed
OsISM05161	LOC_Os01g39310	22123186	ACGCTGTGAGGAAATCGGTA	CCAGGCACGTAATCATGTTG	60.3	183	97	SEY1 putative expressed

ISM IDs	Rice gene locus IDs	Physical positions (bp) on chromosomes	Forward primers (5'-3')	Reverse primers (5'-3')	Melting tempearature (T°C)	Expected product size (bp) in PCR	Expected product size (bp) in Q-PCR	Putative Functions
OsISM05162	LOC_Os01g39310	22123372	TGGGAAGAAGATCAGCAACC	TGGTGCCTTTTCGAAGTTCT	60.0	659	97	SEY1 putative expressed
OsISM05163	LOC_Os01g39310	22124011	AGAACTTCGAAAAGGCACCA	TTTTGGTATGCAGGGTTGAC	59.2	613	63	SEY1 putative expressed
OsISM05164	LOC_Os01g39310	22124704	CTGTTGCTGCTCGTGACTGT	GGTCCCATTTCACTTGTTGG	60.2	695	88	SEY1 putative expressed
OsISM05165	LOC_Os01g39310	22125440	CATGTGGCATCAGTTCGTG	GAGCTTCAACTGGTTCTGCC	59.8	178	92	SEY1 putative expressed
OsISM05166	LOC_Os01g39310	22125798	CAAAAGAGGAAGCTGCAAGG	TCAGCATCACGGCTGAATAG	60.0	144	71	SEY1 putative expressed
OsISM05167	LOC_Os01g39310	22125943	TCAATGCCAAGAGTGTGGAC	CGAATTGCAGCCATTGTAGA	59.8	191	99	SEY1 putative expressed
OsISM05168	LOC_Os01g39310	22126237	CTTGCCTCTAGCTCATGGGA	TTCAGCCTTAAATTGCCTCC	59.9	228	91	SEY1 putative expressed
OsISM05169	LOC_Os01g39310	22126444	TGGAGGCAATTTAAGGCTGA	GCAGCCAGTTGTTGTTCCTC	60.8	214	86	SEY1 putative expressed
OsISM05170	LOC_Os01g39310	22126678	GCAATGGCTATCCTTGGATTC	ACATGGCTTTTCCAACAAGG	60.4	186	98	SEY1 putative expressed
OsISM05171	LOC_Os01g39310	22126853	AAAAGCCATGTGGGTACAGC	TCGAAAGTGATAGGACGGCT	59.9	171	75	SEY1 putative expressed
OsISM05172	LOC_Os01g39330	22137198	CTCCACGACCCCTCCTTCT	CATGAGCTCGTCGAAGCA	60.7	189	94	helix-loop-helix DNA-binding domain containing protein expressed
OsISM05173	LOC_Os01g39330	22138057	TCCACACGCTCAGATCACTC	AGCGACTTGAGGTACTTGGC	59.7	2213	86	helix-loop-helix DNA-binding domain containing protein expressed
OsISM05174	LOC_Os01g39330	22140577	GCCAACATCAAGCTCCAGAT	GACGCCTCGTTCCAATCTC	60.5	191	45	helix-loop-helix DNA-binding domain containing protein expressed
OsISM05176	LOC_Os01g39360	22151215	GCTACTCACACTTGCTGCCA	GATTGAGGGATCCGACTTCA	60.1	175	87	retrotransposon protein putative unclassified
OsISM05177	LOC_Os01g39360	22151625	CGCCAAAGATCAGGAAGTCT	CAGAGTCTTTCCCAGACGGA	59.9	139	61	retrotransposon protein putative unclassified
OsISM05178	LOC_Os01g39360	22152046	CATCCGAGTCCACTCATCCT	TGTATGCGGGGAATTTCTTC	60.0	157	89	retrotransposon protein putative unclassified
OsISM05179	LOC_Os01g39360	22152274	TGATAAACCGGCAAAAAGATG	CGCCTCGTTCCTAAGATGAA	60.1	175	99	retrotransposon protein putative unclassified
OsISM05180	LOC_Os01g39360	22152574	GCTCCAGGAGTCCAATTTCA	TCTTGGCAGCTTGCACTACA	60.5	127	63	retrotransposon protein putative unclassified

ISM IDs	Rice gene locus IDs	Physical positions (bp) on chromosomes	Forward primers (5'-3')	Reverse primers (5'-3')	Melting tempearature (T˚C)	Expected product size (bp) in PCR	Expected product size (bp) in Q-PCR	Putative Functions
OsISM05181	LOC_Os01g39360	22152754	GCAAGACAAAGAGACCTGGC	AACCAGGTCGTCGAATTGAG	60.1	151	76	retrotransposon protein putative unclassified
OsISM05182	LOC_Os01g39430	22216767	TCTGCATGACCTATGCGTTC	TTGCAAAGCTTTTACCTGGC	60.1	879	55	anthocyanin regulatory protein putative
OsISM05183	LOC_Os01g39430	22217684	GATAGCAAAGCCTTTCCACG	ATGAAGGGGACGCAGACAAT	60.8	500	56	anthocyanin regulatory protein putative
OsISM05184	LOC_Os01g39464	22227438	GAACGAGAGCGAGAACGAAC	GTTATGATGTGCCGACCCTC	60.2	1343	84	transposon protein putative CACTA En/Spm sub-class
OsISM05185	LOC_Os01g39464	22229880	CCATGTGAGCTGCATATTCC	AGTACCTGAAGGGTCCGTTG	59.1	158	82	transposon protein putative CACTA En/Spm sub-class
OsISM05186	LOC_Os01g39464	22230895	GCAGTCCCTACCCACACAAA	TCCAGTATCGAAAACCCTCC	60.0	358	83	transposon protein putative CACTA En/Spm sub-class
OsISM05187	LOC_Os01g39464	22231334	AAGGCGCAGCATTACAAGAC	GCCGACAGGTCGAAGAGTAA	60.4	179	81	transposon protein putative CACTA En/Spm sub-class
OsISM05188	LOC_Os01g39464	22231493	TTACTCTTCGACCTGTCGGC	AGTTCCCTGCTTTTGCTTTG	59.9	381	97	transposon protein putative CACTA En/Spm sub-class
OsISM05189	LOC_Os01g39464	22231908	ACTGCCTTGCAGACCAAATC	CGCTGCGATAAATTCCTTGT	60.2	171	90	transposon protein putative CACTA En/Spm sub-class
OsISM05191	LOC_Os01g39480	22236434	GCTGGGAGAAGCTGAATGAG	GCTATCGTCTCAGCAAGGCT	59.9	225	95	anthocyanin regulatory Lc protein putative
OsISM05193	LOC_Os01g39520	22270807	GCAACCCACAATTCAAACCT	GAATTTCTTCTTCCTCCGGC	60.0	748	69	retrotransposon putative centromere-specific
OsISM05194	LOC_Os01g39530	22272888	TTATCACCAAATCCCCATCC	CATCATCAAAGTCAAACGAAAC A	59.7	526	63	retrotransposon putative centromere-specific
OsISM05195	LOC_Os01g39550	22298349	TGCCGGCTATCCTTATGAAC	CCGGAGTTTCTTAATTTCCTGA	59.8	130	81	retrotransposon protein putative Ty1-copia subclass
OsISM05196	LOC_Os01g39550	22298568	CGAAGGAGATGGGTTTGAGA	GACTGCGACCAACAGTCAAG	59.8	288	73	retrotransposon protein putative Ty1-copia subclass
OsISM05197	LOC_Os01g39560	22300292	TGCCATCTTTTGGTCCATTT	CCGTTGTAGAACCCGTCATT	60.1	2431	67	anthocyanin regulatory protein putative
OsISM05198	LOC_Os01g39560	22302890	ATCTCGGCAACGTAGAATGG	TTGCAAAGCTTTTACCTGGC	60.2	2398	85	anthocyanin regulatory protein putative
OsISM05199	LOC_Os01g39560	22305311	AACGCTCAGTCTGCCGATAG	TGACGAAGCATGCATGTAGA	59.8	214	96	anthocyanin regulatory protein putative
OsISM05200	LOC_Os01g39560	22305434	TGCATACTCTCTCTTTTGACCA	AGCTCAAGAACGCCAGTCAT	58.8	227	98	anthocyanin regulatory protein putative

ISM IDs	Rice gene locus IDs	Physical positions (bp) on chromosomes	Forward primers (5'-3')	Reverse primers (5'-3')	Melting tempearature (T˚C)	Expected product size (bp) in PCR	Expected product size (bp) in Q-PCR	Putative Functions
OsISM05201	LOC_Os01g39560	22305639	TCATGACTGGCGTTCTTGAG	AGGATAGGGCTCATCCTCCC	60.8	740	89	anthocyanin regulatory protein putative
OsISM05202	LOC_Os01g39560	22306370	CCCTATCCTCCGCCAGAT	ATGAAGCGAGGGAAACACAT	59.8	218	86	anthocyanin regulatory protein putative
OsISM05203	LOC_Os01g39580	22321036	CCCACTAGTTGGGGTTTTGTT	TTACCACGTTTGGTTCCTCC	60.0	2897	62	anthocyanin regulatory Lc protein putative
OsISM05204	LOC_Os01g39580	22324475	CTCAAATCACTGGTCCCGTC	CCTCTCCAGCTCCTTGAGGT	60.7	222	85	anthocyanin regulatory Lc protein putative
OsISM05205	LOC_Os01g39580	22324999	GGATGGTCTTCTCGGACTGA	GCTGTCCGGAGAGTTTCACT	59.8	1312	88	anthocyanin regulatory Lc protein putative
OsISM05206	LOC_Os01g39640	22360139	CTTTTTACTGCCAGCGCTTC	CTATTTTGGCATGGATGCAG	59.6	138	95	retrotransposon protein putative unclassified
OsISM05207	LOC_Os01g39640	22360524	GGGGATCACCTTGAAGGAAG	CTTAATCCTCCACCGTCTGC	60.3	533	70	retrotransposon protein putative unclassified
OsISM05208	LOC_Os01g39670	22375417	TTTGGTGATCAACGAAACCA	CACGCTGGGGATAAGTGTCT	60.0	156	68	OsFBD1 - F-box and FBD domain containing protein expressed
OsISM05209	LOC_Os01g39670	22375606	CGAAAGGAGGCTTTGATGTC	TCTGAAGCCTTGTCCGTTTT	59.8	308	90	OsFBD1 - F-box and FBD domain containing protein expressed
OsISM05210	LOC_Os01g39680	22378322	GTGTGAATCCCAGGAGCAAC	CCCGTAGGGAAATAAGCACA	60.2	152	66	expressed protein
OsISM05211	LOC_Os01g39680	22378489	TCATCAAGATCGGTCGACAA	GCTCTAATGGCATTGCAGAA	59.6	978	86	expressed protein
OsISM05212	LOC_Os01g39680	22379468	CCCGAAGCGTTCAAATTAGT	TCTTCAACAGATGTGGTTTCAC	58.5	151	56	expressed protein
OsISM05213	LOC_Os01g39680	22379640	TGTTTCTACAATTTAAGGCTGCAC	AGTGGGCAATTGGTGTACTTG	59.8	183	98	expressed protein
OsISM05214	LOC_Os01g39680	22379806	TACACCAATTGCCCACTGTC	GTCGTTTGACACAGCACCAG	59.9	1639	83	expressed protein
OsISM05215	LOC_Os01g39700	22390425	TCTTCTTCCAGTGCTCTCCG	CGAGGAACCGATGATAGGAG	60.2	790	74	hypothetical protein
OsISM05216	LOC_Os01g39720	22401048	TCTCGAGATGCTGACAAGGA	TCTCCATCTCGTTGTTCCCT	59.7	419	93	retrotransposon protein putative unclassified
OsISM05217	LOC_Os01g39720	22401765	GACAACAGAGATCGACACCG	GTGGCGCTTGTATGTTCCTT	59.7	477	94	retrotransposon protein putative unclassified
OsISM05218	LOC_Os01g39720	22402387	ATCGCTACACAACCCAGGAG	CGATCTTCTGCTCTGACCAA	59.6	582	91	retrotransposon protein putative unclassified

ISM IDs	Rice gene locus IDs	Physical positions (bp) on chromosomes	Forward primers (5'-3')	Reverse primers (5'-3')	Melting tempearature (T°C)	Expected product size (bp) in PCR	Expected product size (bp) in Q-PCR	Putative Functions
OsISM05219	LOC_Os01g39730	22404387	GGCGATCGAGAACATGAAGT	CTGTGACTCTGTGGGCTTGA	60.1	443	92	retrotransposon protein putative unclassified
OsISM05220	LOC_Os01g39730	22405440	AGACGTCCTGACAAAGCCAT	TGGCAATTTTGTGTGTTTGC	60.1	470	74	retrotransposon protein putative unclassified
OsISM05221	LOC_Os01g39750	22411473	CGCAAGCCAAGTTCCAATAC	ATCGGCATCCATAATGTTCC	60.1	221	92	hypothetical protein
OsISM05222	LOC_Os01g39750	22411768	TTGCAAAAATTAAAGCTCCCA	CAGTTGCTCAAATCCTCGTTC	59.8	393	47	hypothetical protein
OsISM05224	LOC_Os01g39770	22427205	CTGCACCTCCCTCACCCT	TCTTGTAGAGCTCGAACAGCG	61.1	222	96	calcineurin B putative expressed
OsISM05225	LOC_Os01g39770	22427441	ATCATCGACGACGGACTCAT	CAAGAAAAAGATTCTGGCCG	60.2	837	80	calcineurin B putative expressed
OsISM05226	LOC_Os01g39770	22428258	CGGCCAGAATCTTTTTCTTG	GGAAGACGCTAAGAGCATGA	59.0	273	99	calcineurin B putative expressed
OsISM05227	LOC_Os01g39770	22428515	GCTCTTAGCGTCTTCCATCC	TCCTCACGCTCAATAAACCC	59.6	187	99	calcineurin B putative expressed
OsISM05228	LOC_Os01g39770	22428678	AACTGGGTTTATTGAGCGTGA	CCGACAAAATAGCGATAACCA	59.8	151	54	calcineurin B putative expressed
OsISM05229	LOC_Os01g39770	22428848	AGAAGAACTTCTCGAGGCCA	CATCCCTATCAGCATCAGCA	59.5	153	60	calcineurin B putative expressed
OsISM05230	LOC_Os01g39770	22429032	CGTATTGCGGCATCCTAATC	CGCGGTGTTGAAGACAAAA	60.6	277	92	calcineurin B putative expressed
OsISM05231	LOC_Os01g39780	22435114	CATCTTCCCGTTCCTCCTC	GAGCGTTCTTCTTTTCCTGG	59.3	1762	90	expressed protein
OsISM05232	LOC_Os01g39810	22449834	ACCTACTTCAACCCCGACG	TCCACTCCCAAGTAAGGTGG	60.0	208	86	Alg9-like mannosyltransferase protein putative expressed
OsISM05233	LOC_Os01g39810	22450091	CAAGATTCTTTCGCTGCTCC	GAGATCCCCAAAAGATGCAA	60.1	497	92	Alg9-like mannosyltransferase protein putative expressed
OsISM05234	LOC_Os01g39810	22450615	TTCAATAATCACGTTGCCCA	CCATGCTGTTTGAGAGAGTCC	59.9	192	92	Alg9-like mannosyltransferase protein putative expressed
OsISM05235	LOC_Os01g39810	22451015	GATGAAATCAAAATCCCGGTT	AATCAAGAAACGTTGTCACGG	60.0	773	81	Alg9-like mannosyltransferase protein putative expressed
OsISM05236	LOC_Os01g39810	22451967	TGGAGGCTTGCAGGTCTTAT	ATAAAAGGAAGCACCGGGAG	60.1	188	93	Alg9-like mannosyltransferase protein putative expressed
OsISM05237	LOC_Os01g39810	22452263	CCTAACAAATGTTCCAATGGC	TCTTCCATCATGGGCTTCTC	59.7	242	98	Alg9-like mannosyltransferase protein putative expressed

ISM IDs	Rice gene locus IDs	Physical positions (bp) on chromosomes	Forward primers (5'-3')	Reverse primers (5'-3')	Melting tempearature (T˚C)	Expected product size (bp) in PCR	Expected product size (bp) in Q-PCR	Putative Functions
OsISM05238	LOC_Os01g39810	22452570	CCTACCTATGCGCTTTTTGG	TCTGACTCATCCAAGGTCCC	59.9	167	61	Alg9-like mannosyltransferase protein putative expressed
OsISM05239	LOC_Os01g39810	22452843	CTTCTGCGCAATTCATTTCA	CAACCTTGAAGTGGGAGTGA	59.3	862	56	Alg9-like mannosyltransferase protein putative expressed
OsISM05240	LOC_Os01g39830	22465806	ATCCACTACCCGAGGAGCAC	CCGTCCTTGGCCTTCTCTAT	61.0	227	60	beta-galactosidase putative expressed
OsISM05241	LOC_Os01g39830	22466052	ACGTACGTCTTCTGGAACGG	GCTTGATGAAGTGCACCAGA	60.1	170	83	beta-galactosidase putative expressed
OsISM05242	LOC_Os01g39830	22466239	CTCTACGTGAACCTCCGCAT	GGGGACATACTTGAGCCAAA	60.1	389	82	beta-galactosidase putative expressed
OsISM05243	LOC_Os01g39830	22466608	TTTGGCTCAAGTATGTCCCC	TCGACGATCTTTGTGGTGAA	60.1	211	89	beta-galactosidase putative expressed
OsISM05244	LOC_Os01g39830	22466848	GGCAAGGAGGGCCTATAATC	CACCTTGATCCCACTCCAAG	60.2	155	67	beta-galactosidase putative expressed
OsISM05245	LOC_Os01g39830	22467075	TGTGCAAAGAAGATGATGCC	TGGGGAGAACCAATCACAGT	60.1	320	72	beta-galactosidase putative expressed
OsISM05246	LOC_Os01g39830	22467416	ATGTGGACTGAAGCCTGGAC	TGGCGACACCATATGCTAGA	60.2	181	89	beta-galactosidase putative expressed
OsISM05247	LOC_Os01g39830	22467602	ATCCAGAAGGGTGGCTCTTT	ACTAGTGGCAATGAATGGGC	60.0	174	91	beta-galactosidase putative expressed
OsISM05248	LOC_Os01g39830	22467731	ACAAACTTTGGACGCACAGC	CCCCATTTTGGTTCTCTCAA	60.6	724	99	beta-galactosidase putative expressed
OsISM05249	LOC_Os01g39830	22468507	GCAGGAGACCCCATTGTAAC	AAGGAAAGCTGCACAAGCTC	59.6	171	85	beta-galactosidase putative expressed
OsISM05250	LOC_Os01g39830	22468737	GCCTCCGTGGTCCATAAGTA	CAGCCCATTCCATTTTCATC	60.1	383	99	beta-galactosidase putative expressed
OsISM05251	LOC_Os01g39830	22469208	TGTGACGAGGGATAACACAGA	TTGAGATTCTCCCCATTGCT	59.4	176	91	beta-galactosidase putative expressed
OsISM05252	LOC_Os01g39830	22469395	TGTCAGCTGGTCATGCTTTG	GGGTCATCTACGCTGCCATA	61.0	160	74	beta-galactosidase putative expressed
OsISM05253	LOC_Os01g39830	22469588	GGCAGGCAGCAATACAATTT	GAATCCCAGCATTCCAAGTC	59.8	183	87	beta-galactosidase putative expressed
OsISM05254	LOC_Os01g39830	22469816	TGACATGGCAGAAATGGACT	CCCACTCCACTGTTGAGCTT	59.7	175	88	beta-galactosidase putative expressed
OsISM05255	LOC_Os01g39830	22469998	TGTGCAGAAGCAGCCTCTAA	GCCCCTTCCCCATACTACTC	59.8	180	96	beta-galactosidase putative expressed

ISM IDs	Rice gene locus IDs	Physical positions (bp) on chromosomes	Forward primers (5'-3')	Reverse primers (5'-3')	Melting tempearature (T°C)	Expected product size (bp) in PCR	Expected product size (bp) in Q-PCR	Putative Functions
OsISM05256	LOC_Os01g39830	22470282	TGTCAAACCAACTGTGGTGA	GGACTAAGCCATGAACGAGG	59.1	156	63	beta-galactosidase putative expressed
OsISM05257	LOC_Os01g39830	22470672	AAGCTACACCGAAGGAGGCT	TTCTGGAACAACGCTTACCC	60.3	201	98	beta-galactosidase putative expressed
OsISM05258	LOC_Os01g39860	22488655	CCTGTAAGGACTGGGGCTTC	TTCACCTCCTCCATTAACGC	60.5	359	68	1-aminocyclopropane-1-carboxylate oxidase protein putative expressed
OsISM05260	LOC_Os01g39940	22526026	AACCAGATATGGGTGTTGCAC	AGGGGCGTCAAGGTCTATTC	60.1	1274	99	transposon protein putative unclassified
OsISM05261	LOC_Os01g39940	22527279	AGAATAGACCTTGACGCCCC	GTTGGCCAGTAATGGACGTT	60.2	587	93	transposon protein putative unclassified
OsISM05262	LOC_Os01g39960	22534351	CAGCCAGTCCAAGATCTCCT	GCCAATGATGACCAAATCAA	59.4	968	74	lycopene epsilon cyclase chloroplast precursor putative expressed
OsISM05263	LOC_Os01g39960	22535404	CGAACAACTACGGTGTGTGG	TGTTCAATGCAGCTCTCCAG	60.1	154	62	lycopene epsilon cyclase chloroplast precursor putative expressed
OsISM05264	LOC_Os01g39960	22535637	GACTTGCTGCACGAGGAGTT	TCGAGCTGAGGTATGTGACG	60.3	156	62	lycopene epsilon cyclase chloroplast precursor putative expressed
OsISM05265	LOC_Os01g39960	22535912	GAGTACGAGGTTGGTGGTCC	TGAAAACCATTAAGCTGGGA	58.8	268	98	lycopene epsilon cyclase chloroplast precursor putative expressed
OsISM05266	LOC_Os01g39960	22536235	AACGTTCCTCTATGCCATGC	TTTAAGGAGGTCAAAGGGCA	59.9	186	95	lycopene epsilon cyclase chloroplast precursor putative expressed
OsISM05267	LOC_Os01g39960	22536418	AAAAAGCGGTTGATGTCTCG	TGGTAAGGACCCTCCAACAG	60.1	187	97	lycopene epsilon cyclase chloroplast precursor putative expressed
OsISM05268	LOC_Os01g39960	22536617	AAAAATCTCGCATTTGGTGC	CAGACAATGATCTAACCACCGA	60.0	159	74	lycopene epsilon cyclase chloroplast precursor putative expressed
OsISM05269	LOC_Os01g39960	22536815	GAAACCGTGTCTACCCTGGA	TTCTTGGGGCCATAATGTTC	59.9	244	90	lycopene epsilon cyclase chloroplast precursor putative expressed
OsISM05270	LOC_Os01g39960	22537138	TTGAAACCTTTTTCCGGTTG	GAAGAAAGCGTCGAACCAAG	60.0	182	62	lycopene epsilon cyclase chloroplast precursor putative expressed
OsISM05271	LOC_Os01g39970	22538407	CCAACATCATCCTCCTCCAC	CTCATCTCGGCCTCCAAC	59.8	204	98	protein kinase domain containing protein putative expressed
OsISM05272	LOC_Os01g39970	22538610	GGGAGATCTTTGTCCCCTTC	ACACGTCTGCCTCTTCCAGT	59.9	204	82	protein kinase domain containing protein putative expressed
OsISM05273	LOC_Os01g39970	22538839	CGTGAACAAGATCCAGAGCA	CTTCATCAGGCTGGACGG	60.2	211	95	protein kinase domain containing protein putative expressed
OsISM05274	LOC_Os01g39970	22539525	AGAGCAGCTCGCCTCTCTC	CATGTCCATCGTCTGCTTCA	60.5	160	87	protein kinase domain containing protein putative expressed

ISM IDs	Rice gene locus IDs	Physical positions (bp) on chromosomes	Forward primers (5'-3')	Reverse primers (5'-3')	Melting tempearature (T˚C)	Expected product size (bp) in PCR	Expected product size (bp) in Q-PCR	Putative Functions
OsISM05275	LOC_Os01g39980	22542890	GTCAGTGATGTCGCGAAGAG	CTCTACACCGCGGTCTTCTT	59.5	1449	98	hypothetical protein
OsISM05277	LOC_Os01g40094	22617531	CAGCACACTTCTTCGGTGTC	CCGCTCCCGACAGTAATTT	59.8	197	66	protein phosphatase 2C putative expressed
OsISM05278	LOC_Os01g40094	22617979	AGTGCTCTGTCGTGGCAAG	TGCCTCGATCCTTGCATATT	60.4	193	83	protein phosphatase 2C putative expressed
OsISM05279	LOC_Os01g40094	22618210	TTTTGGTGTTCTTGCCATGT	TCCTTTGCTCGAGGAACAAT	59.4	4196	97	protein phosphatase 2C putative expressed
OsISM05280	LOC_Os01g40130	22638375	GGTCTCGCCAACAGCATT	CGACACTTGTCATCAGGGTG	60.0	697	96	retrotransposon protein putative unclassified
OsISM05281	LOC_Os01g40150	22644997	TGAGGAGATTAAAGGGGCTG	CCTCCTCATTGGAGGATTTC	58.9	194	99	eukaryotic translation initiation factor 5B putative expressed
OsISM05282	LOC_Os01g40150	22645640	GCACAAACAATTGAGTCTTTG AA	GGACATCTTTTCCAGCCAAA	59.7	198	99	eukaryotic translation initiation factor 5B putative expressed
OsISM05283	LOC_Os01g40150	22645866	CAGCAAGCTGAAGGTGTGAA	ACAAAATAGTATTGATGCCTTGC	58.8	178	77	eukaryotic translation initiation factor 5B putative expressed
OsISM05284	LOC_Os01g40150	22646078	GAACATTGTACCTACCTGTGC AA	GCCGCTCTTTCATCTTCTTTT	59.5	198	93	eukaryotic translation initiation factor 5B putative expressed
OsISM05285	LOC_Os01g40150	22646249	GGGCCCAAAAGAAGATGAA	TGTAGTGCAGTGGCCTTCAG	60.0	223	93	eukaryotic translation initiation factor 5B putative expressed
OsISM05286	LOC_Os01g40150	22646498	CCTCCGTGAAGGAGATCAAA	GATGAGGTGTTAACAGAGCCC	59.4	174	81	eukaryotic translation initiation factor 5B putative expressed
OsISM05287	LOC_Os01g40150	22646649	CAGGGCTCTGTTAACACCTCA	TGCCTTGAGAAGCTTTGATCT	59.8	1097	87	eukaryotic translation initiation factor 5B putative expressed
OsISM05288	LOC_Os01g40150	22647729	CAAAGCTTCTCAAGGCATCA	TCATCACCTGGCTGCACTAC	59.5	162	88	eukaryotic translation initiation factor 5B putative expressed
OsISM05289	LOC_Os01g40150	22647914	CCATGGCAGAGATGGTTGTT	GGATATTGACAGCTGGGCTC	60.3	129	88	eukaryotic translation initiation factor 5B putative expressed
OsISM05290	LOC_Os01g40150	22648350	CCCAATTGTCTTAGGCGTTG	CCAATACGAGCGAAACCATT	60.2	325	88	eukaryotic translation initiation factor 5B putative expressed
OsISM05291	LOC_Os01g40150	22648672	TGGGAACATTGCCTCTATCC	GCTCATCTGGAGTGCTTCCT	59.7	181	99	eukaryotic translation initiation factor 5B putative expressed
OsISM05292	LOC_Os01g40150	22648878	TGGAATTGACGATGAACTGG	AAGCTCCCACTCTTTCAACG	59.5	187	98	eukaryotic translation initiation factor 5B putative expressed
OsISM05293	LOC_Os01g40170	22656531	GCTCACCAGTCTGCTGCTTT	TCCTGAAGGTCTCATGACCA	60.0	238	73	translation initiation factor putative

ISM IDs	Rice gene locus IDs	Physical positions (bp) on chromosomes	Forward primers (5'-3')	Reverse primers (5'-3')	Melting tempearature (T˚C)	Expected product size (bp) in PCR	Expected product size (bp) in Q-PCR	Putative Functions
OsISM05294	LOC_Os01g40170	22656841	TGCAGACATTAGAGTCATTGGA	TGGATATTGTTTCCAGCCATC	59.1	209	99	translation initiation factor putative
OsISM05295	LOC_Os01g40170	22657056	TCTTTCGAGAAGGCTCTTGC	TGAGCATTGAGTTGGCTCAC	59.9	176	90	translation initiation factor putative
OsISM05296	LOC_Os01g40170	22657289	CATTGTGCCTACCAGTTCAA	CCGTATGGTAAGCCGTTCAT	58.5	202	98	translation initiation factor putative
OsISM05297	LOC_Os01g40170	22657477	GGCTTACCATACGGGACAAA	TGGTAGTGCCATGACCTTCA	60.0	174	70	translation initiation factor putative
OsISM05298	LOC_Os01g40170	22657658	TGTGTTGTCTAGTGGTGTTCTTCA	GGGTGTTAACAAAGCCCTGA	59.9	181	95	translation initiation factor putative
OsISM05299	LOC_Os01g40170	22657819	TCAGGGCTTTGTTAACACCC	TTATGCCTCGAGCACACTTG	60.0	1812	91	translation initiation factor putative
OsISM05300	LOC_Os01g40170	22659614	GTGTGCTCGAGGCATAACAA	TCACCAGGCCGTACAACATA	59.9	153	82	translation initiation factor putative
OsISM05301	LOC_Os01g40170	22660227	GGTGTCGACATGCTGTATGG	TGGATTGAAGCAATTTTCCC	59.9	329	93	translation initiation factor putative
OsISM05302	LOC_Os01g40170	22660535	TGGGAAAATTGCTTCAATCC	TCGTCTTGGTTGCTCCCTAC	60.1	178	97	translation initiation factor putative
OsISM05303	LOC_Os01g40170	22660770	AACAAAGGAGTCGATTGATGTTC	GTCAGCCTCAGGTAACGAGC	59.8	1203	83	translation initiation factor putative
OsISM05304	LOC_Os01g40170	22661951	AAGCTCGTTACCTGAGGCTG	TTGGAACTCTTCAGACGCAA	59.6	172	71	translation initiation factor putative
OsISM05305	LOC_Os01g40170	22662124	TTGAGACCCAGTTTTGTTCTCTC	TGGTTTCATCTTCAACTCTTCATC	59.7	1349	99	translation initiation factor putative
OsISM05306	LOC_Os01g40210	22692109	GCATGATGGGAGTGTCTGC	GTGTCGGAGACACGGTTCTT	60.2	511	77	hypothetical protein
OsISM05307	LOC_Os01g40220	22694265	CGCCCTTCTTGTCCTAGTTG	AGTGGAAGTCCACGCAGC	59.9	229	78	DEFL7 - Defensin and Defensin-like DEFL family
OsISM05308	LOC_Os01g40230	22699699	GTCCATCTGGACAGGCAAGT	GTGGTTCCTGTCAATCCCAC	60.2	462	75	transposon protein putative unclassified
OsISM05310	LOC_Os01g40230	22700672	CTAGCAAGGCTTGGTTGCTC	AACCACAACAGGTACGCCTC	60.1	184	98	transposon protein putative unclassified
OsISM05311	LOC_Os01g40230	22701166	ACGATGGGGACTTACTGGTG	GGAGACGGTCAAACTCGAAC	59.8	187	95	transposon protein putative unclassified
OsISM05312	LOC_Os01g40230	22701525	CGGACTTAGGCAGGTGTTTC	CTAGCTGTCCCCGTCGAGT	60.1	159	81	transposon protein putative unclassified

ISM IDs	Rice gene locus IDs	Physical positions (bp) on chromosomes	Forward primers (5'-3')	Reverse primers (5'-3')	Melting tempearature (T˚C)	Expected product size (bp) in PCR	Expected product size (bp) in Q-PCR	Putative Functions
OsISM05313	LOC_Os01g40230	22701892	GCACCGTGACCGAGACTACT	ATATCGGCACTGATATCCCG	60.0	152	58	transposon protein putative unclassified
OsISM05315	LOC_Os01g40230	22702600	AGCTCTTCCTCTCACGGAGC	AGACGAAGAGGCCCCAGTA	60.5	144	82	transposon protein putative unclassified
OsISM05316	LOC_Os01g40230	22702919	ACCGACTCACGTACTCCCAC	GCAAGGGCGATTTTGTACTG	60.3	873	76	transposon protein putative unclassified
OsISM05317	LOC_Os01g40240	22705939	CTACTCGTTCACCGACATGG	ATCGCATTGAAGCTAAACCG	59.7	462	52	conserved hypothetical protein
OsISM05318	LOC_Os01g40250	22708566	GTCAAGTGCCTCAACACGTC	ACGTCGATGTCGATCTCCTC	59.8	226	87	down-regulated in metastasis family protein expressed
OsISM05319	LOC_Os01g40250	22708840	GTTCTTCCTCGACGCCCT	GAGGCAATGTTTGTACCAAATGTA	60.3	2504	99	down-regulated in metastasis family protein expressed
OsISM05320	LOC_Os01g40250	22711400	AAGGCGAGATTGTCACTTGAA	TAGCAAACTCACGGCAAAAA	59.7	664	87	down-regulated in metastasis family protein expressed
OsISM05321	LOC_Os01g40250	22712023	TCCAGGGATATTCTGGAGGA	TTCAGGGTCTCGATCACCTC	59.8	256	94	down-regulated in metastasis family protein expressed
OsISM05322	LOC_Os01g40250	22712259	GAGGTGATCGAGACCCTGAA	TCATTACATATGACCATGACGTG	59.3	136	58	down-regulated in metastasis family protein expressed
OsISM05323	LOC_Os01g40250	22712364	CAGGTCTTCACGTCATGGTC	AAAAATCTCAGTGGAGCTGTG	58.1	208	99	down-regulated in metastasis family protein expressed
OsISM05324	LOC_Os01g40250	22712619	TTCTGAGGAATGCACCCAAT	CGGGTGATGGATTTTTAGCA	60.6	1387	79	down-regulated in metastasis family protein expressed
OsISM05325	LOC_Os01g40250	22714099	TGTTGAGCAAGTCAAACCTCA	TGACCAGCGATTTAACAAGTTT	59.1	667	85	down-regulated in metastasis family protein expressed
OsISM05326	LOC_Os01g40250	22714718	TTTTTCCAGATAAGGCTAACATCC	TGAATACTGGATCATAGAAGGGTG	59.6	198	97	down-regulated in metastasis family protein expressed
OsISM05327	LOC_Os01g40250	22714892	CACCCTTCTATGATCCAGTATTCA	AATTTTTGGACCCTTTGTCA	58.2	206	84	down-regulated in metastasis family protein expressed
OsISM05328	LOC_Os01g40250	22715076	ATTGACAAAGGGTCCAAAAA	TCACATTCTCCTGTCATTCCC	58.5	915	82	down-regulated in metastasis family protein expressed
OsISM05329	LOC_Os01g40250	22716123	AGAGCGTCCACATAAGAGGC	ACAGCCAGAAGTGTGTCCAA	59.4	170	91	down-regulated in metastasis family protein expressed
OsISM05330	LOC_Os01g40250	22716577	TGGAAGCTACAATCCAACCA	CTGTCTCCAGAGGTGTGCAA	59.6	861	85	down-regulated in metastasis family protein expressed
OsISM05331	LOC_Os01g40250	22717510	CATTATTTCTAACCTTCATGGGC	ATCGTCTTCCATTGTTTGCC	59.4	196	95	down-regulated in metastasis family protein expressed

ISM IDs	Rice gene locus IDs	Physical positions (bp) on chromosomes	Forward primers (5'-3')	Reverse primers (5'-3')	Melting tempearature (T°C)	Expected product size (bp) in PCR	Expected product size (bp) in Q-PCR	Putative Functions
OsISM05332	LOC_Os01g40250	22717760	TCAGAGCAAGATTCTTCAGGA	TCCAGTTTAAAAGGCAATCCA	58.7	188	96	down-regulated in metastasis family protein expressed
OsISM05333	LOC_Os01g40250	22718080	TTGTTCCTTTGGTTATCCGTG	TTCAAGTCGAAGGTTCCCAG	60.0	298	96	down-regulated in metastasis family protein expressed
OsISM05334	LOC_Os01g40250	22718714	CTGCCCTCAGCAGACCATA	TGATGGGGGTTACCACAGAT	60.0	1383	95	down-regulated in metastasis family protein expressed
OsISM05335	LOC_Os01g40250	22720523	TGGATGTTCTTCTCCATAGTCTCA	AACAGCCTTAGTTCGCGCT	60.1	785	98	down-regulated in metastasis family protein expressed
OsISM05336	LOC_Os01g40250	22721364	TCATCCTTCCGTTTTTCAGC	TTTGCAAGTATTCCCCCAAC	60.0	199	83	down-regulated in metastasis family protein expressed
OsISM05337	LOC_Os01g40250	22721670	CATTTCACGAGCCTTCAGTG	ACTGCATTGAGATCTCGCAC	59.2	163	56	down-regulated in metastasis family protein expressed
OsISM05338	LOC_Os01g40250	22722151	GGTGGCAATGAGCAAAGATA	GGAGTTTAAGGATGGGACATGA	59.5	177	95	down-regulated in metastasis family protein expressed
OsISM05339	LOC_Os01g40250	22722343	AAGGAAGACCTGGAAGAGGA	ATCCGCTGCTTGAAAAGAGA	59.0	181	90	down-regulated in metastasis family protein expressed
OsISM05340	LOC_Os01g40250	22722514	AGCAGCGGATCAAAGATACC	ACCCTTCCCAGCTTTGACAT	60.1	456	99	down-regulated in metastasis family protein expressed
OsISM05341	LOC_Os01g40250	22724637	TGAGAAGCGTTTACAGCAACA	TTGTGGAGCATCTCAAGCAC	59.8	170	94	down-regulated in metastasis family protein expressed
OsISM05342	LOC_Os01g40250	22725012	CAGAATCTGTGGAGTGCTTCAG	TAAGGTGTTTGCCAATTCCC	59.9	271	77	down-regulated in metastasis family protein expressed
OsISM05343	LOC_Os01g40250	22725421	TTGCACGATTTCCAGAGTTG	ATTGAATGGGGATGGATCAG	59.7	538	86	down-regulated in metastasis family protein expressed
OsISM05344	LOC_Os01g40250	22726410	GCAATGCAGATGCAGGATAC	TGATACTTCAGCACCAAGCG	59.6	178	82	down-regulated in metastasis family protein expressed
OsISM05345	LOC_Os01g40250	22726632	TGAAGGATTTGCTGGGAAAG	AATTTCTCGGTGCCAATCAG	60.1	217	94	down-regulated in metastasis family protein expressed
OsISM05346	LOC_Os01g40260	22731215	GAAGCCGCCGTCAATCTT	CTTGCTGCCGTTCTTGCT	60.8	203	97	OsWRKY77 - Superfamily of TFs having WRKY and zinc finger domains expressed
OsISM05347	LOC_Os01g40260	22731468	GTGGAGGAAGTACGGCAAGA	CTCCACCCGCTTCTTCAC	59.8	200	98	OsWRKY77 - Superfamily of TFs having WRKY and zinc finger domains expressed
OsISM05348	LOC_Os01g40280	22741566	GTCGGAGGTGGAGGACGAT	CAGAATCTCCTTCGCTTGGT	60.9	235	95	integral membrane protein putative expressed
OsISM05349	LOC_Os01g40280	22741792	GAGATTCTGTCCAAGCAGGC	ATCCAAGAACACCCAGCAAG	60.0	571	95	integral membrane protein putative expressed

ISM IDs	Rice gene locus IDs	Physical positions (bp) on chromosomes	Forward primers (5'-3')	Reverse primers (5'-3')	Melting tempearature (T˚C)	Expected product size (bp) in PCR	Expected product size (bp) in Q-PCR	Putative Functions
OsISM05350	LOC_Os01g40280	22742364	TGGGACATTTTGCTACCTCC	GTAGTAGATCCACCGGAGCG	59.8	193	98	integral membrane protein putative expressed
OsISM05351	LOC_Os01g40280	22742537	CGCTCCGGTGGATCTACTAC	CAAGGAGAAAAGTGTTGGCA	59.3	505	82	integral membrane protein putative expressed
OsISM05352	LOC_Os01g40280	22743079	CATGGTTTGCTTCTCATTTGC	CAATTAATGCCCAAGCAAGC	60.6	141	48	integral membrane protein putative expressed
OsISM05353	LOC_Os01g40280	22743226	GCTGCAGCTTGGTTTTTAGC	TCCACCACCGGATAGTGAAT	60.2	1445	91	integral membrane protein putative expressed
OsISM05354	LOC_Os01g40280	22744818	TAGTTAATGTGCTGCGTCGG	TGTTGTTTGCTTTCTGTGCTTT	59.9	720	94	integral membrane protein putative expressed
OsISM05355	LOC_Os01g40320	22755133	CTGCGTCGGCAACTTCAC	TCCTGAAAGTTGTTGTACGGTC	60.4	694	65	DNA damage-binding protein 1 putative
OsISM05356	LOC_Os01g40320	22755804	TGACCGTACAACAACTTTCAG G	TACACAGGTGCATCAAGCAT	58.9	356	95	DNA damage-binding protein 1 putative
OsISM05357	LOC_Os01g40320	22756135	AGCCTATGCTTGATGCACCT	GAGGGAGAGGGGTTGGTAAC	59.8	600	99	DNA damage-binding protein 1 putative
OsISM05358	LOC_Os01g40320	22756715	GTTACCAACCCCTCTCCCTC	GTTTGTAGGGCGTCCAATGT	59.8	758	96	DNA damage-binding protein 1 putative
OsISM05359	LOC_Os01g40320	22757453	ACATTGGACGCCCTACAAAC	CGTAGTCGTCGAGCATCTCA	60.0	177	94	DNA damage-binding protein 1 putative
OsISM05360	LOC_Os01g40330	22768688	TTTTGGACTCTTCTGGTGGG	AAAAACCTTGGCGGATTTG	60.0	357	81	DUF630/DUF632 domains containing protein putative expressed
OsISM05361	LOC_Os01g40330	22769128	TGGTTACCATGGCAAAACAC	TGGAGCCTTGCAAATTCTTC	59.8	353	88	DUF630/DUF632 domains containing protein putative expressed
OsISM05362	LOC_Os01g40330	22769629	TCAAAGCTCAGAGATGAGGAG TT	TTCTGAACCTGATGGCACTC	59.2	683	99	DUF630/DUF632 domains containing protein putative expressed
OsISM05363	LOC_Os01g40330	22770567	GCGAGCTCTTACAGGATTACC	GACGCTCCTCAGTCTGCTGT	59.4	319	99	DUF630/DUF632 domains containing protein putative expressed
OsISM05364	LOC_Os01g40340	22776260	TTGTGAAATCCGATCATCCA	CTTCCGCAGCATCAAACATA	59.8	251	78	SIT4 phosphatase-associated protein domain containing protein expressed
OsISM05365	LOC_Os01g40340	22776491	TATGTTTGATGCTGCGGAAG	ATGTGATGCCAATCAGGTCA	59.9	405	94	SIT4 phosphatase-associated protein domain containing protein expressed
OsISM05366	LOC_Os01g40340	22776876	TGACCTGATTGGCATCACAT	TGGTCTCATCAGCACCAATC	59.8	138	63	SIT4 phosphatase-associated protein domain containing protein expressed
OsISM05367	LOC_Os01g40340	22777060	TGTCCTTGAAATGATTGCTGA T	AAAGAATTTCGGCAGCATTG	59.9	273	75	SIT4 phosphatase-associated protein domain containing protein expressed

ISM IDs	Rice gene locus IDs	Physical positions (bp) on chromosomes	Forward primers (5'-3')	Reverse primers (5'-3')	Melting tempearature (T°C)	Expected product size (bp) in PCR	Expected product size (bp) in Q-PCR	Putative Functions
OsISM05368	LOC_Os01g40340	22777314	AATGCTGCCGAAATTCTTTG	GACGAAATAGCCTCCCAACA	60.1	365	95	SIT4 phosphatase-associated protein domain containing protein expressed
OsISM05369	LOC_Os01g40340	22777834	GTTGATGGCATGCTTGAGAG	GGTGGGCGTAAACATCCATA	60.0	264	99	SIT4 phosphatase-associated protein domain containing protein expressed
OsISM05370	LOC_Os01g40340	22778078	TATGGATGTTTACGCCCACC	TGCCAATTGTCAGCAGAACT	60.0	920	77	SIT4 phosphatase-associated protein domain containing protein expressed
OsISM05371	LOC_Os01g40340	22779029	ATCGGCAATAAAGCGATCTG	TACCCTCCAGGCAAGAGACA	60.5	181	99	SIT4 phosphatase-associated protein domain containing protein expressed
OsISM05372	LOC_Os01g40340	22779271	CGCGGAAAAACTTTCTTCTTT	TGGGAATCGATTTACCATCTG	59.8	145	72	SIT4 phosphatase-associated protein domain containing protein expressed
OsISM05373	LOC_Os01g40340	22779471	AAGCAGTAACGGCACAATCC	ATCTGTTTGCCAGTCAACCC	60.1	170	65	SIT4 phosphatase-associated protein domain containing protein expressed
OsISM05374	LOC_Os01g40340	22779621	GGGTTGACTGGCAAACAGAT	CGGTCATGAAGTGATGTTGG	60.0	191	92	SIT4 phosphatase-associated protein domain containing protein expressed
OsISM05375	LOC_Os01g40340	22779870	GCTAATAACTTGAGCCAGGCA	ACCTCATCATCCCGTTCAAG	59.7	341	90	SIT4 phosphatase-associated protein domain containing protein expressed
OsISM05376	LOC_Os01g40340	22780183	AAGGGACACTTGAACGGGAT	GATCATCTCCCAGTCGCAGT	60.5	313	88	SIT4 phosphatase-associated protein domain containing protein expressed
OsISM05377	LOC_Os01g40340	22780475	CACTGCGACTGGGAGATGAT	GCAGCCAAACGGTCAGTAAT	60.7	181	98	SIT4 phosphatase-associated protein domain containing protein expressed
OsISM05378	LOC_Os01g40360	22784622	TCAGCCTGGTGCTCTTCAC	TCGTCGAGAGGATGACAATG	60.0	311	76	amino acid transporter family protein putative
OsISM05379	LOC_Os01g40360	22785058	TTCATCTATTTCATCGGCCA	CACAGCACCGAAGAGATCAG	59.3	1465	99	amino acid transporter family protein putative
OsISM05381	LOC_Os01g40370	22790928	CTCGGCTCTGTACGGCTC	AATCCATGTCTCGGAAGTCG	59.9	1104	99	phosphatase-associated family protein putative
OsISM05382	LOC_Os01g40370	22792065	AGGCATTGCGATATGGGATA	TCATCATGTTCAAGTGTCCCT	59.1	314	68	phosphatase-associated family protein putative
OsISM05383	LOC_Os01g40370	22792343	TTGAATGCAGAATCAAGGGA	CTCCCAGTCGCAGAGAACAT	59.8	320	96	phosphatase-associated family protein putative
OsISM05384	LOC_Os01g40380	22796612	TATCACCCCAGCGGAATAAA	TGAGACTGCACAGCACTGAA	59.8	805	70	amino acid/polyamine transporter II putative
OsISM05385	LOC_Os01g40380	22797452	TATGGTGAAGTTGTGCAGGC	GTAAGCCCGTTGTGCTCG	60.1	197	99	amino acid/polyamine transporter II putative
OsISM05386	LOC_Os01g40410	22814837	TCCCGACTGTGTACTCCTCA	GCACAGCACCAAAGAGATCA	59.6	1528	72	transmembrane amino acid transporter protein putative

ISM IDs	Rice gene locus IDs	Physical positions (bp) on chromosomes	Forward primers (5'-3')	Reverse primers (5'-3')	Melting tempearature (T°C)	Expected product size (bp) in PCR	Expected product size (bp) in Q-PCR	Putative Functions
OsISM05387	LOC_Os01g40520	22895304	GGAATTCAAAGGCGGAGG	CCGAGTCTTCCTTGAACCAG	60.2	388	74	hypothetical protein
OsISM05388	LOC_Os01g40560	22905469	TCCGTCAGCTCGAGATCC	AGCACGGTACTGAACACACG	59.9	269	81	hypersensitive-induced response protein putative expressed
OsISM05389	LOC_Os01g40560	22905788	ACAGATCCAGGCTCACGTCT	TCTTGTCGAACACCTCCTCC	60.1	396	75	hypersensitive-induced response protein putative expressed
OsISM05390	LOC_Os01g40610	22931274	CTGCGGCACCTACATCTACA	AAACCTAAATATTTGTATCCCCAGG	59.8	1036	92	coiled-coil domain-containing protein putative expressed
OsISM05391	LOC_Os01g40610	22932389	GGGCTAGTCGCAATTTTGAA	ATTGCATCACCCATCTCCTC	60.1	188	92	coiled-coil domain-containing protein putative expressed
OsISM05392	LOC_Os01g40610	22932595	AGCAATGGATTCAAAGCAGG	GGTCAACTGTGACTCCAGCA	60.0	192	90	coiled-coil domain-containing protein putative expressed
OsISM05393	LOC_Os01g40610	22932797	AATTTTGAAGCGCTCTGCTC	GGATTACTCGAAAAGTGATTGATTT	59.4	1640	99	coiled-coil domain-containing protein putative expressed
OsISM05394	LOC_Os01g40610	22934354	GCAATGACAGGAGGAGAAAA	TCCGTTTTACATAATCTTGGGA	58.2	220	99	coiled-coil domain-containing protein putative expressed
OsISM05395	LOC_Os01g40610	22934597	GAAGATTTGGTGATACCGGG	TTGGTCAGGACATCTGTTGC	59.5	712	90	coiled-coil domain-containing protein putative expressed
OsISM05396	LOC_Os01g40610	22935289	GCAACAGATGTCCTGACCAA	TTGGCTTGACTATAAATTTTGGC	59.6	307	98	coiled-coil domain-containing protein putative expressed
OsISM05397	LOC_Os01g40620	22947390	CTCCAGTCTCAGCATCACCA	GAGGTGCTTCACTAGCTCGG	60.1	196	95	Uncharacterized ACR COG1565 containing protein putative expressed
OsISM05398	LOC_Os01g40620	22947566	CCGAGCTAGTGAAGCACCTC	CCTCCATGTACTCGGCTACG	60.4	175	73	Uncharacterized ACR COG1565 containing protein putative expressed
OsISM05399	LOC_Os01g40620	22947793	GACATCCGGGGATTTCATC	ACATTGCCCACACACCAGT	60.0	281	72	Uncharacterized ACR COG1565 containing protein putative expressed
OsISM05400	LOC_Os01g40620	22948135	CGGAACTCTCTTGGCAGATT	TTGTAACGTGGGGCTACATTC	59.7	179	98	Uncharacterized ACR COG1565 containing protein putative expressed
OsISM05401	LOC_Os01g40620	22948414	CCCTTGAACAGGTTCCTTCA	TGAATTGGCAAGGCATCATA	60.1	934	71	Uncharacterized ACR COG1565 containing protein putative expressed
OsISM05402	LOC_Os01g40620	22949332	ATGCCTTGCCAATTCATCA	GATCCACCATCTTCTCGCAC	60.3	164	61	Uncharacterized ACR COG1565 containing protein putative expressed
OsISM05403	LOC_Os01g40620	22949477	TGCGAGAAGATGGTGGATCT	AAGAGGCTGTAGGCTGTGGA	60.4	142	71	Uncharacterized ACR COG1565 containing protein putative expressed
OsISM05404	LOC_Os01g40620	22949730	GAATTAGCTCGGATGGAGGG	TACAAACTTGTGCTTGCGGA	60.7	372	96	Uncharacterized ACR COG1565 containing protein putative expressed

ISM IDs	Rice gene locus IDs	Physical positions (bp) on chromosomes	Forward primers (5'-3')	Reverse primers (5'-3')	Melting tempearature (T˚C)	Expected product size (bp) in PCR	Expected product size (bp) in Q-PCR	Putative Functions
OsISM05405	LOC_Os01g40620	22950152	TGCATCAATCAGACACTCCG	CCAAGAGAGCCCAAGAACTG	60.4	179	88	Uncharacterized ACR COG1565 containing protein putative expressed
OsISM05406	LOC_Os01g40650	22982516	CTACAAGGACCACCTCCTCG	AGACCCGCTGATTTAGGGAT	59.8	1971	99	expressed protein
OsISM05409	LOC_Os01g40670	22987642	GCTGAGATCCAATGTTGACC	GCTACCCTTGCCCTCTCTCT	59.0	712	88	single myb histone putative expressed
OsISM05410	LOC_Os01g40670	22988473	AGCCCATGCAACTTGAGAGT	CAGACGGTTCCTTAAGCTTCTT	59.5	1081	97	single myb histone putative expressed
OsISM05411	LOC_Os01g40670	22989537	GCTTAAGGAACCGTCTGGGT	TCGTTGAAAATCTTCAGGTGG	60.3	294	83	single myb histone putative expressed
OsISM05412	LOC_Os01g40670	22989852	GCATTGGTTGCTACTGGAAAA	GTTCGAACTTGGTGCAATCC	60.3	143	64	single myb histone putative expressed
OsISM05413	LOC_Os01g40670	22990256	GTGACCACGATGCAAAACAG	CACTGGCTGAAACCCAATCT	60.1	177	88	single myb histone putative expressed
OsISM05414	LOC_Os01g40700	23000473	CAATGGACTGATACGTGTGGA	CTGAAACCCTCATTTTCCGT	59.2	441	99	expressed protein
OsISM05415	LOC_Os01g40710	23003151	TGGGCAAGAATGAGATTTCC	TCCCTACTGCAAACCCAAAC	60.0	558	55	high light inducible protein putative expressed
OsISM05416	LOC_Os01g40770	23032393	TAATGGACTCACCCCGATGT	GCATGGACTAAGGATGCCAA	60.6	645	97	retrotransposon protein putative unclassified
OsISM05417	LOC_Os01g40770	23033077	CACTGAAAATGTCAAGGCGA	CTCTTGCATGGCGTCTAACA	59.9	289	83	retrotransposon protein putative unclassified
OsISM05418	LOC_Os01g40780	23035027	GCGAGAATACTCACCGAAGC	TGGTTTGTAGGGGAATGGTT	59.6	762	81	retrotransposon protein putative unclassified
OsISM05419	LOC_Os01g40800	23052429	CGAGGGAAGAGGAAGCAA	GCCAGAAGACAAACAAGATGG	59.4	295	98	retrotransposon protein putative unclassified
OsISM05420	LOC_Os01g40800	23053689	GCCTGTGCCGAAGAAGAGT	GTTCAGTGCGGCCCTCTC	61.3	189	85	retrotransposon protein putative unclassified
OsISM05421	LOC_Os01g40820	23072919	ATACCACCACCTCTGGATCG	CGACTTTCGCCATCTGTTTC	60.3	2251	91	peptidase family M41 containing protein expressed
OsISM05422	LOC_Os01g40820	23076124	GATGAGTACTCCGGCTCACC	TGGTACACCAGCTCTTGCAG	59.9	196	88	peptidase family M41 containing protein expressed
OsISM05423	LOC_Os01g40820	23076321	TCTTTTGCCAAACAGCCTCT	GGGTCAAGTTGAAAGGACGA	60.0	346	95	peptidase family M41 containing protein expressed
OsISM05424	LOC_Os01g40820	23076647	TCGTCCTTTCAACTTGACCC	TGCACAAAGGATTGTCTTGC	60.0	289	92	peptidase family M41 containing protein expressed

ISM IDs	Rice gene locus IDs	Physical positions (bp) on chromosomes	Forward primers (5'-3')	Reverse primers (5'-3')	Melting tempearature (T˚C)	Expected product size (bp) in PCR	Expected product size (bp) in Q-PCR	Putative Functions
OsISM05425	LOC_Os01g40820	23076915	TGCAAGACAATCCTTTGTGC	TCCTGTCCTTCAGCACCTTC	60.1	426	92	peptidase family M41 containing protein expressed
OsISM05426	LOC_Os01g40820	23077374	CATGGGAAATCACAAGAGGC	CAATAGCATGCCCACTTTCA	60.1	208	81	peptidase family M41 containing protein expressed
OsISM05427	LOC_Os01g40820	23077803	ACTGCTAAAGAGCTTGCCCA	ACACTTGGCCATTTTTCCAG	60.1	162	87	peptidase family M41 containing protein expressed
OsISM05429	LOC_Os01g40840	23090025	ACCTCGGCGAGAACTTGAAC	TCCAACCTTCTCCCATTTTG	60.8	477	58	serine/threonine-protein kinase AFC3 putative expressed
OsISM05430	LOC_Os01g40840	23090470	CAAAATCTTGAGCAAAATGGG	GTCCCAGCATTCCAAAACAC	60.0	149	56	serine/threonine-protein kinase AFC3 putative expressed
OsISM05431	LOC_Os01g40840	23090666	CAAGTACAGGGATGCTGCAA	CAAACCATCCTTGAATCTGGA	59.9	180	99	serine/threonine-protein kinase AFC3 putative expressed
OsISM05432	LOC_Os01g40840	23090816	CCAGCTGTGTCCAGATTCAA	AAATCATACAAGCTTGGCCC	59.4	421	86	serine/threonine-protein kinase AFC3 putative expressed
OsISM05433	LOC_Os01g40840	23091290	GACGGCAACTGTTGGAATCT	TCTGGCTTCAGATCAGTGTGA	59.8	190	68	serine/threonine-protein kinase AFC3 putative expressed
OsISM05434	LOC_Os01g40840	23091481	AACATACTCCTTGTGTCTTCG GA	GGCACTGGACTTTGGTAAGC	59.9	194	97	serine/threonine-protein kinase AFC3 putative expressed
OsISM05435	LOC_Os01g40840	23091700	CCGCCTTTGATAATCAGGAA	AGGTCACAAGGGAAACTCCA	59.8	529	98	serine/threonine-protein kinase AFC3 putative expressed
OsISM05436	LOC_Os01g40840	23092232	GAGTGTCGGATGCATCATTG	GACCCAAAACCCTCTCCATC	60.4	175	99	serine/threonine-protein kinase AFC3 putative expressed
OsISM05437	LOC_Os01g40840	23092405	TCCACTCCCAGAACACATGA	CTTCAGGCCAATTTAGACGC	60.0	649	84	serine/threonine-protein kinase AFC3 putative expressed
OsISM05438	LOC_Os01g40840	23093034	GCGTCTAAATTGGCCTGAAG	AATGATCAGCCTTCCTCGC	60.1	639	99	serine/threonine-protein kinase AFC3 putative expressed
OsISM05439	LOC_Os01g40860	23096413	TGGAGATCAAGTTCACCAAGC	GTCACGGGTTTCGAATGTCT	60.1	6397	78	aldehyde dehydrogenase putative expressed
OsISM05440	LOC_Os01g40860	23102906	CCCTGGCCAAGAATGTCC	GTGCAGGATCCTTCCCCT	60.7	206	43	aldehyde dehydrogenase putative expressed
OsISM05441	LOC_Os01g40860	23103539	TCTCCTCCCACATGGACATT	GGGCTTCAGGTTGCTCTTC	60.1	328	96	aldehyde dehydrogenase putative expressed
OsISM05442	LOC_Os01g40860	23103942	GTCCACATGGCCTCCTACAC	TCTTCACGAACGCATCGTAG	60.2	200	92	aldehyde dehydrogenase putative expressed
OsISM05443	LOC_Os01g40860	23104171	GTCGGAGATCCGTTCAACC	CGCTTACCGATGTCGATGTA	60.1	216	93	aldehyde dehydrogenase putative expressed

ISM IDs	Rice gene locus IDs	Physical positions (bp) on chromosomes	Forward primers (5'-3')	Reverse primers (5'-3')	Melting tempearature (T˚C)	Expected product size (bp) in PCR	Expected product size (bp) in Q-PCR	Putative Functions
OsISM05444	LOC_Os01g40860	23104437	ACTACATCGAGCCCACCATC	CTTGAATTTCATGAGGGCCA	60.5	222	96	aldehyde dehydrogenase putative expressed
OsISM05445	LOC_Os01g40860	23104634	GGTGATGGCCCTCATGAAAT	TGTTCTTGGTGACTATGCCG	60.9	195	96	aldehyde dehydrogenase putative expressed
OsISM05446	LOC_Os01g40870	23112843	ACCAAGCTCTTCATCAACGG	TGGGTCCCTTGTCTTGAATG	60.6	3391	67	aldehyde dehydrogenase putative expressed
OsISM05447	LOC_Os01g40870	23116305	GAGAGAGGCCTTCGAGCAC	TTGTTCATCACCCTGCTCCT	60.4	189	67	aldehyde dehydrogenase putative expressed
OsISM05448	LOC_Os01g40870	23116822	TCCGCTCTGTACTACGCTCA	TGATCACTCCATCCGGAACT	60.1	178	56	aldehyde dehydrogenase putative expressed
OsISM05449	LOC_Os01g40870	23117036	CACTCTCCTCCCACATGGAC	TTCAGCAGAGCCAGTGAAAG	59.9	143	51	aldehyde dehydrogenase putative expressed
OsISM05450	LOC_Os01g40870	23117295	CAGTCTGTCATCCCTTGCAG	CCCTTCTTGGACATAGACGC	59.6	203	77	aldehyde dehydrogenase putative expressed
OsISM05451	LOC_Os01g40870	23117562	TTGATGCTGCCACCAATATG	AGTAGGGTTGCTCCCTCGTT	60.3	238	98	aldehyde dehydrogenase putative expressed
OsISM05452	LOC_Os01g40870	23117810	AAACCCACTGGTGACAAAGG	CTCTTGCGCAATCGTCATT	59.9	153	79	aldehyde dehydrogenase putative expressed
OsISM05453	LOC_Os01g40870	23117933	ATGCAGGAGGAAATGACGAT	CAGTTGGCCTTCTCTATCGC	59.7	213	99	aldehyde dehydrogenase putative expressed
OsISM05454	LOC_Os01g40890	23125414	TGTGCATTTGTTGCATCTGA	TGGCAAATTGTCATACGAGG	59.7	154	97	transposon protein putative unclassified
OsISM05455	LOC_Os01g40910	23136710	TGTGGCATACCAGATGCAGT	GACCGGTGTCGGTGATCTAT	60.0	876	99	transposon protein putative unclassified
OsISM05456	LOC_Os01g40910	23137643	TGAGCTACTCACGGTTCACG	CCGACACATCGGTAGGAGG	60.8	145	68	transposon protein putative unclassified
OsISM05457	LOC_Os01g40910	23137878	CCAAAGACGCACACTTTTCA	AGAGCAAGTACGCCTCCAAA	59.9	395	96	transposon protein putative unclassified
OsISM05458	LOC_Os01g40910	23138334	GCACTACGCCAGGAGCAT	GCTCCTCGTACAGTGACTCG	59.3	233	95	transposon protein putative unclassified
OsISM05459	LOC_Os01g40910	23138589	GATGGGTACTCTGTGGTGCC	GATACGACCGCCTAACCTGA	60.2	145	63	transposon protein putative unclassified
OsISM05460	LOC_Os01g40910	23138947	AGCCCTTTCCAGTACCGTTT	AGCTGACTGCTGACCCTTG	59.6	181	75	transposon protein putative unclassified
OsISM05461	LOC_Os01g40910	23139302	CACGTTGCGAGCACTAGAGA	GTTGAACCGTCAACGACGA	60.5	190	96	transposon protein putative unclassified

ISM IDs	Rice gene locus IDs	Physical positions (bp) on chromosomes	Forward primers (5'-3')	Reverse primers (5'-3')	Melting tempearature (T°C)	Expected product size (bp) in PCR	Expected product size (bp) in Q-PCR	Putative Functions
OsISM05462	LOC_Os01g40910	23139665	ATCTAGCTGCGCATGTTCCT	ACAACAGAAGGCCTAGCCG	60.2	172	95	transposon protein putative unclassified
OsISM05463	LOC_Os01g40910	23139774	GATTGCTTCAGGGTTTCGTG	CTGCGTCATAGCGAACTGG	60.6	186	98	transposon protein putative unclassified
OsISM05464	LOC_Os01g40910	23140165	CTTCAGGCTCGACAGAGGAC	CCCTTCTGGTGTCTATCCATTC	60.0	1337	88	transposon protein putative unclassified
OsISM05465	LOC_Os01g40920	23144144	ATGACGGTTGTGACGGTTG	TCCGTCGATACATGTGTGCT	60.3	1769	63	hypothetical protein
OsISM05466	LOC_Os01g40920	23146031	CCGCTAGATCTCAACAATCAA	CCGAGCATGTAAACTGGGAT	59.0	205	89	hypothetical protein
OsISM05467	LOC_Os01g40930	23152367	TTTGGGATCAGTCTTGGACC	ACTGCAGTCTTGGGGTCTTC	59.6	268	69	hypothetical protein
OsISM05468	LOC_Os01g40930	23152612	CATGAAGACCCCAAGACTGC	CTGTGTGTGCCCAAGGTTT	60.1	392	99	hypothetical protein
OsISM05469	LOC_Os01g40930	23153014	AAATGGCATCCCAGATGTTC	CGCCTTATGCTTGTGCTTCT	60.1	279	80	hypothetical protein
OsISM05470	LOC_Os01g40930	23153525	ATTGTGCCCAAGAATTGGAG	CACCCTTTTGCTGCTTTCAT	60.1	124	78	hypothetical protein
OsISM05471	LOC_Os01g40930	23153711	TGGTTGGATGAGTAATTGGG	GGCGAGGTTATCCTTGTGAA	59.2	1177	98	hypothetical protein
OsISM05472	LOC_Os01g40960	23170434	AAGGACCCCCACCCTAGAC	TGGCCTTCAGGATCCTCTTA	60.0	182	99	nodulin MtN3 family protein putative
OsISM05473	LOC_Os01g41010	23224820	GCGGTGAGAAACTCGAAGAA	ATGAAATTCTCCCGGCACTC	60.9	228	86	DUF581 domain containing protein expressed
OsISM05474	LOC_Os01g41020	23226951	CTCCTCGCCAAGTTTGTGTT	ACCACTTGCAGATACCCCAC	60.1	855	97	hypothetical protein
OsISM05475	LOC_Os01g41020	23227786	GTGGGGTATCTGCAAGTGGT	TGTTGTGCCTGCTCTGAATC	59.9	393	55	hypothetical protein
OsISM05476	LOC_Os01g41020	23228190	ATTGATGCCAAAGTGGCTTG	TATAGGTGGCCACTCTGGGT	60.2	666	97	hypothetical protein
OsISM05477	LOC_Os01g41020	23228925	CGTATTTCAAAATGCCCTGC	AAAGATGCTCATGCCACACA	60.4	94	49	hypothetical protein
OsISM05478	LOC_Os01g41020	23228937	TGCCCTGCTACAGGGTAAAA	TCCTAATTTTGACTGACCACCA	60.0	324	98	hypothetical protein
OsISM05479	LOC_Os01g41020	23229249	TCAAAATTAGGAACTCCCCA	GAGCTGTTCGAAGAACCCAC	58.5	1039	88	hypothetical protein

ISM IDs	Rice gene locus IDs	Physical positions (bp) on chromosomes	Forward primers (5'-3')	Reverse primers (5'-3')	Melting tempearature (T˚C)	Expected product size (bp) in PCR	Expected product size (bp) in Q-PCR	Putative Functions
OsISM05480	LOC_Os01g41020	23230209	TTGTCTCCTTCAATCAGGGG	CATGGACGTTCCAATTTTCC	60.1	1112	92	hypothetical protein
OsISM05482	LOC_Os01g41040	23240331	ATTCCAATATCACGGCTTCG	TAACAAGCATCTCTTGGGGG	60.0	1858	95	SCF apoptosis response protein putative expressed
OsISM05483	LOC_Os01g41050	23246654	CATCAGCTACGCCACCCT	GTTCCTCGAGCTCCCCAT	60.0	196	98	sulfate transporter putative expressed
OsISM05484	LOC_Os01g41050	23247144	GTCCTCCGCTACATCTTCCA	GAAGATGAGGAAGCAGACGC	60.2	612	76	sulfate transporter putative expressed
OsISM05485	LOC_Os01g41050	23247741	TGCTTCCTCATCTTCCTCGT	CAGAGGAGACATCGCTGACA	60.0	175	82	sulfate transporter putative expressed
OsISM05486	LOC_Os01g41050	23247927	CGTAGGATGCGTCTTCTCGT	ATATCGAGCTCGGGTTGATG	60.2	184	93	sulfate transporter putative expressed
OsISM05487	LOC_Os01g41050	23248160	GTTCGTGTCAGGGATGCTC	CGATGCGCTCTTTCTTCATC	60.1	181	78	sulfate transporter putative expressed
OsISM05488	LOC_Os01g41050	23248370	CTCATGAACCTCATCGGCTC	GGTAGTTCACCGCCGTCTT	60.4	165	74	sulfate transporter putative expressed
OsISM05489	LOC_Os01g41060	23254173	TGGTCGTTTAGTCGACCTCC	CTGTCTCCTCGGCCTCAG	59.9	185	86	retrotransposon protein putative unclassified
OsISM05490	LOC_Os01g41060	23254804	AACACGACGGTGCTGGAC	GACGCTGCCTCGTTCTCC	61.4	339	78	retrotransposon protein putative unclassified
OsISM05491	LOC_Os01g41080	23263624	GAAACTACAGGAGGATGGCG	AGAAGGCGGAGGAAGAAAAC	59.8	139	83	retrotransposon protein putative unclassified
OsISM05492	LOC_Os01g41080	23264174	GTACGAGGTTTGTCGGGGTA	CTGATCATCAATTGGCCGAC	60.6	258	90	retrotransposon protein putative unclassified
OsISM05493	LOC_Os01g41080	23264439	ACAACGGCAAGCAACGTC	CCGATGATATCAGTCAGAGGG	60.2	162	93	retrotransposon protein putative unclassified
OsISM05494	LOC_Os01g41080	23264870	CAGCAGCTTGTGACGCTTG	AGAGGTCCCTCCAGATTCGT	61.0	145	96	retrotransposon protein putative unclassified
OsISM05495	LOC_Os01g41080	23265706	AGTCAATCCTCCCGCTTTTT	CTCGAGTTCCGCTACCTCAA	60.3	494	51	retrotransposon protein putative unclassified
OsISM05496	LOC_Os01g41110	23279051	CTGCTCATGCAGACTGACCT	TTGGCGTTGTACTCCTTGCT	60.0	307	99	hypothetical protein
OsISM05497	LOC_Os01g41190	23315804	TTCCAAGAGGAGAGTCGTGG	CCATAACTTCCTCCCACCAA	60.1	1721	55	glycine-rich protein putative expressed
OsISM05498	LOC_Os01g41190	23317571	TGAATTTGCATGAGGAGCAC	TAAAGCGACCCCTTGACATT	59.7	134	52	glycine-rich protein putative expressed

ISM IDs	Rice gene locus IDs	Physical positions (bp) on chromosomes	Forward primers (5'-3')	Reverse primers (5'-3')	Melting tempearature (T˚C)	Expected product size (bp) in PCR	Expected product size (bp) in Q-PCR	Putative Functions
OsISM05499	LOC_Os01g41190	23317856	TGTTGCAAAGAACAACCGAG	CTGAAGCACTTGCAGCAGAC	59.9	239	81	glycine-rich protein putative expressed
OsISM05500	LOC_Os01g41190	23318853	CAAAATTTTCCTGGCACTCC	AGGAACTCCAACACCACCAG	59.8	197	76	glycine-rich protein putative expressed
OsISM05501	LOC_Os01g41190	23319093	TGGGAATGGGAAATAACGAA	ATGATCCTCCAAAAGCACCA	60.3	582	70	glycine-rich protein putative expressed
OsISM05502	LOC_Os01g41190	23319720	CAGGACAGACCTCATCGTCA	GCCCCCTTGTTAGCAATAGA	59.5	559	53	glycine-rich protein putative expressed
OsISM05503	LOC_Os01g41190	23320262	ATTGCTAACAAGGGGGCTTC	AGGCTTGTTCTGACGCTGAT	60.2	170	73	glycine-rich protein putative expressed
OsISM05504	LOC_Os01g41190	23320412	ATCAGCGTCAGAACAAGCCT	TTCACCTTACATGGTGCTTCA	59.6	163	69	glycine-rich protein putative expressed
OsISM05505	LOC_Os01g41200	23323561	TGATCTTGAAGACGAACCGA	TGGCAGTGCATAGACACCTT	59.3	250	82	heavy metal-associated domain containing protein expressed
OsISM05506	LOC_Os01g41200	23323819	CGAGGAAGGTTGAGAAGCAA	GACCACGGTCACCTTCTTGT	60.3	185	84	heavy metal-associated domain containing protein expressed
OsISM05507	LOC_Os01g41210	23327975	CGCCTCCAACTTGAATCG	CTCATGCGTGGGATCTACAA	60.0	716	95	transposon protein putative unclassified expressed
OsISM05508	LOC_Os01g41240	23342409	ACAGGGTGCTGCCTCATCT	GGAGAAGTCCCAGTCGAACA	60.5	149	60	hydrolase alpha/beta fold family domain containing protein expressed
OsISM05509	LOC_Os01g41240	23342707	CTGCATTGCCTCCATCAAC	CCCTCGTAGTCATCCGAGTT	59.7	930	91	hydrolase alpha/beta fold family domain containing protein expressed
OsISM05510	LOC_Os01g41250	23347541	TTGGGTACTTCGATGTCGGT	ATTGCACTTGGCTTCACCTT	60.1	174	83	OsFBX17 - F-box domain containing protein expressed
OsISM05511	LOC_Os01g41250	23347790	GCTTAGATGCTTCCCATGCT	GATCCCAGAACTCCACTCCA	59.7	175	87	OsFBX17 - F-box domain containing protein expressed
OsISM05512	LOC_Os01g41260	23351736	ACTTGGAGCCAAGAATCCAC	GGACCTTGACAGTTGGAACC	59.3	196	97	OsFBD2 - F-box and FBD domain containing protein
OsISM05513	LOC_Os01g41260	23351969	CAAGATGCTGCTCTCCTTCC	CAGTGGGTTCATCAGCTTCA	60.0	567	81	OsFBD2 - F-box and FBD domain containing protein
OsISM05514	LOC_Os01g41270	23357109	GAGCTGGAGATCGGCAATAC	CACCTTCAAGGCCAAGATTT	59.5	230	91	OsFBD3 - F-box and FBD domain containing protein expressed
OsISM05515	LOC_Os01g41270	23357372	CCCGTCTTTCTCAGATGCTT	GAGATTGAGCTTGCCGGTAG	59.7	196	85	OsFBD3 - F-box and FBD domain containing protein expressed
OsISM05516	LOC_Os01g41280	23360494	CTCGGTTACTTGGACATGGG	CTAGGAACTTTTGCCATCGG	60.0	203	96	OsFBD4 - F-box and FBD domain containing protein expressed

ISM IDs	Rice gene locus IDs	Physical positions (bp) on chromosomes	Forward primers (5'-3')	Reverse primers (5'-3')	Melting tempearature (T°C)	Expected product size (bp) in PCR	Expected product size (bp) in Q-PCR	Putative Functions
OsISM05517	LOC_Os01g41280	23360744	TGGAGATTTTGCCCAGTTTC	GAACTTGGAGTTGAGCTCGC	60.1	191	99	OsFBD4 - F-box and FBD domain containing protein expressed
OsISM05518	LOC_Os01g41290	23363787	GTTGCTGGGATACTTGAGGC	CTTGGGATGATGGTTCTTGG	60.0	208	97	OsFBD5 - F-box and FBD domain containing protein expressed
OsISM05519	LOC_Os01g41290	23364070	CTTTCCCAATGTCGAGAAGC	GCCAGAACTTGAGGTTGAGC	59.9	282	78	OsFBD5 - F-box and FBD domain containing protein expressed
OsISM05520	LOC_Os01g41300	23366589	TGAAGACTATGGGCAGGAGG	CATAGCGTGCATTGATTTGG	60.2	728	78	hypothetical protein
OsISM05521	LOC_Os01g41310	23376472	ATCTGGAGCCAAGAGCTCAC	ACCATGGAGCTTGGAATCAC	59.7	203	80	OsFBX18 - F-box domain containing protein expressed
OsISM05522	LOC_Os01g41310	23376744	CTTCCTCAGATGCTTTCCCA	TGGCCAGTGAACTCATCATC	60.0	195	70	OsFBX18 - F-box domain containing protein expressed
OsISM05523	LOC_Os01g41350	23396510	CACATGGATACTGGTGCCAA	TGAAAGCTCCATCCCTTGAG	60.4	131	99	hypothetical protein
OsISM05524	LOC_Os01g41350	23397311	TCTATCAGATGGGGGAGTGG	TCAAGCTGGGTAGACTGAGGA	59.9	1524	78	hypothetical protein
OsISM05526	LOC_Os01g41360	23402128	CATCCTTTGGAGGGATGATG	CAGCGCTAGCCTTCATGTC	60.0	304	96	expressed protein
OsISM05527	LOC_Os01g41360	23402509	TGCTTCCTCAAGTGTTTCCC	TCCAGAACTCCAAGTTAACCTCA	60.2	205	92	expressed protein
OsISM05528	LOC_Os01g41370	23408694	CTGTCATGGCCTGCATATTG	GCTTGGAACAGCCACTTCAT	60.2	627	77	FBD domain containing protein putative expressed
OsISM05529	LOC_Os01g41370	23409381	TGCATGCTCAGATGTTTTCC	TCAAGCTGGGTAGACTGAGGA	59.9	184	75	FBD domain containing protein putative expressed
OsISM05530	LOC_Os01g41400	23439209	CCGACCGTGTACTCCTCAAT	CAACACCGAAGAGATCAGCA	60.0	626	67	transmembrane amino acid transporter protein putative expressed
OsISM05531	LOC_Os01g41420	23447418	TCTGCTGCTAGGAGTGTTCG	GGAGGAGAACCCCGTCAC	59.9	1862	80	transmembrane amino acid transporter protein putative expressed
OsISM05532	LOC_Os01g41450	23463168	GCGGTTGTGCAACGAGAT	AGCTCGTGGAAGCTGTTGA	60.3	193	43	UDP-glucoronosyl and UDP-glucosyl transferase domain containing protein
OsISM05533	LOC_Os01g41460	23472132	GCCTCGGTCCCAGTAAATTC	GTGAAGATCGACGAGACGC	60.2	229	87	retrotransposon protein putative Ty3-gypsy subclass
OsISM05534	LOC_Os01g41480	23481617	ATGAACAACGGTGACCGAG	TGTTGCTCCGCAGAGATATG	59.8	1314	84	retrotransposon protein putative Ty3-gypsy subclass
OsISM05535	LOC_Os01g41480	23483102	TCTTGATCCCTTTGATCCCA	TATCAGATGGTTTCCACGCA	60.2	125	78	retrotransposon protein putative Ty3-gypsy subclass

ISM IDs	Rice gene locus IDs	Physical positions (bp) on chromosomes	Forward primers (5'-3')	Reverse primers (5'-3')	Melting tempearature (T°C)	Expected product size (bp) in PCR	Expected product size (bp) in Q-PCR	Putative Functions
OsISM05536	LOC_Os01g41490	23484298	GCCGACTCCAAGACGAGATA	ACCGTGACCGAGTAACCTTG	60.2	104	64	retrotransposon protein putative Ty3-gypsy subclass
OsISM05537	LOC_Os01g41490	23485316	CCATTGTTGGCAAGGCTTAT	TGCAACTCCTGAGCTGGTAA	59.8	133	96	retrotransposon protein putative Ty3-gypsy subclass
OsISM05538	LOC_Os01g41510	23498654	ACCAACAAAGCAAGTGGGTC	TGCCTCAACTTCATTCACAG	58.7	516	98	calcineurin B putative expressed
OsISM05539	LOC_Os01g41510	23499162	TTGAGGCATTATACGATCTCTTCA	TGTTCCTAAAGAGAGCAAGATGG	60.0	182	97	calcineurin B putative expressed
OsISM05540	LOC_Os01g41510	23499310	CAGGAAGAATTCCATCTTGCTC	TCGATGACACCATTTCCTTTT	59.8	580	99	calcineurin B putative expressed
OsISM05541	LOC_Os01g41510	23499933	GCACCTGAGGAACAGAAAGC	CATGCCTTTCAATAAACCCTG	59.7	193	74	calcineurin B putative expressed
OsISM05542	LOC_Os01g41510	23500103	GACAGGGTTTATTGAAAGGCA	GAAGAGCCAACACCATCTCC	59.4	411	48	calcineurin B putative expressed
OsISM05543	LOC_Os01g41510	23500494	GGAGATGGTGTTGGCTCTTC	CTTTTGTGTCAGCCTGGTCA	59.8	191	99	calcineurin B putative expressed
OsISM05544	LOC_Os01g41510	23500745	TGAGGAATATGACCCTCCCA	TCTGCTTCGCTGACTTCTGA	60.1	361	83	calcineurin B putative expressed
OsISM05545	LOC_Os01g41600	23550976	GTGCCTCCTCTTCCTCCTTC	CAGTAGTTTTCGCGCATTCC	60.6	189	97	Sad1 / UNC-like C-terminal domain containing protein putative expressed
OsISM05546	LOC_Os01g41600	23552328	ACCTCGTTGAGTGGAAGGG	GATGAGATCGTCCAGCTTGC	60.5	204	90	Sad1 / UNC-like C-terminal domain containing protein putative expressed
OsISM05547	LOC_Os01g41600	23552513	CAAGCTGGACGATCTCATCA	TTGTTCTGCATCGTCTCCTG	60.0	176	85	Sad1 / UNC-like C-terminal domain containing protein putative expressed
OsISM05548	LOC_Os01g41630	23569151	GTTCTCGCAGGTGTTCGG	GGCAAGATGCTGTCCTGATT	60.3	1496	98	serine/threonine protein phosphatase 2A 55 kDa regulatory subunit B putative expressed
OsISM05549	LOC_Os01g41630	23570677	TTTGAAAGGACAGATGCCAA	GGATGCCTGCTAACGGAATA	59.7	179	75	serine/threonine protein phosphatase 2A 55 kDa regulatory subunit B putative expressed
OsISM05550	LOC_Os01g41630	23570836	TATTCCGTTAGCAGGCATCC	TCTTCAATTTCCAAGCTCTTCA	59.6	175	93	serine/threonine protein phosphatase 2A 55 kDa regulatory subunit B putative expressed
OsISM05551	LOC_Os01g41630	23571048	GGAGCATTGTTTCTTCTGTCC	GAAGGGCTCAAATTCATCTCA	59.0	390	99	serine/threonine protein phosphatase 2A 55 kDa regulatory subunit B putative expressed
OsISM05552	LOC_Os01g41630	23571519	ACCTGTTGGACTCAATTCGC	ATGCGTAAAGACTCTTCGGC	59.8	301	89	serine/threonine protein phosphatase 2A 55 kDa regulatory subunit B putative expressed
OsISM05553	LOC_Os01g41630	23571815	CGCATGCACATGACTACCAT	TTCCAAAGATTTATCCGCAAA	60.1	820	92	serine/threonine protein phosphatase 2A 55 kDa regulatory subunit B putative expressed

ISM IDs	Rice gene locus IDs	Physical positions (bp) on chromosomes	Forward primers (5'-3')	Reverse primers (5'-3')	Melting tempearature (T°C)	Expected product size (bp) in PCR	Expected product size (bp) in Q-PCR	Putative Functions
OsISM05554	LOC_Os01g41630	23572678	CCTACAAACATGGAGGACCTG	AGGGTGGAACTCAGCAGATG	59.9	743	52	serine/threonine protein phosphatase 2A 55 kDa regulatory subunit B putative expressed
OsISM05555	LOC_Os01g41630	23573487	TCAGCCCTTTGTGACACTCA	GAATGATCTTGAACCTGGGG	59.9	196	64	serine/threonine protein phosphatase 2A 55 kDa regulatory subunit B putative expressed
OsISM05556	LOC_Os01g41630	23573719	TTTTCCAAGGATGGGAGACA	TGTTGCAACTGGACCAGAGT	59.9	456	88	serine/threonine protein phosphatase 2A 55 kDa regulatory subunit B putative expressed
OsISM05557	LOC_Os01g41630	23574167	TTGCAACATTCCAGGTTCAT	ATCTCCGCTTTGACAGCACT	59.5	211	99	serine/threonine protein phosphatase 2A 55 kDa regulatory subunit B putative expressed
OsISM05558	LOC_Os01g41630	23574358	AGTGCTGTCAAAGCGGAGAT	TGTCGTTGCCTCATTGCTAC	59.9	184	99	serine/threonine protein phosphatase 2A 55 kDa regulatory subunit B putative expressed
OsISM05559	LOC_Os01g41630	23574523	TAGCAATGAGGCAACGACAC	GCCTTGTGGGATTCTGAAGT	59.5	275	69	serine/threonine protein phosphatase 2A 55 kDa regulatory subunit B putative expressed
OsISM05560	LOC_Os01g41630	23574766	AACCAGGAGGCAACTTCAGA	CCTCTATTCCGGTGCTTTCA	60.0	176	96	serine/threonine protein phosphatase 2A 55 kDa regulatory subunit B putative expressed
OsISM05561	LOC_Os01g41640	23577061	CTCCATCTCGGTCTTCCAGA	AACAGGTAGCGGAGGATTCA	60.0	179	74	expressed protein
OsISM05562	LOC_Os01g41640	23577210	AACATGCTCGTGAATCCTCC	CCACCAAAATCAGAGCATCA	59.9	1018	98	expressed protein
OsISM05563	LOC_Os01g41640	23578282	TGCCCCTTTATGTGTCAAGA	ATTCATGAAAGACATCGCCC	59.5	168	93	expressed protein
OsISM05564	LOC_Os01g41640	23578528	CCAGCTGCTATCACACCTCA	CCTGGAAACATTCTCTCCAAA	59.6	190	88	expressed protein
OsISM05565	LOC_Os01g41690	23594844	AAGGACATGGCCCTAAAAGG	TAGTCTGGGGCTCTTTGGAA	60.1	316	88	transposon protein putative unclassified
OsISM05566	LOC_Os01g41690	23596344	CAGCGGATCGTCACATGC	CTCCATGCGAAGACGTCAG	61.5	155	81	transposon protein putative unclassified
OsISM05567	LOC_Os01g41730	23624568	TGTTCTTACATGGGAAGCCC	GCTCTATCCCCTCATGCAAA	60.1	164	78	serine/threonine-protein kinase putative expressed
OsISM05568	LOC_Os01g41730	23624819	AGCTCTTACGTGCTGGGAAA	TTCTCGTTTAGCTGCCCAGT	60.0	266	98	serine/threonine-protein kinase putative expressed
OsISM05569	LOC_Os01g41730	23625112	ACTCTTGGAAGCAGTGACCG	GCTAGCCATCAGCTTGATCC	60.2	301	92	serine/threonine-protein kinase putative expressed
OsISM05571	LOC_Os01g41740	23629488	CGCTGAGGGATCTAGTTTGC	ATCGAGAGGCCCATTTACG	60.0	202	51	hypothetical protein
OsISM05572	LOC_Os01g41870	23728181	ATCTTGCTTCTCGTCGTCGT	TAGTCTTGTTGCCACTTGCG	60.0	347	95	protein kinase putative expressed

ISM IDs	Rice gene locus IDs	Physical positions (bp) on chromosomes	Forward primers (5'-3')	Reverse primers (5'-3')	Melting tempearature (T°C)	Expected product size (bp) in PCR	Expected product size (bp) in Q-PCR	Putative Functions
OsISM05573	LOC_Os01g41870	23728552	AGCTGTCTTCTCAAATGATGGA	GGTGACCCAGACACAACCTT	59.4	177	86	protein kinase putative expressed
OsISM05574	LOC_Os01g41870	23728729	TCCTGGTGTTGTAGCTGCTG	CCACGCAATGTTTCTCTTCA	59.9	181	90	protein kinase putative expressed
OsISM05575	LOC_Os01g41870	23729877	TGGATCCTTGGATTCACATCT	CCCCAACTGCAATCTTTTGT	59.7	315	78	protein kinase putative expressed
OsISM05576	LOC_Os01g41870	23730247	TCGTGACATGAGACCAAACAA	ACCCATGTCACCATCAGGTT	60.1	462	98	protein kinase putative expressed
OsISM05577	LOC_Os01g41870	23730700	GACATGGGTGTTGACACGAG	CTCTGTGCGTATTCTGGTGC	59.7	150	63	protein kinase putative expressed
OsISM05578	LOC_Os01g41870	23730945	AGGGCCAGCAATTTTTGACT	TCACCCAAGCGTGGATCTAT	60.7	323	83	protein kinase putative expressed
OsISM05579	LOC_Os01g41870	23731325	ACGTGATCCTCATTCAAGGC	GAGCCAGAATCGACAACCAT	60.1	175	76	protein kinase putative expressed
OsISM05580	LOC_Os01g41890	23746611	GAGCTGAGAAGTTTTCTCCAGAGT	TTCCACACATTTTCATCCCA	59.7	1984	72	MLA1 putative expressed
OsISM05581	LOC_Os01g41900	23759891	ATGGCAGAATTGTCCAGGAG	GAGCCCGACGAGGAACAG	61.0	531	75	Myb transcription factor putative expressed
OsISM05582	LOC_Os01g41900	23760501	ACGCCCAGAAGTTCTTCCTC	GCACCGTTCTCGCAAATC	60.6	192	92	Myb transcription factor putative expressed
OsISM05583	LOC_Os01g41910	23769613	CCTTGCATATAACAGGATATCAGGA	CAAGAGATGTTATTTCCTCTGGG	59.8	290	99	receptor-like protein kinase 5 precursor putative
OsISM05584	LOC_Os01g41910	23770161	TCTGTGGACCTCCTCTTTCAA	TGCAGATGTTTTCCACACAGA	60.1	236	75	receptor-like protein kinase 5 precursor putative
OsISM05585	LOC_Os01g41960	23788229	AGAGGTTGTTGTGGGTTCCA	TGCACTGAAGGATTTGAAGG	59.6	519	99	retrotransposon protein putative unclassified
OsISM05586	LOC_Os01g41960	23789662	CTCCTGGCTAAGGAAAGGCT	GACCAATCATTGTTGCCCTC	60.2	1441	89	retrotransposon protein putative unclassified
OsISM05587	LOC_Os01g41960	23792297	GAGAGGAAGGAACCACCACA	AGTCGCATCGACCATACAGC	60.7	191	56	retrotransposon protein putative unclassified
OsISM05588	LOC_Os01g41960	23792563	CGGCCTTCAGCTGGTTAGTA	CTGGGTTATTTAGTCCGCGA	60.2	250	90	retrotransposon protein putative unclassified
OsISM05589	LOC_Os01g41960	23793236	TGAACAGGCATCTAATGGGG	TTGTTTCCAGGGCTACATCC	60.4	486	49	retrotransposon protein putative unclassified
OsISM05590	LOC_Os01g41970	23800336	AATATGCCCTTTCGGATCAA	GTGCCATTTGAGAAGACGGT	59.7	195	98	hypothetical protein

ISM IDs	Rice gene locus IDs	Physical positions (bp) on chromosomes	Forward primers (5'-3')	Reverse primers (5'-3')	Melting tempearature (T˚C)	Expected product size (bp) in PCR	Expected product size (bp) in Q-PCR	Putative Functions
OsISM05592	LOC_Os01g41980	23806232	GGGATGATGCTGGTGCTACT	CCTACCACGTCGAGAACGA	60.0	871	93	retrotransposon protein putative unclassified
OsISM05593	LOC_Os01g41980	23807150	CTCTGAGGAGGAACTCTGGC	GAGGAGGACGACGTCACAAC	59.9	369	92	retrotransposon protein putative unclassified
OsISM05594	LOC_Os01g42030	23838897	GCTACAACAACGGGAGGAAG	TCCACGAAGTTGAGTAGCCC	60.0	184	88	mitochondrial chaperone BCS1 putative expressed
OsISM05595	LOC_Os01g42040	23843375	GTGCCTTGTCATCGTTGTTG	TGGATCTTCAGAACAAAAACTCC	59.9	227	89	ubiquitin-conjugating enzyme putative expressed
OsISM05596	LOC_Os01g42040	23843543	GGAATACAGGGACCTGATGAA	TGTCACATTGGGAGGTTGAA	59.4	176	97	ubiquitin-conjugating enzyme putative expressed
OsISM05597	LOC_Os01g42040	23843750	ACAATGGAGGACGAATTTGC	AGCAATGTTGAGCGATGGTT	60.3	211	75	ubiquitin-conjugating enzyme putative expressed
OsISM05598	LOC_Os01g42040	23843984	GCTGCTAAGTGACCCAAACC	CCAGGACCGAGCATTTATGT	59.8	235	98	ubiquitin-conjugating enzyme putative expressed
OsISM05599	LOC_Os01g42040	23844251	CGCTAGTGGTTGGAGCTCTG	AGGCTCCAGGCTTAGCTTCT	60.4	188	74	ubiquitin-conjugating enzyme putative expressed
OsISM05600	LOC_Os01g42060	23846091	CAACAACAACAACAACCGCT	GTCATGCCCAATCAGGGG	61.0	254	84	expressed protein
OsISM05601	LOC_Os01g42060	23846403	CCATCGCTTGGATGGTTACT	CCTTGTTATCTTCTGCCACCA	60.0	346	97	expressed protein
OsISM05602	LOC_Os01g42060	23847023	TGTTGATCGATGTGAGGGAA	GGAGTAGAGCTGGCCTTCAG	59.6	171	56	expressed protein
OsISM05603	LOC_Os01g42060	23847175	TGAAGGCCAGCTCTACTCCT	ATGTGCAAGTGAATGCAACC	59.4	319	91	expressed protein
OsISM05604	LOC_Os01g42060	23847477	TGCATTCACTTGCACATTCA	GGTCCCATCTAGTGAGCCAA	60.0	140	67	expressed protein
OsISM05605	LOC_Os01g42060	23847816	TTACTTTGTTGACACCGGCA	TGCAAAATCATTCAGGTCAG	58.7	215	99	expressed protein
OsISM05606	LOC_Os01g42060	23848011	CTGACCTGAATGATTTTGCAC T	CTTCTCGTGTAGCACTGCGT	59.3	233	92	expressed protein
OsISM05607	LOC_Os01g42060	23848225	CGCAGTGCTACACGAGAAGT	AGAACAAGACGGATGTTGCG	60.3	270	59	expressed protein
OsISM05608	LOC_Os01g42060	23848579	TGGCTTGAAAAAGTTCCTGG	TCGTGGACAGTCTTCTGCTC	59.7	190	97	expressed protein
OsISM05609	LOC_Os01g42080	23854815	CATGCACGATGAGTACGACC	CTTGTGATTCCAGCCCATCT	60.1	857	99	zinc ion binding protein putative expressed

ISM IDs	Rice gene locus IDs	Physical positions (bp) on chromosomes	Forward primers (5'-3')	Reverse primers (5'-3')	Melting tempearature (T°C)	Expected product size (bp) in PCR	Expected product size (bp) in Q-PCR	Putative Functions
OsISM05610	LOC_Os01g42080	23855946	CTCCTCCAAGCCCTCTTCC	ACCATCCCGAACCTCCTC	60.6	206	94	zinc ion binding protein putative expressed
OsISM05611	LOC_Os01g42080	23856267	ATGACCTGGTTCGCCAAA	ATCCTCTCCTTGCGCAGTTA	60.0	522	56	zinc ion binding protein putative expressed
OsISM05612	LOC_Os01g42080	23856846	TCTCCACTCCTTCCTCATGG	GCTAAAAACAGCAGCAGCCT	60.0	310	57	zinc ion binding protein putative expressed
OsISM05613	LOC_Os01g42080	23857445	TGGATCACACTTTTCAAGATGC	GGTTCCCAACCACACAAACT	59.9	960	71	zinc ion binding protein putative expressed
OsISM05614	LOC_Os01g42080	23859337	CGTCTAACGATGGGGAAAAG	GAAAGTCCTGTGCTGCTTCA	59.4	462	99	zinc ion binding protein putative expressed
OsISM05615	LOC_Os01g42080	23859894	TTCACAAGTGGAGCCTCAGA	GAAGGTGGTGTCATGAAAAGC	59.6	239	85	zinc ion binding protein putative expressed
OsISM05616	LOC_Os01g42090	23863741	ATCGGCAATGTCATCTCCTT	CTTGAACTGCTCCACGTCCT	60.0	210	88	nodulin MtN3 family protein putative expressed
OsISM05617	LOC_Os01g42090	23864092	TTCCTCTACTCCCCCAACAA	CAGCATGAACACCAACTCCA	60.1	186	70	nodulin MtN3 family protein putative expressed
OsISM05618	LOC_Os01g42090	23864366	TCATGTACTTCTCCCCGCTC	CAGGAAGAACGGCATGTACTC	60.0	1377	75	nodulin MtN3 family protein putative expressed
OsISM05619	LOC_Os01g42110	23876619	ATCGGCAATGTCATCTCCTT	AACTCCTCCACGTCCTTCCT	59.8	203	84	nodulin MtN3 family protein putative
OsISM05620	LOC_Os01g42110	23876945	GGGCACCTACCTCCTCATCT	ACATGAACACCAACTCCACG	60.0	202	90	nodulin MtN3 family protein putative
OsISM05621	LOC_Os01g42110	23877223	CTTCTTCGGCTCCATCATGT	GGCATGTACTCCACGCTCTT	60.3	1697	79	nodulin MtN3 family protein putative
OsISM05622	LOC_Os01g42120	23882305	GCTTCACTGCCCTAATGACC	GCGGGATATGAGGCATAAAA	59.8	183	89	expressed protein
OsISM05623	LOC_Os01g42120	23882468	TTTTATGCCTCATATCCCGC	GGACCCCAGTCATCTATTTCG	60.3	241	99	expressed protein
OsISM05624	LOC_Os01g42120	23882731	AAACATTTTGATCGGCAAGG	GGAGAGAAGGTGTGCCATGT	60.0	232	67	expressed protein
OsISM05625	LOC_Os01g42190	23910724	TCGTACTACGCCGTGCTC	GTGATCTTGTCCGGGTGC	59.3	221	99	heat shock protein DnaJ putative expressed
OsISM05626	LOC_Os01g42190	23910964	GAGGAAGCCAAGAGCAGGTT	GTAGAGCGCCCTCCTCTTCT	60.5	195	76	heat shock protein DnaJ putative expressed
OsISM05627	LOC_Os01g42190	23911159	GACTCAGGCATGTACGACCC	CATGAGGGACACCATCTCCT	60.2	212	88	heat shock protein DnaJ putative expressed

ISM IDs	Rice gene locus IDs	Physical positions (bp) on chromosomes	Forward primers (5'-3')	Reverse primers (5'-3')	Melting tempearature (T˚C)	Expected product size (bp) in PCR	Expected product size (bp) in Q-PCR	Putative Functions
OsISM05628	LOC_Os01g42190	23911344	TTCCTCCAGGAGATGGTGTC	AGCTCGTCCAGGCAGTACAC	60.3	221	69	heat shock protein DnaJ putative expressed
OsISM05630	LOC_Os01g42210	23919412	GCCAGAGATGCGTTTCCTAC	GGGCATTGATCCTGGGAC	60.6	530	74	LTPL47 - Protease inhibitor/seed storage/LTP family protein precursor putative
OsISM05631	LOC_Os01g42234	23922839	GACTACGACGAGCGGAAGC	GACACGATCGAGAACGACAC	60.2	1083	99	amino acid permease family protein putative expressed
OsISM05632	LOC_Os01g42234	23924073	GGCCTCTACTTCTGGAGCG	CGGTTTACCTGTCCGACAAT	60.0	6230	99	amino acid permease family protein putative expressed
OsISM05633	LOC_Os01g42234	23930271	TGCAGGTTTAACATTGTCGG	AGTGAGAAGTCCACGCTGGT	59.7	132	54	amino acid permease family protein putative expressed
OsISM05634	LOC_Os01g42234	23930541	CACCTGGCTTTCCTTCTTTG	GCACAGCAATCATGAGGACA	60.3	202	75	amino acid permease family protein putative expressed
OsISM05635	LOC_Os01g42234	23930848	TCCTGATGAGCCAGTACACG	CCTGTCAGCATTCTTCGTCTC	59.9	182	75	amino acid permease family protein putative expressed
OsISM05636	LOC_Os01g42234	23931268	TGAGCTCGGTAACAAGCAACT	TTGTTGACCTTGTGCCAGAC	59.9	264	89	amino acid permease family protein putative expressed
OsISM05637	LOC_Os01g42234	23931567	CCCTCATCTCCCTCTGCAT	GACACCATCGCCTGGAAC	60.1	204	65	amino acid permease family protein putative expressed
OsISM05638	LOC_Os01g42250	23933262	GAGATCCCATCTCATCCAGG	GCTCCAGCTCCTCCTCATC	59.7	169	82	expressed protein
OsISM05639	LOC_Os01g42250	23933430	CCGCTCTTCGACTACTCCC	GTGGACGAAGATGTCGGACT	60.0	214	85	expressed protein
OsISM05640	LOC_Os01g42250	23933625	GTCCGACATCTTCGTCCACT	GGCCTTCTGCTCACCCTTAT	60.4	180	98	expressed protein
OsISM05641	LOC_Os01g42250	23933896	CAGAGCAGAGCAGCGTATTG	GATTCAGCAAATTTCGCCAT	60.0	1260	65	expressed protein
OsISM05642	LOC_Os01g42250	23935289	CAGGACAAAGCTGGACATCA	TATGCCCTAAAAAGCTTGCC	59.6	396	69	expressed protein
OsISM05643	LOC_Os01g42310	24000545	AGGAATGCCCTTATGGGTTC	TCCCGACAAAGCAGTAGTCC	60.2	438	89	retrotransposon protein putative unclassified
OsISM05644	LOC_Os01g42310	24000963	GGACTACTGCTTTGTCGGGA	CGGTGTTTCGTCGGTTAGAC	60.4	787	59	retrotransposon protein putative unclassified
OsISM05645	LOC_Os01g42320	24014641	GGACTAAACCATGGTGGTGC	GCAAATATTTCCCACATACCG	59.7	965	71	aspartic proteinase nepenthesin-2 precursor putative
OsISM05646	LOC_Os01g42320	24015664	GCCTTCTTTTGGGTTGTGTG	ATCTGAGGATGACGGAATGG	60.2	1965	65	aspartic proteinase nepenthesin-2 precursor putative

ISM IDs	Rice gene locus IDs	Physical positions (bp) on chromosomes	Forward primers (5'-3')	Reverse primers (5'-3')	Melting tempearature (T˚C)	Expected product size (bp) in PCR	Expected product size (bp) in Q-PCR	Putative Functions
OsISM05647	LOC_Os01g42430	24125842	CAGATCTCGCCCTACACCTT	CTGCTCCCCGTGATGAAG	59.8	211	96	vacuolar ATP synthase putative expressed
OsISM05648	LOC_Os01g42430	24126080	CAGGATCACCTCCAAGAACC	TTGCAACAGCCTCACAGAAG	59.8	1831	51	vacuolar ATP synthase putative expressed
OsISM05649	LOC_Os01g42430	24128003	TATGCAATCTTTGCGTCTGG	AGCATCAGACAATGCACAGC	59.9	176	97	vacuolar ATP synthase putative expressed
OsISM05650	LOC_Os01g42460	24138002	GTGGCCCTGTAATGGAAAAG	ACAGTGCATTTCCCACCAAT	59.8	886	98	BAH domain containing protein expressed
OsISM05651	LOC_Os01g42460	24139007	CTGAGAAAATTGCAGGGGTG	CATGCACTTCCTGGTCTGAA	60.2	200	85	BAH domain containing protein expressed
OsISM05652	LOC_Os01g42460	24139541	TGAAAGACCAAGTCGCACTG	TCACAATCTTGAACCATTTGCT	59.8	872	75	BAH domain containing protein expressed
OsISM05653	LOC_Os01g42460	24140384	GGACAGAAGCAAATGGTTCAA	ACAAGTGTCCCCTGCTCATC	60.1	244	70	BAH domain containing protein expressed
OsISM05654	LOC_Os01g42460	24140637	AAATCTTGACATACAGTTTGC AGC	GGGTGGTTAATAGGCTTAGCG	59.9	1070	91	BAH domain containing protein expressed
OsISM05655	LOC_Os01g42460	24141686	CGCTAAGCCTATTAACCACCC	AGTCTGCGGCACTTTTTGTT	60.0	180	84	BAH domain containing protein expressed
OsISM05656	LOC_Os01g42460	24141882	TAATTCAGGCTTGGTGGTCG	GTGCCCGAGAAGAGTTTCTG	60.5	506	89	BAH domain containing protein expressed
OsISM05657	LOC_Os01g42460	24142368	CAGAAACTCTTCTCGGGCAC	TTGAGAACAATGCGAGGTTG	59.9	182	99	BAH domain containing protein expressed
OsISM05658	LOC_Os01g42460	24142592	GTCCGAGCAAAGCAAGAAAC	TCCCATCTTACTCGCAAAGG	60.1	178	82	BAH domain containing protein expressed
OsISM05659	LOC_Os01g42470	24145122	TACTACCACGACACCATCGC	CTTCCTTCTTCTCCAGGCG	59.8	522	98	acetyltransferase GNAT family putative expressed
OsISM05660	LOC_Os01g42470	24145639	GGAAGGAGGAGCAGTCTGTG	ACATGGTTCAGCAGCTTCG	60.0	1750	91	acetyltransferase GNAT family putative expressed
OsISM05661	LOC_Os01g42520	24180197	GGTGCTCGTTCTGCTCCTC	CTCCTCGAAGTTGCCGTTC	61.0	1273	89	expressed protein
OsISM05662	LOC_Os01g42530	24186295	AACTAATCCCCCACACCTCC	AGTCCACCGTCTGCTGATTC	60.2	203	91	ras-related protein putative expressed
OsISM05663	LOC_Os01g42530	24186491	GTGGACTACCCCAGCTTCAA	ATGCCTCTTCACAAAGGTGG	60.1	583	73	ras-related protein putative expressed
OsISM05664	LOC_Os01g42530	24187079	TGGCGAGTTCGAGAAGAAGT	CAGTAGAAGCGGATCTTGCC	60.1	181	91	ras-related protein putative expressed

ISM IDs	Rice gene locus IDs	Physical positions (bp) on chromosomes	Forward primers (5'-3')	Reverse primers (5'-3')	Melting tempearature (T°C)	Expected product size (bp) in PCR	Expected product size (bp) in Q-PCR	Putative Functions
OsISM05665	LOC_Os01g42530	24187284	ATTTGGTGGACTCAGGGATG	TTCTTGTACGTCAGCCGAGA	59.7	665	85	ras-related protein putative expressed
OsISM05666	LOC_Os01g42530	24187954	CAACTTGGCACCGTGATTTA	GCTTTGCTTTAACCTGCCTG	59.8	174	97	ras-related protein putative expressed
OsISM05667	LOC_Os01g42530	24188199	AAGCCATTCCTGTACCTTGC	GAGCAAGAGCAGGTGTTTCC	59.6	161	74	ras-related protein putative expressed
OsISM05668	LOC_Os01g42530	24188373	AATTGATCTAGCAGCCCAGC	CATCGTCATCATCTGGCAGT	59.6	170	78	ras-related protein putative expressed
OsISM05669	LOC_Os01g42550	24194776	TGAACAGGAAGGAGAGCTGG	GGTGTTTGTGTCAATGTTGAATG	60.4	179	81	expressed protein
OsISM05670	LOC_Os01g42550	24194945	CACAAACACCTTAACTGATCGAC	CAAGCAACTGCTGTCAGGAA	59.4	205	95	expressed protein
OsISM05671	LOC_Os01g42560	24198699	TATGCTACAGTGTGGGTGGC	GAGTGTGCTCCTCATGCAAA	59.8	145	53	retrotransposon protein putative unclassified
OsISM05672	LOC_Os01g42610	24233704	ACTGCTGTCCTCCCTGCAC	TGGTGTTAATCTGCTCCTCAAA	60.6	501	99	terpene synthase putative
OsISM05673	LOC_Os01g42610	24234279	AGGCAACAAGGGTATTGGGT	AGCCCTTCGGGTCATTATTT	60.2	345	95	terpene synthase putative
OsISM05674	LOC_Os01g42610	24234784	GACCTTGAAGAGAGTAGAGGCG	TCGCGGGTGTAGTCTAGGTT	59.7	279	85	terpene synthase putative
OsISM05675	LOC_Os01g42610	24235195	ATTGGAAGAGGCTCGGAAGT	GAAAGAACCTCTTCAGCCCC	60.2	1241	90	terpene synthase putative
OsISM05676	LOC_Os01g42610	24236470	TGAGTTGGAACCACATGAGAA	GATGGAACCACTCAGCTTCC	59.4	323	96	terpene synthase putative
OsISM05677	LOC_Os01g42610	24236965	TTTCATGGATGATTTGGCTG	TTCATGTAACACTCCACGGC	59.5	713	76	terpene synthase putative
OsISM05678	LOC_Os01g42640	24249085	GCAGCTGCCTCAAGACTACC	GATTCTCGCCACCAGCTATC	60.0	172	90	ribosomal protein putative
OsISM05680	LOC_Os01g42650	24254697	GAGGACGTCATGCCCATC	CCTACGGGAGGATCCATGT	59.9	219	93	cytochrome c oxidase subunit 5B mitochondrial precursor putative expressed
OsISM05681	LOC_Os01g42650	24254860	GATCTGTCTGTGGTTTCGCA	AGGATTCAATGACAGCTGGG	60.0	3254	95	cytochrome c oxidase subunit 5B mitochondrial precursor putative expressed
OsISM05682	LOC_Os01g42650	24258130	AGTTGGTTGTCCTGGTGGTG	GTGAACAGACTGGGCACTCA	60.4	189	93	cytochrome c oxidase subunit 5B mitochondrial precursor putative expressed
OsISM05683	LOC_Os01g42650	24258301	AGTGCCCAGTCTGTTCACAA	ACCACCATCACCAATGACCT	59.7	199	51	cytochrome c oxidase subunit 5B mitochondrial precursor putative expressed

ISM IDs	Rice gene locus IDs	Physical positions (bp) on chromosomes	Forward primers (5'-3')	Reverse primers (5'-3')	Melting tempearature (T°C)	Expected product size (bp) in PCR	Expected product size (bp) in Q-PCR	Putative Functions
OsISM05684	LOC_Os01g42660	24263549	GTATGATGGCGGCTTGATCT	TAGTCAGGATGGGAACTGGG	60.0	1735	85	hypothetical protein
OsISM05685	LOC_Os01g42670	24268767	AGAGGGCACGAGTCCATCTA	AAGTCCTATTCGCGCTTGC	60.2	1829	92	hypothetical protein
OsISM05686	LOC_Os01g42670	24270578	CAAGCGCGAATAGGACTTGT	TCGTTAGGTACGGTTCCCTG	60.2	726	57	hypothetical protein
OsISM05687	LOC_Os01g42690	24276151	CTCTCAAGCTTCTTGGTCGC	GTACGTCCTCCCGTACTCCA	60.1	193	89	OsPOP2 - Putative Prolyl Oligopeptidase homologue expressed
OsISM05688	LOC_Os01g42690	24276373	CACCATTGTGTGGCTCCAT	AGGCAGAGGAAGAGAATCCA	59.7	1935	74	OsPOP2 - Putative Prolyl Oligopeptidase homologue expressed
OsISM05689	LOC_Os01g42690	24278275	TGGTCCCAGCTACTGGATTC	GTTGGTGCAGTAGGGCAAAT	60.0	338	63	OsPOP2 - Putative Prolyl Oligopeptidase homologue expressed
OsISM05690	LOC_Os01g42690	24278593	ATTTGCCCTACTGCACCAAC	ACGGAAGTGTCCTCCACATC	60.0	255	84	OsPOP2 - Putative Prolyl Oligopeptidase homologue expressed
OsISM05691	LOC_Os01g42690	24278902	CCTACTGTCATCCGAGCCAT	GCTGAAACCACCTATCCCAA	60.0	174	50	OsPOP2 - Putative Prolyl Oligopeptidase homologue expressed
OsISM05692	LOC_Os01g42690	24279167	TCATCGGTTTGAGCGGTT	CAGGATTGGTAAGGCACCAG	60.4	199	99	OsPOP2 - Putative Prolyl Oligopeptidase homologue expressed
OsISM05693	LOC_Os01g42690	24279353	CTTACCAATCCTGCTCAGCC	CGGCTGATTTCTCACCATTT	60.0	153	69	OsPOP2 - Putative Prolyl Oligopeptidase homologue expressed
OsISM05694	LOC_Os01g42690	24279486	AAATGGTGAGAAATCAGCCG	CAGGGATGGTATAATGCCCC	60.7	178	93	OsPOP2 - Putative Prolyl Oligopeptidase homologue expressed
OsISM05695	LOC_Os01g42710	24295565	CACCACCTTCCCCTTACCTA	GGCAGCTTCCACATACCATT	59.4	1047	55	retrotransposon protein putative unclassified expressed
OsISM05696	LOC_Os01g42710	24296658	TTGTTGCCAGACAATGAACTATG	TCCACAATGCACCTTACCAA	60.0	549	59	retrotransposon protein putative unclassified expressed
OsISM05697	LOC_Os01g42710	24297257	CTCGTTGTGCCTCTTTGTCA	TGTGGGTTGCTCGATTGATA	60.0	838	71	retrotransposon protein putative unclassified expressed
OsISM05698	LOC_Os01g42720	24299281	CTCCCTCCGCTCCCTCTC	CGTAGGTCCCCTTCCCCT	61.8	187	78	expressed protein
OsISM05699	LOC_Os01g42720	24299538	GTGCCTCTGCCCTACCTGT	CAAGCGTAGCCTTTTCCAAG	60.1	189	70	expressed protein
OsISM05700	LOC_Os01g42730	24311991	CCTACGGCCAGACCTTCTTC	AACGGCAGACCAAATGCTT	60.9	940	92	GDSL-like lipase/acylhydrolase putative expressed
OsISM05701	LOC_Os01g42730	24313066	GCAGATGCAGTGGTTCAAGA	CCACAAGGAAAAGTGACTTGG	59.8	940	90	GDSL-like lipase/acylhydrolase putative expressed

ISM IDs	Rice gene locus IDs	Physical positions (bp) on chromosomes	Forward primers (5'-3')	Reverse primers (5'-3')	Melting tempearature (T˚C)	Expected product size (bp) in PCR	Expected product size (bp) in Q-PCR	Putative Functions
OsISM05702	LOC_Os01g42730	24314088	TGTCGGTACCATAACCTCAGC	CAATGGTACACAGCCTATTGGA	59.9	177	95	GDSL-like lipase/acylhydrolase putative expressed
OsISM05703	LOC_Os01g42730	24314447	TATATTCCTTGCCCCACTCC	TTCCACAACACGAGTTCAGC	59.4	1154	57	GDSL-like lipase/acylhydrolase putative expressed
OsISM05704	LOC_Os01g42770	24340520	CATCACCTCCAACTCCATCC	CTTCATCTGCTCCGTGTTCA	60.2	202	68	expressed protein
OsISM05705	LOC_Os01g42770	24340772	TGAGAGAGTCAAGGCCGTCT	GCATACGCCACTTGAACAGA	60.0	1574	86	expressed protein
OsISM05706	LOC_Os01g42810	24363869	TCCGACTACAGGATCTTCCG	GAACTCCACCGAGCAGTTGT	60.3	1913	49	leaf senescence related protein putative expressed
OsISM05707	LOC_Os01g42810	24365885	ACGTCCTCGTCTTCAACACC	GTCCGCCTCCAGATGGTC	60.9	159	75	leaf senescence related protein putative expressed
OsISM05708	LOC_Os01g42810	24366153	GAAGCATCTCCCCAGAACAC	ATTGGGGACGTCTGGTTGTA	59.9	138	56	leaf senescence related protein putative expressed
OsISM05709	LOC_Os01g42830	24375149	GGTCGCTGAGTGCTACGTCT	ATACCTGTAGGAGGCGGCTG	61.1	255	59	adaptin ear-binding coat-associated protein 2 putative expressed
OsISM05710	LOC_Os01g42830	24375360	TTACGCAGATACCTCCCAGG	AAGCCCACTTGTTGACGTTC	60.1	388	70	adaptin ear-binding coat-associated protein 2 putative expressed
OsISM05711	LOC_Os01g42830	24375748	GGGAAGGGACTCTCAAGGTT	GAGGAATGCCCTAGCGTACA	59.9	173	93	adaptin ear-binding coat-associated protein 2 putative expressed
OsISM05712	LOC_Os01g42830	24375911	GGCATTCCTCAGAGAAGACG	TTCTCTTCAACACGGAGTACAAA	59.4	228	82	adaptin ear-binding coat-associated protein 2 putative expressed
OsISM05713	LOC_Os01g42830	24376092	CTCCAACATTGTTTTTGCAGAT	TCACGGAAGCCTAAACCAAT	59.4	278	93	adaptin ear-binding coat-associated protein 2 putative expressed
OsISM05714	LOC_Os01g42830	24376364	CCGTGAAAGACCTCAAGCAT	CCATCTCTTCAGCAGTCTTTTTC	59.9	532	87	adaptin ear-binding coat-associated protein 2 putative expressed
OsISM05715	LOC_Os01g42830	24376928	TACAGCCTCAAAGAAGGGGA	GCAGATTTTATCTTTGCGCC	59.8	1406	72	adaptin ear-binding coat-associated protein 2 putative expressed
OsISM05716	LOC_Os01g42950	24427770	GTGCCCAAGGAGCTCAAG	TTTAACCTCCAGTAGCCCCA	59.5	619	46	protein kinase family protein putative expressed
OsISM05717	LOC_Os01g42950	24428440	CATCTTGCCCATCGAGCTAT	CAAAACCAAACCCACCAAGT	60.0	554	80	protein kinase family protein putative expressed
OsISM05718	LOC_Os01g42950	24429027	CATCAACACAGCAATTCCACT	ACTTCGGACCAATTCTGGTG	59.3	568	99	protein kinase family protein putative expressed
OsISM05719	LOC_Os01g42950	24429670	CCACTTCTGGATTGCCATAAC	CCACCACTAAATCAGCAGGAA	59.8	308	98	protein kinase family protein putative expressed

ISM IDs	Rice gene locus IDs	Physical positions (bp) on chromosomes	Forward primers (5'-3')	Reverse primers (5'-3')	Melting tempearature (T°C)	Expected product size (bp) in PCR	Expected product size (bp) in Q-PCR	Putative Functions
OsISM05720	LOC_Os01g42950	24430000	TGATGTAGCATCACGCCCTA	AGCAAATGGTCAAGGAAACG	60.2	242	97	protein kinase family protein putative expressed
OsISM05721	LOC_Os01g42950	24430188	CTTCTTCCGGAATGATACGC	TGCCTTCAGAAACTCCGTCT	59.8	334	92	protein kinase family protein putative expressed
OsISM05722	LOC_Os01g42950	24430538	AGGACTTCGATTCCCGAGTT	AACCATGGGCAAAATCATTG	60.3	1076	96	protein kinase family protein putative expressed
OsISM05723	LOC_Os01g42950	24431595	AATGATTTTGCCCATGGTTC	GGCAGTGTTGAAAGCTCAAA	59.3	492	70	protein kinase family protein putative expressed
OsISM05724	LOC_Os01g42950	24432125	CACTTCTTTTGCTCGTGAAGC	TAAGCCCGTACCAGCATAGG	60.1	280	95	protein kinase family protein putative expressed
OsISM05725	LOC_Os01g42950	24432449	CAGTGCCATTGTCTCGTCAG	GCATACCGCAGCAACTGTAG	60.0	299	99	protein kinase family protein putative expressed
OsISM05726	LOC_Os01g42950	24432703	CGCGTACATGGATTAGCCTT	CTAAACAGCGCAAGGCATTT	60.3	503	65	protein kinase family protein putative expressed
OsISM05727	LOC_Os01g42950	24433296	CACTTATGTGCACCCTTGGA	GAGCAGTGGGAAGACGAGAG	59.9	214	96	protein kinase family protein putative expressed
OsISM05728	LOC_Os01g42950	24433502	CACTGCTCACAGCACATCAA	AGTCACACCACGCTTCTCCT	59.8	172	99	protein kinase family protein putative expressed
OsISM05729	LOC_Os01g42960	24460804	ACTGCGAGGAGGCAGTCC	TCCTCGCCTTGCTAATCATC	60.9	688	97	TTL1 putative expressed
OsISM05730	LOC_Os01g42960	24461642	CTGCTGGAGCTGACTCCTCT	CGGAGAAGTGCTTCTGACCT	59.7	131	56	TTL1 putative expressed
OsISM05731	LOC_Os01g42960	24461887	CTCTATTGTCCGAGCCCAAG	CATTATCAGCTGCCTCCACA	59.8	160	69	TTL1 putative expressed
OsISM05732	LOC_Os01g42960	24462324	AAGGCGAGCTTCATCCTATG	TCATAGTCCCGCACACAATC	59.5	170	58	TTL1 putative expressed
OsISM05733	LOC_Os01g42960	24462638	ACTCCGTGCTGCCATAGGAT	AAAGGGAGTTCACCGAAGGT	61.0	304	99	TTL1 putative expressed
OsISM05734	LOC_Os01g42960	24462922	ACCTTCGGTGAACTCCCTTT	CGCGACCATAGGGCTATCT	60.1	173	80	TTL1 putative expressed
OsISM05735	LOC_Os01g42980	24480175	AGCTATAGCGGCAGCAGTG	AAAAGCTTGTGCCCTCAAAA	59.6	528	79	ribosomal protein L22 putative expressed
OsISM05736	LOC_Os01g42980	24480698	CTTTTGCATCCAGGGGAGT	TAGCATCATCTGCAGCAAGC	60.2	423	94	ribosomal protein L22 putative expressed
OsISM05737	LOC_Os01g42980	24481203	GAAGGTCCAAGCCATCAAGA	CCTCGAACCAGCTTTGCTAC	60.1	565	73	ribosomal protein L22 putative expressed

ISM IDs	Rice gene locus IDs	Physical positions (bp) on chromosomes	Forward primers (5'-3')	Reverse primers (5'-3')	Melting tempearature (T˚C)	Expected product size (bp) in PCR	Expected product size (bp) in Q-PCR	Putative Functions
OsISM05738	LOC_Os01g42980	24481778	GAAGATGCCCTGTTGCAGTT	GTGATTGTGAGCTGCATTGG	60.3	204	97	ribosomal protein L22 putative expressed
OsISM05739	LOC_Os01g42980	24481962	CCAATGCAGCTCACAATCAC	GTCCTTTCCCAACAAAAGCC	60.6	176	70	ribosomal protein L22 putative expressed
OsISM05740	LOC_Os01g42990	24484567	GAGGATGAGGAGAGCGATGT	CGAGGATGGAGATGTGTTT	59.6	632	92	PPR repeat domain containing protein putative
OsISM05741	LOC_Os01g42990	24485179	AAACACATCTCCCATCCTCG	CCAAGAGGAAGAGGACCCC	60.5	679	97	PPR repeat domain containing protein putative
OsISM05742	LOC_Os01g42990	24486074	CTCTCCTCCACCACTTCTCG	TCTATGCCATTGATCTCCCA	59.5	271	97	PPR repeat domain containing protein putative
OsISM05743	LOC_Os01g42990	24486342	AGACAGATCGGGTTTGGATG	CGAGGCGATTTGCACTAGAT	60.2	110	65	PPR repeat domain containing protein putative
OsISM05745	LOC_Os01g43050	24558886	GATTTCGGCTCAAGACCAAG	GTAGTCCACATTTTGCACCG	59.4	179	54	CENP-C1 putative expressed
OsISM05746	LOC_Os01g43050	24559102	TTTTGACCCTGGAAAAGCTC	TGGTTCAATGCCTCGGTTAT	59.8	179	99	CENP-C1 putative expressed
OsISM05747	LOC_Os01g43050	24559282	CTAAGCTACGACCTGGGCTG	TCAGAGCTGGCGCTAAACTT	60.2	403	65	CENP-C1 putative expressed
OsISM05748	LOC_Os01g43050	24559817	TGCTATACCAGATATCTCAGCGA	ATCCAGATCTTTGAATGCGG	59.5	173	95	CENP-C1 putative expressed
OsISM05749	LOC_Os01g43050	24560488	GAGAAGGGCACATCTTCCAG	CAAATGATCGGGAGGAGAAA	59.9	189	85	CENP-C1 putative expressed
OsISM05750	LOC_Os01g43050	24560736	TCATGGAGATGGCAATTCTG	AGTACCTTGGGCCACATCAC	59.7	182	59	CENP-C1 putative expressed
OsISM05751	LOC_Os01g43050	24560849	TGCGTACGGAACTTTGATTTC	GGGGGTGTTTCCAGTGTATG	60.1	1544	93	CENP-C1 putative expressed
OsISM05752	LOC_Os01g43050	24562388	CCCCCAGAAGATACTCCACA	GGGAGAGTGCGTTGCTTTTA	60.2	364	86	CENP-C1 putative expressed
OsISM05753	LOC_Os01g43050	24562759	AGGGAAGCAACATAGAGGCA	TATCCATTGGGGGTGTTTCT	59.5	678	99	CENP-C1 putative expressed
OsISM05754	LOC_Os01g43050	24563417	AGAAACACCCCCAATGGATA	GGGGAGAGTGCATTGCTTT	59.7	378	97	CENP-C1 putative expressed
OsISM05755	LOC_Os01g43050	24563828	GAAGCAGCCATCGAAGAGAG	ATCTTCTGGGGGTATTTCCG	60.2	651	71	CENP-C1 putative expressed
OsISM05756	LOC_Os01g43050	24564501	CGGAAATTCCTCCAGAAGAT	TGGGGAGAGTGCATTGTTTA	58.7	381	99	CENP-C1 putative expressed

ISM IDs	Rice gene locus IDs	Physical positions (bp) on chromosomes	Forward primers (5'-3')	Reverse primers (5'-3')	Melting tempearature (T˚C)	Expected product size (bp) in PCR	Expected product size (bp) in Q-PCR	Putative Functions
OsISM05757	LOC_Os01g43050	24564888	GGCAAGGAACAAAGAGGAAA	TCAAAGTTTGGTGCGTGTGT	59.7	644	99	CENP-C1 putative expressed
OsISM05758	LOC_Os01g43050	24565542	CAACCTCATGTGCAGGACAC	TACTCGGTGATGGTGACTGG	59.9	438	68	CENP-C1 putative expressed
OsISM05759	LOC_Os01g43050	24566017	GAAGCAAGATTTGAACAGAAGAAA	TTTTGACCGTATTCGTGTGC	59.3	206	89	CENP-C1 putative expressed
OsISM05760	LOC_Os01g43050	24566231	GCATTGGCTTGGTGAAAGAT	TTAACACCGATGACTGTGGC	59.8	960	67	CENP-C1 putative expressed
OsISM05761	LOC_Os01g43070	24572276	GTCTTCGTTCGTGCTCGTC	ACATCTTCATCCATCTCGGC	59.8	421	64	psbP-related thylakoid lumenal protein 4 chloroplast precursor putative expressed
OsISM05762	LOC_Os01g43070	24572756	TGCCAGGAAAGAAATTCACA	CGAGATAGTGGTGCAGCAGA	59.7	173	77	psbP-related thylakoid lumenal protein 4 chloroplast precursor putative expressed
OsISM05763	LOC_Os01g43070	24572904	ATTCCTCTGCTGCACCACTA	CGATACAATACGTTGCCGTG	59.2	170	48	psbP-related thylakoid lumenal protein 4 chloroplast precursor putative expressed
OsISM05764	LOC_Os01g43070	24573072	CGGAGCGAGTTGACATGATA	TCCCACTTGCTCTTGTCCTT	59.8	325	95	psbP-related thylakoid lumenal protein 4 chloroplast precursor putative expressed
OsISM05765	LOC_Os01g43070	24573377	AAGGACAAGAGCAAGTGGGA	AACTCTCTGTCATGCGTTGG	59.4	190	98	psbP-related thylakoid lumenal protein 4 chloroplast precursor putative expressed
OsISM05766	LOC_Os01g43070	24573521	ATCCCATTGCAGAAGGTGAC	TCATAGTACCAGTATGGTTCTCCA	58.8	216	99	psbP-related thylakoid lumenal protein 4 chloroplast precursor putative expressed
OsISM05767	LOC_Os01g43070	24573703	TGTAGGTCGATGGAGAACCA	GGCTACGTTATGGCGAAACA	60.1	253	99	psbP-related thylakoid lumenal protein 4 chloroplast precursor putative expressed
OsISM05768	LOC_Os01g43070	24573919	GGCACCAGAACCAAATCTGT	TGCTTGCTGAGTGTTGAAGC	60.2	183	94	psbP-related thylakoid lumenal protein 4 chloroplast precursor putative expressed
OsISM05769	LOC_Os01g43070	24574082	GCTTCAACACTCAGCAAGCA	GGTGAAAGGATGCCACTGTT	60.2	190	68	psbP-related thylakoid lumenal protein 4 chloroplast precursor putative expressed
OsISM05770	LOC_Os01g43100	24583904	GAGGCTAGGTACATTTGCGG	CAACAAACGGAAAAGCATCA	59.7	269	73	protein phosphatase 2C putative expressed
OsISM05771	LOC_Os01g43100	24584221	ATATGCAGTTGCATCCAGGC	TCCATGTTTGCTCTTCTCCC	60.6	1423	84	protein phosphatase 2C putative expressed
OsISM05772	LOC_Os01g43100	24585681	AACCAAATAAACCTCTTTGGAAT	CTCAGCAGCATGTGAACCAC	58.9	128	55	protein phosphatase 2C putative expressed
OsISM05773	LOC_Os01g43100	24585840	TGAAACATCCGTCATTCATCA	TCTCAGCATCCAAGAAATCAGA	59.9	176	85	protein phosphatase 2C putative expressed
OsISM05774	LOC_Os01g43100	24586102	CGAACTGTGATGTCAAAGGC	CCAGCATTCTCAATCCGTTT	59.7	923	93	protein phosphatase 2C putative expressed

ISM IDs	Rice gene locus IDs	Physical positions (bp) on chromosomes	Forward primers (5'-3')	Reverse primers (5'-3')	Melting tempearature (T˚C)	Expected product size (bp) in PCR	Expected product size (bp) in Q-PCR	Putative Functions
OsISM05775	LOC_Os01g43100	24587005	AAACGGATTGAGAATGCTGG	AAAAGCCCGAGACATTGCTA	60.0	208	82	protein phosphatase 2C putative expressed
OsISM05776	LOC_Os01g43100	24587193	TAGCAATGTCTCGGGCTTTT	CCTCCTCCGCCTTTACAAAT	60.1	940	97	protein phosphatase 2C putative expressed
OsISM05777	LOC_Os01g43120	24597554	GTGGATGGCTCGTACCTCAG	ATACCCAGCATCTTTGACGG	60.3	556	85	DEAD/DEAH box helicase putative expressed
OsISM05778	LOC_Os01g43120	24598129	AGGAGGCAACTCTGCCAGTA	TCCCAGTTCCTGTCTTTGCT	59.9	172	67	DEAD/DEAH box helicase putative expressed
OsISM05779	LOC_Os01g43120	24598281	AGCAAAGACAGGAACTGGGA	GACGTAGTTGATCCCTCCGA	60.0	184	93	DEAD/DEAH box helicase putative expressed
OsISM05780	LOC_Os01g43120	24598561	GGGTGCAAGTTGTAATCGGT	AAGCCTTCCAGGTGTAGCAA	59.9	182	96	DEAD/DEAH box helicase putative expressed
OsISM05781	LOC_Os01g43120	24598870	CCGTTCCTAAAGAGCGACAG	GCAATATGAGAAATTTGGCG	59.1	155	74	DEAD/DEAH box helicase putative expressed
OsISM05782	LOC_Os01g43120	24599065	GGTGATGAGGAGACACATGC	AGATCTAGTGGCGCGATCAT	59.4	146	57	DEAD/DEAH box helicase putative expressed
OsISM05783	LOC_Os01g43120	24599241	GAAGCATGTTGCAGAGGATG	TGGGAAAGAATTTCAGCGAC	59.8	163	88	DEAD/DEAH box helicase putative expressed
OsISM05784	LOC_Os01g43120	24599536	TCCTGATGTCACCCTTGTCA	CCTTCCGTCCAGTTCTACCA	60.1	278	84	DEAD/DEAH box helicase putative expressed
OsISM05785	LOC_Os01g43120	24599896	GGCTACGGTACCTTCTGTTGA	TAGGCACACTCCTTGCTCCT	59.6	221	88	DEAD/DEAH box helicase putative expressed
OsISM05786	LOC_Os01g43130	24601466	GTGGTTGGCTCGCATCTC	TGCTTTCAATGACAACGGAG	60.1	532	67	DEAD/DEAH box helicase putative expressed
OsISM05787	LOC_Os01g43130	24602017	AAAAGATGACCCAGGTGCAG	GTCCCTGTCTTTGCTTTTGC	60.0	181	80	DEAD/DEAH box helicase putative expressed
OsISM05788	LOC_Os01g43130	24602193	GGGACAGGAAAAACTGTTGC	GGGGCATATCACAAGCAAAT	59.7	190	94	DEAD/DEAH box helicase putative expressed
OsISM05789	LOC_Os01g43130	24602463	AATTGGCGGCACAAAATTAC	ATGATCCTTAAGCCTTCCCG	60.1	175	92	DEAD/DEAH box helicase putative expressed
OsISM05790	LOC_Os01g43130	24602756	TCATTCCTAAAGAACGACAGACA	TTGCAATGTGCGAAATTTGA	60.0	151	76	DEAD/DEAH box helicase putative expressed
OsISM05791	LOC_Os01g43130	24602941	AGAGGGTGATGAGGAGACACA	GAAGTGCAGGTCAAGTGGTG	59.5	155	68	DEAD/DEAH box helicase putative expressed
OsISM05792	LOC_Os01g43130	24603122	GCATATCGCAGAAGATGCAG	GCTGGGAAAGAACTTCAGCA	60.0	190	87	DEAD/DEAH box helicase putative expressed

ISM IDs	Rice gene locus IDs	Physical positions (bp) on chromosomes	Forward primers (5'-3')	Reverse primers (5'-3')	Melting tempearature (T˚C)	Expected product size (bp) in PCR	Expected product size (bp) in Q-PCR	Putative Functions
OsISM05793	LOC_Os01g43130	24603442	TCCTGATGTCACCCTTGTCA	TGCCTTTCCGTCCAGTTCTA	60.4	160	87	DEAD/DEAH box helicase putative expressed
OsISM05794	LOC_Os01g43130	24603695	CAATTGATTCGAGCATTCAGA	GGCTGATTCCTTGCTCTTTG	59.4	212	72	DEAD/DEAH box helicase putative expressed
OsISM05795	LOC_Os01g43140	24611168	TCTGCAACATGGCTTACGTC	CGAACCGCAATCCGTAGTAT	59.9	1330	70	lipase putative expressed
OsISM05796	LOC_Os01g43140	24612967	GCAAGCCAACCTCCTCTTC	AGCTGCTCGTAGATGCCCT	60.0	221	97	lipase putative expressed
OsISM05797	LOC_Os01g43140	24613489	CTCCTCAAGCGCCTCAAC	GATGTACGTCGTGCCCATC	59.8	196	88	lipase putative expressed
OsISM05798	LOC_Os01g43160	24628827	AACCTCACCTCCCACATGAC	TATGACGGCAATGGTTCGAT	60.5	481	99	polygalacturonase putative expressed
OsISM05799	LOC_Os01g43160	24629361	ATGGCCTTCAGGATGTTGTC	GGTAGTGTGCCCTTCTTCCA	60.0	257	92	polygalacturonase putative expressed
OsISM05800	LOC_Os01g43160	24629695	CGTTTTGGAACATCCATCCT	GTGTTGGGTGAGTCATGTGG	59.8	657	83	polygalacturonase putative expressed
OsISM05801	LOC_Os01g43160	24630332	CCACATGACTCACCCAACAC	TGTAGCAGTCCTCGATGCAG	60.0	191	71	polygalacturonase putative expressed
OsISM05802	LOC_Os01g43210	24678645	GGAGGGACATTCACAGGGTA	CAAGAGAGACCAAGTTAGAATA TGTGA	59.6	190	98	retrotransposon protein putative Ty1-copia subclass
OsISM05803	LOC_Os01g43210	24678733	CGTTTTTATGAGCCTTTCTTTG A	CCTTCATGATCTCCATGTTCTC	59.2	655	94	retrotransposon protein putative Ty1-copia subclass
OsISM05805	LOC_Os01g43210	24680529	TTTTGGTGGCGATACAGAGC	GAGGATGATCAAGTGCCACA	60.4	448	99	retrotransposon protein putative Ty1-copia subclass
OsISM05806	LOC_Os01g43210	24681057	GAGGAGATTGTGGTGGAAGC	GAACTTGGCCTCAAGTGCAT	60.0	993	65	retrotransposon protein putative Ty1-copia subclass
OsISM05807	LOC_Os01g43210	24682651	GGTGGGTTGATACTGGTGCT	TGACTCGTTTCCCATCAACA	60.0	104	66	retrotransposon protein putative Ty1-copia subclass
OsISM05808	LOC_Os01g43210	24682735	TGTTGATGGGAAACGAGTCA	ATTCAGGACATGACACGCAG	59.9	910	96	retrotransposon protein putative Ty1-copia subclass
OsISM05809	LOC_Os01g43210	24684475	CCTAAACGGAGAGTTGGAGG	CCTTGACCTCCTCAATCACA	58.7	296	72	retrotransposon protein putative Ty1-copia subclass
OsISM05810	LOC_Os01g43220	24692651	CTGGTCAAGATGGGCTGTTT	AGGCAAAGTTTTCCCCAGAT	60.0	1492	92	chitinase domain-containing protein 1 precursor putative expressed
OsISM05811	LOC_Os01g43220	24694388	TTCTCATGGGCATTCTCACA	CCCTTCCAGTGATAGCATTACC	60.0	410	99	chitinase domain-containing protein 1 precursor putative expressed

ISM IDs	Rice gene locus IDs	Physical positions (bp) on chromosomes	Forward primers (5'-3')	Reverse primers (5'-3')	Melting tempearature (T˚C)	Expected product size (bp) in PCR	Expected product size (bp) in Q-PCR	Putative Functions
OsISM05813	LOC_Os01g43240	24698496	GAAAGGATCACCCTGTGCAT	ACGTACTGGTACACCGACCA	59.4	1367	97	hypothetical protein
OsISM05816	LOC_Os01g43340	24773476	TACCACTGGAGCAATCCAAC	AGAAGCGTTGAACCCCTGTA	59.2	469	99	retrotransposon protein putative unclassified
OsISM05817	LOC_Os01g43340	24775040	ATGATCAAGTTCACACGGCA	TCGACCCCTAAGCAGAGTTC	59.8	239	93	retrotransposon protein putative unclassified
OsISM05818	LOC_Os01g43340	24775380	TCGTCTTGGTCATCCTTCCT	CATGATGAGTGATGCCAACTTT	59.8	431	98	retrotransposon protein putative unclassified
OsISM05819	LOC_Os01g43340	24775844	CCGCAGAAAGAAAACATCGT	AGCATTGGGATGAAGTTTGG	60.1	403	85	retrotransposon protein putative unclassified
OsISM05820	LOC_Os01g43340	24776701	GTGGCATCCGAAAAGAAAAA	CATTTCTACCTTGTTGCGGTG	60.3	193	88	retrotransposon protein putative unclassified
OsISM05821	LOC_Os01g43360	24791341	CCTCACCAAGATCCTCTCCA	GATGCGCCTTTTCATCTTTC	60.0	767	95	myosin-Ie putative expressed
OsISM05822	LOC_Os01g43360	24792330	CAGCCTCAAGTGGCTTCTTC	TCAGCAGCAGCATGGTATTC	60.1	427	48	myosin-Ie putative expressed
OsISM05823	LOC_Os01g43360	24792737	GAATACCATGCTGCTGCTGA	GCCTTGTGCGTGATCCTAGT	60.1	374	84	myosin-Ie putative expressed
OsISM05824	LOC_Os01g43410	24838818	GCCACTGACTTCGGTCTTTC	TCAGGTGCAACATAATACGCA	60.0	1001	84	CAMK CAMK like.9 - CAMK includes calcium/calmodulin depedent protein kinases expressed
OsISM05825	LOC_Os01g43410	24839890	CTGGTGTTCCACCTTTTTGG	TATCGAAGGCCAAGGTTCAG	60.3	183	96	CAMK CAMK like.9 - CAMK includes calcium/calmodulin depedent protein kinases expressed
OsISM05826	LOC_Os01g43410	24840123	AGAGCGCCTTACTTCTGCTG	AATGTCAAGTGGCTTGTCTGG	60.0	402	80	CAMK CAMK like.9 - CAMK includes calcium/calmodulin depedent protein kinases expressed
OsISM05827	LOC_Os01g43410	24840540	ATGAAGCAGTTCAGGGCAAT	TTCATCTGACAAGTTCTCTGCAA	59.9	158	73	CAMK CAMK like.9 - CAMK includes calcium/calmodulin depedent protein kinases expressed
OsISM05828	LOC_Os01g43410	24840784	ACCAAAACTTGGCACCAAAA	TGGTTCCATTTCCATCAACA	60.1	696	78	CAMK CAMK like.9 - CAMK includes calcium/calmodulin depedent protein kinases expressed
OsISM05829	LOC_Os01g43410	24841520	CAGATTGGAGAAGGAAGACCA	TTCAGAGCTTCTTCCAACTCATC	59.6	188	97	CAMK CAMK like.9 - CAMK includes calcium/calmodulin depedent protein kinases expressed
OsISM05830	LOC_Os01g43410	24841717	TATGGGGGACGATAAAACGA	ATGGCGACAAACTCCTGGTA	60.3	282	88	CAMK CAMK like.9 - CAMK includes calcium/calmodulin depedent protein kinases expressed
OsISM05831	LOC_Os01g43450	24859532	CAGTAGCGGTGGTGACTGAC	AAGAAGGGGAACGACACCTAA	59.7	186	96	hypothetical protein
OsISM05832	LOC_Os01g43470	24891189	CAGCGATGGGGAGGACTG	CGGAGGAAGAGCAAGAGGA	61.7	258	90	hypothetical protein

ISM IDs	Rice gene locus IDs	Physical positions (bp) on chromosomes	Forward primers (5'-3')	Reverse primers (5'-3')	Melting tempearature (T˚C)	Expected product size (bp) in PCR	Expected product size (bp) in Q-PCR	Putative Functions
OsISM05834	LOC_Os01g43480	24893968	CGACGACTTCCCGTACTACC	AGGATCGTTGGGCTCAGAT	59.6	351	82	AAA-type ATPase family protein putative expressed
OsISM05835	LOC_Os01g43480	24894350	AGCCGCACCATACTGCTCT	CACTGAACTGGTTTGCGAGA	60.5	176	77	AAA-type ATPase family protein putative expressed
OsISM05836	LOC_Os01g43480	24894506	TCTCGCAAACCAGTTCAGTG	GGACCGCCGTACTTATGATG	60.2	175	82	AAA-type ATPase family protein putative expressed
OsISM05837	LOC_Os01g43480	24894660	CCATCATAAGTACGGCGGTC	GCTGCCTCTGTCATGGATCT	60.4	482	61	AAA-type ATPase family protein putative expressed
OsISM05838	LOC_Os01g43480	24895185	CGCAAGAAGGAAGAACCAAC	GTCCTGAGATCAAGGATGCC	59.7	224	66	AAA-type ATPase family protein putative expressed
OsISM05839	LOC_Os01g43480	24895362	AATGTAGGACCATTGAGCCG	CCAAGGATAACTGAACCCGT	59.4	178	98	AAA-type ATPase family protein putative expressed
OsISM05840	LOC_Os01g43480	24895530	TTATCCTTGGTACCTCCCGA	CATTCAAACTCCAGCATGGTT	59.7	323	83	AAA-type ATPase family protein putative expressed
OsISM05841	LOC_Os01g43480	24895834	CCATGCTGGAGTTTGAATGA	TGGACTGATTTCAGAAGCAGAA	59.8	155	76	AAA-type ATPase family protein putative expressed
OsISM05842	LOC_Os01g43480	24896426	CAGAAACGGGAATCTTGTCA	AAGGTCGTTGCTCTCTTGGA	59.3	153	74	AAA-type ATPase family protein putative expressed
OsISM05843	LOC_Os01g43480	24896592	TGGAAGCAAAGGATGAGACA	TAACCGTTGGTCGGATCTTC	59.7	163	70	AAA-type ATPase family protein putative expressed
OsISM05844	LOC_Os01g43480	24897248	TGCGGTGATAAGGAGATTTG	AACCGTCTCTTTCGACAACAA	59.2	190	98	AAA-type ATPase family protein putative expressed
OsISM05845	LOC_Os01g43480	24897450	TTCAAGGAGCTTGCCAAAAT	ACAGGATGATAAGCTGCCGT	59.8	177	81	AAA-type ATPase family protein putative expressed
OsISM05846	LOC_Os01g43480	24897607	ACGGCAGCTTATCATCCTGT	GGTTCTTGCTTCGTTTCTGG	59.8	218	87	AAA-type ATPase family protein putative expressed
OsISM05847	LOC_Os01g43480	24897976	AGACCGCTAAACATGGAGGA	TGATTCATCACAACGCCTTC	59.7	192	81	AAA-type ATPase family protein putative expressed
OsISM05848	LOC_Os01g43500	24903909	GGGAGACTGTGGTGGCTAGA	CCGAGTTTGTTTGACATCCC	60.3	255	82	hypothetical protein
OsISM05849	LOC_Os01g43500	24904065	GGTGGCTGACATGTGGGT	AGTCAAGGTCATCCTGCGAT	60.0	1032	97	hypothetical protein
OsISM05850	LOC_Os01g43500	24905126	CCCGGAATTAAGCACACATT	AAGCCAAGTACTGCAGGAACA	59.9	606	96	hypothetical protein
OsISM05852	LOC_Os01g43530	24932122	ACCTACACCTTCAAGAGCGG	CAGCTCCAGATTTGTCGTCC	60.1	220	94	late embryogenesis abundant protein putative expressed

ISM IDs	Rice gene locus IDs	Physical positions (bp) on chromosomes	Forward primers (5'-3')	Reverse primers (5'-3')	Melting tempearature (T°C)	Expected product size (bp) in PCR	Expected product size (bp) in Q-PCR	Putative Functions
OsISM05853	LOC_Os01g43540	24936849	GCCCATATCAAGCTAGGCAA	TGTGCATTGATGGGTCAAGT	60.1	2082	77	suppressor of G2 allele of SKP1 putative expressed
OsISM05854	LOC_Os01g43540	24938918	CCATCAATGCACAAGGCTTA	GCGAACGAGTAACCCAATTC	59.6	188	99	suppressor of G2 allele of SKP1 putative expressed
OsISM05855	LOC_Os01g43540	24939126	CTCGCCTAATGAAGGAGTGTG	CTGCTCCATCTTCAGCCTTC	60.0	303	79	suppressor of G2 allele of SKP1 putative expressed
OsISM05856	LOC_Os01g43540	24939494	CCAATGGTAGAAGTGAAGCCA	TCTCAGCAGGAACACCCTTT	60.0	345	95	suppressor of G2 allele of SKP1 putative expressed
OsISM05857	LOC_Os01g43540	24939819	AAAGGGTGTTCCTGCTGAGA	TGAAAATGGTACGGCTCCTC	60.0	178	94	suppressor of G2 allele of SKP1 putative expressed
OsISM05858	LOC_Os01g43540	24939977	GAGGAGCCGTACCATTTTCA	CCTTCGTGGATAGCACTTGG	60.4	389	80	suppressor of G2 allele of SKP1 putative expressed
OsISM05859	LOC_Os01g43540	24940423	AAAAACCAAAGGCTGTTCCA	AAGGATATGATGGCCTCTGG	59.3	628	67	suppressor of G2 allele of SKP1 putative expressed
OsISM05860	LOC_Os01g43540	24941036	GGCCATCATATCCTTCCTCA	GCCTTCAAGCTTCTCCTCCT	60.0	188	90	suppressor of G2 allele of SKP1 putative expressed
OsISM05861	LOC_Os01g43540	24941264	ATGCTGATGAAGACATGCGA	CGAGCCAACATCTTTCCAAT	60.2	211	90	suppressor of G2 allele of SKP1 putative expressed
OsISM05863	LOC_Os01g43580	24957578	GGAGCAGCTCATGTTCGAC	ACACGAAGATCTCCCCATCC	60.4	214	99	kinesin motor domain containing protein putative expressed
OsISM05864	LOC_Os01g43580	24957906	CCTTCGACACCGAGCTTATC	ATCATCAAAGCCTCCATTGC	59.9	687	96	kinesin motor domain containing protein putative expressed
OsISM05865	LOC_Os01g43580	24958663	AGGCCTTCCCTGAGAAAGAG	TTCTTATTAAGTGGTCTTTTGCG	58.8	190	77	kinesin motor domain containing protein putative expressed
OsISM05866	LOC_Os01g43580	24958906	CCTTGACTGTTCATGAGACCA A	GGACAGCATCAAACACAAATTC	59.8	202	79	kinesin motor domain containing protein putative expressed
OsISM05867	LOC_Os01g43580	24959086	GAATTTGTGTTTGATGCTGTCC	GCAGGAACAACAGGTTCCAC	60.0	177	81	kinesin motor domain containing protein putative expressed
OsISM05868	LOC_Os01g43580	24959243	GTGGAACCTGTTGTTCCTGC	GGACGCATGGTGTAGGTCTT	60.3	254	93	kinesin motor domain containing protein putative expressed
OsISM05869	LOC_Os01g43580	24959558	GGCTATCAGTTATTCGTGAGC TT	CATCCTCCCTCATGCAAAGT	59.5	187	92	kinesin motor domain containing protein putative expressed
OsISM05870	LOC_Os01g43580	24959999	ACAACGGACAACGACAAACA	AGTGCTCTGATGCATTCCTTT	59.5	201	84	kinesin motor domain containing protein putative expressed
OsISM05871	LOC_Os01g43580	24960295	CATGCATTTCTCCCAGCTCT	CTTGGTGTTGCTTCCTTTCG	60.6	182	96	kinesin motor domain containing protein putative expressed

ISM IDs	Rice gene locus IDs	Physical positions (bp) on chromosomes	Forward primers (5'-3')	Reverse primers (5'-3')	Melting tempearature (T°C)	Expected product size (bp) in PCR	Expected product size (bp) in Q-PCR	Putative Functions
OsISM05872	LOC_Os01g43580	24960878	TGAGCATCTGAACGAACTCCT	CGATGAGCAGACACCAAATC	59.6	171	52	kinesin motor domain containing protein putative expressed
OsISM05873	LOC_Os01g43580	24961050	AAGCAGGTGGAAGAGACCCT	CGTCTAGTTGGTTGCCAGGT	60.2	176	83	kinesin motor domain containing protein putative expressed
OsISM05874	LOC_Os01g43590	24966672	CTTCAAGCACGGCAACTTCT	TTAATCATCGGGAGTACGGC	60.2	100	46	HSF-type DNA-binding domain containing protein expressed
OsISM05875	LOC_Os01g43630	24993692	CGTAGTCGTCTTCTTCACGC	TCAGGTTGGGGATGAAAGAG	59.4	181	74	adaptin putative expressed
OsISM05876	LOC_Os01g43630	24994342	GCTGCGAAGCTGTATCACAT	TTGCAACAGGTAGCAGCATC	59.5	162	77	adaptin putative expressed
OsISM05877	LOC_Os01g43630	24994487	GCTGCTACCTGTTGCAATGA	CTGTGGTTTCAATTACTTTGGG	59.3	2017	91	adaptin putative expressed
OsISM05878	LOC_Os01g43630	24996482	CCCAAAGTAATTGAAACCACAG	CACTAACCAGCCACCTAAAAGG	59.3	1161	84	adaptin putative expressed
OsISM05879	LOC_Os01g43630	24997653	TGAATTTGCAGGTAGTTGCG	TGAGTATTCCCTCCGTCCCT	60.4	561	89	adaptin putative expressed
OsISM05880	LOC_Os01g43630	24998357	TTCTTGAGGACAGACTTCAGCA	CAATGGAGTATGCAAACAATGA	59.4	394	96	adaptin putative expressed
OsISM05881	LOC_Os01g43630	24998882	CAAAAGTTTCTACTGCCAGTTCAG	GAACGAAAGAAGGCCACAAA	59.9	219	96	adaptin putative expressed
OsISM05882	LOC_Os01g43630	24999164	GAGCAGTTGGGAAAATAGCG	CAGGAACAAAGACACCCAGAG	59.8	188	86	adaptin putative expressed
OsISM05883	LOC_Os01g43630	24999454	TATTCAGGAGCCAAAAGGCA	TCAAAAACAAGTAGCTACAGGCA	59.9	598	90	adaptin putative expressed
OsISM05884	LOC_Os01g43630	25000105	CTGACTGCGGTGATGAAATG	TGCAATCAATTCTTGGCAGT	59.8	175	93	adaptin putative expressed
OsISM05885	LOC_Os01g43630	25000359	CCCAAGCAAGCTGTCTCTGT	GCTGCCAAATGCAAAGATAA	59.8	313	98	adaptin putative expressed
OsISM05886	LOC_Os01g43630	25001018	ACAATCCTCTGCACCTCCAC	AGCGCACAACGAAAGAAAAT	60.0	488	95	adaptin putative expressed
OsISM05887	LOC_Os01g43650	25008983	AGGAGGAGGACGACAAGGAT	CAGCTTTCTTCTTGGGCTTG	60.1	992	70	OsWRKY11 - Superfamily of TFs having WRKY and zinc finger domains expressed
OsISM05888	LOC_Os01g43650	25010062	GCCAGAAGGCAGTCAAGAAC	CGTCGTGCACCGGTAGTAG	60.2	219	54	OsWRKY11 - Superfamily of TFs having WRKY and zinc finger domains expressed
OsISM05890	LOC_Os01g43670	25026026	CCGTGGTGGTCTACTTCCAT	AACTCCCATAGCCCCTTCTG	60.1	421	80	hypothetical protein

ISM IDs	Rice gene locus IDs	Physical positions (bp) on chromosomes	Forward primers (5'-3')	Reverse primers (5'-3')	Melting tempearature (T°C)	Expected product size (bp) in PCR	Expected product size (bp) in Q-PCR	Putative Functions
OsISM05891	LOC_Os01g43670	25026475	GATGACCCCCAGATGAACC	TTGTCACGTTGCTTACTGGC	60.0	540	77	hypothetical protein
OsISM05893	LOC_Os01g43700	25040021	GACCTCACCGAGGACCTG	ATTGTAACCCTTGGCATTGG	59.4	369	84	cytochrome P450 72A1 putative expressed
OsISM05894	LOC_Os01g43700	25040580	GGATTTACTGAAAGAATTCGTGA	GAGCATTTTTAGAAGTGTGAAACAA	58.2	736	79	cytochrome P450 72A1 putative expressed
OsISM05895	LOC_Os01g43700	25041631	CCAACTTTCATTGTTTCGGG	TTGCCTTCATCCTTCTGTTG	59.6	161	64	cytochrome P450 72A1 putative expressed
OsISM05896	LOC_Os01g43740	25060107	TTCAGGACATCGTCAAGGAG	GCTCAGCGATGACCACTCTC	60.0	578	76	cytochrome P450 72A1 putative expressed
OsISM05897	LOC_Os01g43740	25060813	AGGATCCTGAACCCTGCTTT	TTACTGTCCCAACGGTCGAT	60.2	204	96	cytochrome P450 72A1 putative expressed
OsISM05898	LOC_Os01g43740	25061162	CGAATCATCAAGACCATCCA	CTCGGCTGTTCTCCTTCATC	59.7	211	89	cytochrome P450 72A1 putative expressed
OsISM05899	LOC_Os01g43740	25061631	CCAGGGAAGAGGTCTTGAGC	CCTGAGCACCTCATGCAGTA	60.7	212	99	cytochrome P450 72A1 putative expressed
OsISM05900	LOC_Os01g43750	25065681	AGAAGTGGGTCAAGCACAGG	TCCTCACAGCATGCAGAAAA	60.4	199	92	cytochrome P450 72A1 putative expressed
OsISM05901	LOC_Os01g43750	25066050	TCCTGAAATGCATGCAGAAG	GATGCATCTTCCGGTTGTTT	59.9	195	73	cytochrome P450 72A1 putative expressed
OsISM05902	LOC_Os01g43750	25066563	CTGAATACGAAGGCCTGAGC	TTCGAGTGAAGGTTACGGCT	59.9	200	91	cytochrome P450 72A1 putative expressed
OsISM05903	LOC_Os01g43760	25070173	AACCCGGCTTTCCATCTC	GACTCCATCCACCTGCTGAT	60.0	242	96	cytochrome P450 72A1 putative expressed
OsISM05904	LOC_Os01g43760	25070558	GCCTCTTGAAATGCGTTCAG	GATGCATCTTCCGGTTGTTT	60.4	218	76	cytochrome P450 72A1 putative expressed
OsISM05905	LOC_Os01g43760	25070890	ACATGAGACACACGGCAGAG	TGGATACAGCCGAAGAACCT	59.8	385	93	cytochrome P450 72A1 putative expressed
OsISM05906	LOC_Os01g43774	25072821	TTCCTGGAATTGAAATTGTCTC	GACGAACGCCCAAACTTTTA	59.1	964	90	cytochrome P450 72A1 putative expressed
OsISM05908	LOC_Os01g43774	25075861	ACGGTGAGAAGTGGGTCAAG	TCACAGCACGTAGAAAACGC	60.1	190	95	cytochrome P450 72A1 putative expressed
OsISM05909	LOC_Os01g43774	25076228	AGGAGCCATTCAGAAGTTCG	ACCTCACTGTTGATCCTCCG	59.8	226	85	cytochrome P450 72A1 putative expressed
OsISM05910	LOC_Os01g43774	25076735	GTCTGTTTGGGAGGGACAAA	CGGGTACAACCGAAGAACCT	60.6	207	90	cytochrome P450 72A1 putative expressed

ISM IDs	Rice gene locus IDs	Physical positions (bp) on chromosomes	Forward primers (5'-3')	Reverse primers (5'-3')	Melting tempearature (T°C)	Expected product size (bp) in PCR	Expected product size (bp) in Q-PCR	Putative Functions
OsISM05912	LOC_Os01g43800	25102269	GATGATCACGTGCGACGA	GGTCTCCAGGCGAAGACG	61.2	153	79	retrotransposon protein putative unclassified
OsISM05913	LOC_Os01g43800	25102856	CAATGCCTTTCGGGTTAAAG	TTTCCGCTAGATCCGAGAGT	59.3	156	65	retrotransposon protein putative unclassified
OsISM05914	LOC_Os01g43844	25113486	CACCAGGCCATGAAGGAG	ACAGCACTTCTCGCACCAG	60.2	2006	98	cytochrome P450 72A1 putative expressed
OsISM05915	LOC_Os01g43844	25115590	CACAGGAGGATCATCAACCC	GTGCAACATGCAGCGAAC	60.2	167	75	cytochrome P450 72A1 putative expressed
OsISM05916	LOC_Os01g43844	25115934	CGTCGCCACCATGAATAAG	ATCTGCTTCATCCTCCGGTT	60.5	224	76	cytochrome P450 72A1 putative expressed
OsISM05917	LOC_Os01g43844	25116447	GAGGTCCTCCACGTCTTCG	CAGCACCTCGTACAACACCA	60.8	176	88	cytochrome P450 72A1 putative expressed
OsISM05918	LOC_Os01g43851	25118982	CTTCCGTTCGAACTCGCC	ATCACTCACGATCACTCGCA	61.2	179	85	cytochrome P450 72A1 putative expressed
OsISM05919	LOC_Os01g43851	25119284	GCTCACCGGAGGATAATGAA	GCTTATCAGCTCGCTGGAAC	60.1	251	91	cytochrome P450 72A1 putative expressed
OsISM05920	LOC_Os01g43851	25119682	CAGGCACGAAACGTTATGAA	TGCTTTCGTCCTTCTGTTGA	59.6	198	88	cytochrome P450 72A1 putative expressed
OsISM05921	LOC_Os01g43851	25120195	CCAGATTTCGAGGGCATAAA	AGCAGGATCGGTGGATACAG	60.1	159	81	cytochrome P450 72A1 putative expressed
OsISM05923	LOC_Os01g43870	25132873	TTGCACTCGATAGAACAGCG	TCAGCGATTCCAAAGTTTCC	60.2	942	95	NLI interacting factor-like phosphatase putative expressed
OsISM05924	LOC_Os01g43870	25133863	GCCACCATCATTTTTGTCCT	TCAGAAAAGAGGACAGTCTCCA	59.4	974	94	NLI interacting factor-like phosphatase putative expressed
OsISM05925	LOC_Os01g43870	25134831	TTCTGACTCAACGTACAGCGA	TCAGCTGAAGGAACTCATTGTT	59.3	227	87	NLI interacting factor-like phosphatase putative expressed
OsISM05926	LOC_Os01g43870	25135438	GACTCACCCGGTCGTCTATC	CACAATGATCCAATGTTGAATGT	59.6	634	98	NLI interacting factor-like phosphatase putative expressed
OsISM05927	LOC_Os01g43870	25136332	GGAGTTGACCTGGCAAAAGT	GGGTCATCAAACCAGCTCTT	59.2	379	99	NLI interacting factor-like phosphatase putative expressed
OsISM05928	LOC_Os01g43920	25168179	GGTCAGACCGGTTCAACAAC	TCCAAATATCATAATCTTCAGCCA	60.1	318	66	retrotransposon protein putative unclassified
OsISM05929	LOC_Os01g43930	25172675	ACCGGTCAGACTGGCTACAG	TCCACACGTTCTCCTTCCTT	60.0	283	95	retrotransposon protein putative unclassified
OsISM05930	LOC_Os01g43930	25173052	TGATGCTTCTACAAGTGCCG	TCAACACAGAATCACCACAAGA	59.6	197	87	retrotransposon protein putative unclassified

ISM IDs	Rice gene locus IDs	Physical positions (bp) on chromosomes	Forward primers (5'-3')	Reverse primers (5'-3')	Melting tempearature (T°C)	Expected product size (bp) in PCR	Expected product size (bp) in Q-PCR	Putative Functions
OsISM05931	LOC_Os01g43930	25174170	AAATCGTGGAGACTTGCGAA	CAGCATCAACAGAACCCTCA	60.3	264	94	retrotransposon protein putative unclassified
OsISM05932	LOC_Os01g43980	25202311	CTGCTCGCTGCTCCACTAC	CAAGTGAAAACGCCAACAGA	59.9	320	93	retrotransposon protein putative unclassified
OsISM05933	LOC_Os01g43980	25202611	TCTGTTGGCGTTTTCACTTG	AGCACAGGATGAGGATGGTT	59.7	1729	83	retrotransposon protein putative unclassified
OsISM05934	LOC_Os01g43980	25204350	GCCCAATGTTCATAGGTGGT	ACTGACGGTGAATACGGGAT	59.5	647	59	retrotransposon protein putative unclassified
OsISM05935	LOC_Os01g43980	25204987	CACCGTCAGTGAGGTTCTCAT	CAGGACCACCATAGTAAGAACG	59.4	212	91	retrotransposon protein putative unclassified
OsISM05936	LOC_Os01g43980	25206031	GAGTCGTGATGAGGTGTCCA	ATGGAAGTTGAGGCGAGATG	59.9	154	98	retrotransposon protein putative unclassified
OsISM05937	LOC_Os01g43980	25206236	GCTGTTGCTAGGGGAGACAC	TGGCCTAGCTCAAAACGAAT	59.9	212	66	retrotransposon protein putative unclassified
OsISM05938	LOC_Os01g43980	25206531	AGGCATTCGGTCCAATACAA	GGCTATCAATGAATGCAGGTG	60.4	246	49	retrotransposon protein putative unclassified
OsISM05939	LOC_Os01g43990	25210112	TTGCGCCTTGATTTAGTCCT	AATACACAGCGCCTTCCTTT	59.4	190	65	hypothetical protein
OsISM05940	LOC_Os01g43990	25210459	ACTCAGAGCTCGGGCTTACA	CAAGAAACAGTCTGCTTTGGC	60.1	201	85	hypothetical protein
OsISM05941	LOC_Os01g43990	25210661	GCAGGTGTTATGAAAGTTGTGC	CGTGGAATTGTGTAGCATGG	59.8	524	95	hypothetical protein
OsISM05942	LOC_Os01g44020	25219475	TCTATGCCACCTTCTACGCC	GGGATATCACATGGGAGGCT	60.7	611	66	hypothetical protein
OsISM05943	LOC_Os01g44040	25226214	GGGAATGGAGGTCTTCGG	CATGCAATGAGAACAGGGAA	60.0	957	76	CAS1 domain-containing protein 1 precursor putative expressed
OsISM05944	LOC_Os01g44040	25227154	CCTGTTCTCATTGCATGGAT	TTGACAGTTCCATTCTCCAAA	58.4	223	99	CAS1 domain-containing protein 1 precursor putative expressed
OsISM05945	LOC_Os01g44040	25227403	AAGGAGGCCTGTCAAAGTCA	CCAGAAGGAAAGATTCATCCA	59.5	184	97	CAS1 domain-containing protein 1 precursor putative expressed
OsISM05946	LOC_Os01g44040	25227578	TCCTTCTGGAAAATCGTGCT	ACCAGAACGATGCCAAATTC	59.9	135	56	CAS1 domain-containing protein 1 precursor putative expressed
OsISM05947	LOC_Os01g44040	25227693	GAATTTGGCATCGTTCTGGT	AAACAGAAACAGATCACGGTTG	59.5	688	94	CAS1 domain-containing protein 1 precursor putative expressed
OsISM05948	LOC_Os01g44040	25228487	CAGAGGAATGGAAAGGTTGG	TAGGCAGCAATGAAAACACG	59.7	1187	98	CAS1 domain-containing protein 1 precursor putative expressed

ISM IDs	Rice gene locus IDs	Physical positions (bp) on chromosomes	Forward primers (5'-3')	Reverse primers (5'-3')	Melting tempearature (T˚C)	Expected product size (bp) in PCR	Expected product size (bp) in Q-PCR	Putative Functions
OsISM05949	LOC_Os01g44040	25229723	GACTTTAGTCTTGCCAGATTTGC	GTCAAGGACAATGCAGCAGA	59.7	186	76	CAS1 domain-containing protein 1 precursor putative expressed
OsISM05950	LOC_Os01g44040	25230054	TGATATGGGAAATTCCTGGTG	TTTGCTTTTGAAGGCTCTGG	60.1	180	89	CAS1 domain-containing protein 1 precursor putative expressed
OsISM05951	LOC_Os01g44040	25230262	CGATCTGGCCTTGATCGTTA	TTCTTCAAGCTTTTCCATCCA	60.5	266	91	CAS1 domain-containing protein 1 precursor putative expressed
OsISM05952	LOC_Os01g44040	25230557	GGGAACCATCATTAGTATTTCCTT	TGTGTACGGATGGTACTTGTTG	58.6	202	98	CAS1 domain-containing protein 1 precursor putative expressed
OsISM05953	LOC_Os01g44040	25230746	CCATCCGTACACATCATGGA	AGCTGCTGAGTGCAATTCCT	60.2	143	64	CAS1 domain-containing protein 1 precursor putative expressed
OsISM05954	LOC_Os01g44040	25230876	GCACTCAGCAGCTTAGAAACG	TGAGCCAAATGTGGATCTGA	60.3	193	94	CAS1 domain-containing protein 1 precursor putative expressed
OsISM05955	LOC_Os01g44040	25231003	TTACCAATAGGTGGCTTGGC	CTGCCCGTTAGGCATGTTA	59.8	1026	87	CAS1 domain-containing protein 1 precursor putative expressed
OsISM05956	LOC_Os01g44040	25232055	GGGTTACCCACTGCTCAACT	TCAACACACCTGTCAGCTCAA	59.8	175	84	CAS1 domain-containing protein 1 precursor putative expressed
OsISM05957	LOC_Os01g44050	25234371	CTGCTCTACGTCGGCAAGAC	GCGAAGCTTAGTAGCAGCTCA	60.6	190	75	siroheme synthase putative expressed
OsISM05958	LOC_Os01g44050	25234545	TGCTACTAAGCTTCGCAGAGG	ATCCATCTCTTCTCCACCCC	60.1	920	90	siroheme synthase putative expressed
OsISM05959	LOC_Os01g44050	25235445	GGGGTGGAGAAGAGATGGAT	TGCAATTCCTGAAGCTGATG	60.1	169	84	siroheme synthase putative expressed
OsISM05960	LOC_Os01g44050	25235621	TTGGCATTCCACTAACCCAT	GATCTGGCCCACCATTTCTA	60.0	530	79	siroheme synthase putative expressed
OsISM05961	LOC_Os01g44050	25236243	ATGAAGCATGGTCTTCCACC	CAAAATCCTTTAACATGGCGA	59.9	173	95	siroheme synthase putative expressed
OsISM05962	LOC_Os01g44069	25256958	CACCCCTGCTTCCCATACT	TCTTGGTGCTCTCCCTGTTC	60.2	4958	56	glycerol-3-phosphate acyltransferase putative expressed
OsISM05963	LOC_Os01g44090	25270737	ACTTGGGCCTCCTTGTTCTT	CTGCTTCTGCTCCGTGGT	60.1	393	64	X8 domain containing protein expressed
OsISM05964	LOC_Os01g44090	25271484	GCTACTACCAGAAGAACCCCG	CTGAATACTGGCACGACCCT	60.1	187	97	X8 domain containing protein expressed
OsISM05965	LOC_Os01g44090	25271647	GTTCAGGGTCGTGCCAGTAT	GCAGCATGTTCTGACCACC	60.1	170	52	X8 domain containing protein expressed
OsISM05966	LOC_Os01g44110	25282286	TTGTGTATCGTGGCCGTCTA	CGACCTCCACTCTGAACTCC	60.0	192	97	serine/threonine-protein kinase putative expressed

ISM IDs	Rice gene locus IDs	Physical positions (bp) on chromosomes	Forward primers (5'-3')	Reverse primers (5'-3')	Melting tempearature (T˚C)	Expected product size (bp) in PCR	Expected product size (bp) in Q-PCR	Putative Functions
OsISM05967	LOC_Os01g44110	25282502	AGAATTTGGTTCGGCTGTTG	ATGAAGCCACTGCTCCAAGT	60.0	1344	96	serine/threonine-protein kinase putative expressed
OsISM05968	LOC_Os01g44110	25283861	CATGGTGTTTTTAGCTGGGAA	CGGTGCACAACTTTTGGTT	59.8	196	99	serine/threonine-protein kinase putative expressed
OsISM05969	LOC_Os01g44110	25284135	GGGGTCTGATAAAAGCCACA	AACATGCCCGTGTTAGCATA	59.5	186	82	serine/threonine-protein kinase putative expressed
OsISM05970	LOC_Os01g44110	25284387	TGTTGATTACAGCCGCTCTG	CGGTTTGCCACCATTATTTT	59.9	296	67	serine/threonine-protein kinase putative expressed
OsISM05971	LOC_Os01g44110	25284800	GATGGGCCAAGTTGTTAGGA	CGATATCCATGCTTCCTGTG	59.5	172	93	serine/threonine-protein kinase putative expressed
OsISM05972	LOC_Os01g44130	25290739	CGCAGAATTTTACGGTGGTC	TTCCGATGGAAGTAACAGGC	60.3	207	95	aspartic proteinase oryzasin-1 precursor putative expressed
OsISM05973	LOC_Os01g44130	25290909	TGTTGTGGGAACAGCTTGC	TCCATAGTGAATGGAAGCAGG	60.5	602	99	aspartic proteinase oryzasin-1 precursor putative expressed
OsISM05974	LOC_Os01g44130	25291550	ACCATTGGTGATCTAGTTGTC A	GTCCAAGGATGCCATCAAAT	58.4	192	98	aspartic proteinase oryzasin-1 precursor putative expressed
OsISM05975	LOC_Os01g44130	25291761	AGTTGAAGGTGCAGACCCAG	CGTTTGCATTTCGATTTAACC	59.9	635	93	aspartic proteinase oryzasin-1 precursor putative expressed
OsISM05976	LOC_Os01g44130	25292433	CGAGAGCCATTACAAAGGTGA	GTGCTCCTGCCTCCAATAAG	60.0	227	97	aspartic proteinase oryzasin-1 precursor putative expressed
OsISM05977	LOC_Os01g44130	25292602	CATCATCGGTAAACGTGCAG	AGCACAACCGTCAACACAAA	60.2	172	81	aspartic proteinase oryzasin-1 precursor putative expressed
OsISM05978	LOC_Os01g44130	25292792	ACTTCGTTGATTGCTGGTCC	TGTTTGCACTCTTCGTTTGC	60.1	268	87	aspartic proteinase oryzasin-1 precursor putative expressed
OsISM05979	LOC_Os01g44130	25293040	GCAAACGAAGAGTGCAAACA	GAACACACTTGTGCTGGTGG	60.1	226	90	aspartic proteinase oryzasin-1 precursor putative expressed
OsISM05980	LOC_Os01g44130	25293266	CAAAATTGGGCTCTGCAAAT	CTCACCCAGCACACTCTCAA	60.0	179	68	aspartic proteinase oryzasin-1 precursor putative expressed
OsISM05981	LOC_Os01g44130	25293537	TACAAAGGAGGGCAAGTTGG	GCAAGTGACCGATGTGTCTG	60.2	170	96	aspartic proteinase oryzasin-1 precursor putative expressed
OsISM05982	LOC_Os01g44130	25293719	TCAGCATAGGTGGAAGAGCA	AATGAGCCAGGAATCCCTCT	59.8	2164	79	aspartic proteinase oryzasin-1 precursor putative expressed
OsISM05983	LOC_Os01g44130	25295863	AGAGGGATTCCTGGCTCATT	CCCCATAAACACATCGCCTA	60.4	1106	95	aspartic proteinase oryzasin-1 precursor putative expressed
OsISM05984	LOC_Os01g44150	25303550	ATGGTTGATGGTTCAATGGC	GGTGCAGGAGCTGATACTGG	60.7	170	69	retrotransposon protein putative Ty3-gypsy subclass

ISM IDs	Rice gene locus IDs	Physical positions (bp) on chromosomes	Forward primers (5'-3')	Reverse primers (5'-3')	Melting tempearature (T˚C)	Expected product size (bp) in PCR	Expected product size (bp) in Q-PCR	Putative Functions
OsISM05985	LOC_Os01g44150	25303735	GGCTCCAGGCCAATTAGTTA	GCTGGGTTGGATCAACAGTA	58.9	140	81	retrotransposon protein putative Ty3-gypsy subclass
OsISM05986	LOC_Os01g44150	25304126	CCAGAGCACTTGGTTCATCA	CTCTCTCAAACCATTCGGGA	60.0	355	92	retrotransposon protein putative Ty3-gypsy subclass
OsISM05987	LOC_Os01g44150	25305443	GGAGGGTGTGCAAGAGGTT	TGATTTCCTGTTCGACCTCC	60.1	416	83	retrotransposon protein putative Ty3-gypsy subclass
OsISM05988	LOC_Os01g44150	25306570	CTGGAAAAGTTTGGGATGGA	CACGACGCCCTCAAAATAAT	59.9	99	66	retrotransposon protein putative Ty3-gypsy subclass
OsISM05989	LOC_Os01g44150	25306978	GCCGATATGCTTGAACCTGT	TGGATATCTTCTTCGGCCAT	59.8	293	87	retrotransposon protein putative Ty3-gypsy subclass
OsISM05990	LOC_Os01g44150	25307645	AATTGCAAGAGATGATCGGC	TTCATTGGACAATAGATAATGCCT	59.6	403	90	retrotransposon protein putative Ty3-gypsy subclass
OsISM05991	LOC_Os01g44170	25316103	TATGCTTGGAATGAAAGGGG	TAACTTCCTCGTCTCCAGCG	60.2	231	96	conserved hypothetical protein
OsISM05992	LOC_Os01g44170	25316344	CAATGACATTCATCGTCGCT	GAGAGCACAGTTTGTTGGCA	59.9	4122	93	conserved hypothetical protein
OsISM05993	LOC_Os01g44170	25320446	TGCCAACAAACTGTGCTCTC	CACTCCTTACATATGATTCAGCCA	60.2	726	87	conserved hypothetical protein
OsISM05994	LOC_Os01g44170	25321136	TTTGCAGGTACTTGGCTGAA	TCGCTTTGGAAATCAAAACA	59.4	164	95	conserved hypothetical protein
OsISM05995	LOC_Os01g44170	25322018	CTTAAATTGCTGCGTCCCAT	CATCTGATGCCTCATCTGGTAA	60.1	133	97	conserved hypothetical protein
OsISM05996	LOC_Os01g44170	25322189	GGGTGCAGAGGTAGAAGCTG	GCATTCACTCCCACAGTTCA	59.8	112	50	conserved hypothetical protein
OsISM05997	LOC_Os01g44200	25335534	GGTTGTTCAGAATGGTTGGG	CGAATGCAATACGCTCTTCA	60.1	1871	75	retrotransposon protein putative unclassified
OsISM05998	LOC_Os01g44200	25337807	GCGGATCAAATTCTGAATCAA	TGTTGCTTGCAAAACGGTAG	60.0	142	75	retrotransposon protein putative unclassified
OsISM05999	LOC_Os01g44200	25338056	ACATGACCAACACTCACCCA	TGATGAACAGCTTGGACAGG	59.8	160	70	retrotransposon protein putative unclassified
OsISM06000	LOC_Os01g44200	25338216	GGATGTTCCAAGTTCCGATT	ATGTCTGCGTCAGTTCGACA	59.7	212	99	retrotransposon protein putative unclassified
OsISM06001	LOC_Os01g44200	25338403	GGTAATGTCGAACTGACGCA	TCCAAGCGATATGACTCGTAA	59.1	187	93	retrotransposon protein putative unclassified
OsISM06002	LOC_Os01g44200	25339505	AGAAAACACTTTTGGCGAGC	TGCATGACCTGTTATCCACC	59.4	197	90	retrotransposon protein putative unclassified

ISM IDs	Rice gene locus IDs	Physical positions (bp) on chromosomes	Forward primers (5'-3')	Reverse primers (5'-3')	Melting tempearature (T˚C)	Expected product size (bp) in PCR	Expected product size (bp) in Q-PCR	Putative Functions
OsISM06003	LOC_Os01g44200	25339696	CATGCAAGCGGAAGAAAGAT	CAGAATTGATCACGTTGACGA	60.0	330	85	retrotransposon protein putative unclassified
OsISM06004	LOC_Os01g44200	25340489	AAAGCATATGTTGCATGGGC	TGCATGACCTGTTATCCACC	60.1	310	90	retrotransposon protein putative unclassified
OsISM06005	LOC_Os01g44200	25340793	CATGCAAGCGGAAGAAAGAT	CAGAATTGATCACGTTGACGA	60.0	330	85	retrotransposon protein putative unclassified
OsISM06006	LOC_Os01g44200	25341586	AAAGCATATGTTGCATGGGC	TGCATGACCTGTTATCCACC	60.1	308	90	retrotransposon protein putative unclassified
OsISM06007	LOC_Os01g44200	25341888	CATGCAAGCGGAAGAAAGAT	CAGAATTGATCACGTTGACGA	60.0	330	85	retrotransposon protein putative unclassified
OsISM06008	LOC_Os01g44200	25342681	AAAGCATATGTTGCATGGGC	ATTTTTACATGCCACCCGTC	60.3	169	91	retrotransposon protein putative unclassified
OsISM06009	LOC_Os01g44200	25343754	TCCATGGGAAAGGATATCTCA	TAATCGGTGTGTCGTTGGAA	59.6	165	77	retrotransposon protein putative unclassified
OsISM06010	LOC_Os01g44220	25356689	GGTGCTTTGGTGATGGAGTT	TCTTTCGGGAGGATTGTGTC	60.0	491	97	glucose-1-phosphate adenylyltransferase large subunit chloroplast precursor putative expressed
OsISM06011	LOC_Os01g44220	25357202	TTCTGCTGTCATTTTGGGTG	GCCTGTAACATCCTCCAACAG	59.6	703	96	glucose-1-phosphate adenylyltransferase large subunit chloroplast precursor putative expressed
OsISM06012	LOC_Os01g44220	25358003	CATACATACCTTGGTGGGGG	TCAGGCATTTGTGTAGCAGC	60.0	171	72	glucose-1-phosphate adenylyltransferase large subunit chloroplast precursor putative expressed
OsISM06013	LOC_Os01g44220	25358186	ATGGTTCCAGGGTACAGCAG	CGTGCTCAATGTTGTTTTGA	59.2	187	81	glucose-1-phosphate adenylyltransferase large subunit chloroplast precursor putative expressed
OsISM06014	LOC_Os01g44220	25358384	TGTGGAGATCAGCTTTACAGGA	GTCGATGGGAGCACATGATA	59.7	176	94	glucose-1-phosphate adenylyltransferase large subunit chloroplast precursor putative expressed
OsISM06015	LOC_Os01g44220	25358538	CATATCATGTGCTCCCATCG	AAGAAATTGGATCACACGGC	59.9	180	86	glucose-1-phosphate adenylyltransferase large subunit chloroplast precursor putative expressed
OsISM06016	LOC_Os01g44220	25358698	GCCGTGTGATCCAATTTCTT	GCATAGCTGAGGAAGCTGGT	59.8	208	80	glucose-1-phosphate adenylyltransferase large subunit chloroplast precursor putative expressed
OsISM06017	LOC_Os01g44220	25358929	CCTACATTGCATCAATGGGAA	TCCAAAGTCTTGCAGGTGTG	60.3	158	90	glucose-1-phosphate adenylyltransferase large subunit chloroplast precursor putative expressed
OsISM06018	LOC_Os01g44220	25359082	TTGGATCAGAAATTCTCCCG	GATTGTTCCAATATCCTCCCAA	60.0	408	87	glucose-1-phosphate adenylyltransferase large subunit chloroplast precursor putative expressed
OsISM06019	LOC_Os01g44220	25359498	TTTTGATGCTAACTTGGCCC	CGAGGTGAGGTGAAGAAAGG	60.0	265	82	glucose-1-phosphate adenylyltransferase large subunit chloroplast precursor putative expressed
OsISM06020	LOC_Os01g44220	25359743	CCTTTCTTCACCTCACCTCG	GGAACAGCCATCGGAGATTA	59.9	166	85	glucose-1-phosphate adenylyltransferase large subunit chloroplast precursor putative expressed

ISM IDs	Rice gene locus IDs	Physical positions (bp) on chromosomes	Forward primers (5'-3')	Reverse primers (5'-3')	Melting tempearature (T˚C)	Expected product size (bp) in PCR	Expected product size (bp) in Q-PCR	Putative Functions
OsISM06021	LOC_Os01g44220	25359918	TGCACCATTGAGCATTCTGT	TTGATCTGCACCCATCATCA	60.7	233	88	glucose-1-phosphate adenylyltransferase large subunit chloroplast precursor putative expressed
OsISM06022	LOC_Os01g44220	25360197	TCCCAATTGGTATAGGCGAG	ATCACGTTCCTGCCAATTCT	59.7	174	77	glucose-1-phosphate adenylyltransferase large subunit chloroplast precursor putative expressed
OsISM06023	LOC_Os01g44220	25360351	AGAATTGGCAGGAACGTGAT	CCCTCTTCAGGGTGATCAGA	59.9	160	66	glucose-1-phosphate adenylyltransferase large subunit chloroplast precursor putative expressed
OsISM06024	LOC_Os01g44230	25363474	GAGAAGATGATGGGGCAAAG	CTAGCTGCTGCATCTTGCG	60.3	211	71	transcription factor X1 putative expressed
OsISM06025	LOC_Os01g44230	25363797	AAAAAGTGATTATGACCGAAGGA	CAGCTCTCTCTTGTTCCAACG	59.6	340	99	transcription factor X1 putative expressed
OsISM06026	LOC_Os01g44230	25364131	GAGCTGATGAAAATGTGCTGA	TGCATCCACCTGCTGTTCTA	59.7	193	96	transcription factor X1 putative expressed
OsISM06027	LOC_Os01g44230	25364523	AAATGCAAGATGCCAGGAAG	TCGCCCATTCTCTTTATTCC	59.7	222	86	transcription factor X1 putative expressed
OsISM06028	LOC_Os01g44230	25364859	TTGGCACCCTTTTAGGTTTG	TCTTCGCCATGCTCTTCTTT	60.0	602	97	transcription factor X1 putative expressed
OsISM06029	LOC_Os01g44250	25367560	TCAAGTCGTTCCTGGACCAT	GAGCCATCGAGCCTGATCT	60.5	192	92	CBS domain containing membrane protein putative expressed
OsISM06030	LOC_Os01g44250	25367844	ATCGCGACATAGGATTCGTC	CGGAGTTTTTGTCACCTGCT	60.2	272	94	CBS domain containing membrane protein putative expressed
OsISM06031	LOC_Os01g44250	25368136	CAGCATCCTCAGATTGCAGA	TCCCATAGGAACGACTTTGC	60.1	229	57	CBS domain containing membrane protein putative expressed
OsISM06032	LOC_Os01g44250	25368427	ACCGCAGGATCAATGTGGTA	CTTGAACTGAGAAGCAATTCCA	60.4	185	98	CBS domain containing membrane protein putative expressed
OsISM06033	LOC_Os01g44250	25368611	GTGGTCTTGAGTGGCTCGAT	ACAGGCTTGGTCGTATTTGC	60.2	674	74	CBS domain containing membrane protein putative expressed
OsISM06034	LOC_Os01g44250	25369385	GGATCAATACAGTGCAGCGA	AGTTTGACGAATTCTTCCGC	59.6	183	93	CBS domain containing membrane protein putative expressed
OsISM06035	LOC_Os01g44260	25382308	CTCCTACGACCACGACGACT	CCAATGACTTGGACACGAAG	59.7	476	87	dihydroflavonol-4-reductase putative expressed
OsISM06036	LOC_Os01g44360	25447453	AGGCAGAAGAACGAGTGCAG	CCATGGAAGCGTAGTGCTTT	60.5	401	91	CBS domain containing membrane protein putative expressed
OsISM06037	LOC_Os01g44360	25448038	TGCTGCACCAGTCATTTTGT	CAGACTTGAGCACCACCAGA	60.2	153	80	CBS domain containing membrane protein putative expressed
OsISM06038	LOC_Os01g44360	25448184	AAGTCTGGGGATGAGAAGCA	CTTGTTTCTTCTGAAGGCCG	59.9	849	93	CBS domain containing membrane protein putative expressed

ISM IDs	Rice gene locus IDs	Physical positions (bp) on chromosomes	Forward primers (5'-3')	Reverse primers (5'-3')	Melting tempearature (T˚C)	Expected product size (bp) in PCR	Expected product size (bp) in Q-PCR	Putative Functions
OsISM06039	LOC_Os01g44360	25449013	CGGCCTTCAGAAGAAACAAG	CATTGCCTGCAGAATATTGG	59.6	188	91	CBS domain containing membrane protein putative expressed
OsISM06040	LOC_Os01g44360	25449189	CTGCAGGCAATGGAGCTAAT	AAAAACAGGAACATGGCGAA	60.4	167	52	CBS domain containing membrane protein putative expressed
OsISM06041	LOC_Os01g44390	25461202	GTCAAGCGCATCGAGACC	CGTTCAGATCGAGGTGCTTT	60.5	1212	89	MYB family transcription factor putative expressed
OsISM06042	LOC_Os01g44394	25465450	ACAACAACCCCAACAAGTCC	CTGAAGCTGAGGCTGGAAAC	59.9	191	68	mitotic checkpoint protein putative expressed
OsISM06043	LOC_Os01g44394	25465622	TTTCCAGCCTCAGCTTCAGT	CTGGATCTCCCAGCAACG	60.2	206	81	mitotic checkpoint protein putative expressed
OsISM06044	LOC_Os01g44394	25465805	AGGTTCGTTGCTGGGAGAT	ATCATCCTTCCATGCCGAG	60.3	213	96	mitotic checkpoint protein putative expressed
OsISM06045	LOC_Os01g44394	25466154	TTGTTAGTGGGAGCTGGGAC	ACAGCGCTCAGGTAGTTGCT	60.2	381	87	mitotic checkpoint protein putative expressed
OsISM06046	LOC_Os01g44394	25466571	GGCACTGCTGATCGAAATGT	GCAAGACACCTTGTCTGGAG	59.8	841	99	mitotic checkpoint protein putative expressed
OsISM06047	LOC_Os01g44394	25467402	GGTGTCTTGCTGCATTTCCT	TGATGAACACCAACTCGTCC	59.9	849	71	mitotic checkpoint protein putative expressed
OsISM06048	LOC_Os01g44394	25468286	TTAAATGTCACCGTGATGGC	CCGAAGTGGCAAAAGTATGG	59.9	395	82	mitotic checkpoint protein putative expressed
OsISM06049	LOC_Os01g44394	25468688	TGGAGGTTTCAACTTTTGGG	TGATGGGTGACGGACACTTA	60.0	2689	72	mitotic checkpoint protein putative expressed
OsISM06050	LOC_Os01g44394	25471357	TAAGTGTCCGTCACCCATCA	ACCCTTGCTCCAATCATAGC	59.6	1159	86	mitotic checkpoint protein putative expressed
OsISM06051	LOC_Os01g44394	25472516	GCCGAGAAGCACAATCCTT	ATTAACACGGGGCTTTCCTT	60.1	370	88	mitotic checkpoint protein putative expressed
OsISM06052	LOC_Os01g44970	25513404	AACGTGGTTGATTTTGGAGC	CTGTAGATGCTCCCTGCTTG	59.3	1239	98	polygalacturonase putative expressed
OsISM06053	LOC_Os01g44970	25514666	TTTCTTGTTGGGCCAATCTC	TTAGCACTGGTTGGGGCTAC	60.1	527	93	polygalacturonase putative expressed
OsISM06054	LOC_Os01g44970	25515281	GGACAACAATGGTGGACCTA	GTGGCATCCTTTCAAACTCC	58.9	173	83	polygalacturonase putative expressed
OsISM06055	LOC_Os01g44970	25515435	GAGTTTGAAAGGATGCCACAA	CACTGCGAGCTGTTGACAAT	60.1	186	99	polygalacturonase putative expressed
OsISM06056	LOC_Os01g44970	25515687	CAGCCTCAACACTGATGGAA	TTTGAATGGAGACGCAGTCA	60.1	411	96	polygalacturonase putative expressed

ISM IDs	Rice gene locus IDs	Physical positions (bp) on chromosomes	Forward primers (5'-3')	Reverse primers (5'-3')	Melting tempearature (T˚C)	Expected product size (bp) in PCR	Expected product size (bp) in Q-PCR	Putative Functions
OsISM06057	LOC_Os01g44970	25516224	TGTTCAGAACCATGACTGGTG	ACTTGGATGTTCGAGAACCG	59.8	290	86	polygalacturonase putative expressed
OsISM06058	LOC_Os01g45000	25531422	TGGAGTGTACATGGCAAGAA	ATCTACCGCTCCTGCATCAC	59.0	210	90	hypothetical protein
OsISM06059	LOC_Os01g45010	25533726	GCGGAGTTGAATTCTGAACC	GTCGTAGTTTCCAACGTCGC	60.2	3574	64	hypothetical protein
OsISM06060	LOC_Os01g45010	25537790	AACACGCGCGTCAATCTC	TGTAGGTACCAGCTGAGCCA	60.2	1007	93	hypothetical protein
OsISM06061	LOC_Os01g45030	25554298	GAACTCGTTCATGCAGACCC	CACCATCTGGAGCTTGTTGT	59.7	122	89	retrotransposon protein putative Ty3-gypsy subclass
OsISM06062	LOC_Os01g45030	25554527	TCTCAGGGATTTCAAGGTGG	AAAATGCTTCTCCAAATCGG	59.6	1219	68	retrotransposon protein putative Ty3-gypsy subclass
OsISM06063	LOC_Os01g45030	25556257	ATACAATCCCATCGGCTGAA	CTGTTCGACCAGCTGTATGC	59.9	213	97	retrotransposon protein putative Ty3-gypsy subclass
OsISM06064	LOC_Os01g45030	25556706	AGAGGCAATCAGCTAAGGCA	CTTAACACGCCACTCCTGCT	60.3	240	85	retrotransposon protein putative Ty3-gypsy subclass
OsISM06065	LOC_Os01g45030	25557496	GGCAAGGCGACAAGATTAAA	AGTTTGAACCCTCCACGATG	60.1	211	84	retrotransposon protein putative Ty3-gypsy subclass
OsISM06066	LOC_Os01g45030	25558542	CCAGGAAATATGGCCTGAAG	ACTTGACAACATCGGCTTTG	59.1	594	94	retrotransposon protein putative Ty3-gypsy subclass
OsISM06067	LOC_Os01g45030	25559582	TGCCAAGAGCTAATGAAGGAA	TAACGCAATTTCCGAGAAGC	60.2	222	63	retrotransposon protein putative Ty3-gypsy subclass
OsISM06068	LOC_Os01g45030	25560748	ACAACAAGAAAGTGGTGCCC	TTCTACCTCCCCTCCCTGTT	60.0	485	75	retrotransposon protein putative Ty3-gypsy subclass
OsISM06069	LOC_Os01g45060	25578829	GACGACGCTAGTCAAGTGGC	GTCGGCCAAAATACTGTGCT	60.6	1246	98	polygalacturonase putative
OsISM06071	LOC_Os01g45060	25581161	TCAGACACAGATGACGACGA	GGCATCCTTTCAAACTCCAC	59.2	150	60	polygalacturonase putative
OsISM06072	LOC_Os01g45060	25581294	GAGTTTGAAAGGATGCCACAA	CACTGCGAGCTGTTGACAAT	60.1	186	99	polygalacturonase putative
OsISM06073	LOC_Os01g45060	25581546	CAGCCTCAACACTGATGGAA	TTTGAATGGAGACGCAGTCA	60.1	411	96	polygalacturonase putative
OsISM06074	LOC_Os01g45060	25582083	TGTTCAGAACCATGACTGGTG	ACTTGGATGTTCGAGAACCG	59.8	290	86	polygalacturonase putative
OsISM06075	LOC_Os01g45080	25589554	CACGGACACAAGATACTGGG	TCATTGACAGTTGATTGACGTG	59.3	132	92	hypothetical protein

ISM IDs	Rice gene locus IDs	Physical positions (bp) on chromosomes	Forward primers (5'-3')	Reverse primers (5'-3')	Melting tempearature (T˚C)	Expected product size (bp) in PCR	Expected product size (bp) in Q-PCR	Putative Functions
OsISM06076	LOC_Os01g45090	25591787	AAGGATCCTGGGACAACCTC	GCCTCAGGTAGTTGAGCCAC	60.1	199	85	MYB family transcription factor putative expressed
OsISM06078	LOC_Os01g45190	25636224	CATGATCTCGCTCTCCGTCT	TTCTGTTTGTGCAGCAAGTTC	59.8	384	98	DEAD-box ATP-dependent RNA helicase putative expressed
OsISM06079	LOC_Os01g45190	25636750	TCAAGAGAAGGACCTTGCGT	GATCCTTAAAGCCTCTGCCC	60.1	167	85	DEAD-box ATP-dependent RNA helicase putative expressed
OsISM06080	LOC_Os01g45190	25636898	GGCAGAGGCTTTAAGGATCA	ATCTCGTGAGGCAGAGTTGC	60.0	494	93	DEAD-box ATP-dependent RNA helicase putative expressed
OsISM06081	LOC_Os01g45190	25637433	TCCTTGTGAAGCGTGATGAA	CAAAGCGTGTCAAACTTCCA	60.1	203	89	DEAD-box ATP-dependent RNA helicase putative expressed
OsISM06082	LOC_Os01g45190	25637653	TGACAATCACCCAAGCTGTC	GCATTCTTTCCGTAAGCCAA	59.9	426	67	DEAD-box ATP-dependent RNA helicase putative expressed
OsISM06083	LOC_Os01g45190	25638183	ACGACAGATGTGTGGGCTC	CCAATGCGATGGATGTAAAG	59.3	191	99	DEAD-box ATP-dependent RNA helicase putative expressed
OsISM06084	LOC_Os01g45190	25638356	TTACATCCATCGCATTGGTC	CGGATGTCTTCCTTTTTGACA	59.7	732	79	DEAD-box ATP-dependent RNA helicase putative expressed
OsISM06085	LOC_Os01g45210	25655664	AAGAGGAAATCCGACTGGCT	CATATCCGAAGGTTTCCACG	60.3	230	93	retrotransposon protein putative Ty3-gypsy subclass
OsISM06086	LOC_Os01g45274	25691958	AGTTATCTACTTACTCTCTCTG TCCGA	GCATAATGCACGTACCGC	58.2	3943	91	carbonic anhydrase chloroplast precursor putative expressed
OsISM06088	LOC_Os01g45274	25696012	CAAGGCTGCATATACATACAC GA	TGGAGTATCTGATTGTTGATAGT TGA	59.6	2445	93	carbonic anhydrase chloroplast precursor putative expressed
OsISM06089	LOC_Os01g45274	25698473	CGAGGCAACCCTACTGCTAA	TCGTGTGGATCGTCCTTTATT	59.9	223	99	carbonic anhydrase chloroplast precursor putative expressed
OsISM06090	LOC_Os01g45274	25698646	AGCCGTCACGAGTCAAATTC	GCAGAGGCATCCACTCATCT	60.3	545	89	carbonic anhydrase chloroplast precursor putative expressed
OsISM06091	LOC_Os01g45274	25699200	TATAAAAAGGCCCCAAACGG	AGAGGAGAAGAGGATGGATGG	60.3	240	79	carbonic anhydrase chloroplast precursor putative expressed
OsISM06092	LOC_Os01g45274	25699450	TTCTTGATTCATCCGTCGAT	TCCATCTGCAAGCAAAAATAA	58.3	291	99	carbonic anhydrase chloroplast precursor putative expressed
OsISM06093	LOC_Os01g45290	25707393	CACCCCCGTTTATCACCTAC	CTCGAACCCAGTCTTGAAGC	59.6	160	90	retrotransposon protein putative unclassified expressed
OsISM06094	LOC_Os01g45290	25707581	TTTCTGATGCCCTGAGAGTTC	TGGCAGCACTATACCTTTGG	59.1	145	86	retrotransposon protein putative unclassified expressed
OsISM06095	LOC_Os01g45290	25707648	TTTGTCGGTTTTCAGCAAGA	GTGAGGTTCTTCGCGATTTC	59.6	1034	94	retrotransposon protein putative unclassified expressed

ISM IDs	Rice gene locus IDs	Physical positions (bp) on chromosomes	Forward primers (5'-3')	Reverse primers (5'-3')	Melting tempearature (T˚C)	Expected product size (bp) in PCR	Expected product size (bp) in Q-PCR	Putative Functions
OsISM06096	LOC_Os01g45290	25713034	TGGATGAATTCCGTGCATTA	GTCAGCACACGAGAAAACCA	59.9	611	93	retrotransposon protein putative unclassified expressed
OsISM06097	LOC_Os01g45290	25713705	AATATTGCCAGCATGGTTCC	TGACCTTGAGGACAACCACA	60.0	203	89	retrotransposon protein putative unclassified expressed
OsISM06098	LOC_Os01g45350	25744282	CCTATACGCGCCTCTCTTCA	GGCCGTCCTGGTAAATGTAA	60.2	1404	93	exostosin family protein putative expressed
OsISM06099	LOC_Os01g45350	25745927	GTTCTGGAATCGCACCAGAG	CGCCGTCGTCGTGTAAGA	61.4	167	74	exostosin family protein putative expressed
OsISM06100	LOC_Os01g45380	25757301	GTCTACGACGGCCAGGAC	CTGATCTATGGCCATCCTCC	59.3	1487	62	protein kinase domain containing protein expressed
OsISM06101	LOC_Os01g45380	25758809	GAGCAGCATTTGCACAAGAG	TCTTGCACCCATTATAGCCC	59.8	810	87	protein kinase domain containing protein expressed
OsISM06102	LOC_Os01g45380	25759761	TCCAATTGGCTCTTGACCTT	TCAGTCTTCACATCACGATGAAC	59.9	179	77	protein kinase domain containing protein expressed
OsISM06103	LOC_Os01g45380	25760038	GCACACTTGGTTACATGGCA	CTGTTGTAAGGATGGCCGTT	60.3	362	50	protein kinase domain containing protein expressed
OsISM06104	LOC_Os01g45380	25760456	TGCCGTATCCTGATCTGAGTT	CAAGGAGCTAGGGCAGCAG	60.5	208	93	protein kinase domain containing protein expressed
OsISM06105	LOC_Os01g45430	25772938	TGGCACTGAGATCAAAAACG	CGGAGGTTGTACACATCGGT	60.3	640	85	hypothetical protein
OsISM06106	LOC_Os01g45430	25773584	CACCCTCAACCTCAATCTCC	TTATCCAACTCGGGCCTGTA	60.0	814	52	hypothetical protein
OsISM06107	LOC_Os01g45430	25774908	CAGGCAGCATGAACATCAAG	GCACCCCTTTGTAGATGAGC	60.1	145	96	hypothetical protein
OsISM06108	LOC_Os01g45450	25788589	ATGGAGGTCCTGACGGAAG	GTTGAGCAGCCCAGACAAAG	60.5	813	66	AAA family ATPase putative expressed
OsISM06109	LOC_Os01g45460	25791981	CTTCAGCCTCCTGCAGTACC	TTGCATAGATTGTCAGGGCA	60.1	1317	72	serine esterase putative expressed
OsISM06110	LOC_Os01g45460	25793278	TGCCCTGACAATCTATGCAA	CGAACCTGACTTGAGAAGAAA	58.7	560	79	serine esterase putative expressed
OsISM06111	LOC_Os01g45460	25793817	TTTCTTCTCAAGTCAGGTTCG	CTGGGCAGGGTCTGTTGATA	59.1	181	99	serine esterase putative expressed
OsISM06112	LOC_Os01g45460	25793995	CAGTGGTGGCCTTCGTAACT	GATTCCTCTCTCAGCCAACC	59.5	171	90	serine esterase putative expressed
OsISM06113	LOC_Os01g45460	25794146	GGTTGGCTGAGAGAGGAATC	AAGCATCGCTAACCATGTCA	59.1	194	85	serine esterase putative expressed

ISM IDs	Rice gene locus IDs	Physical positions (bp) on chromosomes	Forward primers (5'-3')	Reverse primers (5'-3')	Melting tempearature (T˚C)	Expected product size (bp) in PCR	Expected product size (bp) in Q-PCR	Putative Functions
OsISM06114	LOC_Os01g45460	25794322	ACATGGTTAGCGATGCTTCC	GCTGACTGACCCACCAAGTAG	59.9	539	98	serine esterase putative expressed
OsISM06115	LOC_Os01g45460	25794917	GGCAGTCCATTTCTTGGAGT	TCATGAAAATGGTCAACCAAA	59.0	986	92	serine esterase putative expressed
OsISM06116	LOC_Os01g45460	25795896	TTCATGAACGTGGTCTTAATCG	CTGCTTGACAACAATTTCCG	59.7	175	93	serine esterase putative expressed
OsISM06117	LOC_Os01g45480	25802590	GACCCTCCAATCCGCATC	GACCTCCTCCCCATCGTC	60.9	368	49	retrotransposon protein putative unclassified
OsISM06118	LOC_Os01g45520	25835207	GCCAAAGGCTTATCTCGTCC	GGTACCATCTGTTAGGCGCT	60.2	1548	94	D-mannose binding lectin family protein
OsISM06119	LOC_Os01g45520	25837654	AAGTTGCCTGACAAAGCCTG	CAGTTGCCTTCCTCGTTAGC	60.2	222	71	D-mannose binding lectin family protein
OsISM06120	LOC_Os01g45550	25864088	GAAATCCACATGGTCATCCC	CACTGCGCCGTACTCCTC	60.3	180	76	auxin efflux carrier component putative expressed
OsISM06121	LOC_Os01g45550	25864546	CACTTACTCCAGCCTCCTCG	ATTTGCCACACGCGATGAT	61.2	989	91	auxin efflux carrier component putative expressed
OsISM06122	LOC_Os01g45550	25865596	GTCAATCGCCATCGGACT	ACAAAAGGCACAATCCCTTG	59.8	1173	82	auxin efflux carrier component putative expressed
OsISM06123	LOC_Os01g45550	25866780	AATACAATGTCCACCCGGC	CAGCAATGTGATTGGAAGAGC	60.7	167	75	auxin efflux carrier component putative expressed
OsISM06124	LOC_Os01g45580	25889416	AAGGTGGTCATTCAACCGAC	GATCTTCGGCCTCGGACT	60.1	182	87	transposon protein putative unclassified
OsISM06125	LOC_Os01g45640	25925491	TGATGACTGTGACTTGCTGC	TGACAGTGCATCTCTACGGG	59.2	1293	90	tat pathway signal sequence family protein expressed
OsISM06127	LOC_Os01g45740	25969214	TTCAAAGGCTTATGATCGGG	AACAGAAAGGGGGATAACGG	60.1	193	64	retrotransposon protein putative unclassified
OsISM06128	LOC_Os01g45740	25971168	TGTTGATGGCTCCTTTGATG	TCCCTCTCCTTTGATTCGAG	59.5	243	94	retrotransposon protein putative unclassified
OsISM06130	LOC_Os01g45750	25973073	AGGAGAAGGAAGAATGGGGA	GTTAGCTTCAGCCTTGCACA	59.6	1447	99	bile acid sodium symporter family protein putative expressed
OsISM06131	LOC_Os01g45750	25974582	GCTGCTTACCACTCTTTTCCC	GATGGCTTGTAGATGCCAATAA	59.7	190	64	bile acid sodium symporter family protein putative expressed
OsISM06132	LOC_Os01g45750	25974717	GAGCTCACCTTTTGCAGGTC	CCTAGGCCCACAGTGAAAAG	59.9	194	98	bile acid sodium symporter family protein putative expressed
OsISM06133	LOC_Os01g45750	25974954	TTCAGGAGATGCATGAGGAA	ACCATGGCAATAGCAAATCC	59.6	202	93	bile acid sodium symporter family protein putative expressed

ISM IDs	Rice gene locus IDs	Physical positions (bp) on chromosomes	Forward primers (5'-3')	Reverse primers (5'-3')	Melting tempearature (T˚C)	Expected product size (bp) in PCR	Expected product size (bp) in Q-PCR	Putative Functions
OsISM06134	LOC_Os01g45750	25975098	TGGGATTTCTTGCGCAGTAT	AGACCAGTTGCAAGAGGAGC	60.1	926	89	bile acid sodium symporter family protein putative expressed
OsISM06135	LOC_Os01g45750	25976085	GGAAATGTCGCACTTTCAGTT	GAACCAGTTGACCAGCTAGGA	59.3	1429	98	bile acid sodium symporter family protein putative expressed
OsISM06136	LOC_Os01g45750	25977497	AGCTGGTCAACTGGTTCCTG	GGGCTTCTTTAGTACCTCCGA	60.0	190	96	bile acid sodium symporter family protein putative expressed
OsISM06137	LOC_Os01g45750	25977606	TTACATCTGTCCAGGGATTGG	AGGAAAATACTCATGCGCCA	60.2	180	87	bile acid sodium symporter family protein putative expressed
OsISM06138	LOC_Os01g45750	25977819	TTGATTGGGGTTCTCCTCAC	TTGACCACCTTGAGCTTTCA	59.7	316	82	bile acid sodium symporter family protein putative expressed
OsISM06139	LOC_Os01g45750	25978200	TTCCTCTTTTGGGGAATCAA	ATTCGTGAAGTGCTTTTGGG	59.8	577	98	bile acid sodium symporter family protein putative expressed
OsISM06140	LOC_Os01g45750	25978757	CCCAAAAGCACTTCACGAAT	AACTGCAAGAGCACTCCCTC	59.9	215	87	bile acid sodium symporter family protein putative expressed
OsISM06141	LOC_Os01g45760	25994259	AGTGAGCTTGGATTTGTGCC	CGAACGTGGATAGACCGAAC	60.4	477	99	YUC4 putative expressed
OsISM06142	LOC_Os01g45760	25994903	GTTGGGACGCTAGACCACAT	CTTGCCTCGTCATCTCCTTC	60.0	331	71	YUC4 putative expressed
OsISM06143	LOC_Os01g45760	25995272	ACAATAATCCTCGCCACAGG	CCCTCCCTCGTGAACAAGT	60.0	251	78	YUC4 putative expressed
OsISM06144	LOC_Os01g45790	26008800	CAGTCTCGGTTCTGGTTGGT	TAGGGTGACAAAGGCTGGTC	60.1	701	90	hypothetical protein
OsISM06145	LOC_Os01g45800	26017021	CCCTCCAAGGTGGTCATTTA	GATCTTCGGCCTCGGACT	60.0	193	93	transposon protein putative unclassified expressed
OsISM06146	LOC_Os01g45840	26036511	AACACAGGCTGTTGATGAAGG	GCTCTCCCCAGGAGGTCTAC	60.2	1020	98	CRR2 putative expressed
OsISM06147	LOC_Os01g45890	26070579	GTCGACAAGTCGGAGATTGG	CAAGTGGAATGCAAAGCTGA	60.3	440	75	hypothetical protein
OsISM06148	LOC_Os01g45890	26070999	TCAGCTTTGCATTCCACTTG	CAATCACCCGATTTTCAGATG	60.2	564	89	hypothetical protein
OsISM06150	LOC_Os01g45900	26074564	CTTCGGCCTCAAGCTCGT	TACTCGAAGAGGAGTGGCGT	60.8	257	94	OsFBX20 - F-box domain containing protein expressed
OsISM06151	LOC_Os01g45960	26106337	GAGCTCGCTCGTGGACATAC	AGCCCAGAAGCATCAGACAT	60.4	625	85	hydrolase alpha/beta fold family domain containing protein expressed
OsISM06152	LOC_Os01g45960	26107212	CTTATCCATGGCATTGACCC	TGTGGGAGATCGAGGAGTCT	60.0	226	82	hydrolase alpha/beta fold family domain containing protein expressed

ISM IDs	Rice gene locus IDs	Physical positions (bp) on chromosomes	Forward primers (5'-3')	Reverse primers (5'-3')	Melting tempearature (T°C)	Expected product size (bp) in PCR	Expected product size (bp) in Q-PCR	Putative Functions
OsISM06153	LOC_Os01g45960	26108030	CATCTCGACCTCGCAGAAAG	CATGTCCGAGCAAAATGAAA	60.4	589	99	hydrolase alpha/beta fold family domain containing protein expressed
OsISM06154	LOC_Os01g45960	26108684	AAAGTTGCGAGCATCTGGAG	TACGAGCAAATGCAGGAATG	60.2	228	99	hydrolase alpha/beta fold family domain containing protein expressed
OsISM06155	LOC_Os01g45960	26108992	CGTGCATGGTTTGATGCTAC	CATCCCAGCCTTCAACAAAT	60.0	386	80	hydrolase alpha/beta fold family domain containing protein expressed
OsISM06156	LOC_Os01g45960	26109516	CAACGCAAGTAATGGCTTCA	CCTTGGGACACTCCTCATGT	59.9	224	88	hydrolase alpha/beta fold family domain containing protein expressed
OsISM06157	LOC_Os01g46000	26142872	AAAAATTGCAAAGCTCCCAA	TTTTTGAAAAGATCACGACGAA	59.7	160	75	retrotransposon protein putative unclassified
OsISM06158	LOC_Os01g46000	26144936	CGGTACAGAAGCGGTCAGA	CCTCAACCAAAGCTACATTCTTC	59.7	426	70	retrotransposon protein putative unclassified
OsISM06159	LOC_Os01g46000	26145915	TCGATTGGAGTTTGAATTTCCT	AAAATACGACCATCCTCCCG	60.5	585	94	retrotransposon protein putative unclassified
OsISM06160	LOC_Os01g46000	26146507	GAGAGCGACAACAACGATGA	CAGCATCAACAGAACCCTCA	59.9	151	77	retrotransposon protein putative unclassified
OsISM06161	LOC_Os01g46010	26153708	AGTACAGCCCCACCTAGCCT	TTTGTGTCGAAGCTTGTGCT	59.9	555	77	hypothetical protein
OsISM06162	LOC_Os01g46010	26154515	GTGGCAATTAAGGGCTCAAG	TCACAAATAAGGATCAACTTCAT AGT	58.6	221	88	hypothetical protein
OsISM06163	LOC_Os01g46020	26162948	CCTCCCTCGATCTCTCCTTC	CATAAATGTGCCACTTCTTCCA	60.1	1036	48	TATA-binding protein-associated factor TAFII55 family protein putative expressed
OsISM06164	LOC_Os01g46020	26164063	TGCTAATTAAAACAGCTGATA TTGG	TTCCTTCTGGAGCAGGATCA	59.9	149	70	TATA-binding protein-associated factor TAFII55 family protein putative expressed
OsISM06165	LOC_Os01g46020	26164266	TCCGGAGAGAGCCTGACTT	TGAGATGCTTTTCAACTCGG	59.5	140	55	TATA-binding protein-associated factor TAFII55 family protein putative expressed
OsISM06166	LOC_Os01g46020	26164380	GGTTCACCGAGTTGAAAAGC	CAGCTTTCTTGCCACCTTCT	59.7	588	99	TATA-binding protein-associated factor TAFII55 family protein putative expressed
OsISM06167	LOC_Os01g46030	26168873	GGCGATATCTAGAAGGCCA	GGCCTCTACGTCTGGCATAA	59.1	147	44	receptor-like protein kinase putative
OsISM06168	LOC_Os01g46060	26183010	AGCTCTTCGCTGCCGTCT	ATCATCTGGGGCTGAAATTG	60.7	753	99	NUC189 domain containing protein expressed
OsISM06169	LOC_Os01g46060	26183743	CAATTTCAGCCCCAGATGAT	CCAACAGCAACAAGAATAAGGT T	59.7	179	95	NUC189 domain containing protein expressed
OsISM06170	LOC_Os01g46060	26183961	TGTCATATGGAAGAATGCTCC T	ATAATTCCATCCATGCCAGC	59.2	822	97	NUC189 domain containing protein expressed

ISM IDs	Rice gene locus IDs	Physical positions (bp) on chromosomes	Forward primers (5'-3')	Reverse primers (5'-3')	Melting tempearature (T˚C)	Expected product size (bp) in PCR	Expected product size (bp) in Q-PCR	Putative Functions
OsISM06171	LOC_Os01g46060	26184804	CGGGGAATCCAAAGACACTA	TCTTACTTGATACACCCATAAAC TTC	58.5	614	93	NUC189 domain containing protein expressed
OsISM06172	LOC_Os01g46060	26185448	AAGAGATCTTGAGGATTCCTT CTG	ACAGACATCAGCTGGACAGG	59.1	176	50	NUC189 domain containing protein expressed
OsISM06173	LOC_Os01g46060	26186077	AGATATGGTGTCTCCGCAGG	ACAGGTTCTGCATCCTTGCT	60.0	764	70	NUC189 domain containing protein expressed
OsISM06174	LOC_Os01g46060	26186859	AAGGACATCATCCGTTTTGG	CCATGGTCGGTTCATCAAG	59.8	242	81	NUC189 domain containing protein expressed
OsISM06175	LOC_Os01g46060	26187209	AAGCTTTGCGTGCTGATGAT	CTGCCGGTGTTAGGAGTGAT	60.5	733	97	NUC189 domain containing protein expressed
OsISM06176	LOC_Os01g46060	26187922	ATCACTCCTAACACCGGCAG	AGCCATGGGAGCATACAAAC	60.0	185	97	NUC189 domain containing protein expressed
OsISM06177	LOC_Os01g46060	26188125	GCACATGAGCAGCATAGTGTC	TGAAGGTTGATGTTCTTGCATC	59.8	357	87	NUC189 domain containing protein expressed
OsISM06178	LOC_Os01g46060	26188462	TGCAAGAACATCAACCTTCAA	GCGTCCTCCTCATCAGTTTC	59.6	164	97	NUC189 domain containing protein expressed
OsISM06179	LOC_Os01g46070	26190046	CGACGTCTCCCACATCAAC	GATGATCACGATGTCCGACC	60.7	202	95	lactate/malate dehydrogenase putative expressed
OsISM06180	LOC_Os01g46070	26190314	GTTAAGAACCTCTGCACCGC	CTGGGTTGCTGATCATGTTG	60.0	553	68	lactate/malate dehydrogenase putative expressed
OsISM06181	LOC_Os01g46070	26190975	TCTATGCTGGAAAGGCCAAT	CTGTGAGAACAGTGGCAGGA	59.8	198	93	lactate/malate dehydrogenase putative expressed
OsISM06182	LOC_Os01g46070	26191149	ACCATCCTGCCACTGTTCTC	CCCACCATCCTGTGTTCTCT	60.0	677	94	lactate/malate dehydrogenase putative expressed
OsISM06183	LOC_Os01g46070	26191816	GGATGGTGGGACTGAAGTTG	CTTTAAGCAAGCATTTGCGA	59.8	204	98	lactate/malate dehydrogenase putative expressed
OsISM06184	LOC_Os01g46070	26192074	ACAGAGCTGCCATTCTTTGC	CCTCAACTCCATTCTTCCCA	60.3	962	53	lactate/malate dehydrogenase putative expressed
OsISM06186	LOC_Os01g46080	26197653	TCGTGATCTGCTGAAACTGC	TCTCCGACCAGGAAAAGAGA	60.0	1293	82	GDSL-like lipase/acylhydrolase putative expressed
OsISM06187	LOC_Os01g46080	26198998	AAGATTCGCAGCTTCACTCC	GGTTACCAGGAACAACCAGC	59.5	206	98	GDSL-like lipase/acylhydrolase putative expressed
OsISM06188	LOC_Os01g46080	26199411	TTTTCCTTTCCCCTGAACAA	TCCACATCTTGCAGTTCCAG	59.7	594	96	GDSL-like lipase/acylhydrolase putative expressed
OsISM06189	LOC_Os01g46090	26202348	GATCTGCTCGACATGCTCTG	CGATTTCTCCGACCAAAAAG	59.7	2435	83	GDSL-like lipase/acylhydrolase putative

ISM IDs	Rice gene locus IDs	Physical positions (bp) on chromosomes	Forward primers (5'-3')	Reverse primers (5'-3')	Melting tempearature (T˚C)	Expected product size (bp) in PCR	Expected product size (bp) in Q-PCR	Putative Functions
OsISM06190	LOC_Os01g46090	26204826	TGAAAAGATTCGCAGCTTCA	GCTCCAAGTCCGATCAATTC	59.7	178	88	GDSL-like lipase/acylhydrolase putative
OsISM06191	LOC_Os01g46090	26205218	TATGGAGCTGCCATGGAAAT	CATAAGGTCCTCCTCCACCA	60.2	422	89	GDSL-like lipase/acylhydrolase putative
OsISM06193	LOC_Os01g46120	26222227	AGTGGTTCCGTGATCTGCTC	CCGATTTCTCCAACCAAGAA	60.2	619	98	GDSL-like lipase/acylhydrolase putative expressed
OsISM06194	LOC_Os01g46120	26222883	TCCATTGAAAAGATTCGCAAC	ACAACTAGTGTCTTGGCCCC	59.6	203	96	GDSL-like lipase/acylhydrolase putative expressed
OsISM06195	LOC_Os01g46120	26223298	TGGAAGTTTTCCTTTCCCCT	AAGGTCCTCTTCCACCACAG	59.5	401	73	GDSL-like lipase/acylhydrolase putative expressed
OsISM06197	LOC_Os01g46140	26234805	GGGAATCTTCTTTTCCCCTG	AGCTGCCAAAGGATTCTCAA	59.9	1838	50	expressed protein
OsISM06198	LOC_Os01g46169	26248659	CCGCCTCATCATCGACTT	CAGTACGGCCGCAGGAAC	61.3	165	58	GDSL-like lipase/acylhydrolase putative expressed
OsISM06199	LOC_Os01g46169	26248981	CTGCTCTGCCCAGACGAT	GTGATGACTTGCCTTGCTCA	60.0	12955	92	GDSL-like lipase/acylhydrolase putative expressed
OsISM06200	LOC_Os01g46169	26261862	TATAGAGACCGAGGGAGCCA	TTGATTCATCATGTCGTTGC	58.9	9250	83	GDSL-like lipase/acylhydrolase putative expressed
OsISM06201	LOC_Os01g46169	26271198	CACTCCGAGTATCATTGCCA	AACTAACGTCTTGGCTCCGA	59.8	577	71	GDSL-like lipase/acylhydrolase putative expressed
OsISM06202	LOC_Os01g46210	26285629	GCATGACCTACTTCAACCGC	GGTAGCAGCGACAGGTTGAG	60.8	197	92	GDSL-like lipase/acylhydrolase putative
OsISM06203	LOC_Os01g46210	26285982	GAAAGTGCTCGGTCGGATAG	GACTCGCCTAGCAGGCTCT	59.9	182	58	GDSL-like lipase/acylhydrolase putative
OsISM06204	LOC_Os01g46210	26286498	AGGCTCATCTACGCCGACTA	AGCACTCCAGCAGAGGGTC	60.3	193	89	GDSL-like lipase/acylhydrolase putative
OsISM06205	LOC_Os01g46220	26289382	CCGTTTGGCATGACCTACTT	GGTACAAACGGAAGCTGGAG	59.9	599	99	GDSL-like lipase/acylhydrolase putative expressed
OsISM06206	LOC_Os01g46220	26290123	GAGTGGTTCAAGCAAACGCT	CCGATCTCACCCATCAAGATA	60.2	247	93	GDSL-like lipase/acylhydrolase putative expressed
OsISM06208	LOC_Os01g46220	26290792	AGGCTCATCTACGCCGACTA	ACGCGAGCAGAGGATCATT	60.5	207	86	GDSL-like lipase/acylhydrolase putative expressed
OsISM06209	LOC_Os01g46240	26295992	TCAAATTGGATGGGGTTTGT	CACACAAGGGTCATCAGCAC	60.1	183	76	lipase class 3 family protein putative expressed
OsISM06210	LOC_Os01g46240	26296191	CTGTGTACACGAGTGCCGAT	CGTACATATCCTGGAGCCGT	59.9	828	94	lipase class 3 family protein putative expressed

ISM IDs	Rice gene locus IDs	Physical positions (bp) on chromosomes	Forward primers (5'-3')	Reverse primers (5'-3')	Melting tempearature (T°C)	Expected product size (bp) in PCR	Expected product size (bp) in Q-PCR	Putative Functions
OsISM06211	LOC_Os01g46250	26299691	AATGCTCGGAGCTGAACAAG	GTCGCATAGGATCAAATCCC	59.9	1347	67	lipase putative expressed
OsISM06212	LOC_Os01g46250	26301018	GGGATTTGATCCTATGCGAC	CCATCAGCCTCGCTATCTCT	59.5	251	60	lipase putative expressed
OsISM06213	LOC_Os01g46290	26321117	GCCGTTCAACAAACTTAGCG	TGATGCTGCAGTTCTCGTTC	60.5	1867	87	lipase putative expressed
OsISM06215	LOC_Os01g46330	26355069	CCATGTGAGCTGCATATTCC	AGTACCTGAAGGGTCCGTTG	59.1	158	82	transposon protein putative CACTA En/Spm sub-class
OsISM06216	LOC_Os01g46330	26356233	AACTATACCAGCTGGACGCC	TCCAGTATCGAAAACCCTCC	59.1	207	96	transposon protein putative CACTA En/Spm sub-class
OsISM06217	LOC_Os01g46330	26356521	AAGGCGCAGCATTACAAGAC	GCCTCCAGGTCGAAGAGTAA	59.9	179	81	transposon protein putative CACTA En/Spm sub-class
OsISM06218	LOC_Os01g46330	26356700	CTGCACCGTCAACGTATATGA	AATGACGGAACCGATACCAA	59.9	182	96	transposon protein putative CACTA En/Spm sub-class
OsISM06219	LOC_Os01g46330	26356861	CTTGGTATCGGTTCCGTCAT	AGTTCCCTGCTTTTGCTTTG	59.7	189	93	transposon protein putative CACTA En/Spm sub-class
OsISM06220	LOC_Os01g46330	26357084	ACTGCCTTGCAGACCAAATC	CCGCGATAAATTCCTTGTGT	60.1	169	88	transposon protein putative CACTA En/Spm sub-class
OsISM06221	LOC_Os01g46340	26364824	AAATCGAGAATCGTTCGGCT	CACCTCCTTGATCAGGAACC	60.3	899	87	chloroplast unusual positioning protein putative expressed
OsISM06222	LOC_Os01g46340	26365751	ACGTTGTCACGTTCGTCAAG	CTTGTTCTCCGGCCACTC	59.6	189	93	chloroplast unusual positioning protein putative expressed
OsISM06223	LOC_Os01g46340	26366036	TCCACCGCACTGAAGAAGAT	AGGTTGTAGACGCCGTGC	60.3	174	60	chloroplast unusual positioning protein putative expressed
OsISM06224	LOC_Os01g46340	26366266	AGTGGATGCAGCCAGACAC	CAAGTTCAGAAGAGACCCGC	59.9	1494	94	chloroplast unusual positioning protein putative expressed
OsISM06225	LOC_Os01g46340	26367795	ATGCTTCAAGGAGTGCGATT	TCCTTGAGCTCTTGGAATGC	60.2	217	90	chloroplast unusual positioning protein putative expressed
OsISM06226	LOC_Os01g46360	26376394	GCTGGGTCTGCCATAATCAA	CCTTGGGGAAGTCATTGTCA	60.9	242	83	hypothetical protein
OsISM06227	LOC_Os01g46360	26376679	TCTTTCCAAGTCCTCTTCCG	CGCCATAAATTGAACCTCCA	60.1	209	99	hypothetical protein
OsISM06228	LOC_Os01g46380	26391415	GTGTCTCTAGCTGGCCATGA	GATCTGCTTGATCCCCTTGA	59.6	194	91	ketol-acid reductoisomerase chloroplast precursor putative expressed
OsISM06229	LOC_Os01g46380	26391587	GTTCAAGGGGATCAAGCAGA	TGATTTTGCCTCAGCAAGTG	60.1	688	95	ketol-acid reductoisomerase chloroplast precursor putative expressed

ISM IDs	Rice gene locus IDs	Physical positions (bp) on chromosomes	Forward primers (5'-3')	Reverse primers (5'-3')	Melting tempearature (T°C)	Expected product size (bp) in PCR	Expected product size (bp) in Q-PCR	Putative Functions
OsISM06230	LOC_Os01g46380	26392255	CACTTGCTGAGGCAAAATCA	CGCGTGCTTCTTCAAAAGAT	60.3	358	76	ketol-acid reductoisomerase chloroplast precursor putative expressed
OsISM06231	LOC_Os01g46380	26392673	GCGATCTTTTGTTGTTGTTGA	TGTTGGGTTTCATGTGTGAGA	59.4	533	79	ketol-acid reductoisomerase chloroplast precursor putative expressed
OsISM06232	LOC_Os01g46380	26393339	GGGAAAAGAAGTAAACGGTGC	CGACATCTGTTGCTCTTCCA	60.0	195	78	ketol-acid reductoisomerase chloroplast precursor putative expressed
OsISM06233	LOC_Os01g46380	26393600	GAAGCGATATTTTTGGCGAG	TCCAACCCATGCTCTGTGTA	60.0	161	92	ketol-acid reductoisomerase chloroplast precursor putative expressed
OsISM06234	LOC_Os01g46380	26393782	CACTGTTGAGTGCATCACTGG	TGCTTCTTTCCTTCTTCTGTCA	59.8	222	94	ketol-acid reductoisomerase chloroplast precursor putative expressed
OsISM06235	LOC_Os01g46380	26394098	GTTGTTTTGGCTGGAAGGAG	CGTGTTTGATCAATGTTGCC	59.8	170	75	ketol-acid reductoisomerase chloroplast precursor putative expressed
OsISM06236	LOC_Os01g46380	26394318	TTGGCCCATTACATCCTTTC	TCCGAGTAAGAGTGGCCCTT	60.5	191	92	ketol-acid reductoisomerase chloroplast precursor putative expressed
OsISM06237	LOC_Os01g46420	26417578	GGCGGGAGCTAAAGAGGC	GGCATTAGAAATAAAAATATACATGGG	60.9	688	53	hypothetical protein
OsISM06239	LOC_Os01g46500	26448821	AGGCCTCGGATCTCTCTACC	GGTGCATTTCTTGCCCATAC	60.1	964	72	OsFBX21 - F-box domain containing protein expressed
OsISM06240	LOC_Os01g46500	26450770	CACCGAGCAAATTCTTCAAA	TGAGAACAACAAATGGTTCGAT	59.2	182	94	OsFBX21 - F-box domain containing protein expressed
OsISM06241	LOC_Os01g46500	26451041	CCAGCCTTGGAGTCATTACA	GCAGAGATCACCTGCAGTTCT	59.2	168	67	OsFBX21 - F-box domain containing protein expressed
OsISM06242	LOC_Os01g46530	26491997	CTAGTTGGTGTGCAGGGTCA	TCCCAACCCATCCATAACAT	59.8	639	63	hypothetical protein
OsISM06243	LOC_Os01g46540	26495785	AGGCATTTGGCGATTTATTG	CACGTCGATGTCGTTACCAG	60.1	642	99	transposon protein putative CACTA En/Spm sub-class
OsISM06244	LOC_Os01g46554	26500287	CGTGAATGAGAACAAGCGAA	ATCTGTTTCTCATCGCGGTC	60.1	1013	91	transposon protein putative CACTA En/Spm sub-class
OsISM06245	LOC_Os01g46554	26502654	CCATGTGAGCTGCATATTCC	AGTACCTGAAGGGTCCGTTG	59.1	158	82	transposon protein putative CACTA En/Spm sub-class
OsISM06246	LOC_Os01g46554	26503834	CGCCCTCGACGTCTCTATTA	TCCAGTATCGAAAACCCTCC	59.7	191	80	transposon protein putative CACTA En/Spm sub-class
OsISM06247	LOC_Os01g46554	26504091	CTCGTCCATCTCCTGAAGGT	GCCTCCAGGTCGATGAGTAA	59.7	194	96	transposon protein putative CACTA En/Spm sub-class
OsISM06248	LOC_Os01g46554	26504285	CTGCACCGTCAACGTATATGA	AATGACGGAACCGATACCAA	59.9	182	96	transposon protein putative CACTA En/Spm sub-class

ISM IDs	Rice gene locus IDs	Physical positions (bp) on chromosomes	Forward primers (5'-3')	Reverse primers (5'-3')	Melting tempearature (T˚C)	Expected product size (bp) in PCR	Expected product size (bp) in Q-PCR	Putative Functions
OsISM06249	LOC_Os01g46554	26504474	CGGCAAATGGAGAGAAAGAC	GGCAATACTCGCACACGTAA	59.8	195	99	transposon protein putative CACTA En/Spm sub-class
OsISM06250	LOC_Os01g46554	26504669	ACTGCCTTGCAGACCAAATC	CCGCGATAAATTCCTTGTGT	60.1	169	88	transposon protein putative CACTA En/Spm sub-class
OsISM06251	LOC_Os01g46570	26513782	CGCGTCACCACCATCAAG	TCCAAAACGAAGACCTCACC	61.2	1381	87	CTP synthase putative expressed
OsISM06252	LOC_Os01g46570	26515143	GGTGAGGTCTTCGTTTTGGA	TGATGTCCAGAAAACGTTCG	59.9	271	74	CTP synthase putative expressed
OsISM06253	LOC_Os01g46570	26515430	CAACATAACCACGGGAAAAA	CTGGTCACACACCTGAATGG	59.2	777	89	CTP synthase putative expressed
OsISM06254	LOC_Os01g46570	26516163	GGAGAGGAGACTACTTGGGGA	TCGCTACACGTTCAATCCAA	60.0	168	82	CTP synthase putative expressed
OsISM06255	LOC_Os01g46570	26516337	CGGTCGATGGTAAAGAAGGA	TGCTTCAATAAAGGGCATTG	59.4	261	90	CTP synthase putative expressed
OsISM06256	LOC_Os01g46570	26516546	CAGTGGCATTCTTGATGCAG	GGACCAGACAGAAGTTTCCAG	59.6	191	99	CTP synthase putative expressed
OsISM06257	LOC_Os01g46570	26516732	GGTCCATGTCAGTCTTGTTCC	CCTCTTAGCCCACGAACACT	59.4	561	85	CTP synthase putative expressed
OsISM06258	LOC_Os01g46570	26517297	GGATTGGCACCTGACATTTT	TGCTTTCACGTTTTCTTCCA	59.6	166	61	CTP synthase putative expressed
OsISM06259	LOC_Os01g46570	26517443	TGGAAGAAAACGTGAAAGCA	AAGGGGATGTGCCAAATGT	59.8	182	98	CTP synthase putative expressed
OsISM06260	LOC_Os01g46570	26517602	ACAAACATTTGGCACATCCC	TTTCAGAATAGCTTCATGGGC	60.0	142	61	CTP synthase putative expressed
OsISM06261	LOC_Os01g46570	26517720	AGGGCCCATGAAGCTATTCT	CAACCAACTTGGGTTCTCGT	60.0	168	74	CTP synthase putative expressed
OsISM06262	LOC_Os01g46570	26517875	CCCAAGTTGGTTGAATGGAC	ATTTTCCAACCATCGCAATC	60.0	213	80	CTP synthase putative expressed
OsISM06263	LOC_Os01g46570	26518068	GATTGCGATGGTTGGAAAAT	TTCTTCCCATAGCAACCGAT	59.6	425	90	CTP synthase putative expressed
OsISM06264	LOC_Os01g46570	26518524	TCTTGAAGATTCTGCAGCCA	GGGGCCATTCGAGTATGTAA	59.7	176	93	CTP synthase putative expressed
OsISM06265	LOC_Os01g46570	26518622	TTGTACAGACCCCTGAAGCC	CCTCCAGGGACAAGTATACCC	59.9	159	74	CTP synthase putative expressed
OsISM06266	LOC_Os01g46570	26518950	ATGTCACCCTGCGTCATTTT	TGTTCTCCTTGATCCAAGCC	60.3	171	79	CTP synthase putative expressed

ISM IDs	Rice gene locus IDs	Physical positions (bp) on chromosomes	Forward primers (5'-3')	Reverse primers (5'-3')	Melting tempearature (T°C)	Expected product size (bp) in PCR	Expected product size (bp) in Q-PCR	Putative Functions
OsISM06267	LOC_Os01g46570	26519101	GGCTTGGATCAAGGAGAACA	TCTGTGTCGATGTCTTTCGTCT	60.1	190	96	CTP synthase putative expressed
OsISM06268	LOC_Os01g46570	26519266	CGTAGACGAAAGACATCGACA	CACTCTCATCCTTGCCAACA	59.4	185	96	CTP synthase putative expressed
OsISM06269	LOC_Os01g46570	26519440	GGATGAGAGTGGAAGACGCA	GCTTCCCTGGTCTTGACTTG	60.6	193	99	CTP synthase putative expressed
OsISM06270	LOC_Os01g46570	26519625	AGGGAAGCCATCTCCACTTT	TTGTCCAGATGCTGCTGCTA	60.4	487	50	CTP synthase putative expressed
OsISM06271	LOC_Os01g46580	26526688	CCAGGTTCCTGCTCCAGAT	GATACCGCACATCGTCGAAT	60.6	182	97	actin-related protein 2/3 complex subunit 2 putative expressed
OsISM06272	LOC_Os01g46580	26526849	AATTCGACGATGTGCGGTAT	GGCAATGCTACTGATAGCACC	60.1	706	74	actin-related protein 2/3 complex subunit 2 putative expressed
OsISM06273	LOC_Os01g46580	26527687	AAACTTGACAAAGCTTCCGC	CAGCACCACCTCTCTCACAG	59.5	618	80	actin-related protein 2/3 complex subunit 2 putative expressed
OsISM06274	LOC_Os01g46580	26528373	TGTTGCTCTTGTTCACCGTC	CAATGGAATCCTGGAACCTC	59.6	278	96	actin-related protein 2/3 complex subunit 2 putative expressed
OsISM06275	LOC_Os01g46580	26528630	TGAGGTTCCAGGATTCCATT	TGTTAAGAGCGCTAGTCCGC	60.0	376	85	actin-related protein 2/3 complex subunit 2 putative expressed
OsISM06276	LOC_Os01g46580	26529071	CTAAATGCAAATGCCGGTTT	TTTTTACCCTCAACATGCCG	60.4	145	60	actin-related protein 2/3 complex subunit 2 putative expressed
OsISM06277	LOC_Os01g46580	26529217	TTGGACAAAACAGTTTGGAGTTT	TCATCCTGGTGTGCATGAAT	59.9	700	89	actin-related protein 2/3 complex subunit 2 putative expressed
OsISM06278	LOC_Os01g46580	26529897	ATTCATGCACACCAGGATGA	ATCAGATTTTGCACGATCCA	59.5	551	68	actin-related protein 2/3 complex subunit 2 putative expressed
OsISM06279	LOC_Os01g46580	26530426	TTTGGATCGTGCAAAATCTG	GACGCTAGTTGCCGTCATTA	59.3	168	96	actin-related protein 2/3 complex subunit 2 putative expressed
OsISM06280	LOC_Os01g46650	26564440	TCGACGAGGAAATGAAATCC	CCACTCTCTCCAGCACACAA	60.0	1111	98	hypothetical protein
OsISM06281	LOC_Os01g46650	26565492	TCCTGTAGCACCTGGGTTTC	GGATTTTGGAGGCCCATTAT	60.0	552	61	hypothetical protein
OsISM06282	LOC_Os01g46660	26569750	GTCCATCTGGACAGGCAAGT	GTGGTTCCTGTCAATCCCAC	60.2	452	75	transposon protein putative unclassified
OsISM06283	LOC_Os01g46660	26570182	GTGGGATTGACAGGAACCAC	TAGACCACAAAAGGGGATGC	60.1	877	89	transposon protein putative unclassified
OsISM06284	LOC_Os01g46660	26571254	GATGAGGTTTTGCCCAGATG	GGAGAGTGATGCCCAGTAGC	60.1	152	66	transposon protein putative unclassified

ISM IDs	Rice gene locus IDs	Physical positions (bp) on chromosomes	Forward primers (5'-3')	Reverse primers (5'-3')	Melting tempearature (T˚C)	Expected product size (bp) in PCR	Expected product size (bp) in Q-PCR	Putative Functions
OsISM06285	LOC_Os01g46660	26571486	ATGCAGGTGACATCAGAAGC	GGGGCAGATCAACAATCACT	59.4	917	92	transposon protein putative unclassified
OsISM06286	LOC_Os01g46660	26572504	GTGGATGCGGGTGTAACTCT	ACAAGACCGCAGATGTAACG	59.4	182	95	transposon protein putative unclassified
OsISM06287	LOC_Os01g46690	26582284	TTCGTGTATGCTCGTTCTGG	TTCTCGTTGATAGCCTTCGC	60.2	630	97	hypothetical protein
OsISM06288	LOC_Os01g46690	26582894	GCGAAGGCTATCAACGAGAA	CAGTGGACCACGAAAGGAGT	60.3	717	99	hypothetical protein
OsISM06289	LOC_Os01g46690	26583814	GCCCGAGTGGTGTTATGAGT	CGATAACATTGTGGTGTGGC	59.9	128	48	hypothetical protein
OsISM06290	LOC_Os01g46690	26584038	CTGACACAGGTGGCATCTTC	TCTGATTCGAGGAACCTTGG	59.7	508	97	hypothetical protein
OsISM06291	LOC_Os01g46690	26584608	AGGCTGCTGCATCTATCGTT	TCGAAGATGGAGATGTAAGAAG C	59.9	194	97	hypothetical protein
OsISM06292	LOC_Os01g46700	26592046	GCTGTGCTTTGTGTGATGCT	ATATCCCTCCTTGCCTCACC	60.2	184	83	SET domain containing protein expressed
OsISM06293	LOC_Os01g46700	26592210	GGTGAGGCAAGGAGGGATA	CTGGAAATCACCAGGTTTGC	60.3	284	92	SET domain containing protein expressed
OsISM06294	LOC_Os01g46700	26592619	AAATGAGCTGGAGTGCCAGT	TTGGAAAGCAATTTCCACAAC	59.9	524	78	SET domain containing protein expressed
OsISM06295	LOC_Os01g46700	26593173	TGGAACAACTTGCTTGCAGA	CCTCCACATTTATGTTGCCA	60.0	193	92	SET domain containing protein expressed
OsISM06296	LOC_Os01g46700	26593346	TGGCAACATAAATGTGGAGG	AACCTTGAGGTTGCTGCACT	59.7	181	94	SET domain containing protein expressed
OsISM06297	LOC_Os01g46700	26593499	TGACAAAAAGTGCAGCAACC	CTTCTTGCTGGACAGATTTGAG	59.5	185	95	SET domain containing protein expressed
OsISM06298	LOC_Os01g46700	26593662	CTCAAATCTGTCCAGCAAGAA G	GCAACTTTGTCAGGCTTGCT	59.9	187	85	SET domain containing protein expressed
OsISM06299	LOC_Os01g46700	26593861	TGGCATGGAAGGAACTTACC	ATGGACCACAATGGCAAGA	59.9	306	57	SET domain containing protein expressed
OsISM06300	LOC_Os01g46700	26594247	ATCAAGGACACGCTTATGCC	TTTTCTGAGAGGAGCGCTTT	59.7	214	92	SET domain containing protein expressed
OsISM06301	LOC_Os01g46700	26594443	AGCGCTCCTCTCAGAAAATG	TGCACATCTTTCTGTTGTCCA	60.0	203	96	SET domain containing protein expressed
OsISM06302	LOC_Os01g46700	26594652	AGATGGGTTGAAGACTGGGA	CACGCCATAGCATTCCTGAT	60.3	183	82	SET domain containing protein expressed

ISM IDs	Rice gene locus IDs	Physical positions (bp) on chromosomes	Forward primers (5'-3')	Reverse primers (5'-3')	Melting tempearature (T˚C)	Expected product size (bp) in PCR	Expected product size (bp) in Q-PCR	Putative Functions
OsISM06303	LOC_Os01g46700	26594834	GAGGGGAAAGCAAGACTTCA	TCCCAATAGCAGGTTCCATC	59.6	575	75	SET domain containing protein expressed
OsISM06304	LOC_Os01g46700	26595389	GATGGAACCTGCTATTGGGA	CACGAACCATGTATTTGCCT	59.2	212	85	SET domain containing protein expressed
OsISM06305	LOC_Os01g46700	26595638	ATCTGTGCATCAAGGGCAG	TGTTTGTTGTCTGTTTGCCG	60.3	202	71	SET domain containing protein expressed
OsISM06306	LOC_Os01g46700	26595820	CGGCAAACAGACAACAAACA	AGTTCCAGCAGGAGTTTGAA	59.1	298	92	SET domain containing protein expressed
OsISM06307	LOC_Os01g46700	26596227	TATCTGCAGCAAGATGTCGG	ACAGGATCCCCTTACACGGT	60.3	589	87	SET domain containing protein expressed
OsISM06308	LOC_Os01g46700	26596787	CAATTGCACACCGTGTAAGG	GAAAAGGACTCTGGATCCCTC	59.6	175	95	SET domain containing protein expressed
OsISM06309	LOC_Os01g46700	26596933	CAGGAAGAGAGGGATCCAGA	ATTCCTGACCTACCAAAGCA	58.3	175	99	SET domain containing protein expressed
OsISM06310	LOC_Os01g46700	26597129	TCGTAGGGGCATTCAAGAAG	GCAACACTTCGTCTGACCTG	59.8	182	67	SET domain containing protein expressed
OsISM06311	LOC_Os01g46700	26597291	CAGGTCAGACGAAGTGTTGC	CATCAACGACTACTTCCTCGC	59.7	645	98	SET domain containing protein expressed
OsISM06312	LOC_Os01g46700	26597901	GCTTTTCAAAATCAGCGAGG	TCCTTGCATAGCAGTTTGGAG	60.2	185	99	SET domain containing protein expressed
OsISM06313	LOC_Os01g46700	26598103	GACGAGAGCCGTATTGTCCT	GCCTCGTCAGGGTCAAATAA	59.7	190	87	SET domain containing protein expressed
OsISM06314	LOC_Os01g46720	26611098	ACTTCGACGTCGCTGACC	CATGCCACCAACCTGCTC	60.6	196	98	expressed protein
OsISM06315	LOC_Os01g46720	26611211	GTGGTGAATTTCTGCTCCGT	ACCGTCGAACCGTACAGC	59.9	167	90	expressed protein
OsISM06316	LOC_Os01g46720	26611391	CTGATGAGCTTTCAGGCGA	TGCATCGCGTAAGAGTGGTA	60.3	182	96	expressed protein
OsISM06317	LOC_Os01g46720	26611614	ACGATGGATCCTTCATCCTG	GGTTGGAAGCCAATCAGAGA	60.0	441	84	expressed protein
OsISM06318	LOC_Os01g46720	26612114	AGGAGGACCTGCTGTAACCC	AGCCCATGGCTATGCAGATA	60.6	172	91	expressed protein
OsISM06319	LOC_Os01g46720	26612307	GGAGAATGTTCATGTAAGCCC	GAAAATCAACAGCATTCCCAA	59.2	1571	69	expressed protein
OsISM06320	LOC_Os01g46720	26613919	GAAAGGAGAAAAATGATGATTGC	TTGCCATGATAAAGCCAACA	59.6	189	56	expressed protein

ISM IDs	Rice gene locus IDs	Physical positions (bp) on chromosomes	Forward primers (5'-3')	Reverse primers (5'-3')	Melting tempearature (T˚C)	Expected product size (bp) in PCR	Expected product size (bp) in Q-PCR	Putative Functions
OsISM06321	LOC_Os01g46720	26614099	TCATGGCAAAGATGGTTCTG	GCAGACATGCTGGGTCATC	59.9	426	94	expressed protein
OsISM06322	LOC_Os01g46720	26614560	CCATCCGGGAATATTGGTCT	TTTGTCAGGTTTTGTTGCCA	60.5	190	91	expressed protein
OsISM06323	LOC_Os01g46720	26614730	TGGCAACAAAACCTGACAAA	AACTGATGGATTTATGCGCC	60.0	215	90	expressed protein
OsISM06324	LOC_Os01g46720	26614966	GGTTCTACTGCTGTCCGCAT	GGGTTTAGCACCTCCATCAA	60.1	327	96	expressed protein
OsISM06325	LOC_Os01g46720	26615273	TTGATGGAGGTGCTAAACCC	AGACGCCAGTGACCAGGTAG	60.1	443	69	expressed protein
OsISM06327	LOC_Os01g46740	26621916	GGGCAGAGGAATGAAAATGA	CCATCGATGATAGGTGCCAT	60.4	1004	91	hypothetical protein
OsISM06328	LOC_Os01g46750	26628107	TGGACTGCTGCTACGACATC	GAGGTACCTTCCCGTCAACA	60.0	818	94	AMP-binding enzyme putative expressed
OsISM06329	LOC_Os01g46750	26628885	ATGCTTGGTCACCGTGAGAT	TCGAAAGGTCAAACACCTCC	60.3	172	80	AMP-binding enzyme putative expressed
OsISM06330	LOC_Os01g46750	26629036	AGGAGGTGTTTGACCTTTCG	CACCATAGATACCACAACGGC	59.7	275	85	AMP-binding enzyme putative expressed
OsISM06331	LOC_Os01g46750	26629290	GCCGTTGTGGTATCTATGGTG	CGCAGTAGATTCCATGAGCA	60.1	268	79	AMP-binding enzyme putative expressed
OsISM06332	LOC_Os01g46750	26629538	TGCTCATGGAATCTACTGCG	TCTCAACTTCTGCATGGCAC	60.0	525	84	AMP-binding enzyme putative expressed
OsISM06333	LOC_Os01g46750	26630043	GTGCCATGCAGAAGTTGAGA	TCAGCTGACTTGGGGAAAGT	59.9	602	79	AMP-binding enzyme putative expressed
OsISM06334	LOC_Os01g46750	26630619	TTCAAGACTTTCCCCAAGTCA	TTGGAGGCATTTTCCTTTTG	59.9	168	87	AMP-binding enzyme putative expressed
OsISM06335	LOC_Os01g46750	26630781	CTCCAAATATGGGCTGGAGA	TTGCTTTCACTGGAAGATCG	59.5	476	81	AMP-binding enzyme putative expressed
OsISM06336	LOC_Os01g46750	26631357	AGATCGGCTGCTTAACTGTG	GCTCTTCCACAACACGATCA	59.0	188	99	AMP-binding enzyme putative expressed
OsISM06337	LOC_Os01g46750	26631560	GTGCTTCCATTGGATTTTGG	CGTGGAACAGCACACAAGAT	60.0	174	89	AMP-binding enzyme putative expressed
OsISM06338	LOC_Os01g46750	26631714	ATCTTGTGTGCTGTTCCACG	TTTATGAAACCCCCAGATGC	59.8	736	78	AMP-binding enzyme putative expressed
OsISM06339	LOC_Os01g46750	26632425	AAATTGCATCTGGGGGTTTC	GGCAGCTTCATTGTGCTTAG	59.9	307	99	AMP-binding enzyme putative expressed

ISM IDs	Rice gene locus IDs	Physical positions (bp) on chromosomes	Forward primers (5'-3')	Reverse primers (5'-3')	Melting tempearature (T˚C)	Expected product size (bp) in PCR	Expected product size (bp) in Q-PCR	Putative Functions
OsISM06340	LOC_Os01g46750	26632721	ATGAAGCTGCCGCAATATGT	TTTCCCCCAAGACCTTCTTT	60.3	155	62	AMP-binding enzyme putative expressed
OsISM06341	LOC_Os01g46750	26632898	GCTGCTCCACTTGCTACTCA	GATCCAGCACAAGTCTCCGT	59.8	1170	96	AMP-binding enzyme putative expressed
OsISM06342	LOC_Os01g46750	26634262	TTTTGATTGATGGATGGTTCC	ACTTCCATCAGGTTGCCACT	59.6	424	57	AMP-binding enzyme putative expressed
OsISM06343	LOC_Os01g46750	26634756	TGGAGAACATTTATGGTCTTGTTT	TTGACCACAGCAACAAGGAA	59.6	254	86	AMP-binding enzyme putative expressed
OsISM06344	LOC_Os01g46750	26635108	TGGGAGAACTGTCAAAAGTAGGA	CAAATGGCACTGGTTCAAGA	59.9	202	85	AMP-binding enzyme putative expressed
OsISM06345	LOC_Os01g46750	26635340	ATAAAAAGAAGCGCCCTCAA	GGATGTTCTAGACGGCATGG	59.7	351	97	AMP-binding enzyme putative expressed
OsISM06347	LOC_Os01g46760	26656224	TCCTCGGTGTCCTTGCTACT	TCCTGTCCATGACTGTGACC	59.7	479	91	protein phosphatase 2C putative expressed
OsISM06348	LOC_Os01g46770	26668292	GTGGTGACGAGAGGCTTGG	GCAAGAAGGAGACGAGCG	60.8	171	97	transposon protein putative unclassified
OsISM06349	LOC_Os01g46840	26718945	TGAAGGACAACAAACTGGGA	GGCCCTTTAAGCTTACCGTC	59.6	1827	97	expressed protein
OsISM06350	LOC_Os01g46850	26721887	CACCTTGGTGAATGCTCAGG	GAACATCAGCACATAGGCGA	60.7	1519	86	expressed protein
OsISM06351	LOC_Os01g46880	26744165	CGTACAAGCTGAGATCCGTG	GTGTTGCCACCTGATTCTCC	60.0	383	99	retrotransposon protein putative unclassified
OsISM06352	LOC_Os01g46880	26745158	CATCCACGAAGGACATACGA	AGGAATTCTTCTGGGTCGGT	59.7	155	85	retrotransposon protein putative unclassified
OsISM06353	LOC_Os01g46880	26745310	CCTTCAGGTCTACTCCACGG	GTGGCGCTTGTAAGTTCCTT	59.5	190	95	retrotransposon protein putative unclassified
OsISM06354	LOC_Os01g46880	26746592	GCCGCATCTCACTACGTGTAT	TTGTGCTCAATCACCTCCCT	60.4	373	81	retrotransposon protein putative unclassified
OsISM06355	LOC_Os01g46900	26762962	AAACCGCCGAAGGATCTATT	GGTAAAGCCCCTTGATCTCC	59.9	618	62	transposon protein putative Mariner sub-class
OsISM06356	LOC_Os01g46900	26763701	TTTGATCTCAACATGGTGGC	GCTCAAATTGCAATGCTTCA	59.7	907	71	transposon protein putative Mariner sub-class
OsISM06357	LOC_Os01g46900	26765401	TGGAAAGATAGGCATTTGGC	TACGACTTCTCCTTCGGGCT	60.7	148	57	transposon protein putative Mariner sub-class
OsISM06358	LOC_Os01g46940	26789387	GTCCAAGAAGGAGGTGGTGA	GCCACCGTCTTTCTCACTTG	60.5	642	98	sphingolipid C4-hydroxylase SUR2 putative expressed

ISM IDs	Rice gene locus IDs	Physical positions (bp) on chromosomes	Forward primers (5'-3')	Reverse primers (5'-3')	Melting tempearature (T˚C)	Expected product size (bp) in PCR	Expected product size (bp) in Q-PCR	Putative Functions
OsISM06359	LOC_Os01g46980	26811101	CAGCGAGATCTCCGTCTCC	TTCCGTTCATATTCTTGCCTG	60.6	1798	97	vacuolar ATP synthase subunit E putative expressed
OsISM06360	LOC_Os01g46980	26812893	ACGGAAAGAGAAGCAAGTCG	TTGATGCGAGAAGCATTCAG	59.9	717	70	vacuolar ATP synthase subunit E putative expressed
OsISM06361	LOC_Os01g46980	26813680	AGCCACAACCACCATGAATA	AAGTACCGCTGGCTCTTTCA	59.4	437	82	vacuolar ATP synthase subunit E putative expressed
OsISM06362	LOC_Os01g46980	26814227	CGATGTGTACCTACCGCCTT	GAGAAGCCAGCACAACACCT	60.2	685	78	vacuolar ATP synthase subunit E putative expressed
OsISM06363	LOC_Os01g46980	26814935	AACACACTTGATGCCAGGCT	CATGCACAGATTATGCCGTC	60.4	310	95	vacuolar ATP synthase subunit E putative expressed
OsISM06364	LOC_Os01g47000	26838049	GTCCATCTGGACAGGCAAGT	GTGGTTCCTGTCAATCCCAC	60.2	452	75	transposon protein putative unclassified
OsISM06366	LOC_Os01g47000	26839012	CTAGCAAGGCTTGGTTGCTC	AACCACAACAGGTACGCCTC	60.1	184	98	transposon protein putative unclassified
OsISM06367	LOC_Os01g47000	26839506	ACGATGGGGACTTACTGGTG	GGAGACGGTCAAACTCGAAC	59.8	190	95	transposon protein putative unclassified
OsISM06368	LOC_Os01g47000	26839868	CGGACTTAGGCAGGTGTTTC	CTAGCTGTCCCCGTCGAGT	60.1	159	81	transposon protein putative unclassified
OsISM06369	LOC_Os01g47000	26840235	GCACCGTGACCGAGACTACT	GCAAACCTCTGCTCAACCTC	60.2	187	93	transposon protein putative unclassified
OsISM06370	LOC_Os01g47000	26840575	GACCTCGTTTGTCTTCGAGC	AGTACCGAAGATCCCGGCT	60.5	377	93	transposon protein putative unclassified
OsISM06371	LOC_Os01g47000	26841153	TACTCCCAGGGGCACATTAG	GCCGAAAGTGTGTTGTTGTG	60.1	235	98	transposon protein putative unclassified
OsISM06372	LOC_Os01g47020	26850531	AAGGTGGTCATTCAACCGAC	GATCTTCGGCCTCGGACT	60.1	183	87	transposon protein putative unclassified
OsISM06374	LOC_Os01g47060	26876725	CAAACCCTTTGGCTTCTCAC	CCCCACGAAGTTGCAGTAAT	59.9	358	90	expressed protein
OsISM06375	LOC_Os01g47060	26877229	GGTTCAAGATGTGCCATGTG	GTTGAGGGTGGATGGAAGGT	60.6	683	79	expressed protein
OsISM06377	LOC_Os01g47140	26944305	GGTTGTTCAGAATGGTTGGG	CTCCTGGACAAAACCCTCAA	60.1	1061	85	retrotransposon protein putative Ty3-gypsy subclass
OsISM06378	LOC_Os01g47160	26954972	CTCTGCTCGATGGTAGCGT	GCGAAGATGGCCAGTATCAT	59.6	286	83	retrotransposon protein putative unclassified
OsISM06379	LOC_Os01g47160	26956182	CCCTCCAAAGTGGTCGTTTA	GGATCCTCGGCCTCAGACT	60.8	191	94	retrotransposon protein putative unclassified

ISM IDs	Rice gene locus IDs	Physical positions (bp) on chromosomes	Forward primers (5'-3')	Reverse primers (5'-3')	Melting tempearature (T˚C)	Expected product size (bp) in PCR	Expected product size (bp) in Q-PCR	Putative Functions
OsISM06380	LOC_Os01g47160	26957197	CGGAGCTAGGGAAGAAGCTC	CCAGATCCCTCTCCACGAC	60.6	166	61	retrotransposon protein putative unclassified
OsISM06381	LOC_Os01g47190	26970846	CCGCCAACGAGATCAACC	TAGGCTCTTCCCTTCAAACG	61.0	238	87	phosphoglycerate mutase putative expressed
OsISM06382	LOC_Os01g47190	26971064	CGTTTGAAGGGAAGAGCCTA	AGGAGGTGGCTTGATGTCTG	59.9	810	78	phosphoglycerate mutase putative expressed
OsISM06383	LOC_Os01g47190	26972015	GCTGGGTTGATAGGGCTAGA	TATGCTGGCTGGATCAACAG	59.6	282	85	phosphoglycerate mutase putative expressed
OsISM06384	LOC_Os01g47200	26975560	CTGGTCCGGTGACTCCTTC	CAACTGGAACCCCGAGTAAG	60.1	186	89	retrotransposon protein putative unclassified expressed
OsISM06385	LOC_Os01g47200	26975788	CGAGACCCATCAGGGAAGTA	AGTGCTGCTGAAGATGCTGA	60.0	829	81	retrotransposon protein putative unclassified expressed
OsISM06386	LOC_Os01g47240	26992143	CCCTCCAAGGTGGTCATTTA	GACTTGAGATGGTGGGAGGA	59.9	171	70	transposon protein putative unclassified
OsISM06387	LOC_Os01g47256	27003334	GTGCAATGGAATCGAGGAAG	GTCTGGTTGGTCATCTCGG	59.8	194	98	exosome complex exonuclease RRP40 putative expressed
OsISM06388	LOC_Os01g47256	27003515	TGACCAACCAGACCATCAAG	GTAGCCTGTATGGTGTCGCA	59.6	172	62	exosome complex exonuclease RRP40 putative expressed
OsISM06389	LOC_Os01g47256	27003708	TTCGAAGCCCAGCAAATACT	GGTGTCAACCACAACACCAA	60.1	604	89	exosome complex exonuclease RRP40 putative expressed
OsISM06390	LOC_Os01g47256	27004292	TTGGTGTTGTGGTTGACACC	TGCAAGAACTGGTAAAAAGGC	59.9	327	81	exosome complex exonuclease RRP40 putative expressed
OsISM06391	LOC_Os01g47256	27004597	GGCCTTTTTACCAGTTCTTGC	GCTTTCACCACTCGTGCATA	60.0	664	94	exosome complex exonuclease RRP40 putative expressed
OsISM06392	LOC_Os01g47256	27005241	TATGCACGAGTGGTGAAAGC	TTAGTTGACCAAATTCGGCTG	60.0	320	89	exosome complex exonuclease RRP40 putative expressed
OsISM06393	LOC_Os01g47256	27005575	TTTGACACGTCTACTGGCCT	CAAGGGCCTCTAGAATTGGA	58.8	145	68	exosome complex exonuclease RRP40 putative expressed
OsISM06394	LOC_Os01g47256	27005738	AGATTGCTGTTGGCCTGAAT	CGCATTTGATACAACAATGACA	59.6	164	75	exosome complex exonuclease RRP40 putative expressed
OsISM06395	LOC_Os01g47262	27008446	GTCTGGTGCCAAGACCTAGC	GGATGCGTCCCACATTTATT	59.8	158	69	AMSH-like protease putative expressed
OsISM06396	LOC_Os01g47262	27008824	TCTTCAGTGGGGCAGTCTTC	TCTCCTTTGCAAGTTCGATG	59.7	196	92	AMSH-like protease putative expressed
OsISM06397	LOC_Os01g47262	27009042	TTGTGGAATCCTAGGTGCTTC	TGAGCAGTTGCTTCTTGCTT	59.1	194	82	AMSH-like protease putative expressed

ISM IDs	Rice gene locus IDs	Physical positions (bp) on chromosomes	Forward primers (5'-3')	Reverse primers (5'-3')	Melting tempearature (T˚C)	Expected product size (bp) in PCR	Expected product size (bp) in Q-PCR	Putative Functions
OsISM06398	LOC_Os01g47262	27009216	AAGCAAGAAGCAACTGCTCA	GGGTAAAGTGATTGCTCCGA	59.5	219	81	AMSH-like protease putative expressed
OsISM06399	LOC_Os01g47262	27009415	TCGGAGCAATCACTTTACCC	GAAACACGTTTGTGAAGGGTG	60.3	229	58	AMSH-like protease putative expressed
OsISM06400	LOC_Os01g47262	27009620	ACACACCCTTCACAAACGTG	GGAGCAATCACAATCGCAAC	60.5	830	99	AMSH-like protease putative expressed
OsISM06401	LOC_Os01g47262	27010439	GTGATTGCTCCCACTGATCC	CACTCCCTAAGCACACCCAT	60.2	201	81	AMSH-like protease putative expressed
OsISM06403	LOC_Os01g47310	27036622	GCTGATTCGCTTGGTAGGAC	CACCCTTCAACTCCAGATTCA	60.0	700	96	hypothetical protein
OsISM06404	LOC_Os01g47380	27066754	TGTGCCTTCGCTTGTATGAG	CAGTGTCGTTGTGGTTGCTT	59.9	893	94	hypothetical protein
OsISM06406	LOC_Os01g47400	27081200	CGGCAACTACAACGAGAACA	TCCTAGCCTCAGACAGCACA	59.8	378	57	OsMan01 - Endo-Beta-Mannanase expressed
OsISM06407	LOC_Os01g47400	27081684	CGACGATGAGTTCTTCACCA	CGTGTTCTTCCTCGTCAACA	59.9	1743	80	OsMan01 - Endo-Beta-Mannanase expressed
OsISM06408	LOC_Os01g47400	27083709	ACCGTCCACTCCTACCCC	AGCCACCTCCCCATGAAC	60.3	197	75	OsMan01 - Endo-Beta-Mannanase expressed
OsISM06410	LOC_Os01g47420	27094729	TATCCAAGAACCTTCCGCTG	CGTCTTGACTTGCATACATCG	59.8	926	64	pterin-4-alpha-carbinolamine dehydratase putative expressed
OsISM06411	LOC_Os01g47420	27095674	AAGATGGTGCCATGGTTCTC	AGCAGAATCTTCTGGCATGG	60.1	1557	98	pterin-4-alpha-carbinolamine dehydratase putative expressed
OsISM06412	LOC_Os01g47420	27097206	ACATGCCATGCCAGAAGATT	GAATGTCACCTTCGGTTGCT	60.3	158	78	pterin-4-alpha-carbinolamine dehydratase putative expressed
OsISM06413	LOC_Os01g47420	27097418	TTCAGCTTGTTGCTGCTGTT	TTTCACATTGTTCCAACCGA	59.9	702	75	pterin-4-alpha-carbinolamine dehydratase putative expressed
OsISM06414	LOC_Os01g47420	27098100	TCGGTTGGAACAATGTGAAA	CTTCGCAGCAAGGATGAAAT	60.1	833	81	pterin-4-alpha-carbinolamine dehydratase putative expressed
OsISM06415	LOC_Os01g47450	27114917	GGTCAACGAGGGGTTCCT	GGGATCTGATTGAGGTTTGG	59.6	300	99	OsCttP1 - Putative C-terminal processing peptidase homologue expressed
OsISM06416	LOC_Os01g47450	27115242	GAAAATGCTGGCTAACCTGG	AATCCTATTCCCGTCATGTCA	59.5	798	94	OsCttP1 - Putative C-terminal processing peptidase homologue expressed
OsISM06417	LOC_Os01g47450	27116113	GCCCACTCTGCTGGTGTTAG	CCTTGCAACATGGATGACAC	60.4	342	99	OsCttP1 - Putative C-terminal processing peptidase homologue expressed
OsISM06418	LOC_Os01g47450	27116435	GTGTCATCCATGTTGCAAGG	GGCTCAACAGGACCACAGTT	60.1	178	78	OsCttP1 - Putative C-terminal processing peptidase homologue expressed

ISM IDs	Rice gene locus IDs	Physical positions (bp) on chromosomes	Forward primers (5'-3')	Reverse primers (5'-3')	Melting tempearature (T˚C)	Expected product size (bp) in PCR	Expected product size (bp) in Q-PCR	Putative Functions
OsISM06419	LOC_Os01g47450	27116715	ATTCAATGCAGTGGCCAAA	TGTCCCTCAAATCCAAAACA	59.5	547	90	OsCttP1 - Putative C-terminal processing peptidase homologue expressed
OsISM06420	LOC_Os01g47450	27117254	GAGGGACAATCTTGGTGGAC	TTCAGGAAGAGCTTTGCAGTC	59.6	272	58	OsCttP1 - Putative C-terminal processing peptidase homologue expressed
OsISM06421	LOC_Os01g47450	27117493	GGCTGGGATAGAGACTGCAA	GCCAGGAAGTACCTTCATTTTG	60.2	240	95	OsCttP1 - Putative C-terminal processing peptidase homologue expressed
OsISM06422	LOC_Os01g47450	27117669	GTGCCCACCTCAGCTAAAAG	GACGATCACGACCAGCAGTA	59.9	431	78	OsCttP1 - Putative C-terminal processing peptidase homologue expressed
OsISM06423	LOC_Os01g47450	27118115	TTGTTGCTGAAAGGGAACCT	TTTCACTGGCACTTGCTGTC	59.9	466	76	OsCttP1 - Putative C-terminal processing peptidase homologue expressed
OsISM06424	LOC_Os01g47450	27118555	TAACAGGACAGCAAGTGCCA	TTCACCGACCAGAACAGCTT	60.6	154	74	OsCttP1 - Putative C-terminal processing peptidase homologue expressed
OsISM06425	LOC_Os01g47450	27118690	AGCTGTTCTGGTCGGTGAAA	CATACTTGCCCACTGTGACG	60.5	184	99	OsCttP1 - Putative C-terminal processing peptidase homologue expressed
OsISM06426	LOC_Os01g47450	27118863	GGGCAAGTATGTGACACCAA	AAAGGTACTCGGTGGCTTCA	59.6	181	99	OsCttP1 - Putative C-terminal processing peptidase homologue expressed
OsISM06427	LOC_Os01g47460	27124273	GAAAGCCGGCTGATAAGTGA	TTCAAATAGCAGTCCCCCAG	60.2	1243	79	expressed protein
OsISM06428	LOC_Os01g47460	27125620	CAAAGGCCCTTCATCCAATA	TGTTTGACTTCCACCAGCAC	59.8	176	77	expressed protein
OsISM06429	LOC_Os01g47460	27125815	TAATTGCACATTTGATCGGC	TCATCTGCTTCACCAAAGACA	59.5	1045	77	expressed protein
OsISM06430	LOC_Os01g47460	27126839	TGTCTTTGGTGAAGCAGATGA	TTTCTTGATTCAATATGATGCTGAC	59.5	232	93	expressed protein
OsISM06431	LOC_Os01g47460	27127016	CGCAGGAGAAATCATGAAGA	TGCAGAGACCATTTAACTCTGA	58.3	387	99	expressed protein
OsISM06432	LOC_Os01g47460	27127522	GTGGCCGCTTGTTCACTATT	ATTGTGGGTGATACGAAGGC	60.0	161	84	expressed protein
OsISM06433	LOC_Os01g47490	27145875	TGAGGCTCGAAGGAGATGTT	TGCTTCCTGAACATATTCGC	59.4	698	93	Lg106 putative expressed
OsISM06434	LOC_Os01g47490	27146565	AGGAAGCAGCCATCAAGAAA	GCAGAATCAAAGTAAGCCCG	59.9	232	92	Lg106 putative expressed
OsISM06435	LOC_Os01g47490	27146710	GGTTAAGTTGCATTTCTTACTGAGC	CAAGCAAAAGGAAAAACATGG	59.7	206	98	Lg106 putative expressed
OsISM06436	LOC_Os01g47490	27146898	TGTTTTTCCTTTTGCTTGCC	TGAAAGTGGATTGGGACACA	60.1	529	78	Lg106 putative expressed

ISM IDs	Rice gene locus IDs	Physical positions (bp) on chromosomes	Forward primers (5'-3')	Reverse primers (5'-3')	Melting tempearature (T˚C)	Expected product size (bp) in PCR	Expected product size (bp) in Q-PCR	Putative Functions
OsISM06437	LOC_Os01g47490	27147412	CCCAATCCACTTTCAGCCTA	ATCCTCAGGAGGCAAATTCA	59.8	193	98	Lg106 putative expressed
OsISM06438	LOC_Os01g47500	27151438	GCTCAGCAGCGTGGAGAC	AGCGTCGTCATCATCGAACT	60.9	374	89	hypothetical protein
OsISM06439	LOC_Os01g47520	27158235	GTGGAACCGGATTCACTTGT	TGAAGGGAACAAGTGTGCAA	60.1	287	96	hypothetical protein
OsISM06440	LOC_Os01g47520	27158528	AAGCTTCGAATGCTGTCCAT	ATCAAACGGCCAAAAAGATG	59.9	830	50	hypothetical protein
OsISM06441	LOC_Os01g47520	27159400	GCCAAAAGGAAAGCTGCATA	ATTACCGGAGGGAGTACGTG	59.6	1147	94	hypothetical protein
OsISM06442	LOC_Os01g47520	27160490	AGAAATTCCAATTTGCACGG	TTGAACTTGGAGTCTTTCGGA	59.9	660	94	hypothetical protein
OsISM06443	LOC_Os01g47530	27171031	AGACGATGCAGACGGAGC	AAGAAATCCATCTCTGGCGA	59.9	1349	62	CGMC MAPKCMGC 2.6 - CGMC includes CDA MAPK GSK3 and CLKC kinases expressed
OsISM06444	LOC_Os01g47530	27172732	CAGATGCTTCGTGCTTTGAA	TGCCAATATGTTCTTTGGCTT	59.9	676	76	CGMC MAPKCMGC 2.6 - CGMC includes CDA MAPK GSK3 and CLKC kinases expressed
OsISM06445	LOC_Os01g47530	27173441	GGTCTAGCACGAGTGGCATT	AACCACACAACTCAGGAGCC	60.2	208	95	CGMC MAPKCMGC 2.6 - CGMC includes CDA MAPK GSK3 and CLKC kinases expressed
OsISM06446	LOC_Os01g47530	27173629	GGCTCCTGAGTTGTGTGGTT	CAGCAAAAATGCAGCCAATA	60.0	1241	78	CGMC MAPKCMGC 2.6 - CGMC includes CDA MAPK GSK3 and CLKC kinases expressed
OsISM06447	LOC_Os01g47530	27174905	AATGTGGTTCACCAGTTGGA	CTCCTTGCTTTCTCATTCCG	59.4	174	90	CGMC MAPKCMGC 2.6 - CGMC includes CDA MAPK GSK3 and CLKC kinases expressed
OsISM06448	LOC_Os01g47530	27175188	AAGGATAGACCAACTGCCGA	TTTGTTATTGGCTGGCATGA	59.9	224	93	CGMC MAPKCMGC 2.6 - CGMC includes CDA MAPK GSK3 and CLKC kinases expressed
OsISM06449	LOC_Os01g47530	27175508	GGATTACATGAATGGAACCGA	CCCTCCATTTTCTTCCAGGT	60.0	225	95	CGMC MAPKCMGC 2.6 - CGMC includes CDA MAPK GSK3 and CLKC kinases expressed
OsISM06450	LOC_Os01g47530	27175726	TGGAGGGAAGAATGGAGATG	TGATCTTTGGGAGGAATTGG	59.9	365	97	CGMC MAPKCMGC 2.6 - CGMC includes CDA MAPK GSK3 and CLKC kinases expressed
OsISM06451	LOC_Os01g47530	27176105	CCCAAGTACCCCAAAGGATT	CCTTCGTTGATTGTATGGGC	60.2	185	99	CGMC MAPKCMGC 2.6 - CGMC includes CDA MAPK GSK3 and CLKC kinases expressed
OsISM06452	LOC_Os01g47550	27185238	ATACCCGATTGGGTCAACG	CGTCCAAGATAACAGGCACA	60.2	2128	68	kinase pfkB family putative expressed
OsISM06453	LOC_Os01g47550	27187450	TGACTGGGATGCCTACAGAA	TGATCCGAGTTTAACCAGCA	59.3	1604	96	kinase pfkB family putative expressed
OsISM06454	LOC_Os01g47550	27189154	CCTCAGCTTTTGCTGTAGCTC	GGCTCCCTTTACTTGAACACA	59.3	205	99	kinase pfkB family putative expressed

ISM IDs	Rice gene locus IDs	Physical positions (bp) on chromosomes	Forward primers (5'-3')	Reverse primers (5'-3')	Melting tempearature (T˚C)	Expected product size (bp) in PCR	Expected product size (bp) in Q-PCR	Putative Functions
OsISM06455	LOC_Os01g47600	27235732	CCCACCGCCTTCTTCTACT	TTTCTTCCTTGTAAGTTTTCCGA	59.3	1100	53	HMG1/2 putative expressed
OsISM06456	LOC_Os01g47600	27236791	CTGCTGAAGGGAGGATTTTC	TTGTATTCCATTTCTCGCCA	59.0	171	99	HMG1/2 putative expressed
OsISM06457	LOC_Os01g47600	27236938	CATGTGGCGAGAAATGGAAT	CATACTCTGCACGCTTCTCG	60.3	1520	82	HMG1/2 putative expressed
OsISM06458	LOC_Os01g47600	27238448	GCAGAGTATGAGAAGGCGGT	CAACTACCCGACGTTCACCT	59.7	193	99	HMG1/2 putative expressed
OsISM06459	LOC_Os01g47630	27245397	GTATCCCCGGTGCAGAGAT	TGTTAAGCCATTTGGCAACA	60.0	787	68	expressed protein
OsISM06460	LOC_Os01g47630	27246196	GTTCTGAAGTTTCTTCGGCG	GCCATTGTGATTAGTTGCCA	59.8	163	66	expressed protein
OsISM06461	LOC_Os01g47650	27257246	CATCCCTACGGATTTCTCCA	AAAGAAGCACGCTGTCCAAT	59.9	679	99	vacuolar protein sorting-associated protein 16 putative expressed
OsISM06462	LOC_Os01g47650	27258113	GCATTGCTGTATGATGCTAGAGA	CCTCAGGCAAGGAAGAAGAA	59.5	360	89	vacuolar protein sorting-associated protein 16 putative expressed
OsISM06463	LOC_Os01g47650	27258521	GCCAACATGCGTTACTGAGA	CCCGTAATGTTTTGCACATTT	59.8	1933	94	vacuolar protein sorting-associated protein 16 putative expressed
OsISM06464	LOC_Os01g47650	27260487	TGCCACTTACCATTCAGCAG	CAGACGTCCAATGAGTACCG	59.5	142	57	vacuolar protein sorting-associated protein 16 putative expressed
OsISM06465	LOC_Os01g47650	27260652	GGCACTTCGCATCTCTGAGT	CATGCCCAATGCATAATCAC	60.2	470	61	vacuolar protein sorting-associated protein 16 putative expressed
OsISM06466	LOC_Os01g47650	27261162	CTTCTTGAGGGCTTGCTTGA	CTTTCGCCTGCCACTGTTAT	60.5	207	91	vacuolar protein sorting-associated protein 16 putative expressed
OsISM06467	LOC_Os01g47650	27261385	TTGACCATGAGTCCCAATCA	TATCCCCACTCTCAATTGCC	59.9	783	94	vacuolar protein sorting-associated protein 16 putative expressed
OsISM06468	LOC_Os01g47650	27262175	TGTGTACCTTGTGCTTTTCCA	TGCATTGATTACGCCAAAAA	59.7	267	83	vacuolar protein sorting-associated protein 16 putative expressed
OsISM06469	LOC_Os01g47650	27262445	CCTTTGGCTCGTGATTTGTT	GCACCTACCTGAAGTCTCCCT	59.9	194	96	vacuolar protein sorting-associated protein 16 putative expressed
OsISM06470	LOC_Os01g47650	27262577	CATTCCAAGCATGAAGCTCT	TTCCTCTCCAATTCCCTTGA	58.8	309	99	vacuolar protein sorting-associated protein 16 putative expressed
OsISM06471	LOC_Os01g47650	27262992	GCTTCTGAAGAGCATGCAAA	CGCTGACACTAGAACCCACA	59.6	727	89	vacuolar protein sorting-associated protein 16 putative expressed
OsISM06472	LOC_Os01g47650	27263753	ACGAGCTGCACTCAAAGTGA	TCCCAGTTCCCAACTGTAGC	59.9	177	94	vacuolar protein sorting-associated protein 16 putative expressed

ISM IDs	Rice gene locus IDs	Physical positions (bp) on chromosomes	Forward primers (5'-3')	Reverse primers (5'-3')	Melting tempearature (T°C)	Expected product size (bp) in PCR	Expected product size (bp) in Q-PCR	Putative Functions
OsISM06473	LOC_Os01g47650	27263910	GCTACAGTTGGGAACTGGGA	CAGCATCAATACATGCTTCCA	59.9	252	95	vacuolar protein sorting-associated protein 16 putative expressed
OsISM06474	LOC_Os01g47650	27264190	TCCAAAGTTGACAGATCCTCG	TTCCTTTGCCATCTTGATCC	60.1	790	62	vacuolar protein sorting-associated protein 16 putative expressed
OsISM06476	LOC_Os01g47730	27300861	ACGACAGGCTCGTCAAGG	GTAGTAGGCGCTCGTGATGG	60.4	475	75	ras-related protein putative expressed
OsISM06477	LOC_Os01g47770	27336206	CACATGTGTATGCCAGAGCC	TTTGGTCCCACTCAAAGACC	60.0	200	99	CS domain containing protein putative expressed
OsISM06478	LOC_Os01g47770	27336478	AGGCCACTCATGTTGAGGTC	TCTTCACTGGGAGTGTCAGG	59.5	194	73	CS domain containing protein putative expressed
OsISM06479	LOC_Os01g47770	27336638	TTTTTGCAGCATGACCTGAC	TTTGCAGTGTGATGTGCATTT	60.0	657	86	CS domain containing protein putative expressed
OsISM06480	LOC_Os01g47770	27337351	CCATATGCTGCAGACCAAGA	GTGAACTGAGCCTGGGAGAA	60.1	574	90	CS domain containing protein putative expressed
OsISM06481	LOC_Os01g47800	27356736	GGAGGGAAACGGTGATATTC	CCCACAAATCAATTCTAGGCA	59.1	1469	99	AAA-type ATPase family protein putative expressed
OsISM06482	LOC_Os01g47800	27358317	TTCGTTGGTTGATCAGATGG	TCCACTAGGGAGATCTTGAAACT T	59.6	1068	92	AAA-type ATPase family protein putative expressed
OsISM06483	LOC_Os01g47800	27360269	TGATATCATGAGCAGCTGGG	TTGTGGATGACTTTCCAACAG	59.2	278	99	AAA-type ATPase family protein putative expressed
OsISM06484	LOC_Os01g47830	27379697	TTTTGCCTCTGGTCTATGGG	GTAAAATTGGGTTACCGGGG	60.2	395	93	retrotransposon protein putative unclassified
OsISM06485	LOC_Os01g47830	27381249	TACGAGCCTCTTTGGACCAC	TTGCTAGATGTGTGGCATCC	60.0	169	92	retrotransposon protein putative unclassified
OsISM06487	LOC_Os01g47850	27394369	TGACCTCAACCAGTGACCAG	GTTGTGGATAGTCGGGGAGA	59.8	314	99	transposon protein putative unclassified
OsISM06488	LOC_Os01g47860	27396263	CTCTACTTGGCTACGACCCC	GTTGTTAGGGGCGGATGAC	59.6	254	55	retrotransposon protein putative unclassified
OsISM06489	LOC_Os01g47940	27438117	CCCTCCAAAGTGGTCTTTCA	ATTGGCCAGACTTGGAATGA	60.3	177	78	transposon protein putative unclassified
OsISM06490	LOC_Os01g47970	27449376	GGCAAGGAGGAATGAGAAGG	CGTCGAGCTTTTTCCCTAGT	59.8	1146	58	retrotransposon protein putative unclassified
OsISM06491	LOC_Os01g48010	27482075	GCGCTGGCTCTTGTTCGT	TATTCTCCTGGTCCTCGGTG	61.4	2488	71	expressed protein
OsISM06494	LOC_Os01g48150	27570237	CTCTGCGTCCCGTGACCT	TCAACTTAACAGCGAGATTTTTG A	61.4	651	84	expressed protein

ISM IDs	Rice gene locus IDs	Physical positions (bp) on chromosomes	Forward primers (5'-3')	Reverse primers (5'-3')	Melting tempearature (T°C)	Expected product size (bp) in PCR	Expected product size (bp) in Q-PCR	Putative Functions
OsISM06495	LOC_Os01g48160	27576770	GCCTCTGCATTAGCCTACCA	GCCTCATCACCACCATTCTC	60.4	523	87	hypothetical protein
OsISM06496	LOC_Os01g48170	27581255	TGATGAAGAGCCCCTACCAG	AGGTCTCCGACGGGTACTG	60.2	190	70	homeobox domain containing protein
OsISM06498	LOC_Os01g48180	27586775	ACGGCTCTCGAGTGGATAGA	CCTTTGCAATTTTTGCTTCC	59.8	157	55	DDT domain containing protein putative expressed
OsISM06499	LOC_Os01g48180	27586923	TTGCAAAGGAAGTTGAAGCTC	ATCTCCTTGCGCATCTGTTC	60.0	177	98	DDT domain containing protein putative expressed
OsISM06500	LOC_Os01g48180	27587175	CCTAAGAGAGCAAAGGCGTG	CTTTCGGAGCTCCTCCTTTT	60.1	199	98	DDT domain containing protein putative expressed
OsISM06501	LOC_Os01g48180	27587535	ATTGCAGCAGCTTGATTCCT	CGAAAATGGCTCCTTCAGTC	59.9	545	77	DDT domain containing protein putative expressed
OsISM06502	LOC_Os01g48180	27588098	TCTGAGGATAATGTTGGGAACC	GCAACACCTCTCCTATAGACCTG	59.8	334	96	DDT domain containing protein putative expressed
OsISM06503	LOC_Os01g48180	27588392	CATTAGCATCACTTCATCAGGTC	GGATGCAGCATTTACCATCTG	59.6	298	93	DDT domain containing protein putative expressed
OsISM06504	LOC_Os01g48180	27588600	TCATTTCATGCACTAAATCTGG	GCAAAAGTAATCGAGAATTTCC	57.4	210	99	DDT domain containing protein putative expressed
OsISM06505	LOC_Os01g48180	27588810	TGATGTTCTCGGGCTTTCTT	CAGCAGAGCAATGTGCAGTT	60.0	672	98	DDT domain containing protein putative expressed
OsISM06506	LOC_Os01g48180	27589532	ATTAGCGGTGAATCCAGCAG	AAGAACATTCAGGTGGCGTT	59.9	205	98	DDT domain containing protein putative expressed
OsISM06507	LOC_Os01g48180	27589776	GGTTTTGGGCCTCAGTTAAA	TTTCGGAGGGTGGATATGAC	59.4	1296	99	DDT domain containing protein putative expressed
OsISM06508	LOC_Os01g48180	27591185	TGCTGTCCCTTGAAGGAAGT	CAGGCGTCTTGCTTGTTGT	59.9	194	97	DDT domain containing protein putative expressed
OsISM06509	LOC_Os01g48180	27591990	GCCCTGGTTGCACTTGTTAG	AAGGCATTTGCTGATTCCAG	60.5	163	84	DDT domain containing protein putative expressed
OsISM06510	LOC_Os01g48180	27592634	TGCAGAGATGGATACTGGAGG	GAATGCAATTGTGCTTCCCT	60.2	998	99	DDT domain containing protein putative expressed
OsISM06511	LOC_Os01g48180	27594121	GAAGATGCAAACTGCAGATCA	TTGAATTGCTTCTGGTGGAA	59.1	199	98	DDT domain containing protein putative expressed
OsISM06512	LOC_Os01g48180	27594351	GGGGTGTGAAGTTGCACTCT	TCTGAAGATAGGTGGTCCCG	60.1	1857	95	DDT domain containing protein putative expressed
OsISM06513	LOC_Os01g48180	27596382	GATGGAGAGAAATGGTGGAGA	CGGTTGTTTCCAGTGGATCT	59.5	319	90	DDT domain containing protein putative expressed

ISM IDs	Rice gene locus IDs	Physical positions (bp) on chromosomes	Forward primers (5'-3')	Reverse primers (5'-3')	Melting tempearature (T°C)	Expected product size (bp) in PCR	Expected product size (bp) in Q-PCR	Putative Functions
OsISM06514	LOC_Os01g48190	27600833	AGCACTGGATCTCGAGCCT	ATCTCCACCTCCTCCATTCC	60.2	197	98	drought induced 19 protein putative expressed
OsISM06515	LOC_Os01g48190	27601116	ACTGCTACGAGGACCACGAC	CTTTTCGAAGCAGATGGGAC	60.1	1248	99	drought induced 19 protein putative expressed
OsISM06516	LOC_Os01g48190	27602347	CCATCTGCTTCGAAAAGATTACA	AATATCGAATCTGCGCATCC	60.3	217	99	drought induced 19 protein putative expressed
OsISM06517	LOC_Os01g48190	27602784	AGGATACTCCTGCTCAGCCA	GCTTGTTTCCGCTTTTGTTC	59.9	660	80	drought induced 19 protein putative expressed
OsISM06518	LOC_Os01g48210	27624214	GGTTCAGGAGCCCTATGCT	GCAGCTGTTCGTGAAGATTG	59.4	508	95	PAP/25A associated domain containing protein putative expressed
OsISM06519	LOC_Os01g48210	27624720	GCTGTGAAAGCTGTCTCCAA	GCCCTGTTCTAAAAGATCCG	59.0	178	80	PAP/25A associated domain containing protein putative expressed
OsISM06520	LOC_Os01g48210	27624878	CGGATCTTTTAGAACAGGGC	ACAGGCCAACTTGTGGAGTC	59.5	643	90	PAP/25A associated domain containing protein putative expressed
OsISM06521	LOC_Os01g48210	27625539	TGTCTCAGAAAGGGGTTGCT	AACTTCACAATTGGCACACG	59.7	378	65	PAP/25A associated domain containing protein putative expressed
OsISM06522	LOC_Os01g48210	27625897	CGTGTGCCAATTGTGAAGTT	TGAAGTCTGCTGCTTGTGGT	59.6	182	92	PAP/25A associated domain containing protein putative expressed
OsISM06523	LOC_Os01g48210	27626059	ACCACAAGCAGCAGACTTCA	CACAGATGTCTTAAAGCAGGAAA	59.1	179	61	PAP/25A associated domain containing protein putative expressed
OsISM06524	LOC_Os01g48210	27626215	TTTCCTGCTTTAAGACATCTGTG	ATGAACCAATTCCACCCGTA	59.3	192	89	PAP/25A associated domain containing protein putative expressed
OsISM06525	LOC_Os01g48210	27626387	TACGGGTGGAATTGGTTCAT	CCAGTATATCTTTGCCTCCCC	59.9	357	81	PAP/25A associated domain containing protein putative expressed
OsISM06526	LOC_Os01g48210	27626756	AGGAGCACAATTTGGGGATT	AATTGTTCAACTTGCGTCCA	59.9	340	64	PAP/25A associated domain containing protein putative expressed
OsISM06527	LOC_Os01g48210	27627087	TGAACAATTGGGATGTTGGA	TATGCTCGATCAGGATTTGC	59.3	429	89	PAP/25A associated domain containing protein putative expressed
OsISM06528	LOC_Os01g48210	27627490	AGCTTTGCAAATCCTGATCG	TGAGTTCTTCCCAATATCATTATCTG	60.1	171	79	PAP/25A associated domain containing protein putative expressed
OsISM06529	LOC_Os01g48210	27627623	TGGCCTAGGTACCAGATAATGA	TCGCATCGGTTAACACAGAA	59.4	966	97	PAP/25A associated domain containing protein putative expressed
OsISM06530	LOC_Os01g48339	27703302	CTTGGAGATCGGCATCATCT	AACGGCTCGATCTACTCGAA	60.1	167	88	expressed protein
OsISM06531	LOC_Os01g48360	27713731	AGGAAGAGAGGGGGATCGT	ATTCCTCAACCATCCATCCA	60.1	216	99	expressed protein

ISM IDs	Rice gene locus IDs	Physical positions (bp) on chromosomes	Forward primers (5'-3')	Reverse primers (5'-3')	Melting tempearature (T˚C)	Expected product size (bp) in PCR	Expected product size (bp) in Q-PCR	Putative Functions
OsISM06532	LOC_Os01g48380	27725593	GGGTGATATTGATTATAACAAGGC	GCATCTAGATAACATGATCACAAAAA	58.3	888	92	PPR repeat domain containing protein putative expressed
OsISM06533	LOC_Os01g48380	27726575	GCATCTTGCTTTCCTATACATTTTT	CAACAATATAAGTCTATAACAGCGGTA	58.3	414	99	PPR repeat domain containing protein putative expressed
OsISM06535	LOC_Os01g48380	27727208	GATGTTCTTGGGAAGGCAAA	TCTTTTACAAGACCAGATTGGC	59.2	221	95	PPR repeat domain containing protein putative expressed
OsISM06536	LOC_Os01g48380	27727493	AATTGGGATCCTAGGTTGGAA	AACATCGATGCAGAAATGGTC	59.8	470	76	PPR repeat domain containing protein putative expressed
OsISM06537	LOC_Os01g48380	27727966	CTTACCTGGATTCTGACAAGCA	GCTGCATAAAGAATTGTGCC	58.9	165	92	PPR repeat domain containing protein putative expressed
OsISM06538	LOC_Os01g48380	27728180	TGTGGCAAGTACAACTTGGTG	TTCCAGCCTATTGGTTCATCT	59.2	704	91	PPR repeat domain containing protein putative expressed
OsISM06539	LOC_Os01g48380	27729209	CGAAATGTGCAAATACTGCTC	TCCTTTTAAATCTAGTGAGGTTTGTTC	59.0	621	91	PPR repeat domain containing protein putative expressed
OsISM06540	LOC_Os01g48390	27732854	CGCTGGTTCTTCATTTCGTC	CCCTCGACTGAAACTTGAGC	60.4	189	79	expressed protein
OsISM06541	LOC_Os01g48390	27733087	GATGGCGATCCTTGCAGTT	TCTGGACCCAAGGTACCTCTTA	60.6	1722	99	expressed protein
OsISM06542	LOC_Os01g48390	27734790	GAGGTACCTTGGGTCCAGAA	TCCTTCGGTATTGGTCCATC	59.4	590	89	expressed protein
OsISM06543	LOC_Os01g48390	27735439	AAGTTCCGCAGGAGATAGCA	GAATTTTGGATGAGAGGCCA	60.0	172	86	expressed protein
OsISM06544	LOC_Os01g48390	27735657	AGGGGCGATGTGTATCAAAG	TCTTCTGATGAAGAATCAGACTCG	60.0	170	57	expressed protein
OsISM06545	LOC_Os01g48390	27735817	CATCAGAAGAGAACACAAAAGACAA	CGCCGGCTAAGTTGTACAGT	60.1	185	92	expressed protein
OsISM06546	LOC_Os01g48390	27736300	CTCAGTGGACAGCTGCAGAA	CACAAGCACCTTCCAGCTCT	60.5	616	74	expressed protein
OsISM06547	LOC_Os01g48390	27737035	AAGCATTCCGAAGGGAGATT	CCCAAGCAGGTTGATGAAGT	60.1	212	79	expressed protein
OsISM06548	LOC_Os01g48390	27737295	GCACCCAATGGAACACTCTA	ATGCAGTATGCTACCCCCAT	58.9	185	99	expressed protein
OsISM06549	LOC_Os01g48390	27737541	TTGTTGTCTGAGGATGGAGC	GTCATCGTTCTTGGGCATTT	59.4	408	88	expressed protein
OsISM06550	LOC_Os01g48390	27738046	ATTCTGAACACAAGGGCTCC	TTCCGGTCATCTTTAATGCAC	59.5	171	59	expressed protein

ISM IDs	Rice gene locus IDs	Physical positions (bp) on chromosomes	Forward primers (5'-3')	Reverse primers (5'-3')	Melting tempearature (T˚C)	Expected product size (bp) in PCR	Expected product size (bp) in Q-PCR	Putative Functions
OsISM06551	LOC_Os01g48410	27741905	GAAGAACGTCCGTGACAGTG	GCGAACTTCTCCTTCACCAG	59.6	158	58	ATROPGEF7/ROPGEF7 putative expressed
OsISM06552	LOC_Os01g48410	27742126	CTCCAACGCCATCACAAATC	CTTGTTCCATCGAGCTCTCC	60.7	199	92	ATROPGEF7/ROPGEF7 putative expressed
OsISM06553	LOC_Os01g48410	27742358	ATCGTCGAGTTCGTCCCTT	GAGAAGATCTCTGCGCTGCT	59.8	169	85	ATROPGEF7/ROPGEF7 putative expressed
OsISM06554	LOC_Os01g48410	27742541	ATTGCGAAAACTCGACGC	CACCCTCATCTGCGTCTTTT	60.1	194	93	ATROPGEF7/ROPGEF7 putative expressed
OsISM06555	LOC_Os01g48410	27742910	CCCGGGAGAGTACATTGAAA	CGAGTCTCCAAGACTAGACCTTC	59.3	246	56	ATROPGEF7/ROPGEF7 putative expressed
OsISM06556	LOC_Os01g48410	27743371	CATCCTCAAGCACCAGTTCC	CTCTCCAAGATGGCATACCC	60.1	197	94	ATROPGEF7/ROPGEF7 putative expressed
OsISM06558	LOC_Os01g48444	27777089	AGGACCTCTCCATGGCTCTC	GATGATTTACGCAAACCGCT	60.4	585	71	OsIAA5 - Auxin-responsive Aux/IAA gene family member expressed
OsISM06559	LOC_Os01g48444	27777751	AGGATGCTGACTGGATGCTT	GATTTTCAATTTCCGGCAGA	59.9	1427	72	OsIAA5 - Auxin-responsive Aux/IAA gene family member expressed
OsISM06560	LOC_Os01g48444	27779158	TCTGCCGGAAATTGAAAATC	GCTCTGCCCTGACTGCTCTA	60.4	460	78	OsIAA5 - Auxin-responsive Aux/IAA gene family member expressed
OsISM06561	LOC_Os01g48470	27784909	GAAGAAGAGCTTAAACTGAACGAA	CCCTGGCATCAACTTCTCAT	59.3	151	79	RUNX1 protein putative
OsISM06562	LOC_Os01g48480	27786907	CGCACCGTCAGCTGAGTA	ACCAATAACGAACTCGGTGG	59.8	220	74	retrotransposon protein putative Ty3-gypsy subclass
OsISM06563	LOC_Os01g48480	27787757	CTGGCCGTCAGCTCAGTAGT	TTCAAGTCTTGGGAATTCGG	60.3	126	49	retrotransposon protein putative Ty3-gypsy subclass
OsISM06564	LOC_Os01g48480	27788164	TATCCTCCCTGTTGGCATTC	CCACTATTTCTCTGGCGAGG	59.9	454	76	retrotransposon protein putative Ty3-gypsy subclass
OsISM06565	LOC_Os01g48480	27788860	CCATTGAGCAAGCTCTGGAT	GCAGCCAATTTCTTCCTCAC	60.1	254	95	retrotransposon protein putative Ty3-gypsy subclass
OsISM06566	LOC_Os01g48480	27789102	GAAATTGGCTGCAACAACCT	TATTCGCTGCACAGGAGATG	60.0	137	71	retrotransposon protein putative Ty3-gypsy subclass
OsISM06567	LOC_Os01g48480	27789256	GTCGGCTGCAGATAATCACA	TCAAATGAGAACAGATCGGC	59.3	169	85	retrotransposon protein putative Ty3-gypsy subclass
OsISM06568	LOC_Os01g48530	27833179	GGGAAGCTTGATCCACAGAA	CGTACGTCTCCTCGTTCTCC	60.0	968	64	expressed protein
OsISM06569	LOC_Os01g48530	27834118	ATGGCATATGGAGAACGAGG	GTAATGTGGGGCACCATAGA	59.1	240	97	expressed protein

ISM IDs	Rice gene locus IDs	Physical positions (bp) on chromosomes	Forward primers (5'-3')	Reverse primers (5'-3')	Melting tempearature (T˚C)	Expected product size (bp) in PCR	Expected product size (bp) in Q-PCR	Putative Functions
OsISM06570	LOC_Os01g48530	27834366	TCCTAGCATCACATTGCCAC	CATACTTGTCGAATCTGCCG	59.5	228	92	expressed protein
OsISM06571	LOC_Os01g48540	27839703	AGTTCGACTACAACACCGGC	AGCACCACGTGTCCGTCA	61.3	161	58	glyoxal oxidase-related putative expressed
OsISM06572	LOC_Os01g48540	27839967	GAAGGAGTTTCCCAAGAGCC	GCCGATCACGATGAAACTG	60.2	180	83	glyoxal oxidase-related putative expressed
OsISM06573	LOC_Os01g48570	27851581	CGGGTTCTTGGCGATATG	AAGCCAGGATCCACTGAAGA	59.9	383	99	expressed protein
OsISM06574	LOC_Os01g48570	27851983	CCTTCAAATGGCGCTATCAT	CAAATGACATCCACAGCGAG	60.2	234	75	expressed protein
OsISM06575	LOC_Os01g48570	27852352	TCTACCTCAGCCTGATCGGT	TGCGGCGAAATATGATGTAA	59.9	212	97	expressed protein
OsISM06576	LOC_Os01g48570	27852635	GGCGCACTGAACACAAAGTA	CCACTGCAACCTGCATTGTA	60.3	186	62	expressed protein
OsISM06577	LOC_Os01g48570	27852796	CAAGGTACAATGCAGGTTGC	TGGTGTATCATCAGCCCTCA	59.6	162	64	expressed protein
OsISM06578	LOC_Os01g48570	27852977	TATTGGTGCGTGAATGGGTA	GGGGTACAACTGGAAGATGG	59.5	196	94	expressed protein
OsISM06579	LOC_Os01g48570	27853186	TTGGGAGATTGCTATGCCTC	TGTGCATACCACTCCGACAT	60.1	227	98	expressed protein
OsISM06580	LOC_Os01g48570	27853374	CTCGTATCAGACCACTGGCA	CAATGGCTGTGAAGGACAAC	59.5	165	70	expressed protein
OsISM06581	LOC_Os01g48570	27853561	GTCCAAAGGTTTCCACTGCT	CTTACCAAGTATCAGCCGCC	59.5	904	81	expressed protein
OsISM06582	LOC_Os01g48580	27856915	CTCGCCTGTACCCTCTTCC	CCCCCAATGATAAGCTTCCT	60.0	200	72	ubiquitin-conjugating enzyme E2 putative
OsISM06583	LOC_Os01g48580	27857161	GAGTTCTGCTCGCCAAAAAC	CACGCGATACGTGGTAGAAA	59.9	848	63	ubiquitin-conjugating enzyme E2 putative
OsISM06584	LOC_Os01g48580	27858113	GATTTCTACCACGTATCGCGT	AATAGAACTTGGGCCAGCAA	59.4	1322	91	ubiquitin-conjugating enzyme E2 putative
OsISM06585	LOC_Os01g48580	27859717	TCGAACCCAAGAGAAAGGAA	CCATTCATGTTGAATCCGCT	60.3	158	76	ubiquitin-conjugating enzyme E2 putative
OsISM06586	LOC_Os01g48580	27859851	GTGAAGCGGATTCAACATGA	GCAGCTCCAACCTATCCTCA	60.0	254	89	ubiquitin-conjugating enzyme E2 putative
OsISM06587	LOC_Os01g48580	27860142	CCATGACGGGCTATTCTTTT	GATTAAGCCGCATACCTCCA	59.6	203	96	ubiquitin-conjugating enzyme E2 putative

ISM IDs	Rice gene locus IDs	Physical positions (bp) on chromosomes	Forward primers (5'-3')	Reverse primers (5'-3')	Melting tempearature (T°C)	Expected product size (bp) in PCR	Expected product size (bp) in Q-PCR	Putative Functions
OsISM06588	LOC_Os01g48580	27860593	TATTCTCTTCGCAATCCACC	TCCATATAGGCATTGCATCC	58.1	317	99	ubiquitin-conjugating enzyme E2 putative
OsISM06590	LOC_Os01g48690	27921718	AGATGCGCACCAGGATGA	AGGTGTGGCATCAGCTTTCT	60.6	1097	69	ribosomal protein rpS20 putative expressed
OsISM06592	LOC_Os01g48720	27938105	GTGAAGAACTCGCTCCTCGT	CCAGGTAGTGCGTTACCGTC	60.1	186	95	RNA polymerase III subunit RPC82 family protein putative expressed
OsISM06593	LOC_Os01g48720	27938310	GCACAGACAGCGTTTCTCAA	GAAGCAATCCTTCAAGCAGC	60.1	1249	81	RNA polymerase III subunit RPC82 family protein putative expressed
OsISM06594	LOC_Os01g48720	27939582	CAACTTGTGGAGCGAACAAT	TATTTCCTCCCTTGCTGGTG	59.4	226	64	RNA polymerase III subunit RPC82 family protein putative expressed
OsISM06595	LOC_Os01g48720	27939903	CTCGTCAAGGAAACGTGCTC	TCTGCATCAGAAAGAGCTGC	60.2	536	88	RNA polymerase III subunit RPC82 family protein putative expressed
OsISM06596	LOC_Os01g48720	27940466	GGAAGATGCATCCAATGCTAA	GAATCGAGCTCTGCATCTCC	60.1	292	94	RNA polymerase III subunit RPC82 family protein putative expressed
OsISM06597	LOC_Os01g48720	27940769	AGAACGAAGTACTTTGGCGG	AGGTTTCTTTCGGTCTGCAC	59.4	501	81	RNA polymerase III subunit RPC82 family protein putative expressed
OsISM06598	LOC_Os01g48720	27941311	CGAGGCCAGTTTAATGGAAA	TCCTTCTGTCCTAGCCTCTCC	60.0	548	79	RNA polymerase III subunit RPC82 family protein putative expressed
OsISM06599	LOC_Os01g48720	27941924	CCTCAGAGGACCCAGATTCA	CCTCATTTCGAGATGCTTCA	59.6	1107	64	RNA polymerase III subunit RPC82 family protein putative expressed
OsISM06600	LOC_Os01g48720	27943002	CAGAATTGTTGAAGCATCTCG	TGTAAAAGCTTCTTGCCCGT	59.2	189	74	RNA polymerase III subunit RPC82 family protein putative expressed
OsISM06601	LOC_Os01g48720	27943216	GCGGTCCAGTTGAGACAGA	TCGATGTAGTCGTCCTTCCA	59.6	303	98	RNA polymerase III subunit RPC82 family protein putative expressed
OsISM06602	LOC_Os01g48720	27943497	TTTGGAAGGACGACTACATCG	TTGTGTGTTCCCTGTTCCTG	59.8	1338	63	RNA polymerase III subunit RPC82 family protein putative expressed
OsISM06603	LOC_Os01g48720	27944899	CTTTAAACCTGCGGCAAATG	TTCCGCAACTTTGTGTCATC	60.2	733	77	RNA polymerase III subunit RPC82 family protein putative expressed
OsISM06604	LOC_Os01g48760	27960884	AGCTCCTCTTCTCCCACCAC	CCTCTGCGTGTACGAGATCA	60.4	134	51	THAP domain-containing protein 4 putative expressed
OsISM06605	LOC_Os01g48760	27961086	CTCCGTCGAGGTTGTCATCT	GCTTTGGAGCGTCACTGTTT	60.4	2579	92	THAP domain-containing protein 4 putative expressed
OsISM06606	LOC_Os01g48760	27963645	AAACAGTGACGCTCCAAAGC	TGAGAGCTCCCCATCTACCA	60.6	415	90	THAP domain-containing protein 4 putative expressed
OsISM06607	LOC_Os01g48780	27977256	GAAAACGGGGAAGATTGGTT	AACTTTCCGGACCACACAAA	60.3	1041	88	hypothetical protein

ISM IDs	Rice gene locus IDs	Physical positions (bp) on chromosomes	Forward primers (5'-3')	Reverse primers (5'-3')	Melting tempearature (T°C)	Expected product size (bp) in PCR	Expected product size (bp) in Q-PCR	Putative Functions
OsISM06608	LOC_Os01g48790	27982933	GTAGCCCCGGGTTCTTCT	AAGTTCTCCATTGCTTCTTGC	58.8	1117	95	YT521-B-like family domain containing protein expressed
OsISM06609	LOC_Os01g48790	27984024	GCTATGCAAGAAGCAATGGA	GAACTTGCGCCTTTTGTAGC	59.5	236	87	YT521-B-like family domain containing protein expressed
OsISM06610	LOC_Os01g48790	27984342	GGGGACCAAGGGATGTATTA	TGCACCTCCAGTCCATTGTA	59.3	211	99	YT521-B-like family domain containing protein expressed
OsISM06611	LOC_Os01g48790	27984526	CGTTGGATACAATGGACTGG	CATTTTCATACCCCGGAAGA	59.3	857	87	YT521-B-like family domain containing protein expressed
OsISM06612	LOC_Os01g48790	27985739	AATGGCTATCCATCCTCGAA	TGGTTAGTATGCACCGCAAA	59.8	187	96	YT521-B-like family domain containing protein expressed
OsISM06613	LOC_Os01g48790	27986337	TGCTGAGAAAGGAACCAAATG	AAATCTACCGGACCGACCAT	60.4	419	97	YT521-B-like family domain containing protein expressed
OsISM06614	LOC_Os01g48790	27986840	TCCACAATTTCGCCACATAA	GCATCTCTGAACCTTGTGGA	59.4	536	99	YT521-B-like family domain containing protein expressed
OsISM06615	LOC_Os01g48790	27987469	GCAAGCCACTTGCTACAACA	TTGGCCTCTTAGTTTCTTCAGG	60.0	1019	70	YT521-B-like family domain containing protein expressed
OsISM06616	LOC_Os01g48810	28008278	ACCTCGACAAGCTGCCCT	CCGGTTCATCTGGTCTTCTG	60.8	723	93	transcription initiation factor TFIID subunit 11 putative expressed
OsISM06617	LOC_Os01g48810	28009008	CATTCCGCAGATCTGGATTC	TTGACTGCCAGTAATGCTCG	60.3	1013	66	transcription initiation factor TFIID subunit 11 putative expressed
OsISM06618	LOC_Os01g48810	28010067	TGTTTGTTGGTGAGCTTGTTG	TAGGCTTCTCTGATGTGGCA	59.7	335	92	transcription initiation factor TFIID subunit 11 putative expressed
OsISM06619	LOC_Os01g48900	28056453	TATGGACAAGGGCACAAATG	CGATTGACAACACCAATCCA	59.9	214	82	dynamin-related protein 1A putative
OsISM06620	LOC_Os01g48900	28056771	TGGGATCAGAGCACTTAGCA	TGGATCCCTGGAATTCTTGA	60.0	175	74	dynamin-related protein 1A putative
OsISM06621	LOC_Os01g48900	28056995	CTCGGAAAACCTATTGCTGC	CATGCGGCATATTTCCATAA	59.6	331	64	dynamin-related protein 1A putative
OsISM06622	LOC_Os01g48900	28057319	CCGCATGTTCGATGGTATCT	GAGCAACCGGAAATTGATTG	60.7	222	96	dynamin-related protein 1A putative
OsISM06623	LOC_Os01g48900	28057677	TATTAGAGGTCCGGCTGAGG	CTCACTGATAGCCTTGCGAA	59.2	175	77	dynamin-related protein 1A putative
OsISM06624	LOC_Os01g48900	28057832	TTCGCAAGGCTATCAGTGAG	ACGGAGAGTGGGGAATTGAT	59.9	400	51	dynamin-related protein 1A putative
OsISM06625	LOC_Os01g48900	28058369	GAAGGGTGGAAATCCAACAA	CCATATTGACATATGCCAGAACA	59.8	317	93	dynamin-related protein 1A putative

ISM IDs	Rice gene locus IDs	Physical positions (bp) on chromosomes	Forward primers (5'-3')	Reverse primers (5'-3')	Melting tempearature (T˚C)	Expected product size (bp) in PCR	Expected product size (bp) in Q-PCR	Putative Functions
OsISM06626	LOC_Os01g48900	28058754	ACATTGCTCGATCGCTTCTT	TTAGCTCTACGCTCCATCACC	59.7	574	99	dynamin-related protein 1A putative
OsISM06627	LOC_Os01g48930	28080055	GTGGGGAAGATGCCAAGAAG	TCCATTTCACTGTCACTATCGC	61.0	588	89	splicing factor-related putative expressed
OsISM06628	LOC_Os01g48930	28081079	CAGGAACCACTGGACCTGAC	AGCTTGAGTCTCTCCATGCC	60.1	885	72	splicing factor-related putative expressed
OsISM06629	LOC_Os01g48970	28108680	TGGTCTGCAGTTGTTGCTCT	AAAGGATGCAGACCCTTCCT	59.8	3668	87	hypothetical protein
OsISM06630	LOC_Os01g48970	28112325	TTTAGGAAGGGTCTGCATCC	GGCCGTGAGGCAATAATAAA	59.5	341	98	hypothetical protein
OsISM06632	LOC_Os01g48990	28126612	AGCACAGGGCCAAGTACAAG	GTCTTTCCACTGCCTTGGG	60.5	435	80	uncharacterized kinase mug58 putative expressed
OsISM06633	LOC_Os01g48990	28127065	TGATTATCTTTTTCGGGTTGC	TGTTCTGCTGCCGTCAAGTA	59.8	160	76	uncharacterized kinase mug58 putative expressed
OsISM06634	LOC_Os01g48990	28127156	GACTTTTCCGTGCCTTAGGA	TCCAGGATTTCTTTCCCTCA	59.5	190	95	uncharacterized kinase mug58 putative expressed
OsISM06635	LOC_Os01g48990	28127313	TTCCAGGGTAAATTGAGGGA	ATCATGGCTTCCAGCATTTC	59.7	162	73	uncharacterized kinase mug58 putative expressed
OsISM06636	LOC_Os01g48990	28127489	TGAAACACTTGAATCGCTGC	GCGACGATAACTGCAACCTT	60.1	256	92	uncharacterized kinase mug58 putative expressed
OsISM06637	LOC_Os01g48990	28127693	GAAGATGAAGCTTCCACGGT	CTCTGGCCATGTTGAAGGAT	59.7	158	77	uncharacterized kinase mug58 putative expressed
OsISM06638	LOC_Os01g48990	28127831	ATCCTTCAACATGGCCAGAG	ATCCAAGCATCCATCCTTCA	60.2	155	67	uncharacterized kinase mug58 putative expressed
OsISM06639	LOC_Os01g48990	28127978	GCTTGGATTCAAACCTCTTCC	CATGCATCGTAGTATGCCTCA	59.9	176	94	uncharacterized kinase mug58 putative expressed
OsISM06640	LOC_Os01g48990	28128201	GTTGCGTGTACCAGTGGAGA	TCAGACATTCCTGGTTTCCC	59.8	158	68	uncharacterized kinase mug58 putative expressed
OsISM06641	LOC_Os01g48990	28128339	GGGAAACCAGGAATGTCTGA	AGGTAGCGCGACACAAAATC	60.1	560	51	uncharacterized kinase mug58 putative expressed
OsISM06642	LOC_Os01g49000	28135510	GGGACTCTACGACACCTCCA	CATTTTGTGCGAATCAGAGC	59.8	907	97	katanin p60 ATPase-containing subunit putative expressed
OsISM06643	LOC_Os01g49000	28136802	GGGAGGAAAGGGAAGTCAAG	TTCATATTGCCCCTTCTTGG	60.0	2546	85	katanin p60 ATPase-containing subunit putative expressed
OsISM06644	LOC_Os01g49000	28139473	TCCTCTCTGGATGCCTGAAT	CCAGTACCAGGTGGACCAAA	60.3	446	79	katanin p60 ATPase-containing subunit putative expressed

ISM IDs	Rice gene locus IDs	Physical positions (bp) on chromosomes	Forward primers (5'-3')	Reverse primers (5'-3')	Melting tempearature (T˚C)	Expected product size (bp) in PCR	Expected product size (bp) in Q-PCR	Putative Functions
OsISM06645	LOC_Os01g49000	28140045	TGCAAGGTTCTATGCTCCAA	CCTCCTTGATGACTCATGCTC	59.6	433	95	katanin p60 ATPase-containing subunit putative expressed
OsISM06646	LOC_Os01g49000	28140562	CTGGCAGCTACCAACTTTCC	AAATACGCTTCTCCAGCCTC	59.2	650	65	katanin p60 ATPase-containing subunit putative expressed
OsISM06647	LOC_Os01g49000	28141231	TGAAAGCAGAAAGGCACTCA	CTTCTGTCCTCCGTGCAACT	60.1	496	90	katanin p60 ATPase-containing subunit putative expressed
OsISM06649	LOC_Os01g49010	28161136	TACGCTATGGGTTGTTTCGG	CCACTGATATTGCATCAGGA	59.0	442	87	ORC4 - Putative origin recognition complex subunit 4 expressed
OsISM06650	LOC_Os01g49010	28161505	TTCAATTCTTCGCAGGTGGT	ATGTAGCATCCCATTCAGCC	60.3	391	94	ORC4 - Putative origin recognition complex subunit 4 expressed
OsISM06651	LOC_Os01g49010	28161870	AGATCAGGCTGAATGGGATG	AAACAAAGTTGCCTCGCAAT	59.9	250	71	ORC4 - Putative origin recognition complex subunit 4 expressed
OsISM06652	LOC_Os01g49010	28162093	TAAGGAAATTGCGAGGCAAC	TGAATTCCGTGTTGTCATCTG	59.9	162	81	ORC4 - Putative origin recognition complex subunit 4 expressed
OsISM06653	LOC_Os01g49010	28162241	CAACACGGAATTCATGATTGA	CAACCTGAGCAAAGAGGTCA	59.2	198	96	ORC4 - Putative origin recognition complex subunit 4 expressed
OsISM06654	LOC_Os01g49010	28162407	CCTAGAAGAGTTTGACCTCTTTGC	GCTTGTGAGGTGAGAGATTGC	60.0	277	85	ORC4 - Putative origin recognition complex subunit 4 expressed
OsISM06655	LOC_Os01g49010	28162657	TGCAATGCAATCTCTCACCT	CCGGGATCTAACCCTTTTCT	59.4	384	92	ORC4 - Putative origin recognition complex subunit 4 expressed
OsISM06656	LOC_Os01g49010	28163027	GGGTTAGATCCCGGTTCTCT	AAGTGCTAGCAAGTGCTCCAT	59.3	185	90	ORC4 - Putative origin recognition complex subunit 4 expressed
OsISM06657	LOC_Os01g49010	28163242	GTCAGGGAGTACAATGCACG	GTTGTTGCGTCAGCATCAGT	59.5	621	90	ORC4 - Putative origin recognition complex subunit 4 expressed
OsISM06658	LOC_Os01g49010	28163846	GATGCTGACGCAACAACAAG	GCTTTGCATTGACAGAAGTCC	60.2	190	91	ORC4 - Putative origin recognition complex subunit 4 expressed
OsISM06659	LOC_Os01g49010	28164037	TCATGAATGCGCTATCTTCG	CCTGTTCATGCATACAAGAATGT	59.5	254	96	ORC4 - Putative origin recognition complex subunit 4 expressed
OsISM06660	LOC_Os01g49010	28164271	TTCTTGTATGCATGAACAGGCT	TTGTAGGCATCCTGCACAGA	60.1	172	95	ORC4 - Putative origin recognition complex subunit 4 expressed
OsISM06661	LOC_Os01g49010	28164430	AGGATGCCTACAAAACGTCG	ATCAACTCGCGATCCAAAAG	60.2	176	80	ORC4 - Putative origin recognition complex subunit 4 expressed
OsISM06662	LOC_Os01g49010	28164677	GTGAGCTTGCTGAGTCCCTC	TGTATCTTTCACGGTCAAGAAGC	60.4	214	76	ORC4 - Putative origin recognition complex subunit 4 expressed
OsISM06663	LOC_Os01g49026	28172923	ATCCTCCACTCGGAGAACCT	CGGCAACAACTCCACTGATA	59.9	676	86	transmembrane protein 93 putative expressed

ISM IDs	Rice gene locus IDs	Physical positions (bp) on chromosomes	Forward primers (5'-3')	Reverse primers (5'-3')	Melting tempearature (T˚C)	Expected product size (bp) in PCR	Expected product size (bp) in Q-PCR	Putative Functions
OsISM06664	LOC_Os01g49026	28173731	TTGAAGGAGTTTTTGGTGGC	CAGCATCAAGGATACGTCCA	59.9	657	59	transmembrane protein 93 putative expressed
OsISM06665	LOC_Os01g49026	28174344	TTTTTGCAGTCTTTTGTGCTG	TTTATGCCGTGCATCAGAAG	59.5	370	71	transmembrane protein 93 putative expressed
OsISM06667	LOC_Os01g49050	28179886	CATCGGGGTAATGTGTGTCA	CCCAAATCACCTCAAACCAC	60.2	230	96	hypothetical protein
OsISM06668	LOC_Os01g49050	28180056	TGCTAATCGACTTTGTGTGC	CAGTGCTCAAGGCTGCAATA	58.8	420	97	hypothetical protein
OsISM06669	LOC_Os01g49060	28189463	CCACTTTGAGCAAATCCTCC	ACTGTGTCGGGTGCAAGAAC	60.4	217	66	prenyltransferase putative expressed
OsISM06670	LOC_Os01g49060	28189898	ATTCTTTGCCCTGACATGCT	CAATTGCAGCTCATCCTTGA	59.8	243	94	prenyltransferase putative expressed
OsISM06671	LOC_Os01g49060	28190168	AAAAACGAAAGCACCTGGAG	CATCCTGGTTGTGCTCTGAA	59.6	386	65	prenyltransferase putative expressed
OsISM06672	LOC_Os01g49060	28190531	GGTTTCAGAGCACAACCAGG	CGCACGGTAGTTCATCTTTG	60.0	337	95	prenyltransferase putative expressed
OsISM06673	LOC_Os01g49060	28190878	GCTGCTGGCTTATTCCCTAC	TGTCAGCGACATGTAACTCGT	59.2	279	88	prenyltransferase putative expressed
OsISM06674	LOC_Os01g49060	28191134	GAACGAGTTACATGTCGCTGA	TCTTCTGTGTCCTCTACATGGC	59.1	241	94	prenyltransferase putative expressed
OsISM06675	LOC_Os01g49060	28191363	GGACACAGAAGATTCCCTTGA	TGGTAGCGCCTGATAAGTTTC	59.3	263	91	prenyltransferase putative expressed
OsISM06676	LOC_Os01g49060	28191609	CTTATCAGGCGCTACCAAGG	TCCTCACTGCTGAGCAAGAA	59.9	194	90	prenyltransferase putative expressed
OsISM06677	LOC_Os01g49060	28191903	GAGGCATGTGTCCAATCTGA	GCTTTAGTTTCACAGCCGGA	60.0	191	77	prenyltransferase putative expressed
OsISM06678	LOC_Os01g49080	28208146	CTTCAACGGCTGGAGGTG	CGTAGCACCACGTCTCCTCT	60.4	1069	99	hypothetical protein
OsISM06679	LOC_Os01g49080	28209198	GGAGACGTGGTGCTACGATT	AGCCTAAATTGGTTGTCCCC	60.2	688	72	hypothetical protein
OsISM06680	LOC_Os01g49090	28218008	GTTTAGGGTGGTGGCGACT	TGGAACACATCAATTTCCCC	60.3	1139	89	retrotransposon protein putative unclassified
OsISM06681	LOC_Os01g49110	28231302	ATTCTTTGCCCTGACATGCT	CAATTGCAGCTCATCCTTGA	59.8	243	94	prenyltransferase putative expressed
OsISM06682	LOC_Os01g49110	28231552	TGATCCTTTCATGCTCACCA	TCCTGGTTGTGTTCTGAAACC	60.1	533	83	prenyltransferase putative expressed

ISM IDs	Rice gene locus IDs	Physical positions (bp) on chromosomes	Forward primers (5'-3')	Reverse primers (5'-3')	Melting tempearature (T°C)	Expected product size (bp) in PCR	Expected product size (bp) in Q-PCR	Putative Functions
OsISM06683	LOC_Os01g49110	28232075	ACAACCAGGATGTGTCGGAG	CGCACGGTAGTTCATCTTTG	60.2	257	84	prenyltransferase putative expressed
OsISM06684	LOC_Os01g49110	28232342	GCTGCTGGCTTATTCCCTAC	TGTCAGCGACATGTAACTCGT	59.2	279	88	prenyltransferase putative expressed
OsISM06685	LOC_Os01g49110	28232598	GAACGAGTTACATGTCGCTGA	TCTTCTGTGTCCTCTACATGGC	59.1	241	94	prenyltransferase putative expressed
OsISM06687	LOC_Os01g49160	28249184	AGCTACGTCCGCAGCCAC	GCAGCCTACAGCTCTTCCC	61.3	178	89	MYB family transcription factor putative expressed
OsISM06688	LOC_Os01g49160	28249433	TCCCTCTACAACTCCATCGG	ATTCTTGACGTCGTTGTCCG	60.6	180	79	MYB family transcription factor putative expressed
OsISM06689	LOC_Os01g49190	28277215	GTGCTCAACACCGGCTCT	AAGTGTGGCCCTACCAACAG	60.0	929	49	ATP synthase putative expressed
OsISM06690	LOC_Os01g49190	28278135	GCCACACTTGGGCGTATTAT	GCTTCACGATGGATAGGGAG	59.8	197	93	ATP synthase putative expressed
OsISM06691	LOC_Os01g49190	28278312	CTCCCTATCCATCGTGAAGC	TAGGGTGCAAGGAGATCCAC	59.9	241	96	ATP synthase putative expressed
OsISM06692	LOC_Os01g49190	28278590	GGGGTGGGCAAAACTGTACT	CCAACACCAGCAAAGACAGA	60.5	168	84	ATP synthase putative expressed
OsISM06693	LOC_Os01g49190	28278788	CAGGGAAATGATTGAGAGTGG	CATTCATTTGCCCGTAGACA	59.5	175	84	ATP synthase putative expressed
OsISM06694	LOC_Os01g49190	28279035	GGCAGGATGTGCTTCTCTTC	ACAGCAGATGGGATACGTCC	60.0	236	92	ATP synthase putative expressed
OsISM06695	LOC_Os01g49190	28279391	TCCTGCCCCTGCTACTACAT	CAAAGGATCGACAGCAGGAT	60.0	179	95	ATP synthase putative expressed
OsISM06696	LOC_Os01g49190	28279814	AGCTGAAGGAGAGCGTTCAA	GACCTCATCGATTCCTCCAA	60.1	774	99	ATP synthase putative expressed
OsISM06697	LOC_Os01g49200	28282250	CGATTCGCTTCTACTGGAGC	ATCCTGTCCCTCGTCGTGT	60.3	177	70	microtubule associated protein putative expressed
OsISM06699	LOC_Os01g49200	28283410	AGGACCTGGAATCCCTTCAG	TGCCAAGCACCTCACATAAG	60.2	226	94	microtubule associated protein putative expressed
OsISM06700	LOC_Os01g49200	28283747	TGCGTGAAATGAAGATCCAA	CGAGCATGCTAGAAGCAAAA	59.8	137	58	microtubule associated protein putative expressed
OsISM06701	LOC_Os01g49200	28283978	ACCAAACACCCTCTCCACAG	TGCTTTCAGTTGTTCAAGCC	59.5	170	71	microtubule associated protein putative expressed
OsISM06702	LOC_Os01g49200	28284221	GCGAGGAAGGTTATGCAGAG	TGTTGCAATGTGAGCTTCAA	59.5	194	99	microtubule associated protein putative expressed

ISM IDs	Rice gene locus IDs	Physical positions (bp) on chromosomes	Forward primers (5'-3')	Reverse primers (5'-3')	Melting tempearature (T°C)	Expected product size (bp) in PCR	Expected product size (bp) in Q-PCR	Putative Functions
OsISM06703	LOC_Os01g49200	28284476	CTTGTGAAGAGGAAGCCTGG	TGTTAGATGTGCTCCCCTCC	60.0	170	78	microtubule associated protein putative expressed
OsISM06704	LOC_Os01g49200	28284648	AAAGAGGGCTGAAAAGGCTC	TCGTTCATTTTTCCATGCAG	59.8	211	92	microtubule associated protein putative expressed
OsISM06705	LOC_Os01g49200	28284836	TTGCTGCATGGAAAAATGAA	ATCAAGCATTGACGAAAGGC	60.2	157	72	microtubule associated protein putative expressed
OsISM06706	LOC_Os01g49200	28285004	CGTTCGTCAGGAGAAAGAGC	TCCGTACAAAGCTTCCTGCT	60.1	183	92	microtubule associated protein putative expressed
OsISM06707	LOC_Os01g49200	28285313	TCAAAGTCTGTTCGTGCTGC	CGGCAATGTCCAAACCTCTA	60.6	169	77	microtubule associated protein putative expressed
OsISM06708	LOC_Os01g49219	28294361	GGAGTCCTGCCTGGGTCTAC	GAGGCGTTGTCCTTCACAT	59.9	156	57	expressed protein
OsISM06709	LOC_Os01g49219	28294581	CGGTGATTTCTTCCCTCCTT	TTTGGCTAATTGCCTCATCC	60.2	166	70	expressed protein
OsISM06711	LOC_Os01g49250	28311751	GTTATTGGGCAAGTTGGCAT	CAGCATTGAGCCTTCAACCT	60.1	911	99	methyltransferase putative expressed
OsISM06712	LOC_Os01g49250	28312706	AAATTTTCAGGAACATGGGC	AAACAGCCATGGTGTCCATT	59.6	693	58	methyltransferase putative expressed
OsISM06713	LOC_Os01g49250	28313515	ACTGGGAATGGGCTTCTTTT	GCAAGTTCAATGGCTCCTTC	59.9	563	90	methyltransferase putative expressed
OsISM06714	LOC_Os01g49250	28314091	GCACGTGATGGGTTTACTTCT	TCCAATGTCCCTTTGTCTGT	58.5	832	99	methyltransferase putative expressed
OsISM06715	LOC_Os01g49250	28314915	ACATTGGATGCCATTGGATT	ACCAGGTTCAACCAAGTTCG	60.0	560	85	methyltransferase putative expressed
OsISM06716	LOC_Os01g49250	28315457	AACTTGGTTGAACCTGGTGG	CATCCTTTGTGTGGTTGCAC	59.9	312	56	methyltransferase putative expressed
OsISM06717	LOC_Os01g49270	28322661	GAGCTTCTCAACGGCGTC	ATCTTCACAGCGTGCTTGGT	60.3	172	99	XPA-binding protein 2 putative expressed
OsISM06719	LOC_Os01g49290	28330719	CGAGATCCCTGTCTCCAAGA	GCATAGAGGGTGCTTCCATC	60.0	1240	76	WD repeat-containing protein putative expressed
OsISM06720	LOC_Os01g49300	28337923	ATATCGCCTGGGCAAAGTTA	TTGGTTCTTCTTCTGCCTGC	60.0	191	90	retrotransposon protein putative unclassified
OsISM06721	LOC_Os01g49300	28339427	GGCTACGAGCAAGGATCAAA	TGGTGAGGTACTGCCAGTTG	60.0	248	82	retrotransposon protein putative unclassified
OsISM06722	LOC_Os01g49300	28340672	GCAACACTGCTCAAGTTCCA	TGGGAGCCTCCATGAATCTA	60.3	177	97	retrotransposon protein putative unclassified

ISM IDs	Rice gene locus IDs	Physical positions (bp) on chromosomes	Forward primers (5'-3')	Reverse primers (5'-3')	Melting tempearature (T°C)	Expected product size (bp) in PCR	Expected product size (bp) in Q-PCR	Putative Functions
OsISM06723	LOC_Os01g49310	28348180	GAAGCTCACCATGCATCAAA	AATGTGGACTTGTGGAGTGCT	59.7	344	81	expressed protein
OsISM06724	LOC_Os01g49310	28348470	AGGATTTGCTTCAGCATCAAT	GCAGAGGATGGATAACCCAA	59.3	519	49	expressed protein
OsISM06725	LOC_Os01g49330	28363101	GCGGCACTAGCAAGAAGAAT	ACCAGCAAAGTTTCCAGGAC	59.4	180	74	formyl transferase putative expressed
OsISM06726	LOC_Os01g49330	28363410	CGGCGACCTCATCTTCAC	TGGTCTCACCTCCTTCAGGT	60.0	152	71	formyl transferase putative expressed
OsISM06727	LOC_Os01g49330	28363605	GGTTTCTTGATATCCCGCCT	TGCACCACGATATAGAGGCA	60.3	805	69	formyl transferase putative expressed
OsISM06728	LOC_Os01g49330	28364391	GCCTCTATATCGTGGTGCAG	GGCACGGACAGTATATGCAA	59.0	175	89	formyl transferase putative expressed
OsISM06729	LOC_Os01g49330	28364546	TTGCATATACTGTCCGTGCC	CAATATAGCAAGCAGCTCCG	59.1	548	99	formyl transferase putative expressed
OsISM06730	LOC_Os01g49330	28365073	CCGGAGCTGCTTGCTATATT	GCCTTTTCTTTAGCCGTTCC	59.8	302	90	formyl transferase putative expressed
OsISM06731	LOC_Os01g49330	28365355	GGAACGGCTAAAGAAAAGGC	TTGGTTGAATGACAACCATGA	60.0	752	94	formyl transferase putative expressed
OsISM06732	LOC_Os01g49330	28366103	CCAAGAAGCTAAATTGCTCCA	TTTTGGTTCACAAGCTGCAA	59.9	143	85	formyl transferase putative expressed
OsISM06733	LOC_Os01g49330	28366332	TCTTCTCGGGGAACTCATTG	GCTGTCGTCACCTTCTTTCC	60.0	176	92	formyl transferase putative expressed
OsISM06734	LOC_Os01g49420	28422077	GAAAATGGCATTCATGTCCC	TTTGGCTCCATATCCAGCAT	60.3	147	61	retrotransposon protein putative unclassified
OsISM06735	LOC_Os01g49420	28422344	CGTGCATTTGGGTCACTAAA	TGCCACTGTTGTTGTCCATT	59.8	884	99	retrotransposon protein putative unclassified
OsISM06736	LOC_Os01g49420	28423451	GCTGGTTGGGATGAGAAAAA	AATAATCCGCATCAGCCTTG	60.1	177	89	retrotransposon protein putative unclassified
OsISM06738	LOC_Os01g49510	28478696	CAGATGTACGGCCTCTTCGT	GTAGACCGCCCATATCCAGA	60.1	1466	84	OsPOP3 - Putative Prolyl Oligopeptidase homologue expressed
OsISM06739	LOC_Os01g49510	28480142	TCTGGATATGGGCGGTCTAC	TGCTGCTTCAATGTCTGCAT	60.2	173	64	OsPOP3 - Putative Prolyl Oligopeptidase homologue expressed
OsISM06740	LOC_Os01g49510	28480477	CCTGTCAAAAGGACGTATTGG	ATGAATTACGAGCACTGGGC	59.8	160	82	OsPOP3 - Putative Prolyl Oligopeptidase homologue expressed
OsISM06741	LOC_Os01g49510	28480607	CTGGTAAATTGCCCAGTGCT	CAAAGTTCCCACAGCTGCTT	60.3	410	84	OsPOP3 - Putative Prolyl Oligopeptidase homologue expressed

ISM IDs	Rice gene locus IDs	Physical positions (bp) on chromosomes	Forward primers (5'-3')	Reverse primers (5'-3')	Melting tempearature (T˚C)	Expected product size (bp) in PCR	Expected product size (bp) in Q-PCR	Putative Functions
OsISM06742	LOC_Os01g49550	28497651	AATGGTCGTCAACTCGTCGT	GTTTGGACCGGGGTTACTG	60.4	1076	93	hypothetical protein
OsISM06743	LOC_Os01g49550	28498708	CAGTAACCCCGGTCCAAAC	AAGAGCTTCAACCTCCTGGT	59.1	1308	89	hypothetical protein
OsISM06744	LOC_Os01g49580	28512132	TCTTCCACCCACTTACGACC	TTGGATAAAAGGGGTCTGCTT	60.0	168	68	Protein kinase domain containing protein expressed
OsISM06745	LOC_Os01g49580	28512876	AAGGATTGCAGAAACAACGG	GGAGACACAGCGAGAGAAGC	60.2	786	65	Protein kinase domain containing protein expressed
OsISM06746	LOC_Os01g49580	28514445	GAGTGCGCTCTCTCAGGTG	TTTGGGACAGAGAAAGGCAT	59.8	1011	95	Protein kinase domain containing protein expressed
OsISM06747	LOC_Os01g49600	28522171	CCACCATGGATTTCACCTGT	CGAATTCGATATCCACGGC	61.0	150	78	expressed protein
OsISM06748	LOC_Os01g49614	28523941	GTGCCAGCAACTCGTCTACA	CAAAGTTTCATTCTCCCCTTT	58.7	6826	56	Protein kinase domain containing protein expressed
OsISM06749	LOC_Os01g49614	28530732	GGGTATTCAAGCTTAAAGGGG	ACGCTTGAGATGCCTGCTAT	59.5	883	57	Protein kinase domain containing protein expressed
OsISM06750	LOC_Os01g49630	28534994	CCTACGACAAGGACTCCAGC	TCTTGGTATGTGAGGATGCG	59.8	235	94	proteophosphoglycan ppg4 putative expressed
OsISM06751	LOC_Os01g49660	28539024	CACAAGCTTCTTGGTGGAGG	GATCACAAGCATGGCAAGAA	60.3	281	82	reticulon domain containing protein putative
OsISM06752	LOC_Os01g49660	28539288	TTGCCATGCTTGTGATCTTC	ATTTCTGGGATCCTTGGAGG	60.0	194	74	reticulon domain containing protein putative
OsISM06753	LOC_Os01g49660	28539566	TGCATGTGGAAAGGATCTGA	TCGCCGACAATACCAATAGA	59.7	306	99	reticulon domain containing protein putative
OsISM06754	LOC_Os01g49660	28539873	TGGAAGTTCTTGCAGCTTGA	TCTTTGGTACAACACTGGCAA	59.5	211	77	reticulon domain containing protein putative
OsISM06755	LOC_Os01g49680	28544625	CTCCTCGCTCCAGGGCTT	GAGGACTTGTGCCGCTTC	61.2	183	75	DNA repair helicase XPB2 putative expressed
OsISM06756	LOC_Os01g49680	28544837	CCGAACTGGACTACACCGAT	GTCCCTCTTCTTCACCTCCC	60.0	198	69	DNA repair helicase XPB2 putative expressed
OsISM06757	LOC_Os01g49680	28545137	GCCTACGATTTCTTGATCGC	CGCAGCATATAGTGAGTGGG	59.6	1454	91	DNA repair helicase XPB2 putative expressed
OsISM06758	LOC_Os01g49680	28546721	TGAAGAAGAATCGATATTTTGTGG	TCCGTGCTCTGCAAATGATA	59.9	171	85	DNA repair helicase XPB2 putative expressed
OsISM06759	LOC_Os01g49680	28546872	TATCATTTGCAGAGCACGGA	GCCATTTCACCAGCTGTTTT	60.2	597	82	DNA repair helicase XPB2 putative expressed

ISM IDs	Rice gene locus IDs	Physical positions (bp) on chromosomes	Forward primers (5'-3')	Reverse primers (5'-3')	Melting tempearature (T°C)	Expected product size (bp) in PCR	Expected product size (bp) in Q-PCR	Putative Functions
OsISM06760	LOC_Os01g49680	28547508	TGCTGCAACTGAAGACAAGG	GAAGTTCAGTGCATTTGGCA	60.0	174	95	DNA repair helicase XPB2 putative expressed
OsISM06761	LOC_Os01g49680	28547683	CCATGCTGGAGGAGTACGAT	TTTCTTGATATGGCCTTGGG	60.0	1102	94	DNA repair helicase XPB2 putative expressed
OsISM06762	LOC_Os01g49680	28548787	AGCCTCAGCAAGATGTTTGG	CGACATGCAGCAGATACACC	60.3	566	99	DNA repair helicase XPB2 putative expressed
OsISM06763	LOC_Os01g49680	28549576	ATTCGAAATAGGGAGTGGGG	TGCTGATGACCTTTCTGAACA	59.8	176	80	DNA repair helicase XPB2 putative expressed
OsISM06764	LOC_Os01g49680	28549710	TCCATGTTGTTCCTGCACAT	GCGTTCATCCTCTCTCACAAG	60.0	188	99	DNA repair helicase XPB2 putative expressed
OsISM06765	LOC_Os01g49680	28549995	AAGTTTGGTGTCCCATGACC	AATTTGTTTGGGTTCATCGC	59.7	578	98	DNA repair helicase XPB2 putative expressed
OsISM06766	LOC_Os01g49680	28550667	GCAATGAAGCTCCGTAAACC	GCAGAATTCTCGTCCTCTCAA	59.6	137	65	DNA repair helicase XPB2 putative expressed
OsISM06767	LOC_Os01g49680	28550802	GCATCAATTCAAGAACAGCTCA	TGATCACATTTGCTTCTGGA	59.1	176	90	DNA repair helicase XPB2 putative expressed
OsISM06768	LOC_Os01g49680	28551019	CAACGGTTGGGACGTATTCT	GCCATCCTGTCTTGATGCTT	60.0	541	51	DNA repair helicase XPB2 putative expressed
OsISM06769	LOC_Os01g49680	28551555	ATGGCTGGTGGAAAAGAAGA	AAGAAATTGTTGCCTTTTAGTTGA	59.1	222	97	DNA repair helicase XPB2 putative expressed
OsISM06770	LOC_Os01g49680	28551763	GGCAACAATTTCTTATTGACCA	AGGTAATTCTTCAGGTGGCG	59.1	151	72	DNA repair helicase XPB2 putative expressed
OsISM06771	LOC_Os01g49680	28551894	CGCCACCTGAAGAATTACCT	ATGCCAATCATGTCATCCCT	59.7	195	98	DNA repair helicase XPB2 putative expressed
OsISM06772	LOC_Os01g49680	28552141	TCTGCTGGATCAATGAGTGC	CTTCGGTTTCTTGGAAGCTC	59.5	196	97	DNA repair helicase XPB2 putative expressed
OsISM06773	LOC_Os01g49690	28555169	CTAAGGTGAAGGAGGGCCAG	GCACGTTGGACTCCTCGAT	61.2	331	91	Ser/Thr protein phosphatase family protein putative expressed
OsISM06774	LOC_Os01g49690	28555553	GACCTGATGAAGCTCTTCGC	ACCTCCAGACTGTTGAAGCC	59.7	210	96	Ser/Thr protein phosphatase family protein putative expressed
OsISM06775	LOC_Os01g49690	28555743	GGCTTCAACAGTCTGGAGGT	TTTCATGATTTCCACGCAGA	59.8	767	89	Ser/Thr protein phosphatase family protein putative expressed
OsISM06776	LOC_Os01g49690	28556490	TCTGCGTGGAAATCATGAAA	TCCCATACTTCCTTTGGCAC	60.1	722	75	Ser/Thr protein phosphatase family protein putative expressed
OsISM06777	LOC_Os01g49690	28557226	CGGTATTGCACCGATGTTTT	CGCACATCAGGAGAAAGTCC	60.8	178	93	Ser/Thr protein phosphatase family protein putative expressed

ISM IDs	Rice gene locus IDs	Physical positions (bp) on chromosomes	Forward primers (5'-3')	Reverse primers (5'-3')	Melting tempearature (T°C)	Expected product size (bp) in PCR	Expected product size (bp) in Q-PCR	Putative Functions
OsISM06778	LOC_Os01g49690	28557384	GGACTTTCTCCTGATGTGCG	ACAATTGCGATCAATCGTCC	60.8	646	55	Ser/Thr protein phosphatase family protein putative expressed
OsISM06779	LOC_Os01g49690	28558126	TTTGGATCACGAGTGACAGC	GGTGAGCTCGGCAAACTAGA	60.2	185	65	Ser/Thr protein phosphatase family protein putative expressed
OsISM06780	LOC_Os01g49690	28558311	AGCTGGTTCAGGAAGGCTTA	GCAATAATTAGGAGCGGACC	58.9	192	75	Ser/Thr protein phosphatase family protein putative expressed
OsISM06781	LOC_Os01g49690	28558501	GCTACAGATGTGGAAATGTTGC	TCGCATCTGGTTATTCTCCTC	59.5	640	99	Ser/Thr protein phosphatase family protein putative expressed
OsISM06782	LOC_Os01g49740	28572288	AAGCATCAACGGGAGGAAG	AGACCGGATTGTGCTAGCTG	60.3	242	94	expressed protein
OsISM06783	LOC_Os01g49740	28572771	GACCAAGCCAAGAAGGAGC	CCGCTTCATCTCGTCGAT	59.9	141	70	expressed protein
OsISM06784	LOC_Os01g49760	28577423	GAGCTGCACTACGTGCCC	ATCAGCGGGTGATTCCTCTT	60.8	187	96	expressed protein
OsISM06785	LOC_Os01g49760	28577632	AATGCGATTGGATTTGACCT	CAAACCCTTGCATGGACATA	59.4	1120	65	expressed protein
OsISM06786	LOC_Os01g49760	28579380	TGACCATTATTTGGAGGAGGA	AGTTTGCCATCCTTTGTTGG	59.7	173	76	expressed protein
OsISM06787	LOC_Os01g49760	28579550	ACTGCTTGCCATTGGTCATT	ATTGCTACCAGCTCTGGCTC	60.1	278	94	expressed protein
OsISM06788	LOC_Os01g49760	28579836	TGGCCTCATCAGTTGACTACA	ACAGGGATAGCAGGAACACG	59.7	175	98	expressed protein
OsISM06789	LOC_Os01g49760	28580115	TGCTTTCGAAGCTTGTCTTG	CATCACGGAATGCAGTTGTC	59.7	477	79	expressed protein
OsISM06790	LOC_Os01g49760	28580650	AGTGCCAATACTTGCCCTTG	TGTCACCAAATGCTGAGGAA	60.2	617	95	expressed protein
OsISM06791	LOC_Os01g49760	28581308	TTATGACTTGGTTGGAGGGC	CACTTCATCATGCTGGGAGA	59.9	153	80	expressed protein
OsISM06792	LOC_Os01g49780	28588983	GGCGAGAAAGAGAGAGGAAGA	AGGGGCCCTAAAGGGTATTT	60.1	418	86	hypothetical protein
OsISM06793	LOC_Os01g49800	28596014	CAAAAATTTGGCAGCATTCA	AACAAACCGTGGCAAAATAG	58.7	415	85	hypothetical protein
OsISM06794	LOC_Os01g49800	28596444	AGACAAGGCGTGACCATAGG	CATGGCATCCCTTCTCTTGT	60.1	187	92	hypothetical protein
OsISM06795	LOC_Os01g49820	28604728	ACACAGTCCCATTTTGGGC	TGGAAATCATACACATTCCTCTTC	60.1	190	98	lipid phosphatase protein putative expressed

ISM IDs	Rice gene locus IDs	Physical positions (bp) on chromosomes	Forward primers (5'-3')	Reverse primers (5'-3')	Melting tempearature (T°C)	Expected product size (bp) in PCR	Expected product size (bp) in Q-PCR	Putative Functions
OsISM06796	LOC_Os01g49820	28604905	TGTATGATTTCCATCATGCCATA	GTCGACCAACCCCATCTTTA	59.9	181	91	lipid phosphatase protein putative expressed
OsISM06797	LOC_Os01g49820	28605096	TTTCTTTTGGCGCTGTTTTC	TTCTTTGATAACGCTCGCCT	60.2	200	92	lipid phosphatase protein putative expressed
OsISM06798	LOC_Os01g49820	28605277	GGCGAGCGTTATCAAAGAAG	ACCATGACAGGAAACCAAGG	59.9	278	84	lipid phosphatase protein putative expressed
OsISM06799	LOC_Os01g49820	28605665	GACTATTGGCATCATTGGCA	TCATCAGATGGCATTGGAAA	59.8	222	99	lipid phosphatase protein putative expressed
OsISM06800	LOC_Os01g49820	28605868	TTCCAATGCCATCTGATGAA	ATCAGGGTTGAGAATGTGCC	60.0	364	66	lipid phosphatase protein putative expressed
OsISM06801	LOC_Os01g49870	28644744	TGAGGTGGAGGAATTAGGCA	AAAACCTCTTCCTCTTCGGC	60.2	1395	88	transposon protein putative CACTA En/Spm sub-class expressed
OsISM06802	LOC_Os01g49870	28646848	GATGCAACGACAACTCATGC	CCCGCATCAACCACATAGTA	59.9	154	69	transposon protein putative CACTA En/Spm sub-class expressed
OsISM06803	LOC_Os01g49870	28647050	TCATGTGGAGCAATGGAAAA	GGAAAATAATCTGCATCCGC	59.8	267	99	transposon protein putative CACTA En/Spm sub-class expressed
OsISM06804	LOC_Os01g49880	28649763	AGAAGGGTGACTGACAAATGG	GGACCTCATATGATCCGGTTT	59.5	358	89	expressed protein
OsISM06805	LOC_Os01g49880	28650085	CCCTCACAATTTACAAAACCG	ACAAGGATCGTGGAGGTGAC	59.7	279	75	expressed protein
OsISM06806	LOC_Os01g49880	28650766	CAACCAACACAAGGAGCAGA	AGTTGATTCCTTCCCTTCCC	59.6	918	87	expressed protein
OsISM06807	LOC_Os01g49910	28666339	GGAACAATCGGATACATGGC	ATGACCCCGTAGCTGAACAC	60.1	179	81	expressed protein
OsISM06808	LOC_Os01g49920	28671503	CGAGTATGCCAGGCACTGTA	CGTGATGCTTTTCCTGTTGA	59.9	152	62	protein kinase putative expressed
OsISM06809	LOC_Os01g49950	28700611	TGACCCACCAACAATGAGAA	AACACCCAGTCGTCAACGTA	59.5	452	97	transposon protein putative unclassified
OsISM06810	LOC_Os01g49950	28701219	GCACCGTGACCGAGACTACT	ATATCGGCACTGATATCCCG	60.0	152	58	transposon protein putative unclassified
OsISM06811	LOC_Os01g49950	28701563	TCGACCTACGGCACCTATTT	AGTCCTACCGGCGTGAGAC	59.9	523	83	transposon protein putative unclassified
OsISM06812	LOC_Os01g49950	28702256	GTACTCCCACGGGCACATTA	CCGAAAGTGTGTTGTCGTGA	60.8	235	98	transposon protein putative unclassified
OsISM06813	LOC_Os01g49950	28702471	TCACGACAACACACTTTCGG	TCTGTCAACGCCCTGAAGTA	60.1	1801	87	transposon protein putative unclassified

ISM IDs	Rice gene locus IDs	Physical positions (bp) on chromosomes	Forward primers (5'-3')	Reverse primers (5'-3')	Melting tempearature (T°C)	Expected product size (bp) in PCR	Expected product size (bp) in Q-PCR	Putative Functions
OsISM06814	LOC_Os01g49950	28704212	GACTGCAGGTCCCCTCATAA	TTCTGCGTTTGGATGTTACG	59.9	573	99	transposon protein putative unclassified
OsISM06815	LOC_Os01g49950	28704845	TGGTGCTACAAGCCTCTTCA	GTCTGCTTCCATGCCTTGAC	60.2	3290	97	transposon protein putative unclassified
OsISM06816	LOC_Os01g49950	28708133	ACAGGACGTATTCGCAAACC	ACGACGAGCTCCATGGTC	59.9	714	84	transposon protein putative unclassified
OsISM06817	LOC_Os01g49970	28713565	GCACCTGTGCGAGATGTTTA	AGTAGCAGCCACCGACTACC	59.6	107	60	retrotransposon protein putative unclassified
OsISM06818	LOC_Os01g49970	28714104	AAGGAGTCCTCCCCCTCTG	GACTTGGAATGGCGGGAG	61.1	560	89	retrotransposon protein putative unclassified
OsISM06819	LOC_Os01g50010	28728282	AGCCTCGCCAATCTCAACT	CTTCACAATCGCCACATCC	60.0	234	52	clathrin assembly protein putative expressed
OsISM06820	LOC_Os01g50010	28728497	GGATGTGGCGATTGTGAAG	GAGGCCTACCGATGGACG	60.8	296	96	clathrin assembly protein putative expressed
OsISM06821	LOC_Os01g50010	28728807	TACTGCATCCATGCTCTTGC	CACCACAAGTGTCTTCAGTGC	59.7	944	73	clathrin assembly protein putative expressed
OsISM06822	LOC_Os01g50010	28729840	CTTCAAGGATGACTCCACCC	TTTCCTCCAAAAACAAACCG	59.7	152	75	clathrin assembly protein putative expressed
OsISM06823	LOC_Os01g50010	28730028	AAGCTGAGCGTTTGTCGAAG	CAGTGCTGGTAATTGCTCCA	60.3	230	99	clathrin assembly protein putative expressed
OsISM06824	LOC_Os01g50010	28730236	GCTGGAGCAATTACCAGCAC	TTGCTATTTGCAGCTCCTTC	59.5	192	76	clathrin assembly protein putative expressed
OsISM06825	LOC_Os01g50010	28730404	GCCAGAAGGAGCTGCAAATA	AATTCCATCGTTTATTGCACA	59.3	747	98	clathrin assembly protein putative expressed
OsISM06826	LOC_Os01g50010	28731129	TTGTGCAATAAACGATGGAA	TTCAAGGGCTTTGAGTGCTT	58.8	179	80	clathrin assembly protein putative expressed
OsISM06827	LOC_Os01g50010	28731289	AGCACTCAAAGCCCTTGAAA	GCAAGTTCTAATCCCCGACA	60.0	183	91	clathrin assembly protein putative expressed
OsISM06828	LOC_Os01g50010	28731452	TGTCGGGGATTAGAACTTGC	CCATGGTCGAAAGGAATGAT	59.9	174	77	clathrin assembly protein putative expressed
OsISM06829	LOC_Os01g50010	28731648	ACGCATGGTTCCAATTAAGG	AGCAGAAACAGGCTCAGGAA	60.0	370	98	clathrin assembly protein putative expressed
OsISM06830	LOC_Os01g50010	28732081	CCACCACCTCCTAAACCTGA	TACACTAGGGGTTGGATCGC	60.0	146	70	clathrin assembly protein putative expressed
OsISM06831	LOC_Os01g50010	28732207	GCGATCCAACCCCTAGTGTA	GTGCCACTTGTTGACGTCTC	59.6	176	92	clathrin assembly protein putative expressed

ISM IDs	Rice gene locus IDs	Physical positions (bp) on chromosomes	Forward primers (5'-3')	Reverse primers (5'-3')	Melting tempearature (T˚C)	Expected product size (bp) in PCR	Expected product size (bp) in Q-PCR	Putative Functions
OsISM06832	LOC_Os01g50010	28732436	TTACTACCCCGAGCACCAAC	TTCCCTCGTCGTATAGGCTG	60.1	880	97	clathrin assembly protein putative expressed
OsISM06833	LOC_Os01g50030	28739145	CCCCTCTAAAATCGCACTGA	CCTCTCCTCCACCTCAAGC	60.1	319	98	CPuORF25 - conserved peptide uORF-containing transcript expressed
OsISM06834	LOC_Os01g50030	28739543	ATCTCGACAAGGAGGAGCG	ATTCCAGCACCAAGTTCCAG	60.3	1288	83	CPuORF25 - conserved peptide uORF-containing transcript expressed
OsISM06835	LOC_Os01g50030	28740857	AAACAGCTGGGCATGTTCTT	AGGATGCATTCTTGTGGTGA	59.4	235	88	CPuORF25 - conserved peptide uORF-containing transcript expressed
OsISM06836	LOC_Os01g50030	28741163	TGGTTACTGATGTATCTTTCAGACG	TATAGCCGCCAACCTTTAGC	59.5	287	80	CPuORF25 - conserved peptide uORF-containing transcript expressed
OsISM06837	LOC_Os01g50030	28741505	TCCTACACATTACCGGGAGC	CAAAGGAATTCCCATCTTGA	58.8	304	81	CPuORF25 - conserved peptide uORF-containing transcript expressed
OsISM06838	LOC_Os01g50030	28741837	TGGAGCTTACGTGAAAAGCA	AAAAATCTTTGAAACCCCCG	59.9	180	94	CPuORF25 - conserved peptide uORF-containing transcript expressed
OsISM06839	LOC_Os01g50030	28742073	AAGGCTTTGTGAGCACTGGT	GTTTGAGATCCAGCCTGTCC	59.8	163	64	CPuORF25 - conserved peptide uORF-containing transcript expressed
OsISM06840	LOC_Os01g50030	28742434	CGTTTGACGTCATCTACAGTCG	ACCTTACCCCCAGGTTTGAG	60.5	377	98	CPuORF25 - conserved peptide uORF-containing transcript expressed
OsISM06841	LOC_Os01g50030	28742845	CCTTCAGAAGAGTTCGCAGC	CATGGAAACCAGCATTCTCA	60.0	191	95	CPuORF25 - conserved peptide uORF-containing transcript expressed
OsISM06842	LOC_Os01g50030	28743013	GCTTGAGAATGCTGGTTTCC	TTCAACTTTAGCAAGCTCCCTC	59.9	196	89	CPuORF25 - conserved peptide uORF-containing transcript expressed
OsISM06843	LOC_Os01g50030	28743187	GAGGGAGCTTGCTAAAGTTGAA	GAAGTTTTGCCTTCCATCCA	60.0	190	99	CPuORF25 - conserved peptide uORF-containing transcript expressed
OsISM06845	LOC_Os01g50032	28739543	ATCTCGACAAGGAGGAGCG	GCGCCACTTACTACCACGTA	59.7	211	98	expressed protein
OsISM06846	LOC_Os01g50032	28739702	TTTTGGTACTTTTCCCAACCA	ATTCCAGCACCAAGTTCCAG	59.7	1129	98	expressed protein
OsISM06847	LOC_Os01g50032	28740857	AAACAGCTGGGCATGTTCTT	AGGATGCATTCTTGTGGTGA	59.4	235	88	expressed protein
OsISM06848	LOC_Os01g50032	28741163	TGGTTACTGATGTATCTTTCAGACG	TATAGCCGCCAACCTTTAGC	59.5	287	80	expressed protein
OsISM06849	LOC_Os01g50032	28741505	TCCTACACATTACCGGGAGC	CAAAGGAATTCCCATCTTGA	58.8	304	81	expressed protein
OsISM06850	LOC_Os01g50032	28741837	TGGAGCTTACGTGAAAAGCA	AAAAATCTTTGAAACCCCCG	59.9	180	94	expressed protein

ISM IDs	Rice gene locus IDs	Physical positions (bp) on chromosomes	Forward primers (5'-3')	Reverse primers (5'-3')	Melting tempearature (T˚C)	Expected product size (bp) in PCR	Expected product size (bp) in Q-PCR	Putative Functions
OsISM06851	LOC_Os01g50032	28742073	AAGGCTTTGTGAGCACTGGT	GTTTGAGATCCAGCCTGTCC	59.8	163	94	expressed protein
OsISM06852	LOC_Os01g50032	28742434	CGTTTGACGTCATCTACAGTCG	ACCTTACCCCCAGGTTTGAG	60.5	377	98	expressed protein
OsISM06853	LOC_Os01g50032	28742845	CCTTCAGAAGAGTTCGCAGC	CATGGAAACCAGCATTCTCA	60.0	191	95	expressed protein
OsISM06854	LOC_Os01g50032	28743013	GCTTGAGAATGCTGGTTTCC	TTCAACTTTAGCAAGCTCCCTC	59.9	196	89	expressed protein
OsISM06855	LOC_Os01g50032	28743187	GAGGGAGCTTGCTAAAGTTGAA	GAAGTTTTGCCTTCCATCCA	60.0	190	99	expressed protein
OsISM06856	LOC_Os01g50040	28745860	CTAGGGTCGCCTTCCTCTTC	TACACCGAGAACTTGCCCTC	60.3	203	98	DNA binding protein putative expressed
OsISM06857	LOC_Os01g50040	28746102	CACGGGATCGTCGTACTTCT	AACATCCTCTCTGCCTCCAC	59.7	200	91	DNA binding protein putative expressed
OsISM06858	LOC_Os01g50040	28746334	AGCGCTTTGTTTTGCTCTCT	AAAACTTTTAGTCGAAGCCATCA	59.4	679	87	DNA binding protein putative expressed
OsISM06859	LOC_Os01g50040	28746944	TGCAGCTGTGTTCCTCTGTA	CGCTTCTCGGTCTTGTCAAC	59.6	211	99	DNA binding protein putative expressed
OsISM06860	LOC_Os01g50040	28747158	CAATCCAAGCATGTCTCCTG	GCATGTCTTCTGGTCAATGC	59.2	173	79	DNA binding protein putative expressed
OsISM06861	LOC_Os01g50040	28747329	GCAGAAGTAGTCGTTGGCG	AGGCTTCTGTCCAAGCAAAG	59.6	953	91	DNA binding protein putative expressed
OsISM06862	LOC_Os01g50040	28748258	AAGGCTTTGCTTGGACAGAA	ACCGATATCTGCTTTCGACG	60.1	490	69	DNA binding protein putative expressed
OsISM06863	LOC_Os01g50040	28748721	TGGACTTCGTCGAAAGCAG	CATCTGGAATGCAATCATGC	60.1	295	63	DNA binding protein putative expressed
OsISM06864	LOC_Os01g50040	28748996	GCATGATTGCATTCCAGATG	TCCAGTTCATTTTCATGTCCA	59.5	172	70	DNA binding protein putative expressed
OsISM06865	LOC_Os01g50040	28749259	GCAAGTCCTGAGCAAATCAA	CCACTCAGTTCTGTGCTCCA	59.5	495	73	DNA binding protein putative expressed
OsISM06867	LOC_Os01g50080	28773364	CGTACTGATCCAAGATGCCC	TGAATCCGATGACAAAACCA	60.2	691	87	MDR-like ABC transporter putative expressed
OsISM06868	LOC_Os01g50080	28774187	TTGTTGAGCAGACCATTGGA	TTGCTCTCTTCTCACCATTGAA	60.1	172	61	MDR-like ABC transporter putative expressed
OsISM06869	LOC_Os01g50080	28774520	GGAGGGCAAGTCATCAATGT	CAGATTGGCCTTCTGCAATA	59.4	730	98	MDR-like ABC transporter putative expressed

ISM IDs	Rice gene locus IDs	Physical positions (bp) on chromosomes	Forward primers (5'-3')	Reverse primers (5'-3')	Melting tempearature (T˚C)	Expected product size (bp) in PCR	Expected product size (bp) in Q-PCR	Putative Functions
OsISM06870	LOC_Os01g50080	28775685	TTGCAAATGCAGCAAACTTC	AATCCTTTGCTTTTGTCCCC	60.1	191	99	MDR-like ABC transporter putative expressed
OsISM06871	LOC_Os01g50080	28776035	AAGAATGCCGACTGCATAGC	TCGGGATCCTTGATCAACTC	60.2	189	81	MDR-like ABC transporter putative expressed
OsISM06872	LOC_Os01g50080	28776780	CAAAGCATCGTCCATCAAGA	TCGGATGTTCAAAGCATCAA	60.0	184	97	MDR-like ABC transporter putative expressed
OsISM06873	LOC_Os01g50080	28777122	GAAAGGGTTCAGTGAAGATGC	CTGCACAGAAAGACGCTACAG	58.8	334	99	MDR-like ABC transporter putative expressed
OsISM06874	LOC_Os01g50080	28777598	TGCACAGTTTGTACATGGGG	TGAGAAATTCCAAAAGCTGTGA	60.1	172	88	MDR-like ABC transporter putative expressed
OsISM06875	LOC_Os01g50080	28777967	TTACCTTAGGCATCCCCTCC	ACTGTGGACTTTCCACTGCC	60.2	475	68	MDR-like ABC transporter putative expressed
OsISM06876	LOC_Os01g50110	28795767	GGAGGACCTCATCCTCCG	TCTTGATCTCGTTGTCCGTG	60.2	188	93	MYB family transcription factor putative expressed
OsISM06877	LOC_Os01g50120	28798023	AGCAGCTCCTACAGCTCCAC	TTCTCAGCAGCCTCAGTCAA	59.8	378	95	expressed protein
OsISM06878	LOC_Os01g50120	28798510	CGCATTGAGGTGCTTGATAA	AGACATCCCTGGATGCAATG	60.4	463	97	expressed protein
OsISM06879	LOC_Os01g50120	28799323	GTGGCAAAAATTGTCTGACG	CCTCAGATCCAGCCTCCTTT	59.9	218	96	expressed protein
OsISM06880	LOC_Os01g50160	28824962	AGGTCATCAACGCCTTCG	CGATGCCCAGGTAGACAAAG	60.2	1308	88	MDR-like ABC transporter putative expressed
OsISM06881	LOC_Os01g50160	28826250	CTTTGTCTACCTGGGCATCG	CGTTCTCCAGTCATTGTCCA	60.2	658	73	MDR-like ABC transporter putative expressed
OsISM06882	LOC_Os01g50160	28827026	TTAGTTCAGGATGCCATTGGT	GCCAACCTTTCACAAATGCT	59.8	223	95	MDR-like ABC transporter putative expressed
OsISM06883	LOC_Os01g50160	28827357	ATGCAGCGAATGTTGTTGAA	GCTTTTCACCGTTGAAGGAA	60.2	162	67	MDR-like ABC transporter putative expressed
OsISM06884	LOC_Os01g50160	28827709	GTTTGCTGTCATGACTGGGG	TAAGCTGCAGATTGCCCTTC	61.0	156	82	MDR-like ABC transporter putative expressed
OsISM06885	LOC_Os01g50160	28828289	GAGCTTGCTAATGCAGCAAAT	CTTTTGTCCCCCTGAGAGC	59.7	189	94	MDR-like ABC transporter putative expressed
OsISM06886	LOC_Os01g50160	28828634	TTGACCACAGTGAGGAATGC	ACCATTGGGGTTCATTACCA	59.8	197	97	MDR-like ABC transporter putative expressed
OsISM06887	LOC_Os01g50160	28829371	GCCTTGTCATTCCGGAGTAT	TGATAATCTTGCACCAAGCG	59.4	185	88	MDR-like ABC transporter putative expressed

ISM IDs	Rice gene locus IDs	Physical positions (bp) on chromosomes	Forward primers (5'-3')	Reverse primers (5'-3')	Melting tempearature (T˚C)	Expected product size (bp) in PCR	Expected product size (bp) in Q-PCR	Putative Functions
OsISM06888	LOC_Os01g50160	28829694	CCACTTGTGGGTCTTCAAGG	AGCCACTTGACTTGCATCTTC	60.0	333	94	MDR-like ABC transporter putative expressed
OsISM06889	LOC_Os01g50160	28830217	CACAATTCGTACGCCACAAT	TCCAATAGTTGCCAACACCA	59.7	214	78	MDR-like ABC transporter putative expressed
OsISM06890	LOC_Os01g50160	28830593	TTCAAGTATCCAACCCGCC	CCGCTACCACTCTCTCCAAC	60.4	182	99	MDR-like ABC transporter putative expressed
OsISM06891	LOC_Os01g50180	28838811	CTGTGTCCGGTGTTCTGTGT	TGAAAACTAGCATGAACGCA	58.8	1041	81	retrotransposon protein putative Ty1-copia subclass
OsISM06892	LOC_Os01g50180	28843611	TGCCGGCTATCCTTATGAAC	CCGGAGTTTCTTAATTTCCTGA	59.8	130	81	retrotransposon protein putative Ty1-copia subclass
OsISM06893	LOC_Os01g50180	28843830	CGAAGGAGATGGGTTTGAGA	GACTGCGACCAACAGTCAAG	59.8	288	73	retrotransposon protein putative Ty1-copia subclass
OsISM06894	LOC_Os01g50320	28884795	GCTCTCAAGATGCGGTCTCT	CCCAACCTGTATCTGTCCCA	60.2	1116	46	surp module family protein putative expressed
OsISM06895	LOC_Os01g50320	28886002	CGTGCCTGACAGATTGTTGA	CCCCATGCTCACTGACAAAT	60.9	513	90	surp module family protein putative expressed
OsISM06896	LOC_Os01g50320	28887160	GGTTTCCATTTCTTCTGCCA	GAAGAACCACCACGGGACT	60.0	1129	92	surp module family protein putative expressed
OsISM06897	LOC_Os01g50340	28894647	CCTTCAACAGTGCAGGGATT	TTTGTCATCTGCATGACCGT	60.1	177	88	transposon protein putative unclassified expressed
OsISM06898	LOC_Os01g50360	28912781	TCGAACAAATTGAGGTTTCTGA	CTCCTCCTCTCCCGCATC	60.3	767	85	NAC domain containing protein putative expressed
OsISM06899	LOC_Os01g50360	28913919	GATGAAGACGACGATGACGA	TGCTTGGAGCCCTTGTTTAC	60.0	288	75	NAC domain containing protein putative expressed
OsISM06900	LOC_Os01g50360	28914267	GGGAGTCAGCAGGATTACCA	GAGGTTTGGACACCACGAAC	60.2	148	60	NAC domain containing protein putative expressed
OsISM06901	LOC_Os01g50430	28951145	TGGTGATACTCACTCAAAACCG	CTCCTTGAGCTCGGGTGTAG	60.0	1291	89	hypothetical protein
OsISM06902	LOC_Os01g50440	28954858	ATCAACCACGCCATCCAC	GGGAAGTAGATCGGGGAGAA	60.4	263	78	AWPM-19-like membrane family protein putative expressed
OsISM06903	LOC_Os01g50440	28955259	GTTCATCGCATGGACGCT	TCTTACCAATCTGGCGTTCC	60.4	178	86	AWPM-19-like membrane family protein putative expressed
OsISM06904	LOC_Os01g50440	28955374	TACGTTTTCAGATTGGCCGT	TGCCGAGAGTATGATGGTGA	60.4	198	91	AWPM-19-like membrane family protein putative expressed
OsISM06905	LOC_Os01g50450	28969875	CTACCGTCGCGACCAAAC	ATCTCCTCGGAGCTCTCCAT	60.3	359	66	expressed protein

ISM IDs	Rice gene locus IDs	Physical positions (bp) on chromosomes	Forward primers (5'-3')	Reverse primers (5'-3')	Melting tempearature (T˚C)	Expected product size (bp) in PCR	Expected product size (bp) in Q-PCR	Putative Functions
OsISM06906	LOC_Os01g50450	28970649	TCTCTGGGTGCAGCTGATACT	AATACCCCACAGCAATTCCA	60.1	198	99	expressed protein
OsISM06907	LOC_Os01g50460	28974561	TCCAACAACCATGGACTCCT	TTACACTGGCGACAGGAAGA	59.9	226	95	nodulin MtN3 family protein putative expressed
OsISM06908	LOC_Os01g50460	28974748	AACATCTTTGCGCTTGCTCT	TGGCCTTCAGGATCCTCTTA	60.0	144	62	nodulin MtN3 family protein putative expressed
OsISM06909	LOC_Os01g50460	28975046	CCTCTTCATCTTCTACGCGG	GCATGTGAAACTAGCGCAAA	60.0	268	94	nodulin MtN3 family protein putative expressed
OsISM06910	LOC_Os01g50460	28975390	TTCGCTTCTCCGTTAGCTGT	GCGCTCATCAAGAACGTAGA	59.7	213	96	nodulin MtN3 family protein putative expressed
OsISM06911	LOC_Os01g50460	28975600	CGCCAGTTTTGCATTGTATG	TTGCTCCCAGGATAAGTCCA	60.4	207	81	nodulin MtN3 family protein putative expressed
OsISM06912	LOC_Os01g50470	28977708	TGTTGCTGTTTAGATGACACCA	ACGGATCTTGGGTTTTCCTC	59.8	213	98	regulator of chromosome condensation putative expressed
OsISM06913	LOC_Os01g50470	28977913	AGATCCGTGCGTTCAGACTT	TTTTCCTTTTTGTGAGAAAACCA	59.9	155	65	regulator of chromosome condensation putative expressed
OsISM06914	LOC_Os01g50470	28978045	TGGTTTTCTCACAAAAAGGAAAA	CAGGGTGTAAAAACCTCCCA	59.9	197	98	regulator of chromosome condensation putative expressed
OsISM06915	LOC_Os01g50470	28978269	TATTTAAAAACGGGCAACGC	AACCCCTCAAGTGCTGAAAA	59.8	243	83	regulator of chromosome condensation putative expressed
OsISM06916	LOC_Os01g50470	28978507	GGGTTAATAAGTTCATTCCGCA	AGGAATGGCGATCTTGATAA	58.7	807	98	regulator of chromosome condensation putative expressed
OsISM06917	LOC_Os01g50470	28981239	TTGCAGAAGTCTAACGAATTGC	GGCCTCGCACTTTTGTTTTA	59.9	159	82	regulator of chromosome condensation putative expressed
OsISM06918	LOC_Os01g50470	28981475	CAGTGCTGTGGTCGAGTTTG	GGCTTTCAAGCTTTCTGCTG	60.4	275	92	regulator of chromosome condensation putative expressed
OsISM06919	LOC_Os01g50470	28982102	GCAAAGTATTCAAGCGGGTC	TTCAGCCTGATTCTCTGCAA	59.7	150	57	regulator of chromosome condensation putative expressed
OsISM06920	LOC_Os01g50490	28991049	CATGGACAACGTAAAGGGCT	ACACTGTTCGACGTGGTGTC	59.8	204	67	cytochrome P450 putative expressed
OsISM06921	LOC_Os01g50520	29019989	TCTCAGTGCTCACGAGGAAG	TCCTTGCACAACTCAGCATC	59.6	204	92	cytochrome P450 monooxygenase CYP711A12 putative
OsISM06922	LOC_Os01g50530	29024174	AACAGCTCGAAATTGGAGGA	CTGGCTCTGGGAAATTTTTG	59.7	339	97	cytochrome P450 putative expressed
OsISM06923	LOC_Os01g50570	29035521	GTGCTCGCAAGGAAGTACG	CACCATGACCAATGGTTGTC	59.6	178	64	cytochrome P450 monooxygenase CYP711A12 putative

ISM IDs	Rice gene locus IDs	Physical positions (bp) on chromosomes	Forward primers (5'-3')	Reverse primers (5'-3')	Melting tempearature (T°C)	Expected product size (bp) in PCR	Expected product size (bp) in Q-PCR	Putative Functions
OsISM06925	LOC_Os01g50580	29039699	CCTTCTTCGACATTGGTCGT	GGAAGGGCATATCCTCCAAT	60.1	198	71	cytochrome P450 putative expressed
OsISM06926	LOC_Os01g50580	29039859	ACGTGCGAACAGGTTGAAAT	AGTTCTTGGGATCCTTCGCT	60.4	177	92	cytochrome P450 putative expressed
OsISM06927	LOC_Os01g50590	29044185	ATGTGTTCTCCGTTCTCGCT	CACAATGATCAGTGGTTGCC	59.9	362	75	cytochrome P450 putative
OsISM06928	LOC_Os01g50590	29044592	AGCATTCCAAATCGAAGCAT	TTTCTCATCGACGTCCACCT	60.2	622	99	cytochrome P450 putative
OsISM06929	LOC_Os01g50590	29045962	GATCTGCAGACCAAGTTCCC	GTTCAAGTGTTTCCCTGGCT	59.4	193	95	cytochrome P450 putative
OsISM06930	LOC_Os01g50590	29046132	GCTAGCCAGGGAAACACTTG	ATCCTTCGCTAGCACTCCTG	59.7	590	95	cytochrome P450 putative
OsISM06931	LOC_Os01g50600	29048502	CCATCTCCTCCTCCAACCTC	AGCAGTATTGAGGGCTGGTT	59.9	281	58	hypothetical protein
OsISM06932	LOC_Os01g50640	29086242	GATGATCACGTGCGACGA	CTGGTCTCCAGGCGAAGA	60.2	155	90	retrotransposon protein putative unclassified
OsISM06933	LOC_Os01g50640	29086658	CTAAGGACCCTTACCCCCTG	TCCGCTAGATCCGAGTAAGG	59.6	323	77	retrotransposon protein putative unclassified
OsISM06934	LOC_Os01g50640	29088224	GAGTACGAGGGACTCCTTGC	CAGAGCACTGGTACTCCTTGG	59.4	121	86	retrotransposon protein putative unclassified
OsISM06935	LOC_Os01g50680	29103886	TGGTTTGCTTGCTACAGTCG	GGCAGCTCTGAGTCCAAAGT	59.8	331	85	OsSub2 - Putative Subtilisin homologue expressed
OsISM06936	LOC_Os01g50680	29104332	AAGTACGGGGTGGACGCTAT	ATAGCCGTCGTCTTTGAAGC	60.1	898	79	OsSub2 - Putative Subtilisin homologue expressed
OsISM06937	LOC_Os01g50680	29105270	ACGACACGACCTTCAAATGC	TAGGAAGCCGAGGTTGAAGA	60.5	154	63	OsSub2 - Putative Subtilisin homologue expressed
OsISM06938	LOC_Os01g50680	29105880	GACTTCCCGGCTTATGTCAC	TTGAGGCAGAGTGCTGTTTG	59.9	183	85	OsSub2 - Putative Subtilisin homologue expressed
OsISM06939	LOC_Os01g50680	29106105	GCAAACGTACCGAGCGAG	TGCAGACAACTATCTTGCCG	60.3	174	83	OsSub2 - Putative Subtilisin homologue expressed
OsISM06940	LOC_Os01g50680	29106340	CATGGTGCTCTGCAACTACG	ACTCGAAAAGAATCGACCCC	60.5	1377	93	OsSub2 - Putative Subtilisin homologue expressed
OsISM06941	LOC_Os01g50690	29109506	CTGTTGCCTCTGGTCACCTT	CTATGGGAAATGGGTCGCTA	60.1	187	97	WD domain G-beta repeat domain containing protein expressed
OsISM06942	LOC_Os01g50690	29109673	TAGCGACCCATTTCCCATAG	GCGCATAATTCAGTTTCACG	59.6	655	94	WD domain G-beta repeat domain containing protein expressed

ISM IDs	Rice gene locus IDs	Physical positions (bp) on chromosomes	Forward primers (5'-3')	Reverse primers (5'-3')	Melting tempearature (T˚C)	Expected product size (bp) in PCR	Expected product size (bp) in Q-PCR	Putative Functions
OsISM06943	LOC_Os01g50690	29110338	TCATGGTCTCAACAAGGGTTT	ATTCACAAAATGGTGACCGA	59.1	223	98	WD domain G-beta repeat domain containing protein expressed
OsISM06944	LOC_Os01g50690	29110552	TTTGTGAATTCTGTGATGAATGG	TAGGACCATCATTTTCCCCA	60.0	630	97	WD domain G-beta repeat domain containing protein expressed
OsISM06945	LOC_Os01g50690	29111162	TGGGGAAAATGATGGTCCTA	AGAGGCTCTTGCAGTTTACCA	59.6	225	94	WD domain G-beta repeat domain containing protein expressed
OsISM06946	LOC_Os01g50690	29111391	AGCATGGGTACAGAACGGAG	ATCCATTGGAGCATCTACGC	60.1	884	91	WD domain G-beta repeat domain containing protein expressed
OsISM06947	LOC_Os01g50690	29112424	GGATGCAGACTTTTCATTGGA	AGCATCGTCAAGTCCAGGTC	60.2	279	83	WD domain G-beta repeat domain containing protein expressed
OsISM06948	LOC_Os01g50690	29113077	TTTGTTGCTTTTGGCAACTG	GCACTTCTTTTGCGCATGTA	60.0	320	96	WD domain G-beta repeat domain containing protein expressed
OsISM06949	LOC_Os01g50690	29113377	TACATGCGCAAAAGAAGTGC	ATGCTGAGCTGGTGCAGATA	59.8	2294	98	WD domain G-beta repeat domain containing protein expressed
OsISM06950	LOC_Os01g50690	29115757	GATTCATGCATGTGACGCTC	AAACAATGCCCATGAAAACC	60.0	139	67	WD domain G-beta repeat domain containing protein expressed
OsISM06951	LOC_Os01g50690	29115862	CAGGTCTGTCATGGGGTTTT	CGTTGAACATCCAACAATGTG	59.8	507	79	WD domain G-beta repeat domain containing protein expressed
OsISM06952	LOC_Os01g50690	29116388	TCCTCTGCACACAGATGGTC	TTCTCCAGGTGCTAGTGCAA	59.7	173	92	WD domain G-beta repeat domain containing protein expressed
OsISM06953	LOC_Os01g50690	29116541	TTGCACTAGCACCTGGAGAA	AAGTTTGAGTCCAAGCTGCG	60.1	172	56	WD domain G-beta repeat domain containing protein expressed
OsISM06954	LOC_Os01g50690	29116693	CGCAGCTTGGACTCAAACTT	CCAAGGAACTGACCACCAAT	60.2	221	93	WD domain G-beta repeat domain containing protein expressed
OsISM06955	LOC_Os01g50690	29117083	TCTGCACCTACCTTTCTTGAGAC	ATTGTTCCACAAATCAGCCA	59.5	240	82	WD domain G-beta repeat domain containing protein expressed
OsISM06956	LOC_Os01g50690	29117478	GAACGATGGAGAAGTGAGGG	CAACTGAGTACTCGCACACTGA	59.4	241	93	WD domain G-beta repeat domain containing protein expressed
OsISM06957	LOC_Os01g50700	29118749	TACTACGACGTCTTGGGGGT	TTGGGATTCTTGTCAGGGTG	60.2	231	99	dehydrin family protein expressed
OsISM06958	LOC_Os01g50700	29118932	GTTTGGGTTTCTGTAGGCCA	ATTTGATAGGCCTCACCCAG	59.5	893	97	dehydrin family protein expressed
OsISM06959	LOC_Os01g50700	29119857	ACAAGCATGGAAAAGAAGGC	CCCAAAAAGCATTCCAAAGA	59.7	281	72	dehydrin family protein expressed
OsISM06960	LOC_Os01g50700	29120216	AATACAGAGGCAAGGGCAAG	TTTGCTCTCTTTCGGTTTGC	59.9	202	62	dehydrin family protein expressed

ISM IDs	Rice gene locus IDs	Physical positions (bp) on chromosomes	Forward primers (5'-3')	Reverse primers (5'-3')	Melting tempearature (T˚C)	Expected product size (bp) in PCR	Expected product size (bp) in Q-PCR	Putative Functions
OsISM06961	LOC_Os01g50700	29120470	GGATGAATTTGGTGATTGGG	GTGTGGAGCATAGCCTCACC	60.3	269	76	dehydrin family protein expressed
OsISM06962	LOC_Os01g50700	29120848	AAGTCACAGGTTAATGCGGC	TCATAAGCTGGCCTTCCTTG	60.2	220	98	dehydrin family protein expressed
OsISM06963	LOC_Os01g50700	29121132	TGAATCAACGCTTTCTCGTG	AGCTCGCACCTTCAGAACAT	60.0	204	77	dehydrin family protein expressed
OsISM06964	LOC_Os01g50700	29121325	AGGTGCGAGCTAGAGGACTG	AAGCATCAAAGGCTCGAAAA	59.9	905	82	dehydrin family protein expressed
OsISM06965	LOC_Os01g50700	29122189	GCCACGCAATATTCTTCGAT	TCCGGGTGAACAGTAAAAGG	60.0	131	69	dehydrin family protein expressed
OsISM06966	LOC_Os01g50700	29123020	CAAATCCAGCTCCAGCTCC	ATGCTCTTCTTCTTCCTCCG	59.8	149	70	dehydrin family protein expressed
OsISM06967	LOC_Os01g50710	29130136	GCAGGAGTTTGGAACTTTGG	CTGCTAGCGACAGAAGCAAG	59.4	464	96	hypothetical protein
OsISM06968	LOC_Os01g50750	29152089	GACGATCCCGAGATGCAC	ACCACATCGCGAAGAACAG	60.0	175	98	zinc finger C3HC4 type domain containing protein expressed
OsISM06969	LOC_Os01g50750	29152503	CAGTGGCGGTTCAAGGAG	CCTTGTACTGAGCCAGGCAT	60.3	680	86	zinc finger C3HC4 type domain containing protein expressed
OsISM06970	LOC_Os01g50760	29157449	CTTCGAGTTCACCGACGAGT	ATCAATGACAGAGATCCCGC	60.2	1704	95	polyprenyl synthetase putative expressed
OsISM06971	LOC_Os01g50760	29159196	AGACATTTCTTGCCTGCACC	CCACGTCGTGTTTGAGAGTT	59.5	199	98	polyprenyl synthetase putative expressed
OsISM06972	LOC_Os01g50760	29159376	ACTCTCAAACACGACGTGGC	TCGTGAAATATGGTTGCGAA	60.7	344	99	polyprenyl synthetase putative expressed
OsISM06973	LOC_Os01g50760	29159727	AACGCCATTTTAAAGGAAAGC	CCAACAATTGACCTGAAGCA	59.7	182	88	polyprenyl synthetase putative expressed
OsISM06974	LOC_Os01g50760	29159889	TGCTTCAGGTCAATTGTTGG	CTTGTACTGAACAATGCGGC	59.5	344	98	polyprenyl synthetase putative expressed
OsISM06975	LOC_Os01g50760	29160197	ATATTGTGCAGTCACCGCC	CACCAGATAGCAGCAAAGCA	59.8	196	89	polyprenyl synthetase putative expressed
OsISM06976	LOC_Os01g50760	29160427	TCTTGTGGAAATGGGGACAT	GGCACACTAACCTTGCCAAT	60.1	162	88	polyprenyl synthetase putative expressed
OsISM06977	LOC_Os01g50760	29160553	TGGTGATCCTGAGTTTATTGG	CGAGAGCTTGCACAACTAACC	58.8	202	78	polyprenyl synthetase putative expressed
OsISM06978	LOC_Os01g50760	29160762	TGACGAGAACCAAAAGCACA	CAGGATCTGGCTTCCCATAA	60.2	125	51	polyprenyl synthetase putative expressed

ISM IDs	Rice gene locus IDs	Physical positions (bp) on chromosomes	Forward primers (5'-3')	Reverse primers (5'-3')	Melting tempearature (T˚C)	Expected product size (bp) in PCR	Expected product size (bp) in Q-PCR	Putative Functions
OsISM06979	LOC_Os01g50760	29160891	TGTTGCAAAAGTGAAGGATCTG	CTTCGATGTCGGCAATCAG	60.3	355	99	polyprenyl synthetase putative expressed
OsISM06980	LOC_Os01g50770	29165717	CTTCTTCACCAAGCTCCTCG	TGTGCTGGATGAACATGTAGG	59.9	199	96	adaptor complexes medium subunit family domain containing protein expressed
OsISM06981	LOC_Os01g50770	29165965	CATCCTCCTCTTCCTCCACC	ATCCCTCAGCGACTCTTCCT	60.7	166	77	adaptor complexes medium subunit family domain containing protein expressed
OsISM06982	LOC_Os01g50770	29166111	AGGAAGAGTCGCTGAGGGAT	ATTTTCGCCTCCGTGTATTG	60.2	186	92	adaptor complexes medium subunit family domain containing protein expressed
OsISM06983	LOC_Os01g50770	29166382	CGGAGTGAGGGTATTCGGTA	CTGATCTCACAATCTGCCCA	59.9	179	98	adaptor complexes medium subunit family domain containing protein expressed
OsISM06984	LOC_Os01g50770	29166563	GTTGTTGGGGCACTGAAGAT	ATCCAAGTTTGCACTCAGGC	60.1	1303	59	adaptor complexes medium subunit family domain containing protein expressed
OsISM06985	LOC_Os01g50770	29167889	GCTCAAGGCCGAGCTACTAA	TCAAATCTAGCCAACCTCACG	60.0	169	81	adaptor complexes medium subunit family domain containing protein expressed
OsISM06986	LOC_Os01g50770	29168074	TCCTTCATACCTCCTGACGG	AATTTGTGCTTCCACCCAGA	60.3	197	88	adaptor complexes medium subunit family domain containing protein expressed
OsISM06987	LOC_Os01g50770	29168271	GAGAAACATTCAAGAAGCCGA	GGAACAGGCACTTCAATTTCA	59.8	181	99	adaptor complexes medium subunit family domain containing protein expressed
OsISM06988	LOC_Os01g50770	29168518	TGCAATGGTCTGGAAAGTAAAA	CAGGAGCTGCTTCTTCTGC	59.3	182	99	adaptor complexes medium subunit family domain containing protein expressed
OsISM06989	LOC_Os01g50770	29168704	GAAGGCACCAATACGAGTGAA	CGCCTGGTACCCACTCTTT	60.1	197	98	adaptor complexes medium subunit family domain containing protein expressed
OsISM06990	LOC_Os01g50780	29170486	CTGGACGCTCACACTGACAT	AGCCTCCACAAGAACTCCAC	59.6	263	89	hypothetical protein
OsISM06993	LOC_Os01g50830	29191462	GGTTTTGAAAGATCACTATCACTCC	ACCTTTTAGGGCGTCCAAGT	59.7	191	99	expressed protein
OsISM06994	LOC_Os01g50830	29191927	GGGGTAATGGAAAATCAGGG	AGAATGCTGCACATAGGGTTC	59.8	431	68	expressed protein
OsISM06995	LOC_Os01g50830	29192645	CAACGATGGTTTTGAGCTTG	AGCGATTCACTCAGGCATCT	59.6	619	68	expressed protein
OsISM06996	LOC_Os01g50860	29218735	ATTCACTGTTCCAAATCCCG	AGAGGCACCTTGCATACACC	60.0	217	98	chloride transporter chloride channel family putative expressed
OsISM06997	LOC_Os01g50860	29219417	AACTGCGAAAACTACCAGGG	AGGTGTCACAGGCCACATTT	59.8	616	89	chloride transporter chloride channel family putative expressed
OsISM06998	LOC_Os01g50860	29220125	AGGACTCTTGGTTGGTTTTGT	CCCCCTTTATCTCAGATGTAAAAC	58.5	190	99	chloride transporter chloride channel family putative expressed

ISM IDs	Rice gene locus IDs	Physical positions (bp) on chromosomes	Forward primers (5'-3')	Reverse primers (5'-3')	Melting tempearature (T°C)	Expected product size (bp) in PCR	Expected product size (bp) in Q-PCR	Putative Functions
OsISM06999	LOC_Os01g50880	29225512	GGGGAACATGTATAATGACATCAA	GCATGGATCAGCAAACTCAA	59.8	130	77	hypothetical protein
OsISM07001	LOC_Os01g50934	29246040	AGCTGAGGGTCTTCGTGGT	GGCCTAACAACAGCTGGAAG	59.9	1874	89	fiber protein Fb11 putative expressed
OsISM07002	LOC_Os01g50970	29284938	GGGGTGCAGGAATTACATTG	TGAACTTGGTCACCTTCTTCG	60.2	209	98	expressed protein
OsISM07003	LOC_Os01g50970	29285757	CTTTCCATCTGTGGAGAACCT	GCTCAAACAAGTCAATAATGCG	59.0	167	93	expressed protein
OsISM07004	LOC_Os01g50980	29289565	GACAATCAGGCATCACATCG	CATATCCTGGAGGGACGGTA	59.9	289	50	cullin putative
OsISM07005	LOC_Os01g50980	29289959	AATCAGAGGTCACTTCCACCT	TTCTGGATCCTTTCCTCTTCC	58.7	613	96	cullin putative
OsISM07006	LOC_Os01g50980	29290575	TTTGAAGCCATGGACTGAGG	AAGTAGCCATTCAGCTTGCC	60.3	222	77	cullin putative
OsISM07007	LOC_Os01g50980	29290817	TAGAGGTATCCTTGCGGCAG	CCACCAGAAATCCTGGAGAA	60.2	178	68	cullin putative
OsISM07008	LOC_Os01g50980	29290978	TCCAGGATTTCTGGTGGTTT	CACCTTTTCCATGTGTGCAG	59.8	186	99	cullin putative
OsISM07009	LOC_Os01g50980	29291114	TTAGGGACATCCTGCAACAG	CAGGATTCTACAAATGCTGACC	58.7	1112	96	cullin putative
OsISM07010	LOC_Os01g50980	29292160	TTGTGCAATCCACAGGGTAA	CATGCGCTTCGTATTTTTGA	59.9	221	92	cullin putative
OsISM07011	LOC_Os01g50980	29292364	AAAATACGAAGCGCATGTGA	CAGCAATGTTTCTGTTGCAG	58.7	221	99	cullin putative
OsISM07012	LOC_Os01g50980	29292634	ACAACGTACATGTGGCAAGC	TGTGCAGGTACGGTAGAAGC	59.3	174	81	cullin putative
OsISM07013	LOC_Os01g50980	29292788	GCTTCTACCGTACCTGCACA	TGTCTCGACCTCAATGTTGC	59.4	201	98	cullin putative
OsISM07014	LOC_Os01g50980	29293175	AAACAGCATTTATCGCTCCC	TCTCGAAAGCTTCAGCACAA	59.5	148	53	cullin putative
OsISM07015	LOC_Os01g50980	29293411	TGTTGGAAACTTTGAAGGCA	CTTCATTGAATAGCAGCAGCA	59.3	169	78	cullin putative
OsISM07016	LOC_Os01g50980	29293701	CAAAACAATTGCTCCAGACG	AATCACCCCTGTCAACATGA	59.0	184	99	cullin putative
OsISM07017	LOC_Os01g50980	29293996	TGGAGGAATGCAAGAAGTTG	GCGAGCAAGATAATTTTCGG	59.3	369	99	cullin putative

ISM IDs	Rice gene locus IDs	Physical positions (bp) on chromosomes	Forward primers (5'-3')	Reverse primers (5'-3')	Melting tempearature (T˚C)	Expected product size (bp) in PCR	Expected product size (bp) in Q-PCR	Putative Functions
OsISM07018	LOC_Os01g50990	29297641	CAGGGGTTGAGGTCACTAGG	GGTACTTGGGGTTGCTCTTG	59.6	178	97	FBD domain containing protein putative expressed
OsISM07019	LOC_Os01g50990	29297883	TGTTGTGCAGTTTCCTCCAG	CAGTGGCATTGTTGAGTTCG	60.1	187	85	FBD domain containing protein putative expressed
OsISM07020	LOC_Os01g51000	29300298	TGCCTCCTTCCTCTCAATTC	GCATATGCCACATGTTCTTTG	59.2	714	94	hypothetical protein
OsISM07021	LOC_Os01g51000	29301037	GAGAAAGTGGGTGGTGTTGAG	TGAATTCCCATGCTTCTTGA	59.4	335	98	hypothetical protein
OsISM07022	LOC_Os01g51010	29305698	GTCCTGCACAAGGGCTTC	GTTCCTCAGCAGCTTGATCC	59.7	211	79	DUF292 domain containing protein expressed
OsISM07023	LOC_Os01g51010	29305931	AGATGCGTCGAGAGGTCG	GCTTGCATGAACTTCTCTTCC	59.6	862	98	DUF292 domain containing protein expressed
OsISM07024	LOC_Os01g51010	29306815	TGTGAACTTATGGTGGCACG	CACTGGCTATGGCTTCTTTCA	60.5	1122	80	DUF292 domain containing protein expressed
OsISM07025	LOC_Os01g51010	29308018	AGTTTGCAGCAGCAGCTCTT	TTTTGGTCTGTGTATCAGGTGC	60.3	214	97	DUF292 domain containing protein expressed
OsISM07026	LOC_Os01g51010	29308265	ACATCAAATGGGAGCCAAAA	TTCCTCCCGGATATGTTGAG	60.1	168	88	DUF292 domain containing protein expressed
OsISM07027	LOC_Os01g51010	29308692	TGCTGGAGGTTCAAATGTTTC	TGAACTCTTCCCTCCTGGAC	59.7	166	87	DUF292 domain containing protein expressed
OsISM07028	LOC_Os01g51020	29314422	GGTTCAGTTCAGCAAGGAGC	CCGTCACGGTTTTCTTGTTT	60.0	185	84	60S ribosomal protein L28-1 putative expressed
OsISM07029	LOC_Os01g51020	29314674	GCCTGCTAGCCTCTACCACA	GGTCAGGCCGGTAGTAGTTG	60.1	799	99	60S ribosomal protein L28-1 putative expressed
OsISM07030	LOC_Os01g51080	29345296	GGAGGATCTGTGCCAACAGT	AGGGATGCATGGTATGGTGT	60.1	160	93	retrotransposon protein putative unclassified
OsISM07031	LOC_Os01g51080	29346753	GGTCAAGGAGGTTGCCCT	GGTGCTCAATCACCTCCCT	60.1	170	87	retrotransposon protein putative unclassified
OsISM07032	LOC_Os01g51090	29348688	ATTTTGTGGCAGAGTGGACC	GGCGAACCAAATATCTGAGG	59.8	188	97	retrotransposon protein putative unclassified
OsISM07033	LOC_Os01g51110	29356606	AGGGGTGCTCCAGGACAG	AAAGCAAAACCAGACATCGG	60.7	359	91	hypothetical protein
OsISM07034	LOC_Os01g51120	29363791	GTACTGCGAGGTGTCCGC	CTCGGACAGCTTCTGCTTCT	60.2	198	86	vesicle transport v-SNARE protein putative expressed
OsISM07035	LOC_Os01g51120	29363969	AGAAGCAGAAGCTGTCCGAG	GTTGTAGGCTCCTCGCCTC	59.9	325	88	vesicle transport v-SNARE protein putative expressed

ISM IDs	Rice gene locus IDs	Physical positions (bp) on chromosomes	Forward primers (5'-3')	Reverse primers (5'-3')	Melting tempearature (T˚C)	Expected product size (bp) in PCR	Expected product size (bp) in Q-PCR	Putative Functions
OsISM07036	LOC_Os01g51120	29364420	AGTCAGGCTTGGCTGACACT	TTGTCATCATCAATCTTCCCC	59.9	1253	61	vesicle transport v-SNARE protein putative expressed
OsISM07037	LOC_Os01g51120	29365762	GATCTTCATCAACAACGGCA	GCCGCCAGGATCTTTTTACT	60.1	754	93	vesicle transport v-SNARE protein putative expressed
OsISM07038	LOC_Os01g51154	29404768	ATCCAGAAGGATCCGGAGTT	CCCAGTCCACTTGCACTAAAA	60.0	2692	99	single myb histone putative expressed
OsISM07039	LOC_Os01g51154	29407574	ATCCGCAGATCCTGAGAAAA	TCCCCAATTTCACAAAGAGC	59.9	572	85	single myb histone putative expressed
OsISM07040	LOC_Os01g51154	29408161	TGTTGATGCAATTTGCCACT	TTTCGCTGCTATAAGCCTCC	59.9	647	98	single myb histone putative expressed
OsISM07041	LOC_Os01g51154	29408788	GGAGGCTTATAGCAGCGAAA	TGGAGACTTATTTGCTTGCG	59.3	829	96	single myb histone putative expressed
OsISM07042	LOC_Os01g51154	29409786	TCAAGCTTGCTGAGGAGACC	GCGCCACTTGCATTATTGTT	60.9	179	97	single myb histone putative expressed
OsISM07043	LOC_Os01g51180	29417838	GTCCATCTGGACAGGCAAGT	GTGGTTCCTGTCAATCCCAC	60.2	480	75	transposon protein putative unclassified
OsISM07045	LOC_Os01g51180	29418829	CTAGCAAGGCTTGGTTGCTC	AACCACAACAGGTACGCCTC	60.1	184	98	transposon protein putative unclassified
OsISM07046	LOC_Os01g51180	29419323	ACGATGGGGACTTACTGGTG	GGAGACGGTCAAACTCGAAC	59.8	186	95	transposon protein putative unclassified
OsISM07047	LOC_Os01g51180	29419681	CGGACTTAGGCAGGTGTTTC	CTAGCTGTCCCCGTCGAGT	60.1	159	81	transposon protein putative unclassified
OsISM07048	LOC_Os01g51180	29420055	GACCGAGACTACTTCGTGGC	ATATCGGCACTGATATCCCG	59.8	156	75	transposon protein putative unclassified
OsISM07050	LOC_Os01g51180	29420767	AGCTCTTCCTCTCACGGAGC	AGACGAAGAGGCCCCAGTA	60.5	144	82	transposon protein putative unclassified
OsISM07051	LOC_Os01g51180	29421086	ACCGACTCACGTACTCCCAC	GCAAGGGCGATTTTGTACTG	60.3	873	76	transposon protein putative unclassified
OsISM07052	LOC_Os01g51190	29422756	AAGGTCCTCCAGAAGAAGGG	GATCCAGAGCCTGTTCAACG	60.2	191	91	expressed protein
OsISM07053	LOC_Os01g51190	29422955	CCTCATGGACATGTGCGG	AGTATGGCGAGGCCACAG	61.0	192	97	expressed protein
OsISM07054	LOC_Os01g51190	29423194	ACTGGGTCGCCATCACAT	ACTTTCTGCTCCTCGCCTC	59.8	208	68	expressed protein
OsISM07055	LOC_Os01g51190	29423434	TGGTGCTCTCCATTGTCATC	ACGCCTTTGCCTTTTGTAGA	59.8	475	69	expressed protein

ISM IDs	Rice gene locus IDs	Physical positions (bp) on chromosomes	Forward primers (5'-3')	Reverse primers (5'-3')	Melting tempearature (T°C)	Expected product size (bp) in PCR	Expected product size (bp) in Q-PCR	Putative Functions
OsISM07056	LOC_Os01g51190	29423889	TCTACAAAAGGCAAAGGCGT	CCCAAACAGAATGCCAGATT	59.9	168	90	expressed protein
OsISM07057	LOC_Os01g51190	29424028	ATGGATTGGAATCTGGCATT	GCCGGTACAACAATCTTTGG	59.8	942	98	expressed protein
OsISM07058	LOC_Os01g51190	29424953	AAGATTGTTGTACCGGCTGC	CCTTCCATGCTTCAGACCTC	60.0	952	88	expressed protein
OsISM07059	LOC_Os01g51190	29425980	GAACATCTTCCCACAGCTCC	TCCGGGTTGAACTAACAAGAA	59.6	440	95	expressed protein
OsISM07060	LOC_Os01g51200	29430874	TTTTTCCTCTGGGGAAGGAG	ACACCAGAAACCGATCAAGC	60.3	178	62	CK1 CaseinKinase 1.3 - CK1 includes the casein kinase 1 kinases expressed
OsISM07061	LOC_Os01g51200	29431030	GTGCTTGATCGGTTTCTGGT	AACAAAGCAATCACATGCAAA	59.7	755	86	CK1 CaseinKinase 1.3 - CK1 includes the casein kinase 1 kinases expressed
OsISM07062	LOC_Os01g51200	29431772	TGATTGCTTTGTTTCTTATACAGG	AATTCCGGCTGCAAGAATAA	58.7	185	99	CK1 CaseinKinase 1.3 - CK1 includes the casein kinase 1 kinases expressed
OsISM07063	LOC_Os01g51200	29432000	TGGTTATGGATTTGCTGGGT	CAAGAGAGGGACCGAGATCA	60.3	349	93	CK1 CaseinKinase 1.3 - CK1 includes the casein kinase 1 kinases expressed
OsISM07064	LOC_Os01g51200	29432329	TGATCTCGGTCCCTCTCTTG	TGGTTAAACAACCCAGCAAA	59.7	189	87	CK1 CaseinKinase 1.3 - CK1 includes the casein kinase 1 kinases expressed
OsISM07065	LOC_Os01g51200	29432500	TGCTGGGTTGTTTAACCACTT	GAACCTTGACAAAATTGACCC	59.0	422	94	CK1 CaseinKinase 1.3 - CK1 includes the casein kinase 1 kinases expressed
OsISM07066	LOC_Os01g51200	29432883	AGGGCATTGCACATAAAAGG	AGTCAGACCTATGTGCAGCG	59.5	277	94	CK1 CaseinKinase 1.3 - CK1 includes the casein kinase 1 kinases expressed
OsISM07067	LOC_Os01g51200	29433140	CGCTGCACATAGGTCTGACT	TTGATGCATTGTACATAAAAGGG	59.0	165	87	CK1 CaseinKinase 1.3 - CK1 includes the casein kinase 1 kinases expressed
OsISM07068	LOC_Os01g51200	29433326	AGTCTTCCTTGGCAGGGTCT	AATTGAAAGGAAGCACTGGC	59.8	136	56	CK1 CaseinKinase 1.3 - CK1 includes the casein kinase 1 kinases expressed
OsISM07069	LOC_Os01g51200	29433528	ATTTCCACTATTGCCGCTCA	TGAAAACCTGCAAAGCAGAA	60.1	269	95	CK1 CaseinKinase 1.3 - CK1 includes the casein kinase 1 kinases expressed
OsISM07070	LOC_Os01g51200	29433777	TTCTGCTTTGCAGGTTTTCA	GCCCTGCAACATAGGCTACT	59.5	195	97	CK1 CaseinKinase 1.3 - CK1 includes the casein kinase 1 kinases expressed
OsISM07071	LOC_Os01g51200	29433929	CATCTGTTGGGGAAGTTTTGA	AAACAAACTCATTATTTGTGTGCAA	59.9	281	77	CK1 CaseinKinase 1.3 - CK1 includes the casein kinase 1 kinases expressed
OsISM07072	LOC_Os01g51200	29434286	CACCCCCTATTGCAAGTGTT	CAGCCAATAAGTAACAAACTGGAA	59.7	383	68	CK1 CaseinKinase 1.3 - CK1 includes the casein kinase 1 kinases expressed
OsISM07073	LOC_Os01g51220	29441739	GAAGCAGTACGCCAACGTG	TGAGAACAGGAACGCAAATG	60.2	1902	83	transport protein particle component Bet3 domain containing protein expressed

ISM IDs	Rice gene locus IDs	Physical positions (bp) on chromosomes	Forward primers (5'-3')	Reverse primers (5'-3')	Melting tempearature (T˚C)	Expected product size (bp) in PCR	Expected product size (bp) in Q-PCR	Putative Functions
OsISM07074	LOC_Os01g51220	29443625	TGCGTTCCTGTTCTCAGAGTT	CACCAACAGCATAACCAGCA	60.4	612	99	transport protein particle component Bet3 domain containing protein expressed
OsISM07075	LOC_Os01g51220	29444206	AGGCTGGAAGATGCTGGTTA	CTAGCAGTCGAGTCTCTCGC	59.0	211	92	transport protein particle component Bet3 domain containing protein expressed
OsISM07076	LOC_Os01g51220	29444429	TCATTCACAGCACTGTATGGAA	TCATCCTCATGTTCTGTTCCTTT	59.6	523	77	transport protein particle component Bet3 domain containing protein expressed
OsISM07077	LOC_Os01g51220	29444930	AAGGAACAGAACATGAGGATGAA	CCACAATTGAATGCTCCCAT	60.4	168	98	transport protein particle component Bet3 domain containing protein expressed
OsISM07078	LOC_Os01g51220	29445092	TTGTGGAGCTTTTGTTGCAG	AATTGGCACAAAGTGTGCAG	59.9	171	86	transport protein particle component Bet3 domain containing protein expressed
OsISM07079	LOC_Os01g51220	29445272	CAAAGGCCAAGGACAACAAT	GCCAAATCTAGCTTCTCGCT	59.4	592	67	transport protein particle component Bet3 domain containing protein expressed
OsISM07081	LOC_Os01g51250	29458036	GAGCGCCTCAAGATATTGCT	AGGACGCCAAGAGAACTGAA	59.8	929	57	mitochondrial carrier protein putative expressed
OsISM07082	LOC_Os01g51250	29458990	CATGATGGAATTTTGGGCTT	CCTGTATCGTTCATACGCCA	59.7	184	97	mitochondrial carrier protein putative expressed
OsISM07083	LOC_Os01g51250	29459271	ATCCCTTGGATCTGGCTAGG	TTGGTTGAAAGTTTGTCCTCTTC	60.0	734	91	mitochondrial carrier protein putative expressed
OsISM07084	LOC_Os01g51250	29460055	GTGCGAGCTCTCTATCGTGG	ATGTGCCTTCAGTCCTTCGT	60.4	275	94	mitochondrial carrier protein putative expressed
OsISM07085	LOC_Os01g51250	29460384	GCTGGATTATTTGGGCAGAC	GCCCACCAAATTTATCGTGA	60.1	209	95	mitochondrial carrier protein putative expressed
OsISM07086	LOC_Os01g51250	29460644	GATGGAGGCAATTGTTTGCT	TTCATCGTGTCATATGCGGT	60.0	848	89	mitochondrial carrier protein putative expressed
OsISM07087	LOC_Os01g51260	29468695	CAAGTACATCTCCACGCACG	CAGCTCTTGCCACATCTCTG	60.0	248	79	MYB family transcription factor putative expressed
OsISM07088	LOC_Os01g51260	29469014	TCATCATTGAGCTCCACAGG	GGTAGGTGCTTGGCAATCTG	60.2	319	59	MYB family transcription factor putative expressed
OsISM07089	LOC_Os01g51260	29469382	AAGAAGCTCATCTCCCAGGC	GTCCTGCTCCGTCCAGAAT	60.5	272	80	MYB family transcription factor putative expressed
OsISM07090	LOC_Os01g51280	29478506	GATGCGACGTCCACGTATG	CACCGATGTTGCTGTAATGG	60.6	251	90	5-nucleotidase surE putative expressed
OsISM07091	LOC_Os01g51280	29478738	CATTACAGCAACATCGGTGG	AATGCCAAAGAGACGCAATC	60.1	186	82	5-nucleotidase surE putative expressed
OsISM07092	LOC_Os01g51280	29478904	GATTGCGTCTCTTTGGCATT	CTCATATCCACAGTTCGCCC	60.4	183	97	5-nucleotidase surE putative expressed

ISM IDs	Rice gene locus IDs	Physical positions (bp) on chromosomes	Forward primers (5'-3')	Reverse primers (5'-3')	Melting tempearature (T˚C)	Expected product size (bp) in PCR	Expected product size (bp) in Q-PCR	Putative Functions
OsISM07093	LOC_Os01g51280	29479048	ATCAGTGGGATCAATGCAGG	AGCAGCAATGGCAGAAGAAT	60.4	142	64	5-nucleotidase surE putative expressed
OsISM07094	LOC_Os01g51280	29479190	GCAAGGGAGGCCTTACTGTAT	ACCTCAGCAGCATCCTTGAA	60.3	213	99	5-nucleotidase surE putative expressed
OsISM07095	LOC_Os01g51280	29479470	CTGAACATTGGGGTTCCAAG	GTGGACACACCTTCCCAACT	60.1	943	99	5-nucleotidase surE putative expressed
OsISM07096	LOC_Os01g51280	29480452	CCAAAGCCTTGGTATTCAGC	TGAGCATTAATTCTGCGTGC	59.8	162	76	5-nucleotidase surE putative expressed
OsISM07097	LOC_Os01g51280	29480675	GTGAAGAAGCTTTTCCGTGC	GCATTCATGCTGCTTTTCAG	59.8	258	46	5-nucleotidase surE putative expressed
OsISM07098	LOC_Os01g51280	29480920	GCAGCATGAATGCTTAGACG	GGAGCCACATTTCCGTGTAT	59.7	183	94	5-nucleotidase surE putative expressed
OsISM07099	LOC_Os01g51300	29500310	CGGTGCTCTACGACTGGTTC	AGCCTTCTCAAACTGGGGTC	60.7	816	81	WD domain G-beta repeat domain containing protein expressed
OsISM07100	LOC_Os01g51300	29501138	CGTCAGCGTCTTTACCTTTCA	GCAACCCTTGGTTTGACAAC	60.4	271	90	WD domain G-beta repeat domain containing protein expressed
OsISM07101	LOC_Os01g51300	29501385	TGAAGTTGTCAAACCAAGGG	GGTGATCGTGCTTCCTCATT	59.3	445	67	WD domain G-beta repeat domain containing protein expressed
OsISM07102	LOC_Os01g51300	29501810	AATGAGGAAGCACGATCACC	CGGAAGCTCCCTAATCCTGT	60.3	330	82	WD domain G-beta repeat domain containing protein expressed
OsISM07103	LOC_Os01g51300	29502153	TCATTGCCACTCATACCGAC	TCTGTGCCAGTTGAGCTTGT	59.6	187	82	WD domain G-beta repeat domain containing protein expressed
OsISM07104	LOC_Os01g51300	29502326	TCAACTGGCACAGATGGAGT	CAAGCGCAAACTCAGCAATA	59.7	295	99	WD domain G-beta repeat domain containing protein expressed
OsISM07105	LOC_Os01g51300	29502601	TATTGCTGAGTTTGCGCTTG	GGATGCTCCACCATACAACA	59.8	634	87	WD domain G-beta repeat domain containing protein expressed
OsISM07106	LOC_Os01g51300	29503378	GAAGATGTCCAGTTCTGCCC	GGGCATCCCAAAGAATAAGA	59.3	344	80	WD domain G-beta repeat domain containing protein expressed
OsISM07107	LOC_Os01g51300	29503703	CTTATTCTTTGGGATGCCCG	TGGAGGTTCCAGTCAACACA	60.9	198	93	WD domain G-beta repeat domain containing protein expressed
OsISM07108	LOC_Os01g51300	29503860	GCTCATGGTGGTGATGTTCA	GGTCCCACATACGGACAGAA	60.7	245	95	WD domain G-beta repeat domain containing protein expressed
OsISM07109	LOC_Os01g51300	29504129	CTGGCATTCCAGTTCACAAA	TCCGCAGAGCTTCCAAATAC	60.0	202	95	WD domain G-beta repeat domain containing protein expressed
OsISM07110	LOC_Os01g51300	29504334	CGGCTTCTTAAATGTGTGGG	GCATGTTGAAAGAAAAGCCC	60.1	236	82	WD domain G-beta repeat domain containing protein expressed

ISM IDs	Rice gene locus IDs	Physical positions (bp) on chromosomes	Forward primers (5'-3')	Reverse primers (5'-3')	Melting tempearature (T˚C)	Expected product size (bp) in PCR	Expected product size (bp) in Q-PCR	Putative Functions
OsISM07111	LOC_Os01g51300	29504550	GGGCTTTTCTTTCAACATGC	CAAGGATCCGAAGAATTCCA	59.8	749	69	WD domain G-beta repeat domain containing protein expressed
OsISM07112	LOC_Os01g51300	29505333	ACTGGTGGAGGTGGAACATT	TGCAAGAACTTCATCTTCTGGA	59.6	212	73	WD domain G-beta repeat domain containing protein expressed
OsISM07113	LOC_Os01g51380	29536793	CACGGAGGAAGGAACTCTTG	AGAGGACCAGCAACTCCAGA	59.9	186	64	ATP synthase putative expressed
OsISM07114	LOC_Os01g51380	29536959	TCTGGAGTTGCTGGTCCTCT	ACGCCTAGTTGTGCCATCTC	60.1	203	97	ATP synthase putative expressed
OsISM07115	LOC_Os01g51380	29537161	TGGTCAAGTGCTTGAAGTCG	CCGGTGAATTGTACAGTTGTG	59.5	582	97	ATP synthase putative expressed
OsISM07116	LOC_Os01g51380	29537686	GGTTTTCGAAGGAACATCTGG	CGTCCAAGCATATCCAGTGA	60.3	335	97	ATP synthase putative expressed
OsISM07117	LOC_Os01g51380	29538025	TTCAATGGTTCTGGGAAACC	TGTTCGTTCACTGGGATTGA	59.9	188	97	ATP synthase putative expressed
OsISM07118	LOC_Os01g51380	29538344	TCAGGCTGGTCTGGTAAAGC	CAGCAAAGACAATCGCAAAA	60.2	483	84	ATP synthase putative expressed
OsISM07119	LOC_Os01g51380	29538872	TTGAGGAAAATGGTTCAATGG	GAGAGCAATTCTTGGGGTGA	60.0	199	93	ATP synthase putative expressed
OsISM07120	LOC_Os01g51380	29539136	CTCATATGCCGATGCACTTC	CGTCCAGGTACTTCCTCTCG	59.6	637	55	ATP synthase putative expressed
OsISM07121	LOC_Os01g51380	29539825	GCTGGTCGCATAGAAGGAAG	CCAGTAAGATCAGGAGTTGGAT G	60.0	298	96	ATP synthase putative expressed
OsISM07122	LOC_Os01g51380	29540100	CATCCAACTCCTGATCTTACT GG	GGAAGGACATTAATCGGTGG	59.6	282	99	ATP synthase putative expressed
OsISM07123	LOC_Os01g51380	29540350	TGGCAGATCTATCCACCGAT	ATCAGAATGATCCCTGCGAG	60.3	218	94	ATP synthase putative expressed
OsISM07124	LOC_Os01g51380	29540544	ATGACTCGCAGGGATCATTC	CTTCATCGCCTGTACGTCCT	60.2	170	79	ATP synthase putative expressed
OsISM07125	LOC_Os01g51380	29540694	AGGACGTACAGGCGATGAAG	AACTTCCGCTCGAACTTGTC	59.9	194	95	ATP synthase putative expressed
OsISM07126	LOC_Os01g51390	29544203	ATCCTCCAGTACCCGTGCTT	ACCTCGTCCATCATTCCTTG	60.4	708	96	mitochondrial-processing peptidase subunit mitochondrial precursor putative expressed
OsISM07127	LOC_Os01g51390	29544994	CGATCTCTAAGAAAGATTTGG AGC	ACAGCTCCAGCAGCAGACAC	60.9	175	80	mitochondrial-processing peptidase subunit mitochondrial precursor putative expressed
OsISM07128	LOC_Os01g51390	29545245	CTTGTTGAAGCAAATCCAGC	GCAAAGTGCGTTAGAGGCAT	59.5	179	78	mitochondrial-processing peptidase subunit mitochondrial precursor putative expressed

ISM IDs	Rice gene locus IDs	Physical positions (bp) on chromosomes	Forward primers (5'-3')	Reverse primers (5'-3')	Melting tempearature (T˚C)	Expected product size (bp) in PCR	Expected product size (bp) in Q-PCR	Putative Functions
OsISM07129	LOC_Os01g51390	29545501	GGAATAGGAGCATTGGGGTT	TACCGTTGCTGATACCACGA	60.1	178	64	mitochondrial-processing peptidase subunit mitochondrial precursor putative expressed
OsISM07130	LOC_Os01g51390	29545727	GATTGTTTGGCATCTGCACT	GACACTTCAAATGCAAGTCTTCT	58.2	320	95	mitochondrial-processing peptidase subunit mitochondrial precursor putative expressed
OsISM07131	LOC_Os01g51390	29546049	AAACAGAGGTTGCTCGTGCT	AGAAACAGCAGTGGATCCGT	59.9	748	75	mitochondrial-processing peptidase subunit mitochondrial precursor putative expressed
OsISM07132	LOC_Os01g51390	29546777	ACGGATCCACTGCTGTTTCT	GCATTACTCGCCCATACGTT	59.9	154	61	mitochondrial-processing peptidase subunit mitochondrial precursor putative expressed
OsISM07133	LOC_Os01g51390	29546940	AGCTTTTTGCTCGCATTGAT	CACTGCAGCTAGGGCGAT	60.1	207	93	mitochondrial-processing peptidase subunit mitochondrial precursor putative expressed
OsISM07134	LOC_Os01g51400	29552030	GTCTTCGACTACGTCCCCAA	TGCCACTCCAGTGGAACAC	60.4	168	81	Leucine Rich Repeat family protein expressed
OsISM07135	LOC_Os01g51410	29560579	CACCAGCAACATCTGCACTG	GGGACCATGGTAGACAGCAT	60.7	218	71	glycine dehydrogenase putative expressed
OsISM07136	LOC_Os01g51410	29560955	CAAGAACGAGATGAACCTTCG	ACACCTTGAACAGCTTGTCG	59.4	191	99	glycine dehydrogenase putative expressed
OsISM07137	LOC_Os01g51410	29561125	TCGACAAGCTGTTCAAGGTG	CTCTGATGCGAGGGATTCAG	60.5	163	69	glycine dehydrogenase putative expressed
OsISM07138	LOC_Os01g51410	29561326	CCCCTACTTAACACACCCGA	CAACTTGTGCAGGTATCGGA	59.8	160	71	glycine dehydrogenase putative expressed
OsISM07139	LOC_Os01g51410	29561600	CACCCATTTGCACCTACTGA	GATCGCCCAAGTCATCAAAC	60.0	145	62	glycine dehydrogenase putative expressed
OsISM07140	LOC_Os01g51410	29561793	CTGGTGCATCAGGGGAGTAT	ATACAGACGTCCCGGTGGTA	60.1	168	83	glycine dehydrogenase putative expressed
OsISM07141	LOC_Os01g51410	29562089	ACAAGGACAACTTGGCTGCT	TAGACTCCATGGGTCGAAGG	60.0	559	53	glycine dehydrogenase putative expressed
OsISM07142	LOC_Os01g51410	29562696	GCAGGTATACATGGATGGGG	AAGGTGGCAAACATCTGCTC	60.1	572	83	glycine dehydrogenase putative expressed
OsISM07143	LOC_Os01g51410	29563312	TGGAATGGGTCCTATTGGTG	GGTTTTCTCAGGGAGAGGGA	60.6	709	95	glycine dehydrogenase putative expressed
OsISM07144	LOC_Os01g51410	29564146	TGCAAACTACATGGCAAAGC	CCACGGAAAAGAACTGGGTA	59.9	146	52	glycine dehydrogenase putative expressed
OsISM07145	LOC_Os01g51410	29564292	AGTCAATGGAACTGTTGCCC	AACGCTTTGCAACATCCTCT	59.9	174	90	glycine dehydrogenase putative expressed
OsISM07146	LOC_Os01g51410	29564513	GGCACACTTATGATTGAACCC	TTTCTGCAATTTCTTCCCTGA	59.6	185	95	glycine dehydrogenase putative expressed

ISM IDs	Rice gene locus IDs	Physical positions (bp) on chromosomes	Forward primers (5'-3')	Reverse primers (5'-3')	Melting tempearature (T˚C)	Expected product size (bp) in PCR	Expected product size (bp) in Q-PCR	Putative Functions
OsISM07147	LOC_Os01g51410	29564706	GGCAAAGCAGATGTGAACAA	CCATGAGTCGCTCATCAGAA	59.9	349	73	glycine dehydrogenase putative expressed
OsISM07148	LOC_Os01g51410	29565065	ACTCCAGGGAGTACGCTGC	GTTGCGATCCCCATACACAT	60.5	196	96	glycine dehydrogenase putative expressed
OsISM07149	LOC_Os01g51420	29567625	GATGGACTCCTCCCGCTC	CTCCCCCAGCGTCAGAGA	61.7	204	59	calcineurin B putative expressed
OsISM07150	LOC_Os01g51420	29567968	GCCTCACCTTCCGTGAGC	GTACAGTGCCTCCACCTCGT	61.1	171	75	calcineurin B putative expressed
OsISM07151	LOC_Os01g51420	29568152	AGATCAGCTGCTCCATCGTC	CTTTCCAGCCGGTGTTCTAA	60.4	653	81	calcineurin B putative expressed
OsISM07152	LOC_Os01g51420	29568785	TTAGAACACCGGCTGGAAAG	GCGTGAATAAACTCTTCAAATTC A	60.0	501	98	calcineurin B putative expressed
OsISM07153	LOC_Os01g51420	29569280	TCACGCAATAAGTGTATTTCA TCC	CCGGTTTGCCTCAAATCATA	60.5	184	85	calcineurin B putative expressed
OsISM07154	LOC_Os01g51420	29569458	AACCGGTTTCATTGAACGTG	TGGCTTCCACAAGATCATCA	60.5	580	93	calcineurin B putative expressed
OsISM07155	LOC_Os01g51420	29570004	GAGGTGCAGCTGTCTGATGA	CCTTGTCAGTGTCAGCATCC	59.7	153	71	calcineurin B putative expressed
OsISM07156	LOC_Os01g51420	29570191	TGTACTGAAGCACCCGTCTG	TGAAAATGAAGCTGGGGAAC	60.0	229	84	calcineurin B putative expressed
OsISM07157	LOC_Os01g51430	29573271	TCAAATCTGGTGAGCATGGA	CGCGAGATTAACAGGAAAGC	60.1	867	94	green ripe-like putative expressed
OsISM07158	LOC_Os01g51480	29586222	GTCCACCCTGCCAAGAAG	CTCCTTCTCCTCACCAGCTC	59.2	207	99	hypothetical protein
OsISM07159	LOC_Os01g51550	29610198	CCTCTTCTTCCACGACTGCT	GCTTCCCTCTCCGACTTGT	59.5	193	85	peroxidase family protein
OsISM07160	LOC_Os01g51550	29610619	CTACACCGTGACGCTCGAT	CACCTTCCCGATGCTATGTG	60.7	173	89	peroxidase family protein
OsISM07161	LOC_Os01g51550	29611080	GTGTTCCGCGAGGACTTC	TTTGCAGAACTGGTTTTCCC	59.7	1159	67	peroxidase family protein
OsISM07162	LOC_Os01g51550	29612566	GACGTTGAAGACAGGGGAGA	ACGCAGATACGAGACCAACG	60.7	716	97	peroxidase family protein
OsISM07163	LOC_Os01g51570	29619930	CTAAGATGGGTTTCGCTCCC	GTAACACACGCCGATGGAG	60.5	276	93	glycosyl hydrolases family 17 putative expressed
OsISM07165	LOC_Os01g51610	29638971	CGTAAGCCAGCTAGGGAGAA	TTGGTAGGTAAGCCTCTGCC	59.5	667	57	B3 DNA binding domain containing protein expressed

ISM IDs	Rice gene locus IDs	Physical positions (bp) on chromosomes	Forward primers (5'-3')	Reverse primers (5'-3')	Melting tempearature (T˚C)	Expected product size (bp) in PCR	Expected product size (bp) in Q-PCR	Putative Functions
OsISM07166	LOC_Os01g51610	29639684	GCAAAATGCACAACTCTGGA	ATCCTGCTCTTGTTGTTGGG	60.0	1287	58	B3 DNA binding domain containing protein expressed
OsISM07167	LOC_Os01g51610	29640951	CCCAACAACAAGAGCAGGAT	TGAAGATCATGGGTTCTGACA	59.6	155	66	B3 DNA binding domain containing protein expressed
OsISM07168	LOC_Os01g51610	29641085	TGTCAGAACCCATGATCTTCA	ATCACCTGCCTTCTTTGCTC	59.3	176	95	B3 DNA binding domain containing protein expressed
OsISM07169	LOC_Os01g51620	29645086	ATCGATTGGTTTTGCCTGGT	GACCCCAAACTCCTTCAGTG	60.6	147	82	KRR1 small subunit processome component putative expressed
OsISM07170	LOC_Os01g51620	29645213	CACTGAAGGAGTTTGGGGTC	TTCCTTGTGGTTGAAACGGT	60.0	159	71	KRR1 small subunit processome component putative expressed
OsISM07171	LOC_Os01g51620	29645420	GCTATCGAGGAGTGTCCCTG	GCACAGCTCATCTCATCATTG	59.6	257	61	KRR1 small subunit processome component putative expressed
OsISM07172	LOC_Os01g51620	29645656	CAATGATGAGATGAGCTGTGC	AAATTAGGCCCCAGAAGACG	59.9	736	97	KRR1 small subunit processome component putative expressed
OsISM07173	LOC_Os01g51620	29646372	CGTCTTCTGGGGCCTAATTT	TGTAGCAGCCAGTCAAAATCTC	60.0	168	59	KRR1 small subunit processome component putative expressed
OsISM07174	LOC_Os01g51620	29646507	TGCAGGCCATAGAGATTTTG	CAATCCCTTCCAGTAACCCA	59.3	187	81	KRR1 small subunit processome component putative expressed
OsISM07175	LOC_Os01g51620	29646699	AGTTGTGAGGGTTGTGGAGG	GCAGGATTTTTGACCAGCTC	59.9	195	97	KRR1 small subunit processome component putative expressed
OsISM07176	LOC_Os01g51620	29646911	GGGACAAGTTTCTCCCGAA	GGAGGTGGAAAGGGTGTGTA	59.9	186	92	KRR1 small subunit processome component putative expressed
OsISM07177	LOC_Os01g51620	29647085	CTTTCCACCTCCTCAGCAAC	GCCATTCCTTTGCTGACTTC	59.8	440	93	KRR1 small subunit processome component putative expressed
OsISM07178	LOC_Os01g51620	29647542	CAATCGCAGAAAGCTGAAGA	TGATGGACTTGGCAGATTCA	59.7	183	98	KRR1 small subunit processome component putative expressed
OsISM07179	LOC_Os01g51620	29647720	CATCATCAACAATGGCGAAA	CGAGTCTCACATTTTCCTGTTC	59.7	196	96	KRR1 small subunit processome component putative expressed
OsISM07180	LOC_Os01g51660	29695010	CTAGGAGGGCAAGGCTCA	GACTGTCGCTGCCAATGC	60.3	148	99	hypothetical protein
OsISM07181	LOC_Os01g51680	29716920	GCAATGGAGTTCAACCTTCC	CTGCTAGCCGCTCTTGAACT	59.7	260	71	hypothetical protein
OsISM07183	LOC_Os01g51720	29740011	TCTCAGCGGATTGAACTGTG	AACCTGACCTCAGCCAGTCA	60.4	575	96	hypothetical protein
OsISM07184	LOC_Os01g51720	29740559	ATTCTGCTGACTGGCTGAGG	TGGGCAACCTCATTGGTAAT	60.4	359	65	hypothetical protein

ISM IDs	Rice gene locus IDs	Physical positions (bp) on chromosomes	Forward primers (5'-3')	Reverse primers (5'-3')	Melting tempearature (T°C)	Expected product size (bp) in PCR	Expected product size (bp) in Q-PCR	Putative Functions
OsISM07185	LOC_Os01g51720	29741007	ACAGCTCTCCGAACCTCAAG	ACCCATGTTCTTCTCCCAGA	59.6	200	99	hypothetical protein
OsISM07186	LOC_Os01g51740	29754297	GCTTCATGCTTGCTCAACAG	TCCCAGAACTCTGCAGCTAA	59.2	185	99	hypothetical protein
OsISM07187	LOC_Os01g51754	29762713	CCAGAACCACATCCACCTG	GTTGCCTGTTCCATCAGCTT	60.1	911	85	alpha-amylase precursor putative expressed
OsISM07188	LOC_Os01g51754	29764134	TGGTCACCACCACCTACTGA	TTCAACTCTTCCATGGTCCC	60.0	1379	96	alpha-amylase precursor putative expressed
OsISM07189	LOC_Os01g51754	29765638	GGGATGATCGAGCAGTTGTT	GAGTGATCAATGTTTGGGGC	60.2	587	98	alpha-amylase precursor putative expressed
OsISM07190	LOC_Os01g51754	29766208	CCAAACATTGATCACTCGCA	AAGTCGCCATCCATCGTATC	60.3	826	94	alpha-amylase precursor putative expressed
OsISM07191	LOC_Os01g51754	29767209	CTGCTGGTGCATTTGATGTT	TTTCATCAGACAGACGCCAG	59.9	518	82	alpha-amylase precursor putative expressed
OsISM07192	LOC_Os01g51754	29767762	TCGCGTGCTGTCACATTTAT	CAACTCCATACCAAAGGGGA	60.0	440	79	alpha-amylase precursor putative expressed
OsISM07193	LOC_Os01g51754	29768304	AGAAATCGCCAAAAGATCCA	CCCTCTCATCAATCTCTGCC	59.7	190	86	alpha-amylase precursor putative expressed
OsISM07194	LOC_Os01g51790	29779235	TGCTTGTAAAAGGGGGTCTC	ACCGACGAAGGAAGAGTGAA	59.5	726	98	conserved hypothetical protein
OsISM07195	LOC_Os01g51790	29779921	TCCCCGATAATCCACTGGT	GAACGCGACTACGAACCCT	60.2	236	90	conserved hypothetical protein
OsISM07196	LOC_Os01g51820	29790105	AAGTTGTTCCCGAGCATTTG	AACCACTCAGGGTAAGGCTG	59.7	298	62	retrotransposon protein putative Ty3-gypsy subclass
OsISM07197	LOC_Os01g51820	29790785	GACGCTCAGTTAGCCGATCT	TTTACTCCTCACCCATTCGG	59.8	239	82	retrotransposon protein putative Ty3-gypsy subclass
OsISM07198	LOC_Os01g51820	29792391	TCAATGCTTGACTCAGTGGC	CACGACGCCCTCAAAATAAT	60.0	215	98	retrotransposon protein putative Ty3-gypsy subclass
OsISM07199	LOC_Os01g51820	29794602	CAGCAATAACAGCCGATGAT	CTCCACCCACTTGGTGAAAT	59.3	473	60	retrotransposon protein putative Ty3-gypsy subclass
OsISM07200	LOC_Os01g51860	29816028	GTCTCCATAGTAGGCGACGG	CCTTCGTCAACCCACTTCAT	59.8	1147	99	violaxanthin de-epoxidase putative expressed
OsISM07202	LOC_Os01g51890	29829430	GCAAGGGAAGGAAGCAGAA	ATGTTGAGCCATTTCCGAAG	60.3	188	59	endonuclease/exonuclease/phosphatase family domain containing protein expressed
OsISM07203	LOC_Os01g51890	29829670	GACGACACCGATAGCGAGTT	CGCAGTCTCCTCTCCTCATC	60.2	1180	78	endonuclease/exonuclease/phosphatase family domain containing protein expressed

ISM IDs	Rice gene locus IDs	Physical positions (bp) on chromosomes	Forward primers (5'-3')	Reverse primers (5'-3')	Melting tempearature (T˚C)	Expected product size (bp) in PCR	Expected product size (bp) in Q-PCR	Putative Functions
OsISM07204	LOC_Os01g51890	29830830	GATGAGGAGAGGAGACTGCG	GTAGGGAGCACCCTCCATTT	60.2	463	67	endonuclease/exonuclease/phosphatase family domain containing protein expressed
OsISM07205	LOC_Os01g51890	29831302	AGAAGGCGCAAGTCAGAGAC	ACATTCCATGTTCCTACGCA	59.4	310	78	endonuclease/exonuclease/phosphatase family domain containing protein expressed
OsISM07206	LOC_Os01g51890	29831662	TGGAGGAGCCAGCTGATATT	AGGACGGTTGTCTTCAGCAC	60.1	200	90	endonuclease/exonuclease/phosphatase family domain containing protein expressed
OsISM07207	LOC_Os01g51890	29832570	GTAGGTGTCGGTGCTATGGG	CAGGTGGCAACATATAAAGCAA	60.2	406	97	endonuclease/exonuclease/phosphatase family domain containing protein expressed
OsISM07208	LOC_Os01g51890	29833053	TCCGGTTTCCAGAGTTAGCA	TCCTATAATTGAGATCTCCTAAC CA	59.1	607	78	endonuclease/exonuclease/phosphatase family domain containing protein expressed
OsISM07209	LOC_Os01g51890	29833696	CCATGAAGGACTGGAATGGT	CACCCATCAAATAAATGCCC	59.9	169	77	endonuclease/exonuclease/phosphatase family domain containing protein expressed
OsISM07210	LOC_Os01g51890	29833924	ACACGAGTGATGAGCCAAAA	CCCTTGCCAAAGGAAAGAAT	59.9	620	71	endonuclease/exonuclease/phosphatase family domain containing protein expressed
OsISM07212	LOC_Os01g51940	29853802	ATTGTCTCCGCCACCGTAG	TGTTCTCTGGTCCTGTTTCTGA	60.5	588	89	hypothetical protein
OsISM07213	LOC_Os01g51950	29860162	AGATGAAACCTGGAGAAGCG	AGACATGTCAACAGACTCCTGA A	59.1	193	69	retrotransposon protein putative unclassified
OsISM07214	LOC_Os01g51950	29860858	CATCTCCGATGTTTGCTTCA	TTGAGCAATCCAAAACATCA	58.7	447	93	retrotransposon protein putative unclassified
OsISM07215	LOC_Os01g51950	29861513	CCTCAACATGATTTTGGATCA	TCTCACCACTTCTAGCACCAG	58.3	125	50	retrotransposon protein putative unclassified
OsISM07216	LOC_Os01g51950	29861605	TTGTGTTAGGTGCTGGTGCT	ATTCTCCTTCCTTGCCACAA	59.5	669	49	retrotransposon protein putative unclassified
OsISM07217	LOC_Os01g51950	29862967	ATGGCAACTAAGGACGAAGC	GTCGTCGTTGTCATCGCTC	59.9	661	91	retrotransposon protein putative unclassified
OsISM07218	LOC_Os01g51970	29870214	GACTAAACCCGACGATGAGC	CAGAAGATGCAACGATGTGG	60.0	690	96	hypothetical protein
OsISM07219	LOC_Os01g51970	29870884	CCACATCGTTGCATCTTCTG	CACGAACACATTGGTGATTGA	60.3	350	78	hypothetical protein
OsISM07220	LOC_Os01g51970	29871207	TCCACTTCAATCACCAATGTG	TCACAAGTGCAATGACACTGA	59.1	1030	70	hypothetical protein
OsISM07221	LOC_Os01g51970	29872163	TTTTCATTGGGAATCATGTGA C	TGAGGACATATAAATAACTGCTC TGAA	59.7	386	98	hypothetical protein
OsISM07223	LOC_Os01g51980	29873772	AACAGCTGCTTCTCCGGTC	AATACGGCTCCAGGAACAAC	59.8	949	92	alliin lyase precursor putative expressed

ISM IDs	Rice gene locus IDs	Physical positions (bp) on chromosomes	Forward primers (5'-3')	Reverse primers (5'-3')	Melting tempearature (T˚C)	Expected product size (bp) in PCR	Expected product size (bp) in Q-PCR	Putative Functions
OsISM07225	LOC_Os01g51980	29875340	CTTCACCACGTCCAAGCTCT	TGTAGGATATCGCCCTGCTC	60.3	200	93	alliin lyase precursor putative expressed
OsISM07226	LOC_Os01g51980	29875749	TTCAACCGGATCAAAGAACC	AGCGTCTCGTAGCAGTCGTC	60.3	196	81	alliin lyase precursor putative expressed
OsISM07227	LOC_Os01g52010	29893315	AGTGCAACCGCTGCTTCT	AGTACGGCTCCAGGAACAAC	59.5	2377	97	alliin lyase precursor putative expressed
OsISM07229	LOC_Os01g52010	29896318	AGGACGTCATGCTCTTCACC	GCCACCTTCTCGTCCCTTAT	60.4	193	86	alliin lyase precursor putative expressed
OsISM07230	LOC_Os01g52010	29896739	CTTCAACCGGATCAAAGAGC	AGCAGCGTCTCGTAGCAGTC	60.4	188	85	alliin lyase precursor putative expressed
OsISM07232	LOC_Os01g52070	29942933	CGTCGTTAACCCTCACACCT	GTGTACAGCACCAGCACGAT	59.9	166	67	potassium channel AKT1 putative expressed
OsISM07233	LOC_Os01g52070	29943247	GTAACCGACCGCAAGAAGAT	GTAAAGGAATCGTTGAGGCG	59.5	442	80	potassium channel AKT1 putative expressed
OsISM07234	LOC_Os01g52070	29943739	CTCAATCTTCTCCGGCTCTG	TTGATAAGCCTGGTCCACAA	59.6	1621	99	potassium channel AKT1 putative expressed
OsISM07235	LOC_Os01g52070	29945340	TTGTGGACCAGGCTTATCAA	CATCCACAGGTAGATGCACG	59.6	174	76	potassium channel AKT1 putative expressed
OsISM07236	LOC_Os01g52070	29945758	ACGAAACCACCAACACCTTC	ACGTCCTCTGCACCATGTC	59.8	122	46	potassium channel AKT1 putative expressed
OsISM07237	LOC_Os01g52070	29946046	CCAGAGCTGCTACCTGTTCC	GGGAAATGACTCTGCCTTCA	60.1	171	80	potassium channel AKT1 putative expressed
OsISM07238	LOC_Os01g52070	29946223	GCGGATATCATCCTGGAGAA	ACCATCCTCAAGGGTTGTCA	60.2	178	88	potassium channel AKT1 putative expressed
OsISM07239	LOC_Os01g52070	29946384	CAACCCTTGAGGATGGTACAG	CGTGGCCCTATTCTCATCAC	60.0	520	47	potassium channel AKT1 putative expressed
OsISM07240	LOC_Os01g52070	29947022	CCTAACACTGCAGATGGGGT	GCTGTTTCCTTTGCCTTCAC	59.9	156	81	potassium channel AKT1 putative expressed
OsISM07241	LOC_Os01g52070	29947350	TCATCTGGGAAGCTCGTCAT	CCACGGTGAGAATCCCTCT	60.4	195	98	potassium channel AKT1 putative expressed
OsISM07242	LOC_Os01g52100	29961463	CTGCAGCAGGACAGGGATA	TAGTCCCTCCTGCACTCGTT	59.9	180	72	DUF581 domain containing protein expressed
OsISM07243	LOC_Os01g52120	29970770	GAAGGCAAGCAGCAGCAG	TCAATTGAATCAGGGAGAGGA	60.3	129	57	expressed protein
OsISM07244	LOC_Os01g52130	29987601	AACCTCCCTCCAATCATCG	CGAACACCGCGTACATCA	60.1	160	62	sulfate transporter putative expressed

ISM IDs	Rice gene locus IDs	Physical positions (bp) on chromosomes	Forward primers (5'-3')	Reverse primers (5'-3')	Melting tempearature (T˚C)	Expected product size (bp) in PCR	Expected product size (bp) in Q-PCR	Putative Functions
OsISM07245	LOC_Os01g52130	29987888	CTTCTTCACCGGCCTCTTC	CGCGAGAGGAAATCCACTAT	59.6	213	70	sulfate transporter putative expressed
OsISM07246	LOC_Os01g52130	29988212	CCTGCACTCCACCTACCATT	AGAGGAAGCATATGCCGAGA	60.0	1994	69	sulfate transporter putative expressed
OsISM07247	LOC_Os01g52130	29990186	TCTCGGCATATGCTTCCTCT	CCGACACCCAGAACAACTTT	60.0	204	78	sulfate transporter putative expressed
OsISM07248	LOC_Os01g52130	29990423	GCATCTTCGCTTTCCTTGTC	GGGGATTGATCCCTTTATTCA	60.0	186	76	sulfate transporter putative expressed
OsISM07249	LOC_Os01g52130	29990661	AGCTGGTCTCTTGTCTGGGA	TCGATCTGCTCGTTCTTGAC	59.6	187	85	sulfate transporter putative expressed
OsISM07250	LOC_Os01g52130	29990871	TTGGTATCATGAACATCGCC	GAAGTTCACCGCCGACTTT	59.8	173	78	sulfate transporter putative expressed
OsISM07251	LOC_Os01g52130	29991231	ACTTCTGCATCTGCATGGTC	CAACGCCCTGACAACTGAC	59.6	195	96	sulfate transporter putative expressed
OsISM07252	LOC_Os01g52130	29991559	ACTTCGTCAATGCGGGTTAC	AAGTATTGCAGATCGTGCCC	60.1	186	95	sulfate transporter putative expressed
OsISM07253	LOC_Os01g52130	29991727	GCACGATCTGCAATACTTGG	TCGTTCGAGGCTCTTGTGTA	59.4	211	98	sulfate transporter putative expressed
OsISM07254	LOC_Os01g52130	29991921	ACAAGAGCCTCGAACGAAGA	CCTGACGTATCCAGACAGCA	60.0	569	90	sulfate transporter putative expressed
OsISM07255	LOC_Os01g52140	29994836	TACATGCACGATGACCGTTT	TAACTTTTGGCCACCGAAAT	59.7	646	79	expressed protein
OsISM07256	LOC_Os01g52140	29995549	GGCTTTATGAGACGAACTCCC	CCACCCCATGCTCTAAAAGA	60.1	193	77	expressed protein
OsISM07257	LOC_Os01g52140	29996085	CATTGGAGGAGTGATCCAGAG	CTTCTGCATTGGAAGAAGCC	59.8	172	77	expressed protein
OsISM07258	LOC_Os01g52160	30002900	CCAAGACTCTCGCTTTCCAT	GCTTCTGAACTTTCTTGGCG	59.8	202	88	heavy metal-associated domain containing protein expressed
OsISM07259	LOC_Os01g52160	30003082	CGCCAAGAAAGTTCAGAAGC	ATCCCCTATCACCACCACCT	60.3	1476	92	heavy metal-associated domain containing protein expressed
OsISM07262	LOC_Os01g52170	30007894	CAAGGACCTGGAGATTAGCG	CGTCAGCTCCAGACGAAAC	59.7	148	56	expressed protein
OsISM07263	LOC_Os01g52170	30008000	TTGGCTCTGCTTTTTCAGGT	GCAACAACCTCATTTGCCTT	60.1	366	96	expressed protein
OsISM07264	LOC_Os01g52170	30008437	AGCTTCCAGAAGGATTGCAG	GCAACTGTAGACTTTCCAACTCC	59.5	151	80	expressed protein

ISM IDs	Rice gene locus IDs	Physical positions (bp) on chromosomes	Forward primers (5'-3')	Reverse primers (5'-3')	Melting tempearature (T˚C)	Expected product size (bp) in PCR	Expected product size (bp) in Q-PCR	Putative Functions
OsISM07265	LOC_Os01g52170	30008648	TGTCTTCGGTCCGAGCTTAC	CCCAAATACTCGGTTGGAAG	59.9	173	97	expressed protein
OsISM07266	LOC_Os01g52170	30008864	ATAATGCGTGGTCCAATGGT	GTTCCTGGAGGCATGTCAAT	60.0	252	99	expressed protein
OsISM07267	LOC_Os01g52170	30009096	ATTGACATGCCTCCAGGAAC	GGGGTGGTAACAATGACAGC	60.1	180	84	expressed protein
OsISM07268	LOC_Os01g52170	30009292	ATCGATGTTGCAAAGGGAGT	CCATCAGCATCGAAGTAGCA	59.8	193	90	expressed protein
OsISM07269	LOC_Os01g52170	30009465	TGCTACTTCGATGCTGATGG	GCAAATCGAAGAGGTGAGGT	59.6	178	98	expressed protein
OsISM07270	LOC_Os01g52170	30009629	CCTCTTCGATTTGCCGATAC	AACATCCCCTTGTGGATCAG	59.7	161	86	expressed protein
OsISM07271	LOC_Os01g52170	30009817	TGTGTTGTTCAGCAGTGTGC	TGGTACTTTCACTCGGATTGC	59.8	357	91	expressed protein
OsISM07272	LOC_Os01g52170	30010197	GCATCCAGCAACAGTCAGAA	TTCTGCTCACCTGTCCATTC	59.4	244	67	expressed protein
OsISM07273	LOC_Os01g52170	30010487	CGTCCTATGGGAAACTACGC	AAGCCTCTCAAGCATTTCCA	59.8	198	91	expressed protein
OsISM07275	LOC_Os01g52400	30102172	CCACGAGCTTCGTCCACC	CAAAATGGGCCTATCGAGAC	61.2	359	77	bacterial transferase hexapeptide domain containing protein expressed
OsISM07276	LOC_Os01g52400	30102603	CGAGCTAGGGGAGAGATGTG	GATAATGGTTTGCCCAGGAA	59.9	543	71	bacterial transferase hexapeptide domain containing protein expressed
OsISM07277	LOC_Os01g52400	30103160	CATTGGACACCACGCTGTAG	CCAATCTGTAGAAAGCATTCATCT	59.5	262	79	bacterial transferase hexapeptide domain containing protein expressed
OsISM07278	LOC_Os01g52400	30103458	TCGCTCTTCTAAATCTTGTGACTG	TATTGTTGCCAATCCTGCAA	60.1	175	96	bacterial transferase hexapeptide domain containing protein expressed
OsISM07279	LOC_Os01g52400	30103614	TGCAGGATTGGCAACAATAA	AACAACAGCCCCTGCAGTAT	59.8	180	94	bacterial transferase hexapeptide domain containing protein expressed
OsISM07280	LOC_Os01g52400	30103781	AGGGGCTGTTGTTGTCCAT	CAACCATCATGTATCTTGGCA	59.9	761	99	bacterial transferase hexapeptide domain containing protein expressed
OsISM07281	LOC_Os01g52400	30104577	GAAGGTCTTAAGCGCAATGG	TAAGCTTTCCTCAACATCCG	58.9	560	57	bacterial transferase hexapeptide domain containing protein expressed
OsISM07282	LOC_Os01g52400	30105175	GCTTTGATGAGAGACTTGCTGA	CATGCGAATAGATTCCACCA	59.6	162	84	bacterial transferase hexapeptide domain containing protein expressed
OsISM07283	LOC_Os01g52410	30116232	GAGGAGGACGCCATACTCAG	CTCTTGCCGCACCGTTTA	60.4	423	99	myb-like DNA-binding domain containing protein putative expressed

ISM IDs	Rice gene locus IDs	Physical positions (bp) on chromosomes	Forward primers (5'-3')	Reverse primers (5'-3')	Melting tempearature (T˚C)	Expected product size (bp) in PCR	Expected product size (bp) in Q-PCR	Putative Functions
OsISM07284	LOC_Os01g52410	30116720	AGGACAACCTCATCCTGTCG	CTTGACGTCGTTGTCCGTC	60.2	203	93	myb-like DNA-binding domain containing protein putative expressed
OsISM07286	LOC_Os01g52430	30124721	CTTTGAAGGCAGCTGAGCA	GGCAAGTATCTGAAATTAGAGG TAACA	60.4	215	98	hAT dimerisation domain-containing protein putative expressed
OsISM07287	LOC_Os01g52430	30124914	CCTCTAATTTCAGATACTTGCC TT	GCTTGTTTTGGTCCAGCTTC	58.6	251	95	hAT dimerisation domain-containing protein putative expressed
OsISM07288	LOC_Os01g52430	30126918	AGGCAGCGAAAGAACAAAAA	CAAAATCCTTGCAGCCAACT	60.1	172	94	hAT dimerisation domain-containing protein putative expressed
OsISM07289	LOC_Os01g52430	30127103	GCTCCTCAGCTTGTGAGAGG	TCATGAAGTAGCCTGTTGCG	60.1	193	74	hAT dimerisation domain-containing protein putative expressed
OsISM07290	LOC_Os01g52450	30130643	ACCTACGTTGACTCCCTCCC	GTTCCTCCAAGGTCAAGTGC	60.0	808	66	hexokinase putative expressed
OsISM07291	LOC_Os01g52450	30131501	TCAAGCAAGACTCAGAAGGG A	GCCACAGCAACAAAATCAAA	59.9	178	83	hexokinase putative expressed
OsISM07292	LOC_Os01g52450	30131774	AAAAATCATTGGCATCTGGC	AATTCAGCCACAACATCCTTG	59.9	283	86	hexokinase putative expressed
OsISM07293	LOC_Os01g52450	30132047	TGGCTGAATTAAACATGGCT	CCCAGCAGCTAATGTCCCTA	59.2	219	90	hexokinase putative expressed
OsISM07294	LOC_Os01g52450	30132335	CATGCAGATGCAATTCCAAA	AAAGTTACCCCATTCCATGTTT	59.6	299	79	hexokinase putative expressed
OsISM07295	LOC_Os01g52450	30132682	GAAAGTTTGAACCCTGGCAA	TTCGAACAAGTTCCCCCATA	60.2	140	71	hexokinase putative expressed
OsISM07296	LOC_Os01g52450	30132834	AGATGGCTCAAGAAACTCGC	TGCATGATCAGCATGTCAAGT	59.9	578	92	hexokinase putative expressed
OsISM07297	LOC_Os01g52450	30133430	TCTCAGAACAGTTGCCAACAA	AAACCAGTTTCCTCGTCGTG	59.8	151	75	hexokinase putative expressed
OsISM07298	LOC_Os01g52480	30155278	ATCTTCATGTGCAGCGAGG	CGGTAGCTTGAGGACGAATC	59.9	152	71	senescence-associated protein putative expressed
OsISM07299	LOC_Os01g52480	30155508	GAGGAGTCAACAAGAGCCTC A	AGCAACGACTGGCTCATCTT	59.8	160	71	senescence-associated protein putative expressed
OsISM07300	LOC_Os01g52480	30155762	ATCCTGGCCTCGCTTGAT	GACAACATTGGATGTGGCAG	60.4	237	88	senescence-associated protein putative expressed
OsISM07302	LOC_Os01g52500	30165577	GACACCTACGGCGAGGACT	CGAGGATCCCTCAGCAAAT	60.2	657	93	NADP-dependent malic enzyme putative expressed
OsISM07303	LOC_Os01g52500	30166295	CTGCCACCAGCAATCGTAT	AGAGGCAGCTGGTATTGTCG	60.1	161	75	NADP-dependent malic enzyme putative expressed

ISM IDs	Rice gene locus IDs	Physical positions (bp) on chromosomes	Forward primers (5'-3')	Reverse primers (5'-3')	Melting tempearature (T˚C)	Expected product size (bp) in PCR	Expected product size (bp) in Q-PCR	Putative Functions
OsISM07304	LOC_Os01g52500	30166436	CGACAATACCAGCTGCCTCT	AAAAGCCTCTCATTCCCCTC	60.0	159	69	NADP-dependent malic enzyme putative expressed
OsISM07305	LOC_Os01g52500	30166690	GCCGTCCTCAGGGTCTTTAT	ACCTGAATGCTCCTCTCTGG	59.9	162	86	NADP-dependent malic enzyme putative expressed
OsISM07306	LOC_Os01g52500	30166875	GAATTTTGGGGCTTGGAGAT	TACAGCAGATGGACGCACTC	60.1	190	99	NADP-dependent malic enzyme putative expressed
OsISM07307	LOC_Os01g52500	30167019	GCTTGCCCTTTACACTGCTC	AGCGCCTCGTTATTTGTACC	59.6	165	85	NADP-dependent malic enzyme putative expressed
OsISM07308	LOC_Os01g52500	30167205	TGGCTTAAGACAAAAGAGGG	TTTTGTTTCACGGCAGTCAT	58.1	163	76	NADP-dependent malic enzyme putative expressed
OsISM07309	LOC_Os01g52500	30167349	TGACTGCCGTGAAACAAAAC	TTGCAAAATCCTCAAACTGC	59.3	214	58	NADP-dependent malic enzyme putative expressed
OsISM07310	LOC_Os01g52500	30167571	GCATTTGAACTCCTTGCAAAA	AAAGGACCACGGAAGCTGTT	60.6	257	80	NADP-dependent malic enzyme putative expressed
OsISM07311	LOC_Os01g52500	30167862	TTCATTGTCGGAGCACAGTT	AAGTTCTGCAATGCCTGTCC	59.8	701	65	NADP-dependent malic enzyme putative expressed
OsISM07312	LOC_Os01g52500	30168543	GGACAGGCATTGCAGAACTT	CCATATTTTCTTGCGGCACT	60.2	180	81	NADP-dependent malic enzyme putative expressed
OsISM07313	LOC_Os01g52500	30168703	AGTGCCGCAAGAAAATATGG	TTGAAGGGTCTCCTTTCGTG	60.2	168	69	NADP-dependent malic enzyme putative expressed
OsISM07314	LOC_Os01g52500	30168892	CATGAGCATGAACCTGTTGG	TTTCCAGATGTGCCAATCAA	60.1	165	78	NADP-dependent malic enzyme putative expressed
OsISM07315	LOC_Os01g52500	30169048	ATCTGGAAAGGGGCAAACTT	TGGACAAGGCAAAAATGACA	60.0	178	84	NADP-dependent malic enzyme putative expressed
OsISM07316	LOC_Os01g52500	30169219	TTGTCCAACCCAACATCTCA	TGGACTTCCACTTGCAAACA	60.1	208	88	NADP-dependent malic enzyme putative expressed
OsISM07317	LOC_Os01g52500	30169430	GATCCGGTGGAGTATGATGG	CCAGACATCACCACACCAAG	60.1	187	93	NADP-dependent malic enzyme putative expressed
OsISM07318	LOC_Os01g52500	30169604	TGGTGATGTCTGGTGCAATC	AAGGCCCTTGTCGAAATTCT	60.3	426	99	NADP-dependent malic enzyme putative expressed
OsISM07319	LOC_Os01g52500	30170057	AAGATCTCTGCCCACATTGC	ACTTAACCAGGTCCTTTGGC	59.2	184	79	NADP-dependent malic enzyme putative expressed
OsISM07320	LOC_Os01g52514	30172456	CTTCTTCAAGGTGCTCGTCG	GGGATGTGCTTGCAGAAGTT	60.7	368	70	B3 DNA binding domain containing protein expressed
OsISM07321	LOC_Os01g52514	30173222	CGTTGCAAATCGTGGACTC	GCGTCTTCCTCGTTCTTGAC	60.1	4043	68	B3 DNA binding domain containing protein expressed

ISM IDs	Rice gene locus IDs	Physical positions (bp) on chromosomes	Forward primers (5'-3')	Reverse primers (5'-3')	Melting tempearature (T˚C)	Expected product size (bp) in PCR	Expected product size (bp) in Q-PCR	Putative Functions
OsISM07322	LOC_Os01g52514	30177542	ATGAGCACCATGCACGTCTA	CACCACGTCCGTCCTCTC	60.3	219	91	B3 DNA binding domain containing protein expressed
OsISM07323	LOC_Os01g52530	30180012	GAACGACGACATCGAGCTG	TGGGCATTCGAGATGAATAA	59.8	855	79	phosphoribosyl transferase putative expressed
OsISM07324	LOC_Os01g52530	30181090	AGAGGCGGTCCTTCAAGTCT	CCACTCTCAAAACATGGCAA	60.0	178	93	phosphoribosyl transferase putative expressed
OsISM07325	LOC_Os01g52530	30181264	GTGGCATTCCCCTACTCAAA	TAGAACCGTTTCCATGCACC	60.4	188	95	phosphoribosyl transferase putative expressed
OsISM07326	LOC_Os01g52530	30181416	AGCTTTTCCTGATGACGGTG	TCCCCTTCTCTAACTTTGTTGC	60.0	189	88	phosphoribosyl transferase putative expressed
OsISM07327	LOC_Os01g52530	30181642	GGCCGCCATGTTGTTATAGT	GCACTAACCTTTGCTGCTCC	59.9	259	99	phosphoribosyl transferase putative expressed
OsISM07328	LOC_Os01g52530	30181916	CATTTTCCCCAACAAGTCGT	AGAAGTGGCTTAGCCCATGA	59.8	1133	75	phosphoribosyl transferase putative expressed
OsISM07329	LOC_Os01g52540	30184497	CTTCATCAAGGTCATGGTCG	CTGTGGTCCACCTCTGGAAT	59.5	973	85	B3 DNA binding domain containing protein expressed
OsISM07330	LOC_Os01g52540	30185786	ACAGAGGCAGCCAACAGTCT	CATGGCCTTGAGTTGTCCTT	60.1	520	65	B3 DNA binding domain containing protein expressed
OsISM07331	LOC_Os01g52540	30186544	ACCGCAAATTCAGAACCTGT	TCTGGAGGGAAGAACTTTCG	59.5	163	88	B3 DNA binding domain containing protein expressed
OsISM07332	LOC_Os01g52540	30187020	GAGCAAGCATGTTTATGCCA	AATCCACCTCACCATGTGCT	60.1	174	89	B3 DNA binding domain containing protein expressed
OsISM07333	LOC_Os01g52560	30215270	CCAGAAGAAGCGCCTCAC	GAAGGTGGTGGAGCTGTCC	60.2	208	98	Plant PDR ABC transporter associated domain containing protein expressed
OsISM07334	LOC_Os01g52560	30215669	CGAGAGGAAAGGTGTTGCTG	GCGATAAGGTTTCTCGCTCT	59.8	300	89	Plant PDR ABC transporter associated domain containing protein expressed
OsISM07335	LOC_Os01g52560	30216151	CATGAAGCGGAATTCATTTG	GGTCCGCAAGAAAACTGTTG	59.9	287	86	Plant PDR ABC transporter associated domain containing protein expressed
OsISM07336	LOC_Os01g52560	30216674	CTACTACACCATGGGGTTTGC	GAACAGTCCTGCAGCCATCT	59.9	195	92	Plant PDR ABC transporter associated domain containing protein expressed
OsISM07337	LOC_Os01g52560	30216936	TCATGTTTGTGCTTGGAGGA	CACCCACCATTTAGGGATTG	60.1	341	57	Plant PDR ABC transporter associated domain containing protein expressed
OsISM07338	LOC_Os01g52560	30217332	AAATGCATTCTCCAAGGTGG	CCACTCCCAATCTCTTTCCA	60.0	146	58	Plant PDR ABC transporter associated domain containing protein expressed
OsISM07339	LOC_Os01g52560	30217537	GCCCTTCTAGGGTTCACCA	TCTTCAGGGAGGATGGATTG	60.0	633	96	Plant PDR ABC transporter associated domain containing protein expressed

ISM IDs	Rice gene locus IDs	Physical positions (bp) on chromosomes	Forward primers (5'-3')	Reverse primers (5'-3')	Melting tempearature (T°C)	Expected product size (bp) in PCR	Expected product size (bp) in Q-PCR	Putative Functions
OsISM07340	LOC_Os01g52560	30218244	CAGAACCTGTATCCCCGAAC	CCACGCAATTGTTGAATCAC	59.7	170	59	Plant PDR ABC transporter associated domain containing protein expressed
OsISM07341	LOC_Os01g52560	30218492	TCTTCCATTTGAACCGCTCT	TCGGCAGTTACTCCCTGACT	59.8	720	91	Plant PDR ABC transporter associated domain containing protein expressed
OsISM07342	LOC_Os01g52560	30219482	CCTGCCTAAGGAGGTCAATG	GGCCCACAATAGCATCTTTC	59.6	261	96	Plant PDR ABC transporter associated domain containing protein expressed
OsISM07343	LOC_Os01g52560	30219929	TTCATCAACCAAGCATTGACA	TTGTACCCAATGGTCCAGAA	59.5	180	94	Plant PDR ABC transporter associated domain containing protein expressed
OsISM07344	LOC_Os01g52560	30220089	TTCTGGACCATTGGGTACAA	TAGCTGGGTTGCGATTCTCT	59.4	275	93	Plant PDR ABC transporter associated domain containing protein expressed
OsISM07345	LOC_Os01g52560	30220402	CGATTGGAAATTGATTTTGCT	CAGGAGGTGGATTGCTCAGT	59.7	286	92	Plant PDR ABC transporter associated domain containing protein expressed
OsISM07346	LOC_Os01g52560	30220848	ATTTTGGAGGGTTGGTCACA	AAACCAACAAACAAGACGGC	60.1	189	88	Plant PDR ABC transporter associated domain containing protein expressed
OsISM07347	LOC_Os01g52560	30221102	CAGCTGGAATGTACTCGGCT	CGAACACATACGGTATCTCCA	59.5	245	67	Plant PDR ABC transporter associated domain containing protein expressed
OsISM07348	LOC_Os01g52560	30221510	CATATTGGGTGCTGCCTTTT	CCAATAGTACCAAACCCACCA	59.8	184	95	Plant PDR ABC transporter associated domain containing protein expressed
OsISM07349	LOC_Os01g52600	30236996	CCAACCTTTGCCGACAACTA	TATCACCACATTTGCCTCCA	60.5	2446	97	hypothetical protein
OsISM07351	LOC_Os01g52610	30241235	CATCGAGATCCCGCTTTATG	AAACTGCAGACATCCCAACC	60.3	168	72	membrane associated DUF588 domain containing protein putative expressed
OsISM07352	LOC_Os01g52610	30241486	GCCTGGCTTCTGTTTACTGG	CTGCTGATCCAGCACTCATC	59.7	8207	62	membrane associated DUF588 domain containing protein putative expressed
OsISM07354	LOC_Os01g52630	30250816	CTACCACAGCCTTGCGCT	TCGCCAAAGCTCAAATTACA	59.8	1315	96	regulator of chromosome condensation putative expressed
OsISM07355	LOC_Os01g52630	30252049	ATTGCAGATGAAGGTTCGCT	GAATTCTCTTCCCCGTAGCC	59.9	177	72	regulator of chromosome condensation putative expressed
OsISM07356	LOC_Os01g52630	30252245	GAGCGTTTTCAAGATTTGGG	CCCCCAGCTTTAACAGAAGA	59.5	211	99	regulator of chromosome condensation putative expressed
OsISM07357	LOC_Os01g52690	30293078	CTACTGGCCAGCACCACTCT	GTTGTAGCCTCCACCACCTC	60.0	204	93	retrotransposon protein putative unclassified expressed
OsISM07359	LOC_Os01g52710	30310846	ATCCCTCTGCCCTTCCAC	AGACTCACCTCCATCGCGT	60.4	202	94	glycosyl transferase 8 domain containing protein putative expressed
OsISM07360	LOC_Os01g52710	30310968	GCTCCAGGATGCTTGGACT	CGACCTCTCCTCCACTCCTT	60.6	1358	99	glycosyl transferase 8 domain containing protein putative expressed

ISM IDs	Rice gene locus IDs	Physical positions (bp) on chromosomes	Forward primers (5'-3')	Reverse primers (5'-3')	Melting tempearature (T°C)	Expected product size (bp) in PCR	Expected product size (bp) in Q-PCR	Putative Functions
OsISM07361	LOC_Os01g52710	30312441	GCCGTGGTGCTCAAGATTAT	CTGGACGACTTAACCTTCCG	59.9	701	60	glycosyl transferase 8 domain containing protein putative expressed
OsISM07362	LOC_Os01g52710	30313628	GCCAAGCACGTTCTCTCTTC	CAAGAAGTATCACCCTCCCG	59.8	189	81	glycosyl transferase 8 domain containing protein putative expressed
OsISM07363	LOC_Os01g52710	30314051	GAACAAATGTCACCGACACG	CCATCTTCCACAGCCTGAAT	60.0	177	73	glycosyl transferase 8 domain containing protein putative expressed
OsISM07364	LOC_Os01g52720	30320029	CTCCGTTAACGCCACCAG	TGGTGGGATTAGGTCTTCTGA	59.9	467	56	Complex I intermediate-associated protein 30 domain containing protein putative expressed
OsISM07365	LOC_Os01g52720	30320533	AGCTGAAAAGGTGGCACTTG	ATGAAGTATCCCCACCAGCA	60.4	1098	97	Complex I intermediate-associated protein 30 domain containing protein putative expressed
OsISM07366	LOC_Os01g52720	30321612	GCTGGTGGGGATACTTCATC	CCACAGAAACCACTACGCCT	59.8	451	99	Complex I intermediate-associated protein 30 domain containing protein putative expressed
OsISM07367	LOC_Os01g52720	30322043	AGGCGTAGTGGTTTCTGTGG	TCCATCCCCTCTGAGTTTCA	60.4	182	97	Complex I intermediate-associated protein 30 domain containing protein putative expressed
OsISM07368	LOC_Os01g52720	30322210	CTCAGAGGGGATGGAAGATG	TCTTCCTGTTGTCCAGGTGA	59.4	161	75	Complex I intermediate-associated protein 30 domain containing protein putative expressed
OsISM07369	LOC_Os01g52720	30322367	AAGATAATTCGTGGCAAGCCT	CATTTCCTCTCCATGTGGGT	59.9	166	97	Complex I intermediate-associated protein 30 domain containing protein putative expressed
OsISM07370	LOC_Os01g52760	30350989	CAGGATGTCTTCCTCATCGG	AATCGGAGGTAGCGAGGG	60.4	640	99	expressed protein
OsISM07371	LOC_Os01g52760	30352093	TCCTCAACGGTCTCCTTGTT	TCCTCCACTTCCCACTTCAC	59.9	175	80	expressed protein
OsISM07372	LOC_Os01g52770	30361124	GTTCACCAACGGCCTCAC	ATGAAGTTGTCGAAGCCCAG	60.2	608	61	GDSL-like lipase/acylhydrolase putative expressed
OsISM07373	LOC_Os01g52770	30361766	CTCAACGGCGCCAACTTC	TAGTTCTGCACCTGCCCG	62.0	191	99	GDSL-like lipase/acylhydrolase putative expressed
OsISM07374	LOC_Os01g52864	30398349	GTCCTCGTCGCCTCCTACTT	CCTTCCTCCTCTTTTCTCCG	60.5	1207	97	transmembrane protein putative expressed
OsISM07375	LOC_Os01g52864	30399710	AAATAGGGAAGGCCTACGGA	ATCGCACCTTCTGTACCTCG	60.1	214	80	transmembrane protein putative expressed
OsISM07376	LOC_Os01g52864	30399941	TAGCTGGAATTCTTGCATCG	CACATACTGCGCCTTGTGAT	59.4	269	82	transmembrane protein putative expressed
OsISM07377	LOC_Os01g52864	30400249	TTGGTGCCACACTACAAGATAAA	CAGAACAGCCAATATACCACCA	59.7	3942	96	transmembrane protein putative expressed
OsISM07378	LOC_Os01g52920	30427007	GGCTGCGAGATTGGTACTTT	TCTCAATGTCGTGGTTCCTG	59.5	676	92	expressed protein

ISM IDs	Rice gene locus IDs	Physical positions (bp) on chromosomes	Forward primers (5'-3')	Reverse primers (5'-3')	Melting tempearature (T˚C)	Expected product size (bp) in PCR	Expected product size (bp) in Q-PCR	Putative Functions
OsISM07379	LOC_Os01g52920	30427837	GCAACATCTGCTTTGGTATGG	CCATTTCCGATGTTTTGACC	60.3	223	83	expressed protein
OsISM07380	LOC_Os01g52920	30428073	TGGTTTGCCTCAATCCAGTA	CCACTTGCTGTAATCCCCTC	59.3	466	95	expressed protein
OsISM07381	LOC_Os01g52920	30428573	CATGGGAAATACCCTGGAGA	CTTGTCAGGCCATTGAAACA	59.7	602	91	expressed protein
OsISM07382	LOC_Os01g52920	30429195	TCAAGCCTTAGCATGGAAAA	CACGTACCCCAGGATCATTT	59.1	501	96	expressed protein
OsISM07383	LOC_Os01g52920	30429749	TGCTCAAGACATCCATCAGC	TCCAAGGTAACGACCACTCC	60.0	171	93	expressed protein
OsISM07384	LOC_Os01g52930	30433741	GGTCAAATGGGACTGGGTC	GGTCCTCGGCCTCAGACT	60.3	663	91	retrotransposon protein putative unclassified
OsISM07386	LOC_Os01g52960	30445497	ACTACGTCGATTCGGTGGAC	AAGGCCGCTGTAGTGGTAGA	59.9	230	99	subtilisin N-terminal Region family protein expressed
OsISM07387	LOC_Os01g52960	30445748	CCAGAACAAGCTGAGAGGCT	CTTGAAGATTGCAATGCCCT	60.0	146	49	subtilisin N-terminal Region family protein expressed
OsISM07388	LOC_Os01g53000	30461116	CTGCCGAACATGGCTACTTT	TTCAATTGCCATTGCTCATC	59.9	925	53	trehalose synthase putative expressed
OsISM07389	LOC_Os01g53000	30462255	GGCCAACAGATTGTGGAAGT	TCTGCCTGTCTCTTGTTCTCTG	59.9	503	96	trehalose synthase putative expressed
OsISM07390	LOC_Os01g53020	30471874	GCGTCGCCGAAGACTACTAC	TTGATCTGCTGAGGTGTTGC	60.0	254	62	heat shock protein DnaJ putative expressed
OsISM07391	LOC_Os01g53020	30472159	CATCCTGATCTCAGTGGCAA	GCTGGATGGGATCGGTAAG	60.1	192	89	heat shock protein DnaJ putative expressed
OsISM07392	LOC_Os01g53020	30472422	AGGGATCATGTGTTTGTCGAT	CCGACCAAAATCTTCCTCAA	59.7	199	97	heat shock protein DnaJ putative expressed
OsISM07393	LOC_Os01g53020	30472615	GGTCGGGCAAGAGTTTACAA	CAGCTGATGTCCAATGGATG	60.1	226	95	heat shock protein DnaJ putative expressed
OsISM07394	LOC_Os01g53020	30472853	TCCTTGAAGATGAAATGCGA	TCAACTGAACTTCCCATCCC	59.6	217	74	heat shock protein DnaJ putative expressed
OsISM07395	LOC_Os01g53020	30473020	CCATTTGAGCAGGTTGGTTT	CCTGTCTTTTCTCCCATCGT	59.6	357	89	heat shock protein DnaJ putative expressed
OsISM07396	LOC_Os01g53020	30473339	TGTCTGCCAGGCAAGTACAC	TGGCTCACACGTCTTCTGAC	60.0	221	73	heat shock protein DnaJ putative expressed
OsISM07397	LOC_Os01g53020	30473565	ATTCTGGTAAGGGCAGCTCA	TTTGCTCTCTCCCTTGCTTC	59.8	204	86	heat shock protein DnaJ putative expressed

ISM IDs	Rice gene locus IDs	Physical positions (bp) on chromosomes	Forward primers (5'-3')	Reverse primers (5'-3')	Melting tempearature (T°C)	Expected product size (bp) in PCR	Expected product size (bp) in Q-PCR	Putative Functions
OsISM07398	LOC_Os01g53060	30487439	CTTACGGGTGACACCATCG	ATCAACAAGTCCGGTACCAA	58.9	583	99	peroxisomal membrane protein putative expressed
OsISM07399	LOC_Os01g53060	30488117	GTATGCCGAAGCCAACATTT	TCCAGGCAAAAATTACACCA	59.5	186	94	peroxisomal membrane protein putative expressed
OsISM07400	LOC_Os01g53060	30488348	GGAATGATGCCCTTCCTACA	TGATCGATACAGGAATCCAAAA	59.7	151	58	peroxisomal membrane protein putative expressed
OsISM07401	LOC_Os01g53060	30488451	TTTGTTGTGAAATTGCAGGG	CAACACGAGCAGACAAAGGA	59.8	296	82	peroxisomal membrane protein putative expressed
OsISM07402	LOC_Os01g53100	30515982	GACTCCGTCGTCTTCCTCAG	GGATCTTGCCTTCTCCTCGT	60.4	226	68	ubiquitin family protein putative expressed
OsISM07403	LOC_Os01g53140	30532946	GTCCCCATCCTCGTCTACAA	CAAGAACTTCCTCGGTGGTC	59.8	2481	82	expressed protein
OsISM07404	LOC_Os01g53150	30536660	CGCGCTCACTTCCAAGAA	AACCAGGCAAATCAACAAGG	60.6	278	87	GTPase of unknown function domain containing protein putative expressed
OsISM07405	LOC_Os01g53150	30536918	CCTTGTTGATTTGCCTGGTT	AATTCGTGCGAGCTTCTTGT	60.0	313	57	GTPase of unknown function domain containing protein putative expressed
OsISM07406	LOC_Os01g53150	30537332	TATGCTAGCTGGCTTGGTCA	CCGTTTTTCTTCTTCTTGCG	59.8	2481	81	GTPase of unknown function domain containing protein putative expressed
OsISM07407	LOC_Os01g53180	30553301	ATGGACAAGCTCAAAAGGCA	ATTGTCGTCTCGGTCATCCT	60.2	176	87	retrotransposon protein putative Ty1-copia subclass
OsISM07408	LOC_Os01g53190	30554378	CGCTGTCAAATCACATGTCC	AAGCTATTGGCATTCTGCAT	58.8	351	78	hypothetical protein
OsISM07409	LOC_Os01g53190	30554755	CAAGAACGATGTTCTAAGTTTGATG	AAATTGCCCATGGCACAG	59.9	440	63	hypothetical protein
OsISM07410	LOC_Os01g53190	30555176	ACTGTGCCATGGGCAATTT	ATTAGGCAGCCTCTTCATGC	60.4	1225	96	hypothetical protein
OsISM07411	LOC_Os01g53190	30556436	AACCATCGGCCACATCTTAG	TAGATAGTTGGCACCAGGGC	60.0	249	87	hypothetical protein
OsISM07412	LOC_Os01g53200	30561148	CTGCAACCACAATGCAAATC	GTGGACTGACCCAGCATCTC	60.4	1096	79	flavin monooxygenase putative expressed
OsISM07413	LOC_Os01g53200	30562418	CGGCACATTTGCAAAGATTA	CTCCAACCTTGTGTCCATGA	59.6	232	98	flavin monooxygenase putative expressed
OsISM07414	LOC_Os01g53200	30562656	GATGTCATTGTGCTTGCCAC	CTTGGCAACCCATCCTTTT	60.0	267	90	flavin monooxygenase putative expressed
OsISM07415	LOC_Os01g53220	30581780	CCTCCCCTCCTACTTCAAGC	CTGTCCGGATCCACCTTG	60.1	196	85	HSF-type DNA-binding domain containing protein expressed

ISM IDs	Rice gene locus IDs	Physical positions (bp) on chromosomes	Forward primers (5'-3')	Reverse primers (5'-3')	Melting tempearature (T˚C)	Expected product size (bp) in PCR	Expected product size (bp) in Q-PCR	Putative Functions
OsISM07416	LOC_Os01g53240	30588884	TCTCCATGGATAGGCTCCTC	TGATTTCCAGTATTGCTCCG	59.0	667	96	BURP domain containing protein expressed
OsISM07417	LOC_Os01g53240	30589528	CTCCGGAGCAATACTGGAAA	ACGTTCACCGACGTGCCT	61.5	569	98	BURP domain containing protein expressed
OsISM07418	LOC_Os01g53260	30603835	TACCGATGGAGGAAGTACGG	CTTGCTTCTTCACGTTGCAC	59.8	2728	98	OsWRKY23 - Superfamily of TFs having WRKY and zinc finger domains expressed
OsISM07419	LOC_Os01g53280	30608721	CAATGCTGGTACACGATTGC	GGTTGAGCTGGATGTTCTCC	59.9	260	78	50S ribosomal protein L20 putative expressed
OsISM07420	LOC_Os01g53294	30621906	CCGTCTCCAAATCTTCTTCG	GGCGTGGATTATGTTTTGCT	59.9	214	94	respiratory burst oxidase protein B putative expressed
OsISM07421	LOC_Os01g53294	30622033	CTGCATTTTTACCAGGGTGG	CGACAGTTTATTGGCAGACG	59.8	1262	98	respiratory burst oxidase protein B putative expressed
OsISM07422	LOC_Os01g53294	30623312	AGAAGAGTACGCCGCTTTGA	TCTGCAACAGAAGTGTCTCCA	59.9	6265	90	respiratory burst oxidase protein B putative expressed
OsISM07423	LOC_Os01g53294	30629929	ACGGGCACTACCTTTTGATG	GTTCCCTGCATGGAGGATTA	59.9	340	86	respiratory burst oxidase protein B putative expressed
OsISM07424	LOC_Os01g53294	30630537	TTGTGTATATCGCGCTCATTG	CAAGCAAACAGGCACTGAAA	59.9	185	99	respiratory burst oxidase protein B putative expressed
OsISM07425	LOC_Os01g53294	30630742	AAGGTTCTTCAGGTCTGGCA	GAGGCTTGGACATTTGCAGT	60.1	166	90	respiratory burst oxidase protein B putative expressed
OsISM07426	LOC_Os01g53294	30630947	AATGTCCAGCAGTGTCTCCC	ATCCCCAGGTGCTGAAGTAA	59.8	216	60	respiratory burst oxidase protein B putative expressed
OsISM07427	LOC_Os01g53294	30631255	TAAAAGCGGCCTTCTTAGGG	ACGGTCCATCAATCAGAAGC	60.4	157	69	respiratory burst oxidase protein B putative expressed
OsISM07428	LOC_Os01g53294	30631476	TGCGACACCCTTTATTAGCA	CCCATTGGTGGATAAAGATCA	59.5	253	97	respiratory burst oxidase protein B putative expressed
OsISM07429	LOC_Os01g53294	30631870	TGAAATTGCTGACTTGGATCA	CATGGTGATGAGTGCTGACC	59.7	207	98	respiratory burst oxidase protein B putative expressed
OsISM07430	LOC_Os01g53294	30632068	ATCACCATGCTCCAAGCTCT	TTTGGTCGTGCAAAATGTGT	59.9	251	87	respiratory burst oxidase protein B putative expressed
OsISM07431	LOC_Os01g53294	30632339	AAATTTCCTCCAAGCATCCA	TGCTTAGTTCTTGTGCCAGG	59.3	414	76	respiratory burst oxidase protein B putative expressed
OsISM07432	LOC_Os01g53310	30633694	CCCGTTTATCTTACCAGCCA	TTTCATGCAGCACCATTCAT	60.0	912	98	hypothetical protein
OsISM07433	LOC_Os01g53310	30634521	CCACTGTAACTAGACTGTAAG TGGTGA	CAACTGTCCTTGTTACTTGAGCC	60.1	1328	75	hypothetical protein

ISM IDs	Rice gene locus IDs	Physical positions (bp) on chromosomes	Forward primers (5'-3')	Reverse primers (5'-3')	Melting tempearature (T°C)	Expected product size (bp) in PCR	Expected product size (bp) in Q-PCR	Putative Functions
OsISM07434	LOC_Os01g53310	30635809	GGGGTTGATAGAACAAAGGC	GCCCCACACCCATATACTTT	58.6	292	64	hypothetical protein
OsISM07435	LOC_Os01g53320	30640214	TTGTGGATGAAATGGAGAAGG	CGCTCCCTGAATAACCTCAT	59.5	182	99	transposon protein putative unclassified
OsISM07436	LOC_Os01g53450	30711328	TAAACTCGTCAAACGCCACA	GACGGCGGACAAAGGAAT	60.7	386	87	aminotransferase classes I and II domain containing protein expressed
OsISM07438	LOC_Os01g53450	30711990	GGACCGACGCAGTACACC	CTCGTGCAGGACAGAGAGG	59.9	182	73	aminotransferase classes I and II domain containing protein expressed
OsISM07439	LOC_Os01g53450	30712365	GCGCAGAATATGTCGAGGAC	ATCACCTTCCACCACCTCC	60.3	161	72	aminotransferase classes I and II domain containing protein expressed
OsISM07440	LOC_Os01g53450	30712457	CATGAGCAAGAGGGGGTTC	CGCCAAGCTCTTCCTCAC	60.1	184	81	aminotransferase classes I and II domain containing protein expressed
OsISM07441	LOC_Os01g53450	30712682	GTATAGCACGACGTTGCGG	TCACAAGGCAAGTGACCAAC	60.0	128	46	aminotransferase classes I and II domain containing protein expressed
OsISM07442	LOC_Os01g53450	30712864	CTTCCAGCTACGAAGGCG	CAAACCAAACTTTGCCATGA	59.6	675	78	aminotransferase classes I and II domain containing protein expressed
OsISM07443	LOC_Os01g53450	30713555	ACTGGCCAAGAAGAGGAGGT	CACATATCAAGGCAGAGCCC	60.4	282	74	aminotransferase classes I and II domain containing protein expressed
OsISM07444	LOC_Os01g53450	30713922	GAACACCTGTGTTTGCAGAAA T	CACACTTCCCAGAGCAGTCA	59.6	147	68	aminotransferase classes I and II domain containing protein expressed
OsISM07445	LOC_Os01g53450	30714081	TCTGTTGGGAGAAAACCTGC	CAACAAAAATTAGCTTCAGAGTT CA	59.7	378	79	aminotransferase classes I and II domain containing protein expressed
OsISM07446	LOC_Os01g53480	30720271	CTCCCAGCTCGCTTGACC	TGTTTCTCAGAAGACCGGTGA	61.5	142	97	retrotransposon protein putative Ty3-gypsy subclass
OsISM07447	LOC_Os01g53480	30720385	ACAGCTGTCACCGGTCTTCT	TCGTTGGTGATGAAGTCGAG	59.9	163	78	retrotransposon protein putative Ty3-gypsy subclass
OsISM07448	LOC_Os01g53480	30721502	CTATCACCAACCAAGCCTCAG	TGATAGTTTCGCGAAGATCG	59.4	167	83	retrotransposon protein putative Ty3-gypsy subclass
OsISM07449	LOC_Os01g53520	30737929	CAAGATCTACCGCCTCTTCG	TGTTCCTCCATAGCACGAGA	59.7	486	87	reticulon domain containing protein putative expressed
OsISM07450	LOC_Os01g53520	30738522	CTGGCTGTCCTCTTCCTCTG	TCACCTCAGGAATGTTTGGA	59.6	196	71	reticulon domain containing protein putative expressed
OsISM07451	LOC_Os01g53520	30738805	ATGGCCGTGATCTGAAGAAG	ACAGAATCCAGAGTCCAGCG	60.3	146	52	reticulon domain containing protein putative expressed
OsISM07452	LOC_Os01g53520	30738926	GTGATCGCTGGACTCTGGAT	GCACAGTGTAGAGCGCCA	60.0	179	92	reticulon domain containing protein putative expressed

ISM IDs	Rice gene locus IDs	Physical positions (bp) on chromosomes	Forward primers (5'-3')	Reverse primers (5'-3')	Melting tempearature (T°C)	Expected product size (bp) in PCR	Expected product size (bp) in Q-PCR	Putative Functions
OsISM07454	LOC_Os01g53560	30752678	GTCGAGAAGGTCAAGGGGTT	GCCGTTTCAACAGGTAAGGA	60.3	168	96	phosphoesterase putative expressed
OsISM07455	LOC_Os01g53560	30752793	GAGGAGGTGCGTGAGCTAAA	CCTGGGCTGGCTTTTACTTT	60.6	533	96	phosphoesterase putative expressed
OsISM07456	LOC_Os01g53560	30753495	TCAACTTACCCAGCATTCCC	TTGATCAGTCACATTGCCATC	59.7	718	85	phosphoesterase putative expressed
OsISM07457	LOC_Os01g53560	30754187	AGAGAGATGGCAATGTGACTGA	GCCCTTAGAGTCTTCCATGC	59.4	181	81	phosphoesterase putative expressed
OsISM07458	LOC_Os01g53560	30754364	GGGCACTGGAAATAATATCGAA	TGAAAATGCAAGTTGGATGC	59.9	232	90	phosphoesterase putative expressed
OsISM07459	LOC_Os01g53560	30754480	TGTTTCCCTGAAAGTTTTGCT	GACCAGCTCTTTTGTCCCCT	59.7	800	99	phosphoesterase putative expressed
OsISM07460	LOC_Os01g53560	30755278	TCCAGTGATACCCTTTTCGG	ATTGAAAACCTTCCAATGCG	59.9	187	80	phosphoesterase putative expressed
OsISM07461	LOC_Os01g53560	30755412	GTCATGTGTGGATGCAGGAT	TTAGCTGAAGACTGAAGGCCA	59.7	472	98	phosphoesterase putative expressed
OsISM07462	LOC_Os01g53560	30755861	GATGGCCTTCAGTCTTCAGC	AAGACCACTTGTCTGCACCA	59.6	873	95	phosphoesterase putative expressed
OsISM07463	LOC_Os01g53560	30756750	TCCAGAAAAGCTGCAAATCC	TCCATCGTTCAATGTTCGAC	59.9	570	78	phosphoesterase putative expressed
OsISM07464	LOC_Os01g53560	30757355	GGGCGTCTTGACAAGTTCAT	CCTTTCCCTAAACCACATGC	59.8	455	72	phosphoesterase putative expressed
OsISM07465	LOC_Os01g53560	30757869	GAGAGCATTTCAATTCGGGA	GATCATTCTCTCTCCCAGCG	60.0	944	79	phosphoesterase putative expressed
OsISM07466	LOC_Os01g53560	30758775	TTTCTATGTTGGTGATGCCG	GGAACGTGAAACTTCAGACCA	59.8	1100	91	phosphoesterase putative expressed
OsISM07467	LOC_Os01g53570	30763443	GTCGGCATCATGTTCGAGTA	AACCCTCGGTTGAAACTGG	59.8	162	48	aluminum-activated malate transporter putative expressed
OsISM07468	LOC_Os01g53570	30763695	CGTGATTGGCCTAAGCATCT	GCTTCACGAACGACGTCAC	60.4	297	51	aluminum-activated malate transporter putative expressed
OsISM07470	LOC_Os01g53590	30769520	AGGAGGCTGGACAGGAGTG	ACGATCGAGGTCGACAAATC	60.2	1830	87	3-5 exonuclease domain-containing protein putative
OsISM07471	LOC_Os01g53610	30778531	CCTCCTAGCTGCCCGTTACT	GCCACAGACAACCTCTGTCA	60.3	582	99	selenium-binding protein related putative expressed
OsISM07472	LOC_Os01g53620	30785277	CTTCTCTCTGCCTCGCTCC	CTGTGCAGCGGCATCAAC	61.5	179	94	expressed protein

ISM IDs	Rice gene locus IDs	Physical positions (bp) on chromosomes	Forward primers (5'-3')	Reverse primers (5'-3')	Melting tempearature (T˚C)	Expected product size (bp) in PCR	Expected product size (bp) in Q-PCR	Putative Functions
OsISM07473	LOC_Os01g53620	30785470	CTTTGCTCACCTCCATGCTC	GGTGACCAAAACACCACTCC	60.6	1284	97	expressed protein
OsISM07474	LOC_Os01g53640	30819248	ACCGCCAAACTACTTTCCCT	ACCGGATGATATCCAGGTGA	60.1	178	79	retrotransposon protein putative unclassified expressed
OsISM07475	LOC_Os01g53640	30819742	CAGCGGATGCTTGTTTATGA	TTAACCCTTGTTCGCCAATC	59.9	209	96	retrotransposon protein putative unclassified expressed
OsISM07476	LOC_Os01g53640	30819968	CCCGTGGAATTGCTTATCTG	AGCTTCGAAGTTGTCATCCAA	60.2	181	96	retrotransposon protein putative unclassified expressed
OsISM07477	LOC_Os01g53640	30820078	CAGGTCATCCTCGGATTATACA	AGCCTTGCAAGTCCAAAATC	59.1	199	98	retrotransposon protein putative unclassified expressed
OsISM07478	LOC_Os01g53640	30820271	AAGGCTAGCAGCTGACTCCA	TTGCCAGACAACGCATACTC	60.1	201	88	retrotransposon protein putative unclassified expressed
OsISM07479	LOC_Os01g53640	30820561	TGGGAGATGAGAGCCTTGTT	TCAGGGAGGTCACCAAATTC	59.9	1071	80	retrotransposon protein putative unclassified expressed
OsISM07480	LOC_Os01g53640	30821691	CTGCATGCATTCGTCATTCT	TTGAAGGCCATTGTTCAAGTT	59.7	180	99	retrotransposon protein putative unclassified expressed
OsISM07481	LOC_Os01g53660	30828600	GCAGCAGAGCATGTATCCAA	TGTCGCAACTTCAACTGCTC	60.1	203	99	transposon protein putative unclassified expressed
OsISM07482	LOC_Os01g53660	30830046	CAGTATCAAGCCAAGGCTCC	TGGCCTTCTTTACCATACCG	59.9	237	68	transposon protein putative unclassified expressed
OsISM07483	LOC_Os01g53670	30831940	TTGGTCCGATCTCGTCTCTC	CCTCCCTCCTTCTGAAAGTACA	60.0	172	99	ENTH domain containing protein expressed
OsISM07484	LOC_Os01g53670	30832117	CTTAAGGTGCTCAAGGTGCC	CGATATCTGCCAAATCGGAA	60.4	222	95	ENTH domain containing protein expressed
OsISM07485	LOC_Os01g53670	30832316	TGGTTCCGATTTGGCAGATA	AGGCGTTGCCATAACACATT	60.9	228	82	ENTH domain containing protein expressed
OsISM07486	LOC_Os01g53670	30832524	AATGTGTTATGGCAACGCCT	AAGGTACTCGATCACAGCCAA	60.1	160	79	ENTH domain containing protein expressed
OsISM07487	LOC_Os01g53670	30832706	CAGCTGATGGCATTGTTGAC	TTGAGCCCAACATCTTTTCC	60.2	176	89	ENTH domain containing protein expressed
OsISM07488	LOC_Os01g53670	30832915	TTTGGATGACAGGGAGAAGC	TCGATTTGTGCGTTATTCCA	60.1	186	99	ENTH domain containing protein expressed
OsISM07489	LOC_Os01g53670	30833301	AAAAAGAGTGAAAGGCATGGT	GGCTTCAGATGTGGTGCTAA	58.4	238	88	ENTH domain containing protein expressed
OsISM07490	LOC_Os01g53670	30833578	TTCAACCCACGAGGATTTTC	TTGGACCAAAGAGATCCAGG	60.0	237	74	ENTH domain containing protein expressed

ISM IDs	Rice gene locus IDs	Physical positions (bp) on chromosomes	Forward primers (5'-3')	Reverse primers (5'-3')	Melting tempearature (T˚C)	Expected product size (bp) in PCR	Expected product size (bp) in Q-PCR	Putative Functions
OsISM07491	LOC_Os01g53670	30833908	CAGATGCAGCTTTCCAATCA	GACAGTCTGCCAGCAAACAA	60.0	366	92	ENTH domain containing protein expressed
OsISM07492	LOC_Os01g53670	30834798	AATCTGGCATATGGGCTGAC	ACAATCCCAACATCGGAGAG	59.9	408	95	ENTH domain containing protein expressed
OsISM07493	LOC_Os01g53690	30845944	CGTTTTCCCAACTCGAACTC	CCAAGGATGCACCTCAAAAC	60.1	303	59	hypothetical protein
OsISM07494	LOC_Os01g53710	30867809	ACGCTGCTCTACAACGTGC	TGGGAAAGGAACAGCACCTA	60.4	621	97	dual specificity protein phosphatase putative expressed
OsISM07495	LOC_Os01g53710	30868497	CCCTGGTCCCAACATCTTTA	TGACAAATATCCTGGAGGGC	59.8	379	98	dual specificity protein phosphatase putative expressed
OsISM07496	LOC_Os01g53710	30868856	GCCCTCCAGGATATTTGTCA	TACGCCCTCTTCCAGCTTTA	59.9	718	95	dual specificity protein phosphatase putative expressed
OsISM07497	LOC_Os01g53710	30869563	AAGAGGGCGTAGCACGACTA	TCTAGGCCTAATGGATCGGA	59.8	182	98	dual specificity protein phosphatase putative expressed
OsISM07498	LOC_Os01g53710	30869744	AGTACTTTTGGCACCATCGC	TGGCCCAGGTTTTTACTCTG	60.1	195	91	dual specificity protein phosphatase putative expressed
OsISM07499	LOC_Os01g53720	30878405	GATGACACGCCCGAAGAC	TAGTGCAATTTTCATCGCCA	60.2	735	82	expressed protein
OsISM07500	LOC_Os01g53720	30879289	TGAAATGCTCTCAATGTCACA A	GCCTTGCAGCTGTCTCAACT	60.0	178	93	expressed protein
OsISM07501	LOC_Os01g53720	30879469	GCTGAGGAGCGAGGTCATAC	TTGGTGATCAAAACCCAGAAG	60.0	273	85	expressed protein
OsISM07502	LOC_Os01g53720	30879829	AGTGTTCTTGATTCGGGCAA	GCTTCCAGCTCTTTTTAGACCA	60.3	192	76	expressed protein
OsISM07503	LOC_Os01g53720	30880014	TGGAAGCCTAAGATTTTATAG ACCA	TGCATTCCTTGCTGTAACTTG	59.3	180	98	expressed protein
OsISM07504	LOC_Os01g53720	30880200	CTGAGGGAAGCAAGGTCAAG	TGAGAGCATCAATGACAGCC	60.0	436	77	expressed protein
OsISM07505	LOC_Os01g53720	30880670	CTCAGAATCATCTCCTGGCA	TCCTTGCAAACCATAAACCA	59.0	1073	56	expressed protein
OsISM07506	LOC_Os01g53720	30881723	TGGTTTATGGTTTGCAAGGA	TGGTACCTTCTGCTCCAAAGA	59.4	517	96	expressed protein
OsISM07507	LOC_Os01g53720	30882284	TCCTTTGGTTGATTCTCTCCA	GCTGTTCCTAAAAGCTGATCAAT	59.3	164	82	expressed protein
OsISM07508	LOC_Os01g53720	30882389	TTGTGACTCTTGTTGCAGGC	TCCCTCAGGAGTTTCAAGGA	59.9	960	97	expressed protein

ISM IDs	Rice gene locus IDs	Physical positions (bp) on chromosomes	Forward primers (5'-3')	Reverse primers (5'-3')	Melting tempearature (T˚C)	Expected product size (bp) in PCR	Expected product size (bp) in Q-PCR	Putative Functions
OsISM07509	LOC_Os01g53740	30888861	ACTGTAGTGGATGGGCGAAC	TAGCGAACCCTGTACGCAAC	60.6	751	95	hypothetical protein
OsISM07510	LOC_Os01g53750	30896944	TTTGTTGCGCTCCTCTTCTT	GTAGCAGATGCCAACCTTGC	60.5	413	64	glucan endo-1 3-beta-glucosidase precursor putative expressed
OsISM07511	LOC_Os01g53750	30898547	TGTAGCTTGTGACTTTGGCG	TACCCGGCAGCCATATAAAC	59.9	960	82	glucan endo-1 3-beta-glucosidase precursor putative expressed
OsISM07512	LOC_Os01g53790	30917723	GTGTTCACCTGCCGGTTC	GAGTCATGGACGACTCCGAC	60.4	1668	74	stress-induced protein putative expressed
OsISM07513	LOC_Os01g53860	30961016	ATTAGTCGGAGTCACCCACC	TAGAGCACACATGGAAGGGC	60.0	154	84	hypothetical protein
OsISM07514	LOC_Os01g53860	30961330	TCTATCTCCTTTGGCCGGTA	CTTCCCTATTCGGCTTCTCC	59.9	1305	80	hypothetical protein
OsISM07515	LOC_Os01g53860	30962615	GGAGAAGCCGAATAGGGAAG	ACTGTCCTCCTCACCTTGGA	59.9	1120	97	hypothetical protein
OsISM07516	LOC_Os01g53870	30966802	CAAGTGCTAGCAACAAACGG	CCATCAGCATTAAACCGGAG	60.0	232	56	transposon protein putative unclassified
OsISM07517	LOC_Os01g53880	30975871	GGAGCAAAGAGGGGATTCAT	CTGCAAGTTCCTTCCCACAT	60.3	227	98	OsIAA6 - Auxin-responsive Aux/IAA gene family member expressed
OsISM07518	LOC_Os01g53880	30976164	ATGGTGCTCCTGCTACACCT	CTGAAGGATCGAATTGGAGG	59.7	955	95	OsIAA6 - Auxin-responsive Aux/IAA gene family member expressed
OsISM07519	LOC_Os01g53880	30977289	GGCTATCGTCAGCTGTCAAAC	TGTCATCTGCACCTTGCTGT	60.2	177	94	OsIAA6 - Auxin-responsive Aux/IAA gene family member expressed
OsISM07520	LOC_Os01g53880	30977527	AGGAGACAGGATGCTGGTTG	CAGGGTATGGGAAAGCTCTG	60.0	254	98	OsIAA6 - Auxin-responsive Aux/IAA gene family member expressed
OsISM07521	LOC_Os01g53900	30992950	GTCTGTTGTCGCTCATGTGG	TGGGCTATAATTCCAGCAGC	60.3	625	76	elongation factor putative expressed
OsISM07522	LOC_Os01g53920	31007761	CAGGCTCGTTCGGCTACAT	ACCCCAAAGCTGTACACGTC	60.7	212	80	receptor-like protein kinase 5 precursor putative expressed
OsISM07523	LOC_Os01g53950	31018355	GCCGTCCTCTCCTCTTTTCT	GTCGTTTGTGTACCCTGGCT	60.0	207	83	retrotransposon protein putative Ty3-gypsy subclass
OsISM07524	LOC_Os01g53980	31046709	GTGGAAAATGCCATAGCTCG	AGAGACCTGAGAGCGCGA	60.3	737	99	IQ calmodulin-binding motif family protein expressed
OsISM07525	LOC_Os01g53980	31047561	GCTTCCTACCGGAAATCCAT	GTCTTCAACCGGGTCTCCTC	60.7	168	60	IQ calmodulin-binding motif family protein expressed
OsISM07526	LOC_Os01g54000	31061962	TGCGAAATCCCAGTTTTACC	CAGTCCATCAGGACTTTTGGT	59.5	162	76	hypothetical protein

ISM IDs	Rice gene locus IDs	Physical positions (bp) on chromosomes	Forward primers (5'-3')	Reverse primers (5'-3')	Melting tempearature (T˚C)	Expected product size (bp) in PCR	Expected product size (bp) in Q-PCR	Putative Functions
OsISM07527	LOC_Os01g54010	31065185	GCGGTGCTCAATCTCACCTA	CAGGTTCTTCTTTCCCTTCG	60.1	186	86	peptidase putative expressed
OsISM07528	LOC_Os01g54010	31065351	CGAAGGGAAAGAAGAACCTG	AAGGCTGCTGTTCCACTGTT	59.4	611	97	peptidase putative expressed
OsISM07529	LOC_Os01g54010	31065942	AACAGTGGAACAGCAGCCTT	GCCTCCAACCCTTCTTTTGT	60.2	138	68	peptidase putative expressed
OsISM07530	LOC_Os01g54010	31066102	TAACTGGGATGCCGAAGAGT	AGCCCTGTTCTCTTCAACCC	60.2	148	62	peptidase putative expressed
OsISM07531	LOC_Os01g54010	31066338	ATGAGTTGCTTAAGGAGGCG	GAAGAACTATCAGAAGCCATCCA	59.6	325	92	peptidase putative expressed
OsISM07532	LOC_Os01g54010	31066633	TGACTTATGGATGGCTTCTGA	CCAATGTGTTGAACAAAGGC	58.7	218	94	peptidase putative expressed
OsISM07533	LOC_Os01g54010	31066831	GCCTTTGTTCAACACATTGG	TCCATCCACACAAAGTCATCA	59.5	507	96	peptidase putative expressed
OsISM07534	LOC_Os01g54010	31067326	TGTGTGGATGGAGAAGTTCG	ATCTCCTCATCGGAAAGCCT	59.9	554	97	peptidase putative expressed
OsISM07535	LOC_Os01g54010	31067867	CCGATGAGGAGATCCTACCC	AGGCACTCCCAGCAATCTCT	61.1	213	93	peptidase putative expressed
OsISM07536	LOC_Os01g54010	31068122	AGGGCAGCTCTACAAATGGA	TCAGGGGATTGTTTCTCCAG	59.9	192	80	peptidase putative expressed
OsISM07537	LOC_Os01g54010	31068372	AGGACTATCTGGGAGGCCAT	TCATGCAGTGATGGTGCATA	59.8	172	55	peptidase putative expressed
OsISM07538	LOC_Os01g54030	31075730	CGAGGACCAGCCCATCAC	TTCTCCGTGAATGATAGCCC	61.4	486	97	NADP-dependent malic enzyme putative expressed
OsISM07539	LOC_Os01g54030	31076255	AGTCGTGCTCTCCCAAGAAC	GCAGAGGAACCTGAAACTGC	59.7	158	69	NADP-dependent malic enzyme putative expressed
OsISM07540	LOC_Os01g54030	31076393	GCAGTTTCAGGTTCCTCTGC	ACGTTGTCGATCAGGAGCTT	59.9	212	91	NADP-dependent malic enzyme putative expressed
OsISM07541	LOC_Os01g54030	31076652	GCCAGAAGTACGGGTCAATC	TCTCTGGCCAGTTCCTCAGT	59.8	255	97	NADP-dependent malic enzyme putative expressed
OsISM07542	LOC_Os01g54030	31076932	TGATGGTGAGCGCATTTTAG	CACCTCCAAGGGCTGTGTAT	59.9	227	93	NADP-dependent malic enzyme putative expressed
OsISM07543	LOC_Os01g54030	31077139	ATACACAGCCCTTGGAGGTG	CCTCATTGTTTGTTCCCACA	59.7	161	72	NADP-dependent malic enzyme putative expressed
OsISM07544	LOC_Os01g54030	31077331	AAGACAAAAACGAGCCACTG	GGACTAGCACCTTCTGTCCA	57.9	201	93	NADP-dependent malic enzyme putative expressed

ISM IDs	Rice gene locus IDs	Physical positions (bp) on chromosomes	Forward primers (5'-3')	Reverse primers (5'-3')	Melting tempearature (T˚C)	Expected product size (bp) in PCR	Expected product size (bp) in Q-PCR	Putative Functions
OsISM07545	LOC_Os01g54030	31077487	TCATGGCTGCTATTAAGCAGAA	TGCATTGTAATTTGCGAAGTC	59.4	163	72	NADP-dependent malic enzyme putative expressed
OsISM07546	LOC_Os01g54030	31077661	TGAGAAGTACCGTGCAAACAA	GAGGCCTGCAAGCACTACAG	60.3	214	74	NADP-dependent malic enzyme putative expressed
OsISM07547	LOC_Os01g54030	31077920	TTCTTATTCTTTGGCGCAGG	CGAGAGCCACTAGTTCAGCA	59.8	533	56	NADP-dependent malic enzyme putative expressed
OsISM07548	LOC_Os01g54030	31078433	TGCTGAACTAGTGGCTCTCG	CCAGATCTTTTTGCGAGCAT	59.8	161	71	NADP-dependent malic enzyme putative expressed
OsISM07549	LOC_Os01g54030	31078574	ATGCTCGCAAAAAGATCTGG	TCGCTTCTTAAAGGGCTGAA	60.2	166	84	NADP-dependent malic enzyme putative expressed
OsISM07550	LOC_Os01g54030	31078731	AAGAAGCGATATGCCCATGA	CCCACACCAGCTGAACCTAT	60.3	190	96	NADP-dependent malic enzyme putative expressed
OsISM07551	LOC_Os01g54030	31078909	AGCTGGTGTGGGTCAAAGTT	TGTTGGATTGGATAGTGCGA	59.8	193	92	NADP-dependent malic enzyme putative expressed
OsISM07552	LOC_Os01g54030	31079082	TCGCACTATCCAATCCAACA	TCAAATGGACTTCCACTCCC	60.0	235	98	NADP-dependent malic enzyme putative expressed
OsISM07553	LOC_Os01g54030	31079331	ATGACAAGCTTTTCGTGCCT	GCTCCTGAAATCACTACGCC	59.9	229	80	NADP-dependent malic enzyme putative expressed
OsISM07554	LOC_Os01g54030	31079553	CAGGAGCGATAAGGGTGAAA	TCACCTGGTCAGCTAAGCCT	60.1	183	64	NADP-dependent malic enzyme putative expressed
OsISM07555	LOC_Os01g54030	31079790	AGATATCAGCCAACATCGCA	CTTCACCAAGTCTTTTGGGC	59.5	184	90	NADP-dependent malic enzyme putative expressed
OsISM07556	LOC_Os01g54050	31092115	CAAGCTGCATCCATGAAAAA	TGCAAATCTTCCACCTGACA	60.0	269	75	histidine-containing phosphotransfer protein putative expressed
OsISM07557	LOC_Os01g54050	31092441	GGGCAGGCTAATGTCAAACA	AGCTGCCTTTTAGTTGCTGC	60.4	230	93	histidine-containing phosphotransfer protein putative expressed
OsISM07558	LOC_Os01g54050	31092604	TGCAGGGAGAAGTACCCAAG	TTCTTCATCCTAGAAGCGCC	59.9	650	96	histidine-containing phosphotransfer protein putative expressed
OsISM07559	LOC_Os01g54050	31093272	GGATAGTTGTGGCCAAGGAA	ATGCTCCCGCTTCACTTTCT	60.6	190	65	histidine-containing phosphotransfer protein putative expressed
OsISM07560	LOC_Os01g54050	31093462	GCTGTCCTGAGGCAGAAACT	CCGGTTCTTACATGACAGGC	60.1	195	92	histidine-containing phosphotransfer protein putative expressed
OsISM07561	LOC_Os01g54060	31095772	TGGCCTATTTCTCAGGCATT	GGCTAGGAGCGTCCATACTC	59.3	215	88	hypothetical protein
OsISM07562	LOC_Os01g54080	31098591	GCGAGTACGAGGACTTCGAT	CGAGCCAATCTATCACCTCC	59.6	489	55	kinesin motor protein-related putative expressed

ISM IDs	Rice gene locus IDs	Physical positions (bp) on chromosomes	Forward primers (5'-3')	Reverse primers (5'-3')	Melting tempearature (T°C)	Expected product size (bp) in PCR	Expected product size (bp) in Q-PCR	Putative Functions
OsISM07563	LOC_Os01g54080	31099165	CTATGTTGCGGACAAGCTCA	TCTTCACATTTGACCGTTGG	59.8	149	81	kinesin motor protein-related putative expressed
OsISM07564	LOC_Os01g54080	31099351	CTTTGGCGTGAAGGATCTTG	ACCACCCAATTGTGTAGCCA	61.0	699	92	kinesin motor protein-related putative expressed
OsISM07565	LOC_Os01g54080	31100131	GCCACACCAGGGAAAAGATA	CCATGCTTAAATTGGACAACC	59.6	462	90	kinesin motor protein-related putative expressed
OsISM07566	LOC_Os01g54080	31100622	TCTCAGAGATGCTGCATTCAA	AGAAGGGATTGGGTAGGGG	59.9	151	53	kinesin motor protein-related putative expressed
OsISM07567	LOC_Os01g54080	31100786	GGCATTCTGGATGAAAGTATTGA	CGGTGCTCAATCTCCTGAAT	60.3	186	93	kinesin motor protein-related putative expressed
OsISM07568	LOC_Os01g54080	31100970	CGCATTGGTATTCAAGCAGA	CCCGTGTCTTGATGATGCTA	59.8	215	56	kinesin motor protein-related putative expressed
OsISM07569	LOC_Os01g54080	31101181	CGGGAGGATAAGTATCGTTCAA	ACCTCAAGCCGATTTATTGC	59.8	186	93	kinesin motor protein-related putative expressed
OsISM07570	LOC_Os01g54080	31101341	CAGATGGCAATAAATCGGCT	GCCGAATCATGTCTTGCTCT	60.2	347	89	kinesin motor protein-related putative expressed
OsISM07571	LOC_Os01g54080	31101924	GCAAACATTTTTCAGAGTTTTATGA	TTTGAAGGGCATACATTTCTTG	59.0	179	98	kinesin motor protein-related putative expressed
OsISM07572	LOC_Os01g54080	31102098	TCAAATGACATGGAGGGATG	TCTGGTTTTCAGCAAGAACCT	59.1	193	99	kinesin motor protein-related putative expressed
OsISM07573	LOC_Os01g54080	31102270	AGGTTCTTGCTGAAAACCAGA	GCCAGGGAGAAATGGTCTAA	59.0	200	93	kinesin motor protein-related putative expressed
OsISM07574	LOC_Os01g54080	31102538	CAAGCAAGGAAAGGAAGGGT	TCAAAGGTTGGATATCGGAGA	60.1	192	96	kinesin motor protein-related putative expressed
OsISM07575	LOC_Os01g54080	31102773	TGGACAAACTGGTTCAGGAAA	TCTCTTGATGTCCCTGGTCC	60.3	155	55	kinesin motor protein-related putative expressed
OsISM07576	LOC_Os01g54080	31103045	TCGCGATCTTCTGTCAAATG	GGGTGTAAACTTGCATCAGGA	60.0	218	97	kinesin motor protein-related putative expressed
OsISM07577	LOC_Os01g54080	31103338	AAATGAAAGGAGCAGTCGCT	CCATTCTTCACGTCCAGTCC	59.8	151	67	kinesin motor protein-related putative expressed
OsISM07578	LOC_Os01g54080	31103644	ATGCCCATGTGCCATATAGA	CAAACATTAGTGTCTTTGCTTGTC	58.4	158	82	kinesin motor protein-related putative expressed
OsISM07579	LOC_Os01g54080	31103908	GGGGAAAGACATAAAAGAGCTG	GGAGCTGAAGCTGTTCGATT	59.4	415	90	kinesin motor protein-related putative expressed
OsISM07580	LOC_Os01g54080	31104397	GGTTTGTCATCACTTCCCGT	ACTGAGCAACCATCGTCAGA	59.6	217	99	kinesin motor protein-related putative expressed

ISM IDs	Rice gene locus IDs	Physical positions (bp) on chromosomes	Forward primers (5'-3')	Reverse primers (5'-3')	Melting tempearature (T°C)	Expected product size (bp) in PCR	Expected product size (bp) in Q-PCR	Putative Functions
OsISM07581	LOC_Os01g54080	31104595	CTGACGATGGTTGCTCAGTG	TGGTTATTCTTGATGGCGCT	60.5	690	97	kinesin motor protein-related putative expressed
OsISM07582	LOC_Os01g54080	31105323	AGACCAAAACCAAGGGAGGT	TCTTTTGGAAGGCTTGGCTA	59.9	240	97	kinesin motor protein-related putative expressed
OsISM07583	LOC_Os01g54090	31107830	GTGACGGAGGCCAAGAAAT	TGAATGAACAAAGCGTCTGC	60.0	345	86	expressed protein
OsISM07584	LOC_Os01g54090	31108441	GAGGCTGGTCCAGATTCAG	CTCTTGTAGAGCCCACCGTT	58.8	316	89	expressed protein
OsISM07585	LOC_Os01g54150	31139172	TCCTCCATTTGTGGTTTCGT	GCCAAGAAATTTCCGAACTG	60.0	170	71	retrotransposon protein putative unclassified
OsISM07586	LOC_Os01g54150	31140380	TCCTCTTGGACAGCACTTGA	GAGCAACACCACGTTGAAGA	59.7	166	71	retrotransposon protein putative unclassified
OsISM07587	LOC_Os01g54180	31162343	GGGAACACCAGGATATTGGA	ACCGCCACGCTCAACTCT	60.8	1304	74	DUF640 domain containing protein putative
OsISM07588	LOC_Os01g54190	31165010	CACCTCACCCATGGCAGT	CCGGGTCCTTCTTCACCTA	60.3	201	93	expressed protein
OsISM07589	LOC_Os01g54190	31165210	GGCAAGACTTACAGGCAAGC	AGCCGAGCTAGATTGGTTCA	60.0	745	99	expressed protein
OsISM07590	LOC_Os01g54220	31186843	AGGGTGATCATGTACCCTGG	GTAGCACTTGTGCTCGGTCA	59.9	257	90	transposon protein putative unclassified
OsISM07591	LOC_Os01g54220	31188835	GTCCATCTGGACAGGCAAGT	GTGGTTCCTGTCAATCCCAC	60.2	515	75	transposon protein putative unclassified
OsISM07592	LOC_Os01g54220	31189413	GAGTGGTTGCGTGTTGACC	CGCCTCAACAAGCCTACAAA	60.7	153	79	transposon protein putative unclassified
OsISM07593	LOC_Os01g54220	31189865	TAAGGCTTGGTTGCTCCAGT	CCGAGTAATCATCAGCGTCA	59.8	146	60	transposon protein putative unclassified
OsISM07594	LOC_Os01g54220	31190355	ACGATGGGGACTTACTGGTG	GGAGACGGTCAAACTCGAAC	59.8	188	95	transposon protein putative unclassified
OsISM07595	LOC_Os01g54220	31190715	CGGACTTAGGCAGGTGTTTC	CTAGCTGTCCCCGTCGAGT	60.1	159	81	transposon protein putative unclassified
OsISM07596	LOC_Os01g54220	31191082	GCACCGTGACCGAGACTACT	ATATCGGCACTGATATCCCG	60.0	152	58	transposon protein putative unclassified
OsISM07597	LOC_Os01g54220	31191416	GAGCAAGACCTCGACCTACG	ACGCGCTCGAAGGCATAC	61.2	306	58	transposon protein putative unclassified
OsISM07598	LOC_Os01g54220	31191799	TCTCATGGAGTGTCGATCCC	AGACGAAGAGGCCCCAGTA	60.4	135	73	transposon protein putative unclassified

ISM IDs	Rice gene locus IDs	Physical positions (bp) on chromosomes	Forward primers (5'-3')	Reverse primers (5'-3')	Melting tempearature (T˚C)	Expected product size (bp) in PCR	Expected product size (bp) in Q-PCR	Putative Functions
OsISM07599	LOC_Os01g54220	31192120	TACTCCCAGGGGCACATTAG	CCGAAAGTGTGTTGTCGTGA	60.4	234	97	transposon protein putative unclassified
OsISM07600	LOC_Os01g54220	31192334	TCACGACAACACACTTTCGG	GGGTTCTCCATGTCTACCCA	60.3	586	77	transposon protein putative unclassified
OsISM07601	LOC_Os01g54220	31192965	GGACTACCTAAGGCGGAAGG	TCCATCGAGTAAATCCACCC	59.9	693	96	transposon protein putative unclassified
OsISM07602	LOC_Os01g54230	31197524	CCTTCTTCCAGTCCACGGT	CGTTGATGGTGATGAGCAG	59.4	322	97	conserved hypothetical protein
OsISM07603	LOC_Os01g54240	31202921	CTTGCCCTCAGTCTCGGA	TCCCAAGGAGACTACCGATG	60.1	2016	78	nucleoporin putative expressed
OsISM07604	LOC_Os01g54240	31204926	TCTCCTTGGGAACCCAGATT	CCCACGCAGCCTTTAAACTA	60.5	211	99	nucleoporin putative expressed
OsISM07605	LOC_Os01g54240	31205185	CAGAGCGTCTTGTTGATTGG	TCTGGAAATTGATCAGCGTG	59.6	191	88	nucleoporin putative expressed
OsISM07606	LOC_Os01g54240	31205356	CACGCTGATCAATTTCCAGA	CCATCCAACCGATAGAGCTG	60.2	317	98	nucleoporin putative expressed
OsISM07607	LOC_Os01g54240	31205660	ATCGGTTGGATGGCTTGATA	ACTACCTCACGGCTGTCCAT	60.0	173	85	nucleoporin putative expressed
OsISM07608	LOC_Os01g54240	31205803	ATTGGATCAGATGGACAGCC	CAGCTTCAACCAACCCATTT	59.9	347	48	nucleoporin putative expressed
OsISM07609	LOC_Os01g54240	31206191	TTGCCATCGGGTAAATTAGG	ACTTGACCTCGCCATTTTTC	59.5	834	78	nucleoporin putative expressed
OsISM07610	LOC_Os01g54240	31207144	CACATTGGCTGGAGCTCTTT	AACTGATGCATCCCTTCAAA	59.3	530	77	nucleoporin putative expressed
OsISM07611	LOC_Os01g54240	31207709	ACAATAGCGGATTGACAGGC	TTTTTGTGCATTCAGCCAAG	60.0	179	76	nucleoporin putative expressed
OsISM07612	LOC_Os01g54240	31207868	CTTGGCTGAATGCACAAAAA	TCCGCATAGTCATTGTCAGC	59.8	168	82	nucleoporin putative expressed
OsISM07613	LOC_Os01g54240	31208089	TGCACAGGCTTGTTTATGCT	GCCCTAGGCCTTGGTTTAGA	60.0	355	97	nucleoporin putative expressed
OsISM07614	LOC_Os01g54240	31208441	GGCTGTTGGAGATTCTGCTT	AGCTCATACAAGCGGCAAAT	59.7	164	86	nucleoporin putative expressed
OsISM07615	LOC_Os01g54240	31208581	GGAAATTTGCCGCTTGTATG	TTTCCAATGATAAATGCCAGC	60.2	254	74	nucleoporin putative expressed
OsISM07616	LOC_Os01g54240	31208924	TATTGGCAAGTCGGTCACAG	CCTGCACTACCGATGTCAGA	59.8	253	82	nucleoporin putative expressed

ISM IDs	Rice gene locus IDs	Physical positions (bp) on chromosomes	Forward primers (5'-3')	Reverse primers (5'-3')	Melting tempearature (T˚C)	Expected product size (bp) in PCR	Expected product size (bp) in Q-PCR	Putative Functions
OsISM07617	LOC_Os01g54240	31209157	TCTGACATCGGTAGTGCAGG	GCCAGTCCTTCCTTCTTGTG	59.8	175	91	nucleoporin putative expressed
OsISM07618	LOC_Os01g54240	31209311	GCACAAGAAGGAAGGACTGG	AGCCAGAAACGTTGAGGTGT	59.8	235	93	nucleoporin putative expressed
OsISM07619	LOC_Os01g54240	31209526	ACACCTCAACGTTTCTGGCT	GCAACAAAGTCGTTTCAGCA	59.9	229	98	nucleoporin putative expressed
OsISM07620	LOC_Os01g54250	31213701	GAAGTGGACTTTCAATCGCC	GACGGAGTGCTGCTGGAT	59.8	630	77	retrotransposon protein putative unclassified
OsISM07621	LOC_Os01g54250	31214888	AGACCACGATTCATCGGC	CTCTTCACGAAGGCGAAGG	60.3	215	96	retrotransposon protein putative unclassified
OsISM07622	LOC_Os01g54290	31236687	CCACCAGGATACGAGGAGAA	AACTGCTTGACCAGAAGAGC	58.7	243	64	retrotransposon protein putative unclassified
OsISM07623	LOC_Os01g54290	31236971	AGATCTTGGGGATCTGCACT	CCCAGTCTGCATCAGAGAAA	58.8	425	99	retrotransposon protein putative unclassified
OsISM07624	LOC_Os01g54330	31265755	ATTTTTACACAGGTTCGGGC	GAAGTTCTGAATAAGGAAGGAC AA	58.5	471	64	retrotransposon protein putative unclassified
OsISM07625	LOC_Os01g54330	31266203	TGTCCTTCCTTATTCAGAACTT CTTC	TATCGCACATCCTTCAATGG	60.0	922	98	retrotransposon protein putative unclassified
OsISM07626	LOC_Os01g54340	31271504	TCTGGCAGAACCAGCACTC	CTCTACCTGGCTGGTCTTGG	60.0	269	57	plant-specific domain TIGR01615 family protein expressed
OsISM07627	LOC_Os01g54340	31271915	GGTACAACAGCGCCATCTG	ACGTCCACGTAGCTGTGCT	59.9	794	77	plant-specific domain TIGR01615 family protein expressed
OsISM07628	LOC_Os01g54370	31283048	GACGACTGGCACCTCCAC	GATGGCCCTCCCAAAATG	60.7	216	82	dihydropyrimidinase putative expressed
OsISM07629	LOC_Os01g54370	31283395	TAGCCCAGAGGAGATCAAGC	GGGAGTTGGTAGTTGCTCCA	59.8	370	84	dihydropyrimidinase putative expressed
OsISM07630	LOC_Os01g54370	31283804	TGCTTGAGGAAATGGCTAGG	TGCTGATCTGTGACTTCTCCA	60.0	301	62	dihydropyrimidinase putative expressed
OsISM07631	LOC_Os01g54370	31284201	CACCACTATGGATGCGGTTA	CGCATTCCTGTTGAGAAGAAG	59.7	165	95	dihydropyrimidinase putative expressed
OsISM07632	LOC_Os01g54370	31284365	GTTATTTCAGGGTGGCTTGC	ACTCCCACTTGTTACCGCAG	59.9	406	98	dihydropyrimidinase putative expressed
OsISM07633	LOC_Os01g54370	31284849	TATAGCGCTCCTGTGGCTCT	GGGCCATTGAAGCTTGTAAA	60.1	327	93	dihydropyrimidinase putative expressed
OsISM07634	LOC_Os01g54390	31287271	CCCCACATCGTACCACTTCT	TATCCAAGCTCGCCTTAACC	59.6	187	99	S4 domain containing protein putative expressed

ISM IDs	Rice gene locus IDs	Physical positions (bp) on chromosomes	Forward primers (5'-3')	Reverse primers (5'-3')	Melting tempearature (T˚C)	Expected product size (bp) in PCR	Expected product size (bp) in Q-PCR	Putative Functions
OsISM07635	LOC_Os01g54390	31287441	TAAGGCGAGCTTGGATAGGA	GAAAACATCTCTCCGTTGGG	59.7	1155	86	S4 domain containing protein putative expressed
OsISM07636	LOC_Os01g54390	31288657	CAGACATCAAAGCGGTAGCA	CCGGATGTCCAACTGAAACT	60.0	156	70	S4 domain containing protein putative expressed
OsISM07637	LOC_Os01g54390	31288802	TGGACATCCGGACTGTATGA	CACCATGAGAACAGGGTTCC	60.1	158	90	S4 domain containing protein putative expressed
OsISM07638	LOC_Os01g54390	31288979	TGGGACAGGAATAACAAGGG	CAAGGACCTGAGCACCTCTC	59.9	172	69	S4 domain containing protein putative expressed
OsISM07639	LOC_Os01g54390	31289131	GAGAGGTGCTCAGGTCCTTG	TGCATGAAACTCCAACTTTCC	60.0	445	87	S4 domain containing protein putative expressed
OsISM07640	LOC_Os01g54390	31289572	TGCACACAGATTCCTTTGCT	ACTACCCAGGGCATCAACC	59.6	206	97	S4 domain containing protein putative expressed
OsISM07641	LOC_Os01g54390	31289767	CCCTGGGTAGTGCAGGATT	TGACCAATTCACACGGACAT	59.9	267	81	S4 domain containing protein putative expressed
OsISM07642	LOC_Os01g54390	31290064	GCTGGGGATGTTGTTTCTGT	CGAATCAGCTCAACTGCGTA	60.1	915	93	S4 domain containing protein putative expressed
OsISM07643	LOC_Os01g54410	31301791	TAGTATTTCTTCCGAGCCGC	ACCTTGACCACGGTAGTCCC	60.3	128	71	hypothetical protein
OsISM07644	LOC_Os01g54470	31330694	GTTCAGCAACGGCCTCAC	ATGTAGTCGTCGAAGCCCAG	60.3	162	61	GDSL-like lipase/acylhydrolase putative expressed
OsISM07645	LOC_Os01g54470	31330924	GAATCCGGGACGACACTG	AGGATGCTAACCAGCTGCC	60.2	240	98	GDSL-like lipase/acylhydrolase putative expressed
OsISM07646	LOC_Os01g54470	31331306	GTGCTCATCAACCAGTACGC	GAATACCGCCACCTTCCTG	59.9	176	70	GDSL-like lipase/acylhydrolase putative expressed
OsISM07647	LOC_Os01g54470	31331659	GGCATCTTCGAGAGCATCAT	GTCACCTGCCCGTTGTTC	60.2	199	96	GDSL-like lipase/acylhydrolase putative expressed
OsISM07648	LOC_Os01g54490	31342498	CCGACCTGAGGGTGTTCTAC	CTAGTGATGGGTTGCTTGGG	60.0	194	70	osFTL9 FT-Like9 homologous to Flowering Locus T gene; contains Pfam profile PF01161: Phosphatidylethanolamine-binding protein
OsISM07649	LOC_Os01g54490	31342652	GTGGATCCAGATGCTCCAAG	TGACTCCAGTTGTTCCTGGG	60.9	385	77	osFTL9 FT-Like9 homologous to Flowering Locus T gene; contains Pfam profile PF01161: Phosphatidylethanolamine-binding protein
OsISM07650	LOC_Os01g54490	31343016	TCCCAGGAACAACTGGAGTC	ACCATCCGGTGGATACCATA	60.0	1285	80	osFTL9 FT-Like9 homologous to Flowering Locus T gene; contains Pfam profile PF01161: Phosphatidylethanolamine-binding protein
OsISM07652	LOC_Os01g54510	31349470	GTTGGGATCCTTCAGCATTG	TCATCCTTACCACCAACCGT	60.3	215	83	MAC/Perforin domain containing protein putative expressed
OsISM07653	LOC_Os01g54510	31349759	GCGAGAGATTCTCAGATGGG	CACAACCACCCCTCCTTCT	59.9	395	97	MAC/Perforin domain containing protein putative expressed

ISM IDs	Rice gene locus IDs	Physical positions (bp) on chromosomes	Forward primers (5'-3')	Reverse primers (5'-3')	Melting tempearature (T˚C)	Expected product size (bp) in PCR	Expected product size (bp) in Q-PCR	Putative Functions
OsISM07654	LOC_Os01g54510	31350251	TATGCCTGGAAAGAAGCACC	TGCAACTCTTCAATTTGGGG	60.6	456	73	MAC/Perforin domain containing protein putative expressed
OsISM07655	LOC_Os01g54510	31350805	TTAGTTTGATGGGCCCAAAG	CCTTCCAGGCATAGCTTCAG	60.0	397	89	MAC/Perforin domain containing protein putative expressed
OsISM07656	LOC_Os01g54515	31355889	GTCTTCGCGGACACTTTCAT	TTGGAGTGAGCTTGCAGTGT	59.9	211	99	peptide transporter PTR2 putative expressed
OsISM07657	LOC_Os01g54515	31356482	ACCGCGCTTCTTGAAATCT	ATTCCTGTTCCTGATGGCAG	60.0	162	52	peptide transporter PTR2 putative expressed
OsISM07658	LOC_Os01g54515	31356680	ATCAGCAAGAGCAAAGCTCC	CTGTACTATCGCCGCCTTGT	60.0	177	88	peptide transporter PTR2 putative expressed
OsISM07659	LOC_Os01g54520	31358463	CTCCAGTGCGCCGTCTAC	AGATCTTCCGCGACACGATA	60.7	171	77	DUF1264 domain containing protein putative expressed
OsISM07660	LOC_Os01g54530	31361877	ACGTATCCAGCTCCAGCCT	TGGGACTAAGGAAGTTCAAGC	59.1	611	88	hydrolase acting on carbon-nitrogen putative expressed
OsISM07661	LOC_Os01g54530	31362619	CATGCTGAGGCTACCAGGTT	CCACAGTGCACATTAACAGTTTC	59.9	189	99	hydrolase acting on carbon-nitrogen putative expressed
OsISM07662	LOC_Os01g54530	31362884	TCTACTTGTGCGTTTGGTGG	ACCTCATCCCAGAAAGCAAC	59.4	198	99	hydrolase acting on carbon-nitrogen putative expressed
OsISM07663	LOC_Os01g54530	31363145	GCGCATAATTGTTGTCAGGA	ATCTTGCCTCTGGGAAAAGG	60.1	365	96	hydrolase acting on carbon-nitrogen putative expressed
OsISM07664	LOC_Os01g54530	31363714	ATGGGATGCCAGATGAAAAA	CCAGCTCCCTCACAATGACT	60.3	212	73	hydrolase acting on carbon-nitrogen putative expressed
OsISM07665	LOC_Os01g54540	31366298	GATTCATGTTCGAAGGCGAC	GGGTACCATGTTGTGTTCCA	59.9	622	59	ribosomal protein L13 putative expressed
OsISM07666	LOC_Os01g54540	31367031	TGAGGCCACATACACTCCAA	GAGCTTCTGTGACCGCTTTT	59.9	381	98	ribosomal protein L13 putative expressed
OsISM07667	LOC_Os01g54540	31367509	GTGGCATGCTTCCTAAGGG	TGGAACAGGTTTTTGAGCCT	60.2	574	99	ribosomal protein L13 putative expressed
OsISM07668	LOC_Os01g54550	31369701	GCTCCCCAAGTACTTCAAGC	GCAAATTCCCATTGTTCTGG	59.6	681	97	HSF-type DNA-binding domain containing protein expressed
OsISM07669	LOC_Os01g54580	31394465	GAGTTGGTACACGCTGAGCA	AACTTCCACATGTGCTTCCC	60.0	179	70	transporter family protein putative expressed
OsISM07670	LOC_Os01g54580	31394740	GGCTAGTGCAATGACATGGA	CAAGAGCAGTAGCCAAAGCTG	60.0	840	66	transporter family protein putative expressed
OsISM07671	LOC_Os01g54580	31395590	GAGCAACATTTCACTTGTTTTCA	CAGAAGAATGAAAATTGGAGCTG	59.8	182	77	transporter family protein putative expressed

ISM IDs	Rice gene locus IDs	Physical positions (bp) on chromosomes	Forward primers (5'-3')	Reverse primers (5'-3')	Melting tempearature (T˚C)	Expected product size (bp) in PCR	Expected product size (bp) in Q-PCR	Putative Functions
OsISM07672	LOC_Os01g54580	31395747	TGCAGCTCCAATTTTCATTC	CAAGAAGGTTAAAGCTGGGC	58.9	578	69	transporter family protein putative expressed
OsISM07673	LOC_Os01g54580	31396305	GCCCAGCTTTAACCTTCTTG	AAATACAAATCCCCAAAGATTGA	58.9	367	98	transporter family protein putative expressed
OsISM07674	LOC_Os01g54580	31396703	GCTGGTGCATGACACAGATT	TCCTCTCAATGGACAAAAAGG	59.4	261	79	transporter family protein putative expressed
OsISM07675	LOC_Os01g54580	31396897	TGGAATCTACTGAAGAAAGAGGAA	GCCATAACCGGGGTAACATA	59.1	340	75	transporter family protein putative expressed
OsISM07676	LOC_Os01g54580	31397340	ATGCTTCTTGGTGGTGCTTT	ACAATCCCAGCTACGGTCAC	59.9	307	96	transporter family protein putative expressed
OsISM07677	LOC_Os01g54580	31397627	GTGACCGTAGCTGGGATTGT	CAAGCCCCTTTAACCAGGTA	59.5	195	89	transporter family protein putative expressed
OsISM07678	LOC_Os01g54580	31397802	TACCTGGTTAAAGGGGCTTG	TGGCCTTTCTTGAACCTATGA	59.4	429	88	transporter family protein putative expressed
OsISM07679	LOC_Os01g54590	31403060	GTCGTCAAGGCCCAGATTT	AGCAGTGCACCTACTGCTCC	60.3	2512	90	ras-related protein putative expressed
OsISM07680	LOC_Os01g54610	31418895	GGTGATGATGGAGGGGATCT	AGCTGGTAGACCGCAAAGAA	60.6	1064	46	retrotransposon protein putative unclassified
OsISM07682	LOC_Os01g54620	31422387	CAGCCAACTCAGCGATCA	ATGGGTGCTCATAGTCCTGG	59.8	155	56	CESA4 - cellulose synthase expressed
OsISM07683	LOC_Os01g54620	31422541	TGCCAGGACAATTAGCAGTG	TTTCTCCTTCCAGCTCTCCA	60.0	207	98	CESA4 - cellulose synthase expressed
OsISM07684	LOC_Os01g54620	31422772	AAGAAAGCTGCAGCAAAAGC	GAATGATCCGAGAAAGTGGC	59.8	831	98	CESA4 - cellulose synthase expressed
OsISM07685	LOC_Os01g54620	31423775	GTTCCCCAAGTGGTGTCCTA	ATCTACAGGTGCCAACCCAG	59.9	197	95	CESA4 - cellulose synthase expressed
OsISM07686	LOC_Os01g54620	31424243	CATCTTTCGTCAAGGAGCGT	CCTCAGGTGTCTTCTGAGCC	60.2	187	97	CESA4 - cellulose synthase expressed
OsISM07687	LOC_Os01g54620	31424468	AACAACCCTCGTGACCACC	AGGAAGCTCATTTCCGTCAA	60.3	670	82	CESA4 - cellulose synthase expressed
OsISM07688	LOC_Os01g54620	31425166	GACCAGGGTACCAACACCAC	TGTAAGGGGCATTTGTCAGA	59.6	195	82	CESA4 - cellulose synthase expressed
OsISM07689	LOC_Os01g54620	31425467	ATTCCCACAAAGGTTCGATG	CCATCAAGCCCTTTCATGTT	59.9	172	91	CESA4 - cellulose synthase expressed
OsISM07690	LOC_Os01g54620	31425827	TTGAGTCCGCCATTTTCAAT	GCATCGAGCGTTCATACTCA	60.2	145	61	CESA4 - cellulose synthase expressed

ISM IDs	Rice gene locus IDs	Physical positions (bp) on chromosomes	Forward primers (5'-3')	Reverse primers (5'-3')	Melting tempearature (T°C)	Expected product size (bp) in PCR	Expected product size (bp) in Q-PCR	Putative Functions
OsISM07691	LOC_Os01g54620	31426084	CAAAGAGGCCATTCATGTCA	ACTGAACCGTAGATCCAGCC	59.4	175	82	CESA4 - cellulose synthase expressed
OsISM07692	LOC_Os01g54620	31426562	GCTCACAGGCAAATTCATCA	GATGGAGATGAAGAGGCCAA	60.0	168	74	CESA4 - cellulose synthase expressed
OsISM07693	LOC_Os01g54650	31440554	CCCTCCAAAGTGGTCGTTTA	ACTGGCCGGACTTGGAAT	60.2	171	78	retrotransposon protein putative unclassified
OsISM07694	LOC_Os01g54680	31461818	CAAGGAGTCCTCCCCCTCT	GGTACCCTCCTGCTTCCTCT	60.1	231	97	retrotransposon protein putative unclassified
OsISM07695	LOC_Os01g54680	31462186	CCGGGGAAACCTTATCTCTC	GACTGTCCGGACTTGGAATG	60.2	202	97	retrotransposon protein putative unclassified
OsISM07696	LOC_Os01g54714	31480153	GTGCAAGTGCAACGTGGT	GCAAGCTTCATACTCGGAGC	59.7	463	57	retrotransposon protein putative unclassified
OsISM07697	LOC_Os01g54714	31480581	GCGGTATGTTTTTCTGCTCC	ACCACCACAGGCTGTCGAA	61.2	4303	96	retrotransposon protein putative unclassified
OsISM07698	LOC_Os01g54760	31503000	TTCGCAGCGTACCAAAAGAT	GCGTACCTTTGTAAATACATGTCG	60.3	132	85	hypothetical protein
OsISM07699	LOC_Os01g54760	31503048	GCAACAAGGTAATTGGTAGATTGG	CCTACTCGAGTGCTCAATGG	59.7	420	99	hypothetical protein
OsISM07700	LOC_Os01g54760	31503554	TATCTCATAGCACCGGCCAT	TCTCTTGGACACCAATGGAA	59.8	439	85	hypothetical protein
OsISM07701	LOC_Os01g54760	31504189	AATCTCTCGCACCGCAAC	TCGCCTTAGTCGAACTTGGT	59.9	855	99	hypothetical protein
OsISM07702	LOC_Os01g54770	31508484	CGCTGCGAAGAACGACACT	TGACACCAAGCAGACGAAAC	61.3	130	91	hypothetical protein
OsISM07703	LOC_Os01g54784	31510846	ATCGACGCTTCCATTTCAGT	TACCTTTTGCCGAGTCCATC	59.9	575	88	expressed protein
OsISM07704	LOC_Os01g54784	31511366	GCTAGCAAGAATCCACAGGC	TGCATAGGCCAATCATAAAATG	59.9	942	78	expressed protein
OsISM07705	LOC_Os01g54784	31512262	ATGCCTACGTTCTTGCAGAT	GCGATGATGGTAGACACGAA	58.6	758	84	expressed protein
OsISM07706	LOC_Os01g54784	31513044	GGTCCATGGATGTGGATTTG	AAGTGCCGACTCACCAGAAT	60.4	224	93	expressed protein
OsISM07707	LOC_Os01g54784	31513221	TCTTGCTTCTTTTCTGTTGCTG	AAGCCTCATGGTGACTCCTC	59.5	1492	96	expressed protein
OsISM07708	LOC_Os01g54784	31514774	CCCAGGAGTACAGCTTGGAA	CGAAGGCGATTGTAACAACA	60.0	272	78	expressed protein

ISM IDs	Rice gene locus IDs	Physical positions (bp) on chromosomes	Forward primers (5'-3')	Reverse primers (5'-3')	Melting tempearature (T˚C)	Expected product size (bp) in PCR	Expected product size (bp) in Q-PCR	Putative Functions
OsISM07709	LOC_Os01g54784	31515052	TCATCTGGATTGGTAGAGGAGAA	CGCACCACCAAGTACAAGAA	59.9	731	99	expressed protein
OsISM07710	LOC_Os01g54784	31515763	TTCTTGTACTTGGTGGTGCG	CGCACTTTGCTCATCTTTGA	59.9	356	65	expressed protein
OsISM07711	LOC_Os01g54784	31516099	TCAAAGATGAGCAAAGTGCG	CGGACGACGAGAAACAAATC	60.4	749	95	expressed protein
OsISM07712	LOC_Os01g54784	31516828	GATTTGTTTCTCGTCGTCCG	TGTTGATGATGTATTCATCTGCTG	60.3	189	90	expressed protein
OsISM07713	LOC_Os01g54784	31516993	CAGCAGATGAATACATCATCAACA	CCATAGAACAAATCCTGACGG	59.7	877	87	expressed protein
OsISM07714	LOC_Os01g54784	31517962	GGATCCACAATGGAGGACTG	TGAAGTTTCCACATCAACGG	59.9	190	94	expressed protein
OsISM07715	LOC_Os01g54820	31528726	GTCTTCCACCAGCCCTTCTA	TTTTTCGAGAGATGGCCAAG	59.8	335	77	retrotransposon protein putative Ty3-gypsy subclass
OsISM07716	LOC_Os01g54820	31529839	TCAAGGACTGGTTCTGCAGTT	TCCACCTCCATTCGGAATTA	60.1	672	51	retrotransposon protein putative Ty3-gypsy subclass
OsISM07717	LOC_Os01g54820	31530826	CAAACGCCTCCAACTCCTTA	GCCGATGGAGTCTGATCACT	60.2	301	69	retrotransposon protein putative Ty3-gypsy subclass
OsISM07718	LOC_Os01g54820	31531158	CAACCAGCTGCTCCTGCTAT	GGGCTTTGCTTGAAGTGTCT	60.0	178	98	retrotransposon protein putative Ty3-gypsy subclass
OsISM07719	LOC_Os01g54850	31550518	GTTGACCTGCCTCACGCT	CAGCCAAGTTTGGAGTGTCTT	59.7	659	75	OsFBX24 - F-box domain containing protein expressed
OsISM07720	LOC_Os01g54850	31551250	GAAGCTGCTTGAGAGTTGCC	TGCAGTGTCGTCTTTTGCAT	60.4	180	71	OsFBX24 - F-box domain containing protein expressed
OsISM07721	LOC_Os01g54890	31567446	GAAGCAGGTCGGCATGAG	AAAATGTGAGCACTAAGCACAGA	59.8	215	76	ethylene-responsive transcription factor 2 putative expressed
OsISM07722	LOC_Os01g54910	31575007	TGGACTCCATCAGCCTCG	TTGAGTTCGTTCTCCTTCCAA	60.4	558	83	GTP-binding protein typA/bipA putative expressed
OsISM07723	LOC_Os01g54910	31575582	GTCACGCTGATTTTGGTGG	CTTGGTCTGCGCTAAAGGTC	60.1	203	99	GTP-binding protein typA/bipA putative expressed
OsISM07724	LOC_Os01g54910	31575799	GGCTTTGAAGTATGGCTTGC	TCATTGCAGGTCTCTTCCG	59.9	637	79	GTP-binding protein typA/bipA putative expressed
OsISM07725	LOC_Os01g54910	31576417	CGGAAGAGACCTGCAATGA	CGATGCATAAAGAACTGGGAA	60.0	180	99	GTP-binding protein typA/bipA putative expressed
OsISM07726	LOC_Os01g54910	31576706	CTGACCTTGAGGCTCCATTC	CCCCAGATCTTACTCGTCCA	59.9	530	91	GTP-binding protein typA/bipA putative expressed

ISM IDs	Rice gene locus IDs	Physical positions (bp) on chromosomes	Forward primers (5'-3')	Reverse primers (5'-3')	Melting tempearature (T˚C)	Expected product size (bp) in PCR	Expected product size (bp) in Q-PCR	Putative Functions
OsISM07727	LOC_Os01g54910	31577272	GCACAGATGATGGTGTTCAGA	CAGCTCCTGCAGCCTCTACT	59.8	234	97	GTP-binding protein typA/bipA putative expressed
OsISM07728	LOC_Os01g54910	31577536	CTCCAGCAATTGGGCATACT	GATATTGTGGGAGGGTCCAA	59.9	160	80	GTP-binding protein typA/bipA putative expressed
OsISM07729	LOC_Os01g54910	31577676	TTGGACCCTCCCACAATATC	TTGCCTATTTTCGCTCCAGT	59.7	1220	93	GTP-binding protein typA/bipA putative expressed
OsISM07730	LOC_Os01g54910	31578965	TGAAGTTCAAGGAAGGGGAG	CTTTTGGGGGTGAAACTGAA	59.6	258	90	GTP-binding protein typA/bipA putative expressed
OsISM07731	LOC_Os01g54910	31579201	CTTTCAGTTTCACCCCCAAA	CCTTTCTCCACGGTCTGTTC	59.8	320	49	GTP-binding protein typA/bipA putative expressed
OsISM07732	LOC_Os01g54910	31579501	GAACAGACCGTGGAGAAAGG	CCATGACAAATCCAACATGC	59.7	179	79	GTP-binding protein typA/bipA putative expressed
OsISM07733	LOC_Os01g54910	31579746	CTCGGATCTTTTTGACCTGC	CCACGTGTAAAGCTGCTGAA	59.9	225	71	GTP-binding protein typA/bipA putative expressed
OsISM07734	LOC_Os01g54910	31579965	ACGTGGAACTGGGTTTATGC	TAACATTGCCCAGCTGACCT	60.3	205	69	GTP-binding protein typA/bipA putative expressed
OsISM07735	LOC_Os01g54910	31580153	TCAGCTGGGCAATGTTAGAA	AGTGATAAGTCCCTTGCCGA	59.6	294	56	GTP-binding protein typA/bipA putative expressed
OsISM07736	LOC_Os01g54910	31580481	TTCTGTTTGTTTCTCCTGGGAT	TCACGTGAGTGCTCTCCAAC	60.0	152	62	GTP-binding protein typA/bipA putative expressed
OsISM07737	LOC_Os01g54910	31580603	AGGCATGATTGTTGGAGAGC	TTCCTGGAGCTCGAATGTTT	60.0	169	90	GTP-binding protein typA/bipA putative expressed
OsISM07738	LOC_Os01g54910	31580753	AACATTCGAGCTCCAGGAAA	TCCAATTGCCTCTTCCAAAC	59.9	369	76	GTP-binding protein typA/bipA putative expressed
OsISM07739	LOC_Os01g54910	31581102	GTTTGGAAGAGGCAATTGGA	GATATTTCTTCCGAAGCCGA	59.7	814	82	GTP-binding protein typA/bipA putative expressed
OsISM07740	LOC_Os01g54940	31598459	TCTCCATCAACCCTCTGGAC	CACTTATATCCCGGCCAATG	60.1	623	94	dehydrogenase putative expressed
OsISM07741	LOC_Os01g54940	31599195	TCACAGGATGAGCTTACTTCCA	GTACCATGTAAAGCACGCCA	59.7	540	99	dehydrogenase putative expressed
OsISM07742	LOC_Os01g54940	31599726	ACATGGTACGGCTCGAATTT	AACAGCAGCTAGCCCAACAG	60.0	619	77	dehydrogenase putative expressed
OsISM07743	LOC_Os01g54940	31600398	TATTGAACAAGTGTTGGCCG	CGGCATCGAACTTTCCTTTA	59.9	1316	90	dehydrogenase putative expressed
OsISM07744	LOC_Os01g54940	31601765	TGAGGAGAGGCGGACACTAT	AGTAGCAGCAGGAAGTCCCA	59.9	178	90	dehydrogenase putative expressed

ISM IDs	Rice gene locus IDs	Physical positions (bp) on chromosomes	Forward primers (5'-3')	Reverse primers (5'-3')	Melting tempearature (T˚C)	Expected product size (bp) in PCR	Expected product size (bp) in Q-PCR	Putative Functions
OsISM07745	LOC_Os01g54940	31601923	TGGGACTTCCTGCTGCTACT	TCAGCTCTCATGTATGTCCACC	60.1	1250	95	dehydrogenase putative expressed
OsISM07746	LOC_Os01g54969	31610475	ACATGTTCGACCACGTCCC	AGTATCCGCACGCACTCCT	61.3	347	86	O-methyltransferase putative
OsISM07747	LOC_Os01g54980	31612345	CGTCAGCTCCATCCTACCTC	TCTGCTTTGCGTAAGCTCAA	59.9	253	78	expressed protein
OsISM07749	LOC_Os01g55020	31634924	GCTCATGACGTCGCTCCT	GCTGGGCCAATACAAAAGAA	60.1	1488	58	expressed protein
OsISM07750	LOC_Os01g55150	31727455	GATCCGGGACCCCAAGAG	ACTGAAGCTTCCTCACTCGC	61.3	210	78	bZIP transcription factor domain containing protein expressed
OsISM07751	LOC_Os01g55150	31727638	AGAGGTCGCGAGTGAGGAAG	GAGGGGACAGCACGGATAC	61.3	1037	88	bZIP transcription factor domain containing protein expressed
OsISM07752	LOC_Os01g55150	31728744	CGGCTCTAGCACAGGACAAG	CTCGATCTCCTTCCTCAACG	60.5	223	63	bZIP transcription factor domain containing protein expressed
OsISM07754	LOC_Os01g55180	31744403	ATCACGAGTGGACCTGCCT	GTCGAGAGTTGGCGTGTACC	60.7	109	63	hypothetical protein
OsISM07755	LOC_Os01g55180	31744604	ATCACGCCTGGGTTTTACC	TTTGGGTCATTAAGTTTCTGGA	59.2	1471	99	hypothetical protein
OsISM07756	LOC_Os01g55180	31746054	CCAGAAACTTAATGACCCAAACA	ATTGTACCCGCGTATGCTCT	59.9	233	97	hypothetical protein
OsISM07757	LOC_Os01g55180	31746307	ATTCCCTTCCGTTCATTGTG	GGGAGCACAATGTGGATGTT	60.3	694	75	hypothetical protein
OsISM07760	LOC_Os01g55190	31750819	AATGGGACAGTCGAGCTCAT	CGTTTCCAAGACGTACAGCA	59.8	190	92	expressed protein
OsISM07761	LOC_Os01g55190	31751274	CAACGTTGCCATAAAGAAGC	GGACCCTCCTCAAAGTCTCC	59.2	209	79	expressed protein
OsISM07762	LOC_Os01g55200	31760411	CAGGAAATACCTCGTCTCGC	CGAGTACACCACCAGGATGA	59.7	189	77	potassium channel KAT1 putative expressed
OsISM07763	LOC_Os01g55200	31760718	CGTTCGTGGACAAGAAGTCC	CGAGCATACATCGAAGACGA	60.3	203	93	potassium channel KAT1 putative expressed
OsISM07764	LOC_Os01g55200	31760965	GCATGACCTTGGCTTCAAGT	TTGAACCGGATGTCCTTCTC	60.2	203	97	potassium channel KAT1 putative expressed
OsISM07765	LOC_Os01g55200	31761148	GAGAAGGACATCCGGTTCAA	CAGTGTATCGCGAAGAGGGT	60.2	191	75	potassium channel KAT1 putative expressed
OsISM07766	LOC_Os01g55200	31761573	CTACCTCATCGGCAACATGA	ATTCTGAGGCGGCCTGAAC	61.2	179	87	potassium channel KAT1 putative expressed

ISM IDs	Rice gene locus IDs	Physical positions (bp) on chromosomes	Forward primers (5'-3')	Reverse primers (5'-3')	Melting tempearature (T˚C)	Expected product size (bp) in PCR	Expected product size (bp) in Q-PCR	Putative Functions
OsISM07767	LOC_Os01g55200	31761893	ACCTCTTCTTCCGGGTTGTT	GCGAAGTACTCTGCTTGCATC	60.1	179	98	potassium channel KAT1 putative expressed
OsISM07768	LOC_Os01g55200	31762104	GCCCATCGGACTTGTATCTT	CAGAAACAGCAACTCAGGAGA	58.6	186	99	potassium channel KAT1 putative expressed
OsISM07769	LOC_Os01g55200	31762189	TGCACAAATCGTAACGGAAA	CAGTTCTCCCTCAGCTGCTC	60.2	209	99	potassium channel KAT1 putative expressed
OsISM07770	LOC_Os01g55240	31794533	AGTACCTCCTCCTCGCCCT	CCCGAGATGTACTCGTTCAGA	60.2	202	98	gibberellin 2-beta-dioxygenase putative expressed
OsISM07771	LOC_Os01g55240	31795016	GCGACTCCTTCTTCGTCAAC	CTTCACGCTCTTGAACCTCC	60.0	757	69	gibberellin 2-beta-dioxygenase putative expressed
OsISM07772	LOC_Os01g55250	31798912	CGGGAGGGGAGGTACTTG	TGGTGGTACGGTTGTCTTCA	60.2	549	79	hypothetical protein
OsISM07774	LOC_Os01g55260	31806477	GTCTACGAGCAGTTCGAGCA	CTCACATCGCATCAGCATTC	59.9	207	83	AAA-type ATPase family protein putative expressed
OsISM07775	LOC_Os01g55260	31806603	GATCGGTGTTGCTGTTTCAG	ACGAAGGTAGGAGGGGCTT	59.7	860	97	AAA-type ATPase family protein putative expressed
OsISM07776	LOC_Os01g55260	31807462	TTTGAGTCAGCTGAAATGCG	CAGTCCTTTGATGCTTTCCC	59.9	179	94	AAA-type ATPase family protein putative expressed
OsISM07777	LOC_Os01g55260	31807642	AGAATGCCAAACGCCTTCTA	AAATGCCTTTCCATGGTGAC	59.8	320	91	AAA-type ATPase family protein putative expressed
OsISM07778	LOC_Os01g55260	31807959	TTTTACTTTTTGGTCCCCCA	TAGCAACAGCCTTTGCCAG	59.7	480	55	AAA-type ATPase family protein putative expressed
OsISM07779	LOC_Os01g55260	31808459	TTCAACATTTCAGCATCATCA A	GGTGCATGATGTCTAGCAAGC	60.0	463	93	AAA-type ATPase family protein putative expressed
OsISM07780	LOC_Os01g55260	31808955	TATCAGCCAACGTGGTGAAG	TTCGTCTTTGTCAAGCCATC	59.3	256	94	AAA-type ATPase family protein putative expressed
OsISM07781	LOC_Os01g55260	31809241	TTTACCTTGGGAACTGGACG	CAGCCTCAGGAAGAGGTACG	60.0	289	72	AAA-type ATPase family protein putative expressed
OsISM07782	LOC_Os01g55260	31809659	CTGCCATGCAACCACTAAGA	GTTTGAGAGGACCAACCTCG	59.8	221	94	AAA-type ATPase family protein putative expressed
OsISM07783	LOC_Os01g55300	31829407	CGACATGGATTGCTTCTACG	TCCAGTCGTTGTACTGCACC	59.5	216	90	DNA-directed polymerase putative expressed
OsISM07784	LOC_Os01g55300	31829609	GTACAACGACTGGAAAGGCG	GACCATCTTGGCCTCATCAC	60.6	198	98	DNA-directed polymerase putative expressed
OsISM07785	LOC_Os01g55300	31829819	AATTTGGTTCAGGTTCCGGT	CCTTGGTCGAGAGGATTGTG	60.6	182	92	DNA-directed polymerase putative expressed

ISM IDs	Rice gene locus IDs	Physical positions (bp) on chromosomes	Forward primers (5'-3')	Reverse primers (5'-3')	Melting tempearature (T˚C)	Expected product size (bp) in PCR	Expected product size (bp) in Q-PCR	Putative Functions
OsISM07786	LOC_Os01g55300	31830095	GTTGATTTTTGAGGAGGCCA	TGCCCTCACGCTATCTTCTC	60.3	163	80	DNA-directed polymerase putative expressed
OsISM07787	LOC_Os01g55300	31830338	GGTTTTGGAGGAAACCCAAT	TGTGCATCCCACTGACAAGT	60.1	186	81	DNA-directed polymerase putative expressed
OsISM07788	LOC_Os01g55300	31830532	CAGCAGACAGTTGTTCCATCA	CTCCCAAGCTTACCACCAAG	59.8	210	90	DNA-directed polymerase putative expressed
OsISM07789	LOC_Os01g55300	31830760	TGGGGTCAATACTGTTGGTG	TCTTGCAATCTTCCATAACCA	58.7	307	98	DNA-directed polymerase putative expressed
OsISM07790	LOC_Os01g55300	31831119	GTGGAAAGACATTTCCTGGC	ACTGAATCCGTTCGCTCAGT	59.7	1198	97	DNA-directed polymerase putative expressed
OsISM07791	LOC_Os01g55300	31832347	AAACACTAACTCTCTATGCCA GGG	CGGACAAGATTTGGAAGGAA	60.1	188	78	DNA-directed polymerase putative expressed
OsISM07792	LOC_Os01g55300	31832650	CAGCAAGCAAAATATTCGACA	CGAGGTTAGCTGGTGAAACA	58.9	440	91	DNA-directed polymerase putative expressed
OsISM07793	LOC_Os01g55300	31833074	TCACCAGCTAACCTCGATTG	TGATGGTTCACAGTGTTCTTCA	59.0	179	97	DNA-directed polymerase putative expressed
OsISM07794	LOC_Os01g55300	31833292	GCCAAACAGTGTCAAATAAAG G	TGAAAGAAATTTCAGAATAGAG CAA	58.7	1009	88	DNA-directed polymerase putative expressed
OsISM07795	LOC_Os01g55300	31834327	AAAAATTGACAGCTTAATTTG CAG	ATACTGCGCAGCCTTGTGTT	59.9	166	86	DNA-directed polymerase putative expressed
OsISM07796	LOC_Os01g55300	31834660	ACGGGGTTCTACCATTTCGT	CTGGCAGCATCTGTTTTCCT	60.5	716	98	DNA-directed polymerase putative expressed
OsISM07797	LOC_Os01g55380	31891139	TGACGGCAAAGGGAATAATG	GGATAACGACCGAAGGAGGT	60.6	252	62	hypothetical protein
OsISM07798	LOC_Os01g55380	31891371	ACCTCCTTCGGTCGTTATCC	TGGAACAGCTTTATGCATCTTG	60.3	168	97	hypothetical protein
OsISM07799	LOC_Os01g55410	31919758	AAGCATCTTGGGTGTGCTTC	CCTGGACGAACATACTTCTGC	60.0	191	78	expressed protein
OsISM07800	LOC_Os01g55410	31920707	CCTCAATTCAGCCAAAGGAG	TCAGCATCCAATGAATTAGGC	59.9	183	95	expressed protein
OsISM07802	LOC_Os01g55490	31968737	GCTATCGTGGACTACGCTCA	CATCCAAACCCGATCTGTCT	59.5	175	89	transcriptional regulator family protein putative expressed
OsISM07803	LOC_Os01g55490	31968892	AGACAGATCGGGTTTGGATG	TTGGGGTCAGTATGTGAACAA	59.4	1167	84	transcriptional regulator family protein putative expressed
OsISM07804	LOC_Os01g55490	31970214	CTTCCTCCTCCTGTGACTGC	GAAGCTCTTTCCAGCCCTTT	60.0	612	88	transcriptional regulator family protein putative expressed

ISM IDs	Rice gene locus IDs	Physical positions (bp) on chromosomes	Forward primers (5'-3')	Reverse primers (5'-3')	Melting tempearature (T˚C)	Expected product size (bp) in PCR	Expected product size (bp) in Q-PCR	Putative Functions
OsISM07805	LOC_Os01g55490	31970940	CCTTATGGGTACGATAGAAGCG	TCCTTTCTCTCAACTTCACGC	59.8	585	72	transcriptional regulator family protein putative expressed
OsISM07806	LOC_Os01g55490	31971580	CCAATTAGTGCGGCAATACC	CTGTACGCCTTCTCCCGATA	60.3	180	94	transcriptional regulator family protein putative expressed
OsISM07807	LOC_Os01g55490	31971740	TATCGGGAGAAGGCGTACAG	TTGGAGCCACAGGGTTCTTA	60.4	174	91	transcriptional regulator family protein putative expressed
OsISM07808	LOC_Os01g55490	31971954	AGCAAATGTTGGTGCTGGAT	CGCTTGTTTTCTTTTCTTCAGC	60.5	511	72	transcriptional regulator family protein putative expressed
OsISM07809	LOC_Os01g55500	31975426	CAGGGGTTCGAGTACTGCAT	TGGAATCCCAGTATGATCGC	60.5	160	63	nucleobase-ascorbate transporter putative expressed
OsISM07810	LOC_Os01g55500	31975582	TCCAGCACTACATCCTGGC	CACCACCCTCACCCTGTC	59.9	196	96	nucleobase-ascorbate transporter putative expressed
OsISM07811	LOC_Os01g55500	31975884	TGGCCATCATACAGGACTCA	CTGTATCGCCCTCATGGTCT	60.1	211	78	nucleobase-ascorbate transporter putative expressed
OsISM07812	LOC_Os01g55500	31976118	CATTCAGATCATCCTCGGCT	CGAGCAATGCAACCACTG	60.1	250	90	nucleobase-ascorbate transporter putative expressed
OsISM07813	LOC_Os01g55500	31976350	CAGTGGTTGCATTGCTCG	AAGTCCAACCTCAACGCATC	60.0	202	75	nucleobase-ascorbate transporter putative expressed
OsISM07814	LOC_Os01g55500	31976551	TCCAATGTTAATTCTGTTTGTCG	GCAGATGAACAGGGAGAACC	59.4	189	95	nucleobase-ascorbate transporter putative expressed
OsISM07815	LOC_Os01g55500	31976832	CCGTGCCAATCTCATCTCTT	GATTGACCGGCACTGAAAGT	60.2	548	85	nucleobase-ascorbate transporter putative expressed
OsISM07816	LOC_Os01g55500	31977360	ACTTTCAGTGCCGGTCAATC	GAGCCGCAGCTTTGTAAGAA	60.4	170	86	nucleobase-ascorbate transporter putative expressed
OsISM07817	LOC_Os01g55500	31977535	GCAAGTGCTACTCCACCTCC	GCCCATCAAGAAGGATACCA	59.9	169	77	nucleobase-ascorbate transporter putative expressed
OsISM07818	LOC_Os01g55500	31977684	TGGTATCCTTCTTGATGGGC	CCGATCCTCGTTGATCCTAA	60.0	160	88	nucleobase-ascorbate transporter putative expressed
OsISM07819	LOC_Os01g55500	31977842	GGGAGCCGACGAGTTATACA	GGATGGAAGCAAACAATGCT	60.1	186	86	nucleobase-ascorbate transporter putative expressed
OsISM07820	LOC_Os01g55500	31978008	AGCATTGTTTGCTTCCATCC	AACTGCAAGAAGGAGAGCCC	60.5	181	94	nucleobase-ascorbate transporter putative expressed
OsISM07821	LOC_Os01g55500	31978264	CTTTTTCCGGTACAGCATGG	CGGAGGCGATGAGAAGATAG	60.2	437	95	nucleobase-ascorbate transporter putative expressed
OsISM07822	LOC_Os01g55510	31981033	GTACAAGGCCCTCGACCG	CCCGTGGATCATGTCAAA	60.2	269	83	dynein light chain type 1 domain containing protein expressed

ISM IDs	Rice gene locus IDs	Physical positions (bp) on chromosomes	Forward primers (5'-3')	Reverse primers (5'-3')	Melting tempearature (T˚C)	Expected product size (bp) in PCR	Expected product size (bp) in Q-PCR	Putative Functions
OsISM07823	LOC_Os01g55520	31985999	AAGGACACCGGCGAACAC	TTGGCGAACTTCTCCTTCAT	61.0	158	66	ATROPGEF7/ROPGEF7 putative expressed
OsISM07824	LOC_Os01g55520	31986220	CAACGCCATCACCAATCTT	CTTTCTTCCACCTCCCCTTC	59.8	185	90	ATROPGEF7/ROPGEF7 putative expressed
OsISM07825	LOC_Os01g55520	31986459	AACAAGTCTTGGACAACGGC	CCTGGAGATCTCTCCGCTG	60.6	183	64	ATROPGEF7/ROPGEF7 putative expressed
OsISM07826	LOC_Os01g55520	31986632	GATCTCCAGGCGAACATACC	TCCGATCCTTGAAGTTGTCC	59.8	174	77	ATROPGEF7/ROPGEF7 putative expressed
OsISM07827	LOC_Os01g55520	31987020	ATCCTACCTCGAATCCCTCC	AACTCCACGTCGGTGATGAT	59.9	170	79	ATROPGEF7/ROPGEF7 putative expressed
OsISM07828	LOC_Os01g55520	31987425	CTCGACATCGCCAAGATACA	TCTCCAGCACCCTGGAGTAG	60.1	177	74	ATROPGEF7/ROPGEF7 putative expressed
OsISM07829	LOC_Os01g55570	32013609	GTGCCAAGCACCAGATTTGT	GACATTGCGACTCCTCATCC	60.9	177	46	expressed protein
OsISM07830	LOC_Os01g55570	32013829	GGAGCGTTGTGAAGTATGTCC	GCAATCCCTGCAAATCCTAT	59.3	368	83	expressed protein
OsISM07831	LOC_Os01g55570	32014177	ATAGGATTTGCAGGGATTGC	GGAGAACAGGAAGCTTGTCG	59.5	166	74	expressed protein
OsISM07832	LOC_Os01g55570	32014323	CGACAAGCTTCCTGTTCTCC	CCTGTCTGGCTTGAATAGTAGG	59.0	178	95	expressed protein
OsISM07833	LOC_Os01g55570	32014442	CACTGTTGAACTCTTTGCAGTTG	ACGCGAGATACGGAACTCTT	59.5	536	98	expressed protein
OsISM07834	LOC_Os01g55590	32026288	TCATTGCATCTGCACTGGAT	TCCAGTTCCATTTATTCCTTTGA	60.0	254	99	AMP-binding enzyme putative expressed
OsISM07835	LOC_Os01g55590	32026618	TTTCTGGTGGAGTTGCAGTG	TGGTGCTTAAAATCAGCGAA	59.7	549	97	AMP-binding enzyme putative expressed
OsISM07836	LOC_Os01g55590	32027313	GCTGAGATTGACAGTTCAAAGG	TGTGGACCACTCCTTTTGGT	59.7	249	95	AMP-binding enzyme putative expressed
OsISM07837	LOC_Os01g55590	32027553	TGGTCCACACTCATAAGGGG	CAGTGGGAGACAGTGCAGAA	60.4	265	96	AMP-binding enzyme putative expressed
OsISM07838	LOC_Os01g55590	32027798	TTCTGCACTGTCTCCCACTG	CCAGAATAGAGGGGTGCAAA	60.0	221	68	AMP-binding enzyme putative expressed
OsISM07839	LOC_Os01g55590	32027999	TTTGCACCCCTCTATTCTGG	GGATATGACTCACGCCACCT	60.0	328	87	AMP-binding enzyme putative expressed
OsISM07840	LOC_Os01g55590	32028307	AGGTGGCGTGAGTCATATCC	CCTTGCAATAGCCGTGTGTA	59.9	206	96	AMP-binding enzyme putative expressed

ISM IDs	Rice gene locus IDs	Physical positions (bp) on chromosomes	Forward primers (5'-3')	Reverse primers (5'-3')	Melting tempearature (T˚C)	Expected product size (bp) in PCR	Expected product size (bp) in Q-PCR	Putative Functions
OsISM07841	LOC_Os01g55590	32028542	CTGCATCTTCCTTTGCTGC	CTTCCTCCCACCGTTTCATA	59.8	724	88	AMP-binding enzyme putative expressed
OsISM07842	LOC_Os01g55590	32029280	CTTTTGGAACGATACGGCAT	CGGTTTGCCAACTGTACCTT	60.0	181	88	AMP-binding enzyme putative expressed
OsISM07843	LOC_Os01g55590	32029421	ATTGCATGGTGCACGTAAAG	CAGCCCCATCCTCCATAATA	59.7	174	81	AMP-binding enzyme putative expressed
OsISM07844	LOC_Os01g55590	32029605	TGAAGTTGGCGAGCTTTGTA	TGAAGAACCCACCATCAATAAA	59.5	618	99	AMP-binding enzyme putative expressed
OsISM07845	LOC_Os01g55590	32030210	GGTGGGTTCTTCAAGACTGG	CACCAACTTTCATGATGTCAGC	60.1	211	95	AMP-binding enzyme putative expressed
OsISM07846	LOC_Os01g55590	32030398	TGCTGACATCATGAAAGTTGG	GAACAGCACATTCCAGGACA	59.7	336	93	AMP-binding enzyme putative expressed
OsISM07847	LOC_Os01g55590	32030839	TGGAAGCACTGACAAGTTGG	GAGGAAGCGAATCCCATAAG	59.3	416	82	AMP-binding enzyme putative expressed
OsISM07848	LOC_Os01g55590	32031235	CTTATGGGATTCGCTTCCTC	CCTTCCTGGAACCACAGCTA	59.5	162	91	AMP-binding enzyme putative expressed
OsISM07850	LOC_Os01g55630	32052190	CAGTGCTAACCAGCTTGATCC	TTCTTCGAACTCTCCCTTGC	59.7	217	56	glutelin putative
OsISM07851	LOC_Os01g55630	32052644	TGGTTTGGATGAGACCTTTTG	TCCAAAAAGGTGAAAGGAGC	59.6	276	54	glutelin putative
OsISM07852	LOC_Os01g55630	32052959	ATGCCGGGTTCAAGTTGTC	GATGGAATTCTTTCCCACGA	60.4	190	75	glutelin putative
OsISM07853	LOC_Os01g55640	32056170	GGTACTCGACTGCACCGTCT	GAACAAATGACATGCCCAACT	60.1	217	76	seven in absentia protein family protein putative
OsISM07854	LOC_Os01g55690	32075997	AGAGGCCTTCTACTACCCCA	GGGAAAGTTGGCCCTGTTAT	59.0	175	84	glutelin putative expressed
OsISM07855	LOC_Os01g55690	32076374	ACTGATCTCAACAACGGTGC	AACCTCACGCCTGTATGCTT	59.2	203	97	glutelin putative expressed
OsISM07856	LOC_Os01g55690	32076941	CCCAGAATTTCCCCATTCTT	TGAGCGTTGATGTTCCAAAA	60.2	182	98	glutelin putative expressed
OsISM07857	LOC_Os01g55740	32093630	GCTCGCTCGAGCTCTTCTC	GAGCATCAACACTGCCCC	60.4	217	75	OsRhmbd4 - Putative Rhomboid homologue expressed
OsISM07858	LOC_Os01g55740	32093892	CCTAGCTTCATGAGGAGGCA	AGCAACGTGTGTAACCTCCC	60.3	152	69	OsRhmbd4 - Putative Rhomboid homologue expressed
OsISM07859	LOC_Os01g55740	32094092	GATCGGTCTCTACTTCTTCGG A	CAGATCCAGCAAGTGCTCCT	60.2	1351	93	OsRhmbd4 - Putative Rhomboid homologue expressed

ISM IDs	Rice gene locus IDs	Physical positions (bp) on chromosomes	Forward primers (5'-3')	Reverse primers (5'-3')	Melting tempearature (T°C)	Expected product size (bp) in PCR	Expected product size (bp) in Q-PCR	Putative Functions
OsISM07860	LOC_Os01g55740	32095423	AGGAGCACTTGCTGGATCTG	TTCTTGAATTGTCCCATCCG	60.7	387	90	OsRhmbd4 - Putative Rhomboid homologue expressed
OsISM07861	LOC_Os01g55740	32095773	ATTTTGCAGTTTTATGGCGG	TTGCATTAGCAGCAGCACTT	59.9	915	71	OsRhmbd4 - Putative Rhomboid homologue expressed
OsISM07862	LOC_Os01g55740	32096733	TTTACTTCTTCATTCCTATTCCAGC	CAAATCAGCACCGATCAAAA	59.5	141	60	OsRhmbd4 - Putative Rhomboid homologue expressed
OsISM07863	LOC_Os01g55740	32096834	TTTGACTTTTCAGGGGGCTA	GGGCACCACCTAAATGAGAA	59.8	581	93	OsRhmbd4 - Putative Rhomboid homologue expressed
OsISM07864	LOC_Os01g55780	32129703	AGATCGAGAGGGAGAGGGAG	CACATGTGGAAGTAGCAAAAGAA	59.6	2063	94	glycerophosphoryl diester phosphodiesterase family protein putative expressed
OsISM07865	LOC_Os01g55780	32131866	CAAATGGGGAAATTCCTGAG	AAAATCCGCACCTTCTTCAA	59.5	619	66	glycerophosphoryl diester phosphodiesterase family protein putative expressed
OsISM07866	LOC_Os01g55780	32132612	AATGGTTCAACGCTACTGGC	GGTCTCTGAATGGGTATCGC	59.8	863	91	glycerophosphoryl diester phosphodiesterase family protein putative expressed
OsISM07867	LOC_Os01g55780	32133455	GCGATACCCATTCAGAGACC	GCTTGCATCCAAGGCAATAG	60.1	378	86	glycerophosphoryl diester phosphodiesterase family protein putative expressed
OsISM07868	LOC_Os01g55780	32133822	TGGATGCAAGCAGAACTGTC	CCTCAAACTTCTTCCCACCA	60.0	654	94	glycerophosphoryl diester phosphodiesterase family protein putative expressed
OsISM07869	LOC_Os01g55780	32134621	CCTGATTGACGATTTCTCAGTG	TCATCAGAAGTGATGTCCCAG	59.2	227	67	glycerophosphoryl diester phosphodiesterase family protein putative expressed
OsISM07870	LOC_Os01g55780	32134924	TATACGATGGCACCGACTGA	GTGCAGGAATTGGTTCTCGT	60.1	575	94	glycerophosphoryl diester phosphodiesterase family protein putative expressed
OsISM07871	LOC_Os01g55860	32167215	CAAGGCGTACTTCCTCTCCA	CGCCTGCAAACTCTTCAAAT	60.4	240	47	expressed protein
OsISM07872	LOC_Os01g55860	32167511	CCGATACTCAGAATTCCCTCA	GCCGAACCATATTGGAACAC	59.7	269	94	expressed protein
OsISM07873	LOC_Os01g55860	32167837	ACATGCTTCAGGATGGCTTC	TCATCCATGTTGTCAGGGAG	59.8	492	78	expressed protein
OsISM07874	LOC_Os01g55860	32168393	TGTTCTTGGCCAAAGTATTGC	CCAGTCTTCTCCATGACACG	59.7	193	97	expressed protein
OsISM07875	LOC_Os01g55860	32168657	TCATCAGACTTGGCCTTTTTG	TTCAGCTCATATTCTTCCCGA	60.0	857	95	expressed protein
OsISM07876	LOC_Os01g55860	32169523	TGGAAGCCTCGACTTCAAAT	ACACCCCACTCCAGCAAAT	60.1	196	97	expressed protein
OsISM07877	LOC_Os01g55900	32189581	AAATAGGATCGCATTGCCTC	GTTACGAAATCATCCGCCAT	59.5	991	76	holliday junction resolvase putative expressed

ISM IDs	Rice gene locus IDs	Physical positions (bp) on chromosomes	Forward primers (5'-3')	Reverse primers (5'-3')	Melting tempearature (T˚C)	Expected product size (bp) in PCR	Expected product size (bp) in Q-PCR	Putative Functions
OsISM07878	LOC_Os01g55900	32190552	ATGGCGGATGATTTCGTAAC	ACAAAGCCTGCAAGGGAGTA	59.8	526	51	holliday junction resolvase putative expressed
OsISM07879	LOC_Os01g55900	32191053	CAAAATACTCCCTTGCAGGC	GAGCTCTCCAACAAGAAGCC	59.4	189	99	holliday junction resolvase putative expressed
OsISM07880	LOC_Os01g55900	32191233	GGAGAGCTCTGTAAAACTGGGA	TCAGAGGATGTAAAAGGGCTTC	59.8	243	98	holliday junction resolvase putative expressed
OsISM07881	LOC_Os01g55900	32191512	ATAAATTTGCTGCGGTTTGC	CAGGTGTCACCCTGTTTTCC	60.3	260	95	holliday junction resolvase putative expressed
OsISM07882	LOC_Os01g55910	32206686	AGGAGGAGAAGAGGCAGGAG	CCTATGGGGGTAATGAATGC	59.6	457	75	retrotransposon protein putative unclassified
OsISM07883	LOC_Os01g55910	32208228	TTTTGCATAACCGAGAGGGT	CAGGTATCTGAGGGCGTCTC	59.7	293	87	retrotransposon protein putative unclassified
OsISM07884	LOC_Os01g55940	32220735	CAACATCATCTCCTCCCACC	GCTCATCTTCTATCGTCGGC	60.1	263	93	OsGH3.2 - Probable indole-3-acetic acid-amido synthetase expressed
OsISM07885	LOC_Os01g55940	32221015	CTCTACAGCCTCCTCATGCC	CGTCTCCGACTTGATGAACA	59.9	1303	91	OsGH3.2 - Probable indole-3-acetic acid-amido synthetase expressed
OsISM07886	LOC_Os01g56000	32240603	GGTGTACCTCTCGCTCATCG	GCAGTAGGGCAGTATGAGGC	60.3	1516	97	RNA recognition motif containing protein expressed
OsISM07887	LOC_Os01g56000	32242759	ATGTTGCAGCCAAATGAGTG	AGCCTTTCAAAGATGGATGC	59.5	879	62	RNA recognition motif containing protein expressed
OsISM07888	LOC_Os01g56000	32244922	TAGCCCCGAGTGTTTCTGAC	TGGTCGCTCTGTTGGTACTG	60.1	1230	93	RNA recognition motif containing protein expressed
OsISM07889	LOC_Os01g56000	32246132	CAGTACCAACAGAGCGACCA	ATTACAGGACCACGGTGTGC	60.4	183	98	RNA recognition motif containing protein expressed
OsISM07890	LOC_Os01g56000	32246819	ACATGCAGTTTCTGAACCGA	GGTAATCCGTTCGCTTTGTC	59.4	149	63	RNA recognition motif containing protein expressed
OsISM07891	LOC_Os01g56000	32246948	GACAAAGCGAACGGATTACC	TTCGAACAGGGTAATAGCCG	59.8	1595	91	RNA recognition motif containing protein expressed
OsISM07892	LOC_Os01g56000	32248508	CAGAGAGAGGTCTGTCGGCT	GCGCATTCTCTCTGTTTCAG	59.2	193	90	RNA recognition motif containing protein expressed
OsISM07893	LOC_Os01g56040	32266199	ATCTCCGTGTTACCTTTGCC	CCTCTTTACCGACCCAAACA	59.5	1421	83	zinc finger A20 and AN1 domain-containing stress-associated protein putative
OsISM07896	LOC_Os01g56090	32293346	CCATGTCCGAAGAGGACG	AGAAGATGACCTGTGTCTGGC	59.8	180	92	expressed protein
OsISM07897	LOC_Os01g56090	32293446	AATTTTACCAACCTGTGTGCG	TGGTTGGTATCCTCATCCTTG	59.9	237	96	expressed protein

ISM IDs	Rice gene locus IDs	Physical positions (bp) on chromosomes	Forward primers (5'-3')	Reverse primers (5'-3')	Melting tempearature (T°C)	Expected product size (bp) in PCR	Expected product size (bp) in Q-PCR	Putative Functions
OsISM07898	LOC_Os01g56100	32298468	CTTCCCCTTGTTTGTCAACG	AGCTTGGAACAACACGCTCT	60.3	1552	96	BSD domain containing protein expressed
OsISM07899	LOC_Os01g56100	32300121	GCTGAACTTCTTTCAACGCC	ATCAGGTCCCAGCCTTTTCT	60.0	1156	85	BSD domain containing protein expressed
OsISM07901	LOC_Os01g56120	32307817	GAGGAAGCTGCTGAGCCAC	TCAAGGAGGAAGGAAGAGCA	60.7	1062	68	RNA recognition motif containing protein putative
OsISM07902	LOC_Os01g56120	32308943	CCAGATTTTCCACGGGATTA	GCCAAATGCAGACTCACAAA	59.8	186	89	RNA recognition motif containing protein putative
OsISM07903	LOC_Os01g56120	32309109	TTTGTGAGTCTGCATTTGGC	TATGCACCCAGCCTTAGTCC	60.0	482	50	RNA recognition motif containing protein putative
OsISM07904	LOC_Os01g56120	32309615	CTTCTTGTGCTTGCTAGGGC	TAAGGTCGGTGACCACGATT	60.3	676	93	RNA recognition motif containing protein putative
OsISM07905	LOC_Os01g56120	32310370	ACACTTCCTCTGTGGCCAGT	GAAAGCAAGACGCTAGTGGG	59.9	418	55	RNA recognition motif containing protein putative
OsISM07906	LOC_Os01g56120	32310777	GTCTTGCTTTCCCCGAGAG	GGAAGTGCCTGTCCTTCTTG	59.9	1000	98	RNA recognition motif containing protein putative
OsISM07908	LOC_Os01g56180	32346504	GGCGACGACGTTAGGCTC	TACCACGGCACCATCTGATA	60.9	1619	82	expressed protein
OsISM07909	LOC_Os01g56190	32349810	AGGACTTCCAAGCCTCCG	ACACATCTCATGTATCGCCG	59.9	1276	84	HVT1 putative expressed
OsISM07910	LOC_Os01g56190	32351044	GGCATATCGAAACAAGAGCG	CAGATAGGCATCGTCGTTCC	60.7	264	98	HVT1 putative expressed
OsISM07911	LOC_Os01g56190	32351538	AAGAAGGTGGAGATGCTTGC	CATCCTTGTATGAGGAAATTGGA	59.8	229	98	HVT1 putative expressed
OsISM07912	LOC_Os01g56190	32351759	CAAGGATGCAATTTCTTCGAC	GAACTGGCACCTGTGTTGTTT	59.9	464	90	HVT1 putative expressed
OsISM07913	LOC_Os01g56190	32352156	CATCCCTGTCAGGTGGTTCT	CCCTTACCCCACATATGATCC	60.1	469	93	HVT1 putative expressed
OsISM07914	LOC_Os01g56190	32352640	TGTATGCACTCAACCAAGGC	TCCTCTTTCCGCAGAGATTC	59.6	147	65	HVT1 putative expressed
OsISM07915	LOC_Os01g56190	32352772	TCTGCGGAAAGAGGAGAGTC	CTTCCCTCCTTTTGACTCCA	59.5	574	70	HVT1 putative expressed
OsISM07916	LOC_Os01g56190	32353436	CGCTCATTTGATGATGCAGT	AAAACCTGTCCCTTTCATGG	59.4	875	71	HVT1 putative expressed
OsISM07917	LOC_Os01g56190	32354290	TCCATGAAAGGGACAGGTTT	CAGATGAGGATACAACGGCA	59.5	160	72	HVT1 putative expressed

ISM IDs	Rice gene locus IDs	Physical positions (bp) on chromosomes	Forward primers (5'-3')	Reverse primers (5'-3')	Melting tempearature (T˚C)	Expected product size (bp) in PCR	Expected product size (bp) in Q-PCR	Putative Functions
OsISM07918	LOC_Os01g56190	32354430	TGCCGTTGTATCCTCATCTG	ATTTGAGAACCGCTCAGCAT	59.8	476	66	HVT1 putative expressed
OsISM07919	LOC_Os01g56190	32354919	GCCCATTTATTCAAGTCCCA	ATCGCCTACAGATTGCAAAA	59.1	167	87	HVT1 putative expressed
OsISM07920	LOC_Os01g56190	32355991	CCCTGAAATAAAGAGGATGCC	CAATTCTGCAGTTCGGATCA	59.9	215	72	HVT1 putative expressed
OsISM07921	LOC_Os01g56190	32356673	GCCCAAAGAGGATTTGTTCC	GCCATAAGGACAGCACGAAT	60.5	923	84	HVT1 putative expressed
OsISM07922	LOC_Os01g56190	32358042	AGCGCCTAGCATCAGCTATT	GTTGCTCCAAGATCAGGTGG	60.0	493	71	HVT1 putative expressed
OsISM07923	LOC_Os01g56200	32367808	AGGTTCAACGTCGAGCAAAT	TCGACGAAGTTAAGAAGCCG	60.1	632	90	BTBA2 - Bric-a-Brac Tramtrack Broad Complex BTB domain with Ankyrin repeat region expressed
OsISM07924	LOC_Os01g56200	32369116	CCTTTGTTGGCTGACGATCT	GCATTGCAACCTTTGCTTCT	60.3	301	98	BTBA2 - Bric-a-Brac Tramtrack Broad Complex BTB domain with Ankyrin repeat region expressed
OsISM07925	LOC_Os01g56200	32369516	CACAAAACAAAAGGCTCCGT	CTGCGAGCAGTTAGGGAAGT	59.9	573	84	BTBA2 - Bric-a-Brac Tramtrack Broad Complex BTB domain with Ankyrin repeat region expressed
OsISM07926	LOC_Os01g56250	32393088	GAGAGTTTTGGGTTGCCAAG	TCAGATGCAACAAATGCACA	59.8	205	74	transposon protein putative unclassified
OsISM07927	LOC_Os01g56250	32393343	TCCGACACATCGAAGAGACA	TGATTGTTCCCATCTACCCC	60.0	352	80	transposon protein putative unclassified
OsISM07928	LOC_Os01g56250	32393735	ACCGACAGCTGGTACTGGTT	ACATCTTCCCACTCACCAGG	59.8	164	72	transposon protein putative unclassified
OsISM07929	LOC_Os01g56250	32394513	GTCAAGATAAGGCAGGCAGG	GTCTTTGTGAACTCCCCAGC	59.8	258	80	transposon protein putative unclassified
OsISM07930	LOC_Os01g56270	32410313	GGAGGTCCTGTGTGTTACCG	TCCTTCAGGATGTGCCTTATT	59.5	177	91	transposon protein putative CACTA En/Spm sub-class expressed
OsISM07931	LOC_Os01g56270	32410731	ACTGTCCAGATGTCAACCCA	ATAATCTTTGGGGTGATGCG	59.4	166	86	transposon protein putative CACTA En/Spm sub-class expressed
OsISM07932	LOC_Os01g56270	32410877	CGCATCACCCCAAAGATTAT	TGCATATGCCATTTTTCCTC	59.2	168	98	transposon protein putative CACTA En/Spm sub-class expressed
OsISM07933	LOC_Os01g56280	32414157	ACGGTTCACGACTCCTTTGT	ACCGACACATTGGTAGGAGG	59.7	136	59	transposon protein putative unclassified
OsISM07934	LOC_Os01g56280	32414593	GACCTACGAAGAGGTGGCTG	GGTCCCAACATGGTCCAAT	60.2	195	99	transposon protein putative unclassified
OsISM07935	LOC_Os01g56280	32414965	CCACCGGATCATTTCACCTA	AAGCGCCTTCCTCGAATATC	60.7	637	60	transposon protein putative unclassified

ISM IDs	Rice gene locus IDs	Physical positions (bp) on chromosomes	Forward primers (5'-3')	Reverse primers (5'-3')	Melting tempearature (T°C)	Expected product size (bp) in PCR	Expected product size (bp) in Q-PCR	Putative Functions
OsISM07936	LOC_Os01g56280	32415578	TCCTGATATTCGAGGAAGGC	CCAGTCGCACTGTTGTTCAC	59.8	305	98	transposon protein putative unclassified
OsISM07937	LOC_Os01g56280	32415967	AAGCCTACAACATTGGGTCG	TGTATTGACATCTCCTTGGTGG	59.9	173	86	transposon protein putative unclassified
OsISM07938	LOC_Os01g56290	32424101	ATTGTTGGAGTTGTTCGGGA	CCTCACATGTGCATGACTCC	60.2	506	96	expressed protein
OsISM07939	LOC_Os01g56290	32424595	GCACATGTGAGGAGTATTCAGC	TGCTCATAACCAACATCGTCA	59.9	558	83	expressed protein
OsISM07940	LOC_Os01g56300	32432284	GCGTCTTCTTCCTCCCCC	CAACTGAGCTCGAGTCCCTC	61.1	494	93	expressed protein
OsISM07941	LOC_Os01g56300	32432835	GCTGTACCGGCTCTCTTCTG	CAAAAGTGCTCGCAAGATCA	60.1	1385	81	expressed protein
OsISM07942	LOC_Os01g56300	32434200	TGATCTTGCGAGCACTTTTG	CCAATAACACCAGGATTCCG	60.2	891	67	expressed protein
OsISM07943	LOC_Os01g56300	32435071	CGGAATCCTGGTGTTATTGG	CCAGTTGGGAAACTCAGCAT	60.1	625	91	expressed protein
OsISM07945	LOC_Os01g56310	32439677	CCCTGGAGCCTCTGAACAC	TACCACCATCAGGCAAGTCA	60.5	312	97	radical SAM enzyme putative expressed
OsISM07946	LOC_Os01g56310	32439969	TGACTTGCCTGATGGTGGTA	CCACCAGTATCCCAGGTTTG	60.2	167	63	radical SAM enzyme putative expressed
OsISM07947	LOC_Os01g56310	32440218	CATCGAGACTGTGAGGAGCC	TGCAACTTTTTGCATGCTTC	60.5	1091	93	radical SAM enzyme putative expressed
OsISM07948	LOC_Os01g56310	32441390	TAAGGGCCATTGGTGTTGAT	TCCCTTACTGTCAAATGTCTTTCA	60.1	154	74	radical SAM enzyme putative expressed
OsISM07949	LOC_Os01g56310	32441595	GGGTTTCCGCTATGTTGCTA	AGCTCTCCTGCACGATATGAA	60.0	329	58	radical SAM enzyme putative expressed
OsISM07950	LOC_Os01g56320	32450392	CCGTCCACTAGCATCCTCAT	AATTCATAGGTTGGGCTGGA	59.7	491	53	OsSub4 - Putative Subtilisin homologue expressed
OsISM07951	LOC_Os01g56320	32450986	TGCTGTTGTCATCACTCCTCA	TGTTGCCGTCCTAACTGAGA	59.9	544	92	OsSub4 - Putative Subtilisin homologue expressed
OsISM07952	LOC_Os01g56320	32451793	AACCAGGGGAGTGTTCAATG	TTACCAGCTGCTATGGATGC	59.4	408	91	OsSub4 - Putative Subtilisin homologue expressed
OsISM07953	LOC_Os01g56320	32452235	TTTGGGAATGCAAGTGGAAT	CTACATCAGCAGCAAACCCTC	60.1	169	89	OsSub4 - Putative Subtilisin homologue expressed
OsISM07954	LOC_Os01g56320	32452383	GAGGGTTTGCTGCTGATGTAG	TCCAGGAGGCCTTCTATTTG	59.6	232	99	OsSub4 - Putative Subtilisin homologue expressed

ISM IDs	Rice gene locus IDs	Physical positions (bp) on chromosomes	Forward primers (5'-3')	Reverse primers (5'-3')	Melting tempearature (T°C)	Expected product size (bp) in PCR	Expected product size (bp) in Q-PCR	Putative Functions
OsISM07955	LOC_Os01g56320	32452794	ACTTGGCAACAATCTGACCA	TTTTTCAGTGCATGAGGTGC	59.5	187	97	OsSub4 - Putative Subtilisin homologue expressed
OsISM07956	LOC_Os01g56320	32453207	CCAATGGATATGCCTGGACT	GGTCTCATCTCTCACAAGGGA	59.5	178	91	OsSub4 - Putative Subtilisin homologue expressed
OsISM07957	LOC_Os01g56320	32453568	GGTGCTTGGAGTTCTCTTGG	TGTGGTGCAGCCATACTTGT	60.0	678	87	OsSub4 - Putative Subtilisin homologue expressed
OsISM07958	LOC_Os01g56320	32454452	CTTTGGATCCTGGGCTCATA	CTACTGGAGCAGAGCCGTTT	59.8	406	82	OsSub4 - Putative Subtilisin homologue expressed
OsISM07959	LOC_Os01g56350	32469340	GGTTCCAACCGCAGCATC	AGGAACAGTCTGGGCGGT	61.7	175	97	PTAC12 putative expressed
OsISM07960	LOC_Os01g56350	32469497	ACCGCCCAGACTGTTCCT	CTGCTCGACACACCAGAAAA	60.4	217	93	PTAC12 putative expressed
OsISM07961	LOC_Os01g56350	32470258	TGGAGGAGGAAGTCTGAGGA	CCAGTTTCTGCCATTGCTTT	60.1	984	96	PTAC12 putative expressed
OsISM07962	LOC_Os01g56350	32471274	GAACAGAAGCTGCTCTTGCC	TGCTATTGGCCCTATTTCTTC	59.3	469	96	PTAC12 putative expressed
OsISM07963	LOC_Os01g56350	32471911	CAACGAGATCCTTTGGCAAT	GTAAACAGGATCACCACCCC	59.6	613	70	PTAC12 putative expressed
OsISM07964	LOC_Os01g56350	32472572	GGTCCAGAATTTCATGAGCCT	CGGGCATATAGCTCTTCCTTT	60.1	340	87	PTAC12 putative expressed
OsISM07965	LOC_Os01g56350	32472891	AAAGGAAGAGCTATATGCCCG	GCCATGGGATCTCTGACTTC	59.7	189	88	PTAC12 putative expressed
OsISM07966	LOC_Os01g56350	32473068	AGATCCCATGGCTACTGCTG	GTCAGCATCCTCCTCATCGT	60.2	259	77	PTAC12 putative expressed
OsISM07967	LOC_Os01g56350	32473319	ATGCTGACAAAGTTGTTGCG	CTTCCCTTCAGCATCCTCAG	59.9	327	81	PTAC12 putative expressed
OsISM07968	LOC_Os01g56350	32473675	TACCGGACAGGCTGAAAATC	GCGACAGTTGATCTTTCTTCG	60.0	354	51	PTAC12 putative expressed
OsISM07969	LOC_Os01g56410	32516877	AATTCCATCTCAGTCGGCAG	CTCCGTGGGTTGTTGATCTT	60.1	420	77	retrotransposon protein putative unclassified
OsISM07970	LOC_Os01g56410	32517255	AATAGGGTTGATCTGCACCG	TCTCGACGAAGATGTCGATG	59.9	1666	97	retrotransposon protein putative unclassified
OsISM07971	LOC_Os01g56410	32518912	TCGTCGAGACATGAGGAGTG	CAATGGAATTGGTGGCTCTT	60.0	667	95	retrotransposon protein putative unclassified
OsISM07972	LOC_Os01g56410	32520465	AATTCCATCTCAGTCGGCAG	CTCCGTGGGTTGTTGATCTT	60.1	420	77	retrotransposon protein putative unclassified

ISM IDs	Rice gene locus IDs	Physical positions (bp) on chromosomes	Forward primers (5'-3')	Reverse primers (5'-3')	Melting tempearature (T°C)	Expected product size (bp) in PCR	Expected product size (bp) in Q-PCR	Putative Functions
OsISM07973	LOC_Os01g56470	32542560	CAAGTGCGAGAAGACCGAG	TTTTTGATTCCACCAGTTCAGA	59.6	996	69	mal d 1-associated protein putative expressed
OsISM07974	LOC_Os01g56560	32607920	GCGTGCTCTCCAAGGTCTAC	ATAGAGGTCTACTGCCGCGA	60.0	195	87	cyclase/dehydrase family protein expressed
OsISM07975	LOC_Os01g56560	32608232	GTGGAGATGGAGAAGCCAAA	GAACAGGACCAGGCTTGAAC	59.9	1498	98	cyclase/dehydrase family protein expressed
OsISM07976	LOC_Os01g56560	32609728	TCCTGGAACATGTGACCTTT	CGGGAAACAACTTCCTTGAA	59.0	186	97	cyclase/dehydrase family protein expressed
OsISM07977	LOC_Os01g56580	32615814	GGGAGTGCTGAGAAGATTGG	TATTGCGGAGAATGGCCTAC	59.9	1172	87	CK1 CaseinKinase 1a.3 - CK1 includes the casein kinase 1 kinases expressed
OsISM07978	LOC_Os01g56580	32617037	CAGAAGGGTTTCTGGTGGAA	GGCCATAATTGCAACCCTTA	59.9	187	96	CK1 CaseinKinase 1a.3 - CK1 includes the casein kinase 1 kinases expressed
OsISM07979	LOC_Os01g56580	32617222	CCCTCCATATGAGTGGCAAG	GGCCTTTGTAGTGAACCCAG	60.0	467	72	CK1 CaseinKinase 1a.3 - CK1 includes the casein kinase 1 kinases expressed
OsISM07980	LOC_Os01g56580	32617668	CCTGGGTTCACTACAAAGGC	GCCCAACAGAATTCCAAACA	60.2	170	94	CK1 CaseinKinase 1a.3 - CK1 includes the casein kinase 1 kinases expressed
OsISM07981	LOC_Os01g56580	32617818	TGTTTGGAATTCTGTTGGGC	CAAGCAACCATACTTGGGGT	60.4	140	46	CK1 CaseinKinase 1a.3 - CK1 includes the casein kinase 1 kinases expressed
OsISM07982	LOC_Os01g56580	32617958	CATCGCTGTAGAGGCAATTT	CCAGGTTGGCCAAGTAGAAA	59.1	175	97	CK1 CaseinKinase 1a.3 - CK1 includes the casein kinase 1 kinases expressed
OsISM07983	LOC_Os01g56580	32618115	TCTACTTGGCCAACCTGGAT	TGGCGTTTCCTTCCATTTAG	59.8	266	83	CK1 CaseinKinase 1a.3 - CK1 includes the casein kinase 1 kinases expressed
OsISM07984	LOC_Os01g56580	32618361	CTAAATGGAAGGAAACGCCA	AAATGGGCATGGACACTAGC	60.0	182	98	CK1 CaseinKinase 1a.3 - CK1 includes the casein kinase 1 kinases expressed
OsISM07986	LOC_Os01g56580	32618993	ATGAATTGATTGAGCCCCAG	GCAACCTTCCACGTTTTTGT	60.0	191	79	CK1 CaseinKinase 1a.3 - CK1 includes the casein kinase 1 kinases expressed
OsISM07987	LOC_Os01g56580	32619235	GCCCAGCAAATCAGTGGATA	TGTGTTGATGAAGTCTGGCA	59.9	548	94	CK1 CaseinKinase 1a.3 - CK1 includes the casein kinase 1 kinases expressed
OsISM07988	LOC_Os01g56580	32619867	CTGGGTTCACATCCCAAGTT	CTCCCATTGCTCCATAATCC	59.6	147	72	CK1 CaseinKinase 1a.3 - CK1 includes the casein kinase 1 kinases expressed
OsISM07989	LOC_Os01g56580	32620040	GGGTGCAACAAATGGAAGTT	ATCCATTTGAAGGGAAACGA	59.6	369	97	CK1 CaseinKinase 1a.3 - CK1 includes the casein kinase 1 kinases expressed
OsISM07990	LOC_Os01g56580	32620463	CCGCTGGGGTGTAGTTATGT	GCCGGTGGATACCTTCACTA	59.9	340	90	CK1 CaseinKinase 1a.3 - CK1 includes the casein kinase 1 kinases expressed
OsISM07991	LOC_Os01g56580	32620922	GCTTTTCCAAGCAACCATGT	CTGTCCGGCCATAGCATATC	60.3	216	74	CK1 CaseinKinase 1a.3 - CK1 includes the casein kinase 1 kinases expressed

ISM IDs	Rice gene locus IDs	Physical positions (bp) on chromosomes	Forward primers (5'-3')	Reverse primers (5'-3')	Melting tempearature (T˚C)	Expected product size (bp) in PCR	Expected product size (bp) in Q-PCR	Putative Functions
OsISM07992	LOC_Os01g56590	32623189	CACCCCTCCGTCAAGGAC	TTACAGAAGCATTCTAAGCAAGTG	59.8	734	99	guanine nucleotide exchange family protein putative expressed
OsISM07993	LOC_Os01g56590	32623864	GAACTCATGGGCTCATGCTC	TCCATCGGACTAGATAGAGAACG	60.3	1055	93	guanine nucleotide exchange family protein putative expressed
OsISM07994	LOC_Os01g56590	32625031	GCTGTAGCTTCTTCTGCATTGA	AACAATCTCATCTGTCATCTCTGC	59.8	381	73	guanine nucleotide exchange family protein putative expressed
OsISM07995	LOC_Os01g56590	32625448	CAGTCGCACCTACAACCTGA	CGGACACTGTCAGAAGAACG	59.7	710	96	guanine nucleotide exchange family protein putative expressed
OsISM07996	LOC_Os01g56590	32626128	TGAAAGCAACCGTTCTTCTG	CGGTAGGCTCCACAGAGTTC	59.5	751	63	guanine nucleotide exchange family protein putative expressed
OsISM07997	LOC_Os01g56590	32626896	AATGTTGGCAAGCTTGGTCT	TGAATAAACACGAAGCCACG	59.7	239	85	guanine nucleotide exchange family protein putative expressed
OsISM07998	LOC_Os01g56590	32627142	GGACATTTGCCCTTGACATT	GTAAGGTAACAATGCCCGGA	59.8	431	72	guanine nucleotide exchange family protein putative expressed
OsISM07999	LOC_Os01g56590	32627553	TCCGGGCATTGTTACCTTAC	AAGCGAGCATATCTGATGCC	60.1	137	51	guanine nucleotide exchange family protein putative expressed
OsISM08000	LOC_Os01g56590	32627670	GGCATCAGATATGCTCGCTT	GACTAAACGACGGAAGGCAG	60.1	766	90	guanine nucleotide exchange family protein putative expressed
OsISM08001	LOC_Os01g56590	32628439	CGTTTGGTAGCTCATGTCATC	TCCAGACGAGTAACCTTTACGA	58.6	764	93	guanine nucleotide exchange family protein putative expressed
OsISM08002	LOC_Os01g56590	32629213	TGGCATCAGATACTCGTTCTTG	ATAGCAATCGCAGTGTGTGG	60.0	278	68	guanine nucleotide exchange family protein putative expressed
OsISM08003	LOC_Os01g56590	32629471	CCACACACTGCGATTGCTA	TAGTGGCTACCACCAAAGCA	59.4	1537	99	guanine nucleotide exchange family protein putative expressed
OsISM08004	LOC_Os01g56590	32630984	CACTTGCTTTGGTGGTAGCC	GCTGAACATTCCAGCAACAG	60.1	1305	78	guanine nucleotide exchange family protein putative expressed
OsISM08005	LOC_Os01g56590	32632263	TAGCAGCTGTTGCTGGAATG	CGTTATCCATGCTCCACTCA	59.9	798	61	guanine nucleotide exchange family protein putative expressed
OsISM08006	LOC_Os01g56590	32633149	TGCCACTTTGACTGATGAGG	CCAATTCACATTTTGGCGAT	60.3	138	60	guanine nucleotide exchange family protein putative expressed
OsISM08007	LOC_Os01g56590	32633343	TCATGGCTAACCATCCTTGAC	ATAGCACGGAGAACTCCACA	59.1	389	96	guanine nucleotide exchange family protein putative expressed
OsISM08008	LOC_Os01g56590	32633782	ACCCAAATGAAGGGGAGAAG	TCACGCTGATCCACTAACATC	59.5	733	77	guanine nucleotide exchange family protein putative expressed
OsISM08009	LOC_Os01g56590	32634492	GAGATGTTAGTGGATCAGCGTG	CATTGTGTAACCGATGTGCC	59.8	181	95	guanine nucleotide exchange family protein putative expressed

ISM IDs	Rice gene locus IDs	Physical positions (bp) on chromosomes	Forward primers (5'-3')	Reverse primers (5'-3')	Melting tempearature (T°C)	Expected product size (bp) in PCR	Expected product size (bp) in Q-PCR	Putative Functions
OsISM08010	LOC_Os01g56590	32634667	ACAATGTGCTTGGTCCTTCC	GAACCTGGGTTGAAGCATGT	60.0	161	91	guanine nucleotide exchange family protein putative expressed
OsISM08011	LOC_Os01g56590	32634799	CCATTCACCACATGCTTCAA	TGAGAGTCTTGAAACAGAAGCAG	59.7	337	53	guanine nucleotide exchange family protein putative expressed
OsISM08012	LOC_Os01g56590	32635156	CAGTGACTTCCATATTCTCTCATCTT	TCCCAAATTGGTTCAACTCC	59.5	878	64	guanine nucleotide exchange family protein putative expressed
OsISM08013	LOC_Os01g56590	32636014	GGAGTTGAACCAATTTGGGA	TTGAAAATGATGAGGAGCTGAA	59.8	252	73	guanine nucleotide exchange family protein putative expressed
OsISM08014	LOC_Os01g56590	32636237	GCATCAGTTCAGCTCCTCATC	CACCAATGGAGATAAAACAGCA	60.0	169	93	guanine nucleotide exchange family protein putative expressed
OsISM08015	LOC_Os01g56590	32636433	GATGTTCAAATGGGTGCACTT	TGCTAGGCCAGCTATAGGACA	59.9	161	80	guanine nucleotide exchange family protein putative expressed
OsISM08016	LOC_Os01g56590	32636581	AGCTGGCCTAGCATTCTTCA	ACCTGAAAACCCAAGGAGAT	58.8	152	78	guanine nucleotide exchange family protein putative expressed
OsISM08017	LOC_Os01g56590	32636671	TCTTTTGCCAGGGCTGTTAC	ATGGTTGCCAATCCTTCATT	59.7	1305	96	guanine nucleotide exchange family protein putative expressed
OsISM08018	LOC_Os01g56590	32637955	GAATGAAGGATTGGCAACCA	GTGCCATAAGCTCCGGTTAC	60.2	884	70	guanine nucleotide exchange family protein putative expressed
OsISM08019	LOC_Os01g56590	32638934	AGAAAGCAAATGGCATGGAT	GCGCTTCCTCTTTCACTGTT	59.6	138	58	guanine nucleotide exchange family protein putative expressed
OsISM08020	LOC_Os01g56590	32639153	CTGTGCTTCAAAAACTGGGC	CCATGGGTGCTGAGTGTCT	60.2	314	86	guanine nucleotide exchange family protein putative expressed
OsISM08021	LOC_Os01g56590	32639488	GTGGGAGGATTGTCTCTGGA	TCCTTTCCTTGCCACTCATC	60.1	180	94	guanine nucleotide exchange family protein putative expressed
OsISM08022	LOC_Os01g56590	32639666	GAACTTGGAACTCGAGCAGG	TCATCCCATTGTTTTTGTGC	59.7	282	78	guanine nucleotide exchange family protein putative expressed
OsISM08023	LOC_Os01g56590	32640001	CTTTCCTCCAGCAATTGAGC	CAAGAGCGACCTCCTTGCTA	60.3	192	97	guanine nucleotide exchange family protein putative expressed
OsISM08024	LOC_Os01g56590	32640208	TGCAGACGTTTGTTGGCTC	TGGCGAAGTTTGAAGAACAA	60.2	894	99	guanine nucleotide exchange family protein putative expressed
OsISM08025	LOC_Os01g56590	32641132	AAGCAAGAGGTTCTTCGTGG	CGGCCATGAGCCTTAGATAC	59.6	169	86	guanine nucleotide exchange family protein putative expressed
OsISM08026	LOC_Os01g56590	32641286	TAAGGCTCATGGCCGTTATG	CGTTGCAGAGCAGGGATACT	60.7	724	92	guanine nucleotide exchange family protein putative expressed
OsISM08027	LOC_Os01g56590	32642162	CAGCAAATGGTGCAACACA	CATAATACCCCAGCCACAGC	60.3	193	96	guanine nucleotide exchange family protein putative expressed

ISM IDs	Rice gene locus IDs	Physical positions (bp) on chromosomes	Forward primers (5'-3')	Reverse primers (5'-3')	Melting tempearature (T˚C)	Expected product size (bp) in PCR	Expected product size (bp) in Q-PCR	Putative Functions
OsISM08028	LOC_Os01g56590	32642396	GGAGGCTCCACAGAATGAAA	CCCTTTGGATTGTCTCTCCTT	59.9	1187	91	guanine nucleotide exchange family protein putative expressed
OsISM08029	LOC_Os01g56590	32643846	TGAAATTGCAGAAGGATGCTC	AAGGATCCTGTTCGTGATGC	60.2	255	83	guanine nucleotide exchange family protein putative expressed
OsISM08030	LOC_Os01g56590	32644129	TTGCACTGCAGCAGATTTTC	CATAAGCTTGTCCCCGATGT	60.0	531	78	guanine nucleotide exchange family protein putative expressed
OsISM08031	LOC_Os01g56590	32644694	TCAGTTCTTACGAAGCGGTG	TCCTTACACTAGGCAATGGTCC	59.7	200	89	guanine nucleotide exchange family protein putative expressed
OsISM08032	LOC_Os01g56590	32645029	TGGCTTTGCTACCAACCTTT	CCTAGTTCCGAGGCAATGAG	59.8	625	98	guanine nucleotide exchange family protein putative expressed
OsISM08033	LOC_Os01g56600	32651894	AGTCCAACATCAAGCGCATC	GTTGCAGAGCTCCGTGAAG	60.5	179	96	signal recognition particle 54 kDa protein putative expressed
OsISM08034	LOC_Os01g56600	32652125	TGGTCATGTTCGTTGGTTTG	GTGCTGGCTTAAATCCCTTG	60.1	1098	91	signal recognition particle 54 kDa protein putative expressed
OsISM08035	LOC_Os01g56600	32653271	AGAATGCAACAAAGGCCAAG	GAACCTCTCCACACCCTCAA	60.2	168	87	signal recognition particle 54 kDa protein putative expressed
OsISM08036	LOC_Os01g56600	32653510	AGAAATGCGCCAAGTTTCAG	AAATGCAGCCTGACCGATAC	60.2	167	80	signal recognition particle 54 kDa protein putative expressed
OsISM08037	LOC_Os01g56600	32653749	AAAGGAGGTGGCGCACTTA	CGATGTGTTCTCCTGTTCCA	60.2	559	74	signal recognition particle 54 kDa protein putative expressed
OsISM08038	LOC_Os01g56600	32654288	TGGAACAGGAGAACACATCG	TCTTGTCCATAAACCCCGAC	59.7	173	99	signal recognition particle 54 kDa protein putative expressed
OsISM08039	LOC_Os01g56600	32654552	CCAGAACATCTTGAAAATGGG	TCCTTCTCATGTCCCTTTGG	59.7	233	91	signal recognition particle 54 kDa protein putative expressed
OsISM08040	LOC_Os01g56600	32654803	ACGGTACATGACGATGATGG	GAGCAATCCGAAGAATACGC	59.5	164	93	signal recognition particle 54 kDa protein putative expressed
OsISM08041	LOC_Os01g56630	32668780	GTACCCGATCTCCTCCGAGT	GCACAGGCTAGTAAGCACGA	59.9	621	81	dymeclin putative expressed
OsISM08042	LOC_Os01g56630	32669570	AAATGCAAAGACTGATAATTGGC	CCACGCTTTGTTCTGAGAGA	59.3	220	90	dymeclin putative expressed
OsISM08043	LOC_Os01g56630	32669749	TGATTTGTCATGGTTTCCTGA	GTGAGATGCAAATGGCAAAA	59.3	177	69	dymeclin putative expressed
OsISM08044	LOC_Os01g56630	32669958	TGCTACCTGCACCATGAACT	AAGGATGCGCATTCCAAAT	59.9	204	99	dymeclin putative expressed
OsISM08045	LOC_Os01g56630	32670233	AACGACCACGGATTCCTTTA	CAAAGTTTGCTGCAGTAACAAATAA	59.6	280	97	dymeclin putative expressed

ISM IDs	Rice gene locus IDs	Physical positions (bp) on chromosomes	Forward primers (5'-3')	Reverse primers (5'-3')	Melting tempearature (T˚C)	Expected product size (bp) in PCR	Expected product size (bp) in Q-PCR	Putative Functions
OsISM08046	LOC_Os01g56630	32670688	CAATGTCAAGGATGCACCG	TTTCCTTCAACATCAGCACG	60.5	203	97	dymeclin putative expressed
OsISM08047	LOC_Os01g56630	32670860	CTGCAGATGATCGTGCTGAT	AGCATCTGCATGGATAGCG	60.0	452	94	dymeclin putative expressed
OsISM08048	LOC_Os01g56630	32671319	TGAGAGCTCGGTTCTACTGCT	GGCATCAGCTGTTCACAAAA	59.6	668	97	dymeclin putative expressed
OsISM08049	LOC_Os01g56630	32672018	AAGACCTCCAATCAGATATACATGC	CGCTCGGAAGCACCTAGAAT	60.8	169	98	dymeclin putative expressed
OsISM08050	LOC_Os01g56630	32672202	AGCGCCTTATGCATCAAACT	AAGCTTGTCAGTAAACAAGAACCA	59.6	254	73	dymeclin putative expressed
OsISM08051	LOC_Os01g56630	32672500	CTAGCAAATATGGCCCCTCA	CCTGAAGAATGTTGCATAAAAGC	60.1	192	85	dymeclin putative expressed
OsISM08052	LOC_Os01g56630	32672686	TTCAGGTATACCAAATTAGCCGA	GGACTGCAAAAGTAAACCGC	59.8	169	94	dymeclin putative expressed
OsISM08053	LOC_Os01g56630	32672835	GCGGTTTACTTTTGCAGTCC	AAGCAGTAGTATTCTCCCTATGAGGT	59.5	169	89	dymeclin putative expressed
OsISM08054	LOC_Os01g56630	32673020	CAGGTCGTGTATGCTATCTTGC	TCCAACACCTTCCAGACAAA	59.5	208	99	dymeclin putative expressed
OsISM08055	LOC_Os01g56630	32673237	TAGTCGAATGGACATGCAGC	TGTCAAGCCAATCAATCCG	60.2	221	79	dymeclin putative expressed
OsISM08056	LOC_Os01g56630	32673476	TCAGATGTTTACCCAGTTACGG	AGCGACAGGGTTAGGACAAA	59.4	521	84	dymeclin putative expressed
OsISM08057	LOC_Os01g56630	32673977	TTTGTCCTAACCCTGTCGCT	CCTGGAGGGGAATTCATTTT	59.9	380	84	dymeclin putative expressed
OsISM08058	LOC_Os01g56660	32692716	ATGGGTAATGAGATGGGCAA	ATGGTTCTGTCGAAACCCTG	60.1	161	78	expressed protein
OsISM08059	LOC_Os01g56660	32692853	AGCTCAGGGTTTCGACAGAA	TAGATCCTTGGTGCCATCCT	59.7	197	92	expressed protein
OsISM08060	LOC_Os01g56660	32693157	GAACAGGGGTTCACTTCAGG	CCTTGTTGCTCAATGGTTCA	59.6	433	96	expressed protein
OsISM08062	LOC_Os01g56670	32698958	TCACTTGGTGGCTATGCACT	ACGGCAGCATCAAATCTCTC	59.8	181	84	glycosyl hydrolase family 47 domain contain protein expressed
OsISM08063	LOC_Os01g56670	32699143	TGGGTTGGTAAGAATGTCCG	CTGATAGCAGACCCCCAAGA	60.5	810	86	glycosyl hydrolase family 47 domain contain protein expressed
OsISM08064	LOC_Os01g56670	32699954	TCACCTGATTGCCAGTGATT	CTTTGAGCTAAGTCAGCCGC	59.7	634	88	glycosyl hydrolase family 47 domain contain protein expressed

ISM IDs	Rice gene locus IDs	Physical positions (bp) on chromosomes	Forward primers (5'-3')	Reverse primers (5'-3')	Melting tempearature (T˚C)	Expected product size (bp) in PCR	Expected product size (bp) in Q-PCR	Putative Functions
OsISM08065	LOC_Os01g56670	32700591	GCTACCTGCATTTGACACTCC	GCTTGCCTTGCTTTCATTTT	59.6	1233	86	glycosyl hydrolase family 47 domain contain protein expressed
OsISM08066	LOC_Os01g56670	32701754	CGTTGCAGGTATTCCATTTG	CAGTGTCAAAGTGCCACCAC	59.6	533	98	glycosyl hydrolase family 47 domain contain protein expressed
OsISM08067	LOC_Os01g56670	32702267	GTGGTGGCACTTTGACACTG	CCATATTCCACGCACAGAGTT	60.1	545	96	glycosyl hydrolase family 47 domain contain protein expressed
OsISM08068	LOC_Os01g56670	32702825	AACTTGACCTTGTTGGTGCC	ATGAGTCAATGCTTGTGCCA	60.1	512	85	glycosyl hydrolase family 47 domain contain protein expressed
OsISM08069	LOC_Os01g56670	32703317	TGGCACAAGCATTGACTCAT	CAGCCTTGTAAGCCTCCTGA	60.4	596	99	glycosyl hydrolase family 47 domain contain protein expressed
OsISM08070	LOC_Os01g56670	32703907	AGGCTGCCATGCATTATCTC	AGTGGCCAAACAGTAGCACC	60.2	354	77	glycosyl hydrolase family 47 domain contain protein expressed
OsISM08071	LOC_Os01g56670	32704241	GGTGCTACTGTTTGGCCACT	ATGGCAGGATCAACATCTCC	60.0	166	84	glycosyl hydrolase family 47 domain contain protein expressed
OsISM08072	LOC_Os01g56670	32704442	AATATGGCTTCACCCCAGAA	CTTTCAATCAATTCAGGCCG	60.0	315	92	glycosyl hydrolase family 47 domain contain protein expressed
OsISM08073	LOC_Os01g56670	32704756	GCACATATTGGCTGTTCAAGG	GCACCATATTGAAGGCTTGC	60.6	739	83	glycosyl hydrolase family 47 domain contain protein expressed
OsISM08074	LOC_Os01g56670	32705529	GTAGAAACCCACCAGCAGGA	GGCCTGCAGCTAAATCAAAA	60.2	419	92	glycosyl hydrolase family 47 domain contain protein expressed
OsISM08075	LOC_Os01g56670	32705937	AGCTGCAGGCCCTGATAATA	AGCAGGCAACAGATGACCTT	59.9	161	77	glycosyl hydrolase family 47 domain contain protein expressed
OsISM08076	LOC_Os01g56670	32706235	TCGAACACCTTCCGATCAAT	CCAAGTTTCTGGGCATGAGT	60.3	510	97	glycosyl hydrolase family 47 domain contain protein expressed
OsISM08077	LOC_Os01g56690	32714203	GGCGCATCATCATCAAAAGT	GGCGACGATGGTGTTCTT	60.3	146	76	helix-loop-helix DNA-binding domain containing protein expressed
OsISM08079	LOC_Os01g56710	32721957	CTTCCTCTCCACTGCTCCAC	GGGACCGACGTTGATGATT	60.4	625	71	OsFBDUF6 - F-box and DUF domain containing protein
OsISM08080	LOC_Os01g56730	32737203	TGTTCCTGGAGGTGCTATCC	TGTCAAGATTGTGATATGCGG	59.8	178	96	expressed protein
OsISM08081	LOC_Os01g56730	32737395	GCTGTTACACCAGGCATTGA	TATCAATTTCAGCCTCACGC	59.3	357	82	expressed protein
OsISM08082	LOC_Os01g56730	32737870	CTCGTTCTGAGCTTCCATCC	AATTTGGTAGGCAGCATTGG	60.0	192	95	expressed protein
OsISM08083	LOC_Os01g56730	32738155	TCCTTCCTTGGAGACTTTGAA	CCATCTGCCTCAAATGTGACT	59.5	180	96	expressed protein

ISM IDs	Rice gene locus IDs	Physical positions (bp) on chromosomes	Forward primers (5'-3')	Reverse primers (5'-3')	Melting tempearature (T˚C)	Expected product size (bp) in PCR	Expected product size (bp) in Q-PCR	Putative Functions
OsISM08084	LOC_Os01g56790	32769805	CAGCATGATCCCCTAGCTGT	TTGATGCAACTGGAACTTGG	60.0	161	74	expressed protein
OsISM08085	LOC_Os01g56790	32770077	TTTCAGAGCACAACAGCACA	CCAATGCACTTTTCTGGTGA	59.4	182	94	expressed protein
OsISM08086	LOC_Os01g56790	32770239	TCACCAGAAAAGTGCATTGG	TCAGCTTCATCAACAGCTTCA	59.7	188	97	expressed protein
OsISM08087	LOC_Os01g56790	32770465	TAACACATGAAGCGGCACTC	GCCTGTGGTGATATAGGAGCTT	59.8	171	95	expressed protein
OsISM08088	LOC_Os01g56830	32821451	CACTTTGAATCACGCAATGG	TCCCATGGACGAACAGTACC	60.4	198	69	transposon protein putative unclassified
OsISM08089	LOC_Os01g56830	32822625	TTTAGAAATGTTGTTGTTCCCA	ACCAAAAATCTTTCCCCAGG	58.7	303	73	transposon protein putative unclassified
OsISM08090	LOC_Os01g56870	32843222	GGCAACAAAATTGAAGGCAA	GAAATATTTGGGGGTGGCTT	60.5	1608	98	expressed protein
OsISM08091	LOC_Os01g56870	32844732	TCATGTTGATGCTCTTTCGC	CACTCAAATTGATCCTCATCACA	60.0	420	95	expressed protein
OsISM08092	LOC_Os01g56870	32845119	TGCAGATGTTTGTGATGAGGA	AAGTGTTGACCAAAACCATGC	60.1	198	90	expressed protein
OsISM08093	LOC_Os01g56870	32845393	TCGAAATGTTGTCTCGCTTG	AAAAACCTGTGCATGCAAAATA	59.8	138	63	expressed protein
OsISM08094	LOC_Os01g56870	32845582	GAAAATCTGGCGATCGTAGC	TTTTGTTTGGAAGCAACACTTC	59.6	146	64	expressed protein
OsISM08095	LOC_Os01g56870	32845945	GCTTCGGTACGCCAATTTTA	AATAAGAGGCCCTCCAATCC	59.7	163	53	expressed protein
OsISM08096	LOC_Os01g56870	32846188	CCACGTTCTGTCACGTTTTG	ATGAAGTGTCACCATTGCCA	60.1	185	63	expressed protein
OsISM08097	LOC_Os01g56870	32846370	CATCTGGTGTGCTTGATTGG	GGATGACACCAAAATGGCTT	60.0	195	86	expressed protein
OsISM08098	LOC_Os01g56910	32870423	TTCATTGTCCTTATCAACCCC	GCTATTTGCATCTCCAAGGG	59.2	681	91	expressed protein
OsISM08099	LOC_Os01g56910	32871203	GCAAAACTTTGCATGGGACT	CCCCTTTTCTGAAACCAGAG	59.4	783	98	expressed protein
OsISM08100	LOC_Os01g56910	32872198	ATTGGATCCGATAGACTGGG	GTTGAACCAAGAAGCCGAAA	59.5	241	99	expressed protein
OsISM08101	LOC_Os01g56910	32872512	CTCCCAAGTCCCAAGATTCA	TGGAATCATCGGATGTTGACT	60.2	385	96	expressed protein

ISM IDs	Rice gene locus IDs	Physical positions (bp) on chromosomes	Forward primers (5'-3')	Reverse primers (5'-3')	Melting tempearature (T°C)	Expected product size (bp) in PCR	Expected product size (bp) in Q-PCR	Putative Functions
OsISM08102	LOC_Os01g56910	32872981	CTGCTCCATTGCTCAAGTCA	TGTTCTCCCCAAATTTGCTC	60.1	3447	55	expressed protein
OsISM08103	LOC_Os01g56930	32886285	GTGGTGGACTTCCCCGTG	TATTCTTTTGGTGGGCTTCC	60.7	1897	90	eukaryotic aspartyl protease domain containing protein expressed
OsISM08104	LOC_Os01g56930	32888239	CGGTTGCCCATCTTCAAGT	TGAGCATGGTATTTTGGATGA	60.0	223	86	eukaryotic aspartyl protease domain containing protein expressed
OsISM08105	LOC_Os01g56930	32888609	ATGGGAAATGAGCAAACTGC	TGCCCTGTCTGTCTTTGTCA	60.3	760	91	eukaryotic aspartyl protease domain containing protein expressed
OsISM08106	LOC_Os01g56930	32889503	TGAAATTGTCGAGCCAGGAT	CCATTGACAACGATACTTTCCA	60.2	299	88	eukaryotic aspartyl protease domain containing protein expressed
OsISM08107	LOC_Os01g56930	32889880	AGCATATCTTGCAGATGGGG	AGCGCACAGATGGAGAGACT	60.1	192	72	eukaryotic aspartyl protease domain containing protein expressed
OsISM08108	LOC_Os01g56930	32890049	TGCAGTCTCTCCATCTGTGC	ACACGGTAGGAAACGACGAG	60.2	180	90	eukaryotic aspartyl protease domain containing protein expressed
OsISM08109	LOC_Os01g56930	32890247	GTGTTGCAATGACGGTGAAG	GGTTCCTTTGCCAACCAATA	60.0	213	91	eukaryotic aspartyl protease domain containing protein expressed
OsISM08110	LOC_Os01g56930	32890440	TATTGGTTGGCAAAGGAACC	GCATGTTCGCCAAGTCATAC	59.5	458	93	eukaryotic aspartyl protease domain containing protein expressed
OsISM08111	LOC_Os01g56930	32890898	GCATGGGCTGGACTGACTAC	TCACGTACTGGTTCTTCCCC	60.3	201	73	eukaryotic aspartyl protease domain containing protein expressed
OsISM08112	LOC_Os01g56940	32892483	CTACTCCTGGCTCCACAAGG	CCCTATGCATGCAATACTCAA	59.3	830	93	flap endonuclease putative expressed
OsISM08113	LOC_Os01g56940	32893353	ATTTGACGGAGGTCATCTGC	CGCTCAAGATTTTCCTTTCG	60.0	347	76	flap endonuclease putative expressed
OsISM08114	LOC_Os01g56940	32893771	CACCTAGGATTGCTTTTGAACTG	GCTTCATAAGGCGCAACAAT	60.2	198	74	flap endonuclease putative expressed
OsISM08115	LOC_Os01g56940	32894008	TGATGCCGTTATCACAGAGG	CCTTGTCCAAACTTGTCCATC	59.6	214	82	flap endonuclease putative expressed
OsISM08116	LOC_Os01g56940	32894374	TCCAAAAGCTCAAGGGTCAT	CCAGATCGCCTTTCTGAAGT	59.6	484	99	flap endonuclease putative expressed
OsISM08117	LOC_Os01g56940	32894918	ATTCCCCATGGCAGTAGTGA	GGCAATGCCTTTAGCAACA	60.1	332	73	flap endonuclease putative expressed
OsISM08118	LOC_Os01g56940	32895260	TCGATCCGATTACCAAGGAA	TATCAAAAGCGAGTGCGCTA	60.1	509	61	flap endonuclease putative expressed
OsISM08119	LOC_Os01g56940	32895795	GCTCCTTCAAATGGAAAGAAAA	CTGAACTTCCGTTTTGCCTC	59.8	392	99	flap endonuclease putative expressed

ISM IDs	Rice gene locus IDs	Physical positions (bp) on chromosomes	Forward primers (5'-3')	Reverse primers (5'-3')	Melting tempearature (T˚C)	Expected product size (bp) in PCR	Expected product size (bp) in Q-PCR	Putative Functions
OsISM08120	LOC_Os01g56940	32896468	CTGCTGAGGACCCAGACATT	AAGAAAGAGCCTGTCGGTATTG	60.0	189	80	flap endonuclease putative expressed
OsISM08121	LOC_Os01g56940	32896635	CAATACCGACAGGCTCTTTCTT	AGCTCTGGATGGCTCTGTGT	59.9	212	87	flap endonuclease putative expressed
OsISM08122	LOC_Os01g56940	32897014	TCAACAAAGAAGCTCTGGAGG	TGAGGACTAGTGGGTTGAAACA	59.6	190	91	flap endonuclease putative expressed
OsISM08123	LOC_Os01g56940	32897169	TTTGTGCAGGGAATGTTTCA	ATACCGTCGTGGGAATCAGA	60.2	297	87	flap endonuclease putative expressed
OsISM08124	LOC_Os01g56940	32897586	CTCTCGTGCCAGTGGTCTTC	CCGAATCTCTGTTCTGGAGG	60.4	209	91	flap endonuclease putative expressed
OsISM08125	LOC_Os01g56949	32900186	ATTGCTGCCTCCCTCGAC	ACTTCTCAAGTCATTCGGCG	60.9	1009	94	homeobox-leucine zipper protein ATHB-54 putative expressed
OsISM08126	LOC_Os01g56969	32906538	ATTGCTGCCTCCCTCGAC	ACTTCTCAAGTCATTCGGCG	60.9	1009	94	homeobox-leucine zipper protein ATHB-54 putative expressed
OsISM08128	LOC_Os01g57004	32933452	GAGCCGAGCTTCCTCGAA	GGTCACAAATATGACCGATGG	60.9	198	91	adhesive/proline-rich protein putative
OsISM08129	LOC_Os01g57020	32939171	CTCTTCCGCGTCAACCAC	AAATTGTCCGCCATGTCCT	60.4	207	93	expressed protein
OsISM08130	LOC_Os01g57020	32939328	GTTAAGAGGTGGCTGTCGGA	CCTGCCATATATACCCAGCC	59.8	292	95	expressed protein
OsISM08131	LOC_Os01g57020	32939661	AACGAGTACGTGCTCAAGGG	TGACTGTCCAGTTTCTTTGGC	60.3	192	99	expressed protein
OsISM08133	LOC_Os01g57020	32940434	AGAGGCAGGAAAGACAAGGC	CTTGAACTCCTTCCCGCAC	60.8	540	97	expressed protein
OsISM08135	LOC_Os01g57066	32957928	TCCTGGAATTTTACTTGCGG	GTGAAACCCACCACAACACA	60.2	230	93	transmembrane protein 49 putative expressed
OsISM08136	LOC_Os01g57066	32958151	GTTTCACTAGGCGTGGCTTC	TGAAGAGGGCAATATGAGGG	60.0	860	86	transmembrane protein 49 putative expressed
OsISM08137	LOC_Os01g57066	32959194	AAGCTGTTCTTTGGGGCATT	CAGCTTCTGGTTCACTTCCTG	60.5	679	94	transmembrane protein 49 putative expressed
OsISM08138	LOC_Os01g57066	32959949	CCTTTCCCACTCTCAGCATC	GCAAGGTCAAACAGTGGGTT	59.9	624	76	transmembrane protein 49 putative expressed
OsISM08139	LOC_Os01g57066	32960624	TACTTTGATTGGGAAGGCCA	TTGATTGTTGCACAAGGAGA	59.1	669	71	transmembrane protein 49 putative expressed
OsISM08140	LOC_Os01g57066	32961404	TTATCAACACCAACATCGGC	CAAGCCACACCACAGTGTTC	59.8	221	95	transmembrane protein 49 putative expressed

ISM IDs	Rice gene locus IDs	Physical positions (bp) on chromosomes	Forward primers (5'-3')	Reverse primers (5'-3')	Melting tempearature (T˚C)	Expected product size (bp) in PCR	Expected product size (bp) in Q-PCR	Putative Functions
OsISM08141	LOC_Os01g57073	32964561	CTCATCAGCGACTCCGAAAC	CACGCCAACCTCCATACAC	60.5	188	52	insulin-degrading enzyme putative expressed
OsISM08142	LOC_Os01g57073	32964730	GTGTATGGAGGTTGGCGTG	TTTCTCACTGGCATAAAATAGCA	59.5	1650	95	insulin-degrading enzyme putative expressed
OsISM08143	LOC_Os01g57073	32966382	CCCTGGGGAACAAGATTACA	TCTCCGAACTTGTGTAGGCA	59.6	623	72	insulin-degrading enzyme putative expressed
OsISM08144	LOC_Os01g57073	32967031	GTGGCCAATTTTGAAGAAGC	CTTGCGACATCAATGGCTTA	59.8	210	65	insulin-degrading enzyme putative expressed
OsISM08145	LOC_Os01g57073	32967226	CATTGATGTCGCAAGATGCT	TCCAGCCATCAGACAACAAG	59.8	282	82	insulin-degrading enzyme putative expressed
OsISM08146	LOC_Os01g57073	32967488	CTTGTTGTCTGATGGCTGGA	GGTGATCCTTTGAAGCCAGA	60.0	396	60	insulin-degrading enzyme putative expressed
OsISM08147	LOC_Os01g57073	32967864	TCTGGCTTCAAAGGATCACC	CCAATCCCCTCTCTTTTGGT	60.2	464	84	insulin-degrading enzyme putative expressed
OsISM08148	LOC_Os01g57073	32968367	CAGCAAACCTGATGCACCT	AAACAAGCGCTCAACAAAGC	60.2	149	75	insulin-degrading enzyme putative expressed
OsISM08149	LOC_Os01g57073	32968537	CAGAGAAGCTTCAAATGCCC	CCTTCAGATATCGGGATTGC	59.7	216	81	insulin-degrading enzyme putative expressed
OsISM08150	LOC_Os01g57073	32968849	CGAGGGATCCATCTTCCATA	GCCGACAAATTCATAGCCC	60.1	172	55	insulin-degrading enzyme putative expressed
OsISM08151	LOC_Os01g57073	32969060	AATTAGCATGAGGCTCACCG	CAAGCCAATGATGTCTTCCA	59.9	156	56	insulin-degrading enzyme putative expressed
OsISM08152	LOC_Os01g57073	32969203	CATCATTGGCTTGGTTTTCA	TCCGTTTCATTTATCGCCAC	60.2	690	94	insulin-degrading enzyme putative expressed
OsISM08153	LOC_Os01g57073	32969902	TCAGGACAAAGTCCATCCAA	AACCAGCCATTCTTCTGGTG	59.6	314	80	insulin-degrading enzyme putative expressed
OsISM08154	LOC_Os01g57073	32970227	ACCGTCCAAGTATGCTCCAA	CCTTTGAACTTTTTAGATTCCCA	59.6	497	94	insulin-degrading enzyme putative expressed
OsISM08155	LOC_Os01g57073	32970768	TTGAAAATGTTACTCCTTCCATGA	GGCTTTGGGATACAGAGCTTC	60.1	227	74	insulin-degrading enzyme putative expressed
OsISM08156	LOC_Os01g57073	32971018	TATCGCTCAAAGAAGCGCA	CACAACCGTGAAAGTGGTGT	60.1	183	71	insulin-degrading enzyme putative expressed
OsISM08157	LOC_Os01g57073	32971280	CCCGAGGCAGTCATTTCTAC	AACCAGCAATTTGAGCATCAT	59.6	219	86	insulin-degrading enzyme putative expressed
OsISM08158	LOC_Os01g57073	32971474	GCGTATGATGCTCAAATTGCT	GAATCCGCATTTTGTCATTG	59.6	275	98	insulin-degrading enzyme putative expressed

ISM IDs	Rice gene locus IDs	Physical positions (bp) on chromosomes	Forward primers (5'-3')	Reverse primers (5'-3')	Melting tempearature (T°C)	Expected product size (bp) in PCR	Expected product size (bp) in Q-PCR	Putative Functions
OsISM08159	LOC_Os01g57073	32971790	AAACCGAACAGGTTTTGTGC	TGGCTGAACTTGAAATTTTGG	60.1	731	66	insulin-degrading enzyme putative expressed
OsISM08160	LOC_Os01g57073	32972622	CTCTTGCAAAGTTCATGCCA	TGTTGCCTCATTTGGTTCAA	60.0	194	84	insulin-degrading enzyme putative expressed
OsISM08161	LOC_Os01g57073	32972936	TACCACCAAATTGAGGGCTT	TATTTGAAAGGGCATCGTCC	59.7	1339	89	insulin-degrading enzyme putative expressed
OsISM08162	LOC_Os01g57073	32974330	AACTGTTGAGCAGCTTGGCT	ATAACAACCTCGAGTGCCCA	60.4	203	76	insulin-degrading enzyme putative expressed
OsISM08163	LOC_Os01g57073	32974501	GATTGTGGAGTCTGGGCACT	CAATCCTAGCATCAAGATGTGAA	59.7	160	74	insulin-degrading enzyme putative expressed
OsISM08164	LOC_Os01g57073	32974667	TCTTCAAGATGTTTGAAAGCAA	TTTCGAATCGACAAGTGATTTT	58.2	380	81	insulin-degrading enzyme putative expressed
OsISM08165	LOC_Os01g57073	32975113	CTCAAGTTTGACAGGGTCGAG	GGGGCACCAAGTCTTATATGC	60.3	265	99	insulin-degrading enzyme putative expressed
OsISM08166	LOC_Os01g57082	32987718	CATGAGGCTCACTGATGCTG	TCCACTCATGGATCCCATTT	60.4	199	99	insulin-degrading enzyme putative
OsISM08167	LOC_Os01g57082	32987855	CATCATTGGCTTGGTCTTCA	TCCGTTTCATTTATCGCCAC	60.2	698	94	insulin-degrading enzyme putative
OsISM08168	LOC_Os01g57082	32988549	CGGAATTCCATTATCAGGACA	AACCAGCCATTCTTCTGGTG	59.9	325	93	insulin-degrading enzyme putative
OsISM08169	LOC_Os01g57082	32988882	ATTGCCGTCCAAGTATGCTC	CCTTCAAACTTTTTAGATTCCCA	59.4	453	97	insulin-degrading enzyme putative
OsISM08170	LOC_Os01g57082	32989379	TTGAAAATGTCACTCCTTCCA	GGCTTTGGGATACAGAGCTTC	59.2	227	74	insulin-degrading enzyme putative
OsISM08171	LOC_Os01g57082	32989628	TTTTCACTCAAAGAAGCGCA	CGTGAAAGCGGTGTCTTTCT	60.1	178	66	insulin-degrading enzyme putative
OsISM08172	LOC_Os01g57082	32989890	CCCCGAAGCAGTCATTTCTA	AACCAGCAATTTGAGCATCAT	59.9	184	87	insulin-degrading enzyme putative
OsISM08173	LOC_Os01g57082	32990049	GCGTATGATGCTCAAATTGCT	GAATCCGCATTTTGTCATTG	59.6	246	98	insulin-degrading enzyme putative
OsISM08174	LOC_Os01g57082	32990337	AACCGAACAGATTTTGTGCC	CGGTTGGCTGAACTTGAAAT	60.0	569	69	insulin-degrading enzyme putative
OsISM08175	LOC_Os01g57082	32991032	TCGAAGACATTTTTGGAATGC	TGTAGCATCATTTGGTTCAATG	59.3	165	55	insulin-degrading enzyme putative
OsISM08176	LOC_Os01g57082	32991317	TACCACCAAATTGAGGGCTT	TTGATATTTGAAAGAGCATCATCC	59.5	826	93	insulin-degrading enzyme putative

ISM IDs	Rice gene locus IDs	Physical positions (bp) on chromosomes	Forward primers (5'-3')	Reverse primers (5'-3')	Melting tempearature (T˚C)	Expected product size (bp) in PCR	Expected product size (bp) in Q-PCR	Putative Functions
OsISM08177	LOC_Os01g57082	32992192	CGAACTATTGAGCAGCTTGG	ACAATCTCGAGTGCCCTGAC	59.5	183	75	insulin-degrading enzyme putative
OsISM08178	LOC_Os01g57082	32992355	GTCAGGGCACTCGAGATTGT	GAAGAATTCATCAACTCTAGCATCA	59.6	154	76	insulin-degrading enzyme putative
OsISM08179	LOC_Os01g57082	32992513	TGTTTGAAAACAAAATCCATGA	TTTTGGACTTCTCCAGTTTCG	58.7	356	88	insulin-degrading enzyme putative
OsISM08180	LOC_Os01g57082	32992906	TGAGGCAGGAACTCTCCAGT	CGATGAATTCTTCTTTCTTGAGC	59.8	207	78	insulin-degrading enzyme putative
OsISM08181	LOC_Os01g57130	33021527	TATTTCCCCTCCAAGGTGGT	GGTCCTCGGCCTCAGACT	60.5	196	99	retrotransposon protein putative Ty3-gypsy subclass
OsISM08182	LOC_Os01g57130	33022255	GGTGTTGGACGGGTTAAGTG	TTCGAGCTCTGAGACGTGC	60.4	157	95	retrotransposon protein putative Ty3-gypsy subclass
OsISM08183	LOC_Os01g57160	33033484	TTGTGCTACTTTTTCAACGCA	TGTGATCATGGGTGACGAAT	59.7	420	88	transposon protein putative CACTA En/Spm sub-class
OsISM08184	LOC_Os01g57160	33034520	ATTGGGAGTGGATGGAGTTG	CCCTCCAACATGTTATCTAAAAA	58.5	1552	91	transposon protein putative CACTA En/Spm sub-class
OsISM08185	LOC_Os01g57160	33036009	GGGTATGTTCAATTTTGCAGG	CATCCCCAAGTACGGATACA	58.8	456	84	transposon protein putative CACTA En/Spm sub-class
OsISM08186	LOC_Os01g57160	33037238	CGTCGGGATAAGATGGATGT	TCCAAGAAGTCGATGGGTTC	59.9	178	93	transposon protein putative CACTA En/Spm sub-class
OsISM08187	LOC_Os01g57160	33037560	ACGAGCCTTCTTGCACTTTC	TTCCGTCTTTAGGGTGGATG	59.8	178	96	transposon protein putative CACTA En/Spm sub-class
OsISM08188	LOC_Os01g57160	33037755	CCTCTCGATTACAATCACAAGC	GGTCTGTTCTCCACCCTTGA	59.5	180	91	transposon protein putative CACTA En/Spm sub-class
OsISM08189	LOC_Os01g57160	33037915	TCAAGGGTGGAGAACAGACC	CGCTGCACATTTGTGATACC	60.1	371	98	transposon protein putative CACTA En/Spm sub-class
OsISM08190	LOC_Os01g57210	33049968	GAGAGACGGGAGGATGGAC	GCCGATAGACAAGGACCTCA	59.9	684	89	katanin p80 WD40 repeat-containing subunit B1 homolog 1 putative expressed
OsISM08191	LOC_Os01g57210	33050711	CATTGGCAAACAGACACCAC	GCACAAGCACCTCTGCTGTA	60.1	176	87	katanin p80 WD40 repeat-containing subunit B1 homolog 1 putative expressed
OsISM08192	LOC_Os01g57210	33050910	ATCAAGCTCTGGGACTTGGA	AAAAACTCGCCAAATGGATG	59.9	1067	99	katanin p80 WD40 repeat-containing subunit B1 homolog 1 putative expressed
OsISM08193	LOC_Os01g57210	33052186	GGCAAATCCGGTGTATTGAT	TTCAAAAGTTTCAAGATCCCAAA	59.8	365	96	katanin p80 WD40 repeat-containing subunit B1 homolog 1 putative expressed
OsISM08194	LOC_Os01g57210	33052548	GAATTGATTGGTTCTGCTGGA	TTCCATCTGGGTGAAAGACC	60.0	473	65	katanin p80 WD40 repeat-containing subunit B1 homolog 1 putative expressed

ISM IDs	Rice gene locus IDs	Physical positions (bp) on chromosomes	Forward primers (5'-3')	Reverse primers (5'-3')	Melting tempearature (T˚C)	Expected product size (bp) in PCR	Expected product size (bp) in Q-PCR	Putative Functions
OsISM08195	LOC_Os01g57210	33053006	TTCACCCAGATGGAAAAACC	ATCCCATGTCAACAACGTCA	59.8	176	97	katanin p80 WD40 repeat-containing subunit B1 homolog 1 putative expressed
OsISM08196	LOC_Os01g57210	33053252	TGGTGTTTGGGTTGCTGATA	AAACACCAAGAGCATAGGGC	59.6	119	51	katanin p80 WD40 repeat-containing subunit B1 homolog 1 putative expressed
OsISM08197	LOC_Os01g57210	33053457	CCTGCTCTAGCAACGACACA	ATTTGAGCCACGAATTCCAC	60.1	203	82	katanin p80 WD40 repeat-containing subunit B1 homolog 1 putative expressed
OsISM08198	LOC_Os01g57210	33054284	TCGTTGGAGCATCAACTTCA	AACGTACACACACCTCCTCTGA	60.0	195	99	katanin p80 WD40 repeat-containing subunit B1 homolog 1 putative expressed
OsISM08199	LOC_Os01g57210	33054409	CCTCTGAGCCACCATGTTCT	TTTGTTTTTCCTAATGGCACA	59.2	164	80	katanin p80 WD40 repeat-containing subunit B1 homolog 1 putative expressed
OsISM08200	LOC_Os01g57210	33054643	GGTGACCGTGCAGTGAGAT	GGAATCATCGCCTCATCAAT	59.8	599	99	katanin p80 WD40 repeat-containing subunit B1 homolog 1 putative expressed
OsISM08201	LOC_Os01g57210	33055276	AATGCCGTAAAATCTCGGCT	TTTGGTCAAAGACATGACGC	60.3	668	56	katanin p80 WD40 repeat-containing subunit B1 homolog 1 putative expressed
OsISM08202	LOC_Os01g57210	33055945	TGGTATCAAAGGAGCGATTG	GAGATCAAGTTTCCCCTTCAGA	59.2	185	92	katanin p80 WD40 repeat-containing subunit B1 homolog 1 putative expressed
OsISM08203	LOC_Os01g57210	33056151	AGCTTTCTGCCTGTTCTTGC	CAATTCCAAAGAAACAATGGC	59.6	361	70	katanin p80 WD40 repeat-containing subunit B1 homolog 1 putative expressed
OsISM08204	LOC_Os01g57210	33056560	TCAGCTCATTCAGCAGTTGG	TTAAAACACCGACTGCAACG	60.0	294	69	katanin p80 WD40 repeat-containing subunit B1 homolog 1 putative expressed
OsISM08205	LOC_Os01g57210	33056832	AACGTTGCAGTCGGTGTTTT	GTTCTTGGGCAAGTTGTGCT	60.4	285	97	katanin p80 WD40 repeat-containing subunit B1 homolog 1 putative expressed
OsISM08206	LOC_Os01g57220	33058329	AACCCCTTCGACGAGGAC	TAGAACCCAGGTTCGTGAGG	60.1	959	99	secretory carrier-associated membrane protein putative expressed
OsISM08207	LOC_Os01g57220	33059300	TGCCACAGTGGACATACCTC	TCCTTTGCCTGCAGTTCTTT	59.8	382	70	secretory carrier-associated membrane protein putative expressed
OsISM08208	LOC_Os01g57220	33059662	AAAGAACTGCAGGCAAAGGA	TGGATGCAGCTTCTTCTCTTC	59.9	756	74	secretory carrier-associated membrane protein putative expressed
OsISM08209	LOC_Os01g57220	33060366	TTGTGATTCCCTGATACGCA	AAGAAGGGTGGCCAGTTTTT	60.0	625	93	secretory carrier-associated membrane protein putative expressed
OsISM08210	LOC_Os01g57220	33061029	TCCACCTACAAAGGATGCAA	GTTCCAGAAAAGACATGCCG	59.9	666	69	secretory carrier-associated membrane protein putative expressed
OsISM08211	LOC_Os01g57220	33061675	CGGCATGTCTTTTCTGGAAC	AATGGCTAGCAACCAGATCA	59.5	179	81	secretory carrier-associated membrane protein putative expressed
OsISM08212	LOC_Os01g57220	33061885	ATGTGTTATGGTATCGCCCC	AAAGCACTCTCAGTCCTGCAA	59.9	139	50	secretory carrier-associated membrane protein putative expressed

ISM IDs	Rice gene locus IDs	Physical positions (bp) on chromosomes	Forward primers (5'-3')	Reverse primers (5'-3')	Melting tempearature (T˚C)	Expected product size (bp) in PCR	Expected product size (bp) in Q-PCR	Putative Functions
OsISM08213	LOC_Os01g57220	33062001	TGTTGCAGGACTGAGAGTGC	CAGCAGACCAGATGCAGAAC	59.9	213	83	secretory carrier-associated membrane protein putative expressed
OsISM08214	LOC_Os01g57220	33062195	TTCTGCATCTGGTCTGCTGT	CATCAATAGCCGGCAAAATC	60.0	449	77	secretory carrier-associated membrane protein putative expressed
OsISM08215	LOC_Os01g57220	33062610	TTTTGCCACAGTGGGATTTT	TCAAGGCAGAACAGTCCAAA	59.9	334	96	secretory carrier-associated membrane protein putative expressed
OsISM08216	LOC_Os01g57220	33062924	TTTGGACTGTTCTGCCTTGA	ACGTTTCATCTCAGCAGCCT	59.7	369	97	secretory carrier-associated membrane protein putative expressed
OsISM08217	LOC_Os01g57280	33097129	TAGGGGAGAACGGAGAAGGT	TTCAAACCCACAACCAGTGA	60.0	338	65	rp1 putative expressed
OsISM08218	LOC_Os01g57294	33114283	AATTCGTTACAGGAGCGTGG	AGTGTAGCAACAAACGTGGC	59.5	144	97	hypothetical protein
OsISM08219	LOC_Os01g57324	33134911	AATTCGTTACAGGAGCGTGG	AGTGTAGCAACAAACGTGGC	59.5	144	97	hypothetical protein
OsISM08220	LOC_Os01g57430	33193170	ATTGGCTAATGGCAACCAAG	GAAACCTACAGCGCCAAAAA	60.1	171	96	hypothetical protein
OsISM08221	LOC_Os01g57460	33204417	GACTCGTCCGGTGTCTCTGT	AATTTGTGATACTCATCCTCTGG	58.8	530	91	frataxin putative expressed
OsISM08222	LOC_Os01g57460	33204953	TGAGACAATACATGACCTGCT TG	TGTCAAAGCCATCCATCTGA	60.2	187	69	frataxin putative expressed
OsISM08223	LOC_Os01g57460	33205120	TCAGATGGATGGCTTTGACA	GTCTGTTTGGTGCTTGCTTG	59.8	202	99	frataxin putative expressed
OsISM08224	LOC_Os01g57460	33205305	GCAAGCACCAAACAGACAAA	GCATCCCAATCGAACCTAGA	60.0	993	67	frataxin putative expressed
OsISM08225	LOC_Os01g57480	33211589	TGGCAGCGTCAGAAGTAGAA	AACAGGTGGAATCACACACG	59.6	184	81	serine/threonine-protein kinase receptor precursor putative expressed
OsISM08226	LOC_Os01g57480	33211780	TTTCCTTTTGCTCCCTTTCC	TCAAGGAGTGGTGAGCATGT	59.9	197	85	serine/threonine-protein kinase receptor precursor putative expressed
OsISM08227	LOC_Os01g57480	33212090	CAAAACAAGCTTGGTGAGGG	TGTTGCAAGCCTCTTGACAG	60.4	1941	88	serine/threonine-protein kinase receptor precursor putative expressed
OsISM08228	LOC_Os01g57480	33214159	CATGCCAAATAAAAGCTTGG	TTCGCTTTCTCCAGTCCAAT	59.1	177	72	serine/threonine-protein kinase receptor precursor putative expressed
OsISM08229	LOC_Os01g57480	33214474	TGGCGAGAATATTTGGCTCT	CTTGCATGGCATACTCAGGA	59.8	193	91	serine/threonine-protein kinase receptor precursor putative expressed
OsISM08230	LOC_Os01g57480	33214760	GGCGCTCTCTTAACCTCCTT	CATCTGCCTTCCCTCCATAA	60.0	122	53	serine/threonine-protein kinase receptor precursor putative expressed

ISM IDs	Rice gene locus IDs	Physical positions (bp) on chromosomes	Forward primers (5'-3')	Reverse primers (5'-3')	Melting tempearature (T˚C)	Expected product size (bp) in PCR	Expected product size (bp) in Q-PCR	Putative Functions
OsISM08231	LOC_Os01g57490	33218069	CAATGACGCCAGGACTACG	TATGCTTTGCAGCTTCCCTC	60.4	165	99	D-mannose binding lectin family protein
OsISM08232	LOC_Os01g57490	33218323	GGGTTGGATGGTCTAGTGGA	TTCCATACCACAAGCTGCAC	59.8	225	59	D-mannose binding lectin family protein
OsISM08233	LOC_Os01g57490	33218574	AGTGGTGATGCGGTAGGAAC	AGCTGCAATCAAGACCACTG	59.5	211	85	D-mannose binding lectin family protein
OsISM08234	LOC_Os01g57490	33218826	TTTTCTTTGGATGTGGAGGC	CCTGTCTCCTCACTCTCCCA	60.2	164	85	D-mannose binding lectin family protein
OsISM08235	LOC_Os01g57490	33219069	GGGGGATTTGGTCCTGTCTA	TAACAGCAATCTCCTGCCCT	60.5	556	56	D-mannose binding lectin family protein
OsISM08236	LOC_Os01g57490	33219727	GCTGCATCCAAGGAGAAGAA	AACCCACACTGTATGACCTGC	60.2	833	98	D-mannose binding lectin family protein
OsISM08237	LOC_Os01g57490	33220726	ACAGAAGCAAACACCAACCG	CCGAGAAAATGCCTTCCATA	60.6	178	77	D-mannose binding lectin family protein
OsISM08238	LOC_Os01g57490	33220884	TATGGAAGGCATTTTCTCGG	GGACCACCTTCCTTCTTTCC	60.0	246	95	D-mannose binding lectin family protein
OsISM08239	LOC_Os01g57490	33221110	GGAAAGAAGGAAGGTGGTCC	CTCGGTCATGGTAGGTCGAT	59.9	137	96	D-mannose binding lectin family protein
OsISM08240	LOC_Os01g57490	33221362	ACGGAATGACAATTTCCGAC	CGACACTTGTTGCCTGGAC	60.0	1059	47	D-mannose binding lectin family protein
OsISM08241	LOC_Os01g57500	33223092	ACTGCCATGAAACTTCGCTC	GACGGTGACGATGGAGATG	60.2	1463	69	hypothetical protein
OsISM08242	LOC_Os01g57510	33227890	AGTCCGAGGTAGACGCCTTG	AGGAGAACACCGGAAATGGT	61.2	1251	98	receptor protein kinase putative expressed
OsISM08243	LOC_Os01g57510	33229391	AGCAACAAGATCGGGGAAG	TACTTCTTGCCCATCCTCCA	60.4	197	67	receptor protein kinase putative expressed
OsISM08244	LOC_Os01g57510	33229736	ACATGCACAACCAGAGCCTA	CCCCGACAATGATCTCAAAC	59.8	193	91	receptor protein kinase putative expressed
OsISM08245	LOC_Os01g57510	33230062	TGGAGGTGATCAGACCACTG	GATTTCATGGAGAACACGCC	60.1	179	97	receptor protein kinase putative expressed
OsISM08246	LOC_Os01g57510	33230316	GCTCGATCTCAACCTCCTCA	CTCCTGCCTTCTTTCCACAG	60.2	144	52	receptor protein kinase putative expressed
OsISM08247	LOC_Os01g57540	33241643	ATCGCGAGAATGTTTGAAGG	AACACACCGTCCATTGCATA	60.0	178	99	protein kinase putative expressed
OsISM08248	LOC_Os01g57540	33241857	AGTACTGGAGATTGTCGCCG	CCTTCTTTCCACAACATCCA	59.4	180	97	protein kinase putative expressed

ISM IDs	Rice gene locus IDs	Physical positions (bp) on chromosomes	Forward primers (5'-3')	Reverse primers (5'-3')	Melting tempearature (T˚C)	Expected product size (bp) in PCR	Expected product size (bp) in Q-PCR	Putative Functions
OsISM08249	LOC_Os01g57550	33244101	GGAGGCAGGAAGAGAGAAGG	CGCTTGGGTTGACTGTATCC	60.5	337	84	nodulation protein-related putative expressed
OsISM08250	LOC_Os01g57550	33244587	GAAAAACTTTGCCAGGACCC	GCATGCACATTCTTTCCTAGC	60.4	190	94	nodulation protein-related putative expressed
OsISM08251	LOC_Os01g57550	33244945	AATCTGGACTGGCTCAGCA	TGTTGCACTAGACCCTGCAA	60.0	1427	87	nodulation protein-related putative expressed
OsISM08252	LOC_Os01g57550	33246466	GCAAATGCTCATGATAGTAAGGC	GAGCAAGAATTTGAGCCTGC	60.1	170	86	nodulation protein-related putative expressed
OsISM08253	LOC_Os01g57550	33246698	GCCGCTGATGCTTTACTGT	TCCAGGACATCCATGAGCTT	59.8	196	96	nodulation protein-related putative expressed
OsISM08254	LOC_Os01g57570	33263399	AATGGCGGTGAAGGTCTATG	TGAAGCACCCTTCTTGATCTC	59.7	2171	83	NADPH-dependent FMN reductase domain containing protein expressed
OsISM08255	LOC_Os01g57570	33265545	TGAAGAGATCAAGAAGGGTGC	TTCCTCATGGAGAGTTTCAGG	59.3	855	80	NADPH-dependent FMN reductase domain containing protein expressed
OsISM08256	LOC_Os01g57570	33266616	GGCGGTGGTCAGGAGACTA	ATCTTGGCACCGAAGGTGTA	60.9	206	96	NADPH-dependent FMN reductase domain containing protein expressed
OsISM08257	LOC_Os01g57610	33307838	CTTCTCCGGGAAGCCTATCT	CATGAGCTTCCTCTCCCCTC	60.5	212	68	OsGH3.1 - Probable indole-3-acetic acid-amido synthetase expressed
OsISM08258	LOC_Os01g57610	33308858	ACCTGGTCGACGTAAAGCTC	CCCACGCGATAACGATACA	59.9	189	77	OsGH3.1 - Probable indole-3-acetic acid-amido synthetase expressed
OsISM08259	LOC_Os01g57660	33340891	ATGACGAATATGAGGAGCCG	CAAGGGGAACTACCAACAGC	59.8	86	50	conserved hypothetical protein
OsISM08260	LOC_Os01g57670	33343213	TATCGTCCATTTCCTGGTGC	TCGAAAAGAAGGACCCAATG	60.5	174	85	transposon protein putative CACTA En/Spm sub-class
OsISM08261	LOC_Os01g57670	33343443	TCGCAAAGATTTTCGAAGTG	TCGGGTGAGTTTTTCATTCC	59.5	177	84	transposon protein putative CACTA En/Spm sub-class
OsISM08262	LOC_Os01g57670	33343600	GGAATGAAAAACTCACCCGA	GCATACGTAGTAGCCGCACA	59.9	174	81	transposon protein putative CACTA En/Spm sub-class
OsISM08263	LOC_Os01g57670	33343782	GCACTGCTTAGCACACCAAA	CTGTTGTACCACGGCGAGT	59.9	1840	91	transposon protein putative CACTA En/Spm sub-class
OsISM08265	LOC_Os01g57740	33391915	AGATGGAGTCCTCCTTCGCT	CTTCTCATACACGGCCAACA	60.0	1130	93	expressed protein
OsISM08266	LOC_Os01g57740	33393159	GACCCGAGGAAGATCCTTTT	CTGTCATCCTCCCCATTGAG	60.0	1216	92	expressed protein
OsISM08267	LOC_Os01g57750	33395913	ACTCCTCCTAACCGGGTGTT	GAGGCCGAGTATGAATTTCG	59.8	728	62	hypothetical protein

ISM IDs	Rice gene locus IDs	Physical positions (bp) on chromosomes	Forward primers (5'-3')	Reverse primers (5'-3')	Melting tempearature (T˚C)	Expected product size (bp) in PCR	Expected product size (bp) in Q-PCR	Putative Functions
OsISM08268	LOC_Os01g57770	33408984	GTGTTCCTCTGCGCCTTC	AACTCCGTGTCCATCCAGTC	59.7	388	99	OsPOP4 - Putative Prolyl Oligopeptidase homologue expressed
OsISM08269	LOC_Os01g57770	33409402	CACTTCCATGTTGTTCGGG	CTCACCCTCAGCAGAGATCC	59.9	4647	97	OsPOP4 - Putative Prolyl Oligopeptidase homologue expressed
OsISM08270	LOC_Os01g57780	33416484	GACCAAGCCTTGTCCTTCTG	GTAGCAGCCTCCTCGAATGT	59.7	651	91	retrotransposon protein putative unclassified
OsISM08271	LOC_Os01g57780	33418017	GCGCTTTAATGAGTGCAGAA	ATCGATCGACAACCTCGAAC	59.6	154	87	retrotransposon protein putative unclassified
OsISM08272	LOC_Os01g57780	33418875	CATGGAATCACCCCTCAATC	TAGTGATTGTTCCCTTCGGC	60.1	229	89	retrotransposon protein putative unclassified
OsISM08273	LOC_Os01g57790	33422027	GGATTGGTGGGAGGAGGC	GAACGGTGTGATGAATGCTG	61.5	619	95	retrotransposon protein putative unclassified
OsISM08275	LOC_Os01g57790	33423840	AGCGTGTAGTCCAACTGCCT	GATGTGTCGGTTTCCGACTT	60.0	754	62	retrotransposon protein putative unclassified
OsISM08276	LOC_Os01g57790	33424939	ACTTGTCGAGATCCATTGCC	CATTGGCAGACTTCGTGATG	60.2	544	87	retrotransposon protein putative unclassified
OsISM08277	LOC_Os01g57800	33428022	GATCGGGTTGATTACGCTGT	ACGGAGGGAACTGCTTGAAC	60.8	192	72	retrotransposon protein putative Ty3-gypsy subclass
OsISM08278	LOC_Os01g57800	33428558	AGCCGATCCAACAGACTCC	TGCATGGTGAACCAAATGTT	60.0	155	60	retrotransposon protein putative Ty3-gypsy subclass
OsISM08279	LOC_Os01g57800	33428672	TTGTGAATCAGGTCATCCCA	GACCAATCCCCCTAGCTGAT	60.1	191	91	retrotransposon protein putative Ty3-gypsy subclass
OsISM08280	LOC_Os01g57800	33430171	CATCCATTGAAAACTGCCCT	ACTCTTCCCTTTTGCCAACC	60.2	371	99	retrotransposon protein putative Ty3-gypsy subclass
OsISM08281	LOC_Os01g57800	33431024	TCAATGCTTGATTCAATGGC	CACGACGCCCTCAAAGTAAT	59.9	215	98	retrotransposon protein putative Ty3-gypsy subclass
OsISM08282	LOC_Os01g57800	33432131	TTCTTGTTCATGAAAGGGGG	AACTAAGCCGATGCCACAAC	60.0	745	90	retrotransposon protein putative Ty3-gypsy subclass
OsISM08283	LOC_Os01g57800	33433001	TACCATTGAAATCATGGGGG	AACCAGCCGAAACTCTTTCA	60.1	130	62	retrotransposon protein putative Ty3-gypsy subclass
OsISM08284	LOC_Os01g57830	33442510	TTAGGGTTGCCAAGTCGAAC	TGAGGTGTATCCTGACGTGC	59.9	392	63	transposon protein putative unclassified
OsISM08285	LOC_Os01g57830	33442932	CCAGTCTCACCTTACTGCGG	CCGACACATCGGTAGGAGG	61.2	208	94	transposon protein putative unclassified
OsISM08286	LOC_Os01g57830	33443441	GACCTACGAAGAGGTGGCTG	GCCACAGAACATCACCCAG	60.0	213	57	transposon protein putative unclassified

ISM IDs	Rice gene locus IDs	Physical positions (bp) on chromosomes	Forward primers (5'-3')	Reverse primers (5'-3')	Melting tempearature (T°C)	Expected product size (bp) in PCR	Expected product size (bp) in Q-PCR	Putative Functions
OsISM08287	LOC_Os01g57830	33443944	GATGGGTACTCTGTGGTGCC	TCAGGATACGACCGCCTAAC	60.2	149	67	transposon protein putative unclassified
OsISM08288	LOC_Os01g57830	33444302	AGCCCTTTCCAGTACCGTTT	AGCTGAGTGCTGACCCTTG	59.6	181	75	transposon protein putative unclassified
OsISM08289	LOC_Os01g57830	33444674	AGACCTCTATGCCAGGCACC	CCTAAACGTTGGATCGTCGT	60.8	191	97	transposon protein putative unclassified
OsISM08290	LOC_Os01g57830	33445031	CACGTTCCTAGGCCGACTC	ACAACAGAAGGCCTAGCCG	60.6	161	84	transposon protein putative unclassified
OsISM08291	LOC_Os01g57830	33445128	CGATTGCTTCAGGGTTTCAT	GTCATAGCGAACTGGGCAA	59.9	183	95	transposon protein putative unclassified
OsISM08292	LOC_Os01g57840	33447735	GTCTTCGTCAGTGGCAACCT	AAGAAGCTTCCCTGCTCGTT	60.4	222	96	expressed protein
OsISM08293	LOC_Os01g57854	33453369	ACAACTTTCAACTCCGCCAC	TGTTCTCCACCTTGAGGTCC	60.1	4859	71	pectinesterase putative expressed
OsISM08294	LOC_Os01g57880	33467743	CAACGTCAGCGCCTACAAC	TGCCGGGTTGGTAAGAGA	60.3	805	81	plastocyanin-like domain containing protein putative expressed
OsISM08295	LOC_Os01g57890	33470992	CCTTGATGAACGGTTTTGCT	CTCATTCTCTGCTCCCCATC	59.9	2167	65	Homeobox domain containing protein expressed
OsISM08296	LOC_Os01g57890	33473252	TCTACAGAGGCTCACTGGCA	CTCTTTTGTCCATCATCGGG	60.1	807	82	Homeobox domain containing protein expressed
OsISM08297	LOC_Os01g57890	33474064	TGAGTGAGACAACAGGTCTTG G	AGTTCTCTTCCTTCCAGCACA	59.7	179	94	Homeobox domain containing protein expressed
OsISM08298	LOC_Os01g57890	33474372	GGTTAATGGGGCAGAGTGAG	TGAAAGGCAAGGTTTGCAG	59.8	381	80	Homeobox domain containing protein expressed
OsISM08299	LOC_Os01g57890	33475012	AAAAGCGTTGTGGATTTCCT	GGCCACGTGTAGATCCTGTT	59.3	222	96	Homeobox domain containing protein expressed
OsISM08300	LOC_Os01g57890	33475223	TACACGTGGCCTACCAGTGA	TGGGAACATCATCTCGACAG	59.9	195	67	Homeobox domain containing protein expressed
OsISM08301	LOC_Os01g57890	33475539	AAATGCCATCAGGGTTTCTG	AAGGACGTGCTCTATGGCAG	60.2	176	72	Homeobox domain containing protein expressed
OsISM08302	LOC_Os01g57890	33475809	CTGCACGCATGAGAGATGTT	AGATTCTTCCTCCCCTTCGT	59.6	228	68	Homeobox domain containing protein expressed
OsISM08303	LOC_Os01g57890	33476241	CTTCGATCTGCTCAGGAACC	GATGGTGACCTCCTCCTTCA	60.0	175	83	Homeobox domain containing protein expressed
OsISM08304	LOC_Os01g57890	33476438	ACACTGTCACCGTCCTCCAC	ATCATGGTCCTTCCCCTGTC	61.1	155	59	Homeobox domain containing protein expressed

ISM IDs	Rice gene locus IDs	Physical positions (bp) on chromosomes	Forward primers (5'-3')	Reverse primers (5'-3')	Melting tempearature (T°C)	Expected product size (bp) in PCR	Expected product size (bp) in Q-PCR	Putative Functions
OsISM08305	LOC_Os01g57940	33493906	CCGACCTCGTGAACGACATA	GGTGATCCGTAGAATCCCCT	61.1	188	75	tyrosine protein kinase domain containing protein putative expressed
OsISM08306	LOC_Os01g57940	33494202	AGAGGCCACCTCGAAGAGAT	AGCACCTCCACCAGGAACT	60.0	181	93	tyrosine protein kinase domain containing protein putative expressed
OsISM08307	LOC_Os01g57940	33494437	CGACATGGACCAGAGAATCC	GAGAGGCTTTGCCTTTGGAG	60.9	175	92	tyrosine protein kinase domain containing protein putative expressed
OsISM08308	LOC_Os01g57940	33494950	GACGCACGAGCAGGTTCT	GGTCAGCCAGTCTCACGAAT	60.2	164	75	tyrosine protein kinase domain containing protein putative expressed
OsISM08309	LOC_Os01g57960	33505931	TCTAACAGGCCTTGGAGGAC	GCCATTGGGGTACACTAGAAGA	59.8	226	99	retrotransposon protein putative unclassified expressed
OsISM08310	LOC_Os01g57960	33506401	TGCTTGGCCTAACATGAAGA	TTTGAGGCAACCCATGTAACT	59.5	312	96	retrotransposon protein putative unclassified expressed
OsISM08311	LOC_Os01g57990	33535122	CGTTCTGCCAGACACTGGAC	GGGGCTGATTGCAAAAGATA	61.0	291	81	hypothetical protein
OsISM08312	LOC_Os01g58010	33548902	CTCGCATCTCGGTCACTTTT	AATTCCAGACCGGTTAATGC	59.7	415	95	ATP synthase A subunit family protein putative
OsISM08314	LOC_Os01g58160	33632755	AATGGAGGGAGGCCGAAT	GCCAACACACATGACAGGAG	61.0	532	72	conserved hypothetical protein
OsISM08315	LOC_Os01g58160	33633267	CTCCTGTCATGTGTGTTGGC	TTCATCATCTCCACGTTCTCA	59.7	458	57	conserved hypothetical protein
OsISM08316	LOC_Os01g58160	33633700	ACATTGAGAACGTGGAGATGA	CTTCACCGTCTCGCCATAAT	59.1	514	88	conserved hypothetical protein
OsISM08317	LOC_Os01g58194	33641113	CCTCGCTCATCTCTCCAACT	CCAGTTTGCAGCGAAGTACA	59.8	958	84	protein disulfide isomerase putative expressed
OsISM08318	LOC_Os01g58194	33642458	AGCGGGAAAGACTACGTGAA	TAGAGCCCTACCGTCTTCCC	60.2	6642	75	protein disulfide isomerase putative expressed
OsISM08319	LOC_Os01g58240	33658807	TGGATGCTGTTCATCAGAGC	TGTGTGCATGCTTTCTGTCA	60.0	150	74	OsSub6 - Putative Subtilisin homologue expressed
OsISM08320	LOC_Os01g58240	33658971	GCTTCCCACCATGATACACTC	GCAGCAAATCCTGAGAATCC	59.6	273	99	OsSub6 - Putative Subtilisin homologue expressed
OsISM08321	LOC_Os01g58240	33659228	TCTCAGGATTTGCTGCAATG	GCTTCGCTGCACACTGATTA	60.1	260	81	OsSub6 - Putative Subtilisin homologue expressed
OsISM08322	LOC_Os01g58240	33659532	GCTGAACTACCAAAACCCCA	TTCTCGACTCCGGCCATA	60.1	196	99	OsSub6 - Putative Subtilisin homologue expressed
OsISM08323	LOC_Os01g58240	33659709	ATATGGCCGGAGTCGAGAA	GTTGCTGCCCCATCCTTC	61.1	9397	97	OsSub6 - Putative Subtilisin homologue expressed

ISM IDs	Rice gene locus IDs	Physical positions (bp) on chromosomes	Forward primers (5'-3')	Reverse primers (5'-3')	Melting tempearature (T˚C)	Expected product size (bp) in PCR	Expected product size (bp) in Q-PCR	Putative Functions
OsISM08324	LOC_Os01g58240	33669594	GATCACTCTGGGGGACAAGA	TGAGTTCTTCCCTTCGCTGT	60.0	157	62	OsSub6 - Putative Subtilisin homologue expressed
OsISM08325	LOC_Os01g58240	33669748	TCATCAGGAAGCACCTTCAA	TCTTCCCTTTATGTCCGTGC	59.7	169	82	OsSub6 - Putative Subtilisin homologue expressed
OsISM08326	LOC_Os01g58240	33670064	GCCTGTGTTCTTGTGGACCT	GTGGTTCGATCTTTGCCACT	60.1	154	86	OsSub6 - Putative Subtilisin homologue expressed
OsISM08327	LOC_Os01g58240	33670267	CTCCTCGAGAGGTCCATCAG	CGGCTGCTAAAATGTTGGAT	60.0	186	81	OsSub6 - Putative Subtilisin homologue expressed
OsISM08328	LOC_Os01g58240	33670521	CTGCTGAAAGCTCTCCATCC	AGTATTGGCATGCCTCGTTC	60.1	181	96	OsSub6 - Putative Subtilisin homologue expressed
OsISM08329	LOC_Os01g58270	33673575	ACGAAAGGCCTGAGCCAT	CGTGCTCTTTCCTTTGAAGTG	60.4	216	96	OsSub7 - Putative Subtilisin homologue
OsISM08330	LOC_Os01g58270	33673761	CCCTTTTACCACTTCAAAGGAA	GCATATCATGGTGTGAAGCG	59.8	760	65	OsSub7 - Putative Subtilisin homologue
OsISM08331	LOC_Os01g58270	33674493	GATGTCATCGCTTCACACCA	TGTGGATGATAGAGGCCAAA	59.9	207	77	OsSub7 - Putative Subtilisin homologue
OsISM08332	LOC_Os01g58270	33674726	GCCGTGATGCTTACTGAAGA	GTCGCCGTCGTATACGTTTT	59.5	506	90	OsSub7 - Putative Subtilisin homologue
OsISM08333	LOC_Os01g58270	33675266	AGAATGCCCACTGAACTGCT	TCGCTGAAGCTTCTCGACTC	60.4	207	99	OsSub7 - Putative Subtilisin homologue
OsISM08334	LOC_Os01g58270	33676032	GTGATCACGTTGGGTGACAA	GCTGAGCGATGAGTTCTTCC	60.3	192	73	OsSub7 - Putative Subtilisin homologue
OsISM08335	LOC_Os01g58270	33676225	GTTTCAGACGCCTTGTCGTT	CAGCACAATGCTTCCTTTGA	60.1	1076	84	OsSub7 - Putative Subtilisin homologue
OsISM08336	LOC_Os01g58270	33677429	ATGCAACGGTATTGCGTGT	GGATCGATCTTCACAATGGG	60.1	243	97	OsSub7 - Putative Subtilisin homologue
OsISM08337	LOC_Os01g58270	33677697	ATAATGGCACCAAAAGTGGC	TGAATCCTGGTGCAGCTATG	59.8	200	92	OsSub7 - Putative Subtilisin homologue
OsISM08338	LOC_Os01g58270	33678003	CTGGTCACCTGCTGCACTAA	GCCACGCTCATCAGTTACAG	59.8	152	62	OsSub7 - Putative Subtilisin homologue
OsISM08339	LOC_Os01g58280	33683763	CGACTGTTTTCATCTTGCCTC	CTCACCAAGGTAGGCGATGT	60.0	182	91	OsSub8 - Putative Subtilisin homologue expressed
OsISM08340	LOC_Os01g58280	33683968	TGTGACTGGTTCACACCATGA	GTAGGTGATGGAAGCCTTGG	60.3	204	74	OsSub8 - Putative Subtilisin homologue expressed
OsISM08341	LOC_Os01g58280	33684191	CAGGCTTTGCAGCAATGTTA	AGCTCATGCTTCTGATTTGGA	60.0	377	92	OsSub8 - Putative Subtilisin homologue expressed

ISM IDs	Rice gene locus IDs	Physical positions (bp) on chromosomes	Forward primers (5'-3')	Reverse primers (5'-3')	Melting tempearature (T˚C)	Expected product size (bp) in PCR	Expected product size (bp) in Q-PCR	Putative Functions
OsISM08342	LOC_Os01g58280	33684612	CACCCAGTGAGTTTCTGCAA	AGCTTTTTGATTCAGGCCAA	59.8	216	88	OsSub8 - Putative Subtilisin homologue expressed
OsISM08343	LOC_Os01g58280	33685405	CCTGGGAAACAAGCAAACAT	TGCCACCATTTACAAGTGATTC	59.9	189	93	OsSub8 - Putative Subtilisin homologue expressed
OsISM08344	LOC_Os01g58280	33685517	TTTGCAGGGACAATCACTCT	CATTTAACGCCTCCCTTGAA	59.2	191	99	OsSub8 - Putative Subtilisin homologue expressed
OsISM08345	LOC_Os01g58280	33685871	GGGGATAGCCTGTGTTTTTG	TTGCTGGCTCAATCTTAACAG	58.8	193	96	OsSub8 - Putative Subtilisin homologue expressed
OsISM08346	LOC_Os01g58280	33686106	TATTCTCCTCAAGAGGCCCA	TGCATCTTCTTTGGCAGCTA	59.7	238	96	OsSub8 - Putative Subtilisin homologue expressed
OsISM08347	LOC_Os01g58280	33686421	TCATTGGTCTCATGCTGCTC	GGCGTGTCATATTCGTCCTT	60.0	159	70	OsSub8 - Putative Subtilisin homologue expressed
OsISM08348	LOC_Os01g58290	33690804	GGCTTTTGATGATCAGAGGAA	TTTCTTCTCGCCCAGATAGG	59.3	155	66	OsSub9 - Putative Subtilisin homologue expressed
OsISM08349	LOC_Os01g58290	33690975	TTGTCACTGCCTCACACCAT	GCCTGAGAAGCCATGTTTGT	60.2	268	99	OsSub9 - Putative Subtilisin homologue expressed
OsISM08350	LOC_Os01g58290	33691253	TGCTAACAGAGGAGCAAGCA	CCAACTTCTTGTGGTCAGCA	59.9	1181	96	OsSub9 - Putative Subtilisin homologue expressed
OsISM08351	LOC_Os01g58290	33692474	TGCAGAGGAGCAAATATGGA	AAGCTTCTTGATTCAGGCCA	59.7	176	74	OsSub9 - Putative Subtilisin homologue expressed
OsISM08352	LOC_Os01g58290	33693220	CCTGGGAAACAAGCAAACAT	CCCACTCTTGGATTCGTTGT	60.0	411	68	OsSub9 - Putative Subtilisin homologue expressed
OsISM08353	LOC_Os01g58290	33693616	GAATCCAAGAGTGGGTTCCA	CGATGCACAGAACAATGCTT	59.9	213	98	OsSub9 - Putative Subtilisin homologue expressed
OsISM08354	LOC_Os01g58290	33693953	CCAAGGCATACCCTGTGTTT	GGTTCAATCTTTGCAACCG	59.5	195	94	OsSub9 - Putative Subtilisin homologue expressed
OsISM08355	LOC_Os01g58290	33694205	AAGAGGCCCATCCACTAGGT	TTTGGCTGCTAGGATGTTGA	59.7	206	77	OsSub9 - Putative Subtilisin homologue expressed
OsISM08356	LOC_Os01g58290	33694497	TGATTGGTCTCATGCTGCTC	GGCATGCCATATTCATCCTT	59.9	157	70	OsSub9 - Putative Subtilisin homologue expressed
OsISM08357	LOC_Os01g58320	33702484	GAGGACAGCATACCATTCGG	CCAAACACCAATCCAAATCC	60.3	263	99	expressed protein
OsISM08358	LOC_Os01g58340	33709259	GGGTAGGGGAGGAGGCAG	GCACACACCACACCTTACCA	61.2	203	98	conserved hypothetical protein
OsISM08359	LOC_Os01g58340	33709415	TGCAGTGTGTTTGGGAAGTG	TTGAGTGCTCCAAAATCAGC	59.9	706	79	conserved hypothetical protein

ISM IDs	Rice gene locus IDs	Physical positions (bp) on chromosomes	Forward primers (5'-3')	Reverse primers (5'-3')	Melting tempearature (T°C)	Expected product size (bp) in PCR	Expected product size (bp) in Q-PCR	Putative Functions
OsISM08360	LOC_Os01g58350	33712743	GTATTTGACGGGGAAGTGGA	TGCACTGATTAGGCTAGGGC	60.1	1326	73	conserved hypothetical protein
OsISM08361	LOC_Os01g58380	33729592	ACTCCATCTCCTCCTCCCTC	AATCTGCTCGATGGAATGCT	59.7	171	81	3-hydroxybutyryl-CoA dehydrogenase putative expressed
OsISM08362	LOC_Os01g58380	33729926	TGGCTTCAGCCACTAATCG	TGTATCAGCCCCACGTATGA	60.0	633	99	3-hydroxybutyryl-CoA dehydrogenase putative expressed
OsISM08363	LOC_Os01g58380	33730539	TCATACGTGGGGCTGATACA	CCAGGGTAGTCTTGTGAGCA	59.4	779	98	3-hydroxybutyryl-CoA dehydrogenase putative expressed
OsISM08364	LOC_Os01g58430	33769315	AATTTAGCCCGGACTCCTTC	ATCGTTGAGGCTTTGTTCGT	59.6	380	90	hypothetical protein
OsISM08365	LOC_Os01g58450	33787696	CCAACTATCTGAGGCAGCG	AGACCTGCTCTCCCGATCTC	60.2	792	88	retrotransposon protein putative Ty1-copia subclass
OsISM08366	LOC_Os01g58450	33788521	AGAAGGCGGCAATAAGGTTT	GACGTGTGCCTGGGGTAG	60.1	238	78	retrotransposon protein putative Ty1-copia subclass
OsISM08368	LOC_Os01g58459	33791598	TGTACAGCTGAAGTGGTGCC	GGAGGTTGGATAGCAGCAAC	59.8	752	84	expressed protein
OsISM08369	LOC_Os01g58470	33802487	CCCGAGGACTTGGAGGATA	AGACTGCTTACCTCGCCAAA	60.0	193	90	expressed protein
OsISM08370	LOC_Os01g58470	33802644	GAACAGGGATATGACCTTTGG	TCAGGATCCTCTATGCCGAG	59.3	197	97	expressed protein
OsISM08371	LOC_Os01g58470	33802853	GGATGATTTGGCTGATGCTT	GCAACACGGTTTTCTGGAAT	60.0	559	61	expressed protein
OsISM08372	LOC_Os01g58470	33803405	GTGTTGCCCTTCAGTTGCTT	TGCATAGACAGATTTCCCAGG	60.2	379	99	expressed protein
OsISM08373	LOC_Os01g58470	33803859	TTGGATGATGAAGAGGAGGA A	CCAATAATGACAGGAAAGGCA	59.8	239	96	expressed protein
OsISM08374	LOC_Os01g58480	33809136	GTCGTCTGGCACGAGCAC	AATCCCTCGTGGGAACTAGAA	60.8	712	88	conserved hypothetical protein
OsISM08375	LOC_Os01g58540	33831452	CTCCTCCTTCTCCGGTCATC	CCCGAACTATCGTGGTCG	60.6	637	69	hypothetical protein
OsISM08376	LOC_Os01g58550	33837060	ATGCAGCTTCATCACTCCAT	GAAGGGCAAACTCACTCGTC	59.1	181	97	methyladenine glycosylase putative expressed
OsISM08377	LOC_Os01g58550	33837182	GCCTACCACGACGAGGAGT	GACAACGTGAGCATCTCGAA	60.1	163	69	methyladenine glycosylase putative expressed
OsISM08378	LOC_Os01g58550	33837364	GACTGGACCTCGATCCTGAA	GTGTACTTGGCGACAGCGT	60.1	198	81	methyladenine glycosylase putative expressed

ISM IDs	Rice gene locus IDs	Physical positions (bp) on chromosomes	Forward primers (5'-3')	Reverse primers (5'-3')	Melting tempearature (T˚C)	Expected product size (bp) in PCR	Expected product size (bp) in Q-PCR	Putative Functions
OsISM08379	LOC_Os01g58550	33837603	ATTTGGGCACCATCAGAGG	ACGTATTTGCTGAACGACCC	60.4	1321	83	methyladenine glycosylase putative expressed
OsISM08380	LOC_Os01g58580	33868587	CCCAGCGACATGAACCTTAT	TGTGGCAAGAATCAGAGACG	60.0	1149	98	ICE-like protease p20 domain containing protein putative expressed
OsISM08381	LOC_Os01g58610	33884231	TCAAACACCTCATTGGCAAC	GGGAGTGATACCCTTAGGACG	59.7	685	72	phosphoglycerate kinase protein putative expressed
OsISM08382	LOC_Os01g58610	33884898	CCTAAGGGTATCACTCCCAAA	GGACCAATAACATCATCTGCC	58.7	192	99	phosphoglycerate kinase protein putative expressed
OsISM08383	LOC_Os01g58610	33885173	AGAATGACCCAGAGTTTGCG	CTTGGGGTTTGAAACAGCTC	60.0	353	87	phosphoglycerate kinase protein putative expressed
OsISM08384	LOC_Os01g58610	33885774	TGATAAGTTTGCTCCTGAGGC	TTGTTGTTACACCCTTGCCA	59.7	450	78	phosphoglycerate kinase protein putative expressed
OsISM08385	LOC_Os01g58640	33896709	CATGATCACGCCTTCAAACT	AATTGACACCGTTCAGAGGG	59.3	192	54	nucleotide pyrophosphatase/phosphodiesterase putative expressed
OsISM08386	LOC_Os01g58640	33896930	CCTCTTCTGTGCCACTACCC	CGGGTCGCTTGTCAGGTACT	61.1	3045	49	nucleotide pyrophosphatase/phosphodiesterase putative expressed
OsISM08387	LOC_Os01g58640	33900193	CTCCAGCGTCGACTCCAAG	GTCCCGTACTGAACCTGCTG	61.4	177	82	nucleotide pyrophosphatase/phosphodiesterase putative expressed
OsISM08388	LOC_Os01g58640	33900391	CGCCACGACATTCACTCATA	ATGTAACCCGGATCATGCC	60.6	198	88	nucleotide pyrophosphatase/phosphodiesterase putative expressed
OsISM08389	LOC_Os01g58640	33900610	TCTCCAGCCATCTCAGTCCT	GGGTTCTGAATTCGGTTGTG	60.1	180	78	nucleotide pyrophosphatase/phosphodiesterase putative expressed
OsISM08390	LOC_Os01g58640	33900851	ACTGGACCCATCAGTGGAAC	CTTTCCGGTTTGCATCTCTG	60.3	207	83	nucleotide pyrophosphatase/phosphodiesterase putative expressed
OsISM08391	LOC_Os01g58640	33901163	TACATGACCGCCATTGGTAA	TCGTAGGGGACTCCACATTC	59.9	170	96	nucleotide pyrophosphatase/phosphodiesterase putative expressed
OsISM08392	LOC_Os01g58640	33901395	GCGTTCACTTCGTTGTGATG	CCGATGACAAATCCATTTCC	60.2	184	85	nucleotide pyrophosphatase/phosphodiesterase putative expressed
OsISM08393	LOC_Os01g58640	33901559	GGAAATGGATTTGTCATCGG	AATACCGCTGCTGGATGAAT	59.8	266	89	nucleotide pyrophosphatase/phosphodiesterase putative expressed
OsISM08394	LOC_Os01g58640	33901836	TGATCCGAACTTCGTTTCCT	TGGCCAAAGAAGACCAAATC	59.9	169	70	nucleotide pyrophosphatase/phosphodiesterase putative expressed
OsISM08395	LOC_Os01g58640	33902146	CAACTTGGATGGCTTCCCTA	AGTTTTCGTCGCATGTACCC	60.0	274	92	nucleotide pyrophosphatase/phosphodiesterase putative expressed
OsISM08396	LOC_Os01g58640	33902399	AGGGTACATGCGACGAAAAC	TAAATTGGTCCTGGACTGCC	60.0	702	74	nucleotide pyrophosphatase/phosphodiesterase putative expressed

ISM IDs	Rice gene locus IDs	Physical positions (bp) on chromosomes	Forward primers (5'-3')	Reverse primers (5'-3')	Melting tempearature (T°C)	Expected product size (bp) in PCR	Expected product size (bp) in Q-PCR	Putative Functions
OsISM08397	LOC_Os01g58650	33905598	TTGCATGGACAAGGTTGTGT	TAAGCGAGGGAAGCAGGAAC	60.9	2950	97	LTPL35 - Protease inhibitor/seed storage/LTP family protein precursor putative
OsISM08398	LOC_Os01g58660	33910605	GCAAGAAGCCACTACAGCCT	CAAAGCCATCATCTTCATGC	59.4	191	97	LTPL29 - Protease inhibitor/seed storage/LTP family protein precursor expressed
OsISM08399	LOC_Os01g58670	33913316	CGGGAGGTTAATTTCTGGGT	GCTACACTGACCCTGAAGGC	60.0	135	96	conserved hypothetical protein
OsISM08400	LOC_Os01g58670	33913398	AATTCCTCCTGTGAAATGCG	ATCGAGCAGGTGAAGGAGC	60.3	289	91	conserved hypothetical protein
OsISM08401	LOC_Os01g58670	33914156	AGGAGGTTAATTTCGGGGTG	CGCAAGCAAGCATATCAATC	59.8	2385	76	conserved hypothetical protein
OsISM08402	LOC_Os01g58680	33918492	GGCTCCCTCTTCCGTCTCT	TCAGTGCTAGTTTGCTGGGA	60.2	504	74	apurinic endonuclease-redox protein putative expressed
OsISM08403	LOC_Os01g58680	33919040	CGTCGAGCTGCCTCTAAGAA	ATCTCCAATTTGCTTTCAGC	58.9	179	87	apurinic endonuclease-redox protein putative expressed
OsISM08404	LOC_Os01g58680	33919258	GACAGTGAAGGAGCTCAGGG	CCATCAGGGCTGATACCAAA	60.4	176	84	apurinic endonuclease-redox protein putative expressed
OsISM08405	LOC_Os01g58680	33919416	TGGTATCAGCCCTGATGGAT	TTCTCAACTGATGAAGTTCCCA	60.0	770	53	apurinic endonuclease-redox protein putative expressed
OsISM08406	LOC_Os01g58680	33920315	TGAGGAGAGTTCTGGGGCTA	TCCAAGGTTCTTCATGGTCC	59.9	197	99	apurinic endonuclease-redox protein putative expressed
OsISM08407	LOC_Os01g58680	33920679	GCTCAGAGGGAGGACTTCG	TGGGTATAGCCTTCAAGTAGACC T	59.8	256	99	apurinic endonuclease-redox protein putative expressed
OsISM08408	LOC_Os01g58680	33920967	GCTATTCGGGGACTGCAATA	CGACCCTCCCTTCAGTATCA	60.1	247	91	apurinic endonuclease-redox protein putative expressed
OsISM08409	LOC_Os01g58680	33921212	CGTCACAGTGGAGTTCAATGA T	GATCCCATTCTGTGACCCTG	60.2	185	99	apurinic endonuclease-redox protein putative expressed
OsISM08410	LOC_Os01g58680	33921365	CGTGCAGACTTACAGGGTCA	AACAGGCTTCGATTTTTCCA	59.8	685	77	apurinic endonuclease-redox protein putative expressed
OsISM08411	LOC_Os01g58680	33922070	CTGTGCCCATCAAGAGATTG	TCGAATGATTCCCTTTCTTCA	59.4	383	85	apurinic endonuclease-redox protein putative expressed
OsISM08412	LOC_Os01g58680	33922539	ATAATGCCCGGAAGACCAAC	TGGACTCTTTCTGCAATGGA	60.2	622	74	apurinic endonuclease-redox protein putative expressed
OsISM08413	LOC_Os01g58690	33925572	TTTCGTCTCCAGAGGTTCGT	AGCAACGATTGCAACAACAG	59.9	692	99	endonuclease/exonuclease/phosphatase family domain containing protein expressed
OsISM08414	LOC_Os01g58690	33926499	AACGGGCTTCATGATGTTGT	CATAATGAAAACACAGTACACG ATG	59.5	270	93	endonuclease/exonuclease/phosphatase family domain containing protein expressed

ISM IDs	Rice gene locus IDs	Physical positions (bp) on chromosomes	Forward primers (5'-3')	Reverse primers (5'-3')	Melting tempearature (T˚C)	Expected product size (bp) in PCR	Expected product size (bp) in Q-PCR	Putative Functions
OsISM08415	LOC_Os01g58690	33926773	CAGGAAAAGGATGTTGAAAAGTT	GGCAATGGAAGCACAACATA	59.1	225	99	endonuclease/exonuclease/phosphatase family domain containing protein expressed
OsISM08416	LOC_Os01g58690	33927048	ATGATCATGAGGGCAGGGT	TCTGAAAATCAAAATGCAAGAAA	59.6	193	94	endonuclease/exonuclease/phosphatase family domain containing protein expressed
OsISM08417	LOC_Os01g58690	33927141	TGGTAAGACTGCTTTTCCCAT	TCAGGGATCAAATCTGTTCAA	58.4	270	99	endonuclease/exonuclease/phosphatase family domain containing protein expressed
OsISM08418	LOC_Os01g58690	33927429	AAGCTGATTTATACCTTTTGATCTG	GGGTTCAGAATGAGGATCTGTG	59.1	245	99	endonuclease/exonuclease/phosphatase family domain containing protein expressed
OsISM08419	LOC_Os01g58690	33927696	TGCAGGTTTCACAATTGAAGAG	TTTTCAAAGGGGAGGAAAAA	59.5	612	95	endonuclease/exonuclease/phosphatase family domain containing protein expressed
OsISM08420	LOC_Os01g58710	33936920	AACACTGGCGCTGGAGAG	TGTCGGTGGCACTTTTGTTA	60.1	258	92	retrotransposon protein putative Ty3-gypsy subclass
OsISM08421	LOC_Os01g58710	33937419	AGCCGATGGTTCAATAGCTG	CCGTCACAACACTCTTCCCT	60.2	591	99	retrotransposon protein putative Ty3-gypsy subclass
OsISM08422	LOC_Os01g58710	33938319	GTGGCAATCCATCAGAAACC	GTGAATCCAATCCCTTCCAA	60.0	135	96	retrotransposon protein putative Ty3-gypsy subclass
OsISM08423	LOC_Os01g58710	33938500	GGCAAGGCGACAAAATAGAG	AGACATTTGAACCCTCCACG	59.9	214	97	retrotransposon protein putative Ty3-gypsy subclass
OsISM08424	LOC_Os01g58710	33938721	GGACACGGTAGATGATCTCG	TACTCCCAACCGAAGCAATC	59.1	189	79	retrotransposon protein putative Ty3-gypsy subclass
OsISM08425	LOC_Os01g58710	33939663	ACAAGAGATGATCGGCAAGA	CGTTGGACAGTAGATAATGCCTC	59.2	398	84	retrotransposon protein putative Ty3-gypsy subclass
OsISM08426	LOC_Os01g58790	33970434	AGCAAGGGAGGAGGCAAGT	CCCAGCATTCTGGTCAATTT	60.6	991	84	GHMP kinases ATP-binding protein putative expressed
OsISM08427	LOC_Os01g58790	33971414	AGAATGCTGGGATTACACGG	GTCATGGAACCCATCTGGTC	60.1	190	93	GHMP kinases ATP-binding protein putative expressed
OsISM08428	LOC_Os01g58790	33971595	TTCCATGACCTGGCTTCTCT	GGTTGACAAGCGATCTTTGC	60.3	816	94	GHMP kinases ATP-binding protein putative expressed
OsISM08429	LOC_Os01g58790	33972408	ACCAATGTAGCAGGTGTCCC	GCGGTAAAGATTGAGTGCCT	59.6	169	64	GHMP kinases ATP-binding protein putative expressed
OsISM08430	LOC_Os01g58790	33972546	GCAGATCATCAAGGCACTCA	AAGACCAGCACCAGTAGGGA	59.8	256	98	GHMP kinases ATP-binding protein putative expressed
OsISM08431	LOC_Os01g58790	33972928	TCACAAGGAGCAGCGTATTG	AATTTGCTGGCAATGGATTC	60.0	722	71	GHMP kinases ATP-binding protein putative expressed
OsISM08432	LOC_Os01g58790	33973677	AAGCATGCTCAACAGCTGAA	CCTTGAGCAATACCAAGGGA	59.9	143	79	GHMP kinases ATP-binding protein putative expressed

ISM IDs	Rice gene locus IDs	Physical positions (bp) on chromosomes	Forward primers (5'-3')	Reverse primers (5'-3')	Melting tempearature (T˚C)	Expected product size (bp) in PCR	Expected product size (bp) in Q-PCR	Putative Functions
OsISM08433	LOC_Os01g58790	33973832	ATGGGATATCACAGGATGCC	TGATGGCAACACCTCAAATG	60.1	217	63	GHMP kinases ATP-binding protein putative expressed
OsISM08434	LOC_Os01g58790	33974085	CGGGGAGATTATGATGCTGT	ATCTGGTGAACCAATCCCAA	60.0	516	67	GHMP kinases ATP-binding protein putative expressed
OsISM08435	LOC_Os01g58790	33974599	ATCCGCCTGCATTTGTGTAT	CATTACGAGTGAGGAAGCAGG	60.1	417	76	GHMP kinases ATP-binding protein putative expressed
OsISM08436	LOC_Os01g58890	34032447	GTCACCGAGCACAACAAGAA	TACAAAGTGCCAGCGACAAC	59.9	425	87	cysteine proteinase inhibitor precursor protein putative expressed
OsISM08437	LOC_Os01g58950	34074936	AGCTGCAAGGAGGACGACT	CTCTGGCCTGAACTCCTCAC	60.1	132	94	cytochrome P450 putative expressed
OsISM08438	LOC_Os01g59009	34112257	ACGAACAGCCACATCCTCTC	GTCGTGGCACACCATCATC	60.6	799	78	hypothetical protein
OsISM08439	LOC_Os01g59050	34131637	AAGGACGACTTCCTGTCGC	CGATCCTGTCCTCCACGTC	61.0	165	83	cytochrome P450 putative
OsISM08440	LOC_Os01g59090	34139089	CCAAGGACACTCCTGCACTT	GTGGGTGGCAGAATGAACTC	60.4	192	62	thylakoid lumenal 20 kDa protein putative expressed
OsISM08441	LOC_Os01g59090	34139525	GTCGATACCTCCGTGGAGAA	GGTCAGTGCCAATGGAGTCT	60.1	200	73	thylakoid lumenal 20 kDa protein putative expressed
OsISM08442	LOC_Os01g59100	34141244	GCGCTCTACAACATCCACCT	CAGCCTGGGACATATTGCTC	60.5	1824	93	cytokinin-N-glucosyltransferase 1 putative expressed
OsISM08443	LOC_Os01g59110	34145661	CGTCGTGGCTCGCTAAAC	AAGCCAAGCTTGAGATATAGTG C	59.8	1519	94	indole-3-acetate beta-glucosyltransferase putative expressed
OsISM08444	LOC_Os01g59150	34168210	GATGTTGTGAGGAAGGAGGC	CCAGATCCAGTACCACCACC	59.9	793	84	tubulin/FtsZ domain containing protein putative expressed
OsISM08445	LOC_Os01g59150	34169201	CAGGACTCTCAAGCTGACCA	TCATGGTGGCAGAAATCAAA	59.6	477	63	tubulin/FtsZ domain containing protein putative expressed
OsISM08446	LOC_Os01g59220	34207080	ACTATGTCACGTCTTCCGCC	TCTTCTCTTTTGCCTCTCCC	59.4	1132	55	retrotransposon protein putative unclassified
OsISM08447	LOC_Os01g59220	34208368	CATCTCCGATGTTTGCTTCA	TTGAGCAATCCAAAACACCA	59.9	1808	93	retrotransposon protein putative unclassified
OsISM08448	LOC_Os01g59220	34210381	GAACCTCAATATGATTTTGGA TCA	TCTCACCACTTCTAGCACCAG	58.7	128	53	retrotransposon protein putative unclassified
OsISM08449	LOC_Os01g59220	34210476	TTGTGTTAGGTGCTGGTGCT	TCCACACGTTCTCCTTCCTT	59.5	444	56	retrotransposon protein putative unclassified
OsISM08450	LOC_Os01g59220	34211035	TCTCGCTACAGGTTTGGTCA	CCACGAACAAAATCGAAACC	59.9	348	97	retrotransposon protein putative unclassified

ISM IDs	Rice gene locus IDs	Physical positions (bp) on chromosomes	Forward primers (5'-3')	Reverse primers (5'-3')	Melting tempearature (T°C)	Expected product size (bp) in PCR	Expected product size (bp) in Q-PCR	Putative Functions
OsISM08451	LOC_Os01g59220	34211653	TCGATTGGATCTTGAATTTCCT	AAAATACGACCATCCTCCCC	60.0	585	94	retrotransposon protein putative unclassified
OsISM08452	LOC_Os01g59220	34213552	AGCTTCTCCTCGTGCTTCAC	CTTTGTGCGTGAATGTTGAA	59.0	406	92	retrotransposon protein putative unclassified
OsISM08453	LOC_Os01g59280	34267669	TGAAATTCCAAGGAAATCGG	CCATCAAGAAGCTGAATAGCC	59.4	2868	83	retrotransposon protein putative unclassified
OsISM08456	LOC_Os01g59340	34301230	CGACGTGTACGTGGAGGAC	TTTTCCAGTTGCATCAATCA	58.9	778	92	dihydropyrimidinase putative expressed
OsISM08457	LOC_Os01g59340	34301988	TGATTGATGCAACTGGAAAA	CTCCAGGTGAGTGTGAGGGT	58.9	149	60	dihydropyrimidinase putative expressed
OsISM08458	LOC_Os01g59340	34302338	GATGTTTCGAGGGAAATGGA	CATCCGTGACCATCAATGAA	60.1	298	95	dihydropyrimidinase putative expressed
OsISM08459	LOC_Os01g59340	34302738	TCGATCTTGGGATAACTGGC	GAGCAGTTGCTTCCCCTTC	60.0	258	73	dihydropyrimidinase putative expressed
OsISM08460	LOC_Os01g59340	34303063	AATGGATGAGATTGCCAAGG	GATCCCAAAGCCAAGAATCA	60.0	375	96	dihydropyrimidinase putative expressed
OsISM08461	LOC_Os01g59340	34303416	GATGATTCTTGGCTTTGGGA	TGTCCTGCTTCTCGGATAGG	60.2	152	78	dihydropyrimidinase putative expressed
OsISM08462	LOC_Os01g59340	34303548	CCTATCCGAGAAGCAGGACA	TGGAGTTGAAGGTGCAATGA	60.3	276	95	dihydropyrimidinase putative expressed
OsISM08463	LOC_Os01g59340	34303829	AAAGCTTTCGGCTCTGATGA	CTTACCACCATACTGTCCCAAA	59.5	227	93	dihydropyrimidinase putative expressed
OsISM08464	LOC_Os01g59340	34303990	TCTTAAACGCTTGACCCCAG	TGACAGAAATTCGGCCAGT	59.7	207	81	dihydropyrimidinase putative expressed
OsISM08465	LOC_Os01g59340	34304173	AGGAGACTGGCCGAATTTCT	CAGAATTGCTCCTTTCCGAG	60.1	191	96	dihydropyrimidinase putative expressed
OsISM08466	LOC_Os01g59340	34304452	ATGTGTACGAGGGCAGGAAA	GAACAACGTTCAGAATGCCA	60.1	175	91	dihydropyrimidinase putative expressed
OsISM08467	LOC_Os01g59360	34324953	TTCCTCCTCCTCAGCAAGG	CCGACGATGTCCTTGAACAC	61.0	194	99	CAMK CAMK like.10 - CAMK includes calcium/calmodulin depedent protein kinases expressed
OsISM08468	LOC_Os01g59360	34325225	CCTCTACATCCTCCTCTGCG	CCCTCTCAGGATGGAATTGA	60.0	173	77	CAMK CAMK like.10 - CAMK includes calcium/calmodulin depedent protein kinases expressed
OsISM08469	LOC_Os01g59360	34325460	GAAGATGCTCAACTCCGACC	CAGGTGCTTCTCCATCTTCC	59.8	180	87	CAMK CAMK like.10 - CAMK includes calcium/calmodulin depedent protein kinases expressed
OsISM08470	LOC_Os01g59360	34325660	GTCATGAACAGGCTCAAGCA	CTCTTCCTCCGACAAGCATC	60.0	203	88	CAMK CAMK like.10 - CAMK includes calcium/calmodulin depedent protein kinases expressed

ISM IDs	Rice gene locus IDs	Physical positions (bp) on chromosomes	Forward primers (5'-3')	Reverse primers (5'-3')	Melting tempearature (T°C)	Expected product size (bp) in PCR	Expected product size (bp) in Q-PCR	Putative Functions
OsISM08471	LOC_Os01g59360	34325938	AAAGGGCTGTCCAAGCAAG	GTCGTAATCGATGGTTCCGT	60.1	220	97	CAMK CAMK like.10 - CAMK includes calcium/calmodulin depedent protein kinases expressed
OsISM08472	LOC_Os01g59360	34326200	GAGGAGCATCTTTACACCGC	AAGACCTTTCTCCCTCAGCG	60.4	192	97	CAMK CAMK like.10 - CAMK includes calcium/calmodulin depedent protein kinases expressed
OsISM08473	LOC_Os01g59360	34326382	GAAAGGTCTTCTCGATGGCA	CGCGAACTCGCTGTAGTCTAT	60.0	212	89	CAMK CAMK like.10 - CAMK includes calcium/calmodulin depedent protein kinases expressed
OsISM08474	LOC_Os01g59440	34378349	ACCTGGTTCCACGTCACCT	AACTTTCCAAGCTCAGGCAC	60.0	879	99	BRASSINOSTEROID INSENSITIVE 1-associated receptor kinase 1 precursor putative expressed
OsISM08475	LOC_Os01g59440	34379211	CCTGAGCTTGGAAAGTTGGA	GATGGGATCGTTCCTTGAAT	59.6	442	72	BRASSINOSTEROID INSENSITIVE 1-associated receptor kinase 1 precursor putative expressed
OsISM08476	LOC_Os01g59440	34379731	TGGGAAATTAACGTCCCTTG	TAGATATTCCGGCCAGTTCC	59.4	503	84	BRASSINOSTEROID INSENSITIVE 1-associated receptor kinase 1 precursor putative expressed
OsISM08477	LOC_Os01g59440	34380214	GGAACTGGCCGGAATATCTA	GGGAATGTGCTCAAATGGTC	59.7	176	95	BRASSINOSTEROID INSENSITIVE 1-associated receptor kinase 1 precursor putative expressed
OsISM08478	LOC_Os01g59440	34380370	GACCATTTGAGCACATTCCC	CCAGGCCTTGTAGTTCTGGA	60.3	1329	73	BRASSINOSTEROID INSENSITIVE 1-associated receptor kinase 1 precursor putative expressed
OsISM08479	LOC_Os01g59450	34385721	AAGATTTCAGAACAGGAACCTCC	TCAATCCTCAATATCTTAGCATCA	59.0	167	75	ZOS1-13 - C2H2 zinc finger protein expressed
OsISM08480	LOC_Os01g59450	34385948	TGCCATCATGCCTATTTTGA	CACCACAAGAGGAAGCCTTT	59.7	143	63	ZOS1-13 - C2H2 zinc finger protein expressed
OsISM08481	LOC_Os01g59450	34386071	AAAGGCTTCCTCTTGTGGTG	TGCTGGTCCATCTGAAATTG	59.5	201	94	ZOS1-13 - C2H2 zinc finger protein expressed
OsISM08482	LOC_Os01g59450	34386252	CAATTTCAGATGGACCAGCA	GCTTTCTCGACCTTGGTCCT	60.2	803	81	ZOS1-13 - C2H2 zinc finger protein expressed
OsISM08483	LOC_Os01g59450	34387056	CGGTGCTTTGTTTCAGATCA	GATGTCAATCTGAGCGCTTG	59.7	233	88	ZOS1-13 - C2H2 zinc finger protein expressed
OsISM08484	LOC_Os01g59450	34387772	AAGGAATCAAAAGATTGCTGTCA	TGACATTGGATTCCACACAGA	60.0	482	93	ZOS1-13 - C2H2 zinc finger protein expressed
OsISM08485	LOC_Os01g59450	34388237	TGTGGAATCCAATGTCAAGC	TCTCAAACTTCAATGCAAAATGA	59.6	122	77	ZOS1-13 - C2H2 zinc finger protein expressed
OsISM08486	LOC_Os01g59470	34397387	ATGGCTACCCATGCACTTCT	AGCAGATGCAGCTTCCTTTT	59.4	1460	79	hypothetical protein
OsISM08487	LOC_Os01g59480	34400815	AGCCTATCGTCTCGCCAAG	GTGCCATATTCGTGATGTGC	60.2	181	58	hypothetical protein
OsISM08488	LOC_Os01g59480	34400976	GCACATCACGAATATGGCAC	AGCTGCCAGCCTACAATCTC	59.8	806	58	hypothetical protein

ISM IDs	Rice gene locus IDs	Physical positions (bp) on chromosomes	Forward primers (5'-3')	Reverse primers (5'-3')	Melting tempearature (T˚C)	Expected product size (bp) in PCR	Expected product size (bp) in Q-PCR	Putative Functions
OsISM08489	LOC_Os01g59490	34405580	GCCGGCATCTACTACCCTC	GGGAATTTCTCGTTCTGCAC	59.7	729	94	FAD dependent oxidoreductase domain containing protein expressed
OsISM08490	LOC_Os01g59490	34406503	TCATTCATTCATGCTCTCCCT	CCATTTGTAACCGTTGTGTTG	59.3	448	79	FAD dependent oxidoreductase domain containing protein expressed
OsISM08491	LOC_Os01g59490	34407294	GATGGTGGTAAGGATGTCACG	GAACATCTGGTAGGATTGACTGA	59.2	237	69	FAD dependent oxidoreductase domain containing protein expressed
OsISM08492	LOC_Os01g59490	34407630	GCAGCCTCCTTCAGATTTTG	CGTCAGACCAGGGGATTCTA	60.0	168	92	FAD dependent oxidoreductase domain containing protein expressed
OsISM08493	LOC_Os01g59520	34416483	GTGGCTACTTGACCCCGTC	CTGGATCTGTCCCACGTGTA	60.0	1763	89	cupin RmlC-type putative expressed
OsISM08494	LOC_Os01g59520	34418302	CTGGGGTGCAAAGACAAAAT	ATGGCTTCCCCATATCCTCT	59.9	141	67	cupin RmlC-type putative expressed
OsISM08495	LOC_Os01g59530	34420067	CCGAAGAGCAGATTGGAGAG	ACGGTTCCTAGCTCCTTGGT	60.1	1700	89	OsCML1 - Calmodulin-related calcium sensor protein expressed
OsISM08496	LOC_Os01g59530	34422082	GTTCGTCAAGTGCATGATGG	GTCGTGGTCCCTCTTCTCCT	60.4	190	62	OsCML1 - Calmodulin-related calcium sensor protein expressed
OsISM08497	LOC_Os01g59540	34424936	GGAGGATTCTGACAACGAGC	CCGGTCGGATTTGTAATGAA	60.3	244	86	GRF zinc finger family protein
OsISM08498	LOC_Os01g59540	34425345	AGGTGCCCTAACTATGACCG	GGTTAGGTCGAGAACGGGAT	59.7	169	88	GRF zinc finger family protein
OsISM08499	LOC_Os01g59540	34425719	TGGGTCGAAGGTTCTTTGTT	TGTATTGACATCTCCTTGGTGG	59.7	160	73	GRF zinc finger family protein
OsISM08500	LOC_Os01g59600	34470687	CAGGTTGAGTATGCCACGAA	TGCAGACATTACCAGGACGA	60.0	187	88	peptidase T1 family putative expressed
OsISM08501	LOC_Os01g59600	34470837	CAAATGCAAAGATGGAATCG	TGCACTGAATGGATCCTCCT	59.9	174	91	peptidase T1 family putative expressed
OsISM08502	LOC_Os01g59600	34470966	GAAGATGATGCTGGAGGGAT	AAACAATTTGCCTGCCATCT	59.3	354	99	peptidase T1 family putative expressed
OsISM08503	LOC_Os01g59600	34471319	TCAAGAGCAAAATCGGAAGC	CGTAGCTAGCCACACGATCA	60.3	161	89	peptidase T1 family putative expressed
OsISM08504	LOC_Os01g59600	34471464	CGTGTGGCTAGCTACGTTCA	GGCCCATCCCTATCATAACC	60.2	204	93	peptidase T1 family putative expressed
OsISM08505	LOC_Os01g59600	34471663	GGGCCACAGCTCTACATGAT	TTTTGCAGCCTGTCTTCCTT	60.0	295	85	peptidase T1 family putative expressed
OsISM08506	LOC_Os01g59600	34471923	GTGCTGCTCTGGGTAAAGGA	GGTAAGCTCGGAAAGCTTCA	60.0	857	69	peptidase T1 family putative expressed

ISM IDs	Rice gene locus IDs	Physical positions (bp) on chromosomes	Forward primers (5'-3')	Reverse primers (5'-3')	Melting tempearature (T˚C)	Expected product size (bp) in PCR	Expected product size (bp) in Q-PCR	Putative Functions
OsISM08507	LOC_Os01g59600	34472760	TGAAGCTTTCCGAGCTTACC	TCCAACTCAAAAGCTTTGTCC	59.5	1293	98	peptidase T1 family putative expressed
OsISM08508	LOC_Os01g59600	34474070	ATGAATCAAATCGCCAGCAT	CAGCATCCATCTCCTCAAGC	60.7	172	91	peptidase T1 family putative expressed
OsISM08509	LOC_Os01g59660	34508624	AACCACCTGAGGCCCAAC	TGCAAAGAGTTCAGGATAAATGA	59.9	1317	58	MYB family transcription factor putative expressed
OsISM08510	LOC_Os01g59660	34510895	CAACATCCTGGTCCATTCCT	AGTCCAACCTGGGGAAACTT	59.8	129	57	MYB family transcription factor putative expressed
OsISM08512	LOC_Os01g59670	34516850	ATTTTGTCCCCGAGACAGTG	GAGGTCCTTGTGGTCCTCCT	60.2	140	90	expressed protein
OsISM08513	LOC_Os01g59670	34517266	GCTGACCTGTTTAGCTGGGA	GATGAAACCCAGTCCGAGAA	60.2	299	96	expressed protein
OsISM08514	LOC_Os01g59700	34536121	GGCATGCATCTTAGGGTTGT	GTTGCCCAGTTGGCTTCTTA	60.1	146	86	hypothetical protein
OsISM08515	LOC_Os01g59710	34545178	GATGGCCAGTCTCTCCTACG	AGGTGGCGATCTTCTCTTGA	59.9	211	98	hypothetical protein
OsISM08516	LOC_Os01g59710	34545422	TCAGCGAGATGCTACAGGAA	TTCTTGGTGAACAACGAGACC	59.9	946	99	hypothetical protein
OsISM08517	LOC_Os01g59780	34574015	CCTCCATCTACAAGGGCGT	TCCTTGTCTGGTTTGGGTTC	60.0	209	94	AP2 domain containing protein expressed
OsISM08518	LOC_Os01g59780	34574204	GAACCCAAACCAGACAAGGA	TGTGAACTTTTCCCACGATG	59.7	331	82	AP2 domain containing protein expressed
OsISM08519	LOC_Os01g59780	34574464	GATGTCTCATCGCCTCCATT	TGAGTGCCGCAAGATCATAA	60.2	492	83	AP2 domain containing protein expressed
OsISM08520	LOC_Os01g59780	34574936	TTATGATCTTGCGGCACTCA	TGCATCCTCTCAAGCTCCTT	60.2	203	88	AP2 domain containing protein expressed
OsISM08521	LOC_Os01g59780	34575205	CTCCAAGTACAGGGGAGTGG	CGAATGTTCCCAGGTAGAGG	59.6	395	99	AP2 domain containing protein expressed
OsISM08522	LOC_Os01g59780	34575550	CAGGATAGGCCGAGCTGTAG	CTGTATCGCCGCCAGGTC	61.4	405	95	AP2 domain containing protein expressed
OsISM08523	LOC_Os01g59790	34580103	AGCTCAAGCTTGGCGAGAT	CCCAAACAGTGAAGCTGATG	59.8	646	91	ADP-ribosylation factor putative expressed
OsISM08524	LOC_Os01g59790	34580729	CATCAGCTTCACTGTTTGGG	TGAGTCCCTGTGTGTTCTGG	59.5	261	84	ADP-ribosylation factor putative expressed
OsISM08525	LOC_Os01g59790	34581005	GCAATGATAGGGAGCGTGTT	GCAAATACAAGCAGCACAGC	59.9	436	92	ADP-ribosylation factor putative expressed

ISM IDs	Rice gene locus IDs	Physical positions (bp) on chromosomes	Forward primers (5'-3')	Reverse primers (5'-3')	Melting tempearature (T°C)	Expected product size (bp) in PCR	Expected product size (bp) in Q-PCR	Putative Functions
OsISM08526	LOC_Os01g59790	34581490	AAGCTTGGTTTGCATTCCCT	AAAGCCAGTCAAGACCCTCA	60.4	514	95	ADP-ribosylation factor putative expressed
OsISM08527	LOC_Os01g59790	34581984	TGAGGGTCTTGACTGGCTTT	GATATCCAGCTGCCAAAACG	60.2	278	65	ADP-ribosylation factor putative expressed
OsISM08528	LOC_Os01g59850	34611933	CCAATCGCAAGTGCATCAAT	TCCACTGCATGACAAGCATA	60.4	992	87	GTPase-activating protein putative expressed
OsISM08529	LOC_Os01g59850	34612906	ATGCTTGTCATGCAGTGGAA	TGACTTGACACGGTGGGTAA	60.1	752	50	GTPase-activating protein putative expressed
OsISM08530	LOC_Os01g59850	34613694	TCTCTTGAACAGGGAGGGAA	AACCTCATTCGTTGCCAATC	59.9	158	72	GTPase-activating protein putative expressed
OsISM08531	LOC_Os01g59850	34613839	AACGAATGAGGTTGCCTGAC	GTGGACTTCCCACCAGCATA	60.5	177	95	GTPase-activating protein putative expressed
OsISM08532	LOC_Os01g59850	34614013	CACGGACAAACCAGTCAATG	AAGGTGGGCTTTGGGAATAC	60.1	254	96	GTPase-activating protein putative expressed
OsISM08533	LOC_Os01g59850	34614556	TGAAAATCCTCCATCCCATC	CCAAAACTTCCTGAAGAACCTG	59.9	210	97	GTPase-activating protein putative expressed
OsISM08534	LOC_Os01g59850	34615720	TGAAGAGGTGAAGACCCCTG	TGTTCCCAGTAGATGCACCA	60.2	163	72	GTPase-activating protein putative expressed
OsISM08535	LOC_Os01g59850	34615956	ACATGCTGAAGCCAGAGGTC	TCCCCAGGGGTTAGTCTTTC	60.4	137	64	GTPase-activating protein putative expressed
OsISM08536	LOC_Os01g59850	34616139	TCATTTCGTGGACCACTTCA	TGTTGATTTTGCAGGCTGAG	60.0	574	61	GTPase-activating protein putative expressed
OsISM08537	LOC_Os01g59850	34616693	CTCAGCCTGCAAAATCAACA	TTCTTGCAGCGAGGTCAGAT	60.5	353	93	GTPase-activating protein putative expressed
OsISM08538	LOC_Os01g59850	34617119	TGCCTTATATTTCCTCTGGGC	GGAGCTTACAGCATGAGGGA	60.4	181	80	GTPase-activating protein putative expressed
OsISM08539	LOC_Os01g59850	34617229	TCTGTTGTTGTTTCAGGAGGG	ATTTCTGGGCGGAAATGAC	60.0	166	94	GTPase-activating protein putative expressed
OsISM08540	LOC_Os01g59860	34619746	GAATAGCAGTGCTGGGAAGG	GATGCCATCTGGCTTGATCT	60.0	766	59	expressed protein
OsISM08541	LOC_Os01g59860	34620632	GGAAGGGGAAGGTCTTTCAA	TGCTGGACTTGGACTGTCAC	60.1	392	97	expressed protein
OsISM08542	LOC_Os01g59860	34621046	GTCCAGCATCTGGAGGACAC	GTTCTTCTTGCGTGCTTCAA	59.9	538	87	expressed protein
OsISM08543	LOC_Os01g59860	34621563	CTTGAAGCACGCAAGAAGAA	CACCATCTGGTCGACTCTCA	59.6	177	74	expressed protein

ISM IDs	Rice gene locus IDs	Physical positions (bp) on chromosomes	Forward primers (5'-3')	Reverse primers (5'-3')	Melting tempearature (T°C)	Expected product size (bp) in PCR	Expected product size (bp) in Q-PCR	Putative Functions
OsISM08544	LOC_Os01g59860	34621745	AAGGATGAAGAGGCCCAAAT	CCAGGTGGTAGTTCAGGGAG	59.7	324	93	expressed protein
OsISM08545	LOC_Os01g59870	34625594	CAGCGTCGACTACAAGCG	CCATAGGCACAGGTACTCCAA	59.6	900	99	LTPL65 - Protease inhibitor/seed storage/LTP family protein precursor expressed
OsISM08546	LOC_Os01g59870	34626523	GGGCCATCTACTGAACCACA	ATGGTTACTCGACTCGGACG	60.5	510	67	LTPL65 - Protease inhibitor/seed storage/LTP family protein precursor expressed
OsISM08547	LOC_Os01g59890	34636239	TTGCCTTGAAGCTTTTCTCAA	AGCACAGGCTGACAACACAC	60.0	565	84	pentatricopeptide putative expressed
OsISM08548	LOC_Os01g59980	34677514	CTCTGTTCAAGGACGGGGAG	GATGGTTTCCGCTCTCTCTG	61.0	580	96	zinc finger family protein putative expressed
OsISM08549	LOC_Os01g59980	34678299	AGCGAGGTGACTGGATCTGT	ACTCAAGGCATCTCGCATTT	59.9	488	67	zinc finger family protein putative expressed
OsISM08550	LOC_Os01g59980	34678816	ACTGGTGGAGAGTGGGAATG	AAGCATGACATGTTCCTCCC	59.9	158	63	zinc finger family protein putative expressed
OsISM08551	LOC_Os01g59990	34682500	CAGGCAGGACATTGTGAAAG	TCTTCTGCCCACTAAAACGG	59.8	193	79	ribosomal protein L24 putative expressed
OsISM08552	LOC_Os01g59990	34682695	TACCCAGGCAAGGGTATCAG	GGAAGTAGCGCTTGCATTTC	60.0	174	80	ribosomal protein L24 putative expressed
OsISM08553	LOC_Os01g59990	34682904	GCTATGTACAGGAAGCAGCAC A	CACCAACAATTGACCGTGAG	60.2	574	98	ribosomal protein L24 putative expressed
OsISM08554	LOC_Os01g59990	34683513	AAACCTGAAGTCCGTGATGC	TGCCTTTTTCTCATCCTTGG	60.2	445	85	ribosomal protein L24 putative expressed
OsISM08555	LOC_Os01g60060	34738594	GGATGGAATTTCCTGTTCCA	TTGCTTAATGTGCCTTGCAG	59.9	155	76	leucine-rich repeat family protein putative expressed
OsISM08556	LOC_Os01g60060	34738734	AGGCACATTAAGCAATGCAA	ACAATGCTTGGAGGAAGTGG	59.7	183	97	leucine-rich repeat family protein putative expressed
OsISM08557	LOC_Os01g60060	34738897	CCACTTCCTCCAAGCATTGT	TCACCAGTGAAACTGCATCC	59.9	179	72	leucine-rich repeat family protein putative expressed
OsISM08558	LOC_Os01g60060	34739087	AGATCGGAGCATTAAGGCAA	GGGATTCCACCAGTGAACTT	59.3	161	65	leucine-rich repeat family protein putative expressed
OsISM08559	LOC_Os01g60060	34739341	AGGGCTGGATCAGCTTGTTA	GTCAATTGGGCCTGTCAACT	59.9	174	68	leucine-rich repeat family protein putative expressed
OsISM08560	LOC_Os01g60060	34739495	AGTTGACAGGCCCAATTGAC	ATCCTGGGATTGGTCCAGTA	59.6	176	97	leucine-rich repeat family protein putative expressed
OsISM08561	LOC_Os01g60060	34739670	TCTCTCGGGAGAGTCTCATCA	ACTTCCTGGAACTGGACCAC	59.5	160	73	leucine-rich repeat family protein putative expressed

ISM IDs	Rice gene locus IDs	Physical positions (bp) on chromosomes	Forward primers (5'-3')	Reverse primers (5'-3')	Melting tempearature (T˚C)	Expected product size (bp) in PCR	Expected product size (bp) in Q-PCR	Putative Functions
OsISM08562	LOC_Os01g60060	34739806	TTCAGTGGTCCAGTTCCAGG	GTCCCATTCAGTTGGTTGCT	60.5	167	81	leucine-rich repeat family protein putative expressed
OsISM08563	LOC_Os01g60060	34739981	ACCTCACCAGTGCAAATGCT	TGTTAATGTTGAAAACCATCGC	60.3	166	90	leucine-rich repeat family protein putative expressed
OsISM08564	LOC_Os01g60060	34740103	ACTTCATGAGCTCACCAGCA	CACTGGGGATTGTTCCAGTAA	59.7	193	97	leucine-rich repeat family protein putative expressed
OsISM08565	LOC_Os01g60060	34740298	CTGTTCAGTTTCCCGCAAT	CAACCCGCAGTAGTGAACTG	59.0	203	98	leucine-rich repeat family protein putative expressed
OsISM08566	LOC_Os01g60060	34740535	AGTTGACCCAAGCTACACCG	GCTGCTTTTGCTTGAGTGTG	60.0	171	90	leucine-rich repeat family protein putative expressed
OsISM08567	LOC_Os01g60060	34741050	CAAACCTACAAAGCTCCAGCA	GCCCATTGACGACCTTTTAC	59.9	167	91	leucine-rich repeat family protein putative expressed
OsISM08568	LOC_Os01g60060	34741310	GCTAAGGAGGCAGTAGAACG AA	GCTGTGGCACATCTCCATTA	59.9	174	80	leucine-rich repeat family protein putative expressed
OsISM08569	LOC_Os01g60060	34741511	AGGAACTGAAGAGGTGCACA A	TTTGCGAGCATTCCTTTGTA	59.7	184	89	leucine-rich repeat family protein putative expressed
OsISM08570	LOC_Os01g60060	34741831	GGGGAGCAGATGTTGGTTTA	TGAAGGCGTTTCTTCCAATC	60.1	212	99	leucine-rich repeat family protein putative expressed
OsISM08571	LOC_Os01g60060	34742215	TCTACCCAAGTGAAGGGCAC	CTCAGATAGCTGCTGAGTCATGT	59.4	155	67	leucine-rich repeat family protein putative expressed
OsISM08572	LOC_Os01g60080	34746189	CCAACAACAACATCGTCGTC	CCATCCACGAGTTCTTCCTC	59.8	201	94	monocopper oxidase putative expressed
OsISM08573	LOC_Os01g60080	34747417	CATCGACTCCTTCCACATCA	TGTCCAGCAGGTTGTACGTC	59.7	202	99	monocopper oxidase putative expressed
OsISM08574	LOC_Os01g60100	34756407	TGCTTCAAGCTGGATAAGAGC	TGGGGAAGTCCACTATTTCG	59.8	181	96	transposon protein putative unclassified
OsISM08575	LOC_Os01g60140	34787715	CCTCGTCGTCCTCCTCAAG	AGACTTCCACCTGCCCCAT	61.4	197	99	ATCHX15 putative expressed
OsISM08576	LOC_Os01g60140	34788841	CTCAACATCGGACGTGACAA	CGACACCAGCACCATCAC	60.2	151	64	ATCHX15 putative expressed
OsISM08577	LOC_Os01g60140	34789332	ACGCCTTCGAGAACTACGAG	GAGCGTCTGGATGGAAATG	59.4	116	51	ATCHX15 putative expressed
OsISM08578	LOC_Os01g60190	34803784	TACAACTGCATCCATGTCGC	ACAGCTGTTCCATGAGCCTT	60.3	414	96	2 3-bisphosphoglycerate-independent phosphoglycerate mutase putative expressed
OsISM08579	LOC_Os01g60190	34804227	ACAGCGAAGTGGGTCACAAT	ATTTTCCCAGAGGCAAGAGC	60.6	182	89	2 3-bisphosphoglycerate-independent phosphoglycerate mutase putative expressed

ISM IDs	Rice gene locus IDs	Physical positions (bp) on chromosomes	Forward primers (5'-3')	Reverse primers (5'-3')	Melting tempearature (T˚C)	Expected product size (bp) in PCR	Expected product size (bp) in Q-PCR	Putative Functions
OsISM08580	LOC_Os01g60190	34804459	CCCTGCACCTTATTGGTTTG	TTTTTGCTCCCCTCTCACTG	60.4	196	97	2 3-bisphosphoglycerate-independent phosphoglycerate mutase putative expressed
OsISM08581	LOC_Os01g60190	34804796	TGTATGTTACCATGGACCGC	ACGTTTGACCACATCCCAGT	59.8	200	48	2 3-bisphosphoglycerate-independent phosphoglycerate mutase putative expressed
OsISM08582	LOC_Os01g60190	34805361	CTTGGTAAAGAATGGCGTCC	TATCCAGATCGGTTTCCGTT	59.5	485	91	2 3-bisphosphoglycerate-independent phosphoglycerate mutase putative expressed
OsISM08583	LOC_Os01g60190	34805950	GGGATGCCATTCTTAGTGGA	GCAACTACCGTGGCTTCAAT	60.0	179	98	2 3-bisphosphoglycerate-independent phosphoglycerate mutase putative expressed
OsISM08584	LOC_Os01g60190	34806109	ATTGAAGCCACGGTAGTTGC	ACTTGTTCAATCGCGTCCA	60.2	163	72	2 3-bisphosphoglycerate-independent phosphoglycerate mutase putative expressed
OsISM08585	LOC_Os01g60190	34806353	CGACAAGAACGGTGGTATCC	AAGACCGGGACCTCCAATAG	60.3	232	77	2 3-bisphosphoglycerate-independent phosphoglycerate mutase putative expressed
OsISM08586	LOC_Os01g60230	34834317	CCTGGTTGTGGAAGTCAGGA	AGAATGAACAAGGCTCACCC	60.1	281	97	auxin efflux carrier component putative expressed
OsISM08587	LOC_Os01g60230	34834582	GAGCCTTGTTCATTCTCTTGC	CCAGATGGATTTTGCCAGTAA	59.5	553	99	auxin efflux carrier component putative expressed
OsISM08588	LOC_Os01g60230	34835159	TAAGGCCATCCAATCCTTGT	AAGAGCCTGACACTGCACCT	59.7	182	93	auxin efflux carrier component putative expressed
OsISM08589	LOC_Os01g60230	34835366	CATCCGACCACCTTATCCAT	CGCATTCCCATCTTCATTACA	60.2	241	98	auxin efflux carrier component putative expressed
OsISM08590	LOC_Os01g60230	34835759	AATGTTTCAGGTTGGTGCAA	GTGCGAGTGCTTGTTGGATA	59.4	165	73	auxin efflux carrier component putative expressed
OsISM08591	LOC_Os01g60230	34835904	TATCCAACAAGCACTCGCAC	TCTGCATCAACAGAGCATGA	59.5	187	92	auxin efflux carrier component putative expressed
OsISM08592	LOC_Os01g60230	34836095	GGGCATTGTTAGAAGCATGG	TCAAGACTGGAACAACACCAA	59.8	200	80	auxin efflux carrier component putative expressed
OsISM08593	LOC_Os01g60230	34836270	GCCATTGGTGTTGTTCCAGT	CATTGCTTCCCTGAAAAAGG	60.3	217	98	auxin efflux carrier component putative expressed
OsISM08594	LOC_Os01g60230	34836595	GATGCATCTCCAGATCTTATCCT	TTCTTAGTACCTGACAGCACAGATG	59.3	257	66	auxin efflux carrier component putative expressed
OsISM08595	LOC_Os01g60260	34851552	GGAGAAATGCTTCTCCCCTG	GCAGCAACTCTGCCAGGTA	60.6	279	99	KH domain containing protein putative expressed
OsISM08596	LOC_Os01g60260	34851843	AGATTGGTCCCTTTGTGCAG	CAAGGTGCGAAACAATAGCA	60.0	426	88	KH domain containing protein putative expressed
OsISM08597	LOC_Os01g60260	34852335	CTAATTTTTGCGGGAATGGA	CTTCCCAACCCATAGTTCCC	60.2	873	85	KH domain containing protein putative expressed

ISM IDs	Rice gene locus IDs	Physical positions (bp) on chromosomes	Forward primers (5'-3')	Reverse primers (5'-3')	Melting tempearature (T˚C)	Expected product size (bp) in PCR	Expected product size (bp) in Q-PCR	Putative Functions
OsISM08598	LOC_Os01g60260	34853231	ATTGTGAAGAAGATCGTGCG	TTCAGTGAGTTTCCCCTTGG	59.5	216	96	KH domain containing protein putative expressed
OsISM08599	LOC_Os01g60260	34853487	GGGAAGGGCTCCATAAAAGA	AGGCCTTCCCTTCAATTGTT	60.2	248	52	KH domain containing protein putative expressed
OsISM08600	LOC_Os01g60260	34853800	TGACACAAGACTAGCACAAGCTC	CAAGCTCTCTAAGCTGTTGCC	59.6	507	99	KH domain containing protein putative expressed
OsISM08601	LOC_Os01g60270	34862375	GCGCAGCAGGACTCCATT	GCCTTGCTGTGATCTTGTGA	61.3	1244	89	homeobox domain containing protein expressed
OsISM08602	LOC_Os01g60270	34864027	TGGGCCAGATGTCCTTCTAC	GCTCTCCACCTTACCAAGGA	59.7	157	72	homeobox domain containing protein expressed
OsISM08603	LOC_Os01g60309	34887669	AAGTGGTGGGTTTGAGATGG	AGGAGCATGAGGTGATCCTG	60.0	995	95	retrotransposon protein putative Ty1-copia subclass
OsISM08604	LOC_Os01g60309	34888692	CCTCAGATGTCCCTAAACGC	GTGGAATGCCCATTCTCTGT	59.8	1020	92	retrotransposon protein putative Ty1-copia subclass
OsISM08605	LOC_Os01g60309	34890766	GGCACAGATAATGGCTTTGG	CGAATACCACCCAGGTTTTG	60.3	212	93	retrotransposon protein putative Ty1-copia subclass
OsISM08606	LOC_Os01g60330	34899370	CCATCTGGTAGCCTTGCTGT	TCCAATGGAGCTTTTCCAGT	60.0	410	60	inactive receptor kinase At2g26730 precursor putative expressed
OsISM08607	LOC_Os01g60360	34916673	ACCTGCAGAAAGATCCTCCA	CCTGCCAATGGAACATATCC	60.0	669	67	ubiquitin-conjugating enzyme putative
OsISM08608	LOC_Os01g60360	34917411	CGGACTACCCCTTCAAACCT	AGGCATATGCTTCCATTGCT	60.0	371	83	ubiquitin-conjugating enzyme putative
OsISM08609	LOC_Os01g60360	34917802	GGAGCCCTGCTTTGACAATA	GCAATCTCAGGGACAAGAGG	60.0	489	92	ubiquitin-conjugating enzyme putative
OsISM08611	LOC_Os01g60369	34920866	TTGAAGAGTTCCAAGGCCAG	ACCATTCTGAACAACCACCC	60.0	956	98	retrotransposon protein putative Ty3-gypsy subclass
OsISM08612	LOC_Os01g60369	34921806	GGTTGTTCAGAATGGTTGGG	CTCCTGGACAAAACCCTCAA	60.1	1066	85	retrotransposon protein putative Ty3-gypsy subclass
OsISM08613	LOC_Os01g60380	34926805	TAGAGGTTTCGTTGCAGCCT	CGGCAGGTTTCTGATGTTCT	60.1	374	78	retrotransposon protein putative Ty3-gypsy subclass
OsISM08614	LOC_Os01g60380	34927262	TCAGGCCATGATTCTATTTGG	TCTGATTCACCCTTTCGGTT	59.7	277	92	retrotransposon protein putative Ty3-gypsy subclass
OsISM08616	LOC_Os01g60389	34929841	TTGAAGAGTTCCAAGGCCAG	ACCATTCTGAACAACCACCC	60.0	956	98	retrotransposon protein putative Ty3-gypsy subclass
OsISM08617	LOC_Os01g60389	34930781	GGTTGTTCAGAATGGTTGGG	CTCCTGGACAAAACCCTCAA	60.1	1066	85	retrotransposon protein putative Ty3-gypsy subclass

ISM IDs	Rice gene locus IDs	Physical positions (bp) on chromosomes	Forward primers (5'-3')	Reverse primers (5'-3')	Melting tempearature (T°C)	Expected product size (bp) in PCR	Expected product size (bp) in Q-PCR	Putative Functions
OsISM08618	LOC_Os01g60400	34933271	TCCGAAAGACGCATATACCA	AGGGGAACAAAACAAAGGAA	58.8	286	84	retrotransposon protein putative Ty3-gypsy subclass
OsISM08619	LOC_Os01g60410	34938020	ACCTGCAGAAAGATCCTCCA	CCTGCCAATGGAACATATCC	60.0	624	67	ubiquitin-conjugating enzyme putative expressed
OsISM08620	LOC_Os01g60410	34938715	GACTACCCCTTCAAGCCTCC	AGGCATATGCTTCCATTGCT	59.9	559	81	ubiquitin-conjugating enzyme putative expressed
OsISM08621	LOC_Os01g60410	34939295	GAGCCCTGCTTTGACAATTT	GCAATCTCAGGGACAAGAGG	59.6	1179	91	ubiquitin-conjugating enzyme putative expressed
OsISM08622	LOC_Os01g60420	34943518	TCTACTACTGCAGCAAGTGCC	GAGAGGATCTCGAGCTGGG	59.2	200	95	expressed protein
OsISM08623	LOC_Os01g60470	34967901	ATTGCTCTGCCTGTCTGCTT	GCAGAAGGCTCTGGAAGAAC	59.7	1319	78	retrotransposon protein putative unclassified
OsISM08624	LOC_Os01g60480	34971591	CCATGCACATTGATTGGGTA	TTCTCCAGGTTCCATCCTTC	59.6	463	94	retrotransposon protein putative unclassified
OsISM08625	LOC_Os01g60480	34972169	TCTCAGGGCAGGATATCACC	TAGCTTTCAGGAAGGTGGCT	59.6	141	84	retrotransposon protein putative unclassified
OsISM08626	LOC_Os01g60480	34973537	CACTTCCTGTACCCTCAGGC	GTGGAGTGGAATGAAATGGG	59.9	137	78	retrotransposon protein putative unclassified
OsISM08627	LOC_Os01g60490	34980486	GGCTTCTGCAGGACAGTAGG	CAGTTCTTGTTCCGATGCAA	59.9	348	70	OsWRKY22 - Superfamily of TFs having WRKY and zinc finger domains expressed
OsISM08628	LOC_Os01g60490	34980868	GTGGAGAAAATACGGGCAAA	CATGGTCCTGATGGTACGTG	59.9	2989	81	OsWRKY22 - Superfamily of TFs having WRKY and zinc finger domains expressed
OsISM08629	LOC_Os01g60510	34992547	AGGAGACTGTACCCGGTGG	ACATCAAGAACGTTCCCGTC	60.0	492	95	hypothetical protein
OsISM08630	LOC_Os01g60520	34995856	CGGCGTCTTTGGCATCTC	CGTATGTTTCACCAATGATGG	60.8	4924	86	WRKY DNA-binding domain containing protein
OsISM08631	LOC_Os01g60520	35000819	GGCCAGAAGAACATCAAGGA	CAAGGCACTGTCTGTCCTCA	60.1	826	77	WRKY DNA-binding domain containing protein
OsISM08632	LOC_Os01g60540	35008056	GACGAGGACTGACGATAGCA	CCACCTCTTCTCTGCCATTT	59.1	194	96	OsWRKY20 - Superfamily of TFs having WRKY and zinc finger domains
OsISM08633	LOC_Os01g60540	35008312	GGGCAAAAGAACATTCAGGA	CTGCTGCACTTGCTTCTTCG	61.2	1108	97	OsWRKY20 - Superfamily of TFs having WRKY and zinc finger domains
OsISM08634	LOC_Os01g60560	35015407	GCCAAGTCAATGTCAGACCA	GCACTGCTTCTCGTCCTTTT	59.7	129	93	retrotransposon protein putative unclassified
OsISM08635	LOC_Os01g60610	35037215	GATCTGGGAGATGGCTACGA	CACCCGTTGGACCACTATCT	60.0	231	58	retrotransposon protein putative unclassified

ISM IDs	Rice gene locus IDs	Physical positions (bp) on chromosomes	Forward primers (5'-3')	Reverse primers (5'-3')	Melting tempearature (T˚C)	Expected product size (bp) in PCR	Expected product size (bp) in Q-PCR	Putative Functions
OsISM08636	LOC_Os01g60610	35037880	GTCGTTCAACCGACCTCCT	CGTATCCTCGGCCTCAGAC	60.4	179	83	retrotransposon protein putative unclassified
OsISM08637	LOC_Os01g60640	35062004	CGTGACCTCCCCTTCTCTC	GCTCCTCACCTCCCTCACC	61.0	293	77	OsWRKY21 - Superfamily of TFs having WRKY and zinc finger domains expressed
OsISM08638	LOC_Os01g60770	35144642	ACTACGGCTCCTGGCAGA	GGTGCTGTACAGGTTCCCAT	59.7	208	99	expansin precursor putative expressed
OsISM08639	LOC_Os01g60770	35145105	ATCGTGCCCGTCTCCTACA	AGTAGGAGTGCCCGTTGATG	60.9	440	77	expansin precursor putative expressed
OsISM08640	LOC_Os01g60780	35147794	CTCATGAGCACACTCGGCT	AAATGTCACCAGGAGATGCC	60.0	1668	64	solute carrier family 35 member E3 putative expressed
OsISM08641	LOC_Os01g60780	35149560	TGGGTTGCTTAATCTGAGCC	GGAATTATTGCCAGCTTGGT	59.6	402	70	solute carrier family 35 member E3 putative expressed
OsISM08642	LOC_Os01g60780	35149931	TGCATCAGGTAACCAAGCTG	CCAGTGCCATTTGGATTCTC	60.2	352	97	solute carrier family 35 member E3 putative expressed
OsISM08644	LOC_Os01g60790	35152879	GTACATCCGCTGCTCCAACT	CTCCACGATGTTCCTCACCT	60.2	183	80	40S ribosomal protein S26 putative expressed
OsISM08645	LOC_Os01g60790	35153042	AGGTGAGGAACATCGTGGAG	GCGTAAAGCTTGGGGAGAAC	60.6	269	86	40S ribosomal protein S26 putative expressed
OsISM08647	LOC_Os01g60800	35155914	GGCTCGACGTACCAGGTGT	TTTGGATGCAACAATCTCCA	60.6	876	49	expressed protein
OsISM08648	LOC_Os01g60800	35157014	TCAGAGCGGGTCAACTTTCT	TCTTGAACTCCCCATTGGAC	59.9	517	93	expressed protein
OsISM08649	LOC_Os01g60830	35174754	CACAAAGGATGGAGGAAGGA	CTTGTAGGTGCCACCGATCT	60.1	207	71	expressed protein
OsISM08650	LOC_Os01g60830	35174941	AGATCGGTGGCACCTACAAG	TTTGCAGGTCTTATCCACCC	60.0	205	90	expressed protein
OsISM08652	LOC_Os01g60880	35210532	GTCCATCTGGACAGGCAAGT	GTGGTTCCTGTCAATCCCAC	60.2	501	75	transposon protein putative unclassified
OsISM08653	LOC_Os01g60880	35211058	GTGCACAGCTCGACACCTT	AGTGGTAGAAGGCCTGCCG	61.4	172	98	transposon protein putative unclassified
OsISM08654	LOC_Os01g60880	35211550	CAGTGGAGCTGGGGTTCTG	AAACTCGAACACGAAGTCCG	61.1	385	90	transposon protein putative unclassified
OsISM08655	LOC_Os01g60880	35212096	GCAGCGTGTGATGAGACAGT	CGTCGAGTCAACCGAGGTA	60.0	161	61	transposon protein putative unclassified
OsISM08656	LOC_Os01g60880	35212476	GCACCGTGACCGAGACTACT	ATATCGGCACTGATATCCCG	60.0	152	58	transposon protein putative unclassified

ISM IDs	Rice gene locus IDs	Physical positions (bp) on chromosomes	Forward primers (5'-3')	Reverse primers (5'-3')	Melting tempearature (T˚C)	Expected product size (bp) in PCR	Expected product size (bp) in Q-PCR	Putative Functions
OsISM08658	LOC_Os01g60880	35213184	AGCTCTTCCTCTCACGGAGC	AGACGAAGAGGCCCCAGTA	60.5	144	82	transposon protein putative unclassified
OsISM08659	LOC_Os01g60880	35213514	TACTCCCAGGGGCACATTAG	TCGAGTAAATCCACCCATCC	59.8	615	71	transposon protein putative unclassified
OsISM08660	LOC_Os01g60900	35223940	GTGGCTGTGGTGGATTCAG	CACCTGATTAGCGCCAAAAT	60.1	430	87	expressed protein
OsISM08662	LOC_Os01g60920	35238649	CGCTGTCAAACCAACTTTGTA	TTGACTCCTCGTACCGTTCA	59.1	198	95	OsFBX30 - F-box domain containing protein expressed
OsISM08663	LOC_Os01g60930	35242946	GCTTCGGTCTCGTATTCTCG	ACCTGCATTTGCAGTTCCTC	60.1	1266	98	expressed protein
OsISM08664	LOC_Os01g60960	35256637	TGTCCATCTCCATCTCGTCA	TGATCTTCTTGCCGATCTCA	59.8	381	94	DUF260 domain containing protein putative expressed
OsISM08665	LOC_Os01g60960	35257261	CCAGCAACGTCTCCAAGATG	CTCGTACACCAGGCTGCTC	60.7	168	75	DUF260 domain containing protein putative expressed
OsISM08666	LOC_Os01g60970	35273899	GTGGTGACGAGAGGCTTGG	CCGCAGGAAGGAGACGAG	62.0	173	99	transposon protein putative unclassified
OsISM08667	LOC_Os01g60980	35278850	GTCGATGGACATTCTCCAGG	AAGCCAGTCATGTCCAAACC	60.2	1189	92	retrotransposon protein putative Ty3-gypsy subclass
OsISM08668	LOC_Os01g60980	35280745	GTTGGTAGCTCGGAGGGTG	AAAACCCCACACAGAACCAG	60.3	305	91	retrotransposon protein putative Ty3-gypsy subclass
OsISM08669	LOC_Os01g60990	35288562	TTGTCTTTGAGTGTGCTGGG	GATAGAGGGTGTCACGACCG	60.2	174	73	retrotransposon protein putative unclassified
OsISM08670	LOC_Os01g61070	35331664	TCGAAGCTGTGTAAACCTGTG	GTGCATCCCAACCTTCAACT	59.5	262	80	heavy metal-associated domain containing protein expressed
OsISM08671	LOC_Os01g61070	35331943	GGCGATCAAGAAGGCAATAA	CTGGGGTAACATTTCCTGTCA	60.0	198	96	heavy metal-associated domain containing protein expressed
OsISM08672	LOC_Os01g61080	35347289	AACCAGGCCGACTTCCTC	GGCCTCCAGTCAAAGTGCT	60.3	215	99	OsWRKY24 - Superfamily of TFs having WRKY and zinc finger domains expressed
OsISM08673	LOC_Os01g61080	35347611	GAACGACGACTTTCCAGCC	CTGCTGCTGCTGACTTCTGT	60.2	271	72	OsWRKY24 - Superfamily of TFs having WRKY and zinc finger domains expressed
OsISM08674	LOC_Os01g61080	35348357	AGATCAGAGTTGGCTCCCCT	CTCGCCGTCACCGTCTTT	61.3	188	99	OsWRKY24 - Superfamily of TFs having WRKY and zinc finger domains expressed
OsISM08675	LOC_Os01g61080	35348632	TACCGCTGGAGAAAGTACGG	GCCGTGGTGCACTTGTAGTA	60.0	186	75	OsWRKY24 - Superfamily of TFs having WRKY and zinc finger domains expressed
OsISM08676	LOC_Os01g61100	35361389	TGTGTGATGTAAATGAAGGTGTAAA	TCAGAAGTTCCTGCATTTGTGT	59.2	151	89	transposon protein putative unclassified

ISM IDs	Rice gene locus IDs	Physical positions (bp) on chromosomes	Forward primers (5'-3')	Reverse primers (5'-3')	Melting tempearature (T°C)	Expected product size (bp) in PCR	Expected product size (bp) in Q-PCR	Putative Functions
OsISM08677	LOC_Os01g61100	35361584	CATCAGCTCCAGAATCAGCA	TTTAAAACTTACATAACAATTGAACCA	58.8	516	79	transposon protein putative unclassified
OsISM08678	LOC_Os01g61100	35362044	CATGTGTGATGTAAATGAAGGTG	TTCAGAAGTTCTTGCATTTGTGT	58.4	154	92	transposon protein putative unclassified
OsISM08679	LOC_Os01g61100	35362243	TCAGCTCCAGAATCAGCAAA	TGACTCAATCAGGGCTTTGTC	60.0	455	67	transposon protein putative unclassified
OsISM08680	LOC_Os01g61100	35362683	GCCCTGATTGAGTCATACAACA	TTGCTTCTGATATTTGTCCATCTC	59.8	171	97	transposon protein putative unclassified
OsISM08681	LOC_Os01g61100	35362883	TGATGAATTAGCTCTGTGGCA	GAAGATCGAAACCGATGCAG	60.1	582	87	transposon protein putative unclassified
OsISM08682	LOC_Os01g61100	35363445	CTGCATCGGTTTCGATCTTC	CCTGGAATGCTTTTGGTGTT	60.4	1177	69	transposon protein putative unclassified
OsISM08683	LOC_Os01g61100	35364575	ACAGGGGCAAGTCTCAATTA	TGACAGCTGACTGCATTGAA	58.2	311	81	transposon protein putative unclassified
OsISM08684	LOC_Os01g61100	35364867	TCAATGCAGTCAGCTGTCAA	TCTCTGAATCATCTGAATCACCAT	59.5	184	95	transposon protein putative unclassified
OsISM08685	LOC_Os01g61100	35365052	TGAACAAAATCCAGTAGACCCA	GCCCATTGTTCAGACTCTCC	59.6	210	93	transposon protein putative unclassified
OsISM08686	LOC_Os01g61100	35365360	CAGCATGAAGCATTACAAGGA	ATTGTTTGCTTGTGGTGTGG	59.2	171	96	transposon protein putative unclassified
OsISM08687	LOC_Os01g61100	35365612	GGAGACAGTTGTTTCAAAGAGG	GGCAGTGTTGTCATTCCTCC	59.2	284	76	transposon protein putative unclassified
OsISM08688	LOC_Os01g61100	35367562	CACATAGAGTTCAATTTAGCAAAGAAA	TTGGAGTTTTTAATTTCTTCAACA	58.3	204	99	transposon protein putative unclassified
OsISM08689	LOC_Os01g61140	35393741	GCAACAACAATAACGGTGGG	GCATCTTGCACGGTTCTGT	60.5	136	99	retrotransposon protein putative unclassified
OsISM08690	LOC_Os01g61160	35401887	CTGCTCTTCCTCCTCTCGTG	CTCTTCACCGGCGTCTCTT	60.4	232	93	laccase precursor protein putative expressed
OsISM08691	LOC_Os01g61160	35402223	CGCAGTACAACGTCACCATC	GGGCACTGTGTCACGAACT	60.0	232	86	laccase precursor protein putative expressed
OsISM08692	LOC_Os01g61160	35402580	GGGAGAACAAGACCTACCCC	CTGATGACTTGGATCGGGTC	60.1	199	92	laccase precursor protein putative expressed
OsISM08693	LOC_Os01g61160	35402834	GGTCAGCCTGGTGATCTGTA	GTTGATGAACCGCAGCAG	58.8	189	91	laccase precursor protein putative expressed
OsISM08694	LOC_Os01g61190	35413712	TGCGACTTGAGGATGTGAGA	GTTCTGCTTGGCAGGTCTTC	60.3	1669	83	exo70 exocyst complex subunit putative expressed

ISM IDs	Rice gene locus IDs	Physical positions (bp) on chromosomes	Forward primers (5'-3')	Reverse primers (5'-3')	Melting tempearature (T°C)	Expected product size (bp) in PCR	Expected product size (bp) in Q-PCR	Putative Functions
OsISM08695	LOC_Os01g61230	35430047	CACGCCACCGTCAAGAAC	AGGAGGTCCATCTGGAACAG	60.2	208	99	dihydroflavonol-4-reductase putative expressed
OsISM08696	LOC_Os01g61230	35430316	CCTCTCACACTTCACCCGAC	GAGGACGTTGAGAGTGCCA	60.3	209	76	dihydroflavonol-4-reductase putative expressed
OsISM08697	LOC_Os01g61230	35430640	ACGTCGAGCTCCTCAAGAAA	CCTTCTCTGCCAGCGTTTTA	60.3	456	61	dihydroflavonol-4-reductase putative expressed
OsISM08698	LOC_Os01g61230	35431170	TTGATGCCATCCCCTACTTC	CTGACATCGACACAGCCAAT	59.8	178	99	dihydroflavonol-4-reductase putative expressed
OsISM08699	LOC_Os01g61230	35431462	TTACCCTGAATTCCCGATCC	GAGGCAGCCTTTGATCTCAC	60.5	159	70	dihydroflavonol-4-reductase putative expressed
OsISM08700	LOC_Os01g61240	35432994	CCGACGGATCGATTACCTG	ACGCAAGCTTCTATCGCATT	60.7	184	65	gamma-interferon-inducible lysosomal thiol reductase precursor putative
OsISM08701	LOC_Os01g61240	35433158	AATGCGATAGAAGCTTGCGT	TCAGCGCTAAATGCTCAATG	60.1	323	86	gamma-interferon-inducible lysosomal thiol reductase precursor putative
OsISM08702	LOC_Os01g61240	35433550	GCTATAACTCAGGGTACGGCA	ACTGATGAGGAGGCTGAAGG	59.3	542	76	gamma-interferon-inducible lysosomal thiol reductase precursor putative
OsISM08703	LOC_Os01g61240	35434094	GTACCCTGGGTGACCGTAAA	GCCTCAGGAAGTTCACCATC	59.7	193	96	gamma-interferon-inducible lysosomal thiol reductase precursor putative
OsISM08704	LOC_Os01g61240	35434306	GCCATTGCTCAACAGACAAG	TTTGTGGATCCCTCTGCATT	60.0	335	90	gamma-interferon-inducible lysosomal thiol reductase precursor putative
OsISM08705	LOC_Os01g61250	35436455	GTACCGGTCACCCTTGCC	CCCAGTTCTGGTCCTTCTTG	60.5	701	99	methyltransferase putative expressed
OsISM08706	LOC_Os01g61250	35437184	CCCTGGAGGTGGAACTCAAT	AGTTCTTGTATGGGTCCCCC	60.7	370	98	methyltransferase putative expressed
OsISM08707	LOC_Os01g61250	35437744	TGATCCATTGTGCAAGGTGT	TCCAGGCCTGAGCACTCTAT	60.0	244	84	methyltransferase putative expressed
OsISM08708	LOC_Os01g61250	35438011	TCCTGTATATCGTCGTGGCA	CTCCAGCAAATTGACTTGGTC	59.9	696	76	methyltransferase putative expressed
OsISM08709	LOC_Os01g61250	35438802	AACCACCCCTATGCCCTAGT	CCTGAACTTGACACAGCAGG	59.6	325	92	methyltransferase putative expressed
OsISM08710	LOC_Os01g61250	35439295	GTCTACCGTTCGCAATGTCA	AAGGTTTGTGGATGAGTGACG	59.9	601	72	methyltransferase putative expressed
OsISM08711	LOC_Os01g61250	35440036	TATCTCCTTCAGGGCCTCAC	AACACGAACCATTTTCCAGG	59.5	203	90	methyltransferase putative expressed
OsISM08713	LOC_Os01g61270	35447031	TCGGCGTAGCTCTGTGACTA	ATCAAAACCAAAGCGGACTG	59.9	219	92	retrotransposon protein putative Ty3-gypsy subclass

ISM IDs	Rice gene locus IDs	Physical positions (bp) on chromosomes	Forward primers (5'-3')	Reverse primers (5'-3')	Melting tempearature (T°C)	Expected product size (bp) in PCR	Expected product size (bp) in Q-PCR	Putative Functions
OsISM08714	LOC_Os01g61270	35448144	CGCAAGTCTAACCATCAGGG	ACTAGGGCATGCTTCTGTGG	60.5	167	94	retrotransposon protein putative Ty3-gypsy subclass
OsISM08715	LOC_Os01g61320	35477010	ATCGTTCTTGTCTTCGAGCG	AAACTTTAGCCGCCTTTTCC	60.1	358	80	thioredoxin putative expressed
OsISM08716	LOC_Os01g61320	35477345	ATTGGAAAAGGCGGCTAAAG	GCAAATGCCAGGTCTCTCTC	60.3	192	97	thioredoxin putative expressed
OsISM08717	LOC_Os01g61320	35477567	ATTCCTCAGGGGAAACAAGA	GAAATGGGCAATCATTTGAA	58.5	261	80	thioredoxin putative expressed
OsISM08718	LOC_Os01g61335	35485106	TGCATGTAGGAGTAGATGGGA A	TTCAGCTTCATATCCCTGGC	59.9	788	94	expressed protein
OsISM08719	LOC_Os01g61410	35508396	CCGGCTCCTACTTCCTCCT	TCACCTTCAGTAACTGTCTCGC	60.1	792	83	NADH-ubiquinone oxidoreductase mitochondrial precursor putative expressed
OsISM08720	LOC_Os01g61410	35509341	GCTCCTTCTGAACCTGATGC	AACAACAACGCAATGCAAAA	60.1	427	80	NADH-ubiquinone oxidoreductase mitochondrial precursor putative expressed
OsISM08721	LOC_Os01g61410	35509817	TCATCAGAGATGTTAAACAGC GTTA	GAGGCGAACATCAAAAGATGA	60.2	179	99	NADH-ubiquinone oxidoreductase mitochondrial precursor putative expressed
OsISM08722	LOC_Os01g61410	35509990	CGCCTCAGGCAATATGCTAC	TCACAATGCCTCTCACAAGC	60.4	213	65	NADH-ubiquinone oxidoreductase mitochondrial precursor putative expressed
OsISM08723	LOC_Os01g61410	35510303	GTCGTTGCCATTTCCAAAGT	CGTAACCACTCATCAACCCC	60.1	333	55	NADH-ubiquinone oxidoreductase mitochondrial precursor putative expressed
OsISM08724	LOC_Os01g61410	35510663	TATTGGGGATTGCAGTGGAT	TACTTGCCTTGTCGTTCTGC	59.6	843	85	NADH-ubiquinone oxidoreductase mitochondrial precursor putative expressed
OsISM08725	LOC_Os01g61410	35511611	CATGGCAACTGTCGGAAGAT	CCAGATGAACCAGCTCACAA	60.4	185	98	NADH-ubiquinone oxidoreductase mitochondrial precursor putative expressed
OsISM08726	LOC_Os01g61450	35529793	AAGGGAAGGAGAGGGAGGAT	CCCAACTTGCCCTTCTTGTA	60.3	1569	90	hypothetical protein
OsISM08727	LOC_Os01g61460	35538165	GACACCTACCAGAAGTGGCG	GCCTCTCCATGAACAGCTTC	60.3	227	77	leaf senescence related protein putative expressed
OsISM08728	LOC_Os01g61460	35538490	AAATACGTCACCTGGGAGGA	GAGTTGGACTCCACCAGGAA	59.7	422	99	leaf senescence related protein putative expressed
OsISM08729	LOC_Os01g61460	35539025	GGTGGATGAACACCCTCAAC	CTCACGACCTCGTCATGCT	60.1	185	74	leaf senescence related protein putative expressed
OsISM08730	LOC_Os01g61460	35539289	TTCTTCATGAGCATCTCCCC	ATCGTCTCCTTGGCGCAC	61.3	3338	81	leaf senescence related protein putative expressed
OsISM08731	LOC_Os01g61490	35568219	AGCCTCACCCTCCAAGAAGT	TCTCTCCTTTCTCCTTCCCA	59.6	151	98	retrotransposon protein putative unclassified

ISM IDs	Rice gene locus IDs	Physical positions (bp) on chromosomes	Forward primers (5'-3')	Reverse primers (5'-3')	Melting tempearature (T˚C)	Expected product size (bp) in PCR	Expected product size (bp) in Q-PCR	Putative Functions
OsISM08732	LOC_Os01g61490	35569368	TTGCTCCAAGTGTTGTGAGG	TCACCGGTCATGTGTTGAGT	59.9	319	97	retrotransposon protein putative unclassified
OsISM08733	LOC_Os01g61490	35571362	GACCACAATGTCATAGGGACAA	AGCTCAACTCTCCAATCATGG	59.5	160	86	retrotransposon protein putative unclassified
OsISM08734	LOC_Os01g61490	35571626	CCCATCAAGACACCTATGGC	GCAACTACCGGATGTGCTCT	60.3	326	97	retrotransposon protein putative unclassified
OsISM08735	LOC_Os01g61490	35572284	CAAGCCTCTTGATGAAACCC	CGCTTGATTCCTCATTCTCA	59.3	1796	99	retrotransposon protein putative unclassified
OsISM08736	LOC_Os01g61500	35581655	AGGCTCAGGGTCTCCCAC	GGGTAAGGACCCCCTTCAAT	60.7	950	86	BAG domain containing protein expressed
OsISM08737	LOC_Os01g61500	35582784	ATGAAGGCTTGTGAGGCTGT	TCTTTCTCCCTGCAAACACA	59.6	195	98	BAG domain containing protein expressed
OsISM08738	LOC_Os01g61500	35583045	TAGAGGCAGAGGGAGAAGCA	CCAACGTCTCCACAAGACCT	60.2	1256	73	BAG domain containing protein expressed
OsISM08739	LOC_Os01g61570	35605315	GGCAAGCTCCGATCGTACA	GTCATCTGCACCGGGAGG	62.5	670	99	GDSL-like lipase/acylhydrolase putative expressed
OsISM08740	LOC_Os01g61570	35606131	TCTACACCCCTCTCACGGAC	CATCTCCAGCAGCCCAGT	60.0	212	93	GDSL-like lipase/acylhydrolase putative expressed
OsISM08741	LOC_Os01g61590	35619485	ATTTCGGCCTCTCCATCTTC	TATGGGCTTCCCACAATTTC	60.2	2056	62	CAMK CAMK like.1 - CAMK includes calcium/calmodulin depedent protein kinases expressed
OsISM08742	LOC_Os01g61590	35621627	GTGGAGTTCCTCCATTTTGG	TTCCACGAAGAATGGCTTGT	60.0	142	61	CAMK CAMK like.1 - CAMK includes calcium/calmodulin depedent protein kinases expressed
OsISM08743	LOC_Os01g61590	35621839	GGAGCCTGATCCAAAACTCA	CCAAGAGGAACATTTGGAGC	59.9	480	91	CAMK CAMK like.1 - CAMK includes calcium/calmodulin depedent protein kinases expressed
OsISM08744	LOC_Os01g61590	35622325	TGTGAAGTCAAGGCTCAAACA	TGTCCTCAACTTCCTCAGCA	59.5	213	99	CAMK CAMK like.1 - CAMK includes calcium/calmodulin depedent protein kinases expressed
OsISM08745	LOC_Os01g61590	35622637	AATCGGAAGTGCAGATGCTT	GAGACAGCCAAAAATTCACCA	60.0	1392	80	CAMK CAMK like.1 - CAMK includes calcium/calmodulin depedent protein kinases expressed
OsISM08746	LOC_Os01g61590	35624154	GTGACAGCATGGAAGTGGTG	GCCCGTCTTCATCATTGCTA	60.7	192	99	CAMK CAMK like.1 - CAMK includes calcium/calmodulin depedent protein kinases expressed
OsISM08747	LOC_Os01g61610	35636266	CTACTTCCTCGTCCTCTCGC	CTCTCCCGTACTCCTCGGAC	60.2	2075	99	flavonol synthase/flavanone 3-hydroxylase putative expressed
OsISM08748	LOC_Os01g61610	35638618	CTTCATCGTCAACGTCGG	CTCCACGCTCTTGTACATCG	59.1	753	62	flavonol synthase/flavanone 3-hydroxylase putative expressed
OsISM08749	LOC_Os01g61620	35652602	CTTCGTCAGCATGCGCTC	AAACTCCATCTCCGATGACG	61.0	497	95	protein kinase family protein putative expressed

ISM IDs	Rice gene locus IDs	Physical positions (bp) on chromosomes	Forward primers (5'-3')	Reverse primers (5'-3')	Melting tempearature (T°C)	Expected product size (bp) in PCR	Expected product size (bp) in Q-PCR	Putative Functions
OsISM08750	LOC_Os01g61620	35653625	GGGTTGATGCAGATGCTTTT	CCAGTTTGGCATTGTCTGTG	60.1	847	64	protein kinase family protein putative expressed
OsISM08751	LOC_Os01g61620	35654665	CGCTCTCGCTAATTTCCATC	CAATGGGGAAATCTTTGCTT	59.5	205	99	protein kinase family protein putative expressed
OsISM08752	LOC_Os01g61620	35655072	TGAGTATCATGTGTTGCGTCTCT	GCTCGCAGTAATTCAGTGACA	59.5	376	76	protein kinase family protein putative expressed
OsISM08753	LOC_Os01g61620	35655478	GGAATCTGGTGGTGAAGCAT	GCTTCTAAACATTGCCGAGC	60.0	334	67	protein kinase family protein putative expressed
OsISM08754	LOC_Os01g61620	35656036	GCATCCTTGCGGAACTCTAC	AGCATTATGGGCACTGGTTC	59.9	502	62	protein kinase family protein putative expressed
OsISM08755	LOC_Os01g61620	35656619	TGATTACGACCTTTTCACCAA	GGCATTGTAAGTGATGCTGC	58.7	234	90	protein kinase family protein putative expressed
OsISM08756	LOC_Os01g61650	35669903	GGGAAGAGGAAACAGGAGGA	ACTTGGAATGGCGGGAAG	60.8	351	86	retrotransposon protein putative unclassified
OsISM08758	LOC_Os01g61670	35682476	TACAACCTGGAGCTCAGCAA	TTACCCCGTCGTCTTTCTTG	59.8	1892	71	ureidoglycolate hydrolase putative expressed
OsISM08759	LOC_Os01g61680	35685574	GACAGCTACCCCTCCATCAA	GTCGACGAGCTCAGGTTCA	60.1	264	75	expressed protein
OsISM08760	LOC_Os01g61730	35713699	ACCTGCAGCTTTGTTGATTG	TTAGCCACCTGGGTTAGTGC	59.5	249	49	hypothetical protein
OsISM08761	LOC_Os01g61750	35719720	GCCTGACAGGATGGATGC	ACCGTGGTAAAGCACAAACC	60.0	193	83	hypothetical protein
OsISM08762	LOC_Os01g61780	35731906	CTCGCCGTCACCTACCTC	CGCTGAAAAGGACTCTTATCC	58.7	1256	75	vacuolar ATP synthase 98 kDa subunit putative expressed
OsISM08763	LOC_Os01g61780	35733141	GGATAAGAGTCCTTTTCAGCG	CGTGACATTTCAGAGCATCG	59.2	1153	61	vacuolar ATP synthase 98 kDa subunit putative expressed
OsISM08764	LOC_Os01g61780	35734337	AAGTCCTCTGTTCGACCTGC	CAGATCATTCTCATGCTCGC	59.5	195	82	vacuolar ATP synthase 98 kDa subunit putative expressed
OsISM08766	LOC_Os01g61780	35735024	AAATGGATGATGGAAATGCC	AATTCTCCGAAGCCCCTAGA	59.9	142	61	vacuolar ATP synthase 98 kDa subunit putative expressed
OsISM08767	LOC_Os01g61780	35735273	GCAGGGGAACCTGTTACTGA	TTAGCCTGATCCCCAGAAAA	59.9	311	84	vacuolar ATP synthase 98 kDa subunit putative expressed
OsISM08768	LOC_Os01g61780	35735618	GCAAGTTGCTACCCTGTTCC	AAAGTGGCTTCCAGATCAGC	59.6	280	90	vacuolar ATP synthase 98 kDa subunit putative expressed
OsISM08769	LOC_Os01g61780	35735941	GGTTCACAATTATGGAGGTGG	CAAGGCATTTCTTCGTGACA	59.5	216	98	vacuolar ATP synthase 98 kDa subunit putative expressed

ISM IDs	Rice gene locus IDs	Physical positions (bp) on chromosomes	Forward primers (5'-3')	Reverse primers (5'-3')	Melting tempearature (T˚C)	Expected product size (bp) in PCR	Expected product size (bp) in Q-PCR	Putative Functions
OsISM08770	LOC_Os01g61780	35736141	ACGAAGAAATGCCTTGTTGG	CACGCTGCAAAACATCCTT	60.0	174	74	vacuolar ATP synthase 98 kDa subunit putative expressed
OsISM08771	LOC_Os01g61780	35736417	TCCAGGAGATAGTTGACGCA	CACAGCAAAAAGAAATGGGAA	59.7	606	93	vacuolar ATP synthase 98 kDa subunit putative expressed
OsISM08772	LOC_Os01g61780	35737062	GGAGCATGTGTTCTTATACTTCG	GGATAACATAACGCCCACCA	59.3	1172	92	vacuolar ATP synthase 98 kDa subunit putative expressed
OsISM08773	LOC_Os01g61780	35738307	CGGGAAATCAGCGTATGAAT	TCACGGACTTTAATGAGCCC	60.0	1166	73	vacuolar ATP synthase 98 kDa subunit putative expressed
OsISM08774	LOC_Os01g61780	35739612	GCAAAGTTTCATGGAAATGCT	AGCCAAAGAGGCTGTTCAGA	59.7	172	77	vacuolar ATP synthase 98 kDa subunit putative expressed
OsISM08775	LOC_Os01g61780	35739893	ACTATTTTGGGGCCAGAAGG	AAAGGCTTTGGGAAGAGCAT	60.3	169	85	vacuolar ATP synthase 98 kDa subunit putative expressed
OsISM08776	LOC_Os01g61780	35740025	TTGTAGCTGTTCCGTGGATG	TCCCAAGAAAGCGATAGGTG	60.0	1377	94	vacuolar ATP synthase 98 kDa subunit putative expressed
OsISM08777	LOC_Os01g61780	35741530	ACTGCATCGTATCTTCGGCT	TACCCCCAAGCAAGTACCAG	59.9	183	93	vacuolar ATP synthase 98 kDa subunit putative expressed
OsISM08778	LOC_Os01g61780	35741650	TTTGGCGCATTCTGAGCTAT	GCCCCACTAACTTCACAACAA	60.4	283	90	vacuolar ATP synthase 98 kDa subunit putative expressed
OsISM08779	LOC_Os01g61800	35751490	TGTCTGCGCACTACATAGCC	ACGACGGATCCACATGAAGT	60.2	402	79	ZmEBE-1 protein putative
OsISM08780	LOC_Os01g61800	35751929	ACCCGATGAAGGAGCCTATT	TGAGTTCATCTCGTATCCTGC	58.9	204	50	ZmEBE-1 protein putative
OsISM08781	LOC_Os01g61800	35752215	CAATTGCCAACAAGGGAGAT	AGCGTGTGCATCACCATTTA	60.0	238	90	ZmEBE-1 protein putative
OsISM08782	LOC_Os01g61800	35752439	GTGATGCACACGCTCATTTC	AGCCTGTCTTCCGATATCCA	60.0	141	58	ZmEBE-1 protein putative
OsISM08783	LOC_Os01g61800	35752653	AGTCTCAGAGCCTGGAGGAA	AGTCCCATCCGCAGTGTAGT	59.4	195	90	ZmEBE-1 protein putative
OsISM08784	LOC_Os01g61830	35766854	GAAGTACGTCCAGTCCCTGC	GCCTTCTCCTCCTCCTTCTT	59.4	199	88	zinc finger C-x8-C-x5-C-x3-H type family protein expressed
OsISM08785	LOC_Os01g61830	35767075	CTATTCAAGGTCGCCGTCAG	CACACTATGGACTTGGGATCG	60.6	525	63	zinc finger C-x8-C-x5-C-x3-H type family protein expressed
OsISM08786	LOC_Os01g61830	35767663	TGTCCAAAGAAAGGGTGAAAA	CCTGGTCCCAGTCTTCCAT	59.7	177	75	zinc finger C-x8-C-x5-C-x3-H type family protein expressed
OsISM08787	LOC_Os01g61830	35767851	AAGGTTGTTGCGTCAAAAGG	CTCCACGGCATCAAGAAAGT	60.2	1386	82	zinc finger C-x8-C-x5-C-x3-H type family protein expressed

ISM IDs	Rice gene locus IDs	Physical positions (bp) on chromosomes	Forward primers (5'-3')	Reverse primers (5'-3')	Melting tempearature (T°C)	Expected product size (bp) in PCR	Expected product size (bp) in Q-PCR	Putative Functions
OsISM08788	LOC_Os01g61830	35769367	TTGCTATCGAAGATGAGATTGAA	TCTGCCTTCTTCCTTTTCCA	59.5	210	98	zinc finger C-x8-C-x5-C-x3-H type family protein expressed
OsISM08789	LOC_Os01g61830	35769606	GGGCAGAGAGGGCTAAGAAT	GCTTCAGCATCATCAACGAA	59.9	169	83	zinc finger C-x8-C-x5-C-x3-H type family protein expressed
OsISM08790	LOC_Os01g61830	35769751	TGTGTTCGTTGATGATGCTG	GCCCCTCATCCTGGTTTTTA	60.2	1095	96	zinc finger C-x8-C-x5-C-x3-H type family protein expressed
OsISM08791	LOC_Os01g61880	35812607	AGGAGAGCTTCGGCAAGTG	GAACTCCTTGGAGTCCCCC	60.8	631	45	respiratory burst oxidase putative expressed
OsISM08792	LOC_Os01g61880	35813325	GAGGAGATGACCGACCAGAA	ACCTCATCTTCCGTGAGCAT	59.9	204	93	respiratory burst oxidase putative expressed
OsISM08793	LOC_Os01g61880	35813459	TCCTTCCCCCTCTCTCTGAT	CGCCGACGCACTCAGTATAA	61.2	206	99	respiratory burst oxidase putative expressed
OsISM08794	LOC_Os01g61880	35814534	ATTCCTCACCAGGGAGTGGT	TCGCATGCATAGAAGAGCAC	60.4	162	73	respiratory burst oxidase putative expressed
OsISM08795	LOC_Os01g61880	35814689	CATGCGAGAGAACGATCAGA	GAGAGAGCACATTTCCTGGG	59.9	182	88	respiratory burst oxidase putative expressed
OsISM08796	LOC_Os01g61880	35814918	CTGTTTGTGAAGTGCCCTGA	GCAGAAGTGATGGAGAAGGG	59.8	149	60	respiratory burst oxidase putative expressed
OsISM08797	LOC_Os01g61880	35815107	GGTGACTGGACGACTGAACTC	AGAAGTAACCTGTGCCTCGC	59.6	185	61	respiratory burst oxidase putative expressed
OsISM08798	LOC_Os01g61880	35815315	TGAAACTACAGTTGTGGCGG	CACCATAGGGCCCATCAATA	60.2	168	75	respiratory burst oxidase putative expressed
OsISM08799	LOC_Os01g61880	35815524	ATTGGTCTTGGAATTGGTGC	CAGAACCATGTATGCTTTCCA	59.2	194	98	respiratory burst oxidase putative expressed
OsISM08800	LOC_Os01g61880	35815812	GAACGATGTCGCTGAAAGTG	AATCAAAGCTGACCTTGCGT	59.7	193	95	respiratory burst oxidase putative expressed
OsISM08801	LOC_Os01g61880	35816027	ATGCCAAAAATGGTGTGGAT	CGCCAAGTCAGAGAACACCT	60.3	176	90	respiratory burst oxidase putative expressed
OsISM08802	LOC_Os01g61880	35816183	AGGTGTTCTCTGACTTGGCG	TGTTTCGTGAGTGTAGGGGA	59.8	168	80	respiratory burst oxidase putative expressed
OsISM08804	LOC_Os01g61930	35846967	GAGAAGTGATGCTGGTGCAA	TTTGAAACACTGGGGTTCCT	59.7	201	63	SAC3/GANP family protein putative expressed
OsISM08805	LOC_Os01g61930	35847608	GTCGCAGTGCAAAAGAATGA	AGTACGAAGCGAAACAGGGA	59.9	389	85	SAC3/GANP family protein putative expressed
OsISM08806	LOC_Os01g61930	35847996	TTATGTTGGGCGGAATGTTT	TACCATCAGCAGTTGCCTTG	60.0	1091	99	SAC3/GANP family protein putative expressed

ISM IDs	Rice gene locus IDs	Physical positions (bp) on chromosomes	Forward primers (5'-3')	Reverse primers (5'-3')	Melting tempearature (T˚C)	Expected product size (bp) in PCR	Expected product size (bp) in Q-PCR	Putative Functions
OsISM08807	LOC_Os01g61930	35849121	GGTTCCTTTGCCAGAAAACA	GTTGAGAAGGAGAAGGGGCT	60.0	1134	76	SAC3/GANP family protein putative expressed
OsISM08808	LOC_Os01g61930	35850341	CCAAAGAATCAGCAAAGACCA	ACGGGATTGGAGAAACTTCC	60.3	170	93	SAC3/GANP family protein putative expressed
OsISM08809	LOC_Os01g61930	35850873	TGTCAAGGGAACATGTCAGG	GCTTTCTCTAAAACATCTTCTGGC	59.7	237	97	SAC3/GANP family protein putative expressed
OsISM08810	LOC_Os01g61930	35851158	CCAACTAAAATCAATTCGGCA	CTAAGCGCGCATGAGTTTC	59.8	855	87	SAC3/GANP family protein putative expressed
OsISM08811	LOC_Os01g61930	35852004	CGCGCTTAGCACTTCAATCT	CAGCCTTTGATTCCTTCTGC	60.3	554	89	SAC3/GANP family protein putative expressed
OsISM08812	LOC_Os01g61930	35852615	AAGAGACTTGCTTTCTTCAATGG	TGTCTTGCTTGGCTTCCTTT	59.5	174	51	SAC3/GANP family protein putative expressed
OsISM08814	LOC_Os01g62000	35889534	CAACAACTACACCAGGGGCT	TCGTAAATGTTGCACTGGGA	60.1	318	82	pectate lyase 4 precursor putative
OsISM08815	LOC_Os01g62000	35889852	GGCAGGGGAGAAGAAGAAAG	AACAGGTCGCCTTCAGACC	60.3	239	97	pectate lyase 4 precursor putative
OsISM08816	LOC_Os01g62010	35892241	GCATCTGAAGACATAGACGCA	GGTGGGATCGCTTATCAGG	59.7	195	91	hydrolase alpha/beta fold family domain containing protein expressed
OsISM08817	LOC_Os01g62010	35892335	GGCTGAGTTCCTCAACCTGT	TGTCACGGGAGTTCACAAAA	59.7	182	95	hydrolase alpha/beta fold family domain containing protein expressed
OsISM08818	LOC_Os01g62010	35892537	TGGACACCGCATAAACAAGA	TACCTCTCATCGATACGCCG	60.6	180	83	hydrolase alpha/beta fold family domain containing protein expressed
OsISM08819	LOC_Os01g62010	35892669	ATTCTTCTCCAGGAATCGCA	AATCCCGTAAACGGCATATC	59.3	165	90	hydrolase alpha/beta fold family domain containing protein expressed
OsISM08820	LOC_Os01g62010	35892907	GCGCAAACTTTTTCACGAGT	AGATGCCGTACAGGAACCTC	59.8	143	70	hydrolase alpha/beta fold family domain containing protein expressed
OsISM08821	LOC_Os01g62010	35893110	CCGTCTTGCTTGCTCCTATG	AGCGCACACCATTTTCAGAG	61.1	241	84	hydrolase alpha/beta fold family domain containing protein expressed
OsISM08822	LOC_Os01g62010	35893369	ATCATACCCACACCGGACAT	GTGGGAGCTTTCCCCTGTAT	60.1	183	98	hydrolase alpha/beta fold family domain containing protein expressed
OsISM08823	LOC_Os01g62010	35893566	GAACTCCTGATGGTCAGCCT	GAAGGATCGGTCACGATGTC	59.9	291	99	hydrolase alpha/beta fold family domain containing protein expressed
OsISM08824	LOC_Os01g62020	35896538	CGTCCCCTGTGCACTACAAG	TGCCAGACCCAACATATTCA	60.8	868	81	NAD dependent epimerase/dehydratase family domain containing protein expressed
OsISM08825	LOC_Os01g62020	35897479	AGGAGACTTACTGGGGGCAT	TTTCCCCTCGTCATAACAGC	60.0	151	60	NAD dependent epimerase/dehydratase family domain containing protein expressed

ISM IDs	Rice gene locus IDs	Physical positions (bp) on chromosomes	Forward primers (5'-3')	Reverse primers (5'-3')	Melting tempearature (T˚C)	Expected product size (bp) in PCR	Expected product size (bp) in Q-PCR	Putative Functions
OsISM08826	LOC_Os01g62020	35897623	GGGGAAAAGAACAGCAGAGA	TCGAGGGCCATATGTATTGA	59.2	579	95	NAD dependent epimerase/dehydratase family domain containing protein expressed
OsISM08827	LOC_Os01g62020	35898218	GCCGTGTGGTTAGCAATTTT	CTGTCATAGGCTGTCTACGCAA	60.2	149	52	NAD dependent epimerase/dehydratase family domain containing protein expressed
OsISM08828	LOC_Os01g62020	35898339	TAGGCATTGCGTAGACAGCC	ATCAGAGCCATCAGTCCAGC	60.9	532	99	NAD dependent epimerase/dehydratase family domain containing protein expressed
OsISM08829	LOC_Os01g62020	35898879	ATCACATTGGCCCTTTCAAC	CCATTGGGTCAATTGTTTCC	59.9	162	88	NAD dependent epimerase/dehydratase family domain containing protein expressed
OsISM08830	LOC_Os01g62040	35902065	CACCCACACCCACATCAAG	CCCGAAGCCAGCCCTATC	61.9	213	64	ruvB-like putative expressed
OsISM08831	LOC_Os01g62040	35902408	GGCATCTCTCAGGAGCTAGG	TCCCACCATAGGACAGAAGG	59.5	1131	49	ruvB-like putative expressed
OsISM08832	LOC_Os01g62040	35903594	GTCGAGCTATAGGCTTGCGT	ACTCTCAGCTTCTTCTGGCG	59.8	230	84	ruvB-like putative expressed
OsISM08833	LOC_Os01g62040	35903890	AAGCAGCTGAAGTTAGATCCTACAA	TCACTGCACCACTATTAGCTTCA	60.0	421	98	ruvB-like putative expressed
OsISM08834	LOC_Os01g62040	35904375	GATTCCCAAAGGAGAGGTCC	CCTCCTTGTGGTTGAGCATT	60.0	835	91	ruvB-like putative expressed
OsISM08835	LOC_Os01g62040	35905271	AAGCTTCGGCAAGAAATCAA	CAAGCTCTGCAATTCCTTCA	59.6	173	62	ruvB-like putative expressed
OsISM08836	LOC_Os01g62040	35905433	TGCAGAGCTTGTACCTGGTG	GGCTCTCCAGAGCACGATTA	60.3	336	90	ruvB-like putative expressed
OsISM08837	LOC_Os01g62040	35905761	GGAGAGCCCATTATCACCAA	CCATGCGGACTTGTCATATC	59.4	222	85	ruvB-like putative expressed
OsISM08838	LOC_Os01g62040	35906030	CATACGGTCCCACTGAGATG	CCGATCTCTCCTAAAAATGCAA	59.8	178	98	ruvB-like putative expressed
OsISM08839	LOC_Os01g62040	35906197	GGAGAGATCGGACAACAGACA	TCCCATTAGCCTTTGCAACT	60.0	580	80	ruvB-like putative expressed
OsISM08840	LOC_Os01g62040	35906758	GTTGCAAAGGCTAATGGGAG	CCGACGATTTAGCATCCAAG	60.2	218	83	ruvB-like putative expressed
OsISM08841	LOC_Os01g62060	35911274	GCCGGCTTCGTACATCAG	CTCCATGCATTCCTCCTTGT	60.2	215	80	plant-specific domain TIGR01589 family protein expressed
OsISM08842	LOC_Os01g62060	35911469	ACAAGGAGGAATGCATGGAG	CGCTGTTCTCCTTCTCAAGC	60.2	441	94	plant-specific domain TIGR01589 family protein expressed
OsISM08843	LOC_Os01g62100	35939213	TTTTTGAGTCTTTCCTTAAGTGCAT	GGTAAAGGGAACAGCAGCAC	59.7	145	93	retrotransposon protein putative unclassified

ISM IDs	Rice gene locus IDs	Physical positions (bp) on chromosomes	Forward primers (5'-3')	Reverse primers (5'-3')	Melting tempearature (T˚C)	Expected product size (bp) in PCR	Expected product size (bp) in Q-PCR	Putative Functions
OsISM08844	LOC_Os01g62100	35939338	GTGCTGCTGTTCCCTTTACC	GTACGGGCACAGAAGATGTG	59.5	200	63	retrotransposon protein putative unclassified
OsISM08845	LOC_Os01g62100	35939494	TCTTATTCCCTGCAGGTTGC	TCTTTCCACCCAGGAAAAGAT	60.1	325	82	retrotransposon protein putative unclassified
OsISM08846	LOC_Os01g62100	35939859	AGCTGCCAGCGAATATGTTT	CGATACTCAGGAGGGCCATA	60.0	523	88	retrotransposon protein putative unclassified
OsISM08847	LOC_Os01g62100	35940947	ATTGTAGGAGGAAGGGCCTG	AAGGCCATGTAAGCAGGATG	60.3	168	78	retrotransposon protein putative unclassified
OsISM08848	LOC_Os01g62100	35941688	TATGGCTGGTGTTCCATTTG	CACGCCACCAGTATATGCAC	59.7	195	84	retrotransposon protein putative unclassified
OsISM08849	LOC_Os01g62100	35942493	TGATATTCATTTTAGAAACCCTTCTG	TCCTCATAACTTCTAGCGCCTT	59.3	353	91	retrotransposon protein putative unclassified
OsISM08850	LOC_Os01g62100	35943345	CGTATTGTGCAAAGCGTCAT	TTTTAAACCGGGAATGAGCA	60.1	198	95	retrotransposon protein putative unclassified
OsISM08851	LOC_Os01g62100	35944316	CCTGATCATCAATTGTGCTTAAA	AACCAGTTATGATACGCGCC	59.6	145	62	retrotransposon protein putative unclassified
OsISM08852	LOC_Os01g62100	35944657	CGTTGCTCTTTCTCGTGTCA	GATCTTCCAGCCTCTGCTTG	60.1	415	92	retrotransposon protein putative unclassified
OsISM08853	LOC_Os01g62100	35945110	AGCTGACGATCTGCCTCCTA	CGCCTGGAATTTCAGCATAC	60.4	165	81	retrotransposon protein putative unclassified
OsISM08854	LOC_Os01g62100	35945477	ATATCTAAGCCTGCGCGTTG	TTTAGACGCCATTCGGACAT	60.4	190	98	retrotransposon protein putative unclassified
OsISM08855	LOC_Os01g62100	35945649	GTCCGAATGGCGTCTAAAAC	TGCTCTCCCCATAGGACAAG	59.9	212	96	retrotransposon protein putative unclassified
OsISM08856	LOC_Os01g62100	35945898	GGCCAAGATAATGCTGTCGT	GCAGGCTGATCCTCCACTAA	60.2	216	88	retrotransposon protein putative unclassified
OsISM08857	LOC_Os01g62100	35946102	GGATCAGCCTGCAAATGGTA	CCACTGGATCTTTGGAGCAC	60.8	181	91	retrotransposon protein putative unclassified
OsISM08858	LOC_Os01g62100	35946361	AAGATTGATCCATGGGAAGC	TCTGTGAAACAACAGCAGCA	59.1	520	71	retrotransposon protein putative unclassified
OsISM08859	LOC_Os01g62100	35946911	GGAGCCTTATGCACAGGAAT	AGAAGCAGCAAGAGCCAAAG	59.5	137	96	retrotransposon protein putative unclassified
OsISM08860	LOC_Os01g62100	35947141	GACAGCAAGAAGCGGAAATC	GGATGGAGCTGTTTCAAAGG	59.8	161	76	retrotransposon protein putative unclassified
OsISM08861	LOC_Os01g62100	35947292	AGCTCCATCCAGCAAAGAAA	CTTGGTCATCAGTGCGAGAA	60.0	705	69	retrotransposon protein putative unclassified

ISM IDs	Rice gene locus IDs	Physical positions (bp) on chromosomes	Forward primers (5'-3')	Reverse primers (5'-3')	Melting tempearature (T°C)	Expected product size (bp) in PCR	Expected product size (bp) in Q-PCR	Putative Functions
OsISM08862	LOC_Os01g62100	35947984	ACTGATGACCAAGCTGCTGA	TTTTGGCTTCAGTTGGTAGGA	59.7	211	74	retrotransposon protein putative unclassified
OsISM08863	LOC_Os01g62100	35948136	TGGCAACCTAATTATTCCCG	GTCTTGCGTGTGCCTGTAGA	59.9	265	97	retrotransposon protein putative unclassified
OsISM08864	LOC_Os01g62150	35980702	TCCCCTATACTGCAAATCCG	GTTCTTCACCGAAGGACAGG	59.8	310	55	clathrin adaptor complex small chain domain containing protein expressed
OsISM08865	LOC_Os01g62150	35981116	TAAAACCCAGAAAACGAGCG	AAAAGTGTAGATCTTGGATGAACTTG	59.7	738	93	clathrin adaptor complex small chain domain containing protein expressed
OsISM08866	LOC_Os01g62150	35981891	AGCATCTGTTCTTCAGGGCT	ATCCAAGTTTTCAAGCGCAG	59.7	192	86	clathrin adaptor complex small chain domain containing protein expressed
OsISM08867	LOC_Os01g62150	35982063	CTGCGCTTGAAAACTTGGAT	AGAATCGCCTTTGCATCTGT	60.1	522	89	clathrin adaptor complex small chain domain containing protein expressed
OsISM08868	LOC_Os01g62150	35982611	AAGGATCCGGGTCTCTCACT	GTTCTCTTGCCGTTGCTAGG	60.0	185	58	clathrin adaptor complex small chain domain containing protein expressed
OsISM08869	LOC_Os01g62170	35992960	GCTCAGAAAGATCGGATGGT	TTCATTAGCTTGCTTAGCACATT	58.8	1137	77	hypothetical protein
OsISM08870	LOC_Os01g62170	35994023	TTGTTAGTGGCTGGTTGCAG	CCGTGACAACCTCCAAGTTC	60.2	359	53	hypothetical protein
OsISM08871	LOC_Os01g62210	36006473	CCCACCGAGTATCCATGC	GAGTACGTACCTTGGCGACG	60.3	175	85	50S ribosomal protein L17 putative expressed
OsISM08872	LOC_Os01g62210	36006614	CGAATCGAGACCACCGTC	TTTACCTCTTTCCCGAGCTG	59.8	176	78	50S ribosomal protein L17 putative expressed
OsISM08873	LOC_Os01g62210	36006714	GGAGTCGTGGTTGATTCCAG	GAAGCACGTCTTGCTGCAT	60.3	975	98	50S ribosomal protein L17 putative expressed
OsISM08874	LOC_Os01g62210	36007697	TTCGGGGAGATGATGTTGTT	TCTGGTGTACCCACCAGCTC	60.7	327	78	50S ribosomal protein L17 putative expressed
OsISM08875	LOC_Os01g62210	36008039	ATACGAGTTGGTGATGCTGC	GTGTTGCAGGTTTGGCTTCT	59.5	484	83	50S ribosomal protein L17 putative expressed
OsISM08876	LOC_Os01g62280	36037946	CTAGAAGGAAGGGCCGAATC	TTCGCATCCCTCAACAGTAA	59.7	228	93	hypothetical protein
OsISM08877	LOC_Os01g62290	36038986	CCATGAACCCCATCAACAC	CTTCATGTCACTCTGCACGG	60.0	1265	84	DnaK family protein putative expressed
OsISM08878	LOC_Os01g62320	36068245	AGCCGATGGTTCAATAGCTG	AATTTGGGGTGTCTGCTGAA	60.4	367	99	retrotransposon protein putative unclassified
OsISM08879	LOC_Os01g62320	36068773	CCAAGGTTTAGTGAGCGCAT	CAGGAACAACCTGAGGCATT	60.2	140	59	retrotransposon protein putative unclassified

ISM IDs	Rice gene locus IDs	Physical positions (bp) on chromosomes	Forward primers (5'-3')	Reverse primers (5'-3')	Melting tempearature (T°C)	Expected product size (bp) in PCR	Expected product size (bp) in Q-PCR	Putative Functions
OsISM08880	LOC_Os01g62320	36069588	AGTTAGCCGATTTGGCCTTT	AGCTCTTCCTAACTTCCGCA	59.7	138	45	retrotransposon protein putative unclassified
OsISM08881	LOC_Os01g62320	36070142	TAGCCAAACAACCGATAGGG	ATGCTTCTCCTGCTGCTTGT	60.1	103	68	retrotransposon protein putative unclassified
OsISM08882	LOC_Os01g62320	36070267	TTGATCCTCATTGGGGTTGT	CCATTGATTCTTCCTGACCA	59.3	227	75	retrotransposon protein putative unclassified
OsISM08883	LOC_Os01g62320	36071267	TTGGGAGATGGATAGCCTTG	CACGACGCCCTCAAAATAAT	60.0	128	95	retrotransposon protein putative unclassified
OsISM08884	LOC_Os01g62330	36073348	TCGGGATATAAGCCGATGAT	TTCTGACAATCTTGACACCCC	59.7	371	96	retrotransposon protein putative Ty3-gypsy subclass
OsISM08885	LOC_Os01g62370	36094253	CGCTCACCGATGTTACCC	AGGCTCATCGTCTTCACGTT	60.0	202	86	ENTH domain containing protein expressed
OsISM08886	LOC_Os01g62370	36094473	CGAGATCGACGAGTACGTCA	GGAGGAGGAGGGCCTTGTA	60.8	219	99	ENTH domain containing protein expressed
OsISM08887	LOC_Os01g62370	36094753	GGTCATCGAGCAGATGGTCT	CTTGCTCTTCACCGTCATCC	60.5	178	74	ENTH domain containing protein expressed
OsISM08888	LOC_Os01g62400	36116263	GACCACGGGTTCTGCAAG	AACCCTTCTGCCTCATTTCC	60.3	906	97	hypothetical protein
OsISM08889	LOC_Os01g62400	36117141	TTGATCATGGAAATGAGGCA	GACCATGTCATCGAGCAGAG	59.7	129	75	hypothetical protein
OsISM08890	LOC_Os01g62410	36122339	CTAGCTCGGCGAATTCCAT	GAGGTGTTGACCTCCTCTGG	60.0	1204	92	MYB family transcription factor putative expressed
OsISM08891	LOC_Os01g62410	36123492	GCCAATAAGGAGAGCAAAGG	CCTCAACTGCCTTCCTCAAT	59.1	422	66	MYB family transcription factor putative expressed
OsISM08892	LOC_Os01g62410	36123886	GACGAGACATTGAGGAAGGC	CGATGCAAGCATTGTACCTC	59.6	506	99	MYB family transcription factor putative expressed
OsISM08893	LOC_Os01g62410	36124387	CATCGATGGCAAAAGGTTCT	TGGTCCATACTTCTTTACAAGATCA	59.6	184	97	MYB family transcription factor putative expressed
OsISM08894	LOC_Os01g62410	36124605	GCCGTATAGGGAAGCAATGT	GAGTGCCTGTTCCTCCTCAG	59.5	647	93	MYB family transcription factor putative expressed
OsISM08895	LOC_Os01g62410	36125272	TGGGAATAAATGGGCAGAGAT	TCTTTCTGAGAGAACTATTCCAATGA	60.2	372	87	MYB family transcription factor putative expressed
OsISM08896	LOC_Os01g62420	36128495	ATGGCTGCCAGGAAGTTCT	GTTGAGGACGGTGACGATCT	60.0	206	85	triosephosphate isomerase cytosolic putative expressed
OsISM08897	LOC_Os01g62420	36128681	AGATCGTCACCGTCCTCAAC	CAAACCCTTCACCTGCGG	61.4	212	99	triosephosphate isomerase cytosolic putative expressed

ISM IDs	Rice gene locus IDs	Physical positions (bp) on chromosomes	Forward primers (5'-3')	Reverse primers (5'-3')	Melting tempearature (T˚C)	Expected product size (bp) in PCR	Expected product size (bp) in Q-PCR	Putative Functions
OsISM08899	LOC_Os01g62420	36129403	TTGGGTGATTCTGGGACACT	CGTACGCAATTTTGTCAGCA	60.6	396	78	triosephosphate isomerase cytosolic putative expressed
OsISM08900	LOC_Os01g62420	36129881	TGCAGCGCAAACTAAAGCTA	CCCAAACTGGTTCATATGCC	60.1	580	75	triosephosphate isomerase cytosolic putative expressed
OsISM08901	LOC_Os01g62420	36130441	GGCATATGAACCAGTTTGGG	CAAGCCACTTTCTCAGACCA	59.6	267	90	triosephosphate isomerase cytosolic putative expressed
OsISM08902	LOC_Os01g62420	36130725	GCAGTTGCTGAATCAACCAG	TGCAGTTTGCTCCATTTACG	59.7	202	56	triosephosphate isomerase cytosolic putative expressed
OsISM08903	LOC_Os01g62420	36130955	TGGATTCCTTGTTGGAGGAG	GCAACCCAGAACACTAAGCAG	60.0	312	93	triosephosphate isomerase cytosolic putative expressed
OsISM08904	LOC_Os01g62430	36135070	GCAGGCAAGATAGACCCGTA	CAGCTCGGGTTCTTCCCT	60.3	210	90	C2 domain containing protein putative expressed
OsISM08905	LOC_Os01g62430	36135350	ATGGACCACGACACCTTCTC	TGCTCCATGCCTAAGCTGAT	60.4	383	84	C2 domain containing protein putative expressed
OsISM08906	LOC_Os01g62430	36135802	CAGAGTCAGCCTCACGTTCA	AACCTGTTCTGCGTGGTCTT	60.0	182	56	C2 domain containing protein putative expressed
OsISM08908	LOC_Os01g62520	36207332	TGGATCTAAAGATGAAGATGAGG	CGCCCTAAAGCATCTTTCAA	58.9	589	79	retrotransposon protein putative unclassified
OsISM08909	LOC_Os01g62520	36207921	TGTTAAACACATGGAGGAAATTG	CCTCTCCTAGCAACACCCAA	59.6	282	99	retrotransposon protein putative unclassified
OsISM08910	LOC_Os01g62520	36208173	TCCTACTGTTTTGGGTGTTGC	CCGTGGTCCTCGAATTAAAA	60.0	323	97	retrotransposon protein putative unclassified
OsISM08911	LOC_Os01g62520	36208476	TTTTAATTCGAGGACCACGG	CCTCAAACATTAGCACATGAAA	58.9	473	93	retrotransposon protein putative unclassified
OsISM08912	LOC_Os01g62520	36208926	TTTTCATGTGCTAATGTTTGAGGT	TTCTCTTTTTCACAATGAGCAAT	59.0	546	76	retrotransposon protein putative unclassified
OsISM08913	LOC_Os01g62540	36213298	GAGGAGGGAGTCGAGGAAGT	ATGAGGAACATGGCCTCCAC	61.0	209	97	hypothetical protein
OsISM08914	LOC_Os01g62540	36213571	CGCCTGCTCGTCTACAGAAT	ATGCCTCGATTGTTCTCCAG	60.4	195	77	hypothetical protein
OsISM08915	LOC_Os01g62540	36213932	GGAGTCTGTGCGCTCGAT	TGTAAGCTATTGCAACCTTTATTTC	59.1	1119	99	hypothetical protein
OsISM08916	LOC_Os01g62570	36221386	AAGTCCAGCCTCGTCGTGT	ATCAGCTTCGACCTCTGCTC	60.3	247	69	ATP/GTP/Ca++ binding protein putative
OsISM08917	LOC_Os01g62570	36221701	GGCTCAGCTCATTCTGGTTC	TCAGGTCCAGCTTACATCCC	60.0	175	82	ATP/GTP/Ca++ binding protein putative

ISM IDs	Rice gene locus IDs	Physical positions (bp) on chromosomes	Forward primers (5'-3')	Reverse primers (5'-3')	Melting tempearature (T°C)	Expected product size (bp) in PCR	Expected product size (bp) in Q-PCR	Putative Functions
OsISM08918	LOC_Os01g62570	36221943	GACCTGCATTGAGTGCTCTG	AGGAGCTGTTGGGTAAAGCA	59.7	981	89	ATP/GTP/Ca++ binding protein putative
OsISM08919	LOC_Os01g62570	36222999	CATGACAGGGATGGAGCTTT	GGTTGGAGAGGAGCATTGAA	60.1	197	75	ATP/GTP/Ca++ binding protein putative
OsISM08920	LOC_Os01g62570	36223385	CATTCCAGCAATTAAACGAGC	AAGAGTGCGGATATTTTACCACA	59.8	297	96	ATP/GTP/Ca++ binding protein putative
OsISM08921	LOC_Os01g62570	36223625	CACCCTTTGGTATTTTCATCA	ATTTCAGCAGGTTGCAAAGC	58.9	463	62	ATP/GTP/Ca++ binding protein putative
OsISM08922	LOC_Os01g62570	36224055	TATCCAGGATGGAGCTTTGC	CAGCACAGTTCTCGTACAGATG	59.4	317	96	ATP/GTP/Ca++ binding protein putative
OsISM08923	LOC_Os01g62570	36224380	GTCTTGGGTGGTTTGTCGTT	TGGCAAAACTATTTGACGGG	60.4	621	80	ATP/GTP/Ca++ binding protein putative
OsISM08924	LOC_Os01g62570	36225143	TGCATTGCTACAATCATTCCTT	CCATTGACAGGAGGAGCATC	60.1	148	99	ATP/GTP/Ca++ binding protein putative
OsISM08925	LOC_Os01g62570	36225272	ATGCTCCTCCTGTCAATGGT	CGAATACATGAGTAAATGTTGCAC	59.1	152	92	ATP/GTP/Ca++ binding protein putative
OsISM08926	LOC_Os01g62570	36225356	TGCTCACACCATATGCAACC	TTCTCGCATCACAAGTGTCTTT	60.2	290	95	ATP/GTP/Ca++ binding protein putative
OsISM08927	LOC_Os01g62570	36225690	TTTGGCACCCTGTGATGTAG	GCTCTTTGCCAAGAAACTTCA	59.6	873	64	ATP/GTP/Ca++ binding protein putative
OsISM08928	LOC_Os01g62570	36226636	GCTAAGGATGACCTCGACCA	GGTGTTTCGATTCCCATGTC	60.2	443	78	ATP/GTP/Ca++ binding protein putative
OsISM08929	LOC_Os01g62570	36227206	CTTCTGAACCGTTCCCTCAC	GATGACGCGTTCTTCCTAGC	59.8	330	99	ATP/GTP/Ca++ binding protein putative
OsISM08930	LOC_Os01g62584	36229657	GAGTTTGCGCATCATCGTG	AGCTGGCTGTTGGTGAGAAT	60.6	557	67	peptidase aspartic family protein putative expressed
OsISM08932	LOC_Os01g62600	36235901	GGCCTTACCCGTCACTACAC	GATGCTCTTGGTCACGCAC	60.0	172	70	laccase precursor protein putative
OsISM08933	LOC_Os01g62600	36236139	AAGGTGCACAACCACATGAA	CACTGCGTGATGTACGACG	60.0	210	99	laccase precursor protein putative
OsISM08934	LOC_Os01g62600	36236508	CTACAAGGAGCTGCCTCCC	AGATATTAGGGCCTGCTCCC	59.7	181	99	laccase precursor protein putative
OsISM08935	LOC_Os01g62600	36236692	ACGCCTACACTTTCAATGGG	CTGCACCTTCACCTTGTACG	59.7	159	75	laccase precursor protein putative
OsISM08936	LOC_Os01g62600	36237379	AACAACAACTCCTTCGTCCG	CAGCTCCACCGACGTGTT	60.5	198	94	laccase precursor protein putative

ISM IDs	Rice gene locus IDs	Physical positions (bp) on chromosomes	Forward primers (5'-3')	Reverse primers (5'-3')	Melting tempearature (T˚C)	Expected product size (bp) in PCR	Expected product size (bp) in Q-PCR	Putative Functions
OsISM08938	LOC_Os01g62610	36254618	AGGAGAGCAAAGGATGATGC	TCAAACACTTCCCCGTTCTC	59.7	194	96	peptidyl-prolyl cis-trans isomerase FKBP-type putative expressed
OsISM08939	LOC_Os01g62610	36254863	TGTCATCAAGGCATGGGATA	GAACCTGCAGCTCCATAAGC	59.9	190	97	peptidyl-prolyl cis-trans isomerase FKBP-type putative expressed
OsISM08940	LOC_Os01g62610	36255050	TTCACCTCCAGAGATACCGC	CTAGAACCTTTCCTCGGCCT	60.0	179	79	peptidyl-prolyl cis-trans isomerase FKBP-type putative expressed
OsISM08941	LOC_Os01g62610	36255210	GGCCGAGGAAAGGTTCTAGT	AGCGATCTCCCTCTGCTTCT	60.2	322	81	peptidyl-prolyl cis-trans isomerase FKBP-type putative expressed
OsISM08942	LOC_Os01g62650	36278981	GATGGGCTGATTGGTTTGTC	AGAGGGATGCTCTGAAGCAG	60.0	981	97	pumilio-family RNA binding protein putative expressed
OsISM08943	LOC_Os01g62650	36281435	ATTGCCAGTCATGTGGTTGA	ATGAAACGGCTTCCGTATTG	60.0	639	51	pumilio-family RNA binding protein putative expressed
OsISM08944	LOC_Os01g62650	36282137	TCAGGCTCTCTCGTTGATGA	TTGATCAGCCAGCTCCCTAC	60.0	231	98	pumilio-family RNA binding protein putative expressed
OsISM08945	LOC_Os01g62650	36282396	TTCAGATGTATGGATGCCGA	CTGGTCCAGGTCAACCACTT	60.0	143	57	pumilio-family RNA binding protein putative expressed
OsISM08946	LOC_Os01g62650	36282698	CATCCCTATGGCTGCAGAGT	TGTGTTTTTGGATCAGTGCAA	60.2	148	60	pumilio-family RNA binding protein putative expressed
OsISM08947	LOC_Os01g62650	36282869	TGCAATCTGTGTGCATGTTG	CTCATGAGGCTTCCCGTG	60.3	915	81	pumilio-family RNA binding protein putative expressed
OsISM08948	LOC_Os01g62650	36283917	TCGGAACTACTGACGAGAATG A	GCTGATCATCACAAGTCTCCAG	59.9	722	97	pumilio-family RNA binding protein putative expressed
OsISM08949	LOC_Os01g62650	36284699	ATGGGAAGCACATAGTTGCC	AGGATATTGTGCCTGAAGCC	59.6	220	78	pumilio-family RNA binding protein putative expressed
OsISM08950	LOC_Os01g62710	36312116	AGGAGAAGAGAAGAAGGGGC	CCGATATCTACAATACGCACTTC	58.5	185	94	transposon protein putative unclassified
OsISM08951	LOC_Os01g62710	36312264	GTTGTACTAATCTAGAAGTGC GTATTG	GTAACGGACGTTTCCTGGTC	58.3	218	90	transposon protein putative unclassified
OsISM08952	LOC_Os01g62710	36312572	TGGTTAATGAGGGGCTTGAG	CATCATCCATGTTCGTCTGC	60.1	223	89	transposon protein putative unclassified
OsISM08953	LOC_Os01g62710	36313723	AGGAAAGTATCGGTGCCAGA	GGCCCATTTCTCTTTTGGTT	60.0	575	86	transposon protein putative unclassified
OsISM08954	LOC_Os01g62720	36316318	AGGTGCCCTAACTATGACCG	GGTTAGGTCGAGAACGGGAT	59.7	169	88	GRF zinc finger family protein
OsISM08955	LOC_Os01g62720	36316692	TGGGTCGAAGGTTCTTTGTT	TGTATTGACATCTCCTTGGTGG	59.7	160	73	GRF zinc finger family protein

ISM IDs	Rice gene locus IDs	Physical positions (bp) on chromosomes	Forward primers (5'-3')	Reverse primers (5'-3')	Melting tempearature (T°C)	Expected product size (bp) in PCR	Expected product size (bp) in Q-PCR	Putative Functions
OsISM08956	LOC_Os01g62740	36322797	CCAGTTGGTATGGAGGAACC	ACGTTTTGGACCTGATCTGC	59.7	388	62	expressed protein
OsISM08957	LOC_Os01g62760	36343634	TCCATTTTTACGGCGTCTTC	AATCTCGTGCATCCTGTCCT	59.9	168	75	protein phosphatase 2C putative expressed
OsISM08958	LOC_Os01g62760	36344071	CGTCGCACTATCCGTTGAC	CCTCGATTCGTTCCAGCTC	60.6	184	57	protein phosphatase 2C putative expressed
OsISM08959	LOC_Os01g62760	36344269	GTCATTTACTGGGACGGCG	CGTACGGCTTAAGGTACCCA	60.7	175	80	protein phosphatase 2C putative expressed
OsISM08961	LOC_Os01g62780	36354429	TTGAGGGAATTAATGAGAGTGC	ACAAATTGCACATATCCGGC	59.5	377	57	hypothetical protein
OsISM08962	LOC_Os01g62780	36354932	TGTGCGTTTTGGTGATATGG	GGTCCCAGTTTTGGGATCAT	60.7	164	77	hypothetical protein
OsISM08963	LOC_Os01g62780	36355078	GATCCCAAAACTGGGACCTT	GAAGGATTGCAGGCTCACA	60.1	167	83	hypothetical protein
OsISM08964	LOC_Os01g62780	36355264	TGAAGGGAACATTGAAGAAGG	TGTTCCTCATCATACACCGTC	58.8	130	88	hypothetical protein
OsISM08965	LOC_Os01g62780	36355372	TGACGGTGTATGATGAGGAA	GCTCTAGCGGCATTATCAGG	58.7	1021	71	hypothetical protein
OsISM08966	LOC_Os01g62790	36360166	AATCTATTCAGCTGGGGCCT	TGATACGAAGGATCCAAAAGG	59.5	1348	59	expressed protein
OsISM08967	LOC_Os01g62790	36361605	CCCTGAGAGAAGTAAGGAGAGC	CTGAAGGACTGGAACTCTTGC	58.9	425	87	expressed protein
OsISM08968	LOC_Os01g62790	36362121	CTTTGGATGATCGCTTTGGT	ATGTTTTGAGCTTTGGCCC	60.1	138	62	expressed protein
OsISM08969	LOC_Os01g62790	36362269	AAAAATGGAACCCTGAATTCCT	AGCGGTGGTAATATTGCAGG	60.0	177	84	expressed protein
OsISM08970	LOC_Os01g62810	36378982	GATGGGTGGAGGGACGTAT	ATCATCAGCAGAACCCCAAG	59.8	944	91	regulator of chromosome condensation putative expressed
OsISM08971	LOC_Os01g62810	36379917	GCTGATGATATGGGCCAAAG	CAGCTCTCACTATTGCCACG	60.0	181	95	regulator of chromosome condensation putative expressed
OsISM08972	LOC_Os01g62810	36380078	CGTGGCAATAGTGAGAGCTG	ACACATTCTTTCCAACCCCA	59.9	190	91	regulator of chromosome condensation putative expressed
OsISM08973	LOC_Os01g62810	36380324	AAAGACAGAGTGCGATTGCC	GATGCTGCTCCGCTACTTGT	60.5	177	77	regulator of chromosome condensation putative expressed
OsISM08974	LOC_Os01g62810	36380660	TGCTGGTGGTCGTCATACAT	CCTAGTTGCCCTTCTCCTCC	60.1	174	85	regulator of chromosome condensation putative expressed

ISM IDs	Rice gene locus IDs	Physical positions (bp) on chromosomes	Forward primers (5'-3')	Reverse primers (5'-3')	Melting tempearature (T˚C)	Expected product size (bp) in PCR	Expected product size (bp) in Q-PCR	Putative Functions
OsISM08975	LOC_Os01g62810	36380963	CATGGGAAATTGCGTGAAG	CGTACAGTCCCCATCCAAAT	59.9	711	96	regulator of chromosome condensation putative expressed
OsISM08976	LOC_Os01g62810	36381654	ATTTGGATGGGGACTGTACG	TGCATCTTGACTCCCAGGAT	60.1	577	94	regulator of chromosome condensation putative expressed
OsISM08977	LOC_Os01g62810	36382278	GCGATGTCTATTCCTTCGGA	CAAGCTTGAGGCATCTACCA	59.6	800	96	regulator of chromosome condensation putative expressed
OsISM08978	LOC_Os01g62810	36383105	TGTGGAGCTCGTCATAGTGC	GTGCAGAGCTGAAATACGCA	60.1	617	94	regulator of chromosome condensation putative expressed
OsISM08979	LOC_Os01g62810	36383656	GATGAAGGCGAGGTCTTCTG	GTTTCTGTCCATGGAATCGC	60.2	157	73	regulator of chromosome condensation putative expressed
OsISM08980	LOC_Os01g62830	36391271	CTTCGTCTTCAGGCTTCCAC	GCACTTGCATATGCGTACCT	59.4	448	69	expressed protein
OsISM08981	LOC_Os01g62840	36394963	GAACAAGCCCATGATTCTGC	TTACCTCCGGTCGGTAGTTG	60.3	579	90	mannose-1-phosphate guanyltransferase putative expressed
OsISM08982	LOC_Os01g62840	36395513	TTTAGCCATCAACTACCGACC	CTTGGGAACACGTGATTGTG	59.6	179	87	mannose-1-phosphate guanyltransferase putative expressed
OsISM08983	LOC_Os01g62840	36395843	TGAGGCAACGATTATGGTCA	CACCATTCCAGTGACCTCCT	60.0	157	80	mannose-1-phosphate guanyltransferase putative expressed
OsISM08985	LOC_Os01g62860	36402860	TGCGCCAGAATGTACAAAAA	TCTTCACGCTTGACAACACC	60.1	572	81	oxidoreductase aldo/keto reductase family protein putative expressed
OsISM08986	LOC_Os01g62860	36403411	AGGTGTTGTCAAGCGTGAAG	TGACTCAGGCACATCCTCTG	59.5	200	80	oxidoreductase aldo/keto reductase family protein putative expressed
OsISM08987	LOC_Os01g62860	36403590	CCAGAGGATGTGCCTGAGTC	CCCTTCTTGACTCTGAATGGC	61.0	753	99	oxidoreductase aldo/keto reductase family protein putative expressed
OsISM08988	LOC_Os01g62860	36404566	CAGTCAACTGGCGTTCATCT	CAGGTGAACCTAGAGGCGAG	59.4	159	47	oxidoreductase aldo/keto reductase family protein putative expressed
OsISM08989	LOC_Os01g62860	36404901	TGGTCTATTCCAGAGGACTTGC	GATTAACAATGACGTCGCCC	60.5	1146	80	oxidoreductase aldo/keto reductase family protein putative expressed
OsISM08990	LOC_Os01g62870	36410024	GTCGGCAACGCCGTCTAC	CCTTTTCGTTGTTGTACGCA	61.3	190	77	oxidoreductase aldo/keto reductase family protein putative expressed
OsISM08991	LOC_Os01g62870	36410190	CCAGTGCGTACAACAACGAA	ACAGATCTTCGCGTTTGACC	60.5	870	82	oxidoreductase aldo/keto reductase family protein putative expressed
OsISM08992	LOC_Os01g62870	36411029	GAAGAGGGTGTGGTCAAACG	ACATCCTCTGGGGCATGAT	60.4	195	75	oxidoreductase aldo/keto reductase family protein putative expressed
OsISM08993	LOC_Os01g62870	36411225	CCTGAGGCACTAGATGCTACC	TGGATCCCTTCTTGGTTCTG	59.5	617	92	oxidoreductase aldo/keto reductase family protein putative expressed

ISM IDs	Rice gene locus IDs	Physical positions (bp) on chromosomes	Forward primers (5'-3')	Reverse primers (5'-3')	Melting tempearature (T°C)	Expected product size (bp) in PCR	Expected product size (bp) in Q-PCR	Putative Functions
OsISM08994	LOC_Os01g62870	36412060	CAGTCAACGGGCATTCATC	CCTTAAGGACATTGCCGTTC	59.8	201	80	oxidoreductase aldo/keto reductase family protein putative expressed
OsISM08995	LOC_Os01g62870	36412411	TTCCAGAGGACTTGCTTGCT	ACAATGAAGTTGCCCCTGAG	60.1	874	68	oxidoreductase aldo/keto reductase family protein putative expressed
OsISM08996	LOC_Os01g62880	36417282	GGCCTCGGCACCTACAAG	CGCAGTCTATGTGCCGGT	61.3	179	86	oxidoreductase aldo/keto reductase family protein putative expressed
OsISM08997	LOC_Os01g62880	36417437	CAGGGTACCGGCACATAGAC	AGCTTCTTCAGAGCACCACC	60.0	182	71	oxidoreductase aldo/keto reductase family protein putative expressed
OsISM08998	LOC_Os01g62880	36417617	CTCTTCGATGATGGGGTTGT	ATTGCGAGTGGGACATCTTC	60.0	228	93	oxidoreductase aldo/keto reductase family protein putative expressed
OsISM08999	LOC_Os01g62880	36417864	CTGCAGCTGGATTACGTTGA	GGCTTGACGAAGTTCTCTGG	60.0	185	90	oxidoreductase aldo/keto reductase family protein putative expressed
OsISM09000	LOC_Os01g62880	36418237	ACACCAGTGGCGTTCATCT	TTCCTAGCATCTCGGCTACC	59.3	188	97	oxidoreductase aldo/keto reductase family protein putative expressed
OsISM09001	LOC_Os01g62880	36418550	CTGTGTGCCAAGCTCTCTGA	TAGACACTCTCGGGGTGAGC	60.4	324	75	oxidoreductase aldo/keto reductase family protein putative expressed
OsISM09002	LOC_Os01g62900	36430178	GACGTGGAGAACACCGACTC	CAGTCACAACAGCGGTGC	60.4	754	83	amino acid kinase putative expressed
OsISM09003	LOC_Os01g62900	36430960	GCTCGGTGCTCTGTGTGA	GGTGACCAGAATCACCTCGT	59.8	166	65	amino acid kinase putative expressed
OsISM09004	LOC_Os01g62900	36431159	AAGTACCGGAAGCTCGTCAA	GTCCATGTCCATCTGTGGGT	60.3	143	61	amino acid kinase putative expressed
OsISM09005	LOC_Os01g62900	36431333	TGCTGATGGCGATCTATGAC	TCACGATCCGTAACAAGAAGC	60.0	163	74	amino acid kinase putative expressed
OsISM09006	LOC_Os01g62900	36431592	CAGTACCAGGAGGCAACCAT	TTCTTGCGCTAACAGTCTGG	59.6	206	80	amino acid kinase putative expressed
OsISM09007	LOC_Os01g62900	36431969	GCAGCTGCTTTTACTGCTTCA	TTCTCTCCTCGCATGACCTT	60.4	3202	93	amino acid kinase putative expressed
OsISM09008	LOC_Os01g62900	36435248	CTGCAAGAGATTGCTCCAGA	TTACGCTCCTCAGACGACAA	59.4	268	50	amino acid kinase putative expressed
OsISM09009	LOC_Os01g62900	36435619	AAATCATTGGTTGCTCGGAT	TAGGGTCCTCCATATCAGCG	59.7	208	89	amino acid kinase putative expressed
OsISM09010	LOC_Os01g62900	36435807	CGCTGATATGGAGGACCCTA	ACACCCAATGGGCAATATGT	60.0	222	91	amino acid kinase putative expressed
OsISM09011	LOC_Os01g62900	36436009	ACATATTGCCCATTGGGTGT	CATTTCCACTTCGGATTGCT	60.0	180	92	amino acid kinase putative expressed

ISM IDs	Rice gene locus IDs	Physical positions (bp) on chromosomes	Forward primers (5'-3')	Reverse primers (5'-3')	Melting tempearature (T˚C)	Expected product size (bp) in PCR	Expected product size (bp) in Q-PCR	Putative Functions
OsISM09012	LOC_Os01g62900	36436174	TCCGAAGTGGAAATGGTCTT	CAACATCTGGAATTGCACCA	60.0	188	97	amino acid kinase putative expressed
OsISM09013	LOC_Os01g62900	36436377	TTGGCCTAGTGAAAAACAAAGA	TGGGAAACGAGCTTATTGCT	59.4	171	98	amino acid kinase putative expressed
OsISM09014	LOC_Os01g62900	36436552	AAAGCAGCAACCAAGATTCC	CAATACGTTTTGCCATGTCC	59.1	239	95	amino acid kinase putative expressed
OsISM09015	LOC_Os01g62900	36436804	GGTTGATTATCCAGCAGCGT	CAAGGCCTTCTGTCCTGTTC	60.0	375	75	amino acid kinase putative expressed
OsISM09016	LOC_Os01g62900	36437161	ACAGGACAGAAGGCCTTGAC	AAGTGTCATGTGCAACAGGC	59.5	530	85	amino acid kinase putative expressed
OsISM09017	LOC_Os01g62900	36437766	TGCAGTCAGCAATTGACCAT	GTCTCTGCTGCCTTTCCATC	60.1	226	83	amino acid kinase putative expressed
OsISM09018	LOC_Os01g62900	36437972	GATGGAAAGGCAGCAGAGAC	CATCACAGAACCGTGTGCTT	59.9	175	80	amino acid kinase putative expressed
OsISM09019	LOC_Os01g62900	36438126	CAAGCACACGGTTCTGTGAT	GTATGCGCCCTGTGCTTATG	60.7	222	70	amino acid kinase putative expressed
OsISM09020	LOC_Os01g62900	36438364	TGGCGTTGATGGACTCCTA	TTGTCGCCGTTCACTACTTG	60.1	282	67	amino acid kinase putative expressed
OsISM09022	LOC_Os01g62970	36479601	CCTGTTTTTCGTATAAGCCCC	ACAACGAAAGAACACCCTCG	60.0	501	49	expressed protein
OsISM09023	LOC_Os01g62970	36480216	GCTGTTGCACACGCATAAAT	CGGCAAAACAATACTTTGGG	60.1	220	87	expressed protein
OsISM09024	LOC_Os01g62970	36480392	TGATGGTGATGGTATTTATCCAA	CACAACTACCTGCAGTCCCA	59.4	174	90	expressed protein
OsISM09025	LOC_Os01g63020	36499058	GGGTGCGAAGACAGACAAAG	CAAACCCTCTGCCTTGTGAT	60.5	203	51	hypothetical protein
OsISM09026	LOC_Os01g63020	36499326	CAATGCAGGCCAGATGTCTA	AGGCCACCTCTCCAATATCA	59.7	328	68	hypothetical protein
OsISM09027	LOC_Os01g63020	36499721	TATCAGGCTTCTGTACCGGG	GCCTGTACCTGTGACCTCGT	60.1	1016	72	hypothetical protein
OsISM09028	LOC_Os01g63020	36500700	CAGGCAATAGGAATCAGACGA	TCAAATCCTAAGTCCGCCAC	60.1	239	96	hypothetical protein
OsISM09029	LOC_Os01g63020	36500919	GTGGCGGACTTAGGATTTGA	TCTCTGGACTCCGGTGAGTAA	60.0	574	61	hypothetical protein
OsISM09030	LOC_Os01g63020	36501880	ATTGCACGGGAGAGAGAAGA	TGCTACCGGTTGATGTTTCA	60.0	288	80	hypothetical protein

ISM IDs	Rice gene locus IDs	Physical positions (bp) on chromosomes	Forward primers (5'-3')	Reverse primers (5'-3')	Melting tempearature (T˚C)	Expected product size (bp) in PCR	Expected product size (bp) in Q-PCR	Putative Functions
OsISM09031	LOC_Os01g63030	36511651	CCTTCTGATGGTACGGATGG	CTATGTCCATGGTAGGGTTGC	59.8	169	65	transposon protein putative unclassified
OsISM09032	LOC_Os01g63030	36511838	CTGTCCATGATTCACAACCG	ACGTCCTCCTCCATTGCTAC	59.6	139	59	transposon protein putative unclassified
OsISM09033	LOC_Os01g63030	36511988	TTGGATCACATGATTCAGGG	CTGTCCAATCCAGTGAAGGG	59.9	703	67	transposon protein putative unclassified
OsISM09034	LOC_Os01g63030	36512683	TTGGACAGAAGGAAACCCAT	CAGCAGGAGGTATGACAGCA	59.7	194	99	transposon protein putative unclassified
OsISM09035	LOC_Os01g63030	36513006	AAAAGCAGAGAACAATGCGG	AGGCTTCTTGGTTTTTGGGT	60.2	161	66	transposon protein putative unclassified
OsISM09036	LOC_Os01g63030	36513794	GAGTGCATGAATGGGAAGGT	CACGATGGATTGTATTGGGA	59.6	214	98	transposon protein putative unclassified
OsISM09037	LOC_Os01g63030	36514365	AGCTGCAAAAAGAGGACTCG	TGCGTGAATCTCTATCGGTG	59.8	164	84	transposon protein putative unclassified
OsISM09038	LOC_Os01g63030	36514734	ATAAGGGGATTCTGTGCAGC	GCATCCTTCGTCCATCTTTT	59.1	203	98	transposon protein putative unclassified
OsISM09039	LOC_Os01g63050	36527236	CGAGGGTGGAAAGGGAAC	GTTGGGGCTATCCTCCCC	61.0	193	98	expressed protein
OsISM09040	LOC_Os01g63050	36527668	CAACAACATGCTCACCCAAC	CTGCTTCAACAGCTGCCTCT	60.4	168	74	expressed protein
OsISM09041	LOC_Os01g63050	36528070	GCCGAGCCCAAGAAAAAG	ATCGTCGACGTTGAGGTTG	60.3	181	82	expressed protein
OsISM09042	LOC_Os01g63050	36528239	AACGTCGACGATCTTGGTG	ATTGCTCCGACTGACCAGAG	60.1	167	79	expressed protein
OsISM09043	LOC_Os01g63060	36539499	GTTTACATCGCTCGGGAGG	GCTATCTCCGCACACACCTT	60.4	132	54	phosphatidic acid phosphatase-related putative expressed
OsISM09044	LOC_Os01g63060	36539644	CTTCTTTTCGAGAAGTGGCG	CCCAGGTCGATGCAAGTAAT	60.0	1700	97	phosphatidic acid phosphatase-related putative expressed
OsISM09045	LOC_Os01g63060	36541356	GACTTGGGATTCATGGCTCT	CCACCTACCAACAGGAAGGA	59.5	194	99	phosphatidic acid phosphatase-related putative expressed
OsISM09046	LOC_Os01g63060	36541502	TGAGAGCGTGTTCACATTCA	TGCGCCAGAGTAGAACAGTG	59.6	729	99	phosphatidic acid phosphatase-related putative expressed
OsISM09047	LOC_Os01g63060	36542211	CACTGTTCTACTCTGGCGCA	TAATTTGGACCTGGGAGCTG	60.1	187	97	phosphatidic acid phosphatase-related putative expressed
OsISM09048	LOC_Os01g63060	36542378	CAGCTCCCAGGTCCAAATTA	CAAGTACATTATGGGGCGGT	59.9	177	74	phosphatidic acid phosphatase-related putative expressed

ISM IDs	Rice gene locus IDs	Physical positions (bp) on chromosomes	Forward primers (5'-3')	Reverse primers (5'-3')	Melting tempearature (T˚C)	Expected product size (bp) in PCR	Expected product size (bp) in Q-PCR	Putative Functions
OsISM09049	LOC_Os01g63060	36542535	ACCGCCCCATAATGTACTTG	AAAATCAGATCGCCACAACC	59.8	163	76	phosphatidic acid phosphatase-related putative expressed
OsISM09050	LOC_Os01g63060	36542718	CACTTGTTTTCGTGCGTACA	ATAGCCATCAGCCAAGCAAG	59.4	236	74	phosphatidic acid phosphatase-related putative expressed
OsISM09051	LOC_Os01g63060	36542982	CGAAAACACTACAGCGTGGA	GGAAGCTAAAAGAGGGTACCTG	59.2	176	99	phosphatidic acid phosphatase-related putative expressed
OsISM09052	LOC_Os01g63060	36543071	TGGTGCATTCTTTTTCGTCA	TGATCCATTTGTGCGATCTG	60.4	176	92	phosphatidic acid phosphatase-related putative expressed
OsISM09053	LOC_Os01g63060	36543315	GGATTGCAAGATGAAGGAAGA	CCGTTCATCTGCACACGTT	60.0	327	88	phosphatidic acid phosphatase-related putative expressed
OsISM09054	LOC_Os01g63100	36565146	CGACGAGGTCTCGGTCAA	GTTTGCCCACAGGAAGGAG	60.8	118	53	retrotransposon protein putative unclassified
OsISM09055	LOC_Os01g63100	36565989	CAACCCCGAGAAGATACGAG	CCTCCTCCGTCCAGGTAAAC	60.3	178	78	retrotransposon protein putative unclassified
OsISM09056	LOC_Os01g63100	36567702	GAGCTTCTCGCCGATGTC	GCCACGAAAGAGGTATGGTC	59.6	208	98	retrotransposon protein putative unclassified
OsISM09057	LOC_Os01g63170	36611762	CTTGTTGCTATCCTGGAGGG	AGGAAAACACCTCCATCGTG	59.8	214	79	transposon protein putative unclassified
OsISM09058	LOC_Os01g63170	36612012	ATTACTGACACGTTTGCCCC	ACGTGAACGTGCTCAACAAA	60.1	328	72	transposon protein putative unclassified
OsISM09059	LOC_Os01g63170	36612366	TAGAGGCGAGACCTCCAGTC	ATGGACCACTCCGATCCAC	60.2	186	98	transposon protein putative unclassified
OsISM09060	LOC_Os01g63170	36612627	AGTACACCAGGGGGTCAGC	GTCGACGCCGAACCATAC	60.0	344	88	transposon protein putative unclassified
OsISM09061	LOC_Os01g63170	36613042	AGGGGGAAGTAGGGATGGAT	GACAGGATGTCCGTCGAACT	60.5	603	83	transposon protein putative unclassified
OsISM09062	LOC_Os01g63180	36618186	CGGGAGATTCGTACCTCTGT	GTTGATGATCCGGAGCAAGT	59.6	168	90	laccase-6 precursor putative expressed
OsISM09063	LOC_Os01g63180	36619251	GCAGTCATCCGCTTCGTC	CCAATGGCGAAGTGAGAATC	60.6	721	75	laccase-6 precursor putative expressed
OsISM09064	LOC_Os01g63190	36621898	GTTCTTGTTCGCGCTTCTTC	TGTTGCATAGCCGACTGACT	59.8	429	99	laccase precursor protein putative expressed
OsISM09065	LOC_Os01g63190	36622394	AGTTGCTGTCGAGGTCATCA	ATGGCGTCAGTAGCTGGAGT	59.7	2225	78	laccase precursor protein putative expressed
OsISM09066	LOC_Os01g63190	36624813	CAAGAGGTCCCCATCGTG	GATTCTCTGGGAAGCTGGC	60.0	184	93	laccase precursor protein putative expressed

ISM IDs	Rice gene locus IDs	Physical positions (bp) on chromosomes	Forward primers (5'-3')	Reverse primers (5'-3')	Melting tempearature (T˚C)	Expected product size (bp) in PCR	Expected product size (bp) in Q-PCR	Putative Functions
OsISM09067	LOC_Os01g63190	36625010	GTTAATGGTGTTACCGGCGA	TTGATGACCCGGAGTAGCAC	60.9	212	93	laccase precursor protein putative expressed
OsISM09068	LOC_Os01g63190	36626057	GTCATCCGTTTCGTTGCC	AGGTGGCAATGGAAGAACC	60.0	172	51	laccase precursor protein putative expressed
OsISM09069	LOC_Os01g63200	36633313	CGCTCGTGCTGGTTCTCT	GTTCATGCATAGCTGGGTGA	60.0	290	96	laccase precursor protein putative expressed
OsISM09070	LOC_Os01g63200	36633688	ACGACTCACCGTACCCTCTG	GGCATTGCGTGATCTTGTT	59.9	296	91	laccase precursor protein putative expressed
OsISM09071	LOC_Os01g63200	36634127	TACCCGTTCCCTGAACCATA	CCACATCGTCCACATTTCTG	60.1	189	74	laccase precursor protein putative expressed
OsISM09072	LOC_Os01g63200	36634348	CAGATTTCAGATGCGCTCAC	GTCTTGCCGTACTCCACCTC	59.6	187	96	laccase precursor protein putative expressed
OsISM09073	LOC_Os01g63200	36635391	ATCCGGTTCATGGCGAAC	TTCAGCACCTCGAAAACCAT	61.3	223	96	laccase precursor protein putative expressed
OsISM09074	LOC_Os01g63220	36640798	CAGCAAGGCTGAGATCAATG	AGTGTGCTTTCCATTGAGGG	59.8	484	94	kinase pfkB family putative expressed
OsISM09075	LOC_Os01g63370	36724386	GGCGTCACCAAAAGAAGATT	AATTTTGCAGCGTTTGGTTC	59.6	339	95	hypothetical protein
OsISM09076	LOC_Os01g63370	36724698	AGGATTTGAACCAAACGCTG	GTCTCAAGATGGCTGTGGGT	60.1	293	79	hypothetical protein
OsISM09077	LOC_Os01g63380	36726716	CCAGTCCACCCTTCATCTTC	TTTGCACACAAGCATCATCA	59.7	280	86	transposon protein putative unclassified
OsISM09078	LOC_Os01g63380	36728203	CAATGCCCTGCTGAAAAGAT	GAAACCATGCTCTCCATGCT	60.2	313	62	transposon protein putative unclassified
OsISM09079	LOC_Os01g63380	36728855	GACCAAATCATGCAAGCAAA	CCAGCATTCCTGTCCACTTT	59.9	301	57	transposon protein putative unclassified
OsISM09080	LOC_Os01g63380	36729190	TTCACCGTATCGAATGAGAATG	TTTTCTGATGGGTCATTGGG	60.3	149	60	transposon protein putative unclassified
OsISM09082	LOC_Os01g63420	36747022	CTTCTCCACTGACGCCCTC	GCCATTCACCACCCTTATCA	60.8	1264	99	OsFBL4 - F-box domain and LRR containing protein expressed
OsISM09083	LOC_Os01g63420	36748663	CAGATTATTGCAAAATGTCCCA	CACCAACAACTTCTAGCCCTC	59.3	162	80	OsFBL4 - F-box domain and LRR containing protein expressed
OsISM09084	LOC_Os01g63520	36819242	AAGAATTTGGAAAGGACGCA	ACACGTCGATGTCGTTACCA	59.9	209	67	transposon protein putative CACTA En/Spm sub-class
OsISM09085	LOC_Os01g63530	36823152	GAACGAGAGCGAGAACGAAC	CAGTACTGGCTGCTTCCCTC	60.1	1261	91	transposon protein putative CACTA En/Spm sub-class

ISM IDs	Rice gene locus IDs	Physical positions (bp) on chromosomes	Forward primers (5'-3')	Reverse primers (5'-3')	Melting tempearature (T˚C)	Expected product size (bp) in PCR	Expected product size (bp) in Q-PCR	Putative Functions
OsISM09086	LOC_Os01g63530	36825309	CGTTCCGACCAGACAGAGAT	CTAGGTCTGCAATCTTCGCC	60.1	180	91	transposon protein putative CACTA En/Spm sub-class
OsISM09087	LOC_Os01g63530	36825590	ATTCCTCCTGCAATGGTGAG	GTACCTGAAGGGTCCGTTGG	60.9	295	99	transposon protein putative CACTA En/Spm sub-class
OsISM09088	LOC_Os01g63530	36826397	AATTCAAGCCTCGAAGTCCA	CACTTCCGCCTTTTCGATT	60.0	296	93	transposon protein putative CACTA En/Spm sub-class
OsISM09089	LOC_Os01g63530	36826905	CGCCCTCGACGTCTCTATTA	TCCAGTATCGAAAACCCTCC	59.7	191	80	transposon protein putative CACTA En/Spm sub-class
OsISM09090	LOC_Os01g63530	36827177	AAGGCGCAGCATTACAAGAC	GCCTCCAGGTCGATGAGTAA	60.3	179	81	transposon protein putative CACTA En/Spm sub-class
OsISM09091	LOC_Os01g63530	36827356	CTGCACCGTCAACGTATATGA	AATGACGGAACCGATACCAA	59.9	182	96	transposon protein putative CACTA En/Spm sub-class
OsISM09092	LOC_Os01g63530	36827545	CGGCAAATGGAGAGAAAGAC	GGCAATACTCGCACACGTAA	59.8	195	99	transposon protein putative CACTA En/Spm sub-class
OsISM09093	LOC_Os01g63530	36827740	ACTGCCTTGCAGACCAAATC	CCGCGATAAATTCCTTGTGT	60.1	169	88	transposon protein putative CACTA En/Spm sub-class
OsISM09094	LOC_Os01g63620	36895231	CAACTTTGCCCAGTTCCTGT	GCCCGCTTGAAGTAGTGCT	60.4	174	67	expressed protein
OsISM09096	LOC_Os01g63630	36901074	CACGACCTACTCCTTGAGGC	TAAACTCCCAGAAAAGGGGC	60.1	790	92	retrotransposon protein putative Ty3-gypsy subclass
OsISM09097	LOC_Os01g63630	36902112	TTATTCCTTGTCCCAGGAGG	ATGGCTGTGGCTAAACCTTG	59.6	1114	72	retrotransposon protein putative Ty3-gypsy subclass
OsISM09098	LOC_Os01g63630	36903523	TGTATTAATCCCCGTCCCAG	TCTGTTGCAAAGCACTACCG	59.8	1043	93	retrotransposon protein putative Ty3-gypsy subclass
OsISM09099	LOC_Os01g63630	36904649	AGGGGCTTTGAAAGAGGAAG	GCCTTGCCTATTTACCTTTGTT	59.3	273	90	retrotransposon protein putative Ty3-gypsy subclass
OsISM09100	LOC_Os01g63630	36904827	CCCACTAGTTGGGGTTTTGTT	CGGAAAGCTGTAGCGAACTC	60.1	1878	99	retrotransposon protein putative Ty3-gypsy subclass
OsISM09101	LOC_Os01g63630	36906966	CAGAAAAGAGGCAACCGAAG	AGGTAGTTGGAGGCCCTTGT	60.0	960	62	retrotransposon protein putative Ty3-gypsy subclass
OsISM09103	LOC_Os01g63640	36911185	CTGGCACACCTCACAGCTC	GGCGTCGAGCTTCTCCTC	60.9	175	77	retrotransposon protein putative unclassified
OsISM09104	LOC_Os01g63660	36918845	CGAATGGAAATGGGAAGAGA	CTCTTTCTGTCTGACCATCGG	59.9	326	80	retrotransposon protein putative Ty3-gypsy subclass
OsISM09105	LOC_Os01g63680	36936335	TAACCCTGGGTCGTTCAAAG	GTTGCCCACTTACGATCTCC	59.8	180	91	MYB family transcription factor putative expressed

ISM IDs	Rice gene locus IDs	Physical positions (bp) on chromosomes	Forward primers (5'-3')	Reverse primers (5'-3')	Melting tempearature (T˚C)	Expected product size (bp) in PCR	Expected product size (bp) in Q-PCR	Putative Functions
OsISM09106	LOC_Os01g63680	36936545	GAAGCAGTGTCGCGAAAGAT	TCTTGTGTGCTTCGATCAGC	60.3	720	99	MYB family transcription factor putative expressed
OsISM09107	LOC_Os01g63710	36950495	AGTTGGAGCATGTGGTTGTG	GAGAACACAAGTAAAATTGACAAGG	59.0	218	94	CDC6 - Putative DNA replication initiation protein expressed
OsISM09108	LOC_Os01g63710	36950727	CATTGAATCCTACTTGACCTCCA	AAGAAGCGATGCCATGTTTT	60.0	131	51	CDC6 - Putative DNA replication initiation protein expressed
OsISM09109	LOC_Os01g63710	36950982	CAGGTTTTTCTTGCAGCGAT	AAGATCGAGGTACCCCAACA	59.9	179	97	CDC6 - Putative DNA replication initiation protein expressed
OsISM09110	LOC_Os01g63710	36951144	TGGGGTACCTCGATCTTCAG	TTTCACCCATTCATTTTAGAGGA	59.9	175	98	CDC6 - Putative DNA replication initiation protein expressed
OsISM09111	LOC_Os01g63710	36951351	TTTCTCTCGCGTCAGAAGGT	CACGTCTGCCGTCGTAACTA	60.0	182	80	CDC6 - Putative DNA replication initiation protein expressed
OsISM09112	LOC_Os01g63710	36951575	CATACCCGCGAAGCATTAAG	GGAAATTGGGGAGGAATTGT	60.3	189	95	CDC6 - Putative DNA replication initiation protein expressed
OsISM09115	LOC_Os01g63710	36952016	CCCCTCGATCTCTAGTGCAG	CCCAATCAGAACTCTGAGCC	59.9	255	83	CDC6 - Putative DNA replication initiation protein expressed
OsISM09116	LOC_Os01g63710	36952250	GGGCTCAGAGTTCTGATTGG	CACACATACAGGCTCCCTGA	59.8	202	98	CDC6 - Putative DNA replication initiation protein expressed
OsISM09117	LOC_Os01g63710	36952429	CGTTCAGGGAGCCTGTATGT	CGACCAACCTACAGAGGAGC	60.0	541	61	CDC6 - Putative DNA replication initiation protein expressed
OsISM09118	LOC_Os01g63710	36952957	TGTAGGTTGGTCGTTGTGGA	TTGTCGATTGGCAAAACAGT	59.6	186	99	CDC6 - Putative DNA replication initiation protein expressed
OsISM09119	LOC_Os01g63710	36953133	CAATCGACAATTGGTGCTATTTT	CACAACAAGTGGTTTGCCTG	60.2	198	98	CDC6 - Putative DNA replication initiation protein expressed
OsISM09120	LOC_Os01g63710	36953333	TTTCCGTGCTTATTCCAAGG	TGTAGTATCACAAAAGGTAAGCAACC	60.0	148	77	CDC6 - Putative DNA replication initiation protein expressed
OsISM09121	LOC_Os01g63710	36953512	ACTGGCCCTTGAATTTTGTG	TGAACACGTCAATTTCATGCT	59.6	159	65	CDC6 - Putative DNA replication initiation protein expressed
OsISM09122	LOC_Os01g63730	36963324	TCTGAACTGTCCTGGCTGG	ACGAGAACGAAATTCCAAGC	59.6	222	99	hypothetical protein
OsISM09123	LOC_Os01g63750	36980492	TCGAGAGAGAGAGAGGCGAG	CTCACCTGTGACGGCTCC	60.3	191	77	glycine-rich protein putative
OsISM09124	LOC_Os01g63770	36998152	GGTTCAGCTGCGCCTCTA	GAGCTGCGAGAAGGAGTACG	60.3	200	66	transmembrane amino acid transporter protein putative expressed
OsISM09125	LOC_Os01g63770	36998468	CAGCTTCAAGAACCACGTCA	GAGGAACGTGCAGTTGAAGG	60.4	520	98	transmembrane amino acid transporter protein putative expressed

ISM IDs	Rice gene locus IDs	Physical positions (bp) on chromosomes	Forward primers (5'-3')	Reverse primers (5'-3')	Melting tempearature (T°C)	Expected product size (bp) in PCR	Expected product size (bp) in Q-PCR	Putative Functions
OsISM09126	LOC_Os01g63770	36998972	CAACTGCACGTTCCTCCTCT	CAGCCGGTCGTTGATGTAGT	60.8	258	80	transmembrane amino acid transporter protein putative expressed
OsISM09127	LOC_Os01g63770	36999330	GCATGACCACCTATACCGCT	CACCAGCTTGGTTGGACC	60.0	206	93	transmembrane amino acid transporter protein putative expressed
OsISM09128	LOC_Os01g63770	36999558	ACATCCTCTACACCTTCGGC	TACTTGAACTTGGCCGGTTT	59.4	1745	77	transmembrane amino acid transporter protein putative expressed
OsISM09129	LOC_Os01g63770	37001667	GTCTACATCATCCCGGCACT	AGGAAGAACGGTGGCTTCTC	60.4	1205	87	transmembrane amino acid transporter protein putative expressed
OsISM09130	LOC_Os01g63810	37020894	AAGACACTCCCACCTCCTGA	CTTTTGCAGCACGAATTTGA	59.8	145	64	starch binding domain containing protein putative expressed
OsISM09131	LOC_Os01g63810	37021118	CTCTGGATTGGTCCGAAGAC	ATCGATCTTTCTGCTGGCAA	60.3	142	62	starch binding domain containing protein putative expressed
OsISM09132	LOC_Os01g63810	37021556	AAATGATGGGAGCAAACGAC	CCCCACTAAGCTCATCTGGA	60.1	438	79	starch binding domain containing protein putative expressed
OsISM09133	LOC_Os01g63870	37059762	AGGTGCATTCCAAGTGCTGT	GACGGTTGGTGAGATGACAA	60.1	898	86	nucleobase-ascorbate transporter putative expressed
OsISM09134	LOC_Os01g63870	37060706	GGTCATGTATAGAGATGGGCCT	TAAAAACACCCGGTAGCCAA	59.8	189	96	nucleobase-ascorbate transporter putative expressed
OsISM09135	LOC_Os01g63870	37060875	TTGGCTACCGGGTGTTTTTA	AAAAGCGATAGCCCATGTGA	60.5	173	66	nucleobase-ascorbate transporter putative expressed
OsISM09136	LOC_Os01g63870	37061256	TTGTGATGTGTGTTGCATCTG	CAGCTGTTGGTGGTCTTGTG	59.8	393	94	nucleobase-ascorbate transporter putative expressed
OsISM09137	LOC_Os01g63870	37061756	GCGGTTACTAAAATGGGCAA	AAGCAATGAAGGCACCAACT	59.9	203	95	nucleobase-ascorbate transporter putative expressed
OsISM09138	LOC_Os01g63870	37062181	TTGTTGCATCTCATGGACCT	AACGCAATGACCATGTTGAG	59.3	867	89	nucleobase-ascorbate transporter putative expressed
OsISM09139	LOC_Os01g63900	37077273	CGGCACATTATGGTGCTGT	TCCCAAAGTCTTAAGCGAGAA	59.8	225	92	WD domain G-beta repeat domain containing protein expressed
OsISM09140	LOC_Os01g63900	37077585	TCCATCACTTGTATTCATCCCA	AACAGGTTCATAGTGCCCCC	60.7	176	98	WD domain G-beta repeat domain containing protein expressed
OsISM09141	LOC_Os01g63900	37077724	GACATTTCAAACATTCCGGG	AATTTGCATGTCATTGCTGC	59.9	182	98	WD domain G-beta repeat domain containing protein expressed
OsISM09142	LOC_Os01g63900	37077910	TGTGGTCTCCATCAACTCCA	GGGTGAAACTGCAGGATGAA	60.6	497	84	WD domain G-beta repeat domain containing protein expressed
OsISM09143	LOC_Os01g63950	37122346	GTAGGTTCCATGCCTTCCAA	TGCAGGTAGCTAATGTCCCC	60.0	177	75	expressed protein

ISM IDs	Rice gene locus IDs	Physical positions (bp) on chromosomes	Forward primers (5'-3')	Reverse primers (5'-3')	Melting tempearature (T°C)	Expected product size (bp) in PCR	Expected product size (bp) in Q-PCR	Putative Functions
OsISM09144	LOC_Os01g63950	37122570	AAGAGGATTTCTGTGGCACG	TGCATCAGTACGAAGCCACT	59.9	301	54	expressed protein
OsISM09145	LOC_Os01g63950	37123289	ATGGTGGCACATCATCTGAA	CGAACTCTTCTTGCTCCCAG	60.0	645	82	expressed protein
OsISM09146	LOC_Os01g63950	37123971	CCAAGCTGGAAAGAAGCAAA	CAGCTTCCTCCAAGCAGTCT	60.1	775	93	expressed protein
OsISM09147	LOC_Os01g63990	37153575	AGTGTTTCACCGGATGTTCA	CTAACTTGGAGAGCGCAACC	59.5	1183	87	hydrolase alpha/beta fold family protein putative expressed
OsISM09148	LOC_Os01g63990	37154938	TCATTTTCTCCCATGGCTTC	CAGGTAAATCCCCAGCTCCT	60.2	182	84	hydrolase alpha/beta fold family protein putative expressed
OsISM09149	LOC_Os01g63990	37155277	GCTGCCTCAACTACATCCCT	ATCGGCCACCAGTAGTTCAC	59.7	159	74	hydrolase alpha/beta fold family protein putative expressed
OsISM09150	LOC_Os01g63990	37155600	AGGACTGGGAGATCCTAGCTG	AGTGGTAGGCTCCCTGCTTC	60.3	228	79	hydrolase alpha/beta fold family protein putative expressed
OsISM09151	LOC_Os01g64000	37174469	GCATGATCAAGAACCGTGAG	AGCTTCGAGCTCCACCGTAT	60.3	162	72	bZIP transcription factor putative expressed
OsISM09152	LOC_Os01g64000	37174651	GGAGAACGCTCGTCTCAAAG	CATGGGAGAGGACAATACCA	59.2	199	74	bZIP transcription factor putative expressed
OsISM09153	LOC_Os01g64000	37174806	CGGTTCTACTGACAAAGAAGCA	CCTTGGACTGCTCCATCATT	59.8	199	55	bZIP transcription factor putative expressed
OsISM09154	LOC_Os01g64010	37177563	GAGATGGGCACTGAGAAAGC	AAGAAGTGCACACATCACGC	59.9	649	95	dual specificity protein phosphatase putative expressed
OsISM09156	LOC_Os01g64050	37200207	ATGCCGTCTTCCACTAGCC	CACAGCTGGATCCACAGAAG	59.8	760	83	retrotransposon protein putative Ty3-gypsy subclass
OsISM09157	LOC_Os01g64050	37201770	TTTCATCAATCGACCACAATG	CTGATGTGGAGGGGAGGAT	59.6	160	82	retrotransposon protein putative Ty3-gypsy subclass
OsISM09158	LOC_Os01g64050	37202218	GATGATGTTGAACAGGCTGC	TGGTGGAGCTGATTTCTTCTT	59.1	222	64	retrotransposon protein putative Ty3-gypsy subclass
OsISM09159	LOC_Os01g64050	37202369	CAGCCGACTACTCATAGGTTTTG	ACAGGAGAAGGAGGAGGAGG	60.0	159	92	retrotransposon protein putative Ty3-gypsy subclass
OsISM09160	LOC_Os01g64050	37202560	CCGTGGGGAGTCATCATATT	GGGAGCTAGAGCTTTGCTTG	59.5	208	90	retrotransposon protein putative Ty3-gypsy subclass
OsISM09161	LOC_Os01g64050	37202963	GCCAAGCTAAGATTAGCCGA	GCTTCAAGCCGATCGATATT	59.4	146	99	retrotransposon protein putative Ty3-gypsy subclass
OsISM09162	LOC_Os01g64090	37230639	TTTTGTCTCATCCTGACCCC	GCCTCCCTAAGAGCATCACTC	60.1	722	99	L1P family of ribosomal proteins domain containing protein expressed

ISM IDs	Rice gene locus IDs	Physical positions (bp) on chromosomes	Forward primers (5'-3')	Reverse primers (5'-3')	Melting tempearature (T˚C)	Expected product size (bp) in PCR	Expected product size (bp) in Q-PCR	Putative Functions
OsISM09163	LOC_Os01g64090	37231501	GCCCTAAGATGAAGGTGTGC	CTTGAGAGCTTCCACATCCA	59.3	589	93	L1P family of ribosomal proteins domain containing protein expressed
OsISM09164	LOC_Os01g64090	37232176	TCCTCGACTTCTTGGACCTG	TTGGACTCAAGTGATTCCTGG	60.2	570	79	L1P family of ribosomal proteins domain containing protein expressed
OsISM09165	LOC_Os01g64090	37232850	ATGTGCAAATGAGCGTGAAC	CCGGATCACTTTTCCCATAG	59.6	474	99	L1P family of ribosomal proteins domain containing protein expressed
OsISM09166	LOC_Os01g64100	37235275	CCAAGTTCTGGGACGACCT	CTCAGCACCACCCTCCTC	59.7	153	71	glycosyl hydrolase putative expressed
OsISM09167	LOC_Os01g64170	37269158	CTCCTGCTCCAGCTCCTTT	GCGATCTGGCCATAGTTGAT	59.9	806	75	glycosyl hydrolases family 17 putative expressed
OsISM09168	LOC_Os01g64200	37280174	CGAAGGATTTAGAATCTGCAAAC	TCTTGTCCTATTGTAATTTTGCTCA	59.3	890	93	transposon protein putative unclassified
OsISM09169	LOC_Os01g64200	37281078	TGCAAAGAAAAGTAGTACACGATCA	TTCTCAATGTTTTTGTCTCTCTCTG	59.7	175	82	transposon protein putative unclassified
OsISM09170	LOC_Os01g64200	37281200	TGTTGTGCAGGTATTTTGAAGG	AATTTTGCTCCAGTAACCCAA	59.3	212	98	transposon protein putative unclassified
OsISM09171	LOC_Os01g64200	37281434	GCTTGAAGATGGCAAAGGTG	CAGCTATTCTCTTTCCCTTGTCA	60.3	156	78	transposon protein putative unclassified
OsISM09172	LOC_Os01g64200	37281567	TGACAAGGGAAAGAGAATAGCTG	CATTGCATCTTGTAGGTTGTGAA	60.0	372	99	transposon protein putative unclassified
OsISM09173	LOC_Os01g64200	37281916	TTCACAACCTACAAGATGCAATG	CAACATTTGTGCTGCTGCTT	60.1	150	63	transposon protein putative unclassified
OsISM09174	LOC_Os01g64200	37282048	GCAGCAGCACAAATGTTGAC	CAGAATCAACTTGAGCTTCAAAA	59.6	243	82	transposon protein putative unclassified
OsISM09175	LOC_Os01g64200	37282282	TTGATTCTGTACAACCTTGGTCTC	TCCTTTTGATGCTTCCTGTTG	59.9	290	90	transposon protein putative unclassified
OsISM09176	LOC_Os01g64200	37282661	TGCATACATGGCTGGATTTT	TTGGGATCTTCTTCCTGCTT	58.9	530	70	transposon protein putative unclassified
OsISM09177	LOC_Os01g64230	37299103	CTGACCAGCTTGATGCTGAA	TCACTCGGCTCACCATTGTA	60.2	288	71	long cell-linked locus protein putative expressed
OsISM09178	LOC_Os01g64240	37300691	GTATCGAGCTTCGTCGGAGA	AAAAGCCGTGTCCGTGTG	60.6	184	94	hypothetical protein
OsISM09179	LOC_Os01g64250	37303254	TCTTCCCTGACATCCAGAGG	ATCTTCCTTGATGCCGTTCA	60.4	1016	99	hemerythrin family protein expressed
OsISM09180	LOC_Os01g64250	37304304	CCCTCTTCCAAGAAGTGCTG	GCAGCTCCCTCTCTTCTTCC	60.3	2033	94	hemerythrin family protein expressed

ISM IDs	Rice gene locus IDs	Physical positions (bp) on chromosomes	Forward primers (5'-3')	Reverse primers (5'-3')	Melting tempearature (T˚C)	Expected product size (bp) in PCR	Expected product size (bp) in Q-PCR	Putative Functions
OsISM09181	LOC_Os01g64270	37314585	ATAGCTCGGTTCGAGACGCT	TCTTCAGTTCGCTTACCCTCA	60.7	194	98	expressed protein
OsISM09182	LOC_Os01g64270	37314702	ACTACAGTGGGGGAACAGCC	CATGAATCCTAGCCGGACAC	60.9	191	98	expressed protein
OsISM09183	LOC_Os01g64290	37321227	ATCCACCACGCTCCCTTCT	GTGCTTGCTCCTCGTATCGT	61.2	174	73	expressed protein
OsISM09184	LOC_Os01g64310	37333597	CCGTCTTCGACGTCTACTCC	GAAGTAGAACCACGGCTCCC	60.4	185	99	no apical meristem protein putative expressed
OsISM09185	LOC_Os01g64310	37334036	GCCACCAAACCATCTTCCT	GGTGTACAGTCGACACAGGC	59.6	541	59	no apical meristem protein putative expressed
OsISM09186	LOC_Os01g64360	37357663	GTACCCTGGTCCGAAGACG	GCGAGATGTTCCTCCAGTCT	60.0	332	80	MYB family transcription factor putative expressed
OsISM09187	LOC_Os01g64390	37375315	AATGCAATCATGGCGGAG	AGCGTAATTCATCCAAACCG	60.3	428	77	hypothetical protein
OsISM09188	LOC_Os01g64390	37375748	TGTCATAAACCGACGAGACG	TGTGCACCGATTAGCAAAAG	59.8	179	76	hypothetical protein
OsISM09190	LOC_Os01g64400	37378878	CGTCTGAGTGTGCCAAATGA	ACTTGAGTATCCATGACTTGATG A	59.3	121	71	hypothetical protein
OsISM09191	LOC_Os01g64420	37384633	GTCCATCTGGACAGGCAAGT	TCCTGTTAATCCCACGGTTC	60.0	457	70	transposon protein putative unclassified
OsISM09193	LOC_Os01g64420	37385606	CTAGCAAGGCTTGGTTGCTC	AACCACAACAGGTACGCCTC	60.1	184	98	transposon protein putative unclassified
OsISM09194	LOC_Os01g64420	37386101	CGATGGGGACTTACTGGTGT	CTCACCTGGATGTGAGCGTA	59.8	145	53	transposon protein putative unclassified
OsISM09195	LOC_Os01g64420	37386459	CGGACTTAGGCAGGTGTTTC	CTAGCTGTCCCCGTCGAGT	60.1	159	81	transposon protein putative unclassified
OsISM09196	LOC_Os01g64420	37386826	GCACCGTGACCGAGACTACT	ATATCGGCACTGATATCCCG	60.0	152	58	transposon protein putative unclassified
OsISM09198	LOC_Os01g64420	37387534	AGCTCTTCCTCTCACGGAGC	AGACGAAGAGGCCCCAGTA	60.5	144	82	transposon protein putative unclassified
OsISM09199	LOC_Os01g64420	37387853	ACCGACTCACGTACTCCCAC	GCAAGGGCGATTTTGTACTG	60.3	892	76	transposon protein putative unclassified
OsISM09200	LOC_Os01g64460	37409649	AAGGGCTACATATATGCACCT GA	GGAAGGCTTCTTTTTGAGCA	59.7	435	71	hypothetical protein
OsISM09201	LOC_Os01g64510	37429808	TCATGGCAAGTTACGGTTGA	CTTCAGCCTGGTCGAGAGTC	60.1	189	98	retrotransposon protein putative unclassified

ISM IDs	Rice gene locus IDs	Physical positions (bp) on chromosomes	Forward primers (5'-3')	Reverse primers (5'-3')	Melting tempearature (T˚C)	Expected product size (bp) in PCR	Expected product size (bp) in Q-PCR	Putative Functions
OsISM09202	LOC_Os01g64510	37430337	CACCCCTGGCTACACTCCTA	CGATGAGAACACAACTAGGGC	59.9	121	69	retrotransposon protein putative unclassified
OsISM09203	LOC_Os01g64510	37430748	GAAGCATCTGCCTCGGAGTA	CGAAGAAGGGAGAAAAAGCA	60.0	216	84	retrotransposon protein putative unclassified
OsISM09204	LOC_Os01g64510	37430898	ATTCGATGAGGGGTGTTCTG	GCTCTGTCGAACAGTGAGCC	60.5	164	69	retrotransposon protein putative unclassified
OsISM09205	LOC_Os01g64550	37454555	CTGAGGGGATAGGCACCG	GAGCACGGCTGATTGTTT	59.4	807	99	hypothetical protein
OsISM09206	LOC_Os01g64560	37456758	CTGGTCCTACGCCGTCTTCT	CGCTCATAGTGCCCATCC	61.0	395	85	helix-loop-helix DNA-binding protein putative expressed
OsISM09207	LOC_Os01g64560	37457292	AGCCATGACAGGGTGGAC	CAGTGCAGCCTGACCAATC	59.9	193	88	helix-loop-helix DNA-binding protein putative expressed
OsISM09208	LOC_Os01g64560	37457474	AGGCTGCACTGACAGGGTT	CAAAACTGGCCCTTCATCTC	60.3	412	98	helix-loop-helix DNA-binding protein putative expressed
OsISM09209	LOC_Os01g64560	37457866	GAGATGAAGGGCCAGTTTTG	ACCATTTTTGTGGAGCCAAG	59.8	179	93	helix-loop-helix DNA-binding protein putative expressed
OsISM09210	LOC_Os01g64560	37458009	ACGAGGCGTTATTCAGCTTG	ACCCAGCTGCTGAAACAAAG	60.4	766	89	helix-loop-helix DNA-binding protein putative expressed
OsISM09211	LOC_Os01g64560	37460303	GGCAAATGATTCAAGACCGT	CATATGCTTAATGGTCTTCTCCA	59.1	200	99	helix-loop-helix DNA-binding protein putative expressed
OsISM09212	LOC_Os01g64560	37460537	AGTTGAAGGGATCTGGGGAG	ACTTCAAAGGCCCAGGTTG	60.3	1078	95	helix-loop-helix DNA-binding protein putative expressed
OsISM09213	LOC_Os01g64560	37461648	AGAGGATCTCAATCCACCCC	TCAGCTATTTCCAAGAAGATTCC	59.6	168	76	helix-loop-helix DNA-binding protein putative expressed
OsISM09214	LOC_Os01g64560	37461873	TAAGATTTGGGCTCGCTTTG	CTCCCAGTGCTAGGTTCCAG	60.1	272	97	helix-loop-helix DNA-binding protein putative expressed
OsISM09215	LOC_Os01g64580	37469134	CATCGGAAAGAATGCATCAA	TTGTGGTTTCGCGATGATAA	59.8	397	79	hypothetical protein
OsISM09216	LOC_Os01g64580	37469557	GGCCATTTGTTTACGTCGTT	AATGAACTGTACGTCCTTGGG	59.4	665	64	hypothetical protein
OsISM09217	LOC_Os01g64580	37470232	CCACACAACTCACTTTACAGCA	GCTCGTCTGGATCCACTTGT	59.6	311	96	hypothetical protein
OsISM09218	LOC_Os01g64600	37491423	CTACTCATGAGGCGACGTGT	CTCCCCCATCAATGAGGAC	59.6	162	64	retrotransposon protein putative unclassified
OsISM09219	LOC_Os01g64600	37492018	GATGATCACAGGCGACGAG	CTCCATGCGAAGACGTCAG	60.5	150	76	retrotransposon protein putative unclassified

ISM IDs	Rice gene locus IDs	Physical positions (bp) on chromosomes	Forward primers (5'-3')	Reverse primers (5'-3')	Melting tempearature (T˚C)	Expected product size (bp) in PCR	Expected product size (bp) in Q-PCR	Putative Functions
OsISM09220	LOC_Os01g64600	37495363	TTCATGGATCGAGGAGTTGC	GCGCCGTAGACAAGAAGAAA	60.8	95	64	retrotransposon protein putative unclassified
OsISM09221	LOC_Os01g64630	37509904	GTGACAATGGCACTGGAATG	TGCATCATCACCAGCAAAAC	60.3	175	48	actin putative expressed
OsISM09222	LOC_Os01g64630	37510417	TCTTTCGTTGTACGCCAGTG	CGGGAGTGTAAATCCTTCGT	59.5	214	98	actin putative expressed
OsISM09223	LOC_Os01g64630	37511108	CCGCCAGAGAGGAAATACAG	ATTCATCGTATTCGGCCTTG	59.9	642	98	actin putative expressed
OsISM09224	LOC_Os01g64650	37514043	GGAGTTCATCGTCTTCGTCG	TGTTGTGTGAGTGGACCTTG	59.4	691	99	VAMP-like protein YKT62 putative expressed
OsISM09225	LOC_Os01g64650	37514741	TCTTTGCGCAGTTGCATTTA	CCCGAAAGCCTTCTGGTATT	60.5	778	95	VAMP-like protein YKT62 putative expressed
OsISM09226	LOC_Os01g64650	37515568	GGCCTTTCTTGACAGATGCT	TTTCATCCAAGTCCCTCTGG	59.7	543	85	VAMP-like protein YKT62 putative expressed
OsISM09227	LOC_Os01g64650	37516091	CCAGAGGGACTTGGATGAAA	ATCTTTCCCCTCTTTGGAGC	59.8	306	72	VAMP-like protein YKT62 putative expressed
OsISM09228	LOC_Os01g64650	37516420	TCAGACCTGAGTGCTGCTTC	GGTTTGTTTTCTTTGCCTGC	59.5	531	53	VAMP-like protein YKT62 putative expressed
OsISM09229	LOC_Os01g64660	37518774	GCTTGGCTGCAAGTTCGT	ACTTGCCTGAACGTTGGTCT	60.0	195	89	fructose-1 6-bisphosphatase putative expressed
OsISM09230	LOC_Os01g64660	37518891	AAACTTCTCGGGTTGTGCAT	TCCAGCTTCTTCTGCTCCTC	59.7	187	93	fructose-1 6-bisphosphatase putative expressed
OsISM09231	LOC_Os01g64660	37519058	GAGGAGCAGAAGAAGCTGGA	TCCTCCTCGGAGACAAGAAC	59.6	183	93	fructose-1 6-bisphosphatase putative expressed
OsISM09232	LOC_Os01g64660	37519212	GTGCAGTGCGTTCTTGTCTC	GTCGAGTGGATCAAAGCACA	59.7	204	94	fructose-1 6-bisphosphatase putative expressed
OsISM09233	LOC_Os01g64660	37519426	ACATCGACTGTGGCGTCTC	CAGGCTGTAACACGTCCTCA	59.9	497	91	fructose-1 6-bisphosphatase putative expressed
OsISM09234	LOC_Os01g64660	37519927	AAACATGGTTGCTGCTGGAT	TGTGAAGCCATTGACACCAT	60.2	167	83	fructose-1 6-bisphosphatase putative expressed
OsISM09235	LOC_Os01g64660	37520098	ATCCATCTCTCGGGGAGTTC	TTTTCGCATTCCCTTCATTC	60.2	220	91	fructose-1 6-bisphosphatase putative expressed
OsISM09236	LOC_Os01g64660	37520298	GAATGAAGGGAATGCGAAAA	AGGGAACTTGCACTTCTCCA	59.9	143	65	fructose-1 6-bisphosphatase putative expressed
OsISM09237	LOC_Os01g64660	37520429	TGCAAGTTCCCTAAGGATGG	CAACGTACGGTGGACATCAG	60.0	238	79	fructose-1 6-bisphosphatase putative expressed

ISM IDs	Rice gene locus IDs	Physical positions (bp) on chromosomes	Forward primers (5'-3')	Reverse primers (5'-3')	Melting tempearature (T˚C)	Expected product size (bp) in PCR	Expected product size (bp) in Q-PCR	Putative Functions
OsISM09238	LOC_Os01g64700	37536527	CCCAGAGTTTTGTGGGAAAC	TCATACCATCTCGAGGACCA	59.2	205	66	OsFBT3 - F-box and tubby domain containing protein expressed
OsISM09239	LOC_Os01g64700	37536765	CCACCTACCATCTCTATCTGTGC	TTACGGTGTCTTTTAGCGGC	60.1	337	83	OsFBT3 - F-box and tubby domain containing protein expressed
OsISM09240	LOC_Os01g64700	37537141	CAACATCTCAAGGTCGAGCA	GTAAGGGGGCTGTGTGTCAT	59.9	176	92	OsFBT3 - F-box and tubby domain containing protein expressed
OsISM09242	LOC_Os01g64720	37551233	CTCCTGCTCCGCCTACCT	CTTGCCCTTGGACAACATCT	60.3	380	83	expressed protein
OsISM09243	LOC_Os01g64720	37551576	GATAGCCATGGAGGGTGAGA	GATCCACCACTGACGAGCTT	60.2	177	90	expressed protein
OsISM09244	LOC_Os01g64720	37551758	CCACATCGTTCAGAGATCCA	TTCCAAGAATCAAACAGCCA	59.4	1019	68	expressed protein
OsISM09245	LOC_Os01g64720	37552806	TCTCGGTTAGATACGGTGGC	GACACCACAATCACGAGGTG	60.1	188	70	expressed protein
OsISM09246	LOC_Os01g64720	37552999	AGGAAGTTGGGCTGGGTTAT	CACAATTCACGAGCAACAGC	60.1	232	67	expressed protein
OsISM09247	LOC_Os01g64720	37553253	CAGGACATTCATGTTCGCAG	ATGCACTGGGCAAAGCTAAA	60.5	394	66	expressed protein
OsISM09248	LOC_Os01g64720	37553674	GCTAGCAATCGCTGAAGGTT	CCTCCAATAGGGCCAGAAAT	59.9	174	94	expressed protein
OsISM09249	LOC_Os01g64720	37553873	CCTTCTGGACCAATTGCTGT	CAACCCATCTTGACAAGGCT	60.1	275	86	expressed protein
OsISM09250	LOC_Os01g64720	37554127	CAGCCTTGTCAAGATGGGTT	TCAACTGATGAAAATGAAAGGC	59.7	221	65	expressed protein
OsISM09251	LOC_Os01g64720	37554323	CAGGCCTTTCATTTTCATCA	TTTCCCTTCCAAAGTCCACA	59.6	514	86	expressed protein
OsISM09252	LOC_Os01g64720	37554841	CGAGCTTTCTCATTTCGAGG	ACTTCCAGATTCGCACCATC	60.1	263	91	expressed protein
OsISM09253	LOC_Os01g64750	37565464	CTGATGGGTTCCCTTCCTCT	TTGCCCTTTAGAACAATCCG	60.3	884	81	sterol 3-beta-glucosyltransferase putative expressed
OsISM09254	LOC_Os01g64750	37566387	AAGTGTGACCTCACGTTGGA	TCCAGTGTACCATCATTCTGGA	59.8	613	90	sterol 3-beta-glucosyltransferase putative expressed
OsISM09255	LOC_Os01g64750	37567151	GGGGATGTCCAGCCATTTAT	GTACGGAAATTGGCGTGAGT	60.5	1602	87	sterol 3-beta-glucosyltransferase putative expressed
OsISM09256	LOC_Os01g64750	37568794	GTGATCCTCGCATATTGGCT	GAAATTTCTGTGGGTCCAGC	59.8	446	71	sterol 3-beta-glucosyltransferase putative expressed

ISM IDs	Rice gene locus IDs	Physical positions (bp) on chromosomes	Forward primers (5'-3')	Reverse primers (5'-3')	Melting tempearature (T˚C)	Expected product size (bp) in PCR	Expected product size (bp) in Q-PCR	Putative Functions
OsISM09257	LOC_Os01g64750	37569289	TGCACAGAACCTGATTTGGA	CCAAGTGCCTCTGCAATATG	59.8	1058	99	sterol 3-beta-glucosyltransferase putative expressed
OsISM09258	LOC_Os01g64750	37570328	ATATTGCAGAGGCACTTGGG	GCCAATGGATGAGGGAACT	60.0	711	83	sterol 3-beta-glucosyltransferase putative expressed
OsISM09259	LOC_Os01g64750	37571020	AGTTCCCTCATCCATTGGC	GGTGCCCCACCAAATTACTA	59.8	231	87	sterol 3-beta-glucosyltransferase putative expressed
OsISM09260	LOC_Os01g64750	37571342	CTGGATACATGTGGAGCCCT	ACAACATCCACGAGAGGACC	60.0	644	65	sterol 3-beta-glucosyltransferase putative expressed
OsISM09261	LOC_Os01g64750	37572037	TTCTCAGTGGCTTCAACAGG	TGCGTCCAGAATGACAGAAG	59.5	549	98	sterol 3-beta-glucosyltransferase putative expressed
OsISM09262	LOC_Os01g64750	37572608	GAATCATTAGTCGGGGTTGG	AGGAAACAGCCAGTCGTGAG	59.8	474	96	sterol 3-beta-glucosyltransferase putative expressed
OsISM09263	LOC_Os01g64750	37573062	CTCACGACTGGCTGTTTCCT	TGCTTACCCCAGCTACAAGC	60.4	504	88	sterol 3-beta-glucosyltransferase putative expressed
OsISM09264	LOC_Os01g64750	37573508	TAGGTACATCATGGTGGGGC	CCCCAGAAGAATTGATCTCC	59.6	399	96	sterol 3-beta-glucosyltransferase putative expressed
OsISM09265	LOC_Os01g64750	37573967	CGAGGCACTTTCAAATGCTA	TGCTATTGCCAGTTCCATTG	59.4	574	74	sterol 3-beta-glucosyltransferase putative expressed
OsISM09266	LOC_Os01g64760	37576408	GGCCCTTCTTCTTCATCGTT	TAGGACAGCGAATGCAACAG	60.3	1141	93	Yip1 domain containing protein expressed
OsISM09267	LOC_Os01g64760	37577545	CCTAGCAGCTGGGGCTATAA	CAAGAAGACTGAGGCTCTGGA	59.6	199	84	Yip1 domain containing protein expressed
OsISM09268	LOC_Os01g64770	37579854	GAACGTCGGGCATGAGAT	GTTCTTAGCACGCAGCATGA	59.9	188	92	polypyrimidine tract-binding protein putative expressed
OsISM09269	LOC_Os01g64770	37580021	GTCATGCTGCGTGCTAAGAA	TTGGATCACATTCACAGCAGA	60.2	162	70	polypyrimidine tract-binding protein putative expressed
OsISM09270	LOC_Os01g64770	37580162	TCTGCTGTGAATGTGATCCAA	TCAATTCTTGGTGGGATGAA	59.6	477	92	polypyrimidine tract-binding protein putative expressed
OsISM09271	LOC_Os01g64770	37580653	TCTCATGGACGAAATCCTGA	CTTGATGAAGCACTTCAATGG	58.8	742	98	polypyrimidine tract-binding protein putative expressed
OsISM09272	LOC_Os01g64770	37581399	TTCTCCCTATGGATTTGTGGA	TTGCACTTTGACGTGACTGA	59.2	346	90	polypyrimidine tract-binding protein putative expressed
OsISM09273	LOC_Os01g64770	37581725	TCAGTCACGTCAAAGTGCAA	ATGTCTAGCTGGCAGCAACC	59.7	189	79	polypyrimidine tract-binding protein putative expressed
OsISM09274	LOC_Os01g64770	37581894	GGTTGCTGCCAGCTAGACAT	GTGAACTTGCAATTCGCTG	59.2	139	52	polypyrimidine tract-binding protein putative expressed

ISM IDs	Rice gene locus IDs	Physical positions (bp) on chromosomes	Forward primers (5'-3')	Reverse primers (5'-3')	Melting tempearature (T°C)	Expected product size (bp) in PCR	Expected product size (bp) in Q-PCR	Putative Functions
OsISM09275	LOC_Os01g64770	37581999	TTGAATCTGCAGTCTCAGCG	TGAGCGTTGTTCTGTAGGCA	60.4	191	92	polypyrimidine tract-binding protein putative expressed
OsISM09276	LOC_Os01g64770	37582170	TGCCTACAGAACAACGCTCA	AGGTTGCTGGAAACCAAACA	60.6	204	75	polypyrimidine tract-binding protein putative expressed
OsISM09277	LOC_Os01g64770	37582351	GTTTGTTTGGTTTCCAGCAA	CGGCTATTATCTGCAGGGTT	58.9	185	79	polypyrimidine tract-binding protein putative expressed
OsISM09278	LOC_Os01g64770	37582453	TCTTTTGATCGTCTCTCTGCTG	AGGCAATGTTCCACCAAAAG	59.9	235	89	polypyrimidine tract-binding protein putative expressed
OsISM09279	LOC_Os01g64770	37582704	TCAATGACCGTTGCACACTT	ACGCACGATATTCCCATACA	59.5	180	99	polypyrimidine tract-binding protein putative expressed
OsISM09280	LOC_Os01g64770	37582939	TTCAGGCTGAGCTTGCTGTA	GATCAGCAGTAACGGTTGGG	60.2	247	97	polypyrimidine tract-binding protein putative expressed
OsISM09281	LOC_Os01g64830	37637368	GCTGGAGTGCCTGACGAT	CACGTCTGCGACATGACAAT	60.4	323	93	aspartic proteinase nepenthesin precursor putative
OsISM09282	LOC_Os01g64830	37637684	AGACGTGCAGATTTCACCAA	CCAAACGGTGAACAACTTCC	59.8	977	76	aspartic proteinase nepenthesin precursor putative
OsISM09284	LOC_Os01g64890	37663923	GAGCACGCATGCAAAGACT	GGTTCAGCGTGCTAACCTTC	60.0	548	98	CorA-like magnesium transporter protein putative expressed
OsISM09285	LOC_Os01g64890	37664479	GTCAGGAATCTGAAGAGCCG	CATGTCGTCATCCAGCAAGT	59.8	194	88	CorA-like magnesium transporter protein putative expressed
OsISM09286	LOC_Os01g64890	37664740	CGGATTCACACAAGTACGCA	ACGGCCACTCTCTGTCTCAT	60.3	243	81	CorA-like magnesium transporter protein putative expressed
OsISM09287	LOC_Os01g64890	37665056	ATGGAACGCTGAATACCCTG	AGCAGCTGGTTCTGCTTCTC	59.9	682	95	CorA-like magnesium transporter protein putative expressed
OsISM09288	LOC_Os01g64930	37689584	GTTTCGCTCCTGCACTCCT	TATGCCATCGGTTGACAAGA	60.3	1060	65	expressed protein
OsISM09289	LOC_Os01g64930	37690846	TCGGAATGTGACGATCAACT	CCATTCGCCTCAATTTCCT	59.6	760	87	expressed protein
OsISM09290	LOC_Os01g64960	37696222	CTGTTCGGCAGGTCCAAG	CGGTCTTGAACTTTGGCTTC	60.1	186	68	chlorophyll A-B binding protein putative expressed
OsISM09291	LOC_Os01g64960	37696440	GGTTTCACCAAGGAGAACGA	GATTCCCTTCCCGGTGAT	59.9	1475	94	chlorophyll A-B binding protein putative expressed
OsISM09292	LOC_Os01g64970	37709481	GTCGCCGTCAAGTGCATC	GGTGGTTGATGATCTCCCTG	60.9	224	68	CAMK CAMK like.11 - CAMK includes calcium/calmodulin depedent protein kinases expressed
OsISM09293	LOC_Os01g64970	37709685	CAGGGAGATCATCAACCACC	ATCATAAGATGCGTTGGCGT	60.4	640	85	CAMK CAMK like.11 - CAMK includes calcium/calmodulin depedent protein kinases expressed

ISM IDs	Rice gene locus IDs	Physical positions (bp) on chromosomes	Forward primers (5'-3')	Reverse primers (5'-3')	Melting tempearature (T°C)	Expected product size (bp) in PCR	Expected product size (bp) in Q-PCR	Putative Functions
OsISM09294	LOC_Os01g64970	37710358	CGATCGAATCTGTGATCGTG	GCATACCATGTGATGGCAGT	59.8	192	98	CAMK CAMK like.11 - CAMK includes calcium/calmodulin depedent protein kinases expressed
OsISM09295	LOC_Os01g64970	37710507	GCAGCTGATCTGTGGAGTGA	GCTGCCATCCAAGAGAACAT	60.2	272	86	CAMK CAMK like.11 - CAMK includes calcium/calmodulin depedent protein kinases expressed
OsISM09296	LOC_Os01g64970	37710759	ATGTTCTCTTGGATGGCAGC	CCACTGCTGATTTGGGTCTT	60.2	191	97	CAMK CAMK like.11 - CAMK includes calcium/calmodulin depedent protein kinases expressed
OsISM09297	LOC_Os01g64970	37710980	TATCCCGCCGTGAGTATGAT	TGGTCTTCAAATGGGTAGGC	60.1	1939	95	CAMK CAMK like.11 - CAMK includes calcium/calmodulin depedent protein kinases expressed
OsISM09298	LOC_Os01g64970	37712928	CAAGAACATTCGCAAAACCA	GGCAATAAGCTGTTTGCATTC	59.7	198	95	CAMK CAMK like.11 - CAMK includes calcium/calmodulin depedent protein kinases expressed
OsISM09299	LOC_Os01g64970	37713117	CTTATTGCCCGCATTTTTGT	ACGGGTGGCTCTTTATTTCC	60.1	173	68	CAMK CAMK like.11 - CAMK includes calcium/calmodulin depedent protein kinases expressed
OsISM09300	LOC_Os01g64990	37722743	ACTTGAGCTCACTGAGGCAG	AGTAACTATCGCCCAGGGGT	59.1	444	95	GPI transamidase subunit PIG-U domain containing protein expressed
OsISM09301	LOC_Os01g64990	37723193	GGTTCATGTACATCACCAATCG	CATATCCAAAGGCAGCAAGG	60.4	290	92	GPI transamidase subunit PIG-U domain containing protein expressed
OsISM09302	LOC_Os01g64990	37723466	TGCTGCCTTTGGATATGTTATG	TGGACCATATCCCAATAAAAGAA	59.8	193	89	GPI transamidase subunit PIG-U domain containing protein expressed
OsISM09303	LOC_Os01g64990	37723852	TGGATGGTCTTCAGGAGATG	TTGGTGATAGGTCCTTAACTGTGA	59.3	390	67	GPI transamidase subunit PIG-U domain containing protein expressed
OsISM09304	LOC_Os01g64990	37724218	TCACAGTTAAGGACCTATCACCAA	GCCAACGGCAATACCATAAA	60.3	202	95	GPI transamidase subunit PIG-U domain containing protein expressed
OsISM09305	LOC_Os01g64990	37724450	TGCCTTCGTTTACACAGCAA	CAAATAGGCCCAGAAGACCA	60.3	235	90	GPI transamidase subunit PIG-U domain containing protein expressed
OsISM09306	LOC_Os01g64990	37724665	TGGTCTTCTGGGCCTATTTG	GCATGACTGGGCTAAGGAGA	60.2	209	99	GPI transamidase subunit PIG-U domain containing protein expressed
OsISM09307	LOC_Os01g64990	37724858	CTTAGCCCAGTCATGCACAA	ATCCGGTGGCAAAGTAAAAA	59.7	147	71	GPI transamidase subunit PIG-U domain containing protein expressed
OsISM09308	LOC_Os01g64990	37724998	ACCGGATTGGCTTATACCTG	CAAGCCGTAACAAGCAGTCTT	59.2	311	99	GPI transamidase subunit PIG-U domain containing protein expressed
OsISM09309	LOC_Os01g64990	37725221	TTCTGTTTTTGCAGACCGTG	GCTTCCATTCTCAGGTAGCG	59.9	1062	65	GPI transamidase subunit PIG-U domain containing protein expressed
OsISM09310	LOC_Os01g64990	37726630	GCAAGTCCCAGCTGCTTC	CATGACCACTTTACCCAGGC	60.0	640	93	GPI transamidase subunit PIG-U domain containing protein expressed
OsISM09311	LOC_Os01g64990	37727316	TTCTGAGAAATCGCATTGGA	ATTTGTAGCGCCATCCTTTG	59.7	372	78	GPI transamidase subunit PIG-U domain containing protein expressed

ISM IDs	Rice gene locus IDs	Physical positions (bp) on chromosomes	Forward primers (5'-3')	Reverse primers (5'-3')	Melting tempearature (T˚C)	Expected product size (bp) in PCR	Expected product size (bp) in Q-PCR	Putative Functions
OsISM09312	LOC_Os01g64990	37727709	TTTTCTGAGTGCATGAAGCG	TTGCGTAATTTTTGGACAACTG	60.1	2108	78	GPI transamidase subunit PIG-U domain containing protein expressed
OsISM09313	LOC_Os01g64990	37729870	GCGAAGGATTCACTGGAAAT	CCTCCTGACCTATGCATCTCA	59.7	380	85	GPI transamidase subunit PIG-U domain containing protein expressed
OsISM09314	LOC_Os01g64990	37730267	TACCGTGAATTCATGCCGT	TCATGAACCACATAAAAAGACCA	59.7	292	97	GPI transamidase subunit PIG-U domain containing protein expressed
OsISM09315	LOC_Os01g64990	37730471	TGAATTTGTGTTCTTGCAGTCA	GCTCCTTGTTTTCCTGACCA	59.8	174	96	GPI transamidase subunit PIG-U domain containing protein expressed
OsISM09317	LOC_Os01g65000	37735167	GGCCTCGTCTGCATCACT	AGAGGATGCCCATCACGA	60.1	935	68	ammonium transporter protein putative expressed
OsISM09319	LOC_Os01g65070	37762789	GAGATCTACCACCGCGTAGC	ACAGTGTCTGCAAATGTGGG	59.7	274	92	hydrolase alpha/beta fold family domain containing protein expressed
OsISM09320	LOC_Os01g65070	37763081	GGCCACCTTCGTTCACATAC	CTTTGAATCAGTGGCAAGCA	60.2	1565	90	hydrolase alpha/beta fold family domain containing protein expressed
OsISM09321	LOC_Os01g65070	37764609	CATTGCTTTGGACTGGCTG	CATCCTCAGAAAGGATCCCA	60.2	137	63	hydrolase alpha/beta fold family domain containing protein expressed
OsISM09322	LOC_Os01g65070	37764742	GATGCTTCAGCACCACTTGT	TTCGTTGCCATGGAATAAGC	59.9	217	93	hydrolase alpha/beta fold family domain containing protein expressed
OsISM09323	LOC_Os01g65070	37764977	AAGCAATCACAGGGGTCTTG	CATCCAGCATTGTAGAGGCA	60.0	267	61	hydrolase alpha/beta fold family domain containing protein expressed
OsISM09324	LOC_Os01g65070	37765315	GTACAAGCATAGGGGCCAAC	CAGATCCCAGGGAGAACAAA	59.7	1189	99	hydrolase alpha/beta fold family domain containing protein expressed
OsISM09325	LOC_Os01g65070	37766456	AATACTCCTGTGGCTGGTGC	ACAAGCTTGCGGGAAATAAA	59.9	706	81	hydrolase alpha/beta fold family domain containing protein expressed
OsISM09326	LOC_Os01g65070	37767169	TTTTACGACAAAGCCCTTGC	TTTGCAAGTCGAACCAACAC	60.0	218	75	hydrolase alpha/beta fold family domain containing protein expressed
OsISM09327	LOC_Os01g65070	37767358	CATGAACCTGTGTTGGTTCG	AAGTGGCATGGTGGTCAAAT	60.1	527	83	hydrolase alpha/beta fold family domain containing protein expressed
OsISM09328	LOC_Os01g65070	37767865	ATTTGACCACCATGCCACTT	GCAACGGCGGTAGTAGGTAT	59.7	379	65	hydrolase alpha/beta fold family domain containing protein expressed
OsISM09329	LOC_Os01g65070	37768289	GTCAATGCTTTGGATGACCC	GCCATTCGGAGTAGTAGCCA	60.3	180	97	hydrolase alpha/beta fold family domain containing protein expressed
OsISM09330	LOC_Os01g65070	37768453	TACTACTCCGAATGGCGGTC	GGAACTCAGAAACAGCTCCG	60.0	205	84	hydrolase alpha/beta fold family domain containing protein expressed
OsISM09331	LOC_Os01g65070	37768638	CGGAGCTGTTTCTGAGTTCC	GACGACTCCAAGGAAGAACG	59.9	196	97	hydrolase alpha/beta fold family domain containing protein expressed

ISM IDs	Rice gene locus IDs	Physical positions (bp) on chromosomes	Forward primers (5'-3')	Reverse primers (5'-3')	Melting tempearature (T˚C)	Expected product size (bp) in PCR	Expected product size (bp) in Q-PCR	Putative Functions
OsISM09332	LOC_Os01g65090	37778591	CCTCCTCCTCCTCCTCAAGT	GACGCCATCCTGCACCTC	61.3	173	68	aminotransferase classes I and II domain containing protein expressed
OsISM09333	LOC_Os01g65090	37778905	GGAGCCCGACTTCGACAC	CCAAAGTCCCAGCATTAGGA	61.0	503	96	aminotransferase classes I and II domain containing protein expressed
OsISM09334	LOC_Os01g65090	37779389	CCTAATGCTGGGACTTTGGA	ATTGCTCACCAGCACCTGAT	60.4	186	97	aminotransferase classes I and II domain containing protein expressed
OsISM09335	LOC_Os01g65090	37779588	GCATTACGCAAGCCGTACTT	CAGCCAATGTAGCCATCTCA	60.1	535	97	aminotransferase classes I and II domain containing protein expressed
OsISM09336	LOC_Os01g65090	37780261	CAAAGGAGCTGCTTGAAGAAA	TGCTGGCTGATAGATGATATGC	60.0	188	99	aminotransferase classes I and II domain containing protein expressed
OsISM09337	LOC_Os01g65090	37780472	GCCTGGAATGTGGGATAGAA	GGCTGCAAGGTATCCAAGTC	59.8	278	86	aminotransferase classes I and II domain containing protein expressed
OsISM09338	LOC_Os01g65090	37780755	ACATTTTGTTGCAGCCTGTG	GAGCCCTGCCTTCTGTGATA	60.1	277	77	aminotransferase classes I and II domain containing protein expressed
OsISM09339	LOC_Os01g65090	37781126	AGGAACTGCCAGGTGTGAAG	AGGAGCTGAAGTCGATGAACA	60.2	150	67	aminotransferase classes I and II domain containing protein expressed
OsISM09340	LOC_Os01g65090	37781323	TCCCTCTGCATGTTCCTCTT	ACATGCGGATGCATTTGTC	60.2	178	83	aminotransferase classes I and II domain containing protein expressed
OsISM09342	LOC_Os01g65100	37789646	AACTCCCGAGGATCGATTG	GAAATTTGGCTGTGGAGCAT	60.1	528	91	peptide transporter putative expressed
OsISM09343	LOC_Os01g65100	37790579	GGAGCTCCTTCTTCAACTGG	GTAGTTCCTTGTCCCGAGCA	59.6	170	79	peptide transporter putative expressed
OsISM09344	LOC_Os01g65110	37794672	GAAGTCGTCGGTGTTCGTC	CCCTTGTACGCGAACCTCT	59.8	146	55	POT family protein expressed
OsISM09345	LOC_Os01g65110	37794978	CTCGCCTCCGTCATCTTC	GACGACATCGACAGCGTG	59.7	126	48	POT family protein expressed
OsISM09346	LOC_Os01g65110	37795491	GAGCTGGAGATCAAACCGTC	ACCTCCTCCACGAGGTCAC	60.0	183	85	POT family protein expressed
OsISM09348	LOC_Os01g65120	37800320	CTCCGGCGTTCTCTATCTCC	GCTACCATGGATGAGACCGT	60.6	148	59	peptide transporter putative
OsISM09351	LOC_Os01g65140	37809966	GTCGCGGAGCTCCTACTTC	TTGACGAATAATCCCTTGCC	60.0	406	70	peptide transporter PTR2 putative expressed
OsISM09354	LOC_Os01g65150	37814684	CGGAAGGATACCTCTTGCAC	GAAGCAACTTGGCAAGGAAC	59.8	174	77	proton-dependent oligopeptide transport putative expressed
OsISM09357	LOC_Os01g65169	37824786	CCAAGAAGTCCTTGTCAGCC	CTCTTCTTCTTTCGCCAGCA	60.3	188	99	proton-dependent oligopeptide transport putative expressed

ISM IDs	Rice gene locus IDs	Physical positions (bp) on chromosomes	Forward primers (5'-3')	Reverse primers (5'-3')	Melting tempearature (T˚C)	Expected product size (bp) in PCR	Expected product size (bp) in Q-PCR	Putative Functions
OsISM09360	LOC_Os01g65190	37828893	GTGGACGAAGAGGGTGTTTT	CGCTTCTCCCATAAGCACTG	60.0	874	75	POT domain containing peptide transporter putative expressed
OsISM09361	LOC_Os01g65190	37830131	TAATGAGTTGATGGGCGACA	ACAGGGTGATATCCTGCGAG	60.1	531	90	POT domain containing peptide transporter putative expressed
OsISM09363	LOC_Os01g65200	37833634	CGCTACCGCATCATCATCT	AGAACCGGTGATAGCGTGAG	60.0	520	72	proton-dependent oligopeptide transport putative expressed
OsISM09364	LOC_Os01g65200	37834545	CTATTCGACCAGGTTGGGAA	TGCAGTGAAGATCCCCTGAT	60.3	173	89	proton-dependent oligopeptide transport putative expressed
OsISM09365	LOC_Os01g65200	37834833	AATTGTTTTTGCCCAGTGGA	ATACCGCGAGGGTTCTTTG	60.2	192	90	proton-dependent oligopeptide transport putative expressed
OsISM09367	LOC_Os01g65210	37836578	GCTACCGCACCATCGTCT	GACTCTTCCGGTGCAAGAAC	59.8	1245	86	proton-dependent oligopeptide transport putative expressed
OsISM09368	LOC_Os01g65210	37838167	GAAACCCATTCGGAGAGGTC	CGATGATGACGGTAGCAACT	59.8	166	99	proton-dependent oligopeptide transport putative expressed
OsISM09369	LOC_Os01g65210	37839048	TGTCCCTGGCCTGTTTCTTA	TCATCTTGTACGCCATTGGT	59.8	1682	97	proton-dependent oligopeptide transport putative expressed
OsISM09370	LOC_Os01g65210	37840742	AGGGCCTCAAGGTCATCTCT	CCTATGTGCGTGACATGCTT	60.0	216	92	proton-dependent oligopeptide transport putative expressed
OsISM09371	LOC_Os01g65210	37840936	TCAAGCATGTCACGCACATA	AATAGTCAGGCGACCCCTTC	60.2	195	91	proton-dependent oligopeptide transport putative expressed
OsISM09372	LOC_Os01g65210	37841111	GAAGGGGTCGCCTGACTATT	TGAGGGTGATCGAAATCTGTC	60.3	1285	97	proton-dependent oligopeptide transport putative expressed
OsISM09373	LOC_Os01g65270	37873481	CCCCACTTGGAAGCTCATTA	GACATGCCAGGAGTTCTGTG	59.7	205	98	retrotransposon protein putative unclassified
OsISM09374	LOC_Os01g65310	37886874	TCATCGTCAAGAAGAAGGGG	GGCTCCAAGAGGTACCCATA	59.6	243	83	DUF803 domain containing putative expressed
OsISM09375	LOC_Os01g65310	37887083	CAGGTGTTGGAGGATATGGG	AATGAAGTTGGCAATCTCCC	59.6	181	87	DUF803 domain containing putative expressed
OsISM09376	LOC_Os01g65310	37887298	CGCTAGGAGCACTCAGCATA	CAAAGAACACATCCCAGCAC	59.2	228	95	DUF803 domain containing putative expressed
OsISM09377	LOC_Os01g65310	37887581	TCAGTGGATGAGATTTGGCA	AGCGGTAGCATAGCAAAGGA	60.1	142	58	DUF803 domain containing putative expressed
OsISM09378	LOC_Os01g65310	37887798	GGAATTTGCTCAGTGATTGGA	GTAGCCAGCCTGGTTTATGC	59.9	184	97	DUF803 domain containing putative expressed
OsISM09379	LOC_Os01g65310	37887991	TGGCTGTTTGCAGTGATTTC	TGAAACAACTGCTGCATTGAA	60.1	405	91	DUF803 domain containing putative expressed

ISM IDs	Rice gene locus IDs	Physical positions (bp) on chromosomes	Forward primers (5'-3')	Reverse primers (5'-3')	Melting tempearature (T°C)	Expected product size (bp) in PCR	Expected product size (bp) in Q-PCR	Putative Functions
OsISM09380	LOC_Os01g65310	37888404	TTATGCCATGTTCACAACCC	TGCAATTTTGCTTGCACTCT	59.4	185	83	DUF803 domain containing putative expressed
OsISM09381	LOC_Os01g65310	37888599	GCGGATTTCTGACAGTTCTTG	CTTTGGAGGAAGAGGTGCAT	59.6	189	99	DUF803 domain containing putative expressed
OsISM09382	LOC_Os01g65340	37909610	TTGGTTCGGTGTGCAGACTA	TCGCATATCTTCGGTCTTCA	59.8	519	99	hypothetical protein
OsISM09383	LOC_Os01g65360	37926368	CTCGCTAACCTGGACACTGC	AGGAGAGAACCTGTGGCTGA	60.5	1643	60	hypothetical protein
OsISM09384	LOC_Os01g65370	37931532	GAGCTCATCATCAAGCTCCA	TTGATCTCGTTGTCCGTCCT	59.9	171	87	MYB family transcription factor putative
OsISM09385	LOC_Os01g65380	37949370	GGACAACTACCCCGAGTTCA	GAACGGGCTCATCATTTTGT	60.0	527	86	patellin protein putative expressed
OsISM09386	LOC_Os01g65380	37949940	CCAAATCAGCTGAGACCCTC	TCCTTGAAGAGACCTCCGAA	59.9	167	74	patellin protein putative expressed
OsISM09387	LOC_Os01g65380	37950162	TCATCGAAAGAAACCGTTGA	GTAGCTCACCTCCCATCCAA	59.7	189	85	patellin protein putative expressed
OsISM09388	LOC_Os01g65410	37962833	CATTGCTGATAAGTGCGGTG	TAATCAAAGGGACTACGGCA	59.3	1448	91	serine hydroxymethyltransferase mitochondrial precursor putative expressed
OsISM09389	LOC_Os01g65410	37964591	TGCAGATTGGTTACTGGTGG	TGCCTCACAGACCTTCTCAA	59.6	506	97	serine hydroxymethyltransferase mitochondrial precursor putative expressed
OsISM09390	LOC_Os01g65410	37965140	GCTCAATATCTCCTGGGGGT	CTGCGATCACCTCAAAGTCA	60.1	184	85	serine hydroxymethyltransferase mitochondrial precursor putative expressed
OsISM09391	LOC_Os01g65440	37982272	GAGGAGACCCCCTCCCTC	CCAGCTTTGGTCCTTGGAT	60.5	1223	98	universal stress protein domain containing protein putative expressed
OsISM09392	LOC_Os01g65440	37983553	CAAGCCATCCCATTTCTGTT	ATGCATTGCTCGACGAACTC	60.6	728	70	universal stress protein domain containing protein putative expressed
OsISM09393	LOC_Os01g65460	37993902	CACCTCCATCCACTACCCC	GAACACGTAGGTCTCGACGC	60.5	177	98	beta-galactosidase precursor putative expressed
OsISM09394	LOC_Os01g65460	37994067	ACCTACGTGTTCTGGAACGG	AATCGCACCAAATCGAACC	60.4	174	75	beta-galactosidase precursor putative expressed
OsISM09395	LOC_Os01g65460	37994268	GTACATGATCCTGCGCATTG	GCGTAATGCAGCCACACC	60.7	196	76	beta-galactosidase precursor putative expressed
OsISM09396	LOC_Os01g65460	37994474	GTGTTCCGGACGAACAATG	CGTCGTGAATCTCTTCATGTG	59.6	139	52	beta-galactosidase precursor putative expressed
OsISM09397	LOC_Os01g65460	37994636	AGAACAATTCTTCGCTTCGC	CGCTTGTTCCATATCTCCGT	59.8	190	77	beta-galactosidase precursor putative expressed

ISM IDs	Rice gene locus IDs	Physical positions (bp) on chromosomes	Forward primers (5'-3')	Reverse primers (5'-3')	Melting tempearature (T˚C)	Expected product size (bp) in PCR	Expected product size (bp) in Q-PCR	Putative Functions
OsISM09398	LOC_Os01g65460	37994898	TGGATCATGTGCCAGCAGTA	CTTGGTTGGTGAATTTGGCT	60.6	538	94	beta-galactosidase precursor putative expressed
OsISM09399	LOC_Os01g65460	37995422	ATTCACCAACCAAGCCAAAA	AAATGCAACATCTTCAGGGG	60.1	243	99	beta-galactosidase precursor putative expressed
OsISM09400	LOC_Os01g65460	37995645	CCCCTGAAGATGTTGCATTT	GACCGAAATTTGTTCCACCA	60.3	302	94	beta-galactosidase precursor putative expressed
OsISM09401	LOC_Os01g65460	37995937	AATTTCGGTCGCACTACTGG	CCCATTTCGGAAGTCTCCTA	59.6	180	95	beta-galactosidase precursor putative expressed
OsISM09402	LOC_Os01g65460	37996181	GTCATTTGTTTCTCTGGGGC	GGAATGCAACACATCCTCCT	59.7	346	72	beta-galactosidase precursor putative expressed
OsISM09403	LOC_Os01g65460	37996589	CCTGCTTGGTCGGTCAGTAT	TTTGCAGGGACCATATCCAT	60.1	372	99	beta-galactosidase precursor putative expressed
OsISM09404	LOC_Os01g65460	37997036	CCGAAATGGATTTGTAGACCA	ACCTGCCAGATGACTTCCAT	59.7	261	98	beta-galactosidase precursor putative expressed
OsISM09405	LOC_Os01g65460	37997341	GGCTTTCCTGAACAATGAGC	GTTCTTCCCAGCCCTCAAAT	60.1	391	98	beta-galactosidase precursor putative expressed
OsISM09406	LOC_Os01g65460	37997717	AGGGCTGGGAAGAACAAACT	CCATTCGTACATGGGTCCTC	60.2	292	73	beta-galactosidase precursor putative expressed
OsISM09407	LOC_Os01g65460	37998040	CAGGAATGGAAAACAGAATA ATTG	CCCCTTATCAGCCTTGAACA	59.5	302	99	beta-galactosidase precursor putative expressed
OsISM09408	LOC_Os01g65460	37998361	CACAGTCTGAGCCACCAAAA	TCTCCTTGGGGAACATCAAC	59.9	154	68	beta-galactosidase precursor putative expressed
OsISM09409	LOC_Os01g65460	37998663	AAGTGCAGGAGAGGATGTGG	ATGGATGAAACCATGACCGT	60.2	154	68	beta-galactosidase precursor putative expressed
OsISM09410	LOC_Os01g65460	37999087	GCCACCATCCGAACTCAATA	CAAGGAGACGGTACAGCCAT	60.5	414	69	beta-galactosidase precursor putative expressed
OsISM09411	LOC_Os01g65470	38005226	TCATGTCGCTCAGTTTGTTTG	CCAAAAGTGGAGCCAGTAGG	59.8	249	60	conserved hypothetical protein
OsISM09412	LOC_Os01g65480	38008918	CTTCAAGGCCATCACCGAG	GTTCTCCTGCTGGTCAAGGA	61.1	142	50	dnaJ domain containing protein expressed
OsISM09413	LOC_Os01g65480	38009349	AGGAGGTCAAGTTCACCCG	TTCTTCCACCCTGGTTTCAC	60.0	940	95	dnaJ domain containing protein expressed
OsISM09414	LOC_Os01g65500	38017305	GTCTGCCCCATCGAGAGC	AGATTTGCAATTTCCCCCTT	60.9	818	86	chloride channel protein putative expressed
OsISM09415	LOC_Os01g65500	38018209	GAAAACATAGCCGGGTTCAA	GCATTGCAGCTGATAAACCA	59.9	187	93	chloride channel protein putative expressed

ISM IDs	Rice gene locus IDs	Physical positions (bp) on chromosomes	Forward primers (5'-3')	Reverse primers (5'-3')	Melting tempearature (T°C)	Expected product size (bp) in PCR	Expected product size (bp) in Q-PCR	Putative Functions
OsISM09416	LOC_Os01g65500	38018505	ATCTTAGCGCCCAGTACCCT	ACCATAGGACCTTCCTTGCC	60.2	201	90	chloride channel protein putative expressed
OsISM09417	LOC_Os01g65500	38018879	TCTTCGCACTTGAAGAAGCA	GAATGTCCTCCAGAGAAGCG	59.9	230	57	chloride channel protein putative expressed
OsISM09418	LOC_Os01g65500	38019310	CTCAATCGGATCCTTCGTGT	AGCGAGATGACCACCGTAAG	60.2	186	81	chloride channel protein putative expressed
OsISM09419	LOC_Os01g65500	38020532	GTGCTCTTCCGCCAGCTC	CATGAAGTCGTGTCGCGT	60.8	180	97	chloride channel protein putative expressed
OsISM09420	LOC_Os01g65530	38030455	ATGGCGAGGAACCCTACC	CACGACCTGGCTGAATAAGA	59.4	898	95	RNA recognition motif containing protein putative expressed
OsISM09421	LOC_Os01g65530	38031459	TTCAGGACTTGTTCGGCTTT	GCCATTTGATGAGGGTTTGT	59.8	383	74	RNA recognition motif containing protein putative expressed
OsISM09422	LOC_Os01g65530	38031882	CAAAGCAGCCTCAGTTTGTG	CTGTAGCTATGCACGGGTCC	60.2	252	94	RNA recognition motif containing protein putative expressed
OsISM09423	LOC_Os01g65530	38032100	CAGCATTACCAGTTGGACCC	GCCAGCAGCATTCAAAACAT	60.8	128	77	RNA recognition motif containing protein putative expressed
OsISM09424	LOC_Os01g65530	38032258	CCGTCCTACTAGGCACCAAA	CATCTTCATCGCCTCCTCTC	60.0	1536	54	RNA recognition motif containing protein putative expressed
OsISM09425	LOC_Os01g65550	38047346	CAAGAACACTGGCAGATCCA	ATCAACGCAAGCCCTCATC	60.5	250	77	RNA recognition motif containing protein putative expressed
OsISM09426	LOC_Os01g65550	38047629	TGTAATCTGGCTTCCCTTGG	CACCCTAAAGCTCCTAGCCC	60.1	908	76	RNA recognition motif containing protein putative expressed
OsISM09427	LOC_Os01g65550	38048617	TGCTATCCCACAAGGTCTCC	GCTGGATTTGCAAGAGGAAC	59.9	473	95	RNA recognition motif containing protein putative expressed
OsISM09428	LOC_Os01g65550	38049025	GCAGGTACTCTCCCTATTCTC C	GGGTATTGAGCTCCTCCGTA	58.8	2314	74	RNA recognition motif containing protein putative expressed
OsISM09429	LOC_Os01g65550	38051547	CAATATGGAGGGCCATTGTC	GTTGGAGTCGGCAAAGTAGG	59.9	578	90	RNA recognition motif containing protein putative expressed
OsISM09430	LOC_Os01g65580	38063668	ATGGTCTGCACGTACCACTG	TGTGGCTCGATACTCCTGAA	59.5	434	94	mitotic checkpoint family protein putative expressed
OsISM09431	LOC_Os01g65580	38064082	TTCAGGAGTATCGAGCCACA	CAGTTTGTCCTGGCATGTTG	59.8	378	84	mitotic checkpoint family protein putative expressed
OsISM09432	LOC_Os01g65580	38064529	GAGGTGGGTGACTTTCAGGA	CAGCAGCAATTCGAGAATCA	60.1	375	98	mitotic checkpoint family protein putative expressed
OsISM09433	LOC_Os01g65580	38065011	TCATACTTGCAAGACGACACA A	TTAGCGCCCTGAGTCTCATT	59.7	157	77	mitotic checkpoint family protein putative expressed

ISM IDs	Rice gene locus IDs	Physical positions (bp) on chromosomes	Forward primers (5'-3')	Reverse primers (5'-3')	Melting tempearature (T˚C)	Expected product size (bp) in PCR	Expected product size (bp) in Q-PCR	Putative Functions
OsISM09434	LOC_Os01g65580	38065176	GACGAAGCAAAGTTGCTGAA	AGCCTGTGCTCCATCTCATT	59.5	289	90	mitotic checkpoint family protein putative expressed
OsISM09435	LOC_Os01g65580	38065522	AAAGCTTTTGCAAGAGGAGC	TTGTGTGGGAAGACTTCAACC	59.4	859	75	mitotic checkpoint family protein putative expressed
OsISM09436	LOC_Os01g65580	38066550	TGATATACCTCAAAAGATCGCAGA	TGCAACCTTTAATTCTTTCAACTG	59.9	219	98	mitotic checkpoint family protein putative expressed
OsISM09437	LOC_Os01g65580	38066843	TACTAGGGAGATCAAGCGGC	TCCCTTTCTTCACTGATGGC	59.8	288	55	mitotic checkpoint family protein putative expressed
OsISM09438	LOC_Os01g65580	38067144	GACCATGCTGTGTCGAAATC	TTCGAGATAGATCCGATTCCA	59.4	162	71	mitotic checkpoint family protein putative expressed
OsISM09439	LOC_Os01g65580	38067450	AACTACGCTCACAGGTGGCT	CCATACGCAGTACTTTTGTGC	59.2	712	82	mitotic checkpoint family protein putative expressed
OsISM09440	LOC_Os01g65580	38068206	GCATTGCAAGCTGAATTGAA	TTCACATCAACCACAGTTCCA	60.0	356	93	mitotic checkpoint family protein putative expressed
OsISM09441	LOC_Os01g65580	38068598	TGCAACCCTAGAGAAACGTG	CAAGCCTTTCGGAAAACTGA	59.6	158	73	mitotic checkpoint family protein putative expressed
OsISM09442	LOC_Os01g65580	38068736	TCAGTTTTCCGAAAGGCTTG	GGGATCCCATTTGACTGTTG	60.3	566	78	mitotic checkpoint family protein putative expressed
OsISM09443	LOC_Os01g65580	38069367	ACGAATCTGGAAGCACCAAT	CAAATGCAACCAATACGCTG	59.8	168	88	mitotic checkpoint family protein putative expressed
OsISM09444	LOC_Os01g65580	38069482	CACTTCTCAACATGAAATTGCTC	TTGAAGGATTCCATTGTCAGG	59.4	609	97	mitotic checkpoint family protein putative expressed
OsISM09446	LOC_Os01g65590	38071425	GTCCTTCTCTACTGGCTGCG	GTGGTGAGTCCTGGACACCT	60.1	265	89	galactosyltransferase putative expressed
OsISM09447	LOC_Os01g65590	38071670	AGGTGTCCAGGACTCACCAC	ATCTCCAGAGAAGACACCGC	59.7	156	59	galactosyltransferase putative expressed
OsISM09448	LOC_Os01g65590	38071932	GCCTTCAGCAGCAAGAAGC	ATCACGATCCCCTTCTCCTT	60.6	348	96	galactosyltransferase putative expressed
OsISM09449	LOC_Os01g65590	38072455	GACGCCGACTTCTACGTCA	CCTCGGTCGTGTCCTGTACT	60.1	195	91	galactosyltransferase putative expressed
OsISM09450	LOC_Os01g65590	38072667	GAAGTCCGGACCTGTTCTGT	TACTCCGGCTCGTGGTACTT	59.5	149	52	galactosyltransferase putative expressed
OsISM09451	LOC_Os01g65590	38072866	AAATCTACGCCGTTTCCAAG	AGGGAGACGTCCTCGTTTG	59.7	162	86	galactosyltransferase putative expressed
OsISM09453	LOC_Os01g65600	38080997	GTCGTTGAGGAGGATGTGCT	AAATGGTACCGCTTCCCAC	60.0	575	99	PHD-finger domain containing protein putative expressed

ISM IDs	Rice gene locus IDs	Physical positions (bp) on chromosomes	Forward primers (5'-3')	Reverse primers (5'-3')	Melting tempearature (T˚C)	Expected product size (bp) in PCR	Expected product size (bp) in Q-PCR	Putative Functions
OsISM09454	LOC_Os01g65600	38081639	CCCACAGTCGCTGGAGAAT	CCTCGCCATCCATATCAAAG	60.8	298	56	PHD-finger domain containing protein putative expressed
OsISM09455	LOC_Os01g65600	38082064	GGGCCGTGACATAATGAGTT	CCATTCCATGCTTCTTGGAT	59.9	195	93	PHD-finger domain containing protein putative expressed
OsISM09456	LOC_Os01g65600	38082776	GGACGATGGCCATTTCATAG	GCATCAACTGACTGGTTGGA	60.0	181	91	PHD-finger domain containing protein putative expressed
OsISM09457	LOC_Os01g65610	38087699	GGTTTGGCGACTCGGATG	CCTGACACGTGATGAAAACG	61.4	1271	67	expressed protein
OsISM09458	LOC_Os01g65610	38088950	CGTTTTCATCACGTGTCAGG	GTTAAAGGCCATGCCTCATC	59.8	493	83	expressed protein
OsISM09459	LOC_Os01g65610	38089415	CAAGTGTTGATGAGGCATGG	CAAGTCGGCGTCAAGATCAG	61.0	622	84	expressed protein
OsISM09460	LOC_Os01g65610	38090008	GGCCTAGGGCTGATCTTGAC	CCGAACAGAGTGATGGTTGT	59.8	130	86	expressed protein
OsISM09461	LOC_Os01g65610	38090464	GTTCATGCTCGTCAATGGC	CACCTCCACGGAGGAGAAC	60.4	1861	97	expressed protein
OsISM09462	LOC_Os01g65610	38092300	GATGGGGTTCTCCTCCGT	CCGTCTGCAAGCACGTAGT	60.0	265	74	expressed protein
OsISM09463	LOC_Os01g65610	38092612	CTGAAGCTGCTGGTGGACAA	GAGTCACCGAACTCCTCCAG	61.2	147	99	expressed protein
OsISM09464	LOC_Os01g65610	38092806	ATAGCAACTTCGCCGTCGT	GTTGTGGCAGATCTGGGTG	60.5	195	99	expressed protein
OsISM09465	LOC_Os01g65650	38115307	ACTCAGCTGCTTCGCTGGTA	GCACAATGCCAAAGCTGTAA	60.6	1014	99	receptor-like protein kinase HAIKU2 precursor putative expressed
OsISM09466	LOC_Os01g65720	38153224	CACTTCCTCTCCGTGGGC	TCCTCATCTTCATTCTCCCA	60.0	928	99	expressed protein
OsISM09467	LOC_Os01g65720	38154630	TGGAGAAAACAAAGATGGGC	TGAGTAGCAGCTTACCCCAA	59.3	258	83	expressed protein
OsISM09468	LOC_Os01g65740	38163612	TCTGCAGGTCTTCTTGGGTT	GTCAGCACCATGGCCTACTG	60.8	907	90	plant-specific domain TIGR01615 family protein
OsISM09469	LOC_Os01g65740	38164904	GCCTCTCGGTGGAGCTATC	TCTCCTTCCTCCTTGCAAAA	59.9	924	91	plant-specific domain TIGR01615 family protein
OsISM09470	LOC_Os01g65740	38165808	TTTTGCAAGGAGGAAGGAGA	TCGGCCAGATATAGCGTTTC	60.1	269	96	plant-specific domain TIGR01615 family protein
OsISM09471	LOC_Os01g65780	38196812	GGTTCTTTGGAGACGACGAA	TGCTTTTCTGGGTCCTTCTC	59.8	2005	80	glycosyl transferase putative expressed

ISM IDs	Rice gene locus IDs	Physical positions (bp) on chromosomes	Forward primers (5'-3')	Reverse primers (5'-3')	Melting tempearature (T˚C)	Expected product size (bp) in PCR	Expected product size (bp) in Q-PCR	Putative Functions
OsISM09472	LOC_Os01g65780	38198907	CGATCATGTCTGCTGCATTT	TCCCATATCCACCCACTGAC	60.2	271	98	glycosyl transferase putative expressed
OsISM09473	LOC_Os01g65780	38199751	TGGATCCTGTGAACTTGCTG	TATGCTTCCCTCCGTCTGTC	60.0	382	73	glycosyl transferase putative expressed
OsISM09474	LOC_Os01g65810	38218238	ATGAAGAAGAAGGCGAGCAG	GTTGAGGGACCTTCGGTGTA	59.8	621	73	hypothetical protein
OsISM09475	LOC_Os01g65820	38220142	CTAGTGCTTGGAGGTGGTGG	GGCACATGCAATTCAAGGTT	60.8	450	86	hypothetical protein
OsISM09476	LOC_Os01g65830	38224024	GTCCAGTACCTCATCGGCTC	AGGTACGGGTTGTTCTCCGT	60.2	142	54	acyl-desaturase chloroplast precursor putative expressed
OsISM09477	LOC_Os01g65870	38244847	CACGCATCGTCCATGACTAA	GCAGTATTATCCCAGGCGG	60.6	449	99	hypothetical protein
OsISM09478	LOC_Os01g65870	38245444	CAACAGCGTCGTTCACAGTT	AGGAAGAAGCCTCGGGATAC	59.8	116	71	hypothetical protein
OsISM09479	LOC_Os01g65880	38253167	CACATCGCGAGGTTCTTCTT	AGGCAGCAAGGAATCAGAAA	60.2	304	91	nodulin MtN3 family protein putative expressed
OsISM09480	LOC_Os01g65880	38253416	CATCGCTCTCTTCCTGTTCC	TGTCATGTTGTAAGGCACGC	60.3	533	98	nodulin MtN3 family protein putative expressed
OsISM09481	LOC_Os01g65880	38253929	GCGTGCCTTACAACATGACA	ATCGTCGTCACCAGGATGTT	60.6	990	92	nodulin MtN3 family protein putative expressed
OsISM09482	LOC_Os01g65880	38255112	TTCTCCATCTGCATGTACGC	ATGAACTCCACGCTCTTCGT	59.9	322	72	nodulin MtN3 family protein putative expressed
OsISM09483	LOC_Os01g65880	38255468	GGCACCTCCTGGTTCATCTA	CTGCATCAAGCCCAGAAAG	59.8	638	88	nodulin MtN3 family protein putative expressed
OsISM09485	LOC_Os01g65890	38257348	GTACGACGCCAATCACTGC	CTACCGAATTGACCCTCGAA	60.2	379	58	DNA repair metallo-beta-lactamase putative expressed
OsISM09486	LOC_Os01g65890	38257865	CCTCAAGCTACCAAGCAAGG	GACAAATGGAGCATGAGGATG	60.2	181	77	DNA repair metallo-beta-lactamase putative expressed
OsISM09487	LOC_Os01g65890	38258170	CCCCAGAAATCATCACTGAA	AGCCCTTGCCTCTTCAATCT	59.4	915	99	DNA repair metallo-beta-lactamase putative expressed
OsISM09488	LOC_Os01g65950	38287044	GTCCTCTGCTCCTTCACCGT	CACCAGGTCCACCAGAGAC	60.4	2474	91	thioesterase family protein putative expressed
OsISM09489	LOC_Os01g65950	38289573	TCACCGTCTCCTACCTCGAC	CTCGCCTCCATCTCGATCT	60.4	133	56	thioesterase family protein putative expressed
OsISM09490	LOC_Os01g65986	38299450	GAAGAAGGGGCTCAAGAAGG	TAATCATTCCAGCCCACCAC	60.5	769	96	DUF803 domain containing putative expressed

ISM IDs	Rice gene locus IDs	Physical positions (bp) on chromosomes	Forward primers (5'-3')	Reverse primers (5'-3')	Melting tempearature (T°C)	Expected product size (bp) in PCR	Expected product size (bp) in Q-PCR	Putative Functions
OsISM09491	LOC_Os01g65986	38300187	GTATGAACCATTGTGGTGGG	AGATAGCAGGAGCAAAGGCA	59.3	174	87	DUF803 domain containing putative expressed
OsISM09492	LOC_Os01g65986	38300375	GGTGCGCTAAGCATCATCA	CCCACAACACAGAGGACACA	60.8	200	99	DUF803 domain containing putative expressed
OsISM09493	LOC_Os01g65986	38300634	TGTGGGCTCTTGCTACTGAA	ATATTGGGGGATAAAGCGGA	60.0	180	93	DUF803 domain containing putative expressed
OsISM09494	LOC_Os01g65986	38300804	CCCCCAATATGGTCAGACAC	TTCCAAGTGCTTTCACACTCA	59.8	210	90	DUF803 domain containing putative expressed
OsISM09495	LOC_Os01g65986	38301077	CCATTGTTGTTGTTGCATGT	GCGAAACAACTGCAGTATTGA	58.4	372	82	DUF803 domain containing putative expressed
OsISM09496	LOC_Os01g65986	38301431	ATACTGCAGTTGTTTCGCCT	AGTTGGATTTTGACGATCCC	58.2	2510	99	DUF803 domain containing putative expressed
OsISM09497	LOC_Os01g65986	38303969	TTACCATCCTTTCTGGGACG	CATCATGTTTCGGTATTCGG	59.4	164	94	DUF803 domain containing putative expressed
OsISM09499	LOC_Os01g66050	38328233	TGGTGCACTGTATTCTGGACA	GCAGATCCCGAATAGCTCTC	59.6	242	99	wound-responsive family protein putative expressed
OsISM09500	LOC_Os01g66050	38328459	GCTATTCGGGATCTGCAAAA	TGGCCATTTGGATCTACCTC	60.0	169	78	wound-responsive family protein putative expressed
OsISM09501	LOC_Os01g66050	38328671	CTAGCCAAAGTTGCAAGGCT	ACAAGATGCCCAACTATGCC	59.8	533	90	wound-responsive family protein putative expressed
OsISM09502	LOC_Os01g66050	38329184	GGCATAGTTGGGCATCTTGT	GCCTTCTCTTGTTTAGCGGA	59.8	409	90	wound-responsive family protein putative expressed
OsISM09503	LOC_Os01g66050	38329626	TGAAATGATCAAATCGCGTG	CAGCTGATCCACTGTGGTGT	60.2	158	63	wound-responsive family protein putative expressed
OsISM09504	LOC_Os01g66050	38329845	TGCTTTGGAGGACAGGATCT	GCTTTGAGGGCCCTTATCTT	59.7	320	74	wound-responsive family protein putative expressed
OsISM09505	LOC_Os01g66050	38330145	AAGATAAGGGCCCTCAAAGC	CCGACATTATCCATGGAACC	59.9	587	77	wound-responsive family protein putative expressed
OsISM09506	LOC_Os01g66050	38330753	ATCGAAGGAGCGAAGGAGAT	ATCTTGCAGTTTAGCTGCCG	60.4	326	98	wound-responsive family protein putative expressed
OsISM09507	LOC_Os01g66080	38346459	TCCTCCATTTGTGGTTTCGT	CATCCTTGGTATAAGGGCCA	60.1	186	87	retrotransposon protein putative unclassified
OsISM09508	LOC_Os01g66080	38347664	TCCTCTTGGACAGCACTTGA	GAGCAACACCACGTTGAAGA	59.7	170	71	retrotransposon protein putative unclassified
OsISM09509	LOC_Os01g66100	38381938	CTACTTCTCCAGCACCCTCG	AGCGACAGCTCCTTCATCTC	59.9	189	85	gibberellin 20 oxidase 2 putative expressed

ISM IDs	Rice gene locus IDs	Physical positions (bp) on chromosomes	Forward primers (5'-3')	Reverse primers (5'-3')	Melting tempearature (T°C)	Expected product size (bp) in PCR	Expected product size (bp) in Q-PCR	Putative Functions
OsISM09510	LOC_Os01g66100	38382373	CATGGTCATCAACATCGGC	CCTGTGCAGGCAGCTCTTAT	60.7	1541	68	gibberellin 20 oxidase 2 putative expressed
OsISM09511	LOC_Os01g66130	38409314	GCGACTGAGCTAGTTGGAGG	TTGGCAATAATTGAATCAGCC	60.0	230	78	armadillo/beta-catenin repeat family protein putative expressed
OsISM09512	LOC_Os01g66130	38409580	CAATTCTCTCATGCAGATTGAGTC	TGGAAGCCTCCCTAATGACA	60.4	178	90	armadillo/beta-catenin repeat family protein putative expressed
OsISM09513	LOC_Os01g66130	38411611	CCACCTTTGGTTGCATTGTC	GCTGGTTGCGAAAATAGCTT	60.4	171	83	armadillo/beta-catenin repeat family protein putative expressed
OsISM09514	LOC_Os01g66180	38442706	GATCTTCAGGACCAAGTGCG	TACCAGATTGCCTCCCAAAC	60.4	1198	99	cytochrome c putative expressed
OsISM09515	LOC_Os01g66180	38443941	AGTGCCTGTGATTTGGGAAG	GTCCATTGAACCCCATCTTG	60.1	1090	87	cytochrome c putative expressed
OsISM09516	LOC_Os01g66240	38474746	GTCACCGGGGAGTAGGGTT	AGCAATGGCCTTGATTTGAT	60.3	1607	97	mitochondrion protein putative expressed
OsISM09517	LOC_Os01g66240	38476382	CATTGGCCAACACAAGGATT	GCAATACGTACAAGGCTCCC	60.2	173	95	mitochondrion protein putative expressed
OsISM09518	LOC_Os01g66240	38476515	AGGCCTATGAGCTGGTTCAA	ATGCCATGAAGGAGAACAGC	60.0	221	95	mitochondrion protein putative expressed
OsISM09519	LOC_Os01g66240	38476720	TTCTCCTTCATGGCATCCTT	AAAGTAACCTGCCACATCGG	59.8	254	89	mitochondrion protein putative expressed
OsISM09520	LOC_Os01g66240	38476942	GCTCAGGAATTTCCGATGTG	GCAACAGTATGCGGTCCTCT	60.4	179	93	mitochondrion protein putative expressed
OsISM09521	LOC_Os01g66240	38477108	CGCATACTGTTGCCTCAACT	TTTCCACCAAAGCTGTGACC	60.0	879	95	mitochondrion protein putative expressed
OsISM09522	LOC_Os01g66240	38477958	GTGCTGGTTGGTCACAGCTT	CGGTTTTGCAGCTTGTTCTA	60.2	167	64	mitochondrion protein putative expressed
OsISM09523	LOC_Os01g66240	38478087	CAGTGGCTCTGAGCATGGTA	GAACCTTGCCAGGAGTAGCA	60.2	190	88	mitochondrion protein putative expressed
OsISM09524	LOC_Os01g66240	38478293	CGAAGATCACCCTGCTGAA	AGCATCCACCACTTCTTGCT	59.9	193	80	mitochondrion protein putative expressed
OsISM09525	LOC_Os01g66240	38478466	AGCAAGAAGTGGTGGATGCT	TTCGTCGGAGATTTGTAGCC	60.0	217	79	mitochondrion protein putative expressed
OsISM09526	LOC_Os01g66240	38478721	TGGATTTTTGACCTAAATGGC	TGAACACCCCTTGGTACATTC	59.3	185	93	mitochondrion protein putative expressed
OsISM09527	LOC_Os01g66240	38479012	GAAATGCACGTGCTTGAAGA	ATGCGGAAGGTTGATGAGAG	60.1	330	87	mitochondrion protein putative expressed

ISM IDs	Rice gene locus IDs	Physical positions (bp) on chromosomes	Forward primers (5'-3')	Reverse primers (5'-3')	Melting tempearature (T˚C)	Expected product size (bp) in PCR	Expected product size (bp) in Q-PCR	Putative Functions
OsISM09528	LOC_Os01g66290	38500969	CGAGGACGCCTCTATGAGTT	ATCCTGAAGTGCTGGCAGAG	60.0	1160	86	OsMADS21 - MADS-box family gene with MIKCc type-box expressed
OsISM09529	LOC_Os01g66290	38502125	GGATCCGCTCCAGTGATAGA	TTTGCATTCTGCAAGGTCTG	60.1	287	93	OsMADS21 - MADS-box family gene with MIKCc type-box expressed
OsISM09530	LOC_Os01g66290	38502373	AAAAATGCGCCACCAGATAC	CTCCTTTGCGGTCATATTGC	60.3	282	83	OsMADS21 - MADS-box family gene with MIKCc type-box expressed
OsISM09531	LOC_Os01g66290	38502666	TGAAAATCGGCTTGAAAAGG	TGCATGTATTCGATCTCCGA	60.2	367	79	OsMADS21 - MADS-box family gene with MIKCc type-box expressed
OsISM09532	LOC_Os01g66290	38502995	CAGCATGAGCTGCTGTTCTC	ACTCACCTTGGCTCTCAGGA	59.9	237	91	OsMADS21 - MADS-box family gene with MIKCc type-box expressed
OsISM09533	LOC_Os01g66290	38503179	TGCAGGAAGCAGATCTTCAG	TCATCATGTTCAGCTCGCTC	59.7	202	77	OsMADS21 - MADS-box family gene with MIKCc type-box expressed
OsISM09534	LOC_Os01g66300	38508646	CTCACCCCCTCAGTTCTTTG	TAATTTCTGCCTCTCGGCAA	60.3	669	81	KH domain containing protein putative expressed
OsISM09535	LOC_Os01g66300	38509329	AGTTCTTCCCTTCTGCACCA	TTAGGTGGCAAAGAAGACGC	60.1	752	64	KH domain containing protein putative expressed
OsISM09536	LOC_Os01g66300	38510123	ATTGAGGTTACCTGGCCTCC	AGATGATGCTTGCAGAACCC	60.3	419	98	KH domain containing protein putative expressed
OsISM09537	LOC_Os01g66300	38510564	CAATTACTGGCACCCCTGTT	CAACAACCGACCAACAAAGTT	59.9	186	90	KH domain containing protein putative expressed
OsISM09538	LOC_Os01g66300	38510798	AGGGTTTATATTCGGGGACG	CAGGTGCTCATACCCAGGTT	60.0	797	82	KH domain containing protein putative expressed
OsISM09539	LOC_Os01g66300	38511651	CCGTCTAAACCAAGCTGTGA	GCCAATTCCCTCAGTTGTTG	59.7	193	94	KH domain containing protein putative expressed
OsISM09540	LOC_Os01g66310	38515443	ATCTTGCCACACGAACAGC	GGATCTCAGGTTCGTGCTTC	59.8	110	77	expressed protein
OsISM09542	LOC_Os01g66350	38535380	TCCGTCATACGTCCCATACA	CCCATAGCTCCACCAAAGAA	59.9	155	61	DUF647 domain containing protein putative expressed
OsISM09543	LOC_Os01g66350	38535582	GCAAAGTAACACCAGGAGCC	AAAAGCATTTTTCCGACACG	59.9	497	74	DUF647 domain containing protein putative expressed
OsISM09544	LOC_Os01g66350	38536077	TTTGCACGTCAAGGAAAGAA	CAGCTCCAATCTCCAGCAAT	59.9	181	80	DUF647 domain containing protein putative expressed
OsISM09545	LOC_Os01g66350	38536274	TGCAGCCTTTCCTCAATTTT	GATGTTGATGTAACAGCCGC	59.5	188	79	DUF647 domain containing protein putative expressed
OsISM09546	LOC_Os01g66350	38536527	GCGAATCTGTTGGGAACATT	TGAAGATGCTCAAGCCAGTG	60.0	544	52	DUF647 domain containing protein putative expressed

ISM IDs	Rice gene locus IDs	Physical positions (bp) on chromosomes	Forward primers (5'-3')	Reverse primers (5'-3')	Melting tempearature (T°C)	Expected product size (bp) in PCR	Expected product size (bp) in Q-PCR	Putative Functions
OsISM09547	LOC_Os01g66350	38537104	TTGCCTTCCTTTCATGTGGT	CCACCGCAACAGTAAACCTT	60.3	190	97	DUF647 domain containing protein putative expressed
OsISM09548	LOC_Os01g66350	38537274	AAGGTTTACTGTTGCGGTGG	TCGTCTCCTGTGAGTTCCCT	59.9	463	78	DUF647 domain containing protein putative expressed
OsISM09549	LOC_Os01g66350	38537717	AGGGAACTCACAGGAGACGA	AAACGCTTCTCCAAATCGTG	60.0	171	80	DUF647 domain containing protein putative expressed
OsISM09550	LOC_Os01g66350	38537892	AACCTGCTTCATTTGTTGCC	ATACCTTATCCTTTGCGGGG	60.1	235	82	DUF647 domain containing protein putative expressed
OsISM09551	LOC_Os01g66350	38538132	TTGCTCAAGGATCAAGCAAA	CATGTGACGCATTGATGAAA	59.3	174	86	DUF647 domain containing protein putative expressed
OsISM09552	LOC_Os01g66350	38538386	TCCTGCAAAATTGTTTCTTCC	CCAGGGTTGAGCAGTGATTC	59.9	220	90	DUF647 domain containing protein putative expressed
OsISM09553	LOC_Os01g66360	38541027	CCCCACATGTCGGTAGAGTT	AGACAGCCGAACCCCATT	60.2	1109	95	2-C-methyl-D-erythritol 4-phosphate cytidylyltransferase putative expressed
OsISM09554	LOC_Os01g66360	38542118	AATGGGGTTCGGCTGTCT	GAGACGCTCCTCTCCTTCAC	59.8	184	71	2-C-methyl-D-erythritol 4-phosphate cytidylyltransferase putative expressed
OsISM09555	LOC_Os01g66360	38542297	GTCTCGGTGGTGCTCTTGTC	GCAGCCCCAATAATGGTAGA	60.4	568	83	2-C-methyl-D-erythritol 4-phosphate cytidylyltransferase putative expressed
OsISM09556	LOC_Os01g66360	38542845	TCTACCATTATTGGGGCTGC	TCTGGATCACACACCACTACAA	59.5	188	94	2-C-methyl-D-erythritol 4-phosphate cytidylyltransferase putative expressed
OsISM09557	LOC_Os01g66360	38543009	AGTTGTAGTGGTGTGTGATCCAG	CTCTCTTTCCCTGGGAGTGC	60.2	240	97	2-C-methyl-D-erythritol 4-phosphate cytidylyltransferase putative expressed
OsISM09558	LOC_Os01g66360	38543233	TCCCAGGGAAAGAGAGACAA	TAAGGGCCTTGCAGAATCAT	59.7	201	96	2-C-methyl-D-erythritol 4-phosphate cytidylyltransferase putative expressed
OsISM09559	LOC_Os01g66360	38543422	GCAAGGCCCTTAGTGTCATC	CTTTCACAGGCACACCAAGA	59.8	464	89	2-C-methyl-D-erythritol 4-phosphate cytidylyltransferase putative expressed
OsISM09560	LOC_Os01g66360	38543866	TCTTGGTGTGCCTGTGAAAG	GGAGTTTGCATTTCCCAAAG	59.7	263	97	2-C-methyl-D-erythritol 4-phosphate cytidylyltransferase putative expressed
OsISM09561	LOC_Os01g66360	38544109	CTTTGGGAAATGCAAACTCC	TCTGAGTAAACTGGGCCTCAT	59.2	133	46	2-C-methyl-D-erythritol 4-phosphate cytidylyltransferase putative expressed
OsISM09562	LOC_Os01g66360	38544210	CATTGTAGGTGATGAGGCCC	GGACACGTCATCAGTGACCTC	60.5	175	84	2-C-methyl-D-erythritol 4-phosphate cytidylyltransferase putative expressed
OsISM09563	LOC_Os01g66360	38544416	TCACAGAGGGCTCTTACACAAA	AGCCAGGAGCAAGTCATCAG	60.2	151	57	2-C-methyl-D-erythritol 4-phosphate cytidylyltransferase putative expressed
OsISM09566	LOC_Os01g66440	38573309	ACTACTGCCCTGTGATTGGC	GGCATTTGCAACTTCTTGGT	60.1	1808	66	hypothetical protein

ISM IDs	Rice gene locus IDs	Physical positions (bp) on chromosomes	Forward primers (5'-3')	Reverse primers (5'-3')	Melting tempearature (T°C)	Expected product size (bp) in PCR	Expected product size (bp) in Q-PCR	Putative Functions
OsISM09567	LOC_Os01g66440	38575082	TGGATGGGATATCAGACCAAG	AACAGACGTCCTTCCTCCAA	59.7	199	98	hypothetical protein
OsISM09568	LOC_Os01g66440	38575437	CGACAAGTCACTTCTTGCCA	CGATCGAGATCCTCCAACTC	59.9	1175	78	hypothetical protein
OsISM09569	LOC_Os01g66440	38576660	CTGAGCAGCTCCTCCATTTG	GCTTTTTCTCATCGAATCTCTCA	60.5	538	79	hypothetical protein
OsISM09570	LOC_Os01g66440	38577288	TACTTGGGATGGTACCGCAT	GACTTGTACTTCTGGCACGG	59.3	1548	98	hypothetical protein
OsISM09571	LOC_Os01g66450	38581462	GAAGGAGATCATCGGGTGG	TCCCCATAGGCAGTGAGTTC	60.2	488	96	retrotransposon protein putative unclassified
OsISM09572	LOC_Os01g66450	38582221	GTACGACGCCTTCAACCCT	GGTAAGCGGTGTTGAGCAAT	60.1	156	76	retrotransposon protein putative unclassified
OsISM09573	LOC_Os01g66450	38582555	CAGGCGAATCCAAAGAAGAA	GTCTCCATGTCAGTCGGCTT	60.3	197	99	retrotransposon protein putative unclassified
OsISM09574	LOC_Os01g66450	38582994	GGTCGAGCTCTCCGTCTAAA	TCAGGATTTCTTCTTCATCGC	59.5	171	91	retrotransposon protein putative unclassified
OsISM09575	LOC_Os01g66450	38583283	GAAAAAGGCACTCAGCCAAG	TCAAGAGTTGCGGTCTTCG	60.1	152	76	retrotransposon protein putative unclassified
OsISM09576	LOC_Os01g66450	38583543	GCTCCAAGAATCCAACTTCG	TTCAGAAATCCTGGCAGCTT	59.9	136	62	retrotransposon protein putative unclassified
OsISM09577	LOC_Os01g66450	38583717	CGAACTAGCAAAGCAGAGGG	AGGACATCAAATTGGGCTTG	60.0	143	79	retrotransposon protein putative unclassified
OsISM09578	LOC_Os01g66490	38611002	TTCAACGTCGAGCTCATCAC	AAGAACCACTCCTTCTCCCC	59.8	184	93	no apical meristem protein putative expressed
OsISM09579	LOC_Os01g66490	38611373	ATGAACGAGTATCGCCTTCC	GTACACGCGGCATAGTGAGA	59.5	565	73	no apical meristem protein putative expressed
OsISM09580	LOC_Os01g66510	38627031	CTCATCGTGCTCATCTCCCT	TGAGGAGAGCCTCGAATAGC	60.0	234	98	MLO domain containing protein putative expressed
OsISM09581	LOC_Os01g66510	38627245	GCTATTCGAGGCTCTCCTCA	CTGAAGAATCCCTTGGGACA	59.9	290	89	MLO domain containing protein putative expressed
OsISM09582	LOC_Os01g66510	38627670	CTTGCCGATGGTGGAACAG	TGCAACTGATGGAGTGCTTC	61.3	608	84	MLO domain containing protein putative expressed
OsISM09583	LOC_Os01g66510	38628282	TTCATTTTTGTTCTGGCCATC	TCATTTTCCCAGTGTTTCCA	59.4	189	96	MLO domain containing protein putative expressed
OsISM09584	LOC_Os01g66510	38628457	CACTGGGAAAATGACATCCA	CAAATTGATGCACATGGGTC	59.6	247	71	MLO domain containing protein putative expressed

ISM IDs	Rice gene locus IDs	Physical positions (bp) on chromosomes	Forward primers (5'-3')	Reverse primers (5'-3')	Melting tempearature (T˚C)	Expected product size (bp) in PCR	Expected product size (bp) in Q-PCR	Putative Functions
OsISM09585	LOC_Os01g66510	38628726	GGGTATTGGCAAAGATTCCA	AACTGACCCAGAAAACTGCTT	58.9	196	68	MLO domain containing protein putative expressed
OsISM09586	LOC_Os01g66510	38628936	CATAACAATGCGACTTGGATT T	CTGCTTCCAAAGCTCGTACC	59.5	159	90	MLO domain containing protein putative expressed
OsISM09587	LOC_Os01g66510	38629075	GGTACGAGCTTTGGAAGCAG	CCATTAACATTCAGCAGCAGG	60.3	250	94	MLO domain containing protein putative expressed
OsISM09588	LOC_Os01g66510	38629297	TGATATTCCTGCTGCTGAATG	AGAAGGGGAACGAAGGAGAT	59.0	271	65	MLO domain containing protein putative expressed
OsISM09589	LOC_Os01g66510	38629542	TTTTGGATCTCCTTCGTTCC	CAACCTCATGGGCTAGTTGG	59.8	180	86	MLO domain containing protein putative expressed
OsISM09590	LOC_Os01g66510	38629841	TTCGAGATCGCGTTCTTCTT	TCAGGATAAAAGGGACGTGG	60.0	187	80	MLO domain containing protein putative expressed
OsISM09591	LOC_Os01g66510	38630008	CCACGTCCCTTTTATCCTGA	TGCAGAGGATTTGAACGATG	59.9	147	57	MLO domain containing protein putative expressed
OsISM09592	LOC_Os01g66510	38630133	GCCATCGTTCAAATCCTCTG	TGCTCGTCGAAGATCTCCTT	60.3	210	96	MLO domain containing protein putative expressed
OsISM09594	LOC_Os01g66560	38649864	CCCGATCGACCTCAGCTACT	GCTTCTTGCAGTTTATTTTGCC	61.0	700	61	signal recognition particle 72 kDa protein putative expressed
OsISM09595	LOC_Os01g66560	38650838	TCTGCCTGCTCGTTGATAGA	TTCGTCCACCATAAGCTCTTC	59.5	378	97	signal recognition particle 72 kDa protein putative expressed
OsISM09596	LOC_Os01g66560	38651251	TGTGTCTGCTCAGCTTGCTT	TCCATAGCTTCCTGGGTTTG	60.0	161	58	signal recognition particle 72 kDa protein putative expressed
OsISM09597	LOC_Os01g66560	38651639	TTTTACTCCTCCACGCGAAC	AGCTTGAAGCAAAACAGGGA	60.1	811	99	signal recognition particle 72 kDa protein putative expressed
OsISM09598	LOC_Os01g66590	38676560	TTCGAGCTCTCCAAGTACCA	ATGAACTCCGCCATCGAGT	59.6	201	96	DUF260 domain containing protein putative expressed
OsISM09599	LOC_Os01g66630	38698077	AATGCAAATTACATGCAGCG	TTGGATCCATCTGAAGGGAG	59.9	683	74	S-domain receptor-like protein kinase putative
OsISM09600	LOC_Os01g66630	38699282	ACAAGGCACAAGGCACAGAT	TGCCAATGTTTCTCCCTTCT	60.2	217	85	S-domain receptor-like protein kinase putative
OsISM09601	LOC_Os01g66630	38699498	ATCGGGATCATCATTGTTGG	CCCTTGACACAGGCCTTCTA	60.4	256	85	S-domain receptor-like protein kinase putative
OsISM09602	LOC_Os01g66640	38703799	AAGTTCGCTCTGGGGTTCTT	TTTGCACTGGGATTTTGTTG	59.9	491	89	S-domain receptor-like protein kinase putative
OsISM09603	LOC_Os01g66660	38717956	GCATCGGTCAAGCTCGTG	CCAAACCTTGAGGATCTGGA	60.8	312	63	hypothetical protein

ISM IDs	Rice gene locus IDs	Physical positions (bp) on chromosomes	Forward primers (5'-3')	Reverse primers (5'-3')	Melting tempearature (T°C)	Expected product size (bp) in PCR	Expected product size (bp) in Q-PCR	Putative Functions
OsISM09604	LOC_Os01g66680	38728858	TTTGCACTGCATAAGTTTCAAG	TCTGCAAAATCGTGTTGCTT	58.8	296	72	S-domain receptor-like protein kinase putative expressed
OsISM09605	LOC_Os01g66680	38729133	AAAGCAACACGATTTTGCAG	TTCACTCTTTCCCCCTAGCA	59.4	170	96	S-domain receptor-like protein kinase putative expressed
OsISM09606	LOC_Os01g66680	38729183	GGCTAGAAATCAACATGACGC	GTTACCCGTCCCATCCATCT	60.3	429	93	S-domain receptor-like protein kinase putative expressed
OsISM09607	LOC_Os01g66680	38729596	GGATGGGACGGGTAACAAC	TAACCAGACAGGCAGACAGC	59.5	1623	99	S-domain receptor-like protein kinase putative expressed
OsISM09608	LOC_Os01g66680	38731199	GCTGTCTGCCTGTCTGGTTA	GTACCAAATGCCCACGTACC	59.6	363	76	S-domain receptor-like protein kinase putative expressed
OsISM09609	LOC_Os01g66680	38731517	GCAGCTGGAGGATCTACTGG	CTTTGCCAGAGCACACTTGA	60.1	287	76	S-domain receptor-like protein kinase putative expressed
OsISM09610	LOC_Os01g66680	38731783	TTCAAGTGTGCTCTGGCAAA	ACTGGTTTGAGCCATCTGGA	60.6	176	99	S-domain receptor-like protein kinase putative expressed
OsISM09611	LOC_Os01g66690	38736776	CATGAGCGTGGCACAATG	TCGATCTCTCAATCAAGTCCG	60.6	186	98	ZIP4/SPO22 putative expressed
OsISM09612	LOC_Os01g66690	38737065	CGAGTTCGTCAATGAAGCCT	GGGGTCATTGCAAAGCTAAC	60.0	175	93	ZIP4/SPO22 putative expressed
OsISM09613	LOC_Os01g66690	38737171	TTGAACCCAATATCAAGTGTGC	ATCTGCTTGAATGCCTCGTC	60.1	159	80	ZIP4/SPO22 putative expressed
OsISM09614	LOC_Os01g66690	38737873	TGAGATGCTAAGCAGAGCTGG	GTTGTTGGCCTCCAACTGAT	60.4	189	83	ZIP4/SPO22 putative expressed
OsISM09615	LOC_Os01g66710	38748391	CTCTGGGCCTGCAAGAAG	CTCCACCACCACGTTCCT	59.6	177	91	polygalacturonase putative
OsISM09617	LOC_Os01g66720	38751850	GGACTTCCACGGATCCTACA	CCACTCCAAGCCCTTCAAT	60.0	173	63	NADP-dependent oxidoreductase putative expressed
OsISM09618	LOC_Os01g66720	38752152	CCCTCTCTTACCATTTGGGG	CAAAAACAAACTCGCCCTTC	60.2	734	97	NADP-dependent oxidoreductase putative expressed
OsISM09619	LOC_Os01g66720	38752941	TGGATGCTATGTTGTGGGAA	CATCAAATCCAAACTTGCCC	60.1	149	72	NADP-dependent oxidoreductase putative expressed
OsISM09620	LOC_Os01g66720	38753107	GAGGAGCCTGACTTGACTGC	GCATTGGACCACCTACGTTC	60.3	362	83	NADP-dependent oxidoreductase putative expressed
OsISM09621	LOC_Os01g66740	38758875	GGTGAGATGCCCAAATGAAT	GCATTGGTCCACCTACGTTC	60.1	780	84	inactive receptor kinase At1g27190 precursor putative
OsISM09622	LOC_Os01g66740	38759705	GGGATGGTCTCCCAGAATG	GCATTCTCCAACCCTATGCT	59.7	349	96	inactive receptor kinase At1g27190 precursor putative

ISM IDs	Rice gene locus IDs	Physical positions (bp) on chromosomes	Forward primers (5'-3')	Reverse primers (5'-3')	Melting tempearature (T˚C)	Expected product size (bp) in PCR	Expected product size (bp) in Q-PCR	Putative Functions
OsISM09623	LOC_Os01g66740	38760054	ACCTGCTGCATTTGTTGGTT	TCAGTCGGAGCAGTCTCCAT	60.8	744	94	inactive receptor kinase At1g27190 precursor putative
OsISM09624	LOC_Os01g66740	38760780	GGAGACTGCTCCGACTGAAG	GGTGTTTCAGAAAACTCACCG	59.9	190	78	inactive receptor kinase At1g27190 precursor putative
OsISM09625	LOC_Os01g66740	38760905	AGTTCATCTATTGCAGGCGG	CCATGACGATCTGAGTATTCCA	60.1	367	95	inactive receptor kinase At1g27190 precursor putative
OsISM09626	LOC_Os01g66780	38777054	GCCAGTTCGGTCTGAATCAT	ATATGACGATCTTGGCCAGC	60.1	737	76	retrotransposon protein putative unclassified
OsISM09627	LOC_Os01g66810	38789440	CGGAAACATCGATCCAACA	CGGGACTGCTTGTTCTGTTT	60.4	133	71	conserved hypothetical protein
OsISM09628	LOC_Os01g66810	38789774	GCTCTACTGGCTCTGGTGCT	CTTCATCTGACGTGGCATTG	60.0	315	71	conserved hypothetical protein
OsISM09629	LOC_Os01g66890	38843721	GATCATGTCACATTCCTGCG	GTCGGATGCCACCTTGTACT	60.0	205	87	BTBZ1 - Bric-a-Brac Tramtrack and Broad Complex BTB domain with TAZ zinc finger and Calmodulin-binding domains expressed
OsISM09630	LOC_Os01g66890	38843917	GCATCCGACACATCCTGATA	CCTCTTCATCTGATACTGCTCA	58.3	360	99	BTBZ1 - Bric-a-Brac Tramtrack and Broad Complex BTB domain with TAZ zinc finger and Calmodulin-binding domains expressed
OsISM09631	LOC_Os01g66890	38844496	ATGAGACAAGCCAACCCAAG	TCCAGCCTTCTTGCTCTCTC	60.0	267	90	BTBZ1 - Bric-a-Brac Tramtrack and Broad Complex BTB domain with TAZ zinc finger and Calmodulin-binding domains expressed
OsISM09632	LOC_Os01g66890	38845013	TCTGCACTTGAAACTTGCCA	AAAGATTCCACTTGGCCTCC	60.5	251	92	BTBZ1 - Bric-a-Brac Tramtrack and Broad Complex BTB domain with TAZ zinc finger and Calmodulin-binding domains expressed
OsISM09633	LOC_Os01g66920	38863255	GTCCTCATCACCGGAGACCT	CCACTCCATCTCGTTTTGCT	60.9	1070	73	Ser/Thr protein phosphatase family protein putative expressed
OsISM09634	LOC_Os01g66920	38864492	CCGTGTCCAGAGCATCACT	GCCAACAAACAGATGCTTCC	60.2	237	53	Ser/Thr protein phosphatase family protein putative expressed
OsISM09635	LOC_Os01g66920	38866248	TTCTTCTTTGCCTGGCAGTT	ACTGGCGAGTGGATTACAGG	60.1	179	92	Ser/Thr protein phosphatase family protein putative expressed
OsISM09636	LOC_Os01g66960	38890451	CTCGCGGAGTTCGTCAAG	CATTTACGGCAGTCCTCCAC	60.6	781	48	selenoprotein precursor putative expressed
OsISM09637	LOC_Os01g66960	38891212	GTGGAGGACTGCCGTAAATG	GCTTTCTCATGCACACCTCA	60.3	462	95	selenoprotein precursor putative expressed
OsISM09638	LOC_Os01g66960	38891774	TATGCTTGATGACAAAGGCG	TTGAGAAACTGCCGAATGTG	59.8	525	79	selenoprotein precursor putative expressed
OsISM09639	LOC_Os01g67010	38915300	AGCTCGCAGGACTTCATCC	GACAGCACGAAGTTCCACAC	59.9	161	72	expressed protein
OsISM09640	LOC_Os01g67010	38915843	GGAGGCAGCACATCTACCG	CCTGAGTGCTTGTGCTTGC	61.3	184	99	expressed protein

ISM IDs	Rice gene locus IDs	Physical positions (bp) on chromosomes	Forward primers (5'-3')	Reverse primers (5'-3')	Melting tempearature (T˚C)	Expected product size (bp) in PCR	Expected product size (bp) in Q-PCR	Putative Functions
OsISM09641	LOC_Os01g67030	38924049	TGGAGCTCCGAGAACTTCAT	CACGTACCCTCCCTTGTCC	60.2	214	88	auxin-responsive protein putative expressed
OsISM09642	LOC_Os01g67090	38946539	GGCCCTAAGGACAAGCCTAC	GTGCTGGTGAGAGTACTGCG	59.9	437	55	IQ calmodulin-binding motif domain containing protein expressed
OsISM09644	LOC_Os01g67090	38947944	TCGCCTACGCCTACTCCC	CGTCCACCACCATGGATT	60.7	715	99	IQ calmodulin-binding motif domain containing protein expressed
OsISM09645	LOC_Os01g67100	38953212	AATCAGAGACGGCTCTTTGG	ACTCAGAGGTCCACGCATTC	59.8	174	75	expressed protein
OsISM09646	LOC_Os01g67100	38953366	GAATGCGTGGACCTCTGAGT	CCCGGAGCTTCTAGAACTGAT	60.1	405	98	expressed protein
OsISM09647	LOC_Os01g67100	38953845	AATCATTGGTGGAAAGAGCG	ACCATCTCTTGCATTGGCTC	60.2	772	92	expressed protein
OsISM09648	LOC_Os01g67100	38954653	GGATGACTCTCAGCTGCAAAA	CCTTTTCAACTTGTTTCTGTGC	59.7	528	75	expressed protein
OsISM09649	LOC_Os01g67100	38955633	AACTGGGCTTTGGAGGATCT	TTGGGTTTGTTTCTGATCCC	59.9	251	99	expressed protein
OsISM09650	LOC_Os01g67100	38956112	CAAAAGCAACAGACCTGCAT	ACATCTTGGGCCAACAACA	59.4	165	65	expressed protein
OsISM09651	LOC_Os01g67100	38956264	TGGCCCAAGATGTCCACTTA	CGTCCAAGAGGCTTTCAATC	60.6	771	99	expressed protein
OsISM09652	LOC_Os01g67100	38957356	GTAGTCCTGCTGCTGCTTCA	CGGCATGATCCTATCACCTT	59.6	155	71	expressed protein
OsISM09653	LOC_Os01g67100	38957554	CCGAATGTGTCAAAGTCACAA	CCTAGCTTGCTAAGAACCTCTTTC	59.5	236	99	expressed protein
OsISM09654	LOC_Os01g67100	38957756	TGCAGGTCAAGAAAGAGGTTC	TATGCTCTTGGGCTTTTTGG	59.8	168	75	expressed protein
OsISM09656	LOC_Os01g67126	38968067	TGTCAAGACCCAGAAGACCC	GGCCCTGTAGTCAGTCTTGC	60.0	185	86	ribosomal L18p/L5e family protein putative expressed
OsISM09657	LOC_Os01g67126	38968288	AACACACCGAAGTACCGCTT	ATCACGATATCACCCGCAAT	59.9	201	81	ribosomal L18p/L5e family protein putative expressed
OsISM09658	LOC_Os01g67126	38968533	TTGGTCTCACCAACTATGCG	TCGTACTCCTGGTCCAAACC	59.8	182	89	ribosomal L18p/L5e family protein putative expressed
OsISM09659	LOC_Os01g67126	38968704	CAGGAGTACGAGGGCAATGT	ATGAGGCCAACATCCAAGAG	60.1	183	99	ribosomal L18p/L5e family protein putative expressed
OsISM09660	LOC_Os01g67126	38968895	CCACTGGAAACCGTGTCTTT	TGTCAAGACCACCATCCAAA	60.0	221	55	ribosomal L18p/L5e family protein putative expressed

ISM IDs	Rice gene locus IDs	Physical positions (bp) on chromosomes	Forward primers (5'-3')	Reverse primers (5'-3')	Melting tempearature (T˚C)	Expected product size (bp) in PCR	Expected product size (bp) in Q-PCR	Putative Functions
OsISM09661	LOC_Os01g67126	38969184	CGCAAGTACATCTACGGTGG	GGGCTTGGAATTTCTCAGGT	59.8	265	77	ribosomal L18p/L5e family protein putative expressed
OsISM09662	LOC_Os01g67126	38969545	CCAAATCGACCAAGAAGGAA	AATCTTTCGACGAGACTGGC	59.7	180	92	ribosomal L18p/L5e family protein putative expressed
OsISM09663	LOC_Os01g67134	38972628	CTCTCCCCTCTCCTCGCT	TGGTCTTCTGGGTCTTGACA	59.4	783	98	ribosomal L18p/L5e family protein putative expressed
OsISM09664	LOC_Os01g67134	38973419	CACAAGCGTTTCCAAGTCAA	TTGGTTGGTGAGCCTAATCC	59.9	164	79	ribosomal L18p/L5e family protein putative expressed
OsISM09665	LOC_Os01g67134	38973599	ACACACCGAAGTACCGCTTC	CACGATATCACCAGCAATGG	60.1	198	87	ribosomal L18p/L5e family protein putative expressed
OsISM09666	LOC_Os01g67134	38973843	TTGGTCTCACCAACTATGCG	TCGTACTCCTGGTCCAAACC	59.8	182	89	ribosomal L18p/L5e family protein putative expressed
OsISM09667	LOC_Os01g67134	38974013	CCAGGAGTACGAGGGCAATA	GAGGCCAACATCCAAGAGAG	59.9	188	98	ribosomal L18p/L5e family protein putative expressed
OsISM09668	LOC_Os01g67134	38974211	CCACTGGAAACCGTGTCTTT	TGTCAAGACCACCATCCAAA	60.0	223	55	ribosomal L18p/L5e family protein putative expressed
OsISM09669	LOC_Os01g67134	38974502	CGCAAGTACATCTACGGTGG	CACTGAAGTGGGCTTGGAAT	59.7	288	86	ribosomal L18p/L5e family protein putative expressed
OsISM09670	LOC_Os01g67134	38974876	GCCAAATCAACCAAGAAGGA	TCTTTCGACAAGACTGGCCT	60.0	177	91	ribosomal L18p/L5e family protein putative expressed
OsISM09671	LOC_Os01g67170	38986714	GCAAAGAAATGCCAGATGCT	TCAATTCACCAGTAACCGCA	60.2	867	92	expressed protein
OsISM09672	LOC_Os01g67170	38988616	ATGGCTTTTCTGCTTCATGG	AAAGCAAGCAAACTTTAGCCA	59.7	194	87	expressed protein
OsISM09673	LOC_Os01g67200	38997251	GGCCTTCACCGACAACCT	AAAAGGTTCAAGAGGGCGAT	60.6	1557	92	ribosomal protein L13 family protein putative
OsISM09675	LOC_Os01g67220	39004952	GGGAGGCAAAGGAGATAACA	CTTCTCCACTGCTCCCATCT	59.3	153	75	Os1bglu4 - beta-glucosidase-like protein without signal sequence expressed
OsISM09676	LOC_Os01g67220	39005085	AGATGGGAGCAGTGGAGAAG	CAAACTGGCCATGAGTTCAA	59.5	276	74	Os1bglu4 - beta-glucosidase-like protein without signal sequence expressed
OsISM09677	LOC_Os01g67220	39005355	AGTTTGGGTTTCCGTGCTTA	AAAAGGCAACTCCTTGCTCA	59.8	241	95	Os1bglu4 - beta-glucosidase-like protein without signal sequence expressed
OsISM09678	LOC_Os01g67220	39005576	TGAGCAAGGAGTTGCCTTTT	CAGAGTTGCGTATGGCTCAA	60.0	152	74	Os1bglu4 - beta-glucosidase-like protein without signal sequence expressed
OsISM09679	LOC_Os01g67220	39005751	TCTTCAGCAGACTGTGGGTG	TCTCCAAAATTTGCAAAGCA	59.7	256	88	Os1bglu4 - beta-glucosidase-like protein without signal sequence expressed

ISM IDs	Rice gene locus IDs	Physical positions (bp) on chromosomes	Forward primers (5'-3')	Reverse primers (5'-3')	Melting tempearature (T°C)	Expected product size (bp) in PCR	Expected product size (bp) in Q-PCR	Putative Functions
OsISM09680	LOC_Os01g67220	39006135	AAATCTTGGCTCATGCTGCT	TTCACAATCGACAACCAAGC	59.8	294	90	Os1bglu4 - beta-glucosidase-like protein without signal sequence expressed
OsISM09681	LOC_Os01g67220	39006476	ACGAAGGCTTGACTTTCAGC	TCGCTGACGCATACTTTCTG	59.9	446	77	Os1bglu4 - beta-glucosidase-like protein without signal sequence expressed
OsISM09682	LOC_Os01g67220	39007056	CCGAGTACAACAAGTGGAGAGA	CAGTTCTGACCCTTTCACCAA	59.8	569	69	Os1bglu4 - beta-glucosidase-like protein without signal sequence expressed
OsISM09683	LOC_Os01g67220	39007576	GCAGAAAAATGGAACACTGG	TAAGTAATTTCCGGAGGCCC	59.2	179	89	Os1bglu4 - beta-glucosidase-like protein without signal sequence expressed
OsISM09684	LOC_Os01g67220	39007735	GGGCCTCCGGAAATTACTTA	GCCGATTGATCATCTTCCTC	59.9	636	97	Os1bglu4 - beta-glucosidase-like protein without signal sequence expressed
OsISM09685	LOC_Os01g67220	39008419	AGGATACCTCGCGTCAGTTG	TAGCCCACTCGAAGTTGTCC	60.3	179	93	Os1bglu4 - beta-glucosidase-like protein without signal sequence expressed
OsISM09686	LOC_Os01g67270	39046271	GTTGCTTCTCGGTCAAGGCT	TATTAAGCTCGCCATAGCCC	60.6	449	63	expressed protein
OsISM09687	LOC_Os01g67300	39062699	ACGAGAACCTGCCGATCTTT	TTGCTGAGATCACCTAGGGC	60.8	182	98	expressed protein
OsISM09688	LOC_Os01g67300	39063035	GCCAGTTTTACTGGGAACGA	TTGCCTTCTCCACCTTCTTC	59.8	202	80	expressed protein
OsISM09689	LOC_Os01g67300	39063256	CATGGACAGTCTTTACCGCA	ATCTTCAGGATCCACCATGC	59.8	454	59	expressed protein
OsISM09690	LOC_Os01g67300	39063732	CAGAATTTCTTCCTCCAATGTCA	AGAACATGATTGGCGTTGTG	60.0	404	65	expressed protein
OsISM09691	LOC_Os01g67310	39065269	TTCCTGCTGTCATCCTCTCA	CCAGCAATGGCATCAAAGTA	59.6	333	92	patatin putative expressed
OsISM09692	LOC_Os01g67310	39065673	ATGCCAAGGATATTGTCCCA	TATGATGCCCAGGATGTTGA	60.0	315	99	patatin putative expressed
OsISM09693	LOC_Os01g67310	39066104	TGTGAAGTGTGTGAAGCCAA	GCATTCTTCAAGGGCTTACG	59.3	152	64	patatin putative expressed
OsISM09694	LOC_Os01g67310	39066358	CATTATTGATGGTGGCATGG	TCTTCCGCTTGATCTGCTTT	59.9	306	78	patatin putative expressed
OsISM09695	LOC_Os01g67310	39066873	GACCTCTTTCAAGCAATCGG	TCCTTCGTCGAGAGGTCATT	59.8	218	96	patatin putative expressed
OsISM09697	LOC_Os01g67320	39079004	TTCTAGGTTTGGGATTGTGTGA	GGATAATCAATTCTTTCACGATCA	59.4	397	95	expressed protein
OsISM09698	LOC_Os01g67320	39079377	TGATCGTGAAAGAATTGATTATCC	GAGACATCATGAACAAAGCAAATC	59.7	973	84	expressed protein

ISM IDs	Rice gene locus IDs	Physical positions (bp) on chromosomes	Forward primers (5'-3')	Reverse primers (5'-3')	Melting tempearature (T°C)	Expected product size (bp) in PCR	Expected product size (bp) in Q-PCR	Putative Functions
OsISM09699	LOC_Os01g67320	39080324	CAGATTTGCTTTGTTCATGATGT	TGTTGCCTCGATAACACGAG	59.5	604	83	expressed protein
OsISM09700	LOC_Os01g67330	39084541	CTCAACGTCCTCCTCGTCTC	TGGGCTAAATTTGAACTTCCC	60.1	815	97	nucleotide-sugar transporter family protein putative expressed
OsISM09701	LOC_Os01g67330	39085351	GCCCAGTTAGTGTAAACTTCTTGA	TGGCTTCTCTCCCACCTTTA	59.4	327	99	nucleotide-sugar transporter family protein putative expressed
OsISM09702	LOC_Os01g67330	39085660	AAGGTGGGAGAGAAGCCATT	GCAAGAAGAACATTGTTGCG	59.8	404	69	nucleotide-sugar transporter family protein putative expressed
OsISM09703	LOC_Os01g67330	39086054	TTCTTCTTGCAGTTCCTGCTC	CAACATTTTCACTGTTGCAGG	59.5	230	93	nucleotide-sugar transporter family protein putative expressed
OsISM09704	LOC_Os01g67330	39086232	TTGATCCTGTCTTGTGCAGC	TCACCTTTAGGAGCACAGCA	59.8	369	93	nucleotide-sugar transporter family protein putative expressed
OsISM09705	LOC_Os01g67330	39086571	TTCTGGTGATTGCTGTGCTC	TCAACAAAAGAGCAAGGGCT	60.0	301	82	nucleotide-sugar transporter family protein putative expressed
OsISM09706	LOC_Os01g67330	39086926	GTCTTCCTGTTGCTGCTGGT	GAAGCCAATGCAGGAACTGT	60.4	645	65	nucleotide-sugar transporter family protein putative expressed
OsISM09707	LOC_Os01g67330	39087586	GGCTCTGAAAAGCCAGTTTG	GGCAGTGATCACAAGCCCTA	60.6	228	98	nucleotide-sugar transporter family protein putative expressed
OsISM09708	LOC_Os01g67330	39087797	GGCTTGTGATCACTGCCAT	CGGCATTGTTGCAAATAAGA	59.7	582	94	nucleotide-sugar transporter family protein putative expressed
OsISM09709	LOC_Os01g67330	39088377	CGCTCAGGGCATATTATCGT	GATTGTGGCGATTGTTGATG	60.0	324	80	nucleotide-sugar transporter family protein putative expressed
OsISM09710	LOC_Os01g67330	39088739	TCTGACCATAAATTTTGTTCTTGC	CTGCTCGGAACTTCATCCTT	59.5	174	91	nucleotide-sugar transporter family protein putative expressed
OsISM09711	LOC_Os01g67330	39088893	AAGGATGAAGTTCCGAGCAG	CACGTTGACGACTACAGATTCC	59.5	217	79	nucleotide-sugar transporter family protein putative expressed
OsISM09712	LOC_Os01g67330	39089062	ATGGATGCCTTGTTCCTCAG	TCTCATCAGTACCGTGCCTG	60.0	171	95	nucleotide-sugar transporter family protein putative expressed
OsISM09713	LOC_Os01g67350	39097978	CGCAGAGCAGCAAGTCTTC	CTTGTGGTTCTTCCTGCGTT	60.2	147	57	cysteine protease putative expressed
OsISM09714	LOC_Os01g67350	39098105	AACGCAGGAAGAACCACAAG	GAGTTCACGTTCCCCTGAAG	60.0	1877	64	cysteine protease putative expressed
OsISM09715	LOC_Os01g67350	39099990	AAGGCCTGGTAACACAATGG	CCTCTCATCACTGTCGCTTG	59.7	184	98	cysteine protease putative expressed
OsISM09716	LOC_Os01g67364	39113639	TGGAGCTCAAAAACTCATGTAAA	CGAACACATCAGGATGTCCA	59.7	443	75	retrotransposon protein putative Ty3-gypsy subclass expressed

ISM IDs	Rice gene locus IDs	Physical positions (bp) on chromosomes	Forward primers (5'-3')	Reverse primers (5'-3')	Melting tempearature (T˚C)	Expected product size (bp) in PCR	Expected product size (bp) in Q-PCR	Putative Functions
OsISM09717	LOC_Os01g67364	39114062	TGGACATCCTGATGTGTTCG	TCTGCAAACCTTTGTTCGTG	60.2	466	98	retrotransposon protein putative Ty3-gypsy subclass expressed
OsISM09718	LOC_Os01g67364	39114723	GTTGCTTCGGGAAAAACAAA	CTGCCACACTTTATCCCGTC	60.3	951	79	retrotransposon protein putative Ty3-gypsy subclass expressed
OsISM09719	LOC_Os01g67364	39115641	GTTTAAGCATGTTGACGGGA	AAGCCTTGCTAATGCCTCCT	59.5	187	99	retrotransposon protein putative Ty3-gypsy subclass expressed
OsISM09720	LOC_Os01g67364	39115816	TTAGCAAGGCTTCCTGTGGT	CGTTTCCACGTCTTAGCGTT	60.1	211	76	retrotransposon protein putative Ty3-gypsy subclass expressed
OsISM09722	LOC_Os01g67420	39158216	GCTGGGAGATCTTCAAAACAG	CCACCCACATGATGACGATA	59.6	477	98	lipase putative expressed
OsISM09723	LOC_Os01g67420	39158679	CATCATGTGGGTGGCAACTA	TGCCACAACACATATATCATCTTC	59.6	288	95	lipase putative expressed
OsISM09724	LOC_Os01g67420	39158989	CTTATCGCGCATCTGGAACT	TTTGAAACAACTGCGGATGA	60.3	443	86	lipase putative expressed
OsISM09725	LOC_Os01g67420	39159480	TCATCCATTTGCTGAAGCTG	ATAACACCCTGCCGATTGAG	60.0	174	97	lipase putative expressed
OsISM09726	LOC_Os01g67420	39159634	CTCAATCGGCAGGGTGTTAT	CCACCAATTATCGCCTTCAA	60.4	1383	70	lipase putative expressed
OsISM09727	LOC_Os01g67420	39161050	AGTGTACCCCCGGAAGTTCT	ATCGTGCAGGACAACAACAA	60.0	167	82	lipase putative expressed
OsISM09728	LOC_Os01g67420	39161300	TACGGTGGATGACTTGGATG	CATGTCCATGGTGTGGAAAG	59.6	367	93	lipase putative expressed
OsISM09729	LOC_Os01g67420	39161681	TCAGCTCGAGAACTGTACGC	CGCCGAGCAAAGAAGATAAG	59.7	414	95	lipase putative expressed
OsISM09730	LOC_Os01g67420	39162121	ATTGAAATCGTTGGGCATTC	TGTAATTTTGGAAAGCGTCC	58.8	774	81	lipase putative expressed
OsISM09731	LOC_Os01g67420	39162937	CGCAGAAGCATGTTCACAGT	CGGGCAGAAAATTCATCATT	60.0	162	58	lipase putative expressed
OsISM09732	LOC_Os01g67420	39163813	GGACCTTGTTGTTACACCTCG	ATGCCCTTTTCCGAGTTTCT	59.8	404	93	lipase putative expressed
OsISM09733	LOC_Os01g67480	39210798	CCTCCTCTCCCGCCTCAT	GTAGCCGCCCATCCCTCT	62.6	3152	68	helix-loop-helix DNA-binding domain containing protein expressed
OsISM09734	LOC_Os01g67480	39214276	CAGCTTCAGCAACATCGACT	GGAGCTGCAAGAAGTCATCC	59.6	169	84	helix-loop-helix DNA-binding domain containing protein expressed
OsISM09736	LOC_Os01g67480	39214674	AGGATCTCGTGCCGAACAT	ATACCTCAACTTGGCCCTGA	60.1	247	97	helix-loop-helix DNA-binding domain containing protein expressed

ISM IDs	Rice gene locus IDs	Physical positions (bp) on chromosomes	Forward primers (5'-3')	Reverse primers (5'-3')	Melting tempearature (T°C)	Expected product size (bp) in PCR	Expected product size (bp) in Q-PCR	Putative Functions
OsISM09737	LOC_Os01g67480	39214888	ACATCAAGGAGCTTCAGGGC	GTTCTCCAGCCTCAGCAATC	60.8	277	92	helix-loop-helix DNA-binding domain containing protein expressed
OsISM09738	LOC_Os01g67520	39244450	GAGGAGGAAAATGGCACAAA	GTCATGGTGAAACAAACCCC	60.1	188	91	VTC2 putative expressed
OsISM09739	LOC_Os01g67520	39244634	TGACATCAGTTCCTGCGAGA	CAAATTGAGTAGGCCGCTTC	60.2	191	99	VTC2 putative expressed
OsISM09740	LOC_Os01g67520	39244955	TGCTGTTGCAGCTTCTCCTA	TGGATTCGAGGGATCAGAAG	60.0	178	82	VTC2 putative expressed
OsISM09741	LOC_Os01g67520	39245236	GGTGGTTTCGCAACTATCAA	CTTCTCCGTCAGTGCCTTCT	59.1	167	85	VTC2 putative expressed
OsISM09742	LOC_Os01g67520	39245557	CCCTCATCTCTGAATCTGGC	TCGAGGAATTCTTGGCTCAC	60.1	203	92	VTC2 putative expressed
OsISM09743	LOC_Os01g67530	39252986	GCTTCCGTCTTCCCCAAC	TATGCCTTCGATTCCTCTGG	60.4	244	99	AMP-binding enzyme putative expressed
OsISM09744	LOC_Os01g67530	39253327	GTCAGAGGCCCAATCGTCAT	CACCTGTTTTCAACCAGCCT	61.3	1075	89	AMP-binding enzyme putative expressed
OsISM09745	LOC_Os01g67530	39254363	CAGCAACGGTTGACTCAGAA	GGCTGGCGTGAATGTAGTTT	60.1	172	87	AMP-binding enzyme putative expressed
OsISM09746	LOC_Os01g67540	39258038	GAGGTCTCCCAGCGCTTC	GTCGACTCCGTCAAACCGTA	61.1	535	72	AMP-binding domain containing protein expressed
OsISM09748	LOC_Os01g67540	39259407	TGATCGATTGAAGGAGCTGA	AGCATCAGCGATTTCAGGAC	59.9	235	95	AMP-binding domain containing protein expressed
OsISM09749	LOC_Os01g67540	39259622	GTCCTGAAATCGCTGATGCT	CAGTTGCCCAGCTTCTTCAT	60.4	184	57	AMP-binding domain containing protein expressed
OsISM09750	LOC_Os01g67540	39259857	GTCATGAACTGCGTCGCTAA	GCAGGAGATTTCGGTATTGC	59.8	290	84	AMP-binding domain containing protein expressed
OsISM09751	LOC_Os01g67550	39262068	TCTCCTTCCTCCTCACCAAA	GTGCGGTTGTTGATGAAATG	59.9	219	98	FAD binding domain containing protein expressed
OsISM09752	LOC_Os01g67550	39262267	CATTTCATCAACAACCGCAC	GAGACCTCTCAATGTCCCCA	60.0	169	71	FAD binding domain containing protein expressed
OsISM09753	LOC_Os01g67550	39262480	CTCTCTGGCTCCATTCTTGG	TGTCAAAATCTGCAACAAAACA	59.6	132	80	FAD binding domain containing protein expressed
OsISM09754	LOC_Os01g67550	39263059	TTGCACATGACCTGGAACAT	AACTGGAACCACCTTTGTGC	60.0	217	96	FAD binding domain containing protein expressed
OsISM09755	LOC_Os01g67550	39263223	TTTATCCTCCCCAGCAGATG	CCCATCCAACCAATTTGACT	59.8	344	76	FAD binding domain containing protein expressed

ISM IDs	Rice gene locus IDs	Physical positions (bp) on chromosomes	Forward primers (5'-3')	Reverse primers (5'-3')	Melting tempearature (T˚C)	Expected product size (bp) in PCR	Expected product size (bp) in Q-PCR	Putative Functions
OsISM09756	LOC_Os01g67550	39263667	TGATGCTGCTCATCGTTTTC	GCACAATTTCCAAGCCAAAT	60.0	203	86	FAD binding domain containing protein expressed
OsISM09757	LOC_Os01g67550	39263909	ACTTATGAATCAGAGCGCCG	AAGGGCTGCAGGAATAGACA	60.1	187	91	FAD binding domain containing protein expressed
OsISM09758	LOC_Os01g67550	39264092	CCTTGGTCTTGACCCAACAG	CGATTTCTGCAGATTTCTTGG	60.2	244	89	FAD binding domain containing protein expressed
OsISM09759	LOC_Os01g67550	39264470	GTTCCCAGCAGAGGATCTTG	GCAACAAGCGCTCCTAATTC	59.9	237	49	FAD binding domain containing protein expressed
OsISM09760	LOC_Os01g67550	39264796	CACGGCTTCCACATATGCTA	GAGCAAACTCAGGCTTGTCC	59.9	187	97	FAD binding domain containing protein expressed
OsISM09761	LOC_Os01g67590	39288478	GCCTCAACCTCGCCTTCT	AGCTGAGAGGTTGACTTGCG	60.6	442	90	oligopeptidase putative expressed
OsISM09762	LOC_Os01g67590	39288960	CGAAGGAAACATACGACGCT	GAGCATCTAATTCCGCCAGA	60.3	434	88	oligopeptidase putative expressed
OsISM09763	LOC_Os01g67590	39289486	TTGGATTCTCACTTCCAGCA	CACCTTTCTGTGTGAATGCC	59.3	499	74	oligopeptidase putative expressed
OsISM09764	LOC_Os01g67590	39289993	GGCCTTGAAGCAACAAGATT	CTCCATCTCCGTTTTCTTGC	59.6	692	91	oligopeptidase putative expressed
OsISM09765	LOC_Os01g67590	39290778	GCTGGGATGCCACTAGAGTT	CAGCAGAACCTTCCACTTCC	59.6	314	64	oligopeptidase putative expressed
OsISM09766	LOC_Os01g67590	39291072	GGAAGTGGAAGGTTCTGCTG	GCCATAAGCAACAGCAATCA	59.8	243	96	oligopeptidase putative expressed
OsISM09767	LOC_Os01g67590	39291307	CTTATGGCCAGAAAGGTGGA	TGGGATACCCCAGCAATCTA	60.2	444	91	oligopeptidase putative expressed
OsISM09768	LOC_Os01g67590	39291773	ACCTAGAATGCCGAGGACG	TTGAGTACGCTGAGCTCCCT	60.2	317	97	oligopeptidase putative expressed
OsISM09769	LOC_Os01g67590	39292070	AGGGAGCTCAGCGTACTCAA	CGCTAAATTGAGCATCACCC	60.4	158	59	oligopeptidase putative expressed
OsISM09770	LOC_Os01g67590	39292301	ATTTTTCCCTGTTGACCTGG	GCCAAGTCTCAGCATCCTTC	59.4	214	96	oligopeptidase putative expressed
OsISM09771	LOC_Os01g67590	39292540	CTGGGATGCAAGTTCAAGTG	TATTCTGAAGTGCCACGACG	59.6	385	99	oligopeptidase putative expressed
OsISM09772	LOC_Os01g67590	39292905	CGTCGTGGCACTTCAGAATA	TTCCCATCAAACTCTTTCGG	60.0	371	97	oligopeptidase putative expressed
OsISM09773	LOC_Os01g67590	39293285	GCTGAGGTTCCCAGAAGTTG	AAAGGTGGCTCTGTTGGAGA	59.8	365	77	oligopeptidase putative expressed

ISM IDs	Rice gene locus IDs	Physical positions (bp) on chromosomes	Forward primers (5'-3')	Reverse primers (5'-3')	Melting tempearature (T˚C)	Expected product size (bp) in PCR	Expected product size (bp) in Q-PCR	Putative Functions
OsISM09774	LOC_Os01g67590	39293665	CTTCAACTGGAGGGGGATTT	ACCTGATGGAAGCCAGACAT	59.9	187	99	oligopeptidase putative expressed
OsISM09775	LOC_Os01g67590	39293793	TGCCATTTCAGGTGCTATGA	AGTGATTGGCAAGCCTCACT	60.0	460	96	oligopeptidase putative expressed
OsISM09776	LOC_Os01g67590	39294265	TGACATATTTGCTGGCCTGA	TCAATGTTCACATTTTCGCC	59.9	419	88	oligopeptidase putative expressed
OsISM09777	LOC_Os01g67590	39294658	CATACAGGCGAAAATGTGAA	ATGAAGCTGGGCTATTCCCT	58.6	178	98	oligopeptidase putative expressed
OsISM09778	LOC_Os01g67590	39294825	CCCAGCTTCATGTTTTCCAC	TTGATGCAAACAGATCAGCC	60.2	185	96	oligopeptidase putative expressed
OsISM09779	LOC_Os01g67590	39295027	CTAAATCAGCATGCGGGG	ATCAAGGGGATTTTTCGAGC	60.3	153	70	oligopeptidase putative expressed
OsISM09780	LOC_Os01g67630	39306631	GGACGTCCAAGAAGGATCG	GTAGGCCGTGTACTGCATGA	60.2	181	78	peptide transporter PTR2 putative expressed
OsISM09781	LOC_Os01g67630	39307506	TCGTGCACACCGACCAGT	GCTCGGCTCTGCCTTGTC	62.4	979	72	peptide transporter PTR2 putative expressed
OsISM09782	LOC_Os01g67640	39311842	CCGATACTGGACCATCGTG	GCTCTCCAGCCTGATCACC	60.4	186	89	PTR-like peptide transporter putatuve expressed
OsISM09783	LOC_Os01g67640	39312412	TCGTCCATGCTGTACGAGAG	CTTGAGACCTTGTTCGTTCG	59.3	404	98	PTR-like peptide transporter putatuve expressed
OsISM09784	LOC_Os01g67640	39312789	TAGGGATCGAACGAACAAGG	TGTGAGCGCATACGTTTGTA	59.5	180	73	PTR-like peptide transporter putatuve expressed
OsISM09785	LOC_Os01g67640	39313069	TTCACTGCGAATGTCTTGGT	TTGGTTTCTTTGTGGTGCAA	59.7	132	98	PTR-like peptide transporter putatuve expressed
OsISM09786	LOC_Os01g67640	39313181	TTGCACCACAAAGAAACCAA	GGCAGTCTTCACCGTAGCTC	60.1	724	96	PTR-like peptide transporter putatuve expressed
OsISM09787	LOC_Os01g67660	39320833	CTCAACCTGGTGCTCACCTT	TCATCCTACACGTCAACAAACC	60.1	1243	99	hypothetical protein
OsISM09788	LOC_Os01g67660	39322000	CGTGTAGGAAAAATGGTGACA	CCATCTATTCCCTCATCCTCA	58.7	145	95	hypothetical protein
OsISM09790	LOC_Os01g67730	39363449	CCAGCAGCTGACAGCAAAAT	AACATACCGTCGCCCAAATA	60.9	402	64	1-5-phosphoribosyl-5-5-phosphoribosylaminomethylideneaminoimidazole-4-carboxamide isomerase chloroplast precursor putative expressed
OsISM09791	LOC_Os01g67730	39363831	TATTTGGGCGACGGTATGTT	GTGAAGATCCCCGTCTTGTG	60.4	289	98	1-5-phosphoribosyl-5-5-phosphoribosylaminomethylideneaminoimidazole-4-carboxamide isomerase chloroplast precursor putative expressed
OsISM09793	LOC_Os01g67760	39380927	TAATTCCGCTCGCTTACTGG	GGTCGAAGACCAGTCGTAGC	60.1	149	63	hypothetical protein

ISM IDs	Rice gene locus IDs	Physical positions (bp) on chromosomes	Forward primers (5'-3')	Reverse primers (5'-3')	Melting tempearature (T°C)	Expected product size (bp) in PCR	Expected product size (bp) in Q-PCR	Putative Functions
OsISM09794	LOC_Os01g67810	39407848	GGAGCTCGTCCAGAAATTCA	ATTCCCCCTGAAAAATCGAC	60.2	181	72	transposon protein putative unclassified expressed
OsISM09795	LOC_Os01g67820	39410711	AAAGAACTGCTCCACGCATT	AAAGCGCATTCCATTTGAAG	60.0	177	81	exo70 exocyst complex subunit domain containing protein expressed
OsISM09796	LOC_Os01g67850	39428272	AAGCTGATATCCACCATGCC	ACAGCCTCATCTGCACACAG	60.0	291	79	zinc finger RING-type putative expressed
OsISM09797	LOC_Os01g67850	39428652	GGTCTCCTTGAGGTTCTTGTG	GGCTTGCTCTCCTTTCAATG	59.4	202	80	zinc finger RING-type putative expressed
OsISM09798	LOC_Os01g67850	39428834	CATTGAAAGGAGAGCAAGCC	CGAGTTGTTGCAAGAATGGA	59.9	153	63	zinc finger RING-type putative expressed
OsISM09799	LOC_Os01g67850	39429155	ATGCCATGTGCATCAACTGT	AGAGGTCCCTTGATCGGACT	60.0	1741	97	zinc finger RING-type putative expressed
OsISM09800	LOC_Os01g67950	39483063	GACGTCTCCATCGACTCCAC	TTGTACAGCAGCCTCTGGTC	59.9	195	96	ubiquitin family protein putative expressed
OsISM09801	LOC_Os01g67960	39486126	CGTCCTCCTCTCGTTCCTC	GTGGATCTCGGTGGACTTGA	60.5	159	56	GPI transamidase component PIG-S-related putative expressed
OsISM09802	LOC_Os01g67960	39486885	CTGCTGAATGCTGATCCAAG	CGGTCGTAACGCCTCAATAA	60.3	425	94	GPI transamidase component PIG-S-related putative expressed
OsISM09803	LOC_Os01g67960	39487302	TACGACCGATTGCGAACAT	AGGACATTGCCACCAAGTTT	59.8	192	98	GPI transamidase component PIG-S-related putative expressed
OsISM09804	LOC_Os01g67960	39487494	TAGCGTGGGAGACATTCCTT	CCAAGTGCCACTCATTTGAA	59.7	182	48	GPI transamidase component PIG-S-related putative expressed
OsISM09805	LOC_Os01g67960	39487682	CCATTTCAGCTACAGGACGA	GTTGCAGGTACAAAGGGCAT	59.4	478	82	GPI transamidase component PIG-S-related putative expressed
OsISM09806	LOC_Os01g67960	39488158	ACTTCCAGATGGGGAGCTTT	GAACCAAATGAACAGCCAGG	60.3	172	94	GPI transamidase component PIG-S-related putative expressed
OsISM09807	LOC_Os01g67960	39488343	GCCCTGGACAAAATGTCATC	TGCCTTAGCTGTCCTATGAAGA	59.7	196	66	GPI transamidase component PIG-S-related putative expressed
OsISM09808	LOC_Os01g67960	39488602	TAGTCCAAAAGGGTTTGCCC	GAAAGGGATTCCAAGGTGGT	60.7	813	97	GPI transamidase component PIG-S-related putative expressed
OsISM09809	LOC_Os01g67960	39489395	ACCACCTTGGAATCCCTTTC	GCCTCCCAATTTCATCCATA	59.9	159	71	GPI transamidase component PIG-S-related putative expressed
OsISM09810	LOC_Os01g67960	39489509	TCCAATCACTCCCCAGAATG	GAGGCTTGCGTTTCTTTGAG	60.5	409	96	GPI transamidase component PIG-S-related putative expressed
OsISM09811	LOC_Os01g67960	39489898	CTCAAAGAAACGCAAGCCTC	AAATGCATCCTCAGCCAAAG	60.2	183	81	GPI transamidase component PIG-S-related putative expressed

ISM IDs	Rice gene locus IDs	Physical positions (bp) on chromosomes	Forward primers (5'-3')	Reverse primers (5'-3')	Melting tempearature (T˚C)	Expected product size (bp) in PCR	Expected product size (bp) in Q-PCR	Putative Functions
OsISM09812	LOC_Os01g67960	39490064	TGGCTGAGGATGCATTTTT	GGAAACTGGCGCAAAGAA	59.6	448	99	GPI transamidase component PIG-S-related putative expressed
OsISM09813	LOC_Os01g67970	39493085	GTGCGCGAACATGGCTAC	CTGTTGTTTTCTGCCCCAAT	60.7	170	71	ZOS1-20 - C2H2 zinc finger protein expressed
OsISM09814	LOC_Os01g67970	39493269	GTGCTTGTGGAGTCGGGTAT	CACAAATTCCCCTGCATTCT	60.0	410	58	ZOS1-20 - C2H2 zinc finger protein expressed
OsISM09815	LOC_Os01g67970	39493658	CAGAATGCAGGGGAATTTGT	TTTGATGCTTCCCCACAGTT	60.2	675	84	ZOS1-20 - C2H2 zinc finger protein expressed
OsISM09816	LOC_Os01g67970	39494401	CTCCCATGGTTTCGCACTAT	GAGCTCCGTGTCTCCATTTC	59.9	907	92	ZOS1-20 - C2H2 zinc finger protein expressed
OsISM09817	LOC_Os01g67970	39495796	CTGGAGGGAGTATTCTGGCA	TCATCGGCCATTATTTCGTT	60.2	568	98	ZOS1-20 - C2H2 zinc finger protein expressed
OsISM09818	LOC_Os01g67970	39497057	TGAGCATGCTATTGAGGTTGA	TGCTTCCGCTTCTATTTTGG	59.9	405	98	ZOS1-20 - C2H2 zinc finger protein expressed
OsISM09819	LOC_Os01g68000	39523303	GAACTCCGCGAAATCTTCC	GACTCCCTCACGTCCTTCAC	59.7	164	51	AML1 putative expressed
OsISM09821	LOC_Os01g68000	39524143	ACGAGAACCACCGTGATGAT	GTTGGAGAGGATGCAGTGGT	60.3	223	91	AML1 putative expressed
OsISM09822	LOC_Os01g68000	39524415	ACGATTTCCTCTACCTCCCC	TGAGGTTGACGAAGCCATAG	59.1	1170	67	AML1 putative expressed
OsISM09823	LOC_Os01g68000	39525661	TTGCCAAGTGACATACGCA	CGGGAACTTGGAGTTCTTGA	60.0	164	71	AML1 putative expressed
OsISM09824	LOC_Os01g68020	39543365	TCGACGTCATCACCTCCTC	GCAGGATGCTCTCCAGCAC	61.0	232	88	BTBZ2 - Bric-a-Brac Tramtrack and Broad Complex BTB domain with TAZ zinc finger and Calmodulin-binding domains expressed
OsISM09825	LOC_Os01g68020	39543697	CTTCGTCCGCCTCCTCTAC	CGCTCTTCTCGTCAATCTCC	60.0	1829	60	BTBZ2 - Bric-a-Brac Tramtrack and Broad Complex BTB domain with TAZ zinc finger and Calmodulin-binding domains expressed
OsISM09826	LOC_Os01g68020	39545734	ATTCCTCCAGGAGAACGACC	CCTCTTCCTCCTCCACTTCC	60.3	905	92	BTBZ2 - Bric-a-Brac Tramtrack and Broad Complex BTB domain with TAZ zinc finger and Calmodulin-binding domains expressed
OsISM09827	LOC_Os01g68020	39546897	CACTGCAACACCCCTCTCT	CACTTATCGTCATCATCGCC	59.0	297	96	BTBZ2 - Bric-a-Brac Tramtrack and Broad Complex BTB domain with TAZ zinc finger and Calmodulin-binding domains expressed
OsISM09828	LOC_Os01g68050	39561550	TCAGCTCATGTTTCCTTCCA	CAGTGGCTGCAGTGGTGTAG	59.9	257	83	transmembrane amino acid transporter protein putative expressed
OsISM09829	LOC_Os01g68050	39561953	TCGGTTTTAGTTTAATCGTTGGA	CAATTGGCGTACCAGGAGG	60.7	195	66	transmembrane amino acid transporter protein putative expressed
OsISM09830	LOC_Os01g68050	39562180	AGAGGTTCATCCGCTACAGG	TGAACTGCAGGAACCAAGTG	59.6	208	85	transmembrane amino acid transporter protein putative expressed

ISM IDs	Rice gene locus IDs	Physical positions (bp) on chromosomes	Forward primers (5'-3')	Reverse primers (5'-3')	Melting tempearature (T°C)	Expected product size (bp) in PCR	Expected product size (bp) in Q-PCR	Putative Functions
OsISM09831	LOC_Os01g68050	39562496	GACCAATTGTAAGCTCGCAG	GCTAATGACAATTATATCTCTCCGC	59.1	471	61	transmembrane amino acid transporter protein putative expressed
OsISM09832	LOC_Os01g68050	39563249	ATCCTCGTGTGCAACACCTC	TGAATAATACTCAACCAATCTTACACC	60.0	466	60	transmembrane amino acid transporter protein putative expressed
OsISM09833	LOC_Os01g68050	39563863	CGTTTAATTTGCCGTGGATT	GTTGGCTCCGAACACGAG	60.1	171	76	transmembrane amino acid transporter protein putative expressed
OsISM09834	LOC_Os01g68104	39587354	ATGGAGACGACCATGGGAT	CCTGGTGGATCACACATCAC	60.0	1017	69	ZOS1-21 - C2H2 zinc finger protein expressed
OsISM09835	LOC_Os01g68104	39588351	GTGATGTGTGATCCACCAGG	CGTTCTCATTCAGCTCACCA	59.9	160	77	ZOS1-21 - C2H2 zinc finger protein expressed
OsISM09836	LOC_Os01g68104	39588659	CTACCAAGCTGCGGATGC	AGCGGAAACATCAGAGATGA	59.5	188	99	ZOS1-21 - C2H2 zinc finger protein expressed
OsISM09837	LOC_Os01g68104	39588743	CTTAACCTTTTGCCTGGCTG	TGGAATAGCCAGTGGAACCT	59.7	179	86	ZOS1-21 - C2H2 zinc finger protein expressed
OsISM09838	LOC_Os01g68104	39588912	GGCTATTCCACTATTCCCCA	ATGCAGCAGCAGCAGACTTA	59.4	268	96	ZOS1-21 - C2H2 zinc finger protein expressed
OsISM09839	LOC_Os01g68104	39589266	GCTTCAGGCGAGGATGAATA	TCCGAACTATCACCATCATCA	59.6	229	81	ZOS1-21 - C2H2 zinc finger protein expressed
OsISM09840	LOC_Os01g68130	39607696	GACCCGATGTCACGCTCC	CTGCAGAATCTCGTCGCC	61.7	343	64	expressed protein
OsISM09841	LOC_Os01g68130	39608399	TCCACGAAGATCGTCCTCTC	ATCCACGCGTAGCATATCG	60.0	167	72	expressed protein
OsISM09842	LOC_Os01g68130	39608676	ACGCCTCTGCTCTGCTCTT	CCAAGCTGACTCGCTTATCC	60.2	701	70	expressed protein
OsISM09843	LOC_Os01g68130	39609338	ACATGGAGGGGCTAATGATG	AGGATTGTGCAAACTGAGCC	60.0	211	98	expressed protein
OsISM09844	LOC_Os01g68130	39609585	TCAGAGCCTGGACATAAAGAAA	TTGCACTGCCTCTGAATCTC	59.0	185	98	expressed protein
OsISM09845	LOC_Os01g68130	39609674	CATGTTTTCTGGGTTCCCC	TTTGTGGACGGCTCTCTGTA	59.8	335	99	expressed protein
OsISM09846	LOC_Os01g68130	39609985	CAACTACAGAGAGCCGTCCA	TTAGCATCTTGGGATGACCC	59.5	492	91	expressed protein
OsISM09847	LOC_Os01g68130	39610457	GGGTCATCCCAAGATGCTAA	TGAGGTCTTTCCACCATTCC	59.9	438	73	expressed protein
OsISM09848	LOC_Os01g68200	39637738	ATGGTGAACACTGGCGAATC	GGCCAATGTCTTGTGATGTG	60.5	772	88	expressed protein

ISM IDs	Rice gene locus IDs	Physical positions (bp) on chromosomes	Forward primers (5'-3')	Reverse primers (5'-3')	Melting tempearature (T˚C)	Expected product size (bp) in PCR	Expected product size (bp) in Q-PCR	Putative Functions
OsISM09849	LOC_Os01g68200	39638720	TTTTGGAATGATGTTTCTCAGTG	TCCACAGATGAAGGTCAGGA	59.2	196	99	expressed protein
OsISM09850	LOC_Os01g68200	39638836	CTCTTGCAGGTTTTTGAGCC	TTTGCAGCTACCATGATTTCC	60.0	121	84	expressed protein
OsISM09851	LOC_Os01g68200	39638962	TGGAGAACACCTACAATCCCA	AGCCCCACTTGCTTCATAGA	60.1	857	87	expressed protein
OsISM09852	LOC_Os01g68220	39649045	GTCCATCTGGACAGGCAAGT	GTGGTTCCTGTCAATCCCAC	60.2	460	75	transposon protein putative unclassified
OsISM09854	LOC_Os01g68220	39650016	CTAGCAAGGCTTGGTTGCTC	AACCACAACAGGTACGCCTC	60.1	184	98	transposon protein putative unclassified
OsISM09855	LOC_Os01g68220	39650510	ACGATGGGGACTTACTGGTG	GGAGACGGTCAAACTCGAAC	59.8	188	95	transposon protein putative unclassified
OsISM09856	LOC_Os01g68220	39650870	CGGACTTAGGCAGGTGTTTC	CTAGCTGTCCCCGTCGAGT	60.1	159	81	transposon protein putative unclassified
OsISM09857	LOC_Os01g68220	39651237	GCACCGTGACCGAGACTACT	ATATCGGCACTGATATCCCG	60.0	152	58	transposon protein putative unclassified
OsISM09858	LOC_Os01g68220	39651570	GGAGCAGGACCTCGACCTAC	ACGTGCTCGAAGGCAGAC	60.7	307	59	transposon protein putative unclassified
OsISM09859	LOC_Os01g68220	39651945	AGCTCTTCCTCTCACGGAGC	AGACGAAGAGGCCCCAGTA	60.5	144	82	transposon protein putative unclassified
OsISM09860	LOC_Os01g68220	39652275	TACTCCCAGGGGCACATTAG	CCGAAAGTGTGTTGTCGTGA	60.4	234	97	transposon protein putative unclassified
OsISM09861	LOC_Os01g68240	39656972	AGGGGGCTATGGCTTGGT	TCTCCCTCCCTTCTCCCTAC	60.7	576	55	hypothetical protein
OsISM09862	LOC_Os01g68240	39657528	GTAGGGAGAAGGGAGGGAGA	CACAAGAATAGCAGGGATTGC	59.7	1808	84	hypothetical protein
OsISM09864	LOC_Os01g68250	39662186	TCTTACACCTCCAACCTGCC	TGCCTAGCAGATCCCAAAAT	59.9	249	83	expressed protein
OsISM09865	LOC_Os01g68250	39662475	GATCAGAGGCAACCTAACTGC	CCACGGCGCAATACTATTTT	59.5	305	95	expressed protein
OsISM09866	LOC_Os01g68250	39662760	AAAATAGTATTGCGCCGTGG	ATCCAATCTCTGGCCTTCCT	60.0	836	99	expressed protein
OsISM09867	LOC_Os01g68260	39665616	CAAAGCTTGCCATTCTAGGC	ATGCCCTCATTTGATCTTGG	59.9	307	53	lysine ketoglutarate reductase trans-splicing related 1 putative expressed
OsISM09868	LOC_Os01g68260	39665966	TTGTTTGGGATTTCATTTCCT	TGGGTTGTGATACCAGAGTCTTT	59.2	184	99	lysine ketoglutarate reductase trans-splicing related 1 putative expressed

ISM IDs	Rice gene locus IDs	Physical positions (bp) on chromosomes	Forward primers (5'-3')	Reverse primers (5'-3')	Melting tempearature (T°C)	Expected product size (bp) in PCR	Expected product size (bp) in Q-PCR	Putative Functions
OsISM09869	LOC_Os01g68260	39666160	AATCATGCATGGACATCTGC	GCACCTTTAGGGTTTGTTGG	59.5	485	99	lysine ketoglutarate reductase trans-splicing related 1 putative expressed
OsISM09870	LOC_Os01g68260	39666695	GAAGATTGTGGGGTGAACCT	TGCTGCATCAATATTTGCCT	59.1	225	99	lysine ketoglutarate reductase trans-splicing related 1 putative expressed
OsISM09871	LOC_Os01g68260	39666896	CAAAAGGCAAATATTGATGCAG	TCCCAGTCATTAGTCCGACC	59.8	277	90	lysine ketoglutarate reductase trans-splicing related 1 putative expressed
OsISM09872	LOC_Os01g68260	39667189	AAAAGGGCCATTCATGTGAG	TGTAGTCATACGGGGCAACA	60.0	198	92	lysine ketoglutarate reductase trans-splicing related 1 putative expressed
OsISM09873	LOC_Os01g68260	39667367	TGTTGCCCCGTATGACTACA	CCAACCCATGCTTCCTAACA	60.4	179	99	lysine ketoglutarate reductase trans-splicing related 1 putative expressed
OsISM09874	LOC_Os01g68260	39667597	CCAAACGGCTAGGAGATCAA	GATGTGGATCAGTGCACCAG	60.2	159	70	lysine ketoglutarate reductase trans-splicing related 1 putative expressed
OsISM09875	LOC_Os01g68260	39667736	CTGGTGCACTGATCCACATC	GCCATGCATCCCTAGAGAAC	59.9	329	78	lysine ketoglutarate reductase trans-splicing related 1 putative expressed
OsISM09876	LOC_Os01g68260	39668044	TGTTCTCTAGGGATGCATGG	TCTAAGAGCGAAATCGAGGC	59.2	153	84	lysine ketoglutarate reductase trans-splicing related 1 putative expressed
OsISM09877	LOC_Os01g68260	39668158	GACTTAGTCCATGGATGGGG	ACTCCGATTTTCTCGTGAGC	59.3	462	72	lysine ketoglutarate reductase trans-splicing related 1 putative expressed
OsISM09878	LOC_Os01g68260	39668645	CAGGTTATTCCGTCACTGGG	CCCATGGTGTTCTTCCATTC	60.3	141	56	lysine ketoglutarate reductase trans-splicing related 1 putative expressed
OsISM09879	LOC_Os01g68260	39668740	TCCTGGTACTGGTAGGGCAC	GGAACATCCCCCATTCTTTT	60.0	485	86	lysine ketoglutarate reductase trans-splicing related 1 putative expressed
OsISM09880	LOC_Os01g68324	39695205	TTTCCGACGCCCACAGAT	ACCGCCTCATACGTCGTC	61.1	245	89	dolichyl-diphosphooligosaccharide--protein glycosyltransferase 63 kDasubunit precursor putative expressed
OsISM09881	LOC_Os01g68324	39695591	CAAGTGCAGTGTTGATTCCG	TCCAGCAGCGAGTTAGTGTC	59.7	174	82	dolichyl-diphosphooligosaccharide--protein glycosyltransferase 63 kDasubunit precursor putative expressed
OsISM09882	LOC_Os01g68324	39695772	TATTCTGTGGGAGGGTTGCT	TGCGTGAAATGTGTTATCTGC	59.7	198	79	dolichyl-diphosphooligosaccharide--protein glycosyltransferase 63 kDasubunit precursor putative expressed
OsISM09883	LOC_Os01g68324	39695908	GTTTGCTTCAGGAACAAGGC	CATCTTCCGTCACTTTGGCT	60.1	207	92	dolichyl-diphosphooligosaccharide--protein glycosyltransferase 63 kDasubunit precursor putative expressed
OsISM09884	LOC_Os01g68324	39696102	GTGACGGAAGATGGCGTTAT	TGCAGACAATGCTTCTAGTGC	59.6	369	81	dolichyl-diphosphooligosaccharide--protein glycosyltransferase 63 kDasubunit precursor putative expressed
OsISM09885	LOC_Os01g68324	39696478	CACTGGCAGATTCGGAAGTT	CCATAGCTCTTGATCGTGTCA	59.6	684	86	dolichyl-diphosphooligosaccharide--protein glycosyltransferase 63 kDasubunit precursor putative expressed
OsISM09886	LOC_Os01g68324	39697113	TGGTGAAAAATGACATTGTGAAG	TTGTATTCGGCAGCATCAAC	59.8	341	98	dolichyl-diphosphooligosaccharide--protein glycosyltransferase 63 kDasubunit precursor putative expressed

ISM IDs	Rice gene locus IDs	Physical positions (bp) on chromosomes	Forward primers (5'-3')	Reverse primers (5'-3')	Melting tempearature (T˚C)	Expected product size (bp) in PCR	Expected product size (bp) in Q-PCR	Putative Functions
OsISM09887	LOC_Os01g68324	39697499	TTTTGCAGCTGTTGCTTCTG	TCGCCAAACCCAGTATTTTC	60.1	149	60	dolichyl-diphosphooligosaccharide--protein glycosyltransferase 63 kDasubunit precursor putative expressed
OsISM09888	LOC_Os01g68324	39697678	GCGCAAAGGATTGCTTTAAC	GGAAGGAAGTGAAAGGACCA	59.5	259	87	dolichyl-diphosphooligosaccharide--protein glycosyltransferase 63 kDasubunit precursor putative expressed
OsISM09889	LOC_Os01g68324	39697944	TTTCGCTGACCTCCAAAGAC	GAGGTGCAGCAGATCCAAAT	60.3	514	64	dolichyl-diphosphooligosaccharide--protein glycosyltransferase 63 kDasubunit precursor putative expressed
OsISM09890	LOC_Os01g68324	39698479	AAGTGCTGGGCTCTGACTCT	TTCAATGGAGCAATATCCAGG	59.6	196	98	dolichyl-diphosphooligosaccharide--protein glycosyltransferase 63 kDasubunit precursor putative expressed
OsISM09891	LOC_Os01g68324	39698681	TGTTGGGAAGTACTCGCTTG	TCGCCTCAGTATTTGTCCCT	59.3	165	90	dolichyl-diphosphooligosaccharide--protein glycosyltransferase 63 kDasubunit precursor putative expressed
OsISM09892	LOC_Os01g68324	39698903	ATGCTGGGACTGTGGAGTCT	TGCAGATGGTTTGCAGAAAG	59.9	466	77	dolichyl-diphosphooligosaccharide--protein glycosyltransferase 63 kDasubunit precursor putative expressed
OsISM09893	LOC_Os01g68324	39699398	CACCACTCGGGAAGACATTT	TGCAGAGCCTGGTACAACAA	60.2	180	99	dolichyl-diphosphooligosaccharide--protein glycosyltransferase 63 kDasubunit precursor putative expressed
OsISM09894	LOC_Os01g68324	39699568	AGGCTCTGCAAGGCAGTTTA	AACTGCAAGCTCCAGGTCAT	60.0	1528	95	dolichyl-diphosphooligosaccharide--protein glycosyltransferase 63 kDasubunit precursor putative expressed
OsISM09895	LOC_Os01g68324	39701085	AGCTTGCAGTTGGTGATGCT	CAATTCAATATGGCCAAGGG	60.6	133	63	dolichyl-diphosphooligosaccharide--protein glycosyltransferase 63 kDasubunit precursor putative expressed
OsISM09896	LOC_Os01g68324	39701371	ACGCTTCTGCCTATTGTTGG	TTCAAGTTTACCCCGAGACG	60.2	168	57	dolichyl-diphosphooligosaccharide--protein glycosyltransferase 63 kDasubunit precursor putative expressed
OsISM09897	LOC_Os01g68324	39701604	TGCTGCTGCTCTACGTTCTC	GGAAGACACCAAGGAAGCTG	59.7	164	79	dolichyl-diphosphooligosaccharide--protein glycosyltransferase 63 kDasubunit precursor putative expressed
OsISM09898	LOC_Os01g68340	39710278	ACCCCCTCTCTTCAGTCGTT	GCCGCTCAGACCTGTTAATC	60.0	266	73	retrotransposon protein putative unclassified
OsISM09899	LOC_Os01g68340	39710634	GCAGAACGTGATGAAGCCAT	TCACTTTCAGAGATGACGCC	60.1	158	89	retrotransposon protein putative unclassified
OsISM09900	LOC_Os01g68340	39710836	AATGTTCCAACAAAGCCGAG	TACTTCTCGCCGATCGTCAC	60.7	201	93	retrotransposon protein putative unclassified
OsISM09901	LOC_Os01g68340	39710995	ACCTCCACGACGACAACG	GACGCACTGGAAGCTCCTC	60.9	163	91	retrotransposon protein putative unclassified
OsISM09902	LOC_Os01g68380	39729353	CTCGCGGAAGAAGGATCTC	GTCTGCAGTTTCAGCAGCCT	60.4	435	95	expressed protein
OsISM09903	LOC_Os01g68380	39730304	GGAAAGTTAGAAGGCACGCA	CCATCTGCTTGGGAAGAGTC	60.1	198	96	expressed protein
OsISM09904	LOC_Os01g68380	39730931	CACAATTCCTGGGAAAGATGA	AGGCGAACTTCTCTTTTGTCA	59.5	587	75	expressed protein

ISM IDs	Rice gene locus IDs	Physical positions (bp) on chromosomes	Forward primers (5'-3')	Reverse primers (5'-3')	Melting tempearature (T˚C)	Expected product size (bp) in PCR	Expected product size (bp) in Q-PCR	Putative Functions
OsISM09905	LOC_Os01g68380	39731557	CTTTGGCCACAGACAACTCA	TTCGTTCTGCAATCTCTCCA	59.7	548	96	expressed protein
OsISM09906	LOC_Os01g68380	39732085	TGGAGAGATTGCAGAACGAA	ATTGCATTCTAGCTGGGCAT	59.6	813	87	expressed protein
OsISM09907	LOC_Os01g68380	39732906	TGAGAGGGCCAAAGTTCTTG	CCCTCAATTTCCTTTTCAAGC	60.2	492	97	expressed protein
OsISM09908	LOC_Os01g68380	39733377	GCTTGAAAAGGAAATTGAGGG	TTGAAGATGTTGACGCTCCTT	60.0	360	89	expressed protein
OsISM09909	LOC_Os01g68380	39733716	AAGGAGCGTCAACATCTTCAA	CCTTCTCAGTCACAGAAGTATTC C	59.2	417	95	expressed protein
OsISM09910	LOC_Os01g68380	39734168	GATGCATCAACTGCAACAGAA	CAATGGCACTGGTAAGGGTT	59.9	168	71	expressed protein
OsISM09911	LOC_Os01g68380	39734415	TAGCATCCCACATGATCAGC	AGCTCGCAGCAACTCTTCTC	59.8	242	89	expressed protein
OsISM09912	LOC_Os01g68380	39734665	TGAATCATCCAATTGCTCCA	CCAGCTTTTGCGTTAGATCC	59.9	358	57	expressed protein
OsISM09913	LOC_Os01g68380	39735093	GATCGACACACGCTCCATC	CATTATCCATCCAAGCACCC	60.2	292	83	expressed protein
OsISM09914	LOC_Os01g68410	39750178	TTCCTTCTGGCCACTCAGAC	TGATTTACTTCTGGCTCGGC	60.4	995	81	retrotransposon protein putative Ty3-gypsy subclass
OsISM09915	LOC_Os01g68410	39753098	ATGTGCGACCTAGCTCATCC	CATCGATGAACACCACGAC	59.3	381	94	retrotransposon protein putative Ty3-gypsy subclass
OsISM09916	LOC_Os01g68410	39755032	CAGACCGATGGTCAGACAGA	CTTCCGAACATTGCCTCATT	59.9	173	51	retrotransposon protein putative Ty3-gypsy subclass
OsISM09917	LOC_Os01g68430	39766515	ACCGACAGCTGGTACTGGTT	GAAATCAGCCCCCATATGAC	59.4	204	97	transposon protein putative unclassified
OsISM09918	LOC_Os01g68430	39767439	TGTGGCATACCAGATGCAAT	GTCTCTGTGAACTCCCCAGC	59.9	113	75	transposon protein putative unclassified
OsISM09919	LOC_Os01g68430	39767754	TCAGGATCAACAGCAGATGG	GACCGGTGTCGGTGATCTAT	59.8	509	93	transposon protein putative unclassified
OsISM09920	LOC_Os01g68430	39768252	CGACACCGGTCTCACCTTAC	CCGACACATCGGTAGGAGG	61.0	213	99	transposon protein putative unclassified
OsISM09921	LOC_Os01g68430	39768493	ACCCGGCCAGACAATAGAC	AGTCCTCAACAAAAGTCGCC	59.6	246	53	transposon protein putative unclassified
OsISM09922	LOC_Os01g68430	39768873	CACGTTGCGAGCACTAGAGA	GTTGAACCATCAACGACGAC	59.7	190	96	transposon protein putative unclassified

ISM IDs	Rice gene locus IDs	Physical positions (bp) on chromosomes	Forward primers (5'-3')	Reverse primers (5'-3')	Melting tempearature (T˚C)	Expected product size (bp) in PCR	Expected product size (bp) in Q-PCR	Putative Functions
OsISM09923	LOC_Os01g68430	39769247	CACGTTCCTAGGCCGACTC	ACAACAGAAGGCCTAGCCG	60.6	161	84	transposon protein putative unclassified
OsISM09924	LOC_Os01g68430	39769345	GATTGCTTCAGGGTTTCGTG	CTGCGTCATAGCGAACTGG	60.6	184	98	transposon protein putative unclassified
OsISM09925	LOC_Os01g68440	39772134	GTTCGTCCCCTTCATGGTC	ATCGCCCAATTCATCTTTCA	60.4	190	95	expressed protein
OsISM09926	LOC_Os01g68440	39772304	TGAAAGATGAATTGGGCGAT	CTCCACCAGCTCCTCCTCT	60.0	200	90	expressed protein
OsISM09927	LOC_Os01g68440	39772481	TCTCAGAGGAGGAGCTGGTG	GCTGTGACAAAGCAGTGGAA	60.4	177	76	expressed protein
OsISM09928	LOC_Os01g68440	39772653	ACAGCTGGAGGATCAGAGGA	TCCAAAGGTGGAACTAGCTGA	59.9	357	53	expressed protein
OsISM09929	LOC_Os01g68440	39773442	AAGAGAGGCAGGCATTTTGA	TTCTGCCATCTTCACTGCTTT	60.0	273	82	expressed protein
OsISM09930	LOC_Os01g68440	39773720	GGCCAAACAAGAGGAAGACA	CTTTGGCCAACACAGAATCA	60.0	679	69	expressed protein
OsISM09931	LOC_Os01g68440	39774405	AGCCATCAGCTGAGAAAGTTG	GCTGGCTTCCCAACACTAGA	60.0	376	91	expressed protein
OsISM09932	LOC_Os01g68440	39774820	ACAAGCTGATGAATGTGGCA	TCAACAGTAGAACGCCTTGTG	59.6	172	92	expressed protein
OsISM09933	LOC_Os01g68440	39775183	TCGGTGCTGGGTACCTCTAC	GTGGATCAACCTCATGTCCC	60.2	311	75	expressed protein
OsISM09934	LOC_Os01g68450	39776825	GTCACCGGCTTCCTCTCC	CAGCCGTCTCTACCTCCTTG	60.4	190	95	expressed protein
OsISM09935	LOC_Os01g68450	39777180	TCACCTGCTACGCCGTCT	GTGATCCACAATGGCCTCTT	60.3	739	86	expressed protein
OsISM09936	LOC_Os01g68450	39777888	GGGAGAAGTACAAGAGGCCA	GGTCAGGTTGCTTGTCGATT	59.7	320	96	expressed protein
OsISM09937	LOC_Os01g68450	39778449	CAGCATCTCGGCTATCATCA	CCGGCCTTTGGAAGAACT	60.1	207	88	expressed protein
OsISM09938	LOC_Os01g68470	39788855	CAGGTACAGTGCAGGACGAC	TTTTCTGCGATGTAGCCAAA	59.4	723	91	hypothetical protein
OsISM09939	LOC_Os01g68470	39789529	AAGATCTTCCGTCCCCAAAT	CGCACGTACGCATAACCAT	60.2	118	86	hypothetical protein
OsISM09940	LOC_Os01g68480	39793897	TCGTCCTCGACATGTACACC	ACACAACGTCCTGGTCCTTC	59.8	288	91	thioredoxin putative expressed

ISM IDs	Rice gene locus IDs	Physical positions (bp) on chromosomes	Forward primers (5'-3')	Reverse primers (5'-3')	Melting tempearature (T˚C)	Expected product size (bp) in PCR	Expected product size (bp) in Q-PCR	Putative Functions
OsISM09941	LOC_Os01g68480	39794170	ACCAGGACGTTGTGTTCCTC	ACCTTTATGCCCAGCTCCTT	60.1	197	74	thioredoxin putative expressed
OsISM09942	LOC_Os01g68510	39809487	GAAGTTGTGGCAGGCAAGAG	CAATTGTCGCAACCTTTTCA	60.3	1582	95	peptide transporter PTR2 putative expressed
OsISM09943	LOC_Os01g68510	39811224	CTAGCCTATGGATGCTTCGC	GTGGCTTGAGAGCTGGAAGT	59.8	379	77	peptide transporter PTR2 putative expressed
OsISM09944	LOC_Os01g68510	39812072	GCTCTACAACCCTCCTTCCA	CAATTGCACCCTTGTTCAGA	59.5	888	84	peptide transporter PTR2 putative expressed
OsISM09945	LOC_Os01g68598	39844477	CTGCTCTTCAGTCATGCTCG	TTCAGGTAATGTTTGGTGAGGA	59.7	220	94	expressed protein
OsISM09946	LOC_Os01g68598	39844675	TCCTCACCAAACATTACCTGA A	TTCTCCTTGCATATTTGGGC	59.8	506	90	expressed protein
OsISM09947	LOC_Os01g68620	39851810	GACTCGGAGTTCGAGGACG	GTCCACCCAGTACGTCACCT	60.4	172	85	signal peptide peptidase-like 2B putative expressed
OsISM09948	LOC_Os01g68620	39852203	GCTGCTCCTCATCAACGAC	ACTGGGATACCATCACGACC	59.6	342	99	signal peptide peptidase-like 2B putative expressed
OsISM09949	LOC_Os01g68620	39852530	TGATGGTATCCCAGTCTGCC	ATCGAAACTCGGCTTCTCC	60.1	201	99	signal peptide peptidase-like 2B putative expressed
OsISM09950	LOC_Os01g68620	39852781	GTCCGTTTGGTCCTTCGTC	CCACCCAGTGTAGGAGCATT	60.2	255	58	signal peptide peptidase-like 2B putative expressed
OsISM09951	LOC_Os01g68620	39853157	CTGCTGGTTGTGCTGTTCTG	GTCGAGGCAACATAGTGCAA	60.3	174	57	signal peptide peptidase-like 2B putative expressed
OsISM09952	LOC_Os01g68620	39853307	GGGATTGCACTATGTTGCCT	AACCACTGTCACGTTCCCTAA	59.7	211	98	signal peptide peptidase-like 2B putative expressed
OsISM09953	LOC_Os01g68620	39853573	AGAATTCACCGTTTGCTTGG	ATTTGGCAGATGGACCACTT	59.8	169	84	signal peptide peptidase-like 2B putative expressed
OsISM09954	LOC_Os01g68620	39853680	TCCTTTTCATTCAGGGCATC	CATGAAAGCGCTCACAAGAA	60.1	194	99	signal peptide peptidase-like 2B putative expressed
OsISM09955	LOC_Os01g68620	39853886	TGGGTGTTCATATCACCTTTCA	GGAAGGCTAGGTCCCTCATC	60.1	163	81	signal peptide peptidase-like 2B putative expressed
OsISM09956	LOC_Os01g68620	39854101	ATCGGATTTGGGGACATTCT	GCCGTCAGTCAAGTCCTTTC	60.2	240	88	signal peptide peptidase-like 2B putative expressed
OsISM09957	LOC_Os01g68620	39854333	CTGACGGCTATTTCCTCTGC	AGGCCAACATATGTGCATGA	60.0	149	65	signal peptide peptidase-like 2B putative expressed
OsISM09958	LOC_Os01g68620	39854498	GGACAGCCAGCTCTCCTCTA	TCGCACCTAGCGTGACAATA	60.1	145	62	signal peptide peptidase-like 2B putative expressed

ISM IDs	Rice gene locus IDs	Physical positions (bp) on chromosomes	Forward primers (5'-3')	Reverse primers (5'-3')	Melting tempearature (T˚C)	Expected product size (bp) in PCR	Expected product size (bp) in Q-PCR	Putative Functions
OsISM09959	LOC_Os01g68630	39857617	CGGATTCATCCGACAATCTT	GAATCAAAAACCCAATGTCCA	59.8	231	74	leaf senescence related protein putative expressed
OsISM09960	LOC_Os01g68630	39858054	AGATGGAATCCCTCCTCTGC	AATCCACTTGCTCATCCTGC	60.4	1038	78	leaf senescence related protein putative expressed
OsISM09961	LOC_Os01g68640	39861306	GGGGAACCTGCTCTACGTCT	ACGGCGTAGGAGGTATTGTG	60.3	667	86	expressed protein
OsISM09962	LOC_Os01g68770	39947277	AGCTCCTCTACGTCACCTGC	CCAGGTAATCCGGCTTGTTA	59.8	753	58	selenium-binding protein putative expressed
OsISM09963	LOC_Os01g68770	39948137	AGTTCTTGCCATGGTGATCC	GTCAACGACATACACACGGC	60.0	187	82	selenium-binding protein putative expressed
OsISM09964	LOC_Os01g68770	39948488	CTTCCTCCTGCTGGATTCTG	ATAGCCAAACAAGGGGCTGT	60.4	404	74	selenium-binding protein putative expressed
OsISM09965	LOC_Os01g68770	39949032	TGGTGAGCTCAAGCAGACAC	GCCCACATACCCAGTATCCTT	60.1	187	98	selenium-binding protein putative expressed
OsISM09966	LOC_Os01g68770	39949252	AAGACTGCAGATGGATCATGG	AGAATCCAGTTGCGCACTTT	60.0	186	72	selenium-binding protein putative expressed
OsISM09967	LOC_Os01g68770	39949596	AAAAGGGCAGTGAGGTTGTG	CTGTGGCCCACCTCTAAGTC	59.9	390	96	selenium-binding protein putative expressed
OsISM09968	LOC_Os01g68790	39954265	CAATGCTGCTCGTCTGATGT	GAAGCTGGTGAATGCCTCTT	59.7	180	92	RNA recognition motif containing protein putative expressed
OsISM09969	LOC_Os01g68790	39954425	AAGAGGCATTCACCAGCTTC	CAAACCCTCTTGACCTTCCA	59.8	177	79	RNA recognition motif containing protein putative expressed
OsISM09971	LOC_Os01g68800	39957958	TTTGTTCTGACCGACGAGTG	TCCGACCCTTGTTCTTCTTC	59.6	294	92	expressed protein
OsISM09974	LOC_Os01g68960	40067067	CTCCTCCTCCTCCTCCCC	TGAAACCAACCCAAATTTCC	60.4	921	72	conserved hypothetical protein
OsISM09975	LOC_Os01g68960	40068292	ATGACGCCAATCCAGCTAAG	GAGAACTTAACCGGCTGCTG	60.1	189	71	conserved hypothetical protein
OsISM09976	LOC_Os01g68980	40073258	CGAGTTGGGCCCTATGATTA	CGGGCTCATACCAGTCAAAA	60.5	243	92	conserved hypothetical protein
OsISM09978	LOC_Os01g69000	40083527	GGAGCTAGCGGTTGATAGTGA	GCGGCTCTTTCATGTCTCTC	59.8	533	99	retrotransposon protein putative unclassified
OsISM09979	LOC_Os01g69010	40093451	GTCTGCTCCAGGCCGTATT	GTGCAGGGATCATCATTGGT	60.5	596	91	MATE efflux protein putative
OsISM09980	LOC_Os01g69010	40094126	CAAGGACACCAAGACACCCT	AACGGCAGGTGAATATCAGG	60.0	173	87	MATE efflux protein putative

ISM IDs	Rice gene locus IDs	Physical positions (bp) on chromosomes	Forward primers (5'-3')	Reverse primers (5'-3')	Melting tempearature (T˚C)	Expected product size (bp) in PCR	Expected product size (bp) in Q-PCR	Putative Functions
OsISM09981	LOC_Os01g69010	40094279	CCTGATATTCACCTGCCGTT	GCTTGCACAGCATTATCAGC	59.8	193	90	MATE efflux protein putative
OsISM09982	LOC_Os01g69010	40094486	GATGTCATCCCGTCCAGTCT	AGAACGTTACGGCCACCAC	60.4	400	92	MATE efflux protein putative
OsISM09983	LOC_Os01g69010	40094958	AAGTCTGGCTCGCCTCATC	CGCCACCTTGTAGTGATCC	59.8	195	99	MATE efflux protein putative
OsISM09984	LOC_Os01g69010	40095264	GTCATCAGCACCATCCACAG	GAGAGAAGAACCAAGCACGG	60.1	382	72	MATE efflux protein putative
OsISM09985	LOC_Os01g69010	40095691	CATGAGCGTCAGAGCATTTG	CATTTGCGAAGAAACTTCCAC	60.2	249	73	MATE efflux protein putative
OsISM09986	LOC_Os01g69040	40117205	TGTCCCATTATCGTGGATGA	AGGTTCTGTCCTTGGAGCCT	60.0	230	94	zinc finger C3HC4 type domain containing protein expressed
OsISM09987	LOC_Os01g69040	40117415	AGGCTCCAAGGACAGAACCT	TCCACATGCTCATCAACTGC	60.6	1913	89	zinc finger C3HC4 type domain containing protein expressed
OsISM09988	LOC_Os01g69040	40119393	GCACCAGCCCTGTGATACAT	CAGGGCAATTGAAAGTTGGT	60.5	178	94	zinc finger C3HC4 type domain containing protein expressed
OsISM09989	LOC_Os01g69050	40128287	ATCGTCACGCTTGATCACAA	GTCTCCGTCTCGCTTACGAC	60.1	177	78	lysine ketoglutarate reductase trans-splicing related 1 putative expressed
OsISM09990	LOC_Os01g69050	40128555	GTCCAGCGAGCGGAACTC	GGACTTGTCCTGCACGATG	61.2	882	97	lysine ketoglutarate reductase trans-splicing related 1 putative expressed
OsISM09991	LOC_Os01g69050	40129521	GTCGGCATAAAGCAGAAGGC	ACATCACCGTGAACCTGTCA	61.1	434	71	lysine ketoglutarate reductase trans-splicing related 1 putative expressed
OsISM09992	LOC_Os01g69050	40130218	CACCACCGCCTCACACTC	CCCTCCCCTCGTCTTGTAG	60.5	482	67	lysine ketoglutarate reductase trans-splicing related 1 putative expressed
OsISM09993	LOC_Os01g69060	40132161	CCAGAAGCTCCCCTGCTG	GGCGCATCGATCAAGTAGTA	60.5	149	67	hydrolase alpha/beta fold family domain containing protein expressed
OsISM09994	LOC_Os01g69060	40132290	TACTACTTGATCGATGCGCC	GCAAACCACTCATCGGACTT	59.5	233	96	hydrolase alpha/beta fold family domain containing protein expressed
OsISM09995	LOC_Os01g69060	40132510	ATGAGTGGTTTGCTCAAGGG	CCTTCCCTTTGTTCCCGTAT	60.1	301	61	hydrolase alpha/beta fold family domain containing protein expressed
OsISM09996	LOC_Os01g69060	40132835	CAATGGGAGCTGAATCACAA	ATTCGAAGAACTCCCAGCCT	59.9	233	72	hydrolase alpha/beta fold family domain containing protein expressed
OsISM09997	LOC_Os01g69060	40133068	CCTCCTGTTCTTTTTATTCATG G	AATCAAAAGCAATGGCATGAT	59.2	195	98	hydrolase alpha/beta fold family domain containing protein expressed
OsISM09998	LOC_Os01g69060	40133244	CATGCCATTGCTTTTGATTG	CGGGTTGAGGCTTATCTGAG	60.0	454	53	hydrolase alpha/beta fold family domain containing protein expressed

ISM IDs	Rice gene locus IDs	Physical positions (bp) on chromosomes	Forward primers (5'-3')	Reverse primers (5'-3')	Melting tempearature (T°C)	Expected product size (bp) in PCR	Expected product size (bp) in Q-PCR	Putative Functions
OsISM09999	LOC_Os01g69060	40133678	CTCAGATAAGCCTCAACCCG	ATCAGGAGCAACAGCATTGA	59.6	177	92	hydrolase alpha/beta fold family domain containing protein expressed
OsISM10000	LOC_Os01g69060	40133839	TGCTGTTGCTCCTGATAAGC	TCAGGTGGTTCAACTTGTCCT	59.4	181	99	hydrolase alpha/beta fold family domain containing protein expressed
OsISM10001	LOC_Os01g69060	40133999	AGGACAAGTTGAACCACCTGA	GAAGCTTCGCATGCTTATCC	59.8	198	61	hydrolase alpha/beta fold family domain containing protein expressed
OsISM10002	LOC_Os01g69060	40134216	CAGCAATTTCTTGTTGGGTG	TTTCATCATGTATGGGCCAC	59.2	183	89	hydrolase alpha/beta fold family domain containing protein expressed
OsISM10003	LOC_Os01g69060	40134455	TGCATTGAACGCAATCAGTAG	TTTGTTTTCCATGAATCACTCG	59.9	170	94	hydrolase alpha/beta fold family domain containing protein expressed
OsISM10004	LOC_Os01g69060	40134680	GATTTTTGTGGCAGTGCAAA	TAGCTCTTCACCACGGTCCT	59.8	310	82	hydrolase alpha/beta fold family domain containing protein expressed
OsISM10005	LOC_Os01g69060	40134972	GACCGTGGTGAAGAGCTAGG	TCTAGAGTCCGTCCCCACAT	59.7	1610	77	hydrolase alpha/beta fold family domain containing protein expressed
OsISM10006	LOC_Os01g69100	40167060	ATCTCAAGCGAAAGGAGCAG	CCGCGAGTCAAAGAACATTT	60.0	823	96	expressed protein
OsISM10007	LOC_Os01g69100	40167893	ATTCTGATTGCGAGGACGAC	GGGTTGGTAGTTTGGAGTGC	59.8	597	78	expressed protein
OsISM10008	LOC_Os01g69120	40174900	ATCATCACCCCCACCTTGT	CTTGTCCAGATCCACCTCGT	60.1	193	97	uncharacterized RNA methyltransferase pc1998 putative expressed
OsISM10009	LOC_Os01g69120	40175145	ATTTCACCTTCAGCAGAGGC	CAATCAACGGGTTCTCTGGT	59.7	704	85	uncharacterized RNA methyltransferase pc1998 putative expressed
OsISM10010	LOC_Os01g69120	40175829	ACCAGAGAACCCGTTGATTG	TAGCATTGATGCTTGGGTGA	60.1	299	93	uncharacterized RNA methyltransferase pc1998 putative expressed
OsISM10011	LOC_Os01g69120	40176096	TTCTTCAGCTCATCACCCAA	CATCCTCATCAAATGGCTCA	59.5	197	90	uncharacterized RNA methyltransferase pc1998 putative expressed
OsISM10012	LOC_Os01g69120	40176294	GAGTACCGGCGAACTGAGAT	GAGGAATCAGGCTAACCTTGC	59.8	273	97	uncharacterized RNA methyltransferase pc1998 putative expressed
OsISM10013	LOC_Os01g69120	40176489	TCTCTAGATGGCTGTGACAAC G	CCCTTGAATTCCAAACCAAC	59.9	193	96	uncharacterized RNA methyltransferase pc1998 putative expressed
OsISM10014	LOC_Os01g69120	40176668	TTGGAATTCAAGGGATGAGC	ACTTCTTGGTCCCCCATTTC	60.1	229	86	uncharacterized RNA methyltransferase pc1998 putative expressed
OsISM10015	LOC_Os01g69120	40176881	TGGGGGACCAAGAAGTAATG	GATGTCTCCATTTATGCCCAA	59.8	656	96	uncharacterized RNA methyltransferase pc1998 putative expressed
OsISM10016	LOC_Os01g69120	40177592	TCCTTATAGCTTTGGGCAGG	TGCAACGTATGCAGTAACGC	60.1	313	58	uncharacterized RNA methyltransferase pc1998 putative expressed

ISM IDs	Rice gene locus IDs	Physical positions (bp) on chromosomes	Forward primers (5'-3')	Reverse primers (5'-3')	Melting tempearature (T˚C)	Expected product size (bp) in PCR	Expected product size (bp) in Q-PCR	Putative Functions
OsISM10017	LOC_Os01g69120	40177963	GGATTATCTCTTGCTGCTTCG	AGTCGGCTTGCTGATTTTTC	59.3	865	96	uncharacterized RNA methyltransferase pc1998 putative expressed
OsISM10018	LOC_Os01g69120	40178854	CTTGGCACAACACTGATGCT	CAACACTTGACCCTTCGAGC	60.4	142	58	uncharacterized RNA methyltransferase pc1998 putative expressed
OsISM10019	LOC_Os01g69120	40179052	AAAGTTGCTTTGTCCGAACG	TCCATGGCCTCTTCTCATCT	60.0	219	77	uncharacterized RNA methyltransferase pc1998 putative expressed
OsISM10020	LOC_Os01g69120	40179353	ATACATTAGCTGCGGATGGG	AAATGCCAAGCCTTACTGGA	59.8	305	70	uncharacterized RNA methyltransferase pc1998 putative expressed
OsISM10021	LOC_Os01g69120	40179638	TCCAGTAAGGCTTGGCATTT	GCTTCCGATTCTCGTTTGAA	60.0	625	99	uncharacterized RNA methyltransferase pc1998 putative expressed
OsISM10022	LOC_Os01g69140	40192163	GGAGAAGGCTAACCTGACGA	CAGCTCCTTCTCCTCCTCCT	59.8	179	71	regulatory protein putative expressed
OsISM10023	LOC_Os01g69140	40192654	ATCCTCGAGCTCGGATACAA	GTCACGGAACCACAGGATGT	60.3	566	58	regulatory protein putative expressed
OsISM10024	LOC_Os01g69160	40200147	GGTACCAGCGGTAATTCCAA	GATGATCACCGTCCTGTCGT	60.4	1414	88	regulatory protein putative
OsISM10025	LOC_Os01g69160	40201792	CTCGTCTGGACCAAGCTCTC	GGGTCCCTGAACCACAAGAT	60.6	355	93	regulatory protein putative
OsISM10026	LOC_Os01g69174	40204291	GTCGATGGCTCAGCACCT	TGATGATCACCGTCTTGTCC	59.7	1088	98	conserved hypothetical protein
OsISM10027	LOC_Os01g69190	40210394	GACGCTGCTCCTCCTCCT	GATGATCACCGTCCTGTCCT	60.3	570	91	regulatory protein putative expressed
OsISM10028	LOC_Os01g69190	40211195	CTCGTCTGGACCAAGCTCTC	TCACGGAACAACACCATGTC	60.3	567	90	regulatory protein putative expressed
OsISM10029	LOC_Os01g69200	40214262	CGCAGAGCAGCAAGAACTAA	ATGATCACCGTCCTGTCCTC	59.7	190	99	regulatory protein putative expressed
OsISM10030	LOC_Os01g69200	40214681	TACCTCGACCTCGTCTGGAG	GAACCACGCCAGGTCAAC	60.2	208	94	regulatory protein putative expressed
OsISM10031	LOC_Os01g69210	40220238	CCCAGGATGATGGATCTGC	CCTTGCACGAATTCCAAGAT	60.8	555	97	mannosyl-oligosaccharide glucosidase putative expressed
OsISM10032	LOC_Os01g69210	40220750	GGGTACCTACAGGCCGAATG	GCACCAATCCACATTATCCC	60.8	434	82	mannosyl-oligosaccharide glucosidase putative expressed
OsISM10033	LOC_Os01g69210	40221370	TCGATTGAATGCGAAGACTG	TTGAGTTCCCTGCTTCATCC	60.1	329	96	mannosyl-oligosaccharide glucosidase putative expressed
OsISM10034	LOC_Os01g69210	40221734	CATGTTCTTTTGGCATCTGG	CCAGCTCTGTGAATTTCCAA	59.0	233	90	mannosyl-oligosaccharide glucosidase putative expressed

ISM IDs	Rice gene locus IDs	Physical positions (bp) on chromosomes	Forward primers (5'-3')	Reverse primers (5'-3')	Melting tempearature (T˚C)	Expected product size (bp) in PCR	Expected product size (bp) in Q-PCR	Putative Functions
OsISM10035	LOC_Os01g69210	40221997	TGACCTAGTACAGCAAGCCCT	TTCTGCCATGTCTGGAAGGT	59.8	188	71	mannosyl-oligosaccharide glucosidase putative expressed
OsISM10036	LOC_Os01g69210	40222144	CAATGCAAAGTGGGAACCTT	ACTTCGGCAGAACGTTTCAT	59.9	218	92	mannosyl-oligosaccharide glucosidase putative expressed
OsISM10037	LOC_Os01g69210	40222396	AACCCAATGATTGAAGAGCG	TTGATTCAAGACGAGTGCTCA	59.8	342	65	mannosyl-oligosaccharide glucosidase putative expressed
OsISM10038	LOC_Os01g69210	40222729	TTGAATCAAAACAGAAGGATTTTG	AAAGCAGCAACACCAACAGA	59.5	380	98	mannosyl-oligosaccharide glucosidase putative expressed
OsISM10039	LOC_Os01g69210	40223145	GCAGTCAAAAATTGCACTTCC	TAGTGCAGCTGGCCAATATG	59.8	270	77	mannosyl-oligosaccharide glucosidase putative expressed
OsISM10040	LOC_Os01g69210	40223463	GATGAGGGCTTCCATCAGTT	TCCATCCATCCGCATTTATT	59.6	227	98	mannosyl-oligosaccharide glucosidase putative expressed
OsISM10041	LOC_Os01g69210	40223695	CGGGAGCAAATATTAGGAGC	TGCAAGAAATAAGGTTGGGG	59.4	191	97	mannosyl-oligosaccharide glucosidase putative expressed
OsISM10042	LOC_Os01g69210	40223858	TGGAAATCCCCCAACCTTAT	TTGCATGTATTCCACTTGCC	60.0	356	57	mannosyl-oligosaccharide glucosidase putative expressed
OsISM10043	LOC_Os01g69210	40224285	TGGTTTCAGTGGTTCAATAGCA	ACCTTCGGATTCAACTCCCT	60.2	1138	99	mannosyl-oligosaccharide glucosidase putative expressed
OsISM10044	LOC_Os01g69210	40225374	ATTGGCATGGAAGAGACAGC	CATTAGGATGAGATGCCCGT	60.1	303	94	mannosyl-oligosaccharide glucosidase putative expressed
OsISM10045	LOC_Os01g69210	40225726	TTGTATGTGCTCAATTGCAGAG	CCCAAAATCTGAAAGTTGATTTG	59.4	175	89	mannosyl-oligosaccharide glucosidase putative expressed
OsISM10046	LOC_Os01g69210	40225878	CAAATCAACTTTCAGATTTTGGG	CGAAATAGGCACCGGTTTTA	59.9	532	67	mannosyl-oligosaccharide glucosidase putative expressed
OsISM10047	LOC_Os01g69210	40226394	ACCGGTGCCTATTTCGACTA	CGCAAAAGCTCTCGTCTCAT	60.1	187	96	mannosyl-oligosaccharide glucosidase putative expressed
OsISM10048	LOC_Os01g69210	40226629	TGTCAGCATGTTCCCTTTCA	GCTGCTTCTCAAGAACCCAT	59.8	158	63	mannosyl-oligosaccharide glucosidase putative expressed
OsISM10049	LOC_Os01g69210	40226827	CGGACTTCGGTCACTTTCTC	TATCCAAATGGCACCTCTCC	59.9	451	89	mannosyl-oligosaccharide glucosidase putative expressed
OsISM10050	LOC_Os01g69210	40227258	GGAGAGGTGCCATTTGGATA	AGTTCCTTTGCCCTGTCCTT	60.0	202	98	mannosyl-oligosaccharide glucosidase putative expressed
OsISM10051	LOC_Os01g69210	40227440	AAGGACAGGGCAAAGGAACT	CCCAGAAGAACCCAGTTTCA	60.1	182	89	mannosyl-oligosaccharide glucosidase putative expressed
OsISM10052	LOC_Os01g69220	40229806	ACATGACCGATCCAGAGACC	CCTGCAGATCGTTGCCAATA	61.0	838	92	exostosin family domain containing protein expressed

ISM IDs	Rice gene locus IDs	Physical positions (bp) on chromosomes	Forward primers (5'-3')	Reverse primers (5'-3')	Melting tempearature (T˚C)	Expected product size (bp) in PCR	Expected product size (bp) in Q-PCR	Putative Functions
OsISM10053	LOC_Os01g69220	40230850	CACTGCTTTTCTTTCGAGGG	TTGGCAAGTTTAGCACGGAT	60.3	221	65	exostosin family domain containing protein expressed
OsISM10054	LOC_Os01g69220	40231102	GAGGATAGCCTTGCAACAGG	GAGGAAGGAGTATCCCCAGC	59.9	253	96	exostosin family domain containing protein expressed
OsISM10055	LOC_Os01g69250	40239981	GAACAGAACCCGCACGAC	CTAGGCACTTGAGGCTCTCG	60.3	247	98	mitochondrial import receptor subunit TOM20 putative expressed
OsISM10056	LOC_Os01g69250	40240208	CGAGAGCCTCAAGTGCCTA	TTCAGTGCTTCCTCCAGCTT	59.7	1128	52	mitochondrial import receptor subunit TOM20 putative expressed
OsISM10057	LOC_Os01g69250	40241431	TCTTTGAAAAGGCAACCCAG	TCAGCACCTTGGATGACAAA	60.2	279	97	mitochondrial import receptor subunit TOM20 putative expressed
OsISM10058	LOC_Os01g69250	40241649	TTGTAGGAACCTGCGAATGA	GAGATGCCATTTGCCTATGAA	59.7	384	95	mitochondrial import receptor subunit TOM20 putative expressed
OsISM10059	LOC_Os01g69250	40242038	TCACAAGCTGCATCCTCAAC	CCCACCCGAAGACATCATAC	60.1	1114	86	mitochondrial import receptor subunit TOM20 putative expressed
OsISM10060	LOC_Os01g69800	40302794	CAAGATGTTGGAGGACCACA	GTTGTCTCGGGCAATTTGTT	59.7	185	73	transposon protein putative CACTA En/Spm sub-class
OsISM10062	LOC_Os01g69810	40306209	TATGCGAAGGGTCCTGTCA	GTCACGCCCAGAAATTTAGC	60.0	728	83	transposon protein putative CACTA En/Spm sub-class
OsISM10063	LOC_Os01g69810	40307275	GCCAGCCTATAGAGAGCGAA	CGCGTTGTTCCATATGCTT	59.7	222	88	transposon protein putative CACTA En/Spm sub-class
OsISM10064	LOC_Os01g69810	40308259	AGCACTTGGCTCTAGTTCGC	AATCATTACGCCTCCACCAG	59.9	397	87	transposon protein putative CACTA En/Spm sub-class
OsISM10065	LOC_Os01g69820	40312785	CCATGTGAGCTGCATATTCC	AGTACCTGAAGGGTCCGTTG	59.1	179	82	transposon protein putative CACTA En/Spm sub-class
OsISM10066	LOC_Os01g69820	40313034	ACCTCGAGCTGGATTACCCT	GATCCTGAGGAGGAGTCGGT	60.4	234	56	transposon protein putative CACTA En/Spm sub-class
OsISM10067	LOC_Os01g69820	40313985	CGCCCTCGACGTCTCTATTA	CAGTATCGAAAACCTTCCGC	60.0	189	78	transposon protein putative CACTA En/Spm sub-class
OsISM10068	LOC_Os01g69820	40314257	AAGGCGCAGCATTACAAGAC	GCCGACAGGTCGAAGAGTAA	60.4	179	81	transposon protein putative CACTA En/Spm sub-class
OsISM10069	LOC_Os01g69820	40314476	TCTACGTTTGACAAGGTTTTC G	GTCTTTCTCTCCATTTGCCG	59.6	169	83	transposon protein putative CACTA En/Spm sub-class
OsISM10070	LOC_Os01g69820	40314625	CGGCAAATGGAGAGAAAGAC	GGCAATACTCGCACACGTAA	59.8	195	99	transposon protein putative CACTA En/Spm sub-class
OsISM10071	LOC_Os01g69820	40314820	ACTGCCTTGCAGACCAAATC	CCGCGATAAATTCCTTGTGT	60.1	169	88	transposon protein putative CACTA En/Spm sub-class

ISM IDs	Rice gene locus IDs	Physical positions (bp) on chromosomes	Forward primers (5'-3')	Reverse primers (5'-3')	Melting tempearature (T°C)	Expected product size (bp) in PCR	Expected product size (bp) in Q-PCR	Putative Functions
OsISM10072	LOC_Os01g69830	40331311	AGCGCTTCTGCCAACAGT	TTCCTGCAGCTCCTCTTCTC	59.8	1256	71	OsSPL2 - SBP-box gene family member expressed
OsISM10073	LOC_Os01g69830	40332605	AGCCTGATCCTCTCAACCC	GAGAAGATCTGCGGGTACGA	59.8	902	80	OsSPL2 - SBP-box gene family member expressed
OsISM10074	LOC_Os01g69850	40343620	GTCGGCAAGCTCTACGAGT	TTCGTTCAGGTCTCTCCTGG	59.3	17658	91	OsMADS65 - MADS-box family gene with MIKC* type-box expressed
OsISM10075	LOC_Os01g69850	40361258	CCAGGAGAGACCTGAACGAA	TGTCCCTAAGCCTGGAGTGT	60.0	490	76	OsMADS65 - MADS-box family gene with MIKC* type-box expressed
OsISM10076	LOC_Os01g69850	40361728	ACACTCCAGGCTTAGGGACA	CGTCAGCATTGTTTTGGAGA	59.8	140	51	OsMADS65 - MADS-box family gene with MIKC* type-box expressed
OsISM10077	LOC_Os01g69850	40361904	TGCTGACAAATGCTTTGAGG	TTCACCATTTTGTTTTGCCA	60.0	936	60	OsMADS65 - MADS-box family gene with MIKC* type-box expressed
OsISM10078	LOC_Os01g69900	40384566	AGGTCCTCCGCTTCCATCT	ACCTCTTCACGGTCCTCTCC	60.9	532	65	CBS domain-containing protein putative expressed
OsISM10079	LOC_Os01g69900	40385181	GCTGTGTTGCTGACCGACT	CGCGAGCAATTACTCTTGTG	59.8	171	80	CBS domain-containing protein putative expressed
OsISM10080	LOC_Os01g69900	40385432	GAGGCATTGCAGAAGATGGT	ACTTCACCGTTCTCCACGAC	60.2	147	63	CBS domain-containing protein putative expressed
OsISM10081	LOC_Os01g69900	40385663	TGCTGCTGTTGAGGGTGTAG	AGTGATGGCCTAAACATCCG	60.0	282	97	CBS domain-containing protein putative expressed
OsISM10082	LOC_Os01g69900	40385925	CGGATGTTTAGGCCATCACT	CAGTTGGTGCAACAGTCACC	60.1	173	68	CBS domain-containing protein putative expressed
OsISM10083	LOC_Os01g69900	40386146	CAGCGGTTGTAGCAATTGAA	TGGGCTATAACACGCATCAA	60.0	149	74	CBS domain-containing protein putative expressed
OsISM10084	LOC_Os01g69900	40386275	TTGATGCGTGTTATAGCCCA	GCACATTCAGGAGTCTGGGT	60.1	168	78	CBS domain-containing protein putative expressed
OsISM10085	LOC_Os01g69900	40386480	ATGCACGACGGAAAGTTTTT	CAATTGCAGCATGGGTTATG	59.8	181	95	CBS domain-containing protein putative expressed
OsISM10086	LOC_Os01g69900	40386639	CACATAACCCATGCTGCAAT	CATCATAGCTGATGTCGCCT	59.1	265	73	CBS domain-containing protein putative expressed
OsISM10087	LOC_Os01g69900	40386902	TGCAGAGATTTTGGGATTCA	TGCCTCAGACGCCACTTTAG	60.3	182	93	CBS domain-containing protein putative expressed
OsISM10088	LOC_Os01g69900	40387152	AGACAAACAAGGCAGGATGC	GCCAAGCCTCTGTAGAATGC	60.1	213	83	CBS domain-containing protein putative expressed
OsISM10089	LOC_Os01g69900	40387352	ACAGAGGCTTGGCGATGAC	CAAGGTCACTGTCCGATGAA	60.5	228	99	CBS domain-containing protein putative expressed

ISM IDs	Rice gene locus IDs	Physical positions (bp) on chromosomes	Forward primers (5'-3')	Reverse primers (5'-3')	Melting tempearature (T°C)	Expected product size (bp) in PCR	Expected product size (bp) in Q-PCR	Putative Functions
OsISM10090	LOC_Os01g69900	40387572	TGACCTTGTAGCTGCTGTGG	ACACCGGCGTAATCCAAGT	60.2	162	76	CBS domain-containing protein putative expressed
OsISM10091	LOC_Os01g69910	40396433	TATTTGGATCCGTTCTTCCG	TTCCTGGTGTAGCCTGTGTG	59.8	655	89	calmodulin-binding transcription activator putative expressed
OsISM10092	LOC_Os01g69910	40397139	CATGATCGGTTCATCATCACA	AAGAAACCAAGCACCACCTG	60.2	169	61	calmodulin-binding transcription activator putative expressed
OsISM10093	LOC_Os01g69910	40397342	ATGGGTATGAATGGCGAAAA	AGCAATTCAGTGCATCAACA	59.0	391	91	calmodulin-binding transcription activator putative expressed
OsISM10094	LOC_Os01g69910	40397736	ATGCACACGCAGACAAAAAC	TGTGATCATAAGCCCTGCAA	60.0	735	70	calmodulin-binding transcription activator putative expressed
OsISM10095	LOC_Os01g69910	40398414	GCCAGGCTGAACTTACACCA	GAAATGCTGCCCTGATAAGC	60.5	443	98	calmodulin-binding transcription activator putative expressed
OsISM10096	LOC_Os01g69910	40399256	TCATCCTGAAGAAACCGGAC	TCCTCCTCCAACCAAGTGTC	60.1	186	76	calmodulin-binding transcription activator putative expressed
OsISM10097	LOC_Os01g69910	40399502	TATTCGTGAGGTTTCCCCAG	ACGAAGGAAGTCTCCTGCAA	60.0	305	74	calmodulin-binding transcription activator putative expressed
OsISM10098	LOC_Os01g69910	40400440	GCAAATGGATGGACTGCTTT	TACCGTCTCTTCCCTGTTGG	60.1	176	55	calmodulin-binding transcription activator putative expressed
OsISM10099	LOC_Os01g69910	40401056	AGCCGCGTTATCTATCCAGA	CTGACACGCGCCTATCATAA	59.8	206	97	calmodulin-binding transcription activator putative expressed
OsISM10100	LOC_Os01g69910	40401508	ACAGCAATACCGAAGGATGC	GACTGCCACTGCCATAAACA	59.9	315	99	calmodulin-binding transcription activator putative expressed
OsISM10101	LOC_Os01g69950	40453112	GTCAAGATTTACGGCGACCA	CCTTGCCCATTCCAACATTA	60.9	898	98	ribosomal protein L27 putative expressed
OsISM10102	LOC_Os01g69950	40454031	TCGATGGCCTCGTAAAGTTC	TCAGGTTGTTTCTCGTATGGG	60.1	164	77	ribosomal protein L27 putative expressed
OsISM10104	LOC_Os01g70000	40493088	CGTCGACAACCCTCTACTGG	TGAGAGCAGTAACCATGCCA	60.6	1211	99	expressed protein
OsISM10105	LOC_Os01g70020	40500413	AGACCGTCGAGCGCTACTC	GTCCTTCAGCTTCGATCCAG	60.3	349	93	DEK C terminal domain containing protein expressed
OsISM10106	LOC_Os01g70020	40500742	CTGGATCGAAGCTGAAGGAC	CAGGTTCTCGTCTGCCTTTC	60.0	1516	69	DEK C terminal domain containing protein expressed
OsISM10107	LOC_Os01g70020	40502244	CAGACGAGAACCTGCAAAGTC	AAACAAAACCAGAAAATTGGGA	59.9	177	97	DEK C terminal domain containing protein expressed
OsISM10108	LOC_Os01g70020	40502368	TCTGCAGGTCCATTTCTTGA	GCTTTTCCTTTATCCTGGTCC	59.2	195	96	DEK C terminal domain containing protein expressed

ISM IDs	Rice gene locus IDs	Physical positions (bp) on chromosomes	Forward primers (5'-3')	Reverse primers (5'-3')	Melting tempearature (T˚C)	Expected product size (bp) in PCR	Expected product size (bp) in Q-PCR	Putative Functions
OsISM10109	LOC_Os01g70020	40502619	GTTTCCAGAGCAGCCACAA	AGGGGACTCCAAAAATTCCA	60.3	148	64	DEK C terminal domain containing protein expressed
OsISM10110	LOC_Os01g70020	40502757	GGAGTCCCCTTGCATCACTA	TCTCCTTCCACGTTTCTTCC	59.7	141	68	DEK C terminal domain containing protein expressed
OsISM10111	LOC_Os01g70020	40502896	GATCTAAAGGAAATGGCCAGG	GCAGCTTCAGTTGATTGCTTT	59.8	1774	86	DEK C terminal domain containing protein expressed
OsISM10112	LOC_Os01g70020	40505108	TGCTGCCAAGACAACCAATA	CTCTGTGCTGCCTTTTCCTT	59.9	524	50	DEK C terminal domain containing protein expressed
OsISM10113	LOC_Os01g70020	40505645	CAACCACTGAAGAGCTGCAT	ATTGCCGAAGAATATCTGCC	59.1	166	88	DEK C terminal domain containing protein expressed
OsISM10114	LOC_Os01g70020	40505774	ATCTTGTAGGCGACATTGGC	TTTCCTATCCATGAGGTCCA	59.0	205	73	DEK C terminal domain containing protein expressed
OsISM10116	LOC_Os01g70025	40508447	CAAGAACGTCCTCGAGATGC	GAACATGCAGTGGTTGATGG	60.5	524	95	transferase family protein putative
OsISM10117	LOC_Os01g70090	40563188	AAGCAAGAATTGGATGCTGG	ACAGCCTTTGTTCCCATCAC	60.1	1519	84	enoyl-CoA hydratase/isomerase family protein putative expressed
OsISM10118	LOC_Os01g70110	40572073	CCTCCATCCATGGGACCT	AAGTACCTCTCGCCGTCTCC	60.7	141	46	No apical meristem protein putative expressed
OsISM10119	LOC_Os01g70120	40583690	CGACGAATGCGTCAGAATC	CGAAGCCATGGATTGATCTC	60.5	185	88	expressed protein
OsISM10120	LOC_Os01g70150	40600344	CAGCTGCTGGAGAAGCTAAA	CAACGATCTCTTGTTCGCAA	59.3	190	52	hypothetical protein
OsISM10122	LOC_Os01g70160	40607735	GCAAGGTGTATGCCTAAGGA	TGAGCTGGGTTGCTAGGTCT	58.9	597	94	zinc finger C3HC4 type domain containing protein expressed
OsISM10123	LOC_Os01g70160	40608639	TCCGCAAAATCGTATAACAGC	TCTTCGGATTGTCTGGAGTG	59.5	200	98	zinc finger C3HC4 type domain containing protein expressed
OsISM10124	LOC_Os01g70160	40608882	CGAATGGATGGAAAGAAGTGA	TCCACTGATACAACCAGCGA	60.2	226	96	zinc finger C3HC4 type domain containing protein expressed
OsISM10125	LOC_Os01g70210	40643731	AAGGGGAGTGGCTCAAGGT	CTCCTCAGCGTCAGGTTGTC	61.0	1487	89	CRAL/TRIO domain containing protein expressed
OsISM10126	LOC_Os01g70210	40645426	AAACGCGACATGCCTAAGTT	GATCGATCAGAGCATTTTCCA	60.0	904	91	CRAL/TRIO domain containing protein expressed
OsISM10127	LOC_Os01g70210	40646248	CTTCTGCACCGCAGGTTATT	ACGAACTTCTCCTGGCCTCT	60.3	227	76	CRAL/TRIO domain containing protein expressed
OsISM10128	LOC_Os01g70210	40646505	CTCCAACTGCGACATCAGAG	GGGTACATGGATCATCAGGG	59.8	227	95	CRAL/TRIO domain containing protein expressed

ISM IDs	Rice gene locus IDs	Physical positions (bp) on chromosomes	Forward primers (5'-3')	Reverse primers (5'-3')	Melting tempearature (T˚C)	Expected product size (bp) in PCR	Expected product size (bp) in Q-PCR	Putative Functions
OsISM10129	LOC_Os01g70210	40646735	ATGTTCATGAAGGCCTGGAA	TCTTGCAAGCTCTTGTCGTC	59.9	229	90	CRAL/TRIO domain containing protein expressed
OsISM10130	LOC_Os01g70220	40654137	GCATTCACCAGCCATTACCT	AACACCGCTTGTGCTCTTTT	59.9	664	60	histone-lysine N-methyltransferase putative expressed
OsISM10131	LOC_Os01g70220	40654795	GGTGTTACAGGAAGGCCAGA	CTCTGGATAAAGCACGGCAT	60.2	243	92	histone-lysine N-methyltransferase putative expressed
OsISM10132	LOC_Os01g70220	40655018	ATGCCGTGCTTTATCCAGAG	GAATGCCCAAAACAACCATC	60.2	250	97	histone-lysine N-methyltransferase putative expressed
OsISM10133	LOC_Os01g70220	40655276	TGGCTGAATGGCATTGATTA	TTGCCAGTGGGAAAGTTAGG	60.1	708	77	histone-lysine N-methyltransferase putative expressed
OsISM10134	LOC_Os01g70220	40656090	TGGCTCTCAACAACTGCAAC	ATGGGATTGCCATTATCCTT	59.1	537	64	histone-lysine N-methyltransferase putative expressed
OsISM10135	LOC_Os01g70220	40656670	AAGGTCTACACCTATGATGGG C	ACAACGTGCCCTTGTACTCC	59.7	1154	72	histone-lysine N-methyltransferase putative expressed
OsISM10136	LOC_Os01g70220	40657812	AGGGCACGTTGTTTTCAAAT	GGGAGCTTCAGCACGAGTAA	60.0	841	92	histone-lysine N-methyltransferase putative expressed
OsISM10137	LOC_Os01g70220	40658633	TTACTCGTGCTGAAGCTCCC	TAGGAAGATTCTCCTGCCCA	60.2	299	85	histone-lysine N-methyltransferase putative expressed
OsISM10138	LOC_Os01g70220	40658949	GTTGATGACCCACCTGTTCC	GAATCTTGATGCCCTTTGGA	60.1	436	74	histone-lysine N-methyltransferase putative expressed
OsISM10139	LOC_Os01g70220	40659450	CAGCGCAATGGTTCTGATTT	ACAATAGCTTTGGGCTCCAC	60.2	471	72	histone-lysine N-methyltransferase putative expressed
OsISM10140	LOC_Os01g70220	40659976	CAGAAAGGTCTGCAGTATCGC	TATCCCAAGTCCTGACACCC	59.9	2315	71	histone-lysine N-methyltransferase putative expressed
OsISM10141	LOC_Os01g70220	40662404	ATGAAGGGTCTGGATGGAAG	ACTCTGGTGCAGGTGGATCT	59.3	326	95	histone-lysine N-methyltransferase putative expressed
OsISM10142	LOC_Os01g70220	40662830	TGTTAAGCTGGCAAAGGTGA	CCCAACAACACTGTCCAAGA	59.5	186	98	histone-lysine N-methyltransferase putative expressed
OsISM10144	LOC_Os01g70260	40680124	TTATTCTGCCCCAGAGTGCT	CCAAGGATCATGCCAAAACT	59.9	1538	76	receptor-like protein kinase 2 precursor putative expressed
OsISM10146	LOC_Os01g70270	40695066	TCGTCTTCTACTTCCCGCAG	TTGGAGGGCAGATCGTAGAG	60.4	206	95	auxin response factor putative expressed
OsISM10147	LOC_Os01g70270	40695252	CTCTACGATCTGCCCTCCAA	TAGACCTCGTCGGTGTCTTG	59.6	231	78	auxin response factor putative expressed
OsISM10148	LOC_Os01g70270	40695439	TGTTGGGGAATTTTGTAGGC	GTCTTCTCCACCGCCATCT	60.0	218	99	auxin response factor putative expressed

ISM IDs	Rice gene locus IDs	Physical positions (bp) on chromosomes	Forward primers (5'-3')	Reverse primers (5'-3')	Melting tempearature (T˚C)	Expected product size (bp) in PCR	Expected product size (bp) in Q-PCR	Putative Functions
OsISM10149	LOC_Os01g70270	40695770	CTGATGAGTGCCTCCCTCC	CCATGCTGTGCAGATCCTT	60.3	196	79	auxin response factor putative expressed
OsISM10150	LOC_Os01g70270	40695950	GATCTGCACAGCATGGACTG	CCACTCTGAAGGAGATGCCT	59.9	327	72	auxin response factor putative expressed
OsISM10151	LOC_Os01g70270	40696312	CTGGGGATGCTTTCATTTTC	GGACAGCTGCCTCATAGCTC	59.8	154	78	auxin response factor putative expressed
OsISM10152	LOC_Os01g70270	40696505	CTGGGAGTCCTTGCTACTGC	TGGTATAATGAACTCTGAAGGGC	59.8	242	97	auxin response factor putative expressed
OsISM10153	LOC_Os01g70270	40696813	GAAGGAGAAGAGGCACCAGA	CAACTTGATTCAGGCCACAC	59.3	175	84	auxin response factor putative expressed
OsISM10154	LOC_Os01g70270	40696954	AGAATCTCGACCCAGTGTGG	AGTTGATGGTTCATCCCACC	59.9	150	69	auxin response factor putative expressed
OsISM10155	LOC_Os01g70270	40697208	TCCTCCAGCATCTCCTGAAT	TTGTGCTTGAGCAGGATCAG	59.9	169	77	auxin response factor putative expressed
OsISM10156	LOC_Os01g70270	40698225	TGCTTCGACGAGGAGTTGTA	AGATCTGCCAAGTGCAACG	59.8	471	56	auxin response factor putative expressed
OsISM10157	LOC_Os01g70270	40698826	TGATGCTTGTTGGAGACGAC	TGGACCTCCTCCTTGGTGTA	60.2	169	80	auxin response factor putative expressed
OsISM10158	LOC_Os01g70280	40703977	TTAGCTGCTTCCCGCTAAGA	TGGGAACGAATAGGGAGATG	60.1	128	55	hypothetical protein
OsISM10159	LOC_Os01g70330	40738290	ACGAGGTCTCCCACGACAT	ATTCGTGTGGATTAAACGGC	60.2	193	72	prefoldin subunit family protein expressed
OsISM10160	LOC_Os01g70330	40738463	GCCGTTTAATCCACACGAAT	CCACATAGTACCCCTCCCCT	59.9	155	55	prefoldin subunit family protein expressed
OsISM10161	LOC_Os01g70330	40738728	TTCTTCGAATCAACTGCCG	TGGTTACCCAATGTTGATGA	58.6	839	99	prefoldin subunit family protein expressed
OsISM10162	LOC_Os01g70330	40739490	TGGCAGGAAGGTCTTATTGAA	TGCCTGAAGCAGAACTTGAA	59.7	527	92	prefoldin subunit family protein expressed
OsISM10163	LOC_Os01g70330	40740238	GCCTCATTTTCTGGAAGTGG	TATTTCCCTTCCTCCTGGCT	59.9	198	88	prefoldin subunit family protein expressed
OsISM10164	LOC_Os01g70330	40740474	TCACTCGGTTTTTCCAGGAC	GAGGAAGCTGAATTCCAGGG	60.6	182	54	prefoldin subunit family protein expressed
OsISM10165	LOC_Os01g70330	40740635	ACCCTGGAATTCAGCTTCCT	GGCAATGGAAGAGACTTGGA	60.1	333	89	prefoldin subunit family protein expressed
OsISM10166	LOC_Os01g70330	40740948	TCCAAGTCTCTTCCATTGCC	TTCCGATCATGAGATGTAGGG	60.0	911	87	prefoldin subunit family protein expressed

ISM IDs	Rice gene locus IDs	Physical positions (bp) on chromosomes	Forward primers (5'-3')	Reverse primers (5'-3')	Melting tempearature (T°C)	Expected product size (bp) in PCR	Expected product size (bp) in Q-PCR	Putative Functions
OsISM10167	LOC_Os01g70330	40741838	CCCTACATCTCATGATCGGAA	TTGAAGGTTGAAGGGTTGAAA	59.7	179	99	prefoldin subunit family protein expressed
OsISM10168	LOC_Os01g70330	40742007	CAACCTTCAAAGAGTGCTGCT	GATATGGTCTCAACGCCCTC	59.6	187	97	prefoldin subunit family protein expressed
OsISM10169	LOC_Os01g70390	40779655	TCATGAAGCTCGCCTACCTC	GATACCAGGCCCTAATGGTG	59.9	499	94	expressed protein
OsISM10170	LOC_Os01g70390	40780134	CACCATTAGGGCCTGGTATC	TCTGCTGTTGCAGTTTTTGG	59.6	249	96	expressed protein
OsISM10171	LOC_Os01g70390	40780411	CGAGCAGAACTTCCTGACCT	TGAGCAAGCTCAACCTCATC	59.4	227	85	expressed protein
OsISM10172	LOC_Os01g70390	40780650	TCTGCTGAATCTTCCCATCA	AACTCCGGTAGGTTGTCGTG	59.7	189	97	expressed protein
OsISM10173	LOC_Os01g70390	40780818	TCACGACAACCTACCGGAGT	TAGTGCGATCCAGTCCAATG	60.1	327	69	expressed protein
OsISM10174	LOC_Os01g70390	40781174	ATGATCATAGCTCGTGCGTG	TTGGAATCTGTCTTTGGCCT	59.8	183	96	expressed protein
OsISM10175	LOC_Os01g70390	40781402	TGTTGAACGCATACGACTTGA	GCTCTTGAATTGTAACCTTGCC	60.2	175	72	expressed protein
OsISM10176	LOC_Os01g70390	40781543	CGCAACTTAATTGGCAAGGT	TTGATCTATCATTCTGGCCCTT	60.0	230	98	expressed protein
OsISM10177	LOC_Os01g70390	40781801	AATGGCAGATATGGAGCTGG	AGTCCATTGGTGTGAGGCTT	59.8	161	60	expressed protein
OsISM10178	LOC_Os01g70390	40781952	CCAATGGACTGGGTTCAGTT	CAGCTTTAGGCATTTCGAGC	60.0	159	80	expressed protein
OsISM10179	LOC_Os01g70390	40782122	GTAGTTGCCGTCCTTTCTGC	ATGTTGCCATGTTCTGTTGA	58.7	335	74	expressed protein
OsISM10180	LOC_Os01g70390	40782502	GTGGGAAAGGCACACTTCTC	GCCTTTCCATTTTCCATCAA	59.8	200	98	expressed protein
OsISM10181	LOC_Os01g70390	40782682	TTGATGGAAAATGGAAAGGC	GCAAGCCAAACTCTTCTTGG	59.9	171	77	expressed protein
OsISM10182	LOC_Os01g70390	40782860	TTTTGAGGTGATGGATGCTG	TGGCACGCTTCAGAGATAGA	59.7	280	96	expressed protein
OsISM10183	LOC_Os01g70400	40784358	GAACGAGGACCCCATTGATA	TGGTGACCATCATACACTCCA	59.8	246	79	expressed protein
OsISM10184	LOC_Os01g70400	40784563	TTGCAGGACGACTTCGAGTA	GAGACATCCTCCCAAAAGCC	60.3	217	87	expressed protein

ISM IDs	Rice gene locus IDs	Physical positions (bp) on chromosomes	Forward primers (5'-3')	Reverse primers (5'-3')	Melting tempearature (T°C)	Expected product size (bp) in PCR	Expected product size (bp) in Q-PCR	Putative Functions
OsISM10185	LOC_Os01g70400	40784767	GGGAGGATGTCTCTGAGTGG	CAGGAGGAAACAACCAGGAG	59.7	223	79	expressed protein
OsISM10186	LOC_Os01g70400	40784980	TTTCCTCCTGCAATCATCCT	CTCGATAACGCAGAACACGA	59.8	201	97	expressed protein
OsISM10188	LOC_Os01g70450	40803022	TCTCTGTTGCGCCAGTAGC	GATTTCGCGAGCTCTTTCAC	60.2	169	89	hypothetical protein
OsISM10189	LOC_Os01g70450	40803311	TGGTTGTTTGCATGTGTGTG	GTGCTGCGAAGGTTGAAAAT	60.2	218	75	hypothetical protein
OsISM10190	LOC_Os01g70470	40811954	TCCTCTCCTCCAACAACACC	CCTCCTCCTCCTCCTCCTTA	59.9	274	61	expressed protein
OsISM10191	LOC_Os01g70490	40824808	AGATTCCAGGTGGCCAAAG	CCATAAACCACGCCTATGCT	60.0	340	86	potassium transporter putative expressed
OsISM10192	LOC_Os01g70490	40825247	CACTGTGGTTCTCCTCCCAT	TCAAGGAATAAAGTGCAAATGTTC	59.8	393	93	potassium transporter putative expressed
OsISM10193	LOC_Os01g70490	40825831	GGTGACGGCGTACTAACTCC	TTTGCTGATTCCTTGATCCC	59.8	259	66	potassium transporter putative expressed
OsISM10194	LOC_Os01g70490	40826070	GGGATCAAGGAATCAGCAAA	ACGCTGAACAAGGAAGAGGA	60.0	195	88	potassium transporter putative expressed
OsISM10195	LOC_Os01g70490	40826414	AAGAAATGGGAAGCAAGGCT	GTGACCAAGGTCAGCAAACA	60.0	210	86	potassium transporter putative expressed
OsISM10196	LOC_Os01g70490	40826608	TGCTGACCTTGGTCACTTCA	GCTTGTCCAATGTATGCCAG	59.8	280	91	potassium transporter putative expressed
OsISM10197	LOC_Os01g70490	40826897	TCGCATCTATCCAGAACACG	TAGCCTGGCTTGCAATTATG	59.4	206	78	potassium transporter putative expressed
OsISM10198	LOC_Os01g70490	40827264	CAAACTACAGACAAGATCGGCA	AAACATCACAAAGACAACAGCAA	60.0	640	55	potassium transporter putative expressed
OsISM10199	LOC_Os01g70520	40841345	GGAGGATTTCGTCTTCGGAT	GGCTCCTACCATCCTCATCA	60.2	221	72	Os1bglu5 - beta-glucosidase homologue similar to G. max isohydroxyurate hydrolase expressed
OsISM10200	LOC_Os01g70520	40841546	TGATGAGGATGGTAGGAGCC	GACATCAGCTGTGCTTCTGC	59.9	417	80	Os1bglu5 - beta-glucosidase homologue similar to G. max isohydroxyurate hydrolase expressed
OsISM10201	LOC_Os01g70520	40841922	TGATAACAGGGAAAATGCCG	GGTGTCAACCATCAGCTTCA	60.3	305	93	Os1bglu5 - beta-glucosidase homologue similar to G. max isohydroxyurate hydrolase expressed
OsISM10202	LOC_Os01g70520	40842207	TGAAGCTGATGGTTGACACC	TTTGGATTGACATCACCCCT	59.9	191	92	Os1bglu5 - beta-glucosidase homologue similar to G. max isohydroxyurate hydrolase expressed
OsISM10203	LOC_Os01g70520	40842383	TGATGTCAATCCAAAAGGGC	AGGTCCAACTGATAAAGCATGA	59.8	1893	97	Os1bglu5 - beta-glucosidase homologue similar to G. max isohydroxyurate hydrolase expressed

ISM IDs	Rice gene locus IDs	Physical positions (bp) on chromosomes	Forward primers (5'-3')	Reverse primers (5'-3')	Melting tempearature (T˚C)	Expected product size (bp) in PCR	Expected product size (bp) in Q-PCR	Putative Functions
OsISM10204	LOC_Os01g70520	40844301	GATGGATGGTTGAGTCCGAG	GCAACCCTGTCTCCAAACTC	60.1	593	81	Os1bglu5 - beta-glucosidase homologue similar to G. max isohydroxyurate hydrolase expressed
OsISM10205	LOC_Os01g70520	40845046	TGTTACTGGCACATGCATCA	TGCCGATAATTCCTTTTCCT	59.2	160	73	Os1bglu5 - beta-glucosidase homologue similar to G. max isohydroxyurate hydrolase expressed
OsISM10206	LOC_Os01g70520	40845260	AAGCAACAAAAAGATGTCAGGA	GCGAGAACCGACAATGTTCT	59.6	188	99	Os1bglu5 - beta-glucosidase homologue similar to G. max isohydroxyurate hydrolase expressed
OsISM10207	LOC_Os01g70520	40845537	TGATCGTCCTTTGGGTAAGG	AGGTGGTGGATCTGTCTTGG	59.9	531	83	Os1bglu5 - beta-glucosidase homologue similar to G. max isohydroxyurate hydrolase expressed
OsISM10208	LOC_Os01g70520	40846048	CCAAGACAGATCCACCACCT	CAATCCTTGTGGGTCAGGTC	60.2	282	63	Os1bglu5 - beta-glucosidase homologue similar to G. max isohydroxyurate hydrolase expressed
OsISM10209	LOC_Os01g70520	40846324	GGATTGAGATTGATGGTGCAG	CATGGACAGTGTCATTGCTTG	60.3	214	98	Os1bglu5 - beta-glucosidase homologue similar to G. max isohydroxyurate hydrolase expressed
OsISM10210	LOC_Os01g70520	40846533	CCATGACAATGACAGGGTTG	AAGTAGCCTTTCACGTTGGC	59.6	202	88	Os1bglu5 - beta-glucosidase homologue similar to G. max isohydroxyurate hydrolase expressed
OsISM10213	LOC_Os01g70540	40851845	GCCGCCTCCTACATCACC	CCGGTAGTCGACGAGATCAT	60.9	844	79	retrotransposon protein putative unclassified expressed
OsISM10214	LOC_Os01g70540	40852846	CCGAGAATTCGGTGATATCCT	ACGTCGAGCAAGGTCTCAAT	60.1	195	86	retrotransposon protein putative unclassified expressed
OsISM10215	LOC_Os01g70540	40854841	GGACGTGGTGTTTGATGAAG	GTCCCGATGGTGGTCGTC	60.7	114	64	retrotransposon protein putative unclassified expressed
OsISM10216	LOC_Os01g70540	40855985	GAGAAGGCAGGACTCAAGGA	GACGAGATAGCGCAGACTCC	59.8	138	74	retrotransposon protein putative unclassified expressed
OsISM10217	LOC_Os01g70540	40856184	GGGAAGACCATCTTGCTGTC	AGTAACCAACCAGCATTGGC	59.8	117	78	retrotransposon protein putative unclassified expressed
OsISM10218	LOC_Os01g70600	40876102	CAAATTAGAAGCACACGGTTG	TTTTCTCCCTAGCTTTCGGA	58.7	159	66	DUF567 domain containing protein putative expressed
OsISM10219	LOC_Os01g70600	40876203	GTGTGGACGTGTGCTTTGAC	CATTTCCCAAAAAGCAGGAA	60.1	667	88	DUF567 domain containing protein putative expressed
OsISM10220	LOC_Os01g70600	40876956	CATTAGTAGTATGGACAAGATGCTCA	TGGTGACTGCAATAATGCTCA	59.6	2070	96	DUF567 domain containing protein putative expressed
OsISM10221	LOC_Os01g70600	40878940	TGTGAACATTGCATGCAGAATA	TGGATCCTAGGAGGAGCAAT	59.4	267	91	DUF567 domain containing protein putative expressed
OsISM10222	LOC_Os01g70600	40879391	GAGGTGTTCGTCATGGACAG	AGCCTTGCCATGTCTTGAAC	59.7	389	83	DUF567 domain containing protein putative expressed
OsISM10223	LOC_Os01g70630	40883762	TGCTCCTGCTATGTACGACG	ATCTGTGTAAACCCTGCCCA	60.2	153	73	retrotransposon protein putative unclassified expressed

ISM IDs	Rice gene locus IDs	Physical positions (bp) on chromosomes	Forward primers (5'-3')	Reverse primers (5'-3')	Melting tempearature (T˚C)	Expected product size (bp) in PCR	Expected product size (bp) in Q-PCR	Putative Functions
OsISM10224	LOC_Os01g70630	40883895	TGGGCAGGGTTTACACAGAT	TGCACGTGCTCTGGATACTC	60.2	1648	83	retrotransposon protein putative unclassified expressed
OsISM10225	LOC_Os01g70630	40885577	GGAAATATGCATGAGGAGCG	TGATCCTTGTGAGAGCATGAA	60.0	271	82	retrotransposon protein putative unclassified expressed
OsISM10226	LOC_Os01g70630	40885837	CACAAGGATCATGTTGAGGC	GTGCAAATTGAGGCCTTCTC	59.5	519	68	retrotransposon protein putative unclassified expressed
OsISM10227	LOC_Os01g70630	40886751	TTTCCTTATGGCGAACGAGT	TTCGTAAGCCTGATCGAGAA	59.2	303	92	retrotransposon protein putative unclassified expressed
OsISM10228	LOC_Os01g70630	40887112	TGAAATCCTGGATGCTACGTT	GGAACAGACACACGTTAACAAA A	59.3	310	98	retrotransposon protein putative unclassified expressed
OsISM10229	LOC_Os01g70630	40887345	TGCAGTTGCCATTGTGGTAT	TGTCATCCCCTTCAAAAAGC	60.0	220	85	retrotransposon protein putative unclassified expressed
OsISM10230	LOC_Os01g70630	40887611	TTGCAAGGATGCTACCAAAA	ACCTGGAGAAGTAGGGGGAA	59.6	188	97	retrotransposon protein putative unclassified expressed
OsISM10231	LOC_Os01g70630	40888002	TTGTGGACAAGAATGAGTCCT TT	AAGCTGCTAGCATCCCTTGA	60.1	183	98	retrotransposon protein putative unclassified expressed
OsISM10232	LOC_Os01g70630	40888167	AAGGGATGCTAGCAGCTTGA	TCAAATGCCAATGCTTGTTC	59.9	269	97	retrotransposon protein putative unclassified expressed
OsISM10233	LOC_Os01g70630	40888480	TTCTGTTTGTTGGGACCCTT	TGTACCACTTGCATACAGAGCC	59.8	151	79	retrotransposon protein putative unclassified expressed
OsISM10234	LOC_Os01g70630	40888606	AGAGGCTCTGTATGCAAGTGG	TGAAGCAGTTTGATTGGCTG	59.7	190	99	retrotransposon protein putative unclassified expressed
OsISM10235	LOC_Os01g70630	40888850	ACGCTTGGACATGAATCCTC	TTTCCGAATAGTCACGTTTGC	60.1	158	65	retrotransposon protein putative unclassified expressed
OsISM10237	LOC_Os01g70670	40914655	CTCCATCGCCTCATCATCTC	TATGCAACACCACTGTGGGA	60.9	885	92	BTBM1 - Bric-a-Brac Tramtrack Broad Complex BTB domain with Meprin and TRAF Homology MATH domain expressed
OsISM10238	LOC_Os01g70670	40915642	CAGCTGCATCATTTTTGGAT	TTGCAAAAAGTTTGATGTCCA	59.0	166	87	BTBM1 - Bric-a-Brac Tramtrack Broad Complex BTB domain with Meprin and TRAF Homology MATH domain expressed
OsISM10239	LOC_Os01g70670	40915776	TTTCTGAGCTTTGGACATCAA	TGACACGTTCCCCATGACTA	59.2	231	82	BTBM1 - Bric-a-Brac Tramtrack Broad Complex BTB domain with Meprin and TRAF Homology MATH domain expressed
OsISM10240	LOC_Os01g70670	40915996	GGAACGTGTCAGAAATGCCT	CGCATGCAAAGTTTGTGAAG	60.3	637	98	BTBM1 - Bric-a-Brac Tramtrack Broad Complex BTB domain with Meprin and TRAF Homology MATH domain expressed
OsISM10241	LOC_Os01g70670	40916627	CATGCGCTCCTTACATCTGA	GACAGAGTGACCTTTGCAAGC	60.0	178	93	BTBM1 - Bric-a-Brac Tramtrack Broad Complex BTB domain with Meprin and TRAF Homology MATH domain expressed
OsISM10242	LOC_Os01g70670	40916994	TCCAGATATGGTAATCCCGC	TACCTTCCTCCTGGAAGCCT	60.0	188	74	BTBM1 - Bric-a-Brac Tramtrack Broad Complex BTB domain with Meprin and TRAF Homology MATH domain expressed

ISM IDs	Rice gene locus IDs	Physical positions (bp) on chromosomes	Forward primers (5'-3')	Reverse primers (5'-3')	Melting tempearature (T°C)	Expected product size (bp) in PCR	Expected product size (bp) in Q-PCR	Putative Functions
OsISM10243	LOC_Os01g70670	40917521	TGCAAAGCTGTAAATGATGGTC	AAGGCAACAATTTGTGCAAG	59.5	176	91	BTBM1 - Bric-a-Brac Tramtrack Broad Complex BTB domain with Meprin and TRAF Homology MATH domain expressed
OsISM10244	LOC_Os01g70670	40918250	CGTTGGACATGAAAACACCA	GAGCCTGTGCACATCTTCAA	60.2	413	75	BTBM1 - Bric-a-Brac Tramtrack Broad Complex BTB domain with Meprin and TRAF Homology MATH domain expressed
OsISM10245	LOC_Os01g70670	40918654	CACAGGCTCGCAGATAGTCA	AGGATCCTCGTCGTTGAATG	60.1	180	97	BTBM1 - Bric-a-Brac Tramtrack Broad Complex BTB domain with Meprin and TRAF Homology MATH domain expressed
OsISM10246	LOC_Os01g70670	40918797	GCAGGTAAGTGTCCAGGCAT	TCAATGGGTCTAAAGGCTCAG	59.7	182	99	BTBM1 - Bric-a-Brac Tramtrack Broad Complex BTB domain with Meprin and TRAF Homology MATH domain expressed
OsISM10247	LOC_Os01g70670	40918932	AGGACTCTTTCTGCACCGAC	ACCTTTTCCCGGTGGTCTAC	59.8	148	82	BTBM1 - Bric-a-Brac Tramtrack Broad Complex BTB domain with Meprin and TRAF Homology MATH domain expressed
OsISM10248	LOC_Os01g70670	40919060	GTAGACCACCGGGAAAAGGT	CTGCTTTAGGCTTCCGAAAA	59.9	179	85	BTBM1 - Bric-a-Brac Tramtrack Broad Complex BTB domain with Meprin and TRAF Homology MATH domain expressed
OsISM10249	LOC_Os01g70680	40920865	ATGCTTGCCATTGCCTTACT	GCGGTACATCTTGCTCCTTG	60.3	192	91	DEF1 - Defensin and Defensin-like DEFL family
OsISM10250	LOC_Os01g70700	40925166	ATTCACGTTGCAGCCCAG	GGTCTTCTCCCCACCCTTC	60.8	333	88	conserved hypothetical protein
OsISM10252	LOC_Os01g70700	40926049	CTGTTCCATGGCAGAACTCC	TCGCCTCACAGCAACTAGAA	60.2	2124	72	conserved hypothetical protein
OsISM10254	LOC_Os01g70750	40951828	GACCTCCACCAATCCAACC	TCATGCAACAGACTGGGAAC	59.9	176	85	retrotransposon protein putative Ty3-gypsy subclass
OsISM10255	LOC_Os01g70750	40952975	ATGCCGACTCAATGGATTTC	ATCTGTCAAATGCCCTGCTC	60.1	363	88	retrotransposon protein putative Ty3-gypsy subclass
OsISM10256	LOC_Os01g70750	40953350	GGAAGCTGCATCTGTTCCAT	GCTCTGGTGAGGAGGAGAAG	59.7	182	99	retrotransposon protein putative Ty3-gypsy subclass
OsISM10257	LOC_Os01g70750	40953901	TCTGCTTCAGCTCAATTTGC	TCAACATGATGACTGCCTGC	60.1	306	72	retrotransposon protein putative Ty3-gypsy subclass
OsISM10258	LOC_Os01g70750	40954210	GGAGGAACAATTAGCCACACC	TCAAAGGAGAACAGATCGGC	60.5	135	55	retrotransposon protein putative Ty3-gypsy subclass
OsISM10259	LOC_Os01g70800	40977590	GACGTCATCAAAATCCGCTT	AAGTACACCCCACGAGGTTG	60.0	930	58	mitochondrial carrier protein putative expressed
OsISM10260	LOC_Os01g70800	40978553	GGGCTTTTGCAAGCAAGTAA	AGGGCTGGAACATTTCCTCT	60.2	753	75	mitochondrial carrier protein putative expressed
OsISM10261	LOC_Os01g70800	40979364	TTTGCTTCTGGTTCATCCAA	CCTACTGTTGCTGCACATCC	59.3	280	96	mitochondrial carrier protein putative expressed
OsISM10262	LOC_Os01g70800	40979673	TTCTTGCATCACAGGGTGAA	AAACCGCGGGTTTTCATTAT	60.4	587	77	mitochondrial carrier protein putative expressed

ISM IDs	Rice gene locus IDs	Physical positions (bp) on chromosomes	Forward primers (5'-3')	Reverse primers (5'-3')	Melting tempearature (T°C)	Expected product size (bp) in PCR	Expected product size (bp) in Q-PCR	Putative Functions
OsISM10263	LOC_Os01g70800	40980312	GCTGGCTTACAGTTTGGTTCA	GAATCATCCTCACTTCCTGAGTT	59.5	482	99	mitochondrial carrier protein putative expressed
OsISM10264	LOC_Os01g70800	40980846	AAAGCTGCTTGTCACCCACT	CGCCATATCTTGGATGTCGT	60.4	589	77	mitochondrial carrier protein putative expressed
OsISM10265	LOC_Os01g70800	40981531	CATCTTTGGTGAAATCGGCT	ACTCGACTCCAGCTTTGCTC	59.9	539	91	mitochondrial carrier protein putative expressed
OsISM10266	LOC_Os01g70810	40983697	CAGCTCCAGCTCCTCGTC	TCTTGATAGACAATGCCCCA	59.4	1100	89	homeobox domain containing protein expressed
OsISM10267	LOC_Os01g70810	40984750	TTGCACGTTTTTGTTCTTGC	ATGGACTTGACAGCCTTTGG	60.0	339	94	homeobox domain containing protein expressed
OsISM10268	LOC_Os01g70810	40985146	CGCATCTTTTGGGGTTACTG	CGTTGACTTGCAAAGAACTCC	60.2	419	52	homeobox domain containing protein expressed
OsISM10269	LOC_Os01g70810	40985973	TGGACAGGTCAAATTGATGG	GATACAATGGCCAAACCACC	59.7	174	91	homeobox domain containing protein expressed
OsISM10270	LOC_Os01g70810	40986157	GGCCAAGCAGTTACTGAGGA	TGAGCTGGCAGAGCTTTATG	59.9	168	90	homeobox domain containing protein expressed
OsISM10271	LOC_Os01g70810	40986305	CATAAAGCTCTGCCAGCTCA	GTTCCTCGCCTTGCTTGATA	59.8	658	94	homeobox domain containing protein expressed
OsISM10272	LOC_Os01g70810	40986998	TCAGGCATTGAAGAAATCTCA G	TTTCCAAGACTCATCACGGA	59.3	184	77	homeobox domain containing protein expressed
OsISM10273	LOC_Os01g70810	40987160	TCTCCGTGATGAGTCTTGGA	CACCTGCTCTCACTTGCATC	59.5	198	82	homeobox domain containing protein expressed
OsISM10274	LOC_Os01g70810	40987314	GAGGACATCCTTGCCTTGAC	CAGAAGCAGTGAGCAGCAAT	59.5	209	83	homeobox domain containing protein expressed
OsISM10275	LOC_Os01g70810	40987519	TCTGCGGACGAATCAAATAA	CAGCTTTCCGATTTGGATGT	59.7	623	92	homeobox domain containing protein expressed
OsISM10276	LOC_Os01g70810	40988458	GAAGCTGAACGTAACAGCCA	CCAAGCAGGGTCTATCCAAA	59.6	1368	98	homeobox domain containing protein expressed
OsISM10277	LOC_Os01g70810	40990343	CTGAAGCTATCCCTGCTTCG	TGGGAACTCTGAACTCCAGC	60.2	165	81	homeobox domain containing protein expressed
OsISM10278	LOC_Os01g70820	40993544	ATGCCTCGACTCCGACAAT	TTCTGCTCTCTTCGCTCCTC	60.3	1818	92	lumenal PsbP putative expressed
OsISM10279	LOC_Os01g70820	40995467	GGGGAGCTCAATGTCAGTGT	CCACCTCCCCAACATCTTTA	60.0	345	89	lumenal PsbP putative expressed
OsISM10280	LOC_Os01g70900	41032383	CTAACCCGTGGAACATCGAC	TTTTCTTTCTTCTTCCGAGGC	60.2	628	86	retrotransposon protein putative unclassified

ISM IDs	Rice gene locus IDs	Physical positions (bp) on chromosomes	Forward primers (5'-3')	Reverse primers (5'-3')	Melting tempearature (T˚C)	Expected product size (bp) in PCR	Expected product size (bp) in Q-PCR	Putative Functions
OsISM10281	LOC_Os01g70920	41052527	GTGGAAGGCCAACTGTGAAT	ACTGGTCTTTCTGCACAGCC	60.2	174	73	cullin-1 putative expressed
OsISM10282	LOC_Os01g70920	41052681	GGCTGTGCAGAAAGACCAGT	GCATCAGATGGGGTAAAACC	59.9	676	96	cullin-1 putative expressed
OsISM10283	LOC_Os01g70920	41053480	TACCTTCGCTTGAAGACGCT	TTGAAGTAGGAAAAGACCTGGC	60.0	170	59	cullin-1 putative expressed
OsISM10284	LOC_Os01g70920	41053619	TGTCCTTCTGCCAGGTCTTT	ATTGCATCCATCACGCTCTT	60.2	164	87	cullin-1 putative expressed
OsISM10285	LOC_Os01g70920	41053903	TGGATTGTGCAATACCCTTTG	CTGCACAGTTTCCTGGACCT	60.5	143	55	cullin-1 putative expressed
OsISM10286	LOC_Os01g70920	41054039	TGTGCAGAAGGAAATGAAGAGA	TCAATGGAGCACTAACAGCCT	59.9	637	93	cullin-1 putative expressed
OsISM10287	LOC_Os01g70920	41054697	CGAAAGAGAAACATATTGGTGG	CAATTGTCAAGCGACTGCAT	59.2	178	94	cullin-1 putative expressed
OsISM10289	LOC_Os01g70940	41061381	TTCACACTTATTCCTTTGCTGAA	TGCCTGCAAAGCAGTGAATA	59.7	318	99	potassium transporter putative expressed
OsISM10290	LOC_Os01g70940	41061848	TCATAGTTCTGTGCGGTGCT	AGCTCGAACTTTCAATCCAGA	59.3	204	96	potassium transporter putative expressed
OsISM10291	LOC_Os01g70940	41062001	GTTTTTCCCCCTCCAGTTCT	CCAACACAATGCATGAAAGG	59.7	674	98	potassium transporter putative expressed
OsISM10292	LOC_Os01g70940	41062849	CGACAGGTAAAGATGGTTGGA	ACCAAGGTCGGCAAACATAG	60.0	200	81	potassium transporter putative expressed
OsISM10293	LOC_Os01g70940	41063045	TGGTCACTTCACGAGTGCAT	TTTTTGACAGAAATGCCGC	60.1	579	99	potassium transporter putative expressed
OsISM10294	LOC_Os01g70940	41063625	TATCCTTGACATGCCCACAG	CTTGGCTACCAACAACAGCA	59.7	183	99	potassium transporter putative expressed
OsISM10295	LOC_Os01g70940	41063929	TTGGATCCTTATGGTGCTCTG	CATAACAGTCATGCACGCAA	59.7	223	95	potassium transporter putative expressed
OsISM10296	LOC_Os01g70960	41074050	ATTGTAAATGGCTGCTGGGA	GGATGAAGAGAGCGGAGATG	60.2	1241	83	cytochrome c1-1 heme protein mitochondrial precursor putative expressed
OsISM10297	LOC_Os01g70960	41075413	GTTGGAAGCCCCACACTATC	GATGGCCACGCCTTATTCT	59.7	399	78	cytochrome c1-1 heme protein mitochondrial precursor putative expressed
OsISM10298	LOC_Os01g70960	41076010	ATGAGCAAGCAGCTCGATTT	TAGTTCTGGCCATTGTGCCT	60.4	177	86	cytochrome c1-1 heme protein mitochondrial precursor putative expressed
OsISM10299	LOC_Os01g70960	41076168	GGCACAATGGCCAGAACTAT	TTGTAATGCAAGCCTTCACG	59.9	1279	89	cytochrome c1-1 heme protein mitochondrial precursor putative expressed

ISM IDs	Rice gene locus IDs	Physical positions (bp) on chromosomes	Forward primers (5'-3')	Reverse primers (5'-3')	Melting tempearature (T˚C)	Expected product size (bp) in PCR	Expected product size (bp) in Q-PCR	Putative Functions
OsISM10300	LOC_Os01g70960	41077510	TGAAGATGGCACTCCAGCTA	CCCATGACAAGAACGAGACA	59.6	161	63	cytochrome c1-1 heme protein mitochondrial precursor putative expressed
OsISM10301	LOC_Os01g70960	41077651	TGTCTCGTTCTTGTCATGGG	CAGAAGTGCGAGGGAGAGTAG	59.5	178	92	cytochrome c1-1 heme protein mitochondrial precursor putative expressed
OsISM10302	LOC_Os01g71000	41095253	GGGGCTGACTCCAAGGAC	TCCACCATGTGATCTCGATT	59.8	1095	88	protein kinase APK1B chloroplast precursor putative expressed
OsISM10303	LOC_Os01g71000	41096467	ATTGGGAGAGGGAGGTTTTG	TTGCGATCAAGTTGCTTCAC	60.1	237	82	protein kinase APK1B chloroplast precursor putative expressed
OsISM10304	LOC_Os01g71000	41096838	TGGGATCACTGGAAGACCAT	TCCGTGTGTTCCAGTCAAGA	60.3	271	67	protein kinase APK1B chloroplast precursor putative expressed
OsISM10305	LOC_Os01g71000	41097392	ACTGATTACAGGGCGCAAAG	TTCCTGCGGTCCTTGAATAG	60.2	625	94	protein kinase APK1B chloroplast precursor putative expressed
OsISM10306	LOC_Os01g71130	41166187	CAAGGCGTTCGACACAGTC	AAAGCTCGAACTGCTTCTGC	60.2	159	78	xylanase inhibitor 725OS putative expressed
OsISM10307	LOC_Os01g71190	41186067	GATGAAAGCTCTCGCCGTC	ATGAATGCAGGCTTTGGCTA	60.9	1022	99	photosystem II reaction center PSB28 protein chloroplast precursor putative expressed
OsISM10308	LOC_Os01g71200	41192692	GTCATCAAGGACAAGGCCAC	TTGTGGTAAGCGTTCACTGC	60.2	1198	96	RNA recognition motif containing protein putative expressed
OsISM10309	LOC_Os01g71200	41193870	GCAGTGAACGCTTACCACAA	TCTCCAACTCTCCATCAGCA	59.7	1370	83	RNA recognition motif containing protein putative expressed
OsISM10310	LOC_Os01g71200	41195220	TGCTGATGGAGAGTTGGAGA	TCAATTCGGTGTCCGTTACA	59.7	161	78	RNA recognition motif containing protein putative expressed
OsISM10311	LOC_Os01g71200	41195427	AAGGGGTTCACAACAAACAA	TCAATAGCTGCCACTGCTTG	59.1	193	79	RNA recognition motif containing protein putative expressed
OsISM10312	LOC_Os01g71200	41195600	CAAGCAGTGGCAGCTATTGA	GTATCGGCCCATTTGACAAC	60.2	344	84	RNA recognition motif containing protein putative expressed
OsISM10313	LOC_Os01g71200	41196034	ATTTGGGGCTTTACAGATGG	AGGGGTACTGCATAAGTCCG	59.0	499	87	RNA recognition motif containing protein putative expressed
OsISM10314	LOC_Os01g71200	41196803	GTCCCAGTAGCAATCCTGGT	ATTGGAAAGGTCTTGGTCCC	59.6	470	99	RNA recognition motif containing protein putative expressed
OsISM10315	LOC_Os01g71200	41197311	TTTTGTAGACAAAGCAACGGG	CGGAAGCAGGAGAATCGTAG	60.1	171	66	RNA recognition motif containing protein putative expressed
OsISM10316	LOC_Os01g71230	41213419	CACCTCGAGGAGCAAAAGAT	CATCATCCTCGTCATCATCG	59.7	926	68	nascent polypeptide-associated complex subunit alpha-like protein 3 putative expressed
OsISM10317	LOC_Os01g71230	41214340	TGATGATGATGACGAGGATGA	TCCTGCTCTTTTTCTCGCTC	59.9	905	99	nascent polypeptide-associated complex subunit alpha-like protein 3 putative expressed

ISM IDs	Rice gene locus IDs	Physical positions (bp) on chromosomes	Forward primers (5'-3')	Reverse primers (5'-3')	Melting tempearature (T˚C)	Expected product size (bp) in PCR	Expected product size (bp) in Q-PCR	Putative Functions
OsISM10318	LOC_Os01g71230	41215260	TTGGCATGAAGACCATCACT	GGGCTCTTGAAGACATCTGG	59.4	194	95	nascent polypeptide-associated complex subunit alpha-like protein 3 putative expressed
OsISM10319	LOC_Os01g71240	41219463	GATGGTACCCGACCTCGAC	GAACGAAGAGTGCAACACGA	60.2	2319	84	calcium-transporting ATPase plasma membrane-type putative expressed
OsISM10320	LOC_Os01g71240	41221762	TCGTGTTGCACTCTTCGTTC	CAACGGGTGCTCCGTCTT	61.1	198	71	calcium-transporting ATPase plasma membrane-type putative expressed
OsISM10321	LOC_Os01g71240	41223838	GATCCCGACCAGATGAGAGA	GTCACCAGCGTGTGCTTG	60.1	167	81	calcium-transporting ATPase plasma membrane-type putative expressed
OsISM10323	LOC_Os01g71240	41224623	TGGTGAACTTCATCTCTGCG	ATCAGGTTCACCCACAGCA	60.0	194	74	calcium-transporting ATPase plasma membrane-type putative expressed
OsISM10324	LOC_Os01g71240	41225012	CCCTCAAGCTGACAGCCTAC	TTCTCCATCTCCCTGCTGTT	59.9	681	91	calcium-transporting ATPase plasma membrane-type putative expressed
OsISM10325	LOC_Os01g71280	41245703	TTCCTCGTATCATTACGCCC	TCTCTGAGGCAATGTTTGGG	60.5	1028	97	glycerol-3-phosphate dehydrogenase putative expressed
OsISM10326	LOC_Os01g71280	41246871	AAGAATGTCTACGCCATCGG	TACACCGACTTGCTTGTTGC	60.0	685	72	glycerol-3-phosphate dehydrogenase putative expressed
OsISM10327	LOC_Os01g71280	41247693	TAGCGAAGGGAGAACTGAGC	AAATGCACCAACAGCAGAGA	59.6	225	84	glycerol-3-phosphate dehydrogenase putative expressed
OsISM10328	LOC_Os01g71280	41247975	GTTGCACCTGCTGAACTGTG	TCTCTCAGGGCTTGCAGAAT	60.3	566	96	glycerol-3-phosphate dehydrogenase putative expressed
OsISM10329	LOC_Os01g71300	41250406	GACCTTCTCCGTCTTCCTCC	TGACGAAGTTCTCGTCGTTG	60.1	177	93	S-formylglutathione hydrolase putative expressed
OsISM10331	LOC_Os01g71300	41252097	GAGGGAGAGGCAGACAGTTG	ATACATGCGCCAATTTTTCC	59.9	17159	88	S-formylglutathione hydrolase putative expressed
OsISM10332	LOC_Os01g71300	41269321	GGCATCTATTTTTGGGCACT	ACCGGAGAGAATGCAGACAC	59.7	258	97	S-formylglutathione hydrolase putative expressed
OsISM10333	LOC_Os01g71300	41269582	CAATCCAATCAACTGCCCTT	ATCAGGCAGGTTGCATCATA	59.5	194	94	S-formylglutathione hydrolase putative expressed
OsISM10334	LOC_Os01g71300	41269768	TGCCTGATCAAGAAGTGCAA	GGTAGAAGCTGCTTTGCCAG	60.3	941	87	S-formylglutathione hydrolase putative expressed
OsISM10335	LOC_Os01g71310	41299963	AGGTCAGCAATGTCAACCAA	GCCAAGAGCAGCGTAGAAGA	60.0	222	99	cytokinin dehydrogenase precursor putative expressed
OsISM10336	LOC_Os01g71310	41300224	GCTCTTGAACCTGCTCCAAA	GGTGAAGGTCTCAAAGTCCG	60.1	178	67	cytokinin dehydrogenase precursor putative expressed
OsISM10337	LOC_Os01g71310	41300582	AACTTCAACCACGATGAGGC	TCTGAGTCGAGATAGCAGCG	59.8	200	64	cytokinin dehydrogenase precursor putative expressed

ISM IDs	Rice gene locus IDs	Physical positions (bp) on chromosomes	Forward primers (5'-3')	Reverse primers (5'-3')	Melting tempearature (T˚C)	Expected product size (bp) in PCR	Expected product size (bp) in Q-PCR	Putative Functions
OsISM10338	LOC_Os01g71310	41300983	CCCATACTGCTTTACCCAGTG	GGAACCCAACCAGGTAGAAA	59.2	184	92	cytokinin dehydrogenase precursor putative expressed
OsISM10339	LOC_Os01g71330	41342597	TCGACGAGCGCTTCCTAC	GTCCTCCTCCTCTTCTTCGC	60.4	202	99	expressed protein
OsISM10340	LOC_Os01g71340	41345729	CGTTTTAACACTGGCATTGG	TAGCACACGCCGATAGATTG	59.5	502	70	glycosyl hydrolases family 17 putative expressed
OsISM10341	LOC_Os01g71350	41348754	ATTGCTCCTTGCAGCATTTC	GTAGCACACGCCAATGGACT	60.7	196	50	glycosyl hydrolases family 17 putative expressed
OsISM10342	LOC_Os01g71370	41362362	CTAGCTCTCCGACACGCAC	CGGTTCTCTCAAACCGACAT	59.9	145	92	retrotransposon protein putative unclassified
OsISM10343	LOC_Os01g71380	41364203	TATTGGTCATCGGAGCCTTC	GGGAGGTTGTTCCCTTTCAC	60.4	234	80	glycosyl hydrolases family 17 putative expressed
OsISM10344	LOC_Os01g71390	41366889	GAACCAAGGAGCTCAGAGGC	TCGACAACAGGGTAGCCTTC	60.9	1047	91	retrotransposon protein putative unclassified
OsISM10345	LOC_Os01g71400	41369715	TCCTTGGAGTACTTGCGGTC	GTAGCACACGCCAATGGAT	59.9	222	54	glycosyl hydrolases family 17 putative expressed
OsISM10346	LOC_Os01g71410	41374156	CTAGCCGTCGCATTGTTCAT	ATGGTTTAATTCCTGGCGTG	60.5	104	68	glycosyl hydrolases family 17 putative expressed
OsISM10347	LOC_Os01g71490	41402427	ATGGAGTTTCATGGATGGCT	GGCAACATCGTGACCCATAG	60.3	558	71	hypothetical protein
OsISM10348	LOC_Os01g71510	41411683	AAGGTGGTCATTCAACCGAC	GATCTTCGGCCTCGGACT	60.1	182	87	transposon protein putative unclassified
OsISM10349	LOC_Os01g71530	41423942	AACCCTGGGAGACTTACCGT	CATAATATCGACCGCCGACT	59.9	1609	77	retrotransposon protein putative Ty3-gypsy subclass
OsISM10350	LOC_Os01g71530	41425531	AGTCGGCGGTCGATATTATG	CGCGTGAACCATTCCTTATC	60.2	200	51	retrotransposon protein putative Ty3-gypsy subclass
OsISM10351	LOC_Os01g71540	41438440	CAAGGAGGTCATGTCGGAAT	TGAAAGCATCTGGCAGAACA	60.2	1121	78	expressed protein
OsISM10352	LOC_Os01g71540	41439620	TCCCGCCCTTTTCTAGTTATC	GTTAAGGACTTGGCCATCCA	59.8	170	90	expressed protein
OsISM10353	LOC_Os01g71540	41439799	AATCATCCCAGACCCTACCC	TCTCAAGACGGAGTCCAAGC	60.3	328	77	expressed protein
OsISM10354	LOC_Os01g71540	41440504	TCCTGTGCAGTATCATGAGGA	GCAAGCAGTCTAACTGTATCCAAA	59.6	759	94	expressed protein
OsISM10355	LOC_Os01g71540	41441525	CAAGCTGGTGAGGTTTTGGA	TATATGAAACACGCGCAAGG	60.5	2624	80	expressed protein

ISM IDs	Rice gene locus IDs	Physical positions (bp) on chromosomes	Forward primers (5'-3')	Reverse primers (5'-3')	Melting tempearature (T˚C)	Expected product size (bp) in PCR	Expected product size (bp) in Q-PCR	Putative Functions
OsISM10356	LOC_Os01g71540	41444158	AATTCGGGTTTTGCCCTAAC	CCTTTGTGAACTATGTCGCTG	59.3	179	89	expressed protein
OsISM10357	LOC_Os01g71540	41444302	AACAAGATTTCTTACAGCGACAT	ACATTTGTGCAACAGCCTTG	58.5	138	59	expressed protein
OsISM10358	LOC_Os01g71540	41444414	TGTAACCAAGGCTGTTGCAC	TCCCATCCTTTAAGTGCCTTT	59.9	899	85	expressed protein
OsISM10359	LOC_Os01g71540	41445382	CAATGGAGCAGCAGATGAGA	CTTTAGCACCTTCGTGAGCA	59.7	187	99	expressed protein
OsISM10360	LOC_Os01g71540	41445543	TCAGAATGCTCACGAAGGTG	ATCAACAGCAACATGCCACT	59.6	238	64	expressed protein
OsISM10361	LOC_Os01g71570	41456269	CTTGCAGTTTTTGCCTCCAT	TTCACGGCACAACTCTTCAC	60.1	163	75	hypothetical protein
OsISM10362	LOC_Os01g71570	41456412	GTGAAGAGTTGTGCCGTGAA	GGCAACATCGTGACCCATAG	60.6	2065	95	hypothetical protein
OsISM10363	LOC_Os01g71590	41469414	CAAGGAGGTCATGTCGGAAT	ATCTGGCAGAAAATGATCGG	60.0	1114	71	expressed protein
OsISM10364	LOC_Os01g71590	41470594	TCCCGCCCTTTTCTAGTTATC	GTTAAGGACTTGGCCATCCA	59.8	170	90	expressed protein
OsISM10365	LOC_Os01g71590	41470773	AATCATCCCAGACCCTACCC	TCTCAAGACGGAGTCCAAGC	60.3	328	77	expressed protein
OsISM10366	LOC_Os01g71590	41471478	TCCTGTGCAGTATCATGAGGA	GCAAGCAGTCTAACTGTATCCAAA	59.6	759	94	expressed protein
OsISM10367	LOC_Os01g71590	41472499	CAAGCTGGTGAGGTTTTGGA	TATATGAAACACGCGCAAGG	60.5	2623	80	expressed protein
OsISM10368	LOC_Os01g71590	41475131	AATTCGGGTTTTGCCCTAAC	CCTTTGTGAACTATGTCGCTG	59.3	179	89	expressed protein
OsISM10369	LOC_Os01g71590	41475275	AACAAGATTTCTTACAGCGACAT	ACATTTGTGCAACAGCCTTG	58.5	138	59	expressed protein
OsISM10370	LOC_Os01g71590	41475387	TGTAACCAAGGCTGTTGCAC	TCCCATCCTTTAAGTGCCTTT	59.9	899	85	expressed protein
OsISM10371	LOC_Os01g71630	41516886	TCTTAGTCAGCCGCAGTTCA	TTGTTTTCCACCATCACGTT	59.3	817	77	expressed protein
OsISM10372	LOC_Os01g71630	41518094	TGCTAAGAGATGCTGGAATCG	ATCCTCCCGAGGTTTCTTTG	60.5	187	99	expressed protein
OsISM10373	LOC_Os01g71640	41523511	TCCTTGGAGTACTTGCGGTC	AGAAGCTCCGCACGTAAAAA	60.1	341	58	conserved hypothetical protein

ISM IDs	Rice gene locus IDs	Physical positions (bp) on chromosomes	Forward primers (5'-3')	Reverse primers (5'-3')	Melting tempearature (T˚C)	Expected product size (bp) in PCR	Expected product size (bp) in Q-PCR	Putative Functions
OsISM10374	LOC_Os01g71640	41523832	TTTTTACGTGCGGAGCTTCT	CAAGGCAGCGATGACGAC	60.8	272	76	conserved hypothetical protein
OsISM10375	LOC_Os01g71800	41585018	GTCTCCTTCGGCTCCACC	GCGTATCCCTCCTCTTCCAG	60.9	168	99	lecithin cholesterol acyltransferase putative
OsISM10376	LOC_Os01g71830	41602293	TTGAGCATCTCCGACAACTG	GACAACGCCATAGCAGACAC	59.7	248	68	glycosyl hydrolases family 17 putative expressed
OsISM10377	LOC_Os01g71840	41604710	TGGGAAGAAGGAAAGTGTGG	CCCACTCCTAAGACCCATCA	60.0	109	68	retrotransposon protein putative unclassified expressed
OsISM10378	LOC_Os01g71840	41608127	TGCACCTGTGGCTAAGATGA	TATTTCCAAATCCAGGGGG	60.0	156	99	retrotransposon protein putative unclassified expressed
OsISM10379	LOC_Os01g71840	41608179	AACTTTGGATGGCCTCTTCA	CCATATTGCACAATACACGCC	60.4	209	93	retrotransposon protein putative unclassified expressed
OsISM10380	LOC_Os01g71840	41609326	GCTGATTGTTTAACCAAGGGA	TCTTTGTGATGGCATAATCAGT	58.2	473	74	retrotransposon protein putative unclassified expressed
OsISM10381	LOC_Os01g71840	41609772	TGATTACTGATTATGCCATCA CAA	TTGGAGAATCATCGCAAGTG	59.6	177	97	retrotransposon protein putative unclassified expressed
OsISM10382	LOC_Os01g71840	41609932	TTGCGATGATTCTCCAAGTG	GCCTTGGTTGCTTCACAAAT	60.0	287	73	retrotransposon protein putative unclassified expressed
OsISM10383	LOC_Os01g71840	41610199	ATTTGTGAAGCAACCAAGGC	GCTATTACACCGGGGATTGA	60.0	171	70	retrotransposon protein putative unclassified expressed
OsISM10384	LOC_Os01g71840	41610499	TGCTTTACCACCCTTTCGAC	GCAAAGCGGTTTCCTGAAC	60.4	1725	94	retrotransposon protein putative unclassified expressed
OsISM10385	LOC_Os01g71840	41612281	CATGAGACGGACAGCAACAA	AACCTCTCCGGTGAGCAAAT	60.9	649	64	retrotransposon protein putative unclassified expressed
OsISM10386	LOC_Os01g71840	41612901	GTAGGTCCGATTTGCTCACC	CCCAACCTTGATGTTTGGAC	59.9	909	53	retrotransposon protein putative unclassified expressed
OsISM10387	LOC_Os01g71840	41613805	TTGGGTAAAAATCCCATTGA	CACCATGTTCAGGACCTTCA	58.7	188	98	retrotransposon protein putative unclassified expressed
OsISM10388	LOC_Os01g71840	41613982	TGAACATGGTGAAATTGATGG	TGACAGAAGAAGTGTCAACCAA A	59.5	174	93	retrotransposon protein putative unclassified expressed
OsISM10389	LOC_Os01g71840	41614133	TTTGGTTGACACTTCTTCTGTC A	TTGAGAATCTGCTATATGAGGGC	59.8	1397	93	retrotransposon protein putative unclassified expressed
OsISM10390	LOC_Os01g71840	41616069	AATACCTTGGGTGGATGCTG	CATTCTTGTCACCAAACTCAACA	59.9	167	71	retrotransposon protein putative unclassified expressed
OsISM10391	LOC_Os01g71840	41616333	GCCCATTAAGGATTTCAATGA T	TGGTACCAATAGCAGACTCCAA	59.4	211	99	retrotransposon protein putative unclassified expressed

ISM IDs	Rice gene locus IDs	Physical positions (bp) on chromosomes	Forward primers (5'-3')	Reverse primers (5'-3')	Melting tempearature (T°C)	Expected product size (bp) in PCR	Expected product size (bp) in Q-PCR	Putative Functions
OsISM10392	LOC_Os01g71840	41616767	TGAAATCCCTTGTGATGCTC	CATGAGAAGGTGTATGGCGA	59.2	183	93	retrotransposon protein putative unclassified expressed
OsISM10393	LOC_Os01g71840	41616928	TTTCGCCATACACCTTCTCA	TGAAAGGGAAAATCTAACAGCA	59.1	162	88	retrotransposon protein putative unclassified expressed
OsISM10394	LOC_Os01g71840	41617068	TGCTGTTAGATTTTCCCTTTCA	AGCATATCCAACTTCAGGGG	58.9	146	71	retrotransposon protein putative unclassified expressed
OsISM10395	LOC_Os01g71840	41617194	CCCCTGAAGTTGGATATGCT	CCTAACAGCAGCAACAGCAG	59.4	187	93	retrotransposon protein putative unclassified expressed
OsISM10396	LOC_Os01g71840	41617527	CCCTCGATATGTCACTTCCTCT	CTTGAACAGGTGAATTGTCAAGA	59.4	196	72	retrotransposon protein putative unclassified expressed
OsISM10397	LOC_Os01g71840	41617775	TGTGAAGAAGAGGCGTTTTG	CCTTGCAGCAATCAGGAAAT	59.6	173	84	retrotransposon protein putative unclassified expressed
OsISM10398	LOC_Os01g71840	41618165	GTCCGAGAGCTCTAAATGGC	CCTTTCATGAGAGATTGCCC	59.3	180	91	retrotransposon protein putative unclassified expressed
OsISM10399	LOC_Os01g71840	41618402	TCATCCTTCAAACTCCAAGAGA	CCTTGCTCAAATCAGCACAA	59.4	185	87	retrotransposon protein putative unclassified expressed
OsISM10400	LOC_Os01g71840	41618567	TTGTGCTGATTTGAGCAAGG	AGGTGTTCGATGGGGAAAA	60.1	171	97	retrotransposon protein putative unclassified expressed
OsISM10401	LOC_Os01g71840	41618811	GGTGATCCCTATTTTGAAGACA	GCCATCATAAGAATCCCAGAA	58.5	165	73	retrotransposon protein putative unclassified expressed
OsISM10402	LOC_Os01g71840	41619002	GAAAGTTTACCCTTGTGTTCCAA	ACCCTTGGTGACCACAATTC	59.6	682	88	retrotransposon protein putative unclassified expressed
OsISM10403	LOC_Os01g71840	41619755	TCCATCCAACGAATAAAATGC	TCAGCCTTCACCAGCAAAT	59.6	325	87	retrotransposon protein putative unclassified expressed
OsISM10404	LOC_Os01g71860	41628299	TCCAGAAACTCGATCAAGCA	CACCGTAGCAAACACCATTG	59.8	215	90	glycosyl hydrolases family 17 putative expressed
OsISM10405	LOC_Os01g71860	41629384	GCCTCTTCAACCCTGACAAG	CACCATGCCGTAGCACAC	59.8	207	90	glycosyl hydrolases family 17 putative expressed
OsISM10407	LOC_Os01g71890	41652533	GTCCATCTGGACAGGCAAGT	GTGGTTCCTGTCAATCCCAC	60.2	452	75	transposon protein putative unclassified
OsISM10408	LOC_Os01g71890	41653010	GTGCACAGCTCAGCACCTT	AGTGGTAGAAGGCCTGCCG	61.4	172	98	transposon protein putative unclassified
OsISM10409	LOC_Os01g71890	41653496	CTAGCAAGGCTTGGTTGCTC	AACCACAACAGGTACGCCTC	60.1	184	98	transposon protein putative unclassified
OsISM10410	LOC_Os01g71890	41653990	ACGATGGGGACTTACTGGTG	GGAGACGGTCAAACTCGAAC	59.8	190	95	transposon protein putative unclassified

ISM IDs	Rice gene locus IDs	Physical positions (bp) on chromosomes	Forward primers (5'-3')	Reverse primers (5'-3')	Melting tempearature (T˚C)	Expected product size (bp) in PCR	Expected product size (bp) in Q-PCR	Putative Functions
OsISM10411	LOC_Os01g71890	41654352	CGGACTTAGGCAGGTGTTTC	CTAGCTGTCCCCGTCGAGT	60.1	159	81	transposon protein putative unclassified
OsISM10412	LOC_Os01g71890	41654719	GCACCGTGACCGAGACTACT	ATATCGGCACTGATATCCCG	60.0	152	58	transposon protein putative unclassified
OsISM10413	LOC_Os01g71890	41655053	GAGCAGGACCTCGTTTGTCT	AACGCGCTCGAAGGCATA	61.2	187	95	transposon protein putative unclassified
OsISM10414	LOC_Os01g71890	41655307	AGCTCTTCCTCTCACGGAGC	AGACGAAGAGGCCCCAGTA	60.5	144	82	transposon protein putative unclassified
OsISM10415	LOC_Os01g71890	41655637	TACTCCCAGGGGCACATTAG	GGTTATTACCCACCATGCCA	60.2	1316	86	transposon protein putative unclassified
OsISM10416	LOC_Os01g71890	41657711	AGGCCACTGGAGACGTACAC	GCACGCCGTCAATAAACTCT	60.2	852	80	transposon protein putative unclassified
OsISM10417	LOC_Os01g71900	41660086	AGCCAGCCTATAGAGTGCGA	CCGCGTTGTTCCATATGTTT	60.4	222	90	transposon protein putative CACTA En/Spm sub-class
OsISM10418	LOC_Os01g71900	41660315	CACTCGGAACCAAAGGAGAA	CGCGAGAATACCGATCTCTT	59.8	444	97	transposon protein putative CACTA En/Spm sub-class
OsISM10419	LOC_Os01g71900	41660823	ATAACCGTGCTCGTCCAGAG	CAGCCATGCGAGGAAAGTAT	60.3	325	90	transposon protein putative CACTA En/Spm sub-class
OsISM10420	LOC_Os01g71910	41664647	TAACTGAGGTGGACGCAGAC	CTCCTCCCACTTCGGTATTG	59.2	483	93	transposon protein putative CACTA En/Spm sub-class
OsISM10421	LOC_Os01g71910	41665729	ACGGACACCATGTAAGCTGC	AGTCCCTGAAATGTCCGTTG	60.5	184	89	transposon protein putative CACTA En/Spm sub-class
OsISM10422	LOC_Os01g71910	41666928	CCCTCGACGTCTCTCTTCTG	ACCCAGTATCGTAAACCCCC	60.0	169	79	transposon protein putative CACTA En/Spm sub-class
OsISM10423	LOC_Os01g71910	41667182	GCAGCATTTCAAGACGTTCA	ATGCGTCCAAGTCGAAAAAG	60.1	169	78	transposon protein putative CACTA En/Spm sub-class
OsISM10424	LOC_Os01g71910	41667381	TTGACAAAGGTCTTCCAACTGA	GTTTGATAGGCAGTGGGCAT	59.9	369	99	transposon protein putative CACTA En/Spm sub-class
OsISM10425	LOC_Os01g71910	41667730	ATGCCCACTGCCTATCAAAC	TGTGGGAGTTTTTCCCTCAT	59.7	175	74	transposon protein putative CACTA En/Spm sub-class
OsISM10426	LOC_Os01g71940	41680958	CATTCAACGCCGTGCTATTT	TGACCAACTGATCGAAGACG	60.4	920	98	retrotransposon protein putative unclassified
OsISM10427	LOC_Os01g71940	41681952	CCTCATGAGCATGTGGTGAC	GACGTCCCCGTATCATTGTC	60.2	175	66	retrotransposon protein putative unclassified
OsISM10428	LOC_Os01g71940	41682105	ATGACAATGATACGGGGACG	GTTGGGACTCTCTCGTCCTG	60.2	1191	90	retrotransposon protein putative unclassified

ISM IDs	Rice gene locus IDs	Physical positions (bp) on chromosomes	Forward primers (5'-3')	Reverse primers (5'-3')	Melting tempearature (T°C)	Expected product size (bp) in PCR	Expected product size (bp) in Q-PCR	Putative Functions
OsISM10429	LOC_Os01g71960	41690401	ACAAATCCTACCGCAACGTC	TTGCCTTATGACCCATTTCC	59.9	611	96	endonuclease putative expressed
OsISM10430	LOC_Os01g71960	41691047	ATCTCCCCCGGAAAGTAATG	GCACATAACGCTTTGACCCT	60.1	198	66	endonuclease putative expressed
OsISM10431	LOC_Os01g71960	41691253	AATTCTGCTGCTTACGCGAT	GGCGTCAATAAGCTCTTGCT	59.8	242	88	endonuclease putative expressed
OsISM10432	LOC_Os01g71960	41691475	AGCAAGAGCTTATTGACGCC	CTGAGAACTTGCAAAAGCCC	59.8	190	99	endonuclease putative expressed
OsISM10433	LOC_Os01g71960	41691681	GATGGAGCTGCATGAAAACA	GCAGTCGATTTCCCTTCTTC	59.3	1199	95	endonuclease putative expressed
OsISM10434	LOC_Os01g71960	41693074	CACCAGAAGTGACATATTTGACAAG	CACTAAGAATGATTTCTTCAGCACA	59.9	533	94	endonuclease putative expressed
OsISM10435	LOC_Os01g71960	41693582	TGTGCTGAAGAAATCATTCTTAGTG	GGGGCAACCCTCTCAGTAAA	60.6	181	99	endonuclease putative expressed
OsISM10436	LOC_Os01g71960	41693761	CCTCCAATATTGAATGCAAGC	GTTCCTTCAGACGAACGAGG	59.7	202	96	endonuclease putative expressed
OsISM10437	LOC_Os01g71960	41693940	TCTCCTCGTTCGTCTGAAGG	CAGTATCATTGTCCAGCATATTGAA	60.4	218	86	endonuclease putative expressed
OsISM10438	LOC_Os01g71960	41694233	TGTTGATATTGGATGATCGTGA	CAGGTACACGAAATTGTGAATG	58.5	940	82	endonuclease putative expressed
OsISM10439	LOC_Os01g71960	41695117	CAGGGGTCGTTCAAGAAAAA	CAAATACCATCCCCAACAGG	60.1	568	91	endonuclease putative expressed
OsISM10440	LOC_Os01g71960	41695735	TTGAAAGGAAGAATGTTGCAGA	ATCAGCTTCCTTAGGCCACA	59.8	634	98	endonuclease putative expressed
OsISM10441	LOC_Os01g71960	41696378	TGAAGGTGATCCAAACCCTC	GGGGTTCTCTGGACATCAAA	59.9	246	94	endonuclease putative expressed
OsISM10442	LOC_Os01g71960	41696777	TGTGAGTGATGTATTCGCCC	CCTATCACAGCAAGTGCAGC	59.6	179	73	endonuclease putative expressed
OsISM10443	LOC_Os01g71960	41696932	AGAAGCTGCACTTGCTGTGA	ACATCTTCTCTTGGGCATGG	60.0	304	99	endonuclease putative expressed
OsISM10444	LOC_Os01g71980	41715286	GTCGTCGGCAGGTTCTTC	TTCTTCAGACGATCACCACCT	59.5	685	51	rho-GTPase-activating protein-like putative expressed
OsISM10445	LOC_Os01g71980	41716180	TTGGCAGGCTCTTTTTAAGTG	CCAAAGATACTCCAGCCGAC	59.6	853	69	rho-GTPase-activating protein-like putative expressed
OsISM10446	LOC_Os01g72120	41839251	CTTCTGGGTTCACTTCCTCG	CGTGTCTCCTCCATGGATTT	59.9	442	97	glutathione S-transferase putative expressed

ISM IDs	Rice gene locus IDs	Physical positions (bp) on chromosomes	Forward primers (5'-3')	Reverse primers (5'-3')	Melting tempearature (T˚C)	Expected product size (bp) in PCR	Expected product size (bp) in Q-PCR	Putative Functions
OsISM10447	LOC_Os01g72130	41841000	GTTTCTGGGCGCAGTTCAT	GAACGGGATCCAGAAATTGA	60.5	165	57	glutathione S-transferase putative expressed
OsISM10448	LOC_Os01g72140	41843297	CGTTTCTGGGCTCACTTCAT	GTCTCCTTCAGAAGCCCCTC	60.3	535	96	glutathione S-transferase putative expressed
OsISM10449	LOC_Os01g72150	41845685	CGTTTCTGGGCTCAATTCAT	AGAAACCCCTTCTGCTCCTC	59.9	202	87	glutathione S-transferase putative expressed
OsISM10450	LOC_Os01g72160	41846964	GTTTCTGGGCCCAATTCAT	ATCCAGAACGACAGCCAGAA	60.3	535	59	glutathione S-transferase putative expressed
OsISM10451	LOC_Os01g72190	41861594	CCTGGATCAAGAAGCAGAGG	TCCATTTGAAGCATCGACAC	59.8	207	98	expressed protein
OsISM10452	LOC_Os01g72210	41869198	GTCGTCCGCCTCTTCAAAT	TGCAAGGATGAACAATCCAA	60.1	179	70	early-responsive to dehydration protein-related putative expressed
OsISM10453	LOC_Os01g72210	41869448	CAATGTGGCAAGGGGTTCTA	GAAGCAGAGGCATGCAAAAT	60.6	417	53	early-responsive to dehydration protein-related putative expressed
OsISM10454	LOC_Os01g72210	41869845	ATTTTGCATGCCTCTGCTTC	TGCAATTCTTCTGCTGGACA	60.5	333	84	early-responsive to dehydration protein-related putative expressed
OsISM10455	LOC_Os01g72210	41870330	CATTGGAAACATCAAGGCACT	CTTGTCCTCAAGGGAGGATG	59.8	145	50	early-responsive to dehydration protein-related putative expressed
OsISM10456	LOC_Os01g72210	41870581	ACGTCCATCACTCTATCCGC	GCATCCAGCTGTGATTTGAA	60.0	241	95	early-responsive to dehydration protein-related putative expressed
OsISM10457	LOC_Os01g72210	41871040	AAATATGGTTTCCACCGGCT	TCATGCTTGGAAGATACCCA	59.8	186	79	early-responsive to dehydration protein-related putative expressed
OsISM10458	LOC_Os01g72210	41871423	GACATACCTAGCCGTCTGGC	CTCCAAGGAAAACCCTGACA	59.9	177	94	early-responsive to dehydration protein-related putative expressed
OsISM10459	LOC_Os01g72210	41871784	TTTTGCTTGGTTACGCAGTG	TGTAGCGATGAATATTGGGC	59.3	404	97	early-responsive to dehydration protein-related putative expressed
OsISM10460	LOC_Os01g72210	41872297	CAAGGTTCGGTTTCTTCCAA	ACCTCTCATCCCCTCAGCTT	60.1	213	89	early-responsive to dehydration protein-related putative expressed
OsISM10461	LOC_Os01g72230	41881707	CACCAATTCGTTGATAGCCC	CTCGATGCCATTCCACAAAT	60.6	237	63	stromal membrane-associated protein putative expressed
OsISM10462	LOC_Os01g72230	41881967	ATCAGCAAGGAGGCAATCC	GCACCACAGTCAGCACAAAT	60.0	516	98	stromal membrane-associated protein putative expressed
OsISM10463	LOC_Os01g72230	41882463	ATTTGTGCTGACTGTGGTGC	TCCAGAGCACTTGATGCAAA	60.1	463	79	stromal membrane-associated protein putative expressed
OsISM10464	LOC_Os01g72230	41882932	AGAAGTCTTGGGGTGCACAT	GCAGCATTTCCACCTGAATC	60.1	870	99	stromal membrane-associated protein putative expressed

ISM IDs	Rice gene locus IDs	Physical positions (bp) on chromosomes	Forward primers (5'-3')	Reverse primers (5'-3')	Melting tempearature (T˚C)	Expected product size (bp) in PCR	Expected product size (bp) in Q-PCR	Putative Functions
OsISM10465	LOC_Os01g72230	41883849	CAAGGCAAGATTGCTCATCA	GCAAGTAAGCTGTGTGTTGGA	59.5	226	90	stromal membrane-associated protein putative expressed
OsISM10466	LOC_Os01g72230	41884139	TAGGCCATGCTTTCAGGAAC	TCCAACAAATTCAACCATACCT	59.3	176	93	stromal membrane-associated protein putative expressed
OsISM10467	LOC_Os01g72230	41884360	GATATGATGTCCAGCGACCC	CCATACAGGATTCAGGCTGC	60.5	178	91	stromal membrane-associated protein putative expressed
OsISM10468	LOC_Os01g72230	41884535	TGGAATGAAAGGATTTTGCT	CCCATTCGGTCGTCTGTAGT	58.6	198	96	stromal membrane-associated protein putative expressed
OsISM10469	LOC_Os01g72240	41886877	ATGTGGGAACTGCAAAGGAG	TTCAGCATGCAAATCTCTGG	60.0	739	65	uridine 5-monophosphate synthase putative expressed
OsISM10470	LOC_Os01g72240	41887607	CATGCTGAAAACTCATGTGGA	AAAAAGTTGTGCTTCTCGGC	59.6	191	88	uridine 5-monophosphate synthase putative expressed
OsISM10471	LOC_Os01g72240	41887812	GCAAGTTTGCTGACATTGGA	CATTGACGATATCAGCCCAG	59.5	168	85	uridine 5-monophosphate synthase putative expressed
OsISM10472	LOC_Os01g72240	41887999	TGGAATTGTGGATGGTCTGA	CATTTCAGCAAGCAAAAGCA	60.0	186	68	uridine 5-monophosphate synthase putative expressed
OsISM10473	LOC_Os01g72240	41888337	TGGAGTGCAGATGGTTTCTG	TGTCACTGCCTCTGTCGTTT	59.6	816	84	uridine 5-monophosphate synthase putative expressed
OsISM10474	LOC_Os01g72250	41892483	AGAGCTACTTGATCTCGCCG	GCCAAAATCCGGTGTAAAAT	59.3	885	92	uridine 5-monophosphate synthase putative expressed
OsISM10475	LOC_Os01g72250	41893348	ATTTTACACCGGATTTTGGC	TCAAAAAGCTGTGCCTCTCA	59.3	606	58	uridine 5-monophosphate synthase putative expressed
OsISM10476	LOC_Os01g72250	41893971	TTTGCAGACATTGGAAACACA	GCCCAGTCTAATATGCGGAA	60.1	155	66	uridine 5-monophosphate synthase putative expressed
OsISM10477	LOC_Os01g72250	41894154	GGACCTGGAATTGTAGAAGGC	AGGTTGCCAGCTGAACTCAT	59.9	239	90	uridine 5-monophosphate synthase putative expressed
OsISM10478	LOC_Os01g72250	41894528	TGGAGTTCAGTTGGTTGCTG	TCGCTGCCTCTGTCGTTTAT	60.4	368	82	uridine 5-monophosphate synthase putative expressed
OsISM10479	LOC_Os01g72290	41913880	GCCGACTACAAGTCCCTCAA	ATCAGCCCATCGAAGAAGAA	60.0	221	99	expressed protein
OsISM10480	LOC_Os01g72300	41916938	CCGACTACAAGTCGCTTCAA	CCCGGGAAGAAGAAGTCCT	59.8	380	92	Cupin domain containing protein expressed
OsISM10482	LOC_Os01g72310	41922292	CGGGGGAGTATCAGTCCATA	CAAGCGGCACCAATCTCT	59.9	164	75	OsFBO7 - F-box and other domain containing protein expressed
OsISM10483	LOC_Os01g72310	41922603	CCAGTGCCCAAAGACCTAAT	AGCAACTTTGCTGCTTGTTG	59.2	186	91	OsFBO7 - F-box and other domain containing protein expressed

ISM IDs	Rice gene locus IDs	Physical positions (bp) on chromosomes	Forward primers (5'-3')	Reverse primers (5'-3')	Melting tempearature (T˚C)	Expected product size (bp) in PCR	Expected product size (bp) in Q-PCR	Putative Functions
OsISM10484	LOC_Os01g72310	41922922	CACACACAATGCCTGACAAA	TATGTTATGCCCCCAATCGT	59.6	168	92	OsFBO7 - F-box and other domain containing protein expressed
OsISM10485	LOC_Os01g72310	41923070	ACGATTGGGGGCATAACATA	GCATGCTGGATCATCTTTGA	59.9	213	94	OsFBO7 - F-box and other domain containing protein expressed
OsISM10486	LOC_Os01g72310	41923314	TGGGCCTTGATTTGAGTTTT	CCATACGATGTGCACGACTAA	59.6	220	97	OsFBO7 - F-box and other domain containing protein expressed
OsISM10487	LOC_Os01g72310	41923574	TGGTACCATCGAAGAGCAAA	TTCACCATTTGTCTGCAAGC	59.6	137	64	OsFBO7 - F-box and other domain containing protein expressed
OsISM10488	LOC_Os01g72330	41948388	TCATCGAGATGCTGCTCAAG	CATCACCCTCATCCCTCAAT	60.0	233	97	OsRR4 type-A response regulator expressed
OsISM10489	LOC_Os01g72330	41948632	TCACCTTCTTCCTCCTCACC	CCAGGCATGCAGTAGTCAGT	59.1	190	72	OsRR4 type-A response regulator expressed
OsISM10490	LOC_Os01g72330	41948820	GGCATGACAGGGTATGATCTG	ATCCTTGCAGGCACATTCTC	60.3	222	99	OsRR4 type-A response regulator expressed
OsISM10491	LOC_Os01g72330	41949022	GAGAATGTGCCTGCAAGGAT	TGAGCTTCTTCATGTCAGCG	60.3	1555	92	OsRR4 type-A response regulator expressed
OsISM10492	LOC_Os01g72340	41954019	CCCGTCATTCTTTAGGTGGA	CCTCCGTTCATGCCAATACT	59.9	196	95	beta-galactosidase putative expressed
OsISM10493	LOC_Os01g72340	41954352	TTCCTCGCCAGAATCTGTTC	CATGCATTTGCCAGTTTGAG	60.3	375	96	beta-galactosidase putative expressed
OsISM10494	LOC_Os01g72340	41954823	TTTTTCGCATCCCAAAAGAG	CGCTGAATCAACAGCTTCAA	60.2	713	66	beta-galactosidase putative expressed
OsISM10495	LOC_Os01g72340	41955516	TTGAAGCTGTTGATTCAGCG	CTCAAACTCAGCAGGAAGCC	60.1	721	87	beta-galactosidase putative expressed
OsISM10496	LOC_Os01g72340	41956333	GACCATTGGTGGCTATCTGG	TCATCCAAGGTTGCTTTGAA	59.8	551	96	beta-galactosidase putative expressed
OsISM10497	LOC_Os01g72340	41956878	GGATGAAGGCTTCCGTGTAG	CCGATCCTGCTTTTGTGAGT	60.0	439	65	beta-galactosidase putative expressed
OsISM10498	LOC_Os01g72340	41957475	TGGTGGAAAAGTAGAAAATCCAA	ACTCAATGAGCTTCCCGTTG	60.1	192	99	beta-galactosidase putative expressed
OsISM10499	LOC_Os01g72340	41957774	CCACGTGTTGGAAAGACAAA	TGGGGATAATGGCTGTTTCT	59.5	218	99	beta-galactosidase putative expressed
OsISM10500	LOC_Os01g72340	41958210	ATGGACCAAATCACTCAGCC	TCATAATGGATGGGCCTTGT	60.0	143	68	beta-galactosidase putative expressed
OsISM10501	LOC_Os01g72340	41958440	TTCTGAAAATAGGCCCCTCA	TGTTGTCTATGGCCATCCAAT	59.9	189	93	beta-galactosidase putative expressed

ISM IDs	Rice gene locus IDs	Physical positions (bp) on chromosomes	Forward primers (5'-3')	Reverse primers (5'-3')	Melting tempearature (T˚C)	Expected product size (bp) in PCR	Expected product size (bp) in Q-PCR	Putative Functions
OsISM10502	LOC_Os01g72340	41958633	TGGACTCCAAGGTGGTTTTA	CCACCATATGCCCAGTTTTT	58.9	494	88	beta-galactosidase putative expressed
OsISM10503	LOC_Os01g72340	41959168	ATGGCATTGTATGGCCTGAT	TCATTGTTATTTTGATAGGCTGG	59.2	193	82	beta-galactosidase putative expressed
OsISM10504	LOC_Os01g72340	41959341	GCCTATCAAAATAACAATGATGGA	AGCCAACTGAAGTCCAAAGC	59.4	166	88	beta-galactosidase putative expressed
OsISM10505	LOC_Os01g72340	41959692	ATGGCCATATCTTGGCTTCA	AGCTCCTGGGCAGTGCTAT	60.2	159	82	beta-galactosidase putative expressed
OsISM10506	LOC_Os01g72340	41959898	AATGCCTGGCAAATCAAAGT	CATGGGTGTATTCCCTTGCT	59.7	474	93	beta-galactosidase putative expressed
OsISM10507	LOC_Os01g72340	41961009	CTCGTCAAGGGAGACGACAT	GCCCCATGTGCTTGTGAT	60.4	200	50	beta-galactosidase putative expressed
OsISM10508	LOC_Os01g72350	41963587	ATCTTCCTCCTCCGCCCT	CAGTATCATGTCAGCGACGG	60.7	169	67	amidohydrolase putative expressed
OsISM10509	LOC_Os01g72350	41963836	CGTGGGCGGATACTACTCTG	TCCCGGAAGAACAACATTTC	60.3	282	83	amidohydrolase putative expressed
OsISM10510	LOC_Os01g72350	41964133	GTGCACTTCATTGATGGTGG	AGCTTCCTTGACCCTGTTGA	59.9	222	91	amidohydrolase putative expressed
OsISM10511	LOC_Os01g72350	41964307	CGAGGGGTTACAAGCAAAGA	TCCTCCAAATATCCATTGCC	60.0	620	85	amidohydrolase putative expressed
OsISM10512	LOC_Os01g72350	41964950	AGGTGATTACCCCACTGCTG	ATATGTCCATCCATGCGTGA	59.9	153	79	amidohydrolase putative expressed
OsISM10513	LOC_Os01g72350	41965131	ATGGCTGGGATCAACAAAAA	GCAGCATCAACTAGAAGACCG	60.2	499	93	amidohydrolase putative expressed
OsISM10514	LOC_Os01g72350	41965766	TCAGAAAAGCAAGTCTGGCA	GCATGGGGAAGAATAAGCAA	59.9	413	95	amidohydrolase putative expressed
OsISM10515	LOC_Os01g72350	41966159	TTGCTTATTCTTCCCCATGC	ATCCGACCTTTTTCATGGAT	59.2	160	64	amidohydrolase putative expressed
OsISM10516	LOC_Os01g72350	41966356	TTCCTTGATGGTTCTTTGGG	TGTCAACCAATTGGAGACCAT	60.1	459	89	amidohydrolase putative expressed
OsISM10517	LOC_Os01g72350	41966799	CTCCAATTGGTTGACATGGA	TCTCCAATTGCATGTATGGC	59.4	169	87	amidohydrolase putative expressed
OsISM10518	LOC_Os01g72350	41967003	AGGTGGTTGATTTGAATGGAA	ATACCATGTTTGCCAAAGCG	60.1	216	98	amidohydrolase putative expressed
OsISM10519	LOC_Os01g72350	41967199	CGCTTTGGCAAACATGGTAT	TTTCTTCCCAGCAGAATTGG	60.5	481	76	amidohydrolase putative expressed

ISM IDs	Rice gene locus IDs	Physical positions (bp) on chromosomes	Forward primers (5'-3')	Reverse primers (5'-3')	Melting tempearature (T˚C)	Expected product size (bp) in PCR	Expected product size (bp) in Q-PCR	Putative Functions
OsISM10520	LOC_Os01g72350	41967740	GCACATCTGGCTTTTGGTTC	CAACAGGTTTGCGGGATACT	60.3	168	86	amidohydrolase putative expressed
OsISM10521	LOC_Os01g72350	41967897	CAAACCTGTTGGATGGGAAG	GGCATAGGCGGCAGATATAG	60.0	661	92	amidohydrolase putative expressed
OsISM10522	LOC_Os01g72460	42017075	ACCGAAGGCGTTGAGAGTT	TGGACTCCTCGCATATCTCC	60.0	207	99	NADPH quinone oxidoreductase putative expressed
OsISM10523	LOC_Os01g72460	42017405	ACTGCTTCCTCTTCGCCTCT	CCTTGATCCCCAGTCCAAC	60.5	581	78	NADPH quinone oxidoreductase putative expressed
OsISM10524	LOC_Os01g72480	42024840	CACCACGACTCTCCCCATC	ATCTTATCCAGGCAAATCGC	60.3	200	94	zinc finger C3HC4 type domain containing protein expressed
OsISM10525	LOC_Os01g72480	42025023	ATTTGCCTGGATAAGATCGC	GTTTGTAGGACGCCCACCT	59.6	814	98	zinc finger C3HC4 type domain containing protein expressed
OsISM10526	LOC_Os01g72480	42025868	GTTTGATTTCCTCAGCGTGC	CAAACGCTTTCCTCGAACAT	60.5	155	70	zinc finger C3HC4 type domain containing protein expressed
OsISM10527	LOC_Os01g72500	42041160	CACATGCTCTTGAGTTGGGA	TTCCAGATGGAGAATGCACA	60.0	650	81	retrotransposon protein putative unclassified expressed
OsISM10528	LOC_Os01g72560	42080133	TGGTTGTGATGCCAAGAAAC	ATCGCGTCAAACATTTAGGG	59.8	252	98	expressed protein
OsISM10529	LOC_Os01g72560	42080306	CTGCAGAATGCTTAGGGAGG	GAAGCAGGCCCTTCTCTTCT	60.0	303	88	expressed protein
OsISM10530	LOC_Os01g72560	42080658	GAGATCACCTCGGACGACAT	ACGTCTTGCTGCAACAATTT	59.3	705	99	expressed protein
OsISM10531	LOC_Os01g72560	42081354	GCAAGACGTGCATGAAGATG	GCATAGCCATGTTCCTTGTG	59.8	666	97	expressed protein
OsISM10532	LOC_Os01g72560	42082101	ACAACCACGAGAATGGAAGG	TTGTTCAGCTATGTCACCCG	59.8	861	65	expressed protein
OsISM10533	LOC_Os01g72560	42082935	TAGGGAACGGGTGACATAGC	TCTCCAGGGCAGTCTGAATC	60.2	246	80	expressed protein
OsISM10534	LOC_Os01g72560	42083332	CGAAGATTTCTACCATGCCA	TCGACGGTATCTTGTACTGCAC	59.5	185	81	expressed protein
OsISM10535	LOC_Os01g72560	42083520	AAGCCCTGAAGGAAGCGTA	TCCCTCTTCAACTGTCCCTG	60.1	263	56	expressed protein
OsISM10536	LOC_Os01g72570	42085958	GCGACTACCCCTTCTTCCTC	ATGGCGAAGATACAGGATCG	60.1	2274	78	expressed protein
OsISM10537	LOC_Os01g72570	42088292	GGCCTCTTGGAGGCTTTATC	TATTGGTATCGAAGCCCCAG	60.0	362	73	expressed protein

ISM IDs	Rice gene locus IDs	Physical positions (bp) on chromosomes	Forward primers (5'-3')	Reverse primers (5'-3')	Melting tempearature (T˚C)	Expected product size (bp) in PCR	Expected product size (bp) in Q-PCR	Putative Functions
OsISM10538	LOC_Os01g72570	42088634	CTGGGGCTTCGATACCAATA	GCAGATAAAAGAAGCTGCCAA	59.8	146	59	expressed protein
OsISM10539	LOC_Os01g72570	42088820	GGATGCTTTCTTGTGACGGT	ATGAGCAGTGGCCACAAAAT	60.3	748	99	expressed protein
OsISM10540	LOC_Os01g72570	42089581	TTTCTCCAAGCCGCTGATAC	CAATTTCTTGGCAGCATTCA	60.1	153	61	expressed protein
OsISM10541	LOC_Os01g72570	42089711	TTCTGAATGCTGCCAAGAAA	ACAAAAAGATCAACCGAGCC	59.4	211	47	expressed protein
OsISM10542	LOC_Os01g72570	42089902	GGCTCGGTTGATCTTTTTGT	GGCACTCCCCATAACTTTGA	59.6	180	99	expressed protein
OsISM10543	LOC_Os01g72570	42090078	TGCCATTCCATCAACTACCA	TGGTAATAGTGGTGCCCCTT	59.6	386	99	expressed protein
OsISM10544	LOC_Os01g72570	42090530	GCAGTTGTATCTTCTCTGGCCT	TAGGGTGAAGGCGTAGATGG	60.0	3034	61	expressed protein
OsISM10545	LOC_Os01g72630	42138001	GGCCTTCTACCGCATCATAG	GCAATATCCAGTTCGTCAAGC	59.7	165	64	expressed protein
OsISM10546	LOC_Os01g72630	42138275	GCCCTTGATAACTACCAGCG	AGTGGTATTGATCCCAGTCCA	59.5	1598	68	expressed protein
OsISM10547	LOC_Os01g72630	42139903	CATATGTCAAATTCTGTGAAG AGC	CCTCAACAGCAATGAGTACCAA	59.1	157	76	expressed protein
OsISM10548	LOC_Os01g72630	42140038	TTGGTACTCATTGCTGTTGAG G	GTGCGATCTTCATTGCTTGA	60.1	172	89	expressed protein
OsISM10549	LOC_Os01g72630	42140190	TCAAGCAATGAAGATCGCAC	AACAATCAAACCGCATGCTC	60.5	175	96	expressed protein
OsISM10550	LOC_Os01g72630	42140392	ATTTTGAGGTTTGGAAGCGG	TCTGACAGGGCATCAGTGAA	60.9	177	59	expressed protein
OsISM10551	LOC_Os01g72630	42140623	TCGCAGTACTCGAGGGAGAT	CAATGCAAGCCACGTAGAGA	60.0	155	63	expressed protein
OsISM10552	LOC_Os01g72630	42140759	CTCTACGTGGCTTGCATTGA	TGACGAGCTTCCTTTTCTGG	60.3	128	48	expressed protein
OsISM10553	LOC_Os01g72630	42140867	CCAGAAAAGGAAGCTCGTCA	TTGTTTCTGGCGTAATCCAA	59.8	724	87	expressed protein
OsISM10554	LOC_Os01g72630	42141600	CGCAGAAGATATCGGTTTGA	GCTGTGTTGTGCAGTGACCT	59.4	174	74	expressed protein
OsISM10555	LOC_Os01g72650	42151006	AGGCAGCTGCAAAGGCTAT	AGGGACTCTCTCCTGAAGCA	59.6	695	81	RNA recognition motif containing protein putative expressed

ISM IDs	Rice gene locus IDs	Physical positions (bp) on chromosomes	Forward primers (5'-3')	Reverse primers (5'-3')	Melting tempearature (T˚C)	Expected product size (bp) in PCR	Expected product size (bp) in Q-PCR	Putative Functions
OsISM10556	LOC_Os01g72650	42151968	GATTGCTGGGGTGACAGAAT	CAAGAAGTCAAGCCCCTCAA	60.2	186	85	RNA recognition motif containing protein putative expressed
OsISM10557	LOC_Os01g72650	42152211	CCGTCTACCGTTAAGCTTGC	GGCTTCCTCCAAGCTCATT	59.6	177	58	RNA recognition motif containing protein putative expressed
OsISM10558	LOC_Os01g72650	42152484	TTCTGCGGACATGTTGAATC	AAAGAGGATGCCCAAGAACA	59.7	397	95	RNA recognition motif containing protein putative expressed
OsISM10559	LOC_Os01g72650	42153099	GTACGCTGTGTCTGTGGTGC	GTTTTGCCGAACAATGTCGT	60.7	271	73	RNA recognition motif containing protein putative expressed
OsISM10560	LOC_Os01g72650	42153394	TGGCATCTTCTTGGAAGTTCA	TGGTGAATCTCTAACGACATCAG	60.0	282	94	RNA recognition motif containing protein putative expressed
OsISM10561	LOC_Os01g72650	42153613	GCTGATTCTAAGAAGCCAGTTGA	CTCTAGGAATTCCAGCGATGA	59.7	790	92	RNA recognition motif containing protein putative expressed
OsISM10562	LOC_Os01g72650	42154370	CTCTGCAGATTTTCATCGCT	CAGCCAATTGACCAAATGAA	58.9	196	85	RNA recognition motif containing protein putative expressed
OsISM10563	LOC_Os01g72650	42154584	ATGAAGACCTTGGTGGAGCA	CAGCACATGCCTTTGATGTAA	60.2	157	70	RNA recognition motif containing protein putative expressed
OsISM10564	LOC_Os01g72650	42154779	GCTGTTGGCGTGTTAACTGA	CAGGAATTCCATGAAATGGG	60.0	148	68	RNA recognition motif containing protein putative expressed
OsISM10565	LOC_Os01g72650	42154912	TTCATGGAATTCCTGCCAAT	CAGCGAGTATTCCTCCTGGT	59.8	250	93	RNA recognition motif containing protein putative expressed
OsISM10566	LOC_Os01g72650	42155182	TGCAGTATTGGAAGATGTACGTG	GTGCCTTGGTGTTGTCACTG	60.1	182	99	RNA recognition motif containing protein putative expressed
OsISM10567	LOC_Os01g72690	42176944	GAGGTGAGGGTGAGCCAAC	AATTCCCTCCGAGGATGC	60.3	210	85	NAD kinase putative expressed
OsISM10568	LOC_Os01g72690	42177218	CCCCAAGCTTCAGATCATTG	ATGGACCCAGACCCAATTTC	60.8	1378	95	NAD kinase putative expressed
OsISM10569	LOC_Os01g72690	42178669	ATCAAGTCAACCGATGCACA	TTTCCCTTCTGCAACTCGTC	60.2	217	85	NAD kinase putative expressed
OsISM10570	LOC_Os01g72690	42178939	GGCACACAAACAGCAGAGAA	TGGCTGGCCAACTTTTCTAA	60.4	153	73	NAD kinase putative expressed
OsISM10571	LOC_Os01g72690	42179309	ATAACAACAGCAGAGCGCAG	GAGGATTCCCATTTCAGCAA	59.6	184	51	NAD kinase putative expressed
OsISM10572	LOC_Os01g72690	42179532	CTCCGTGCATGTTCTTTGTG	CCTTGCTAACACGTGGCTCT	60.4	197	87	NAD kinase putative expressed
OsISM10573	LOC_Os01g72690	42179754	CAATTTCATCCAAACATGGG	AGTCCCATCACCACCAAGAG	59.3	731	89	NAD kinase putative expressed

ISM IDs	Rice gene locus IDs	Physical positions (bp) on chromosomes	Forward primers (5'-3')	Reverse primers (5'-3')	Melting tempearature (T˚C)	Expected product size (bp) in PCR	Expected product size (bp) in Q-PCR	Putative Functions
OsISM10574	LOC_Os01g72690	42180465	CTCTTGGTGGTGATGGGACT	TAGGGAGAAAGCAACAACCG	60.1	552	78	NAD kinase putative expressed
OsISM10575	LOC_Os01g72690	42180997	CGGTTGTTGCTTTCTCCCTA	ATGGTCCATTCAGCACATTG	59.8	308	94	NAD kinase putative expressed
OsISM10576	LOC_Os01g72690	42181514	GGAAGCACGGCATATTCATT	CAGATCGGCGTGAATAGGAT	60.0	465	78	NAD kinase putative expressed
OsISM10577	LOC_Os01g72700	42184690	GGCAACTGTTCGTCCCTCTA	TCTCCAGCTTCTTCAGCTCC	60.0	208	92	expressed protein
OsISM10578	LOC_Os01g72710	42190031	GGACGATCTCTTCCTCGCTA	TCTCTTCTCATACTCCGCAGC	59.6	184	80	expressed protein
OsISM10579	LOC_Os01g72710	42190193	TGCTGCGGAGTATGAGAAGA	TGAAATCAGCAACTATTGCCA	59.5	750	90	expressed protein
OsISM10580	LOC_Os01g72710	42190994	CAAATGCTGGGCATATTGCT	CCCTAGCCTCTGCAGAATTG	60.5	196	99	expressed protein
OsISM10581	LOC_Os01g72710	42191170	CAATTCTGCAGAGGCTAGGG	AGCAAAACAAAATGGCAAGC	60.1	211	96	expressed protein
OsISM10582	LOC_Os01g72710	42191306	TTGCAGAGAAATGGTGCAAA	TCAACAGCCTTCCTTGCTTT	60.2	725	99	expressed protein
OsISM10583	LOC_Os01g72710	42192082	TGCTTATGGTGTATACATGGCAG	TGTTCGATTACACCAGCCAA	60.0	323	67	expressed protein
OsISM10584	LOC_Os01g72710	42192475	GGCAACACATTCTTGGGTTC	ACCCACCTGGCATAGTCAAC	60.1	132	48	expressed protein
OsISM10585	LOC_Os01g72720	42198482	AGGAGGAGGTGAGGGAGGT	AGACCATTACATCGGGGAAG	59.5	123	87	hypothetical protein
OsISM10586	LOC_Os01g72720	42198819	GCTGTACGCCTACTTCCACC	GGGACGAACACCTTGTTCAC	60.1	118	79	hypothetical protein
OsISM10587	LOC_Os01g72730	42201478	GAGTTGCTCAGCCCAAGG	CCCAGCTCCTCCAGGTACTC	60.3	198	87	cytochrome P450 putative expressed
OsISM10588	LOC_Os01g72730	42201913	TCAGAGGGATGGTGGGTTAG	GGAACATCCTTCGACCAAAA	59.9	781	90	cytochrome P450 putative expressed
OsISM10589	LOC_Os01g72740	42206171	TGGGATCACCCTCACTAAAGA	GGACATGGCCCAAATTATTG	59.8	613	98	cytochrome P450 putative expressed
OsISM10590	LOC_Os01g72760	42211770	CTAAGGAAATTGTCAGCGCC	GACATGGCCCAGAGTATGGT	59.8	240	83	cytochrome P450 putative expressed
OsISM10591	LOC_Os01g72780	42216516	CTCCTCTGGAGGCTCTCCTT	AGTGCGCAAATAGCTTCTCC	59.9	167	91	expressed protein

ISM IDs	Rice gene locus IDs	Physical positions (bp) on chromosomes	Forward primers (5'-3')	Reverse primers (5'-3')	Melting tempearature (T°C)	Expected product size (bp) in PCR	Expected product size (bp) in Q-PCR	Putative Functions
OsISM10592	LOC_Os01g72780	42217111	CCAAATTTGTGGATGGAGGT	CATTTTCTGTCCAGAATCAGGT	58.9	855	72	expressed protein
OsISM10593	LOC_Os01g72780	42218161	GGCCTTGGTTTTGTTTCTGA	GTCTGTTTTCGCAGCGATTT	60.2	1082	92	expressed protein
OsISM10594	LOC_Os01g72780	42219213	ACATTGTCACAAATCGCTGC	ATTTTATCTGGCCCCAAAGC	60.0	416	99	expressed protein
OsISM10595	LOC_Os01g72780	42219720	GCCTCACCACAACAATTCCT	TCTTCACATTTCCGTGCTGA	60.2	168	93	expressed protein
OsISM10596	LOC_Os01g72780	42220021	AAATCCGCTGTTAAAGCCCT	TTTCTCTGTTTTGCTCACTGCT	59.7	202	74	expressed protein
OsISM10597	LOC_Os01g72780	42220378	GATTCTTCACCAGAGAAGCGTT	TGAGCCAAACATCTCAATGC	59.8	934	54	expressed protein
OsISM10598	LOC_Os01g72780	42221423	AGACAAGAAGGATGAAGAGGG	CAGCAGCTTCAGCGAATGT	58.9	449	96	expressed protein
OsISM10599	LOC_Os01g72780	42221862	GAAGCTGCTGACGAGGATCT	GCAAATAGAGCTTTGCCCTC	59.4	181	96	expressed protein
OsISM10600	LOC_Os01g72780	42222008	AACAAGCAACCTTGTGAGGG	TTATTTCATCCAACCGAGCC	60.0	182	92	expressed protein
OsISM10601	LOC_Os01g72780	42222252	TGCTACCAGTATTTGTTGGTTCA	GCCTTCTTGCTCGTGTTCTC	59.9	835	60	expressed protein
OsISM10602	LOC_Os01g72780	42223067	GAGAACACGAGCAAGAAGGC	GCCAGGAGTACATCATATGCC	59.8	320	99	expressed protein
OsISM10603	LOC_Os01g72780	42223398	GCTTGAGAAGCTCTGAGTCAAAC	TGTGTGGAGCTGGACTTGAA	60.1	1083	91	expressed protein
OsISM10604	LOC_Os01g72780	42224576	TTGCTGAAGCATAAAGCTATTGA	CCAACACCTTTACAAAGCCC	59.3	143	59	expressed protein
OsISM10605	LOC_Os01g72780	42224764	TCATGAGCGGCATATTACCA	TCAAAGCCACATTTCCTAATCA	59.8	201	98	expressed protein
OsISM10606	LOC_Os01g72780	42224949	GGAAATGTGGCTTTGATGCT	GGATTATGATTTCCCTTGCG	59.7	1100	95	expressed protein
OsISM10607	LOC_Os01g72780	42226074	CAACAACCTACGCAACATGC	TGTGTGTTCCCTCTTGTCCA	60.2	174	77	expressed protein
OsISM10608	LOC_Os01g72790	42228693	CTTCGAGAAGCTCGAGCAGA	ATCTCGTTCGTCTCCGTCTC	60.3	164	76	cyclin-dependent kinase putative expressed
OsISM10609	LOC_Os01g72790	42228868	AGAAGATCCGCATGGACAAC	TTCTGGTGGTGGAGCTTCTT	60.0	182	86	cyclin-dependent kinase putative expressed

ISM IDs	Rice gene locus IDs	Physical positions (bp) on chromosomes	Forward primers (5'-3')	Reverse primers (5'-3')	Melting tempearature (T˚C)	Expected product size (bp) in PCR	Expected product size (bp) in Q-PCR	Putative Functions
OsISM10610	LOC_Os01g72790	42229030	AAGAAGCTCCACCACCAGAA	ATGTGCCATACTTGGCTTCC	59.9	192	94	cyclin-dependent kinase putative expressed
OsISM10611	LOC_Os01g72790	42229166	TATATGCCTTTTCAGGCCCA	CCATGTAGATGCTCCCCTTG	60.4	170	79	cyclin-dependent kinase putative expressed
OsISM10612	LOC_Os01g72790	42229361	TACTGGGCTTGCAGATAGGC	CAGGCCTGTAAGTAGCTGCC	60.2	179	80	cyclin-dependent kinase putative expressed
OsISM10613	LOC_Os01g72790	42229546	TGCCATGTTAATCAAGTGCTG	ATCTTGCAAGGCCAAAATCA	60.2	195	98	cyclin-dependent kinase putative expressed
OsISM10614	LOC_Os01g72790	42229728	GGCCTTGCAAGATCATTCTC	CGTGCTCCCGAGTAACAACT	60.0	187	97	cyclin-dependent kinase putative expressed
OsISM10615	LOC_Os01g72790	42229965	GCTTCTCAATGGAAAGCCAA	AGATTAACTCGTCGGGCGTA	60.0	381	93	cyclin-dependent kinase putative expressed
OsISM10616	LOC_Os01g72790	42230391	AACGACCAATGAAGAGACGG	CTCTAAAAGATCCAGCGCGT	59.9	150	69	cyclin-dependent kinase putative expressed
OsISM10617	LOC_Os01g72790	42230521	ACGCGCTGGATCTTTTAGAG	AAATACTCCGCGTCAAGTGC	59.9	182	83	cyclin-dependent kinase putative expressed
OsISM10618	LOC_Os01g72790	42230702	TCTGGACTGATCCCTTACCG	TGAGACGCCTCATACTTCGG	60.7	127	56	cyclin-dependent kinase putative expressed
OsISM10619	LOC_Os01g72820	42240851	TTGGCTTCGTTCTCACTCCT	AGCATTTCCCAAATGAAGCA	60.3	484	95	CRS1/YhbY domain containing protein putative expressed
OsISM10620	LOC_Os01g72820	42241595	CCTGAAGCTCGACTCAAATACA	GGCCTTGTGCTTTAGCAATC	59.7	447	98	CRS1/YhbY domain containing protein putative expressed
OsISM10621	LOC_Os01g72820	42242336	CAGATGTCATGTCACCAGTCG	AAACGCCTCACTGTCTCCAG	60.3	233	89	CRS1/YhbY domain containing protein putative expressed
OsISM10622	LOC_Os01g72820	42242836	TGTTCTTTATGATCATGGAGGCT	GGTGATCATCCACGGAGTTT	59.9	160	54	CRS1/YhbY domain containing protein putative expressed
OsISM10623	LOC_Os01g72834	42247302	TCGTCATCACCGACAAGAAC	AGCAAGCTTTCATAGCAGCC	59.7	450	85	RNA recognition motif containing protein putative expressed
OsISM10624	LOC_Os01g72834	42247795	TCTTGCATATCTTGGGGTCC	CTTCATTGCCCTCATATGCC	60.2	1153	80	RNA recognition motif containing protein putative expressed
OsISM10625	LOC_Os01g72834	42249012	GGATCCCGACCTACAATGTG	GTACCAGCGGATAGCCGTAA	60.2	1846	64	RNA recognition motif containing protein putative expressed
OsISM10626	LOC_Os01g72834	42250824	CCTTATTTCTCGGATTACGGC	CCCACCGTACGCTTGATAGT	60.0	337	52	RNA recognition motif containing protein putative expressed
OsISM10627	LOC_Os01g72834	42251447	CTAACTCTACCGGCCAAGCA	GCAGAGTTGGAGCTGTCAGA	59.8	1679	55	RNA recognition motif containing protein putative expressed

ISM IDs	Rice gene locus IDs	Physical positions (bp) on chromosomes	Forward primers (5'-3')	Reverse primers (5'-3')	Melting tempearature (T˚C)	Expected product size (bp) in PCR	Expected product size (bp) in Q-PCR	Putative Functions
OsISM10628	LOC_Os01g72860	42260212	TCCGCTCTGACGTCTTATCA	ACAATGATAGCTCCCGGTTG	59.8	266	61	MAC/Perforin domain containing protein putative expressed
OsISM10629	LOC_Os01g72860	42260649	TTCCATCAACATGGGAACCT	TCCCATTTTTACACCAACGA	59.5	265	81	MAC/Perforin domain containing protein putative expressed
OsISM10630	LOC_Os01g72860	42261006	ATTTCTTGATGCAAATGGGC	CCACAAATCTCAGTCGTTGC	59.6	345	96	MAC/Perforin domain containing protein putative expressed
OsISM10631	LOC_Os01g72860	42261318	TTGAAGCAAGAGAGCAACGA	CACCTCTTCGCTTAGGCATC	59.9	180	97	MAC/Perforin domain containing protein putative expressed
OsISM10632	LOC_Os01g72860	42261616	TGGATTTTTGAACCATGCAA	CTGCCTTGGAAGCTGAAACT	59.8	798	92	MAC/Perforin domain containing protein putative expressed
OsISM10633	LOC_Os01g72860	42262462	CAAAGCAGTGCATCTTTACCA	ACAGGTCTTTTGCCCACATC	59.5	509	84	MAC/Perforin domain containing protein putative expressed
OsISM10634	LOC_Os01g72880	42268167	TCAAGCTCATCCAGGTCTCC	TCGCACAATATTGCCAAATC	59.9	190	65	DNA mismatch repair protein Mlh1 putative expressed
OsISM10635	LOC_Os01g72880	42268456	CCATGTTACCGTGACAACGA	AGGCTTAGGCTCATTCTCCA	59.7	171	89	DNA mismatch repair protein Mlh1 putative expressed
OsISM10636	LOC_Os01g72880	42268631	CTGCGGTGAAAGGAACTCA	TTCTGCAATGTTTTCTTGCG	60.0	165	74	DNA mismatch repair protein Mlh1 putative expressed
OsISM10637	LOC_Os01g72880	42268828	TCATCAGTCGGTTTGCAGTC	GAACATCTGCTCTATTGGCTCC	60.0	147	76	DNA mismatch repair protein Mlh1 putative expressed
OsISM10638	LOC_Os01g72880	42269110	CAAATGCAAATTATGTGGCAA	TCAAAGCAGTACAGTCTACAAGCC	59.7	401	76	DNA mismatch repair protein Mlh1 putative expressed
OsISM10639	LOC_Os01g72880	42269585	CTTCCATCAGAACACGTGGA	TTGTTTCAATAATACGCTCTTGATTC	59.8	219	83	DNA mismatch repair protein Mlh1 putative expressed
OsISM10640	LOC_Os01g72880	42269811	TGCTATTGAGGAAAAACTGATGA	TTTGTGGGTTAGCTTGAGCA	59.2	744	93	DNA mismatch repair protein Mlh1 putative expressed
OsISM10641	LOC_Os01g72880	42270531	GGATTGCTCAAGCTAACCCA	TCTGACCATTTGGCTCACAG	60.0	284	96	DNA mismatch repair protein Mlh1 putative expressed
OsISM10642	LOC_Os01g72880	42270857	CACGGGCAATCTTCAAATCT	CACCAGCATCTTTTTGGTTTC	59.8	382	89	DNA mismatch repair protein Mlh1 putative expressed
OsISM10643	LOC_Os01g72880	42271252	TCATGAGCTCCTTGTGGAAA	TCATCGGCAAGTCCAACATA	59.7	169	88	DNA mismatch repair protein Mlh1 putative expressed
OsISM10644	LOC_Os01g72880	42271414	CCGATGAAGCCTTTGCTTT	GCACAAAGCTTGCTGGTACA	60.2	351	96	DNA mismatch repair protein Mlh1 putative expressed
OsISM10645	LOC_Os01g72880	42271869	TGATGAGAAACTGGAGATTGC	TGCCATCTTGATCAATGTGAAT	59.1	238	99	DNA mismatch repair protein Mlh1 putative expressed

ISM IDs	Rice gene locus IDs	Physical positions (bp) on chromosomes	Forward primers (5'-3')	Reverse primers (5'-3')	Melting tempearature (T˚C)	Expected product size (bp) in PCR	Expected product size (bp) in Q-PCR	Putative Functions
OsISM10646	LOC_Os01g72880	42272131	TGGACCAGTACACCCCTGAT	GCACTCTTTCTCGTCATCCC	60.0	308	87	DNA mismatch repair protein Mlh1 putative expressed
OsISM10647	LOC_Os01g72880	42272549	GGCTGATGAACATGCTGAGA	CCCATGCTGCTTCTGCTT	60.0	354	84	DNA mismatch repair protein Mlh1 putative expressed
OsISM10648	LOC_Os01g72880	42272934	GTTTCCATCCATGCGACTTT	TTGTAGAGTTTCTCCAAGGAAGC	59.5	604	91	DNA mismatch repair protein Mlh1 putative expressed
OsISM10649	LOC_Os01g72890	42274693	GCTCCTTCTCGTCGTCGT	AGGGGATGAAGGAGATCGAC	59.7	942	99	transposon protein putative CACTA En/Spm sub-class expressed
OsISM10650	LOC_Os01g72890	42275642	GAGGATCGCCATCAAAGAAA	GATTCAGGAGCAGGAGATGC	60.0	501	89	transposon protein putative CACTA En/Spm sub-class expressed
OsISM10651	LOC_Os01g72890	42276152	CCACATTGATCATTTGTCCAG	TCCATGGATAGCTCCACGTT	59.7	935	94	transposon protein putative CACTA En/Spm sub-class expressed
OsISM10652	LOC_Os01g72890	42277054	TGGTGAAGTGGTTAACGTGG	ATCCATACCCACGAGGAAGG	60.3	145	63	transposon protein putative CACTA En/Spm sub-class expressed
OsISM10653	LOC_Os01g72890	42277215	GAGAACTGATGCTGAGAAAGC TC	CGTGGTGCAAGTGTGAATCT	59.8	170	88	transposon protein putative CACTA En/Spm sub-class expressed
OsISM10654	LOC_Os01g72890	42277457	CGCTCCTAGTGCTGAAAAGG	TTGGAGGAGTTCTTTTGCGT	60.0	269	90	transposon protein putative CACTA En/Spm sub-class expressed
OsISM10655	LOC_Os01g72890	42277876	GTTCTCCCTCTCCACCACCT	CACGAAGCCTCCTAGGTGAA	60.4	145	64	transposon protein putative CACTA En/Spm sub-class expressed
OsISM10656	LOC_Os01g72890	42278018	GTGGAAGCCCATCACCAC	TTCTTCCAGGAGGGCTTCTT	60.1	318	94	transposon protein putative CACTA En/Spm sub-class expressed
OsISM10657	LOC_Os01g72890	42278385	CCCTCATTCACGTTCCAGAT	ATGAATCAGACCTTCCACGC	60.0	170	75	transposon protein putative CACTA En/Spm sub-class expressed
OsISM10658	LOC_Os01g72890	42278559	TTCTCGATCACCCAGTCCTC	GTGACATGTTACCTTGGGGG	60.1	483	76	transposon protein putative CACTA En/Spm sub-class expressed
OsISM10659	LOC_Os01g72890	42278998	AGAAGGGTATCAAGGAGCCG	GGCTGTCGCTAGAGATGCTT	60.2	155	65	transposon protein putative CACTA En/Spm sub-class expressed
OsISM10660	LOC_Os01g72910	42281984	CACAAGCACATGGAGCAGAT	GTCCTTCTTCGCCTGCTTCT	60.5	218	85	abscisic stress-ripening putative expressed
OsISM10661	LOC_Os01g72930	42297815	GAGGATTGGTTGATGAGGGA	TGCAATGCTTTATGGTTGGA	60.0	263	79	pentatricopeptide putative expressed
OsISM10662	LOC_Os01g72950	42311373	GCCGAAGAAGATCCTCAAGA	TGCTTACGTTGATGCTGTTCA	60.0	251	99	expressed protein
OsISM10663	LOC_Os01g72950	42311558	CAGTGAAGAAGGGCCAGATT	CTAGCCCCTTGAAGAAAGGC	59.8	225	97	expressed protein

ISM IDs	Rice gene locus IDs	Physical positions (bp) on chromosomes	Forward primers (5'-3')	Reverse primers (5'-3')	Melting tempearature (T˚C)	Expected product size (bp) in PCR	Expected product size (bp) in Q-PCR	Putative Functions
OsISM10664	LOC_Os01g72950	42311764	CCTTTCTTCAAGGGGCTAGA	GGCATACTCCCCTGTTTCAA	59.2	199	82	expressed protein
OsISM10665	LOC_Os01g72950	42311919	TGCAGGTTTTGGACATCAGA	ATGTAAGTTGGCAGCCATGC	60.7	175	95	expressed protein
OsISM10666	LOC_Os01g72950	42312079	GCTGCCAACTTACATGCTCA	GAGCTAGCTTGAACGCAACC	60.1	188	89	expressed protein
OsISM10667	LOC_Os01g72960	42314474	CTACTTCTGGCCCGTCCTC	GTAAAGCCAATTGCTGACCG	60.2	1723	97	expressed protein
OsISM10668	LOC_Os01g72970	42326015	CTTCTTGAGGTGGGGAAGGT	GGCACGGTCAGAGATAGAGG	60.2	207	96	DUF630/DUF632 domains containing protein putative expressed
OsISM10669	LOC_Os01g72970	42326354	ATGTGCTTGGGAAAAGAAGC	CGTCCAGAGATTTTAGCCTCC	59.8	214	96	DUF630/DUF632 domains containing protein putative expressed
OsISM10670	LOC_Os01g72970	42326704	TCCTCAACTCGTTGATCTCAT	ATTTTGCTGTCCTGAATGGC	58.7	198	97	DUF630/DUF632 domains containing protein putative expressed
OsISM10671	LOC_Os01g73040	42376361	ATGAGAAGGCCTCCAACTCC	GCCAACCTCAGCTTCTTCAC	60.3	219	80	CBS domain-containing protein putative expressed
OsISM10672	LOC_Os01g73040	42376662	CTCACTGATTCCAACGGGAT	CTGCTATCACCCGTCCTGAT	60.0	414	68	CBS domain-containing protein putative expressed
OsISM10673	LOC_Os01g73040	42377137	TATGTCCAACTCACCAGCCA	AGCATGGCAATTACCTCACC	60.0	436	97	CBS domain-containing protein putative expressed
OsISM10674	LOC_Os01g73040	42377667	CGACAGTGGGGAAATGACTT	AACAGCTGGTCTCGCAGATT	60.0	182	63	CBS domain-containing protein putative expressed
OsISM10675	LOC_Os01g73040	42377838	GACCAGCTGTTCAAGCCTTC	TGGCAGCTGCTATTACAGGA	59.8	172	92	CBS domain-containing protein putative expressed
OsISM10676	LOC_Os01g73040	42378011	AAAAATGAGAGAATACCGAGTCAA	GGCCACTAATCGCAATACCA	59.6	238	98	CBS domain-containing protein putative expressed
OsISM10677	LOC_Os01g73040	42378242	AGTGGCCCAAAGTTTATCCC	CGATGTATCCAATGTCGCAC	60.1	190	83	CBS domain-containing protein putative expressed
OsISM10678	LOC_Os01g73040	42378448	ATTCAATGCAAGACGGGAAA	TAGAGCATCCAAACATGCGA	60.4	149	78	CBS domain-containing protein putative expressed
OsISM10679	LOC_Os01g73040	42378577	TCGCATGTTTGGATGCTCTA	TGCCATACTGTTCGCTTCAG	60.2	285	78	CBS domain-containing protein putative expressed
OsISM10680	LOC_Os01g73040	42378880	TTCTGGGATTCAGCTCTTGC	TCTGATGTTGCCATTCGAGA	60.4	169	78	CBS domain-containing protein putative expressed
OsISM10681	LOC_Os01g73040	42379117	AAGGGCGTATGCACAGATTC	ACGATACGGCAGAAACAAGC	60.2	163	64	CBS domain-containing protein putative expressed

ISM IDs	Rice gene locus IDs	Physical positions (bp) on chromosomes	Forward primers (5'-3')	Reverse primers (5'-3')	Melting tempearature (T˚C)	Expected product size (bp) in PCR	Expected product size (bp) in Q-PCR	Putative Functions
OsISM10682	LOC_Os01g73040	42379260	GCTTGTTTCTGCCGTATCGT	CAGAACCACCCTGTCACCTT	60.1	575	89	CBS domain-containing protein putative expressed
OsISM10683	LOC_Os01g73040	42379815	AAGGTGACAGGGTGGTTCTG	CTCATCCATATGCAGCCTCA	59.9	198	99	CBS domain-containing protein putative expressed
OsISM10684	LOC_Os01g73070	42391363	CACATTTCCAAGAAATCCCC	GACGCACGATAGATGTGGAA	59.4	185	97	expressed protein
OsISM10685	LOC_Os01g73070	42391548	CAAGGTCAAGGGATGCTGAT	AGTTGATCGATCTCCCCACA	60.3	265	96	expressed protein
OsISM10686	LOC_Os01g73070	42391858	CAGGTTGTCGTGTTTGTTGG	ACCATTTGCATGGTGAGCTA	59.6	172	83	expressed protein
OsISM10687	LOC_Os01g73070	42392006	CTGGTAGCTCACCATGCAAA	CCATTTGATTGGCTGGAAGT	59.9	205	96	expressed protein
OsISM10688	LOC_Os01g73100	42403400	GGCATTCAGCCTTCTCTGTC	ACCCGCGGTATATAAGGGAC	60.0	1043	93	retrotransposon protein putative unclassified
OsISM10689	LOC_Os01g73100	42404461	AACTCGGAGATATCGGACCC	CACTGGAAGGGGATGTAACG	60.3	1027	95	retrotransposon protein putative unclassified
OsISM10690	LOC_Os01g73100	42405816	GCTAGCTTCTGCCTCTCTGG	CGTTGGTGTTGTCGTTCTCA	60.1	390	95	retrotransposon protein putative unclassified
OsISM10691	LOC_Os01g73100	42406215	ACACCGACAAAGCCTCATTC	CGATGATGATGGAAATGCAG	60.1	354	70	retrotransposon protein putative unclassified
OsISM10692	LOC_Os01g73100	42406597	TCTTCCATCAAGTCAAGCCC	CCTGGTGACTCGTGACATTG	60.2	691	73	retrotransposon protein putative unclassified
OsISM10693	LOC_Os01g73110	42411051	AGAGAGCGCAAGGCAAAACT	CTGTTCTCCCACACCAACCT	60.8	150	84	expressed protein
OsISM10694	LOC_Os01g73110	42411291	CTTTGCCAGGATCACTCTCC	GCTTCTTGTCCCACATGGAT	59.9	188	99	expressed protein
OsISM10696	LOC_Os01g73170	42428982	CGTCTCCTGCTCCGACAT	AGCGGCACCTTGTACGAC	59.9	1730	79	peroxidase precursor putative expressed
OsISM10697	LOC_Os01g73190	42435685	CTGTCCCAAGGCGGAGAC	ATGGTCTCGTTGGGTGGAG	61.4	395	97	peroxidase precursor putative expressed
OsISM10698	LOC_Os01g73220	42445359	GTCTCCTGCTCCGACATCCT	CCCAGTGGCACCTTGTACTC	61.2	465	81	peroxidase precursor putative expressed
OsISM10700	LOC_Os01g73234	42451028	TGGAGAAGTCTGAAAAGCCTG	ATTGACATGGACCACACCTG	59.4	656	61	thioredoxin putative expressed
OsISM10701	LOC_Os01g73234	42451785	CAAATTGAAGCATTGCCAAC	TAGTTGATTAGCAGGCAGCG	59.2	205	85	thioredoxin putative expressed

ISM IDs	Rice gene locus IDs	Physical positions (bp) on chromosomes	Forward primers (5'-3')	Reverse primers (5'-3')	Melting tempearature (T°C)	Expected product size (bp) in PCR	Expected product size (bp) in Q-PCR	Putative Functions
OsISM10702	LOC_Os01g73240	42455879	GAGGAGGAGGACGACGAAAT	TCAACGACGTTCTGTTCGAC	60.2	188	99	expressed protein
OsISM10704	LOC_Os01g73260	42466161	GCCGCAATTATTCAGAAAGG	CGTACTGCTCCTTCGCTTTG	60.4	1010	71	hypothetical protein
OsISM10705	LOC_Os01g73270	42469345	CTTGGTTGTCTTGCCGTCTT	CGTGTAGGAGGATGATTCGG	60.4	546	85	expressed protein
OsISM10706	LOC_Os01g73270	42470165	GACACCTCCACCGTGCTC	GCGATCTGAGCACGAACC	60.4	587	93	expressed protein
OsISM10707	LOC_Os01g73280	42471825	TCGGTTCCTCTCTCTTGGAA	GGGATCGTCTTTGTCCCTAA	59.5	338	92	conserved hypothetical protein
OsISM10710	LOC_Os01g73344	42510043	ATGGGGATCTGTTCTTGTGG	AATCAATCATGCCTTCTCCG	59.9	423	96	retrotransposon protein putative Ty3-gypsy subclass expressed
OsISM10711	LOC_Os01g73344	42511130	CACTCTCTCCTTCGCTCCAG	AGGCACATGATATCGACCGT	60.3	292	96	retrotransposon protein putative Ty3-gypsy subclass expressed
OsISM10712	LOC_Os01g73344	42511319	GGCTCGTTTTCCCATTAGTTT	CGGCTACCAGTCCTGGTGTA	60.3	278	99	retrotransposon protein putative Ty3-gypsy subclass expressed
OsISM10713	LOC_Os01g73400	42536703	TTCGTACAAGCGTGTGAAGC	TGGTACTTGAAGTTTACATTTTG GTC	59.9	190	97	hypothetical protein
OsISM10714	LOC_Os01g73410	42543803	TGGAAAAGGTGCTGCTGATA	ACATCGGTGCCTCAGGTTAT	59.4	186	98	FYVE zinc finger domain containing protein expressed
OsISM10715	LOC_Os01g73410	42544070	TCCAAAGCTGCAAAGGAAGT	TCATTCCCATCATAGGCTCC	59.9	566	96	FYVE zinc finger domain containing protein expressed
OsISM10716	LOC_Os01g73410	42545080	AACATTACGCGATGGGACTC	GTTCTCTGCTTGATGCTCCC	60.0	458	77	FYVE zinc finger domain containing protein expressed
OsISM10717	LOC_Os01g73440	42558205	GCCTCCAGCACTTTCAACTC	GGAGGCCATTCTTCTTGACA	60.1	233	60	conserved hypothetical protein
OsISM10718	LOC_Os01g73450	42562019	CTCCGACTCTCCCTCCAACT	AGCATAAGGCTGAATGGTGG	60.4	1270	61	amino acid kinase putative expressed
OsISM10719	LOC_Os01g73450	42563324	GGCAAAGGGTGTTGCTTAAA	CTCTTGCAATTGCCATCGT	60.0	181	91	amino acid kinase putative expressed
OsISM10720	LOC_Os01g73450	42563473	GTTGTTGCAGATCACGATGG	CCGCCACCAACAACAATAG	60.0	317	85	amino acid kinase putative expressed
OsISM10721	LOC_Os01g73450	42563835	CCGCTCATCAGCAGATTACA	TTTGGACACGGGTTGGTATT	60.0	670	99	amino acid kinase putative expressed
OsISM10722	LOC_Os01g73450	42564626	TACAGCTGCTGCTCTTCGTT	GGTCTGCGTCGTACACACC	59.6	699	84	amino acid kinase putative expressed

ISM IDs	Rice gene locus IDs	Physical positions (bp) on chromosomes	Forward primers (5'-3')	Reverse primers (5'-3')	Melting tempearature (T˚C)	Expected product size (bp) in PCR	Expected product size (bp) in Q-PCR	Putative Functions
OsISM10723	LOC_Os01g73450	42565406	TGACTGCCATTACGCTATGC	CACCAACAATAGCTTTCGCA	59.9	174	91	amino acid kinase putative expressed
OsISM10725	LOC_Os01g73460	42567484	GCGGAGAAATATGGGCTTTT	GTGCATAACAAGGGAACGCT	60.3	2898	61	ATXR putative expressed
OsISM10726	LOC_Os01g73460	42570544	ACACTGGAAGAAGGTGGCAT	TGCATGGTTCTGCAAAGTTC	59.7	151	72	ATXR putative expressed
OsISM10727	LOC_Os01g73460	42570721	TTGTGGTGTTTGATTCACGC	CGGCGATGAAGGTCATATCT	60.3	156	73	ATXR putative expressed
OsISM10728	LOC_Os01g73460	42570994	GTGGAAACATTTCCCGTTTC	TCACCATCTATGTCATACCGGA	59.7	501	98	ATXR putative expressed
OsISM10729	LOC_Os01g73480	42579942	ACCTTCCATCCAGATTGCCT	GTTGCTGGCCGTAAATTGTT	60.4	373	92	acetyltransferase GNAT family protein
OsISM10730	LOC_Os01g73480	42580295	AACAATTTACGGCCAGCAAC	ATGTGACATGCAGAGCACG	59.7	145	79	acetyltransferase GNAT family protein
OsISM10731	LOC_Os01g73480	42580354	GGTTGGGTGTTTTGTTTGCT	TTTGCAAAGGACGGCAGTA	60.1	259	89	acetyltransferase GNAT family protein
OsISM10732	LOC_Os01g73480	42580633	GCATACTGCAGCGAAACCTG	CCCAAGAAAAGCCATCTTCA	61.0	520	89	acetyltransferase GNAT family protein
OsISM10733	LOC_Os01g73480	42581290	ATCGATATGCTCCACCAAGC	TTCTCCATCCTTCTCAAGAACC	59.9	330	97	acetyltransferase GNAT family protein
OsISM10734	LOC_Os01g73480	42581597	TGGTTCTTGAGAAGGATGGAG	GAAAGGCATCTCAGCCAGTT	59.4	391	78	acetyltransferase GNAT family protein
OsISM10735	LOC_Os01g73480	42581996	GCTGCCAGCTTACCAGAAAC	GGCCGGGATCACTAGATTTT	60.2	228	98	acetyltransferase GNAT family protein
OsISM10736	LOC_Os01g73530	42599537	TTCCTCCTCCTCCTCCTCTC	TGAACAAAATCATAGCTGATTGC	59.6	870	94	ABC transporter ATP-binding protein putative expressed
OsISM10737	LOC_Os01g73530	42600395	TGATTTTGTTCAGTCTGTGATTTG	GCCTGAAGTTAGCAGTTGGG	59.5	271	93	ABC transporter ATP-binding protein putative expressed
OsISM10738	LOC_Os01g73530	42600727	TGCTGCCCATTTTTAGATCC	GAATATTCTTCCTGACGTTTTTG	58.5	483	95	ABC transporter ATP-binding protein putative expressed
OsISM10739	LOC_Os01g73530	42601367	AATCATCTTCTCGGCTTGATG	TTCTATCAACAAAGCCTATCGAA	58.7	1070	83	ABC transporter ATP-binding protein putative expressed
OsISM10740	LOC_Os01g73530	42602559	CAGTTAAACCCCATAGAGTTAAGTCAC	TCGTCAAGCAATTGACCAAA	60.0	365	85	ABC transporter ATP-binding protein putative expressed
OsISM10741	LOC_Os01g73530	42603294	ACCTCTCCTGCTCTTTCACG	TGATACCGAAGATTGCTCAACA	60.1	555	55	ABC transporter ATP-binding protein putative expressed

ISM IDs	Rice gene locus IDs	Physical positions (bp) on chromosomes	Forward primers (5'-3')	Reverse primers (5'-3')	Melting tempearature (T˚C)	Expected product size (bp) in PCR	Expected product size (bp) in Q-PCR	Putative Functions
OsISM10742	LOC_Os01g73530	42603936	TCCTTGTGTGAAATGCTTGC	CATATGACATCATAAGAACCATCAA	58.9	128	54	ABC transporter ATP-binding protein putative expressed
OsISM10744	LOC_Os01g73530	42604893	GTGCTGATGGGCAAATTGTA	TCCGACAGCAACCTACATCC	60.3	222	87	ABC transporter ATP-binding protein putative expressed
OsISM10745	LOC_Os01g73530	42605331	CTGTGGCCACTTATGTCAGG	GGTTCAGTTTCCTGGTCTGC	59.4	155	67	ABC transporter ATP-binding protein putative expressed
OsISM10746	LOC_Os01g73530	42605488	TTAGCTATGGTGGCATGGTG	GATCCACCTACCAAAAGGGA	59.2	175	94	ABC transporter ATP-binding protein putative expressed
OsISM10747	LOC_Os01g73530	42605756	GGCTGTTCTACCATAGGCCA	TTTTCCTGAACTGTCCACCC	60.0	202	72	ABC transporter ATP-binding protein putative expressed
OsISM10748	LOC_Os01g73530	42605965	TCCTCATGATGATTTTTATCTCCTT	AAAAGCCCTTTGAACAGCAA	59.6	179	99	ABC transporter ATP-binding protein putative expressed
OsISM10749	LOC_Os01g73530	42606169	TTCCATATTGCCTGTTCTGTTG	TTGCAGCACACTAATCCCAA	60.1	614	99	ABC transporter ATP-binding protein putative expressed
OsISM10750	LOC_Os01g73530	42606779	GCAAAGTGCTGCAAATTCAA	TCATCTAACAACAATGAAAATACGAA	59.5	214	96	ABC transporter ATP-binding protein putative expressed
OsISM10751	LOC_Os01g73530	42607014	TGCGTGACTTGTCTTTTTATTTTT	AAACTTAAGCCCACCGACAA	59.5	179	94	ABC transporter ATP-binding protein putative expressed
OsISM10752	LOC_Os01g73530	42607269	TTGTGCATTGGTAACTGATTTTG	CCTTGTAAAAAGCATTTCGTTTC	59.6	154	67	ABC transporter ATP-binding protein putative expressed
OsISM10753	LOC_Os01g73530	42607400	GAAACGAAATGCTTTTTACAAGGT	TCAACAAGTGGTTTCACCATTC	59.9	254	78	ABC transporter ATP-binding protein putative expressed
OsISM10754	LOC_Os01g73530	42607759	ATGAAGATTTTGTCAGGGCG	GTCAACTTTGCCAATGACCG	60.8	176	97	ABC transporter ATP-binding protein putative expressed
OsISM10755	LOC_Os01g73530	42608000	CGGACACATGCTGAGTCAAT	TGTTTGCAGAGAGTGCAACAG	60.0	992	98	ABC transporter ATP-binding protein putative expressed
OsISM10756	LOC_Os01g73530	42609047	GGGTAATTAACTTCTGACATCTGC	ATGGTTTTCAGTGTCCAGGG	59.1	182	90	ABC transporter ATP-binding protein putative expressed
OsISM10757	LOC_Os01g73530	42609351	GCAACATGTGCTGATTTTAGGA	AATAATTTCTTCCGATGCCG	59.6	236	52	ABC transporter ATP-binding protein putative expressed
OsISM10758	LOC_Os01g73530	42609688	ATCATCTGGCAGCTTTTCGT	AAGTATACGGACGTTGCGGA	60.2	209	52	ABC transporter ATP-binding protein putative expressed
OsISM10759	LOC_Os01g73530	42609901	AGGAACCTTGAGGGATCAGA	GCCATAATCAAAATAAAGGAGTGC	59.4	167	81	ABC transporter ATP-binding protein putative expressed
OsISM10760	LOC_Os01g73530	42610044	GCACTCCTTTATTTTGATTATGGC	TCTGCTGTTCTCCCAAGGAT	60.0	263	81	ABC transporter ATP-binding protein putative expressed

ISM IDs	Rice gene locus IDs	Physical positions (bp) on chromosomes	Forward primers (5'-3')	Reverse primers (5'-3')	Melting tempearature (T˚C)	Expected product size (bp) in PCR	Expected product size (bp) in Q-PCR	Putative Functions
OsISM10762	LOC_Os01g73550	42614128	GTCACCACGCTCCCCAAC	GAATCCACGAACACCCCTAC	60.9	174	81	mitochondrial-processing peptidase subunit alpha mitochondrial precursor putative expressed
OsISM10763	LOC_Os01g73550	42614538	TCCTCCAAGACGAAGTCGAG	AACTTCTTCTCTGGCAAAGGC	60.3	139	48	mitochondrial-processing peptidase subunit alpha mitochondrial precursor putative expressed
OsISM10764	LOC_Os01g73550	42614780	TCACACGAATCAATGGCAGT	TACAACCAAGCGATCAGCAG	60.1	241	69	mitochondrial-processing peptidase subunit alpha mitochondrial precursor putative expressed
OsISM10765	LOC_Os01g73550	42615123	ATCGGTGGGGATTTTAGACA	TCTCTCTCTTCAAGCCAGCC	59.5	184	90	mitochondrial-processing peptidase subunit alpha mitochondrial precursor putative expressed
OsISM10766	LOC_Os01g73550	42615287	GGCTGGCTTGAAGAGAGAGA	AAGAGAATGAACCACCACCG	59.9	186	74	mitochondrial-processing peptidase subunit alpha mitochondrial precursor putative expressed
OsISM10767	LOC_Os01g73550	42615453	CGGTGGTGGTTCATTCTCTT	AAATGATTCGACCGTGTGGT	60.1	512	95	mitochondrial-processing peptidase subunit alpha mitochondrial precursor putative expressed
OsISM10768	LOC_Os01g73550	42615951	CGGTCGAATCATTTTCGGTA	CAACAGCCTTTGCAACAAAA	60.4	340	97	mitochondrial-processing peptidase subunit alpha mitochondrial precursor putative expressed
OsISM10769	LOC_Os01g73550	42616312	TTGCTATTGCAACACCTGGA	TGTTGAGTTTTTCGCTCGTG	60.1	150	60	mitochondrial-processing peptidase subunit alpha mitochondrial precursor putative expressed
OsISM10770	LOC_Os01g73550	42616469	CCGTGCTGATGAATCTTGAA	ATTGCCTTACCTGCAACCAT	59.6	202	84	mitochondrial-processing peptidase subunit alpha mitochondrial precursor putative expressed
OsISM10771	LOC_Os01g73550	42616604	GCACCAGGTAATTGTTGCAG	TGAAGAAAATGGTCAACAGGC	59.7	163	79	mitochondrial-processing peptidase subunit alpha mitochondrial precursor putative expressed
OsISM10772	LOC_Os01g73550	42616827	ACCACCCACAATGGCTAGTT	CCACTGATCATTGTGTCGGA	59.9	181	96	mitochondrial-processing peptidase subunit alpha mitochondrial precursor putative expressed
OsISM10773	LOC_Os01g73580	42625579	CACTTCCAGCCTCCAAAGAA	TGTGAAGAAGATGCGTACCG	60.1	816	56	glycosyl hydrolases putative expressed
OsISM10774	LOC_Os01g73580	42626345	GCAAATTTGTGGCAATTCAA	TCCGTTCGGGTTATACTGGT	59.4	176	86	glycosyl hydrolases putative expressed
OsISM10775	LOC_Os01g73580	42627257	GGGGCTGGACTAATGAGACA	TAACCAAATTGTCCTGGGGA	60.1	775	87	glycosyl hydrolases putative expressed
OsISM10776	LOC_Os01g73580	42628135	TGTTTGAGATCAAGGGAACTGA	CCAGGAAGGGTCAAAAGGAT	60.0	185	96	glycosyl hydrolases putative expressed
OsISM10777	LOC_Os01g73580	42628466	CATGGTTCTCCTGTGCTCTG	GAAGCCTCCATATGCTGGTG	60.0	285	74	glycosyl hydrolases putative expressed
OsISM10778	LOC_Os01g73580	42628759	TGACCTTGAAAAGGAGAAGAAGA	CTCTGGCCATGATGCACG	61.0	206	99	glycosyl hydrolases putative expressed
OsISM10779	LOC_Os01g73620	42645527	ATAGCGTCCGAGGAACTCAA	CTGCAGCGACTTGGTTTTC	59.7	167	82	expressed protein

ISM IDs	Rice gene locus IDs	Physical positions (bp) on chromosomes	Forward primers (5'-3')	Reverse primers (5'-3')	Melting tempearature (T˚C)	Expected product size (bp) in PCR	Expected product size (bp) in Q-PCR	Putative Functions
OsISM10780	LOC_Os01g73620	42645858	TAAGAGTGCGACCACAAGCA	TAGGAACATTACCACGCCCT	60.0	700	78	expressed protein
OsISM10781	LOC_Os01g73620	42646786	GACCAAACAATGAAGCTCGC	TCCATCCATCTTGCTTCCTC	60.5	289	93	expressed protein
OsISM10782	LOC_Os01g73620	42647177	AAAGAACGGGTTTTGGTGAA	ATGCCTTGCCCTTAGTCAGA	59.6	336	89	expressed protein
OsISM10783	LOC_Os01g73620	42648156	CAGCCCAGAAACAAGAAAGC	CTGAACCCTTCTTCGTTGGA	60.1	174	93	expressed protein
OsISM10784	LOC_Os01g73620	42648344	TCTCGACACTCGGAGATCCT	CTCTTCCCGGTCATCTTCTG	59.9	169	70	expressed protein
OsISM10785	LOC_Os01g73620	42648489	GAATCAGAAGATGACCGGGA	TTCTTCCAAAATGGACCAGC	60.0	187	90	expressed protein
OsISM10786	LOC_Os01g73620	42648683	CCGATGCTTACTTTCATCAGC	ACATGCCCATCTCAAGCTCT	59.8	178	83	expressed protein
OsISM10787	LOC_Os01g73620	42648893	TCAACTAACTACAGTGGGCCAT	CTTCGCTTTGCTCCAATGAC	59.8	220	86	expressed protein
OsISM10788	LOC_Os01g73620	42649669	CGAGCTGCACAGTATTGACC	TCCTCTCCATCATCCAGCTT	59.6	790	55	expressed protein
OsISM10789	LOC_Os01g73620	42650481	ACGGAGCTGCAGAAAAGACT	CGGATTGCTTTGTCCAACTT	59.7	768	75	expressed protein
OsISM10790	LOC_Os01g73620	42651229	AAGTTGGACAAAGCAATCCG	GCTTGTTCATTGCATGTTGC	60.2	156	71	expressed protein
OsISM10791	LOC_Os01g73620	42651365	GCAACATGCAATGAACAAGC	GCAGCTTTATTGAGACCACCC	60.6	476	94	expressed protein
OsISM10792	LOC_Os01g73620	42651967	TTTGCCAAAGAATGATGCAA	CCTTGGCTCAATGGACTTCT	59.7	185	91	expressed protein
OsISM10793	LOC_Os01g73620	42652099	TCTGCAGATAGGCCAAAGC	TGTTTTGATCCCTTTCTCGG	59.6	243	95	expressed protein
OsISM10795	LOC_Os01g73640	42659661	GTCGAAACTCCACAATCCGT	TGGCAGTGATTTGAATTGGA	60.0	170	94	expressed protein
OsISM10796	LOC_Os01g73640	42659864	CCTTGGCGCAACTTCTTATC	GCATCTCTCATTTCATGCCA	59.8	245	98	expressed protein
OsISM10797	LOC_Os01g73640	42660450	TCAAGCATTGGTGGCTATTG	GATGAGCCTTTGCCATGTTT	59.9	145	63	expressed protein
OsISM10798	LOC_Os01g73650	42667365	CACACACATCCCAGGTTCAG	TGGGGCATATTTACCTTCTTTG	60.1	274	72	hypothetical protein

ISM IDs	Rice gene locus IDs	Physical positions (bp) on chromosomes	Forward primers (5'-3')	Reverse primers (5'-3')	Melting tempearature (T˚C)	Expected product size (bp) in PCR	Expected product size (bp) in Q-PCR	Putative Functions
OsISM10799	LOC_Os01g73650	42667586	CATGACAGGCCTTACATCAAGA	CCTCCGCTCTCACCAATG	60.2	1996	63	hypothetical protein
OsISM10800	LOC_Os01g73650	42669646	AAAAAGGCCCTAATCCCAAC	TCGTGATCAGCCTTCATCTG	59.6	446	96	hypothetical protein
OsISM10801	LOC_Os01g73650	42670098	AGCGAGCAACAAGAGACTCC	CACCATCTTCTGCCTCATCA	59.8	430	92	hypothetical protein
OsISM10802	LOC_Os01g73656	42673697	TTGTCACAAACCAGAATGGC	TTTTTCTGCCCCTTGCTCT	59.7	265	60	expressed protein
OsISM10803	LOC_Os01g73656	42673943	AGAGCAAGGGGCAGAAAAA	CCCTGCAACTTCCTTGTTGT	60.0	171	88	expressed protein
OsISM10804	LOC_Os01g73656	42674367	TATGGTTGGGGAAAAGGTTG	CCACAGAGCCTCAGCATCTT	60.1	165	91	expressed protein
OsISM10805	LOC_Os01g73656	42674583	TGGAAATGAAATCATTGTGAAA	AACAGCCTGGCAGTCCTTAG	58.7	212	92	expressed protein
OsISM10806	LOC_Os01g73656	42674871	GCCTTGCTGAAGAACTTAGGG	AGGATGGCCTCCAATCTTCT	60.2	1112	72	expressed protein
OsISM10807	LOC_Os01g73656	42676198	TGTGCTGTGTGTAAGTGGAATG	CGGGGTTTCTCTCCATGAT	59.8	186	87	expressed protein
OsISM10808	LOC_Os01g73670	42685135	GCGCTATCGCGTCAGATACT	TCCCCAACAAGTTTCATGTG	60.0	229	88	expressed protein
OsISM10809	LOC_Os01g73670	42685451	TGATTACTGCTGGCTTGCTG	GCGGTAGTCGTCCCAATAAG	59.9	780	92	expressed protein
OsISM10810	LOC_Os01g73670	42686266	TCTCGACTTCTGCAAGGCAT	AAGATGGCAGTTTATTCAGGACA	60.6	164	72	expressed protein
OsISM10811	LOC_Os01g73670	42686382	TCTTGAGACAGTGGATGGAAGA	CTTGGTAGGCTCCTCTGCTG	60.0	165	85	expressed protein
OsISM10812	LOC_Os01g73670	42686582	GCTGCTTTGGCCTATAATGC	AAAAGCGCATGTGCTGTAAA	59.7	131	50	expressed protein
OsISM10813	LOC_Os01g73670	42686745	TACTTTGAGATTTGGCAGCG	TATCTTGCCGTGAAGGGAAC	59.6	193	98	expressed protein
OsISM10814	LOC_Os01g73670	42686921	CCCTTCACGGCAAGATAAAAT	TTCACTGGTAACACTTGCCG	60.0	192	95	expressed protein
OsISM10815	LOC_Os01g73670	42687086	GTTTGCACGGCAAGTGTTAC	TCAACTGCCTTTGCAATCAG	59.6	148	69	expressed protein
OsISM10816	LOC_Os01g73670	42687214	CTGATTGCAAAGGCAGTTGA	ATCAATCCAATAGCTTGGGC	59.5	415	96	expressed protein

ISM IDs	Rice gene locus IDs	Physical positions (bp) on chromosomes	Forward primers (5'-3')	Reverse primers (5'-3')	Melting tempearature (T˚C)	Expected product size (bp) in PCR	Expected product size (bp) in Q-PCR	Putative Functions
OsISM10817	LOC_Os01g73670	42687609	GCCCAAGCTATTGGATTGAT	AAGCTAAGCCCTCCCAAAAA	59.6	197	88	expressed protein
OsISM10818	LOC_Os01g73680	42689134	CCTGCTCGACTACCTCAACG	TCTCACTGAGCAGCGTGAAG	60.7	315	96	aspartyl aminopeptidase putative expressed
OsISM10819	LOC_Os01g73680	42689499	ATGTCCTGCTTGATTGCCTT	GAGACATGGGCTATCGGTGT	59.8	556	91	aspartyl aminopeptidase putative expressed
OsISM10820	LOC_Os01g73680	42690210	GCTTGTCAAAGTGAGCAGACC	CCAGATTAGGCTTGAACCCA	60.1	429	93	aspartyl aminopeptidase putative expressed
OsISM10821	LOC_Os01g73680	42690726	TTGTCCATCATCCACTGCTC	TTCACCTGATTTGCAACCAA	59.9	182	60	aspartyl aminopeptidase putative expressed
OsISM10822	LOC_Os01g73680	42691069	TGAGAAGGCTATAAGAATGATTGC	TAGCATGGAACATGGTTGGT	58.9	400	93	aspartyl aminopeptidase putative expressed
OsISM10823	LOC_Os01g73680	42691502	CTATGGGAGAGGGTGCTTTG	CTGGATAATTTGGGTGCAGG	60.0	176	88	aspartyl aminopeptidase putative expressed
OsISM10824	LOC_Os01g73680	42691763	TGTGACAGCTTTTCTGTTCAAA	TTGAACCACAGCCCATATCA	59.3	378	90	aspartyl aminopeptidase putative expressed
OsISM10825	LOC_Os01g73680	42692156	TTGCTTCTGGTGTTGGCATA	TCTTTGCCACACATTTCTCG	60.0	1916	83	aspartyl aminopeptidase putative expressed
OsISM10826	LOC_Os01g73720	42705861	AACCAATGCATCGTCATGTG	ACACGCCGGGGTAGAAGT	60.5	172	65	RCLEA4 - Root cap and Late embryogenesis related family protein precursor expressed
OsISM10827	LOC_Os01g73730	42710046	CTGCGCCTACTGCCTCAC	GGAAGTAGAAGGTGTTGCCG	60.2	190	99	RCLEA5 - Root cap and Late embryogenesis related family protein precursor expressed
OsISM10828	LOC_Os01g73750	42714888	CCTCTTCTCCGGATTCACC	GATTACTGACCTGAAGCTGCC	59.3	130	94	Leucine Rich Repeat family protein expressed
OsISM10829	LOC_Os01g73750	42714965	AGAGCTCCTCCTACTTCCGC	TTCTCAGCAGCCAGAAACAA	59.9	646	67	Leucine Rich Repeat family protein expressed
OsISM10830	LOC_Os01g73750	42715654	CAAAGTTCCTCGCTATTGGC	TCCTGGGCATATATCTTGAACA	59.6	202	97	Leucine Rich Repeat family protein expressed
OsISM10831	LOC_Os01g73750	42715847	TGCCCAGGATATTTAGCTCAG	AACGACCTCCTGGAGATAACC	59.4	755	55	Leucine Rich Repeat family protein expressed
OsISM10832	LOC_Os01g73750	42716554	CCACTGGGTGAATCCCATAC	TCAAGGAATCATACCTGCTGC	60.1	530	91	Leucine Rich Repeat family protein expressed
OsISM10833	LOC_Os01g73750	42717021	CCTGTTCTGCAGGTGAGGAT	CGAACTCAGCCCAGTTTCTC	60.1	192	74	Leucine Rich Repeat family protein expressed
OsISM10834	LOC_Os01g73750	42717257	TTGCGAACAGTATTGGATGC	TGAGAAGTGCAACGAAGTGC	59.9	465	98	Leucine Rich Repeat family protein expressed

ISM IDs	Rice gene locus IDs	Physical positions (bp) on chromosomes	Forward primers (5'-3')	Reverse primers (5'-3')	Melting tempearature (T°C)	Expected product size (bp) in PCR	Expected product size (bp) in Q-PCR	Putative Functions
OsISM10835	LOC_Os01g73750	42717816	TGTGGGTGCCTCATTGAATA	TCTGCATACACACACGCAAA	59.9	587	92	Leucine Rich Repeat family protein expressed
OsISM10836	LOC_Os01g73750	42718318	TTGATTTCTTTATAGTTATGGATTTGG	ACACTCATCCATGCTGTCCA	59.2	293	98	Leucine Rich Repeat family protein expressed
OsISM10837	LOC_Os01g73750	42718630	GAATGCAAGAATTGCACCTG	TTAGCTGAGACATAGCGGCA	59.5	234	61	Leucine Rich Repeat family protein expressed
OsISM10838	LOC_Os01g73750	42718951	TTGAGTTTCTTGATATTTCCGAG	CATCCAGCTGCTTTCAGACA	58.9	700	98	Leucine Rich Repeat family protein expressed
OsISM10839	LOC_Os01g73750	42719885	TTGTCCATTCCTAGAGTCTTTGG	GAAGCTTCGTTGAGGATTTCA	59.5	181	87	Leucine Rich Repeat family protein expressed
OsISM10840	LOC_Os01g73750	42720230	TCTGGTTGCAAACTCCTTGA	GCCACCCAGACGAGATTAAC	59.5	131	53	Leucine Rich Repeat family protein expressed
OsISM10841	LOC_Os01g73750	42720355	GGTGGCCAAACTTGACTTGT	TGAAACCTTGAAATTTTGGCTT	60.0	126	61	Leucine Rich Repeat family protein expressed
OsISM10842	LOC_Os01g73760	42722790	TCGATGTCCTCACCAACAAG	TCAATCACGCTCAGGAGATG	59.8	159	71	IPP transferase putative expressed
OsISM10843	LOC_Os01g73760	42722929	CATCTCCTGAGCGTGATTGA	CGCCACGATCCAATATACCT	59.9	242	95	IPP transferase putative expressed
OsISM10844	LOC_Os01g73760	42723151	AGGTATATTGGATCGTGGCG	TCATCAAAGAGGAATGGGCT	59.7	186	88	IPP transferase putative expressed
OsISM10845	LOC_Os01g73760	42723338	ATGGCACAGGATATTGAGGG	CATACAGACCGGCTTCATCA	59.7	195	83	IPP transferase putative expressed
OsISM10846	LOC_Os01g73760	42723568	AGGATACACCCGAACAACCA	TTGCCCTTGGAAAAGATCAC	60.1	225	91	IPP transferase putative expressed
OsISM10847	LOC_Os01g73760	42723773	GTGATCTTTTCCAAGGGCAA	TCAGCATCTAACCACAAGAAACA	59.9	274	86	IPP transferase putative expressed
OsISM10848	LOC_Os01g73760	42724326	CACTCGCAGACTTGTTCGAC	TGACGCAAGTTCCACTCAAA	60.0	149	73	IPP transferase putative expressed
OsISM10849	LOC_Os01g73760	42724455	TTTGAGTGGAACTTGCGTCA	GTTCCATGAGTCAGCAGTGG	59.8	167	67	IPP transferase putative expressed
OsISM10850	LOC_Os01g73760	42724711	TGGAAGCCCTAGGATGTCTT	GTGTTGCTCCCACTCATGC	59.5	242	98	IPP transferase putative expressed
OsISM10852	LOC_Os01g73820	42760692	ACGTGATGAGAATGGAAGGG	TGCGAGCAGGTACAATAGCA	60.2	761	65	hypothetical protein
OsISM10853	LOC_Os01g73820	42761493	CCATTAGCGGAGATAGCTGG	GGTTCATGCCAAACTTCGTT	59.9	924	50	hypothetical protein

ISM IDs	Rice gene locus IDs	Physical positions (bp) on chromosomes	Forward primers (5'-3')	Reverse primers (5'-3')	Melting tempearature (T°C)	Expected product size (bp) in PCR	Expected product size (bp) in Q-PCR	Putative Functions
OsISM10854	LOC_Os01g73830	42770295	AGCTGCATGAAGCCACCTAT	AAAGAAGGGTCCCATCAACC	60.0	204	60	expressed protein
OsISM10855	LOC_Os01g73830	42770543	TTCACATATCAGCAGTCGCC	AAGACAATCCAGAGCCTTGC	59.6	241	74	expressed protein
OsISM10856	LOC_Os01g73830	42770830	ATGTTCGAGTGAAGGGTGGA	GCAGTAGTATTTCTTGATGTTGCC	59.9	192	80	expressed protein
OsISM10857	LOC_Os01g73830	42771393	ACCTGGTCAAACATTGGCTC	CGGTGTCCACTTCTCCATCT	60.0	185	93	expressed protein
OsISM10858	LOC_Os01g73830	42771566	AAGTGGACACCGAGGATTTG	TGCTGCTGCAACTCAGAAAT	59.9	297	87	expressed protein
OsISM10859	LOC_Os01g73870	42806463	GTCCATCTGGACAGGCAAGT	GTGGTTCCTGTCAATCCCAC	60.2	452	75	transposon protein putative unclassified
OsISM10861	LOC_Os01g73870	42807395	ACCACCCTTCCTCCGTACTC	CCGAGTAATCATCAGCGTCA	60.1	181	95	transposon protein putative unclassified
OsISM10862	LOC_Os01g73870	42807551	CACCCTGACGCTGATGATTA	CCGTGGGTGCTAGTGAACAT	60.3	92	45	transposon protein putative unclassified
OsISM10863	LOC_Os01g73870	42807920	ACGATGGGGACTTACTGGTG	GGAGACGGTCAAACTCGAAC	59.8	189	95	transposon protein putative unclassified
OsISM10864	LOC_Os01g73870	42808281	CGGACTTAGGCAGGTGTTTC	CTAGCTGTCCCCGTCGAGT	60.1	159	81	transposon protein putative unclassified
OsISM10865	LOC_Os01g73870	42808648	GCACCGTGACCGAGACTACT	ATATCGGCACTGATATCCCG	60.0	152	58	transposon protein putative unclassified
OsISM10866	LOC_Os01g73870	42808984	GCAGGACCTCGTTTGTCTTC	TACGAACGCGCTCGAATG	61.0	189	97	transposon protein putative unclassified
OsISM10867	LOC_Os01g73870	42809229	GTTCGCCAGCTCTTCGTC	AGGCCCCAGTACCGAAGAT	60.3	143	81	transposon protein putative unclassified
OsISM10868	LOC_Os01g73870	42809566	TACTCCCAGGGGCACATTAG	GCCGAAAGTGTGTTGTTGTG	60.1	235	98	transposon protein putative unclassified
OsISM10869	LOC_Os01g73880	42811223	ATCCACACCTTCTCCACCGT	GTCAGCTCCCACAACCAACT	61.0	1599	82	eukaryotic translation initiation factor putative expressed
OsISM10870	LOC_Os01g73880	42812897	AGCTGTGGCAGAGGAAAGTC	TCCACAAATTTCATCCCCAT	59.8	176	91	eukaryotic translation initiation factor putative expressed
OsISM10871	LOC_Os01g73880	42813121	AAAAATGCTGCCAATGAAGC	ATTCTTTCCACTGCTTCCCA	59.9	647	53	eukaryotic translation initiation factor putative expressed
OsISM10872	LOC_Os01g73880	42813781	GGACTCGATTGGGTTCATTG	AGCGGTTCTTGAGACCTTTG	59.9	142	63	eukaryotic translation initiation factor putative expressed

ISM IDs	Rice gene locus IDs	Physical positions (bp) on chromosomes	Forward primers (5'-3')	Reverse primers (5'-3')	Melting tempearature (T˚C)	Expected product size (bp) in PCR	Expected product size (bp) in Q-PCR	Putative Functions
OsISM10873	LOC_Os01g73890	42818531	CAAGTCCTCGTGCAGTTTGA	GATGCTCCAAAGCGCTAGTC	60.1	135	47	transcription initiation factor IIA gamma chain putative expressed
OsISM10874	LOC_Os01g73890	42818646	GACTAGCGCTTTGGAGCATC	AAAGTCCACACATTGTCGCA	60.1	991	88	transcription initiation factor IIA gamma chain putative expressed
OsISM10875	LOC_Os01g73960	42844880	GCTATGGCTTCCTTCCTTCA	AAGTACTCCCACAATTCGCC	59.2	715	86	drought induced 19 protein putative expressed
OsISM10876	LOC_Os01g73960	42845629	CCCTTCATCTGCAACCATCT	CTCTCCCTATATTGTCGGCG	59.9	655	86	drought induced 19 protein putative expressed
OsISM10877	LOC_Os01g73960	42846264	CGCCGACAATATAGGGAGAG	CTGGGCCTAGAAGGTTTCCT	59.7	188	85	drought induced 19 protein putative expressed
OsISM10878	LOC_Os01g73960	42846623	TGCAGTTTCAGCAGTCTCACA	CGATTCTTTGCAACCTTTCC	60.2	202	99	drought induced 19 protein putative expressed
OsISM10879	LOC_Os01g73980	42855507	AGGCCTCAGGCAGGAACT	GTATCCAACGGCCGTCAC	59.9	459	93	xylem cysteine proteinase 2 precursor putative expressed
OsISM10880	LOC_Os01g73990	42857970	CACAACACCAAGGACGATTG	AGACATCATCACCTCCAGGG	60.0	859	92	cytochrome b5-like Heme/Steroid binding domain containing protein expressed
OsISM10881	LOC_Os01g73990	42858809	CCCTGGAGGTGATGATGTCT	ACATCTTCGAAGTCGTCGGT	59.8	156	64	cytochrome b5-like Heme/Steroid binding domain containing protein expressed
OsISM10882	LOC_Os01g74080	42896960	ATCCGCTGCTCAGACAGG	TTTTCTTTCTTCTTCCGAGGC	60.0	1025	88	retrotransposon protein putative unclassified
OsISM10883	LOC_Os01g74140	42946116	GTCACAATTTCGCTGCAAGT	TCGTCTTGCTCAACGTCTTC	59.0	269	67	OsWRKY17 - Superfamily of TFs having WRKY and zinc finger domains expressed
OsISM10884	LOC_Os01g74140	42946436	TCGCAGATGATGGGTACAAA	GATTCGTGCACCGGTAGTAG	59.2	474	88	OsWRKY17 - Superfamily of TFs having WRKY and zinc finger domains expressed
OsISM10885	LOC_Os01g74146	42948971	CCAGGAAGGTCCCCAAGAC	CGACCAGTCGACAAGATTGA	60.8	194	84	WD repeat-containing protein putative expressed
OsISM10886	LOC_Os01g74146	42949288	CGAGAAGGCTCCTATCTTGC	GGTGTCCTCCCATGTTCCTA	59.5	560	95	WD repeat-containing protein putative expressed
OsISM10887	LOC_Os01g74146	42949952	AGCAAGTTCTCAGGGCACAG	TTATCATTTCCACCGGATGC	60.6	220	84	WD repeat-containing protein putative expressed
OsISM10888	LOC_Os01g74146	42950346	GAATTCAGTGGACACAGGCA	CCATGAGTGCTCACAAGCTC	59.6	159	76	WD repeat-containing protein putative expressed
OsISM10889	LOC_Os01g74146	42950502	TGGGTATTCCCAAAACCAAA	GCAGCGTGTGTCCAGTTAGA	60.0	501	78	WD repeat-containing protein putative expressed
OsISM10890	LOC_Os01g74146	42950983	TCTAACTGGACACACGCTGC	AATCTGAGGGTTTCATCCCC	60.1	179	91	WD repeat-containing protein putative expressed

ISM IDs	Rice gene locus IDs	Physical positions (bp) on chromosomes	Forward primers (5'-3')	Reverse primers (5'-3')	Melting tempearature (T°C)	Expected product size (bp) in PCR	Expected product size (bp) in Q-PCR	Putative Functions
OsISM10891	LOC_Os01g74146	42951142	GGGGATGAAACCCTCAGATT	AGCCCAATATCACGAACAGG	60.0	150	72	WD repeat-containing protein putative expressed
OsISM10892	LOC_Os01g74152	42953624	CTACAGCAAGGAGCAGAGGC	CGGATACAGCGTGTCATCAA	60.5	446	65	haloacid dehalogenase-like hydrolase family protein putative expressed
OsISM10893	LOC_Os01g74152	42954089	CCGACGTCGTAAGGAACATC	CTGCTTGTAGAGGAGGACGC	60.3	180	93	haloacid dehalogenase-like hydrolase family protein putative expressed
OsISM10894	LOC_Os01g74152	42954249	GCGTCCTCCTCTACAAGCAG	GCCAATACCTGTGGAAGTCG	60.3	215	91	haloacid dehalogenase-like hydrolase family protein putative expressed
OsISM10895	LOC_Os01g74152	42954415	CAGGCTGTTGGGTACCAGTT	AAGCCAACCTTCCATGAACA	60.3	135	62	haloacid dehalogenase-like hydrolase family protein putative expressed
OsISM10896	LOC_Os01g74152	42954587	TCTCCTAAGCTTGCCAATCC	TTGAAGCATGAATTCTGTCACC	59.8	146	60	haloacid dehalogenase-like hydrolase family protein putative expressed
OsISM10897	LOC_Os01g74152	42954933	CAAGCTCTCAAGGTTGCCTC	CTATTAGCTTCGCAGCCTGG	60.1	184	86	haloacid dehalogenase-like hydrolase family protein putative expressed
OsISM10898	LOC_Os01g74152	42955098	CAGGCTGCGAAGCTAATAGG	GCATGGTCAGCACCCTTTAT	60.0	162	72	haloacid dehalogenase-like hydrolase family protein putative expressed
OsISM10899	LOC_Os01g74160	42959259	GCCTCAACAAGCCTCCTCT	GAGGAGAGGATGATCGAACG	59.7	232	99	carboxyl-terminal peptidase putative expressed
OsISM10900	LOC_Os01g74160	42959453	ATATCTCCAAACAGCCTGCG	GCCACTTGGCTGGATAGAAG	60.0	211	81	carboxyl-terminal peptidase putative expressed
OsISM10901	LOC_Os01g74160	42959828	CTTGGCTGGTGTCACAGATG	TGGTTGCCACAAATTGATTG	60.3	174	98	carboxyl-terminal peptidase putative expressed
OsISM10902	LOC_Os01g74160	42960051	CGGGCTCCTATCAAAACAAG	GCACTTGCAACCACCAATTA	59.6	3270	82	carboxyl-terminal peptidase putative expressed
OsISM10903	LOC_Os01g74180	42969547	GATGTCGCCCACTTCTTCC	GACGAGCTTCTTGACCTCCA	60.6	161	70	adaptin putative expressed
OsISM10904	LOC_Os01g74180	42969687	CTGGAGGTCAAGAAGCTCGT	TTGAAAGAAGAGCTTCATTTTGG	59.7	508	74	adaptin putative expressed
OsISM10905	LOC_Os01g74180	42970362	GCACTTTGTAAGCTGCACGA	CTACTCCCGGGGAATTGTC	59.8	183	92	adaptin putative expressed
OsISM10906	LOC_Os01g74180	42971045	CTACGCTCATCTCCTGATGC	AACAATTGTGGGGCTGTCTT	59.0	598	75	adaptin putative expressed
OsISM10907	LOC_Os01g74180	42971720	CCACCATAGCCACAGAGTCC	AGCAGCAACTGTATCTGCCA	60.1	443	99	adaptin putative expressed
OsISM10908	LOC_Os01g74180	42972195	CACTACCAGTTGCCTTGGTG	AACAGCAGCTTCTCCATCAAA	59.6	169	89	adaptin putative expressed

ISM IDs	Rice gene locus IDs	Physical positions (bp) on chromosomes	Forward primers (5'-3')	Reverse primers (5'-3')	Melting tempearature (T˚C)	Expected product size (bp) in PCR	Expected product size (bp) in Q-PCR	Putative Functions
OsISM10909	LOC_Os01g74180	42972399	GAGAACAGATCCAGCATCTCA	TGCTGCAGGCTCCTTAATTT	59.0	957	77	adaptin putative expressed
OsISM10910	LOC_Os01g74180	42973465	TGCTTGAGACAAAGCTTCAGA	CATGGGCCATTATCTTTTTGA	59.4	190	97	adaptin putative expressed
OsISM10911	LOC_Os01g74180	42974521	GGTAGTGAGGATGGCTTGGA	GTTTGTTCGGGAGCCAATAA	60.0	241	96	adaptin putative expressed
OsISM10912	LOC_Os01g74180	42974914	GCAAGGAAAACCAACTGAGG	CGTTGTGCTCAAGCTTCTGA	60.0	485	82	adaptin putative expressed
OsISM10913	LOC_Os01g74220	42993725	CCCTCCAAAGTGGTCGTTTA	ACTGGCCGGACTTGGAAT	60.2	177	78	retrotransposon protein putative unclassified
OsISM10914	LOC_Os01g74250	43016247	ACCACCTCGCCGATAACAT	GGACCATGGAGAGTAGCTCG	60.1	606	73	plant-specific domain TIGR01615 family protein expressed
OsISM10915	LOC_Os01g74250	43016991	GGCAGGGATTCGACAAGATA	GATATCAATGATCAGACGATATT CAG	59.1	199	90	plant-specific domain TIGR01615 family protein expressed
OsISM10916	LOC_Os01g74260	43028098	GTCAAAAGTTTGGCTGAGCG	AAACCTAGGTCATCATCGCC	60.0	161	87	retrotransposon protein putative unclassified
OsISM10917	LOC_Os01g74260	43028949	GAGGCAGCGGATAGTCACAT	CTCCATGCGAAGACGTCAG	60.4	159	85	retrotransposon protein putative unclassified
OsISM10918	LOC_Os01g74260	43031191	CTCCTGGTGTTAGGCGACTC	TCACTTCCTTCGTGGTCCTC	60.1	415	90	retrotransposon protein putative unclassified
OsISM10919	LOC_Os01g74280	43039599	CTCTTCCCTCAAGAGCATCG	CACCTCAACCACTGAATTGC	59.6	1101	80	TGD2 putative expressed
OsISM10920	LOC_Os01g74280	43040957	AGAAGCGCAGCCTCTTCTTT	CGACATCCTTCACCAGATCA	60.2	924	96	TGD2 putative expressed
OsISM10921	LOC_Os01g74280	43041889	GCTAAAGGTCTGGCTGATGC	GATCGGTGTTCTCAGGGGTA	60.0	339	74	TGD2 putative expressed
OsISM10922	LOC_Os01g74280	43042210	CCCCTGAGAACACCGATCT	ACCGGTGAAACCAGAGATGT	59.7	211	99	TGD2 putative expressed
OsISM10923	LOC_Os01g74300	43046205	ATCACCATGTCGTGCTGC	GTGGGTTCCACCTCAGAGTA	58.4	162	96	metallothionein putative expressed
OsISM10924	LOC_Os01g74300	43046367	GACCACGACCACCTTCCTT	GCCCCCATCTCTGATCCT	60.0	185	73	metallothionein putative expressed
OsISM10925	LOC_Os01g74340	43070583	GTGCCTCTTCCTCCTCCTG	GCCATGCTGAGAGAAAGCAT	60.2	247	96	RNA recognition motif containing protein putative expressed
OsISM10926	LOC_Os01g74340	43070810	ATGCTTTCTCTCAGCATGGC	ACCCTTTGGATTTCCCTGTC	60.3	178	73	RNA recognition motif containing protein putative expressed

ISM IDs	Rice gene locus IDs	Physical positions (bp) on chromosomes	Forward primers (5'-3')	Reverse primers (5'-3')	Melting tempearature (T˚C)	Expected product size (bp) in PCR	Expected product size (bp) in Q-PCR	Putative Functions
OsISM10927	LOC_Os01g74340	43071004	TGCCTCAGAAGATGAAGCTG	CATCCGCTGTTTGCATAGTG	59.8	202	94	RNA recognition motif containing protein putative expressed
OsISM10928	LOC_Os01g74410	43095631	GTGCAGTTGGTTTGGTTCGT	GAGACGGCAGCTCTTCCC	61.0	310	97	MYB family transcription factor putative expressed
OsISM10930	LOC_Os01g74500	43142876	GCAAGTCTGGAACCCACATT	GGGCTCCGATGCAAATAAT	59.9	181	81	conserved hypothetical protein
OsISM10931	LOC_Os01g74500	43143063	GAGGGGGACTGTATAATGCAA	TCTCCAACAGCATGGTCAAG	59.4	199	96	conserved hypothetical protein
OsISM10932	LOC_Os01g74500	43143286	CATCATGGAGCAACACTTGG	AATTGAGCTGTTTTGGACCG	60.1	356	71	conserved hypothetical protein
OsISM10933	LOC_Os01g74500	43143647	TGAAGCCATTGAACACAAGG	CAAATGTGGGAAATGGTCAA	59.5	177	77	conserved hypothetical protein
OsISM10934	LOC_Os01g74500	43143843	GGGATTTCATGCATGCTTCT	CAAAACAGAACCAAAAACACCA	60.0	812	63	conserved hypothetical protein
OsISM10935	LOC_Os01g74500	43144627	CCATGATGGTGTTTTTGGTTC	CACCTGTTCACCAACAGCAT	59.8	235	95	conserved hypothetical protein
OsISM10936	LOC_Os01g74500	43144818	TGCAGATCATTGCTGACAGA	TCCAAAGTTGAGTGCCAGTG	59.5	289	93	conserved hypothetical protein
OsISM10937	LOC_Os01g74500	43145087	CACTGGCACTCAACTTTGGA	AGTGGATCTTGGACTGGCAT	59.7	419	86	conserved hypothetical protein
OsISM10939	LOC_Os01g74510	43153216	AAGCAGTCCAAATGGCTTGA	GGAATCAGCTTCCTCTCCAA	60.1	821	88	KIP1 putative expressed
OsISM10940	LOC_Os01g74570	43185086	ACTGCCACATCCTCCAAGAC	CTATCTTCGTCCATGCCACC	60.3	183	82	tetraspanin family protein putative expressed
OsISM10941	LOC_Os01g74580	43188204	CTTCTCCTTCACTCCTCCCC	TTCTGAGGCTCGTTTGAACA	59.9	494	79	dnaJ homolog subfamily C member putative expressed
OsISM10942	LOC_Os01g74580	43188747	TGTTGGAGGTTGACCCAAAT	ATAGTCACCCTCCGCTTCCT	60.2	370	93	dnaJ homolog subfamily C member putative expressed
OsISM10943	LOC_Os01g74580	43189253	TCAACAAAATTGTGCTTGTTTT T	GCAATGCTTTAGCCTTGAGAA	59.2	162	67	dnaJ homolog subfamily C member putative expressed
OsISM10944	LOC_Os01g74580	43189521	CCTCGCTGATCATGATGTTG	TTCAGGGTCTAGACGAAGGC	59.8	190	59	dnaJ homolog subfamily C member putative expressed
OsISM10945	LOC_Os01g74580	43189734	TTTTGGGTTGAAAAATCTTCTG A	TAGCTTACCCTTGGCAGCAT	59.9	144	65	dnaJ homolog subfamily C member putative expressed
OsISM10946	LOC_Os01g74580	43190014	CAGAAGCACTCAACATAGATG GA	TAGATCCTGAACCGCTCCTT	59.1	586	99	dnaJ homolog subfamily C member putative expressed

ISM IDs	Rice gene locus IDs	Physical positions (bp) on chromosomes	Forward primers (5'-3')	Reverse primers (5'-3')	Melting tempearature (T°C)	Expected product size (bp) in PCR	Expected product size (bp) in Q-PCR	Putative Functions
OsISM10947	LOC_Os01g74580	43190579	GAAGGAGCGGTTCAGGATCT	TTAGTGCTTCTCGGATTCCC	60.0	961	71	dnaJ homolog subfamily C member putative expressed
OsISM10948	LOC_Os01g74580	43191685	CCGAGAAGAAGCAGAAAACA	AAGATCCTCTCCTCGGTCGT	59.2	422	98	dnaJ homolog subfamily C member putative expressed
OsISM10949	LOC_Os01g74640	43225305	GTCTTCTTCAGCAGCGCCT	GCTGTGTCGAGAGGAATGGT	60.6	291	63	mitochondrial carrier protein putative expressed
OsISM10950	LOC_Os01g74640	43225764	ACTCCGCATCGCCTTGTAT	AACTGACACCACCAGCAACA	60.4	193	75	mitochondrial carrier protein putative expressed
OsISM10951	LOC_Os01g74640	43225936	CTGTTGCTGGTGGTGTCAGT	TCAGTTGGATTTGCCACAAC	59.7	279	86	mitochondrial carrier protein putative expressed
OsISM10952	LOC_Os01g74640	43226270	TATTCTGGAGCCCTCAATGC	AGCATTGCGTGCTATGTTTG	60.0	184	94	mitochondrial carrier protein putative expressed
OsISM10953	LOC_Os01g74640	43226475	GCCAGCTACGACCAACTCA	AAATCCAGCACCAAGTCCAG	60.1	179	94	mitochondrial carrier protein putative expressed
OsISM10954	LOC_Os01g74640	43226634	CTGGACTTGGTGCTGGATTT	CTCTTGTACGTTGCGTCACC	59.7	192	89	mitochondrial carrier protein putative expressed
OsISM10955	LOC_Os01g74640	43226806	GGTGACGCAACGTACAAGAG	GGCAAAAGTTTGCAATGAAC	58.8	1841	98	mitochondrial carrier protein putative expressed
OsISM10956	LOC_Os01g74640	43228648	CATTGGGTCATGGAATGTGA	CAGTGCAGTGCAGCAGACTT	60.3	260	80	mitochondrial carrier protein putative expressed
OsISM10957	LOC_Os01g74680	43258906	GATGATCACAGGCGACGAG	ATGCGAAGACGTCAGCTTTT	60.2	146	72	retrotransposon protein putative unclassified expressed
OsISM10958	LOC_Os01g74680	43259775	TACGGGCTATAGAGCGGATG	GATACGCCTTCAACTGGGTG	60.4	197	94	retrotransposon protein putative unclassified expressed
OsISM10959	LOC_Os01g74680	43260463	CCCCTGTTATCCTCCGACC	GATCCTCTTCGCTAGGGGC	61.4	109	56	retrotransposon protein putative unclassified expressed
OsISM10961	LOC_Os02g01030	14516	TGTCGAATCTGACCATCAGG	TGTGAGGCATCACGATTTTC	59.6	154	82	tetratricopeptide repeat domain containing protein expressed
OsISM10962	LOC_Os02g01030	14677	AAAAGCAAGGCCATGGAAG	AGCTGAGAGTGGATTGAGCAG	60.0	192	91	tetratricopeptide repeat domain containing protein expressed
OsISM10963	LOC_Os02g01030	14839	AAGGCAATACTGCTCAATCCA	GCAGCAACAGGTTTCTTCATT	59.5	178	84	tetratricopeptide repeat domain containing protein expressed
OsISM10964	LOC_Os02g01030	15000	AAGAAACCTGTTGCTGCCAT	GCATAAGCCATTCCACGAGT	59.9	407	93	tetratricopeptide repeat domain containing protein expressed
OsISM10965	LOC_Os02g01030	15446	CACAGCATCGAACATGGATT	ATTTCCTGCGATGTTCCATT	59.5	175	93	tetratricopeptide repeat domain containing protein expressed

ISM IDs	Rice gene locus IDs	Physical positions (bp) on chromosomes	Forward primers (5'-3')	Reverse primers (5'-3')	Melting tempearature (T˚C)	Expected product size (bp) in PCR	Expected product size (bp) in Q-PCR	Putative Functions
OsISM10966	LOC_Os02g01030	15649	AAAGAGAGCTGAGCGTGACC	GCGACTTGATGATTGCTCCT	60.1	391	89	tetratricopeptide repeat domain containing protein expressed
OsISM10967	LOC_Os02g01030	16185	GTGCTATGCCTGGAGGTGTT	AACCTCAGGGTCACCAAATG	60.0	180	90	tetratricopeptide repeat domain containing protein expressed
OsISM10968	LOC_Os02g01030	16322	ACAGGACCCTGATCTGATGG	AAAGCTGGCAGGATTGTTCA	60.3	1828	83	tetratricopeptide repeat domain containing protein expressed
OsISM10969	LOC_Os02g01050	27750	CTTCACCAACGCAGCAGAC	CGTCTGCAAGCCCTTTTATC	60.2	159	93	retrotransposon protein putative unclassified
OsISM10970	LOC_Os02g01050	28218	ACAACAACAATCGTCCGACA	GACGAGGCTGTGACATTGG	60.1	125	92	retrotransposon protein putative unclassified
OsISM10971	LOC_Os02g01050	28422	AAGGACAAATATGCCGCAAC	CTGAACCACCAATCTGAGCA	59.9	1406	86	retrotransposon protein putative unclassified
OsISM10972	LOC_Os02g01070	34473	CCTTCGCCGTCTTCTTCTC	GTCGTGCTTGATGGGGTAGT	60.0	177	96	FG-GAP repeat-containing protein putative expressed
OsISM10973	LOC_Os02g01070	35095	GAGGAAGGATGGAAATGCAA	TCTACGGTGCTCTTCCCTGT	59.9	531	84	FG-GAP repeat-containing protein putative expressed
OsISM10974	LOC_Os02g01070	35606	ACAGGGAAGAGCACCGTAGA	GGTCTGTGTTGCCTGTCTCA	59.9	242	61	FG-GAP repeat-containing protein putative expressed
OsISM10975	LOC_Os02g01070	35857	TTGCCCTTTATGCTTTTGCT	GGTATCAGCACTGAAGCATCG	60.3	270	98	FG-GAP repeat-containing protein putative expressed
OsISM10976	LOC_Os02g01070	36148	TGCCCATGCCCTTAATAGTC	GCATGACCCCAAGAACTGAT	59.9	604	75	FG-GAP repeat-containing protein putative expressed
OsISM10977	LOC_Os02g01070	36732	ATCAGTTCTTGGGGTCATGC	ATGGGCAAGTTGCAGAAAAG	60.1	160	65	FG-GAP repeat-containing protein putative expressed
OsISM10978	LOC_Os02g01070	37009	AATGTGATTGGGAAAGCTGC	TCGGAACATAAAGCGTCCTT	59.9	1593	74	FG-GAP repeat-containing protein putative expressed
OsISM10979	LOC_Os02g01070	38636	TAATGTTGTTGTTGCCCACG	CATGAAGGCCTCCTTCTGTTA	59.9	434	99	FG-GAP repeat-containing protein putative expressed
OsISM10980	LOC_Os02g01070	39056	AGGAGGCCTTCATGCAGATA	ACTGTTTGCTCGATGCCATT	60.2	861	82	FG-GAP repeat-containing protein putative expressed
OsISM10981	LOC_Os02g01070	40145	AAGCCACGGGGATATCATCT	GCCATCTCCATATTGCATCA	60.1	239	90	FG-GAP repeat-containing protein putative expressed
OsISM10982	LOC_Os02g01110	59471	ATCCCCTCGTCCCTCCAC	TCCTTGGTGCTCCTCTTCAT	61.0	184	74	translation initiation factor IF-3 putative expressed
OsISM10983	LOC_Os02g01110	59681	ACAACGACATCACCTCTCCC	GTGCCTGGGGACAACACTAT	59.9	139	66	translation initiation factor IF-3 putative expressed

ISM IDs	Rice gene locus IDs	Physical positions (bp) on chromosomes	Forward primers (5'-3')	Reverse primers (5'-3')	Melting tempearature (T˚C)	Expected product size (bp) in PCR	Expected product size (bp) in Q-PCR	Putative Functions
OsISM10984	LOC_Os02g01110	59791	TTGCAGGCCATAGTGTTGTC	GACAGGAGGGTCTGATTTTCTG	59.9	381	99	translation initiation factor IF-3 putative expressed
OsISM10985	LOC_Os02g01110	60213	CGTTAAGGAGAAAGAACGCC	GCACTTCTTTGTTCTCCCCA	59.6	365	69	translation initiation factor IF-3 putative expressed
OsISM10986	LOC_Os02g01110	60540	TGCTATTGTCTTACGCGGTG	TCCATCTGCTTTCACCTTCA	59.6	436	86	translation initiation factor IF-3 putative expressed
OsISM10987	LOC_Os02g01110	60980	CCAGATTAATGGAGCGAGGT	CGAGATAGTGGCCCTCCTAA	59.2	2237	86	translation initiation factor IF-3 putative expressed
OsISM10988	LOC_Os02g01110	63197	TTAGGAGGGCCACTATCTCG	GTATGCATGCTTCGAGTCCA	59.6	232	88	translation initiation factor IF-3 putative expressed
OsISM10989	LOC_Os02g01140	78464	GACGCATACCAACCGACTTC	AGCTCCTCAGTCGACAGCTC	60.2	197	89	GDSL-like lipase/acylhydrolase putative expressed
OsISM10990	LOC_Os02g01190	110957	CCTCTTCGACTACCCGCTC	CAGTTGGTGTTGCACTCCC	60.1	365	91	POEI25 - Pollen Ole e I allergen and extensin family protein precursor expressed
OsISM10992	LOC_Os02g01210	119708	CAAGGATTCATGATGGCGTC	CGTCATCTCCATCTCCACCT	60.7	194	84	amino acid transporter putative expressed
OsISM10993	LOC_Os02g01210	120192	CGGCAGAAGGAACTACACCT	TACTGAATGCATCCGCAAAC	59.5	176	82	amino acid transporter putative expressed
OsISM10994	LOC_Os02g01210	120353	CGGATGCATTCAGTACCTCA	AGCAGTTGGAACGCTGGAT	60.2	188	93	amino acid transporter putative expressed
OsISM10995	LOC_Os02g01220	124841	GTAGAGAGCAGGGCTAGGCT	CCACATCCACCGTTACCAC	58.8	236	98	rhodanese-like domain containing protein putative expressed
OsISM10996	LOC_Os02g01220	125085	AGCGACCTTATCACCTCGG	GCCCTTGTTCATTTCCTCCT	60.3	319	67	rhodanese-like domain containing protein putative expressed
OsISM10997	LOC_Os02g01220	125399	AGGGCCATCTACACAACTCC	CGAGAACTGCTCCACAAACA	59.5	489	90	rhodanese-like domain containing protein putative expressed
OsISM10998	LOC_Os02g01220	125868	TGTTTGTGGAGCAGTTCTCG	CATGCTAGCTCCGACCTCTT	59.8	205	86	rhodanese-like domain containing protein putative expressed
OsISM10999	LOC_Os02g01220	126027	CCAACTATTAGGGGTGCCAA	CCTCCCATGTTCTTCACGTT	59.9	818	92	rhodanese-like domain containing protein putative expressed
OsISM11000	LOC_Os02g01240	133545	TGGAACCAGCTCCACGTC	TTGACATCCATGCCATCATC	60.6	426	78	CPuORF11 - conserved peptide uORF-containing transcript expressed
OsISM11001	LOC_Os02g01240	134084	CTCGCACAAAAGGAGCTTGT	TTGTTCCTTTCCTTAGCAGCA	60.3	615	59	CPuORF11 - conserved peptide uORF-containing transcript expressed
OsISM11002	LOC_Os02g01245	133545	TGGAACCAGCTCCACGTC	TTGACATCCATGCCATCATC	60.6	426	78	hypothetical protein

ISM IDs	Rice gene locus IDs	Physical positions (bp) on chromosomes	Forward primers (5'-3')	Reverse primers (5'-3')	Melting tempearature (T°C)	Expected product size (bp) in PCR	Expected product size (bp) in Q-PCR	Putative Functions
OsISM11003	LOC_Os02g01245	134084	CTCGCACAAAAGGAGCTTGT	TTGTTCCTTTCCTTAGCAGCA	60.3	615	59	hypothetical protein
OsISM11004	LOC_Os02g01270	146941	GCTACTTCGTCCTCGACAGC	CCGTCTATCAGCAGCGATTT	60.3	1050	89	START domain containing protein expressed
OsISM11005	LOC_Os02g01270	147991	GAATTGCAGGGTGGAGGATA	CCGTGATCTGATGCTCCTTT	60.1	245	99	START domain containing protein expressed
OsISM11006	LOC_Os02g01270	148182	TGGTCTATGTCCTGTGCGTT	AGGCCAGAGCATCCTCAATA	59.5	175	85	START domain containing protein expressed
OsISM11007	LOC_Os02g01270	148337	TATTGAGGATGCTCTGGCCT	GCGGTTTTTGGAGGTCATATT	60.0	429	77	START domain containing protein expressed
OsISM11008	LOC_Os02g01270	148793	ACGGAACTAGGAGGGCAGTT	AGTTGTCCTGCGAATCAACA	59.7	190	88	START domain containing protein expressed
OsISM11009	LOC_Os02g01270	148956	CGACCTATGTTGATTCGCAG	CATGCTCTTTGGTCCAGTCA	59.6	165	74	START domain containing protein expressed
OsISM11010	LOC_Os02g01270	149146	CCCCATTCAAGTTAGCTCCA	TTCCAGAAGTTCTTCAAAGATGC	60.0	167	80	START domain containing protein expressed
OsISM11011	LOC_Os02g01270	149276	ATTCGCAGGACTACGCATCT	TTCCACAACACCAACAGCTC	59.8	196	92	START domain containing protein expressed
OsISM11012	LOC_Os02g01270	149487	ATTTTTGGGCTGGTGATGAG	CATAGCGAAAGCTGCAGTCC	60.5	165	62	START domain containing protein expressed
OsISM11013	LOC_Os02g01270	149673	ACGGCCACACCGCTATACT	CGCCAATAGCGAACATAACA	60.1	274	92	START domain containing protein expressed
OsISM11014	LOC_Os02g01270	149946	GGCGTAATGACGATGGAAGT	TCCACAGTTTGGATGCTCTG	59.9	140	60	START domain containing protein expressed
OsISM11015	LOC_Os02g01270	150077	AACTGTGGACGTCAAAAAGGA	TGCGACACTTGAGAGGAGAA	59.7	238	77	START domain containing protein expressed
OsISM11016	LOC_Os02g01270	150407	TTGCTGCAGATACAAAACTGTG	TGTGTTTCGTCTGTTTGGGA	59.6	167	63	START domain containing protein expressed
OsISM11017	LOC_Os02g01270	150731	GACGAGGATGAGGACTATCAGG	ACCTGCACGCTTAGCATCTT	60.1	176	73	START domain containing protein expressed
OsISM11018	LOC_Os02g01270	150867	TGTGCAGGAAGATCCTAACAA	ATGCCCGAAAAGCATGATAG	59.2	196	79	START domain containing protein expressed
OsISM11019	LOC_Os02g01270	151134	GTAGCAAGAACTTCCCGCAT	ATCCATACGCTTGCTGTCCT	59.5	199	99	START domain containing protein expressed
OsISM11020	LOC_Os02g01270	151329	GGATAACGTAGGCAGGCAGA	ATGCATCCCTTTCTCAGCAG	60.3	164	59	START domain containing protein expressed

ISM IDs	Rice gene locus IDs	Physical positions (bp) on chromosomes	Forward primers (5'-3')	Reverse primers (5'-3')	Melting tempearature (T°C)	Expected product size (bp) in PCR	Expected product size (bp) in Q-PCR	Putative Functions
OsISM11021	LOC_Os02g01270	151471	AGCTGCTGAGAAAGGGATGC	TCTTCAGCGACTTTGTCACG	61.1	201	99	START domain containing protein expressed
OsISM11022	LOC_Os02g01270	151694	CTTCGATGGGGATGATGAGT	CTACACTCTGCCGCACTATCC	59.9	171	87	START domain containing protein expressed
OsISM11023	LOC_Os02g01280	159774	GGATGGGTTGGCTATCAAGA	TTTTGGACCAAGGGTGACTC	59.9	169	77	T-complex protein putative expressed
OsISM11024	LOC_Os02g01280	159943	GGACGGAATGTTGTCCTTGA	TTTTCAACAGGGTCCTCCAG	60.5	284	99	T-complex protein putative expressed
OsISM11025	LOC_Os02g01280	160314	AGGGATGATCACAGAGGGTG	TCACTCACAAGTGCTTTGGC	60.0	191	97	T-complex protein putative expressed
OsISM11026	LOC_Os02g01280	160485	GCCAAAGCACTTGTGAGTGA	ATTTCCAGCACTTACCGCAG	60.2	521	88	T-complex protein putative expressed
OsISM11027	LOC_Os02g01280	161132	CGCGGCTATATTTCTCCCTA	GCATTGTTGATTTTCTTGTCCA	59.7	203	99	T-complex protein putative expressed
OsISM11028	LOC_Os02g01280	161483	GGTTTTGGAGAACGCAAGAG	GCCAACTTCTTCCCTGATGA	60.0	155	79	T-complex protein putative expressed
OsISM11029	LOC_Os02g01280	161753	GGGTGACACAAATCAAGAACC	GACAGCAACACCACCAGAGA	59.6	294	99	T-complex protein putative expressed
OsISM11030	LOC_Os02g01280	162024	AGCTCTCTGGTGGTGTTGCT	CAGTGCATCTTCAACCCTCA	59.9	192	87	T-complex protein putative expressed
OsISM11031	LOC_Os02g01280	162196	TGAGGGTTGAAGATGCACTG	CAAAAGCGTACAGCCACCTC	60.3	197	78	T-complex protein putative expressed
OsISM11032	LOC_Os02g01280	162373	GAGGTGGCTGTACGCTTTTG	TCCTTACGATTTCAGCCCC	60.4	204	97	T-complex protein putative expressed
OsISM11033	LOC_Os02g01280	162617	TGGTGTTAACGGCAGTGTTG	ATTTTCCTGTAGCGGCATTG	60.4	172	81	T-complex protein putative expressed
OsISM11034	LOC_Os02g01280	162769	CAATGCCGCTACAGGAAAAT	TGCTCCAAGCAACATCTCAC	60.0	363	82	T-complex protein putative expressed
OsISM11035	LOC_Os02g01280	163186	CAAGGAGCCTGAGAAAGCAG	CCAACTGCCAAAAAGGGTACT	60.3	180	97	T-complex protein putative expressed
OsISM11037	LOC_Os02g01355	199505	CTACCAGTTCGCTTCTGCCT	TTTGTGTGGTTCAGGTAGCG	59.7	3340	64	MADS-box transcription factor putative expressed
OsISM11038	LOC_Os02g01355	202825	CGCTACCTGAACCACACAAA	AGGGTTGTAGCCTCCGATCT	59.9	598	87	MADS-box transcription factor putative expressed
OsISM11039	LOC_Os02g01355	203410	AGGCTACAACCCTGGGAAAG	TAGCTCCTGCACTGAACACG	60.3	202	93	MADS-box transcription factor putative expressed

ISM IDs	Rice gene locus IDs	Physical positions (bp) on chromosomes	Forward primers (5'-3')	Reverse primers (5'-3')	Melting tempearature (T°C)	Expected product size (bp) in PCR	Expected product size (bp) in Q-PCR	Putative Functions
OsISM11040	LOC_Os02g01355	203622	AAGTGCAGCTGGAGAAGAGC	CCCTGAGCTCCAAAATCTGA	60.1	290	79	MADS-box transcription factor putative expressed
OsISM11041	LOC_Os02g01355	203872	CAGCAAAAGATGCTGATGGA	CACCTGATCACGGAGTACCA	59.7	437	88	MADS-box transcription factor putative expressed
OsISM11042	LOC_Os02g01355	204244	TACACAAAACATGCACGCAG	GACCATGGCGATGACAGAG	59.8	219	86	MADS-box transcription factor putative expressed
OsISM11043	LOC_Os02g01365	199505	CTACCAGTTCGCTTCTGCCT	TTTGTGTGGTTCAGGTAGCG	59.7	3340	64	expressed protein
OsISM11044	LOC_Os02g01365	202825	CGCTACCTGAACCACACAAA	AGGGTTGTAGCCTCCGATCT	59.9	598	87	expressed protein
OsISM11045	LOC_Os02g01365	203410	AGGCTACAACCCTGGGAAAG	TAGCTCCTGCACTGAACACG	60.3	202	93	expressed protein
OsISM11046	LOC_Os02g01365	203622	AAGTGCAGCTGGAGAAGAGC	CCCTGAGCTCCAAAATCTGA	60.1	290	79	expressed protein
OsISM11047	LOC_Os02g01365	203872	CAGCAAAAGATGCTGATGGA	CACCTGATCACGGAGTACCA	59.7	437	88	expressed protein
OsISM11048	LOC_Os02g01365	204244	TACACAAAACATGCACGCAG	GACCATGGCGATGACAGAG	59.8	219	86	expressed protein
OsISM11049	LOC_Os02g01365	204563	ATGATGGAGGACGTGGAGAC	TTTCTTCAAGACTTTGCCCG	60.1	2032	61	expressed protein
OsISM11050	LOC_Os02g01370	207761	AAGCAACGTCGCAACTAATG	ACTCACCAGGGCAGGAGATA	59.3	283	69	hypothetical protein
OsISM11051	LOC_Os02g01370	207987	TTGTAAGTTTGTAACCATAGCCCA	TGAAGAGTGGAAGCATGCAG	60.0	462	89	hypothetical protein
OsISM11052	LOC_Os02g01370	208429	CTGCATGCTTCCACTCTTCA	CCACCACCAGCAAGCTCT	60.1	830	96	hypothetical protein
OsISM11053	LOC_Os02g01390	232999	GATGATCACAGGCGACGAG	CTCCATGCGAAGACGTCAG	60.5	150	76	retrotransposon protein putative unclassified
OsISM11054	LOC_Os02g01390	233431	CCTGCCTCGCATAGATCAGA	TCCTATGGGGGTAATGAACG	60.5	139	89	retrotransposon protein putative unclassified
OsISM11055	LOC_Os02g01390	234626	GAAGAGTGCCCCAGGGAT	GATTTGACCGAGGGGGTAAG	60.3	222	97	retrotransposon protein putative unclassified
OsISM11057	LOC_Os02g01440	262008	CCAACTCAGGGACAAGCTCT	CAGGAAGCTCCCATTGAAGT	59.4	210	89	nitric oxide synthase 1 putative expressed
OsISM11058	LOC_Os02g01440	262228	GCGATTTTGCTGGTGCTAAT	CGCCAATGCAATTCAAATCT	60.6	156	85	nitric oxide synthase 1 putative expressed

ISM IDs	Rice gene locus IDs	Physical positions (bp) on chromosomes	Forward primers (5'-3')	Reverse primers (5'-3')	Melting tempearature (T˚C)	Expected product size (bp) in PCR	Expected product size (bp) in Q-PCR	Putative Functions
OsISM11059	LOC_Os02g01440	262383	GACTGGGTTGTTGAGGCAGT	GATATAACCCCAGTGACGCC	59.7	195	96	nitric oxide synthase 1 putative expressed
OsISM11060	LOC_Os02g01440	262548	GTCACTCGTTGGCGTCACT	GGAGATGGAGAGTGCTTATTGC	60.1	293	97	nitric oxide synthase 1 putative expressed
OsISM11061	LOC_Os02g01440	262784	TTCTTGTTAGGGCCGAGATG	AGATTTCCCAACATTTGCTGA	59.9	444	53	nitric oxide synthase 1 putative expressed
OsISM11062	LOC_Os02g01440	263193	CACCATTACAGGGTTCAGCA	TGCCACTGGATCCTTATATGC	59.8	201	84	nitric oxide synthase 1 putative expressed
OsISM11063	LOC_Os02g01440	263440	CGACCCTTGGTCCTATTCAA	ATGGATAACTGCTGCTTGCC	60.1	302	99	nitric oxide synthase 1 putative expressed
OsISM11064	LOC_Os02g01440	263757	TCTCTTGCACCACAAAGTCG	CCCACTCAATCCAACATCTG	59.5	158	70	nitric oxide synthase 1 putative expressed
OsISM11065	LOC_Os02g01440	263895	CAGATGTTGGATTGAGTGGG	TAGAACGTCAGTCGTGTGCG	60.0	202	92	nitric oxide synthase 1 putative expressed
OsISM11066	LOC_Os02g01440	264128	CCAACCACAGAAGCAGATGA	TCCTTCAGCCTTCTCTTTGC	59.8	530	82	nitric oxide synthase 1 putative expressed
OsISM11067	LOC_Os02g01440	264650	CTGAAGGATGGGTTGGTCTG	CACCCGAGACCAGAAATTG	59.8	288	98	nitric oxide synthase 1 putative expressed
OsISM11068	LOC_Os02g01450	266070	CAGAAGCTGCCTCCTCGG	TCCTTCTTTAACCATTCGAACTC	60.5	197	92	expressed protein
OsISM11069	LOC_Os02g01450	266284	AGGTGGTGTTCGGCAAGAT	TGAAGAGCACAATTCCAGCA	60.5	280	97	expressed protein
OsISM11070	LOC_Os02g01450	266543	TTGCTGGAATTGTGCTCTTC	CTAGACAGGCAGTCAGTGCG	59.4	674	98	expressed protein
OsISM11071	LOC_Os02g01450	267169	GCGACGCATATTTGTAAGCA	TTTGGCTTTCATTGGACACA	60.0	486	99	expressed protein
OsISM11072	LOC_Os02g01450	267635	TGTGTCCAATGAAAGCCAAA	GCCTGAGTGTGTTCCATCCT	60.1	182	81	expressed protein
OsISM11073	LOC_Os02g01450	267753	GCATTCCAGGATAGAGCAGC	CAGCTCACTTTGAATGGCAC	59.7	180	91	expressed protein
OsISM11074	LOC_Os02g01450	267913	GTGCCATTCAAAGTGAGCTG	GCTCTGGGAAGCTGATTGAG	59.8	194	86	expressed protein
OsISM11075	LOC_Os02g01450	268087	CTCAATCAGCTTCCCAGAGC	TCCTTCTTCACCTGAGAGGC	59.8	412	75	expressed protein
OsISM11076	LOC_Os02g01480	274737	GTCTACACGGTCTGCGACG	GTAGGTGGCGAAGAGATTGG	60.1	167	94	nucleic acid binding protein putative expressed

ISM IDs	Rice gene locus IDs	Physical positions (bp) on chromosomes	Forward primers (5'-3')	Reverse primers (5'-3')	Melting tempearature (T˚C)	Expected product size (bp) in PCR	Expected product size (bp) in Q-PCR	Putative Functions
OsISM11077	LOC_Os02g01480	274875	ATGACCTCGCCAATCTCTTC	GTCGGTGTAGGGGTCACAGT	59.6	158	84	nucleic acid binding protein putative expressed
OsISM11078	LOC_Os02g01480	275046	AGTTCTCGCAGGTCAGCAAT	AACTGAGGGGCGTATGACAC	60.0	392	95	nucleic acid binding protein putative expressed
OsISM11079	LOC_Os02g01480	275481	AAGGAAGTTCTTGGCCGAAT	GATGATCCCTGACCTGGAGA	60.0	229	78	nucleic acid binding protein putative expressed
OsISM11080	LOC_Os02g01480	275712	CTGCAAACTCACACCGTCAG	TGAGTGAAACGAGGATCTTCAA	60.2	735	89	nucleic acid binding protein putative expressed
OsISM11081	LOC_Os02g01480	276425	TTGAAGATCCTCGTTTCACTCA	TGTTGCATTCATCGACTCAGA	60.1	391	69	nucleic acid binding protein putative expressed
OsISM11082	LOC_Os02g01480	276792	CCATCTGAGTCGATGAATGC	TAGCACTGGAATTGTCGCTG	59.6	174	83	nucleic acid binding protein putative expressed
OsISM11083	LOC_Os02g01490	281584	ACCAACTCAATGGCCACAA	CTTTGTCCGTGACCACCTCT	60.1	198	95	expressed protein
OsISM11084	LOC_Os02g01490	282024	TTTGCTTCTGACCCATCACA	TCGCCCATTTGAAAGCTACT	60.0	365	96	expressed protein
OsISM11085	LOC_Os02g01490	282369	AGTAGCTTTCAAATGGGCGA	TTTTGGACCTCTTGTGCAAC	59.3	180	96	expressed protein
OsISM11086	LOC_Os02g01490	282625	CTTGCTTTGCAACTTGGTGA	GATAGAAAATGGCAGTGGGC	59.8	190	69	expressed protein
OsISM11087	LOC_Os02g01490	282852	GGACAACCTGCAGATGTTTTT	CAGTCATCCAACATGCCATT	59.2	401	99	expressed protein
OsISM11088	LOC_Os02g01490	283310	TACCCATCTTGGAGATTGCC	AAATAGCACGAATGAGCAGC	59.0	142	64	expressed protein
OsISM11089	LOC_Os02g01490	283497	ATGTCTCCTTGGTGCAGACC	CAAGCACCTTCGCATATTCA	60.0	352	96	expressed protein
OsISM11090	LOC_Os02g01490	283900	CATACATGCTTGCGGAAGTG	AGAGGCTTTCAGCACCTTCA	60.2	174	78	expressed protein
OsISM11091	LOC_Os02g01490	284125	GACAGGATACGCACTCACCA	TCCAACTAATTTTGCAGGGG	59.8	150	64	expressed protein
OsISM11092	LOC_Os02g01490	284486	GCAGGAAGATTGCACACAAC	CAGTACTCTGTGTGCTGTCCG	59.4	200	91	expressed protein
OsISM11093	LOC_Os02g01490	284748	TTTGTTTCAAAATCCCACCG	AATCTCAGCACCTTCCTCCA	60.3	198	97	expressed protein
OsISM11094	LOC_Os02g01490	285117	CCAAGCCAGAACCAGTTTTC	CCTCCACCCTTGTTGAATGT	59.8	165	78	expressed protein

ISM IDs	Rice gene locus IDs	Physical positions (bp) on chromosomes	Forward primers (5'-3')	Reverse primers (5'-3')	Melting tempearature (T˚C)	Expected product size (bp) in PCR	Expected product size (bp) in Q-PCR	Putative Functions
OsISM11095	LOC_Os02g01500	287699	ACTCCCATTGCCCCCTTC	AGAGCATCACGTAGAGTCCG	60.1	304	86	2-oxo acid dehydrogenases acyltransferase domain containing protein expressed
OsISM11096	LOC_Os02g01500	288051	ATCCTTTCCCACAAAGGGAG	CTTGCACTGAGGGAATCTGG	60.5	801	74	2-oxo acid dehydrogenases acyltransferase domain containing protein expressed
OsISM11097	LOC_Os02g01500	288857	TGGGAAAGAGCTTGAGACCT	CGGTAATAGTGGTTGGGATACA A	59.5	184	99	2-oxo acid dehydrogenases acyltransferase domain containing protein expressed
OsISM11098	LOC_Os02g01500	289036	TACCGGTCTCAACGGTTCAT	TGCTGGGATAAGTACCTGCAC	60.3	360	81	2-oxo acid dehydrogenases acyltransferase domain containing protein expressed
OsISM11099	LOC_Os02g01500	289339	GTGGACAGGTGTCTTCAGCA	TCCCAATTTCCTGATGTGGT	60.0	665	67	2-oxo acid dehydrogenases acyltransferase domain containing protein expressed
OsISM11100	LOC_Os02g01500	289997	ATTGGGATGCCATCACTCTC	TCTCCTTCCTTCTTCAGCCA	60.0	208	69	2-oxo acid dehydrogenases acyltransferase domain containing protein expressed
OsISM11101	LOC_Os02g01500	290185	TGGCTGAAGAAGGAAGGAGA	TAGCCCTCTTCCATGCACTC	60.2	233	99	2-oxo acid dehydrogenases acyltransferase domain containing protein expressed
OsISM11102	LOC_Os02g01500	290403	CATGGAAGAGGGCTATCTCG	GGTCACCCTCTTCTTCCACA	59.9	233	99	2-oxo acid dehydrogenases acyltransferase domain containing protein expressed
OsISM11103	LOC_Os02g01500	290822	TTGCAAGAAAATTGGCAGAA	ACCAGTTCCCATTACGCTTG	59.7	138	57	2-oxo acid dehydrogenases acyltransferase domain containing protein expressed
OsISM11104	LOC_Os02g01500	290940	CAAGCGTAATGGGAACTGGT	CTTTTGCCACCCTTTGCTAC	59.9	766	86	2-oxo acid dehydrogenases acyltransferase domain containing protein expressed
OsISM11105	LOC_Os02g01500	291698	GGCAAAAGGGAAGCTCTTG	TAGAGCTTAGCAGGCGGTTT	59.6	183	95	2-oxo acid dehydrogenases acyltransferase domain containing protein expressed
OsISM11106	LOC_Os02g01500	291913	GACGCCCGTGTTGATAATCT	CAGATGACTCTTGCAACGGA	60.0	192	62	2-oxo acid dehydrogenases acyltransferase domain containing protein expressed
OsISM11107	LOC_Os02g01500	292085	TCCGTTGCAAGAGTCATCTG	GACCTTTCGAAGAGCCAGAG	59.6	222	83	2-oxo acid dehydrogenases acyltransferase domain containing protein expressed
OsISM11108	LOC_Os02g01500	292295	CTTCGAAAGGTCCCTCAGTG	CATTGATGTTCACGTTGTGG	59.1	158	74	2-oxo acid dehydrogenases acyltransferase domain containing protein expressed
OsISM11109	LOC_Os02g01500	292421	CTCGTGCAGATACCACAACG	CCAAGTCCCTTCTTGTCTGC	60.1	172	97	2-oxo acid dehydrogenases acyltransferase domain containing protein expressed
OsISM11110	LOC_Os02g01500	292611	GAAGCAGGTGGCTCAAAGAG	CCGAAAGGACCTCCTAGGTT	59.9	171	94	2-oxo acid dehydrogenases acyltransferase domain containing protein expressed
OsISM11111	LOC_Os02g01500	292771	GGTCCTTTCGGAATCAAACA	TGCCAGGTATCACCCTCTTC	60.0	467	95	2-oxo acid dehydrogenases acyltransferase domain containing protein expressed
OsISM11112	LOC_Os02g01500	293245	CGGCCAGTATGAGTTTGGTT	AGAAATTCTGCGCCGATAG	59.0	173	85	2-oxo acid dehydrogenases acyltransferase domain containing protein expressed

ISM IDs	Rice gene locus IDs	Physical positions (bp) on chromosomes	Forward primers (5'-3')	Reverse primers (5'-3')	Melting tempearature (T˚C)	Expected product size (bp) in PCR	Expected product size (bp) in Q-PCR	Putative Functions
OsISM11113	LOC_Os02g01510	296155	ACCTCGACTCCTCCAGGTTC	CGTGCTCTCCCACCATGTA	60.7	2346	88	lactate/malate dehydrogenase putative expressed
OsISM11114	LOC_Os02g01520	300866	GAGATCGACGACAACACTGC	GGGACAGCTGCTTTCTCTTG	59.8	627	86	IQ calmodulin-binding motif family protein putative expressed
OsISM11115	LOC_Os02g01520	301682	ACAAAATCATTGCCCTGCTC	CAGCTCCTCCAGCATAGACC	60.0	716	90	IQ calmodulin-binding motif family protein putative expressed
OsISM11116	LOC_Os02g01540	307509	ATGACCTTCTGCACCAGACC	GGATGGTTCGTCACAATCAG	59.5	1206	87	ribosomal protein putative
OsISM11117	LOC_Os02g01540	308671	TGGGAGGTGGCATGAATATC	GTCCGTTTGTCCTACGCAAG	60.7	136	72	ribosomal protein putative
OsISM11118	LOC_Os02g01540	308796	GACAAACGGACATATCTTGGG	CAGCTTTGATCTTCATGCCA	59.6	896	97	ribosomal protein putative
OsISM11119	LOC_Os02g01540	309707	CCCCATATGCAGCTATGCTT	TTCCAGAAAACAGTCACCCA	59.6	123	90	ribosomal protein putative
OsISM11120	LOC_Os02g01540	309923	CGGTCTGGGATGAATATTGG	CAGCACACCAGCAAAAGTTG	60.3	662	57	ribosomal protein putative
OsISM11121	LOC_Os02g01540	310563	CACAACTTTTGCTGGTGTGC	CTGGTCTTTCAGGGAAGCCT	60.6	356	93	ribosomal protein putative
OsISM11122	LOC_Os02g01570	323960	CCCCTAGATCCCTCTGATCC	CCTCTCTAGGAATACCGGGC	60.0	148	89	retrotransposon protein putative Ty3-gypsy subclass
OsISM11123	LOC_Os02g01570	325860	TCGTCGAACACCTTTATACGC	TTCCGCATATCCCAGAATGT	60.2	297	70	retrotransposon protein putative Ty3-gypsy subclass
OsISM11124	LOC_Os02g01590	343551	CCGAGAGGAACTGGATGAAC	CAGGTGGTACCAACCCTTGT	59.7	437	60	glycosyl hydrolases putative expressed
OsISM11125	LOC_Os02g01590	344756	GTCCTCTGGGGTTGGGTC	GTGTCCAGCTCCACCGTC	60.3	1103	99	glycosyl hydrolases putative expressed
OsISM11126	LOC_Os02g01620	358258	GTCCATCTGGACAGGCAAGT	GTGGTTCCTGTCAATCCCAC	60.2	460	75	transposon protein putative unclassified
OsISM11128	LOC_Os02g01620	359229	CTAGCAAGGCTTGGTTGCTC	AACCACAACAGGTACGCCTC	60.1	184	98	transposon protein putative unclassified
OsISM11129	LOC_Os02g01690	380527	ACGAACCTCGACGGAGGAG	CACCTGGACGTGAGCGTAAC	62.2	1206	90	transposon protein putative unclassified
OsISM11130	LOC_Os02g01690	381948	CGGACTTAGGCAGGTGTTTC	CTAGCTGTCCCCGTCGAGT	60.1	159	81	transposon protein putative unclassified
OsISM11131	LOC_Os02g01690	382315	GCACCGTGACCGAGACTACT	ATATCGGCACTGATATCCCG	60.0	152	58	transposon protein putative unclassified

ISM IDs	Rice gene locus IDs	Physical positions (bp) on chromosomes	Forward primers (5'-3')	Reverse primers (5'-3')	Melting tempearature (T°C)	Expected product size (bp) in PCR	Expected product size (bp) in Q-PCR	Putative Functions
OsISM11133	LOC_Os02g01690	383023	AGCTCTTCCTCTCACGGAGC	GATGAAGAGGCCCCAGTACC	61.0	143	81	transposon protein putative unclassified
OsISM11134	LOC_Os02g01710	392120	GTCCAGGTTCTTACGGCTGA	AATTCCATGTCGGTTTGGAG	60.0	227	78	peptidase family C78 domain containing protein expressed
OsISM11135	LOC_Os02g01710	392354	CAAATCGCCCTCACTCAATC	TTGAGCATGAGGATCACCAG	60.2	301	91	peptidase family C78 domain containing protein expressed
OsISM11136	LOC_Os02g01710	393339	TTTGCTAGCAGGCATTCTGA	TGGAATGACCTTGATGTTGG	59.5	213	92	peptidase family C78 domain containing protein expressed
OsISM11137	LOC_Os02g01710	393608	ACCCTTTTGATTTTGGACCC	ACTAGACGCTGCCATCCTGT	60.0	279	81	peptidase family C78 domain containing protein expressed
OsISM11138	LOC_Os02g01710	393867	ACAGGATGGCAGCGTCTAGT	AGCAATTCCAGGATCCACAT	59.6	168	85	peptidase family C78 domain containing protein expressed
OsISM11139	LOC_Os02g01720	397571	GCCTCCCTCTCCACCCTC	GCAGCCACGTCACTATCTGA	61.1	968	93	transmembrane protein 120A putative expressed
OsISM11140	LOC_Os02g01720	398519	TCAGATAGTGACGTGGCTGC	TTACAGGGCCAACAAAGGTC	60.0	162	74	transmembrane protein 120A putative expressed
OsISM11141	LOC_Os02g01720	398687	GGGTGGCGAGAAAAGAAGAT	GAGGAGAATGGATGGGAACA	60.2	647	99	transmembrane protein 120A putative expressed
OsISM11142	LOC_Os02g01720	399369	AGCCTTGGCAGTTCAGATGT	ATCAACACATTCTCCCGCA	60.0	180	76	transmembrane protein 120A putative expressed
OsISM11143	LOC_Os02g01720	399530	TGCGGGAGAATGTGTTGAT	GCTAACGAGAGCCATTAGCA	59.2	348	90	transmembrane protein 120A putative expressed
OsISM11144	LOC_Os02g01720	399896	GGACAGCCTGATTGTTCCAG	CATTGCAATTCCTTGCATGA	60.6	176	76	transmembrane protein 120A putative expressed
OsISM11145	LOC_Os02g01720	400106	GCACCCGGATAGCTCTAGG	TTCTCCAGCAACAACATCCA	60.0	453	51	transmembrane protein 120A putative expressed
OsISM11146	LOC_Os02g01720	400593	ACCCGGTCCTTTTCACATT	AATTCTTGCCTGCCATTCC	59.6	347	99	transmembrane protein 120A putative expressed
OsISM11147	LOC_Os02g01720	400892	CTTAAGACGGCTTTGCACG	ACCAGCAAGATCCCACAAAC	59.8	2176	63	transmembrane protein 120A putative expressed
OsISM11148	LOC_Os02g01740	411349	ACGAAGATGACGAGGAGAGC	CATTCGACTCAGGCAAGTCA	59.8	184	82	U5 small nuclear ribonucleoprotein 200 kDa helicase putative expressed
OsISM11149	LOC_Os02g01740	413680	CCGGCAGATATCATGGAGAC	AGCAGATTTGCAGCAGAGTG	59.9	829	75	U5 small nuclear ribonucleoprotein 200 kDa helicase putative expressed
OsISM11150	LOC_Os02g01740	414844	CGCTTAGCTCCGATATGGTC	TAGTTGGGCCCATCCTCTCT	60.4	202	93	U5 small nuclear ribonucleoprotein 200 kDa helicase putative expressed

ISM IDs	Rice gene locus IDs	Physical positions (bp) on chromosomes	Forward primers (5'-3')	Reverse primers (5'-3')	Melting tempearature (T˚C)	Expected product size (bp) in PCR	Expected product size (bp) in Q-PCR	Putative Functions
OsISM11151	LOC_Os02g01760	421525	ACCTGATCTGCACCTGAAGC	TGTATGCAACGCAGATGTCA	60.1	730	81	diphosphomevalonate decarboxylase family protein
OsISM11152	LOC_Os02g01760	422256	GCCTCTTCGTTCTAATGCCA	GTCAGGGATTCGGACTAGCA	60.3	393	56	diphosphomevalonate decarboxylase family protein
OsISM11153	LOC_Os02g01760	422889	GACCGGATGTGGCTCAAC	GAACCTCCCTCCTGACAAGG	60.5	495	49	diphosphomevalonate decarboxylase family protein
OsISM11154	LOC_Os02g01760	423496	ACAACAACTTCCCCACTGCT	CACATTCATTAGATTTCCAAGGG	59.7	270	87	diphosphomevalonate decarboxylase family protein
OsISM11155	LOC_Os02g01760	423739	TTCACCCTTGGAAATCTAATGAA	TATACTGCGGCATGCACTTC	59.8	186	91	diphosphomevalonate decarboxylase family protein
OsISM11156	LOC_Os02g01760	423934	TTCGTGAAATGGTGTATGGG	TCATTCCAGTGTGCCTCATC	59.4	180	81	diphosphomevalonate decarboxylase family protein
OsISM11157	LOC_Os02g01760	424094	GATGAGGCACACTGGAATGA	CACTGTCTCGCATCCCACTA	59.7	292	92	diphosphomevalonate decarboxylase family protein
OsISM11159	LOC_Os02g01780	430240	GGATCAGTGAGGGGATTGAC	CTCCACCAGTTGGGTTGATT	59.6	149	69	hypothetical protein
OsISM11160	LOC_Os02g01800	442352	GTTTTCATTTGGGGCAAAAA	AAGCGGATCATCTTGCTGTT	59.8	133	98	receptor-like protein kinase precursor putative
OsISM11161	LOC_Os02g01800	445435	GCAGCGCCTGGTAACTCTAC	TTGATATTTGCCCACCCATT	60.0	222	89	receptor-like protein kinase precursor putative
OsISM11162	LOC_Os02g01840	463279	ATCCTGATGATGGTGGCAAT	CGTGTGTATTTGCTGCAGGT	60.0	266	79	retrotransposon protein putative unclassified
OsISM11163	LOC_Os02g01840	464149	TGCTTTTCAACATGGTTTGC	AAGGACAAAAATAGTCACACTCCC	59.8	241	92	retrotransposon protein putative unclassified
OsISM11164	LOC_Os02g01840	464486	GGTGATCTGCACTATTTTATGGG	TGACAGACCTTATTTACTGCAAAA	58.9	278	90	retrotransposon protein putative unclassified
OsISM11166	LOC_Os02g01880	487490	AATTCTTCAAGCGTTTCCCC	CAAGTTCGTCATGTCACGCT	60.2	120	69	U5 small nuclear ribonucleoprotein 200 kDa helicase putative
OsISM11167	LOC_Os02g01880	487642	GTACCCAAAGCCCAAGGAAG	TCCTTTCTCCCAGCTTCTGA	60.5	130	88	U5 small nuclear ribonucleoprotein 200 kDa helicase putative
OsISM11168	LOC_Os02g01910	495595	GGACGGAGCTACTTCACGAC	ACCATGGCTGTCTCTTCGAG	60.1	122	76	hypothetical protein
OsISM11170	LOC_Os02g01950	519497	TGCTTTGTTTTCACAGGTTTG	TGTTTAAATCCAAGTCAATCAACTG	59.2	192	92	transposon protein putative unclassified
OsISM11171	LOC_Os02g01950	519659	AACAACAGTTGATTGACTTGGA	TCTTGTCCTATTGTAATTTTGCTCA	58.2	164	84	transposon protein putative unclassified

ISM IDs	Rice gene locus IDs	Physical positions (bp) on chromosomes	Forward primers (5'-3')	Reverse primers (5'-3')	Melting tempearature (T°C)	Expected product size (bp) in PCR	Expected product size (bp) in Q-PCR	Putative Functions
OsISM11172	LOC_Os02g01950	519837	TGCAAAGAAAAGTAGTACACGATCA	TGTCTTCTTCTCAATGTTTTTGTCTC	60.2	181	89	transposon protein putative unclassified
OsISM11173	LOC_Os02g01950	519958	TGTTGTGCAGGTATTTTGAAGG	AATTTTGCTCCAGTAACCCAA	59.3	212	98	transposon protein putative unclassified
OsISM11174	LOC_Os02g01950	520192	GCTTGAAGATGGCAAAGGTG	CAGCTATTCTCTTTCCCTTGTCA	60.3	156	78	transposon protein putative unclassified
OsISM11175	LOC_Os02g01950	520352	CGAGAATGTAAAAACAATGGAAAA	TTGGTTTGATGTATTTCTTCTGC	58.9	169	97	transposon protein putative unclassified
OsISM11176	LOC_Os02g01950	520523	TTCTATCACTATTGGATCAAGATTTCA	TCATTGCATCTTGTAGGTTGTG	59.1	177	98	transposon protein putative unclassified
OsISM11177	LOC_Os02g01950	520676	TTCACAACCTACAAGATGCAATG	CAACATTTGTGCTGCTGCTT	60.1	150	63	transposon protein putative unclassified
OsISM11179	LOC_Os02g01950	521043	TTGATTCTGTACAACCTTGGTCTC	TTTCGCCTTTATCACTAGACTGAA	59.5	193	91	transposon protein putative unclassified
OsISM11180	LOC_Os02g01970	540528	AGGCCTTCCTCCCTGGAC	GCCTTCTCTGTAACTTCATTTGC	60.5	770	86	expressed protein
OsISM11181	LOC_Os02g01970	541453	AAGATTTGAGCAAACCCCG	CCGCAAGAGCAGCTAGAAGT	60.0	162	79	expressed protein
OsISM11182	LOC_Os02g01970	541994	AGTTCTCTTATCCCACGCCC	TGGAATAGAAGGTTTTTCCAGG	60.0	191	96	expressed protein
OsISM11183	LOC_Os02g01970	542182	CCAACTGGAATTCAGAACCA	GAGCTACCAGCCAACAAAGC	59.3	168	80	expressed protein
OsISM11184	LOC_Os02g01970	542416	GGCCACTGTTAGTTCATGTGTC	CAACAACAGATACACAGTAAGCCTG	59.8	695	99	expressed protein
OsISM11185	LOC_Os02g01970	543316	GGAACAAAATGATGACACTCAAG	GGTTTGTTGGCTGAAATCCA	59.7	1314	99	expressed protein
OsISM11186	LOC_Os02g01970	544641	AATCATTGGGCAACAAATCC	AGCTGGGCGAGAAATTCTAA	59.3	199	97	expressed protein
OsISM11187	LOC_Os02g02010	558931	CGATCGAGTTGGTGTTGAAG	CTGCATCTTGTCGACCTCCT	59.8	139	84	conserved hypothetical protein
OsISM11188	LOC_Os02g02010	559183	GGTGAAGAAGATCACCAGCC	TTCTTCTTCTTCTCGCTGGC	59.7	509	73	conserved hypothetical protein
OsISM11189	LOC_Os02g02070	607608	AATTGGACGGGAGAACGG	AGAGTGGAGCCAATTGAAACA	60.3	1262	98	hypothetical protein
OsISM11190	LOC_Os02g02110	623360	ACCTACTTCAACCCCGACG	TCCACTCCCAAGTAAGGTGG	60.0	242	86	Alg9-like mannosyltransferase protein putative expressed

ISM IDs	Rice gene locus IDs	Physical positions (bp) on chromosomes	Forward primers (5'-3')	Reverse primers (5'-3')	Melting tempearature (T˚C)	Expected product size (bp) in PCR	Expected product size (bp) in Q-PCR	Putative Functions
OsISM11191	LOC_Os02g02110	623643	GCGCTTTACAAGATTCTGGC	ACCAGTTAACCAGCTGCGAA	60.4	648	74	Alg9-like mannosyltransferase protein putative expressed
OsISM11192	LOC_Os02g02110	624549	CGAAATCCCGGTTTCTCTTT	ACCTGGGAACCATACATCCA	60.2	775	95	Alg9-like mannosyltransferase protein putative expressed
OsISM11193	LOC_Os02g02110	625484	TGGAGGCTTGCAGGTCTTAT	AAACATCAAAGGAAGCACCG	60.0	190	97	Alg9-like mannosyltransferase protein putative expressed
OsISM11194	LOC_Os02g02110	625781	AACAAATGTTCCAATGGCTTT	GGGCTTCTCGTGACAGGTAA	59.4	226	84	Alg9-like mannosyltransferase protein putative expressed
OsISM11195	LOC_Os02g02110	626087	CCTATGCGCTTTTTGGACTG	AACCTTGCCCACAAAATCTG	60.4	205	98	Alg9-like mannosyltransferase protein putative expressed
OsISM11197	LOC_Os02g02130	633816	AGAGCACCTGCTCCTCCTG	TCCTTCCAGTTCCTGTGGTC	60.4	1591	97	ribosomal protein L37 putative
OsISM11198	LOC_Os02g02130	635387	GACCACAGGAACTGGAAGGA	CTCTTCCTTGGGGTAGCCTC	60.1	203	94	ribosomal protein L37 putative
OsISM11199	LOC_Os02g02200	676523	GGGACTCAAAAGAGCCAAGA	CTGCAGCTGTGACCATAGGA	59.7	617	73	thaumatin putative
OsISM11200	LOC_Os02g02230	713003	GGCCACGTTGATTCTATGGT	AGGATCAGGTTCCCCATCTC	60.0	1185	99	cytochrome P450 putative
OsISM11201	LOC_Os02g02230	715053	TGGGAGAAATTCATTCCAGG	GTTTACGCCATCCCTTCCC	60.7	563	65	cytochrome P450 putative
OsISM11202	LOC_Os02g02240	719200	CGAGCGCTACTACAACACCA	CCCCAGTTTTTACAAGAGTTCC	59.6	283	81	expressed protein
OsISM11203	LOC_Os02g02250	727040	AGCTGCAGTGGTGAACAAAA	AAGGCCCTTCTCTGTGCTCT	60.0	888	93	expressed protein
OsISM11204	LOC_Os02g02250	728002	GGACAATATGCAAACCTGGG	ATAGCACTTCGCAAGAAGCC	59.9	535	72	expressed protein
OsISM11205	LOC_Os02g02250	728604	ATTCATATTGATGCCCTGCC	TGTTATGTGCTTGTCCCAGG	59.7	163	71	expressed protein
OsISM11206	LOC_Os02g02250	729430	ATTTGCTGGTGTTTATCCGC	TCTTGAGTTTTTGCTCCATGTT	59.4	171	79	expressed protein
OsISM11207	LOC_Os02g02250	729640	CGGCTGCTGTTGTAGACCTT	TTAGAAAACGCATTCCACGG	60.7	166	86	expressed protein
OsISM11208	LOC_Os02g02270	744815	TCAAGCGCTACTACAACACCA	CCCCAGTTTTTACAAGAGTTCC	59.3	281	82	expressed protein
OsISM11209	LOC_Os02g02275	747283	TCCAGCTTCATGCGCTACTA	GCGCCAGTTCTTACCAGAGT	59.6	825	90	expressed protein

ISM IDs	Rice gene locus IDs	Physical positions (bp) on chromosomes	Forward primers (5'-3')	Reverse primers (5'-3')	Melting tempearature (T˚C)	Expected product size (bp) in PCR	Expected product size (bp) in Q-PCR	Putative Functions
OsISM11210	LOC_Os02g02360	786336	CTGCCCCTGAATTCGTCA	CTGAAAGCGTCTGGAGGTCT	60.2	482	67	expressed protein
OsISM11211	LOC_Os02g02360	787676	GTGGCATGCAAATACGTCAG	GCAAGCATGACACCTCAGAA	60.1	156	51	expressed protein
OsISM11212	LOC_Os02g02360	787923	ATGGGATCACTATGGCCTCA	ATGGCATCTCTTCCACCTTG	60.2	587	89	expressed protein
OsISM11213	LOC_Os02g02360	788518	ATCCCATTTTGCGTGAAGAC	TCCTCGACACCCAAGAAATC	60.0	323	95	expressed protein
OsISM11214	LOC_Os02g02390	819202	GACAGGATGGAGCTGGAGTT	ATGAAGCATCTGGGTCTGCT	59.5	828	95	S1 RNA binding domain containing protein expressed
OsISM11215	LOC_Os02g02390	820738	GTGCAGTTGACCCAGAAACA	GGAAGCACAGATGACTCACG	59.6	166	80	S1 RNA binding domain containing protein expressed
OsISM11216	LOC_Os02g02390	820884	CGTGAGTCATCTGTGCTTCC	GATCATCCACCACAGAAGGG	59.9	171	95	S1 RNA binding domain containing protein expressed
OsISM11217	LOC_Os02g02390	821054	CAATCAGCCGTCAGCAGTAA	GCATCAACTTGAGATGGAAATG	59.8	316	99	S1 RNA binding domain containing protein expressed
OsISM11218	LOC_Os02g02390	821409	TGATGATGGAGGCAATGTGT	TTCTCCAGCCTTGTCCAGTC	60.2	270	70	S1 RNA binding domain containing protein expressed
OsISM11219	LOC_Os02g02390	821691	CGGAGAAAAAGTTGAGGTGG	GCAAGATTGCGATACGGTAA	59.3	191	97	S1 RNA binding domain containing protein expressed
OsISM11220	LOC_Os02g02390	822077	TCTTCTTCGAACATATAACACT GAGAA	CTGTCAGCCAGGACAACTGA	59.9	166	88	S1 RNA binding domain containing protein expressed
OsISM11221	LOC_Os02g02390	822269	TGAAGCCAAAAGAAAGTGAA GA	TGCATTCAACAATATCTCCCA	59.1	208	82	S1 RNA binding domain containing protein expressed
OsISM11222	LOC_Os02g02390	822442	GGCTAAGCTCAATGTGGGAG	GCTGAATTAATGCGGGAACT	59.5	166	99	S1 RNA binding domain containing protein expressed
OsISM11223	LOC_Os02g02390	822611	GGGAAGTGTCTTGGGATGAA	CCAGTTGAATAACTTTAGCATCC A	59.7	304	82	S1 RNA binding domain containing protein expressed
OsISM11224	LOC_Os02g02390	822882	TCCAGGTTGTGGATGCTAAA	CAATGCGCCTACTGAAGGAT	59.7	205	99	S1 RNA binding domain containing protein expressed
OsISM11225	LOC_Os02g02390	823126	CTTGAACCTGCAGAAGCAGA	CTCTTCCATGAGAGCATCCA	59.1	699	55	S1 RNA binding domain containing protein expressed
OsISM11226	LOC_Os02g02390	823878	CCCAGGACTAGCTCCAACCT	CCATAGCGTGCAAGGAGTTT	60.5	190	76	S1 RNA binding domain containing protein expressed
OsISM11227	LOC_Os02g02390	824048	AAACTCCTTGCACGCTATGG	TTTGTGCACGCTAAAACTGC	60.2	191	96	S1 RNA binding domain containing protein expressed

ISM IDs	Rice gene locus IDs	Physical positions (bp) on chromosomes	Forward primers (5'-3')	Reverse primers (5'-3')	Melting tempearature (T°C)	Expected product size (bp) in PCR	Expected product size (bp) in Q-PCR	Putative Functions
OsISM11228	LOC_Os02g02440	861628	CCGCCATCTCAGACATGAAT	TTGAACACGTTACCAGGGTG	60.2	228	91	hypothetical protein
OsISM11230	LOC_Os02g02450	862711	CATGCACCTCGATCACATTC	CATCCCCTACAGCTGCTTTC	60.0	166	76	transposon protein putative unclassified expressed
OsISM11231	LOC_Os02g02450	863054	CACCTCAAGGTGTCAAGCAA	AAGAACCACTGGAAGAGCGA	59.9	192	92	transposon protein putative unclassified expressed
OsISM11232	LOC_Os02g02450	863266	GCTTCAAATTCTTCCCCACA	ACATTGGCCATGGTGAAATC	60.3	159	74	transposon protein putative unclassified expressed
OsISM11233	LOC_Os02g02470	871150	CGTCTCCTTCGTCGTCACC	CAGCCATCACAACATGACCT	60.7	1447	90	oxidoreductase short chain dehydrogenase/reductase family domain containing protein expressed
OsISM11234	LOC_Os02g02470	872649	TTCAGAGATAGGAAAGCCCC	ATACGAGCATTCCAGGCATC	59.2	394	97	oxidoreductase short chain dehydrogenase/reductase family domain containing protein expressed
OsISM11235	LOC_Os02g02470	873066	ACAACGCTGGCATTTTCTCT	AATAAAGCCGGTGCAAGATG	60.0	222	95	oxidoreductase short chain dehydrogenase/reductase family domain containing protein expressed
OsISM11236	LOC_Os02g02470	873311	TTCTTCGTGGTTCTCCTAGC	CCGTTTTCCAGATGTCAAGTT	58.1	329	99	oxidoreductase short chain dehydrogenase/reductase family domain containing protein expressed
OsISM11237	LOC_Os02g02470	873623	TGACATCTGGAAAACGGAAA	GGAATCCTCCTGTGAAGCAT	59.1	274	95	oxidoreductase short chain dehydrogenase/reductase family domain containing protein expressed
OsISM11238	LOC_Os02g02470	873917	TTATTTGTGCCTCTCCTGGG	CGGAATCAAATGATATGCAGC	60.3	1178	84	oxidoreductase short chain dehydrogenase/reductase family domain containing protein expressed
OsISM11239	LOC_Os02g02470	875056	CTCCCCAAGCTTGTTGTAGC	ATCAGAAGCTGCGAACAGTG	59.5	189	94	oxidoreductase short chain dehydrogenase/reductase family domain containing protein expressed
OsISM11240	LOC_Os02g02500	890395	AGTACGAGCGCATCGAGAAG	CTCCACCCCCAGCAACAT	61.3	221	84	remorin family protein putative expressed
OsISM11241	LOC_Os02g02500	890685	GTGCCCAAGTCCTCTGTCA	TTCTTCGCACATGCTACCTC	59.4	547	67	remorin family protein putative expressed
OsISM11242	LOC_Os02g02500	891725	CAAAGTCTGCACTTGCTGAAG	ATGTTGTCGCGCTCTGAAG	59.5	691	94	remorin family protein putative expressed
OsISM11243	LOC_Os02g02500	892789	AACAGAAATTCGTGCAGCAG	TCTTCCCATGCTTGGATCTT	59.4	202	94	remorin family protein putative expressed
OsISM11244	LOC_Os02g02500	893001	GCAAAAATCGAAGCTGAAATG	CTGGCTCATCAGCTTGTCCT	60.2	868	82	remorin family protein putative expressed
OsISM11245	LOC_Os02g02520	896391	CATGGACGAGGGCAAGAC	AAGAAGAGATGTTGCTCGGC	59.9	213	63	expressed protein
OsISM11246	LOC_Os02g02520	896553	TTTTGGTGGTGGTGCTTGT	AAGAGCAAGCTGGATGTTGG	60.2	395	99	expressed protein

ISM IDs	Rice gene locus IDs	Physical positions (bp) on chromosomes	Forward primers (5'-3')	Reverse primers (5'-3')	Melting tempearature (T˚C)	Expected product size (bp) in PCR	Expected product size (bp) in Q-PCR	Putative Functions
OsISM11247	LOC_Os02g02520	896933	ATCCAGCTTGCTCTTGCTGT	CTGCGCTTTTCTGGTAGGTC	60.1	192	90	expressed protein
OsISM11248	LOC_Os02g02520	897105	GACCTACCAGAAAAGCGCAG	CCAGGGAACTGATAGTTGCAG	59.9	202	80	expressed protein
OsISM11249	LOC_Os02g02520	897358	CCCTGGATTTTGACTTTGCT	TAGCGATGGTGGTTCATCAG	59.4	621	63	expressed protein
OsISM11250	LOC_Os02g02520	897959	CTGATGAACCACCATCGCTA	CTGGGACAGATGAGGCAAAT	59.9	911	97	expressed protein
OsISM11251	LOC_Os02g02520	898850	ATTTGCCTCATCTGTCCCAG	AATGCAAATCTTTTGGTGGG	59.9	184	96	expressed protein
OsISM11252	LOC_Os02g02520	899057	CCAGTATTTTATATCAGGCAGCG	TTGACGGAATACAGCACCAC	59.8	179	88	expressed protein
OsISM11253	LOC_Os02g02520	899216	GTGGTGCTGTATTCCGTCAA	AAGAACCATCGGCTTGCTTA	59.7	295	64	expressed protein
OsISM11254	LOC_Os02g02520	899528	AGTGCAGCTCGGTTCACTTT	CCTTGTTCTTCTTGCAAACCA	60.2	154	72	expressed protein
OsISM11255	LOC_Os02g02520	899665	TTGCAAGAAGAACAAGGAAGC	CATGCTGACGATTTCTCCTG	59.5	403	99	expressed protein
OsISM11256	LOC_Os02g02524	901114	CTCCATCGGCGACAAGTG	CAGACAGAAAAAGAGTTCACTCACA	60.7	392	89	canopy homolog 2 precursor putative expressed
OsISM11257	LOC_Os02g02524	901439	TTGATCTTGTTTGCAGGCAG	GCGCAAGTCCAAGTGATTTC	60.4	304	71	canopy homolog 2 precursor putative expressed
OsISM11258	LOC_Os02g02524	901754	TTCAAAAGGTCAGCGTGAAG	ATAGGCCATCCAGAAGCTCA	59.4	2026	78	canopy homolog 2 precursor putative expressed
OsISM11259	LOC_Os02g02524	903761	GAGCTTCTGGATGGCCTATG	CACCCATCCTTTTTCTCCTG	59.7	281	76	canopy homolog 2 precursor putative expressed
OsISM11260	LOC_Os02g02524	904022	CAGGAGAAAAAGGATGGGTG	AATTTTTGGAGTGTGCTCGTG	59.8	575	85	canopy homolog 2 precursor putative expressed
OsISM11261	LOC_Os02g02524	904556	TGTAGAAAAGAAGGCCGCAG	GCAGCCTACCTCATCCTCAG	60.2	618	92	canopy homolog 2 precursor putative expressed
OsISM11262	LOC_Os02g02524	905131	ATGGTGCAGGTTACTGGAGG	CTGCGGAGCTCGTTTTTATC	60.0	230	63	canopy homolog 2 precursor putative expressed
OsISM11263	LOC_Os02g02524	905356	CGCAGAATCAGGAAATGTCA	CGTCGTCGATCTCGATGGTA	61.0	207	87	canopy homolog 2 precursor putative expressed
OsISM11264	LOC_Os02g02530	907268	AGAGCTGCTAATTTGGCCCT	TGACGTTCACATTTGGCTCT	59.8	199	74	signal peptide peptidase domain containing protein expressed

ISM IDs	Rice gene locus IDs	Physical positions (bp) on chromosomes	Forward primers (5'-3')	Reverse primers (5'-3')	Melting tempearature (T˚C)	Expected product size (bp) in PCR	Expected product size (bp) in Q-PCR	Putative Functions
OsISM11265	LOC_Os02g02530	907493	GTGGGCTGTTACCGTTCTGT	CAAAGGAAACCGCATAGCAT	60.1	207	76	signal peptide peptidase domain containing protein expressed
OsISM11266	LOC_Os02g02530	907761	TCAATGCTGTTCTCACTGCC	CGTTTTATGGAAGGCAGCA	59.9	184	77	signal peptide peptidase domain containing protein expressed
OsISM11267	LOC_Os02g02530	907969	TAATGCCATCGTTTGGTGTG	TGCAAAAGAAAAATCCTGGG	60.2	276	96	signal peptide peptidase domain containing protein expressed
OsISM11268	LOC_Os02g02530	908286	TTGGGGATTTCTTTCTGCAT	CAAACAGCCCATACCAAGAGA	59.8	230	87	signal peptide peptidase domain containing protein expressed
OsISM11269	LOC_Os02g02530	908471	TGGATCATTCAAAACTGGTGC	TGATTTTGCAACACTGACCA	59.8	372	98	signal peptide peptidase domain containing protein expressed
OsISM11270	LOC_Os02g02530	908835	CAAAATCATTTGATGCACCG	ACCAAGCCCAAGCATAGAGA	59.9	183	78	signal peptide peptidase domain containing protein expressed
OsISM11271	LOC_Os02g02530	908998	TCTCTATGCTTGGGCTTGGT	GACATCAAACCGCAGTGCTA	59.9	766	69	signal peptide peptidase domain containing protein expressed
OsISM11272	LOC_Os02g02530	909809	TATACGGTGGGTTTGACCGT	CAAAACCAATCACACCAGGA	59.7	544	95	signal peptide peptidase domain containing protein expressed
OsISM11273	LOC_Os02g02530	910333	TCCTGGTGTGATTGGTTTTG	CAGCATCTTCCTCTTCAGCC	59.7	681	99	signal peptide peptidase domain containing protein expressed
OsISM11274	LOC_Os02g02540	920363	GTGATGATGGTTTCCTTGGG	CAATTGTTGAGCACTGTGGG	60.2	188	88	glutamate receptor putative expressed
OsISM11275	LOC_Os02g02540	921780	TTTGGGGGTGGTAATGAAAA	CAATATCCCCTATTGCTGCG	60.2	169	86	glutamate receptor putative expressed
OsISM11276	LOC_Os02g02540	922147	TAAATGACGAATTCCGTGGC	GGTTGGTTTACTCACTGTGTGC	60.2	434	97	glutamate receptor putative expressed
OsISM11277	LOC_Os02g02540	922533	GGTTCAGCTTTTCAACCTTGTT	TGATCAAGACACCACGTCCT	59.4	163	73	glutamate receptor putative expressed
OsISM11278	LOC_Os02g02540	923035	ATTTCACCAGCAGAGGATGG	TCAATTTGCAAAGGGGAGTC	60.1	207	56	glutamate receptor putative expressed
OsISM11279	LOC_Os02g02550	924814	GTCGCTGGAGGACTACGTCA	TTGAGCCCTTTTCAGCAGTT	60.7	399	87	peptidyl-prolyl cis-trans isomerase FKBP-type putative expressed
OsISM11280	LOC_Os02g02550	925193	AACTGCTGAAAAGGGCTCAA	CCTGCCAGTAGTTTGGCTTC	59.9	178	94	peptidyl-prolyl cis-trans isomerase FKBP-type putative expressed
OsISM11281	LOC_Os02g02550	925351	GAAGCCAAACTACTGGCAGG	TCTACAAATTCCCGCTTTCG	60.0	190	96	peptidyl-prolyl cis-trans isomerase FKBP-type putative expressed
OsISM11282	LOC_Os02g02550	925605	ACTGAAGGGATGAAAGTGGG	CCCTTTTTCCCATATCCGAG	59.8	189	69	peptidyl-prolyl cis-trans isomerase FKBP-type putative expressed

ISM IDs	Rice gene locus IDs	Physical positions (bp) on chromosomes	Forward primers (5'-3')	Reverse primers (5'-3')	Melting tempearature (T˚C)	Expected product size (bp) in PCR	Expected product size (bp) in Q-PCR	Putative Functions
OsISM11283	LOC_Os02g02550	925769	CAGAGCTCGGATATGGGAAA	TCTCTGCAGGAGGGACAACT	60.1	203	97	peptidyl-prolyl cis-trans isomerase FKBP-type putative expressed
OsISM11284	LOC_Os02g02600	950844	GAGAGGGAGAGATGGGGAAC	GCTTGTTTTGGGGGTCATT	59.9	733	99	serine/threonine-protein kinase Cx32 chloroplast precursor putative expressed
OsISM11285	LOC_Os02g02600	951863	GCATGGTTGTTGCTGTCAAG	GTTTGGATGCGAGAGCCTAC	60.1	729	99	serine/threonine-protein kinase Cx32 chloroplast precursor putative expressed
OsISM11286	LOC_Os02g02600	952659	AAGGGAGCTTGGAGAACCAT	ATCTTCAGCCTCAGTTCCCA	59.9	337	68	serine/threonine-protein kinase Cx32 chloroplast precursor putative expressed
OsISM11287	LOC_Os02g02600	953027	CTGCATGCCTCAGAAAAACA	GCAAGCCCAAAATCAGAGAG	60.0	176	99	serine/threonine-protein kinase Cx32 chloroplast precursor putative expressed
OsISM11288	LOC_Os02g02600	953266	TGCAGCACCGGAGTATGTAG	AGCATCACAACTCCGAAACC	60.0	824	73	serine/threonine-protein kinase Cx32 chloroplast precursor putative expressed
OsISM11289	LOC_Os02g02620	961685	GTCTACCTCTCCGGCCTCTC	ACCACACACTTGCTGAAGCC	60.8	187	99	sialyltransferase family domain containing protein expressed
OsISM11290	LOC_Os02g02620	961810	GTTTCCGATGCGGACTTG	CTCCTGGAATTGCTTTCAGC	60.1	463	95	sialyltransferase family domain containing protein expressed
OsISM11291	LOC_Os02g02620	962253	GCTGAAAGCAATTCCAGGAG	TCAGGTTCTCCAGTTTTTGGA	59.8	860	97	sialyltransferase family domain containing protein expressed
OsISM11292	LOC_Os02g02620	963139	GTGAAGCTGTGGCTTCATGG	CATGCATACTCCTCCCAACC	61.1	283	98	sialyltransferase family domain containing protein expressed
OsISM11293	LOC_Os02g02620	963419	ATGGCGCAGGATCAATAAAG	GCAAAGAGTCCTGTTCTGGC	60.0	149	56	sialyltransferase family domain containing protein expressed
OsISM11294	LOC_Os02g02620	963701	TCGTTATTAGAGAGAACGGTG C	GCCTTTGCAGATCCTCTGTT	59.0	174	95	sialyltransferase family domain containing protein expressed
OsISM11295	LOC_Os02g02620	963855	AACAGAGGATCTGCAAAGGC	TGTCTTAACGATAAGGGCTTCC	59.5	147	76	sialyltransferase family domain containing protein expressed
OsISM11296	LOC_Os02g02620	963984	GCCCTTATCGTTAAGACAACA A	AAACGATGTGCCTAGCATGA	58.8	190	97	sialyltransferase family domain containing protein expressed
OsISM11297	LOC_Os02g02620	964248	TGGATTTACAGTTGACCCAGG	GGAGTGTGTCCCTTTCTGGA	60.0	154	70	sialyltransferase family domain containing protein expressed
OsISM11298	LOC_Os02g02620	964378	GGAGTCCAGAAAGGGACACA	CCTCGCATAGGTGAGTGGAT	60.1	220	94	sialyltransferase family domain containing protein expressed
OsISM11299	LOC_Os02g02620	964642	TTGAAGCTGCTAGGGTTGCT	ATGGTGCAAGAGCTCAAAGG	60.3	288	80	sialyltransferase family domain containing protein expressed
OsISM11300	LOC_Os02g02640	967180	TGGTTCTATTGACCTTGGGG	AACCCCATTATCCACACTGC	59.7	1185	97	NBS-LRR disease resistance protein putative

ISM IDs	Rice gene locus IDs	Physical positions (bp) on chromosomes	Forward primers (5'-3')	Reverse primers (5'-3')	Melting tempearature (T˚C)	Expected product size (bp) in PCR	Expected product size (bp) in Q-PCR	Putative Functions
OsISM11301	LOC_Os02g02640	968625	ATTACTACCGGCTCCAGCAA	AGATGGAGTGGATGAAGACACA	59.7	379	99	NBS-LRR disease resistance protein putative
OsISM11302	LOC_Os02g02640	968982	TGTGTCTTCATCCACTCCATC	AATCTCAAGAGGCAGCATGG	59.4	141	92	NBS-LRR disease resistance protein putative
OsISM11303	LOC_Os02g02640	969660	GGATGGGACAAGCTGTTAGC	TTCCCAATGGATTGCAAAGT	60.0	221	99	NBS-LRR disease resistance protein putative
OsISM11304	LOC_Os02g02640	971494	ACAAACTCATCGTCGGTCGT	GGAAGGATCTTCCATGCTCT	59.4	135	98	NBS-LRR disease resistance protein putative
OsISM11305	LOC_Os02g02640	972475	GCTACAAATTAGGGATTGCCC	TGATTGCTGCCTTGTTCTTG	59.9	369	61	NBS-LRR disease resistance protein putative
OsISM11306	LOC_Os02g02640	973236	TAGGATTACACCGGGATGGA	TCACGAGTCCACACTTGCAT	60.2	1117	86	NBS-LRR disease resistance protein putative
OsISM11307	LOC_Os02g02660	978386	CGAGCTTGATTACTACCGGC	AAGATACACGGCTTCCCTCA	59.8	421	90	retrotransposon protein putative unclassified expressed
OsISM11308	LOC_Os02g02660	980537	GAAGAGGCCATTGTCTGGAA	GAAAACAGATTGGGGCAAAA	60.1	129	86	retrotransposon protein putative unclassified expressed
OsISM11309	LOC_Os02g02660	980646	TTTTGCCCCAATCTGTTTTC	CCCAGATATCATCCAATACAAGC	59.8	444	96	retrotransposon protein putative unclassified expressed
OsISM11310	LOC_Os02g02660	981925	GTTCTTTGGTGAAAGCAGCA	CAAGTTGCAACACCAGCAAT	59.4	369	98	retrotransposon protein putative unclassified expressed
OsISM11311	LOC_Os02g02660	982938	TCTCAACAAGCTCACCATCG	AACTCCTTGAGTGCTCGCAT	60.0	169	85	retrotransposon protein putative unclassified expressed
OsISM11312	LOC_Os02g02660	984994	GGTCGGCATTTACTCTGCAT	CTCCCCTTCCATTCTTCTCC	60.1	684	69	retrotransposon protein putative unclassified expressed
OsISM11313	LOC_Os02g02660	985693	GCTCTGTAGGATGGACTGCC	ATCTCGTGTAGACGATTGCG	59.4	643	87	retrotransposon protein putative unclassified expressed
OsISM11314	LOC_Os02g02660	986297	AGACTTCTTGGTGGCTTGGC	TACCGATACATTCTGCGTGC	60.7	581	66	retrotransposon protein putative unclassified expressed
OsISM11316	LOC_Os02g02670	988606	TGACGTTGGTCAGTTCAAAAA	TAGGGTTTCATTCCTCGGTG	59.6	543	95	NBS-LRR disease resistance protein putative
OsISM11317	LOC_Os02g02670	989253	AAATCTCCGCCAAATGGACT	CTCCCAATCCATGATCCAAC	60.5	170	81	NBS-LRR disease resistance protein putative
OsISM11318	LOC_Os02g02670	989459	TGTTGGCAAGGTCATGTGAT	GCCAATTTCGTGAAAGCAAG	60.4	178	71	NBS-LRR disease resistance protein putative
OsISM11319	LOC_Os02g02670	989622	TTTCACGAAATTGGCACAAA	CTGTGGAAACCAATCCCATC	60.1	656	84	NBS-LRR disease resistance protein putative

ISM IDs	Rice gene locus IDs	Physical positions (bp) on chromosomes	Forward primers (5'-3')	Reverse primers (5'-3')	Melting tempearature (T˚C)	Expected product size (bp) in PCR	Expected product size (bp) in Q-PCR	Putative Functions
OsISM11320	LOC_Os02g02670	990461	TGTCCGAGTCACTGGATGAG	GCAGCACTAGAACCTTTCCC	59.4	411	80	NBS-LRR disease resistance protein putative
OsISM11321	LOC_Os02g02670	992430	TTACATACTTTATGCAAGGAG TCAA	AAAATTTTGTCAAAGCTTGAGG	57.4	203	89	NBS-LRR disease resistance protein putative
OsISM11322	LOC_Os02g02670	993277	TGTGTTGGGACTTACGGTCA	TCCAACTCCTTGAGTGCTCC	60.2	309	75	NBS-LRR disease resistance protein putative
OsISM11324	LOC_Os02g02680	1001317	TGATAGGTCACTGTTACCGCA	TTCGACAATAGGCACATCCA	59.6	183	99	NBS-LRR disease resistance protein putative expressed
OsISM11325	LOC_Os02g02680	1003188	TCCTGCAAATCTACGAAGCC	TCCTGGAATTGCTCTCATCC	60.3	564	68	NBS-LRR disease resistance protein putative expressed
OsISM11326	LOC_Os02g02680	1003776	CAGTACCCAAGCGACACAGA	TCAGGTTCTCCAGTTTTTGGA	59.8	284	54	NBS-LRR disease resistance protein putative expressed
OsISM11327	LOC_Os02g02680	1004088	GAAGCTGTAGCCTCATGGGA	TGAAGTGTGTCCCTTTCTGG	59.5	465	62	NBS-LRR disease resistance protein putative expressed
OsISM11328	LOC_Os02g02680	1004493	TTGATGTGATGATCTTTCAGG A	CCTCGCATAGGTGAGTGGAT	59.1	385	53	NBS-LRR disease resistance protein putative expressed
OsISM11329	LOC_Os02g02680	1004909	ACCAAGGACACCATTGAAGC	TCATGGTGCAAGAGCTCAAA	60.3	312	95	NBS-LRR disease resistance protein putative expressed
OsISM11331	LOC_Os02g02710	1017719	GATCTCCCGCACCCTCTAC	CACTCAACTTGTCTGGATGCC	60.2	672	84	zinc carboxypeptidase family protein putative expressed
OsISM11332	LOC_Os02g02710	1018368	CCGGCATCCAGACAAGTT	TGCTCTCCTTGGCATGATTA	59.5	196	99	zinc carboxypeptidase family protein putative expressed
OsISM11333	LOC_Os02g02710	1018556	GGAGAGCACGTCCAATTCTT	TGATAAGCTCTCTGCCATGC	59.2	328	69	zinc carboxypeptidase family protein putative expressed
OsISM11334	LOC_Os02g02710	1018937	GCTGGTGTCGATCTATCATCC T	CTTCAACACGTTTTCGACCA	60.1	665	95	zinc carboxypeptidase family protein putative expressed
OsISM11335	LOC_Os02g02710	1019582	TGGTCGAAAACGTGTTGAAG	TCCCCAATCAACACTCCAAT	60.0	173	89	zinc carboxypeptidase family protein putative expressed
OsISM11336	LOC_Os02g02710	1019742	TGTTGATTGGGGAAAGAAGG	CTAAAAGGAGCAGTGCCAGG	60.0	505	67	zinc carboxypeptidase family protein putative expressed
OsISM11337	LOC_Os02g02710	1020271	AGAGCTATCCAAGTCATTTAA ACC	CGTTTGGTGTAGTATTCTTATGG TCA	58.9	420	99	zinc carboxypeptidase family protein putative expressed
OsISM11338	LOC_Os02g02710	1020739	GCCATTTCCAAGATAGCTGC	ATCAGTTGTGGTGCCATGTG	60.1	221	75	zinc carboxypeptidase family protein putative expressed
OsISM11339	LOC_Os02g02710	1020980	GGTGCCTATGCCCTTTACCT	AGCAATCGCTGGTAGATGCT	60.2	152	60	zinc carboxypeptidase family protein putative expressed

ISM IDs	Rice gene locus IDs	Physical positions (bp) on chromosomes	Forward primers (5'-3')	Reverse primers (5'-3')	Melting tempearature (T˚C)	Expected product size (bp) in PCR	Expected product size (bp) in Q-PCR	Putative Functions
OsISM11340	LOC_Os02g02710	1021141	TCAATCCCGTTGACAAGACA	CGCAGTCCTTCCTCAAAAAG	60.0	192	83	zinc carboxypeptidase family protein putative expressed
OsISM11342	LOC_Os02g02750	1040879	TACATCCTCCAGCCCTTCTG	TGCGGGACTCTCACCATATT	60.3	859	78	CDP-alcohol phosphatidyltransferase putative expressed
OsISM11343	LOC_Os02g02750	1041689	TTTCCCATGTTGGAATCGAC	GGAATGCCGATGTCAATAGAA	60.3	168	76	CDP-alcohol phosphatidyltransferase putative expressed
OsISM11344	LOC_Os02g02750	1041840	ATTGACATCGGCATTCCTTG	GCTGTATCGAGATGAGGGGA	60.5	185	49	CDP-alcohol phosphatidyltransferase putative expressed
OsISM11345	LOC_Os02g02750	1042028	ACCTAGATGGGTGCACCTTG	TGTTTACCATCCACAGCATCA	60.0	165	73	CDP-alcohol phosphatidyltransferase putative expressed
OsISM11346	LOC_Os02g02750	1042211	ATCAAGTCCTTTAGGCGAGC	ATATTGGGACATGCAAGGGA	59.4	190	96	CDP-alcohol phosphatidyltransferase putative expressed
OsISM11347	LOC_Os02g02750	1042326	CTTGAACAGGATGTGATGCG	TAGCTTTTCCACACATTGCC	59.5	164	76	CDP-alcohol phosphatidyltransferase putative expressed
OsISM11348	LOC_Os02g02750	1042495	TGGTATTGGTTCATTGCTGC	CTTCAGTCGGTCCGTTGACT	59.9	455	98	CDP-alcohol phosphatidyltransferase putative expressed
OsISM11349	LOC_Os02g02750	1042930	AGTCAACGGACCGACTGAAG	ATCTTGTGCCCACCATTCA	60.1	254	86	CDP-alcohol phosphatidyltransferase putative expressed
OsISM11350	LOC_Os02g02750	1043185	TCCGGAAGTCAATACCCCTA	CGGAATTACAGCAAAAGCTATCA	60.0	303	99	CDP-alcohol phosphatidyltransferase putative expressed
OsISM11351	LOC_Os02g02750	1043475	TGCTGTAATTCCGACGATTG	TTTCTTGCCTCGACCACTTT	59.8	318	61	CDP-alcohol phosphatidyltransferase putative expressed
OsISM11352	LOC_Os02g02750	1043774	AAGTGGTCGAGGCAAGAAAA	TACCAGACAAGAACTCCGGC	60.1	177	89	CDP-alcohol phosphatidyltransferase putative expressed
OsISM11353	LOC_Os02g02750	1043899	TGTGCCAGCTCTTTCCTTTT	GAAGATGTGGCTGGTTTCTCA	60.1	246	94	CDP-alcohol phosphatidyltransferase putative expressed
OsISM11354	LOC_Os02g02750	1044157	CTGGTTTTGCATTTGGCTTT	GACCTTTGGGCTCATCACAT	60.0	494	67	CDP-alcohol phosphatidyltransferase putative expressed
OsISM11355	LOC_Os02g02750	1044640	GCCCAAAGGTCTGAAGACAG	TAGGCACAAACCCATCATCA	59.9	215	99	CDP-alcohol phosphatidyltransferase putative expressed
OsISM11356	LOC_Os02g02750	1044781	GCAGTCCCTAGCATATTTCCC	TAGCTGCTCATCAACAAGGG	59.5	171	86	CDP-alcohol phosphatidyltransferase putative expressed
OsISM11357	LOC_Os02g02750	1044932	CCCTTGTTGATGAGCAGCTA	AGCATTGGTGATTTCATGGAT	59.1	263	99	CDP-alcohol phosphatidyltransferase putative expressed
OsISM11358	LOC_Os02g02750	1045189	AATGCTCTCGGGATTCATTG	AAGGCAATTGTGGATGGTTC	59.9	154	70	CDP-alcohol phosphatidyltransferase putative expressed

ISM IDs	Rice gene locus IDs	Physical positions (bp) on chromosomes	Forward primers (5'-3')	Reverse primers (5'-3')	Melting tempearature (T°C)	Expected product size (bp) in PCR	Expected product size (bp) in Q-PCR	Putative Functions
OsISM11359	LOC_Os02g02790	1062914	GTCTGGGACGACAAGACCTC	CCTCGTCATGTGTTTGCATC	59.9	153	71	C2 domain containing protein expressed
OsISM11360	LOC_Os02g02790	1063118	GATACCCAAGCAGCAAGCTC	TACATCGTCCCCACAATCTG	59.7	619	60	C2 domain containing protein expressed
OsISM11361	LOC_Os02g02790	1064162	ACAGCCACCTCTACGCCTT	ACGAGGCAATCCTTTCACTG	60.1	173	96	C2 domain containing protein expressed
OsISM11362	LOC_Os02g02790	1064315	CAGTGAAAGGATTGCCTCGT	GTCACGTTCTTGTCGCACAC	60.3	465	74	C2 domain containing protein expressed
OsISM11363	LOC_Os02g02790	1064858	CTTCATCGGATCATCCTTCG	ATCTCCATCGGCACCAGATT	61.2	417	73	C2 domain containing protein expressed
OsISM11364	LOC_Os02g02800	1067159	CAAGCACTACTTCGGCGG	GCCAGACAAACATCCCGTAA	60.7	461	96	AGAP001222-PA putative expressed
OsISM11365	LOC_Os02g02800	1067665	GCTTCACTGCCTCCACAGTT	TGCGATGTCTGTCAGTGTGA	60.2	314	99	AGAP001222-PA putative expressed
OsISM11366	LOC_Os02g02800	1067943	AAGGTTGGAGCAGACGTCAC	CCGCATACTTGTCTGATGTTG	59.8	157	75	AGAP001222-PA putative expressed
OsISM11367	LOC_Os02g02800	1068079	CAACATCAGACAAGTATGCGG	CCCATTCTCCCCAGGTAAGT	59.7	241	69	AGAP001222-PA putative expressed
OsISM11368	LOC_Os02g02800	1068360	AGCCGATGTGCTTTACGACT	CTGTCGCGAAGAGATCATCA	60.0	145	48	AGAP001222-PA putative expressed
OsISM11369	LOC_Os02g02800	1068532	GGGCGATGTTCATAACCACA	CCATCCCGAAGCTTTAAACA	60.9	196	98	AGAP001222-PA putative expressed
OsISM11370	LOC_Os02g02830	1078589	TCAAGGACCTTCAGAGGGAT	CATGATTGTTGCTTGCCAGT	59.2	213	84	ubiquitin-conjugating enzyme putative expressed
OsISM11371	LOC_Os02g02830	1078822	CTCTGGAGGAGTGTTCCTGG	GTTTGGATGGAACACCTTGG	60.0	227	95	ubiquitin-conjugating enzyme putative expressed
OsISM11372	LOC_Os02g02830	1079066	CATCTGCCTTGACATCCTCA	AGCGAGCAGATCGACAGAAG	60.3	185	76	ubiquitin-conjugating enzyme putative expressed
OsISM11373	LOC_Os02g02920	1135581	TAGGGCTCAAGATGGTCGTC	TTGTAGCAATCATAGGCTGGG	60.2	179	83	HVA22 putative expressed
OsISM11374	LOC_Os02g02920	1135765	TGGAGCTGAACAGACCTGAG	TCCCCAACTCTCTCAAAAACA	59.4	181	92	HVA22 putative expressed
OsISM11375	LOC_Os02g02920	1135925	TGTTTTTGAGAGAGTTGGGGA	AATGCCAGCTTTGCTTCACT	59.9	161	67	HVA22 putative expressed
OsISM11376	LOC_Os02g02920	1136072	GCAAAGCTGGCATTTATCGT	TCATGTTTGCCAATGTACGG	60.3	164	96	HVA22 putative expressed

ISM IDs	Rice gene locus IDs	Physical positions (bp) on chromosomes	Forward primers (5'-3')	Reverse primers (5'-3')	Melting tempearature (T°C)	Expected product size (bp) in PCR	Expected product size (bp) in Q-PCR	Putative Functions
OsISM11377	LOC_Os02g02920	1136353	TATTGCTTCGCAGTCAGAGG	GAGTCCGTGGAGGTGGTG	59.6	2249	78	HVA22 putative expressed
OsISM11378	LOC_Os02g02930	1146096	CTACCCACCGACTTCGACAT	CAGCATCTCCTGCACGTC	59.5	637	73	terpene synthase putative expressed
OsISM11379	LOC_Os02g02930	1146919	CGCCTCATGAGAGAAGCTG	GTAATCGAGCCTGAATTCGC	59.8	443	88	terpene synthase putative expressed
OsISM11380	LOC_Os02g02930	1147656	CATCAGATGGAGATGCAGGA	TCCCTGGCAACTGGTATTTC	59.8	1413	75	terpene synthase putative expressed
OsISM11381	LOC_Os02g02930	1149211	CTCTCCTGCTTCACTCAGGC	TGTGTGTAGAGCCCTGAAGC	59.7	428	94	terpene synthase putative expressed
OsISM11382	LOC_Os02g02930	1149650	CATCGCAGATATGGTCGAGA	TCATGAATCCATCAAACAGCA	59.9	222	87	terpene synthase putative expressed
OsISM11383	LOC_Os02g02930	1150020	TGATCACATCCCTCCTGTCA	TCCAATCCTTCTTGTGCCTC	60.1	559	94	terpene synthase putative expressed
OsISM11384	LOC_Os02g02940	1153025	TTGCTCCCTTCAGAGATGCT	GCTGAAAGATGAGCCTGAGC	60.2	747	66	succinate dehydrogenase subunit 3 putative expressed
OsISM11386	LOC_Os02g02970	1163258	GCTGAGCTTCTTCTCGCATT	AATTGGAACTCGAGCACTGG	60.1	375	85	expressed protein
OsISM11387	LOC_Os02g02970	1163804	TATCGTGGAACCCTTTCGAG	ATCTTGGGCTCTCTTTGCTG	59.8	173	72	expressed protein
OsISM11388	LOC_Os02g02970	1163957	CAGCAAAGAGAGCCCAAGAT	CATTCAGTGCCATCGACAAC	59.8	608	78	expressed protein
OsISM11389	LOC_Os02g02970	1164657	GGGCATTCCATCACGATTAC	GCGAGTCGTCCAGTTCTTCT	59.9	220	87	expressed protein
OsISM11390	LOC_Os02g03030	1191096	CGCACTCCAAGAAATCACAC	AATCCACGATCTTCGTCTGG	59.7	172	70	ATXR putative
OsISM11391	LOC_Os02g03030	1191482	CAACGAGCTCACCTACGTCC	TCGACGTCCTCCTTTGGTAG	60.6	186	97	ATXR putative
OsISM11392	LOC_Os02g03030	1191932	CATCAATGGCATCAACAACC	GGACGCACTTGAGATTCTGC	60.4	405	60	ATXR putative
OsISM11393	LOC_Os02g03060	1208038	GAGATCTCGCTGCTCAAGGA	CGAGGTACTCGAAGACGAGG	60.4	230	98	cyclin-dependent kinase A-2 putative expressed
OsISM11394	LOC_Os02g03060	1208303	GATTTCAAGAACCACCGCAT	AAATCTCGGTGGAGAACACG	60.0	260	93	cyclin-dependent kinase A-2 putative expressed
OsISM11395	LOC_Os02g03060	1208635	ATTTGGCATTCCTGTCCG	CAACAGGGGTGGAATAATGC	59.8	1020	99	cyclin-dependent kinase A-2 putative expressed

ISM IDs	Rice gene locus IDs	Physical positions (bp) on chromosomes	Forward primers (5'-3')	Reverse primers (5'-3')	Melting tempearature (T˚C)	Expected product size (bp) in PCR	Expected product size (bp) in Q-PCR	Putative Functions
OsISM11396	LOC_Os02g03060	1209684	GAAATGGTGAATCAGAAGCCA	TTTCTTCATTTGGAGTGCCC	60.1	203	92	cyclin-dependent kinase A-2 putative expressed
OsISM11397	LOC_Os02g03060	1209890	GGCCAGGTGTTGCTTCACTA	AATCAAGTGTTGGGACCACG	61.0	266	88	cyclin-dependent kinase A-2 putative expressed
OsISM11398	LOC_Os02g03060	1210136	CGTGGTCCCAACACTTGATT	GCTTGGATCTAAACGGAGCA	60.6	686	65	cyclin-dependent kinase A-2 putative expressed
OsISM11399	LOC_Os02g03070	1214224	CCATGGAGGCATACAAGCTC	AAGCGCTCAGGAAGTATCCA	60.3	542	98	SSE1 putative expressed
OsISM11400	LOC_Os02g03070	1214749	ATACTTCCTGAGCGCTTTGC	GGCGTTTCAATTATGTGCTG	59.4	1401	99	SSE1 putative expressed
OsISM11401	LOC_Os02g03070	1216248	CCCAGCACTTTGTTGGAGAT	CCGAAAGCAGCTAACCTGAC	60.1	192	86	SSE1 putative expressed
OsISM11402	LOC_Os02g03070	1216701	GTGGATGAAGAAGGCTCTGC	CTTGCCAAAGTTGGCTTCTC	60.0	190	67	SSE1 putative expressed
OsISM11403	LOC_Os02g03070	1217083	AGGGGGAAAAGTTCATCAGC	CCACATCATCTTTCGCCTTT	60.3	164	65	SSE1 putative expressed
OsISM11404	LOC_Os02g03070	1217237	GATGATGTGGGCCCTTTATG	GGCACTGGATCCAACACTTT	60.1	182	94	SSE1 putative expressed
OsISM11405	LOC_Os02g03070	1217399	AAAGTGTTGGATCCAGTGCC	TATATCGTGTCTGAGCCCCC	59.9	180	80	SSE1 putative expressed
OsISM11406	LOC_Os02g03080	1219306	ATGGCATCTCGTTTCTGGG	ATCTCCTGCTCCTCCTCCTC	60.5	185	57	eukaryotic translation initiation factor 3 subunit C putative expressed
OsISM11407	LOC_Os02g03080	1221234	ACAAGGTGTCCAACAAAGCC	CTGCCTTCTTCTCTCAAGCC	59.7	565	68	eukaryotic translation initiation factor 3 subunit C putative expressed
OsISM11409	LOC_Os02g03120	1237773	GTCTCCGACCCCTCCTACTC	GTCGCTGTATGATCCCCTGT	60.0	322	88	endoglucanase putative expressed
OsISM11410	LOC_Os02g03120	1238117	CGTTCTACTGCTCCCACTCC	CGACAGGTACACGTCCTTCTT	59.6	641	93	endoglucanase putative expressed
OsISM11411	LOC_Os02g03120	1238800	GACTTCACCTTCAGCTGGGA	GTTGTCAGTGTGCGCTTTGT	60.2	191	97	endoglucanase putative expressed
OsISM11412	LOC_Os02g03120	1238991	TACATCTGCTCCCTTGTCCC	ATGTTGCTGTCGCCTTCTTT	60.0	503	87	endoglucanase putative expressed
OsISM11413	LOC_Os02g03120	1239593	CTCCACCCTCATCTCCCTC	GATTCGCCCCGAGTATGTAG	59.6	141	54	endoglucanase putative expressed
OsISM11414	LOC_Os02g03130	1245634	CTGCTTTGCACCATGAACAC	GTCCTCTCTTGGAGAAGGGG	60.2	342	83	conserved hypothetical protein

ISM IDs	Rice gene locus IDs	Physical positions (bp) on chromosomes	Forward primers (5'-3')	Reverse primers (5'-3')	Melting tempearature (T˚C)	Expected product size (bp) in PCR	Expected product size (bp) in Q-PCR	Putative Functions
OsISM11415	LOC_Os02g03130	1245956	CCCCTTCTCCAAGAGAGGAC	TCACTGAATTGTAACCGCTCC	60.2	326	84	conserved hypothetical protein
OsISM11416	LOC_Os02g03130	1246242	TTTGTTGTCCATGCAGGAAG	CAGTGCATACCATCAGGATTCA	60.3	252	90	conserved hypothetical protein
OsISM11417	LOC_Os02g03130	1246447	GCAGGAAGGAGTGGGTACAA	CATCCTCAGCCCATACCATC	60.2	259	84	conserved hypothetical protein
OsISM11418	LOC_Os02g03130	1246660	GGAGCGGCTACAATTCAGTC	CGGTTCATACCATTCGGATT	59.7	255	71	conserved hypothetical protein
OsISM11419	LOC_Os02g03130	1246879	TGGTTACAATTCAGTCAATCCG	GTATTTGCATCGTCCTCGGT	59.9	253	83	conserved hypothetical protein
OsISM11420	LOC_Os02g03130	1247059	TGTTGTCCTTGTAGGGAGGG	GCCGTTTCTAGGGACAGAAT	59.1	213	80	conserved hypothetical protein
OsISM11421	LOC_Os02g03160	1256514	TGCCGGCTATCCTTATGAAC	CCGGAGTTTCTTAATTTCCTGA	59.8	130	81	retrotransposon protein putative Ty1-copia subclass
OsISM11422	LOC_Os02g03160	1256733	CGAAGGAGATGGGTTTGAGA	AATCTTCATGGACTGCGACC	60.1	298	83	retrotransposon protein putative Ty1-copia subclass
OsISM11423	LOC_Os02g03220	1283508	TCTTCCCAAGTTGTTGGACC	CAAAACAGACCTTCGCTCGT	60.2	607	98	protein binding protein putative expressed
OsISM11424	LOC_Os02g03220	1284295	AATGCTTTCTCTGGGATTGC	CGTAATGCATCAAGCACAGG	59.8	203	99	protein binding protein putative expressed
OsISM11425	LOC_Os02g03220	1284534	ATTGAACCCAGAGGAGTCCC	CCATTTACATCCTTGCTTCCA	60.1	823	94	protein binding protein putative expressed
OsISM11426	LOC_Os02g03220	1285571	GAGTTTGGTGATTTTAAGCTCG	TCCTCAATTACTGACTTGGCTG	58.7	423	93	protein binding protein putative expressed
OsISM11427	LOC_Os02g03220	1286338	ACAGATCTCGTGGGCTTCAG	ATGCAAACTCTGTGGCCTCT	60.1	330	72	protein binding protein putative expressed
OsISM11428	LOC_Os02g03220	1286816	CAAAAAGATCAGGGAACCGA	CACTATCGCACTTGATTATTCTGA	59.3	175	71	protein binding protein putative expressed
OsISM11429	LOC_Os02g03240	1295273	GTGGCACTAGGGCTGTCGT	TTCCACCACTCTACCACCGT	60.9	810	66	transposon protein putative unclassified
OsISM11430	LOC_Os02g03240	1296127	AACCATTTTGTTTCCCACCC	TTCTTCTCTCCTTCCCCCTC	60.3	377	97	transposon protein putative unclassified
OsISM11431	LOC_Os02g03240	1298391	GGTCACAGAGAGGGGTGGTA	AGAACTGATACGGCATTCGG	60.0	194	97	transposon protein putative unclassified
OsISM11433	LOC_Os02g03250	1302735	GCTCGGCTTGATCATCCTC	CACGAAAAAGCTGTCCCATT	60.5	630	80	expressed protein

ISM IDs	Rice gene locus IDs	Physical positions (bp) on chromosomes	Forward primers (5'-3')	Reverse primers (5'-3')	Melting tempearature (T°C)	Expected product size (bp) in PCR	Expected product size (bp) in Q-PCR	Putative Functions
OsISM11434	LOC_Os02g03270	1314608	CGGTGGTCGTCTCGTCTC	CCAGAAGCACATGCTACCAA	60.1	1073	77	AT hook motif domain containing protein expressed
OsISM11435	LOC_Os02g03270	1315679	GGTGCAAACTTCACACCTCA	TGGACCTTGTTGGGAGAAAG	59.9	197	85	AT hook motif domain containing protein expressed
OsISM11436	LOC_Os02g03270	1315896	TGCTAATGGGGTGATTTCAA	ATCCGGATAAGGAGAGCAGC	59.8	1716	99	AT hook motif domain containing protein expressed
OsISM11437	LOC_Os02g03270	1317692	GTCATCGGAGGCGGAGTT	TCTGGTCCAGCTGGTAGCTC	60.9	639	92	AT hook motif domain containing protein expressed
OsISM11438	LOC_Os02g03294	1331773	GTCTGCTCATCACCAGCGT	CGCGAAGTATGCGTTGTTGT	61.1	1109	72	cyclin putative expressed
OsISM11440	LOC_Os02g03310	1335859	TGTCATGTTTCAGGTAGATTCTGA	CTGCTTGAGATTCTTCTGTTGC	59.0	271	93	KH domain containing protein putative expressed
OsISM11441	LOC_Os02g03310	1336310	TTCAAGAAACTACCGGTGCTA	GGAGCACCATGTATGTCCAC	58.2	481	98	KH domain containing protein putative expressed
OsISM11442	LOC_Os02g03310	1336847	TTGATCACGGTGTGCTTCAC	TGTGCCTCACCTACTGCTTG	60.4	145	56	KH domain containing protein putative expressed
OsISM11443	LOC_Os02g03310	1337068	CCTGACCATCGAACTCCTCT	TGTCCCCTTGCACCAATTAT	59.7	419	90	KH domain containing protein putative expressed
OsISM11444	LOC_Os02g03310	1337556	TGAGGTTTTAGTTATGATCAAAGGC	GGTCGCTCTTTATTGCCTGA	60.1	889	64	KH domain containing protein putative expressed
OsISM11445	LOC_Os02g03320	1341674	AGCCAATTGGAACTGTTGCT	GCCAATGCACTAGGAACAAAA	59.9	351	92	plant neutral invertase domain containing protein expressed
OsISM11446	LOC_Os02g03320	1342394	TGATGGCTGTTCCATGATTG	GAGCCTGGATATCAATCGGA	60.2	669	63	plant neutral invertase domain containing protein expressed
OsISM11448	LOC_Os02g03330	1345059	ACCTCAGGCAGTTCGAGAAG	CAAGCCATTTCCTTCTCCTG	59.7	1194	79	expressed protein
OsISM11449	LOC_Os02g03330	1346326	GATGAAGCGTTTCCATCTCC	ATGCCATGAAGAAGCCAATC	59.8	212	72	expressed protein
OsISM11450	LOC_Os02g03350	1354795	GTCCATCTGGACAGGCAAGT	GTGGTTCCTGTCAATCCCAC	60.2	460	75	transposon protein putative unclassified
OsISM11452	LOC_Os02g03350	1355766	CTAGCAAGGCTTGGTTGCTC	AACCACAACAGGTACGCCTC	60.1	184	98	transposon protein putative unclassified
OsISM11453	LOC_Os02g03350	1355891	CACCCTGACGCTGATGATTA	AAACTCGAACACGAAGTCCG	60.0	548	79	transposon protein putative unclassified
OsISM11454	LOC_Os02g03350	1356621	CGGACTTAGGCAGGTGTTTC	CTAGCTGTCCCCGTCGAGT	60.1	159	81	transposon protein putative unclassified

ISM IDs	Rice gene locus IDs	Physical positions (bp) on chromosomes	Forward primers (5'-3')	Reverse primers (5'-3')	Melting tempearature (T˚C)	Expected product size (bp) in PCR	Expected product size (bp) in Q-PCR	Putative Functions
OsISM11455	LOC_Os02g03350	1356988	GCACCGTGACCGAGACTACT	ATATCGGCACTGATATCCCG	60.0	152	58	transposon protein putative unclassified
OsISM11456	LOC_Os02g03350	1357321	GGAGCAGGACCTCGACCTAC	ACGCGCTCGAAAGCAGAC	62.0	307	59	transposon protein putative unclassified
OsISM11457	LOC_Os02g03350	1357696	AGCTCTTCCTCTCACGGAGC	AGACGAAGAGGCCCCAGTA	60.5	144	82	transposon protein putative unclassified
OsISM11458	LOC_Os02g03360	1360601	GTGATCAGCCTCCCAAGTCT	TTGATCCATCGAACAAACTCC	59.6	2434	84	expressed protein
OsISM11459	LOC_Os02g03360	1363051	CGATGGATCAATATATGACAC TGG	CCCATCCTGGTCAGTCATTT	60.1	405	91	expressed protein
OsISM11460	LOC_Os02g03370	1366570	CGCAACCTCACAGCGTATT	GTCACTGACCCCACCACAAT	60.3	1331	55	hypothetical protein
OsISM11461	LOC_Os02g03370	1367855	TTGATTTGTTACAGGTGGGG	GGTCAAGCATTTGCTCCAGT	59.3	822	83	hypothetical protein
OsISM11462	LOC_Os02g03370	1368716	ACAGCTCTGACCGTGGCTAT	GGGTGTTCCTTCACAGCATT	59.9	261	60	hypothetical protein
OsISM11463	LOC_Os02g03380	1370043	CCGTATTCTTCCTCTGCGAC	GCTGCGAACATGTCAGAGAC	59.7	1654	72	retrotransposon protein putative unclassified
OsISM11464	LOC_Os02g03380	1372313	CAGGATTGGTGGTCATCCTC	ACTTTGTCCAACGCAAGGTC	60.2	534	72	retrotransposon protein putative unclassified
OsISM11465	LOC_Os02g03380	1372794	CAGGTGAATGAGCCACTTGA	GAAGCATTTCCACCCTCTGA	60.0	617	84	retrotransposon protein putative unclassified
OsISM11466	LOC_Os02g03380	1373385	GACCTGTCAGAGGGTGGAAA	CCATCATTGTCGTTATGCCA	60.2	701	98	retrotransposon protein putative unclassified
OsISM11467	LOC_Os02g03390	1375846	GATCCCACGCCCATCTATCT	AGGCATCCTTGGTCAGAGAA	60.5	360	77	expressed protein
OsISM11468	LOC_Os02g03390	1376237	CAAGACGCATCTCAGGATCA	AATGTAGCCAGCCATTTTGC	60.0	1223	84	expressed protein
OsISM11469	LOC_Os02g03410	1388307	GTCAAGCGCATCGACAAG	ACATCCTCCACAGCAACAGG	60.1	2074	51	CAMK CAMK like.12 - CAMK includes calcium/calmodulin depedent protein kinases expressed
OsISM11470	LOC_Os02g03410	1390441	CAATGCGTTTGAAGATGACTC	TACTTTGCCAAAATCCGGTC	59.2	444	85	CAMK CAMK like.12 - CAMK includes calcium/calmodulin depedent protein kinases expressed
OsISM11471	LOC_Os02g03410	1390850	GGTGGTGAACTATTGGACCG	CCACTGCAGCATCTTTCTCA	60.2	215	68	CAMK CAMK like.12 - CAMK includes calcium/calmodulin depedent protein kinases expressed
OsISM11472	LOC_Os02g03410	1391079	CTCAAAGTGGCAGCTGAGTG	GGTGAGTCCTCCTTGGTTGA	59.9	197	93	CAMK CAMK like.12 - CAMK includes calcium/calmodulin depedent protein kinases expressed

ISM IDs	Rice gene locus IDs	Physical positions (bp) on chromosomes	Forward primers (5'-3')	Reverse primers (5'-3')	Melting tempearature (T˚C)	Expected product size (bp) in PCR	Expected product size (bp) in Q-PCR	Putative Functions
OsISM11473	LOC_Os02g03410	1391256	TCAACCAAGGAGGACTCACC	GCACTGCCAACTATATCGTGAA	60.1	216	93	CAMK CAMK like.12 - CAMK includes calcium/calmodulin depedent protein kinases expressed
OsISM11474	LOC_Os02g03410	1391551	ATTTTGCTCTGTGGGAGACG	GCCAAGGCTTCTTACGAAAA	59.9	992	98	CAMK CAMK like.12 - CAMK includes calcium/calmodulin depedent protein kinases expressed
OsISM11475	LOC_Os02g03410	1392590	AAGAACCCAAGGGCAAGATT	GGGATCTCAGATGCTTCTCCT	59.9	184	81	CAMK CAMK like.12 - CAMK includes calcium/calmodulin depedent protein kinases expressed
OsISM11476	LOC_Os02g03410	1392812	TCAAGTACAGCCGTTTTAAGCA	TCAATTGCATCGAACTGGTC	59.8	179	98	CAMK CAMK like.12 - CAMK includes calcium/calmodulin depedent protein kinases expressed
OsISM11477	LOC_Os02g03410	1392968	AAGGACCAGTTCGATGCAAT	CCCTTCAATCTCCAAGGAAG	59.1	185	99	CAMK CAMK like.12 - CAMK includes calcium/calmodulin depedent protein kinases expressed
OsISM11478	LOC_Os02g03410	1393156	CCGTGTTCTCGAGATTATCCA	TATGGAGGGTTGCTGCTACA	59.7	188	84	CAMK CAMK like.12 - CAMK includes calcium/calmodulin depedent protein kinases expressed
OsISM11479	LOC_Os02g03410	1393414	TCTGGATGGTGACGGATACA	GAACCCTTCAAGCCAGTGTG	60.3	426	67	CAMK CAMK like.12 - CAMK includes calcium/calmodulin depedent protein kinases expressed
OsISM11480	LOC_Os02g03430	1397700	TCTGTTTTGCCCTCTTTGCT	TCCGGTTGGTATACTGCTCC	60.0	302	84	expressed protein
OsISM11481	LOC_Os02g03430	1398024	AGGACAAGAAAACCGACAGC	ATCTTGACCAGGTAGCACGG	59.7	178	87	expressed protein
OsISM11482	LOC_Os02g03430	1398174	TTTCAACACCGTGCTACCTG	GGTCTTCGTTGATCAGTTTCC	59.2	361	96	expressed protein
OsISM11483	LOC_Os02g03450	1403009	CGACATCCTCCACGAGTTC	CTGATGGAGCTCAGATTCCC	59.5	155	61	vesicle transport v-SNARE protein putative expressed
OsISM11484	LOC_Os02g03450	1403187	CTGTGCGGGACGACATTACT	TAATGCTGCCATGAATCGAG	60.5	210	94	vesicle transport v-SNARE protein putative expressed
OsISM11485	LOC_Os02g03450	1403377	CTCGATTCATGGCAGCATTA	AAACAATGCCCTCTGATTGG	59.9	540	89	vesicle transport v-SNARE protein putative expressed
OsISM11486	LOC_Os02g03450	1403940	GCAGTTGGGTGAAAAGTTCC	GCGATCACTGCTGAAAGGAT	60.0	287	94	vesicle transport v-SNARE protein putative expressed
OsISM11487	LOC_Os02g03470	1412375	CCTCCTCAACGACTCCTCC	CACTGCCACCTGCTCATACA	60.3	443	71	tetratricopeptide repeat containing protein putative expressed
OsISM11488	LOC_Os02g03470	1412788	GAGTGGACGCTGTATGAGCA	CCAGGAAACTGCTTGGAAAG	59.9	262	99	tetratricopeptide repeat containing protein putative expressed
OsISM11489	LOC_Os02g03470	1413029	GCTTTCCAAGCAGTTTCCTG	GCATCAGTCCATTCACCCTT	60.0	795	82	tetratricopeptide repeat containing protein putative expressed
OsISM11490	LOC_Os02g03470	1413831	GCCTATGCGCTTATCCTTGA	CATGTCACCCTGTGCTTTTG	60.2	241	85	tetratricopeptide repeat containing protein putative expressed

ISM IDs	Rice gene locus IDs	Physical positions (bp) on chromosomes	Forward primers (5'-3')	Reverse primers (5'-3')	Melting tempearature (T°C)	Expected product size (bp) in PCR	Expected product size (bp) in Q-PCR	Putative Functions
OsISM11491	LOC_Os02g03470	1414047	TATTGCAAAAGCACAGGGTG	CAAGCATCATGATCTGCCAT	59.7	162	85	tetratricopeptide repeat containing protein putative expressed
OsISM11492	LOC_Os02g03470	1414192	GCAGATCATGATGCTTGGAG	GGGCCAATATTAGCTCCTCA	59.2	174	95	tetratricopeptide repeat containing protein putative expressed
OsISM11493	LOC_Os02g03470	1414352	GCTAATATTGGCCCAACCAA	TTTTCCAAACCACCCATTGT	59.9	905	79	tetratricopeptide repeat containing protein putative expressed
OsISM11494	LOC_Os02g03470	1415294	CAGTTGACTGGAGGCAAGAA	TCAACTGATTGATTGCCGAG	59.4	175	71	tetratricopeptide repeat containing protein putative expressed
OsISM11495	LOC_Os02g03550	1444348	GGCTTCGACAACATCCTCC	TGGTACATGGACGACGACAT	60.2	218	96	glycosyl hydrolases family 16 putative expressed
OsISM11496	LOC_Os02g03550	1444591	AAGCTCCCCTCCGACTACAC	GAACTCGAAGTCCAGCTCGT	60.1	912	97	glycosyl hydrolases family 16 putative expressed
OsISM11497	LOC_Os02g03560	1447413	AGGATCCGCCACTTCGTCT	TGAAACCGCAACATTGAAAA	61.1	1314	55	expressed protein
OsISM11499	LOC_Os02g03580	1457948	AAGGCAACAAAGGCATCAGA	TGGAGCTGTAGTGCCAGATG	60.4	508	61	transcription factor putative expressed
OsISM11500	LOC_Os02g03580	1458452	TCCAGTTTACCCTGAATGGC	AGCAACAGGAGGTGGAAAGA	59.9	189	77	transcription factor putative expressed
OsISM11501	LOC_Os02g03580	1458621	TCTTTCCACCTCCTGTTGCT	TATAAGGTGGTGGTGTCCCG	60.2	575	94	transcription factor putative expressed
OsISM11502	LOC_Os02g03580	1459210	GGAACTGTATATGCACATCCCTC	TTCAACATTTCCATTTGCCA	59.8	179	79	transcription factor putative expressed
OsISM11503	LOC_Os02g03580	1459369	TGGCAAATGGAAATGTTGAA	CCTTGATGTTGACATTGCAGA	59.8	161	84	transcription factor putative expressed
OsISM11504	LOC_Os02g03580	1459461	TTCTTTCAGGGAACTGCACC	CAGATGTTCTGCCAAGCTCA	60.2	143	65	transcription factor putative expressed
OsISM11505	LOC_Os02g03580	1459586	AGCTTGGCAGAACATCTGGT	TGAGAGTTGGCATCACTTCCT	59.9	172	95	transcription factor putative expressed
OsISM11506	LOC_Os02g03580	1459737	AGGAAGTGATGCCAACTCTCA	CCATCTTCCTTTACATCATTTTCC	60.0	182	61	transcription factor putative expressed
OsISM11507	LOC_Os02g03580	1459873	TGCTCCAGGATTCACATTCA	GCCATCGACATGTTCTGATTT	60.1	190	98	transcription factor putative expressed
OsISM11508	LOC_Os02g03580	1460187	GGTTCAGTTCGAGGAGAGCA	ACGTGCTGATTCCCGATTAG	60.3	426	79	transcription factor putative expressed
OsISM11509	LOC_Os02g03580	1460595	AATCGGGAATCAGCACGTAG	TTGTCAGCGCGTACAGAAAG	60.1	168	72	transcription factor putative expressed

ISM IDs	Rice gene locus IDs	Physical positions (bp) on chromosomes	Forward primers (5'-3')	Reverse primers (5'-3')	Melting tempearature (T˚C)	Expected product size (bp) in PCR	Expected product size (bp) in Q-PCR	Putative Functions
OsISM11510	LOC_Os02g03580	1460820	CGAGGCACTTCTTTCACACA	GCCATTGGTGTCGTTCTGTT	60.5	369	95	transcription factor putative expressed
OsISM11511	LOC_Os02g03610	1476550	CTCTTCGACGCCTTCTTCTC	CCCATGTGCAGCCTATACAA	59.4	719	96	ADP-ribosylation factor putative expressed
OsISM11512	LOC_Os02g03610	1477266	GGGAGAAGTTCTTTCAACGG	GCCAACATCCCACACAGTAA	59.1	1040	89	ADP-ribosylation factor putative expressed
OsISM11513	LOC_Os02g03610	1478298	ATGTTGGCGGACAAGAAAAG	CTGTCCAATGAATCAACAACG	59.6	562	89	ADP-ribosylation factor putative expressed
OsISM11514	LOC_Os02g03610	1478839	CGTTGTTGATTCATTGGACAG	CCATCAGTGGGTCCTTGATAA	59.4	186	78	ADP-ribosylation factor putative expressed
OsISM11515	LOC_Os02g03610	1479016	CACTGATGGCAAACAGCATAA	CTCACCTCATCTGGGCTCAT	60.0	584	77	ADP-ribosylation factor putative expressed
OsISM11516	LOC_Os02g03630	1500916	TTTGGGTTAGCTACAGATGGG	CATGCAAATTCTCCATGCAC	59.6	350	78	transposon protein putative CACTA En/Spm sub-class
OsISM11517	LOC_Os02g03630	1501425	CTATGGGCGTCTGGTATTGG	GGATGGAGCTCCCCTCTAAT	59.9	238	97	transposon protein putative CACTA En/Spm sub-class
OsISM11518	LOC_Os02g03630	1502086	TAAGATTGCTGGGCCAGTTT	TGGAGTTAGCCTTTTGCGTT	59.8	596	95	transposon protein putative CACTA En/Spm sub-class
OsISM11519	LOC_Os02g03630	1502709	CAATTCCCTGATTGGTTCAGA	CACGTGCCAGCCATCTAATA	59.8	165	92	transposon protein putative CACTA En/Spm sub-class
OsISM11520	LOC_Os02g03630	1502852	GATATTAGATGGCTGGCACG	TTGCAAATACAAAGGGCTCA	58.8	384	59	transposon protein putative CACTA En/Spm sub-class
OsISM11521	LOC_Os02g03640	1507531	TGTTCGAGGCAACTGCTAAG	TTCAGTTTTGTCTTGCCACG	59.6	157	77	conserved hypothetical protein
OsISM11522	LOC_Os02g03640	1507851	GGAAAGAGAGAGAAAAATACAAATGAA	CATCCGAGGAGCATAAAGGA	59.9	186	88	conserved hypothetical protein
OsISM11523	LOC_Os02g03640	1508165	TGACCAGTGGATTGCTCTTG	TGCCTTCTTCTTTTGGCAGT	59.9	203	92	conserved hypothetical protein
OsISM11524	LOC_Os02g03640	1508381	GGACAAAGAGTTTTGCTCGC	TGTGGGTACTTCTTTGCAGG	59.4	160	65	conserved hypothetical protein
OsISM11525	LOC_Os02g03640	1508568	CAAGCGCAAAAGTGACAAGA	CATCCGCTAAAGCTGGTTGT	60.2	166	81	conserved hypothetical protein
OsISM11526	LOC_Os02g03640	1509004	CCAAGTTGGAAAAGGCACAA	CCCTCCTTTTTGAATTAGGTACG	60.6	189	97	conserved hypothetical protein
OsISM11527	LOC_Os02g03640	1509121	AGGGAGGTTTAGATGGAGCA	CGAGGGGTTGTCACCATACA	60.3	187	93	conserved hypothetical protein

ISM IDs	Rice gene locus IDs	Physical positions (bp) on chromosomes	Forward primers (5'-3')	Reverse primers (5'-3')	Melting tempearature (T˚C)	Expected product size (bp) in PCR	Expected product size (bp) in Q-PCR	Putative Functions
OsISM11528	LOC_Os02g03640	1509342	TGGATCAGCAGGACAACAGT	GGTTGGTGGTTGCAAATTCT	59.5	158	69	conserved hypothetical protein
OsISM11529	LOC_Os02g03640	1509483	ATTTGCAACCACCAACCAAG	TTCCCTGGTTGCATAAGCTC	60.5	296	80	conserved hypothetical protein
OsISM11530	LOC_Os02g03640	1509757	ATGAGCTTATGCAACCAGGG	TTGTCCTGGTTGTCAGGTTT	59.1	203	68	conserved hypothetical protein
OsISM11531	LOC_Os02g03640	1509940	AAACCTGACAACCAGGACAA	TAACAAGCTTGCGGGTAAGG	59.1	188	89	conserved hypothetical protein
OsISM11532	LOC_Os02g03640	1510099	CAAAGAGAACCTTACCCGCA	AAAAGTCCATTGTGTTGCATTG	60.1	209	90	conserved hypothetical protein
OsISM11533	LOC_Os02g03640	1510285	GCAATGCAACACAATGGACT	AAGGGACCGTCACCATACAC	59.6	144	64	conserved hypothetical protein
OsISM11534	LOC_Os02g03640	1510408	AGTGTATGGTGACGGTCCCT	TCATCTTGAGCATGGCTTTG	59.6	172	85	conserved hypothetical protein
OsISM11535	LOC_Os02g03640	1510556	TTCACAAAGCCATGCTCAAG	TCATTTAGGCCAAAGGTTCG	60.0	252	88	conserved hypothetical protein
OsISM11536	LOC_Os02g03640	1510798	GCCTAAATGACCATGGGAAA	TCCATTTTGCTGCACTGTTG	60.3	410	65	conserved hypothetical protein
OsISM11537	LOC_Os02g03640	1511187	ACAACAGTGCAGCAAAATGG	GCCACGTTTGTATTCCGATT	59.8	181	97	conserved hypothetical protein
OsISM11538	LOC_Os02g03650	1518811	CGTTGTCGATGGTGATGAAG	ACAGATGCTTCACGCATTCA	60.3	4199	94	expressed protein
OsISM11540	LOC_Os02g03690	1540323	TCAGCTCTCTGAATTGTTTGG A	AAGACGACCTCAAGCTCCAC	59.7	619	53	beta-amylase putative expressed
OsISM11541	LOC_Os02g03690	1541143	TGTTTCTGCTGTTATTGCCG	TCTGGTGTAATCACCTGCCA	60.0	1073	71	beta-amylase putative expressed
OsISM11542	LOC_Os02g03690	1542197	GGCAGGTGATTACACCAGAAC	TGCCCTCTGGATCAATCAAT	59.9	1446	96	beta-amylase putative expressed
OsISM11543	LOC_Os02g03690	1543749	TGGTTACAGGGACCTTTTCG	AGATCCAGTCTCCCCAGACC	60.2	479	89	beta-amylase putative expressed
OsISM11544	LOC_Os02g03690	1544339	GACAAAGAGCGAGTCCTTCG	CCTCTTCGGTCAGGTTTCTG	60.0	414	95	beta-amylase putative expressed
OsISM11545	LOC_Os02g03690	1544826	GGGCTGGAGATATCCTGGTA	CAAAACAGATGTCCCCGTG	59.5	438	97	beta-amylase putative expressed
OsISM11546	LOC_Os02g03690	1545429	TGCATTTGATGGAGTCGAAA	CAGCATGGCTTGCAGTTCTA	60.2	673	72	beta-amylase putative expressed

ISM IDs	Rice gene locus IDs	Physical positions (bp) on chromosomes	Forward primers (5'-3')	Reverse primers (5'-3')	Melting tempearature (T˚C)	Expected product size (bp) in PCR	Expected product size (bp) in Q-PCR	Putative Functions
OsISM11547	LOC_Os02g03690	1546239	GCATTTGCTGATCCAGAAGG	TAATCCATGATCCCATGCTG	60.0	156	61	beta-amylase putative expressed
OsISM11548	LOC_Os02g03690	1546549	GCTTCTCAGAGCTTGGCAAC	TGATGAGCAATAATGGTGCG	60.5	1477	89	beta-amylase putative expressed
OsISM11550	LOC_Os02g03720	1561130	CTCGTCGACCCCAGCTACT	TCGCCTCAGATACAGCTTCA	60.1	189	92	expressed protein
OsISM11551	LOC_Os02g03720	1561299	TGAAGCTGTATCTGAGGCGA	CCAGCTGAATGCCCTATCAA	60.4	408	67	expressed protein
OsISM11552	LOC_Os02g03720	1561761	ACTGCTCACACTTGGCACTC	TTTGGTCAATTACCCCAGGA	59.6	350	63	expressed protein
OsISM11553	LOC_Os02g03720	1562141	ATTGTGCCCCAGCAGTTTAC	GTAGCGATGGTGTTCCCAGT	60.0	459	95	expressed protein
OsISM11554	LOC_Os02g03720	1562674	CTACTCAATCAGGCCCAACC	GTCTCCCCACACATCAGCAC	60.6	171	84	expressed protein
OsISM11555	LOC_Os02g03770	1589144	GGGAATCGGGAGGAAGTG	CCAATGGCCACTGTTTCTTC	60.5	777	68	conserved hypothetical protein
OsISM11556	LOC_Os02g03790	1595448	CATCGTCATTGTCATCTCCG	GATTAAGGGACCTACCCCGA	60.1	1222	98	expressed protein
OsISM11557	LOC_Os02g03790	1596606	CTGTCGATAATGCAGGTGGA	ATCCATAGATGGACAACGCC	59.7	213	75	expressed protein
OsISM11558	LOC_Os02g03790	1596939	TTGCTGGATTCTGGGGTTAC	TGGGAAGATGATGAACCAAA	59.4	1074	96	expressed protein
OsISM11559	LOC_Os02g03790	1597993	TTTGGTTCATCATCTTCCCA	AAAACGGCATTTACCGAGTG	59.4	178	86	expressed protein
OsISM11560	LOC_Os02g03790	1598151	CACTCGGTAAATGCCGTTTT	AGAAAGAAATATGCGACCCG	59.6	183	84	expressed protein
OsISM11561	LOC_Os02g03790	1598366	TTCTCCATGCATCAACTCCA	CATCGAGAAATGGGTATGGC	60.2	158	46	expressed protein
OsISM11562	LOC_Os02g03790	1598504	GCCATACCCATTTCTCGATG	GCATGGTAATTGCATGATCG	60.1	210	77	expressed protein
OsISM11563	LOC_Os02g03800	1600818	CTGCAGAATGATCTGCCTGA	GCAGCATCAGCATCGTTTC	60.3	440	76	THION22 - Plant thionin family protein precursor expressed
OsISM11565	LOC_Os02g03820	1604097	AGCATGCTCAGCAAGATTGA	ATTGTCAGCCACATCTTCCC	59.8	945	83	expressed protein
OsISM11566	LOC_Os02g03820	1605824	GTCAGACCTTCATGTTGGGG	TTCTGCAACTTCAAGCAACAA	60.0	247	89	expressed protein

ISM IDs	Rice gene locus IDs	Physical positions (bp) on chromosomes	Forward primers (5'-3')	Reverse primers (5'-3')	Melting tempearature (T˚C)	Expected product size (bp) in PCR	Expected product size (bp) in Q-PCR	Putative Functions
OsISM11567	LOC_Os02g03820	1606212	CATCTGCAGCCCTCTGTTAAG	TCAGCCCTATATTTGGATGACC	60.1	499	99	expressed protein
OsISM11568	LOC_Os02g03860	1637650	GAGATCGGAGCAGCGAAG	CAAGGCAGCTTGAGCAGAGT	60.3	1410	96	mitochondrial ATP synthase precursor putative expressed
OsISM11569	LOC_Os02g03860	1639105	GATCGTACTCCCGTCAATGG	CACCAAGGGCAGTCTCAAAT	60.2	445	80	mitochondrial ATP synthase precursor putative expressed
OsISM11570	LOC_Os02g03860	1639599	TTCTCGTTATGCCCAAGTCA	TGCCCTTTGTAAAGGTGTCG	60.2	670	99	mitochondrial ATP synthase precursor putative expressed
OsISM11571	LOC_Os02g03860	1640270	CCCTGATGCACGGACTTACT	CCATCATCATAGCCTCAGCA	60.0	250	90	mitochondrial ATP synthase precursor putative expressed
OsISM11572	LOC_Os02g03860	1640581	TATGGGTCTCCTCTTGGCAG	GAAGATCCTCCTTGCGGATT	60.4	542	66	mitochondrial ATP synthase precursor putative expressed
OsISM11573	LOC_Os02g03860	1641148	GGAAATTGCCAAAAGTGAGC	AGGCATCTCCTCGTCTTTGA	59.8	271	98	mitochondrial ATP synthase precursor putative expressed
OsISM11574	LOC_Os02g03910	1664317	GAGAGGAGGAGGAGCGAGTC	CTAATTGGGTTGCGGTCG	60.4	459	97	OsFBX35 - F-box domain containing protein
OsISM11575	LOC_Os02g03910	1664972	CGACGAATTCTTGGTCACCT	ATTGACGAGCTGCCGTGT	60.3	198	80	OsFBX35 - F-box domain containing protein
OsISM11576	LOC_Os02g03910	1665581	GTACCTGGACGTGTGGGC	GAGCAGCTGAGCATCCTCTT	59.9	213	88	OsFBX35 - F-box domain containing protein
OsISM11577	LOC_Os02g03950	1681709	TGCTAACGAAGAAACCCACC	TTGCAATGACAAGGTGCAAT	60.1	232	97	zinc finger C3HC4 type family protein expressed
OsISM11578	LOC_Os02g03950	1681957	GTGCATCGATCCTGTCTTGA	TCAGTGCAATGTGAAAAGGC	59.8	189	66	zinc finger C3HC4 type family protein expressed
OsISM11579	LOC_Os02g03950	1682218	TCTGGTTGTTAGAGATCACAC GTT	CTCGTAGTTCATCTCCATAAAAT CTG	59.9	506	99	zinc finger C3HC4 type family protein expressed
OsISM11580	LOC_Os02g03950	1682729	TTTGGTTATGAAGAGCATCCA	TCCATACAGCAAACCAACCA	59.1	796	76	zinc finger C3HC4 type family protein expressed
OsISM11581	LOC_Os02g03950	1683551	GAGGATCACAGAACGGCATT	CAAGGTCAGGAACTCCATCAG	59.9	757	99	zinc finger C3HC4 type family protein expressed
OsISM11582	LOC_Os02g04020	1743626	AGGAGGAGGCTGTGCTTGT	TTGTCATCATTACTTGAAGACCC	59.1	1344	98	expressed protein
OsISM11583	LOC_Os02g04020	1745186	TTTGGATGAGTTGTTGCCTG	TCCACATTTGCTTTGCTTTC	59.3	182	99	expressed protein
OsISM11584	LOC_Os02g04020	1745357	GCAAATGTGGACTGACATGC	TGCAGAACAGCGTCTAACTCA	60.0	178	94	expressed protein

ISM IDs	Rice gene locus IDs	Physical positions (bp) on chromosomes	Forward primers (5'-3')	Reverse primers (5'-3')	Melting tempearature (T˚C)	Expected product size (bp) in PCR	Expected product size (bp) in Q-PCR	Putative Functions
OsISM11585	LOC_Os02g04020	1745720	AAAGGCACATTGATTCGAGC	ATTCACGTGCAGTTTTGCTG	60.1	173	79	expressed protein
OsISM11586	LOC_Os02g04020	1745892	TTAATTGTCCGATTGCAAAAA	CGGACCAGCATTGACAATAA	58.9	167	79	expressed protein
OsISM11587	LOC_Os02g04020	1746100	CCCAAAAACCACTTCCAAGA	TTGCTTTGATATTTGTTCCCG	59.9	131	49	expressed protein
OsISM11588	LOC_Os02g04020	1746210	CGGGAACAAATATCAAAGCA A	CCCTCCATATTCAGGACAGG	59.7	189	95	expressed protein
OsISM11589	LOC_Os02g04020	1746408	GCTGAAAAAGGACCATGGAA	ACACTGAGCCTCGCCACTAA	60.5	169	64	expressed protein
OsISM11590	LOC_Os02g04020	1746622	TCTTATGCCAAGTCCAAGCA	TCCAGACTCAGCAGTGGATG	59.7	156	61	expressed protein
OsISM11591	LOC_Os02g04020	1746827	CCCATCCTAAGTTGACCCCT	GCGAGTAGAAAAGCTCGTGG	60.2	396	66	expressed protein
OsISM11592	LOC_Os02g04020	1747255	GGCTGTAGATGCAACATGGA	AGCCATTGGATGAACTTTCTG	59.4	180	96	expressed protein
OsISM11593	LOC_Os02g04020	1747761	GCGCTGGAAGCTGAACTTAT	CCTCTTGCCTGATAAGAGCC	59.3	170	62	expressed protein
OsISM11594	LOC_Os02g04070	1769341	CGCCTCTACGCACATGAC	CTGCTCAACATCCGAGTGG	59.7	1384	83	retrotransposon protein putative unclassified
OsISM11595	LOC_Os02g04100	1784982	ATCAACCACGCCTTCGTCTA	TCTTCCATGATCGCTGTGTG	60.7	2438	98	peptidase T1 family putative expressed
OsISM11596	LOC_Os02g04110	1789326	TCTTCCACACGTTCAGCAAC	AATGCATCCCCGTATCCTCT	60.3	1007	99	expressed protein
OsISM11597	LOC_Os02g04110	1790321	CGGGGATGCATTGTAGATTC	TTTCTTCAACATGGCAGCAG	60.1	313	85	expressed protein
OsISM11598	LOC_Os02g04110	1790769	TTCGAGATTGTCCTTCATCTAC C	AGATAAAACTGGGGGCAGGT	59.7	1218	96	expressed protein
OsISM11599	LOC_Os02g04120	1793918	ACCAGGGACACCACCTCTTC	ATCACGATCCTCTCCCTCG	60.8	350	59	cytidylyltransferase domain containing protein expressed
OsISM11600	LOC_Os02g04120	1794257	AGGATCGTGATCGGCATCT	CGCTTTTCGATTGGCTGTAT	60.4	1553	78	cytidylyltransferase domain containing protein expressed
OsISM11601	LOC_Os02g04120	1795799	ATCGAAAAGCGAATGGAAAA	ACGATTGGCTCGACATGAAC	60.4	569	78	cytidylyltransferase domain containing protein expressed
OsISM11602	LOC_Os02g04120	1796344	TGAAGTTCATGTCGAGCCAA	GCCCTCCTGGCAAAGTCT	60.4	295	99	cytidylyltransferase domain containing protein expressed

ISM IDs	Rice gene locus IDs	Physical positions (bp) on chromosomes	Forward primers (5'-3')	Reverse primers (5'-3')	Melting tempearature (T˚C)	Expected product size (bp) in PCR	Expected product size (bp) in Q-PCR	Putative Functions
OsISM11603	LOC_Os02g04120	1796663	AGAGAGGCCTCGCACAACT	CCTGTAGACTTCTCCGGCAC	60.0	152	59	cytidylyltransferase domain containing protein expressed
OsISM11604	LOC_Os02g04140	1804947	CCCTCTAAGGTGGTCGTTCA	GATCTTCGGCCTCGGATT	60.1	195	93	retrotransposon protein putative unclassified
OsISM11605	LOC_Os02g04170	1822460	TTCTTCTCTCCCGTCAACAGA	CTCATTCGAAGTTGGGAAGC	59.9	507	96	L-aspartate oxidase 1 putative expressed
OsISM11606	LOC_Os02g04170	1823195	AGAGAGCCACATGCAAGACA	CCGGTCCTTCTGTGCATACT	59.9	220	84	L-aspartate oxidase 1 putative expressed
OsISM11607	LOC_Os02g04170	1823592	CTTTGTTTGGCCACCACTTT	TGAGTTTCAGCGTCCAATGA	60.2	673	95	L-aspartate oxidase 1 putative expressed
OsISM11608	LOC_Os02g04170	1824231	GCTATGGTGTGGATTCATTGG	AGATGAAACGAATTGCCTGG	60.1	192	52	L-aspartate oxidase 1 putative expressed
OsISM11609	LOC_Os02g04170	1824433	ATTGCTTGCATCTGGAGGAG	AGACATTGCAATTCCATCCC	60.1	322	83	L-aspartate oxidase 1 putative expressed
OsISM11610	LOC_Os02g04170	1824743	ATTGCAATGTCTCATCGTGC	TGATAGTGCAGTTGGGTGGA	59.9	278	73	L-aspartate oxidase 1 putative expressed
OsISM11611	LOC_Os02g04200	1843409	GTCCATCTGGACAGGCAAGT	GTGGTTCCTGTCAATCCCAC	60.2	452	75	transposon protein putative unclassified
OsISM11613	LOC_Os02g04200	1844372	CTAGCAAGGCTTGGTTGCTC	AACCACAACAGGTACGCCTC	60.1	184	98	transposon protein putative unclassified
OsISM11614	LOC_Os02g04200	1844866	ACGATGGGGACTTACTGGTG	GGAGACGGTCAAACTCGAAC	59.8	190	95	transposon protein putative unclassified
OsISM11615	LOC_Os02g04200	1845228	CGGACTTAGGCAGGTGTTTC	CTAGCTGTCCCCGTCGAGT	60.1	159	81	transposon protein putative unclassified
OsISM11616	LOC_Os02g04200	1845595	GCACCGTGACCGAGACTACT	GCAAACCTCTGCTCAACCTC	60.2	187	93	transposon protein putative unclassified
OsISM11617	LOC_Os02g04200	1845975	GCGACCTACAGCACCAGTTT	ACGCGCTCGAAGGCATAC	61.4	140	48	transposon protein putative unclassified
OsISM11618	LOC_Os02g04200	1846183	AGCTCTTCCTCTCACGGAGC	AGACGAAGAGGCCCCAGTA	60.5	144	82	transposon protein putative unclassified
OsISM11619	LOC_Os02g04200	1846513	TACTCCCAGGGGCACATTAG	GCCGAAAGTGTGTTGTTGTG	60.1	235	98	transposon protein putative unclassified
OsISM11620	LOC_Os02g04250	1872971	TCAAGGCCAGATACTGCTCC	TGATGCTGGAGTTCATCTGC	60.2	174	73	glycosyltransferase putative expressed
OsISM11621	LOC_Os02g04250	1873125	GCAGATGAACTCCAGCATCA	CTCCTCCTCGTCCTCCTCTT	59.9	156	80	glycosyltransferase putative expressed

ISM IDs	Rice gene locus IDs	Physical positions (bp) on chromosomes	Forward primers (5'-3')	Reverse primers (5'-3')	Melting tempearature (T˚C)	Expected product size (bp) in PCR	Expected product size (bp) in Q-PCR	Putative Functions
OsISM11622	LOC_Os02g04270	1883383	GCTCATTCGCGGTATCTCTT	CACAACTGCAAGACCCAATG	59.8	175	68	DNA polymerase V putative expressed
OsISM11623	LOC_Os02g04270	1883581	CGTCGAAGCAATCATGAAGT	TCGTCCCAAAAGATTGTCCT	59.2	179	89	DNA polymerase V putative expressed
OsISM11624	LOC_Os02g04270	1883886	TTAACTGAGCCCGCTGTTG	ATCTTGCACACTGGGAGCTT	59.9	610	97	DNA polymerase V putative expressed
OsISM11625	LOC_Os02g04270	1884596	TGCCATATCCATTCAGTCCA	TGAATGCGTGGAAGACAAAA	60.1	277	98	DNA polymerase V putative expressed
OsISM11626	LOC_Os02g04270	1885933	AACGACGACAATGCATTCAA	TTAGTCGAATCCAAGCCAGG	60.2	582	90	DNA polymerase V putative expressed
OsISM11627	LOC_Os02g04270	1886825	CATGTGTCTGGCCCTGTATG	CAACATGTCAAGAAGCCCTG	59.6	440	88	DNA polymerase V putative expressed
OsISM11628	LOC_Os02g04270	1887659	TCAAGCTCCGTGTCCTTACA	GAATAGACCTCCAGCACCAGA	59.4	318	77	DNA polymerase V putative expressed
OsISM11629	LOC_Os02g04325	1907611	ACTGAAGGCCCATGAATTTG	GGATGCCCATAATCATCACC	60.0	231	79	expressed protein
OsISM11630	LOC_Os02g04330	1909500	TTGTTCAGGGAAATAGCTATGGA	GCTTCGCTCGGACATATGAG	60.4	333	87	5-AMP-activated protein kinase beta-1 subunit-related putative expressed
OsISM11631	LOC_Os02g04330	1910052	TGGTGAAGTTTATGGTGACCC	AGATCGTTCTGAATCAGCCAA	59.8	784	92	5-AMP-activated protein kinase beta-1 subunit-related putative expressed
OsISM11632	LOC_Os02g04330	1910857	GAAGCAAACCAATACAACCG	TTCAAGTTCCATAACGGCAA	58.7	747	97	5-AMP-activated protein kinase beta-1 subunit-related putative expressed
OsISM11633	LOC_Os02g04330	1911535	TTGCAGTTGCAAATGAAGGA	TCTCTTCAGCTAGGGCAACC	60.0	459	98	5-AMP-activated protein kinase beta-1 subunit-related putative expressed
OsISM11634	LOC_Os02g04330	1912007	TGTTCCATCTGGGTCTAGGAA	CCTTAATTTGTTCCATAACCTTATCA	59.2	172	99	5-AMP-activated protein kinase beta-1 subunit-related putative expressed
OsISM11635	LOC_Os02g04330	1912177	GGATGTAGACTCTTTAAAGCACCA	CGAAGGAGCCCATAATTTGT	59.0	197	81	5-AMP-activated protein kinase beta-1 subunit-related putative expressed
OsISM11636	LOC_Os02g04330	1912420	CCAAGCAAGATTCTCTGCAA	GACAGCCTCCACTCTCCATC	59.5	177	82	5-AMP-activated protein kinase beta-1 subunit-related putative expressed
OsISM11637	LOC_Os02g04430	1949994	ATTCCCCATCCCGTTCTTT	CGTACAGCTCGTTTATCGCA	60.3	151	66	STRUBBELIG-RECEPTOR FAMILY 1 precursor putative expressed
OsISM11638	LOC_Os02g04430	1950223	AATGCCTTGGACCCAACAT	GCGTCTCACTCAGCTTTCCT	60.0	214	70	STRUBBELIG-RECEPTOR FAMILY 1 precursor putative expressed
OsISM11639	LOC_Os02g04430	1950420	AAAGCTGAGTGAGACGCTGG	GCAGGTGGTAAGCTCTCAGG	60.4	185	97	STRUBBELIG-RECEPTOR FAMILY 1 precursor putative expressed

ISM IDs	Rice gene locus IDs	Physical positions (bp) on chromosomes	Forward primers (5'-3')	Reverse primers (5'-3')	Melting tempearature (T˚C)	Expected product size (bp) in PCR	Expected product size (bp) in Q-PCR	Putative Functions
OsISM11640	LOC_Os02g04430	1950585	CCTGAGAGCTTACCACCTGC	AATCTGGGAGTTTGCCACTG	60.1	174	71	STRUBBELIG-RECEPTOR FAMILY 1 precursor putative expressed
OsISM11641	LOC_Os02g04430	1950739	CAGTGGCAAACTCCCAGATT	TGCCAGTGAGCTGATTGTTC	60.1	159	78	STRUBBELIG-RECEPTOR FAMILY 1 precursor putative expressed
OsISM11642	LOC_Os02g04430	1950878	GAACAATCAGCTCACTGGCA	CTGGGATTGGTCCTGAGAAA	60.0	203	93	STRUBBELIG-RECEPTOR FAMILY 1 precursor putative expressed
OsISM11643	LOC_Os02g04430	1951066	CAGGACCAATCCCAGAGAAA	GATGGTAAGATGGTTGCCGT	59.9	157	66	STRUBBELIG-RECEPTOR FAMILY 1 precursor putative expressed
OsISM11644	LOC_Os02g04430	1951556	AAGCCTTCAGGACAAAGTGC	TGAAGAAATGCTACCCTTGG	58.7	271	88	STRUBBELIG-RECEPTOR FAMILY 1 precursor putative expressed
OsISM11645	LOC_Os02g04430	1951970	CCTAGCAGAGCAGCAAGACA	TCCTCAAGAACTCGTCCACC	59.9	212	84	STRUBBELIG-RECEPTOR FAMILY 1 precursor putative expressed
OsISM11646	LOC_Os02g04430	1952340	GCTGCCAAGATCACTCTTCC	TGTCTCTGACCACCTTCGTG	59.9	214	93	STRUBBELIG-RECEPTOR FAMILY 1 precursor putative expressed
OsISM11647	LOC_Os02g04430	1952640	CTGTGAAATCCGGCTTGG	CGGTGGCTCGTAGCTCAG	60.5	145	63	STRUBBELIG-RECEPTOR FAMILY 1 precursor putative expressed
OsISM11648	LOC_Os02g04450	1968187	AGGTTTCTCGCCAGGACATA	GGTCTCAACCACTTCTTTCTGG	59.9	1255	87	plant-specific domain TIGR01589 family protein putative expressed
OsISM11649	LOC_Os02g04450	1969441	CCTATCATTCCAGGCGAAAA	GAACTCACGATTCTCTTCCTCAA	60.0	379	77	plant-specific domain TIGR01589 family protein putative expressed
OsISM11650	LOC_Os02g04450	1969946	ACTAATGGCTCCAACATGGG	TTGCAGTGCTGGGAGTAGTG	59.9	637	71	plant-specific domain TIGR01589 family protein putative expressed
OsISM11651	LOC_Os02g04450	1970945	TTCTTGAGCCAAATTCCTCG	GGGATCTGCCATAATTCTCC	59.4	203	92	plant-specific domain TIGR01589 family protein putative expressed
OsISM11652	LOC_Os02g04450	1971139	GCAGATCCCCTTTTATCCCT	TCGGATATGGTATCTAGCCGTC	59.8	273	98	plant-specific domain TIGR01589 family protein putative expressed
OsISM11653	LOC_Os02g04480	1981574	TTCCCAAGGACGATAGTGTCT	TTTTCATCCCTTCTGTACTTGC	58.5	741	99	cleavage and polyadenylation specificity factor putative expressed
OsISM11654	LOC_Os02g04480	1982705	CCAGGAGCATCCACCATTAT	GCTCACAGAGGTCACCATCA	59.8	273	93	cleavage and polyadenylation specificity factor putative expressed
OsISM11655	LOC_Os02g04490	1986520	GGGCCTTGATACCCAGTTTT	TCGATGATTGCTTCCTTCCT	60.0	472	81	histone acetyltransferase HAC1 putative expressed
OsISM11656	LOC_Os02g04490	1987014	AGAACTTGCTAAACGGCTCG	AATTGTGGCTCAACTGGTCC	59.8	218	94	histone acetyltransferase HAC1 putative expressed
OsISM11657	LOC_Os02g04490	1987439	TGCCAACACTGGTACCTCAC	TAGATGGGATGCTGCTTGTG	59.7	441	99	histone acetyltransferase HAC1 putative expressed

ISM IDs	Rice gene locus IDs	Physical positions (bp) on chromosomes	Forward primers (5'-3')	Reverse primers (5'-3')	Melting tempearature (T°C)	Expected product size (bp) in PCR	Expected product size (bp) in Q-PCR	Putative Functions
OsISM11658	LOC_Os02g04490	1988271	ACAATTCAACCAGCACCCTC	TGCTGTTAAAGCAGAAGAACCA	60.0	801	95	histone acetyltransferase HAC1 putative expressed
OsISM11659	LOC_Os02g04490	1989986	CGGAAGTTGTAAATTCCATCG	TGCTTGCTTGCTAGTTCAGC	59.5	207	91	histone acetyltransferase HAC1 putative expressed
OsISM11660	LOC_Os02g04490	1990728	ACCCCAGAACAAATTCAGGA	TGCCCCATCAACTGATTCTT	59.9	647	90	histone acetyltransferase HAC1 putative expressed
OsISM11661	LOC_Os02g04490	1991579	CTTTTTGAAGGCCAGATTCG	TGCCAGCATTCACACTTGTC	60.4	188	85	histone acetyltransferase HAC1 putative expressed
OsISM11662	LOC_Os02g04490	1991955	AACGGTTGAAGCAGGAGAGA	CTAACCACAAGACCTTCGGC	59.9	1144	86	histone acetyltransferase HAC1 putative expressed
OsISM11663	LOC_Os02g04490	1993167	ACTACCCGACAGAGTTCCCA	CCAAATAGGCACACTTCTACACC	59.8	174	80	histone acetyltransferase HAC1 putative expressed
OsISM11664	LOC_Os02g04490	1993446	GGTGTCTGGAGAGGCGTTAC	CTTGTGAATCCACGCTGCT	59.9	246	85	histone acetyltransferase HAC1 putative expressed
OsISM11665	LOC_Os02g04490	1993767	TCCAAAATCCGACAAATTGA	TCCTCCTTGGTAGCTTTTCG	59.2	147	61	histone acetyltransferase HAC1 putative expressed
OsISM11666	LOC_Os02g04490	1994145	CGATCTCAGTGGGAATGCTT	CATTGGATAAATGGTTTCCCC	60.2	228	68	histone acetyltransferase HAC1 putative expressed
OsISM11667	LOC_Os02g04490	1994433	GTGTCTGGAAAACGTTGGGT	GGGTGCCTCTCCCTATCTTC	60.0	932	99	histone acetyltransferase HAC1 putative expressed
OsISM11668	LOC_Os02g04490	1995345	GAAGATAGGGAGAGGCACCC	CTTGGTATCTTTGGGCAACC	59.7	172	85	histone acetyltransferase HAC1 putative expressed
OsISM11669	LOC_Os02g04490	1995825	TGATCGTGATGCCCAAAATA	CATGTCGAGGCATGTACCAG	60.0	424	88	histone acetyltransferase HAC1 putative expressed
OsISM11670	LOC_Os02g04490	1996362	GGTACATGCTCCAGCTCCAT	ACCTTCGAAGATGCTCCTTG	59.8	185	88	histone acetyltransferase HAC1 putative expressed
OsISM11671	LOC_Os02g04500	1998613	CTTCGTCGACTGCACCCT	ATCGACATCCATCCCAATGT	60.0	274	92	S-adenosyl-L-methionine-dependent methyltransferase mraW putative expressed
OsISM11672	LOC_Os02g04500	1999054	TGTTGGAACGTCTGGAGTTG	CCCATCCTGAAGAACGCTAA	60.0	191	98	S-adenosyl-L-methionine-dependent methyltransferase mraW putative expressed
OsISM11673	LOC_Os02g04500	1999225	TTAGCGTTCTTCAGGATGGG	CAACTTCAAGATCAGGCCAAG	60.0	708	97	S-adenosyl-L-methionine-dependent methyltransferase mraW putative expressed
OsISM11674	LOC_Os02g04500	2000026	CAGGGGAGCTTGTCAAACTTA	CTTAGGGCCTGAAACACCCT	59.9	184	95	S-adenosyl-L-methionine-dependent methyltransferase mraW putative expressed
OsISM11675	LOC_Os02g04510	2002486	CATGCTCCGTGGCTACAAC	TCACCAACCACAATGTCCAC	60.3	406	94	UOS1 putative expressed

ISM IDs	Rice gene locus IDs	Physical positions (bp) on chromosomes	Forward primers (5'-3')	Reverse primers (5'-3')	Melting tempearature (T˚C)	Expected product size (bp) in PCR	Expected product size (bp) in Q-PCR	Putative Functions
OsISM11676	LOC_Os02g04510	2002995	GATAACCAAGGCGTGAGGAA	TGCTCTTGCTGCTTTTACCA	59.9	186	89	UOS1 putative expressed
OsISM11677	LOC_Os02g04510	2003307	CTGAAAATGGACAGGCTGTTT	AGCCGTTTCGACACTTCAAC	59.8	224	71	UOS1 putative expressed
OsISM11678	LOC_Os02g04510	2003511	GTTGAAGTGTCGAAACGGCT	TTCCACCCACGGATAGAAGT	59.9	186	80	UOS1 putative expressed
OsISM11679	LOC_Os02g04510	2003738	GCTGACACCTCACAGAGCAA	TTCACTGGCCTGAAATCTGA	59.8	202	96	UOS1 putative expressed
OsISM11680	LOC_Os02g04510	2003949	CCCTCCTCTTGACCCTTTTC	GCTCCTTGAGACGAGTCACC	60.0	172	85	UOS1 putative expressed
OsISM11681	LOC_Os02g04510	2004113	CAAGGAGCTAGTGACCCAAGA	CCTGTGCATGAAACCAAAAC	59.3	192	99	UOS1 putative expressed
OsISM11682	LOC_Os02g04510	2004303	GGCTCTGGAATCGAACCTAA	CTCCTCAGTGCATCCTCTCC	59.6	161	72	UOS1 putative expressed
OsISM11683	LOC_Os02g04510	2004444	GGAGAGGATGCACTGAGGAG	GATCAAAGATCAGAGCACGC	59.2	429	98	UOS1 putative expressed
OsISM11684	LOC_Os02g04510	2004851	CAGCGTGCTCTGATCTTTGA	TATCTGCTACGTCGGCACAG	60.2	173	68	UOS1 putative expressed
OsISM11685	LOC_Os02g04510	2005040	GCACGATTCAACAGCAAGAA	TCATACAACTCATCCCCTTGC	60.0	169	76	UOS1 putative expressed
OsISM11686	LOC_Os02g04510	2005176	ACATGTCTCAAAGCAAGGGG	GCTGGTGCCAGGTAATTGTT	60.1	198	79	UOS1 putative expressed
OsISM11687	LOC_Os02g04520	2007594	GTGGGGTACGTCGGGAAG	CATGGTTTCAAGCTCGTTCA	60.6	1701	97	AGG2 putative expressed
OsISM11688	LOC_Os02g04520	2009280	GAGCTTGAAACCATGGAACC	CTTACACAGGAAGAAGCGGG	59.7	179	89	AGG2 putative expressed
OsISM11689	LOC_Os02g04520	2009388	TTGTCCTCTTGATCATGCCA	CAAGAAGCGTTCTCTGGACC	60.1	198	91	AGG2 putative expressed
OsISM11690	LOC_Os02g04530	2015394	CGGCGAAAGATTTCTCAGAT	CGTTGTAACCTCACCCCAGA	60.2	1033	94	rust resistance-like protein RP1 putative
OsISM11691	LOC_Os02g04530	2016407	TCTGGGGTGAGGTTACAACG	CCGGCTTTGATCATCGAGTA	61.0	607	71	rust resistance-like protein RP1 putative
OsISM11692	LOC_Os02g04530	2017158	CATCCAACTGATGCCCAAC	CCACTGGGAATAGCTTGGTC	59.7	553	64	rust resistance-like protein RP1 putative
OsISM11693	LOC_Os02g04530	2017745	ACTGTCCTCCAATCAAACCG	TGATTGCACATCGAACAACA	59.8	325	80	rust resistance-like protein RP1 putative

ISM IDs	Rice gene locus IDs	Physical positions (bp) on chromosomes	Forward primers (5'-3')	Reverse primers (5'-3')	Melting tempearature (T˚C)	Expected product size (bp) in PCR	Expected product size (bp) in Q-PCR	Putative Functions
OsISM11694	LOC_Os02g04560	2037885	CTACGTCGACTTCCACTGCC	CTCCAGCCTAAGTTTTCCCC	60.5	326	95	marker Xucw84 putative expressed
OsISM11695	LOC_Os02g04560	2038324	GCTGAATAAGGCTGTTTTGGA	ATTTGTGCTTTCCAGTGCCT	59.5	578	99	marker Xucw84 putative expressed
OsISM11696	LOC_Os02g04560	2039303	AATAGCGAAAAGCAAGCAGC	TGTTGACGTTTTCTGAAGCG	59.9	142	51	marker Xucw84 putative expressed
OsISM11697	LOC_Os02g04570	2043083	AACCATGCTTCGTTATTGCC	CTTTTTAGGATCTTTGCGGC	59.5	748	97	conserved hypothetical protein
OsISM11700	LOC_Os02g04630	2072794	CAAGATCAATGGGACGAACC	TCGAACTCGTCGGGTCTACT	60.1	219	70	sodium/calcium exchanger protein putative expressed
OsISM11701	LOC_Os02g04630	2073132	CCAAGCTCAATGTGTTGATACC	ACCCAGCATGCTTAGAGGAA	59.7	263	96	sodium/calcium exchanger protein putative expressed
OsISM11702	LOC_Os02g04630	2073397	TATTCCTTTGGCTGAGCGAT	CTAAACCTTTTGGCCCAGTG	59.7	151	69	sodium/calcium exchanger protein putative expressed
OsISM11703	LOC_Os02g04630	2073511	AGGCAACTGGCACTTTTCAC	CAAGCAAGTCCACCACTGAA	60.1	303	93	sodium/calcium exchanger protein putative expressed
OsISM11704	LOC_Os02g04630	2073816	GCAAAACCATGCAGACCTTT	GAATGAGCAACCCCATCACT	60.0	157	82	sodium/calcium exchanger protein putative expressed
OsISM11705	LOC_Os02g04630	2074087	AGTTGTCAAACAATCGGCGT	ACCACTGGGAGCAAAACAAC	60.3	534	98	sodium/calcium exchanger protein putative expressed
OsISM11706	LOC_Os02g04630	2074645	TGCTGTCATGTTTGCAGTGA	TCGATGATCCTATTGCCACTC	60.0	162	63	sodium/calcium exchanger protein putative expressed
OsISM11707	LOC_Os02g04630	2074783	TTGGAGTGGCAATAGGATCA	GTTTAGGTCCATTGGCTTGC	59.3	191	96	sodium/calcium exchanger protein putative expressed
OsISM11708	LOC_Os02g04630	2074988	GACGGCAAGCCTCTTAACAA	AGCATCAATCCTTTCACACAA	59.3	181	82	sodium/calcium exchanger protein putative expressed
OsISM11710	LOC_Os02g04670	2097603	ACAAGTGGGTCGACCAGAAC	TCTCCAGGAAAGGCTCGTTA	60.0	1821	88	glucan endo-1 3-beta-glucosidase precursor putative expressed
OsISM11711	LOC_Os02g04680	2107607	TTAAAGTCATCGTTGCGGGT	GGTCGAACTCAGCTAAACCG	60.2	211	76	OsSPL3 - SBP-box gene family member expressed
OsISM11712	LOC_Os02g04680	2107871	AAGCCACAACCAGAAGCAAT	AAAACTTGCCTGTTGCCTTG	60.0	519	82	OsSPL3 - SBP-box gene family member expressed
OsISM11713	LOC_Os02g04680	2108597	ATGGCGTTCAAAGAAACCAA	TCAAAGTCTGGGCCTCTAGC	60.0	1393	90	OsSPL3 - SBP-box gene family member expressed
OsISM11714	LOC_Os02g04700	2125257	CTAGTTAATTCGCCTCGGGA	CTCGTAACCGGGGACACTAA	59.7	149	67	tRNA synthetases class II domain containing protein expressed

ISM IDs	Rice gene locus IDs	Physical positions (bp) on chromosomes	Forward primers (5'-3')	Reverse primers (5'-3')	Melting tempearature (T˚C)	Expected product size (bp) in PCR	Expected product size (bp) in Q-PCR	Putative Functions
OsISM11716	LOC_Os02g04700	2125682	CAGCAGGTGTTCGACGTTT	TTGCTGTGATCAATGGCAGT	60.1	246	73	tRNA synthetases class II domain containing protein expressed
OsISM11717	LOC_Os02g04700	2126104	GATTTGCACATTGAACACGG	CCTTCTGACAGCAGCACTTG	59.9	196	98	tRNA synthetases class II domain containing protein expressed
OsISM11719	LOC_Os02g04700	2126914	CATTTTTAACCCAAAACAAATTTAC	CTTTGACGCTGAAACCATCC	59.0	283	97	tRNA synthetases class II domain containing protein expressed
OsISM11720	LOC_Os02g04700	2127185	TCAGCGTCAAAGTTGCTCAG	GCTTCCTGTCAGAGAGGTCAA	60.0	375	96	tRNA synthetases class II domain containing protein expressed
OsISM11721	LOC_Os02g04700	2127616	GCGAGCTGGTTCATACCAAG	GATGGAAAAGTTGGAAGTTTGTG	60.3	212	97	tRNA synthetases class II domain containing protein expressed
OsISM11722	LOC_Os02g04720	2140788	AAGAAATCGTCAGCGTGGAG	TCTCCTTCTCGGCCTTCATA	60.2	389	97	hypothetical protein
OsISM11723	LOC_Os02g04770	2180622	GATGACCTCCTCCGCCTC	GAGGATGTCAGAGTTGGAGAAAA	60.2	1991	85	hypothetical protein
OsISM11724	LOC_Os02g04780	2192264	ACGGCAAGGAGGTGAAGAC	CGACCTTGGGACGATAGAAG	60.0	228	85	expressed protein
OsISM11725	LOC_Os02g04780	2192693	CGGTGACAGAGAGTTGCAGA	AGGCTCTTCTTCCCCATCAC	60.4	134	95	expressed protein
OsISM11727	LOC_Os02g04800	2205860	ACGCTAATGGCGGAGGAG	CCCCTTGATGCTGAACCTC	61.0	193	97	AIG2-like family domain containing protein expressed
OsISM11728	LOC_Os02g04800	2206029	AGCCAGAGGTTCAGCATCAA	CTGTCAGTGATGCCCTTGAA	60.4	258	96	AIG2-like family domain containing protein expressed
OsISM11729	LOC_Os02g04800	2206287	GGAGCTCAATGTGCTGGATA	GCAAGCAATTTCTCCGATGT	59.5	294	91	AIG2-like family domain containing protein expressed
OsISM11730	LOC_Os02g04800	2206583	ATGCATACATTTGGGGCAAT	TAATCCTCCAAGTGCACCCT	59.8	652	86	AIG2-like family domain containing protein expressed
OsISM11731	LOC_Os02g04870	2248222	AGACTCCCGTCAACACCAAC	CCTTGCTCTTTGCAGCAACT	60.4	560	97	retrotransposon protein putative Ty1-copia subclass
OsISM11732	LOC_Os02g04870	2248718	CTACCCCGGTGCATTTAACA	CTTTCAAACTCCTCGCCCTA	60.1	456	86	retrotransposon protein putative Ty1-copia subclass
OsISM11733	LOC_Os02g04880	2253539	TCCTCTTTCAAATTGCCCTC	GCACATACTGCGCACTCAAA	60.1	951	83	hypothetical protein
OsISM11735	LOC_Os02g04880	2255256	TTTTGCCTACATTTATTTTCTTGA	GCATCAATAACCCCTCCAAA	58.5	166	98	hypothetical protein
OsISM11736	LOC_Os02g04880	2255421	CAGAAATGCAGGATGTGTGG	CGTATGTTTTCAGGCACAATG	59.6	874	99	hypothetical protein

ISM IDs	Rice gene locus IDs	Physical positions (bp) on chromosomes	Forward primers (5'-3')	Reverse primers (5'-3')	Melting tempearature (T°C)	Expected product size (bp) in PCR	Expected product size (bp) in Q-PCR	Putative Functions
OsISM11737	LOC_Os02g04880	2256198	TCCTCTTCTGTTCGTTGCAG	ATCGCCCTCCTTTAGCAACT	59.7	666	83	hypothetical protein
OsISM11738	LOC_Os02g04890	2259459	ATCAATGCCAGTGCTCGTTT	TGGCAGACAGTAGCAACAGC	60.4	428	83	hypothetical protein
OsISM11740	LOC_Os02g04940	2296315	GGTTGTTCAGAATGGTTGGG	CTCCTGGACAAAACCCTCAA	60.1	1065	85	retrotransposon protein putative Ty3-gypsy subclass
OsISM11741	LOC_Os02g04940	2297885	GTCTCACCGCAAGGAAGAGA	GGACAGGGCACAAGAAGAGA	60.5	498	86	retrotransposon protein putative Ty3-gypsy subclass
OsISM11742	LOC_Os02g04960	2326008	TGTCACGCAACTACTCGACG	TCCAATGATCTGTCGTCTCTG	59.9	765	83	expressed protein
OsISM11743	LOC_Os02g04970	2331961	GCGACTCGCAGTGCTATCT	GCTTGGAAACTCCTTGAAAGC	59.8	934	93	expressed protein
OsISM11744	LOC_Os02g04970	2332928	TGCCCGTTTTCCTTCTTATG	CAGATATACAGGTGTGCCACG	59.6	145	58	expressed protein
OsISM11745	LOC_Os02g04970	2333267	CTGCTGTTACAGTCTGGGCA	TCATCTCGGAGCTCATTCAA	59.8	150	64	expressed protein
OsISM11746	LOC_Os02g04970	2333401	ATGAGCTCCGAGATGATGCT	ATCTTGTACAGGGCCCACAG	60.0	505	78	expressed protein
OsISM11747	LOC_Os02g04970	2333966	TTGCGCATATGGGATATTTT	GCATCACCAACCCATATCCT	58.8	1066	99	expressed protein
OsISM11748	LOC_Os02g04970	2335020	GGTTGGTGATGCTGATGATG	GAAACGCAATCACAAGCTCC	60.4	1367	85	expressed protein
OsISM11749	LOC_Os02g04970	2336405	TGGTGCTGGTGAAAGGTTCT	GCTTGTCCATGCTTATTTTCAA	60.0	291	99	expressed protein
OsISM11750	LOC_Os02g04970	2336704	CTTAAGGAAGTTGGATCAATGC	CACTGACAGAAACTTCTTGTAGCA	58.3	476	95	expressed protein
OsISM11751	LOC_Os02g04970	2337210	TGGATGATTTGGTCTGGACA	AAGTAGCCTCCGCAAAAACA	59.9	536	93	expressed protein
OsISM11752	LOC_Os02g04970	2337848	CGGAAAGAAATATTATCCACCA	TGTGGAGATACCGTGCAGAA	59.1	206	98	expressed protein
OsISM11753	LOC_Os02g04970	2338139	TCTCTTCCGTTGTTTGGAGG	TATCAAGATTGCGCAGGTCA	60.3	168	69	expressed protein
OsISM11754	LOC_Os02g04970	2338821	TTATCTGGTCTCCATTGGTGG	TGAATCACTGGGAACAACTTCA	60.2	488	82	expressed protein
OsISM11755	LOC_Os02g04970	2339365	CACCAACTATTGAGGATTTCAGC	TGCAAAGACAGGTCATCAGC	60.0	815	71	expressed protein

ISM IDs	Rice gene locus IDs	Physical positions (bp) on chromosomes	Forward primers (5'-3')	Reverse primers (5'-3')	Melting tempearature (T˚C)	Expected product size (bp) in PCR	Expected product size (bp) in Q-PCR	Putative Functions
OsISM11756	LOC_Os02g04970	2340389	TTCGCCATGGTCAATATGAA	ATGTGTGTGTCGAGAATGCC	59.7	170	56	expressed protein
OsISM11757	LOC_Os02g04970	2340650	GCACGTGATGAAGCAAACAT	AGAGCCTTTGGAGCTTCTTG	59.3	1258	99	expressed protein
OsISM11758	LOC_Os02g04970	2341891	GAAGCTCCAAAGGCTCTTCA	ACGAAGCCTCCATGCTGTAA	60.2	165	88	expressed protein
OsISM11759	LOC_Os02g04970	2342337	AGTTCTTTGTGAGCTTGGCG	CTTGCTCCACTTTTTCCACC	60.1	713	81	expressed protein
OsISM11760	LOC_Os02g04970	2342998	ATTTGCAATGGCGAGATACC	TCTAGACAAAAGATCCGAGCG	59.8	171	91	expressed protein
OsISM11761	LOC_Os02g04970	2343384	TTCTGTTTGTGAAGCGGATG	CAGCTCTGAATCTGTGCCAA	60.0	176	92	expressed protein
OsISM11762	LOC_Os02g04970	2343609	CACTAACAGAAGCACTCTATA TGCTCA	AAGAATATCCGTCAGTGCTTCA	59.6	188	90	expressed protein
OsISM11763	LOC_Os02g04970	2343843	TTCGGAAGGAGTCAGGAATG	CCCACTCTATTAGGGCAGCA	60.2	176	80	expressed protein
OsISM11764	LOC_Os02g04970	2343999	TGCTGCCCTAATAGAGTGGG	CCATCACAGGCATCATCATC	60.1	232	72	expressed protein
OsISM11765	LOC_Os02g04970	2344275	GCCATTGGGAGACTCTTGAA	GTGTTTCCGCAACAATGACA	60.4	245	79	expressed protein
OsISM11766	LOC_Os02g04970	2344503	CATTGTTGCGGAAACACTTC	CATCCTGTTACCAGCCTTGG	59.8	177	95	expressed protein
OsISM11767	LOC_Os02g04970	2344771	TGCTGGTCGAGTACTTGGAA	GATGGCAGTGTATGGGAACC	59.8	184	96	expressed protein
OsISM11768	LOC_Os02g04970	2345011	GTGCGACAGGCTCAAGAAGT	CAAGCGATGAAAGCACATCA	60.8	187	90	expressed protein
OsISM11770	LOC_Os02g04980	2353637	TGCCTCAGCAACAACAGTTC	GGAGCTTTCTCTTCTTTGCG	59.7	217	60	transposon protein putative unclassified
OsISM11771	LOC_Os02g04980	2354059	AATGCATTGGTCTTGCTGTG	TGATTTTCAACTTCAGCTTTGA	58.7	455	83	transposon protein putative unclassified
OsISM11772	LOC_Os02g04980	2354869	GTGTGGGGGAGTCCTGTAGA	TCTGGTGTGGCAACAATTTC	59.8	407	93	transposon protein putative unclassified
OsISM11773	LOC_Os02g04980	2355533	TGAAACTAAACGATGTTTGGG A	TCATAATGTGCCACCAGTGC	60.0	235	98	transposon protein putative unclassified
OsISM11774	LOC_Os02g04980	2355965	TTGGGTTTCAAGAGAATGTCG	ATAGGAGCAGGTGTAGCATTG	58.6	309	98	transposon protein putative unclassified

ISM IDs	Rice gene locus IDs	Physical positions (bp) on chromosomes	Forward primers (5'-3')	Reverse primers (5'-3')	Melting tempearature (T°C)	Expected product size (bp) in PCR	Expected product size (bp) in Q-PCR	Putative Functions
OsISM11775	LOC_Os02g05020	2378098	CCGAAGCGCTTGTACCAAT	GCAGTAAATTCTTCCGACGC	60.5	165	76	expressed protein
OsISM11776	LOC_Os02g05020	2378338	GCCATTGGGAGACTCTTGAA	CAACCTCGGATGCAAATCTT	60.1	8345	57	expressed protein
OsISM11777	LOC_Os02g05020	2386716	GACCCTGAGATTGAGCTACCA	CTGCCTCTGTGCCAGACATA	59.7	212	99	expressed protein
OsISM11778	LOC_Os02g05030	2390283	CGCGTCTCATGATTGTTTCA	AGGGCACCAAATCTAAGCAA	60.3	183	86	sucrose-phosphatase putative expressed
OsISM11779	LOC_Os02g05030	2390682	GGAGACAGCAAATGTTTCCG	GCTGACCTTATGTGGACGCT	60.5	180	65	sucrose-phosphatase putative expressed
OsISM11780	LOC_Os02g05030	2390870	TGACAAAAAGAGTGCTCAGGA A	CCTGGCCACCGCTATAGAT	60.0	249	93	sucrose-phosphatase putative expressed
OsISM11781	LOC_Os02g05030	2391230	CTGGCAACGATGCAGAATTA	TGAAGTAGTTCCTCTTGGGCA	59.8	176	77	sucrose-phosphatase putative expressed
OsISM11782	LOC_Os02g05030	2391613	GGCTGAGGTTCCAAAGTCTG	AAGCCCAGCAGGATGAATAA	59.8	365	86	sucrose-phosphatase putative expressed
OsISM11783	LOC_Os02g05030	2392091	GGGTTGGCTGGTGAGATTTA	TCAGACAACACGACCACACA	59.8	1711	60	sucrose-phosphatase putative expressed
OsISM11784	LOC_Os02g05030	2393782	TGTGTGGTCGTGTTGTCTGA	ACCCTTTGAGCCATGTTTTG	59.9	258	99	sucrose-phosphatase putative expressed
OsISM11785	LOC_Os02g05040	2397781	AGATCATCGCCGACATCTTC	ACGCGATGATGGTTTCTAGC	60.2	654	98	cyclin-related protein putative expressed
OsISM11786	LOC_Os02g05040	2398415	GCTAGAAACCATCATCGCGT	TTCACAAAGCCATACTGCTCA	59.9	510	90	cyclin-related protein putative expressed
OsISM11787	LOC_Os02g05040	2398943	CCGCAAACTACTTGTCTCCTG	TGTCATCCCGTCTTGTTTGA	60.0	280	99	cyclin-related protein putative expressed
OsISM11788	LOC_Os02g05040	2399203	TCAAACAAGACGGGATGACA	CAAATTGAATTGATATGTGCCG	60.1	184	92	cyclin-related protein putative expressed
OsISM11789	LOC_Os02g05040	2399441	AAGGATAACATGCTCCGTGC	GAAAACTCGCCCATAAACCA	60.0	391	69	cyclin-related protein putative expressed
OsISM11790	LOC_Os02g05040	2399831	CTCACATATCCTCCGCGTTT	GCCTGGAGCTTCACTGTCTA	59.1	613	99	cyclin-related protein putative expressed
OsISM11791	LOC_Os02g05040	2400521	TGATGACAAGGGAGAGCTTCA	TTGTGCACACAGACTCGTGA	60.5	155	76	cyclin-related protein putative expressed
OsISM11792	LOC_Os02g05040	2400782	TTTTGTGTTCTTTTGGACATGC	CAGACTTGTGTTCAAGGTGCTT	59.7	191	98	cyclin-related protein putative expressed

ISM IDs	Rice gene locus IDs	Physical positions (bp) on chromosomes	Forward primers (5'-3')	Reverse primers (5'-3')	Melting tempearature (T˚C)	Expected product size (bp) in PCR	Expected product size (bp) in Q-PCR	Putative Functions
OsISM11793	LOC_Os02g05040	2401143	AAGTGGAACGACAAACTCCG	AGAACAGGTCCAGCATCACC	60.1	269	81	cyclin-related protein putative expressed
OsISM11794	LOC_Os02g05040	2401480	CCGTTTATCGTACAGCCCAG	CTGATGAAAGAGTGCTTCTGGA	60.1	180	84	cyclin-related protein putative expressed
OsISM11795	LOC_Os02g05040	2402049	CCCAAAGGAATGCCAAAATA	AAAAGTGTGGCCTGACGACT	59.8	196	88	cyclin-related protein putative expressed
OsISM11796	LOC_Os02g05040	2402326	GCTTCTGTTGTTCTCGGGAT	TCATTAAGGAAAACGCCACC	59.6	341	78	cyclin-related protein putative expressed
OsISM11797	LOC_Os02g05040	2402637	CAAGTTTTCAGGTGGCGTTT	AGTAAACAAGGATCGACGGC	59.7	252	87	cyclin-related protein putative expressed
OsISM11798	LOC_Os02g05040	2402933	GGCACCTCTTATCCCGATCT	TCACAGCCTGAAGTTTGGTG	60.2	360	93	cyclin-related protein putative expressed
OsISM11799	LOC_Os02g05040	2403390	ATCCATGGCCTCTACAATCA	TGTCATCAGGGGAGAAGTCA	58.6	224	99	cyclin-related protein putative expressed
OsISM11800	LOC_Os02g05040	2403657	TCCAGGTTGTGATGAGGATTC	TTCCCCAAAGGTGTCATTGT	60.1	155	77	cyclin-related protein putative expressed
OsISM11801	LOC_Os02g05040	2403841	ATCTGTGCCCACAGCAAATC	CGAGCATCTTGCAGTCTGAG	60.5	326	86	cyclin-related protein putative expressed
OsISM11802	LOC_Os02g05040	2404313	CACGGGAGCAGTCCATATTT	ATCAAGGCTTTGCTGGAGAA	60.0	238	65	cyclin-related protein putative expressed
OsISM11803	LOC_Os02g05080	2415454	GGAGAGGCCGAACCAGAG	GCGACAACTTCGACAGATCA	60.5	470	72	hypothetical protein
OsISM11804	LOC_Os02g05080	2416215	GTCTCTTCTACGCAGACCCG	TTCATCCACGTGAGGTTGTC	59.8	131	92	hypothetical protein
OsISM11805	LOC_Os02g05090	2431150	GTGACAGTGGTGGCTGTGG	AATATGATATCCCGGCCTTTG	60.6	686	87	hypothetical protein
OsISM11807	LOC_Os02g05110	2439961	CCTGATTAACTGTTCTTTACTCACGA	GAGTAGCTCACCGCACGTC	59.9	310	98	transposon protein putative unclassified
OsISM11808	LOC_Os02g05110	2440261	TGAGCTACTCACGGTTCACG	CCGACACATCGGTAGGAGG	60.8	145	68	transposon protein putative unclassified
OsISM11809	LOC_Os02g05110	2440704	CTGGACCTACGAAGAGGTGG	GAGCAAGTACGCCTCCAAAC	59.8	186	99	transposon protein putative unclassified
OsISM11810	LOC_Os02g05110	2440952	GCACTACGCCAGGAGCAT	CCTCGTACAGTGACTCCTCGT	59.7	233	99	transposon protein putative unclassified
OsISM11811	LOC_Os02g05110	2441210	GATGGGTACTCTGTGGTGCC	TACGACCGCCTAATCTGGAC	60.2	143	61	transposon protein putative unclassified

ISM IDs	Rice gene locus IDs	Physical positions (bp) on chromosomes	Forward primers (5'-3')	Reverse primers (5'-3')	Melting tempearature (T˚C)	Expected product size (bp) in PCR	Expected product size (bp) in Q-PCR	Putative Functions
OsISM11812	LOC_Os02g05110	2441568	AGCCCTTTCCAGTACCGTTT	TCTCTAGTGCTCGCAACGTG	60.2	355	98	transposon protein putative unclassified
OsISM11813	LOC_Os02g05110	2441920	AGACCTCTATGCCAGGCACC	CCTAAACGTTGGATCGTCGT	60.8	191	97	transposon protein putative unclassified
OsISM11814	LOC_Os02g05110	2442277	CACGTTCCTAGGCCGACTC	GGGGCACAACAGAAGACCTA	60.5	166	89	transposon protein putative unclassified
OsISM11815	LOC_Os02g05110	2442423	TAGGTCTTCTGTTGTGCCCC	CCAATGACGTCCTCTGTCG	60.2	311	46	transposon protein putative unclassified
OsISM11816	LOC_Os02g05130	2451316	GGTGATAATTGATAATGCAAAAACC	GGCGATGCATGTATTGAAAA	59.7	276	84	hypothetical protein
OsISM11817	LOC_Os02g05130	2451702	TTGAAGACAGTGCGTTCGTC	TCATTTAAAATAGAACCAAATATGCTG	59.5	876	57	hypothetical protein
OsISM11818	LOC_Os02g05150	2461237	AAGACTCGTCAAGGCACGAT	GTCTGACCGCCGGAGTAATA	60.0	430	96	retrotransposon protein putative unclassified
OsISM11819	LOC_Os02g05160	2465212	GCTTCGCATGGGAATATCAT	TCTTCTCCACCGGGACTATG	60.0	225	70	retrotransposon protein putative unclassified
OsISM11820	LOC_Os02g05160	2465530	TGATCAAAGATGCCTCAGGTC	TGCCCTTTGATATGTTGCAC	59.9	188	51	retrotransposon protein putative unclassified
OsISM11821	LOC_Os02g05160	2465872	AGTGTGCTTTTAGCGTGTCG	CCACGTAAAATCGGCTTCTT	59.2	242	84	retrotransposon protein putative unclassified
OsISM11822	LOC_Os02g05160	2466282	ATTACATATTTGAGCCGCCG	ATCAAAGCATACGCCCACTT	59.8	212	84	retrotransposon protein putative unclassified
OsISM11823	LOC_Os02g05170	2470500	ATGTCTTGTTCCGCCAGC	GTCATGCGACAGACTTGGAA	59.8	191	91	retrotransposon protein putative Ty3-gypsy subclass
OsISM11824	LOC_Os02g05170	2471793	CTGTTGGCATTCATCAAGGA	ACAGGAGGAGAAGGAGGAGG	59.7	391	56	retrotransposon protein putative Ty3-gypsy subclass
OsISM11825	LOC_Os02g05170	2472247	ACAACCAACCACTCCTGTCA	TGCTTGATGTTTCTTCTTTTGC	59.3	178	96	retrotransposon protein putative Ty3-gypsy subclass
OsISM11826	LOC_Os02g05220	2498491	CCCCCTCTGCTCCATTTAGT	CGATCCTCCCCATCAAATC	60.3	1396	95	retrotransposon protein putative unclassified
OsISM11827	LOC_Os02g05220	2500036	ACCAGGAGGTCATTCCACAG	GCTTCTATGGCGTTTTGTCC	59.8	177	80	retrotransposon protein putative unclassified
OsISM11829	LOC_Os02g05280	2538195	GCTCCATCGGCTGAGTACG	AGCCGTGCTTGATTCTGAGT	61.0	240	52	hypothetical protein
OsISM11830	LOC_Os02g05290	2540056	GGCGTACCAAAAGACGTCAG	CATGGCCGGTGTATGAGATA	60.0	577	95	hypothetical protein

ISM IDs	Rice gene locus IDs	Physical positions (bp) on chromosomes	Forward primers (5'-3')	Reverse primers (5'-3')	Melting tempearature (T°C)	Expected product size (bp) in PCR	Expected product size (bp) in Q-PCR	Putative Functions
OsISM11832	LOC_Os02g05320	2552767	AGTGTCATGCTTTCACGGTT	CTGCCCAGTACTGCATTTCA	59.0	248	98	phosphatidate cytidylyltransferase family protein putative expressed
OsISM11833	LOC_Os02g05320	2553130	CCTGGCATACTTGTTCCACC	CCACTCCATGACACAGCAAA	60.5	201	87	phosphatidate cytidylyltransferase family protein putative expressed
OsISM11834	LOC_Os02g05320	2553324	GGAGTGGCTGCTGTGATCTT	CCACTTGAGACCAAAATGGC	60.5	368	87	phosphatidate cytidylyltransferase family protein putative expressed
OsISM11835	LOC_Os02g05320	2553704	CTGTGGTGATATGCTGGCTC	GTGCGCGAATAAATGCTTCT	59.8	171	72	phosphatidate cytidylyltransferase family protein putative expressed
OsISM11836	LOC_Os02g05320	2553879	AACTCGAAGCGAGATAGGCA	AGCCAAAACTTGAAGGGAGC	60.4	167	92	phosphatidate cytidylyltransferase family protein putative expressed
OsISM11837	LOC_Os02g05320	2554163	TCATGGACGCGTCTTATTCA	GCATAAAGCAAGCCGTTCAC	60.5	247	94	phosphatidate cytidylyltransferase family protein putative expressed
OsISM11838	LOC_Os02g05320	2554505	CCATCTCGTTGCTGTCCTAA	TGCACCAAATGCTAAGTCCA	59.6	152	74	phosphatidate cytidylyltransferase family protein putative expressed
OsISM11839	LOC_Os02g05320	2554655	CAGCATTTGCCGTTTTCTTA	ACTGATGCACAATATGCCCA	59.5	192	76	phosphatidate cytidylyltransferase family protein putative expressed
OsISM11840	LOC_Os02g05320	2554868	ATCGCGATTCGGAAATTCTA	AGGTCGGTCATTGAATCCTG	59.8	189	93	phosphatidate cytidylyltransferase family protein putative expressed
OsISM11841	LOC_Os02g05320	2555070	CTGGAATTCTCAGCTTGGGT	TCACTTCCTGTCTTGCTCCA	59.4	176	89	phosphatidate cytidylyltransferase family protein putative expressed
OsISM11842	LOC_Os02g05330	2558839	TGACAGCAAGATGCAGGAAC	GGCCCATGTCATCAAAACTC	60.2	225	93	DEAD-box ATP-dependent RNA helicase putative expressed
OsISM11843	LOC_Os02g05330	2559046	GTTTTGATGACATGGGCCTC	CAATCCCCCTTTGCTGAATA	60.1	178	91	DEAD-box ATP-dependent RNA helicase putative expressed
OsISM11844	LOC_Os02g05330	2559568	GTGCTGGATGAAGCTGATGA	TGGATCTTTGGTGGAAGGAG	60.0	570	84	DEAD-box ATP-dependent RNA helicase putative expressed
OsISM11845	LOC_Os02g05340	2563874	GGACGAGAAGAAGGATGACG	CTCCTTGAGCGCCAAATC	59.6	184	50	proteasome/cyclosome repeat containing protein expressed
OsISM11846	LOC_Os02g05340	2564108	GAAGCTCGCGCTTGAGAGTA	GATGTCATTGAGCTTGTGGC	60.1	303	58	proteasome/cyclosome repeat containing protein expressed
OsISM11847	LOC_Os02g05340	2564436	CGGCCACACTATGGAACACT	CACTGATAAAATGTCGGCCA	60.3	183	88	proteasome/cyclosome repeat containing protein expressed
OsISM11848	LOC_Os02g05340	2564623	CTTTGACAATGTCCGCTGAA	CCGATATCACCTTCAGAGCC	59.7	183	71	proteasome/cyclosome repeat containing protein expressed
OsISM11849	LOC_Os02g05340	2564786	GGCTCTGAAGGTGATATCGG	AATTCTTGTGCAATTTCGCC	59.9	764	72	proteasome/cyclosome repeat containing protein expressed

ISM IDs	Rice gene locus IDs	Physical positions (bp) on chromosomes	Forward primers (5'-3')	Reverse primers (5'-3')	Melting tempearature (T˚C)	Expected product size (bp) in PCR	Expected product size (bp) in Q-PCR	Putative Functions
OsISM11850	LOC_Os02g05340	2565529	AGGCGAAATTGCACAAGAAT	ATAAGAGCGTCAATCGGCAT	59.7	526	61	proteasome/cyclosome repeat containing protein expressed
OsISM11851	LOC_Os02g05340	2566035	ATGCCGATTGACGCTCTTAT	TCAACTGCTTCAGGTTCAGC	59.4	228	81	proteasome/cyclosome repeat containing protein expressed
OsISM11852	LOC_Os02g05340	2566242	TGCTGAACCTGAAGCAGTTG	CATGCTCAACTAGCAAATCAAGA	59.9	164	63	proteasome/cyclosome repeat containing protein expressed
OsISM11853	LOC_Os02g05340	2566386	TGATTTGCTAGTTGAGCATGTTG	ATGTCATCAGGAGCAGGGAG	60.3	292	94	proteasome/cyclosome repeat containing protein expressed
OsISM11854	LOC_Os02g05340	2566740	GGATTGCTCTGCTCCTTGAC	CGGTGTAGACCTGCTTCACA	59.9	131	55	proteasome/cyclosome repeat containing protein expressed
OsISM11855	LOC_Os02g05340	2566858	CAGGTCTACACCGCAACTGA	GCAGCTATCTCGTCGTCAATC	59.9	203	99	proteasome/cyclosome repeat containing protein expressed
OsISM11856	LOC_Os02g05340	2567144	TCGAAGTGATGGAAGCAAAA	GAATCAAGACTGGAGCTGGC	59.7	168	83	proteasome/cyclosome repeat containing protein expressed
OsISM11857	LOC_Os02g05340	2567344	ATGCATTCGTAAATGCTGGA	GAAGCTCCGCTACTGGAAGA	59.4	154	74	proteasome/cyclosome repeat containing protein expressed
OsISM11858	LOC_Os02g05340	2567517	AACAAGGAACATGGGAAAGC	TCAAGCTGAGCAAGTCCAGA	59.4	191	84	proteasome/cyclosome repeat containing protein expressed
OsISM11859	LOC_Os02g05340	2567770	TCACATGTGGTGTGAAGAATGA	CCCAATCCGAATGTTTGTGT	60.3	207	87	proteasome/cyclosome repeat containing protein expressed
OsISM11860	LOC_Os02g05340	2567976	GGCAATCTTGGGTCTTGGTA	CAGAGTCCCCCAACACAGTT	60.0	172	84	proteasome/cyclosome repeat containing protein expressed
OsISM11861	LOC_Os02g05340	2568283	CCATTATACGCCTGCTTCCT	GAAACTTCTGCCGTAGCCTCT	59.6	508	80	proteasome/cyclosome repeat containing protein expressed
OsISM11862	LOC_Os02g05340	2568846	GCATATGCTGGAACAGGAAA	TGGTGGGTTTCACCTTTCTC	59.3	173	84	proteasome/cyclosome repeat containing protein expressed
OsISM11863	LOC_Os02g05340	2569133	GGCAGTTCCTCTTGCTCTTG	CAGTCTGCTAAGCGTGTCCA	60.2	571	74	proteasome/cyclosome repeat containing protein expressed
OsISM11864	LOC_Os02g05340	2569700	ACTGAGTCATGATGCGGATG	TACCAGCACCAATGAAACCC	60.2	164	69	proteasome/cyclosome repeat containing protein expressed
OsISM11865	LOC_Os02g05340	2569881	GCTGGTATGCTTCGCAATCT	TTCCCAAGGTGAACAAGTCC	60.2	201	93	proteasome/cyclosome repeat containing protein expressed
OsISM11866	LOC_Os02g05340	2570076	TGGGAAGGGTTTGTTGACTC	AGTAACAAGGCCTCCAAGGG	60.2	160	80	proteasome/cyclosome repeat containing protein expressed
OsISM11867	LOC_Os02g05340	2570249	GCCTCGATATGAAATCCACC	CCTCATCCACCGTTAAAAGC	59.5	187	97	proteasome/cyclosome repeat containing protein expressed

ISM IDs	Rice gene locus IDs	Physical positions (bp) on chromosomes	Forward primers (5'-3')	Reverse primers (5'-3')	Melting tempearature (T˚C)	Expected product size (bp) in PCR	Expected product size (bp) in Q-PCR	Putative Functions
OsISM11868	LOC_Os02g05340	2570529	CACATTCGACACCCGTATTG	ATACCGAAGTCAGCGGAATG	60.0	167	79	proteasome/cyclosome repeat containing protein expressed
OsISM11869	LOC_Os02g05365	2577384	GGAAGAAGAAGACCCCGACT	TTCAGGAGAATCATCGGAGC	60.0	687	89	expressed protein
OsISM11870	LOC_Os02g05365	2578169	TAAGGGCAAAAAGGCAACAC	TGATTTGATTCCATGGTTGG	59.6	429	94	expressed protein
OsISM11871	LOC_Os02g05370	2582172	GAGGCTCATGTGTTGAGCAG	AGCATTAGAACCAGATGGGG	59.3	1052	99	retrotransposon protein putative Ty3-gypsy subclass expressed
OsISM11872	LOC_Os02g05370	2583240	CAAAAGCTGGTCCAGATCAA	CCTGCATGTCTTCTTCAGCA	59.5	173	73	retrotransposon protein putative Ty3-gypsy subclass expressed
OsISM11873	LOC_Os02g05370	2583394	GCTGAAGAAGACATGCAGGA	GATTAATGGCAACCTCTGGG	59.3	446	61	retrotransposon protein putative Ty3-gypsy subclass expressed
OsISM11874	LOC_Os02g05370	2583799	GCTCTTATGCGTTCCAGGTC	TCTATCGAGTCACCATCGCA	60.1	564	98	retrotransposon protein putative Ty3-gypsy subclass expressed
OsISM11875	LOC_Os02g05370	2584408	CAGTCCTAACGGAAATCAGGA	GCAAAATGTCCTGGATGGAA	60.0	262	83	retrotransposon protein putative Ty3-gypsy subclass expressed
OsISM11876	LOC_Os02g05370	2584644	CAATTGTTCCATCCAGGACA	ATCATCGACAACCTTCGCTT	59.5	161	66	retrotransposon protein putative Ty3-gypsy subclass expressed
OsISM11877	LOC_Os02g05370	2584745	TGGACTGTCTCTTTTTCTCAGG	TAAATTGGATTGGGGTTCCA	59.3	275	97	retrotransposon protein putative Ty3-gypsy subclass expressed
OsISM11878	LOC_Os02g05370	2585000	TGGAACCCCAATCCAATTTA	TTCAGGAAGCTTGTCACCCT	59.9	376	99	retrotransposon protein putative Ty3-gypsy subclass expressed
OsISM11879	LOC_Os02g05420	2607345	GCCGGATTATGATCTCTGCT	AGTTGCGCACGAACTCTACA	59.5	480	84	expressed protein
OsISM11880	LOC_Os02g05420	2608573	AGCTATGATGTGGGGAATGG	GGACTGTTGAGTGTGAACCG	59.5	310	79	expressed protein
OsISM11883	LOC_Os02g05420	2610495	AAAATTGCAAATGGGCTGAG	AGGTGCAGATCATGGGAGTC	60.1	470	81	expressed protein
OsISM11884	LOC_Os02g05420	2610995	TTGTCGCATCAAATGCAAAT	CATTCACGTTTCTGTCAAACAA	59.4	380	99	expressed protein
OsISM11885	LOC_Os02g05440	2614402	GGAAGAAGAAGACCCCGACT	TTCAGGAGAATCATCGGAGC	60.0	687	89	expressed protein
OsISM11886	LOC_Os02g05440	2615187	TAAGGGCAAAAAGGCAACAC	TGATTTGATTCCATGGTTGG	59.6	429	94	expressed protein
OsISM11887	LOC_Os02g05440	2615596	CCAACCATGGAATCAAATCA	AGCATTAGAACCAGATGGGG	59.1	465	85	expressed protein

ISM IDs	Rice gene locus IDs	Physical positions (bp) on chromosomes	Forward primers (5'-3')	Reverse primers (5'-3')	Melting tempearature (T˚C)	Expected product size (bp) in PCR	Expected product size (bp) in Q-PCR	Putative Functions
OsISM11888	LOC_Os02g05440	2616033	GGTTAAACCCCCATCTGGTT	TCCCTCTTCTTGATGTTCCAA	59.8	195	95	expressed protein
OsISM11889	LOC_Os02g05440	2616230	TGCTGAAGAAGAAATGCAGG	ATTAATGGCAACCTCTGGGA	59.3	446	61	expressed protein
OsISM11890	LOC_Os02g05440	2616636	GCTCTTATGCGTTCCAGGTC	TCTATCGAGTCACCATCGCA	60.1	564	98	expressed protein
OsISM11891	LOC_Os02g05440	2617245	CAGTCCTAACGGAAATCAGGA	GCAAAATGTCCTGGATGGAA	60.0	262	83	expressed protein
OsISM11892	LOC_Os02g05440	2617481	CAATTGTTCCATCCAGGACA	ATCATCGACAACCTTCGCTT	59.5	161	66	expressed protein
OsISM11893	LOC_Os02g05440	2617582	TGGACTGTCTCTTTTTCTCAGG	TAAATTGGATTGGGGTTCCA	59.3	275	97	expressed protein
OsISM11894	LOC_Os02g05440	2617837	TGGAACCCCAATCCAATTTA	TTCAGGAAGCTTGTCACCCT	59.9	376	99	expressed protein
OsISM11895	LOC_Os02g05440	2618246	GATGGGGATCAACCTAAGGC	CCCTTCTTAGTGGCCTTTCC	60.4	427	99	expressed protein
OsISM11896	LOC_Os02g05450	2621686	TCTGGAGATGGTTGGAATGG	CGCTGTAGCTCCTTCTCTGG	60.6	792	60	Homeobox domain containing protein expressed
OsISM11897	LOC_Os02g05450	2622574	GATTCCACCAGATGGGTGTC	AAATGACTCGTGGGATTTGC	60.1	156	77	Homeobox domain containing protein expressed
OsISM11898	LOC_Os02g05450	2622770	TGGCGTGTCATCAGAAATGT	CATGGCGGGTAGTTGGAA	60.3	632	61	Homeobox domain containing protein expressed
OsISM11899	LOC_Os02g05450	2623456	CCCATCTGGGGACAAGTTTT	GCCCAGTCCATCATCAGAGT	60.6	221	90	Homeobox domain containing protein expressed
OsISM11900	LOC_Os02g05450	2623922	CCAAAGGCCAAGAAGAGATG	TCATTGCCAATAGGCTCTCC	60.0	220	70	Homeobox domain containing protein expressed
OsISM11901	LOC_Os02g05450	2624252	ATAAGAAAGCCCAACCTCGG	GGCAGGAAGCTCATTTTCAG	60.2	292	93	Homeobox domain containing protein expressed
OsISM11902	LOC_Os02g05450	2624542	CCCGGAGTGTCAAAGAAAAT	CCTATCCCTGAGAGCAGCAC	59.5	393	99	Homeobox domain containing protein expressed
OsISM11903	LOC_Os02g05450	2624914	TGTGCTGCTCTCAGGGATAG	TCTTGACCTTTTGCTGGGAC	59.7	413	61	Homeobox domain containing protein expressed
OsISM11904	LOC_Os02g05450	2625487	AAACTTGCTTGTCGCCAACT	GTGATGGAAGGCTTGATCGT	60.0	180	65	Homeobox domain containing protein expressed
OsISM11905	LOC_Os02g05470	2640182	GGCAAGGGGTCGATGTTC	GGGAACAGATCAATATCTGCAA	60.3	1246	93	CCT motif family protein expressed

ISM IDs	Rice gene locus IDs	Physical positions (bp) on chromosomes	Forward primers (5'-3')	Reverse primers (5'-3')	Melting tempearature (T°C)	Expected product size (bp) in PCR	Expected product size (bp) in Q-PCR	Putative Functions
OsISM11906	LOC_Os02g05470	2641521	GTACCAGGTCCGGAAGGTG	TTGAAGAAGAGATGGGCTCC	59.9	266	77	CCT motif family protein expressed
OsISM11907	LOC_Os02g05490	2656541	GCACATGATCAGCATGGAAG	GTGCTTGCATCAAACCTTCA	60.0	220	83	expressed protein
OsISM11908	LOC_Os02g05490	2657366	ACATCAGGCCAACCTAGCAG	GAATCTGAGTGACAACCGCA	60.1	197	98	expressed protein
OsISM11909	LOC_Os02g05490	2657607	GAGGTGAAGGACAAGCCATT	GGTCGAGTGAACTTTGGAACA	59.6	179	96	expressed protein
OsISM11910	LOC_Os02g05490	2657765	TGTTCCAAAGTTCACTCGACC	CGCCAAGTCTCAATGTTGGT	60.6	175	96	expressed protein
OsISM11911	LOC_Os02g05500	2659970	CAGTGATGCGGATGACCA	GAGTTGCTCCCACCACTCTC	60.0	404	97	pyridoxamine 5'-phosphate oxidase family protein putative expressed
OsISM11912	LOC_Os02g05500	2660463	GAGCTGTTCGCAATCTGATG	TGCATCCGAGACATGACAGT	59.9	214	65	pyridoxamine 5'-phosphate oxidase family protein putative expressed
OsISM11913	LOC_Os02g05500	2660711	GTGGATTTTGCACCTGATCC	GATTCCTTGTGTGGATGGCT	60.1	162	71	pyridoxamine 5'-phosphate oxidase family protein putative expressed
OsISM11914	LOC_Os02g05500	2660854	GCCATCCACACAAGGAATCT	TTGACAGTCCACTCCATCCA	60.0	152	77	pyridoxamine 5'-phosphate oxidase family protein putative expressed
OsISM11915	LOC_Os02g05500	2661027	GTGATGTTGTTCCCTTGCCT	TAAAAGTTGCCCCACTGATG	59.3	753	98	pyridoxamine 5'-phosphate oxidase family protein putative expressed
OsISM11916	LOC_Os02g05500	2661734	TTTTGAAGCACCAACAGTGG	AGTGCCAAAACCTCCAATGA	60.1	298	99	pyridoxamine 5'-phosphate oxidase family protein putative expressed
OsISM11917	LOC_Os02g05500	2662089	GATGGTGGGGAGCAAAGTT	TGCATCATCTACCTCTATTTCGG	60.2	260	91	pyridoxamine 5'-phosphate oxidase family protein putative expressed
OsISM11918	LOC_Os02g05500	2662382	ATACGAGTTCGACAAGGTGC	CGCTGTAATGCAGCTCAAAG	58.8	276	62	pyridoxamine 5'-phosphate oxidase family protein putative expressed
OsISM11919	LOC_Os02g05510	2664829	GGTCTACGTCTTCGAGTCCG	CCAAGCAGTAACAGCACAGC	59.8	170	58	GATA transcription factor 25 putative expressed
OsISM11920	LOC_Os02g05510	2665036	TCATCTTCAGCGGCATACAG	TTTCTCTTTTCCCGGAACCT	60.0	175	78	GATA transcription factor 25 putative expressed
OsISM11921	LOC_Os02g05510	2665191	AGGTTCCGGGAAAAGAGAAA	ACTGGCCTCTATTACGCTGC	59.8	338	98	GATA transcription factor 25 putative expressed
OsISM11922	LOC_Os02g05510	2665573	ACTAGTTCGGAAGGTTCGCC	AGGTGTTGACGCTGCACTAA	60.1	1152	97	GATA transcription factor 25 putative expressed
OsISM11923	LOC_Os02g05510	2666770	TGTGGACTTATGTGGGCAAA	CACAGGGGGACCTTTTGTTA	59.9	562	58	GATA transcription factor 25 putative expressed

ISM IDs	Rice gene locus IDs	Physical positions (bp) on chromosomes	Forward primers (5'-3')	Reverse primers (5'-3')	Melting tempearature (T˚C)	Expected product size (bp) in PCR	Expected product size (bp) in Q-PCR	Putative Functions
OsISM11924	LOC_Os02g05510	2667312	TAACAAAAGGTCCCCCTGTG	AATTGTGCTGCTCGACACCT	60.3	433	97	GATA transcription factor 25 putative expressed
OsISM11925	LOC_Os02g05520	2668625	ACGAGGAGTTGGAGTCGCT	TGCAGTTCACCCTTAAAGCC	60.1	445	94	expressed protein
OsISM11926	LOC_Os02g05520	2669061	TGAACTGCAGCTCTGAATCC	CAGCCTTCTCAATCGCTCAT	59.8	241	87	expressed protein
OsISM11927	LOC_Os02g05520	2669283	TGAGCGATTGAGAAGGCTGT	CCTTTTCCAGTTCTTCCTTCA	59.8	596	99	expressed protein
OsISM11928	LOC_Os02g05520	2669867	AACTGGAAAAGGCAAACACG	GCTGTTCATTTCACTGCCAA	60.0	177	90	expressed protein
OsISM11929	LOC_Os02g05520	2670022	GCTTGGCAGTGAAATGAACA	CTTCATTGGTTGCTTGCTGA	59.9	157	75	expressed protein
OsISM11930	LOC_Os02g05520	2670247	TTGGAGCAGGTTACTGCTGA	TGCATGTCATTTTTCTCCTCC	59.8	197	99	expressed protein
OsISM11931	LOC_Os02g05520	2670410	TCATTGTTGAACAGGAGGAGA	GCTCTGCCTGCTTCATCTTC	59.3	323	91	expressed protein
OsISM11932	LOC_Os02g05530	2674198	CCTTCCGGAAACATCAGGT	TCTCCAGTGGGCTGAGTTCT	59.9	1079	77	hypothetical protein
OsISM11933	LOC_Os02g05530	2675315	AGTTGTCTCCCTGCTGCATT	GGAGTTGGACACGACAGCC	60.8	582	94	hypothetical protein
OsISM11934	LOC_Os02g05540	2680007	CAAAAGACAATTCATGGCCC	TACCAACCTCTGGGCGTTAC	60.2	153	85	hypothetical protein
OsISM11935	LOC_Os02g05540	2680129	TAATGGTCAAGGTAACGCCC	ACTGTCATTCCACCTTTGGC	59.9	202	52	hypothetical protein
OsISM11937	LOC_Os02g05550	2681735	AAGTCTATGAGGCGCTCCAA	GCTCAAATCTGCCTCTGACC	60.0	105	72	retrotransposon protein putative Ty1-copia subclass
OsISM11938	LOC_Os02g05550	2681851	AACGACAGCAATGATCGTGA	GGCCTTACCATCAGATTTCC	59.4	153	86	retrotransposon protein putative Ty1-copia subclass
OsISM11939	LOC_Os02g05550	2682307	GCACACTTGATGCAGAAGGA	AGCAGCGGTAACAGAACCAT	59.9	152	79	retrotransposon protein putative Ty1-copia subclass
OsISM11940	LOC_Os02g05550	2683623	GAGCCAGCCACATACACTGA	GCTCAAGATCATGCATAGCAA	59.4	328	98	retrotransposon protein putative Ty1-copia subclass
OsISM11941	LOC_Os02g05550	2684738	TGATTTTGCTGCAGATTTGG	TCAATGTGCTTTGTCCTCTCA	59.6	288	88	retrotransposon protein putative Ty1-copia subclass
OsISM11942	LOC_Os02g05560	2695864	GGAGCACAACCGGTTAATTT	AACTCATCCAACAACTCCTCAA	58.8	248	96	retrotransposon protein putative unclassified

ISM IDs	Rice gene locus IDs	Physical positions (bp) on chromosomes	Forward primers (5'-3')	Reverse primers (5'-3')	Melting tempearature (T°C)	Expected product size (bp) in PCR	Expected product size (bp) in Q-PCR	Putative Functions
OsISM11943	LOC_Os02g05560	2696996	TCCATACACCTTGGCAACAA	TCCTTTTCATACCCACCGAC	59.9	196	77	retrotransposon protein putative unclassified
OsISM11944	LOC_Os02g05560	2697815	ATCCTCAATCGGATGGACAG	CGAAGCTCCTCCATGTCTTC	59.9	727	73	retrotransposon protein putative unclassified
OsISM11945	LOC_Os02g05560	2698757	GGGTCATGTAATGATTCGGG	CTCGTGTTCTCCAGCAGCTT	60.4	326	97	retrotransposon protein putative unclassified
OsISM11947	LOC_Os02g05560	2699520	CAGATTCCTCTGCGAGGGT	GCCAACTTACCAATTCCACC	59.8	223	84	retrotransposon protein putative unclassified
OsISM11949	LOC_Os02g05600	2724093	TGTGCAATGCAAGAACAATTC	TGCCATCATCAATTTTGCTC	59.7	459	96	expressed protein
OsISM11951	LOC_Os02g05610	2729472	GTTTCGTGCAGATTCGGATT	ATCACATCCAGAATCCAGGC	60.0	1067	94	ZOS2-03 - C2H2 zinc finger protein expressed
OsISM11952	LOC_Os02g05610	2730538	TGCCCCCTATAATGGTCAAA	CCCGGTGAAACATCATCTTC	60.2	306	91	ZOS2-03 - C2H2 zinc finger protein expressed
OsISM11953	LOC_Os02g05610	2730824	GAAGATGATGTTTCACCGGG	TGGTGATCAAAATAATCCTGTTTC	59.8	216	93	ZOS2-03 - C2H2 zinc finger protein expressed
OsISM11954	LOC_Os02g05610	2731014	CAGAAACAGGATTATTTTGATCACC	TGTTGGGTGGTACATGTCTTTC	59.9	166	64	ZOS2-03 - C2H2 zinc finger protein expressed
OsISM11955	LOC_Os02g05610	2731156	TTGAAAGACATGTACCACCCA	GCAGCCTTGCAAAGTTCATT	59.6	253	69	ZOS2-03 - C2H2 zinc finger protein expressed
OsISM11956	LOC_Os02g05610	2731422	ATTCTTGATTTGCGGAGTGG	ATCTGCAGGGATTCCATCAC	60.0	193	97	ZOS2-03 - C2H2 zinc finger protein expressed
OsISM11957	LOC_Os02g05610	2732283	CCTCAAGCATCCTGATTTGG	GTGCCACCTGGAGCATTT	60.1	191	97	ZOS2-03 - C2H2 zinc finger protein expressed
OsISM11958	LOC_Os02g05610	2732464	CAGGTGGCACACCAGTTATG	CCAGGCGTTAGTTCAGAAGG	59.9	731	71	ZOS2-03 - C2H2 zinc finger protein expressed
OsISM11959	LOC_Os02g05610	2733224	AAATGCCCACTCCACCAGTA	AGGTGCAGGAACAAATGGAC	60.2	185	69	ZOS2-03 - C2H2 zinc finger protein expressed
OsISM11960	LOC_Os02g05610	2733417	AGTCATGCAGATGATGCGAC	CATCATCAGGAGCGTCAAGA	59.9	532	99	ZOS2-03 - C2H2 zinc finger protein expressed
OsISM11961	LOC_Os02g05630	2744516	GGAGGAGTTGGAGGAGGAGA	ACCCATAACTGAACTTGCCG	60.4	844	90	protein phosphatase 2C putative expressed
OsISM11962	LOC_Os02g05630	2745418	TGGTGTTGATGGAGAGACCA	ATTGCTGAAAAGGTGCTGCT	60.1	188	95	protein phosphatase 2C putative expressed
OsISM11963	LOC_Os02g05630	2745625	TCATCAGTGATATCAAGTCCGC	GTTGAGCCAGCATCCCTAGT	59.7	190	98	protein phosphatase 2C putative expressed

ISM IDs	Rice gene locus IDs	Physical positions (bp) on chromosomes	Forward primers (5'-3')	Reverse primers (5'-3')	Melting tempearature (T˚C)	Expected product size (bp) in PCR	Expected product size (bp) in Q-PCR	Putative Functions
OsISM11964	LOC_Os02g05630	2745841	ATCGCTTGCTGGTTGCTAAT	GGGTTTGTGATCCCTTGAAA	59.8	313	87	protein phosphatase 2C putative expressed
OsISM11965	LOC_Os02g05630	2746149	AACCCGACCAGTCAGATGAG	CAGCAAGAACACCACCAACA	60.4	181	91	protein phosphatase 2C putative expressed
OsISM11966	LOC_Os02g05630	2746325	TGCTGTTTCTCGAGCATTTG	GGAACTCAAGGGAGCTATCG	59.5	335	96	protein phosphatase 2C putative expressed
OsISM11967	LOC_Os02g05630	2746663	TCCTTGCTAGCGATGGACTT	TCCTGAATAGGCCTCACCAT	59.7	174	71	protein phosphatase 2C putative expressed
OsISM11968	LOC_Os02g05640	2758059	CGCCCACAACATCCTCTC	CCTGTTCTGGAACCACACCT	60.1	402	92	homeobox associated leucine zipper putative
OsISM11969	LOC_Os02g05680	2776578	GATCGCTCCTTGCCAAGAC	TCAGCCCAAGGGATCTCTAA	60.3	1031	83	apoptosis-inducing factor 2 putative expressed
OsISM11970	LOC_Os02g05680	2777800	GGAGCAGAAGCGAGAGGATA	CCGCCTCCAATTATCAGAAC	59.6	164	83	apoptosis-inducing factor 2 putative expressed
OsISM11971	LOC_Os02g05680	2778339	TGCAATTGGTGACATCACAG	TAGCAGAGCATGCTTATGGG	59.1	548	68	apoptosis-inducing factor 2 putative expressed
OsISM11972	LOC_Os02g05692	2793830	GAAAGAAATGGCCGAAGACA	TGTTCAATCCTGTCCAGAGC	59.5	161	72	zinc finger C3HC4 type domain containing protein expressed
OsISM11973	LOC_Os02g05692	2793970	AGCTCTGGACAGGATTGAACA	ATCATGGGAGGTGAAAGCAC	59.9	174	95	zinc finger C3HC4 type domain containing protein expressed
OsISM11974	LOC_Os02g05692	2794133	CCTCCCATGATCAGCATAGTG	AGCGGTGCTAACAGAGCCTA	60.3	264	99	zinc finger C3HC4 type domain containing protein expressed
OsISM11975	LOC_Os02g05692	2794461	CAAACGCGCAAGTTGTAGAC	CGTCCAACTTCTTCACCCTC	59.6	709	74	zinc finger C3HC4 type domain containing protein expressed
OsISM11976	LOC_Os02g05700	2799219	CCCCATCATCTACGTCAACC	TGGTCCTCTACACTGCAAGAA	59.4	2225	98	OsFBO8 - F-box and other domain containing protein expressed
OsISM11977	LOC_Os02g05720	2814946	TGGAAATCGGTTCCATCATT	CAAGCATACAAGTCCATGCG	60.2	1096	59	pentatricopeptide repeat protein PPR986-12 putative expressed
OsISM11978	LOC_Os02g05760	2833228	GAGCCTGTGAACGGATCAA	CGGATGGTGACCGGTAGTAG	60.1	606	86	hypothetical protein
OsISM11979	LOC_Os02g05760	2833814	CTACTACCGGTCACCATCCG	TCCATTGCTTGAGAACTTCC	59.1	2464	82	hypothetical protein
OsISM11980	LOC_Os02g05760	2836337	CGGTAATTGGCTTAGAGGGG	CCAGAAGTGGAGCCAGTAGG	60.3	172	98	hypothetical protein
OsISM11981	LOC_Os02g05770	2838516	ATCCTGTCGTCGGAGGCT	CTCCAGCGGTGACGGTAG	60.6	162	73	expressed protein

ISM IDs	Rice gene locus IDs	Physical positions (bp) on chromosomes	Forward primers (5'-3')	Reverse primers (5'-3')	Melting tempearature (T°C)	Expected product size (bp) in PCR	Expected product size (bp) in Q-PCR	Putative Functions
OsISM11982	LOC_Os02g05770	2838744	TCGCCATTCAAGAGTTACCC	TTGCGGCTAAGTGACTGATG	60.0	174	75	expressed protein
OsISM11983	LOC_Os02g05770	2838968	TAGCACTGCCGAAACTCCTT	ATCAATCAACTCTCCCGCAC	60.0	2227	99	expressed protein
OsISM11984	LOC_Os02g05770	2841600	AACCACGCTTCGCTTATACC	CGAGCTTACCCATGATGTGA	59.5	166	77	expressed protein
OsISM11985	LOC_Os02g05780	2843014	GCTTTCTCCAACTCGAGGCT	GGCTTGTCCAGATCCACCT	60.6	1119	75	conserved hypothetical protein
OsISM11986	LOC_Os02g05780	2844110	CATGAGGTGGATCTGGACAA	ATCGACAGGGCAACCAATAA	59.9	1686	62	conserved hypothetical protein
OsISM11987	LOC_Os02g05780	2845776	TTATTGGTTGCCCTGTCGAT	CTGGTTTGTTCCTCTTACAGCA	59.9	557	98	conserved hypothetical protein
OsISM11988	LOC_Os02g05780	2846279	CAGGCTGTGGTCCAGTATATGA	CGATTTGCTTATTGCCCTTG	60.3	320	59	conserved hypothetical protein
OsISM11989	LOC_Os02g05780	2846579	CAAGGGCAATAAGCAAATCG	AATCCCTCAGATTCTGGGCT	60.3	337	72	conserved hypothetical protein
OsISM11990	LOC_Os02g05780	2846898	CCCAGAATCTGAGGGATTCA	TTCATCAAATGACTTGGAGCC	60.0	424	87	conserved hypothetical protein
OsISM11991	LOC_Os02g05820	2869996	CTTCTTCCTCCTCCACGTCC	GAAGCTGCAACATCATCCTG	60.3	503	97	protein kinase domain containing protein expressed
OsISM11992	LOC_Os02g05820	2870520	TCACACCAAAGATCTGCTGC	AGAATGGTTTCCCTGCATTG	60.0	331	73	protein kinase domain containing protein expressed
OsISM11993	LOC_Os02g05820	2870916	TACTGGGGGCATCATCTAGG	TGGCACATCCAACTTCCATA	59.9	188	57	protein kinase domain containing protein expressed
OsISM11994	LOC_Os02g05820	2871236	GAAAATCGGCCGCTAATTG	GAGAGGATGCAAAGCTCACC	60.3	184	88	protein kinase domain containing protein expressed
OsISM11995	LOC_Os02g05820	2871352	CAGGTGAACCAGCACCAATA	AGATAAAGGGTTGCAGCCAG	59.5	212	78	protein kinase domain containing protein expressed
OsISM11996	LOC_Os02g05820	2871547	GCTGCAACCCTTTATCTCCA	TTCCTTATTTCAGCCTCCAAA	59.5	179	89	protein kinase domain containing protein expressed
OsISM11997	LOC_Os02g05820	2871699	GGAAGATTTGGAGGCTGAAA	TGTCTCCGATCCCAAGAGG	60.2	209	99	protein kinase domain containing protein expressed
OsISM11998	LOC_Os02g05820	2872414	GCTGCCAAGATTCTCCATTC	ATGCACCATATTCGGGTGG	60.7	545	97	protein kinase domain containing protein expressed
OsISM11999	LOC_Os02g05820	2873584	TTCACACATTCGTTGCATCA	GCCAAGTCATTTTCCAGAGC	59.7	236	98	protein kinase domain containing protein expressed

ISM IDs	Rice gene locus IDs	Physical positions (bp) on chromosomes	Forward primers (5'-3')	Reverse primers (5'-3')	Melting tempearature (T˚C)	Expected product size (bp) in PCR	Expected product size (bp) in Q-PCR	Putative Functions
OsISM12000	LOC_Os02g05830	2875197	GAAGAGGTGCAGCATCCAAT	AGGGGAGGCAGGTAAGACAT	60.1	260	98	ribulose bisphosphate carboxylase small chain chloroplast precursor putative expressed
OsISM12001	LOC_Os02g05830	2875494	ATGTCCAAAGGATGGGTCC	GTCCAGTACCTCCCGTCGTA	59.8	246	99	ribulose bisphosphate carboxylase small chain chloroplast precursor putative expressed
OsISM12002	LOC_Os02g05850	2887449	GAGGCGCTCAGGAAGGTC	CCTCGACGACATGGAGCC	62.0	306	98	transposon protein putative unclassified
OsISM12003	LOC_Os02g05850	2888237	CAGTTCACCACGAACTTCCA	ATCTTCTCGAGCATGCGGT	60.3	205	96	transposon protein putative unclassified
OsISM12004	LOC_Os02g05850	2889102	CCTCACCTTCGACCAGACC	AGCTCGTATCTTCTCGGGGT	60.4	177	95	transposon protein putative unclassified
OsISM12005	LOC_Os02g05850	2890512	CAGATGGACGCTTACGTGC	CGGTCCTCGGGTAGAGTCTT	60.5	341	57	transposon protein putative unclassified
OsISM12006	LOC_Os02g05900	2929207	ACTTCTGGAAAAACACCGGG	TCTTCTCATCTTCTTCGCCTTC	60.7	164	75	ternary complex factor MIP1 putative expressed
OsISM12007	LOC_Os02g05900	2929751	TGGATGTCTGTTGGAAAGGA	AACCTTTGATAGCTGTTCCACAA	59.6	706	85	ternary complex factor MIP1 putative expressed
OsISM12008	LOC_Os02g05900	2930468	TATGAACTGTGATGAGCGGC	TTTCGTGGAACTCCATATGCT	59.7	247	88	ternary complex factor MIP1 putative expressed
OsISM12009	LOC_Os02g05900	2930694	AGCATATGGAGTTCCACGAAA	TCAGCAGCACTGAAAGACTGA	59.7	178	91	ternary complex factor MIP1 putative expressed
OsISM12010	LOC_Os02g05900	2930894	ATGAAGACTCCAGTGCATCG	CGAGTATTTCTTGTGCTCCTCA	59.2	170	85	ternary complex factor MIP1 putative expressed
OsISM12011	LOC_Os02g05900	2931096	ACTCAGCTGCGGAATGTTCT	GATTCCTGAAGCTCCTGACG	60.0	358	76	ternary complex factor MIP1 putative expressed
OsISM12012	LOC_Os02g06040	3012007	AGGTGGTCATTCACCCGAC	ACTGGCCAGACTTGGGGT	60.7	165	71	retrotransposon protein putative unclassified
OsISM12013	LOC_Os02g06160	3076622	ATCGACGACTCCATCAAAGG	GGCTGTATGCGTCGAAAAAT	60.1	127	51	cysteine-rich receptor-like protein kinase 16 precursor putative
OsISM12014	LOC_Os02g06160	3077634	CTACCAAGACTTTCTCGCCG	TAGGGGCTCTCCTTTGTTGT	59.4	408	98	cysteine-rich receptor-like protein kinase 16 precursor putative
OsISM12015	LOC_Os02g06170	3081057	GATGGACGTCGACTCTGACC	TCCACAAATTCAGAGCATTCC	60.4	914	89	expressed protein
OsISM12016	LOC_Os02g06240	3119666	CAACAGCTTCAGAGGAGAGC	CAAGAGGGATTTCAGGTTGC	58.8	201	62	phytosulfokine receptor precursor putative
OsISM12017	LOC_Os02g06290	3141997	GACGAGGACACAGCAGCATA	ACGATGTGGTGTCCCTGTTT	60.2	217	96	rhodanese-like domain containing protein putative expressed

ISM IDs	Rice gene locus IDs	Physical positions (bp) on chromosomes	Forward primers (5'-3')	Reverse primers (5'-3')	Melting tempearature (T˚C)	Expected product size (bp) in PCR	Expected product size (bp) in Q-PCR	Putative Functions
OsISM12018	LOC_Os02g06290	3142194	AAACAGGGACACCACATCGT	GGCTTCCAGCTCCTTCTTG	60.2	204	68	rhodanese-like domain containing protein putative expressed
OsISM12019	LOC_Os02g06290	3142389	CTGGAAGCCCTCCCAATC	CCTTGTCAAAATCCTTCCCA	60.2	303	98	rhodanese-like domain containing protein putative expressed
OsISM12020	LOC_Os02g06290	3142703	GGTGCTCGCAACATTTCCTA	ATCCTTGCCGAAGAGTGAAG	60.3	251	97	rhodanese-like domain containing protein putative expressed
OsISM12021	LOC_Os02g06290	3142934	CTTCACTCTTCGGCAAGGAT	TAATTACCGCGTCCAAAAGG	59.7	708	94	rhodanese-like domain containing protein putative expressed
OsISM12022	LOC_Os02g06290	3143609	TCGCGACTAAGGACCTTTTG	CAGGTTGCTGGCTTTCACTT	60.4	192	94	rhodanese-like domain containing protein putative expressed
OsISM12023	LOC_Os02g06310	3154656	AGAACCATCTCCACCCACAG	CACTTCTTCCATCCTGACCA	59.3	165	71	hypothetical protein
OsISM12024	LOC_Os02g06310	3154861	TTCTATTTCGTCCCACCCTG	ATCAAAGCCCAAAACTGGTG	59.9	515	60	hypothetical protein
OsISM12025	LOC_Os02g06310	3155342	TTGAGCAGAAAATGCACCAG	CAGATTCTGATATCACCATGCAA	60.0	212	87	hypothetical protein
OsISM12026	LOC_Os02g06310	3156089	AAGATCATTTGGACTTGGCG	AGAAGGGAGCGAGGAGAGAA	60.3	1326	81	hypothetical protein
OsISM12027	LOC_Os02g06320	3159298	GCAACATCCCCAACAAGC	AGTGGTCGTCGAGCAGCC	61.4	197	55	RNA recognition motif 2 domain containing protein expressed
OsISM12028	LOC_Os02g06320	3159545	TACGATCTCGTCTACGTCCG	TTGCTGCTCCTCTCCTTGTT	59.5	546	57	RNA recognition motif 2 domain containing protein expressed
OsISM12029	LOC_Os02g06320	3160193	CAAGATCATCGACATCCGC	TCGGTGTCGCACTCGTAGTA	60.3	260	91	RNA recognition motif 2 domain containing protein expressed
OsISM12030	LOC_Os02g06350	3172119	GTCAGGGAGGTGGGCAGT	CGGTAAATCCTCGGTTTCGT	61.2	2011	97	hypothetical protein
OsISM12031	LOC_Os02g06350	3174247	GTGCTATGGACGTATGCCAA	TCCCGTGGTGCTAGTAGCTG	60.5	503	96	hypothetical protein
OsISM12032	LOC_Os02g06360	3180253	CAAGATTGGCAAATCCCTCA	TGCACAATGGAAGCTCATTT	60.1	723	69	uncharacterized protein CPn 0526/CP 0226/CPj0526/CpB0547 putative expressed
OsISM12033	LOC_Os02g06360	3181111	GTGGGCAAGCAATTTTCAAT	TGTACTGCCATTGCATCAGC	60.4	1267	83	uncharacterized protein CPn 0526/CP 0226/CPj0526/CpB0547 putative expressed
OsISM12034	LOC_Os02g06400	3197546	CTCGCGTTCCTCCTCCTC	AGCGTGATCCCATCCTTCT	60.3	172	93	growth regulator related protein putative expressed
OsISM12035	LOC_Os02g06400	3197675	CCTAGAATGCGCCCTACTTG	AGCACGTGGATTCTCCCATA	60.2	189	93	growth regulator related protein putative expressed

ISM IDs	Rice gene locus IDs	Physical positions (bp) on chromosomes	Forward primers (5'-3')	Reverse primers (5'-3')	Melting tempearature (T˚C)	Expected product size (bp) in PCR	Expected product size (bp) in Q-PCR	Putative Functions
OsISM12036	LOC_Os02g06400	3197850	AGAATCCACGTGCTGCTACC	AGGTTTCATTCTCAGGTGGG	59.6	837	88	growth regulator related protein putative expressed
OsISM12037	LOC_Os02g06400	3198711	AGGGTGGACTGAACCAACAG	GCAAAATAAGCGTTGCCTTC	59.9	526	79	growth regulator related protein putative expressed
OsISM12038	LOC_Os02g06400	3199235	GCCAGTTCTAAAGCAGGATCA	GCGCACATCATCCTTCAAAT	60.2	927	98	growth regulator related protein putative expressed
OsISM12039	LOC_Os02g06400	3200160	GCATTGTCCGAGACATTCCT	GAGCAGATGCATACTTTGGGA	60.2	848	97	growth regulator related protein putative expressed
OsISM12040	LOC_Os02g06400	3201029	TCCCGAGGATCAAAGAGAAA	CTGCATCGTAGCCGGTTAAT	59.9	303	98	growth regulator related protein putative expressed
OsISM12041	LOC_Os02g06400	3201382	ATAAGCTGGCAGCAAGGATG	AAGGACAGGCCTACCATTCC	60.4	200	95	growth regulator related protein putative expressed
OsISM12042	LOC_Os02g06400	3201602	GGGAGGAGAAAGAGATGATGG	AGCTAATGGCCACAGGTGAG	60.1	197	84	growth regulator related protein putative expressed
OsISM12043	LOC_Os02g06400	3201888	GATATACGTTGCGTCAGGGC	CTGGCGAGCTCCTCCTTG	61.4	677	97	growth regulator related protein putative expressed
OsISM12044	LOC_Os02g06410	3204645	CACCCTGTGGACTCTCCATC	CTGAACGTCTCCATGCAATC	59.9	828	55	CBS domain containing membrane protein putative expressed
OsISM12045	LOC_Os02g06430	3212656	GCCTGCTCAACTCCAAGTTC	CTTGACCATCGCTTGCTTCT	60.3	507	99	DUF292 domain containing protein expressed
OsISM12046	LOC_Os02g06430	3213202	CTTGAATCGCACGCCTTT	GCAGGATGCTTGGTTCATCT	60.1	304	61	DUF292 domain containing protein expressed
OsISM12047	LOC_Os02g06430	3213540	TGAAGCAGCTCAACAACCTG	GACACAGCTTCCAAGGCTTC	60.1	853	62	DUF292 domain containing protein expressed
OsISM12048	LOC_Os02g06430	3214472	GGGAGTTTTGTCGAGCCTTT	GCTATGCTTTGCATCACTTGC	60.8	188	99	DUF292 domain containing protein expressed
OsISM12049	LOC_Os02g06430	3214689	AGGCACTGGAGCGAAAGATA	GCTTCAACTTCCACACGCTT	60.2	190	86	DUF292 domain containing protein expressed
OsISM12050	LOC_Os02g06450	3219200	GGAAGCAGAAGCCTCTCAAG	AGGGCCTTCTCCTCCTCTTT	60.0	241	98	coiled-coil domain-containing protein 72 putative
OsISM12051	LOC_Os02g06490	3234810	GTCGAGTTCCCGCAGTTCT	TTGGCCAAGTAAGATTTGCC	60.2	378	93	vacuolar protein sorting-associated protein 4 putative expressed
OsISM12052	LOC_Os02g06490	3235162	GGGACGGGCAAATCTTACTT	CTTGGAGAGAAGATCCGACG	60.1	588	88	vacuolar protein sorting-associated protein 4 putative expressed
OsISM12053	LOC_Os02g06490	3235875	CGAGGCTTCTCGGAGAGTTA	TTTGTGGCAGCAAGAACAAG	59.9	291	91	vacuolar protein sorting-associated protein 4 putative expressed

ISM IDs	Rice gene locus IDs	Physical positions (bp) on chromosomes	Forward primers (5'-3')	Reverse primers (5'-3')	Melting tempearature (T˚C)	Expected product size (bp) in PCR	Expected product size (bp) in Q-PCR	Putative Functions
OsISM12054	LOC_Os02g06490	3236146	CTTGTTCTTGCTGCCACAAA	GTCGGGTAGCGGAATGTAGA	60.1	201	88	vacuolar protein sorting-associated protein 4 putative expressed
OsISM12055	LOC_Os02g06490	3236327	TCTACATTCCGCTACCCGAC	GTGAGGCTGTGAGGGGTGT	60.6	189	77	vacuolar protein sorting-associated protein 4 putative expressed
OsISM12056	LOC_Os02g06490	3236555	CTTCTCCGGCTCCGACAT	AGAAGAACATGGCATCCTGC	60.8	194	81	vacuolar protein sorting-associated protein 4 putative expressed
OsISM12057	LOC_Os02g06490	3236820	CAGATCACCATGCAGGAGC	ACCTTGTCCAAGTCCGTCCT	60.7	615	84	vacuolar protein sorting-associated protein 4 putative expressed
OsISM12058	LOC_Os02g06510	3251807	GAAACCATTGATGAACAAATGA	GAGGATTCTTGGCGGTCATA	58.7	217	99	myosin heavy chain putative expressed
OsISM12059	LOC_Os02g06520	3256031	CCGTACTCACCCTATGCAGC	GGGTGGCTACTGAAGCAGAG	60.3	134	55	OsFBLD1 - F-box LRR and FBD domain containing protein expressed
OsISM12060	LOC_Os02g06520	3256272	TGTCCTCAGTTGGAGAGGCT	CTTCAGGCGGTTCTTCCTCT	60.4	192	98	OsFBLD1 - F-box LRR and FBD domain containing protein expressed
OsISM12061	LOC_Os02g06530	3265017	TGGTAAGCTCACTTGGAGCC	AACGACACGAGGCAAGAAAT	60.1	199	95	armadillo/beta-catenin repeat family protein putative expressed
OsISM12062	LOC_Os02g06540	3266229	GCATCTTCGGCTACGACATC	GAAGATCACCGGGAAGAACA	60.4	172	81	transporter family protein putative expressed
OsISM12063	LOC_Os02g06540	3266595	CGCCATCTCCTTCAACTTCA	TCCGATATGTACAGCGGTGA	60.7	511	97	transporter family protein putative expressed
OsISM12064	LOC_Os02g06540	3267687	AAGGTGGCACCCAAATGAT	AATCCGATGAATGTTCCCAC	59.9	205	53	transporter family protein putative expressed
OsISM12065	LOC_Os02g06580	3291702	CTGCCTCTTCGTTGGTTCTC	TCCCCAAAGCCAAATGTATC	59.9	448	98	formin putative expressed
OsISM12066	LOC_Os02g06580	3292696	CAAAAGCAAGGTCTCGGTTG	CAGGAACATGCACATCAGGT	60.2	178	99	formin putative expressed
OsISM12067	LOC_Os02g06580	3293332	GCCTGGCATGACATCAAAT	CCAGCACCATAACCAAACAA	59.5	151	75	formin putative expressed
OsISM12068	LOC_Os02g06580	3293601	GTGAGGGCAGAGGAAATTCA	TCCTCCTCATCAGTCGGTTT	59.9	600	99	formin putative expressed
OsISM12069	LOC_Os02g06580	3294320	CCTCTTTGCAGGAAGATGCT	AGTTTCAGAAAAAGGCGGTG	59.5	190	92	formin putative expressed
OsISM12070	LOC_Os02g06588	3311888	GCAAGCGATCTCATGTCTCTC	CGCAGCAGGATCACTTTTTC	60.5	182	97	ATP binding protein putative expressed
OsISM12071	LOC_Os02g06588	3312422	CGTCAGGCTACTTGAAATGTG	CTGCCATCCAGACTACAGCA	59.2	229	82	ATP binding protein putative expressed

ISM IDs	Rice gene locus IDs	Physical positions (bp) on chromosomes	Forward primers (5'-3')	Reverse primers (5'-3')	Melting tempearature (T°C)	Expected product size (bp) in PCR	Expected product size (bp) in Q-PCR	Putative Functions
OsISM12072	LOC_Os02g06588	3312678	TTTTGCCTTTAATGGCTGCT	CATCTGCAGCTTTGTCCTGA	60.0	137	64	ATP binding protein putative expressed
OsISM12073	LOC_Os02g06592	3314637	CCGTCTTATCCTCAGCGGTA	TCTGTCCCAAGAAATCCAGG	60.1	261	91	SNF2 family N-terminal domain containing protein expressed
OsISM12074	LOC_Os02g06592	3314878	CCTGGATTTCTTGGGACAGA	GCTGCTAGCAATGGTTTTCC	59.9	892	60	SNF2 family N-terminal domain containing protein expressed
OsISM12075	LOC_Os02g06592	3315800	TGAAGCTGGCATTCTTGCTA	TCTCAGGAAGATCAGATAAAAC TTCA	59.8	198	96	SNF2 family N-terminal domain containing protein expressed
OsISM12076	LOC_Os02g06592	3316127	CTCAACCAAAGGCAACTCGT	ATAATCAGGTGGGCTTTCCC	60.2	196	99	SNF2 family N-terminal domain containing protein expressed
OsISM12077	LOC_Os02g06592	3316465	CAGCTATTGGGCAACACAGA	TGGGATTGAAACAGGTCCTT	59.6	178	83	SNF2 family N-terminal domain containing protein expressed
OsISM12078	LOC_Os02g06592	3316596	CTCCAGGCTTTGCTTGACAT	GAGCCGTCCAACCGTAAATA	60.2	606	81	SNF2 family N-terminal domain containing protein expressed
OsISM12079	LOC_Os02g06592	3317251	CCAACCATTGATGTGCTCCT	CAAATTCAATCCAAGCCCAC	60.6	144	55	SNF2 family N-terminal domain containing protein expressed
OsISM12080	LOC_Os02g06592	3317428	GATTGGAACCCAATGAAGGA	CCACTTTCCTTTGACCCAGA	59.9	172	65	SNF2 family N-terminal domain containing protein expressed
OsISM12081	LOC_Os02g06592	3317735	TGCTTGATCTGTTTGCTTCG	ACTCTGATCACCACCCGAAC	60.1	174	75	SNF2 family N-terminal domain containing protein expressed
OsISM12082	LOC_Os02g06600	3323910	TCCCTACCAACTCAGCAAGC	TGGAATCCATTGTTCGACAG	59.9	1226	88	receptor-like protein kinase 2 precursor putative
OsISM12083	LOC_Os02g06600	3325970	CCCCAAACTACAATTGCTTGA	CTCCCTTCCCTTCCAATCA	60.0	175	95	receptor-like protein kinase 2 precursor putative
OsISM12085	LOC_Os02g06660	3351142	GGGAATATTCAAAGGCCTGA	CCCAATTTCCATCTCATGCT	59.4	289	68	P21-Rho-binding domain containing protein putative
OsISM12086	LOC_Os02g06660	3351461	ATAGGTTTGGGCACCAGTGA	CTTGATGGTGCAGTTGTGCT	60.1	240	99	P21-Rho-binding domain containing protein putative
OsISM12087	LOC_Os02g06660	3351681	AGCACAACTGCACCATCAAG	CATGCTTCTTGGCTGTTCAA	59.9	464	64	P21-Rho-binding domain containing protein putative
OsISM12088	LOC_Os02g06660	3352156	GATCTTGCCGGAGAAATCTG	CGACTGCCATCCAATCATTA	59.6	432	92	P21-Rho-binding domain containing protein putative
OsISM12089	LOC_Os02g06670	3353813	CCGAGAAGATGAGGTTCCAG	ACCAACACCGAGTCCTTGTC	59.9	446	71	retrotransposon protein putative unclassified expressed
OsISM12090	LOC_Os02g06690	3357170	ACGAGCCGTACAACACCTG	ACCAACACCGAGTCCTTGTC	59.9	562	92	plastocyanin-like domain containing protein putative expressed

ISM IDs	Rice gene locus IDs	Physical positions (bp) on chromosomes	Forward primers (5'-3')	Reverse primers (5'-3')	Melting tempearature (T˚C)	Expected product size (bp) in PCR	Expected product size (bp) in Q-PCR	Putative Functions
OsISM12091	LOC_Os02g06740	3392336	CGGGATGGAACAACGAACTA	CGTGAAACGGAGGGAGTAGA	60.6	356	82	retrotransposon protein putative Ty1-copia subclass
OsISM12092	LOC_Os02g06740	3392669	CTTTCTACTCCCTCCGTTTCA	CTTACCTCCGCCCAGAAATA	58.8	205	57	retrotransposon protein putative Ty1-copia subclass
OsISM12093	LOC_Os02g06740	3392833	TTGGCAGTCAAATACGCCTT	TTAGACGCGGCATCTTTTTC	60.5	1229	66	retrotransposon protein putative Ty1-copia subclass
OsISM12094	LOC_Os02g06740	3397139	TGCCGGCTATCCTTATGAAC	CCGGAGTTTCTTAATTTCCTGA	59.8	130	81	retrotransposon protein putative Ty1-copia subclass
OsISM12095	LOC_Os02g06740	3397358	CGAAGGAGATGGGTTTGAGA	GACTGCGACCAACAGTCAAG	59.8	288	73	retrotransposon protein putative Ty1-copia subclass
OsISM12097	LOC_Os02g06754	3408571	TTTTCTTCGTGGGTCGTAGG	TGTGTAACGCTCCACTCCAG	60.0	196	90	expressed protein
OsISM12098	LOC_Os02g06760	3411319	CGTCCTCGATGGCTTGAA	TCTGGATCTTCCTGACTGGG	60.6	182	75	expressed protein
OsISM12099	LOC_Os02g06760	3411563	GCCCACTTGGAAAGACAAGA	AACCTGCACCATTACGGAAC	60.0	1760	74	expressed protein
OsISM12100	LOC_Os02g06760	3413315	TGCAGGTTGGCAATTAAACA	AACGCAAGGACAGCTCAATC	60.3	1744	94	expressed protein
OsISM12101	LOC_Os02g06800	3431526	AAATGAACCAACATCTGCTCC	CATGGTGTGCCCATAGAGTG	59.5	752	59	hypothetical protein
OsISM12102	LOC_Os02g06800	3432266	GGGCACACCATGTTGTATGA	ATTTCGTGCATCAACCCTGT	60.3	189	90	hypothetical protein
OsISM12103	LOC_Os02g06800	3432497	TTTGGGCATGTCCAGAGATA	TCTCAGGAAACCTATTGCTCG	59.3	178	81	hypothetical protein
OsISM12104	LOC_Os02g06800	3432678	ATGAGCAAATGGGAGGTGAA	ATTCTTGCTTGCCCATTGAT	60.0	149	66	hypothetical protein
OsISM12105	LOC_Os02g06800	3432894	CCAGTCGATGGAGCAAAAA	TGTTTTCAACTTCCGGTGTG	59.7	184	91	hypothetical protein
OsISM12107	LOC_Os02g06820	3440228	AACCAAGCATAGGAACTGCC	TCTTGTATCCCAAATTGATTGC	59.1	218	95	transposon protein putative unclassified
OsISM12108	LOC_Os02g06820	3440711	GCATTTTCAAAAAGTTCCAGC	CAGGATATGGGAGCAAAGGA	59.4	220	84	transposon protein putative unclassified
OsISM12109	LOC_Os02g06820	3441331	GGCAGAAAAGATTGGTGGAA	TCAACTGATTCTCATTACTTTGGC	59.9	181	85	transposon protein putative unclassified
OsISM12110	LOC_Os02g06840	3453873	CAAGAACGAACGACTCGTGA	TGAATTTGCGAAAATGGAAC	59.3	755	89	galactosyltransferase putative expressed

ISM IDs	Rice gene locus IDs	Physical positions (bp) on chromosomes	Forward primers (5'-3')	Reverse primers (5'-3')	Melting tempearature (T˚C)	Expected product size (bp) in PCR	Expected product size (bp) in Q-PCR	Putative Functions
OsISM12111	LOC_Os02g06840	3454611	CCATTTTCGCAAATTCAGCTA	AAATTAAAATGGGAAGAAAAGCA	59.3	270	99	galactosyltransferase putative expressed
OsISM12112	LOC_Os02g06840	3454833	GCGGAGTGTGGTCCTAAAAA	TGTTGGACCTGCTAACAAACA	59.7	198	99	galactosyltransferase putative expressed
OsISM12113	LOC_Os02g06840	3455151	TCGGTATTTTTGGATGCTTTG	TTCCAGATTTTAATGTCAACAACC	59.6	263	96	galactosyltransferase putative expressed
OsISM12114	LOC_Os02g06840	3455377	CTGGATGCCTCAAGGTTGTT	TCTTCTTCAAGCTTCTTCCTTTTT	59.7	207	99	galactosyltransferase putative expressed
OsISM12115	LOC_Os02g06840	3455588	GGGATCATCATTCGTTTCGT	CCATGTTTTCTGTCCTCTGCT	59.5	195	65	galactosyltransferase putative expressed
OsISM12116	LOC_Os02g06840	3455838	TGGATCGTATTTATCAAGTGACTG	TGCGAAATATTTGAAACCCTG	59.1	408	97	galactosyltransferase putative expressed
OsISM12117	LOC_Os02g06840	3456239	TTTCGCAAGATAACACGCAG	GGTCCAGATTTCATGCATCC	60.2	191	88	galactosyltransferase putative expressed
OsISM12118	LOC_Os02g06840	3456422	TCTGGACCTGTCCTAACTGAGA	ACCAGTAGCATGCCGAAAAT	59.3	200	99	galactosyltransferase putative expressed
OsISM12119	LOC_Os02g06840	3456681	CACCTGTTTTCATATTTACCTGTG	AGTCTCCGGTCGTCGATATG	58.9	180	55	galactosyltransferase putative expressed
OsISM12120	LOC_Os02g06870	3461605	GCCATGCTTCAAGGTCTCA	CGGTAGATGGAGCAGAGGAT	59.6	151	83	retrotransposon protein putative unclassified
OsISM12121	LOC_Os02g06870	3462639	GGATCTTTCTACCAGGACCG	TCACATTGAATGCTGCGAAT	59.4	492	67	retrotransposon protein putative unclassified
OsISM12122	LOC_Os02g06870	3463212	CACCTCTCCCCAAAATGGTA	ATCGATGAGGTCGACCGAG	60.5	722	95	retrotransposon protein putative unclassified
OsISM12123	LOC_Os02g06890	3471532	AGGTGTTGTTGTGAGGAGGG	GCTCAGAAGCCTTGTTCCTG	60.1	185	96	OTU-like cysteine protease family protein putative expressed
OsISM12124	LOC_Os02g06890	3471697	CAGGAACAAGGCTTCTGAGC	ACGCTTCATTTGGTTTTCCA	60.3	844	99	OTU-like cysteine protease family protein putative expressed
OsISM12125	LOC_Os02g06890	3472521	TGGAAAACCAAATGAAGCGT	CACGCCCATAATATTCCGAT	60.1	544	96	OTU-like cysteine protease family protein putative expressed
OsISM12126	LOC_Os02g06890	3473076	TATGACATCCAGACAACCCG	AAAGCATCATAATGCAGCCC	59.7	179	96	OTU-like cysteine protease family protein putative expressed
OsISM12127	LOC_Os02g06890	3473235	GGGCTGCATTATGATGCTTT	ATTTGTGGTCGACAGGGAAG	60.0	175	86	OTU-like cysteine protease family protein putative expressed
OsISM12128	LOC_Os02g06890	3473421	CCAGTAGAGGGCCTTGCTC	GCAAGGTGAAGTTTGCAGTG	59.7	369	80	OTU-like cysteine protease family protein putative expressed

ISM IDs	Rice gene locus IDs	Physical positions (bp) on chromosomes	Forward primers (5'-3')	Reverse primers (5'-3')	Melting tempearature (T°C)	Expected product size (bp) in PCR	Expected product size (bp) in Q-PCR	Putative Functions
OsISM12129	LOC_Os02g06890	3473800	CTGCCAAATTGGTGTGATTG	GTTGACATGTCCAGTGGCCT	60.5	164	65	OTU-like cysteine protease family protein putative expressed
OsISM12130	LOC_Os02g06910	3487571	AAGAAGAATGAAGCTCTCGCC	TTAGGCACTTCTGCTCCTCTG	59.9	734	87	auxin response factor 6 putative expressed
OsISM12131	LOC_Os02g06910	3488364	CGGGTGGTTTATTTTCCTCA	GCGGCAGGTTAGGATAGTTG	59.8	297	95	auxin response factor 6 putative expressed
OsISM12132	LOC_Os02g06910	3488641	CAACTATCCTAACCTGCCGC	GCGCATAGACTTCATCCGTC	60.3	185	87	auxin response factor 6 putative expressed
OsISM12133	LOC_Os02g06910	3488789	CTTTAGGCCGATGCAGAGAC	GGTAGGAATGGATCCTTGAGC	59.9	191	87	auxin response factor 6 putative expressed
OsISM12134	LOC_Os02g06910	3489077	CGTCGAGCAGCAGAGAAAGT	AACTCTTGAGCTGGAGGCTG	60.3	182	63	auxin response factor 6 putative expressed
OsISM12135	LOC_Os02g06910	3489253	AGAGTTGATGGCGAAAGACC	GCTCCAACCCGTAGTCAGAA	59.8	274	95	auxin response factor 6 putative expressed
OsISM12136	LOC_Os02g06910	3489550	GGTTGCTGGAGACTCTGTCC	TGACCGTTTGTGGCCTATTT	60.1	202	87	auxin response factor 6 putative expressed
OsISM12137	LOC_Os02g06910	3489782	TGCATATTGGTCTGCTTGCT	ATAACAAACTCCGAAGGGCT	58.6	165	98	auxin response factor 6 putative expressed
OsISM12138	LOC_Os02g06910	3490005	TTCCGGATGCTTTTTGAAAC	TCATCCAACGAACAGGATCA	60.1	292	92	auxin response factor 6 putative expressed
OsISM12139	LOC_Os02g06910	3490277	TGATCCTGTTCGTTGGATGA	CACCCTTGGCTGTCTCTCTC	60.0	254	86	auxin response factor 6 putative expressed
OsISM12140	LOC_Os02g06910	3490561	TTCCCGATGTATCCATCTCC	GAAGAAGCCAAGTCATCCTCC	60.0	236	99	auxin response factor 6 putative expressed
OsISM12141	LOC_Os02g06910	3491895	TGCTGATCAAGTGAAGCGAC	ATGTCGAGCAACCTTCCAAC	60.1	184	76	auxin response factor 6 putative expressed
OsISM12142	LOC_Os02g06910	3492181	ATTTGTTGACCGAGAGGACG	TGCGGCGAAAGTATCTTTATG	60.2	185	94	auxin response factor 6 putative expressed
OsISM12143	LOC_Os02g06930	3505871	CAACCCGACAGCAAAGTTCT	AACCCTTGAAAGCTCCTTGT	59.1	197	87	protein kinase putative expressed
OsISM12144	LOC_Os02g06930	3506100	TGAGGGAGGTTTTGGATCTG	TCGCTCCCTGTCTTGACTCT	60.1	194	93	protein kinase putative expressed
OsISM12145	LOC_Os02g06930	3506364	TTGTGTGGAAGGAAACCAAA	TGAACTGGATGTTGCTGTGG	59.9	218	99	protein kinase putative expressed
OsISM12146	LOC_Os02g06930	3506766	TGTTAGCACAAGGGTTGCTG	TGTCACTTGTCCTCGAATGG	59.8	379	68	protein kinase putative expressed

ISM IDs	Rice gene locus IDs	Physical positions (bp) on chromosomes	Forward primers (5'-3')	Reverse primers (5'-3')	Melting tempearature (T˚C)	Expected product size (bp) in PCR	Expected product size (bp) in Q-PCR	Putative Functions
OsISM12147	LOC_Os02g06930	3507193	AGTGGGAGATCCAACACCAA	GAGGAGAACCCCATTTGTGA	60.1	799	88	protein kinase putative expressed
OsISM12150	LOC_Os02g07040	3607638	CCGACGTGCAGTTCAAAG	CATGAGACATCCTGGTGGTG	59.5	544	66	biotin--protein ligase putative expressed
OsISM12151	LOC_Os02g07040	3608253	GACCGAAGCCATCAAAGAAC	AGAGATCATTGGGCCATTTT	59.1	211	78	biotin--protein ligase putative expressed
OsISM12152	LOC_Os02g07040	3608470	AAGGGCTAAAAGTTGGTGGC	GGCTCCTCATTGTCAACATTT	59.8	199	98	biotin--protein ligase putative expressed
OsISM12153	LOC_Os02g07040	3608724	AAACGTGAAGACATACTGGCA	AGTACTGTTCCTCAAGCGCC	58.9	344	95	biotin--protein ligase putative expressed
OsISM12154	LOC_Os02g07040	3609032	CTATTCCAGGATTTCAGGCG	ATCCTGCACAACGACCTTCT	59.7	407	78	biotin--protein ligase putative expressed
OsISM12155	LOC_Os02g07040	3609452	AGCATGCAGACAGTGTGGTT	CCATCAGGGTGCAACTCATA	59.4	172	98	biotin--protein ligase putative expressed
OsISM12156	LOC_Os02g07040	3609557	TGACACCAGCTGGGTACTTG	TTTCCATCTTCCTTCGCACT	59.8	279	95	biotin--protein ligase putative expressed
OsISM12157	LOC_Os02g07044	3611160	TCGGTTGTTTCTGCTCAGTG	ACAGGCATGTACCAGGAAGC	60.1	421	81	rhodanese-like domain containing protein putative expressed
OsISM12158	LOC_Os02g07044	3611559	ATGCTTCCTGGTACATGCCT	TATGTCGAAGAACAGCGCAC	59.8	178	87	rhodanese-like domain containing protein putative expressed
OsISM12159	LOC_Os02g07044	3611717	GTGCGCTGTTCTTCGACATA	TGCAAAAGCCTCTTGTGATG	60.0	326	81	rhodanese-like domain containing protein putative expressed
OsISM12160	LOC_Os02g07044	3612106	TTTTTCAGTGCACCTCGTGT	AATCCAGAAGTTCGCCACTG	59.8	229	99	rhodanese-like domain containing protein putative expressed
OsISM12161	LOC_Os02g07044	3612383	TGCTAACAGTGTTGTTGAAAGGA	TGTCCAGAATATATGAGGCTGAAA	59.9	174	89	rhodanese-like domain containing protein putative expressed
OsISM12162	LOC_Os02g07044	3612505	GGCAAGTGATGTAACATTTCAGA	TGGTGAGACTGTGCATCCAT	59.6	329	91	rhodanese-like domain containing protein putative expressed
OsISM12163	LOC_Os02g07044	3612813	CATGGATGCACAGTCTCACC	TACTCCTTCCCGTGGTTCTG	60.1	281	83	rhodanese-like domain containing protein putative expressed
OsISM12164	LOC_Os02g07044	3613074	CAGAACCACGGGAAGGAGTA	ATCTGCAGGGAGAAGCATTG	60.2	189	99	rhodanese-like domain containing protein putative expressed
OsISM12165	LOC_Os02g07044	3613231	TTGATGATGCACCAATGCTT	ACTACAATGGGGCGATCAAG	60.0	303	86	rhodanese-like domain containing protein putative expressed
OsISM12166	LOC_Os02g07044	3613544	TCTGGTGTAACTGCCTGCAT	TCCCATTCTGTCCAAGATCC	59.6	166	90	rhodanese-like domain containing protein putative expressed

ISM IDs	Rice gene locus IDs	Physical positions (bp) on chromosomes	Forward primers (5'-3')	Reverse primers (5'-3')	Melting tempearature (T˚C)	Expected product size (bp) in PCR	Expected product size (bp) in Q-PCR	Putative Functions
OsISM12167	LOC_Os02g07060	3620240	AGCTCATCGACTCCGTCCT	CCTCCTCCTAGAGAGTGGCA	59.8	914	92	tubulin/FtsZ domain containing protein putative expressed
OsISM12168	LOC_Os02g07060	3621350	TATGACATCTGCTTCCGCAC	ACACCACTCATGGTTGCAGA	60.0	481	84	tubulin/FtsZ domain containing protein putative expressed
OsISM12169	LOC_Os02g07080	3641905	AAGAATTTGGAAAGGACGCA	ACACGTCGATGTCGTTACCA	59.9	209	67	transposon protein putative CACTA En/Spm sub-class
OsISM12170	LOC_Os02g07090	3645899	GAACGAGAGCGAGAACGAAC	GTCGCACCAATTCCGTAGTC	60.3	855	64	transposon protein putative CACTA En/Spm sub-class
OsISM12172	LOC_Os02g07100	3648469	CCATGTGAGCTGCATATTCC	AGTACCTGAAGGGTCCGTTG	59.1	158	82	transposon protein putative CACTA En/Spm sub-class
OsISM12173	LOC_Os02g07100	3649646	GGACACCCTCGACGTCTCTA	TCCAGTATCGAAAACCCTCC	59.6	194	83	transposon protein putative CACTA En/Spm sub-class
OsISM12174	LOC_Os02g07100	3649921	AAGGCGCAGCATTACAAGAC	GCCTCCAGGTCGATGAGTAA	60.3	179	81	transposon protein putative CACTA En/Spm sub-class
OsISM12175	LOC_Os02g07100	3650098	GCCTGCACCATCAACGTAT	AATGACGGAACCGATACCAA	59.9	184	98	transposon protein putative CACTA En/Spm sub-class
OsISM12176	LOC_Os02g07100	3650289	CGGCAAATGGAGAGAAAGAC	GGCAATACTCGCACACGTAA	59.8	195	99	transposon protein putative CACTA En/Spm sub-class
OsISM12177	LOC_Os02g07100	3650484	ACTGCCTTGCAGACCAAATC	CCGCGATAAATTCCTTGTGT	60.1	169	88	transposon protein putative CACTA En/Spm sub-class
OsISM12178	LOC_Os02g07180	3688050	CTTCCTCTCCTGCTTCCG	GTCGTCCAGGAACGCCTC	60.2	204	98	expressed protein
OsISM12180	LOC_Os02g07180	3688429	CGTCGACGAGGAGCTCTG	TTTGGTTTGACCCTTTCAGC	60.5	1253	93	expressed protein
OsISM12181	LOC_Os02g07180	3689662	GCTGAAAGGGTCAAACCAAA	TTTCTTCAGACACCGCTGC	60.1	178	72	expressed protein
OsISM12182	LOC_Os02g07180	3689809	GAGCTTAAAGGAGCAGCGGT	GGTGAGGTGAATCGCTTTGT	60.6	197	98	expressed protein
OsISM12183	LOC_Os02g07180	3690613	AAAGGTGTGGGATGGTAACG	GTTGCGTGCCAACTGACTT	59.8	161	76	expressed protein
OsISM12184	LOC_Os02g07180	3690800	TTCTGTCCGATGAGAAACCG	TGGTCTTCTTGCAAGGGTCT	60.5	183	95	expressed protein
OsISM12185	LOC_Os02g07190	3695634	TGGGACAAGCATTCCTCTTC	TTTGACATCTTCCTGCTCCA	59.8	211	94	expressed protein
OsISM12186	LOC_Os02g07190	3695903	TGGAGGATAATCCAGGTTGC	TTCTCCTTACACCCGTTTGG	59.9	990	71	expressed protein

ISM IDs	Rice gene locus IDs	Physical positions (bp) on chromosomes	Forward primers (5'-3')	Reverse primers (5'-3')	Melting tempearature (T˚C)	Expected product size (bp) in PCR	Expected product size (bp) in Q-PCR	Putative Functions
OsISM12187	LOC_Os02g07190	3696888	GAGAAACAGCCCCATTTTGA	CACGGCTTCCTTCTTTGGTA	60.1	369	97	expressed protein
OsISM12188	LOC_Os02g07190	3698019	TCTGCTGCCCTTAACTTGCT	AAGCAGCCACCATTTTTCAG	60.2	225	93	expressed protein
OsISM12189	LOC_Os02g07190	3698283	TCCTATTGCCCAACATCCTC	CGCTTCCTCTCTGTAAACGC	60.0	182	92	expressed protein
OsISM12190	LOC_Os02g07190	3698620	CTCCCTATCCGAATCCTTCC	GCAAGTTTCTTCAGCCGTTC	59.9	201	97	expressed protein
OsISM12191	LOC_Os02g07190	3699034	TTTGGAAGAGAGGGTGCTTG	TGTCTTTCTTCAGTGGGTAAAGC	60.1	417	90	expressed protein
OsISM12192	LOC_Os02g07230	3715540	CAAGGTCTCGCTCATCCG	CAGCTTGTCTCGGGTTTCAT	60.4	806	77	porphobilinogen deaminase chloroplast precursor putative expressed
OsISM12193	LOC_Os02g07230	3716651	TGGAAGTGCTTCTCTGCGTA	TCGTGTTTGAACATTTCCTCTG	59.9	264	89	porphobilinogen deaminase chloroplast precursor putative expressed
OsISM12194	LOC_Os02g07230	3717052	TGGAATAGCTTGCAGAAGCA	CACATGCAACAGCTAATCTGG	59.5	555	87	porphobilinogen deaminase chloroplast precursor putative expressed
OsISM12195	LOC_Os02g07230	3717706	TGGCTTCACCTGATGGATCT	CTCGTGGCCAGCATCTTTAC	60.7	201	96	porphobilinogen deaminase chloroplast precursor putative expressed
OsISM12196	LOC_Os02g07260	3727877	GTGCCCACTGTCAAGTTCCT	GGCTTCAAGCTGTACTTGGG	60.0	1279	99	phosphoglycerate kinase protein putative expressed
OsISM12197	LOC_Os02g07260	3729156	TCTTGTTCCACGCTTGTCTG	CAACTTCCTCACCAATGCAA	59.9	173	72	phosphoglycerate kinase protein putative expressed
OsISM12198	LOC_Os02g07260	3729489	TGCTTCAACTGAGGGAGTGA	TTTGCAACAGCTCCAACAAG	59.8	464	94	phosphoglycerate kinase protein putative expressed
OsISM12199	LOC_Os02g07260	3730192	CCGATGTTGTAGTGGCTGAC	ACATCAAGACCCATCCAACC	59.4	382	92	phosphoglycerate kinase protein putative expressed
OsISM12200	LOC_Os02g07260	3730637	CGGACCTATGGGAGTCTTTG	GCGCCCTTTGTGGTAGTAAG	59.7	175	91	phosphoglycerate kinase protein putative expressed
OsISM12201	LOC_Os02g07280	3736823	GTGAATGCACCGGAAAGAGT	CCTGTATCCACATAACTGGTGG	59.7	380	93	retrotransposon protein putative unclassified
OsISM12203	LOC_Os02g07300	3742634	GGGCAAAGGAGAAGGAAGAA	GCTCCAGGTAAAAATTGGCA	60.4	285	86	hypothetical protein
OsISM12204	LOC_Os02g07320	3761074	GGAGGAGGAGGAGAAGCAAT	GAGCTCGATTCGTGTCCATAG	59.8	373	59	conserved hypothetical protein
OsISM12205	LOC_Os02g07320	3761795	CGGAAGGACAGACAACAACA	GCAATCCCTTCTCCTCTGTG	59.8	234	95	conserved hypothetical protein

ISM IDs	Rice gene locus IDs	Physical positions (bp) on chromosomes	Forward primers (5'-3')	Reverse primers (5'-3')	Melting tempearature (T°C)	Expected product size (bp) in PCR	Expected product size (bp) in Q-PCR	Putative Functions
OsISM12207	LOC_Os02g07330	3772881	CACTTCTTCCCCGACTTCAA	TTGTAGGTCCCCTTCAAACG	60.1	829	79	expressed protein
OsISM12208	LOC_Os02g07330	3774001	AAAGAGTTCAGCACTCCCCA	ATAAGCACGAGTCCTGCCC	60.0	155	76	expressed protein
OsISM12209	LOC_Os02g07330	3774118	TGTAGGTTCATCGCCTTGTG	CTGACACACCCAGTCCTTGA	59.7	175	90	expressed protein
OsISM12210	LOC_Os02g07330	3774272	TTCAAGGACTGGGTGTGTCA	GGCAAACCAAGCTCTCATCT	59.8	179	97	expressed protein
OsISM12211	LOC_Os02g07330	3774431	AGATGAGAGCTTGGTTTGCC	CACGCCAAATGTAACTGGAA	59.5	463	58	expressed protein
OsISM12212	LOC_Os02g07340	3780293	CTCTTGCCCCACAGCTAATC	AAGGCACTCGCATTTCACTT	59.9	1186	66	HEAT repeat family protein putative expressed
OsISM12213	LOC_Os02g07340	3781541	GCTGACTGCCTTGTTATCCC	TCATCAGACAGACATGCAGC	59.1	200	97	HEAT repeat family protein putative expressed
OsISM12214	LOC_Os02g07340	3781805	ATTGCCCGAACAAATATCCA	ACTTTCCAGCTTGCATCCTC	59.8	739	84	HEAT repeat family protein putative expressed
OsISM12215	LOC_Os02g07340	3782584	TCTCGTCCTGAAATGTTGTCT	TTTCCTCTCTTTCCCTAAACCA	58.3	253	77	HEAT repeat family protein putative expressed
OsISM12216	LOC_Os02g07340	3782788	ATGCAGGCTTGTCCAAAGTT	TGAAAAGCAAAAGGATTGCC	60.0	389	92	HEAT repeat family protein putative expressed
OsISM12217	LOC_Os02g07340	3783226	CAATAGGCAGTTGCGTGAAA	CAAGGGCTTCAATCTTCAGG	59.8	318	75	HEAT repeat family protein putative expressed
OsISM12218	LOC_Os02g07340	3783569	ACATTCCCCAGCTGTGTTTC	AGCAGACAATATTGGGCCAG	60.0	138	59	HEAT repeat family protein putative expressed
OsISM12219	LOC_Os02g07340	3783751	CTGCGAAGAGCTAGTTCGTGT	CGGGCCAAGATAGCCTTATAG	60.0	176	97	HEAT repeat family protein putative expressed
OsISM12220	LOC_Os02g07340	3783927	CTTGGCAAATCAGGATCAGG	GACCATCACCAAACGTGGAT	60.6	169	90	HEAT repeat family protein putative expressed
OsISM12221	LOC_Os02g07340	3784142	GGGTAATGAAATAACTAGACT TACAGC	AGGTTGGGCAGGAATCCAC	59.8	3837	98	HEAT repeat family protein putative expressed
OsISM12222	LOC_Os02g07340	3788077	TATGGAAGCCAAAGGGATTT	CGACCGATCTTCTTATCATGC	59.1	108	69	HEAT repeat family protein putative expressed
OsISM12223	LOC_Os02g07340	3788254	GCCGGTTTATACCACCCATA	ACTTCCTAAGCTTGCAGCCA	59.9	230	90	HEAT repeat family protein putative expressed
OsISM12224	LOC_Os02g07340	3788602	GGAACAAATGGAAATCCACC	CTTGCAACAACGGATGATGT	59.3	968	94	HEAT repeat family protein putative expressed

ISM IDs	Rice gene locus IDs	Physical positions (bp) on chromosomes	Forward primers (5'-3')	Reverse primers (5'-3')	Melting tempearature (T˚C)	Expected product size (bp) in PCR	Expected product size (bp) in Q-PCR	Putative Functions
OsISM12225	LOC_Os02g07340	3789789	ATTACATTGAAAACCGCCGA	CTGGCCAGATTTCTTCGATT	59.8	229	94	HEAT repeat family protein putative expressed
OsISM12226	LOC_Os02g07360	3797744	TCTGTTGCCTTCACCTTGTG	TCTTGGCCTTGTGACAGGAT	60.3	353	69	pentatricopeptide containing protein putative expressed
OsISM12227	LOC_Os02g07360	3798191	AATACAACGGCGCCTACATC	CCCGGCCTCTCATAGCAC	60.9	249	94	pentatricopeptide containing protein putative expressed
OsISM12228	LOC_Os02g07360	3798604	GTCGCTGTCCAATTCCGTAT	CTGTACAGGATCCACTCGCA	59.9	184	69	pentatricopeptide containing protein putative expressed
OsISM12229	LOC_Os02g07360	3798971	AGAAGGCGTGATCTCGGA	TGCCTTCAGTAATCCATCCA	59.3	177	86	pentatricopeptide containing protein putative expressed
OsISM12230	LOC_Os02g07360	3799182	GGATGAAGAGAGAACGGTGC	CATCGATGACATTGGTTGCT	59.7	214	90	pentatricopeptide containing protein putative expressed
OsISM12231	LOC_Os02g07360	3799542	CGTGTATGCTTACAATGCCC	TGTGCTGCATCAAGGAGAAG	59.6	232	87	pentatricopeptide containing protein putative expressed
OsISM12232	LOC_Os02g07360	3799814	AGATGCATATGGAAGAGCCG	AGCATGTGGGACTTCATCGT	60.4	407	97	pentatricopeptide containing protein putative expressed
OsISM12233	LOC_Os02g07370	3804186	CTCGTAAGGAAGAGGCATGG	GTGGACCGATTCCCTTGAC	60.1	1855	80	retrotransposon protein putative unclassified
OsISM12234	LOC_Os02g07380	3811306	AGGAGAGGAGGGATGCTCAC	GCCTCAAAGTCAAGCTCGG	60.9	369	98	retrotransposon protein putative unclassified
OsISM12235	LOC_Os02g07380	3811606	TGGTGGTGAAGTTGGTGCT	TTCTTCCTCCTCCTGTCACG	60.3	1147	97	retrotransposon protein putative unclassified
OsISM12236	LOC_Os02g07380	3813791	GAGGCAGCGGATAGTCACAT	GTTTGCCCGTAGGAAAGAGA	59.8	139	65	retrotransposon protein putative unclassified
OsISM12237	LOC_Os02g07380	3814588	AGTGCGTGTTCGGTGTACCT	ACGCATTGCACATCCCTAAG	60.8	137	59	retrotransposon protein putative unclassified
OsISM12238	LOC_Os02g07380	3814898	AGCCAAATGAACCCTTGCTA	GGTCAGAGGGCGATAGGTG	60.2	287	94	retrotransposon protein putative unclassified
OsISM12239	LOC_Os02g07410	3828082	CAAGATCTCCACTTTCCCCA	CCGAATCACCCTCAACCTTA	60.0	1151	93	glycine cleavage system H protein putative expressed
OsISM12240	LOC_Os02g07410	3829213	TAAGGTTGAGGGTGATTCGG	ACTGCTGCCAACTTCTGGA	59.7	284	98	glycine cleavage system H protein putative expressed
OsISM12241	LOC_Os02g07410	3829583	AGTGAACGATGGACTAGGCG	GATGATCCACCCACTCTCGT	60.1	149	68	glycine cleavage system H protein putative expressed
OsISM12242	LOC_Os02g07440	3843578	TTTCTCTCACGGCCCACTAT	GGTACGTTGACGCTCTGACA	59.8	277	68	DEFL78 - Defensin and Defensin-like DEFL family

ISM IDs	Rice gene locus IDs	Physical positions (bp) on chromosomes	Forward primers (5'-3')	Reverse primers (5'-3')	Melting tempearature (T˚C)	Expected product size (bp) in PCR	Expected product size (bp) in Q-PCR	Putative Functions
[truncated: 502,780 more chars]
